# Molecular signatures of tumor progression in pancreatic adenocarcinoma identified by energy metabolism characteristics

**DOI:** 10.1186/s12885-022-09487-3

**Published:** 2022-04-13

**Authors:** Cong Tan, Xin Wang, Xu Wang, Weiwei Weng, Shu-juan Ni, Meng Zhang, Hesheng Jiang, Lei Wang, Dan Huang, Weiqi Sheng, Mi-die Xu

**Affiliations:** 1 Department of Pathology, Fudan University Shanghai Cancer Center, 270 Dong’an Road, Shanghai, 200032 People’s Republic of China; 2 Department of Oncology, Shanghai Medical College, Fudan University, Shanghai, 200032 China; 3 Institute of Pathology, Fudan University, Shanghai, 200032 China

**Keywords:** Pancreatic adenocarcinoma, Molecular subtype, Energy metabolism-related genes, Prognosis signature

## Abstract

**Background:**

In this study, we performed a molecular evaluation of primary pancreatic adenocarcinoma (PAAD) based on the comprehensive analysis of energy metabolism-related gene (EMRG) expression profiles.

**Methods:**

Molecular subtypes were identified by nonnegative matrix clustering of 565 EMRGs. An overall survival (OS) predictive gene signature was developed and internally and externally validated based on three online PAAD datasets. Hub genes were identified in molecular subtypes by weighted gene correlation network analysis (WGCNA) coexpression algorithm analysis and considered as prognostic genes. LASSO cox regression was conducted to establish a robust prognostic gene model, a four-gene signature, which performed better in survival prediction than four previously reported models. In addition, a novel nomogram constructed by combining clinical features and the 4-gene signature showed high-confidence clinical utility. According to gene set enrichment analysis (GSEA), gene sets related to the high-risk group participate in the neuroactive ligand receptor interaction pathway.

**Conclusions:**

In summary, EMRG-based molecular subtypes and prognostic gene models may provide a novel research direction for patient stratification and trials of targeted therapies.

**Supplementary Information:**

The online version contains supplementary material available at 10.1186/s12885-022-09487-3.

## Introduction

Pancreatic adenocarcinoma (PAAD) is one of the most lethal malignancies, causing 459,000 deaths and 432,000 deaths worldwide, according to GLOBOCAN 2018 [1]. Our current understanding of the complicated genetic and epigenetic alterations and their correlation with the microenvironment has not resulted in a leap in patient survival [2]. Substantial effort is required for further exploration of disease pathogenesis and progression and the identification of early detection and risk evaluation biomarkers that will translate to diverse treatment options.

The reprogramming of cellular metabolism plays an indispensable role in tumorigenesis as both a direct and indirect outcome of oncogenic alteration. Reprogramming enables tumor cells to produce ATP to maintain the reduction-oxidation balance and macromolecular biosynthesis processes required for cell growth, proliferation, and migration. For a long time, it was believed that malignancies mainly restrict their energy metabolism to glycolysis, even in the presence of oxygen, a situation known as the Warburg effect [3]. However, an increasing number of studies have acknowledged the heterogeneous metabolic phenotype of cancer cells [4]. For example, Daemen et al. successfully proposed three highly distinct metabolic subtypes in PAAD through broad metabolite profiling [5]. Although recent bioinformatic analyses have revealed the existence of metabolic subtypes with differential prognosis within PAAD [6], which suggests a relationship between the metabolic genetic expression profile and tumor aggressiveness, almost nothing is known about the potential to define molecular subtypes in PAAD specifically based on the gene expression profiles of energy metabolism-related genes (EMRGs) or how signatures might relate to prognosis. A deep understanding of EMRGs in tumors might provide an important basis for the development of new therapies.

In this study, we constructed energy metabolism-associated molecular subtypes of PAAD by using EMRG expression data from public databases, including TCGA, GEO, and ICGC. Furthermore, we assessed relationships with prognosis and identified differences in clinical and immune characteristics. The prognostic risk model constructed by differentially expressed genes between PAAD molecular subtypes can better evaluate PAAD prognosis. We further used the gene expression datasets from the GEO and ICGC databases to verify the performance of the prognostic risk model.

## Materials and methods

### Data collection and processing

Raw gene expression data and corresponding clinical information of patients with PAAD were obtained from The Cancer Genome Atlas (TCGA), Gene Expression Omnibus (GEO), and the International Cancer Genome Consortium (ICGC). The RNA-seq expression data, RNA-seq count data, and clinical follow-up information of 177 patients diagnosed with PAAD were downloaded through the TCGA GDC API; among them, 171 patients (90%) were randomly selected as the training set for model construction (Table 1). Subsequently, to verify the robustness of the model over different sequencing platforms, all PAAD samples in TCGA database were used as internal verification sets. Furthermore, a GEO dataset, GSE57495, containing transcriptome and clinical data of 63 patients and a series of RNA-seq profiles of 269 samples obtained from the ICGC database, was downloaded for validation datasets (Table 1). Eleven annotated metabolism-related pathways from the Molecular Signature Database v7.0 (MSigDB), which included 594 EMRGs, were downloaded from the Reactome database (https://reactome.org/, Supplementary Table 1). We matched the candidate gene with the TCGA transcriptome matrix, retained genes with detectable signals in more than half of the tissues, and finally obtained 565 genes for subsequent analysis. The workflow is shown in Supplementary Fig. 1.

**Table 1:** Clinical characteristics of the training and validation datasets

Characteristic		TCGA Set	Training Set	GSE57495 Set	ICGC Set
**Age (years)**	< 65	78	71	–	103
	> = 65	93	83	–	154
**Survival state**	Alive	80	74	21	151
	Dead	91	80	42	106
**Gender**	female	78	71	–	120
	male	93	83	–	137
**Pathologic T**	T1	7	6	–	–
	T2	21	20	–	–
	T3	138	123	–	–
	T4/Tx	4	4	–	–
**Pathologic N**	N1	119	107	–	–
	N/Nx	51	46	–	–
**Pathologic M**	Mx	90	81	–	–
	M0/M1	81	72	–	–
**Tumor Stage**	Stage I	19	17	–	–
	Stage II	142	128	–	–
	Stage III	3	3	–	–
	Stage IV	3	3	–	–
**Grade**	G1	28	24	–	–
	G2	92	82	–	–
	G3	47	40	–	–
	G4/Gx	4	4	–	–
**Total**		171	154	63	257

### Identification of energy metabolism molecular subtypes

Among all TCGA and ICGC PAAD samples, 565 EMRGs were extracted. Nonnegative matrix factorization (NMF) [7] was utilized to cluster all PAAD samples, and the optimal numbers of clusters were determined according to indicators including cophenetic correlation [7], silhouette coefficient [8], and residual sum of squares (RSS) [9].

### Analysis of immune scores between molecular subtypes

The fragments per kilobase of exon model per million mapped reads (FPKM) data of genes in the TCGA PAAD dataset were submitted to the TIMER (tumor immune estimation resource) tool [10] and the R software package estimate for calculation of the immune score. Next, the difference in the immune score and stromal score, which represent the relative proportion of immune cells and stromal cells in tumor tissues, was calculated using the R package estimation of stromal and immune cells in malignant tumors using expression data (ESTIMATE) [11]. The estimate score, which refers to the purity of tumor tissues, is the sum of the immune score and stromal score. Then, the differences in the immune scores of the samples between the two subtypes were compared.

### Identify differentially coexpressed genes between molecular subtypes

To identify the differentially coexpressed genes between each subtype, the R software package DESeq2 was used to calculate the differentially expressed genes (DEGs) between the two subtypes, and the thresholds were set to FDR < 0.05 and | log_2_FC | > 1. The weighted gene correlation network analysis (WGCNA) coexpression algorithm was used to detect coexpressed genes and modules by the R package WGCNA [12]. To improve the accuracy of network construction, the TPM profiles of genes were subjected to hierarchical cluster analysis to remove outlier samples. Second, the distance between each gene was calculated using the Pearson correlation coefficient; a weighted coexpression network was constructed using the R package WGCNA, and coexpression modules were screened by setting the soft threshold power *β* to 10. Third, the topology overlap matrix (TOM) was then constructed from the adjacency matrix to avoid the influence of noise and spurious associations. On the basis of TOM, average-linkage hierarchical clustering using the dynamic shear tree method was subsequently conducted to define coexpression modules, and the minimum gene size of each module was set as 30. The feature vector values ​​(eigengenes) of each module were calculated in turn to explore the relationship among modules, and then modules with highly correlated eigengenes were merged into new modules by performing cluster analysis with the following thresholds: height = 0.25, DeepSplit = 2, and minModuleSize = 30. To identify the modules of interest, the correlation between each coexpression module and patients’ clinical features as well as cluster subtypes was further evaluated. Modules with a significant correlation with the energy metabolism subtypes were defined as key modules for the subsequent selection of hub genes (Spearman correlation coefficient > 0.4, *P* < 0.05). Finally, pathway enrichment analysis of differentially coexpressed genes was performed using the R package WebGestaltR (threshold FDR < 0.05).

### Establishment of prognosis prediction model

The R package survival coxph function was used for analysis of the univariate Cox proportional hazards regression model, and log rank *p* < 0.01 was selected as the threshold. To narrow the gene range and maximize the accuracy, least absolute shrinkage and selection operator (LASSO) Cox regression analysis [13], a method for screening signatures with generally effective prognostic performance by performing automatic feature selection, was performed by using the glmnet package of R to identify the prognostic genes. Optimal genes were evaluated by 10-fold cross validation. Genes obtained by LASSO analysis were subjected to multivariate Cox survival analysis to construct a final prognostic risk model. Time-dependent receiver operating characteristic (ROC) curve analysis was conducted to assess the prognostic value of the identified model using the R package timeROC [14]. The risk scores of patients in the internal verification set and the external verification set were analyzed using the same model coefficients as the training set to verify the robustness of the gene signature. Kaplan–Meier curves were used to evaluate the difference in survival time between groups, and then univariate and multivariate Cox regression analyses were performed to evaluate independent prognostic factors. A *P* value < 0.05 was considered statistically significant. Decision curve analysis (DCA), which can evaluate predictive models from the perspective of clinical consequences [15], was performed in the entire cohort to test the clinical usefulness of the nomogram in comparison with the gene signature and clinicopathological parameters. A restricted mean survival time (RMST) curve was drawn to construct the comparison with the R package rms.

### Bioinformatic analysis

Data processing and symbol remapping were conducted using R-4.0.1 software. A P value < 0.05 was considered statistically significant. Single-sample gene set enrichment analysis (ssGSEA) was applied to identify the relationship between the risk scores of different samples and biological functions using the R package GSVA. The classical gene sets of Kyoto Encyclopedia of Genes and Genomes (KEGG) pathways (c2.cp.kegg.v7.0.symbols) were considered to decipher the phenotype [16–18]. For each analytical pathway, the enrichment score (ES) and the significance of ES were calculated, and the normalized enrichment score (NES) and false discovery rate (FDR) were further calculated to examine functional enrichment results.

## Result

### Construction of energy metabolism-related molecular subtypes

**Fig. 1:**
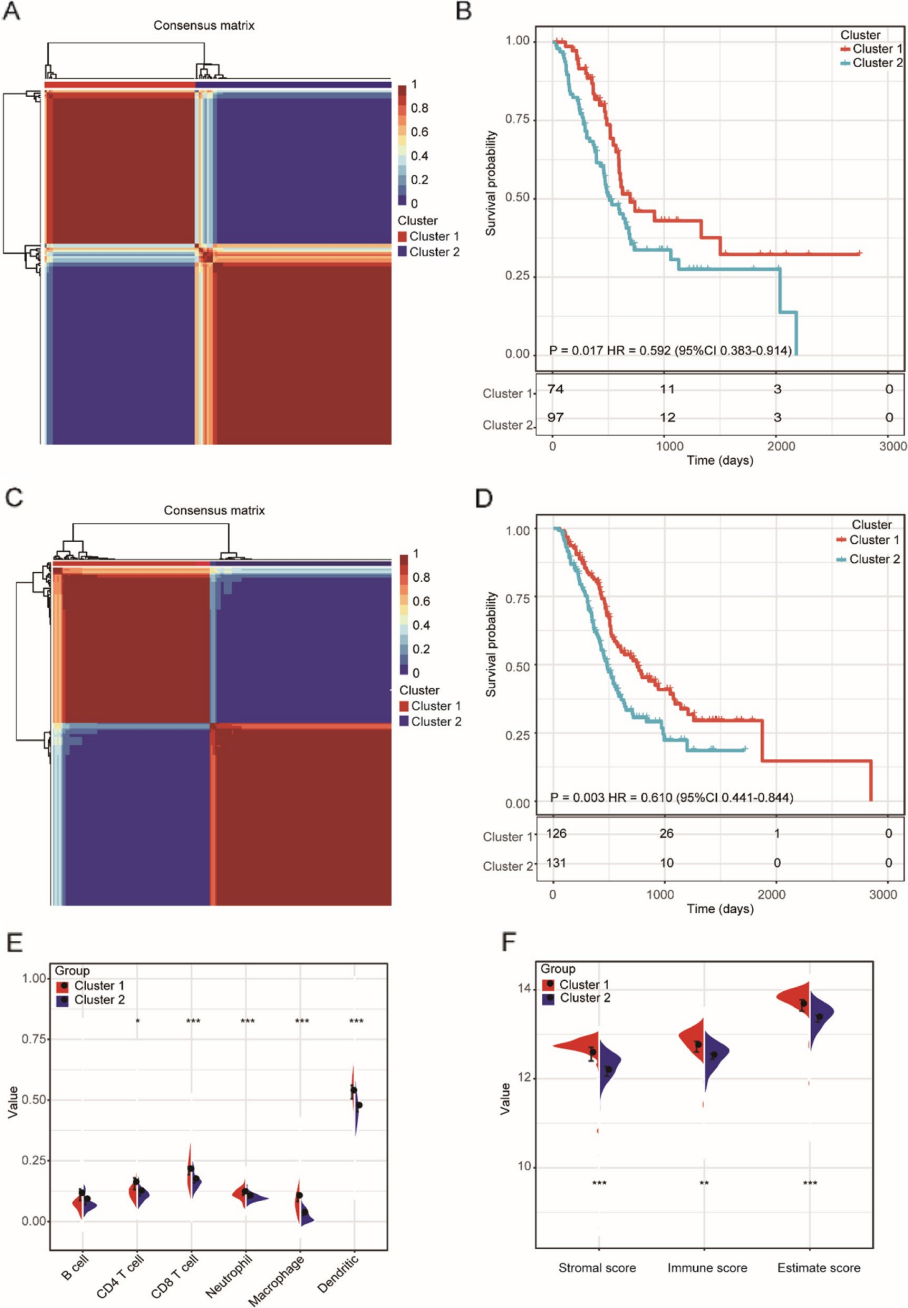
Identification of molecular subtypes in PAAD. **A** Consensus map of NMF clustering in TCGA PAAD dataset; **B** Kaplan–Meier curves showing the overall survival (OS) curve of the two subtypes in TCGA PAAD dataset; **C** Consensus map of NMF clustering in ICGC PAAD dataset; **D** Kaplan–Meier curves showing the overall survival (OS) curve of the two subtypes in ICGC PAAD dataset; **E** The proportions of B cells, CD4 + T cells, CD8 + T cells, neutrophils, macrophages, and dendritic cells (DCs) between the two subtypes; **F** Distribution of the ImmuneScore, StromalScore, and ESTIMATEScore between the two subtypes

By using NMF analysis based on the expression of the 565 EMRGs (Supplementary Fig. 2A), we identified two distinct subtypes (Cluster 1 [*n* = 74], Cluster 2 [*n* = 97]) between the 171 patients in the TCGA PAAD dataset (Fig. 1A-B). Clinically, patients in Cluster 1 showed a significantly higher tumor grade than those in Cluster 2 (Supplementary Fig. 2C). Moreover, we assessed the potential difference in prognosis between the two subtypes, and patients in Cluster 1 had significantly better OS compared with patients in Cluster 2 (*p* = 0.017, HR = 0.597, 95%CI 0.383–0.914, Fig. 1B). Similarly, the expression profiles of these 565 EMRGs also divided 257 patients into two molecular subtypes in the ICGC PAAD dataset (Fig. 1C-D, Supplementary Fig. 2B), and patients in Cluster 1 also showed significantly better OS compared with patients in Cluster 2 (*p* = 0.003, HR = 0.610, 95%CI 0.441–0.844, Fig. 1D). These data show the consistency of these molecular subtypes in PAAD.

Then, we calculated the immune scores of six cell types (B cells, CD4 T cells, CD8 T cells, neutrophils, macrophages, and dendritic cells) in each PAAD sample and analyzed the potential difference between Cluster 1 and Cluster 2. The results showed that except for the B cell immune score, Cluster 1 showed a higher immune score than Cluster 2 (Fig. 1E). We further observed that the scores of immunity, stroma, and tumor purity in Cluster 1 were also significantly higher than those in Cluster 2 (Fig. 1F). These results indicate that lower immune cell infiltration in the tumor environment (TME) may confer worse prognosis in patients with PAAD.

### Identification of differentially coexpressed genes between subtypes

**Fig. 2:**
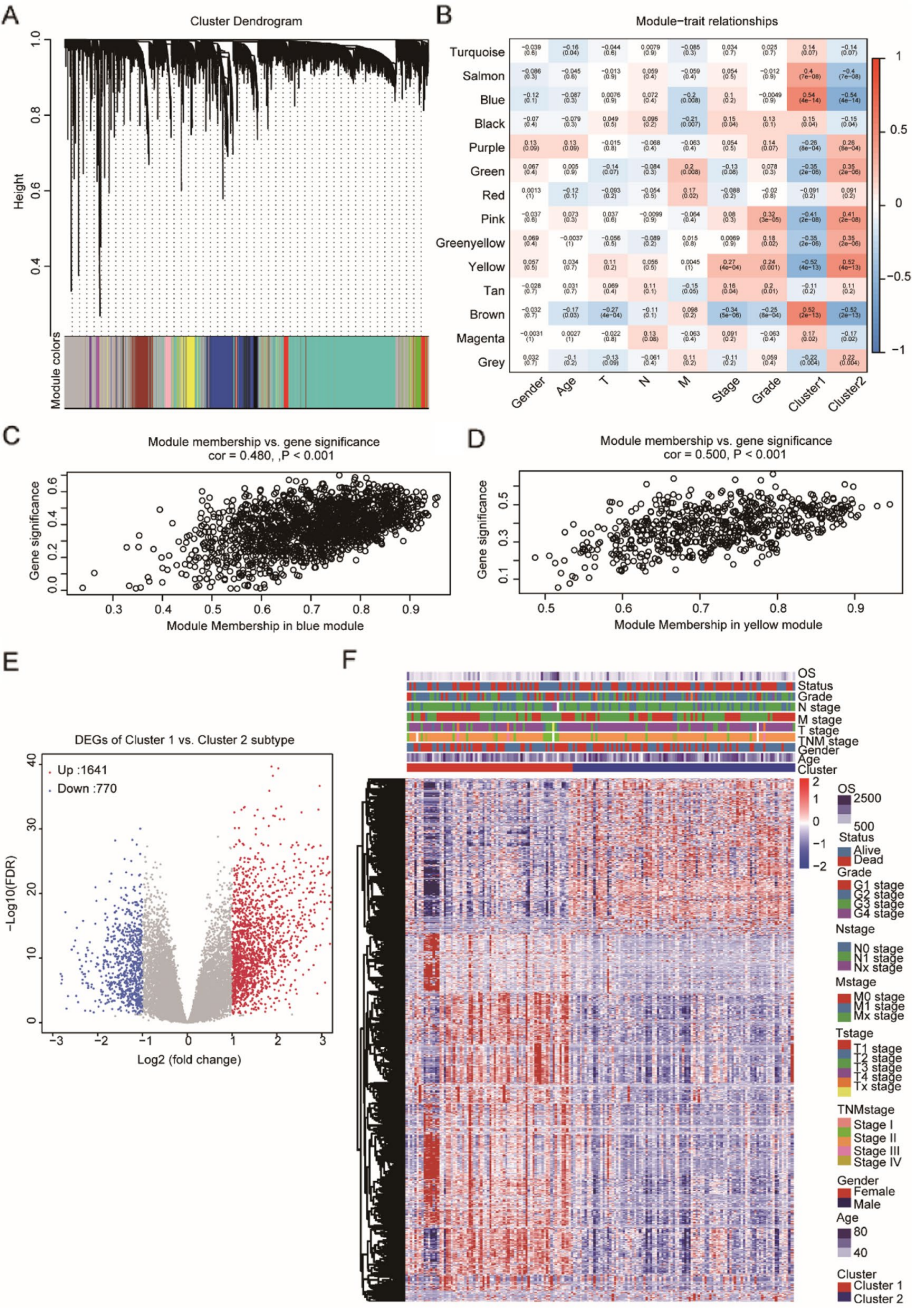
WGCNA coexpression analysis. **A** Gene dendrogram and module colors; **B** Relationship between the 29 modules and the clinical phenotypes and molecular subtypes. **C-D** The correlation of the blue module with Cluster 1 (**C**) and the yellow module with Cluster 2 (**D**) in the TCGA PAAD dataset; **E** Volcano map of differentially expressed genes (DEGs) between the two subtypes; **F** Heat map of the expression profile of 2411 DEGs and the distribution of clinicopathological parameters in the two subtypes

We extracted the expression profile of protein-coding genes from the TCGA PAAD dataset and clustered all samples through hierarchical clustering (Supplementary Fig. 3A), from which we confirmed that there was no outlier sample. To ensure that the network constructed by WGCNA was scale-free, β was set as 10 (Supplementary Fig. 3B). Then, we performed cluster analysis and obtained 14 modules, among which the gray module represented gene sets that could not be aggregated to other modules (Fig. 2A). Moreover, by analyzing the correlation of the module and genes in the module with phenotypes (Supplementary Table 2), we found that the blue module (containing 1692 coexpressed genes) was significantly correlated with Cluster 1, and the yellow module (containing 645 coexpressed genes) was significantly correlated with Cluster 2 (Fig. 2B-D). In addition, we identified 2411 DEGs differentially expressed genes (DEGs) between Cluster 1 and Cluster 2, comprising 1641 upregulated DEGs and 770 downregulated DEGs (Fig. 2E-F, Supplementary Table 3). We further analyzed these 2411 DEGs and their coexpressed genes in the blue and yellow modules and identified 743 overlapping genes (Supplementary Table 4). These 743 coexpressed DEGs were analyzed by GO function and KEGG pathway enrichment (Supplementary Table 5), and 38 KEGG pathways, 52 GO cellular component (CC), 126 GO molecular function (MF), and 977 GO biological process (BP) were enriched. The top enriched pathways included cell adhesion molecules (CAMs), transcriptional misregulation in cancer, immunological synapses, and T cell differentiation (Supplementary Fig. 3C-F), suggesting that these coexpressed DEGs may be involved in the PAAD molecular regulatory network by performing pivotal functions through these pathways.

### Development of a prognostic risk model based on coexpressed DEGs

By analyzing the expression profiles of 743 coexpressed DEGs and the corresponding survival of the training set using a univariate Cox proportional hazard regression model, we identified sixty-seven prognostic coexpression DEGs (*P* < 0.01, Supplementary Table 6). After Lasso Cox regression analysis and 10-fold cross validation, we selected four genes (λ = 0.1042) as candidate genes for construction of the prognostic risk model (Supplementary Fig. 4A-B). We then established a gene-based prognostic model by using univariate Cox regression analysis (Table 2). High expression levels of GJB5, MET, and TMEM139 were identified as risk factors, whereas AFF3 was identified as a protective factor. The final 4-gene signature formula is as follows: RiskScore = − 0.1513* exp^AFF3^ + 0.0156*exp^GJB5^ + 0.0045*exp^MET^ + 0.0164*exp™^EM139^.

**Table 2:** Univariate Cox regression of the 4-gene signature

Symbol	coefficient	Hazard ration	Z-score	*P* value	Low 95%CI	High 95%CI
AFF3	−0.1513	0.8595	−1.7450	0.0809	0.7252	1.0190
GJB5	0.0156	1.0157	3.4580	0.0005	1.0068	1.0250
MET	0.0045	1.0045	2.3600	0.0183	1.0008	1.0080
TMEM139	0.0164	1.0165	1.8980	0.0577	0.9995	1.0340

**Fig. 3:**
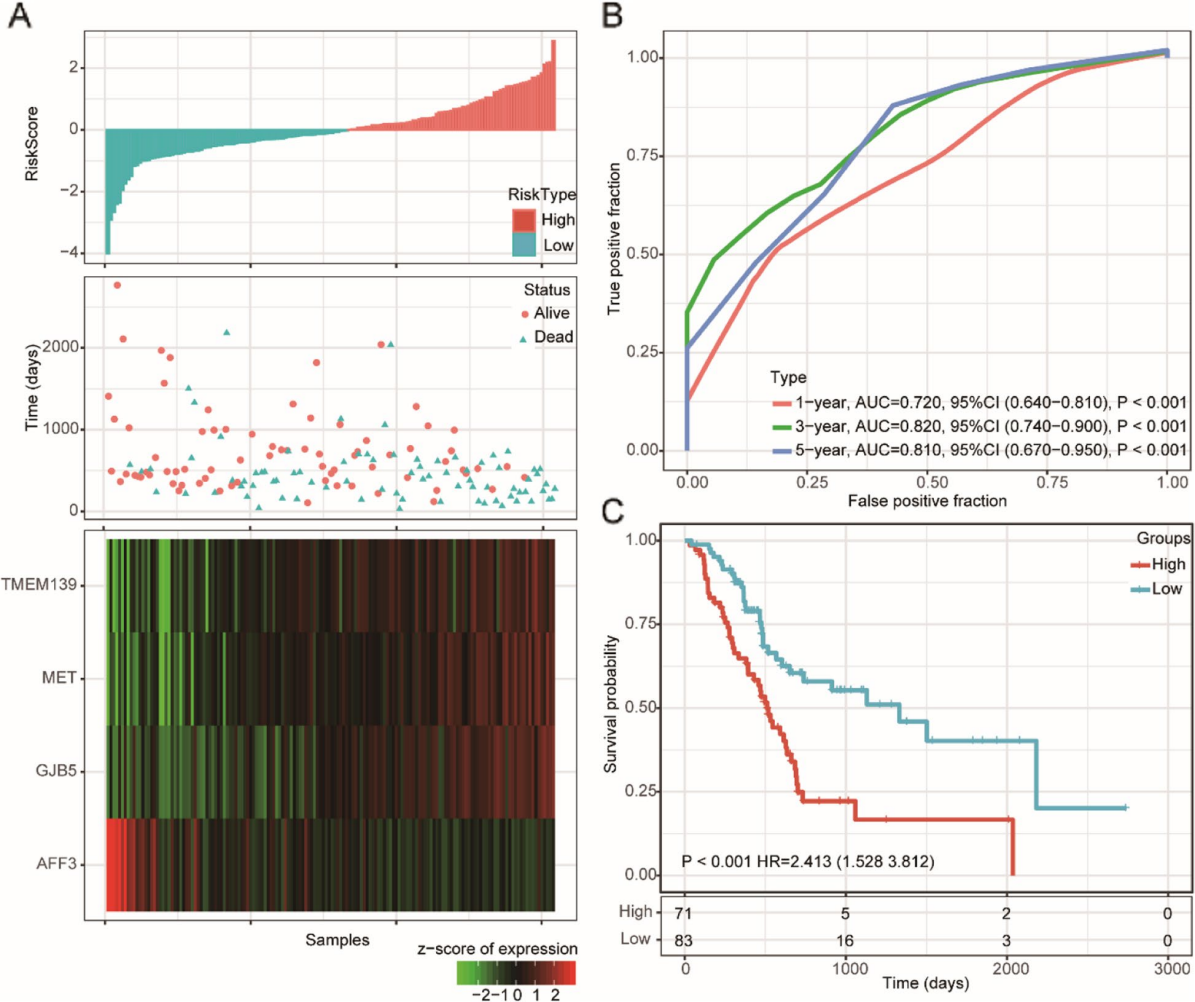
Construction of the 4-gene signature in the training dataset. **A** Risk score, survival time, survival status, and expression of the 4-gene signature in the training set; **B** ROC curve of the 11-gene signature for 1-year, 3-year, and 5-year survival in the training set. **C** Kaplan–Meier survival analysis of overall survival for high-risk or low-risk group patients in the training set. ROC, receiver operating characteristic; AUC, area under the curve; HR, hazard ratio; CI, confidence interval

We calculated the risk score of each sample according to the established model and plotted the risk score distribution, which showed that the survival time of the samples with high risk scores was significantly shorter than that of those with low risk scores (Fig. 3A). In addition, the AUCs of the 1-, 3-, and 5-year ROC curves for the 4-gene signature to predict PAAD survival were all above 0.70 (Fig. 3B). Finally, we performed Z-score normalization on the risk score, which classified samples with a risk score greater than zero into the high-risk group and samples with a risk score less than zero into the low-risk group. Kaplan-Meier survival analysis demonstrated that there were significant differences between the high- and low-risk groups (log rank *P* < 0.001, HR = 2.413, Fig. 3C). We further obtained the subtype schema of samples in the TCGA PAAD cohort [19] and compared the difference in risk scores between basal and classic samples. We observed that basal samples had a significantly higher risk score than classical samples (Supplementary Fig. 4C).

### Internal and external validation of the prognostic risk model

**Fig. 4:**
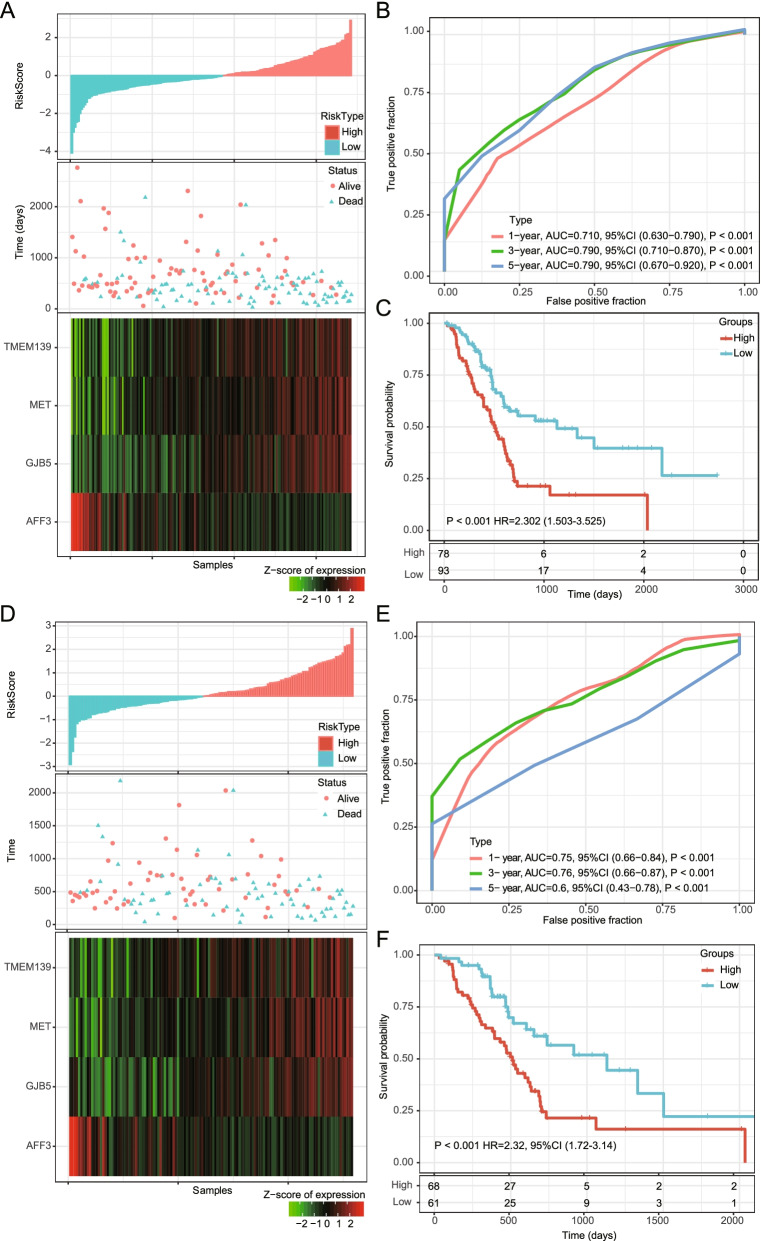
Internal validation of the robustness of the 4-gene signature in the entire TCGA cohort. **A** Risk score, survival time, survival status, and expression of the 4-gene signature in the training set; **B** ROC curve of the 11-gene signature for 1-year, 3-year, and 5-year survival in the entire TCGA cohort; **C** Kaplan–Meier survival analysis of overall survival for high-risk or low-risk group patients in the entire TCGA set. ROC, receiver operating characteristic; AUC, area under the curve; HR, hazard ratio; CI, confidence interval

To determine the robustness of the model, we subjected patient data from the entire TCGA dataset to our prognostic gene signature formula. The risk score distribution of all samples (Fig. 4A), corresponding ROC curves (Fig. 4B), and Kaplan–Meier survival curves (Fig. 4C) showed that the AUCs of the signature remained high, and the high-risk groups had consistently shorter OS than the low-risk groups.

**Fig. 5:**
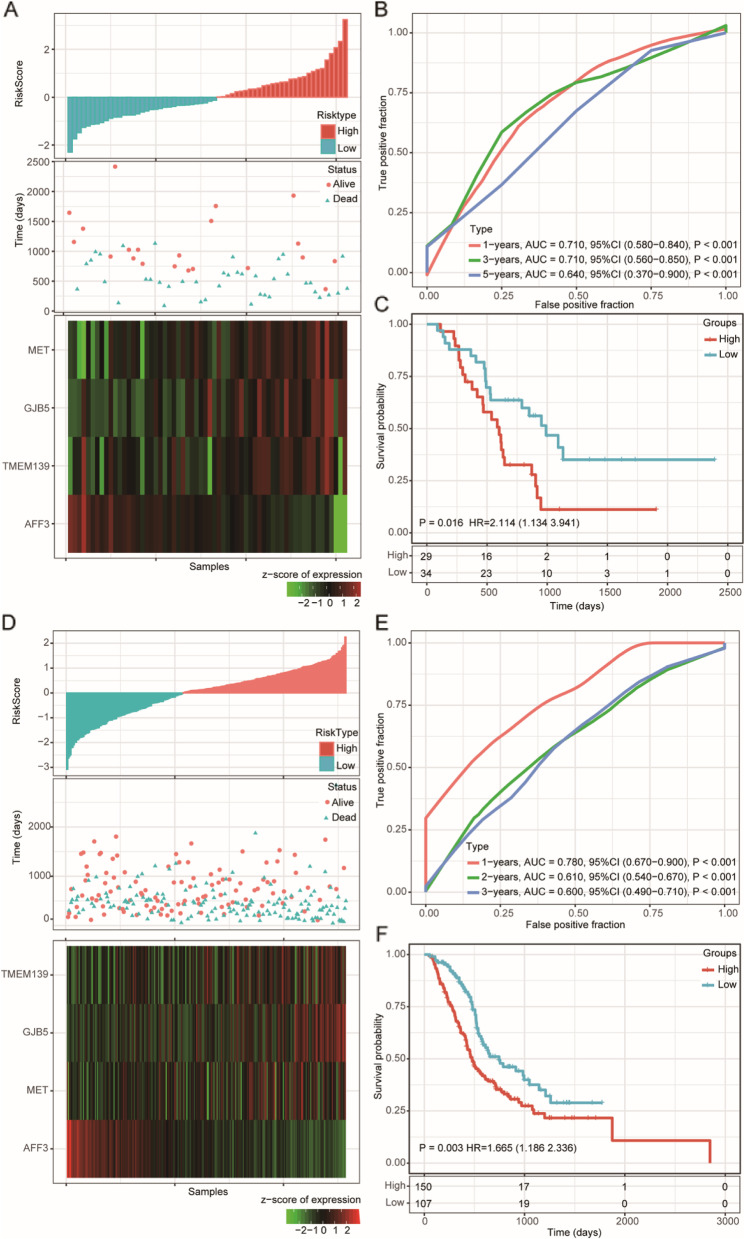
External validation of the robustness of the 4-gene signature in the GSE57495 and ICGC PAAD cohorts. **A** Risk score, survival time, survival status, and expression of the 4-gene signature in the GSE57495 dataset; **B** ROC curve of the 4-gene signature for 1-year, 3-year, and 5-year survival in the GSE57495 cohort; **C** Kaplan–Meier survival curve based on the 4-gene signature in the GSE57495 cohorts. **D** Risk score, survival time, survival status, and expression of the 4-gene signature in the ICGC PAAD dataset; **E** ROC curve of the 4-gene signature for 1-year, 3-year, and 5-year survival in the ICGC PAAD cohort; (**D**) Kaplan–Meier survival curve based on the 4-gene signature in the ICGC PAAD cohorts. ROC, receiver operating characteristic; AUC, area under the curve; HR, hazard ratio; CI, confidence interval

However, we noticed that 27 samples in the TCGA cohort were not exactly pancreatic ductal adenocarcinoma. Among them, ten samples were normal pancreas with atrophy, eight samples were neuroendocrine neoplasms, four samples were tumors derived from other organs (duodenum-ampulla in three cases and undefined location in one case), two samples were intraductal papillary neoplasms, one sample was an acinar cell carcinoma, one was a ductal adenocarcinoma but had received neoadjuvant chemotherapy and one had a normal ampulla [20]. Therefore, we further subjected patients’ data of the 146 exactly PAAD cases from the TCGA cohort to our prognostic gene signature formula (Fig. 4D). The corresponding ROC curves (Fig. 4E) showed high AUCs similar to the whole TCGA cohort. And Kaplan–Meier survival results also showed that patients in the high-risk groups had consistently shorter OS than the low-risk groups (Fig. 4F). Therefore, we believe that the impact of these 27 samples on the remaining 150 samples is acceptable. We further verified the robustness of the 4-gene prognosis signature by external analysis in the GSE57495 dataset (Fig. 5A-C) and ICGC PAAD dataset (Fig. 5D-F) using the same coefficients in our prognostic gene signature formula. Excellent performance was observed in the prognostic risk indication.

### Independence of the 4-gene prognostic signature

**Fig. 6:**
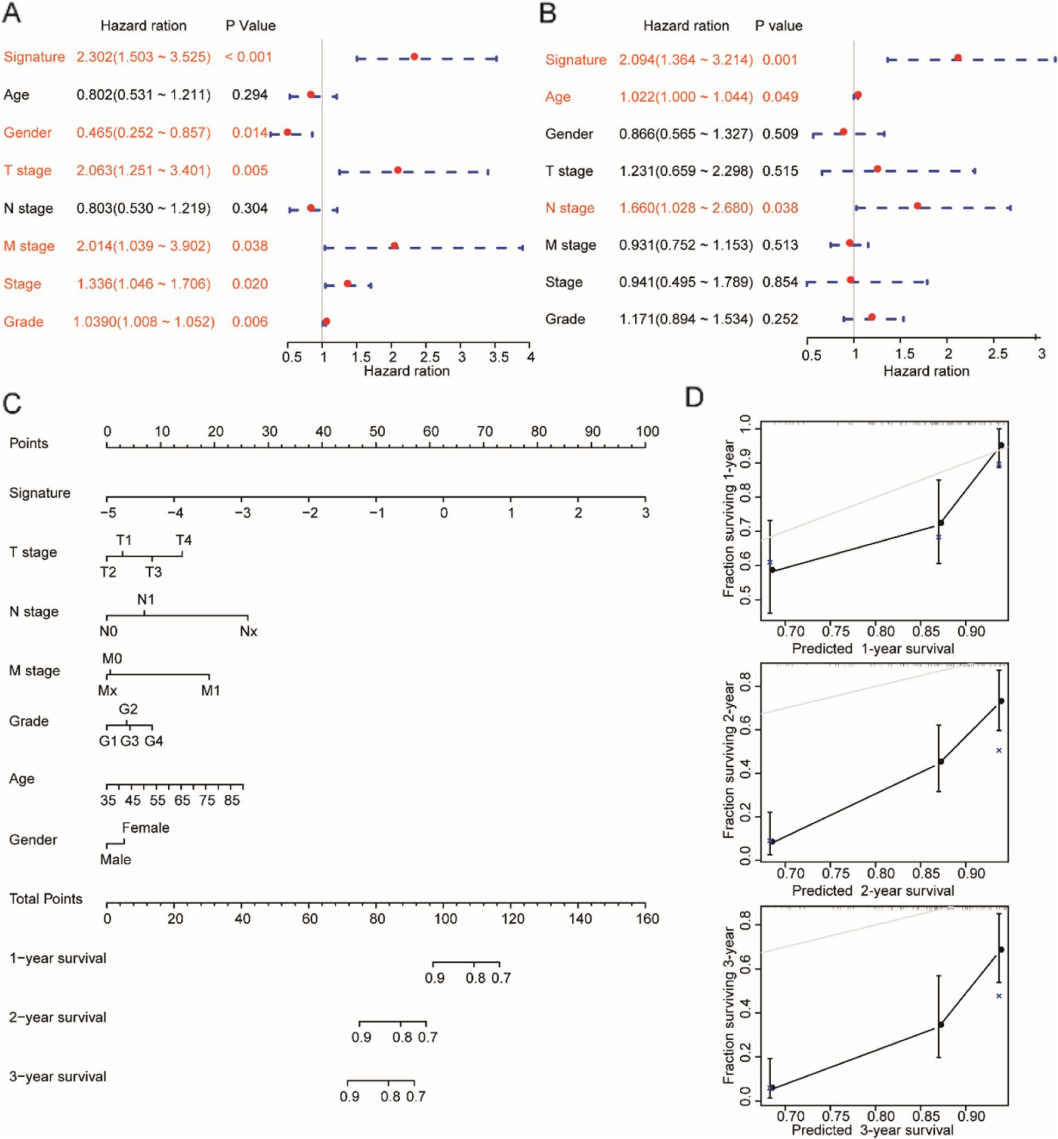
Independence of the 4-gene prognosis signature. **A-B** Forest plot of the univariate (**A**) and multivariate (**B**) Cox regression analyses in the *TCGA* PAAD dataset; **C** A nomogram was developed by integrating the signature risk score with the clinicopathologic features in the TCGA PAAD dataset. **D** Calibration curves of the nomogram for predicting OS at 1 year, 3 years and 5 years in the *TCGA* PAAD dataset

To assess the independence of the 4-gene signature in clinical application, we conducted univariate and multivariate Cox regression in the TCGA PAAD dataset. We systematically analyzed the clinical data of patients, including age, sex, pathologic T stage, pathologic N stage, pathologic M stage, tumor stage, tumor grade, Basal/classical phenotype [19] and the 4-gene signature. Univariate Cox regression analysis showed that age, tumor grade, pathologic T stage, pathologic N stage, tumor stage, and the 4-gene signature were significantly associated with survival (*P* < 0.05, Fig. 6A). However, multivariate Cox regression analysis revealed that only the 4-gene signature (Fig. 6B) were independent prognostic indicators in PAAD. The above conditions indicated that the 4-gene signature has good predictive performance in clinical application.

Furthermore, we combined clinical features and the 4-gene signature and constructed a nomogram using the entire TCGA PAAD dataset (Fig. 6C). The nomogram suggested that the 4-gene signature had the greatest impact on the survival rate prediction. We calibrated the performance of 1-, 2-, and 3-year nomography data for visualization of the nomogram, which further verified the consistency between the predicted and actual survival (Fig. 6D).

### **C**omparison with previous prognostic models

**Fig. 7:**
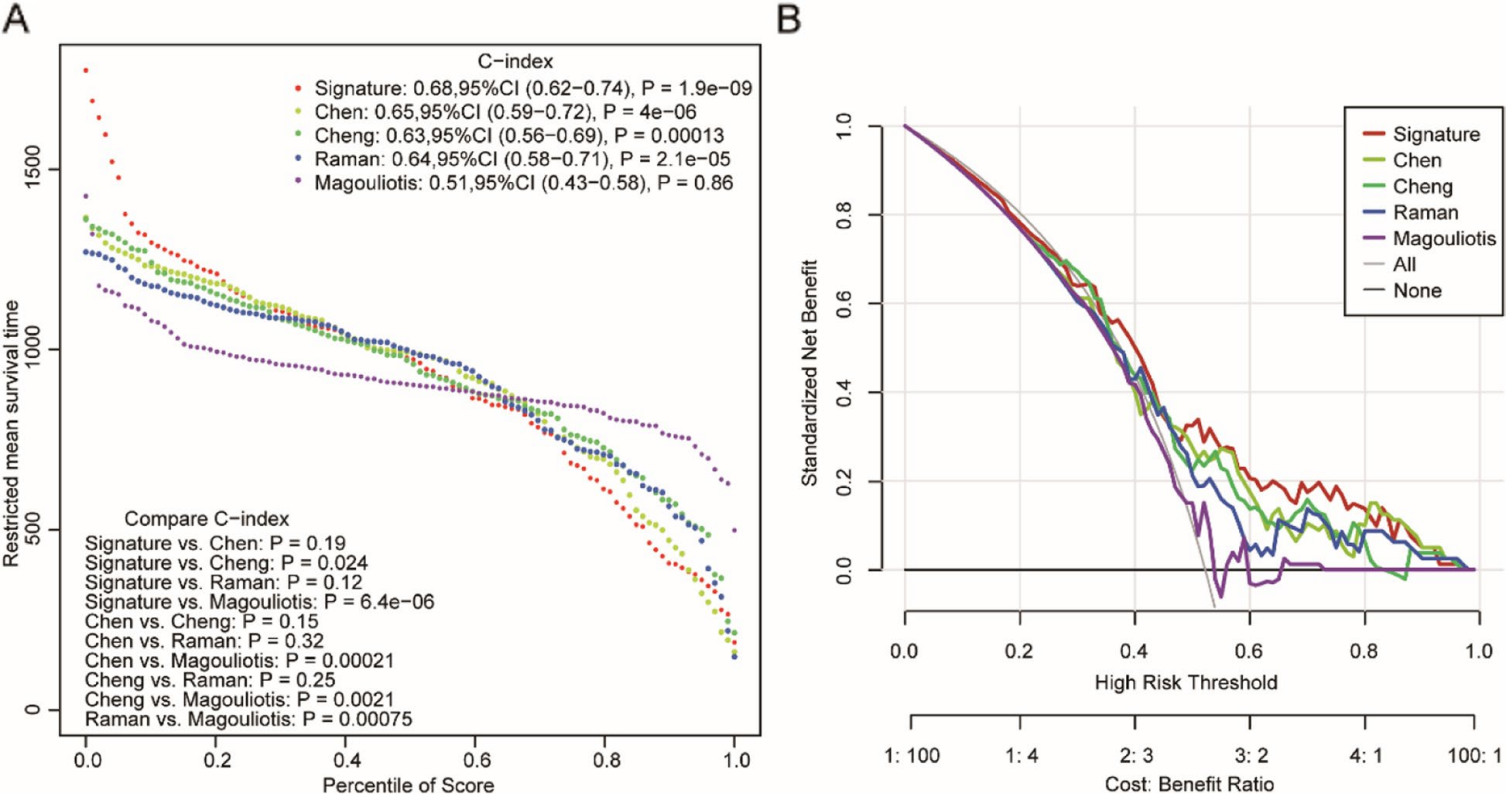
The performance of the 6-gene signature in comparison to previous signatures in the TCGA STAD dataset. **A** Restricted mean survival time (RMST) curve developed by integrating the indicated 5 signatures; **B** DCA plots developed by integrating the indicated 5 signatures

Previous studies have identified several prognostic models for PAAD survival. The predictive performance of the present 4-gene signature was further compared with four previous models (a 15-gene signature proposed by Chen et al. [21], a 7-gene signature proposed by Cheng et al. [22], a 5-gene signature proposed by Raman et al. [23], and a 7-gene signature proposed by Magouliotis et al. [24]). We calculated the risk score of each PAAD sample in the TCGA PAAD dataset based on the corresponding coefficients provided by each model, evaluated the ROC of each model, and divided the samples into high-risk and low-risk groups based on the median risk score of each signature. All four models divided the patients into a high-risk group and a low-risk group (Supplementary Fig. 5). Kaplan–Meier curves showed that there were significant differences between the high-risk and low-risk groups in the Chen, Cheng, and Raman models (*P* < 0.05) but no significant difference for the Li model (*P* = 0.076, Supplementary Fig. 5A-D). Among the four models, the AUCs of the Chen model and Raman model were greater than 0.70, but generally, the predictive efficacy of the four models was worse than that of our four-gene model (Supplementary Fig. 5E-H). Furthermore, RMST curves (Fig. 7A) and DCA curves (Fig. 7B) were used to evaluate the predictive effect of our 4-gene signature and the four published models on the prognosis of PAAD patients, and both curves demonstrated that the performance of our four-gene model was significantly better than that of the previous four models.

## GSEA of enriched pathways based on risk score

**Fig. 8:**
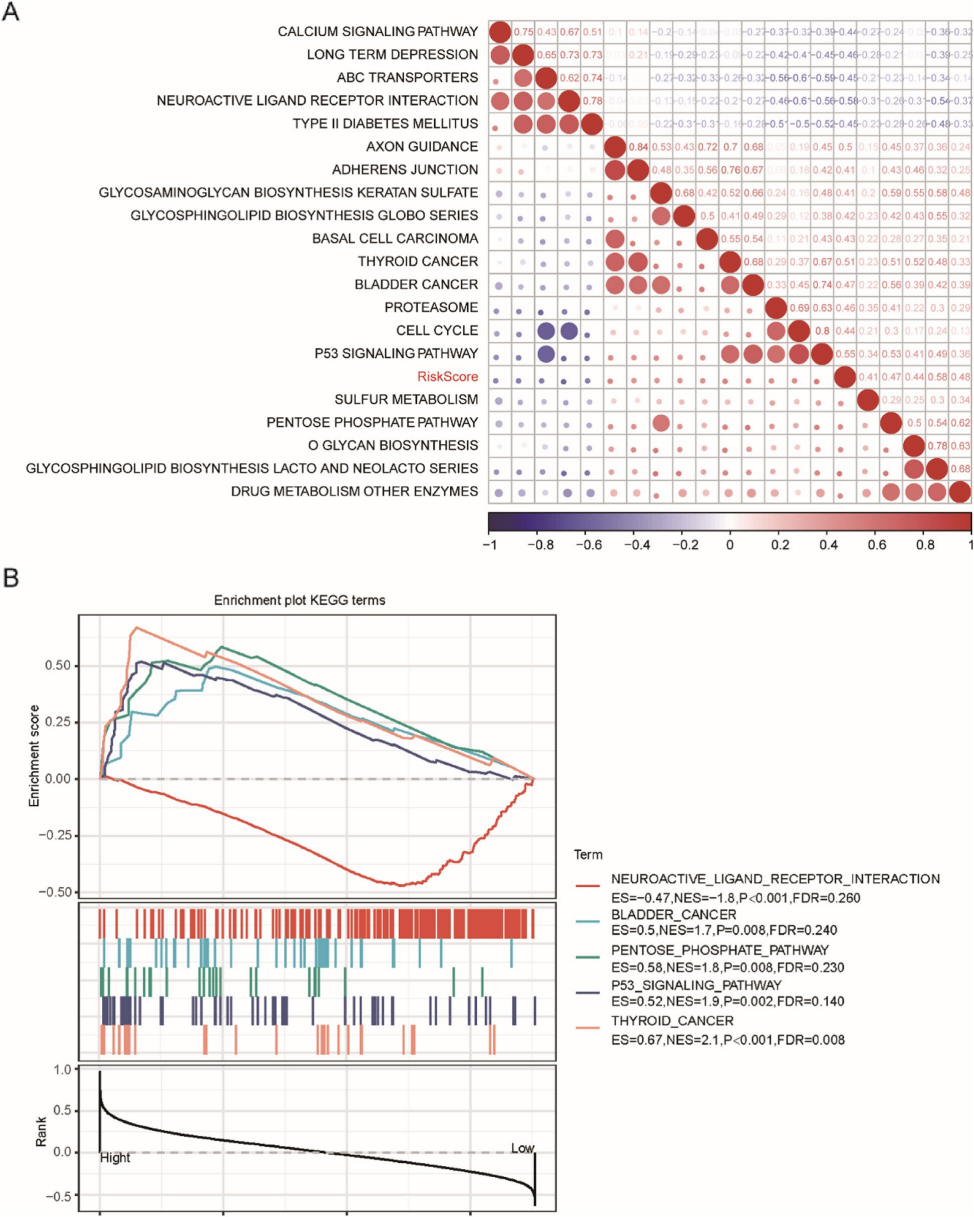
ssGSEA results according to the risk score of PAAD samples in the TCGA dataset. **A** Clustering of KEGG pathways correlated with RiskScore, with correlation coefficients greater than 0.40; **B** Enrichment pathways that were significantly correlated in the high-risk and low-risk groups

To investigate the relationship between the risk score and biological function of different samples, we conducted single sample GSEA (ssGSEA) analysis and calculated the ssGSEA score of each sample on different biological functions. The correlation between these functions and the RiskScore with a coefficient cutoff of 0.4 showed that most of the functional pathways were negatively correlated with the RiskScore of the samples (Fig. 8A). Moreover, we divided the training set into a high-risk group and a low-risk group according to the risk score. GSEA was used to analyze the significantly enriched pathways in the two groups (Supplementary Table 7). Pathways including bladder cancer, the pentose phosphate pathway, the p53 signaling pathway, and thyroid cancer were significantly negatively correlated with the low-risk group, whereas the neuroactive ligand receptor interaction pathway was negatively correlated with the high-risk group (*P* < 0.01, Fig. 8B).

## Discussion

Cumulative evidence has revealed that metabolic reprogramming in cancer is extensively linked to oncogenesis and immune disorders [25, 26]. In PAAD, previous studies suggested that the metabolic alteration in PAAD was typically characterized by the overexpression of glycolytic enzymes and lactate dehydrogenase for glucose, amino acid, and lipid metabolism [27, 28]. Moreover, there is complex crosstalk among these reprogrammed metabolic pathways within the tumor microenvironment, which contributes to the extraordinary growth advantages of tumor cells and unlimited development of PAAD [28].

The detection of aberrant metabolomics also contributes to the identification of novel biomarkers for diagnosis and prognosis and the discovery of potential therapeutic targets for PAAD. For example, there are significant differences in metabolic profiles not only between PAAD patients and normal controls but also among different pathological PAAD subtypes [29, 30], and metabolic alterations have helped identify several promising metabolomics-based diagnostic biomarkers, such as single serum metabolites [31] and even metabolomics-based biomarker signatures in blood [32]. Oliver F. Bathe et al. proposed the utility of serum metabolomic profiling in discriminating PAAD patients from healthy controls [33]. PAAD patients with higher levels of PE in serum exosomes might have a worse prognosis according to a population-based study [34]. Taken together, the distinct characteristics of energy metabolism in PAAD are worth exploring and may shed new light on the development of novel biomarkers related to metabolism. However, the accurate detection of metabolites in biological samples remains hampered by some technical defects, such as a lack of optimized study methods, limited coverage in metabolomics fingerprints, and interference caused by unwanted sources [35]. Moreover, the abundance of some metabolites can be quite low and fall below the detection limit [36]. Gene expression profiling, with the advantage of being convenient and precise, can provide a complete picture of tumor properties based on quantitative data [37]. By analyzing the expression levels of EMRGs in PAAD tumor tissue, the metabolic characteristics of PAAD can be comprehensively interpreted from another dimension.

In the present study, a total of 565 EMRGs were selected from the Reactome database. These genes mainly participate in the key pathways of carbohydrate, fatty acid, and glycogen metabolism. Based on the expression data of the TCGA-PAAD dataset, pancreatic cancer patients were divided into two metabolic subtypes using the NMF algorithm. Significant differences were observed in patients’ immune cell infiltration and survival status between the two subtypes. Moreover, the proportions of nearly all immune cells and the fraction of immune components were significantly higher in the subtype with significantly better clinical outcomes, which strongly indicates the close relationship between tumor energy metabolism and immunology in PAAD. Previous evidence has shown that metabolic interventions can impact the immune functions of immune cells upon activation [38, 39]. This phenomenon revealed the potential influence of the cross-talk between energy metabolism and the immune microenvironment on the development and long-term survival of PAAD.

To select the hub genes that may significantly modulate cancer metabolism in PAAD, WGCNA coexpression analysis was conducted, and a total of 743 genes that strongly correlated with the two metabolic subtypes and were differentially expressed between the two subtypes were identified and considered as candidates for the construction of a prognostic model. Using Lasso regression analysis, a four-gene (AFF3, GJB5, MET, and TMEM139) signature was identified after the verification of the training, internal validation sets, and external validation sets, which comprised a total of 491 patients from the TCGA*,* ICGC, and GEO PAAD datasets. The model translated the gene expression information into a risk score for the accurate estimation of prognosis in PAAD. Notably, the 3-year AUCs for the signature in all datasets were solid (higher than 0.70). When clinicopathologic parameters were taken into consideration, the constructed risk score system still independently predicted the prognosis of PAAD patients. A nomogram integrating the calculated risk score and clinical information constructed for the accurate prediction of survival probability of PAAD patients also showed confident clinical utility in PAAD.

Although there were 27 non-PAAD samples in the TCGA cohort, them are exactly pancreatic carcinoma. We also used those 146 PAAD samples for survival analysis, and observed that the high risk-score samples have a significantly worse prognosis, and the formula also showed similarly higher AUC in these PAAD samples. In addition, we had verified the good robustness of the 4-gene signature by two pancreatic ductal adenocarcinoma datasets from ICGC and GEO database. Therefore, we believe that the impact of these 27 samples on the remaining 146 samples is acceptable. This may also be the reason why other recent PAAD relayed prognostic models also did not excluded these 27 samples [22, 23].

Among the four genes, GJB5, MET, and TMEM139 were risk factors, whereas AFF3 was a protective factor for clinical outcomes in PAAD. The prognostic value of MET in PAAD has been reported in previous studies [40, 41]. MET is a well-recognized regulator in the progression of PAAD, and MET inhibitors have shown promising results in preclinical studies [42, 43]. However, the risk or protective value of the other three genes in PAAD have rarely been identified. Functional enrichment analysis revealed that this metabolism-related signature was significantly involved in some classical cancer-related pathways. The interaction between the four genes and tumor metabolism and progression in PAAD deserves further investigation.

Several previous studies have also identified specific prognostic models for the risk prediction of PAAD. For example, Chen et al. proposed a 15-gene signature that contained C6orf15, CAPN8, HIST1H3H, IGF2BP3, KIF14, KRT6A, PMAIP1, PPBP, RTKN2, SCEL, SERPINB5, SLC2A1, SLC45A3, TMPRSS3, and UCA1 [21]. Cheng et al. identified a biomarker consisting of 7 genes, including SCEL, SLC2A1, and SERPINB5, which were in Chen’s gene signature [22]. Raman et al. discovered another 5-gene signature based on the gene expression levels of ADM, ASPM, DCBLD2, E2F7, and KRT6A [23], which are distinct from the genes in the previous two modules. Magouliotis et al. discovered another gene signature containing 3 protein-coding RNAs and 4 microRNAs that was totally different from that of Jiang et al. [24]. The prognostic performance of the present model was further compared with that of the four previous models. Among the four different signatures, our four-gene biomarker had the highest AUC and C-index values. It could be concluded that these EMRGs outperform some previous biomarkers in the survival prediction of PAAD patients and have great potential to be used in clinical applications in the future.

However, there are still some limitations of this study. For example, the analysis was based on retrospective data and needs to be verified in a prospective cohort containing samples from multiple centers before clinical application. Deeper mechanistic research is also needed to elucidate the exact functions of the identified signature in PAAD.

## Conclusion

In summary, by analyzing the expression levels of EMRGs in PAAD tumor tissues, two different clusters with varied overall survival and immune status were identified in the TCGA PAAD dataset. A 4-gene prognostic signature and a novel nomogram were identified for the accurate risk prediction of PAAD patients.

## Supplementary Information

**Additional file 1.**

**Additional file 2.**

**Additional file 3.**

**Additional file 4.**

**Additional file 5.**

**Additional file 6.**

**Additional file 7.**

**Additional file 8.**

**Additional file 9.**

**Additional file 10.**

**Additional file 11.**

**Additional file 12.**

## Data Availability

The datasets generated and analyzed during the current study are available in the TCGA repository (https://portal.gdc.cancer.gov/), ICGC database (https://dcc.icgc.org/repositories), and GEO repository (GSE57495, https://www.ncbi.nlm.nih.gov/geo/query/acc.cgi?acc=GSE57495).

## Abbreviations

PAAD: Primary pancreatic adenocarcinoma
EMRG: Energy-metabolism-related gene
OS: Overall survival
WGCNA: Weighted gene correlation network analysis
ROC: Receiver operating characteristic
GSEA: Gene set enrichment analysis
TCGA: The Cancer Genome Atlas
GEO: Gene Expression Omnibus
ICGC: International Cancer Genome Consortium
MSigDB: Molecular Signature Database
IRB: Institutional review board
FUSCC: Fudan University Shanghai Cancer Center
NMF: Nonnegative matrix factorization
RSS: Residual sum of squares
FPKM: Fragments per kilobase per million
TOM: Topology overlap matrix
LASSO: Least absolute shrinkage and selection operator
DCA: Decision curve analysis
RMST: Restricted mean survival time
ES: Enrichment score
NES: Normalized enrichment score
FDR: False discovery rate
