# Supplementary material for: Molecular signatures of tumor progression in pancreatic adenocarcinoma identified by energy metabolism characteristics
Source: BMC Cancer. 2022 Apr 13;22:404. doi: 10.1186/s12885-022-09487-3 (PMC9006543; doi:10.1186/s12885-022-09487-3)

Supplementary Figure 1.
Flow chart of the analysis procedure: data collection, analysis, hub gene selection, and validation

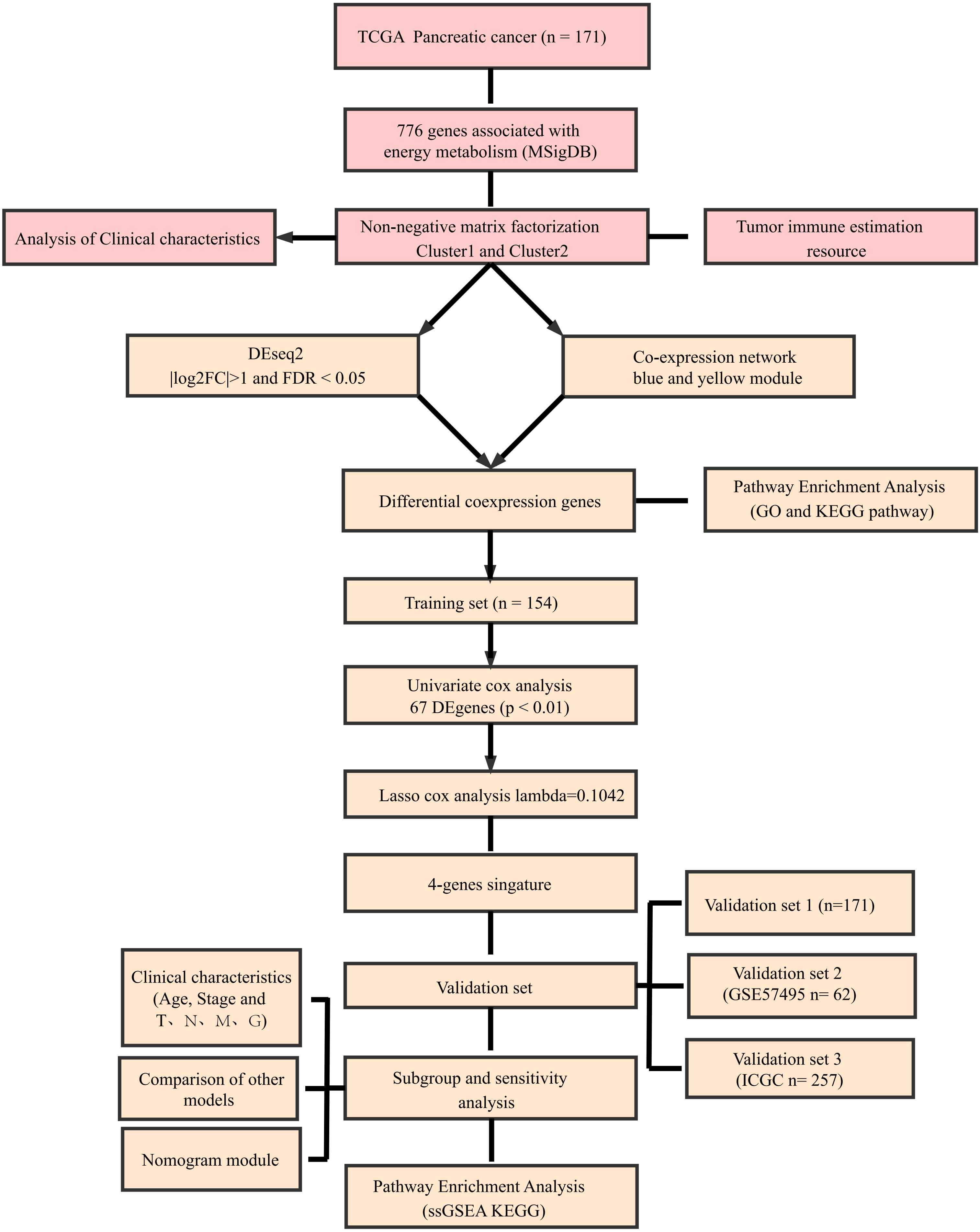

Supplement: Supplementary file 1 — Additional file 1. [file 12885_2022_9487_MOESM1_ESM.pdf]
