# Supplementary material for: Molecular signatures of tumor progression in pancreatic adenocarcinoma identified by energy metabolism characteristics
Source: BMC Cancer. 2022 Apr 13;22:404. doi: 10.1186/s12885-022-09487-3 (PMC9006543; doi:10.1186/s12885-022-09487-3)

Supplementary Figure 2.

A: NMF rank survey of cophenetic, RSS, and dispersion in area under rank=2–1 in the TCGA (A) and ICGC (B datasets; C: Distribution of clinicopathological parameters in the three subtypes.

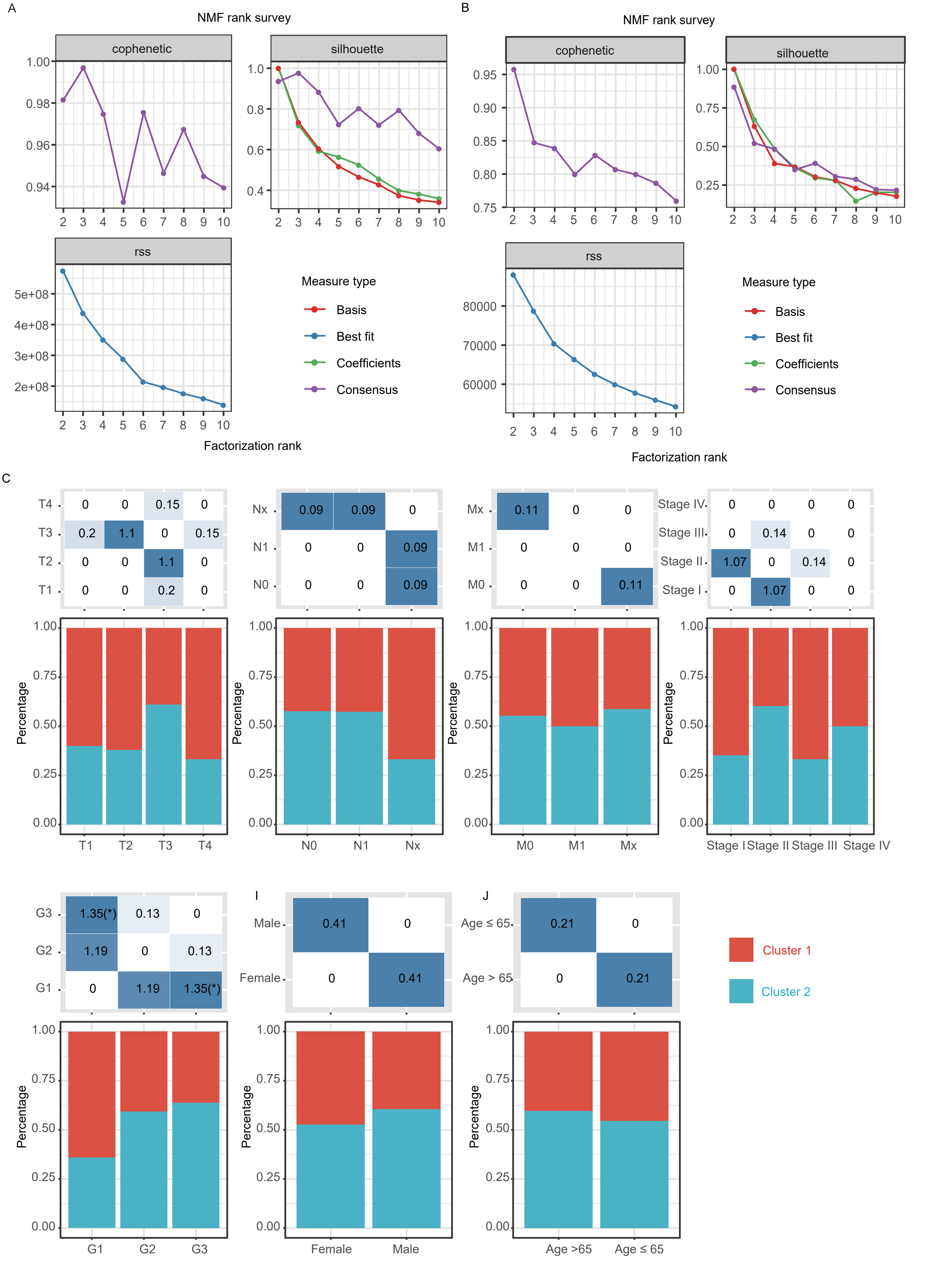

Supplement: Supplementary file 2 — Additional file 2. [file 12885_2022_9487_MOESM2_ESM.pdf]
