# Supplementary material for: Molecular signatures of tumor progression in pancreatic adenocarcinoma identified by energy metabolism characteristics
Source: BMC Cancer. 2022 Apr 13;22:404. doi: 10.1186/s12885-022-09487-3 (PMC9006543; doi:10.1186/s12885-022-09487-3)

Supplementary Figure 3.

A: Hierarchical clustering for identification of samples with outliers; B: Analysis of network topology for various soft-thresholding powers; C: Enriched top 20 KEGG pathways of coexpressed DEGs; D-F: Enriched top 20 gene ontology (GO) cellular component, molecular function, and biological process of coexpressed DEGs. The color from red to blue represents the significance of the P value; redder color represents a smaller P value. The dot size represents the number of genes enriched in the pathway, and a larger number represents a larger value.

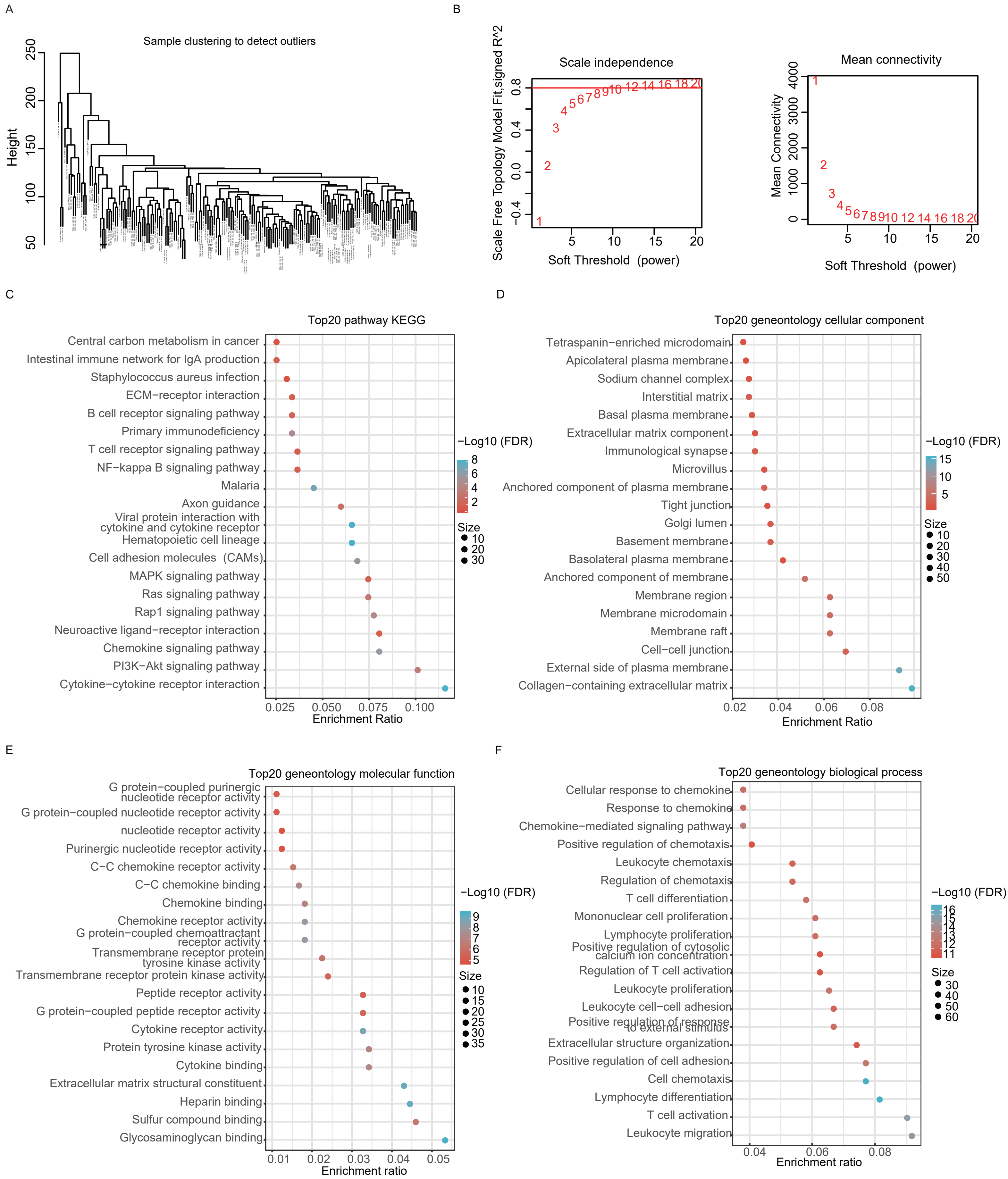

Supplement: Supplementary file 3 — Additional file 3. [file 12885_2022_9487_MOESM3_ESM.pdf]
