# Supplementary material for: Molecular signatures of tumor progression in pancreatic adenocarcinoma identified by energy metabolism characteristics
Source: BMC Cancer. 2022 Apr 13;22:404. doi: 10.1186/s12885-022-09487-3 (PMC9006543; doi:10.1186/s12885-022-09487-3)

Supplementary Figure 4.

A: Trajectory change of each independent variable. The X axis represents the log value of the independent variable lambda, and the Y axis represents the coefficient of the independent variable. B: Confidence intervals of each lambda. C. The difference in risk score between basal and classic samples in the TCGA PAAD cohort.

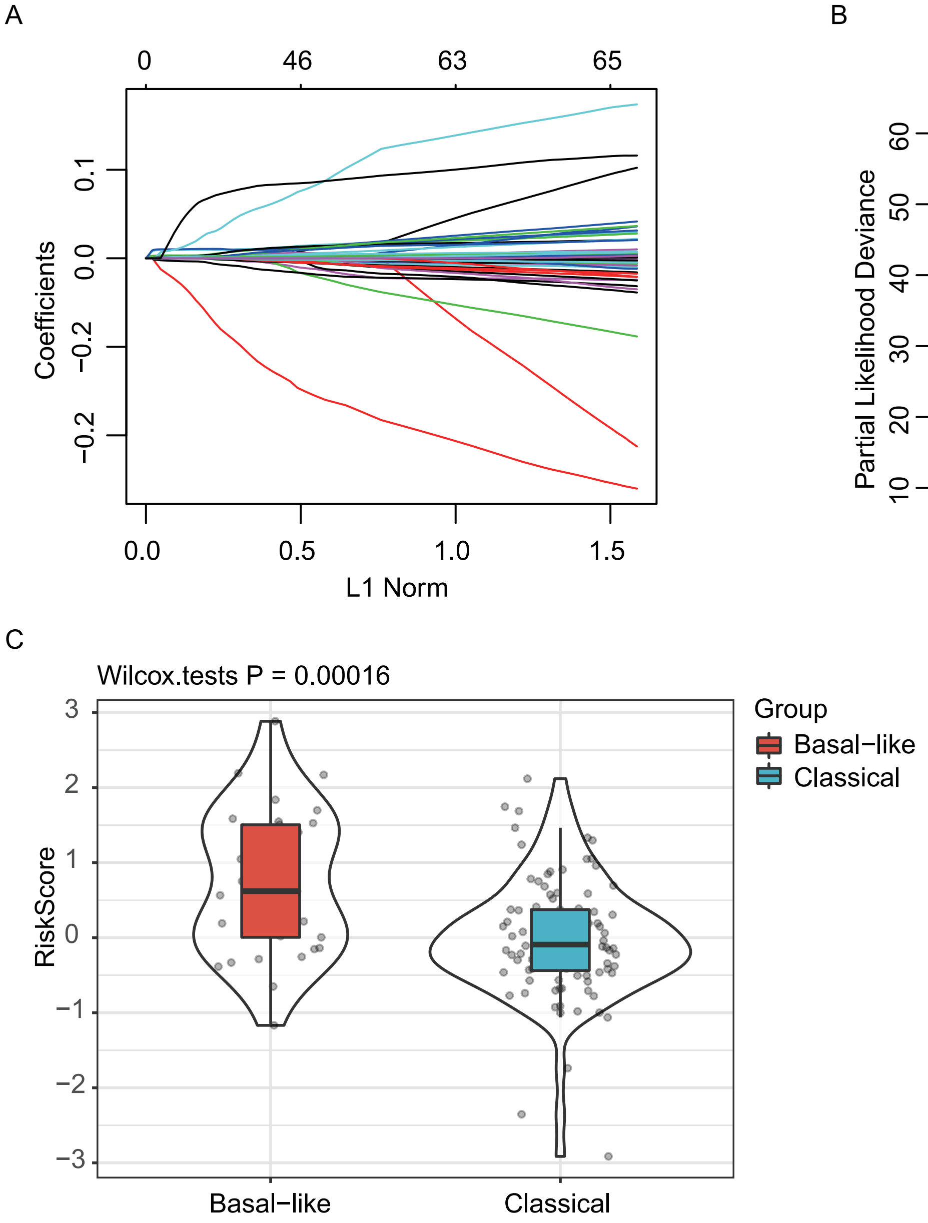

Supplement: Supplementary file 4 — Additional file 4. [file 12885_2022_9487_MOESM4_ESM.pdf]
