# Supplementary material for: Molecular signatures of tumor progression in pancreatic adenocarcinoma identified by energy metabolism characteristics
Source: BMC Cancer. 2022 Apr 13;22:404. doi: 10.1186/s12885-022-09487-3 (PMC9006543; doi:10.1186/s12885-022-09487-3)

Supplementary Figure 5.

A-D: Kaplan–Meier survival analysis of the gene signature of Chen et al. (A), Cheng et al. (B), Raman et al. (C), and Raman et al. (D); E-H: ROC curve (survival analysis of the gene signature of Chen et al. (E), Cheng et al. (F), Raman et al. (G), and Raman et al. (H).

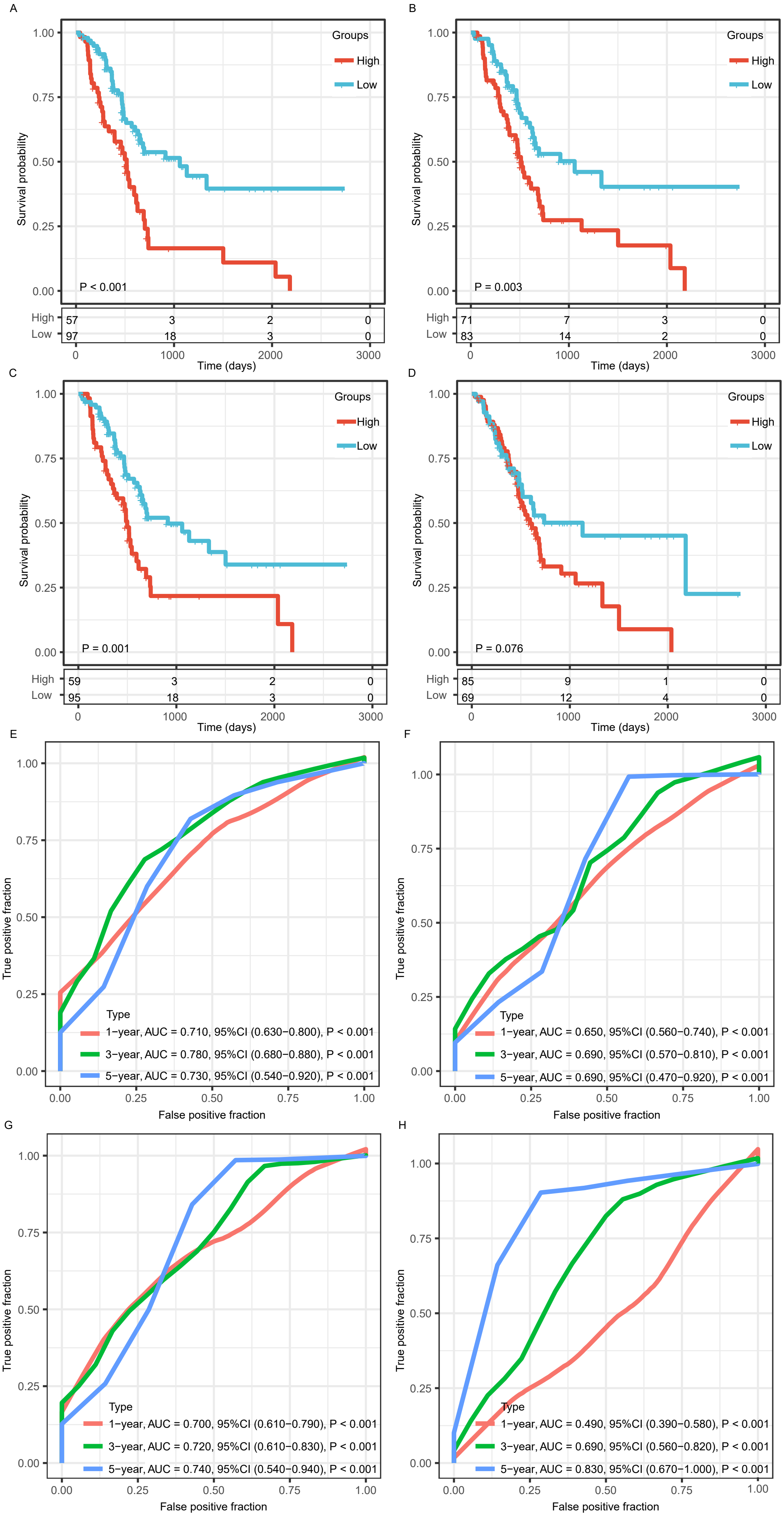

Supplement: Supplementary file 5 — Additional file 5. [file 12885_2022_9487_MOESM5_ESM.pdf]
