# Supplementary material for: Molecular signatures of tumor progression in pancreatic adenocarcinoma identified by energy metabolism characteristics
Source: BMC Cancer. 2022 Apr 13;22:404. doi: 10.1186/s12885-022-09487-3 (PMC9006543; doi:10.1186/s12885-022-09487-3)
Supplement: Supplementary file 6 — Additional file 6. [file 12885_2022_9487_MOESM6_ESM.pdf]

Supplementary Table 1. Pathways associated with energy metabolism in the Reactome pathway database

Metabolic pathways from Reactome	PathwayID	Gene Count
Biological oxidations	R-HSA-211859	216
Metabolism of carbohydrates	R-HSA-71387	290
Mitochondrial Fatty Acid Beta-Oxidation	R-HSA-77289	37
Glycogen synthesis	R-HSA-3322077	16
Glycogen metabolism	R-HSA-8982491	27
Glucose metabolism	R-HSA-70326	90
Glycogen breakdown (glycogenolysis)	R-HSA-70221	15
Glycolysis	R-HSA-70171	71
Pyruvate metabolism	R-HSA-70268	31
Pyruvate metabolism and Citric Acid (TCA) cycle	R-HSA-71406	55
Citric acid cycle (TCA cycle)	R-HSA-71403	22
Sum	871(unique:594)	
