# Supplementary material for: Molecular signatures of tumor progression in pancreatic adenocarcinoma identified by energy metabolism characteristics
Source: BMC Cancer. 2022 Apr 13;22:404. doi: 10.1186/s12885-022-09487-3 (PMC9006543; doi:10.1186/s12885-022-09487-3)
Supplement: Supplementary file 7 — Additional file 7. [file 12885_2022_9487_MOESM7_ESM.pdf]

Supplementary Table 2. The correlation of the module and genes in the module with phenotypes

Genes	GS.Gende	GS.Age	GS.T	GS.N	GS.M	GS.Stage	GS.Grade	GS.Cluster	GS.Cluster	p.GS.Genc	p.GS.Age	p.GS.T	p.GS.N	p.GS.M	p.GS.Stag(	p.GS.Grad	p.GS.Clust	p.GS.Clust	Module
RAB4B	0.0534	-0.08053	-0.05841	-0.10479	0.17753	-0.17461	-0.07425	-0.17885	0.17885	0.48791	0.29508	0.44793	0.17258	0.02018	0.02236	0.33448	0.01926	0.01926	red
TIGAR	0.00096	-0.0114	0.00583	0.05458	-0.02854	0.02281	-0.03224	0.1894	-0.1894	0.99002	0.88236	0.93969	0.47834	0.71096	0.76717	0.67552	0.0131	0.0131	turquoise
RNF44	0.03609	-0.12206	-0.02802	-0.01624	-0.03057	0.08137	-0.01736	-0.11595	0.11595	0.63933	0.11175	0.71603	0.83303	0.69139	0.29005	0.82167	0.13097	0.13097	turquoise
RPL23A	0.10585	0.07405	-0.00484	-0.12046	-0.00076	0.00169	0.07833	-0.18721	0.18721	0.16825	0.3358	0.94994	0.18238	0.99216	0.98253	0.30851	0.01421	0.01421	purple
ARL8B	0.00942	-0.12434	-0.00117	-0.02009	-0.1511	0.10413	0.09166	0.02924	-0.02924	0.90272	0.10517	0.98784	0.7942	0.04853	0.17531	0.23314	0.70421	0.70421	turquoise
CALB2	-0.11876	-0.00129	0.07309	0.0758	-0.08908	0.06843	0.11105	-0.22534	0.22534	0.12185	0.98661	0.34208	0.32442	0.24661	0.37383	0.14819	0.00304	0.00304	black
MFSD3	0.05209	0.10624	-0.15262	-0.14059	0.27385	-0.21953	0.04257	-0.36967	0.36967	0.49863	0.16668	0.04628	0.06665	0.00029	0.00392	0.58033	6.49E-07	6.49E-07	green
PIGV	-0.04127	-0.13787	0.0236	0.03609	-0.08095	0.12926	0.09871	-0.00322	0.00322	0.59197	0.07214	0.75935	0.63936	0.29256	0.092	0.199	0.96665	0.96665	turquoise
ZNF708	-0.00143	-0.13324	-0.13294	-0.03601	0.0585	-0.12345	-0.21875	0.36007	-0.36007	0.98516	0.08233	0.08302	0.64007	0.44726	0.1077	0.00405	1.32E-06	1.32E-06	turquoise
MOGAT2	0.00254	0.04138	0.04571	0.0076	0.23907	0.08056	0.08989	-0.27661	0.27661	0.97368	0.59102	0.5527	0.92141	0.00164	0.29488	0.24233	0.00025	0.00025	grey
PFN2	-0.05144	-0.16854	-0.17646	-0.03966	-0.11535	-0.22984	-0.07021	0.39636	-0.39636	0.504	0.02756	0.02096	0.60655	0.133	0.00249	0.36147	8.03E-08	8.03E-08	brown
RMND5A	0.08925	-0.08476	-0.00765	-0.05994	-0.03509	0.13669	-0.0083	-0.20672	0.20672	0.24572	0.27036	0.92093	0.43612	0.64869	0.07462	0.91424	0.00667	0.00667	turquoise
RAD23A	0.03956	-0.02305	-0.0541	-0.0255	0.00098	-0.04671	-0.02432	-0.20062	0.20062	0.60744	0.76472	0.48223	0.74058	0.98988	0.54405	0.75222	0.00851	0.00851	turquoise
SIN3B	0.00666	-0.20805	-0.09619	0.00142	0.0666	-0.11981	-0.09247	0.19749	-0.19749	0.93109	0.00632	0.21073	0.98529	0.38674	0.11857	0.22899	0.00962	0.00962	red
CTSA	0.01701	-0.08613	0.07922	0.08033	0.00998	-0.01374	0.06718	-0.12973	0.12973	0.82528	0.26266	0.30302	0.29627	0.89689	0.85842	0.38264	0.0908	0.0908	turquoise
IL32	-0.03377	0.08992	0.14288	0.14105	-0.18803	0.27935	0.19805	-0.20273	0.20273	0.66104	0.24218	0.06229	0.06574	0.01379	0.00022	0.00941	0.00783	0.00783	yellow
PTPN4	-0.02787	-0.09842	-0.04574	0.01423	-0.10071	0.02761	0.01349	0.2243	-0.2243	0.71751	0.20031	0.5525	0.85346	0.19	0.72	0.86102	0.00318	0.00318	turquoise
NUCB2	-0.01458	-0.05206	-0.09595	-0.00473	0.04629	-0.25515	-0.25863	0.39939	-0.39939	0.84993	0.49891	0.21187	0.95109	0.54775	0.00076	0.00064	6.26E-08	6.26E-08	yellow
TMEM143	0.00362	0.0499	-0.09218	-0.09484	0.12357	0.02565	-0.03208	-0.1316	0.1316	0.96248	0.5169	0.23049	0.21724	0.10736	0.73911	0.67704	0.08622	0.08622	red
DACH1	-0.03276	-0.05843	-0.11333	-0.09078	0.15899	-0.1413	-0.19278	0.30932	-0.30932	0.67054	0.44782	0.13998	0.23768	0.0378	0.06526	0.01153	3.84E-05	3.84E-05	brown
SERTAD2	-0.00368	-0.15301	0.06355	0.08988	-0.1771	0.16416	0.03902	0.07647	-0.07647	0.96189	0.04572	0.40895	0.24238	0.02049	0.03191	0.61235	0.32018	0.32018	turquoise
ST3GAL6	-0.28454	-0.11541	-0.09488	-0.08494	-0.04587	-0.06037	-0.05645	0.29437	-0.29437	0.00016	0.13281	0.21703	0.26933	0.55135	0.43283	0.46334	9.30E-05	9.30E-05	grey
PKP2	0.02701	0.03818	0.05683	0.0206	-0.08247	0.22022	0.10416	-0.20329	0.20329	0.72581	0.62001	0.46036	0.78909	0.28355	0.0038	0.17516	0.00766	0.00766	turquoise
SLC45A4	0.07647	0.03915	-0.01715	-0.04931	0.0624	-0.05217	0.03367	-0.30338	0.30338	0.32015	0.6112	0.82379	0.52189	0.41746	0.49798	0.66197	5.49E-05	5.49E-05	grey
MTRF1	0.02345	-0.05669	0.0554	0.00544	0.10164	0.04863	-0.00123	-0.12458	0.12458	0.76084	0.46145	0.47169	0.94372	0.18592	0.52765	0.98725	0.10448	0.10448	red
ESM1	-0.07438	-0.17874	-0.047	0.01429	0.01116	-0.12077	-0.10558	-0.01571	0.01571	0.3336	0.01933	0.54154	0.85286	0.88479	0.1156	0.16932	0.83843	0.83843	grey
RWDD2A	-0.08772	-0.11938	-0.20292	-0.02849	0.04276	-0.24398	-0.14065	0.51399	-0.51399	0.25394	0.11989	0.00777	0.71142	0.57864	0.0013	0.06652	6.48E-13	6.48E-13	brown
SCFD2	-0.04073	0.01048	-0.09881	#######	-0.07402	-0.02865	0.07037	0.18591	-0.18591	0.59687	0.89182	0.19852	0.99899	0.33598	0.70991	0.36037	0.01491	0.01491	turquoise
YWHAB	0.02985	-0.02169	0.02892	0.00971	-0.11583	0.06981	0.12073	-0.06431	0.06431	0.69836	0.77829	0.70725	0.89973	0.13138	0.36422	0.11575	0.40336	0.40336	turquoise
TOMM40	0.06474	0.0423	-0.08633	-0.07157	0.04323	-0.09211	0.14763	-0.30909	0.30909	0.40022	0.58278	0.26157	0.35225	0.57451	0.23086	0.05398	3.90E-05	3.90E-05	grey
ADTRP	-0.04771	-0.01211	0.14813	0.07767	-0.07028	0.2437	0.05633	-0.1554	0.1554	0.53551	0.87508	0.05317	0.3126	0.36103	0.00132	0.46431	0.04241	0.04241	grey
NDUFAF2	-0.03074	0.00798	-0.07775	-0.08023	0.02181	-0.13113	-0.00883	0.02317	-0.02317	0.68978	0.91751	0.31212	0.29687	0.77703	0.08734	0.90877	0.76357	0.76357	grey
SEPSECS	-0.03013	-0.05747	-0.08296	-0.04107	0.13546	-0.0107	-0.09022	0.00687	-0.00687	0.69567	0.45531	0.28072	0.59375	0.07731	0.88952	0.24058	0.92895	0.92895	turquoise
CWC27	0.00522	0.00575	-0.01883	-0.03828	-0.00026	-0.02632	0.07239	-0.02036	0.02036	0.946	0.94055	0.80691	0.61913	0.99731	0.73256	0.34672	0.79154	0.79154	turquoise
MYOM2	-0.04012	-0.0918	-0.1825	0.00069	0.01619	-0.10711	-0.05571	0.14812	-0.14812	0.60241	0.23243	0.01689	0.99281	0.83349	0.16321	0.46926	0.05319	0.05319	grey
MAP9	-0.06537	-0.2008	-0.16579	-0.03975	-0.07103	-0.16978	-0.14911	0.45241	-0.45241	0.3956	0.00845	0.03023	0.60568	0.35594	0.02642	0.0516	5.23E-10	5.23E-10	turquoise
APIP	0.03859	-0.13393	-0.0653	-0.07462	0.06277	-0.11237	-0.07228	0.07171	-0.07171	0.61631	0.08074	0.39616	0.33204	0.41472	0.14339	0.34748	0.35131	0.35131	turquoise
MAF1	0.06166	-0.03409	-0.06241	-0.00079	-0.09699	-0.08226	0.11452	-0.22111	0.22111	0.42304	0.65801	0.41739	0.99181	0.20696	0.28479	0.13584	0.00366	0.00366	green
FOXP1	-0.02309	0.01888	0.03779	0.04751	-0.09517	0.17979	0.0459	0.037	-0.037	0.76432	0.80642	0.62362	0.53722	0.21567	0.01862	0.55111	0.63087	0.63087	turquoise
PLCE1	-0.03737	-0.15553	0.09859	0.11966	0.00012	0.05389	-0.0375	0.16166	-0.16166	0.62748	0.04223	0.19954	0.11901	0.99873	0.48391	0.62628	0.03465	0.03465	grey
RAD17	-0.03939	-0.11152	-0.13266	-0.06221	0.03718	-0.19734	-0.13294	0.24426	-0.24426	0.60902	0.14645	0.08369	0.41894	0.62921	0.00968	0.08304	0.00128	0.00128	turquoise
SPCS2	-0.01798	-0.1371	-0.03995	0.06817	-0.0627	-0.10966	-0.02542	0.01923	-0.01923	0.81548	0.07376	0.60388	0.37563	0.41524	0.15336	0.74135	0.80289	0.80289	turquoise
SETDB2	-0.05157	-0.04648	-0.0626	-0.00168	-0.04657	-0.05098	-0.0938	0.40722	-0.40722	0.50298	0.54605	0.41598	0.98256	0.54526	0.50789	0.22237	3.25E-08	3.25E-08	blue
DESI2	-0.02618	-0.07548	0.04433	0.04207	-0.15426	0.17499	0.14661	0.07198	-0.07198	0.73389	0.32653	0.56478	0.58484	0.04396	0.02207	0.05569	0.34946	0.34946	turquoise
COX17	0.12653	-0.04499	-0.23465	-0.2212	0.12516	-0.19632	-0.01694	-0.30922	0.30922	0.09912	0.55905	0.00201	0.00364	0.10287	0.01007	0.82594	3.87E-05	3.87E-05	grey
NLGN2	-0.05025	-0.22616	-0.04401	0.04047	-0.12484	-0.10458	0.00609	0.41136	-0.41136	0.51397	0.00294	0.56761	0.59922	0.10376	0.17341	0.93694	2.28E-08	2.28E-08	blue
PKMYT1	-0.00537	0.12403	0.03914	-0.01387	0.05256	0.03874	0.3144	-0.50485	0.50485	0.94443	0.10603	0.61123	0.85709	0.49478	0.61488	2.82E-05	1.91E-12	1.91E-12	pink
FAM89B	-0.00401	-0.03033	0.02167	0.05824	-0.18341	0.11854	0.13382	-0.16266	0.16266	0.9585	0.69373	0.7785	0.44924	0.01634	0.12254	0.081	0.03354	0.03354	black
MED8	-0.02875	-0.09867	0.02124	-0.06228	-0.07199	0.09761	0.08297	-0.03622	0.03622	0.7089	0.19917	0.7827	0.41838	0.34943	0.20406	0.28066	0.6381	0.6381	turquoise
PHF1	0.06143	-0.09842	-0.07902	-0.07522	0.06909	-0.11127	-0.10018	0.05639	-0.05639	0.42479	0.20032	0.30427	0.32817	0.36923	0.14738	0.19234	0.46385	0.46385	grey
ZNF781	-0.06178	-0.09887	-0.12648	0.0364	0.05849	-0.20715	-0.20155	0.59127	-0.59127	0.42216	0.19825	0.09927	0.63649	0.44732	0.00656	0.00821	1.68E-17	1.68E-17	brown
DIPK1A	-0.09326	-0.11202	0.10604	0.06812	-0.10032	0.14306	-0.00226	0.30397	-0.30397	0.22502	0.14463	0.16747	0.37598	0.19169	0.06196	0.9766	5.30E-05	5.30E-05	blue
ANKMY1	0.14136	0.04709	0.01378	-0.04035	0.06213	0.15255	0.14023	-0.25251	0.25251	0.06514	0.54078	0.85807	0.6003	0.41951	0.04638	0.06735	0.00086	0.00086	red
PSMB6	-0.00216	-0.01169	-0.13906	-0.08704	0.07069	-0.31205	0.02588	-0.01128	0.01128	0.97761	0.87943	0.0697	0.25761	0.35821	3.25E-05	0.73683	0.88354	0.88354	grey
GUCY1A1	-0.11735	-0.10128	-0.03266	0.10771	-0.14719	0.03585	0.01977	0.50956	-0.50956	0.12636	0.18746	0.67156	0.16085	0.05472	0.6416	0.7974	1.10E-12	1.10E-12	blue
PRKG2	0.0251	-0.11305	-0.05915	-0.02559	0.02891	-0.11858	-0.07927	-0.0145	0.0145	0.74455	0.14095	0.44219	0.73973	0.70736	0.1224	0.30273	0.85071	0.85071	grey
FBXO45	0.00429	-0.07781	-0.10966	-0.08719	-0.16466	0.04559	0.16162	-0.02674	0.02674	0.95563	0.31176	0.15336	0.25683	0.03138	0.55376	0.0347	0.72849	0.72849	turquoise
AMZ2	-0.07498	-0.14527	-0.12762	-0.05494	-0.02528	-0.17907	-0.04295	0.19458	-0.19458	0.32971	0.05798	0.09622	0.47537	0.7428	0.01911	0.57703	0.01076	0.01076	turquoise
TMEM52	-0.05863	0.16018	0.08343	0.13818	-0.09825	0.16221	0.02316	-0.03114	0.03114	0.4462	0.03637	0.27799	0.07148	0.20108	0.03404	0.76363	0.68596	0.68596	magenta
CHN2	0.10949	-0.03862	0.01632	-0.01203	0.0151	0.17446	-0.08303	0.1218	-0.1218	0.154	0.61605	0.83221	0.87593	0.84461	0.02248	0.2803	0.11252	0.11252	grey
GCNT4	-0.03824	-0.17585	-0.14285	0.03534	0.01216	-0.03983	-0.12169	0.34697	-0.34697	0.61946	0.02141	0.06234	0.64628	0.87459	0.60499	0.11284	3.33E-06	3.33E-06	turquoise
KLK8	0.04212	0.02185	0.04888	0.01746	0.022	0.08316	0.15086	-0.19543	0.19543	0.58437	0.77671	0.5255	0.82071	0.77514	0.27953	0.04888	0.01042	0.01042	grey
MTF2	-0.12156	-0.16828	0.00194	0.04682	-0.03909	0.03052	0.0031	0.03238	-0.03238	0.11322	0.0278	0.97995	0.54312	0.61174	0.69186	0.96793	0.67421	0.67421	turquoise
RBM15B	-0.11399	-0.0732	-0.09619	-0.05708	-0.11466	0.04628	0.02846	0.09231	-0.09231	0.13766	0.34138	0.21073	0.4584	0.13536	0.54776	0.71178	0.2298	0.2298	turquoise
PIH1D2	0.00562	-0.06585	-0.26364	-0.10471	0.21093	-0.23189	-0.01401	-0.09405	0.09405	0.94183	0.39218	0.00049	0.17288	0.00562	0.00227	0.85569	0.22111	0.22111	grey
MT1A	-0.06557	-0.02004	-0.02436	0.11076	-0.04606	0.04139	-0.02767	0.19742	-0.19742	0.39419	0.79475	0.75184	0.14925	0.54967	0.59094	0.71943	0.00965	0.00965	grey
NCAPG	-0.10335	0.08175	0.05057	-0.02322	-0.07287	0.08173	0.31139	-0.34136	0.34136	0.17857	0.28782	0.51126	0.76302	0.34354	0.28793	3.39E-05	4.90E-06	4.90E-06	pink
FANK1	0.10202	-0.17435	-0.05677	-0.03896	0.09165	-0.02229	0.05954	-0.36714	0.36714	0.18427	0.02257	0.46082	0.6129	0.23318	0.77233	0.43923	7.84E-07	7.84E-07	grey
NOP2	0.02347	-0.00551	0.00252	0.00447	0.05266	-0.00979	0.02311	-0.13296	0.13296	0.76058	0.94296	0.97386	0.95376	0.49392	0.89884	0.76417	0.08299	0.08299	grey
ZNF880	-0.07856	0.02881	0.08264	0.01577	-0.11491	0.20449	-0.01152	0.1915	-0.1915	0.30708	0.70836	0.28258	0.83783	0.1345	0.0073	0.88118	0.0121	0.0121	grey
MELTF	0.09823	0.0108	0.02657	0.013	-0.03362	0.08442	0.22768	-0.39479	0.39479	0.20119	0.88853	0.73008	0.866	0.66247	0.27229	0.00274	9.12E-08	9.12E-08	yellow
PTPRB	-0.05378	-0.20875	-0.10652	0.04255	0.01011	-0.0062	-0.19415	0.36692	-0.36692	0.48476	0.00614	0.16554	0.58053	0.89556	0.9359	0.01094	7.97E-07	7.97E-07	turquoise
DGKD	-0.08665	-0.00192	0.0226	-0.00955	-0.04469	0.07371	-0.02308	0.05095	-0.05095	0.25978	0.98008	0.76922	0.90133	0.56168	0.33798	0.76445	0.50813	0.50813	grey
TMCO1	-0.00894	-0.09522	-0.09561	-0.10477	0.06247	-0.01085	0.05848	-0.187	0.187	0.9076	0.21542	0.21352	0.17264	0.41696	0.88795	0.44742	0.01432	0.01432	turquoise
RAB5C	-0.02928	-0.0964	-0.01581	-0.00095	-0.02682	0.02203	0.04897	0.04164	-0.04164	0.70381	0.20977	0.83742	0.99021	0.72768	0.77491	0.52474	0.58865	0.58865	turquoise
UPP1	0.02015	-0.14767	0.0538	0.08309	-0.0015	0.09405	0.11786	-0.20692	0.20692	0.79364	0.05393	0.4846	0.27993	0.98444	0.2211	0.1247	0.00662	0.00662	grey
TRABD2B	-0.0043	-0.08274	-0.06507	0.06436	-0.14381	0.1255	0.00706	0.31614	-0.31614	0.95549	0.28199	0.39778	0.40297	0.06058	0.10194	0.92698	2.53E-05	2.53E-05	blue
AMN	0.11692	0.08019	0.10749	0.08844	0.23312	0.1897	0.17356	-0.44622	0.44622	0.12778	0.29716	0.16172	0.25003	0.00215	0.01295	0.02319	9.55E-10	9.55E-10	yellow
KLK10	-0.01132	0.15462	0.03894	-0.00232	-0.02807	0.14627	0.20365	-0.28995	0.28995	0.88322	0.04347	0.61305	0.97596	0.71549	0.05627	0.00755	0.00012	0.00012	yellow
KDM5B	0.05204	-0.14545	0.02465	0.02908	-0.10894	0.19204	0.14651	-0.17978	0.17978	0.4991	0.05767	0.74891	0.70574	0.1561	0.01186	0.05586	0.01863	0.01863	turquoise
STPG1	-0.00956	-0.12834	-0.07402	-0.0052	-0.0519	0.038	-0.00786	0.01093	-0.01093	0.9012	0.09434	0.33596	0.9462	0.50022	0.62166	0.91875	0.88715	0.88715	turquoise
FHOD1	-0.03892	-0.06472	0.03346	0.0839	-0.00462	0.11927	0.19673	-0.00506	0.00506	0.6133	0.40035	0.66393	0.27529	0.95215	0.12024	0.00991	0.94768	0.94768	grey
PTPRH	0.17273	-0.00595	0.08573	0.02983	0.11302	0.15391	0.06554	-0.39305	0.39305	0.02387	0.93848	0.26489	0.6985	0.14106	0.04444	0.39442	1.05E-07	1.05E-07	yellow
PCMTD1	-0.06358	-0.0313	0.04121	0.05384	-0.09804	0.05308	-0.05012	0.13483	-0.13483	0.40874	0.68443	0.59251	0.48429	0.20206	0.49047	0.51502	0.07872	0.07872	turquoise
MAP2K7	0.05304	-0.03123	-0.17178	-0.06693	0.15228	-0.15859	0.04261	-0.07391	0.07391	0.49082	0.68515	0.02466	0.3844	0.04677	0.03828	0.57999	0.33668	0.33668	green
SMIM32	0.03292	-0.12319	-0.21159	-0.16723	0.29334	-0.27713	-0.19862	0.1646	-0.1646	0.66906	0.10845	0.00547	0.0288	9.87E-05	0.00024	0.00921	0.03145	0.03145	brown
AP5S1	0.01612	0.01146	-0.19201	-0.07457	0.08799	-0.27368	-0.07352	0.224	-0.224	0.83424	0.88171	0.01187	0.33237	0.25247	0.00029	0.33923	0.00323	0.00323	grey
GRIP1	0.04882	-0.19554	-0.0774	0.11915	0.0087	-0.0637	-0.05307	0.22581	-0.22581	0.52599	0.01038	0.31429	0.12062	0.91013	0.40784	0.49058	0.00298	0.00298	turquoise
VRK3	0.16197	0.00558	-0.07253	-0.05478	0.07254	-0.00367	0.0688	-0.2221	0.2221	0.0343	0.94222	0.34579	0.47668	0.34575	0.96197	0.37124	0.0035	0.0035	grey
SLC2A1	0.01677	-0.07432	0.10655	0.06064	-0.02853	0.1738	0.13479	-0.46176	0.46176	0.82763	0.33398	0.16544	0.43079	0.71104	0.023	0.0788	2.06E-10	2.06E-10	yellow
SPINK1	-0.04156	0.01155	0.04517	0.08779	0.07474	0.18452	-0.14202	-0.07966	0.07966	0.58936	0.88077	0.5574	0.25354	0.33126	0.01569	0.06389	0.30037	0.30037	magenta
GLYCTK	-0.00725	0.06747	-0.09754	-0.12012	0.37819	-0.14131	-0.12688	-0.07517	0.07517	0.92497	0.38059	0.20439	0.1176	3.40E-07	0.06524	0.09819	0.32848	0.32848	grey
TNNT1	0.01438	-0.00524	0.02924	0.02332	-0.08619	0.03997	0.1775	-0.39466	0.39466	0.85194	0.94577	0.7042	0.76204	0.26232	0.6037	0.0202	9.22E-08	9.22E-08	grey
MT-ND1	-0.0169	-0.10541	-0.06945	-0.01071	0.21285	-0.08926	-0.13284	-0.15921	0.15921	0.82634	0.17003	0.36674	0.8894	0.00519	0.24567	0.08327	0.03753	0.03753	grey
PPOX	0.03704	-0.03287	-0.12439	-0.14246	0.20538	-0.10924	-0.03237	-0.18287	0.18287	0.63052	0.66956	0.10501	0.06307	0.00704	0.15494	0.67429	0.01667	0.01667	red
H6PD	-0.05514	-0.06736	0.01032	0.10043	-0.19002	0.21756	0.10556	0.16194	-0.16194	0.47383	0.38134	0.89347	0.19122	0.0128	0.00426	0.16943	0.03434	0.03434	turquoise
RCOR1	0.08324	-0.10848	0.04714	0.04188	0.06211	0.05643	0.06108	-0.20847	0.20847	0.27909	0.15784	0.54037	0.58654	0.41962	0.46348	0.42745	0.00621	0.00621	turquoise
GRAMD1(	0.00106	-0.15604	-0.06541	0.06408	0.0308	-0.00518	-0.04578	-0.05522	0.05522	0.98906	0.04155	0.39536	0.40504	0.68922	0.94637	0.55213	0.47319	0.47319	grey
RPS6KB1	-0.09074	-0.19178	-0.08353	-0.0024	-0.09531	-0.04967	0.01937	0.17845	-0.17845	0.23786	0.01198	0.27737	0.97519	0.21495	0.5188	0.80145	0.01954	0.01954	turquoise
GLI3	-0.10092	-0.0828	0.01846	0.05118	-0.20779	0.04992	0.08882	0.38862	-0.38862	0.18904	0.28164	0.81062	0.50621	0.00639	0.5167	0.24799	1.50E-07	1.50E-07	blue
KCNE4	0.0022	-0.11501	0.04184	0.06861	-0.17258	0.07134	-0.07271	0.47065	-0.47065	0.97718	0.13417	0.58691	0.37259	0.024	0.35378	0.34461	8.26E-11	8.26E-11	blue
FAM217B	-0.03028	-0.15487	-0.05718	-0.02163	-0.18384	-0.09938	0.01466	0.20878	-0.20878	0.69425	0.04312	0.45757	0.7789	0.01609	0.19592	0.84907	0.00614	0.00614	turquoise
FCGR2B	-0.13268	-0.03012	0.11395	0.08074	-0.23507	0.19921	0.09575	0.29402	-0.29402	0.08364	0.69574	0.13779	0.29381	0.00197	0.009	0.21283	9.49E-05	9.49E-05	blue
LAMTOR4	0.12084	-0.00601	-0.08083	-0.08402	0.09702	-0.11567	0.02572	-0.18239	0.18239	0.11541	0.93786	0.2933	0.27461	0.20682	0.13191	0.73848	0.01696	0.01696	grey
PHYKPL	-0.04602	-0.12757	0.03076	0.0212	0.08602	0.05324	0.02947	-0.03611	0.03611	0.55007	0.09637	0.68962	0.78311	0.26327	0.48922	0.70204	0.63914	0.63914	red
KIAA1328	-0.01497	-0.15653	-0.09799	0.02893	-0.1044	-0.07309	-0.06307	0.45075	-0.45075	0.84592	0.0409	0.2023	0.70721	0.17416	0.34209	0.41249	6.15E-10	6.15E-10	turquoise
NME1-NN	-0.10765	0.11095	-0.00465	0.04611	-0.00173	-0.03375	0.0937	-0.21679	0.21679	0.16108	0.14853	0.95187	0.54923	0.9821	0.6612	0.22285	0.0044	0.0044	grey
PHLDA2	0.07559	0.00675	0.06362	-0.03669	0.05889	0.22465	0.2324	-0.52606	0.52606	0.32578	0.93014	0.40842	0.63375	0.44424	0.00314	0.00222	1.48E-13	1.48E-13	yellow
CBL	-0.05288	-0.15991	0.02708	0.02832	-0.23003	0.08041	0.06287	0.20224	-0.20224	0.49212	0.03669	0.72511	0.71313	0.00247	0.29579	0.41396	0.00799	0.00799	turquoise
CLN6	0.00445	0.10616	-0.10867	-0.0516	0.08484	-0.08934	0.12033	-0.3182	0.3182	0.95391	0.16697	0.15713	0.50272	0.26991	0.24522	0.11696	2.22E-05	2.22E-05	grey
EFS	-0.03078	-0.08512	0.00693	0.05577	-0.16512	0.08989	0.0808	0.34622	-0.34622	0.68945	0.26832	0.92832	0.46874	0.03091	0.24229	0.29343	3.51E-06	3.51E-06	blue
ERRFI1	-0.10206	-0.25358	-0.09974	0.04572	-0.07018	-0.09819	-0.075	-0.03121	0.03121	0.18409	0.00082	0.19432	0.55269	0.36174	0.20138	0.32959	0.68534	0.68534	grey
EVPL	0.04623	0.01989	0.1027	0.04247	0.00704	0.22984	0.24529	-0.47054	0.47054	0.54821	0.79622	0.18134	0.58126	0.92714	0.00249	0.00122	8.35E-11	8.35E-11	yellow
IL27RA	-0.10291	-0.13249	0.11588	0.16381	-0.13194	0.15752	0.0971	0.21626	-0.21626	0.18044	0.08408	0.13123	0.03228	0.08538	0.03962	0.20646	0.0045	0.0045	blue
WDR70	-0.00257	0.02972	-0.03308	-0.02258	-0.13224	-0.02422	0.23813	0.05096	-0.05096	0.97334	0.6996	0.66752	0.76945	0.08468	0.75318	0.00171	0.50797	0.50797	grey
OTUD1	0.05016	-0.12288	0.07352	0.06256	-0.19739	0.13506	0.02586	0.12002	-0.12002	0.51472	0.10933	0.33927	0.41628	0.00966	0.07819	0.73704	0.1179	0.1179	turquoise
TTC5	-0.04689	-0.12815	-0.10443	-0.00811	-0.02345	-0.08824	-0.08731	0.19225	-0.19225	0.5425	0.09485	0.17406	0.91616	0.76082	0.25113	0.25615	0.01177	0.01177	turquoise
PDCD6	0.07841	-0.06937	-0.11145	-0.15223	0.1541	-0.1039	0.13866	-0.33848	0.33848	0.30804	0.3673	0.14672	0.04684	0.04417	0.17626	0.07049	5.95E-06	5.95E-06	grey
MRPS11	-0.05753	-0.03828	-0.22483	-0.09541	0.12705	-0.23952	-0.04369	-0.00212	0.00212	0.45483	0.6191	0.00311	0.21448	0.09775	0.0016	0.57045	0.97802	0.97802	grey
EPB41L2	-0.10292	-0.10774	0.00651	0.01237	-0.14377	0.15218	-0.05044	0.46313	-0.46313	0.1804	0.16073	0.93268	0.87238	0.06065	0.04692	0.51232	1.79E-10	1.79E-10	blue
EPHA3	-0.04582	-0.12817	-0.03055	0.03478	-0.17541	0.16004	0.01767	0.43546	-0.43546	0.55181	0.09479	0.69164	0.65152	0.02175	0.03654	0.81854	2.64E-09	2.64E-09	blue
C1orf35	0.09968	0.02878	-0.15488	-0.12342	0.21605	-0.10877	0.12448	-0.46208	0.46208	0.19457	0.70867	0.0431	0.10777	0.00454	0.15674	0.10478	1.99E-10	1.99E-10	green
LDHA	-0.04167	-0.08228	0.08311	0.07833	-0.10437	0.2289	0.21284	-0.36447	0.36447	0.58843	0.28465	0.2798	0.3085	0.17429	0.0026	0.00519	9.55E-07	9.55E-07	turquoise
XCL1	-0.04744	-0.07116	0.04626	0.03546	-0.1182	0.14068	0.10923	0.19937	-0.19937	0.53778	0.35501	0.548	0.64519	0.12361	0.06647	0.155	0.00894	0.00894	grey
UBE3A	-0.07911	-0.142	-0.07792	-0.02996	-0.08833	0.00391	-0.02874	0.16564	-0.16564	0.30372	0.06394	0.31109	0.69731	0.2506	0.95955	0.70903	0.03038	0.03038	turquoise
DNMT3A	-0.0452	-0.18608	-0.17935	-0.00545	0.00671	-0.11271	0.02522	0.16981	-0.16981	0.55722	0.01482	0.01892	0.94365	0.93054	0.14217	0.7433	0.02639	0.02639	red
PDE5A	-0.12006	-0.18127	-0.06831	0.06315	-0.08701	0.07174	-0.10863	0.38071	-0.38071	0.11779	0.01766	0.37465	0.41191	0.25781	0.35111	0.15726	2.80E-07	2.80E-07	turquoise

ZNF304	-0.11057	-0.19765	-0.14342	0.00986	-0.12347	-0.0488	-0.05982	0.35371	-0.35371	0.14994	0.00956	0.0613	0.89818	0.10763	0.52617	0.43704	2.08E-06	2.08E-06	turquoise
ATP6AP2	-0.09299	-0.18805	-0.12059	0.02424	-0.0094	-0.18993	-0.08046	0.25274	-0.25274	0.2264	0.01378	0.11617	0.75296	0.90287	0.01284	0.29551	0.00085	0.00085	turquoise
ATP23	0.09934	-0.01734	-0.02634	0.06857	0.05873	-0.11327	0.02841	-0.09994	0.09994	0.1961	0.82185	0.73241	0.37287	0.44548	0.14021	0.71226	0.19343	0.19343	grey
PLIN4	0.00934	0.01964	0.00258	0.16066	0.01601	0.04259	-0.06919	0.34621	-0.34621	0.90352	0.79871	0.97333	0.03581	0.83534	0.58022	0.36856	3.51E-06	3.51E-06	grey
PLP2	0.01734	-0.09831	0.07484	0.06214	-0.08264	0.04974	0.23107	-0.49373	0.49373	0.82186	0.20083	0.33063	0.41945	0.28254	0.51825	0.00236	6.80E-12	6.80E-12	pink
TREH	-0.02941	-0.08106	-0.04672	0.08308	0.19138	0.03794	-0.01366	0.0971	-0.0971	0.70261	0.29193	0.54402	0.28003	0.01216	0.62226	0.85921	0.20645	0.20645	grey
NOP16	-0.01604	0.05866	0.01606	-0.03855	-0.04055	0.11086	0.14797	-0.32304	0.32304	0.83502	0.44603	0.83481	0.61667	0.59844	0.14886	0.05343	1.64E-05	1.64E-05	grey
GNA12	-0.10819	-0.18123	-0.03796	0.01106	-0.08431	-0.13268	-0.06435	0.41647	-0.41647	0.15896	0.01768	0.62204	0.88581	0.27294	0.08364	0.40305	1.46E-08	1.46E-08	turquoise
HEY2	-0.08666	-0.02978	-0.17413	-0.03613	-0.0308	-0.147	-0.10804	0.31594	-0.31594	0.25975	0.69897	0.02274	0.63894	0.68919	0.05504	0.15955	2.56E-05	2.56E-05	grey
ACOX2	-0.09738	-0.04985	-0.0653	-0.00587	0.01546	0.01036	-0.19667	0.41452	-0.41452	0.20512	0.51729	0.39612	0.93922	0.84098	0.89303	0.00993	1.74E-08	1.74E-08	turquoise
INTS13	0.02601	-0.03583	0.04894	0.08143	-0.1323	0.03076	0.1291	-0.10997	0.10997	0.7356	0.64179	0.52497	0.2897	0.08454	0.68965	0.0924	0.15217	0.15217	turquoise
PROSER2	0.02626	-0.09208	-0.00564	0.04995	-0.21016	0.13148	0.22142	-0.33327	0.33327	0.73312	0.23098	0.94163	0.5165	0.0058	0.08649	0.00361	8.42E-06	8.42E-06	yellow
PPP1R7	0.10543	0.03672	-0.05465	-0.02604	-0.01722	0.00676	0.15023	-0.25847	0.25847	0.16993	0.63347	0.47772	0.73531	0.82307	0.93004	0.04985	0.00064	0.00064	greenyellow
CYP2E1	-0.0025	-0.01912	-0.01029	-0.12917	0.03899	-0.02502	-0.01811	-0.06592	0.06592	0.97412	0.80398	0.89375	0.09221	0.61261	0.74535	0.81418	0.39163	0.39163	grey
PES1	-0.05824	0.09421	-0.13641	-0.0186	-0.06025	-0.07358	0.16653	-0.14688	0.14688	0.44927	0.22032	0.07524	0.80915	0.4337	0.33883	0.02949	0.05524	0.05524	grey
AP002990	-0.07012	-0.09787	-0.12255	-0.00778	0.10408	-0.12603	0.05708	0.17054	-0.17054	0.36215	0.20284	0.11031	0.91953	0.17549	0.10049	0.45835	0.02574	0.02574	grey
HSDL2	-0.05569	-0.04431	0.02439	0.04369	-0.04801	0.03268	0.03906	0.09173	-0.09173	0.46936	0.56502	0.75155	0.57044	0.53292	0.67134	0.61201	0.23278	0.23278	turquoise
STARD5	-0.06877	0.02185	0.02031	-0.006	-0.00769	0.07369	0.0388	0.23318	-0.23318	0.37145	0.77666	0.792	0.93795	0.92044	0.33811	0.61433	0.00215	0.00215	grey
PTCD3	0.01265	-0.02991	-0.05671	-0.06943	0.05063	-0.06494	0.03329	0.01441	-0.01441	0.86955	0.6978	0.4613	0.36686	0.51073	0.39871	0.66556	0.85161	0.85161	turquoise
CYP2D6	-0.00634	-0.04755	-0.02366	0.03254	0.19327	-0.0623	-0.08775	-0.04381	0.04381	0.93445	0.53682	0.75869	0.67264	0.01132	0.41823	0.25374	0.56939	0.56939	grey
FLYWCH2	-0.01327	-0.10723	-0.16425	-0.08053	0.16178	-0.16069	-0.05764	0.00331	-0.00331	0.86325	0.16273	0.03182	0.29507	0.03452	0.03577	0.45395	0.96576	0.96576	green
NTM	-0.03564	-0.06595	0.02149	0.02256	-0.18901	0.16358	0.04423	0.10577	-0.10577	0.64355	0.39144	0.78022	0.7696	0.01329	0.03253	0.56567	0.16854	0.16854	black
ZNF551	-0.0508	-0.11267	-0.06635	0.01464	-0.15949	0.07802	-0.0032	0.29465	-0.29465	0.50932	0.14232	0.38857	0.84931	0.03719	0.31046	0.96688	9.15E-05	9.15E-05	turquoise
RPL41	0.13685	-0.01243	-0.0793	-0.04487	0.01114	-0.06601	-0.0623	-0.116	0.116	0.07429	0.87183	0.30252	0.56004	0.88499	0.39101	0.41827	0.13081	0.13081	purple
SERPINB3	-0.05573	0.1322	0.07992	0.00472	-0.01679	0.01618	0.1429	-0.32465	0.32465	0.46906	0.08477	0.29878	0.95119	0.82744	0.83361	0.06225	1.48E-05	1.48E-05	grey
ASB2	-0.04384	-0.00938	0.01811	0.04593	-0.02107	0.05757	-0.00579	0.30824	-0.30824	0.56913	0.90308	0.81417	0.55083	0.78445	0.45449	0.94007	4.10E-05	4.10E-05	salmon
CD1C	-0.10469	-0.11417	0.01619	-0.00475	-0.09891	0.12401	-0.0517	0.38051	-0.38051	0.17296	0.13704	0.83357	0.95079	0.19806	0.1061	0.50184	2.84E-07	2.84E-07	blue
MZT2A	0.04959	0.07064	-0.05821	0.05895	0.11648	-0.05248	0.17081	-0.40164	0.40164	0.51946	0.35856	0.44947	0.44378	0.12923	0.49541	0.02551	5.19E-08	5.19E-08	green
EXOSC7	0.07131	0.01524	-0.02047	-0.08883	-0.03366	0.03362	0.18065	-0.41734	0.41734	0.35398	0.84314	0.79044	0.24796	0.66211	0.66241	0.01806	1.36E-08	1.36E-08	grey
OGDHL	0.00378	-0.11484	-0.23152	-0.10387	0.10638	-0.31439	-0.23267	0.41054	-0.41054	0.96083	0.13472	0.00231	0.17636	0.16612	2.82E-05	0.0022	2.45E-08	2.45E-08	brown
FGFBP1	-0.00253	0.04145	0.10653	0.0565	-0.03125	0.1212	0.20365	-0.181	0.181	0.9738	0.59035	0.16549	0.46291	0.68491	0.1143	0.00755	0.01783	0.01783	grey
FAM86C1	-0.00829	0.02442	-0.07608	-0.0409	-0.11652	-0.0597	0.15977	-0.0982	0.0982	0.91434	0.75117	0.32264	0.59534	0.12909	0.43797	0.03686	0.20132	0.20132	turquoise
MATN3	0.03315	-0.01204	0.06383	0.05267	-0.22157	0.20868	0.03869	0.19493	-0.19493	0.66685	0.87579	0.40684	0.49385	0.00359	0.00616	0.61535	0.01062	0.01062	black
PIGA	-0.05268	-0.2126	-0.03584	0.08617	-0.10398	0.10049	0.07898	-0.12652	0.12652	0.49376	0.00524	0.64168	0.26243	0.17594	0.19094	0.30449	0.09915	0.09915	turquoise
MNT	-0.01579	-0.2276	-0.09277	-0.02592	0.01012	-0.10003	-0.03062	0.07071	-0.07071	0.8376	0.00276	0.22752	0.73648	0.89548	0.19299	0.69096	0.3581	0.3581	turquoise
MSANTD3	-0.06108	-0.13248	-0.07651	0.06568	-0.19082	-0.03099	0.0591	0.05335	-0.05335	0.42741	0.08412	0.31989	0.3934	0.01242	0.68742	0.4426	0.48832	0.48832	turquoise
PLOD1	-0.04178	-0.08846	0.06582	0.11013	-0.14341	0.08627	0.17438	-0.06311	0.06311	0.58739	0.2499	0.3924	0.15159	0.06131	0.26191	0.02254	0.41223	0.41223	black
CD79B	-0.11391	0.01692	0.01429	-0.00373	-0.06133	0.03423	-0.08674	0.35792	-0.35792	0.13796	0.82616	0.8528	0.96141	0.42551	0.65675	0.25929	1.54E-06	1.54E-06	blue
FARS2	-0.03738	0.11206	-0.05993	-0.11591	-0.02177	0.05388	0.09418	0.13194	-0.13194	0.62736	0.14449	0.43619	0.13111	0.77748	0.48398	0.22046	0.08539	0.08539	grey
DDX60L	-0.09583	-0.06098	0.13348	0.13083	-0.01937	0.17236	0.13078	-0.16347	0.16347	0.21248	0.42822	0.08178	0.08808	0.80149	0.02418	0.08821	0.03264	0.03264	tan
VNN2	-0.03497	-0.12489	0.06286	0.05112	-0.07212	0.20666	0.04835	0.07371	-0.07371	0.64975	0.10362	0.41408	0.50668	0.34857	0.00669	0.53004	0.33804	0.33804	grey
SH3D21	-0.00946	-0.00891	0.17797	0.05436	0.08587	0.16662	0.12987	-0.33575	0.33575	0.90226	0.90792	0.01987	0.48008	0.26412	0.0294	0.09045	7.14E-06	7.14E-06	yellow
RCN3	-0.06437	-0.04206	0.08938	0.08483	-0.07458	0.12946	0.07056	0.11147	-0.11147	0.40288	0.58489	0.24502	0.26994	0.33232	0.09148	0.35911	0.14664	0.14664	black
PPP4C	0.04921	0.07381	0.00572	-0.02072	-0.00453	0.06836	0.22603	-0.48537	0.48537	0.52271	0.33732	0.94081	0.78792	0.95312	0.37433	0.00295	1.72E-11	1.72E-11	green
ALDOA	0.05918	-0.01221	-0.00246	-0.02408	0.05028	0.05151	0.17759	-0.54342	0.54342	0.44199	0.87402	0.97453	0.75459	0.51373	0.50342	0.02014	1.59E-14	1.59E-14	yellow
NCEH1	-0.03229	-0.09101	0.02268	0.093	-0.18624	0.19987	0.14883	-0.08635	0.08635	0.67501	0.23647	0.76845	0.22635	0.01473	0.00877	0.05204	0.26144	0.26144	turquoise
SMARCC2	0.02495	-0.19392	-0.03733	0.01352	0.01491	-0.03575	-0.09211	0.11785	-0.11785	0.74604	0.01104	0.62788	0.86068	0.8465	0.64249	0.23085	0.12474	0.12474	turquoise
RCAN3	-0.05692	-0.17277	-0.14304	-0.10207	0.04415	-0.1134	-0.29639	0.22269	-0.22269	0.45964	0.02384	0.06199	0.18403	0.56638	0.13973	8.28E-05	0.00342	0.00342	turquoise
GALC	0.01233	-0.09544	0.02448	0.05477	-0.03358	0.09585	-0.02238	0.10382	-0.10382	0.87279	0.21436	0.75059	0.47681	0.66283	0.21238	0.77136	0.17657	0.17657	turquoise
ZBTB21	-0.07216	-0.2273	-0.05548	0.04652	-0.04095	0.02589	-0.12521	0.41786	-0.41786	0.34829	0.00279	0.4711	0.54569	0.59491	0.73677	0.10272	1.30E-08	1.30E-08	turquoise
KBTBD8	-0.15148	-0.04299	0.04507	0.00785	-0.14367	0.09669	-0.13522	0.45489	-0.45489	0.04795	0.57666	0.55833	0.91884	0.06083	0.20836	0.07783	4.10E-10	4.10E-10	blue
ZNF451	-0.04442	-0.11901	-0.05351	0.0231	-0.0589	-0.08677	-0.04307	0.24781	-0.24781	0.564	0.12105	0.48703	0.76424	0.44415	0.25913	0.57592	0.00108	0.00108	turquoise
MED31	-0.15191	-0.22024	-0.18523	-0.09296	0.10033	-0.25758	-0.11332	0.22386	-0.22386	0.04732	0.0038	0.01529	0.22654	0.19168	0.00067	0.14	0.00325	0.00325	brown
RNF24	-0.03771	-0.10646	0.04218	0.0666	-0.14113	0.09082	0.08691	0.15594	-0.15594	0.62436	0.16579	0.58384	0.3868	0.06558	0.23749	0.25837	0.04168	0.04168	turquoise
BCL7C	0.01842	0.03253	-0.08018	-0.04793	0.05037	-0.00507	0.19484	-0.35262	0.35262	0.81101	0.6728	0.29718	0.53362	0.51292	0.94751	0.01066	2.24E-06	2.24E-06	green
DROSHA	-0.01191	-0.12267	-0.08648	-0.01001	-0.11756	-0.07685	0.1624	0.0623	-0.0623	0.87718	0.10995	0.26074	0.89664	0.1257	0.31779	0.03382	0.41821	0.41821	turquoise
LEMD2	0.03728	-0.10898	-0.1666	-0.07814	0.10177	-0.17956	0.02782	-0.11219	0.11219	0.62832	0.15593	0.02942	0.30967	0.18534	0.01878	0.71799	0.14405	0.14405	grey
BTLA	-0.13187	-0.01636	0.03796	0.02904	-0.10382	0.03778	-0.1279	0.41316	-0.41316	0.08556	0.83176	0.62206	0.70619	0.17658	0.62369	0.09549	1.95E-08	1.95E-08	blue
PRDM10	-0.01824	-0.12171	-0.00072	0.02063	-0.1355	0.08887	0.04683	0.08006	-0.08006	0.81285	0.11278	0.99251	0.78885	0.07722	0.24772	0.54306	0.29791	0.29791	turquoise
UQCRB	0.08121	0.05209	-0.14026	-0.10356	0.04739	-0.2139	-0.04589	-0.07771	0.07771	0.29103	0.4986	0.06729	0.17766	0.53825	0.00497	0.55115	0.31234	0.31234	purple
DIPK1B	-0.03881	-0.13966	-0.29588	-0.1061	0.16297	-0.30435	-0.16478	0.37312	-0.37312	0.61427	0.06848	8.53E-05	0.16724	0.03319	5.18E-05	0.03127	5.00E-07	5.00E-07	brown
RNF5	0.09356	0.01533	-0.05542	0.00304	-0.03938	0.07429	0.10802	-0.30853	0.30853	0.22353	0.84228	0.47155	0.9685	0.60908	0.33423	0.15965	4.03E-05	4.03E-05	grey
ONECUT1	0.04149	-0.09143	-0.10542	0.01961	0.0153	0.06899	0.01429	0.06272	-0.06272	0.58998	0.2343	0.16999	0.79905	0.84258	0.36993	0.85284	0.41509	0.41509	magenta
CDK8	0.05674	-0.08901	-0.06467	0.08847	-0.0072	-0.09193	0.07938	-0.02869	0.02869	0.46101	0.24699	0.4007	0.24988	0.92558	0.23176	0.30204	0.70953	0.70953	turquoise
PCBP3	-0.05888	-0.08865	-0.11279	-0.02629	0.03496	-0.24044	-0.20075	0.57183	-0.57183	0.44425	0.24889	0.14188	0.73288	0.64985	0.00154	0.00847	3.11E-16	3.11E-16	brown
LAMA1	-0.01557	-0.02544	0.08051	0.0777	-0.11203	0.05488	0.09955	0.25201	-0.25201	0.83982	0.74122	0.29517	0.31245	0.14461	0.4759	0.19515	0.00088	0.00088	grey
TAF9B	-0.00733	-0.15786	-0.13742	-0.01582	0.01983	-0.17347	-0.13522	0.37167	-0.37167	0.9242	0.0392	0.07308	0.83728	0.79684	0.02327	0.07785	5.59E-07	5.59E-07	turquoise
PLK3	-0.04567	-0.09703	0.06049	0.0477	-0.12526	0.22263	0.13999	-0.0049	0.0049	0.55305	0.20675	0.43193	0.53555	0.10259	0.00343	0.06781	0.9493	0.9493	black
MPLKIP	0.039	-0.09121	-0.13717	-0.08333	0.13944	-0.10793	-0.03282	-0.18442	0.18442	0.61256	0.23546	0.0736	0.27855	0.06893	0.15998	0.67001	0.01575	0.01575	grey
RIC8B	0.04293	-0.19781	-0.17372	-0.02672	0.12524	-0.16122	-0.20012	0.37123	-0.37123	0.57721	0.0095	0.02307	0.72862	0.10265	0.03516	0.00868	5.77E-07	5.77E-07	turquoise
WNK4	0.04314	-0.11431	-0.12027	-0.10563	0.25385	-0.19673	-0.15116	0.11165	-0.11165	0.5753	0.13657	0.11714	0.16914	0.00081	0.00991	0.04843	0.14598	0.14598	grey
LNPEP	-0.0093	-0.11859	-0.0562	0.00276	-0.08609	-0.03742	-0.04331	0.18917	-0.18917	0.90389	0.1224	0.46536	0.97142	0.26288	0.62699	0.57383	0.01321	0.01321	turquoise
MRPL22	-0.01056	-0.08091	-0.1029	-0.05836	0.06834	-0.17332	0.03226	-0.24008	0.24008	0.891	0.2928	0.18047	0.44831	0.37449	0.02339	0.6753	0.00156	0.00156	grey
DOCK10	-0.13793	-0.06505	-0.005	-0.00335	-0.15438	-0.02931	-0.08781	0.58188	-0.58188	0.07201	0.39798	0.94828	0.96531	0.04379	0.70354	0.25343	7.05E-17	7.05E-17	blue
DHDDS	-0.05658	-0.09306	-0.00083	#######	-0.07666	0.08948	0.05591	0.09052	-0.09052	0.46231	0.22603	0.99144	0.99903	0.31896	0.2445	0.46766	0.23901	0.23901	turquoise
MGA	-0.04663	-0.23325	-0.06622	-0.02407	-0.14405	0.06079	-0.07108	0.30052	-0.30052	0.54479	0.00214	0.38948	0.7547	0.06015	0.42963	0.35559	6.50E-05	6.50E-05	turquoise
RMI2	-0.03248	0.11562	0.10334	0.03433	-0.16977	0.17288	0.35734	-0.45554	0.45554	0.67324	0.13209	0.17863	0.65578	0.02642	0.02375	1.60E-06	3.84E-10	3.84E-10	pink
GALNT16	-0.02184	-0.11644	-0.11202	0.02916	-0.02824	-0.12732	-0.1317	0.61001	-0.61001	0.77676	0.12936	0.14466	0.70495	0.71383	0.09702	0.08596	8.30E-19	8.30E-19	brown
ARFGEF1	-0.01627	-0.08597	0.03736	0.00037	-0.1016	0.02318	0.09092	-0.01689	0.01689	0.83272	0.26355	0.6276	0.99617	0.18608	0.7635	0.23693	0.82641	0.82641	turquoise
VPS45	0.00773	-0.05776	-0.11964	-0.1312	0.02839	-0.05493	0.07744	0.03211	-0.03211	0.92003	0.45303	0.11909	0.08718	0.71238	0.47549	0.31407	0.67676	0.67676	turquoise
NLGN3	-0.01585	-0.14796	-0.01444	-0.03855	-0.08178	0.13135	-0.07739	0.45182	-0.45182	0.83702	0.05345	0.85132	0.61668	0.28764	0.08681	0.3144	5.54E-10	5.54E-10	blue
ITGB6	0.0355	-0.00245	0.10453	0.09958	-0.11186	0.2456	0.19789	-0.3187	0.3187	0.64484	0.97461	0.17362	0.19501	0.14523	0.0012	0.00947	2.15E-05	2.15E-05	yellow
USP18	-0.05726	-0.02851	0.05343	0.05275	-0.07689	-0.01481	0.02752	0.0633	-0.0633	0.45696	0.71128	0.48765	0.4932	0.3175	0.84757	0.72089	0.41077	0.41077	tan
LPCAT3	-0.04909	-0.13742	0.06241	0.07967	-0.01305	0.07574	-0.03755	0.02992	-0.02992	0.52377	0.07308	0.41744	0.30029	0.86543	0.32486	0.62579	0.69763	0.69763	turquoise
SOSTDC1	0.03211	-0.18788	-0.02147	-0.02331	-0.06933	-0.05869	0.10321	0.00359	-0.00359	0.67669	0.01386	0.7805	0.76215	0.3676	0.44576	0.17917	0.96285	0.96285	grey
ZNF850	0.00449	-0.19404	0.00097	0.15223	-0.03953	0.06887	-0.00077	0.15807	-0.15807	0.95347	0.01099	0.98993	0.04684	0.60772	0.37077	0.992	0.03893	0.03893	turquoise
CAND1	0.00628	-0.2003	-0.03316	0.01143	-0.0432	-0.03238	-0.02243	0.18574	-0.18574	0.93499	0.00862	0.66681	0.88207	0.5748	0.67413	0.7709	0.015	0.015	turquoise
KCNJ5	-0.10473	0.07458	0.0306	0.01239	-0.18504	0.07293	0.00858	0.44357	-0.44357	0.1728	0.33231	0.69118	0.87217	0.0154	0.34316	0.9113	1.23E-09	1.23E-09	magenta
KCNJ11	0.01164	-0.04993	-0.29677	-0.17014	0.1335	-0.29001	-0.18932	0.32441	-0.32441	0.87993	0.51661	8.10E-05	0.02609	0.08173	0.00012	0.01314	1.50E-05	1.50E-05	brown
CRYBB1	-0.0174	0.07221	0.00847	0.04208	-0.10644	-0.0248	0.00658	0.46295	-0.46295	0.8213	0.34793	0.91243	0.58477	0.16587	0.74743	0.93194	1.82E-10	1.82E-10	blue
CAPN3	-0.05477	-0.07483	-0.04839	0.03668	0.09263	#######	-0.10057	0.0918	-0.0918	0.47675	0.3307	0.52966	0.63383	0.22822	0.99922	0.19061	0.23242	0.23242	red
TRAF3IP2	-0.07459	0.06147	-0.0307	-0.07227	-0.01112	0.05985	0.06865	0.06066	-0.06066	0.33227	0.42448	0.69016	0.34758	0.88527	0.43684	0.37228	0.43065	0.43065	turquoise
POGK	0.02785	-0.10524	-0.0394	-0.03448	-0.07611	0.12968	0.06782	-0.07908	0.07908	0.71765	0.17072	0.60886	0.65437	0.32248	0.09094	0.3781	0.30389	0.30389	turquoise
SMARCC1	0.00886	-0.05527	0.02297	-0.04816	-0.1615	0.19384	0.10007	-0.04178	0.04178	0.90845	0.47275	0.76552	0.5316	0.03483	0.01107	0.19282	0.5874	0.5874	turquoise
PRKACB	-0.1154	-0.07874	-0.04704	0.01971	-0.0061	0.029	-0.10474	0.312	-0.312	0.13282	0.30597	0.54123	0.79804	0.93684	0.7065	0.17278	3.27E-05	3.27E-05	turquoise
HOOK3	-0.03947	-0.15435	0.0187	0.12444	-0.1211	0.03313	0.0479	0.30162	-0.30162	0.60827	0.04383	0.8082	0.10489	0.11462	0.66705	0.53382	6.10E-05	6.10E-05	blue
DHX15	-0.09978	-0.09904	0.00609	-0.0092	-0.0602	0.08463	0.10203	-0.05215	0.05215	0.19415	0.19749	0.93698	0.90496	0.43411	0.27109	0.18423	0.49819	0.49819	turquoise
CCDC188	-0.08096	-0.1214	-0.2466	-0.11229	0.19422	-0.35707	-0.2501	0.36339	-0.36339	0.29249	0.11372	0.00115	0.14368	0.01091	1.63E-06	0.00097	1.03E-06	1.03E-06	brown
ATAD3C	0.00214	0.05575	-0.1253	0.07001	0.09401	-0.21835	0.05984	0.07263	-0.07263	0.97781	0.46895	0.10247	0.36288	0.2213	0.00412	0.4369	0.34513	0.34513	grey
RAD54L	-0.06152	0.09838	0.09678	0.00121	-0.07534	0.13681	0.31973	-0.405	0.405	0.42413	0.20049	0.20795	0.98746	0.32737	0.07438	2.02E-05	3.92E-08	3.92E-08	pink
CHORDC1	0.08479	-0.2327	-0.01402	0.06663	0.01747	0.00832	-0.00296	0.05992	-0.05992	0.2702	0.00219	0.85562	0.38655	0.82054	0.91395	0.96932	0.43628	0.43628	turquoise
SLC18A2	-0.02953	-0.07584	-0.11566	-0.06349	-0.0648	-0.07612	-0.0586	0.44461	-0.44461	0.70139	0.32421	0.13197	0.40935	0.3998	0.32239	0.44648	1.11E-09	1.11E-09	turquoise
DENND5A	-0.15291	-0.18831	-0.01302	0.09981	-0.21422	0.02057	-0.03915	0.50373	-0.50373	0.04586	0.01365	0.86576	0.19401	0.0049	0.78944	0.61121	2.17E-12	2.17E-12	blue
ATP6V0E1	-0.00031	-0.10136	-0.0239	-0.01248	-0.07704	-0.01677	0.02798	-0.01898	0.01898	0.99681	0.18711	0.75634	0.87134	0.31657	0.82769	0.71642	0.8054	0.8054	turquoise
BTBD16	0.11915	-0.05606	0.00027	0.00471	0.11337	0.08628	0.06742	-0.47324	0.47324	0.1206	0.46643	0.99723	0.95122	0.13985	0.26181	0.38096	6.30E-11	6.30E-11	grey
N4BP1	0.00013	-0.16554	0.0185	0.005	-0.11328	0.07285	0.07087	-0.02121	0.02121	0.99866	0.03048	0.81021	0.94824	0.14016	0.3437	0.357	0.78309	0.78309	turquoise
ART5	-0.05247	-0.0095	-0.04628	0.13296	-0.06796	0.01569	-0.01797	0.26566	-0.26566	0.49549	0.90182	0.54782	0.08299	0.37711	0.83862	0.81557	0.00045	0.00045	black
TBX19	0.01609	-0.18719	-0.10517	-0.0601	0.06334	-0.14327	-0.10485	0.02853	-0.02853	0.83459	0.01422	0.17102	0.43491	0.41049	0.06157	0.17233	0.71103	0.71103	grey
CXCR1	-0.15129	-0.02773	0.08681	0.01318	-0.02304	0.07518	0.0198	0.24259	-0.24259	0.04823	0.71887	0.25889	0.86416	0.76488	0.32845	0.79716	0.00139	0.00139	grey
ISLR2	-0.04454	-0.18521	0.00676	0.08693	-0.10516	0.08862	0.07445	0.29995	-0.29995	0.56298	0.0153	0.93004	0.25823	0.17102	0.24904	0.33316	6.73E-05	6.73E-05	blue
PDRG1	0.12191	-0.04208	-0.12791	-0.05178	0.19575	-0.14723	-0.00099	-0.23119	0.23119	0.1122	0.5847	0.09548	0.50119	0.01029	0.05465	0.98972	0.00235	0.00235	grey
AKR1C2	0.13317	-0.05524	0.01791	0.09387	-0.02222	0.07612	0.10308	-0.09199	0.09199	0.08249	0.47296	0.81615	0.22199	0.77302	0.32243	0.17972	0.23147	0.23147	grey
ADCY2	0.01293	-0.10481	-0.07496	-0.01749	0.04753	-0.25733	-0.24887	0.40156	-0.40156	0.8667	0.17246	0.32988	0.82037	0.53698	0.00068	0.00103	5.23E-08	5.23E-08	brown
EIF3I	-0.0333	-0.00476	0.10686	0.02038	-0.03857	0.19931	0.124	-0.27518	0.27518	0.66544	0.95077	0.16419	0.79137	0.61645	0.00896	0.10613	0.00027	0.00027	turquoise
TTLL4	0.06916	-0.07104	0.03862	0.04363	0.0223	0.1309	0.1781	-0.25263	0.25263	0.36877	0.35585	0.61603	0.57099	0.77218	0.08792	0.01978	0.00086	0.00086	turquoise
HMGB2	-0.04414	0.04244	0.05775	-0.04589	-0.01468	0.17372	0.11814	-0.27616	0.27616	0.56647	0.58156	0.45307	0.55113	0.8489	0.02307	0.12381	0.00026	0.00026	pink
UBE2N	-0.05161	-0.09014	0.01363	-0.03282	0.00013	-0.05042	-0.09161	0.09295	-0.09295	0.50258	0.24098	0.85952	0.67	0.99862	0.51256	0.23341	0.22662	0.22662	turquoise
FAM185A	0.03114	-0.0504	-0.03695	0.02968	0.07763	0.02558	0.13184	-0.11335	0.11335	0.68597	0.51272	0.63135	0.69994	0.31285	0.73984	0.08563	0.1399	0.1399	turquoise
ABCA1	-0.13132	-0.15268	-0.00723	0.06606	-0.1909	-0.00951	-0.00899	0.45841	-0.45841	0.08689	0.0462	0.92522	0.39062	0.01238	0.90179	0.90714	2.89E-10	2.89E-10	blue
NIF3L1	0.03373	0.02381	-0.0596	-0.04791	-0.0482	0.03011	0.21041	-0.20696	0.20696	0.66145	0.75724	0.43869	0.53378	0.53133	0.69588	0.00574	0.00661	0.00661	turquoise
ANKRD1	-0.04743	-0.17842	-0.03107	0.0324	-0.13364	0.06101	0.13016	-0.0071	0.0071	0.53789	0.01956	0.68663	0.67396	0.0814	0.42798	0.08973	0.92657	0.92657	grey
MCOLN2	-0.10381	0.05113	0.01439	0.02179	-0.1132	0.11529	0.0313	0.31333	-0.31333	0.17662	0.50658	0.85182	0.77727	0.14045	0.13322	0.68447	3.01E-05	3.01E-05	blue
MMUT	-0.03463	-0.09229	-0.02646	-0.01023	0.01759	-0.00229	-0.06505	0.22444	-0.22444	0.65294	0.22991	0.73118	0.89438	0.81938	0.9763	0.39793	0.00317	0.00317	turquoise
VTA1	-0.04965	-0.0468	0.04239	0.0338	-0.08594	0.04376	0.04319	0.0985	-0.0985	0.51903	0.54328	0.58197	0.66073	0.2637	0.56985	0.57489	0.19995	0.19995	turquoise
SIM2	0.10099	-0.0161	0.11424	0.00262	0.01101	0.0712	0.13761	-0.36608	0.36608	0.18873	0.83447	0.13679	0.97285	0.88638	0.35479	0.07267	8.48E-07	8.48E-07	grey
ZCCHC17	-0.07921	-0.16465	-0.03591	0.03242	-0.10531	0.00607	-0.03514	0.0867	-0.0867	0.3031	0.0314	0.64098	0.67375	0.17041	0.93716	0.64816	0.25951	0.25951	turquoise
EML2	0.16908	-0.00339	-0.00528	-0.11053	0.18074	-0.00934	0.08411	-0.35181	0.35181	0.02705	0.96489	0.94533	0.1501	0.018	0.90353	0.27406	2.38E-06	2.38E-06	grey
GPR35	0.08299	0.13045	0.06147	0.01455	0.13358	0.21295	0.16946	-0.43472	0.43472	0.28055	0.08902	0.42444	0.85014	0.08156	0.00517	0.02671	2.83E-09	2.83E-09	yellow
PRG4	-0.0987	-0.13861	-0.11209	-0.05543	-0.18918	-0.02613	-0.07326	0.505	-0.505	0.19903	0.07061	0.14441	0.4715	0.01321	0.73442	0.34094	1.87E-12	1.87E-12	blue

PCDHGA2	-0.06127	-0.19346	-0.11926	-0.00545	-0.0669	-0.07904	-0.08167	0.39604	-0.39604	0.42598	0.01124	0.12027	0.94364	0.38462	0.30414	0.28827	8.24E-08	8.24E-08 turquoise
KATNB1	0.05187	-0.0445	-0.13357	-0.13889	0.14423	-0.11945	0.02248	-0.21877	0.21877	0.5005	0.56328	0.08156	0.07004	0.05982	0.11968	0.77036	0.00404	0.00404 grey
S100A12	-0.08748	-0.08183	-0.01351	-0.04099	-0.10194	-0.0172	0.00019	0.43244	-0.43244	0.25525	0.28734	0.86077	0.59449	0.18461	0.82329	0.99807	3.49E-09	3.49E-09 blue
ZNF248	0.01708	-0.23212	-0.13639	-0.06025	0.05183	-0.18581	-0.07171	0.31121	-0.31121	0.82448	0.00225	0.07527	0.43373	0.50078	0.01496	0.3513	3.43E-05	3.43E-05 turquoise
CREM	-0.11043	-0.16103	0.03064	0.07724	-0.15661	0.03969	-0.10598	0.49068	-0.49068	0.15048	0.03537	0.69077	0.31532	0.0408	0.60626	0.16773	9.57E-12	9.57E-12 blue
ZDHHC6	0.02247	-0.12694	0.00271	-0.01791	-0.03764	0.04461	0.10779	-0.16755	0.16755	0.7705	0.09804	0.97189	0.81616	0.62499	0.56232	0.16055	0.02849	0.02849 turquoise
AL159163	-0.14456	0.07827	-0.0047	0.00564	-0.00569	0.05709	0.0957	-0.23209	0.23209	0.05924	0.30889	0.95138	0.94166	0.94109	0.45827	0.21309	0.00225	0.00225 grey
POU6F1	-0.06558	-0.16893	-0.1308	-0.00659	-0.03439	-0.08851	-0.18431	0.4717	-0.4717	0.39408	0.02719	0.08816	0.93187	0.65517	0.24963	0.01581	7.40E-11	7.40E-11 blue
CSPG4	-0.1423	0.02579	-0.02112	0.03222	0.02147	0.0065	0.07015	0.09497	-0.09497	0.06337	0.73779	0.78391	0.67566	0.78041	0.93276	0.36192	0.2166	0.2166 black
BIN3	-0.03502	0.0318	0.03641	-0.06573	0.14856	0.07687	0.08596	-0.19172	0.19172	0.6493	0.67969	0.63635	0.39299	0.05248	0.31762	0.2636	0.012	0.012 grey
PLP1	-0.04422	-0.05994	-0.12096	0.0442	-0.08474	-0.09845	-0.08045	0.42665	-0.42665	0.56575	0.43614	0.11503	0.56594	0.27049	0.20015	0.29556	5.93E-09	5.93E-09 grey
RTN2	0.12118	-0.11407	-0.11639	-0.09822	0.02832	-0.18478	-0.07517	-0.07138	0.07138	0.11438	0.13738	0.12952	0.20123	0.71314	0.01555	0.32851	0.35352	0.35352 grey
TMED6	-0.07245	0.09167	-0.15895	-0.00788	0.01858	-0.09457	-0.11586	0.31545	-0.31545	0.34633	0.2331	0.03785	0.91849	0.8094	0.21858	0.13127	2.64E-05	2.64E-05 magenta
XYLT2	0.01304	-0.20215	-0.11178	-0.00175	0.0591	-0.05756	0.0467	-0.14143	0.14143	0.8656	0.00801	0.14552	0.98185	0.44257	0.45456	0.54416	0.06502	0.06502 grey
LYZ	-0.07106	0.07471	0.14758	0.01015	0.01032	0.29102	0.09338	-0.09922	0.09922	0.35574	0.3315	0.05408	0.89522	0.89343	0.00011	0.22445	0.19665	0.19665 yellow
DDX21	-0.04241	-0.06011	0.08234	0.04317	-0.18578	0.16205	0.07896	0.06753	-0.06753	0.58179	0.43481	0.28433	0.57506	0.01498	0.03421	0.30463	0.38015	0.38015 turquoise
VAC14	-0.00269	0.03389	-0.04857	-0.037	-0.00096	-0.04301	0.13746	-0.07518	0.07518	0.97219	0.65991	0.52814	0.63088	0.99009	0.57644	0.07299	0.32845	0.32845 turquoise
USP9X	-0.18611	-0.14417	-0.06132	0.0236	-0.13148	0.0396	0.01447	0.22991	-0.22991	0.0148	0.05994	0.42562	0.75931	0.08649	0.60704	0.85097	0.00249	0.00249 turquoise
STX17	-0.03453	-0.15081	-0.03068	0.07123	-0.17025	0.09022	0.04567	0.21203	-0.21203	0.65387	0.04896	0.69036	0.35452	0.026	0.24058	0.55313	0.00537	0.00537 turquoise
SAP30	-0.00303	-0.07894	0.03897	0.0652	-0.02216	0.08677	0.02965	0.05296	-0.05296	0.96864	0.30472	0.61285	0.39685	0.77362	0.25913	0.70023	0.49149	0.49149 turquoise
ISG20L2	0.04486	-0.05686	0.00264	-0.0223	-0.07132	0.14071	0.16571	-0.12141	0.12141	0.56017	0.4601	0.97269	0.7722	0.35397	0.06641	0.03031	0.11369	0.11369 turquoise
ZFYVE19	0.02899	-0.15943	-0.02454	0.01184	-0.06178	0.01329	0.17951	-0.41428	0.41428	0.7066	0.03726	0.75006	0.87784	0.42213	0.86303	0.01881	1.77E-08	1.77E-08 grey
STOX1	0.00154	-0.1376	0.00931	0.06729	-0.05913	0.00197	0.00203	0.14972	-0.14972	0.98409	0.07269	0.90386	0.38185	0.44237	0.97957	0.97898	0.05064	0.05064 grey
NR2F1	-0.06838	-0.14658	-0.09838	0.02156	-0.11068	0.13344	0.01604	0.31352	-0.31352	0.3742	0.05573	0.20051	0.7796	0.14953	0.08187	0.835	2.97E-05	2.97E-05 blue
ZNF566	-0.07195	-0.20369	-0.10188	0.05474	-0.07008	-0.03166	-0.10299	0.42749	-0.42749	0.3497	0.00754	0.18485	0.47699	0.36242	0.68104	0.1801	5.49E-09	5.49E-09 turquoise
MORF4L2	-0.01081	-0.20176	-0.09308	-0.01029	0.03219	-0.06303	-0.01872	0.03571	-0.03571	0.88835	0.00814	0.22594	0.89375	0.67598	0.41282	0.80799	0.64285	0.64285 turquoise
PTGS2	0.00707	-0.0505	0.13209	0.07594	-0.07651	0.16698	0.20296	-0.10907	0.10907	0.92691	0.51184	0.08505	0.32356	0.31992	0.02905	0.00776	0.15561	0.15561 grey
ZZEF1	-0.0934	-0.16339	-0.08789	-0.043	0.02655	-0.17751	-0.16736	0.42816	-0.42816	0.22436	0.03273	0.253	0.57651	0.73035	0.02019	0.02868	5.17E-09	5.17E-09 turquoise
CCDC90B	-0.03932	-0.13347	0.02274	0.0498	-0.14479	0.0605	0.0853	0.02851	-0.02851	0.6096	0.0818	0.7678	0.51775	0.05883	0.43187	0.26733	0.71124	0.71124 turquoise
SYNJ1	-0.01891	-0.21878	-0.10593	-0.00489	0.03698	-0.07559	-0.06893	0.46397	-0.46397	0.80605	0.00404	0.16793	0.94935	0.63109	0.32577	0.37034	1.64E-10	1.64E-10 turquoise
UBE2Z	-0.00023	-0.15939	-0.09873	0.01597	-0.07732	-0.0404	0.07551	-0.08669	0.08669	0.99764	0.03731	0.19889	0.83571	0.31483	0.59983	0.32632	0.25957	0.25957 turquoise
CDK5RAP2	0.02608	-0.08105	-0.08817	-0.0462	-0.22233	0.01391	0.03611	0.10999	-0.10999	0.73492	0.29194	0.25151	0.54851	0.00347	0.85666	0.63914	0.1521	0.1521 turquoise
ZNF132	-0.09343	-0.22076	-0.17317	-0.02147	0.05316	-0.18552	-0.21474	0.44516	-0.44516	0.22417	0.00371	0.02351	0.78045	0.48982	0.01513	0.0048	1.06E-09	1.06E-09 brown
FOCAD	-0.04347	0.02606	-0.14484	-0.10735	-0.07165	-0.11117	-0.00536	0.33276	-0.33276	0.5724	0.73511	0.05874	0.16225	0.35174	0.14774	0.94452	8.71E-06	8.71E-06 turquoise
ICA1	0.11703	-0.04265	-0.00787	-0.05415	0.17156	0.04396	-0.08914	-0.39824	0.39824	0.12741	0.57964	0.91863	0.48176	0.02485	0.56807	0.24628	6.88E-08	6.88E-08 grey
OSBPL5	-0.0286	-0.04222	0.03521	0.06403	-0.07036	0.16075	0.15131	-0.0597	0.0597	0.71037	0.58353	0.64751	0.40539	0.36048	0.0357	0.04821	0.43793	0.43793 black
C11orf53	0.10961	-0.05488	0.06445	0.02383	0.16411	0.17809	0.03713	-0.26673	0.26673	0.15356	0.47592	0.40233	0.75708	0.03197	0.01978	0.62972	0.00042	0.00042 grey
FGFR4	0.06716	0.0645	-0.07976	0.04438	0.24	0.02943	0.00259	-0.07918	0.07918	0.38281	0.40193	0.29973	0.56436	0.00157	0.70237	0.97316	0.30325	0.30325 grey
WDR36	-0.05661	-0.11432	-0.01525	0.04465	-0.07405	0.03403	-0.01712	0.13762	-0.13762	0.4621	0.13653	0.84303	0.56199	0.33578	0.65859	0.82412	0.07265	0.07265 turquoise
TRIM25	0.03829	-0.11436	-0.11985	-0.06913	0.11603	-0.16585	-0.00832	0.03331	-0.03331	0.619	0.13639	0.11842	0.36895	0.13071	0.03016	0.91404	0.66534	0.66534 grey
MBOAT1	0.04809	-0.01128	0.07536	0.10124	-0.02668	0.27832	0.1821	-0.25742	0.25742	0.53219	0.88361	0.32726	0.18766	0.72908	0.00023	0.01714	0.00068	0.00068 turquoise
CRB3	0.10967	0.00626	-0.08812	-0.06725	0.22529	-0.0148	-0.02406	-0.43916	0.43916	0.1533	0.93519	0.25178	0.38213	0.00305	0.84764	0.75479	1.87E-09	1.87E-09 grey
XPO4	-0.01519	-0.03926	-0.0842	-0.08206	-0.09262	-0.02399	-0.018	0.25192	-0.25192	0.84371	0.61014	0.27353	0.28596	0.22826	0.75547	0.81525	0.00089	0.00089 turquoise
TMEM30A	-0.09165	-0.12003	-0.00974	0.01836	-0.11103	-0.00954	-0.0515	0.33341	-0.33341	0.2332	0.11789	0.89937	0.81157	0.14824	0.90144	0.50349	8.35E-06	8.35E-06 turquoise
ECHDC3	0.00657	0.0798	-0.08018	0.09731	-0.03674	-0.05666	-0.09599	0.3652	-0.3652	0.93207	0.29951	0.2972	0.20545	0.63332	0.46168	0.2117	9.04E-07	9.04E-07 grey
WAS	-0.11402	-0.01773	0.04835	0.03349	-0.2001	0.11397	0.03532	0.40001	-0.40001	0.13758	0.81793	0.53	0.66366	0.00869	0.13773	0.64649	5.95E-08	5.95E-08 blue
FBXO6	-0.09008	0.02114	0.09636	0.1713	-0.09396	0.10119	0.08991	-0.06048	0.06048	0.2413	0.78375	0.20993	0.02508	0.22155	0.18787	0.2422	0.43201	0.43201 tan
ZGRF1	-0.16027	-0.02188	-0.04279	-0.05118	0.06578	-0.0972	0.06877	-0.09461	0.09461	0.03626	0.77633	0.57845	0.50618	0.39264	0.20595	0.37144	0.21836	0.21836 red
C8orf76	0.02468	-0.02242	-0.04764	-0.01016	0.10387	-0.21829	0.07869	-0.25441	0.25441	0.74861	0.77103	0.53604	0.89507	0.17639	0.00413	0.30626	0.00079	0.00079 green
DDR1	0.05991	-0.03446	-0.0126	-0.04022	0.11681	0.02533	0.00399	-0.36216	0.36216	0.43634	0.6546	0.87007	0.60149	0.12815	0.74224	0.95869	1.13E-06	1.13E-06 grey
CDH6	-0.0049	-0.21123	-0.14952	0.01182	-0.06126	0.01417	-0.10417	0.30072	-0.30072	0.94924	0.00555	0.05096	0.87806	0.42609	0.85411	0.17515	6.43E-05	6.43E-05 blue
SPI1	-0.11627	0.01841	0.05685	0.05856	-0.17534	0.14452	0.07782	0.32516	-0.32516	0.12992	0.81108	0.46017	0.44676	0.0218	0.05931	0.31166	1.43E-05	1.43E-05 blue
PGBD4	-0.14467	-0.09565	-0.12902	-0.09019	0.04566	-0.10942	-0.01134	0.02169	-0.02169	0.05904	0.21333	0.09259	0.24074	0.55319	0.15427	0.88295	0.7783	0.7783 red
ZNF780A	0.02598	-0.27015	-0.08707	0.0201	-0.06752	-0.05853	-0.06756	0.16494	-0.16494	0.73589	0.00035	0.25745	0.79415	0.3802	0.44699	0.37998	0.0311	0.0311 turquoise
PPARA	-0.0358	-0.04359	-0.01066	0.06197	-0.07196	0.02539	0.07224	0.05542	-0.05542	0.642	0.57132	0.8899	0.4207	0.34965	0.74169	0.34773	0.47155	0.47155 turquoise
ZNF425	-0.01244	-0.12573	-0.02151	0.00946	-0.05189	-0.0414	0.00269	0.13811	-0.13811	0.87169	0.1013	0.78	0.90229	0.50027	0.59086	0.97212	0.07164	0.07164 turquoise
ZNF778	-0.0738	-0.14411	-0.24359	-0.13327	0.0263	-0.27933	-0.20462	0.43218	-0.43218	0.33741	0.06004	0.00133	0.08225	0.73274	0.00022	0.00726	3.58E-09	3.58E-09 brown
SCPEP1	-0.16813	-0.13467	0.01007	0.01472	-0.1546	-0.02832	-0.08828	0.48823	-0.48823	0.02794	0.07907	0.89601	0.84846	0.04348	0.71307	0.25087	1.25E-11	1.25E-11 blue
SGCB	-0.09981	-0.1407	-0.1211	-0.01673	-0.05802	-0.08816	-0.06771	0.30821	-0.30821	0.19401	0.06643	0.11462	0.82808	0.45098	0.25156	0.37889	4.11E-05	4.11E-05 turquoise
GC	0.02399	-0.09386	-0.21282	-0.11995	0.1002	-0.15799	-0.17714	0.32264	-0.32264	0.7555	0.22208	0.0052	0.11812	0.19227	0.03903	0.02046	1.68E-05	1.68E-05 magenta
PLIN1	-0.03283	0.00127	-0.02218	0.10771	0.04254	-0.00088	-0.13057	0.42146	-0.42146	0.66987	0.98686	0.77334	0.16085	0.58068	0.99084	0.08871	9.44E-09	9.44E-09 blue
FBXO28	-0.03007	-0.10974	-0.00615	0.02386	-0.12117	0.117	0.0897	-0.03438	0.03438	0.69625	0.15306	0.93635	0.75669	0.1144	0.12752	0.24332	0.65529	0.65529 turquoise
PDIA3	-0.00871	-0.10954	-0.10766	-0.07075	-0.03151	-0.15116	0.04804	-0.2653	0.2653	0.90999	0.15382	0.16103	0.35779	0.68243	0.04843	0.53268	0.00045	0.00045 turquoise
RPF2	0.02669	0.00051	0.03061	0.01851	-0.16115	0.10675	0.19611	0.00378	-0.00378	0.72897	0.99475	0.691	0.8101	0.03523	0.16461	0.01015	0.96083	0.96083 turquoise
APPL1	-0.09272	-0.05009	-0.0325	0.02163	-0.17594	0.1398	-0.01416	0.34104	-0.34104	0.22777	0.5153	0.67306	0.77884	0.02135	0.06819	0.8542	5.00E-06	5.00E-06 turquoise
UPF2	0.01476	-0.06913	0.0487	0.0503	-0.13248	0.16581	0.06654	0.09101	-0.09101	0.84802	0.36894	0.52706	0.51352	0.0841	0.03021	0.38717	0.23645	0.23645 turquoise
NRK	-0.02777	-0.05286	0.02415	0.0926	-0.16938	0.08825	0.02453	0.6084	-0.6084	0.71843	0.49234	0.75387	0.22837	0.02678	0.25103	0.75012	1.08E-18	1.08E-18 blue
SLPI	0.05099	0.02348	0.024	3.31E-05	-0.01646	0.13408	0.19201	-0.44246	0.44246	0.50777	0.76052	0.75531	0.99966	0.83079	0.08039	0.01187	1.37E-09	1.37E-09 yellow
ITGB3BP	-0.03916	0.01617	-0.02923	-0.04114	-0.00319	-0.06193	0.08259	-0.08237	0.08237	0.61111	0.83375	0.70428	0.59318	0.96698	0.42101	0.28286	0.28414	0.28414 turquoise
TOR4A	0.00797	-0.00637	0.05518	0.03862	-0.09951	0.22996	0.27665	-0.41817	0.41817	0.91756	0.93413	0.47349	0.61603	0.19533	0.00248	0.00025	1.26E-08	1.26E-08 yellow
MED1	-0.04303	-0.11245	-0.04366	0.02188	-0.13704	0.07153	0.05897	0.22325	-0.22325	0.57632	0.1431	0.57068	0.77633	0.07389	0.35255	0.44359	0.00333	0.00333 turquoise
CRH	-0.01934	-0.06247	-0.16042	-0.05611	-0.02264	-0.12035	-0.09998	0.26563	-0.26563	0.80179	0.41696	0.03609	0.46604	0.76882	0.11689	0.19325	0.00045	0.00045 brown
CLEC5A	-0.11053	-0.0408	0.08676	0.10478	-0.21417	0.203	0.09831	0.22404	-0.22404	0.15012	0.59623	0.25917	0.17258	0.00491	0.00775	0.20081	0.00322	0.00322 blue
RBM12	-0.03371	-0.11617	-0.02373	-0.00658	-0.11161	0.09686	0.02117	0.1873	-0.1873	0.66157	0.13026	0.75805	0.93198	0.14613	0.20758	0.7834	0.01417	0.01417 turquoise
PDCD4	-0.07386	-0.03778	-0.02005	0.03673	-0.09326	-0.03085	-0.18944	0.39923	-0.39923	0.33705	0.6237	0.79466	0.63337	0.22503	0.68878	0.01308	6.34E-08	6.34E-08 blue
ERO1B	-0.04634	-0.0906	-0.21652	-0.09282	0.03013	-0.25023	-0.18964	0.50993	-0.50993	0.54731	0.2386	0.00445	0.22722	0.69567	0.00096	0.01298	1.05E-12	1.05E-12 brown
RBM43	-0.04382	-0.05749	0.00633	0.11046	-0.18242	0.14133	0.06741	0.17295	-0.17295	0.56929	0.45516	0.93452	0.15037	0.01694	0.06521	0.381	0.02369	0.02369 turquoise
TTLL1	-0.04078	-0.04618	-0.15479	-0.03687	0.02472	-0.12436	-0.01296	0.14401	-0.14401	0.59639	0.54866	0.04323	0.63207	0.74826	0.1051	0.86639	0.06022	0.06022 grey
WDR90	0.03649	0.00212	-0.05981	-0.01424	0.24238	-0.02712	0.09309	-0.29515	0.29515	0.63564	0.97804	0.43712	0.85337	0.0014	0.7248	0.22591	8.89E-05	8.89E-05 green
MAPK4	-0.01574	-0.12165	-0.26616	-0.15747	0.08172	-0.33946	-0.20472	0.44689	-0.44689	0.83807	0.11298	0.00043	0.03969	0.28799	5.57E-06	0.00723	8.95E-10	8.95E-10 brown
SDAD1	-0.0817	-0.08039	0.00871	0.0014	-0.08867	0.07143	-0.02156	0.1884	-0.1884	0.28812	0.2959	0.91002	0.98546	0.24879	0.35322	0.77953	0.0136	0.0136 turquoise
MSANTD4	-0.06356	-0.18603	-0.16848	-0.09335	-0.13199	-0.10814	-0.08736	0.34727	-0.34727	0.40885	0.01485	0.02761	0.22457	0.08527	0.15918	0.2559	3.26E-06	3.26E-06 turquoise
HAUS5	-0.03053	-0.03501	-0.03254	-0.03142	-0.00453	-0.06378	0.11842	-0.12329	0.12329	0.69182	0.6494	0.67267	0.68335	0.95312	0.40726	0.12293	0.10815	0.10815 red
SBNO2	-0.0118	-0.05446	0.03608	0.0185	-0.03918	0.19381	0.17477	-0.21275	0.21275	0.8783	0.47931	0.6394	0.81018	0.61093	0.01109	0.02224	0.00521	0.00521 yellow
FCGRT	-0.09444	-0.03749	0.1374	0.06889	0.00939	0.15961	0.03695	0.14933	-0.14933	0.21922	0.62641	0.07312	0.37063	0.90298	0.03705	0.6314	0.05125	0.05125 grey
MED28	-0.00206	-0.18717	-0.09745	-0.03239	-0.0148	-0.05895	-0.01188	0.13106	-0.13106	0.97863	0.01423	0.20479	0.6741	0.84767	0.44375	0.87739	0.08751	0.08751 turquoise
ZFHX2	-0.01592	-0.29044	-0.18083	-0.09994	0.13667	-0.1654	-0.25144	0.25466	-0.25466	0.83625	0.00012	0.01794	0.19339	0.07468	0.03063	0.00091	0.00078	0.00078 brown
ZNF622	0.17371	-0.01744	-0.07829	-0.05162	-0.03856	-0.09824	0.16859	-0.13805	0.13805	0.02308	0.82089	0.30874	0.50253	0.61661	0.20112	0.02751	0.07175	0.07175 grey
MYSM1	-0.10159	-0.16686	0.00181	0.03368	-0.01507	0.04746	-0.03282	0.1902	-0.1902	0.18612	0.02916	0.9813	0.66191	0.84491	0.53759	0.66998	0.01271	0.01271 red
ANXA3	-0.06526	0.06049	0.13922	0.05612	-0.16691	0.30326	0.24537	-0.34403	0.34403	0.39645	0.43189	0.06935	0.46596	0.02911	5.53E-05	0.00122	4.08E-06	4.08E-06 yellow
HS3ST3A1	0.00858	-0.04361	0.12849	0.00125	-0.08701	0.0703	0.06127	0.0346	-0.0346	0.91136	0.57117	0.09397	0.98703	0.25781	0.36085	0.42602	0.65324	0.65324 black
CEACAM1	0.00721	-0.17088	-0.2509	-0.08461	0.28758	-0.32735	-0.16825	-0.00275	0.00275	0.92546	0.02544	0.00093	0.27123	0.00014	1.24E-05	0.02783	0.97156	0.97156 grey
SZRD1	-0.10554	-0.0899	0.00749	0.07415	-0.13956	0.08543	0.04978	0.16273	-0.16273	0.16951	0.24229	0.92257	0.33512	0.06868	0.2666	0.51788	0.03346	0.03346 turquoise
PHACTR3	0.04204	-0.1443	-0.06107	-0.01848	-0.03478	0.00851	0.04044	-0.27659	0.27659	0.58508	0.05971	0.42749	0.81044	0.65159	0.91206	0.59948	0.00025	0.00025 grey
GPSM2	-0.00423	-0.00722	0.0957	0.05328	-0.04706	0.14714	0.21492	-0.23792	0.23792	0.95625	0.92531	0.2131	0.48884	0.54105	0.0548	0.00476	0.00173	0.00173 pink
SLC20A1	0.03702	-0.08374	0.12116	0.04402	-0.16775	0.22073	0.15679	-0.1652	0.1652	0.63075	0.2762	0.11445	0.56757	0.0283	0.00372	0.04056	0.03082	0.03082 turquoise
NPIPB3	0.04828	-0.07699	0.06552	0.00137	0.05642	0.10358	-0.00501	-0.06191	0.06191	0.53063	0.31689	0.39451	0.98579	0.4636	0.1776	0.9481	0.42114	0.42114 red
ADGRA3	-0.19085	-0.18947	-0.01849	0.13061	-0.0049	0.10822	0.05865	0.02185	-0.02185	0.01241	0.01306	0.81033	0.08861	0.9493	0.15887	0.44606	0.77667	0.77667 turquoise
POLG	-0.02197	-0.07998	-0.02168	-0.01156	-0.00075	0.08226	0.07661	-0.05487	0.05487	0.77546	0.29838	0.77835	0.88074	0.9922	0.28482	0.31928	0.47595	0.47595 turquoise
IGF1R	-0.0976	-0.22708	-0.07485	-0.03429	-0.1768	-0.00148	0.00782	0.26802	-0.26802	0.2041	0.00282	0.33055	0.65616	0.02071	0.98463	0.9191	0.00039	0.00039 turquoise
F2RL2	-0.00766	-0.07204	-0.05347	-0.02653	-0.18756	0.15944	0.06223	0.26401	-0.26401	0.9208	0.34907	0.48735	0.73047	0.01403	0.03725	0.41872	0.00048	0.00048 blue
SLA	-0.14323	-0.04008	0.05478	0.07247	-0.28403	0.13864	0.0309	0.41059	-0.41059	0.06165	0.60272	0.47669	0.3462	0.00017	0.07054	0.68825	2.44E-08	2.44E-08 blue
RPL38	0.12602	0.03545	-0.04539	-0.09464	0.01293	-0.07983	0.10459	-0.23142	0.23142	0.1005	0.64525	0.55548	0.21822	0.86669	0.29932	0.1734	0.00232	0.00232 purple
PTPN12	-0.01364	-0.10971	0.1301	0.08245	-0.1333	0.21556	0.17568	-0.13229	0.13229	0.85946	0.15317	0.08989	0.28365	0.0822	0.00463	0.02154	0.08457	0.08457 turquoise
FGF10	-0.03695	-0.00372	0.02579	0.03031	-0.19436	0.09978	-0.05414	0.60922	-0.60922	0.63136	0.96151	0.73773	0.6939	0.01086	0.19411	0.48189	9.45E-19	9.45E-19 blue
ZNF318	-0.07832	-0.03419	-0.065	-0.00725	-0.07465	-0.04165	-0.05189	0.38916	-0.38916	0.30855	0.6571	0.39832	0.92497	0.33189	0.58856	0.50031	1.44E-07	1.44E-07 turquoise
RHEB	-0.05408	-0.13294	-0.03231	-0.04329	-0.03247	-0.12527	-0.01743	-0.04492	0.04492	0.48235	0.08302	0.67486	0.57395	0.67331	0.10257	0.82104	0.5596	0.5596 turquoise
RASD2	0.12455	0.0468	-0.04276	0.0617	0.08589	0.05788	0.07284	0.04701	-0.04701	0.10456	0.54327	0.57867	0.42273	0.26399	0.45206	0.34376	0.54151	0.54151 grey
PRRT2	0.00696	-0.16028	-0.08343	-0.08202	0.16147	-0.09086	0.01813	0.08183	-0.08183	0.92806	0.03625	0.27796	0.2862	0.03487	0.23724	0.81393	0.28732	0.28732 red
ZNF350	-0.06034	-0.09696	-0.05452	0.00802	-0.12911	0.04162	-0.02869	0.13079	-0.13079	0.43303	0.20708	0.47878	0.91711	0.09237	0.58887	0.70957	0.08818	0.08818 turquoise
TONSL	0.04284	0.07623	0.01187	-0.06302	0.08917	-0.01027	0.23158	-0.4616	0.4616	0.578	0.32169	0.87759	0.41285	0.24615	0.89391	0.00231	2.09E-10	2.09E-10 green
ZNF664	0.03113	-0.18679	-0.09916	-0.05276	0.09015	-0.09544	-0.12902	0.09016	-0.09016	0.68607	0.01444	0.19695	0.49312	0.24094	0.21435	0.09261	0.24091	0.24091 turquoise
IL10RA	-0.10664	-0.0322	0.05389	0.01702	-0.18213	0.06848	0.02042	0.49679	-0.49679	0.16508	0.67592	0.48389	0.82515	0.01712	0.3735	0.79097	4.82E-12	4.82E-12 blue
TYK2	-0.03422	-0.09231	-0.10973	-0.05773	0.15803	-0.07224	-0.07078	0.09207	-0.09207	0.6568	0.22984	0.1531	0.45324	0.03899	0.34778	0.35763	0.23106	0.23106 red
FAHD2B	-0.0761	0.03523	-0.11119	-0.04021	-0.07411	-0.06034	0.10708	0.06079	-0.06079	0.32255	0.64731	0.14768	0.60153	0.33541	0.43304	0.16331	0.42964	0.42964 grey
TIPIN	-0.17177	0.00093	-0.15514	-0.06627	-0.02825	-0.1492	0.02367	0.07621	-0.07621	0.02468	0.99035	0.04275	0.38917	0.71377	0.05146	0.75865	0.3218	0.3218 grey
BDH1	0.09245	0.11567	-0.06522	-0.20239	0.25781	-0.02183	-0.04873	-0.31964	0.31964	0.22911	0.13192	0.3967	0.00794	0.00066	0.77688	0.5268	2.03E-05	2.03E-05 grey
PSMB5	-0.02911	-0.10918	-0.09626	-0.03631	0.04637	-0.16153	0.02316	-0.12812	0.12812	0.70551	0.15518	0.2104	0.63728	0.54702	0.03481	0.76361	0.09492	0.09492 grey
LRIG1	-0.15319	0.07064	-0.00148	0.07721	-0.08446	0.15274	-0.13035	0.4203	-0.4203	0.04546	0.35858	0.98465	0.3155	0.27208	0.04611	0.08926	1.05E-08	1.05E-08 turquoise
KCTD1	-0.01136	-0.09342	0.00656	0.05025	-0.08531	0.17661	0.09881	-0.13675	0.13675	0.8828	0.22423	0.93217	0.51395	0.26724	0.02085	0.19853	0.07449	0.07449 grey
NR5A2	-0.00432	-0.0967	-0.03421	0.12142	-0.05097	0.11598	-0.00769	0.14322	-0.14322	0.95523	0.20832	0.65694	0.11364	0.50797	0.13088	0.92051	0.06166	0.06166 magenta
CLBA1	0.04019	-0.06244	-0.15906	-0.00301	0.22726	-0.1928	-0.01457	-0.24624	0.24624	0.60174	0.4172	0.03771	0.96883	0.0028	0.01152	0.84999	0.00117	0.00117 grey
NOTCH2	-0.05509	-0.10274	0.10546	0.17723	-0.26546	0.27656	0.18595	0.15115	-0.15115	0.47423	0.18116	0.16983	0.0204	0.00045	0.00025	0.01489	0.04845	0.04845 turquoise
RECQL	-0.10591	-0.12461	0.04954	0.05162	-0.19515	0.14281	0.05554	0.27789	-0.27789	0.16798	0.10441	0.51991	0.5025	0.01053	0.06241	0.47058	0.00023	0.00023 turquoise
FN3KRP	-0.06769	-0.09952	0.01907	-0.02632	-0.0113	0.04771	0.03199	-0.16403	0.16403	0.37905	0.19529	0.80451	0.73256	0.88343	0.53545	0.67783	0.03205	0.03205 turquoise
TMOD1	-0.03258	-0.12312	-0.24021	-0.08972	0.00271	-0.25307	-0.24119	0.4414	-0.4414	0.67229	0.10865	0.00155	0.24322	0.97196	0.00084	0.00148	1.51E-09	1.51E-09 brown
ELAC2	-0.04602	-0.0961	-0.11281	-0.09259	-0.06266	-0.22173	-0.01847	0.20718	-0.20718	0.55005	0.21119	0.14181	0.22841	0.41555	0.00356	0.81046	0.00655	0.00655 turquoise
FXYD6	-0.02744	-0.14608	-0.16961	-0.07362	0.04795	-0.265	-0.24802	0.58337	-0.58337	0.72169	0.05659	0.02657	0.33856	0.53341	0.00046	0.00107	5.63E-17	5.63E-17 brown
MRPL10	0.0265	-0.06627	-0.05319	0.00612	0.04753	-0.03218	0.05664	-0.24207	0.24207	0.73077	0.38917	0.48964	0.93664	0.537	0.67606	0.46184	0.00142	0.00142 turquoise

NCAPH2	0.05083	0.13082	-0.09739	-0.04663	0.06453	-0.16243	0.08955	-0.18367	0.18367	0.50908	0.08809	0.2051	0.54473	0.40175	0.03379	0.24411	0.01619	0.01619	grey
PAQR3	-0.0926	-0.09903	-0.04776	-0.01402	-0.12007	0.08401	0.03252	0.18629	-0.18629	0.22837	0.19751	0.53502	0.8556	0.11774	0.27465	0.67282	0.01471	0.01471	turquoise
DPYSL3	-0.07482	-0.06434	-0.00399	0.02353	-0.1427	0.11968	-0.02716	0.32247	-0.32247	0.33077	0.40315	0.95865	0.76004	0.06262	0.11896	0.72432	1.70E-05	1.70E-05	blue
APBA2	-0.10521	0.02026	0.04509	0.02915	-0.2003	0.09851	0.04287	0.19871	-0.19871	0.17084	0.79253	0.55818	0.70506	0.00862	0.19988	0.57774	0.00918	0.00918	black
ANTXR2	-0.04991	-0.01245	0.11316	0.05046	-0.12582	0.31435	0.1533	-0.10406	0.10406	0.51684	0.87162	0.14056	0.5122	0.10107	2.83E-05	0.04531	0.17558	0.17558	turquoise
MICOS10	0.01352	-0.03703	-0.05796	-0.04263	-0.00907	-0.08093	-0.04228	0.05129	-0.05129	0.86067	0.63059	0.45146	0.57984	0.9063	0.29269	0.58295	0.50527	0.50527	turquoise
SERPINI1	-0.04232	-0.04552	-0.09865	-0.0542	-0.01914	-0.24895	-0.2759	0.5205	-0.5205	0.58262	0.55436	0.19927	0.48136	0.80378	0.00103	0.00026	2.94E-13	2.94E-13	brown
UBE2J1	-0.12805	-0.06765	0.05578	0.0501	-0.11687	0.0144	-0.06277	0.39963	-0.39963	0.09512	0.37928	0.4687	0.51522	0.12795	0.85168	0.41475	6.13E-08	6.13E-08	turquoise
THOC1	-0.05539	-0.21502	-0.04146	-0.01628	0.15797	-0.01883	0.03075	-0.10013	0.10013	0.47179	0.00474	0.59027	0.83262	0.03906	0.80689	0.68975	0.19258	0.19258	red
EOMES	-0.06354	-0.00089	0.03458	0.06778	-0.1482	0.08658	-0.02438	0.43612	-0.43612	0.40898	0.99076	0.65346	0.37837	0.05306	0.2602	0.75164	2.49E-09	2.49E-09	blue
KIAA1324	0.0154	-0.00607	-0.21488	-0.12715	0.16229	-0.2261	-0.23847	0.33974	-0.33974	0.84152	0.93716	0.00477	0.09747	0.03394	0.00294	0.00168	5.47E-06	5.47E-06	brown
REM2	0.06147	-0.13942	-0.14266	-0.15258	0.19829	-0.33556	-0.23602	0.0326	-0.0326	0.42449	0.06896	0.0627	0.04634	0.00933	7.24E-06	0.00189	0.6721	0.6721	brown
MZT2B	0.0343	0.03563	-0.09446	-0.02295	0.18749	-0.08532	0.06308	-0.29135	0.29135	0.65604	0.64362	0.2191	0.76574	0.01407	0.2672	0.41244	0.00011	0.00011	green
MCL1	-0.08269	-0.13283	-0.00135	0.00404	-0.15152	0.20227	0.09495	0.05149	-0.05149	0.28229	0.08328	0.98597	0.95819	0.0479	0.00798	0.21673	0.5036	0.5036	turquoise
POPDC3	0.01905	-0.02888	-0.08767	-0.03985	0.0054	-0.31288	-0.08274	0.25336	-0.25336	0.80471	0.7077	0.25421	0.60486	0.9441	3.09E-05	0.28199	0.00083	0.00083	grey
G2E3	0.00443	-0.09388	0.05942	0.08432	-0.09112	0.07006	0.06454	0.0432	-0.0432	0.95418	0.22194	0.44014	0.27285	0.2359	0.36255	0.40165	0.57476	0.57476	turquoise
ULK1	0.03295	-0.12201	-0.15981	-0.02376	0.18357	-0.19263	-0.10194	0.04787	-0.04787	0.66876	0.11188	0.03681	0.7577	0.01624	0.0116	0.18462	0.53412	0.53412	grey
EXTL2	-0.13784	-0.11423	-0.05467	0.00663	-0.12126	-0.02787	0.05182	0.40181	-0.40181	0.0722	0.13685	0.47759	0.93142	0.11414	0.71747	0.50084	5.12E-08	5.12E-08	blue
PBX1	0.01197	-0.06521	-0.00749	0.07167	-0.14325	0.09247	0.01143	0.38335	-0.38335	0.87654	0.39676	0.92251	0.35155	0.0616	0.22902	0.882	2.28E-07	2.28E-07	turquoise
PNMA6A	0.07402	-0.00515	-0.11702	0.02663	-0.0476	-0.09963	0.03659	0.15622	-0.15622	0.33596	0.94668	0.12746	0.72954	0.53639	0.19482	0.63473	0.04132	0.04132	grey
SLC35D3	-0.03264	0.03751	-0.10825	-0.04932	0.07607	-0.08224	-0.00903	0.23337	-0.23337	0.67168	0.62616	0.15875	0.52178	0.32275	0.28489	0.90672	0.00213	0.00213	grey
EP400	0.00644	-0.14045	-0.04985	-0.0205	0.01163	-0.04467	-0.06193	0.18933	-0.18933	0.93335	0.06692	0.51729	0.79017	0.87999	0.56182	0.421	0.01313	0.01313	turquoise
ADAMTSL	-0.05697	-0.01463	0.15785	0.08097	-0.11371	0.19838	0.16868	0.03757	-0.03757	0.45919	0.84934	0.03922	0.29243	0.13865	0.0093	0.02743	0.62565	0.62565	black
C19orf73	-0.03829	0.05112	-0.20024	-0.22427	0.2518	-0.15773	-0.12428	-0.09485	0.09485	0.61901	0.50665	0.00864	0.00319	0.00089	0.03937	0.10535	0.21718	0.21718	brown
SORBS2	-0.04953	-0.12924	-0.21252	0.04236	0.02478	-0.1412	-0.17474	0.27036	-0.27036	0.52003	0.09203	0.00526	0.58222	0.74764	0.06546	0.02226	0.00035	0.00035	grey
TRMT44	-0.01352	-0.08324	-0.03469	-0.04224	0.03209	0.09077	0.08595	-0.08144	0.08144	0.86073	0.27908	0.65239	0.5833	0.67697	0.2377	0.26366	0.28962	0.28962	grey
ZNF486	0.05435	-0.07056	0.01423	0.1452	-0.16759	0.15461	0.04976	0.21273	-0.21273	0.48019	0.35908	0.85342	0.05812	0.02846	0.04348	0.51811	0.00521	0.00521	grey
ZC3H8	0.13956	0.00872	-0.04307	-0.01066	0.03578	-0.03126	-0.01294	0.0091	-0.0091	0.06868	0.90984	0.57595	0.88995	0.6422	0.68482	0.86664	0.90597	0.90597	turquoise
DENND1A	-0.10771	-0.1172	-0.19539	-0.06093	-0.04407	-0.19399	-0.10375	0.22894	-0.22894	0.16083	0.12686	0.01044	0.42853	0.56712	0.01101	0.1769	0.0026	0.0026	turquoise
GBP5	-0.15159	0.11302	0.07449	0.05786	-0.26775	0.10242	0.16467	0.27211	-0.27211	0.04779	0.14108	0.33292	0.45223	0.0004	0.18255	0.03138	0.00032	0.00032	blue
RARRES1	0.03448	0.02116	0.1087	0.13936	-0.25379	0.1729	0.1966	0.05481	-0.05481	0.65432	0.78354	0.15702	0.06908	0.00081	0.02373	0.00996	0.47648	0.47648	grey
PRPH2	0.04734	-0.09262	-0.09072	0.01697	-0.10541	-0.01511	0.07455	0.49371	-0.49371	0.53863	0.22824	0.23799	0.82565	0.17001	0.84449	0.33254	6.81E-12	6.81E-12	blue
ENTPD6	0.10315	0.0251	0.01021	0.01399	0.22155	-0.09096	0.09441	-0.36397	0.36397	0.1794	0.74454	0.89453	0.85589	0.00359	0.23675	0.21936	9.90E-07	9.90E-07	grey
CFB	-0.0101	0.0447	0.01174	0.0898	-0.06788	0.17394	0.1745	-0.19315	0.19315	0.89566	0.56158	0.8789	0.24278	0.37773	0.02289	0.02245	0.01137	0.01137	grey
STMP1	-0.07234	-0.1366	-0.06249	-0.03512	0.06062	-0.18185	-0.05798	-0.02233	0.02233	0.34711	0.07482	0.41683	0.64837	0.43093	0.01729	0.45131	0.77185	0.77185	turquoise
ANAPC1	0.0196	0.01258	0.03319	0.03432	-0.20594	0.10837	0.15945	0.08169	-0.08169	0.79912	0.87031	0.66648	0.65588	0.00689	0.15827	0.03724	0.28818	0.28818	turquoise
CDO1	-0.05689	-0.18776	-0.10625	0.02432	-0.03319	-0.07751	-0.24658	0.56227	-0.56227	0.45988	0.01393	0.16662	0.75216	0.66654	0.31359	0.00115	1.22E-15	1.22E-15	brown
ASGR1	0.00286	-0.01755	-0.19269	-0.06933	0.10021	-0.21577	-0.09543	0.04754	-0.04754	0.97034	0.81981	0.01157	0.36755	0.19221	0.00459	0.21438	0.53695	0.53695	grey
TNPO3	0.0348	-0.09529	0.07283	0.04916	-0.08721	0.05338	0.10832	-0.09962	0.09962	0.65132	0.21505	0.34382	0.52317	0.25669	0.48808	0.15847	0.19486	0.19486	turquoise
PIGO	0.07263	-0.09067	-0.0647	-0.07828	-0.02994	0.02973	0.05174	-0.02386	0.02386	0.34513	0.23822	0.40053	0.30882	0.6975	0.69951	0.50154	0.7567	0.7567	turquoise
NDUFA3	0.11444	0.06353	-0.13483	-0.15285	0.13576	-0.13878	-0.02179	-0.29384	0.29384	0.13612	0.40912	0.07871	0.04595	0.07666	0.07025	0.77723	9.59E-05	9.59E-05	grey
CTXND1	-0.05862	-0.11664	-0.22519	-0.08749	0.20942	-0.15537	-0.15948	0.34235	-0.34235	0.44629	0.1287	0.00306	0.25517	0.00598	0.04245	0.03721	4.58E-06	4.58E-06	grey
NR6A1	0.03712	-0.1377	0.07289	0.14588	-0.0466	0.07806	0.12032	-0.24491	0.24491	0.62979	0.07249	0.34342	0.05694	0.54499	0.31017	0.117	0.00124	0.00124	grey
MARVELD	0.02971	-0.02396	0.07132	0.0712	-0.20306	0.11591	0.19711	0.07434	-0.07434	0.69969	0.75571	0.35394	0.35478	0.00773	0.13112	0.00976	0.33391	0.33391	black
CPT2	-0.19558	0.02261	-0.04816	-0.08354	0.06656	-0.00804	0.04481	0.119	-0.119	0.01036	0.76907	0.5316	0.27732	0.38705	0.91683	0.56058	0.12107	0.12107	turquoise
PI4K2A	-0.02535	-0.1536	-0.03051	-0.03565	-0.18022	-0.06132	0.08365	0.22759	-0.22759	0.74203	0.04489	0.69204	0.64344	0.01834	0.42562	0.27673	0.00276	0.00276	blue
C14orf93	0.07898	-0.04523	-0.17927	-0.12534	0.21747	-0.18274	-0.07328	-0.03722	0.03722	0.3045	0.55693	0.01897	0.10237	0.00427	0.01674	0.34084	0.62884	0.62884	grey
NREP	-0.05022	-0.10257	-0.03708	0.01382	-0.16234	0.0998	0.00114	0.1809	-0.1809	0.51424	0.18188	0.63021	0.8576	0.03388	0.19405	0.98815	0.0179	0.0179	black
MAFK	0.00621	-0.17208	0.02082	0.02123	0.06376	0.08021	-0.02669	-0.19186	0.19186	0.93576	0.02441	0.7869	0.78289	0.4074	0.29703	0.72891	0.01194	0.01194	grey
PLCD4	-0.058	-0.07498	-0.18339	-0.03724	0.06994	-0.15541	-0.18661	0.31738	-0.31738	0.45114	0.3297	0.01635	0.62874	0.36334	0.04239	0.01453	2.34E-05	2.34E-05	brown
STK35	-0.00158	-0.10336	-0.1141	-0.03189	-0.02749	-0.09681	0.01217	0.01556	-0.01556	0.98362	0.17851	0.13727	0.6788	0.72114	0.20782	0.87447	0.83995	0.83995	turquoise
KIF1B	-0.07427	-0.1893	-0.0495	0.04466	-0.11149	0.0164	-0.04392	0.36549	-0.36549	0.33435	0.01315	0.5203	0.56195	0.14658	0.83137	0.56845	8.85E-07	8.85E-07	turquoise
SIPA1	-0.046	-0.06789	0.02803	0.07647	-0.14636	0.1012	0.14918	0.02216	-0.02216	0.55026	0.37763	0.71594	0.32018	0.05611	0.18783	0.0515	0.77359	0.77359	blue
PTGR1	0.02181	-0.12096	0.03805	0.02269	-0.09477	0.12841	0.15008	-0.05891	0.05891	0.77709	0.11504	0.62119	0.76835	0.21757	0.09417	0.05007	0.44403	0.44403	turquoise
RPS24	0.09248	0.12521	0.03388	-0.0243	-0.03373	0.06191	0.20757	-0.34588	0.34588	0.22896	0.10274	0.65997	0.75239	0.66141	0.42115	0.00645	3.59E-06	3.59E-06	purple
MFN2	-0.0365	-0.10544	0.0305	0.02213	-0.0941	0.07609	-0.02599	0.16305	-0.16305	0.63556	0.16992	0.69212	0.7739	0.22087	0.32259	0.73578	0.03311	0.03311	turquoise
RBMS2	-0.02235	-0.14128	0.00069	0.07761	-0.21785	0.1564	0.13105	0.02509	-0.02509	0.77168	0.0653	0.99286	0.31299	0.0042	0.04107	0.08755	0.74466	0.74466	turquoise
LSAMP	0.00291	-0.08796	-0.02172	0.01879	-0.16849	0.10972	-0.038	0.5098	-0.5098	0.96983	0.25262	0.77796	0.8073	0.02761	0.15313	0.62171	1.07E-12	1.07E-12	blue
GCK	0.0024	-0.08352	-0.21899	-0.1417	0.07771	-0.29493	-0.15367	0.42299	-0.42299	0.9751	0.27743	0.00401	0.06449	0.31234	9.00E-05	0.04479	8.24E-09	8.24E-09	brown
RPL9	0.10775	0.04063	-0.07357	-0.09638	-0.04341	0.02062	0.0388	-0.05884	0.05884	0.16068	0.59771	0.3389	0.20983	0.5729	0.78896	0.61434	0.44458	0.44458	purple
GPR171	-0.14389	-0.0517	0.04898	0.08554	-0.18195	0.122	-0.01448	0.44367	-0.44367	0.06043	0.50188	0.52469	0.26599	0.01723	0.11193	0.85089	1.22E-09	1.22E-09	blue
RXRB	-0.01849	-0.02496	-0.10906	-0.03294	0.12964	-0.09797	-0.01344	0.1394	-0.1394	0.81033	0.74585	0.15562	0.6689	0.09104	0.20236	0.86154	0.06901	0.06901	grey
ABHD5	-0.04576	-0.156	0.06993	0.01334	-0.19299	0.20434	0.14329	0.06446	-0.06446	0.55235	0.0416	0.36345	0.86253	0.01144	0.00734	0.06152	0.40221	0.40221	turquoise
CREG1	-0.05654	-0.04215	0.09611	0.00785	-0.16614	0.17505	0.16631	0.14738	-0.14738	0.46263	0.58413	0.21114	0.9188	0.02987	0.02203	0.0297	0.0544	0.0544	turquoise
GCNT2	-0.02052	0.03095	0.09051	0.08124	-0.02245	0.03488	0.10088	-0.05637	0.05637	0.78991	0.68777	0.23909	0.29083	0.77073	0.65057	0.18925	0.46395	0.46395	grey
POLR2I	0.10007	-0.02357	-0.17898	-0.05983	0.14585	-0.2463	0.0086	-0.30094	0.30094	0.19283	0.75958	0.01917	0.43699	0.05699	0.00117	0.91106	6.35E-05	6.35E-05	green
SNRNP35	0.00436	-0.00934	-0.10424	-0.13084	0.22012	-0.16084	-0.06002	-0.10644	0.10644	0.95489	0.9035	0.17483	0.08806	0.00382	0.0356	0.4355	0.16588	0.16588	grey
LIMD2	-0.16261	-0.01693	0.00162	-0.00692	-0.14126	0.03244	-0.03967	0.2775	-0.2775	0.03359	0.82606	0.98325	0.92845	0.06533	0.67356	0.60647	0.00024	0.00024	blue
DCAF7	-0.0843	-0.22633	-0.03761	-0.01268	-0.08447	-0.02551	-0.0303	0.20281	-0.20281	0.27296	0.00291	0.62527	0.86923	0.27199	0.74055	0.69399	0.00781	0.00781	turquoise
RHOC	-0.01063	-0.0524	0.06414	0.02112	-0.07104	0.23174	0.19776	-0.37404	0.37404	0.89027	0.49612	0.40457	0.78393	0.35585	0.00229	0.00952	4.67E-07	4.67E-07	yellow
CDON	-0.03988	-0.05644	-0.09782	0.0214	-0.11225	-0.12412	-0.05439	0.40803	-0.40803	0.60454	0.4634	0.20306	0.7812	0.14381	0.10577	0.47984	3.03E-08	3.03E-08	turquoise
RIPPLY3	0.09042	-0.11946	-0.23525	-0.06577	0.08321	-0.15867	0.02402	-0.13206	0.13206	0.23953	0.11964	0.00195	0.39274	0.27924	0.03819	0.75517	0.08512	0.08512	grey
CDS2	-0.03024	-0.17396	-0.16285	-0.03229	0.01209	-0.29894	-0.168	0.36703	-0.36703	0.69456	0.02287	0.03332	0.67501	0.87524	7.14E-05	0.02806	7.90E-07	7.90E-07	brown
LRRC32	-0.06263	-0.1493	-0.01797	0.03898	-0.19341	0.11782	0.00161	0.32847	-0.32847	0.41576	0.0513	0.81554	0.61274	0.01126	0.12483	0.9833	1.15E-05	1.15E-05	black
DCP2	0.01008	-0.19802	0.00793	0.03274	-0.15589	0.06755	0.04976	0.18603	-0.18603	0.89589	0.00942	0.91801	0.67077	0.04175	0.38	0.51806	0.01484	0.01484	turquoise
CALHM2	-0.07026	-0.07066	0.07904	0.1206	-0.13195	0.09353	0.02586	0.25649	-0.25649	0.36119	0.35845	0.30414	0.11614	0.08538	0.22368	0.73708	0.00071	0.00071	blue
UQCRC1	0.09941	0.09263	-0.01155	-0.11188	0.06605	0.05106	0.1611	-0.41484	0.41484	0.19578	0.22821	0.88081	0.14515	0.39072	0.50719	0.03529	1.69E-08	1.69E-08	grey
ZMYND10	0.01015	-0.1419	-0.18936	-0.05537	0.13994	-0.14641	-0.08429	-0.049	0.049	0.89518	0.06413	0.01312	0.47193	0.06793	0.05603	0.27304	0.52446	0.52446	grey
CENPE	-0.09199	0.01475	0.06318	-0.00125	-0.10402	0.12646	0.29059	-0.32029	0.32029	0.23144	0.84818	0.41164	0.98701	0.17575	0.09931	0.00012	1.95E-05	1.95E-05	pink
EIF2AK2	-0.03354	-0.10328	0.01959	0.0496	-0.15273	0.08981	0.10582	0.0613	-0.0613	0.66319	0.17887	0.79929	0.51946	0.04612	0.24273	0.16837	0.42574	0.42574	tan
PRICKLE3	0.09446	-0.09231	0.05934	0.037	-0.06502	0.09105	0.18963	-0.44878	0.44878	0.21909	0.22984	0.44075	0.63087	0.39819	0.23626	0.01299	7.45E-10	7.45E-10	yellow
ENDOV	-0.0181	-0.20287	-0.19834	-0.06998	0.18919	-0.14251	-0.00919	-0.11255	0.11255	0.81419	0.00779	0.00931	0.36308	0.0132	0.06298	0.90501	0.14275	0.14275	grey
INTS4	0.0795	-0.07123	0.06519	0.01095	-0.10885	0.05837	0.13439	-0.12101	0.12101	0.30132	0.35454	0.3969	0.88693	0.15644	0.44826	0.07971	0.11488	0.11488	turquoise
MINDY1	0.09854	-0.16239	-0.07796	-0.02504	0.05882	-0.07337	0.03282	0.05162	-0.05162	0.19975	0.03383	0.31081	0.74509	0.44472	0.34024	0.66996	0.50257	0.50257	grey
XDH	0.01793	-0.00143	0.06054	0.01755	-0.04907	0.15915	0.2347	-0.3908	0.3908	0.81595	0.98523	0.43155	0.81982	0.52389	0.03761	0.002	1.26E-07	1.26E-07	yellow
INKA1	-0.09792	-0.01023	-0.12274	-0.03933	-0.00643	-0.14398	-0.13331	0.32235	-0.32235	0.20263	0.89435	0.10976	0.60952	0.9335	0.06028	0.08216	1.71E-05	1.71E-05	grey
ZDHHC20	0.00671	-0.0768	0.04085	-0.00184	-0.09896	0.19291	0.14315	-0.16836	0.16836	0.93054	0.31809	0.59575	0.98096	0.19784	0.01147	0.06178	0.02773	0.02773	turquoise
ANKRD42	0.03928	-0.19953	-0.06814	0.01835	0.02167	0.00931	0.02332	0.02068	-0.02068	0.60997	0.00888	0.37586	0.81173	0.77847	0.90377	0.76212	0.78832	0.78832	turquoise
SLC43A2	-0.02587	-0.14167	-0.08681	-0.09467	0.05865	-0.24318	-0.1814	0.37346	-0.37346	0.737	0.06456	0.25888	0.2181	0.44605	0.00135	0.01758	4.88E-07	4.88E-07	brown
RTEL1-TN	-0.03722	0.01558	-0.02638	-0.04394	0.20679	-0.08277	0.06074	-0.222	0.222	0.62887	0.83973	0.73202	0.56824	0.00666	0.28183	0.43002	0.00352	0.00352	green
C9orf78	-0.00216	-0.11317	-0.09038	-0.06253	-0.12437	-0.03528	-0.03855	0.17285	-0.17285	0.97758	0.14054	0.23975	0.41652	0.10509	0.64686	0.6167	0.02377	0.02377	turquoise
BMP8A	-0.05612	-0.13311	-0.04541	0.04211	-0.23302	0.0125	0.0577	0.34591	-0.34591	0.46595	0.08264	0.55538	0.58444	0.00216	0.87106	0.45346	3.59E-06	3.59E-06	blue
SMARCD1	0.09302	-0.15857	-0.08082	-0.02904	0.04448	-0.12922	-0.10013	0.01803	-0.01803	0.22626	0.03831	0.29334	0.70612	0.56344	0.09209	0.19256	0.81498	0.81498	turquoise
ABCC1	-0.03542	-0.02468	0.11638	0.01981	-0.1423	0.16056	0.07438	-0.0588	0.0588	0.64553	0.74862	0.12956	0.79705	0.06337	0.03592	0.33361	0.44494	0.44494	turquoise
STAT1	-0.06579	0.04644	0.01671	0.0938	-0.19054	0.12384	0.1928	0.03354	-0.03354	0.39256	0.5464	0.82828	0.22237	0.01255	0.10657	0.01152	0.66323	0.66323	tan
IL18BP	-0.13231	-0.00312	0.00994	0.04536	-0.15694	0.09744	0.0101	0.28885	-0.28885	0.08452	0.96769	0.89734	0.55581	0.04037	0.20483	0.89573	0.00013	0.00013	blue
PRX	-0.01529	-0.28274	-0.05205	0.13886	-0.14901	-0.11864	0.0163	0.2298	-0.2298	0.8427	0.00018	0.49896	0.0701	0.05176	0.12221	0.83245	0.0025	0.0025	grey
COQ8B	0.08854	-0.17337	-0.20978	-0.09057	0.18	-0.19265	-0.01554	-0.14934	0.14934	0.24948	0.02335	0.00589	0.23878	0.01848	0.01159	0.84016	0.05123	0.05123	grey
ZC3H18	0.03485	-0.07844	-0.02606	-0.03443	0.00876	0.05614	0.06785	-0.1091	0.1091	0.65088	0.30785	0.73512	0.65485	0.9095	0.46579	0.37793	0.15549	0.15549	turquoise
ZNF500	0.02957	-0.08335	-0.12245	-0.06695	0.01652	-0.01983	0.04639	0.11525	-0.11525	0.70101	0.27841	0.1106	0.3843	0.83016	0.79683	0.54687	0.13335	0.13335	turquoise
TSC2	0.06664	-0.04987	-0.03937	-0.06996	0.12053	-0.03487	0.00487	-0.02453	0.02453	0.38647	0.5171	0.60916	0.36321	0.11635	0.6507	0.94956	0.75017	0.75017	green
POMC	-0.0835	0.02417	-0.12146	-0.06098	0.04775	-0.12887	-0.16006	0.36638	-0.36638	0.27757	0.7537	0.11354	0.42822	0.53512	0.093	0.0365	8.29E-07	8.29E-07	grey
METTL22	-0.03018	-0.05119	-0.0322	0.02002	0.05931	-0.05969	-0.03488	-0.08684	0.08684	0.6952	0.50614	0.67586	0.79495	0.44094	0.43802	0.65064	0.25872	0.25872	red
STRIP1	-0.02302	-0.14543	-0.03008	-0.03124	-0.02188	-0.01986	-0.02873	0.26257	-0.26257	0.76507	0.05771	0.69609	0.685	0.77635	0.79657	0.70914	0.00052	0.00052	turquoise
ZNF598	0.05772	0.07461	0.00504	-0.0337	0.11183	0.0707	0.09484	-0.25687	0.25687	0.45335	0.33211	0.94787	0.66173	0.14532	0.35818	0.21724	0.0007	0.0007	green
HSPA1L	0.01267	-0.17459	-0.15817	-0.07232	-0.01056	-0.11747	-0.08165	0.23676	-0.23676	0.86937	0.02238	0.03881	0.34719	0.89094	0.12597	0.2884	0.00182	0.00182	turquoise
RILP	0.04522	0.08793	0.16895	0.01944	-0.08089	0.25414	0.2667	-0.28378	0.28378	0.55703	0.25276	0.02718	0.80071	0.29293	0.0008	0.00042	0.00017	0.00017	grey
JMJD7-PL	-0.00251	-0.10673	0.00294	0.04995	0.1185	-0.01638	0.01	-0.03517	0.03517	0.97402	0.1647	0.96957	0.51649	0.12266	0.83165	0.89675	0.64789	0.64789	red
ZNF532	-0.09399	-0.13273	-0.00892	0.08626	-0.22302	0.07507	0.0765	0.2238	-0.2238	0.22143	0.08352	0.90777	0.26192	0.00337	0.32915	0.31996	0.00326	0.00326	black
STRA6	-0.03501	-0.10419	0.06145	0.08949	-0.13985	0.09449	0.08095	-0.10381	0.10381	0.64943	0.17504	0.4246	0.24441	0.06811	0.21895	0.29258	0.17662	0.17662	black
CDH11	-0.08467	-0.04444	0.07361	0.07415	-0.24201	0.22082	0.09372	0.23547	-0.23547	0.27084	0.56382	0.33865	0.33512	0.00143	0.0037	0.22275	0.00193	0.00193	black
ATP2A3	0.0009	-0.04665	-0.16092	-0.15224	0.24335	-0.15621	-0.29285	0.25688	-0.25688	0.99067	0.54458	0.03551	0.04684	0.00134	0.04132	0.0001	0.00069	0.00069	brown
ECE2	0.03049	0.09884	-0.23696	-0.08113	0.00347	-0.16235	0.01408	0.03145	-0.03145	0.69217	0.19836	0.00181	0.29149	0.96406	0.03388	0.85498	0.68299	0.68299	grey
HCFC2	-0.04689	-0.15684	-0.07396	-0.00417	-0.01926	-0.11718	-0.12322	0.50494	-0.50494	0.54256	0.0405	0.3364	0.95682	0.8026	0.12692	0.10837	1.89E-12	1.89E-12	turquoise
ZNF700	0.02663	-0.06227	-0.05497	-0.0193	0.17317	0.03393	-0.01529	-0.08021	0.08021	0.72951	0.41846	0.47514	0.80219	0.02351	0.65955	0.84267	0.29698	0.29698	red
CMTM3	-0.08628	-0.13024	0.03094	0.09105	-0.22813	0.13619	0.09564	0.02448	-0.02448	0.26184	0.08953	0.68786	0.23625	0.00269	0.0757	0.21338	0.75062	0.75062	black
ACAD8	-0.07771	-0.07877	-0.10289	-0.10821	0.03472	-0.03058	0.03088	0.06895	-0.06895	0.31239	0.30582	0.18051	0.15888	0.65209	0.69136	0.68846	0.37019	0.37019	turquoise
NKX3-2	-0.01435	-0.01793	0.04215	0.06141	-0.11176	0.13727	0.09771	0.08202	-0.08202	0.85222	0.81595	0.5841	0.4249	0.14557	0.07341	0.20359	0.28623	0.28623	black
KCNE3	-0.07541	0.06843	0.13677	0.08123	0.08379	0.31356	0.1119	-0.20848	0.20848	0.32694	0.37382	0.07446	0.2909	0.27591	2.97E-05	0.14507	0.00621	0.00621	yellow
ZNF528	-0.02524	-0.03962	-0.04799	#######	0.00913	0.05566	0.03727	0.07553	-0.07553	0.74316	0.60686	0.53304	0.99917	0.90566	0.46965	0.62844	0.32617	0.32617	red
NOMO3	-0.01694	-0.05147	-0.00854	0.02798	-0.16055	0.08757	0.03256	0.33951	-0.33951	0.82598	0.5038	0.91172	0.71638	0.03593	0.25474	0.6725	5.55E-06	5.55E-06	grey
NEK2	0.02145	0.05958	0.02725	-0.00019	-0.04572	0.10347	0.3127	-0.48747	0.48747	0.78064	0.43886	0.72352	0.99799	0.55266	0.17808	3.13E-05	1.36E-11	1.36E-11	pink
CTHRC1	-0.04545	-0.02942	0.12287	0.10953	-0.19417	0.1651	0.10956	0.11016	-0.11016	0.55496	0.70252	0.10936	0.15385	0.01094	0.03093	0.15373	0.15149	0.15149	black
PCBP1	-0.02094	-0.04892	0.01109	0.04134	-0.00864	0.12593	0.01129	-0.03627	0.03627	0.7857	0.52514	0.88557	0.59135	0.91071	0.10074	0.88352	0.63769	0.63769	turquoise
TMEM260	0.06261	-0.15024	0.0007	0.04635	0.11282	-0.18419	-0.11958	0.12567	-0.12567	0.41595	0.04984	0.99279	0.54721	0.14178	0.01588	0.11926	0.10145	0.10145	turquoise
TAF3	0.03868	-0.09185	0.00796	0.04177	-0.18385	0.05821	0.00578	0.29914	-0.29914	0.61544	0.23217	0.91774	0.58754	0.01608	0.44947	0.94016	7.06E-05	7.06E-05	turquoise
FOXD1	0.02159	-0.05342	0.06651	0.0707	-0.11542	0.08632	0.29726	-0.29742	0.29742	0.7793	0.48771	0.38738	0.35818	0.13278	0.26162	7.87E-05	7.80E-05	7.80E-05	grey
AOC2	-0.01247	-0.13094	-0.08549	0.04147	0.03844	-0.0598	-0.13653	0.25031	-0.25031	0.87136	0.08781	0.26622	0.59021	0.61764	0.43722	0.07497	0.00096	0.00096	grey
MANBA	-0.00934	-0.07087	0.13195	0.1709	-0.22449	0.20095	0.22155	-0.04641	0.04641	0.90345	0.35698	0.08538	0.02543	0.00316	0.0084	0.00359	0.54666	0.54666	turquoise

ALDH3A1	-0.03325	0.19164	0.14266	0.03794	0.03186	0.22018	0.10552	-0.30381	0.30381	0.66592	0.01204	0.06269	0.62221	0.67907	0.00381	0.16959	5.35E-05	5.35E-05	grey
C9orf16	0.05732	0.01678	-0.13617	-0.11575	0.02837	-0.09328	0.10632	-0.32649	0.32649	0.45648	0.82751	0.07576	0.13166	0.71263	0.22493	0.16636	1.31E-05	1.31E-05	green
SCN2B	-0.02473	-0.0546	-0.02208	0.06447	-0.10175	0.13691	-0.01789	0.55682	-0.55682	0.74819	0.47819	0.77434	0.4022	0.18543	0.07416	0.81633	2.61E-15	2.61E-15	blue
CRIP1	0.03203	0.06566	0.03084	-0.01917	0.2212	0.09108	0.01426	-0.42162	0.42162	0.67754	0.39351	0.68884	0.80349	0.00364	0.23614	0.85312	9.30E-09	9.30E-09	grey
LRRC41	-0.08314	-0.17941	-0.06087	-0.01459	-0.02696	-0.03037	-0.03714	0.20888	-0.20888	0.27967	0.01888	0.42903	0.84975	0.72636	0.69334	0.6296	0.00611	0.00611	turquoise
CASP4	0.02114	0.00338	0.10664	0.09816	-0.19644	0.23854	0.28333	-0.26435	0.26435	0.7837	0.96498	0.16505	0.20148	0.01002	0.00168	0.00017	0.00048	0.00048	turquoise
SYCE1L	0.02111	-0.14777	-0.17304	0.016	0.15374	-0.16012	-0.03945	-0.14577	0.14577	0.78402	0.05376	0.02362	0.83542	0.04469	0.03644	0.60847	0.05712	0.05712	green
POLR1D	0.19345	0.06053	-0.05676	-0.05424	-0.05558	-0.02849	0.11281	-0.33075	0.33075	0.01124	0.43161	0.46092	0.48102	0.4703	0.71144	0.14181	9.94E-06	9.94E-06	purple
TESMIN	0.01061	-0.07012	-0.06071	0.01803	-0.01056	-0.11606	0.17327	-0.3515	0.3515	0.89042	0.36208	0.43021	0.81496	0.89096	0.13062	0.02343	2.43E-06	2.43E-06	grey
RNF112	0.00377	-0.0753	-0.01394	0.089	0.05591	0.05468	0.05634	0.28036	-0.28036	0.96093	0.32767	0.85641	0.24702	0.46763	0.47753	0.46421	0.0002	0.0002	blue
RAN	0.02772	0.0073	0.02658	-0.03443	-0.01807	-0.02173	0.05256	-0.12382	0.12382	0.71888	0.92449	0.73002	0.65488	0.81451	0.77788	0.4948	0.10664	0.10664	turquoise
SLC66A3	-0.09091	-0.02771	0.09349	0.15478	-0.25974	0.3114	0.18762	0.00483	-0.00483	0.23699	0.71898	0.22387	0.04324	0.0006	3.39E-05	0.014	0.95005	0.95005	turquoise
PPT2	-0.05322	-0.11941	-0.1058	-0.04227	0.02188	-0.2673	-0.06933	0.29984	-0.29984	0.48936	0.11979	0.16843	0.58306	0.7764	0.00041	0.36753	6.77E-05	6.77E-05	brown
PHGDH	0.11838	0.02271	0.05319	0.06046	-0.19761	0.11633	0.12658	-0.0888	0.0888	0.12306	0.7681	0.48963	0.43212	0.00958	0.12971	0.09898	0.2481	0.2481	grey
CELSR3	0.16941	0.00722	-0.15643	-0.08091	0.11316	-0.25296	-0.02632	-0.02027	0.02027	0.02676	0.92535	0.04103	0.29279	0.14058	0.00084	0.73259	0.79243	0.79243	grey
TENT5B	-0.05168	0.01334	-0.01416	0.12458	-0.04619	-0.01574	0.04464	0.1224	-0.1224	0.50203	0.86251	0.85415	0.1045	0.54859	0.83812	0.56207	0.11074	0.11074	grey
ANO4	-0.01331	-0.12013	-0.0034	0.0359	-0.00313	0.12789	0.0535	0.14046	-0.14046	0.86279	0.11756	0.96483	0.64111	0.96759	0.09553	0.48707	0.0669	0.0669	black
EFHC1	0.03927	-0.19477	-0.15604	-0.06176	0.2269	-0.15962	-0.14432	0.08954	-0.08954	0.61013	0.01069	0.04155	0.42227	0.00284	0.03703	0.05966	0.24419	0.24419	grey
CRIP3	-0.02441	-0.01466	-0.19441	-0.10014	0.14169	-0.24895	-0.30426	0.29425	-0.29425	0.75128	0.84905	0.01084	0.1925	0.06452	0.00103	5.21E-05	9.37E-05	9.37E-05	brown
PTS	-0.00451	-0.05302	-0.1008	-0.1693	0.19227	-0.20472	0.0001	-0.10297	0.10297	0.95327	0.49101	0.18957	0.02685	0.01175	0.00723	0.99895	0.18017	0.18017	grey
ABCF2	-0.02427	-0.04343	-0.05411	-0.05395	-0.09267	-0.10131	0.09448	0.09062	-0.09062	0.75265	0.57272	0.48211	0.48338	0.22799	0.18736	0.219	0.23848	0.23848	turquoise
SLC39A10	-0.03899	-0.1085	-0.0415	0.06625	-0.18367	0.06117	0.12939	-0.00685	0.00685	0.61268	0.15776	0.58997	0.38928	0.01618	0.42671	0.09167	0.9291	0.9291	turquoise
TREML1	-0.09193	0.08871	0.08342	0.06702	-0.0498	0.10857	-0.03969	0.36331	-0.36331	0.23177	0.24856	0.27803	0.38381	0.51775	0.15749	0.60631	1.04E-06	1.04E-06	blue
RAC2	-0.09104	0.04557	0.07039	0.02555	-0.12912	0.15077	0.14455	-0.06306	0.06306	0.23631	0.55399	0.36028	0.74009	0.09236	0.04902	0.05926	0.41255	0.41255	blue
SRFBP1	-0.06337	-0.08265	-0.00399	0.03327	-0.13013	0.14488	0.03702	0.14266	-0.14266	0.41024	0.28249	0.9587	0.66571	0.08981	0.05867	0.63075	0.06269	0.06269	turquoise
CFL2	-0.09748	-0.08527	-0.09013	0.06876	-0.08233	-0.1037	-0.10317	0.42636	-0.42636	0.20467	0.26749	0.24107	0.37156	0.28441	0.17711	0.17932	6.08E-09	6.08E-09	salmon
TNFAIP3	-0.13474	-0.11368	0.06695	0.10686	-0.18322	0.17805	-0.00968	0.31553	-0.31553	0.07891	0.13874	0.38428	0.16421	0.01646	0.01981	0.90001	2.63E-05	2.63E-05	blue
NRIP3	-0.01504	-0.04778	0.07925	0.16764	-0.17591	0.0919	0.15451	0.1666	-0.1666	0.84516	0.5349	0.30283	0.0284	0.02137	0.23189	0.04362	0.02942	0.02942	black
PLEKHM3	-0.0545	-0.11249	-0.02825	-0.01928	-0.18444	0.12432	0.04212	0.3032	-0.3032	0.47895	0.14296	0.71381	0.80239	0.01574	0.10522	0.58439	5.55E-05	5.55E-05	turquoise
ARMH1	-0.08984	0.01168	-0.06757	-0.13342	0.07791	-0.06434	-0.10296	0.0965	-0.0965	0.2426	0.87948	0.37988	0.08191	0.31114	0.40313	0.18024	0.20926	0.20926	blue
PBK	-0.08082	0.09265	0.05143	-0.07345	-0.03493	0.04019	0.31176	-0.39044	0.39044	0.29335	0.2281	0.50412	0.33972	0.65018	0.60172	3.31E-05	1.30E-07	1.30E-07	pink
ZFHX3	-0.00137	-0.12921	-0.01326	0.06532	-0.18608	0.08338	0.06533	0.28372	-0.28372	0.98578	0.09211	0.86336	0.39602	0.01482	0.27824	0.39589	0.00017	0.00017	turquoise
DACT1	-0.00385	-0.09565	0.07891	0.14341	-0.23023	0.2272	0.09309	0.28183	-0.28183	0.96011	0.21335	0.30492	0.06131	0.00245	0.0028	0.22588	0.00019	0.00019	black
CIZ1	0.10828	-0.04322	-0.08775	-0.03219	-0.09423	-0.05091	0.07595	0.04631	-0.04631	0.15862	0.57455	0.25377	0.67602	0.22023	0.50846	0.32347	0.54752	0.54752	turquoise
ECE1	-0.08405	-0.22958	-0.0197	0.0387	-0.17116	-0.02077	-0.08894	0.09095	-0.09095	0.27442	0.00252	0.79814	0.61533	0.0252	0.78741	0.24733	0.23681	0.23681	turquoise
PGM3	-0.06481	-0.13132	0.04999	0.02099	-0.07892	0.03514	0.0311	0.2794	-0.2794	0.39972	0.08689	0.51612	0.7852	0.30488	0.64818	0.68636	0.00021	0.00021	turquoise
NSMCE1	0.04345	0.15602	-0.05371	-0.05777	-0.0724	0.1215	0.20055	-0.29107	0.29107	0.57258	0.04158	0.48535	0.45292	0.34666	0.11342	0.00854	0.00011	0.00011	grey
EYA2	0.08879	-0.03071	0.05401	0.12297	-0.19526	0.17458	0.17623	-0.11174	0.11174	0.24815	0.69008	0.48291	0.10907	0.01049	0.02239	0.02113	0.14565	0.14565	grey
SLC30A7	-0.08094	-0.13615	0.02537	0.01466	-0.12488	0.12659	0.08771	0.09607	-0.09607	0.29263	0.0758	0.74191	0.8491	0.10365	0.09896	0.25395	0.21132	0.21132	turquoise
SLC36A1	-0.00738	-0.11268	0.02347	0.09264	-0.22055	0.18656	0.12333	0.22291	-0.22291	0.92367	0.14227	0.76056	0.22817	0.00375	0.01456	0.10804	0.00338	0.00338	blue
PRPF19	0.12435	0.00642	-0.06931	-0.08092	-0.07699	-0.07677	0.12514	-0.12917	0.12917	0.10512	0.93355	0.36772	0.29272	0.31687	0.3183	0.10292	0.09223	0.09223	turquoise
FMO5	0.0567	0.05052	0.05985	0.00149	0.05932	0.20893	0.00971	-0.12749	0.12749	0.4614	0.51168	0.4368	0.98453	0.44091	0.0061	0.89975	0.09657	0.09657	grey
MARVELD	0.11116	-0.02729	-0.00594	-0.0358	0.18554	0.08057	0.0712	-0.4345	0.4345	0.14777	0.72307	0.93854	0.64204	0.01511	0.29481	0.35474	2.89E-09	2.89E-09	grey
C2orf76	-0.07838	-0.09557	-0.0502	-0.00161	-0.07929	-0.05459	-0.09753	0.36429	-0.36429	0.30821	0.21373	0.51438	0.98337	0.3026	0.47822	0.20444	9.68E-07	9.68E-07	grey
FOXL1	0.04393	-0.09855	0.03183	-0.03918	-0.02116	0.10337	0.19606	-0.45428	0.45428	0.56831	0.19969	0.67942	0.61087	0.78355	0.17847	0.01017	4.35E-10	4.35E-10	yellow
M1AP	0.01341	-0.0416	0.06879	0.01717	-0.06697	0.12403	0.23364	0.08013	-0.08013	0.86176	0.58904	0.37134	0.82362	0.38412	0.10605	0.0021	0.29747	0.29747	grey
ECM1	-0.03287	0.04834	0.1158	-0.0028	0.02618	0.13181	0.11739	-0.19624	0.19624	0.66952	0.5301	0.13148	0.97105	0.73397	0.0857	0.12624	0.0101	0.0101	grey
TUBA1C	-0.01653	0.00758	0.10556	0.0696	-0.07729	0.25047	0.24007	-0.29729	0.29729	0.83009	0.92166	0.16942	0.36569	0.31498	0.00095	0.00156	7.86E-05	7.86E-05	pink
SCYL2	-0.00187	-0.15693	0.04213	0.07111	-0.02844	0.06302	0.01199	0.13399	-0.13399	0.98063	0.04039	0.58431	0.35538	0.71196	0.41284	0.87629	0.0806	0.0806	turquoise
OARD1	0.09367	0.04134	-0.15571	-0.08294	0.13984	-0.0638	-0.07942	-0.02641	0.02641	0.22299	0.59134	0.04199	0.28083	0.06811	0.40708	0.30179	0.73172	0.73172	grey
CD96	-0.10435	-0.01413	0.07823	0.07421	-0.19493	0.17559	-0.00956	0.3841	-0.3841	0.17438	0.85445	0.30914	0.33475	0.01062	0.02161	0.90121	2.15E-07	2.15E-07	blue
LY86	-0.08125	0.00543	0.03927	0.0195	-0.24805	0.14261	0.00715	0.38667	-0.38667	0.29075	0.94385	0.61009	0.80017	0.00107	0.06279	0.92609	1.75E-07	1.75E-07	blue
CHRNA5	-0.03921	-0.10396	-0.05309	-0.04775	0.07487	-0.093	0.0262	-0.2955	0.2955	0.61063	0.17601	0.49041	0.53514	0.33046	0.22636	0.73376	8.72E-05	8.72E-05	grey
LARP4B	0.08616	-0.0766	0.01848	-0.0541	0.00653	0.03902	0.07702	-0.17972	0.17972	0.2625	0.31938	0.8104	0.48219	0.93248	0.6124	0.31671	0.01867	0.01867	turquoise
SPNS1	0.04891	0.00963	0.03028	-0.00545	0.01513	0.06418	0.15164	-0.10978	0.10978	0.52522	0.90056	0.69424	0.94355	0.84431	0.40434	0.04772	0.15291	0.15291	green
BPGM	-0.06689	-0.22767	-0.06241	0.0165	-0.06651	-0.09029	-0.0135	0.11192	-0.11192	0.3847	0.00275	0.41744	0.83035	0.38739	0.24025	0.86085	0.14502	0.14502	turquoise
GPR89B	0.13654	0.04945	-0.03014	-0.08705	0.15581	-0.01807	0.09588	-0.33556	0.33556	0.07495	0.5207	0.69557	0.2576	0.04186	0.81451	0.21222	7.23E-06	7.23E-06	grey
NRN1	-0.067	-0.00713	-0.02627	0.0545	0.06509	0.01516	-0.13911	0.39523	-0.39523	0.38395	0.92625	0.73303	0.47893	0.39764	0.844	0.06958	8.81E-08	8.81E-08	grey
TSSK6	0.08077	0.0462	-0.19472	-0.07575	0.28405	-0.16668	0.04259	-0.2167	0.2167	0.29363	0.54851	0.01071	0.32479	0.00017	0.02934	0.58021	0.00441	0.00441	green
SLC14A1	-0.03999	-0.21635	-0.01568	0.07588	0.0694	0.0151	0.00105	0.026	-0.026	0.60351	0.00448	0.83872	0.32396	0.36707	0.84459	0.98913	0.73574	0.73574	grey
RXRA	-0.07291	-0.17085	-0.22104	-0.03044	-0.01415	-0.20899	-0.01072	0.29783	-0.29783	0.34326	0.02547	0.00367	0.69269	0.85429	0.00608	0.8893	7.61E-05	7.61E-05	brown
AKR1C1	0.17512	-0.11164	0.00339	0.05925	-0.00685	0.0694	-0.01299	0.01158	-0.01158	0.02196	0.14601	0.96492	0.44145	0.92911	0.3671	0.86612	0.88048	0.88048	grey
MMP11	-0.06322	-0.04908	0.02375	0.03247	-0.08655	0.15311	0.09334	-0.12322	0.12322	0.41135	0.52379	0.7578	0.6733	0.26033	0.04558	0.22463	0.10836	0.10836	black
LSM14A	-0.02273	-0.16674	-0.07	0.0102	-0.11524	0.02764	-0.02999	0.13064	-0.13064	0.76788	0.02928	0.36291	0.89466	0.13339	0.71969	0.69695	0.08855	0.08855	turquoise
SHC4	-0.01195	-0.10049	-0.14598	-0.08529	-0.11168	-0.13612	-0.07444	0.31295	-0.31295	0.87674	0.19095	0.05675	0.26736	0.14587	0.07586	0.33325	3.08E-05	3.08E-05	grey
NFASC	-0.00171	-0.11586	-0.18501	-0.08406	0.09217	-0.2608	-0.27569	0.52594	-0.52594	0.98231	0.13128	0.01542	0.27433	0.23053	0.00057	0.00026	1.50E-13	1.50E-13	brown
APEX2	-0.02774	-0.04539	0.02135	0.05301	-0.09163	0.0925	0.19314	-0.15397	0.15397	0.71869	0.55551	0.78164	0.49105	0.23328	0.22888	0.01138	0.04437	0.04437	turquoise
TMEM214	0.05768	-0.01944	0.01489	0.03549	-0.09176	0.08219	0.1834	-0.12743	0.12743	0.45368	0.8008	0.84669	0.64496	0.2326	0.2852	0.01634	0.09672	0.09672	turquoise
HGH1	0.04602	0.11394	-0.05781	-0.02864	-0.04647	-0.02762	0.18248	-0.2486	0.2486	0.55008	0.13786	0.45263	0.71005	0.54618	0.71986	0.0169	0.00104	0.00104	green
POMT1	-0.04939	-0.19828	-0.17607	-0.00083	0.1115	-0.23808	-0.16016	0.23257	-0.23257	0.52121	0.00933	0.02125	0.9914	0.14654	0.00171	0.03639	0.00221	0.00221	brown
NPNT	-0.02427	-0.18983	-0.11268	0.01835	0.12154	0.07177	-0.08182	0.04758	-0.04758	0.75273	0.01289	0.14227	0.81169	0.11328	0.35087	0.2874	0.53663	0.53663	grey
TMEM164	-0.0223	-0.16021	-0.09702	-0.03838	0.0769	-0.10426	-0.01672	-0.04916	0.04916	0.77221	0.03633	0.20681	0.61825	0.31748	0.17475	0.8282	0.52317	0.52317	turquoise
TP53INP1	-0.11085	-0.14998	-0.12802	0.01443	-0.11288	-0.09338	-0.17377	0.42977	-0.42977	0.1489	0.05024	0.09519	0.85144	0.14157	0.22443	0.02302	4.46E-09	4.46E-09	blue
CREB3L3	0.02881	0.00271	0.09814	0.0881	0.10348	0.10917	0.10123	-0.31679	0.31679	0.70835	0.9719	0.2016	0.25184	0.17803	0.15522	0.1877	2.43E-05	2.43E-05	grey
BMP7	0.0151	0.03365	0.0177	0.00273	0.05805	0.11319	0.0799	0.04601	-0.04601	0.84457	0.66215	0.81831	0.97173	0.45077	0.14048	0.29886	0.55016	0.55016	grey
ACAP1	-0.08852	-0.05996	-0.02093	-0.02888	-0.05568	-0.02127	-0.07014	0.37006	-0.37006	0.24962	0.43598	0.7858	0.7077	0.46945	0.78246	0.36199	6.30E-07	6.30E-07	blue
SRXN1	-0.03628	-0.041	-0.09221	0.01271	0.01715	-0.08358	0.04989	0.11511	-0.11511	0.6376	0.59439	0.23033	0.86897	0.8238	0.27711	0.51696	0.13383	0.13383	turquoise
ATP1B3	0.00311	-0.00843	0.11728	0.09117	-0.21765	0.22364	0.27328	-0.09896	0.09896	0.96779	0.91284	0.12661	0.23565	0.00424	0.00328	0.0003	0.19785	0.19785	turquoise
RBM4	-0.0368	-0.1512	-0.09988	-0.0151	-0.06006	0.00917	0.03338	0.07859	-0.07859	0.63273	0.04838	0.19366	0.84454	0.43516	0.90529	0.66471	0.30693	0.30693	turquoise
ZC3H4	0.02892	-0.05884	-0.07037	-0.02652	-0.02316	-0.04109	-0.05146	0.07298	-0.07298	0.70727	0.44462	0.36041	0.73061	0.76369	0.59365	0.50384	0.34284	0.34284	turquoise
DENND2C	-0.02794	-0.00549	-0.09539	0.09025	-0.16781	-0.02337	0.10035	0.18369	-0.18369	0.71678	0.94318	0.21459	0.24044	0.02825	0.76153	0.19156	0.01617	0.01617	grey
MOB1A	-0.06496	-0.06876	0.08218	0.07165	-0.20281	0.22556	0.1477	-0.01698	0.01698	0.39859	0.37149	0.28526	0.35172	0.00781	0.00301	0.05387	0.82553	0.82553	turquoise
ABCC8	0.00579	-0.0675	-0.26838	-0.14162	0.09032	-0.245	-0.17173	0.38656	-0.38656	0.94004	0.38041	0.00039	0.06466	0.24005	0.00124	0.02471	1.77E-07	1.77E-07	brown
P3H3	0.0155	-0.14037	-0.08981	-0.06535	0.04982	-0.16167	-0.12439	0.33825	-0.33825	0.84055	0.06708	0.24277	0.39577	0.51757	0.03464	0.10501	6.04E-06	6.04E-06	brown
ZFP28	-0.11604	-0.14964	-0.11073	0.02809	-0.04446	-0.00918	-0.15061	0.42738	-0.42738	0.1307	0.05077	0.14937	0.71533	0.56366	0.9051	0.04926	5.55E-09	5.55E-09	turquoise
ANKRD61	0.02066	-0.17963	0.01747	0.04841	-0.01461	0.03001	0.14912	-0.08661	0.08661	0.78851	0.01873	0.82061	0.52953	0.84957	0.69685	0.05158	0.26002	0.26002	red
RPS4X	-0.4611	0.06363	0.013	-0.00033	-0.07004	0.08836	0.00292	-0.06126	0.06126	2.20E-10	0.40832	0.86597	0.99658	0.36268	0.25048	0.96972	0.42608	0.42608	purple
TRAPPC1	-0.04769	-0.0451	-0.10311	-0.12646	-0.00591	-0.17729	-0.00854	-0.04625	0.04625	0.53563	0.55801	0.17959	0.09931	0.93886	0.02036	0.91171	0.54805	0.54805	grey
FBLN7	-0.0693	-0.163	-0.05087	-0.00532	-0.03549	-0.22703	-0.14339	0.41176	-0.41176	0.36776	0.03316	0.50877	0.94498	0.64489	0.00283	0.06135	2.20E-08	2.20E-08	brown
EPB41L4B	0.01812	-0.02619	-0.02704	-0.05619	0.09341	-0.05752	-0.20217	0.06996	-0.06996	0.81408	0.73381	0.72554	0.46545	0.2243	0.4549	0.00801	0.36323	0.36323	grey
TNRC6B	-0.04071	-0.14826	-0.07126	0.00913	-0.06478	-0.04539	-0.10456	0.26837	-0.26837	0.59703	0.05296	0.35436	0.90571	0.39994	0.5555	0.17351	0.00039	0.00039	turquoise
SP6	-0.09064	-0.04147	0.01726	0.10279	-0.0264	0.05853	0.16586	-0.14759	0.14759	0.23839	0.59016	0.82274	0.18094	0.73174	0.44701	0.03016	0.05405	0.05405	grey
COPG2	0.04722	0.04302	-0.03741	-0.04175	0.06163	-0.04363	-0.01349	0.07171	-0.07171	0.53964	0.57641	0.62714	0.5877	0.42329	0.57094	0.86097	0.35129	0.35129	turquoise
VIPR1	0.13523	-0.03876	-0.16765	-0.15138	0.25609	-0.09745	-0.12555	-0.01656	0.01656	0.07782	0.61473	0.0284	0.04811	0.00072	0.20479	0.1018	0.82977	0.82977	grey
BRCA2	-0.05032	0.11542	0.00174	-0.00838	-0.18844	0.10968	0.27674	-0.0736	0.0736	0.5134	0.13276	0.98194	0.91334	0.01358	0.15326	0.00025	0.33875	0.33875	pink
P2RY12	-0.17011	0.01071	-0.04708	0.02753	-0.12631	0.07715	-0.05783	0.50513	-0.50513	0.02612	0.88947	0.54085	0.72077	0.09971	0.31585	0.45246	1.85E-12	1.85E-12	blue
SPTSSA	0.04246	-0.0643	-0.08442	-0.00729	-0.00295	-0.07361	0.02075	-0.00092	0.00092	0.58138	0.40345	0.27229	0.92459	0.96941	0.33864	0.78761	0.99045	0.99045	turquoise
AGO4	-0.08196	-0.21503	0.06771	0.05995	-0.06739	0.11889	-0.01464	0.09781	-0.09781	0.28655	0.00474	0.37892	0.43605	0.38118	0.12144	0.84923	0.20312	0.20312	turquoise
SAAL1	0.02811	0.0858	-0.0416	-0.1387	-0.04879	-0.00414	0.1415	-0.20937	0.20937	0.71514	0.26448	0.58907	0.07041	0.52624	0.95715	0.06487	0.00599	0.00599	turquoise
SETD7	-0.08791	-0.10581	-0.0276	0.01192	-0.14842	0.11829	-0.01599	0.34933	-0.34933	0.25291	0.16841	0.72004	0.87698	0.0527	0.12335	0.83555	2.83E-06	2.83E-06	turquoise
MEF2B	-0.02009	0.0571	0.04168	-0.04964	0.06533	0.06473	-0.04856	0.00639	-0.00639	0.7942	0.45819	0.58831	0.51906	0.3959	0.40031	0.52819	0.93384	0.93384	blue
FPR3	-0.15313	-0.00055	0.0833	0.0491	-0.25105	0.17722	0.06936	0.42305	-0.42305	0.04554	0.99426	0.27871	0.52363	0.00093	0.0204	0.36735	8.19E-09	8.19E-09	blue
BCS1L	0.07315	0.10599	-0.09715	-0.01694	0.16515	-0.07668	0.08107	-0.22746	0.22746	0.34167	0.16767	0.20619	0.82589	0.03088	0.31887	0.29183	0.00277	0.00277	red
SLBP	-0.03701	-0.04859	-0.0553	-0.04635	-0.01593	0.01913	0.03878	-0.1255	0.1255	0.63079	0.52794	0.4725	0.54715	0.83622	0.80382	0.61458	0.10194	0.10194	turquoise
WDR77	-0.05744	-0.01677	0.00821	-0.05445	0.0166	0.05172	0.10063	-0.13405	0.13405	0.45554	0.82765	0.91513	0.47934	0.8294	0.50168	0.19036	0.08046	0.08046	turquoise
LRRC75A	0.00167	-0.1599	-0.09479	-0.13735	0.21965	-0.1215	-0.12586	0.14584	-0.14584	0.9827	0.0367	0.21748	0.07323	0.00389	0.1134	0.10096	0.057	0.057	grey
RPA3	0.02632	-0.05887	-0.04458	-0.07888	-0.06754	-0.04879	0.17533	-0.34349	0.34349	0.73259	0.44433	0.56259	0.30509	0.38007	0.52628	0.02181	4.23E-06	4.23E-06	pink
FKTN	-0.02821	-0.12745	-0.04512	-0.03107	-0.11455	0.04472	0.03146	0.1394	-0.1394	0.71413	0.09668	0.55792	0.68662	0.13573	0.56136	0.68294	0.069	0.069	turquoise
KCTD18	-0.10336	-0.10286	-0.04571	0.06704	0.0385	-0.01259	-0.06575	0.19533	-0.19533	0.17854	0.18067	0.55272	0.38364	0.61715	0.87017	0.39285	0.01046	0.01046	turquoise
CARHSP1	-0.01512	-0.14795	-0.03904	0.03066	0.02379	-0.03444	0.01457	-0.24015	0.24015	0.8444	0.05346	0.61222	0.69057	0.75745	0.65472	0.84996	0.00156	0.00156	grey
SALL1	0.02585	-0.06298	0.03152	0.19017	-0.00886	-0.14115	-0.02565	0.15385	-0.15385	0.73719	0.41313	0.68235	0.01273	0.90845	0.06554	0.73912	0.04453	0.04453	grey
CA4	-0.05655	-0.01754	-0.07314	0.08642	0.03804	-0.06244	-0.01505	0.14626	-0.14626	0.46255	0.81986	0.34174	0.26103	0.62135	0.4172	0.84512	0.05629	0.05629	magenta
MEIS3	-0.02525	-0.16216	-0.08241	0.02914	0.00191	-0.18916	-0.10973	0.36736	-0.36736	0.74305	0.03408	0.28391	0.70517	0.98019	0.01322	0.15308	7.71E-07	7.71E-07	brown
GTPBP2	0.01978	-0.08389	-0.00518	0.00988	-0.01103	0.03106	0.07298	-0.1845	0.1845	0.79738	0.27531	0.94643	0.89795	0.88618	0.68671	0.34285	0.0157	0.0157	turquoise
XXYLT1	-0.06751	-0.04193	-0.04115	0.02909	-0.1431	0.10983	0.16289	0.06091	-0.06091	0.38031	0.58606	0.59309	0.70567	0.06187	0.1527	0.03328	0.42872	0.42872	black
FREM2	0.08327	-0.01255	-0.11	-0.01072	-0.02138	0.04415	0.10532	0.08785	-0.08785	0.27891	0.87063	0.15208	0.88928	0.78133	0.56636	0.17041	0.25319	0.25319	grey
STRN4	0.05411	-0.02536	-0.02838	0.0175	0.02407	0.02472	0.07322	-0.22458	0.22458	0.48209	0.74194	0.71253	0.82027	0.75462	0.7483	0.34126	0.00315	0.00315	grey
TMPRSS3	0.04481	0.11745	0.01765	0.02948	0.02966	0.21824	0.10493	-0.35153	0.35153	0.56055	0.12603	0.81874	0.70188	0.70018	0.00414	0.17197	2.42E-06	2.42E-06	yellow
KLF16	0.10394	-0.02181	-0.05837	-0.10628	0.08573	-0.01935	0.06267	-0.28577	0.28577	0.1761	0.77706	0.44823	0.16652	0.2649	0.80162	0.41546	0.00015	0.00015	green
ARSE	-0.18152	0.10337	-0.12575	0.02326	0.12817	0.07793	0.00864	-0.02919	0.02919	0.0175	0.17848	0.10123	0.76267	0.09479	0.31098	0.91074	0.70469	0.70469	grey
RNASEH2I	0.03635	0.03743	-0.05564	-0.04798	-0.07218	-0.07132	-0.02188	0.22319	-0.22319	0.63689	0.6269	0.46976	0.53315	0.34816	0.35393	0.77637	0.00334	0.00334	grey
TSPAN3	0.04038	-0.09739	-0.00342	-0.0231	0.12356	0.07154	-0.06646	-0.23363	0.23363	0.60005	0.20509	0.96458	0.76424	0.10739	0.35247	0.38775	0.0021	0.0021	grey
ADNP2	-0.06936	-0.13797	-0.03039	0.02776	-0.03231	0.00612	-0.05022	0.19378	-0.19378	0.36738	0.07193	0.69318	0.71856	0.67483	0.93666	0.51421	0.0111	0.0111	turquoise
NLRP1	-0.05701	-0.12236	-0.16549	-0.13694	0.0752	-0.29665	-0.26447	0.40592	-0.40592	0.45893	0.11086	0.03053	0.0741	0.32829	8.16E-05	0.00047	3.63E-08	3.63E-08	brown
SLC9A7	-0.04547	-0.28334	-0.11618	-0.01011	-0.08145	-0.15911	-0.00143	-0.01695	0.01695	0.55485	0.00017	0.13023	0.89562	0.28961	0.03765	0.98522	0.82584	0.82584	turquoise
CEP41	-0.04792	-0.14009	-0.17241	-0.09997	-0.01247	-0.29203	-0.07322	0.31674	-0.31674	0.5337	0.06763	0.02414	0.19327	0.87142	0.00011	0.34121	2.43E-05	2.43E-05	turquoise
PARP16	-0.05581	-0.14667	-0.0985	-0.02071	-0.0618	0.01918	0.06797	0.03793	-0.03793	0.46843	0.05558	0.19994	0.78803	0.42202	0.80332	0.37703	0.62237	0.62237	turquoise
ZNF662	-0.03495	-0.13791	-0.04849	-0.06803	-0.03122	0.05907	-0.04791	-0.01394	0.01394	0.64999	0.07205	0.52881	0.37662	0.68517	0.44283	0.5338	0.85643	0.85643	grey
ZCCHC8	-0.00726	-0.19373	-0.02781	0.00222	0.08056	0.01468	-0.09743	0.07916	-0.07916	0.92495	0.01112	0.71809	0.97701	0.29489	0.84885	0.2049	0.30338	0.30338	turquoise

PAWR	0.07813	-0.06546	0.07293	0.08296	0.00669	0.13093	0.01889	-0.13116	0.13116	0.30974	0.395	0.34312	0.28068	0.93085	0.08784	0.80627	0.08728	0.08728	turquoise
SLC37A3	-0.0302	-0.14854	0.03792	0.05436	0.00352	0.05344	0.03679	0.01777	-0.01777	0.695	0.05251	0.62242	0.48008	0.96351	0.48753	0.63283	0.81755	0.81755	turquoise
CRTAP	-0.03923	-0.04486	0.03218	0.06599	-0.23652	0.16447	0.16832	0.29052	-0.29052	0.61045	0.56017	0.67609	0.39114	0.00184	0.03159	0.02776	0.00012	0.00012	blue
SIRT4	0.06212	-0.2129	-0.16598	0.01237	0.10275	-0.15383	-0.06674	0.22523	-0.22523	0.4196	0.00518	0.03004	0.87238	0.18113	0.04455	0.38577	0.00306	0.00306	grey
LRP1	-0.02589	-0.08797	0.09668	0.09819	-0.19203	0.21965	0.11719	0.30732	-0.30732	0.73673	0.25259	0.20843	0.20135	0.01187	0.00389	0.1269	4.34E-05	4.34E-05	black
THUMPD1	-0.0686	-0.08761	-0.03097	0.05413	-0.07279	0.08946	-0.01299	0.16423	-0.16423	0.37264	0.25452	0.68756	0.48199	0.34411	0.24457	0.86604	0.03183	0.03183	turquoise
DAPL1	0.05495	0.02908	-0.15617	0.03213	-0.02021	-0.07261	0.01736	0.18901	-0.18901	0.47531	0.70577	0.04137	0.67658	0.793	0.34526	0.82174	0.01329	0.01329	grey
SUSD1	-0.05102	-0.18158	0.02538	0.04394	-0.17785	0.08796	0.01563	0.16317	-0.16317	0.50749	0.01746	0.74174	0.56824	0.01995	0.2526	0.8392	0.03298	0.03298	turquoise
DDR2	-0.08791	-0.07701	0.04086	0.08057	-0.22834	0.13974	0.04289	0.4445	-0.4445	0.25291	0.31673	0.59568	0.29487	0.00267	0.06831	0.57752	1.13E-09	1.13E-09	blue
CFAP73	0.02496	-0.0185	0.09571	-0.01017	0.15618	0.07042	0.02405	-0.36496	0.36496	0.74588	0.81021	0.21305	0.89492	0.04137	0.36004	0.75485	9.21E-07	9.21E-07	grey
FUT8	-0.07054	0.01804	0.14827	0.00842	-0.07501	0.18976	0.13104	-0.05205	0.05205	0.35924	0.81481	0.05295	0.91299	0.32952	0.01292	0.08757	0.49894	0.49894	turquoise
SLC25A5	0.13878	0.06736	0.09014	-0.00401	-0.03017	0.06488	0.08182	-0.19506	0.19506	0.07026	0.38138	0.24099	0.95846	0.69531	0.39922	0.28738	0.01057	0.01057	grey
KLK7	-0.03619	0.06236	0.08873	0.02713	-0.01834	0.11639	0.10469	-0.19793	0.19793	0.63839	0.41778	0.24849	0.72465	0.81177	0.12954	0.17297	0.00946	0.00946	yellow
PHF3	-0.02757	-0.15063	-0.01363	0.03898	-0.08897	0.1297	-0.02002	0.22237	-0.22237	0.72041	0.04924	0.85953	0.6127	0.24718	0.09087	0.79489	0.00346	0.00346	turquoise
ADAM8	-0.00447	-0.03362	0.10882	0.00619	-0.02549	0.15716	0.21132	-0.35575	0.35575	0.95373	0.66243	0.15655	0.93591	0.74068	0.04009	0.00553	1.80E-06	1.80E-06	yellow
CYS1	-0.02946	-0.02302	-0.03311	0.16582	-0.13225	0.10688	-0.01291	0.25571	-0.25571	0.70206	0.76506	0.66726	0.0302	0.08466	0.16409	0.86689	0.00074	0.00074	magenta
OSGEP	-0.00373	-0.0572	-0.03997	-0.02548	0.16627	-0.05652	-0.05544	-0.11729	0.11729	0.96137	0.45742	0.60376	0.74076	0.02974	0.4628	0.47138	0.12657	0.12657	red
KLHL9	-0.01533	0.00532	-0.13552	-0.06618	-0.12192	-0.0509	-0.02145	0.38778	-0.38778	0.84226	0.94491	0.07718	0.38976	0.11215	0.50854	0.7807	1.60E-07	1.60E-07	turquoise
ENO1	-0.02965	-0.02636	0.05384	0.06439	-0.12922	0.14988	0.19451	-0.32141	0.32141	0.70024	0.73221	0.4843	0.40278	0.09209	0.05039	0.01079	1.82E-05	1.82E-05	turquoise
CCDC18	-0.13212	-0.05439	0.05688	0.00916	-0.08845	0.05903	0.14151	-0.01525	0.01525	0.08496	0.47983	0.45991	0.90535	0.24996	0.44312	0.06487	0.84304	0.84304	turquoise
DGKQ	0.00173	0.11164	-0.07109	-0.04345	0.20471	0.01282	0.01532	-0.28835	0.28835	0.98211	0.14603	0.35547	0.57254	0.00724	0.86787	0.84235	0.00013	0.00013	grey
LNPK	-0.03032	-0.07666	-0.03245	0.08843	-0.02402	0.00025	0.04768	-0.0009	0.0009	0.69387	0.31898	0.67354	0.2501	0.75516	0.99739	0.53575	0.99067	0.99067	turquoise
HMGCS2	0.07431	-0.04204	0.04141	0.01815	0.13362	0.12213	-0.05379	-0.25176	0.25176	0.3341	0.58509	0.59071	0.8137	0.08146	0.11153	0.48468	0.00089	0.00089	grey
KMT2E	-0.04216	-0.18964	0.03436	0.05496	-0.05642	0.15646	-0.04341	0.1374	-0.1374	0.58404	0.01298	0.65551	0.47525	0.46357	0.041	0.57289	0.07312	0.07312	turquoise
ASAP3	0.0295	-0.04805	0.10212	0.19437	-0.17137	0.22649	0.12732	0.28744	-0.28744	0.70169	0.53258	0.18384	0.01085	0.02502	0.00289	0.09702	0.00014	0.00014	blue
C6orf141	0.0692	0.00362	-0.00421	0.0431	0.07575	0.13962	0.115	-0.26722	0.26722	0.36846	0.96256	0.95644	0.57564	0.32479	0.06857	0.13418	0.00041	0.00041	grey
PDK1	-0.13123	-0.03341	0.04964	0.07952	-0.03742	0.05479	0.10069	-0.19193	0.19193	0.08711	0.66446	0.5191	0.3012	0.62706	0.47665	0.19009	0.01191	0.01191	turquoise
ATP8B2	-0.09747	-0.15175	-0.08718	0.0223	-0.07237	-0.07562	-0.18151	0.61371	-0.61371	0.20471	0.04755	0.25684	0.77219	0.34686	0.32563	0.01751	4.47E-19	4.47E-19	blue
FBXL22	-0.00016	-0.00134	0.0156	-0.0559	-0.06027	0.08316	0.00123	0.3236	-0.3236	0.99829	0.98615	0.83951	0.46775	0.43361	0.27954	0.98723	1.58E-05	1.58E-05	salmon
KLF4	0.00119	-0.07208	0.19751	0.08142	0.05621	0.34123	0.09265	-0.23736	0.23736	0.98764	0.3488	0.00961	0.28978	0.46526	4.94E-06	0.2281	0.00177	0.00177	grey
KIAA1614	-0.11318	-0.04035	-0.189	-0.01559	-0.02876	-0.0641	0.02991	0.41545	-0.41545	0.14051	0.60032	0.0133	0.83964	0.70885	0.40491	0.69777	1.60E-08	1.60E-08	blue
KCNA5	-0.08084	-0.11564	-0.23804	-0.08687	0.07529	-0.31151	-0.23077	0.48113	-0.48113	0.29322	0.13203	0.00172	0.25857	0.32774	3.36E-05	0.00239	2.72E-11	2.72E-11	brown
MOCOS	-0.01626	0.00397	0.06948	0.09636	-0.07747	0.22678	0.27027	-0.36073	0.36073	0.83286	0.95894	0.36653	0.20994	0.3139	0.00286	0.00035	1.26E-06	1.26E-06	yellow
CHMP1A	0.06951	-0.02515	0.00252	-0.01994	-0.01284	0.04256	0.16151	-0.41752	0.41752	0.36631	0.74404	0.97395	0.79576	0.86765	0.58048	0.03483	1.34E-08	1.34E-08	green
HTR1D	0.01656	-0.03813	0.12867	0.05667	0.06189	0.21343	0.17733	-0.22569	0.22569	0.82982	0.62049	0.09351	0.46158	0.42132	0.00507	0.02033	0.003	0.003	grey
LTK	-0.02624	-0.08816	0.04409	0.01809	0.15178	0.1372	-0.0171	-0.32828	0.32828	0.73337	0.25154	0.56689	0.81435	0.04751	0.07355	0.82429	1.17E-05	1.17E-05	grey
GANAB	-0.0203	-0.16488	0.0052	0.01838	-0.14817	0.00749	0.0637	0.0925	-0.0925	0.79212	0.03116	0.94617	0.81137	0.05311	0.92251	0.40786	0.22886	0.22886	turquoise
PPP1R12A	-0.02666	-0.12826	0.03693	0.05752	-0.08417	0.06062	-0.02206	0.29067	-0.29067	0.72923	0.09456	0.63153	0.45486	0.27371	0.4309	0.77455	0.00011	0.00011	turquoise
MORC2	0.05681	-0.00468	-0.03883	0.0552	-0.09554	0.01825	0.22725	-0.16891	0.16891	0.46052	0.95159	0.61409	0.47332	0.21386	0.81269	0.0028	0.02722	0.02722	turquoise
CLMP	-0.11967	-0.01428	0.04949	0.08172	-0.18497	0.11869	0.0795	0.35867	-0.35867	0.11899	0.85293	0.52035	0.28797	0.01544	0.12207	0.30133	1.46E-06	1.46E-06	blue
TREM1	-0.14606	-0.03422	0.14353	0.11553	-0.16382	0.19491	0.13018	0.025	-0.025	0.05662	0.65683	0.06109	0.1324	0.03228	0.01063	0.08968	0.74549	0.74549	black
ARNTL	0.07182	-0.12178	-0.06338	-0.03788	0.03217	0.00808	-0.08855	0.22348	-0.22348	0.35056	0.11257	0.4102	0.6228	0.67621	0.9165	0.24946	0.0033	0.0033	turquoise
DGKE	0.12739	-0.20653	-0.21598	-0.11971	0.14573	-0.25095	-0.19958	0.21039	-0.21039	0.09684	0.00672	0.00455	0.11887	0.05718	0.00093	0.00887	0.00574	0.00574	turquoise
SRRM2	0.02757	-0.06608	0.02611	0.00106	-0.10077	0.13355	0.05937	-0.03098	0.03098	0.72037	0.39053	0.73457	0.98905	0.18881	0.08162	0.44054	0.68754	0.68754	red
SENP1	0.02771	-0.18651	-0.03849	0.01209	-0.09488	-0.00926	-0.0539	0.21819	-0.21819	0.71901	0.01458	0.61718	0.87529	0.21707	0.90434	0.4838	0.00415	0.00415	turquoise
RAD18	-0.00024	-0.01908	-0.00163	-0.08248	-0.17585	0.10003	0.18528	-0.17832	0.17832	0.99747	0.80438	0.98309	0.28348	0.02141	0.19301	0.01526	0.01962	0.01962	turquoise
LGALS8	-0.00888	-0.15741	-0.02959	0.00359	-0.01166	0.02681	0.05937	-0.03986	0.03986	0.90822	0.03976	0.70082	0.96286	0.87964	0.72782	0.44052	0.60473	0.60473	turquoise
IFITM1	-0.0135	0.02668	0.09376	0.12896	-0.18831	0.16324	0.18754	-0.09613	0.09613	0.86086	0.72906	0.22253	0.09277	0.01364	0.0329	0.01404	0.21102	0.21102	tan
GLT8D2	-0.09448	-0.04968	0.00432	0.04397	-0.15639	0.07268	0.03068	0.45577	-0.45577	0.219	0.51871	0.95523	0.56795	0.04109	0.34482	0.69037	3.75E-10	3.75E-10	blue
IRF2BPL	-0.01424	-0.18581	-0.1989	0.04555	0.0012	-0.23027	-0.09044	0.30267	-0.30267	0.85339	0.01497	0.00911	0.55411	0.98756	0.00245	0.23942	5.73E-05	5.73E-05	turquoise
TMEM182	0.14165	-0.06403	0.08023	0.04628	-0.08582	0.09647	0.10318	-0.13424	0.13424	0.0646	0.40538	0.29688	0.54778	0.26442	0.2094	0.17928	0.08004	0.08004	turquoise
PC	-0.05438	-0.06517	-0.0651	-0.03759	-0.11611	0.01333	0.05373	0.02358	-0.02358	0.47993	0.39709	0.39757	0.62548	0.13045	0.86262	0.48521	0.75955	0.75955	grey
MRFAP1L1	-0.07102	-0.12589	-0.14212	-0.04149	0.02406	-0.1089	-0.07422	0.09098	-0.09098	0.35597	0.10086	0.0637	0.59007	0.75478	0.15626	0.33468	0.23663	0.23663	turquoise
DCAF13	-0.01425	-0.02305	0.03419	0.03114	-0.08643	-0.04807	0.09384	-0.11035	0.11035	0.85319	0.76474	0.65704	0.68599	0.26097	0.53243	0.22215	0.15077	0.15077	turquoise
FKBP10	-0.03775	-0.06478	-0.00616	0.06196	-0.12628	0.08275	0.1684	0.02171	-0.02171	0.62403	0.39993	0.93623	0.42076	0.0998	0.28191	0.02769	0.77803	0.77803	black
NEMP1	0.00328	-0.18002	-0.07558	0.0494	-0.1281	-0.02697	0.08167	0.07379	-0.07379	0.96602	0.01847	0.32585	0.52113	0.09498	0.72618	0.28829	0.33748	0.33748	turquoise
CAMKK1	-0.03439	-0.10481	-0.17529	-0.08557	0.06329	-0.21591	-0.15064	0.46054	-0.46054	0.65523	0.1725	0.02184	0.2658	0.41087	0.00456	0.04922	2.33E-10	2.33E-10	brown
BTF3L4	-0.08961	-0.16065	-0.02157	0.0148	-0.13677	0.01876	-0.02519	0.23046	-0.23046	0.24378	0.03582	0.77946	0.84763	0.07446	0.8076	0.74367	0.00243	0.00243	turquoise
C1QTNF4	0.1146	-0.10965	-0.18811	0.01605	0.09309	-0.24689	-0.0875	0.26542	-0.26542	0.13557	0.15338	0.01374	0.83491	0.22589	0.00113	0.25511	0.00045	0.00045	grey
NAALADL1	0.03352	-0.10792	0.01547	0.0907	-0.14028	0.20548	0.10323	-0.0592	0.0592	0.66339	0.16001	0.84087	0.23806	0.06725	0.00701	0.17906	0.44181	0.44181	turquoise
SMAP1	-0.02344	-0.08206	0.05333	0.08445	-0.09563	0.09782	0.06769	0.1471	-0.1471	0.76084	0.28598	0.48849	0.27211	0.21343	0.20309	0.37901	0.05487	0.05487	turquoise
SLC22A15	0.02575	-0.18959	-0.06062	-0.02515	-0.00752	-0.10575	-0.10797	0.0889	-0.0889	0.73814	0.01301	0.43093	0.744	0.92224	0.16863	0.15984	0.24758	0.24758	grey
PROC	0.12243	-0.06783	-0.0033	0.06384	0.16415	-0.07228	-0.0849	-0.28131	0.28131	0.11066	0.37806	0.96583	0.40679	0.03192	0.34748	0.26955	0.00019	0.00019	grey
RRP9	0.12898	0.12307	0.02649	-0.10662	-0.06001	0.09994	0.1811	-0.38498	0.38498	0.09271	0.10878	0.73093	0.16516	0.43557	0.19343	0.01777	2.00E-07	2.00E-07	grey
CBX1	-0.08692	-0.23701	-0.14607	-0.03242	-0.06606	-0.15638	-0.06064	0.22732	-0.22732	0.25827	0.0018	0.0566	0.67377	0.39068	0.04111	0.43077	0.00279	0.00279	turquoise
PLOD3	0.11728	-0.04634	0.0484	0.05178	0.07142	-0.05865	0.10331	-0.25785	0.25785	0.12661	0.54724	0.5296	0.50121	0.35328	0.44607	0.17874	0.00066	0.00066	grey
GALM	0.03571	0.09188	0.12728	0.10892	-0.06337	0.37297	0.26241	-0.21578	0.21578	0.64288	0.23199	0.09714	0.15615	0.41027	5.06E-07	0.00053	0.00459	0.00459	yellow
ILF3	-0.00376	-0.06248	-0.12879	-0.10236	0.04058	-0.05649	0.0143	-0.03443	0.03443	0.96109	0.41688	0.0932	0.18277	0.59822	0.46299	0.85269	0.65487	0.65487	red
PLAGL2	-0.03707	-0.24782	-0.01215	0.01583	0.03406	-0.02835	-0.08329	0.10628	-0.10628	0.63025	0.00108	0.87464	0.83721	0.65832	0.71283	0.2788	0.16649	0.16649	turquoise
CAMKK2	-0.02423	-0.00975	0.09439	0.10325	-0.11175	0.2182	0.16217	0.03325	-0.03325	0.75312	0.89932	0.21944	0.179	0.14563	0.00414	0.03408	0.66595	0.66595	turquoise
INPP4A	-0.08501	-0.1262	-0.00046	0.09204	-0.13436	-0.02238	0.00269	0.42685	-0.42685	0.26894	0.10002	0.99525	0.23119	0.07976	0.77145	0.97214	5.82E-09	5.82E-09	blue
DNAJC28	-0.11322	-0.15394	-0.20931	-0.07815	0.16852	-0.26923	-0.16539	0.3609	-0.3609	0.14038	0.0444	0.006	0.30963	0.02757	0.00037	0.03064	1.24E-06	1.24E-06	brown
SMAD6	0.00171	0.05012	0.00515	0.04223	0.01818	0.10761	0.20585	-0.28445	0.28445	0.98227	0.51504	0.94671	0.58344	0.81346	0.16124	0.00691	0.00016	0.00016	yellow
ASIC1	0.03776	-0.03038	-0.08497	0.06246	0.07997	-0.23963	-0.03469	0.17423	-0.17423	0.62389	0.69321	0.26918	0.41703	0.29845	0.0016	0.65237	0.02266	0.02266	grey
CCDC51	0.00983	0.09908	-0.0017	-0.05549	-0.04295	0.10487	0.23999	-0.29084	0.29084	0.89843	0.19728	0.98243	0.47102	0.57697	0.17222	0.00157	0.00011	0.00011	turquoise
RRAGD	9.54E-05	-0.12859	-0.12062	0.04362	-0.15215	-0.09165	-0.09686	0.44354	-0.44354	0.99901	0.09372	0.11606	0.57105	0.04696	0.23319	0.20756	1.23E-09	1.23E-09	blue
MIGA2	0.06907	-0.11947	-0.09871	-0.07892	0.14433	-0.1235	-0.04338	-0.13882	0.13882	0.36935	0.1196	0.19897	0.30485	0.05965	0.10755	0.57318	0.07019	0.07019	red
FABP5	0.02207	-0.08186	-0.02304	0.0563	-0.04653	-0.01199	-0.04472	0.01904	-0.01904	0.77445	0.28716	0.76484	0.46454	0.54562	0.8763	0.56134	0.80476	0.80476	grey
S100A6	0.04751	0.05802	0.06125	-0.05875	0.01167	0.20401	0.25588	-0.54881	0.54881	0.53723	0.451	0.42611	0.44528	0.87963	0.00744	0.00073	7.77E-15	7.77E-15	yellow
TNNI1	0.11588	0.08583	-0.04972	-0.01857	0.00898	0.10764	0.1594	-0.36088	0.36088	0.13122	0.26436	0.5184	0.80953	0.90718	0.16114	0.0373	1.24E-06	1.24E-06	yellow
LIPE	-0.02114	-0.02285	-0.05219	-0.03662	0.06446	-0.0148	-0.13216	0.23221	-0.23221	0.78374	0.7667	0.49781	0.63441	0.40225	0.84767	0.08487	0.00224	0.00224	grey
RAB19	-0.04092	-0.11762	-0.01788	-0.01347	0.09481	-0.01927	-0.10039	0.03663	-0.03663	0.59514	0.12549	0.81641	0.86116	0.21742	0.8025	0.19142	0.63432	0.63432	turquoise
TWF1	0.072	-0.16776	0.00365	0.02654	0.0671	-0.02197	-0.05438	-0.01337	0.01337	0.34934	0.02829	0.96221	0.73039	0.38318	0.77545	0.47994	0.86222	0.86222	turquoise
C8orf88	-0.0317	-0.0919	-0.10365	-0.02067	-0.08892	-0.13099	-0.00687	0.37264	-0.37264	0.68065	0.23189	0.1773	0.78841	0.24745	0.08769	0.92895	5.19E-07	5.19E-07	turquoise
PCGF5	0.0173	-0.11525	-0.00668	0.01582	-0.08143	0.008	-0.03429	0.19553	-0.19553	0.82227	0.13334	0.93086	0.83726	0.28971	0.91724	0.65614	0.01038	0.01038	turquoise
ATP10A	-0.07844	-0.04494	0.06644	0.1034	-0.12773	0.20203	0.13649	0.17274	-0.17274	0.30784	0.55949	0.38794	0.17837	0.09595	0.00805	0.07506	0.02386	0.02386	black
TMPRSS4	0.0279	0.05914	0.11833	0.0104	-0.01993	0.27042	0.2541	-0.48461	0.48461	0.71717	0.44225	0.12322	0.89258	0.7958	0.00035	0.0008	1.87E-11	1.87E-11	yellow
IDNK	-0.04485	-0.00344	-0.13118	-0.11666	0.08678	-0.12867	-0.20749	0.02753	-0.02753	0.56028	0.96437	0.08723	0.12864	0.25907	0.0935	0.00647	0.72081	0.72081	grey
COL8A2	-0.08117	-0.02364	0.08343	0.08244	-0.18688	0.2102	0.11422	0.14933	-0.14933	0.29122	0.75894	0.27795	0.28371	0.01438	0.00579	0.13689	0.05124	0.05124	black
PTPRM	-0.07321	-0.15316	-0.19707	-0.00455	-0.01132	-0.05987	-0.13925	0.43361	-0.43361	0.34133	0.04551	0.00978	0.95294	0.88313	0.43663	0.0693	3.14E-09	3.14E-09	blue
EMD	0.05249	-0.12861	-0.13985	-0.11599	0.01296	-0.04401	0.08872	-0.19701	0.19701	0.49537	0.09365	0.0681	0.13086	0.86636	0.5676	0.24853	0.0098	0.0098	grey
MRPS16	0.0083	0.03793	-0.02527	-0.01021	-0.05387	-0.07875	0.19052	-0.23206	0.23206	0.91419	0.62232	0.74282	0.89459	0.4841	0.30593	0.01256	0.00226	0.00226	turquoise
RDH12	-0.07526	-0.01034	-0.26295	-0.21763	0.17761	-0.22751	-0.22073	0.14771	-0.14771	0.32793	0.89318	0.00051	0.00425	0.02012	0.00277	0.00372	0.05387	0.05387	grey
SMIM10L1	-0.02913	-0.15646	-0.13994	-0.06747	0.11588	-0.24986	-0.05358	0.12048	-0.12048	0.70526	0.041	0.06793	0.38057	0.13123	0.00098	0.48639	0.1165	0.1165	grey
S100A3	-0.10653	-0.04024	0.10234	0.04756	-0.15578	0.19643	0.17547	-0.26236	0.26236	0.16551	0.60129	0.18289	0.53673	0.04189	0.01002	0.0217	0.00053	0.00053	grey
TBX1	-0.11444	-0.01448	-0.05556	0.03233	0.06835	-0.17231	-0.06706	0.22177	-0.22177	0.13613	0.85086	0.47047	0.67468	0.37438	0.02422	0.38347	0.00356	0.00356	grey
KATNA1	-0.03153	-0.09019	-0.00566	0.01768	-0.09511	0.07687	0.10022	0.13316	-0.13316	0.6823	0.24076	0.94145	0.81842	0.21594	0.31762	0.19218	0.08252	0.08252	turquoise
PPFIBP2	0.04017	-0.00985	0.08596	0.06256	0.10152	0.25412	0.06114	-0.08952	0.08952	0.60191	0.89827	0.2636	0.41626	0.18643	0.0008	0.42693	0.24425	0.24425	grey
F11R	0.1326	-0.00133	0.05067	-0.02576	-0.01478	0.18032	0.19132	-0.51997	0.51997	0.08383	0.98619	0.51042	0.73806	0.84781	0.01827	0.01219	3.14E-13	3.14E-13	yellow
TJP2	0.06217	-0.05346	0.05282	0.07216	-0.09507	0.29401	0.1991	-0.35524	0.35524	0.41917	0.48739	0.4926	0.34832	0.21615	9.50E-05	0.00904	1.86E-06	1.86E-06	yellow
ABCG2	-0.05996	-0.05516	-0.02873	0.0582	-0.01823	-0.01276	-0.10415	0.42824	-0.42824	0.43594	0.47364	0.70909	0.4496	0.81292	0.86844	0.17521	5.13E-09	5.13E-09	grey
NPR3	-0.05852	-0.01543	0.05241	-0.01808	-0.12282	0.21308	0.08456	-0.0076	0.0076	0.44706	0.8412	0.49602	0.81447	0.10952	0.00514	0.27147	0.92143	0.92143	black
SLC7A1	0.00569	-0.04665	-0.04239	-0.0455	-0.09888	-0.02217	-0.06089	0.02599	-0.02599	0.94114	0.54461	0.58198	0.5546	0.19821	0.77346	0.42889	0.73575	0.73575	turquoise
DLGAP5	-0.03699	0.07635	0.09426	0.01346	-0.0851	0.16739	0.32628	-0.42784	0.42784	0.63098	0.32096	0.2201	0.8613	0.26845	0.02864	1.33E-05	5.32E-09	5.32E-09	pink
NUP210	-0.04001	-0.12928	-0.13807	-0.05359	-0.00345	-0.06637	-0.11829	0.13882	-0.13882	0.6034	0.09195	0.07172	0.48638	0.9643	0.38842	0.12335	0.07018	0.07018	grey
TYSND1	0.05155	-0.00666	-0.06178	-0.04259	0.11413	-0.14793	0.07327	-0.25678	0.25678	0.50314	0.93115	0.42217	0.58021	0.13717	0.05349	0.34088	0.0007	0.0007	grey
MOB3A	-0.07223	-0.11048	-0.01429	-0.01649	-0.13554	0.00864	0.01664	0.1685	-0.1685	0.34784	0.15027	0.85284	0.83054	0.07714	0.91071	0.82893	0.02759	0.02759	blue
FGF20	-0.0522	0.13784	0.15537	0.04022	0.05071	0.18863	0.11495	-0.18495	0.18495	0.4977	0.0722	0.04244	0.6015	0.51006	0.01348	0.13436	0.01545	0.01545	grey
MTX3	-0.07179	-0.09799	-0.13149	-0.01288	-0.07811	-0.05551	-0.0299	0.33019	-0.33019	0.35074	0.20228	0.08647	0.86723	0.30987	0.47087	0.69788	1.03E-05	1.03E-05	turquoise
EXTL1	-0.09748	-0.12318	-0.15908	-0.14761	-0.10018	-0.06204	-0.03629	0.29926	-0.29926	0.20467	0.10848	0.03769	0.05402	0.19233	0.42017	0.63746	7.00E-05	7.00E-05	turquoise
SHROOM2	-0.08164	-0.1837	-0.18681	0.00157	-0.08472	-0.21275	0.05828	0.09715	-0.09715	0.28843	0.01617	0.01443	0.9837	0.27056	0.00521	0.44893	0.2062	0.2062	grey
IFITM2	-0.05121	0.00328	0.02265	0.04356	-0.15173	0.16767	0.12782	-0.1205	0.1205	0.5059	0.966	0.76871	0.57161	0.04758	0.02838	0.09571	0.11642	0.11642	tan
TRIQK	0.01986	-0.0798	-0.07263	0.00146	0.04775	-0.01558	-0.02773	0.08561	-0.08561	0.79655	0.29951	0.34512	0.98488	0.53511	0.83976	0.71883	0.26558	0.26558	turquoise
PSTPIP1	-0.04821	0.05417	0.07604	0.0472	-0.08772	0.09774	0.06175	0.27665	-0.27665	0.53122	0.48122	0.32293	0.53986	0.25391	0.20344	0.42234	0.00025	0.00025	blue
SIRT1	-0.08389	-0.19598	0.02314	0.05577	-0.10753	0.11147	-0.09674	0.3615	-0.3615	0.27531	0.0102	0.76384	0.46876	0.16156	0.14663	0.20812	1.19E-06	1.19E-06	turquoise
FUT4	-0.00366	-0.02931	0.00486	0.04194	0.01531	0.23634	0.11405	-0.2073	0.2073	0.96206	0.70356	0.94971	0.586	0.84243	0.00186	0.13748	0.00652	0.00652	yellow
RCC1	-0.01765	-0.06759	0.08752	0.1411	-0.1381	0.18493	0.22373	-0.26042	0.26042	0.81881	0.37974	0.25499	0.06566	0.07167	0.01546	0.00327	0.00058	0.00058	pink
RNF125	-0.1401	0.01849	0.08138	0.03638	-0.07673	0.17159	-0.03636	0.28145	-0.28145	0.06761	0.81031	0.29	0.6366	0.31852	0.02483	0.63682	0.00019	0.00019	turquoise
ZKSCAN3	-0.03384	-0.1013	-0.09088	-0.00419	0.0571	-0.01347	-0.06873	0.29622	-0.29622	0.66036	0.18738	0.23715	0.95659	0.45823	0.86124	0.37173	8.36E-05	8.36E-05	turquoise
PLEKHO2	-0.10447	-0.03751	0.04534	0.06188	-0.2072	0.17563	0.05984	0.38755	-0.38755	0.17387	0.62621	0.55593	0.42139	0.00654	0.02158	0.43685	1.63E-07	1.63E-07	blue
ATP6V0D1	0.05875	-0.07295	-0.135	-0.10622	0.06576	-0.14482	-0.00495	0.1315	-0.1315	0.44528	0.34303	0.07832	0.16675	0.39284	0.05878	0.94875	0.08645	0.08645	grey
PON3	0.10454	-0.08125	-0.09128	-0.03836	0.02928	-0.11007	0.15073	-0.04521	0.04521	0.17359	0.29078	0.23507	0.61836	0.70387	0.15181	0.04908	0.55706	0.55706	grey
C9orf152	0.09774	0.05547	0.07908	0.04004	0.10673	0.2916	0.05735	-0.30116	0.30116	0.20346	0.47114	0.30389	0.6031	0.16471	0.00011	0.45625	6.26E-05	6.26E-05	yellow
ODF2L	-0.02762	-0.17338	-0.02032	0.02488	-0.03701	-0.0482	-0.049	0.08485	-0.08485	0.71989	0.02334	0.79196	0.74668	0.63084	0.53131	0.52448	0.26985	0.26985	turquoise
LANCL2	-0.13038	-0.04242	-0.13916	-0.03988	0.09731	-0.22757	-0.17656	0.2993	-0.2993	0.08919	0.58169	0.06949	0.60458	0.20543	0.00276	0.02088	6.99E-05	6.99E-05	turquoise
SEPTIN6	-0.14321	-0.12506	0.01904	0.02635	-0.17894	0.06945	-0.05387	0.42427	-0.42427	0.06168	0.10314	0.80479	0.73226	0.01919	0.36675	0.48402	7.34E-09	7.34E-09	blue
LPIN2	0.05205	-0.22904	0.04318	0.02168	0.01077	0.11342	-0.0112	0.02795	-0.02795	0.49899	0.00258	0.57495	0.7784	0.88882	0.13967	0.88442	0.7167	0.7167	turquoise
LPAR2	0.05002	0.12225	0.08555	0.02191	0.04989	0.20454	0.23223	-0.46157	0.46157	0.51592	0.11118	0.26588	0.77609	0.51699	0.00729	0.00224	2.10E-10	2.10E-10	yellow
PTPN18	0.08422	0.01928	0.01742	-0.05675	0.03772	0.06322	0.06198	-0.18101	0.18101	0.27341	0.80231	0.82107	0.46094	0.62429	0.41139	0.42063	0.01782	0.01782	grey

TEFM	-0.0176	-0.11892	-0.05209	-0.07181	0.13287	-0.04795	0.02282	0.06824	-0.06824	0.81931	0.12134	0.49866	0.35064	0.08318	0.53347	0.76703	0.37513	0.37513	turquoise
TEKT2	-0.01975	-0.19397	-0.17124	-0.0084	0.11537	-0.23856	-0.15402	0.16092	-0.16092	0.79765	0.01102	0.02513	0.91315	0.13294	0.00168	0.0443	0.03551	0.03551	brown
PPFIA3	0.01124	-0.03965	-0.22117	-0.11826	0.27383	-0.30868	-0.18783	0.07295	-0.07295	0.88401	0.60663	0.00365	0.12343	0.00029	4.00E-05	0.01389	0.34303	0.34303	brown
ESYT1	0.06376	-0.19939	-0.02856	-0.02489	-0.07989	-0.112	-0.17545	0.19391	-0.19391	0.40737	0.00894	0.71081	0.74658	0.29895	0.14473	0.02172	0.01104	0.01104	turquoise
TFEC	-0.10906	0.01626	0.09666	0.07637	-0.23109	0.1471	0.07621	0.43296	-0.43296	0.15561	0.83281	0.20853	0.32079	0.00236	0.05487	0.32183	3.33E-09	3.33E-09	blue
PIK3R3	-0.15355	-0.09749	-0.14131	-0.0256	-0.04207	-0.09132	-0.07344	0.443	-0.443	0.04496	0.2046	0.06525	0.73962	0.58481	0.23489	0.33975	1.30E-09	1.30E-09	turquoise
TCF7L1	-0.15719	-0.09256	-0.01652	0.07087	-0.12627	0.00488	0.00427	0.27192	-0.27192	0.04005	0.22857	0.83021	0.35696	0.09984	0.94951	0.95579	0.00032	0.00032	blue
MS4A4E	-0.06152	-0.04367	-0.07487	0.00707	-0.0266	0.03299	0.00091	0.45053	-0.45053	0.42412	0.57058	0.33046	0.92685	0.72979	0.66844	0.9906	6.29E-10	6.29E-10	blue
FHIT	0.00442	0.06001	-0.06478	-0.05852	0.23065	0.05937	0.01454	-0.03728	0.03728	0.95423	0.43557	0.39994	0.44705	0.0024	0.44054	0.85026	0.62828	0.62828	grey
VCAM1	-0.1406	-0.15264	0.08704	0.14329	-0.22368	0.21576	0.13133	0.19957	-0.19957	0.06662	0.04625	0.25766	0.06153	0.00327	0.00459	0.08686	0.00887	0.00887	blue
RAB3GAP:	-0.0348	-0.11098	-0.04509	-0.00325	-0.06213	-0.0155	0.02379	0.16373	-0.16373	0.65132	0.14843	0.55818	0.96634	0.41947	0.84054	0.75746	0.03237	0.03237	turquoise
TMEM273	-0.14026	-0.05065	0.0709	0.08313	-0.17069	0.08863	-0.01442	0.49523	-0.49523	0.06728	0.51059	0.35679	0.27972	0.0256	0.24903	0.8515	5.75E-12	5.75E-12	blue
GAD1	0.11932	-0.03161	-0.08064	-0.00174	0.13523	0.01033	-0.05277	-0.24491	0.24491	0.12008	0.68152	0.29444	0.98202	0.07782	0.89328	0.49301	0.00124	0.00124	grey
TCTA	-0.05806	-0.1206	-0.20152	-0.1633	0.06166	-0.15007	-0.20106	0.19929	-0.19929	0.45063	0.11614	0.00822	0.03283	0.42305	0.0501	0.00837	0.00897	0.00897	turquoise
CTSC	-0.11997	-0.06602	-0.06381	0.01472	-0.17465	0.12637	0.20102	0.07913	-0.07913	0.11807	0.39094	0.40705	0.84848	0.02233	0.09957	0.00838	0.30357	0.30357	blue
GP2	-0.03324	0.03356	0.02	0.07946	-0.05518	0.06187	-0.02662	0.08216	-0.08216	0.66605	0.66299	0.79514	0.30158	0.47345	0.42149	0.72966	0.28537	0.28537	magenta
CASS4	-0.17372	-0.02594	0.03507	0.04687	-0.16752	0.13673	0.05557	0.56321	-0.56321	0.02307	0.73627	0.64886	0.54266	0.02853	0.07454	0.47034	1.07E-15	1.07E-15	blue
HOXA1	0.09542	0.08873	0.12483	0.13506	-0.21398	0.14377	0.22481	0.06416	-0.06416	0.21442	0.24846	0.10378	0.0782	0.00495	0.06065	0.00312	0.40443	0.40443	grey
MTIF2	0.02594	0.07118	0.03374	-0.01413	-0.0303	0.08566	0.23032	-0.22225	0.22225	0.7363	0.35486	0.66134	0.85444	0.69398	0.26529	0.00244	0.00348	0.00348	turquoise
ATMIN	-0.04262	-0.14836	-0.05809	0.02066	-0.14126	-0.01207	0.08306	0.18447	-0.18447	0.57992	0.0528	0.45047	0.78858	0.06535	0.87546	0.28014	0.01572	0.01572	turquoise
TEPSIN	-0.01232	-0.08387	-0.03127	-0.03411	0.20115	-0.07912	0.02875	-0.15452	0.15452	0.87295	0.27541	0.68471	0.65788	0.00834	0.30366	0.70896	0.04359	0.04359	red
KRBA1	-0.1035	-0.1258	-0.22055	-0.06417	-0.04285	-0.23916	-0.12713	0.435	-0.435	0.17794	0.1011	0.00375	0.40436	0.57788	0.00163	0.09752	2.76E-09	2.76E-09	brown
SMOC1	-0.03101	-0.17819	-0.24476	-0.13991	0.1156	-0.33712	-0.13915	0.14772	-0.14772	0.68718	0.01971	0.00125	0.06798	0.13218	6.52E-06	0.0695	0.05384	0.05384	grey
MBTPS1	-0.09893	-0.18138	-0.0958	-0.02617	-0.10266	-0.00487	-0.02502	0.19913	-0.19913	0.19798	0.01759	0.21263	0.73405	0.18149	0.94962	0.74535	0.00903	0.00903	turquoise
DDT	0.03894	0.19379	-0.10912	-0.11814	0.07304	-0.0313	0.07377	-0.30675	0.30675	0.61307	0.0111	0.1554	0.12381	0.34243	0.68448	0.33762	4.49E-05	4.49E-05	grey
SMG6	-0.05446	-0.1987	-0.05203	-0.01151	-0.09672	-0.02869	-0.07753	0.42161	-0.42161	0.47927	0.00918	0.49918	0.8812	0.20821	0.7095	0.31349	9.31E-09	9.31E-09	turquoise
CUL1	-0.08692	-0.12827	-0.01592	0.00708	-0.05233	-0.07479	0.00163	0.15355	-0.15355	0.2583	0.09454	0.83631	0.92673	0.49664	0.33098	0.98312	0.04495	0.04495	turquoise
TRNT1	0.02016	-0.11069	-0.01053	-0.03664	-0.12038	0.11474	0.16221	-0.03079	0.03079	0.79357	0.14952	0.89129	0.63428	0.1168	0.13508	0.03403	0.68931	0.68931	turquoise
SPCS1	0.03494	0.01949	-0.08337	-0.18662	0.03737	-0.04956	-0.02632	-0.135	0.135	0.65002	0.80029	0.27834	0.01453	0.62749	0.51978	0.73256	0.07833	0.07833	grey
NKTR	-0.00629	-0.1614	-0.04899	-0.05827	0.03835	0.02122	-0.02833	0.02146	-0.02146	0.93489	0.03495	0.52454	0.44905	0.61848	0.7829	0.71305	0.78059	0.78059	red
SIRT2	0.04495	-0.22245	-0.05457	0.10049	-0.07801	0.01001	-0.01222	0.14369	-0.14369	0.55936	0.00345	0.47843	0.19094	0.31051	0.8966	0.87395	0.06079	0.06079	grey
TOR1A	-0.0488	-0.17262	-0.09349	-0.01662	-0.13456	-0.07658	-0.02092	0.20252	-0.20252	0.5262	0.02396	0.22391	0.8292	0.07931	0.3195	0.78589	0.0079	0.0079	turquoise
ITIH5	0.09354	-0.15064	-0.16374	0.03813	-0.07471	0.02846	-0.13939	0.29919	-0.29919	0.22363	0.04922	0.03236	0.62051	0.33148	0.71172	0.06903	7.03E-05	7.03E-05	grey
KIN	0.04585	-0.1164	0.0405	0.00991	-0.01068	0.07384	-0.02089	-0.0231	0.0231	0.55156	0.1295	0.5989	0.89762	0.88972	0.33712	0.78627	0.76426	0.76426	turquoise
VDR	-0.0242	-0.05256	0.04603	0.05327	-0.11462	0.20318	0.19487	-0.02717	0.02717	0.75337	0.49479	0.54992	0.48893	0.1355	0.00769	0.01065	0.72423	0.72423	black
ADGRL2	-0.07354	-0.17008	-0.02813	0.10331	-0.18399	0.09177	-0.03116	0.40544	-0.40544	0.33914	0.02615	0.71499	0.17873	0.016	0.23259	0.68574	3.77E-08	3.77E-08	blue
LGI3	0.02914	-0.13701	-0.15711	-0.08855	0.06926	-0.35284	-0.21687	0.41891	-0.41891	0.70515	0.07395	0.04016	0.24943	0.36803	2.21E-06	0.00438	1.18E-08	1.18E-08	brown
PCDHB14	0.06147	-0.08676	-0.09715	0.0715	0.03996	0.06141	-0.04245	0.02543	-0.02543	0.42448	0.25916	0.20619	0.35276	0.6038	0.42494	0.5814	0.74133	0.74133	grey
SMIM4	0.09006	-0.00844	-0.13731	-0.1881	0.0395	-0.17131	-0.0368	0.04382	-0.04382	0.24144	0.91274	0.07331	0.01375	0.60796	0.02507	0.63273	0.56928	0.56928	grey
GORASP2	0.03309	-0.06159	0.01591	0.0519	-0.08344	0.06799	0.06441	-0.06279	0.06279	0.66745	0.42355	0.83633	0.50019	0.27794	0.3769	0.40264	0.41458	0.41458	turquoise
PHC2	-0.07365	-0.18478	-0.04983	-0.01483	-0.14056	0.0234	0.02926	-0.02905	0.02905	0.33839	0.01555	0.51751	0.84738	0.0667	0.7613	0.70406	0.70601	0.70601	turquoise
RELB	-0.01665	0.02929	0.10288	0.03642	-0.06666	0.25458	0.21696	-0.21078	0.21078	0.82889	0.70374	0.18054	0.6363	0.38634	0.00078	0.00437	0.00565	0.00565	yellow
RACGAP1	0.06252	0.01077	0.05074	0.02357	-0.06462	0.03032	0.22658	-0.29452	0.29452	0.41661	0.88878	0.50989	0.75963	0.4011	0.69386	0.00288	9.22E-05	9.22E-05	pink
NAT14	0.02164	-0.10957	-0.20362	-0.06009	0.0621	-0.28992	-0.04703	-0.02386	0.02386	0.77873	0.15368	0.00756	0.43495	0.41973	0.00012	0.54133	0.75673	0.75673	grey
F2RL3	-0.10223	-0.16077	-0.0808	0.04955	-0.11773	0.01243	-0.17438	0.37395	-0.37395	0.18334	0.03568	0.29348	0.51985	0.12514	0.87176	0.02254	4.70E-07	4.70E-07	blue
ATG2B	-0.01927	-0.15706	-0.09954	0.03377	-0.00646	-0.05756	-0.12172	0.32552	-0.32552	0.80243	0.04021	0.19519	0.66101	0.93321	0.45458	0.11276	1.40E-05	1.40E-05	turquoise
ILDR2	0.03356	-0.13337	-0.15257	-0.08605	-0.06296	-0.01195	-0.00491	0.325	-0.325	0.66298	0.08204	0.04636	0.26312	0.41332	0.87671	0.94921	1.44E-05	1.44E-05	blue
GPCPD1	0.00234	-0.08687	0.04397	0.05727	0.00838	0.05518	0.00254	0.05682	-0.05682	0.97575	0.25859	0.56797	0.45685	0.91333	0.4735	0.97367	0.4604	0.4604	red
ITIH2	0.03699	-0.09861	-0.15846	-0.05439	0.06524	-0.03044	-0.10279	-0.14005	0.14005	0.63097	0.19943	0.03845	0.47983	0.39653	0.69269	0.18093	0.06769	0.06769	grey
PLAG1	0.01661	-0.06828	0.05299	0.0492	-0.18071	-0.02293	0.10546	-0.1733	0.1733	0.82927	0.37485	0.49126	0.52279	0.01802	0.7659	0.1698	0.02341	0.02341	grey
COTL1	-0.07997	-0.02233	0.09954	0.05842	-0.20456	0.10249	0.13425	-0.05789	0.05789	0.29846	0.77188	0.19521	0.4479	0.00728	0.18225	0.08001	0.45202	0.45202	black
PRRX2	-0.12126	0.00936	0.05271	0.08714	-0.09931	0.08149	0.1258	-0.06574	0.06574	0.11413	0.90327	0.49354	0.25711	0.19623	0.28932	0.10111	0.39292	0.39292	black
LRCH2	-0.03469	-0.16153	-0.15966	-0.06708	-0.05135	-0.16409	-0.13072	0.61175	-0.61175	0.65243	0.0348	0.03699	0.38336	0.50473	0.03198	0.08836	6.21E-19	6.21E-19	brown
OPA3	-0.02697	-0.10557	-0.15656	-0.09339	-0.01416	-0.13778	0.00059	0.22827	-0.22827	0.72621	0.16935	0.04086	0.22437	0.8542	0.07232	0.99387	0.00267	0.00267	turquoise
ENTPD3	-0.06268	0.10107	-0.16766	-0.05417	0.02273	-0.01748	0.03489	0.17891	-0.17891	0.41541	0.1884	0.02838	0.48162	0.76788	0.82046	0.65057	0.01922	0.01922	grey
MIDN	0.0502	-0.0567	-0.04519	-0.06801	0.10751	0.1553	0.04082	-0.26889	0.26889	0.51439	0.46135	0.55731	0.37676	0.16161	0.04254	0.59605	0.00038	0.00038	grey
TTK	-0.07499	0.10713	0.05188	0.01204	-0.09017	0.12351	0.32772	-0.36753	0.36753	0.32963	0.16311	0.50035	0.87584	0.24083	0.10753	1.21E-05	7.62E-07	7.62E-07	pink
RPL14	0.1034	0.11637	0.04063	-0.13734	-0.13455	0.17821	0.1481	-0.29494	0.29494	0.17835	0.12959	0.59771	0.07325	0.07933	0.0197	0.05322	9.00E-05	9.00E-05	purple
APOBEC2	0.02084	-0.03564	-0.13398	0.0578	-0.04606	-0.00227	-0.00096	0.18275	-0.18275	0.78674	0.64352	0.08063	0.45273	0.54971	0.97648	0.99003	0.01674	0.01674	magenta
LRPAP1	-0.07475	-0.12685	-0.21389	-0.07107	0.1288	-0.31652	-0.14302	0.17927	-0.17927	0.33121	0.09828	0.00497	0.3556	0.09316	2.47E-05	0.06202	0.01897	0.01897	brown
CHMP6	-0.05392	-0.05557	-0.04485	-0.07142	0.17531	-0.1177	-0.00378	-0.026	0.026	0.48369	0.47033	0.56027	0.35325	0.02182	0.12523	0.96082	0.73566	0.73566	green
PTPDC1	0.03288	-0.16255	-0.04595	-0.0103	-0.18721	0.02168	0.02475	0.26084	-0.26084	0.66946	0.03366	0.55061	0.89359	0.01421	0.77834	0.74799	0.00057	0.00057	turquoise
ZNF616	0.03456	-0.11078	-0.082	0.04955	-0.00638	0.0031	-0.01775	0.17754	-0.17754	0.65361	0.14919	0.28633	0.51985	0.93403	0.96788	0.81774	0.02017	0.02017	turquoise
ZKSCAN8	-0.08769	-0.13979	-0.07871	0.00085	-0.07797	0.03203	-0.06228	0.33625	-0.33625	0.2541	0.06823	0.30614	0.99117	0.31075	0.67748	0.41837	6.91E-06	6.91E-06	turquoise
PDZD4	-0.02483	-0.12608	-0.23301	-0.09007	0.1374	-0.35643	-0.23842	0.44111	-0.44111	0.74714	0.10036	0.00216	0.24136	0.07313	1.71E-06	0.00169	1.56E-09	1.56E-09	brown
ACVR2B	0.04298	-0.17536	-0.19362	-0.10278	-0.09698	-0.15025	-0.02851	0.09609	-0.09609	0.57669	0.02178	0.01117	0.18099	0.20698	0.04982	0.71125	0.21124	0.21124	turquoise
BCL2L2	0.01505	-0.1907	-0.07212	-0.02767	0.06248	-0.16397	-0.13456	0.24257	-0.24257	0.84512	0.01248	0.34854	0.71937	0.41688	0.03212	0.07931	0.00139	0.00139	turquoise
PTX3	-0.05174	-0.13687	-0.06413	0.03993	-0.11202	-0.0049	-0.04183	0.46515	-0.46515	0.50156	0.07425	0.40466	0.60411	0.14464	0.9493	0.58697	1.46E-10	1.46E-10	blue
TRABD2A	0.07356	0.0809	0.17255	0.02339	-0.09504	0.19614	0.14288	-0.22212	0.22212	0.33901	0.29287	0.02402	0.76139	0.21625	0.01014	0.06229	0.0035	0.0035	grey
UBE2D4	0.0286	-0.0817	-0.11863	-0.01455	-0.02238	-0.11465	-0.15295	0.40556	-0.40556	0.71041	0.28808	0.12227	0.8502	0.77139	0.1354	0.0458	3.74E-08	3.74E-08	brown
CD200	-0.08536	-0.16608	-0.15293	-0.06201	-0.0258	-0.17596	-0.22524	0.61684	-0.61684	0.26699	0.02993	0.04583	0.42042	0.73768	0.02133	0.00306	2.64E-19	2.64E-19	brown
TTC1	0.01639	-0.08928	-0.07284	-0.06397	-0.12036	-0.03281	0.07147	-0.00542	0.00542	0.83147	0.24552	0.34372	0.40582	0.11688	0.67007	0.35295	0.94388	0.94388	turquoise
MMP15	0.11159	-0.08201	-0.12613	-0.05765	0.1414	0.07736	0.0423	-0.30673	0.30673	0.14621	0.28624	0.10022	0.45388	0.06507	0.31457	0.58276	4.50E-05	4.50E-05	grey
QKI	-0.09672	-0.10448	0.03394	0.11311	-0.25684	0.12079	0.06066	0.41242	-0.41242	0.20823	0.17385	0.65943	0.14076	0.0007	0.11556	0.4306	2.08E-08	2.08E-08	blue
KIF21B	-0.0458	0.06884	-0.09476	-0.09066	-0.01633	-0.03314	-0.02323	0.17438	-0.17438	0.55192	0.37095	0.21765	0.23828	0.83214	0.667	0.763	0.02254	0.02254	blue
CISH	-0.02942	-0.12855	0.0081	0.01538	-0.073	0.03711	-0.11228	0.3615	-0.3615	0.70247	0.09382	0.91624	0.84177	0.34267	0.62988	0.14372	1.19E-06	1.19E-06	grey
UBE4A	-0.04666	-0.12557	0.00255	0.01219	-0.17349	0.0927	0.10374	0.17655	-0.17655	0.54452	0.10173	0.9736	0.87431	0.02325	0.22783	0.17691	0.02089	0.02089	turquoise
PTPN14	-0.03099	-0.09307	0.02682	0.07209	-0.26911	0.16619	0.21213	0.08985	-0.08985	0.68744	0.226	0.7277	0.34878	0.00037	0.02983	0.00535	0.24255	0.24255	turquoise
VASP	0.07853	0.02619	0.09391	-0.00188	-0.00281	0.24853	0.19746	-0.40244	0.40244	0.30729	0.73381	0.22181	0.98055	0.97094	0.00105	0.00963	4.86E-08	4.86E-08	yellow
PRKX	-0.25587	-0.01651	0.00678	0.03857	-0.26147	0.17252	0.11278	0.09868	-0.09868	0.00073	0.83033	0.92984	0.61651	0.00055	0.02404	0.14191	0.19913	0.19913	turquoise
BSCL2	0.0054	-0.17731	-0.10451	-0.02395	0.09128	-0.20017	-0.13646	0.16209	-0.16209	0.94416	0.02034	0.17372	0.75582	0.23507	0.00866	0.07514	0.03417	0.03417	grey
OBP2A	-0.03835	0.08673	-0.01072	-0.00135	-0.04494	-0.00465	0.04738	-0.10404	0.10404	0.61849	0.25934	0.88933	0.98601	0.55946	0.95192	0.53828	0.17568	0.17568	grey
NPW	0.0186	0.01334	-0.23507	-0.18375	0.25541	-0.11822	-0.05357	-0.04557	0.04557	0.80918	0.86252	0.00197	0.01614	0.00075	0.12357	0.48653	0.55395	0.55395	grey
AMBRA1	0.06378	-0.11201	-0.00171	-0.00411	-0.03051	0.06592	0.05585	-0.08784	0.08784	0.40727	0.1447	0.98225	0.95742	0.69205	0.39169	0.46814	0.25328	0.25328	turquoise
TRIM46	-0.04504	-0.13924	-0.14527	-0.1556	0.1905	-0.27476	-0.11147	0.05241	-0.05241	0.55859	0.06932	0.05799	0.04213	0.01257	0.00028	0.14664	0.49603	0.49603	brown
TLR6	-0.12491	-0.09183	0.12486	0.03965	-0.21144	0.17381	0.11345	0.20312	-0.20312	0.10358	0.23228	0.10369	0.60665	0.0055	0.02299	0.13956	0.00771	0.00771	blue
PRCC	0.06252	-0.02619	-0.09275	-0.07407	-0.00335	0.01357	0.18892	-0.19109	0.19109	0.41658	0.73382	0.22758	0.33562	0.96532	0.86014	0.01334	0.01229	0.01229	greenyellow
EXOC2	-0.03014	-0.07359	-0.02228	-0.05411	-0.09117	-0.04645	-0.0216	0.33138	-0.33138	0.69551	0.33878	0.77236	0.48208	0.23567	0.54629	0.7792	9.54E-06	9.54E-06	turquoise
MAT1A	0.04465	0.10274	0.03417	0.04369	-0.07391	-0.01031	-0.04432	0.18021	-0.18021	0.56198	0.18116	0.65731	0.57043	0.33667	0.89354	0.56489	0.01834	0.01834	magenta
CADPS2	-0.0473	-0.00323	0.14921	0.1616	-0.07278	0.22625	0.05892	0.101	-0.101	0.53899	0.96657	0.05145	0.03473	0.34416	0.00292	0.44397	0.18869	0.18869	turquoise
CCDC124	0.04029	0.00992	-0.13008	-0.00957	0.22689	-0.14417	0.05538	-0.28852	0.28852	0.60079	0.89751	0.08994	0.90111	0.00284	0.05994	0.47185	0.00013	0.00013	green
MYEOV	0.05867	-0.00323	0.13895	0.09028	-0.06104	0.16779	0.23536	-0.44593	0.44593	0.44592	0.96652	0.0699	0.24026	0.42774	0.02827	0.00194	9.81E-10	9.81E-10	yellow
RAB11FIP:	0.02376	-0.02762	0.0875	0.06171	-0.05158	0.21392	0.09296	-0.02788	0.02788	0.75775	0.71994	0.25511	0.42264	0.50284	0.00496	0.22656	0.71742	0.71742	turquoise
IGFBP2	0.01073	-0.09711	0.0197	-0.00141	0.12595	-0.10419	-0.25872	0.09218	-0.09218	0.88919	0.20637	0.79814	0.98537	0.10069	0.17504	0.00063	0.23046	0.23046	grey
CTDSPL	0.00066	-0.09309	-0.03069	-0.05689	-0.14568	0.15656	0.24594	-0.1117	0.1117	0.99312	0.22592	0.69029	0.45987	0.05728	0.04086	0.00119	0.14581	0.14581	turquoise
TNFRSF17	-0.18639	0.08339	0.02902	-0.03092	-0.09448	0.10186	0.03314	0.29698	-0.29698	0.01465	0.27819	0.70635	0.68812	0.21901	0.18495	0.66697	8.00E-05	8.00E-05	blue
MEAF6	-0.06088	-0.2461	-0.12392	-0.12167	0.0113	-0.16135	-0.1941	0.18093	-0.18093	0.42897	0.00118	0.10634	0.1129	0.88338	0.035	0.01097	0.01788	0.01788	turquoise
DNAJB5	-0.06714	-0.14419	-0.09787	-0.0201	-0.07674	-0.1182	-0.15235	0.46426	-0.46426	0.3829	0.0599	0.20285	0.79417	0.31847	0.12362	0.04668	1.60E-10	1.60E-10	salmon
SNRPN	-0.01489	-0.07531	-0.19193	-0.11783	0.02154	-0.20913	-0.26162	0.39474	-0.39474	0.84677	0.32762	0.01191	0.1248	0.77973	0.00605	0.00055	9.16E-08	9.16E-08	grey
USHBP1	-0.11386	0.00411	-0.13422	0.05292	0.08548	-0.09265	-0.09668	0.50372	-0.50372	0.13812	0.95749	0.08008	0.49182	0.26628	0.22809	0.20842	2.18E-12	2.18E-12	blue
DYNC1I1	-0.02702	-0.15326	-0.17213	-0.09795	0.0817	-0.2513	-0.22837	0.45591	-0.45591	0.72572	0.04537	0.02437	0.20246	0.28811	0.00091	0.00266	3.70E-10	3.70E-10	brown
PROCR	0.01804	-0.046	0.13844	0.12582	-0.06672	0.20545	0.03379	0.06086	-0.06086	0.81487	0.55021	0.07095	0.10105	0.38594	0.00702	0.66087	0.42909	0.42909	blue
RASSF7	0.14994	-0.01061	-0.07176	-0.05823	0.25253	-0.00259	0.11956	-0.61973	0.61973	0.05031	0.89048	0.35094	0.44931	0.00086	0.97314	0.11933	1.61E-19	1.61E-19	green
TMEM63C	-0.02004	-0.11696	-0.23026	-0.11112	0.11379	-0.30493	-0.21916	0.41242	-0.41242	0.79475	0.12764	0.00245	0.14792	0.13837	5.01E-05	0.00398	2.08E-08	2.08E-08	brown
TNFSF12	-0.12272	-0.03443	-0.01197	0.06118	-0.16675	0.07786	0.06724	0.31325	-0.31325	0.10981	0.65481	0.87651	0.42664	0.02927	0.3114	0.38225	3.02E-05	3.02E-05	blue
ZCCHC7	-0.00434	0.00954	-0.12368	-0.07938	-0.04683	-0.01758	-0.01343	0.05507	-0.05507	0.95507	0.90149	0.10704	0.30202	0.54304	0.81951	0.86161	0.47434	0.47434	turquoise
GYPC	-0.08672	-0.02041	0.00472	0.09031	-0.19929	0.1046	-0.04024	0.43616	-0.43616	0.25939	0.791	0.95118	0.24013	0.00897	0.17336	0.60127	2.48E-09	2.48E-09	blue
GARS	0.09954	-0.05118	-0.00534	0.00508	-0.0002	-0.07845	0.04869	-0.12382	0.12382	0.19521	0.50617	0.94472	0.94744	0.99788	0.30774	0.52709	0.10663	0.10663	grey
RUNDC1	0.02906	-0.15077	-0.14523	-0.06981	0.18744	-0.14502	-0.12489	-0.01919	0.01919	0.7059	0.04902	0.05805	0.36424	0.01409	0.05842	0.10363	0.80324	0.80324	turquoise
HIPK1	-0.03647	-0.20962	0.02058	0.08129	-0.08082	0.14159	-0.06957	0.22991	-0.22991	0.63577	0.00593	0.78937	0.29054	0.29333	0.06471	0.36589	0.00249	0.00249	turquoise
ZNF268	-0.06033	-0.09591	-0.045	-0.03175	0.0226	-0.0342	-0.09579	0.28431	-0.28431	0.43314	0.21206	0.55889	0.68018	0.76923	0.65701	0.21267	0.00016	0.00016	turquoise
MED26	-0.04746	-0.13559	-0.11611	0.01422	0.07208	-0.03016	-0.13411	0.07577	-0.07577	0.53762	0.07701	0.13045	0.85351	0.34882	0.69538	0.08032	0.32466	0.32466	grey
RTL10	-0.14752	-0.09269	-0.09873	-0.06009	-0.07063	-0.14544	0.02473	0.06353	-0.06353	0.05417	0.2279	0.19891	0.43499	0.35865	0.05769	0.74821	0.40912	0.40912	turquoise
RAB33B	-0.09525	-0.24	-0.06395	0.03318	-0.03734	0.02651	-0.10349	0.28789	-0.28789	0.21527	0.00157	0.40599	0.66659	0.62773	0.73075	0.178	0.00013	0.00013	turquoise
CC2D1B	-0.08358	-0.13392	-0.00303	0.00217	-0.03904	0.04157	0.05009	-0.04233	0.04233	0.27712	0.08076	0.96858	0.97756	0.61217	0.58934	0.51528	0.58251	0.58251	turquoise
DYNLL2	0.08671	-0.17499	-0.21672	-0.10982	0.01009	-0.23455	-0.0743	0.12809	-0.12809	0.25945	0.02207	0.00441	0.15274	0.89584	0.00202	0.33417	0.09499	0.09499	turquoise
RPAP1	-0.11664	-0.16611	-0.06919	-0.01953	-0.01396	-0.08781	0.00833	-0.05502	0.05502	0.12868	0.02991	0.36855	0.7999	0.85623	0.25344	0.91387	0.47476	0.47476	turquoise
ZC3HC1	-0.03326	-0.0273	-0.11864	-0.03614	0.01345	-0.20594	-0.01334	0.08564	-0.08564	0.66585	0.723	0.12221	0.63886	0.86135	0.00689	0.86249	0.26538	0.26538	grey
TRMT112	-0.03488	-0.04239	-0.05161	-0.06429	-0.07613	-0.09592	0.11534	-0.22122	0.22122	0.65057	0.58196	0.50264	0.40352	0.32233	0.21203	0.13303	0.00364	0.00364	grey
FOXA3	0.10574	0.07027	-0.01926	-0.02858	0.2554	0.13937	-0.07235	-0.27631	0.27631	0.1687	0.36112	0.80261	0.71055	0.00075	0.06907	0.34701	0.00025	0.00025	grey
SP110	-0.04803	-0.01884	0.07515	0.11929	-0.22796	0.21437	0.16578	0.0068	-0.0068	0.53272	0.80676	0.32863	0.12017	0.00271	0.00487	0.03024	0.92967	0.92967	turquoise
COMMD4	-0.02476	0.05007	-0.15105	-0.14836	0.10755	-0.15129	0.10712	-0.32801	0.32801	0.74786	0.51542	0.04861	0.0528	0.16146	0.04824	0.16315	1.19E-05	1.19E-05	grey
CNTFR	-0.1374	0.01875	-0.0906	0.13032	0.0923	-0.02614	-0.12841	0.41922	-0.41922	0.07312	0.80764	0.23858	0.08933	0.22988	0.73429	0.09417	1.15E-08	1.15E-08	grey
TEN1-CDI	-0.01399	-0.16009	-0.05704	-0.03403	0.27693	-0.10214	-0.0667	-0.05327	0.05327	0.85586	0.03648	0.45865	0.65862	0.00025	0.18374	0.38605	0.48899	0.48899	red
KCMF1	-0.00557	-0.06032	0.00787	-0.02958	-0.15126	0.08294	0.18247	-0.26397	0.26397	0.94239	0.4332	0.91868	0.70091	0.04829	0.28082	0.01691	0.00049	0.00049	turquoise
DDX56	0.00495	-0.07467	-0.09413	-0.02903	0.10676	-0.11477	0.0618	-0.30036	0.30036	0.94874	0.33172	0.22073	0.70624	0.16461	0.13498	0.42202	6.57E-05	6.57E-05	green
MPC1	-0.02079	0.01145	-0.07905	-0.02133	0.08313	-0.05522	-0.12072	0.20845	-0.20845	0.78728	0.88183	0.30406	0.78189	0.2797	0.47315	0.11577	0.00622	0.00622	grey
PPCDC	0.00462	-0.03724	-0.08515	-0.04724	0.21025	-0.08693	0.00917	-0.28266	0.28266	0.95222	0.62868	0.26816	0.53953	0.00578	0.25826	0.90523	0.00018	0.00018	grey
FAM120C	-0.07303	-0.20576	-0.18982	-0.02838	-0.01033	-0.26028	-0.14317	0.46381	-0.46381	0.34248	0.00694	0.0129	0.71255	0.89333	0.00059	0.06175	1.67E-10	1.67E-10	brown

SLC48A1	0.1233	-0.11863	-0.15251	0.01504	0.10093	-0.14029	-0.09011	0.07139	-0.07139	0.10814	0.12225	0.04644	0.84522	0.18902	0.06723	0.24114	0.35345	0.35345	grey
IQGAP1	-0.06675	-0.1202	0.07725	0.06676	-0.20424	0.20877	0.15961	-0.0159	0.0159	0.38573	0.11737	0.31527	0.38566	0.00737	0.00614	0.03704	0.83644	0.83644	turquoise
PCOLCE	-0.08298	-0.02525	0.07857	0.10358	-0.10885	0.0845	0.06538	0.24154	-0.24154	0.28056	0.74305	0.30702	0.17761	0.15642	0.27182	0.39555	0.00146	0.00146	black
PCK1	0.03243	-0.1654	0.06974	0.08985	0.21117	-0.03508	-0.12336	0.14037	-0.14037	0.67366	0.03062	0.36476	0.24251	0.00556	0.64877	0.10794	0.06708	0.06708	grey
POLR2E	-0.01702	0.02174	-0.13483	-0.07507	0.08512	-0.13764	0.02961	-0.13812	0.13812	0.82517	0.7778	0.07871	0.32912	0.26832	0.07261	0.70064	0.07162	0.07162	green
GNPDA1	0.03201	-0.0762	-0.06996	-0.01701	-0.01094	-0.00786	0.132	0.04147	-0.04147	0.67766	0.32187	0.36325	0.82519	0.88704	0.91877	0.08524	0.59023	0.59023	turquoise
ZNF821	-0.06664	-0.18543	-0.17922	-0.09399	0.0445	-0.19462	-0.20833	0.41977	-0.41977	0.3865	0.01518	0.019	0.22142	0.56327	0.01075	0.00625	1.10E-08	1.10E-08	brown
IQSEC1	-0.05789	-0.16358	-0.09027	-0.04336	-0.13096	-0.08949	-0.06858	0.32652	-0.32652	0.45201	0.03254	0.24034	0.57333	0.08775	0.24441	0.37277	1.31E-05	1.31E-05	turquoise
THPO	-0.04407	-0.00541	-0.08342	-0.02862	-0.13256	0.00171	-0.01128	0.39625	-0.39625	0.56707	0.944	0.27806	0.71022	0.08391	0.98233	0.88356	8.10E-08	8.10E-08	blue
RPL32	0.10365	0.12344	0.0101	-0.10526	-0.11582	0.15275	0.14539	-0.2449	0.2449	0.17731	0.10774	0.89571	0.17065	0.13142	0.04609	0.05778	0.00125	0.00125	purple
F10	-0.02778	-0.12438	-0.21688	-0.15285	0.1207	-0.3182	-0.25449	0.4624	-0.4624	0.71834	0.10506	0.00438	0.04594	0.11581	2.22E-05	0.00078	1.93E-10	1.93E-10	brown
BNIP2	-0.08559	-0.10892	0.0854	0.0511	-0.1376	0.14645	0.11399	0.05968	-0.05968	0.26567	0.15616	0.26674	0.50689	0.0727	0.05595	0.13768	0.43812	0.43812	turquoise
C16orf74	-0.04994	0.00403	0.04928	0.0755	-0.08528	0.03375	0.13092	-0.20167	0.20167	0.51655	0.95827	0.52215	0.32638	0.26741	0.66119	0.08787	0.00817	0.00817	grey
MFSD6L	-0.02936	-0.02787	-0.09515	0.04641	0.11655	0.07026	-0.03162	-0.046	0.046	0.70306	0.71747	0.21573	0.54668	0.129	0.36114	0.68144	0.55025	0.55025	grey
MACC1	0.09642	-0.07376	0.08877	0.10664	-0.03872	0.26087	0.11532	-0.38434	0.38434	0.20964	0.33765	0.24825	0.16506	0.61512	0.00057	0.1331	2.11E-07	2.11E-07	yellow
NTF4	0.0144	0.03006	0.07929	0.00701	-0.07297	0.05749	0.19473	-0.46171	0.46171	0.85172	0.69629	0.30258	0.92749	0.34288	0.4551	0.0107	2.07E-10	2.07E-10	grey
ATP9B	-0.06396	-0.13133	-0.13666	0.00269	0.07169	-0.14021	-0.16023	0.37925	-0.37925	0.40591	0.08686	0.07469	0.97216	0.35144	0.06739	0.0363	3.13E-07	3.13E-07	turquoise
TYMP	-0.04966	0.0673	0.03121	0.12064	-0.08072	0.12416	0.22612	-0.16649	0.16649	0.51892	0.38183	0.68528	0.11601	0.29396	0.10567	0.00294	0.02953	0.02953	black
KIFAP3	-0.00567	-0.08207	-0.11302	-0.08039	-0.04876	-0.0858	-0.05471	0.23075	-0.23075	0.94133	0.2859	0.14108	0.29594	0.52651	0.26448	0.47724	0.00239	0.00239	turquoise
SNAPC3	0.05325	-0.12118	-0.06543	-0.04537	-0.00242	-0.00793	0.01157	0.08942	-0.08942	0.48913	0.11437	0.39519	0.55572	0.9749	0.91806	0.88059	0.24482	0.24482	turquoise
MMRN2	-0.1229	-0.17134	-0.02982	0.09584	-0.11279	0.02083	-0.10282	0.52329	-0.52329	0.10929	0.02504	0.69864	0.21241	0.14188	0.78688	0.18084	2.09E-13	2.09E-13	blue
ITPR1	-0.11789	-0.1087	-0.02669	0.02256	-0.13035	-0.00687	-0.13086	0.58449	-0.58449	0.12461	0.15702	0.72891	0.76956	0.08925	0.92892	0.08801	4.75E-17	4.75E-17	turquoise
TMBIM6	0.09563	-0.18106	0.0296	0.03042	0.02793	-0.00183	-0.08046	-0.1484	0.1484	0.21344	0.01779	0.70072	0.6929	0.71692	0.98109	0.29551	0.05274	0.05274	turquoise
NQO2	-0.09427	0.0263	0.07388	0.09207	0.0782	0.16335	0.14227	0.06464	-0.06464	0.22002	0.73273	0.33689	0.23106	0.3093	0.03278	0.06342	0.40096	0.40096	grey
BTD	0.03727	-0.12216	-0.08047	-0.11392	-0.03777	-0.00207	-0.04177	0.23681	-0.23681	0.62846	0.11144	0.29542	0.1379	0.62379	0.97856	0.58752	0.00182	0.00182	turquoise
PML	0.01154	-0.00273	0.04147	0.0661	-0.07154	0.14023	0.1774	-0.25189	0.25189	0.88092	0.97169	0.59021	0.39033	0.35243	0.06735	0.02028	0.00089	0.00089	yellow
DPP3	0.04408	0.02871	-0.01585	-0.00739	-0.05615	0.03956	0.22673	-0.29799	0.29799	0.567	0.70932	0.837	0.92354	0.4657	0.60748	0.00286	7.54E-05	7.54E-05	turquoise
ABCB4	0.05652	-0.09812	-0.08299	-0.05432	0.05484	-0.22067	-0.19655	0.33424	-0.33424	0.4628	0.20169	0.28055	0.48042	0.47618	0.00373	0.00998	7.90E-06	7.90E-06	grey
TRMT5	-0.03208	-0.06632	-0.04867	-0.02296	-0.04609	-0.00917	0.08598	-0.0112	0.0112	0.67706	0.3888	0.52731	0.76569	0.54943	0.90528	0.26351	0.88443	0.88443	turquoise
GRINA	0.06188	-0.04303	-0.05044	-0.03807	-0.10936	-0.09888	0.13627	-0.08911	0.08911	0.42138	0.57627	0.51237	0.62101	0.1545	0.19822	0.07555	0.24646	0.24646	green
ASF1A	-0.09877	-0.0361	-0.00961	-0.0113	-0.09761	-0.06626	-0.08814	0.31048	-0.31048	0.19869	0.63924	0.9007	0.8834	0.20406	0.38924	0.25167	3.58E-05	3.58E-05	turquoise
CCDC113	0.01505	-0.06248	-0.18713	-0.10304	-0.00584	-0.09325	0.02964	0.03487	-0.03487	0.84505	0.41689	0.01425	0.17987	0.93954	0.2251	0.70038	0.65072	0.65072	grey
CD248	-0.10513	-0.04573	0.02301	0.0752	-0.16223	0.08552	0.05987	0.2938	-0.2938	0.17117	0.55256	0.76517	0.32828	0.03401	0.26609	0.43668	9.61E-05	9.61E-05	black
GCLC	0.01318	-0.00286	-0.03873	0.06994	-0.00604	-0.07635	-0.00632	0.18569	-0.18569	0.86412	0.97043	0.615	0.36332	0.93748	0.32091	0.93461	0.01503	0.01503	turquoise
AFAP1L2	-0.03789	-0.01348	0.1114	0.13655	-0.16269	0.12472	0.18194	-0.25232	0.25232	0.62267	0.86107	0.1469	0.07493	0.03351	0.1041	0.01724	0.00087	0.00087	yellow
ENHO	-0.13495	-0.10547	-0.22494	-0.06446	0.11442	-0.21519	-0.16513	0.32298	-0.32298	0.07844	0.16979	0.0031	0.40223	0.13618	0.00471	0.0309	1.64E-05	1.64E-05	brown
SPAAR	-0.13497	-0.05202	-0.07943	0.00893	-0.11413	-0.06803	-0.10745	0.28902	-0.28902	0.07839	0.49924	0.30174	0.90775	0.1372	0.37663	0.16185	0.00013	0.00013	grey
MAP4K5	-0.07033	-0.20047	0.00855	0.10034	-0.09682	0.05401	0.02758	0.20839	-0.20839	0.36067	0.00856	0.91162	0.19162	0.20777	0.48292	0.7203	0.00623	0.00623	turquoise
SNX7	-0.0859	-0.0785	0.06482	0.04829	-0.09193	0.10399	0.14109	-0.08671	0.08671	0.26396	0.30745	0.39964	0.53056	0.23176	0.17588	0.06567	0.25946	0.25946	turquoise
CCDC74B	0.05192	-0.18364	-0.20272	-0.00332	0.12103	-0.26508	-0.17037	0.12837	-0.12837	0.50003	0.0162	0.00784	0.96561	0.11482	0.00046	0.02589	0.09427	0.09427	grey
P2RX4	0.07704	-0.17796	0.04419	0.09784	0.11066	-0.00957	-0.08635	-0.0668	0.0668	0.31658	0.01988	0.56605	0.203	0.1496	0.90118	0.26146	0.38537	0.38537	grey
FAM83H	0.08025	0.08172	-0.05083	-0.06838	0.00089	0.03965	0.22342	-0.5785	0.5785	0.29676	0.28799	0.50914	0.37415	0.99076	0.60661	0.00331	1.17E-16	1.17E-16	green
TNPO1	-0.01359	-0.13188	-0.03873	-0.00772	-0.09136	0.01178	-0.05057	0.27976	-0.27976	0.86	0.08555	0.61505	0.92017	0.23465	0.87845	0.5113	0.00021	0.00021	turquoise
SLC35F6	0.03086	-0.05178	-0.09861	0.08207	-0.11586	-0.03485	0.19406	0.00534	-0.00534	0.68861	0.50117	0.19942	0.28593	0.1313	0.6509	0.01098	0.94474	0.94474	turquoise
FIBIN	-0.10515	-0.01308	0.04788	0.11542	-0.21892	0.11784	0.09444	0.36541	-0.36541	0.17109	0.86516	0.534	0.13277	0.00402	0.12479	0.2192	8.91E-07	8.91E-07	blue
RAP1GAP	0.0773	-0.02025	-0.12378	-0.00918	0.19713	0.0428	-0.15379	0.01475	-0.01475	0.31494	0.79262	0.10676	0.90518	0.00976	0.57831	0.04461	0.84819	0.84819	grey
AKAP17A	-0.06069	-0.21102	-0.10418	-0.02487	0.21298	-0.01045	-0.06391	-0.00541	0.00541	0.43041	0.0056	0.17509	0.74679	0.00516	0.89214	0.40627	0.94404	0.94404	red
YIF1A	0.02215	0.02351	-0.12795	-0.10085	0.15773	-0.18574	0.10042	-0.27142	0.27142	0.77366	0.76024	0.09538	0.18936	0.03936	0.01501	0.19129	0.00033	0.00033	green
WNT7A	-0.11397	0.01862	0.00233	0.01503	0.00182	0.06829	0.13998	-0.23694	0.23694	0.13775	0.80901	0.97589	0.84528	0.9812	0.3748	0.06785	0.00181	0.00181	grey
RNF170	0.00949	-0.07706	-0.07222	0.03758	0.01701	-0.07765	0.04406	0.0834	-0.0834	0.90196	0.31645	0.34791	0.62559	0.8252	0.31273	0.56716	0.27813	0.27813	turquoise
LZTFL1	0.00453	-0.13517	-0.146	-0.10073	-0.01351	-0.03868	-0.05836	0.22778	-0.22778	0.95308	0.07795	0.05673	0.1899	0.86079	0.61546	0.44831	0.00273	0.00273	turquoise
FAM174C	-0.00296	0.05156	-0.1244	-0.08025	0.08755	-0.10854	0.07349	-0.30855	0.30855	0.96936	0.503	0.105	0.2968	0.25484	0.15764	0.33944	4.03E-05	4.03E-05	green
EHMT1	-0.02786	-0.15032	-0.03377	0.01649	-0.04533	0.0421	0.0108	0.03193	-0.03193	0.71759	0.04971	0.66099	0.8305	0.55606	0.58454	0.88846	0.67841	0.67841	turquoise
ZNF879	-0.12377	-0.09105	-0.11038	-0.0119	-0.04627	-0.09791	-0.12351	0.46055	-0.46055	0.10679	0.23627	0.15067	0.8772	0.54788	0.20266	0.10751	2.33E-10	2.33E-10	turquoise
IQCE	0.00732	-0.07367	-0.12606	-0.02885	0.15179	-0.11398	-0.02614	-0.18238	0.18238	0.92432	0.3383	0.1004	0.708	0.04749	0.13772	0.73429	0.01697	0.01697	grey
ERCC8	-0.01056	-0.08701	-0.05862	-0.03852	-0.0313	-0.08773	-0.0414	0.20809	-0.20809	0.89098	0.25783	0.44631	0.61693	0.68444	0.25388	0.59085	0.00631	0.00631	turquoise
FBP1	-0.04211	0.03939	0.12901	0.10033	-0.0657	0.24403	0.18761	-0.20896	0.20896	0.58446	0.60901	0.09263	0.19168	0.39322	0.0013	0.014	0.00609	0.00609	yellow
AC118549	-0.05737	-0.14228	-0.04281	0.00534	-0.06992	-0.00465	-0.03999	0.25969	-0.25969	0.4561	0.0634	0.57822	0.94478	0.36352	0.95183	0.60351	0.0006	0.0006	turquoise
TNFRSF14	-0.06881	0.05781	0.10975	0.08327	0.10995	0.18761	0.14743	-0.32045	0.32045	0.37118	0.45262	0.15301	0.2789	0.15227	0.014	0.05432	1.93E-05	1.93E-05	green
C1orf56	0.01475	-0.15935	-0.26136	-0.07362	0.22382	-0.36907	-0.08092	0.06483	-0.06483	0.84815	0.03736	0.00055	0.33857	0.00325	6.79E-07	0.29273	0.39953	0.39953	grey
CD1A	-0.09625	-0.11598	0.07242	0.03735	-0.11372	0.12174	0.05728	0.10317	-0.10317	0.21044	0.1309	0.34655	0.62764	0.13862	0.11271	0.4568	0.17933	0.17933	grey
NRROS	-0.12219	-0.02071	0.00533	0.03356	-0.09398	0.07385	-0.02158	0.59152	-0.59152	0.11138	0.78803	0.94479	0.66306	0.22147	0.33707	0.77937	1.61E-17	1.61E-17	blue
ATP2B4	-0.02412	-0.09379	0.05231	0.09892	-0.14718	0.08359	0.05424	0.06769	-0.06769	0.75418	0.2224	0.49683	0.198	0.05474	0.27704	0.48106	0.37906	0.37906	turquoise
ADAT1	-0.11921	-0.10719	-0.0585	-0.0693	-0.12922	0.05929	0.07239	0.1231	-0.1231	0.12041	0.16287	0.44725	0.36777	0.0921	0.44108	0.34672	0.10871	0.10871	turquoise
BRMS1L	-0.03586	-0.13808	-0.00585	0.06136	-0.09941	-0.03512	-0.04275	0.32747	-0.32747	0.64148	0.07169	0.93951	0.42531	0.19581	0.64839	0.57879	1.23E-05	1.23E-05	turquoise
RAB3GAP:	0.04641	-0.08448	0.012	0.06347	-0.11695	0.04352	0.03066	0.14922	-0.14922	0.5467	0.27195	0.87619	0.40953	0.12768	0.57197	0.69055	0.05142	0.05142	turquoise
CCL28	0.01312	-0.0611	-0.02137	0.02408	-0.12568	0.18215	0.14632	-0.12633	0.12633	0.8648	0.42727	0.78142	0.75461	0.10145	0.0171	0.05617	0.09967	0.09967	grey
BICDL2	0.09019	0.04125	0.09893	0.06297	0.08618	0.20765	0.21394	-0.50327	0.50327	0.24074	0.59213	0.19796	0.41326	0.26236	0.00643	0.00496	2.29E-12	2.29E-12	yellow
AGAP4	0.00507	-0.04883	-0.0011	-0.07145	0.15063	-0.13353	-0.13665	-0.0277	0.0277	0.94756	0.52593	0.98856	0.35305	0.04923	0.08166	0.07472	0.71909	0.71909	red
FAM216A	-0.04051	-0.09704	-0.10985	-0.01547	-0.01856	-0.22306	-0.11909	0.38503	-0.38503	0.59888	0.2067	0.15264	0.8408	0.80957	0.00336	0.12079	1.99E-07	1.99E-07	brown
TSPAN32	-0.07838	0.08126	0.01983	0.05963	-0.07459	0.01698	-0.00487	0.29451	-0.29451	0.30819	0.29074	0.79685	0.43847	0.33228	0.8255	0.94957	9.23E-05	9.23E-05	blue
KPNA6	-0.02042	-0.19168	0.02617	-0.01668	-0.1011	0.07814	-0.076	0.15628	-0.15628	0.79094	0.01202	0.734	0.82857	0.18824	0.3097	0.32317	0.04124	0.04124	turquoise
ALG5	0.06737	0.05965	-0.16213	-0.10559	0.1306	-0.22914	-0.03763	-0.16001	0.16001	0.38132	0.43836	0.03412	0.1693	0.08864	0.00257	0.62514	0.03657	0.03657	grey
MOCS2	-0.05489	-0.13843	-0.15275	-0.05784	0.08762	-0.24436	-0.21704	0.42567	-0.42567	0.47583	0.07098	0.0461	0.45238	0.25447	0.00128	0.00435	6.47E-09	6.47E-09	brown
CSNK1G1	-0.07986	-0.20752	-0.10785	0.01476	-0.09655	-0.0684	0.02422	0.17033	-0.17033	0.2991	0.00646	0.16028	0.84809	0.20906	0.37402	0.75323	0.02592	0.02592	turquoise
TTC37	-0.05944	-0.13891	-0.09385	-0.03819	0.0146	-0.08314	-0.09361	0.37054	-0.37054	0.43999	0.06999	0.22209	0.61995	0.84972	0.27963	0.22331	6.08E-07	6.08E-07	turquoise
ZNF75A	-0.05649	-0.15158	-0.08068	0.01475	-0.05275	-0.03449	-0.09253	0.23996	-0.23996	0.46305	0.04781	0.29416	0.84816	0.49318	0.65426	0.22869	0.00157	0.00157	turquoise
CYBRD1	-0.12289	-0.11347	0.05856	0.12391	-0.20982	0.2107	0.02465	0.32768	-0.32768	0.10931	0.13947	0.44681	0.10639	0.00588	0.00567	0.74892	1.21E-05	1.21E-05	blue
TACO1	-0.00619	-0.04623	-0.11065	-0.08444	0.01509	-0.16984	0.08788	-0.10682	0.10682	0.93598	0.5482	0.14967	0.27217	0.84474	0.02637	0.25306	0.16434	0.16434	grey
CDC37	0.07811	0.04405	-0.05844	-0.06156	0.12615	0.03964	0.10364	-0.31536	0.31536	0.30986	0.56723	0.44774	0.4238	0.10016	0.60673	0.17735	2.65E-05	2.65E-05	green
FRS3	-0.02087	-0.00299	-0.09192	-0.09362	0.05971	-0.07663	0.02292	-0.06221	0.06221	0.78647	0.969	0.2318	0.22326	0.43788	0.31919	0.76608	0.41888	0.41888	red
PIGZ	-0.01225	-0.02877	-0.1413	-0.03155	0.15444	-0.06438	0.04387	-0.04013	0.04013	0.87368	0.70875	0.06527	0.68208	0.04371	0.40286	0.56881	0.60228	0.60228	grey
FAM162B	-0.02599	-0.06523	-0.07942	-0.00157	-0.04986	-0.02695	-0.05047	0.36521	-0.36521	0.73583	0.39665	0.30178	0.98378	0.51719	0.72646	0.51209	9.04E-07	9.04E-07	blue
C15orf65	0.01347	-0.07795	-0.18775	-0.1293	0.19193	-0.19782	-0.12879	0.04769	-0.04769	0.86119	0.31089	0.01393	0.09188	0.01191	0.0095	0.09319	0.53567	0.53567	grey
KATNAL1	-0.02779	-0.15859	-0.12445	-0.03044	-0.1851	-0.12854	-0.10643	0.39762	-0.39762	0.71827	0.03829	0.10485	0.6927	0.01536	0.09383	0.16592	7.24E-08	7.24E-08	turquoise
N4BP2L1	0.04353	-0.0469	-0.06591	0.02493	-0.09865	0.0401	-0.03327	0.22118	-0.22118	0.57182	0.54244	0.39169	0.74622	0.19926	0.60259	0.66579	0.00365	0.00365	blue
LAMP5	0.01722	-0.06654	-0.02944	0.02231	-0.17518	0.06231	-0.05197	0.21976	-0.21976	0.82316	0.38718	0.70229	0.77207	0.02192	0.41814	0.49962	0.00388	0.00388	black
PRSS53	-0.0368	-0.09022	0.02394	0.0941	0.12227	-0.01171	0.06534	-0.15111	0.15111	0.63278	0.24061	0.756	0.22089	0.11112	0.87917	0.39581	0.04851	0.04851	red
VPS13C	-0.07498	-0.15527	-0.05613	0.01507	-0.06762	0.02619	-0.07189	0.31087	-0.31087	0.32975	0.04257	0.46586	0.84492	0.37956	0.73388	0.35013	3.50E-05	3.50E-05	turquoise
PDE6D	-0.00017	-0.07465	-0.04433	0.04016	-0.05714	0.01379	0.00928	-0.0686	0.0686	0.99823	0.33184	0.56477	0.60198	0.4579	0.8579	0.90406	0.37266	0.37266	turquoise
KCTD14	0.04576	0.20657	0.11234	0.10781	0.04597	0.32552	0.1567	-0.38723	0.38723	0.55231	0.00671	0.14349	0.16045	0.55051	1.40E-05	0.04068	1.68E-07	1.68E-07	yellow
ATP11B	-0.05061	-0.12299	0.06792	0.0488	-0.14674	0.23945	0.13137	-0.08122	0.08122	0.51094	0.10904	0.37741	0.52621	0.05547	0.00161	0.08676	0.29092	0.29092	turquoise
EML4	-0.00369	-0.01422	0.0872	0.05605	-0.10125	0.24682	0.12739	-0.07379	0.07379	0.96185	0.8536	0.25674	0.4665	0.18761	0.00114	0.09683	0.33748	0.33748	turquoise
PITX1	0.09702	0.07426	0.04275	-0.04373	0.0242	0.09472	0.24862	-0.58846	0.58846	0.2068	0.33437	0.57881	0.57011	0.75343	0.21784	0.00104	2.59E-17	2.59E-17	yellow
DCLK1	-0.0291	-0.10414	0.04931	0.14334	-0.10134	-0.07934	-0.09915	0.58786	-0.58786	0.70559	0.17524	0.5219	0.06144	0.18722	0.30231	0.19696	2.84E-17	2.84E-17	blue
RHBDF1	0.02588	-0.02272	0.08063	0.06413	-0.03604	0.23904	0.23238	-0.38753	0.38753	0.73691	0.76803	0.29449	0.40463	0.63975	0.00164	0.00222	1.64E-07	1.64E-07	yellow
EFNB1	0.06729	0.06265	0.08519	0.07079	0.02216	0.19615	0.21439	-0.43225	0.43225	0.38183	0.41558	0.26791	0.35752	0.77362	0.01014	0.00487	3.56E-09	3.56E-09	yellow
RNF135	0.02894	0.04124	0.15505	0.14994	-0.13594	0.22435	0.26573	-0.11532	0.11532	0.70707	0.5923	0.04288	0.0503	0.07626	0.00318	0.00044	0.13309	0.13309	turquoise
ALS2CL	0.07523	-0.0025	0.07217	0.02628	0.04911	0.1433	0.23657	-0.45772	0.45772	0.32809	0.97409	0.34821	0.73295	0.52357	0.06151	0.00184	3.09E-10	3.09E-10	yellow
CTBS	-0.12396	-0.15286	0.0153	-0.04412	-0.06782	0.00466	-0.08837	0.23036	-0.23036	0.10623	0.04594	0.84257	0.56662	0.37812	0.95176	0.25038	0.00244	0.00244	turquoise
TMSB15A	-0.14239	-0.11366	-0.06349	-0.00586	-0.10692	-0.01799	0.0175	0.12424	-0.12424	0.06319	0.13881	0.40937	0.93941	0.16395	0.81529	0.82029	0.10545	0.10545	grey
INPP5B	-0.05758	-0.1402	-0.02176	0.01092	-0.09098	0.04189	-0.11758	0.39749	-0.39749	0.45444	0.06741	0.77761	0.88725	0.23664	0.5864	0.12561	7.32E-08	7.32E-08	turquoise
RIMBP2	-0.0269	-0.12031	-0.21165	-0.09638	0.13042	-0.33636	-0.23395	0.43205	-0.43205	0.72693	0.11702	0.00545	0.20985	0.08908	6.86E-06	0.00207	3.62E-09	3.62E-09	brown
KCTD15	-0.06336	-0.08352	-0.08204	0.0059	-0.05321	-0.02869	-0.02557	0.23382	-0.23382	0.41031	0.27747	0.28608	0.93897	0.48943	0.70951	0.7399	0.00208	0.00208	blue
ABITRAM	0.0923	-0.08584	0.0087	-0.00847	-0.08465	0.12324	0.07437	-0.18754	0.18754	0.22989	0.2643	0.91009	0.9124	0.27098	0.1083	0.33371	0.01404	0.01404	turquoise
KLC1	0.09861	-0.09497	-0.08838	-0.0172	0.19149	-0.13214	-0.02592	-0.15644	0.15644	0.19942	0.2166	0.25034	0.82331	0.01211	0.08492	0.73647	0.04102	0.04102	red
ADH4	-0.00997	-0.02261	0.03411	-0.07577	0.13597	0.08044	-0.01653	0.0837	-0.0837	0.89704	0.76913	0.6578	0.32464	0.07619	0.29565	0.83009	0.27644	0.27644	grey
IFIT5	-0.02039	-0.08954	-0.07442	0.04818	-0.14017	-0.0703	-0.0074	0.20632	-0.20632	0.79128	0.24418	0.33333	0.53145	0.06746	0.36087	0.92344	0.00678	0.00678	tan
EXD2	-0.04285	-0.20276	-0.05565	0.08789	0.00453	-0.04655	-0.12101	0.19918	-0.19918	0.57784	0.00782	0.46968	0.25298	0.95308	0.54545	0.11488	0.00901	0.00901	turquoise
COASY	0.05632	-0.02803	-0.17235	-0.027	0.14397	-0.16607	0.08505	-0.36825	0.36825	0.46435	0.71591	0.02419	0.72593	0.0603	0.02994	0.26872	7.21E-07	7.21E-07	grey
RPS6KA2	-0.00884	-0.13731	0.01568	0.0842	-0.22412	0.05235	0.00589	0.21857	-0.21857	0.90859	0.07332	0.83869	0.27353	0.00321	0.49649	0.93904	0.00408	0.00408	turquoise
CYP7B1	-0.08059	-0.07386	-0.02071	0.03824	-0.15989	-0.05614	-0.06419	0.51484	-0.51484	0.29469	0.337	0.78801	0.61954	0.03671	0.46581	0.4042	5.85E-13	5.85E-13	blue
IFT52	0.03335	-0.09781	-0.06027	0.02579	-0.1154	-0.0118	0.00474	0.15437	-0.15437	0.66497	0.20311	0.43361	0.73778	0.13283	0.87829	0.95097	0.0438	0.0438	turquoise
NRBP2	-0.0332	-0.08027	0.01757	-0.00399	0.00373	0.01977	0.07624	-0.22808	0.22808	0.66641	0.29668	0.81955	0.95866	0.96137	0.79745	0.32164	0.0027	0.0027	grey
NPIPB15	0.05069	-0.0267	0.0291	0.12011	0.20455	0.06603	0.12349	-0.17689	0.17689	0.51024	0.7289	0.70557	0.11763	0.00728	0.39086	0.10759	0.02064	0.02064	grey
SMARCA5	-0.11906	-0.20142	-0.06874	-0.05454	-0.06622	-0.01217	-0.07312	0.22952	-0.22952	0.12091	0.00825	0.3717	0.47859	0.38951	0.87451	0.34191	0.00253	0.00253	turquoise
FAM24B	0.11483	0.04437	0.09852	-0.06493	0.03077	-0.00702	0.14959	-0.36456	0.36456	0.13478	0.56446	0.19986	0.3988	0.68951	0.9274	0.05085	9.49E-07	9.49E-07	grey
FBXO33	-0.08556	-0.17182	-0.05778	0.02473	-0.0202	-0.07572	-0.09479	0.30913	-0.30913	0.26584	0.02463	0.45289	0.74816	0.79317	0.32498	0.2175	3.89E-05	3.89E-05	turquoise
DNALI1	0.04881	-0.15286	-0.2127	-0.0856	0.22422	-0.26193	-0.17661	0.10735	-0.10735	0.52608	0.04594	0.00522	0.26562	0.0032	0.00054	0.02085	0.16225	0.16225	grey
IPMK	-0.08976	-0.20338	0.03729	0.10062	-0.0674	-0.02542	-0.00843	0.17549	-0.17549	0.24302	0.00763	0.6282	0.19036	0.38111	0.74134	0.91285	0.02168	0.02168	turquoise
RRN3	-0.048	-0.09461	-0.0417	0.02325	-0.09938	0.03726	0.00258	0.0202	-0.0202	0.53297	0.21837	0.58815	0.76278	0.19594	0.62855	0.97328	0.79317	0.79317	turquoise
ANK1	-0.09969	0.00353	-0.10978	0.00849	0.06341	-0.12037	0.04951	-0.16835	0.16835	0.19451	0.96346	0.15289	0.91221	0.40998	0.11684	0.52021	0.02773	0.02773	grey
UGGT1	0.0297	-0.04989	-0.03885	0.00175	-0.22434	0.06476	0.15318	0.09897	-0.09897	0.69977	0.51695	0.61393	0.9819	0.00318	0.40006	0.04548	0.19778	0.19778	turquoise
ASB1	0.01751	-0.14321	-0.19486	-0.12843	0.00357	-0.22005	-0.0838	0.23847	-0.23847	0.82021	0.06167	0.01065	0.09411	0.96306	0.00383	0.27586	0.00168	0.00168	turquoise
GTF2E1	-0.03494	-0.06374	-0.02563	0.02846	-0.15155	0.09316	0.15658	0.06948	-0.06948	0.65005	0.40755	0.73935	0.71175	0.04785	0.22553	0.04084	0.36654	0.36654	turquoise
POLR2H	0.04269	-0.00636	-0.03216	-0.04161	0.0881	0.05492	0.25268	-0.54331	0.54331	0.57926	0.93421	0.67631	0.58893	0.25185	0.47558	0.00086	1.62E-14	1.62E-14	green
TBC1D9	-0.12363	-0.21446	-0.09966	-0.00931	-0.08609	-0.11709	-0.20641	0.50821	-0.50821	0.10718	0.00485	0.19468	0.90385	0.26287	0.12723	0.00676	1.29E-12	1.29E-12	turquoise
SSX2IP	-0.04957	-0.19747	-0.20022	-0.17047	0.12534	-0.22208	-0.1257	0.33957	-0.33957	0.51969	0.00963	0.00865	0.0258	0.10237	0.00351	0.10139	5.53E-06	5.53E-06	turquoise
CMTM4	-0.02355	-0.13956	-0.10006	-0.09186	0.01707	-0.08768	-0.02667	0.03557	-0.03557	0.75983	0.06868	0.19287	0.23211	0.82466	0.25412	0.72912	0.64417	0.64417	turquoise

TSPAN5	-0.10677	-0.11926	0.09142	-0.02352	-0.13643	0.13562	0.15059	-0.16382	0.16382	0.16457	0.12026	0.23437	0.76009	0.0752	0.07696	0.0493	0.03227	0.03227	turquoise
HSPA5	0.0755	-0.13588	-0.05673	0.0128	-0.08467	-0.00896	0.046	-0.05601	0.05601	0.32635	0.07639	0.4611	0.868	0.27089	0.9074	0.55019	0.46684	0.46684	turquoise
ASAH2B	0.02902	-0.08091	-0.04185	-0.07354	0.06754	-0.12442	-0.0377	-0.12488	0.12488	0.70633	0.29278	0.58678	0.33912	0.38008	0.10493	0.62441	0.10366	0.10366	grey
IFT140	0.08697	-0.11971	-0.02484	-0.04046	0.10585	0.02936	-0.00311	-0.11308	0.11308	0.25803	0.11886	0.74707	0.59927	0.16824	0.70301	0.96778	0.14086	0.14086	grey
SCGB3A1	0.01573	0.04521	0.01538	0.00743	0.0606	0.07282	0.11871	0.01645	-0.01645	0.83817	0.5571	0.84173	0.92317	0.43107	0.34385	0.12199	0.83085	0.83085	grey
MFSD4A	-0.03981	-0.02819	-0.03629	-0.00801	0.14049	0.08639	-0.01273	-0.06634	0.06634	0.60518	0.71434	0.63743	0.91721	0.06684	0.26122	0.86876	0.38863	0.38863	grey
CDC25B	0.11156	0.0574	-0.05749	-0.06427	-0.02722	-0.11464	0.16004	-0.26163	0.26163	0.1463	0.45584	0.45516	0.40363	0.72382	0.13543	0.03653	0.00055	0.00055	grey
RER1	-0.01883	-0.03512	0.06772	0.0472	-0.04711	0.2139	0.15153	-0.28256	0.28256	0.80689	0.6484	0.37884	0.53989	0.54061	0.00497	0.04789	0.00018	0.00018	turquoise
MPZ	-0.16182	-0.10923	-0.20038	-0.10489	0.0439	-0.21293	-0.20067	0.38276	-0.38276	0.03447	0.15499	0.00859	0.17215	0.56863	0.00517	0.0085	2.38E-07	2.38E-07	grey
CNPY4	-0.05023	-0.14394	-0.094	-0.02527	-0.08116	-0.11791	-0.11852	0.36534	-0.36534	0.51409	0.06035	0.22138	0.74288	0.29129	0.12456	0.12259	8.95E-07	8.95E-07	blue
MON1A	0.0479	-0.0117	-0.11019	-0.1778	0.11988	-0.06398	-0.02962	0.04745	-0.04745	0.53381	0.87933	0.15136	0.01999	0.11835	0.40579	0.70052	0.53772	0.53772	grey
CIAO3	0.0541	0.0242	-0.06208	-0.04643	0.18961	-0.06093	0.06	-0.30286	0.30286	0.48217	0.7534	0.41986	0.54645	0.013	0.42857	0.43565	5.67E-05	5.67E-05	green
TP63	0.08684	0.01645	0.09933	0.06104	-0.1508	0.0611	0.2048	-0.12133	0.12133	0.25872	0.83094	0.19614	0.4277	0.04898	0.42729	0.00721	0.11394	0.11394	grey
TMEM41A	0.06483	-0.12962	0.05154	0.0495	-0.12869	0.17299	0.29535	-0.33081	0.33081	0.39951	0.09109	0.5032	0.52027	0.09345	0.02366	8.79E-05	9.91E-06	9.91E-06	turquoise
MLPH	0.07829	0.01918	0.003	-0.03943	0.13663	0.1948	0.14212	-0.51377	0.51377	0.30878	0.80336	0.96897	0.60866	0.07476	0.01067	0.0637	6.65E-13	6.65E-13	yellow
RBM24	-0.08181	-0.05815	-0.09914	-0.02269	0.07779	-0.20314	-0.13366	0.32239	-0.32239	0.28743	0.44993	0.19701	0.76833	0.3119	0.0077	0.08136	1.71E-05	1.71E-05	grey
GNAO1	0.01855	-0.12713	-0.22629	-0.10814	0.06759	-0.26225	-0.23045	0.54521	-0.54521	0.80975	0.09753	0.00292	0.15918	0.37974	0.00053	0.00243	1.26E-14	1.26E-14	brown
ZFYVE28	0.02525	-0.07874	-0.10124	-0.00441	0.2059	-0.11781	-0.15482	0.00834	-0.00834	0.74302	0.30597	0.18764	0.95434	0.0069	0.12489	0.04319	0.91379	0.91379	grey
ZNF785	0.10491	-0.161	-0.20222	-0.13318	0.11346	-0.12718	0.06561	-0.06115	0.06115	0.17206	0.0354	0.00799	0.08247	0.13951	0.09738	0.39391	0.42691	0.42691	red
CIRBP	-0.00663	-0.06078	-0.25135	-0.15877	0.2208	-0.31018	-0.27545	0.26073	-0.26073	0.93142	0.42967	0.00091	0.03807	0.00371	3.65E-05	0.00027	0.00057	0.00057	brown
CEP170B	0.05383	-0.01872	-0.01683	0.04397	0.19826	0.02066	0.07587	-0.40406	0.40406	0.48436	0.80804	0.82706	0.56799	0.00934	0.7885	0.324	4.24E-08	4.24E-08	yellow
USP2	-0.05483	-0.14067	-0.18362	-0.03593	0.0281	-0.19635	-0.12523	0.41003	-0.41003	0.47626	0.06648	0.01622	0.64085	0.71525	0.01006	0.10267	2.56E-08	2.56E-08	brown
ZNF69	0.00674	0.01696	-0.02726	0.021	-0.032	0.16788	0.11115	-0.19844	0.19844	0.93026	0.82569	0.72341	0.78517	0.67782	0.02818	0.1478	0.00927	0.00927	grey
ERV3-1	0.02729	-0.14945	0.04793	0.04685	-0.0202	0.19631	0.13393	-0.12336	0.12336	0.72307	0.05107	0.53356	0.54288	0.79316	0.01007	0.08074	0.10796	0.10796	grey
TEAD4	0.07321	0.11522	0.013	0.10844	-0.15062	0.19942	0.23139	-0.22797	0.22797	0.3413	0.13345	0.86604	0.15801	0.04926	0.00892	0.00233	0.00271	0.00271	grey
ADGRL1	0.03132	-0.10773	-0.25563	-0.09012	0.07818	-0.31322	-0.17894	0.34801	-0.34801	0.68427	0.16077	0.00074	0.24112	0.30941	3.03E-05	0.0192	3.10E-06	3.10E-06	brown
CDR2L	0.00444	-0.0278	0.02521	0.09985	-0.01012	0.19328	0.21291	-0.28236	0.28236	0.95401	0.71814	0.74345	0.19383	0.89549	0.01131	0.00517	0.00018	0.00018	yellow
SMYD4	-0.06871	-0.12659	-0.06212	-0.03128	-0.05537	-0.07182	-0.02841	0.41061	-0.41061	0.37185	0.09897	0.41958	0.68469	0.47194	0.35056	0.71221	2.43E-08	2.43E-08	turquoise
EIF3D	-0.00423	0.10579	-0.02616	-0.0086	-0.06348	0.0587	0.14125	-0.18015	0.18015	0.9562	0.16847	0.7341	0.91111	0.40945	0.44565	0.06536	0.01838	0.01838	turquoise
TRRAP	-0.06693	-0.21648	-0.04517	0.01713	-0.01674	-0.03443	-0.0258	0.1349	-0.1349	0.38445	0.00446	0.55745	0.82399	0.82796	0.65485	0.73761	0.07855	0.07855	turquoise
ZNF684	-0.09158	-0.06971	-0.18875	-0.02016	0.01288	-0.07583	-0.14193	0.38752	-0.38752	0.23352	0.36495	0.01342	0.79349	0.86724	0.32428	0.06407	1.64E-07	1.64E-07	turquoise
TFCP2L1	0.02688	0.04023	0.02482	0.0995	-0.0463	0.16764	0.17474	-0.30265	0.30265	0.72712	0.60135	0.74731	0.19537	0.54763	0.02841	0.02226	5.74E-05	5.74E-05	yellow
BORCS5	-0.03979	-0.06992	-0.05286	-0.02906	-0.08477	0.00374	0.05874	0.0897	-0.0897	0.60536	0.36346	0.49234	0.70596	0.27032	0.96129	0.44538	0.24334	0.24334	turquoise
HIGD1A	0.0126	-0.10799	0.03621	-0.09786	0.04269	0.01561	-0.04652	-0.16984	0.16984	0.87006	0.15977	0.63823	0.2029	0.57934	0.83937	0.54573	0.02637	0.02637	turquoise
FOXRED1	-0.07068	0.06841	-0.16213	-0.10847	0.0717	-0.16888	0.11459	-0.17749	0.17749	0.35828	0.37398	0.03412	0.15791	0.35135	0.02724	0.13558	0.02021	0.02021	grey
TMCO6	0.05146	-0.07816	-0.0544	-0.09573	0.13372	-0.04682	0.05058	-0.25606	0.25606	0.50383	0.30954	0.47976	0.21295	0.08123	0.54311	0.51121	0.00072	0.00072	red
SKP1	0.0289	-0.18041	-0.12943	-0.11912	-0.0089	-0.09082	-0.0491	0.05545	-0.05545	0.70752	0.01821	0.09157	0.12072	0.90799	0.23746	0.52366	0.47129	0.47129	turquoise
CEP70	0.14309	-0.10371	-0.08719	0.03688	0.00727	0.09617	0.17587	-0.19686	0.19686	0.06189	0.17707	0.25683	0.632	0.92476	0.21082	0.0214	0.00986	0.00986	grey
CXCL9	-0.10405	0.12951	-0.02983	0.06674	-0.17406	0.10287	0.18211	0.17323	-0.17323	0.17565	0.09135	0.69856	0.38578	0.0228	0.18059	0.01713	0.02346	0.02346	blue
DPY19L1	-0.10256	-0.11104	0.07538	0.11544	-0.09847	0.10632	0.11089	-0.10071	0.10071	0.18192	0.14822	0.32716	0.13271	0.20009	0.16634	0.14878	0.18997	0.18997	turquoise
HLA-F	-0.05975	0.12988	0.03501	0.03378	-0.00986	0.11385	0.122	-0.20186	0.20186	0.43759	0.09044	0.64935	0.66095	0.89812	0.13815	0.11192	0.00811	0.00811	tan
NEK8	0.0282	0.03271	-0.07835	-0.0607	0.19234	-0.10457	-0.02694	-0.12172	0.12172	0.71427	0.67108	0.30837	0.4303	0.01172	0.17347	0.72655	0.11274	0.11274	red
LGALS2	-0.06171	0.04908	-0.00625	0.0788	0.08405	0.13098	-0.01856	0.15335	-0.15335	0.42269	0.52383	0.93534	0.30563	0.27443	0.08771	0.80959	0.04524	0.04524	magenta
CWC25	-0.08715	-0.18654	-0.06826	0.0087	0.08367	-0.01086	-0.06117	0.08439	-0.08439	0.25703	0.01457	0.37506	0.91003	0.27658	0.88788	0.42674	0.27245	0.27245	turquoise
MYZAP	0.01608	-0.01569	0.18662	0.15941	-0.01307	0.29436	0.06684	-0.27124	0.27124	0.83461	0.83863	0.01453	0.03729	0.86532	9.31E-05	0.38504	0.00033	0.00033	grey
CARS	0.11824	-0.05593	0.05435	-0.03175	-0.0392	0.01053	0.12405	-0.29166	0.29166	0.1235	0.4675	0.48017	0.68013	0.61071	0.89128	0.10599	0.00011	0.00011	turquoise
ZNF148	-0.03349	-0.10508	-0.07149	0.0183	-0.13097	0.07722	0.04325	0.18472	-0.18472	0.66369	0.17138	0.35283	0.81225	0.08773	0.31542	0.57429	0.01558	0.01558	turquoise
TRIM7	0.02753	0.05889	0.06528	0.01555	0.03373	0.12902	0.12998	-0.50414	0.50414	0.72073	0.44418	0.39627	0.83999	0.66141	0.09261	0.09019	2.07E-12	2.07E-12	grey
PLK2	-0.03022	-0.21183	-0.08469	-0.02523	0.05859	-0.2274	-0.08717	0.18463	-0.18463	0.69474	0.00541	0.27073	0.74321	0.44655	0.00278	0.25691	0.01563	0.01563	grey
GON4L	-0.00676	-0.13087	0.00772	-0.04206	-0.05986	0.14391	0.0846	0.06317	-0.06317	0.93003	0.08799	0.92018	0.58493	0.43674	0.0604	0.27125	0.41173	0.41173	turquoise
TPPP3	-0.01005	-0.03785	-0.15106	-0.01824	0.16215	-0.11331	-0.12938	0.19604	-0.19604	0.89619	0.6231	0.04858	0.81286	0.0341	0.14006	0.09169	0.01018	0.01018	grey
LONP1	0.00852	0.0101	-0.21237	-0.07871	0.21453	-0.25882	-0.03895	-0.10389	0.10389	0.91192	0.89573	0.00529	0.30619	0.00484	0.00063	0.61304	0.17629	0.17629	green
AKR1B10	0.04852	-0.03229	0.1256	0.09946	0.14046	0.22897	0.09909	-0.21302	0.21302	0.5286	0.67504	0.10165	0.19559	0.06689	0.00259	0.19724	0.00515	0.00515	yellow
MYO5B	0.04519	-0.01714	0.02212	0.05224	0.09951	0.11251	0.07671	-0.29551	0.29551	0.55731	0.82396	0.77402	0.49739	0.19535	0.14289	0.31864	8.71E-05	8.71E-05	turquoise
SMG8	-0.04457	-0.09604	-0.07404	0.00039	-0.07277	-0.06953	-0.01164	0.30712	-0.30712	0.56271	0.0128	0.33582	0.99597	0.34419	0.3662	0.87993	4.39E-05	4.39E-05	turquoise
C1QL3	-0.00555	-0.05654	-0.09659	-0.04539	0.04816	-0.03432	0.04916	0.19919	-0.19919	0.94261	0.46262	0.20884	0.55549	0.53162	0.65588	0.52309	0.00901	0.00901	grey
CACNA2D	-0.11168	-0.10651	-0.07628	-0.00088	-0.06426	-0.08883	-0.13904	0.5668	-0.5668	0.14587	0.16557	0.32141	0.99085	0.40369	0.24792	0.06972	6.42E-16	6.42E-16	turquoise
PTPRZ1	0.05516	0.0145	-0.04074	-0.07598	-0.03016	-0.26668	-0.06036	0.23174	-0.23174	0.47362	0.85067	0.59678	0.32333	0.69534	0.00042	0.43289	0.00229	0.00229	grey
PET117	0.05228	0.1338	-0.13151	-0.16414	0.18319	-0.13886	0.10731	-0.23	0.23	0.49706	0.08105	0.08644	0.03194	0.01647	0.07009	0.1624	0.00248	0.00248	grey
SLC27A3	-0.00301	-0.11514	-0.08595	-0.05279	0.27708	-0.10164	-0.1333	0.01519	-0.01519	0.9688	0.1337	0.26364	0.49291	0.00024	0.18592	0.0822	0.84369	0.84369	grey
TEF	-0.06538	0.00303	-0.14833	-0.02736	-0.12949	-0.16808	-0.08044	0.30921	-0.30921	0.39558	0.96858	0.05284	0.72244	0.09141	0.02799	0.29563	3.87E-05	3.87E-05	grey
DESI1	-0.04448	-0.00021	-0.06565	0.03164	-0.02314	-0.05436	0.10194	-0.14012	0.14012	0.5635	0.99781	0.39357	0.68121	0.76385	0.48011	0.18462	0.06757	0.06757	turquoise
RBSN	-0.04795	-0.16447	-0.04071	-0.01223	-0.19596	0.08623	0.0807	0.31107	-0.31107	0.5334	0.03159	0.59707	0.87391	0.01021	0.26212	0.29404	3.46E-05	3.46E-05	turquoise
ITGA10	-0.01723	-0.115	0.04465	-0.04542	-0.03722	0.08067	0.02938	0.02597	-0.02597	0.82303	0.13419	0.56201	0.55523	0.62883	0.29421	0.70284	0.736	0.736	grey
PXYLP1	-0.03811	-0.08168	0.00509	0.1265	-0.2432	0.13167	0.10229	0.15359	-0.15359	0.62068	0.28822	0.94737	0.09922	0.00135	0.08603	0.18309	0.04489	0.04489	turquoise
LEAP2	0.02982	-0.18048	-0.19564	-0.1058	0.25106	-0.16932	-0.04827	-0.13264	0.13264	0.69863	0.01817	0.01034	0.16843	0.00093	0.02683	0.53066	0.08373	0.08373	grey
ODF2	0.01097	-0.14349	-0.10777	-0.01953	-0.07135	-0.0687	0.02174	0.0745	-0.0745	0.88676	0.06117	0.16061	0.7998	0.35377	0.37198	0.77774	0.33283	0.33283	turquoise
PXDN	-0.06023	-0.09323	0.04199	0.07735	-0.15336	0.08335	0.01891	0.25093	-0.25093	0.43389	0.2252	0.58553	0.3146	0.04522	0.27846	0.80605	0.00093	0.00093	black
PCDHGA1	0.01475	-0.15596	0.07947	0.11165	-0.10993	0.14745	0.11342	0.14399	-0.14399	0.84815	0.04166	0.30151	0.146	0.15234	0.05429	0.13965	0.06025	0.06025	grey
NFKBIE	0.01088	-0.01569	0.07379	0.08044	-0.00184	0.24753	0.15061	-0.25514	0.25514	0.88769	0.83863	0.33751	0.29563	0.98095	0.0011	0.04927	0.00076	0.00076	yellow
SENP6	-0.13726	-0.11911	-0.02663	0.01574	-0.04866	-0.00596	-0.06867	0.37141	-0.37141	0.07342	0.12073	0.72958	0.83811	0.52739	0.93834	0.37217	5.69E-07	5.69E-07	turquoise
MACROD1	0.09868	0.06662	0.10038	0.01332	0.06912	0.06299	0.18492	-0.4578	0.4578	0.1991	0.38666	0.19146	0.86268	0.36904	0.41308	0.01546	3.07E-10	3.07E-10	green
TSPAN8	0.02789	0.10146	0.11491	0.0781	0.10401	0.28287	-0.00797	-0.22313	0.22313	0.7173	0.18669	0.13451	0.30994	0.1758	0.00018	0.91764	0.00335	0.00335	yellow
USP30	-0.00782	-0.14752	-0.16817	-0.03688	0.13859	-0.24369	-0.18628	0.33337	-0.33337	0.91917	0.05417	0.0279	0.63199	0.07065	0.00132	0.01471	8.37E-06	8.37E-06	turquoise
APMAP	0.04596	-0.17051	-0.08992	0.01626	-0.02716	-0.14992	-0.0302	0.16268	-0.16268	0.55061	0.02577	0.24218	0.83286	0.72432	0.05033	0.695	0.03351	0.03351	turquoise
CTNNAL1	-0.01635	-0.17396	-0.00566	-0.00589	-0.10292	0.0031	0.03484	0.02681	-0.02681	0.83192	0.02287	0.94148	0.93903	0.18041	0.96795	0.65099	0.72777	0.72777	grey
OXLD1	-0.00906	-0.02754	-0.12163	-0.10988	0.21941	-0.21258	0.05202	-0.24181	0.24181	0.90634	0.7207	0.11304	0.15252	0.00393	0.00525	0.4992	0.00144	0.00144	green
TSEN2	0.01003	0.01447	-0.01486	-0.11931	0.06796	0.05158	0.11407	-0.19048	0.19048	0.89642	0.85104	0.84706	0.12011	0.37713	0.50287	0.1374	0.01258	0.01258	grey
SCAMP3	0.09977	-0.02636	-0.09118	-0.12759	0.02819	-0.06899	0.14861	-0.24091	0.24091	0.19418	0.73216	0.2356	0.09631	0.71435	0.36994	0.05239	0.0015	0.0015	greenyellow
BEGAIN	0.08229	-0.10483	-0.13829	0.0259	0.06266	-0.05304	-0.168	0.01013	-0.01013	0.28463	0.17242	0.07126	0.73669	0.41555	0.4908	0.02806	0.89542	0.89542	grey
ZSCAN5A	0.05412	0.00371	-0.23866	-0.07054	0.10408	-0.22779	-0.10436	0.19924	-0.19924	0.48202	0.96158	0.00167	0.35922	0.17552	0.00273	0.17433	0.00899	0.00899	turquoise
ANKRD6	-0.04685	-0.11949	-0.02775	0.08401	-0.18156	0.08155	-0.06084	0.42632	-0.42632	0.54283	0.11956	0.71866	0.27465	0.01747	0.28898	0.42923	6.11E-09	6.11E-09	blue
GNAI1	0.06332	-0.19255	-0.13358	-0.04928	0.09889	-0.21846	-0.13263	0.26718	-0.26718	0.41061	0.01163	0.08155	0.52212	0.19814	0.0041	0.08375	0.00041	0.00041	turquoise
IRGQ	0.06369	-0.17117	-0.22678	-0.15356	0.0763	-0.20935	-0.05151	0.19114	-0.19114	0.40793	0.02519	0.00286	0.04494	0.32126	0.006	0.50342	0.01227	0.01227	grey
ARL2	-0.06409	0.02078	-0.14586	-0.022	0.12603	-0.10986	-0.01035	-0.01282	0.01282	0.40495	0.78732	0.05696	0.77521	0.10049	0.15259	0.8931	0.86781	0.86781	green
TTC33	-0.06005	-0.19783	-0.04116	0.0446	-0.03517	-0.00082	-0.0764	0.26801	-0.26801	0.43525	0.0095	0.59295	0.56242	0.64795	0.99146	0.32065	0.00039	0.00039	turquoise
ADAP2	-0.07867	0.03214	0.01591	0.08525	-0.19173	0.06646	0.07391	0.45657	-0.45657	0.30643	0.67644	0.83642	0.26759	0.012	0.3878	0.33668	3.47E-10	3.47E-10	blue
DSC3	0.02271	0.0682	0.08632	0.05801	-0.09773	0.0614	0.06889	-0.07176	0.07176	0.76808	0.37541	0.26162	0.45108	0.2035	0.42499	0.37061	0.351	0.351	grey
BMP8B	-0.02389	-0.06032	-0.06931	0.02176	-0.15415	-0.09259	0.00164	0.14268	-0.14268	0.75642	0.43322	0.3677	0.77759	0.04411	0.22841	0.98297	0.06266	0.06266	grey
STIM1	0.06754	-0.20037	-0.03932	-0.00362	-0.13194	-0.05419	-0.02677	0.17093	-0.17093	0.3801	0.0086	0.60967	0.96256	0.08539	0.48146	0.72822	0.0254	0.0254	turquoise
IFI16	-0.0611	-0.0081	0.06705	0.07259	-0.20074	0.19095	0.09197	0.1143	-0.1143	0.42731	0.91629	0.38359	0.34544	0.00847	0.01236	0.23154	0.13659	0.13659	black
TNS4	0.01642	0.03584	0.14066	0.01061	-0.1422	0.20422	0.21321	-0.31896	0.31896	0.83118	0.64163	0.06651	0.89048	0.06356	0.00738	0.00511	2.12E-05	2.12E-05	yellow
PPIF	0.10803	0.13473	0.16306	0.10545	-0.0529	0.03399	0.13436	-0.21636	0.21636	0.15959	0.07894	0.0331	0.16985	0.49201	0.65892	0.07976	0.00448	0.00448	grey
BRD1	0.00227	-0.05491	-0.11523	0.00981	0.1522	-0.13445	-0.14981	0.10061	-0.10061	0.97653	0.47567	0.13342	0.89863	0.04689	0.07956	0.0505	0.19043	0.19043	turquoise
EIF4A1	-0.06441	-0.06	0.04765	-0.01814	0.06458	0.05178	0.05337	0.06801	-0.06801	0.40259	0.43567	0.53595	0.8138	0.4014	0.50121	0.48813	0.37676	0.37676	red
DOCK9	-0.05026	-0.13695	-0.00877	0.07013	-0.16497	0.03989	0.0379	0.15032	-0.15032	0.51384	0.07408	0.90935	0.36207	0.03107	0.60442	0.62265	0.04971	0.04971	turquoise
ARHGAP3	-0.14172	-0.09336	0.00714	0.04563	-0.22561	0.12449	-0.00641	0.51857	-0.51857	0.06445	0.22452	0.92613	0.55348	0.00301	0.10473	0.93367	3.73E-13	3.73E-13	blue
ARHGEF2!	-0.06477	-0.10093	-0.03687	0.03799	-0.05145	-0.06166	-0.08791	0.43568	-0.43568	0.39997	0.189	0.63209	0.6218	0.50397	0.42304	0.25287	2.59E-09	2.59E-09	blue
DIS3	-0.03557	-0.06851	0.02053	-0.03962	-0.1384	0.08721	0.11052	0.0274	-0.0274	0.64422	0.3733	0.78982	0.60694	0.07103	0.25668	0.15013	0.72207	0.72207	turquoise
UAP1L1	-0.04893	-0.08139	-0.15728	0.02363	0.01045	-0.05713	0.00801	0.1724	-0.1724	0.52507	0.28993	0.03993	0.75897	0.89214	0.45796	0.91721	0.02414	0.02414	grey
NEURL1	0.08703	-0.0522	-0.25802	-0.13282	0.20309	-0.28475	-0.2264	0.31594	-0.31594	0.25769	0.49772	0.00066	0.08332	0.00772	0.00016	0.0029	2.56E-05	2.56E-05	brown
PI3	-0.08509	0.07904	0.17173	0.04926	-0.03749	0.27847	0.2893	-0.28998	0.28998	0.26852	0.30413	0.02471	0.52228	0.62637	0.00023	0.00012	0.00012	0.00012	yellow
GTF3C2	-0.04702	-0.00087	-0.02162	0.00158	-0.08515	0.10601	0.15335	-0.13474	0.13474	0.54137	0.99096	0.77898	0.98365	0.26817	0.16758	0.04523	0.0789	0.0789	turquoise
URI1	0.05138	-0.20301	-0.04089	0.04095	-0.16533	0.01752	0.06696	0.03409	-0.03409	0.50453	0.00775	0.59542	0.59491	0.03069	0.8201	0.38422	0.65804	0.65804	turquoise
UBALD1	0.06026	-0.05415	-0.12549	-0.05495	0.16426	-0.09028	-0.00661	-0.23463	0.23463	0.43363	0.48179	0.10195	0.47533	0.03181	0.24028	0.93166	0.00201	0.00201	green
USP12	-0.00198	-0.19047	-0.0208	-0.03818	0.00802	-0.06709	-0.16496	0.24954	-0.24954	0.97953	0.01258	0.78714	0.62004	0.91704	0.38328	0.03108	0.001	0.001	turquoise
OR2I1P	-0.0683	0.04246	0.00853	0.09796	-0.05276	0.15585	0.07123	-0.02282	0.02282	0.37473	0.58138	0.91181	0.20242	0.49314	0.04179	0.35456	0.76701	0.76701	grey
OS9	0.13104	-0.18394	-0.03725	0.06713	0.04953	-0.17744	-0.11546	0.15722	-0.15722	0.08757	0.01603	0.62863	0.38302	0.52002	0.02024	0.13263	0.04002	0.04002	turquoise
RPUSD3	0.01196	0.00069	-0.06076	-0.08802	0.01173	0.00022	0.20055	-0.26506	0.26506	0.8766	0.9929	0.42982	0.25232	0.87895	0.99773	0.00854	0.00046	0.00046	grey
ADRB1	0.05805	-0.18395	-0.04241	-0.07277	0.13916	-0.12479	-0.13655	0.03219	-0.03219	0.45071	0.01302	0.58177	0.34423	0.06949	0.1039	0.07493	0.67593	0.67593	grey
MSX2	0.06873	0.06206	0.0854	0.07944	-0.11122	0.18923	0.25344	-0.25189	0.25189	0.37175	0.42005	0.26673	0.30169	0.14756	0.01318	0.00082	0.00089	0.00089	yellow
RNF214	-0.02752	-0.06294	-0.0139	0.00408	-0.09826	0.08599	0.10224	0.05073	-0.05073	0.72083	0.41345	0.85682	0.95781	0.20104	0.26344	0.1833	0.50996	0.50996	turquoise
HDAC2	-0.06891	-0.10782	-0.00102	-0.01115	-0.08047	-0.02066	0.0711	0.06836	-0.06836	0.37046	0.16041	0.98939	0.88496	0.29543	0.7885	0.35543	0.37432	0.37432	turquoise
AAMDC	0.01705	-0.06032	0.00268	0.09505	-0.03835	0.07571	0.12828	-0.18091	0.18091	0.82482	0.43317	0.97229	0.2162	0.6185	0.325	0.09449	0.01789	0.01789	grey
ALKBH8	-0.05834	-0.05933	-0.0426	0.03456	-0.12362	0.04327	0.01145	0.19974	-0.19974	0.44845	0.44082	0.58011	0.6536	0.1072	0.57419	0.88182	0.00881	0.00881	turquoise
ZNF213	0.07974	-0.02828	-0.0188	0.01448	-0.00325	0.02864	0.09123	-0.18883	0.18883	0.29984	0.71351	0.80716	0.8509	0.96639	0.71003	0.23533	0.01338	0.01338	green
SRRD	-0.06112	-0.06254	-0.04841	-0.0685	-0.1102	-0.03576	0.03053	0.09192	-0.09192	0.42715	0.41647	0.52948	0.37336	0.15132	0.64237	0.69179	0.2318	0.2318	turquoise
CLSTN1	0.05428	-0.13871	-0.00509	0.02652	-0.09125	0.05546	0.04708	-0.04833	0.04833	0.48076	0.07039	0.94728	0.73063	0.23523	0.47121	0.5409	0.53015	0.53015	turquoise
EHBP1L1	-0.00576	-0.05618	0.05554	-0.0119	-0.00321	0.1218	0.17209	-0.19303	0.19303	0.94041	0.46549	0.47058	0.87725	0.96676	0.11252	0.0244	0.01142	0.01142	grey
PCDH12	-0.18574	-0.12867	-0.0687	0.04584	-0.09972	-0.01086	-0.07428	0.50063	-0.50063	0.015	0.09351	0.37198	0.55164	0.1944	0.88788	0.33425	3.11E-12	3.11E-12	blue
PLSCR4	-0.04926	-0.15781	-0.03502	0.05726	-0.16544	0.11186	-0.11869	0.47597	-0.47597	0.52229	0.03926	0.64927	0.45692	0.03059	0.14521	0.12205	4.72E-11	4.72E-11	turquoise
ERBB3	0.1438	0.07629	0.118	0.08399	0.0759	0.19838	0.11024	-0.4475	0.4475	0.06059	0.32134	0.12426	0.27477	0.32379	0.00929	0.15119	8.44E-10	8.44E-10	yellow
RGL1	-0.13426	-0.09486	-0.10234	0.01787	-0.17156	-0.01941	-0.07599	0.62269	-0.62269	0.07999	0.21715	0.18289	0.81655	0.02486	0.80108	0.32326	9.67E-20	9.67E-20	blue
RARB	-0.08964	-0.16722	0.01344	0.08658	-0.19719	0.10666	0.13149	0.18334	-0.18334	0.24362	0.02881	0.86147	0.26017	0.00974	0.16499	0.08647	0.01638	0.01638	turquoise
ZNF583	-0.09955	-0.12953	-0.15836	-0.01027	-0.03152	-0.09809	-0.17424	0.55393	-0.55393	0.19516	0.09131	0.03858	0.89392	0.68232	0.20182	0.02265	3.88E-15	3.88E-15	turquoise
BLVRB	0.08591	-0.08058	0.07477	0.13205	-0.0029	0.13435	0.22248	-0.47116	0.47116	0.26391	0.29477	0.33107	0.08514	0.97	0.07979	0.00345	7.83E-11	7.83E-11	yellow
UNC119B	-0.01348	-0.19824	-0.20182	-0.07451	0.02652	-0.2497	-0.13997	0.41985	-0.41985	0.86108	0.00935	0.00812	0.33278	0.7306	0.00099	0.06787	1.09E-08	1.09E-08	brown
SATB1	-0.00666	-0.08396	-0.07049	0.04158	-0.12993	1.88E-05	-0.06423	0.46453	-0.46453	0.93106	0.27602	0.35959	0.58921	0.0903	0.99981	0.40394	1.55E-10	1.55E-10	turquoise
FAM81A	0.03275	0.07834	-0.05751	-0.01147	-0.04723	0.04607	0.18732	-0.2306	0.2306	0.67068	0.30844	0.45495	0.88166	0.53964	0.5496	0.01415	0.00241	0.00241	grey

SIK1B	-0.08109	0.00967	0.06789	0.00795	-0.08327	0.03616	0.03941	0.00847	-0.00847	0.29174	0.90009	0.37759	0.91781	0.27891	0.63867	0.60883	0.91246	0.91246	grey
IRF8	-0.05489	0.01408	0.03939	0.01583	-0.11941	0.23228	0.06379	0.19955	-0.19955	0.47581	0.855	0.60897	0.83716	0.1198	0.00223	0.40719	0.00888	0.00888	blue
PRKDC	-0.05426	-0.03022	-0.00369	0.00169	-0.21335	0.01845	0.14935	0.12179	-0.12179	0.48092	0.69482	0.96181	0.98246	0.00508	0.81066	0.05122	0.11254	0.11254	turquoise
TGM1	0.00304	0.04836	-0.03114	0.14084	0.04772	-0.06271	0.04691	-0.0184	0.0184	0.96857	0.52996	0.686	0.06616	0.53537	0.41517	0.54233	0.81123	0.81123	grey
KDM3A	0.01784	-0.19011	0.01782	0.06269	-0.01593	0.0995	0.06921	-0.2288	0.2288	0.81687	0.01275	0.81702	0.41534	0.83618	0.19537	0.3684	0.00261	0.00261	turquoise
ZNF585A	0.00451	-0.19231	-0.08906	0.00823	-0.02282	-0.04026	-0.05099	0.25455	-0.25455	0.95327	0.01174	0.24669	0.91488	0.76707	0.60111	0.50776	0.00078	0.00078	turquoise
PEMT	-0.02556	-0.09951	-0.20303	-0.15718	0.21392	-0.33414	-0.17874	0.17925	-0.17925	0.74001	0.19534	0.00774	0.04006	0.00496	7.95E-06	0.01933	0.01898	0.01898	brown
ANGEL1	-0.05164	-0.09974	-0.22278	-0.04957	0.03797	-0.21695	-0.1715	0.46837	-0.46837	0.50239	0.1943	0.0034	0.51966	0.62201	0.00437	0.0249	1.05E-10	1.05E-10	brown
SOX2	0.14666	0.11718	0.00566	0.00373	-0.07222	0.01647	0.00312	0.07237	-0.07237	0.05561	0.12693	0.94147	0.96143	0.34789	0.83073	0.96773	0.34685	0.34685	grey
PHC1	-0.09097	-0.2044	-0.0731	0.02091	-0.04121	-0.1103	-0.07416	0.32694	-0.32694	0.23666	0.00733	0.34203	0.78605	0.59252	0.15096	0.33507	1.27E-05	1.27E-05	turquoise
RSPH14	-0.02282	-0.12064	-0.28563	-0.06777	0.28575	-0.29827	-0.25843	0.06482	-0.06482	0.76698	0.11601	0.00015	0.37848	0.00015	7.42E-05	0.00064	0.3996	0.3996	grey
SUSD3	-0.10727	-0.04089	0.06217	0.07345	-0.21185	0.11919	0.03925	0.29656	-0.29656	0.16258	0.59543	0.41921	0.33969	0.00541	0.12049	0.61024	8.20E-05	8.20E-05	blue
PI4KA	-0.03527	-0.11768	-0.11464	-0.07201	0.01031	-0.11415	-0.08835	0.32206	-0.32206	0.64701	0.1253	0.13542	0.34932	0.89355	0.1371	0.2505	1.74E-05	1.74E-05	turquoise
PICALM	-0.04879	-0.12085	0.00128	0.04626	-0.18128	0.12647	0.07877	0.09961	-0.09961	0.52629	0.11538	0.98676	0.54798	0.01765	0.09929	0.3058	0.1949	0.1949	turquoise
EIF4H	0.00603	-0.15032	-0.01762	0.01027	-0.03365	0.03025	0.02138	-0.01325	0.01325	0.93761	0.04972	0.81908	0.89395	0.6622	0.69448	0.78131	0.86339	0.86339	turquoise
SCRN1	-0.05508	-0.17794	-0.09051	-0.00607	-0.1608	-0.13448	-0.07696	0.30026	-0.30026	0.47425	0.01989	0.23906	0.9372	0.03564	0.07949	0.31709	6.61E-05	6.61E-05	turquoise
SNRPE	0.06459	0.04099	-0.07177	-0.1019	-0.03693	-0.02725	0.20827	-0.33689	0.33689	0.40132	0.59447	0.35092	0.18477	0.6316	0.7235	0.00627	6.62E-06	6.62E-06	greenyellow
BEAN1	-0.01869	-0.04782	0.03718	0.01594	-0.10654	0.10566	0.17322	-0.34424	0.34424	0.80826	0.53458	0.62927	0.83608	0.16545	0.16899	0.02347	4.02E-06	4.02E-06	yellow
TBX18	-0.14999	-0.02255	0.09422	0.05547	-0.14012	0.19081	0.06772	0.09759	-0.09759	0.05022	0.76974	0.22029	0.47115	0.06756	0.01242	0.37883	0.20413	0.20413	grey
ST6GALN/	-0.03275	-0.13244	-0.08025	-0.00114	-0.10458	-0.08415	-0.11272	0.47383	-0.47383	0.67066	0.0842	0.29678	0.98819	0.17344	0.27381	0.14213	5.92E-11	5.92E-11	blue
FABP4	-0.04198	0.03332	-0.02739	0.07717	-0.03285	0.00583	-0.11658	0.45195	-0.45195	0.58567	0.66532	0.72211	0.31578	0.66968	0.93972	0.1289	5.47E-10	5.47E-10	blue
C2CD2	-0.08848	-0.1139	0.03235	0.10955	-0.05289	0.0946	0.08658	0.19669	-0.19669	0.24981	0.13797	0.67449	0.15376	0.49209	0.21844	0.26018	0.00993	0.00993	turquoise
SUMO2	0.00096	-0.15492	-0.08457	-0.02918	-0.01024	-0.05005	0.06144	-0.12769	0.12769	0.99007	0.04305	0.27144	0.70479	0.8943	0.51563	0.42469	0.09603	0.09603	turquoise
DDX3Y	0.93746	-0.06238	-0.04917	0.00454	0.04617	-0.0449	0.03181	0.02109	-0.02109	2.31E-79	0.41765	0.52305	0.95303	0.5487	0.55979	0.67959	0.78424	0.78424	grey
ARHGAP3	-0.0268	-0.05271	-0.14418	-0.07519	0.21762	-0.16355	-0.05513	0.01914	-0.01914	0.72788	0.49353	0.05991	0.32837	0.00425	0.03257	0.47389	0.80374	0.80374	red
ELN	-0.05557	-0.06164	0.0192	0.07463	-0.14397	0.22738	0.05575	0.37409	-0.37409	0.47034	0.42322	0.80312	0.33198	0.0603	0.00278	0.46892	4.65E-07	4.65E-07	blue
TRAPPC1(	-0.03592	-0.13981	0.00245	-0.00603	-0.08033	0.08637	0.0188	0.22426	-0.22426	0.64087	0.06817	0.97459	0.93758	0.29631	0.26131	0.80723	0.00319	0.00319	turquoise
TGS1	-0.05471	-0.00073	-0.01077	-0.00309	-0.13157	0.05309	0.02242	0.09973	-0.09973	0.47725	0.99243	0.88884	0.96802	0.08628	0.49042	0.771	0.19433	0.19433	turquoise
NAGK	-0.04537	-0.11419	0.00578	0.03974	-0.13528	0.00139	0.06094	0.23307	-0.23307	0.55574	0.13698	0.94017	0.60578	0.0777	0.98561	0.42849	0.00216	0.00216	blue
SERPINF1	-0.09518	-0.06315	0.06218	0.09077	-0.20265	0.18398	0.05561	0.39602	-0.39602	0.21559	0.41189	0.41912	0.23773	0.00786	0.01601	0.47005	8.26E-08	8.26E-08	blue
ATF7IP	0.03174	-0.10714	-0.04577	0.05454	-0.15744	0.02881	-0.01141	0.16848	-0.16848	0.68027	0.16308	0.55223	0.47867	0.03973	0.70834	0.88225	0.02761	0.02761	turquoise
IGIP	0.03668	-0.24147	-0.17393	-0.01194	0.05646	-0.18955	-0.15927	0.36197	-0.36197	0.63386	0.00146	0.0229	0.87678	0.46326	0.01303	0.03745	1.15E-06	1.15E-06	turquoise
CUEDC1	0.04405	-0.171	-0.11314	0.00623	0.09779	-0.14366	-0.03553	-0.09544	0.09544	0.56728	0.02534	0.14066	0.93555	0.2032	0.06085	0.64451	0.21433	0.21433	grey
AGBL3	-0.06086	-0.20386	-0.21143	-0.02148	0.15242	-0.22045	-0.18218	0.20355	-0.20355	0.42913	0.00748	0.0055	0.78035	0.04657	0.00376	0.01709	0.00758	0.00758	grey
TCEANC2	-0.12521	-0.11647	0.00942	0.0409	-0.04294	0.10189	0.04874	0.19557	-0.19557	0.10273	0.12927	0.90264	0.59528	0.57705	0.18481	0.52666	0.01036	0.01036	turquoise
CGREF1	0.18466	0.16178	0.11417	0.14803	-0.02438	0.11045	0.19166	0.08738	-0.08738	0.01561	0.03452	0.13706	0.05334	0.75161	0.15041	0.01203	0.25576	0.25576	grey
OTUB1	0.03144	-0.02246	-0.09727	-0.11552	0.02529	-0.10033	0.1049	-0.37767	0.37767	0.68307	0.77065	0.20563	0.13243	0.74264	0.19166	0.1721	3.54E-07	3.54E-07	green
C8orf37	-0.09057	-0.10242	-0.1059	-0.01632	-0.05693	-0.138	-0.15902	0.29386	-0.29386	0.23875	0.18253	0.16802	0.83223	0.45958	0.07186	0.03776	9.58E-05	9.58E-05	turquoise
HS3ST2	0.02378	0.00547	0.04033	0.09717	-0.20397	0.08335	-0.04919	0.32087	-0.32087	0.75749	0.94342	0.60045	0.2061	0.00745	0.27845	0.52287	1.88E-05	1.88E-05	blue
DTX3L	-0.02168	-0.01269	-0.07474	0.07945	-0.20426	0.14215	0.26284	-0.12375	0.12375	0.77835	0.8692	0.33131	0.30162	0.00737	0.06364	0.00051	0.10684	0.10684	tan
B3GNT3	0.08012	0.05773	0.04426	0.00892	0.04785	0.19011	0.18715	-0.53593	0.53593	0.29756	0.45326	0.56541	0.9078	0.53425	0.01275	0.01424	4.24E-14	4.24E-14	yellow
PPP1R2	-0.0541	-0.0976	-0.05267	-0.01653	-0.06801	0.06165	-0.01258	0.12812	-0.12812	0.4822	0.20412	0.49385	0.83005	0.37675	0.4231	0.87026	0.09492	0.09492	turquoise
FLT3LG	-0.004	0.0217	-0.0466	-0.0038	0.04883	-0.00493	0.02421	0.00253	-0.00253	0.95857	0.77821	0.54503	0.96065	0.52591	0.94902	0.75327	0.9738	0.9738	grey
KIAA1191	0.03005	-0.22493	-0.03373	-0.00182	-0.09558	0.05227	-0.0111	-0.01378	0.01378	0.69639	0.0031	0.66143	0.98116	0.21365	0.49718	0.88547	0.85806	0.85806	turquoise
EID1	-0.14826	-0.13288	-0.1394	-0.05567	-0.04132	-0.13264	-0.18391	0.45775	-0.45775	0.05297	0.08316	0.06901	0.46954	0.5915	0.08373	0.01605	3.08E-10	3.08E-10	turquoise
SF3B5	0.04431	0.0633	-0.07418	-0.07943	0.03969	-0.12847	0.03584	-0.08531	0.08531	0.56495	0.41077	0.33492	0.30176	0.60623	0.09402	0.64167	0.26723	0.26723	purple
CCDC153	0.0987	-0.10543	-0.00857	-0.01507	0.11038	-0.0595	0.04316	-0.29826	0.29826	0.19903	0.16993	0.91144	0.84485	0.15066	0.43953	0.57515	7.42E-05	7.42E-05	grey
FBLN5	-0.04109	-0.06073	-0.05897	0.04977	-0.17591	0.08138	-0.05468	0.53478	-0.53478	0.5936	0.43006	0.44359	0.51796	0.02137	0.29	0.47748	4.91E-14	4.91E-14	blue
UPF1	0.11712	-0.06072	-0.07981	-0.03594	0.12226	0.00381	0.01407	-0.08297	0.08297	0.12712	0.43018	0.29943	0.64068	0.11116	0.96056	0.85509	0.28067	0.28067	turquoise
NME6	0.00823	-0.01948	0.03066	-0.06118	-0.09228	0.18704	0.1692	-0.0934	0.0934	0.9149	0.80037	0.69052	0.42664	0.22996	0.0143	0.02694	0.22436	0.22436	turquoise
NUP58	-0.01134	-0.18479	-0.06881	-0.06784	-0.01962	-0.06848	-0.02539	0.11643	-0.11643	0.88294	0.01554	0.3712	0.37795	0.79898	0.37351	0.74163	0.1294	0.1294	turquoise
CNOT6	-0.03636	-0.18435	-0.01629	0.03448	-0.10562	0.03615	0.11026	-0.00374	0.00374	0.63682	0.01579	0.83256	0.65432	0.16918	0.63874	0.15109	0.96131	0.96131	turquoise
FAM83D	-0.01631	0.05799	0.07091	0.11664	-0.08746	0.13125	0.23548	-0.19973	0.19973	0.83228	0.45118	0.35673	0.12868	0.25535	0.08705	0.00193	0.00882	0.00882	pink
TTC12	-0.04523	0.01616	-0.20288	-0.21067	0.10628	-0.10181	-0.07386	-0.02869	0.02869	0.55694	0.8338	0.00778	0.00568	0.16652	0.18517	0.33703	0.70949	0.70949	grey
NAAA	-0.01834	-0.14742	-0.10227	-0.01978	0.00327	-0.17158	-0.10804	0.3164	-0.3164	0.81182	0.05434	0.1832	0.79739	0.96616	0.02484	0.15955	2.49E-05	2.49E-05	turquoise
TRMT61A	0.09213	0.07145	-0.08166	-0.02275	0.17503	-0.03803	0.07695	-0.35466	0.35466	0.23076	0.35307	0.28836	0.76774	0.02204	0.62138	0.31713	1.94E-06	1.94E-06	green
FKBP8	0.07818	-0.03177	-0.15385	-0.10834	0.1836	-0.2333	-0.08878	-0.11137	0.11137	0.30946	0.67997	0.04453	0.1584	0.01623	0.00213	0.24818	0.147	0.147	green
CAMK2D	0.02889	-0.06182	0.0336	-0.0233	0.02666	0.10192	-0.01069	0.13691	-0.13691	0.70756	0.42187	0.66268	0.76226	0.72922	0.18471	0.88962	0.07417	0.07417	turquoise
RHOF	0.01255	-0.07612	0.10683	0.06915	-0.01846	0.14599	0.20393	-0.36626	0.36626	0.87054	0.32242	0.16433	0.36884	0.81064	0.05674	0.00746	8.37E-07	8.37E-07	yellow
SLC35E1	-0.03366	-0.15704	-0.05235	0.0309	-0.08772	0.02604	0.02511	0.03808	-0.03808	0.66209	0.04025	0.49651	0.68824	0.25393	0.73527	0.7444	0.62095	0.62095	turquoise
CCDC82	-0.12221	-0.12496	-0.00854	0.04409	-0.08431	0.0118	0.00579	0.12548	-0.12548	0.1113	0.10342	0.91176	0.56694	0.2729	0.87825	0.94012	0.102	0.102	turquoise
TMEM178	0.03011	-0.08252	-0.08739	-0.02064	0.20216	-0.06291	-0.13096	-0.02963	0.02963	0.69586	0.28325	0.2557	0.78874	0.00801	0.41369	0.08777	0.7005	0.7005	grey
C19orf53	-0.02283	-0.02624	-0.12492	-0.09959	0.09472	-0.13989	-0.01988	-0.19209	0.19209	0.76692	0.73333	0.10355	0.195	0.21785	0.06803	0.79631	0.01184	0.01184	grey
ZAP70	-0.07848	0.0341	0.01651	0.04621	-0.04972	0.05845	-0.01454	0.36886	-0.36886	0.30756	0.65789	0.83032	0.54836	0.51839	0.44762	0.85027	6.90E-07	6.90E-07	blue
TMEM14A	0.04624	-0.01894	-0.06114	-0.08706	0.08831	-0.06439	-0.07266	-0.01132	0.01132	0.54811	0.80582	0.42698	0.25755	0.25072	0.40277	0.34494	0.88314	0.88314	turquoise
RBP7	-0.10725	0.04053	-0.0454	0.08557	-0.01535	-0.0408	-0.17609	0.51536	-0.51536	0.16264	0.59868	0.55546	0.26578	0.8421	0.59621	0.02123	5.50E-13	5.50E-13	blue
TBC1D22E	-0.01272	-0.16192	-0.11943	0.04047	0.01885	-0.01802	-0.03822	0.05182	-0.05182	0.86886	0.03435	0.11973	0.59923	0.80673	0.81503	0.61968	0.50089	0.50089	turquoise
CYC1	0.10714	0.13708	-0.10395	-0.10335	0.02631	-0.13528	0.14047	-0.33934	0.33934	0.1631	0.0738	0.17606	0.17855	0.73264	0.07771	0.06687	5.61E-06	5.61E-06	green
BRD7	-0.00292	-0.10935	-0.04188	-0.05663	-0.02842	-0.00996	0.04535	-0.03013	0.03013	0.96975	0.15452	0.58655	0.46188	0.71211	0.89712	0.55584	0.69565	0.69565	turquoise
SEPHS1	-0.03094	-0.05273	-0.14212	-0.03247	-0.00124	-0.14914	-0.08932	0.19163	-0.19163	0.68789	0.4934	0.0637	0.67328	0.98716	0.05156	0.24534	0.01204	0.01204	turquoise
NMD3	0.01129	-0.08531	0.03895	0.02777	-0.16297	0.18743	0.18395	-0.08253	0.08253	0.88352	0.26724	0.61296	0.71846	0.0332	0.01409	0.01602	0.28321	0.28321	turquoise
TMTC3	-0.0685	-0.17071	-0.0446	0.02532	-0.14203	-0.04572	-0.02429	0.34665	-0.34665	0.37337	0.02559	0.56245	0.74233	0.06387	0.55263	0.75254	3.41E-06	3.41E-06	blue
NTSR1	0.10176	0.08307	0.05432	-0.01655	-0.04466	0.07264	0.10823	-0.33248	0.33248	0.1854	0.28006	0.48042	0.82988	0.56188	0.3451	0.15882	8.88E-06	8.88E-06	grey
RNF6	-0.01656	-0.13673	-0.09319	-0.02482	-0.01775	-0.08415	-0.06995	0.22033	-0.22033	0.82981	0.07454	0.22539	0.74726	0.81772	0.27383	0.36326	0.00378	0.00378	turquoise
TMEM64	-0.08051	-0.04543	-0.00162	0.05511	-0.11679	-0.07626	-0.01726	0.39217	-0.39217	0.29517	0.55517	0.98323	0.47403	0.12819	0.32153	0.82273	1.13E-07	1.13E-07	turquoise
AFTPH	0.01275	-0.11427	-0.09176	-0.01269	0.06471	0.03728	-0.03384	-0.04845	0.04845	0.8685	0.13669	0.23261	0.86917	0.40043	0.62832	0.66039	0.52918	0.52918	turquoise
KCNH6	0.02882	-0.13781	-0.25737	-0.17716	0.16356	-0.36201	-0.25062	0.38662	-0.38662	0.70826	0.07226	0.00068	0.02045	0.03255	1.14E-06	0.00095	1.76E-07	1.76E-07	brown
RPL37	0.11254	0.1038	-0.00795	-0.05504	-0.08039	0.04771	0.20488	-0.28071	0.28071	0.14278	0.1767	0.91785	0.47461	0.29595	0.5355	0.00719	0.0002	0.0002	purple
HDAC7	0.01486	-0.12914	0.01595	0.07889	-0.06392	0.12489	0.11955	-0.12842	0.12842	0.84708	0.09231	0.83599	0.30506	0.40623	0.10361	0.11935	0.09414	0.09414	black
ENO3	-0.07912	-0.01513	-0.03928	-0.04132	0.16921	-0.04781	-0.00535	-0.05374	0.05374	0.30364	0.84433	0.61002	0.59153	0.02694	0.53465	0.94467	0.4851	0.4851	red
GRHL3	0.0619	0.06322	0.03542	0.00547	0.06357	0.12041	0.15577	-0.16634	0.16634	0.42121	0.4114	0.64557	0.94337	0.40878	0.11673	0.0419	0.02968	0.02968	grey
RUSC1	0.01858	-0.09767	-0.02341	-0.05416	0.11538	-0.0422	0.07064	-0.2752	0.2752	0.80942	0.20377	0.76123	0.48173	0.13292	0.58366	0.35859	0.00027	0.00027	greenyellow
NBDY	0.00914	-0.15838	-0.06079	-0.05703	0.03106	-0.10174	0.00104	-0.13937	0.13937	0.90554	0.03854	0.42961	0.45875	0.68674	0.18547	0.98927	0.06907	0.06907	grey
LAMTOR2	0.07472	0.05771	-0.0637	-0.06927	0.0229	0.05718	0.18953	-0.41539	0.41539	0.33141	0.4534	0.40786	0.36799	0.76624	0.45754	0.01304	1.61E-08	1.61E-08	greenyellow
RHOU	0.01225	-0.04921	-0.07798	0.03765	-0.01683	0.0766	-0.06427	0.27973	-0.27973	0.87365	0.5227	0.31068	0.62489	0.82703	0.31936	0.40365	0.00021	0.00021	turquoise
ST6GALN/	0.07259	0.1292	-0.15729	-0.00146	0.06066	-0.13784	-0.12084	0.08989	-0.08989	0.3454	0.09214	0.03992	0.98489	0.43062	0.07221	0.11539	0.24234	0.24234	grey
DAP3	0.01584	0.02475	0.03575	-0.08631	-0.07944	0.0943	0.17095	-0.19952	0.19952	0.83706	0.74799	0.64245	0.26168	0.30169	0.21988	0.02538	0.00889	0.00889	greenyellow
MSI2	0.02586	-0.15457	-0.15193	-0.06978	0.03905	-0.1093	-0.11726	0.12475	-0.12475	0.73706	0.04354	0.04729	0.36449	0.61208	0.15472	0.12665	0.10402	0.10402	turquoise
DKC1	-0.04373	-0.02273	-0.02869	-0.02162	-0.09897	0.03327	0.13359	-0.16992	0.16992	0.57004	0.76791	0.70955	0.77898	0.1978	0.66578	0.08152	0.02629	0.02629	turquoise
VIRMA	-0.0351	-0.1025	0.04533	0.02724	-0.19461	0.01562	0.09717	0.08973	-0.08973	0.64859	0.18219	0.55605	0.72355	0.01075	0.83935	0.20609	0.24317	0.24317	turquoise
TMEM217	-0.13139	-0.18581	-0.03624	0.11734	-0.15539	0.05316	-0.05445	0.25991	-0.25991	0.08671	0.01497	0.6379	0.12639	0.04242	0.48984	0.47938	0.0006	0.0006	grey
FYN	-0.08295	-0.08518	0.0062	-0.01884	-0.14166	-0.00601	-0.15198	0.51106	-0.51106	0.28078	0.26796	0.9359	0.80682	0.06457	0.93785	0.04722	9.20E-13	9.20E-13	blue
DBNDD2	0.07884	-0.04201	0.025	0.04842	0.18415	0.06877	0.05462	-0.36828	0.36828	0.30538	0.58533	0.7455	0.52941	0.0159	0.37143	0.478	7.20E-07	7.20E-07	green
ZNF782	-0.0292	-0.12619	-0.10604	-0.04415	0.00128	0.00715	-0.06129	0.16527	-0.16527	0.70465	0.10005	0.16746	0.56636	0.98672	0.926	0.42584	0.03075	0.03075	turquoise
DNPH1	0.07947	0.14117	-0.09957	-0.08368	0.18684	-0.1254	0.08021	-0.28605	0.28605	0.30148	0.06552	0.19507	0.27653	0.01441	0.10221	0.297	0.00015	0.00015	grey
NAF1	-0.10725	-0.07778	-0.03507	0.02298	-0.09007	0.10132	-0.02431	0.21565	-0.21565	0.16265	0.31192	0.64884	0.76541	0.24136	0.18732	0.75233	0.00462	0.00462	turquoise
ARHGEF2	0.0157	-0.03164	0.08577	0.0186	-0.01021	0.20685	0.16303	-0.17141	0.17141	0.83849	0.68123	0.26468	0.80924	0.8946	0.00664	0.03313	0.02498	0.02498	yellow
ZNF852	-0.02347	-0.1577	-0.08825	-0.04482	0.05091	0.01674	-0.04961	0.26837	-0.26837	0.76062	0.03941	0.25104	0.5605	0.50843	0.828	0.51935	0.00039	0.00039	turquoise
SEMA3A	-0.03731	-0.10528	0.00721	0.02336	-0.20648	0.01503	0.1511	0.10431	-0.10431	0.62804	0.17057	0.92543	0.76172	0.00674	0.8453	0.04852	0.17455	0.17455	turquoise
TK2	-0.04007	-0.09565	0.03864	-0.0371	-0.14041	0.0991	0.05752	-0.04003	0.04003	0.60284	0.21332	0.61581	0.62997	0.067	0.19719	0.45489	0.60322	0.60322	turquoise
EPB41L5	0.01695	-0.24487	-0.16077	-0.06257	0.03487	-0.05531	-0.09616	0.28137	-0.28137	0.82585	0.00125	0.03568	0.4162	0.65069	0.47246	0.21087	0.00019	0.00019	turquoise
PRSS22	0.13378	0.01265	0.12103	0.04251	0.03162	0.23668	0.18282	-0.45711	0.45711	0.08109	0.86961	0.11483	0.58093	0.68142	0.00183	0.0167	3.28E-10	3.28E-10	yellow
TMEM52B	-0.10538	-0.02129	0.13165	0.06245	-0.22452	0.14929	0.08977	0.28946	-0.28946	0.17013	0.78221	0.08608	0.41709	0.00315	0.05131	0.24297	0.00012	0.00012	blue
HPN	-0.0176	0.07466	-0.14842	-0.01752	0.13222	-0.05731	-0.13058	0.13713	-0.13713	0.81927	0.33181	0.0527	0.82003	0.08474	0.45658	0.0887	0.0737	0.0737	grey
BIRC6	-0.05826	-0.15322	-0.01903	0.02491	-0.10602	0.09019	0.02897	0.19435	-0.19435	0.44912	0.04541	0.8049	0.74643	0.16755	0.24075	0.70683	0.01086	0.01086	turquoise
PCGF1	0.11893	-0.04201	-0.06791	-0.10614	0.0116	-0.02291	0.15963	-0.36915	0.36915	0.12129	0.58538	0.37745	0.16708	0.88028	0.76615	0.03702	6.75E-07	6.75E-07	greenyellow
HTR3A	0.01919	0.02861	0.03471	0.04294	-0.16014	-0.02982	-0.10775	-0.12911	0.12911	0.80328	0.71027	0.65219	0.57709	0.03642	0.69861	0.16067	0.09238	0.09238	grey
GBP2	-0.01771	0.01765	0.09169	0.09181	-0.19358	0.2913	0.27892	-0.23046	0.23046	0.81815	0.81873	0.23295	0.23235	0.01118	0.00011	0.00022	0.00243	0.00243	turquoise
EEF1AKM1	0.04056	-0.02662	-0.07914	-0.13716	-0.03897	-0.13136	-0.04042	0.21282	-0.21282	0.59841	0.72965	0.30353	0.07363	0.61283	0.08679	0.59967	0.0052	0.0052	grey
TOP2A	-0.04672	0.02457	0.05492	-0.00122	-0.08159	0.13734	0.28364	-0.3097	0.3097	0.54401	0.74974	0.47558	0.98739	0.28873	0.07326	0.00017	3.76E-05	3.76E-05	pink
CISD2	-0.08056	-0.1479	-0.0601	-0.00045	0.07791	-0.12117	-0.01671	-0.09824	0.09824	0.29489	0.05355	0.43492	0.9953	0.31112	0.1144	0.8283	0.20112	0.20112	turquoise
TMEM181	0.06872	-0.06637	-0.00095	-0.00082	-0.04458	0.06865	0.04202	0.02642	-0.02642	0.37183	0.38841	0.9902	0.9915	0.56262	0.37231	0.58528	0.73155	0.73155	turquoise
CCL4L2	-0.13275	-0.02382	-0.026	0.03772	0.01468	-0.0357	-0.06508	0.32666	-0.32666	0.08348	0.75713	0.73569	0.62429	0.84886	0.64293	0.39774	1.30E-05	1.30E-05	grey
ANXA1	-0.05493	-0.08508	0.09076	0.06926	-0.19791	0.1749	0.1493	-0.08827	0.08827	0.47548	0.26857	0.23779	0.36803	0.00947	0.02214	0.0513	0.25097	0.25097	black
LAMTOR5	-0.0024	-0.09733	-0.04675	-0.09441	0.11348	-0.01858	0.00568	-0.23611	0.23611	0.97518	0.20536	0.54369	0.21934	0.13946	0.80939	0.94125	0.00188	0.00188	turquoise
SGPP1	-0.0989	-0.14932	-0.11142	0.07298	-0.01989	-0.05526	-0.05515	0.28294	-0.28294	0.19809	0.05127	0.14684	0.34279	0.7962	0.47281	0.47373	0.00018	0.00018	turquoise
TCP11L2	0.02703	-0.20594	-0.02369	0.01224	-0.06085	-0.00316	-0.08328	0.25317	-0.25317	0.72559	0.00689	0.75847	0.87376	0.42916	0.9673	0.27882	0.00083	0.00083	turquoise
FAM98B	-0.07359	-0.06341	-0.11863	-0.083	-0.06362	-0.10615	-0.05117	0.20227	-0.20227	0.33876	0.40995	0.12224	0.28045	0.40844	0.16702	0.50623	0.00798	0.00798	turquoise
RALGPS1	0.06925	-0.14956	-0.11307	-0.11831	0.13795	-0.10729	-0.14563	0.05173	-0.05173	0.36815	0.0509	0.14089	0.12329	0.07197	0.1625	0.05736	0.50161	0.50161	grey
LRRC56	0.1223	-0.09741	-0.21975	-0.18465	0.24269	-0.23621	-0.07808	-0.17367	0.17367	0.11105	0.20498	0.00388	0.01562	0.00138	0.00187	0.31005	0.02311	0.02311	grey
TOR2A	0.04022	-0.001	-0.10524	-0.04836	0.22364	-0.16207	-0.07613	-0.0707	0.0707	0.6015	0.98966	0.17072	0.52992	0.00328	0.03419	0.32231	0.35812	0.35812	grey
SSTR3	-0.00063	-0.09793	-0.22332	-0.17139	0.05929	-0.29608	-0.21039	0.43419	-0.43419	0.99348	0.20254	0.00332	0.025	0.4411	8.43E-05	0.00574	2.97E-09	2.97E-09	brown
TSPAN33	-0.04101	-0.0771	-0.12676	-0.0219	0.04277	-0.20978	-0.23509	0.4908	-0.4908	0.59431	0.31621	0.09851	0.77616	0.57856	0.00589	0.00197	9.44E-12	9.44E-12	brown
C5orf51	-0.08592	-0.17216	-0.02379	0.02045	-0.18674	0.04865	0.05235	0.19354	-0.19354	0.26381	0.02435	0.75741	0.7906	0.01446	0.52746	0.49654	0.0112	0.0112	turquoise
UCN2	-0.06989	0.01328	0.02836	0.1197	0.03882	0.00931	0.17518	-0.16934	0.16934	0.36373	0.8631	0.71268	0.11891	0.61419	0.90378	0.02192	0.02682	0.02682	grey
CCSAP	-0.00751	-0.18207	-0.07341	-0.02586	-0.02519	-0.14558	-0.00841	0.3196	-0.3196	0.92234	0.01716	0.34	0.73707	0.74363	0.05745	0.91311	2.04E-05	2.04E-05	turquoise
CNOT9	-0.03831	-0.06958	-0.04184	-0.01401	-0.0036	0.03557	0.04716	-0.07649	0.07649	0.61882	0.36587	0.5869	0.85572	0.9627	0.64418	0.54019	0.32006	0.32006	turquoise
MT-ND5	-0.14411	-0.17793	-0.04206	0.02415	0.19702	-0.06551	-0.12253	0.01029	-0.01029	0.06005	0.0199	0.58488	0.75388	0.0098	0.3946	0.11036	0.89374	0.89374	grey
SELENOP	-0.02016	-0.09736	0.01221	0.02879	-0.03364	0.07076	-0.12167	0.52593	-0.52593	0.7935	0.20522	0.87403	0.70857	0.66229	0.35772	0.11291	1.50E-13	1.50E-13	turquoise

SLC8A2	-0.0161	-0.07452	-0.23458	-0.10504	0.07952	-0.31881	-0.22035	0.44671	-0.44671	0.83442	0.33273	0.00201	0.17152	0.30118	2.14E-05	0.00378	9.11E-10	9.11E-10	brown
GPR75	-0.10747	-0.24321	-0.17397	-0.09119	-0.05317	-0.16087	-0.14048	0.42877	-0.42877	0.16178	0.00135	0.02287	0.23554	0.48979	0.03556	0.06686	4.89E-09	4.89E-09	turquoise
ENTPD8	0.13022	-0.02621	0.00509	0.07295	0.13799	0.12315	0.04578	-0.32979	0.32979	0.08959	0.73367	0.94737	0.34301	0.07188	0.10856	0.55217	1.06E-05	1.06E-05	grey
KCTD10	-0.08969	-0.14585	0.00106	0.0279	-0.12909	0.01822	0.01462	0.33851	-0.33851	0.24336	0.05698	0.98903	0.71715	0.09243	0.81303	0.84943	5.94E-06	5.94E-06	blue
CARD6	0.01298	0.01203	0.04773	0.08414	-0.13175	0.25062	0.19201	-0.07472	0.07472	0.8662	0.87589	0.53533	0.27391	0.08584	0.00095	0.01188	0.33138	0.33138	turquoise
IL11RA	-0.06059	-0.08237	-0.1689	-0.04501	0.07447	-0.10781	-0.13648	0.36335	-0.36335	0.43118	0.28414	0.02722	0.55881	0.33303	0.16043	0.07509	1.04E-06	1.04E-06	grey
TACC1	-0.01182	-0.18252	-0.06429	0.10587	-0.09838	0.10763	-0.03556	0.43858	-0.43858	0.87801	0.01688	0.40351	0.16817	0.20051	0.16115	0.64424	1.97E-09	1.97E-09	turquoise
PTPRA	-0.00392	-0.08289	-0.11395	-0.04229	-0.0175	-0.1298	-0.04881	0.2345	-0.2345	0.95937	0.29653	0.13782	0.58288	0.82028	0.09062	0.52612	0.00202	0.00202	turquoise
LTF	0.04119	0.04316	0.05284	0.10212	-0.09574	0.16613	0.03419	-0.00456	0.00456	0.59271	0.5751	0.49248	0.18382	0.21288	0.02988	0.65712	0.95281	0.95281	grey
NOL8	-0.01766	-0.08026	-0.04058	0.01427	-0.17477	0.04611	0.08474	0.17544	-0.17544	0.81865	0.29668	0.59821	0.85302	0.02224	0.54924	0.27048	0.02173	0.02173	turquoise
SETDB1	-0.00215	-0.06132	-0.04469	-0.08955	-0.01621	0.07223	0.08555	-0.05692	0.05692	0.97777	0.4256	0.56161	0.24412	0.83336	0.34784	0.26588	0.45958	0.45958	turquoise
SLC41A1	-0.10049	-0.1633	-0.02845	0.00725	-0.17743	0.01469	-0.10104	0.42309	-0.42309	0.19097	0.03283	0.71185	0.92505	0.02025	0.84878	0.18853	8.16E-09	8.16E-09	turquoise
RHOT1	-0.11132	-0.17986	-0.11922	0.03059	0.03077	-0.07184	-0.08465	0.30143	-0.30143	0.14718	0.01857	0.12039	0.69119	0.68954	0.35044	0.27099	6.17E-05	6.17E-05	turquoise
HMCES	-0.03927	-0.05455	-0.13074	-0.09993	-0.01499	0.00076	0.10032	-0.23318	0.23318	0.61012	0.47852	0.08829	0.19345	0.84573	0.99215	0.19173	0.00215	0.00215	turquoise
TOB1	-0.05589	-0.06685	0.07062	0.05785	0.10102	0.11197	0.00328	-0.23925	0.23925	0.46783	0.385	0.35873	0.45233	0.18862	0.14482	0.96603	0.00162	0.00162	turquoise
SQOR	-0.02589	-0.00178	0.06818	-0.03204	-0.03864	0.09283	0.18671	-0.4095	0.4095	0.73677	0.98152	0.37557	0.67736	0.61583	0.22721	0.01448	2.67E-08	2.67E-08	yellow
DSP	0.04395	-0.02385	-0.05083	-0.10855	0.04754	-0.03156	-0.0651	-0.12367	0.12367	0.56818	0.75686	0.50911	0.15758	0.53694	0.68201	0.39761	0.10706	0.10706	turquoise
KCTD7	0.04289	-0.22291	-0.10462	-0.04535	-0.02733	-0.0219	-0.04419	0.3242	-0.3242	0.57752	0.00338	0.17325	0.5559	0.72274	0.77621	0.56602	1.52E-05	1.52E-05	turquoise
FGL1	0.04124	0.04294	-0.06653	0.00881	-0.00583	-0.05978	-0.16214	0.09205	-0.09205	0.59226	0.5771	0.38731	0.90894	0.93969	0.43738	0.03411	0.23113	0.23113	magenta
SKA1	-0.08665	0.04653	-0.00818	-0.05878	0.00529	-0.01193	0.23626	-0.33511	0.33511	0.25979	0.54559	0.91544	0.44508	0.94523	0.87694	0.00186	7.46E-06	7.46E-06	pink
RAB4A	0.07813	-0.00683	-0.105	-0.06384	0.05235	0.04166	0.07524	-0.04338	0.04338	0.30979	0.92931	0.1717	0.40678	0.49653	0.58849	0.32802	0.57319	0.57319	turquoise
PNLIP	0.00623	0.06459	0.06077	0.11819	-0.10909	0.09708	-0.08839	0.14764	-0.14764	0.93556	0.40127	0.42981	0.12366	0.1555	0.20652	0.25032	0.05398	0.05398	magenta
DPH1	-0.05739	-0.17955	-0.19128	-0.10312	0.14659	-0.23211	-0.1761	0.36043	-0.36043	0.45595	0.01878	0.0122	0.17953	0.05573	0.00225	0.02123	1.28E-06	1.28E-06	brown
CYP39A1	0.01133	-0.0998	-0.09261	0.08449	-0.0061	0.00913	-0.09286	0.34811	-0.34811	0.88312	0.19402	0.22831	0.27188	0.93689	0.90569	0.22704	3.08E-06	3.08E-06	magenta
DUSP28	0.05718	-0.11709	-0.08081	-0.13477	0.1537	-0.12548	-0.11615	-0.0931	0.0931	0.45758	0.12722	0.2934	0.07885	0.04474	0.10199	0.13033	0.22585	0.22585	grey
SDC3	-0.15762	-0.09537	-0.009	0.0758	-0.15969	0.10434	0.03986	0.22851	-0.22851	0.0395	0.21469	0.90704	0.32444	0.03695	0.17442	0.60469	0.00265	0.00265	blue
ATP1A2	-0.06184	-0.02759	-0.06354	0.07793	0.03991	-0.04845	-0.0747	0.43836	-0.43836	0.42166	0.72021	0.40904	0.31098	0.60429	0.52918	0.33156	2.02E-09	2.02E-09	salmon
TC2N	0.06591	0.04415	0.12976	0.16177	-0.00063	0.27721	0.05711	-0.23979	0.23979	0.39175	0.56641	0.09073	0.03453	0.99352	0.00024	0.4581	0.00158	0.00158	yellow
ABR	-0.11056	-0.15421	-0.03585	-0.05187	0.0366	-0.12693	-0.0807	0.15221	-0.15221	0.14998	0.04402	0.64161	0.50043	0.63465	0.09804	0.29404	0.04688	0.04688	turquoise
NUDT7	-0.09031	0.06922	-0.16206	-0.02765	-0.00152	-0.10447	-0.07504	0.16896	-0.16896	0.2401	0.36831	0.0342	0.71965	0.98428	0.1739	0.32936	0.02717	0.02717	grey
CATSPER1	0.01781	-0.07811	0.03391	-0.00508	-0.09482	0.08407	0.21431	-0.37245	0.37245	0.81712	0.30985	0.65976	0.94742	0.21732	0.27427	0.00488	5.27E-07	5.27E-07	yellow
SLC25A27	0.00014	-0.17797	-0.25296	-0.08141	0.22008	-0.25542	-0.17688	0.28669	-0.28669	0.99855	0.01987	0.00084	0.28984	0.00382	0.00075	0.02065	0.00014	0.00014	brown
ARHGAP2	-0.00346	0.01679	-0.0283	0.0617	-0.05412	0.07476	0.08	0.21563	-0.21563	0.9642	0.82746	0.71327	0.4227	0.48201	0.33116	0.29829	0.00462	0.00462	black
ATP2A2	0.03129	-0.1759	-0.03064	-0.02884	-0.00411	-0.04434	-0.03464	0.11272	-0.11272	0.68455	0.02138	0.69076	0.70812	0.95741	0.56474	0.65287	0.14215	0.14215	turquoise
ARHGEF1(	0.04082	0.06459	0.05557	-0.03325	0.15028	0.15522	0.10845	-0.48586	0.48586	0.59606	0.40129	0.47039	0.66593	0.04977	0.04264	0.15796	1.63E-11	1.63E-11	yellow
SLF2	0.00048	-0.18214	-0.06768	0.00341	-0.04659	-0.13085	0.04214	0.3004	-0.3004	0.99507	0.01711	0.37914	0.96467	0.54513	0.08803	0.58424	6.55E-05	6.55E-05	turquoise
MTF1	-0.04291	-0.1662	-0.05347	0.01869	-0.12866	0.05345	-0.06229	0.29628	-0.29628	0.57734	0.02981	0.48731	0.80824	0.09353	0.48752	0.41829	8.33E-05	8.33E-05	turquoise
RABEP1	-0.0932	-0.14216	-0.07127	-0.03516	-0.0234	-0.14041	-0.09595	0.49052	-0.49052	0.22533	0.06363	0.35426	0.64803	0.76125	0.067	0.21187	9.73E-12	9.73E-12	turquoise
ARHGAP4	-0.06282	-0.13909	0.06302	0.08011	-0.22243	0.21055	0.16225	0.02095	-0.02095	0.41435	0.06962	0.41288	0.29761	0.00346	0.00571	0.03399	0.78561	0.78561	turquoise
MAPK8IP1	-0.06078	-0.10745	-0.24555	-0.06764	0.04932	-0.3336	-0.29204	0.48337	-0.48337	0.42968	0.16185	0.00121	0.37937	0.52175	8.24E-06	0.00011	2.13E-11	2.13E-11	brown
DOCK7	-0.08882	-0.17962	0.0151	0.02994	-0.14298	0.0976	0.01963	0.24373	-0.24373	0.24798	0.01873	0.84459	0.69752	0.0621	0.20409	0.79885	0.00132	0.00132	turquoise
LPAR1	-0.09281	-0.13479	-0.00182	0.00777	-0.16794	0.00802	0.01891	0.50644	-0.50644	0.22729	0.07881	0.98112	0.9197	0.02812	0.91712	0.80612	1.58E-12	1.58E-12	blue
HSPE1	0.11879	-0.00143	0.00275	0.01132	0.05544	0.00477	0.1351	-0.43019	0.43019	0.12174	0.9852	0.9715	0.88321	0.4714	0.95066	0.0781	4.30E-09	4.30E-09	greenyellow
CCDC92	-0.05539	-0.17025	-0.13227	-0.01681	0.06514	-0.19665	-0.15986	0.29981	-0.29981	0.47182	0.02599	0.08461	0.82724	0.39727	0.00994	0.03675	6.78E-05	6.78E-05	turquoise
ZYX	0.00086	-0.02292	0.08378	0.02301	-0.12503	0.20823	0.1808	-0.09173	0.09173	0.99114	0.76599	0.27595	0.76514	0.10323	0.00628	0.01796	0.23276	0.23276	black
MT-ATP6	-0.02068	-0.08279	-0.11318	-0.10491	0.19319	-0.02857	-0.0974	-0.19795	0.19795	0.78834	0.28168	0.14049	0.17206	0.01135	0.71072	0.20502	0.00945	0.00945	grey
EMP1	-0.09996	-0.16163	0.07487	0.12018	-0.1994	0.18036	0.05869	0.20389	-0.20389	0.19334	0.03468	0.33044	0.11741	0.00893	0.01824	0.4458	0.00748	0.00748	blue
PPIA	0.03675	0.02601	0.02128	-0.00698	-0.05059	0.06998	0.20426	-0.45191	0.45191	0.63326	0.7356	0.78236	0.92784	0.51108	0.3631	0.00737	5.49E-10	5.49E-10	greenyellow
GAL3ST4	-0.08263	-0.0015	0.08102	0.11719	-0.25981	0.19415	0.17017	0.32901	-0.32901	0.28264	0.98449	0.29214	0.12689	0.0006	0.01094	0.02606	1.11E-05	1.11E-05	blue
TMEM198	0.06465	-0.06507	-0.31447	-0.1103	0.2569	-0.42055	-0.23217	0.23263	-0.23263	0.40084	0.3978	2.80E-05	0.15096	0.00069	1.02E-08	0.00225	0.0022	0.0022	brown
FAM183A	-0.05896	-0.11656	-0.20222	-0.18449	0.10022	-0.24365	-0.15994	0.0704	-0.0704	0.44369	0.12895	0.00799	0.01571	0.19217	0.00132	0.03665	0.36017	0.36017	grey
AP3S2	-0.04861	-0.13532	-0.18387	-0.09698	0.01982	-0.13277	-0.12734	0.1781	-0.1781	0.52777	0.07761	0.01607	0.20701	0.79692	0.08342	0.09697	0.01978	0.01978	turquoise
SLC52A3	0.00913	0.0294	0.01659	0.06304	-0.12278	0.1492	0.27281	-0.38252	0.38252	0.90561	0.70269	0.82953	0.41271	0.10962	0.05146	0.00031	2.43E-07	2.43E-07	yellow
DHFR2	-0.0371	-0.09294	-0.15663	-0.04956	-0.05858	-0.01124	-0.06704	0.31122	-0.31122	0.62998	0.22665	0.04078	0.51973	0.44665	0.88395	0.38363	3.42E-05	3.42E-05	turquoise
CST6	0.06474	-0.15931	0.06074	0.07482	-0.13024	0.10264	0.08207	-0.20785	0.20785	0.40019	0.03741	0.43004	0.33079	0.08954	0.18157	0.28591	0.00637	0.00637	yellow
KYAT1	0.12156	-0.07396	-0.06666	-0.09012	-0.02481	-0.08747	0.07656	-0.34064	0.34064	0.11323	0.33638	0.38636	0.24112	0.74742	0.25527	0.31959	5.14E-06	5.14E-06	grey
ALKBH4	-0.02523	-0.02347	-0.15298	-0.036	0.19776	-0.14892	-0.02129	-0.08173	0.08173	0.74325	0.76059	0.04576	0.64017	0.00952	0.0519	0.78223	0.2879	0.2879	green
SDCBP	-0.07839	-0.00368	0.06372	0.09014	-0.14732	0.16507	0.07515	0.10813	-0.10813	0.30814	0.96186	0.40771	0.24098	0.05451	0.03096	0.32864	0.1592	0.1592	turquoise
BLZF1	-0.04388	-0.08281	0.07206	0.02639	-0.00532	0.16631	0.18101	-0.14963	0.14963	0.56874	0.2816	0.34895	0.73191	0.94497	0.02971	0.01782	0.05078	0.05078	turquoise
SMC5	-0.00073	-0.14805	-0.02083	0.05178	-0.14715	0.15602	0.0912	0.01723	-0.01723	0.99243	0.05331	0.78686	0.50123	0.05479	0.04157	0.23551	0.82297	0.82297	turquoise
CHST6	-0.01444	-0.0375	0.00905	0.02499	-0.08643	-0.02517	0.22059	-0.23818	0.23818	0.85127	0.62631	0.90647	0.7456	0.261	0.74387	0.00374	0.00171	0.00171	grey
BCL6B	-0.15264	-0.12859	-0.06361	0.10645	-0.09469	0.03044	-0.12111	0.56608	-0.56608	0.04626	0.09371	0.40853	0.16581	0.218	0.69269	0.1146	7.11E-16	7.11E-16	blue
JAKMIP2	-0.03185	-0.08778	-0.16854	-0.13017	0.04625	-0.34296	-0.23678	0.48409	-0.48409	0.67924	0.2536	0.02755	0.0897	0.54804	4.39E-06	0.00182	1.97E-11	1.97E-11	brown
NINL	-0.05327	-0.17716	-0.1305	0.03162	-0.00529	-0.1934	-0.02425	0.17641	-0.17641	0.48899	0.02045	0.0889	0.6814	0.94528	0.01126	0.75289	0.021	0.021	grey
BRIP1	-0.12168	0.09344	0.0082	-0.04924	-0.09563	0.03577	0.30689	-0.29534	0.29534	0.11288	0.22417	0.91526	0.52242	0.21342	0.64231	4.45E-05	8.80E-05	8.80E-05	pink
SORL1	-0.01725	-0.11707	-0.00617	-0.01215	-0.07257	0.07593	-0.05864	0.17879	-0.17879	0.82276	0.12729	0.93618	0.87465	0.34558	0.32361	0.44614	0.0193	0.0193	turquoise
QDPR	-0.02005	-0.06311	-0.28098	-0.16202	0.15554	-0.39379	-0.25706	0.31877	-0.31877	0.79465	0.41217	0.0002	0.03424	0.04221	9.89E-08	0.00069	2.14E-05	2.14E-05	brown
PCYOX1L	-0.02134	-0.14564	-0.24681	-0.21152	0.13133	-0.16268	-0.20317	0.15619	-0.15619	0.78178	0.05734	0.00114	0.00548	0.08686	0.03352	0.0077	0.04135	0.04135	turquoise
LGR4	-0.00298	0.03813	0.0999	0.02341	-0.0035	0.1944	0.05768	-0.14346	0.14346	0.96917	0.62051	0.19358	0.76119	0.96372	0.01084	0.45361	0.06122	0.06122	turquoise
ODAM	-0.00068	0.04547	0.07951	0.09594	-0.09449	0.24488	0.083	-0.00546	0.00546	0.99299	0.55482	0.30128	0.21196	0.21896	0.00125	0.28048	0.94354	0.94354	grey
RBM38	0.07302	0.01487	0.08676	0.07526	0.02048	0.12557	0.02991	-0.12256	0.12256	0.34255	0.84691	0.25917	0.32792	0.79038	0.10174	0.69777	0.11029	0.11029	grey
COX18	-0.16165	-0.0474	-0.17601	-0.07203	0.1262	-0.12117	-0.04172	0.14338	-0.14338	0.03466	0.53817	0.02129	0.34915	0.10002	0.1144	0.58793	0.06137	0.06137	turquoise
EDF1	0.09218	0.0155	-0.12782	-0.08046	0.05088	-0.11669	0.05546	-0.31906	0.31906	0.23047	0.8405	0.09571	0.29548	0.50872	0.12853	0.47126	2.11E-05	2.11E-05	grey
AKAP9	-0.00074	-0.20527	-0.0748	0.00308	0.0451	-0.0231	-0.05818	0.22221	-0.22221	0.99229	0.00707	0.33086	0.96814	0.55808	0.76422	0.44976	0.00349	0.00349	turquoise
LILRA5	-0.13965	0.018	0.12352	0.05665	-0.18251	0.10274	0.01898	0.34098	-0.34098	0.06851	0.81526	0.10749	0.46176	0.01689	0.18116	0.80541	5.03E-06	5.03E-06	blue
CELA2B	-0.00901	0.05489	0.0654	0.1335	-0.11396	0.07168	-0.08363	0.15453	-0.15453	0.90689	0.4758	0.39541	0.08172	0.13777	0.3515	0.2768	0.04359	0.04359	magenta
PLA2G4C	-0.0576	-0.1649	-0.1451	0.00248	-0.05274	-0.07362	-0.1779	0.42894	-0.42894	0.45424	0.03114	0.05829	0.9743	0.4933	0.33858	0.01992	4.81E-09	4.81E-09	grey
SPPL2B	0.03715	-0.01739	-0.17356	-0.08833	0.36004	-0.18826	-0.11543	-0.1852	0.1852	0.62949	0.82148	0.0232	0.2506	1.32E-06	0.01367	0.13272	0.01531	0.01531	green
FAM171A;	-0.00719	-0.16175	-0.16154	-0.0263	0.12685	-0.36026	-0.12653	0.35096	-0.35096	0.9256	0.03455	0.03479	0.73276	0.09827	1.30E-06	0.09914	2.52E-06	2.52E-06	brown
GFPT2	-0.10979	-0.07685	0.07027	0.11325	-0.25804	0.1622	0.08684	0.37383	-0.37383	0.15287	0.31777	0.3611	0.14025	0.00066	0.03404	0.25874	4.74E-07	4.74E-07	blue
ALPK1	-0.07783	-0.03031	0.12505	0.13092	-0.11941	0.31647	0.29045	-0.17923	0.17923	0.31162	0.69395	0.10316	0.08786	0.1198	2.48E-05	0.00012	0.019	0.019	turquoise
LYPD5	-0.00898	0.04357	0.04609	0.05044	-0.10456	0.17515	0.27952	-0.05296	0.05296	0.90724	0.57152	0.54943	0.5124	0.1735	0.02194	0.00021	0.4915	0.4915	grey
NR2C1	0.01745	-0.11183	0.05333	-0.0218	0.13351	0.02645	-0.03293	-0.12945	0.12945	0.82076	0.14535	0.48846	0.77718	0.0817	0.7313	0.66896	0.0915	0.0915	red
ZNF470	-0.10547	-0.17341	-0.03918	0.09834	0.00702	0.0476	-0.1629	0.36986	-0.36986	0.16977	0.02331	0.61088	0.20065	0.92737	0.5364	0.03327	6.40E-07	6.40E-07	turquoise
SMAD3	-0.01325	-0.1285	0.12609	0.13393	-0.10582	0.21585	0.13778	-0.34287	0.34287	0.86344	0.09393	0.10031	0.08075	0.16837	0.00458	0.07232	4.42E-06	4.42E-06	turquoise
NDN	-0.04878	-0.09076	-0.09023	-0.04253	-0.09184	-0.10579	-0.16193	0.52529	-0.52529	0.52635	0.23776	0.24055	0.58073	0.23222	0.16849	0.03434	1.63E-13	1.63E-13	blue
CARD14	0.06738	-0.03145	0.05561	0.1268	0.08901	0.08677	0.24397	-0.32837	0.32837	0.38122	0.68298	0.47007	0.09841	0.24696	0.2591	0.0013	1.16E-05	1.16E-05	grey
MTMR7	-0.06152	-0.1421	-0.1925	-0.13114	0.08703	-0.35277	-0.20693	0.46645	-0.46645	0.42412	0.06373	0.01166	0.08733	0.25767	2.22E-06	0.00662	1.28E-10	1.28E-10	brown
PLA2G4A	-0.05013	-0.0546	0.10919	0.10332	-0.01273	0.30279	0.08894	-0.13081	0.13081	0.51494	0.47814	0.15514	0.17869	0.86873	5.69E-05	0.24733	0.08812	0.08812	turquoise
TANGO2	-0.09341	-0.02392	-0.15716	-0.06229	-0.00194	-0.17975	-0.04908	0.30565	-0.30565	0.2243	0.75613	0.04009	0.4183	0.9799	0.01865	0.52383	4.80E-05	4.80E-05	grey
NUP98	0.02009	-0.11695	0.03972	-0.04677	-0.14411	0.13288	0.06647	0.07491	-0.07491	0.79424	0.12767	0.60599	0.54359	0.06005	0.08317	0.3877	0.33015	0.33015	turquoise
PIGX	-0.03245	-0.09015	-0.14033	-0.04782	0.00772	-0.10003	0.0592	0.05099	-0.05099	0.67348	0.24093	0.06714	0.53458	0.92019	0.19302	0.44179	0.5078	0.5078	turquoise
SLC19A2	-0.09835	-0.11397	0.00267	0.09995	-0.05422	0.18324	0.03525	0.23545	-0.23545	0.20064	0.13773	0.97236	0.19337	0.48124	0.01644	0.64719	0.00194	0.00194	turquoise
PILRA	-0.11125	0.00923	0.08126	0.04782	-0.2018	0.15574	0.04483	0.33748	-0.33748	0.14745	0.90465	0.2907	0.53454	0.00813	0.04194	0.5604	6.37E-06	6.37E-06	blue
NFATC4	-0.00981	-0.15814	-0.03282	0.02337	0.00425	-0.01993	0.02997	0.16892	-0.16892	0.89868	0.03884	0.67002	0.76163	0.95596	0.79579	0.69717	0.0272	0.0272	black
RAP1GAP;	-0.01509	-0.14508	-0.19592	-0.14805	0.13856	-0.29162	-0.23908	0.23435	-0.23435	0.84466	0.05832	0.01022	0.0533	0.07071	0.00011	0.00164	0.00203	0.00203	brown
TNS2	-0.0465	-0.14722	-0.11028	-0.01636	0.02703	-0.13502	-0.1967	0.51882	-0.51882	0.54585	0.05467	0.15103	0.83185	0.7256	0.07828	0.00992	3.62E-13	3.62E-13	blue
ALYREF	-0.01056	-0.0075	0.00146	-0.06821	0.00932	-0.07739	0.14401	-0.22721	0.22721	0.89101	0.92242	0.98484	0.3754	0.90366	0.31435	0.06023	0.0028	0.0028	grey
SOCS6	-0.06395	-0.19517	-0.09772	0.03733	0.07249	-0.02624	-0.04062	0.11822	-0.11822	0.40602	0.01059	0.20353	0.62783	0.3461	0.73337	0.59786	0.12355	0.12355	turquoise
DEPDC1	-0.10042	0.03859	0.06419	0.0043	-0.09413	0.1095	0.30804	-0.30178	0.30178	0.19128	0.61632	0.40423	0.95553	0.22075	0.15395	4.15E-05	6.04E-05	6.04E-05	pink
HNRNPL	0.05826	-0.14427	0.01042	0.02014	-0.04491	0.07693	0.08759	-0.19479	0.19479	0.44911	0.05975	0.89244	0.79373	0.55973	0.31728	0.25464	0.01068	0.01068	turquoise
IQCN	-0.02589	-0.23203	-0.19722	-0.03804	0.16058	-0.20219	-0.19754	0.22183	-0.22183	0.73681	0.00226	0.00972	0.62129	0.0359	0.008	0.0096	0.00355	0.00355	grey
TRMT6	0.02737	-0.01797	-0.02036	-0.03857	-0.00908	-0.09455	0.03495	0.03701	-0.03701	0.72234	0.81551	0.79151	0.61645	0.9062	0.21864	0.64998	0.63085	0.63085	turquoise
VWA7	0.02591	-0.1146	-0.1862	-0.11491	0.20086	-0.28799	-0.24724	0.06729	-0.06729	0.73654	0.13556	0.01475	0.1345	0.00843	0.00013	0.00111	0.38187	0.38187	brown
CC2D1A	0.08339	-0.02987	-0.12292	-0.03659	0.26906	-0.12612	0.01472	-0.27051	0.27051	0.27823	0.69811	0.10923	0.6347	0.00037	0.10024	0.84843	0.00035	0.00035	green
AVL9	-0.01623	-0.08601	0.00327	0.0374	0.00042	0.04741	0.05707	-0.17851	0.17851	0.83314	0.26334	0.96615	0.62721	0.9956	0.53806	0.45841	0.01949	0.01949	turquoise
TDRD5	0.01419	-0.01335	0.08345	-0.04697	0.05377	0.1	-0.02949	-0.04908	0.04908	0.85387	0.86236	0.27787	0.54186	0.48488	0.19314	0.70176	0.52383	0.52383	grey
CCL11	-0.14596	0.03345	-0.02664	-0.06241	-0.09893	0.02306	0.01191	0.37711	-0.37711	0.0568	0.66407	0.72942	0.4174	0.19797	0.76465	0.87713	3.69E-07	3.69E-07	blue
DSG3	0.07417	0.13711	0.14912	0.09385	-0.06766	0.08065	0.13916	-0.24337	0.24337	0.33496	0.07374	0.05158	0.22209	0.37923	0.29438	0.06948	0.00134	0.00134	grey
ARL1	-0.01673	-0.12816	-0.06578	-0.06062	0.10047	-0.12735	-0.15275	0.21824	-0.21824	0.82804	0.09481	0.39265	0.43093	0.19105	0.09693	0.04609	0.00414	0.00414	turquoise
C3orf52	0.09241	-0.03017	0.1026	0.07428	-0.0753	0.2949	0.18932	-0.43073	0.43073	0.2293	0.69523	0.18176	0.3343	0.32765	9.02E-05	0.01314	4.09E-09	4.09E-09	yellow
ANKRD49	-0.03831	-0.13413	-0.0458	-0.03091	0.03987	-0.02332	-0.00834	-0.04484	0.04484	0.61882	0.08029	0.55196	0.68819	0.60466	0.76207	0.91382	0.56031	0.56031	red
CAVIN4	0.02952	-0.23226	0.10122	0.073	-0.27392	0.07186	0.10715	0.1792	-0.1792	0.70156	0.00224	0.18774	0.34266	0.00029	0.3503	0.16305	0.01902	0.01902	grey
PPIE	0.06424	-0.00996	-0.03441	-0.02647	-0.02727	-0.01774	0.02342	-0.16537	0.16537	0.40383	0.89715	0.65505	0.73115	0.72329	0.81784	0.76106	0.03066	0.03066	turquoise
ERP44	0.09514	-0.05154	-0.08954	0.00021	-0.10464	-0.04653	0.02411	-0.07759	0.07759	0.2158	0.50317	0.24416	0.99786	0.1732	0.54562	0.75427	0.31309	0.31309	turquoise
TAF4B	-0.05722	0.0078	0.05404	0.06143	-0.21835	0.20296	0.07076	0.17107	-0.17107	0.45729	0.91934	0.48265	0.42475	0.00412	0.00776	0.35771	0.02528	0.02528	turquoise
PCID2	0.01688	-0.01834	-0.05026	-0.05936	0.06133	-0.11007	-0.02632	-0.00143	0.00143	0.82656	0.8118	0.51386	0.44055	0.42553	0.1518	0.73259	0.98516	0.98516	red
SARM1	-0.10234	-0.17123	-0.2112	-0.0856	0.11678	-0.22861	-0.24394	0.37248	-0.37248	0.18288	0.02514	0.00556	0.26561	0.12822	0.00263	0.0013	5.25E-07	5.25E-07	brown
SMAP2	-0.07918	-0.06753	-0.0344	-0.02162	-0.14093	-0.03795	-0.16324	0.46673	-0.46673	0.30326	0.38019	0.65507	0.779	0.06598	0.62212	0.0329	1.24E-10	1.24E-10	blue
LIPT2	-0.10319	-0.04304	-0.06893	-0.01487	0.15704	-0.06897	0.0215	-0.11511	0.11511	0.17925	0.57622	0.37034	0.84692	0.04024	0.37009	0.78013	0.13382	0.13382	grey
SRF	-0.02511	-0.04164	-0.04792	0.02701	-0.14063	0.04444	-0.02577	0.30516	-0.30516	0.74446	0.58872	0.53368	0.72586	0.06657	0.56386	0.73798	4.94E-05	4.94E-05	blue
LTB	-0.13668	-0.00667	0.03277	-0.01579	-0.0689	0.12472	0.00903	0.16286	-0.16286	0.07466	0.93099	0.67052	0.83755	0.37059	0.10409	0.90671	0.03331	0.03331	blue
HS6ST3	-0.00991	-0.12092	-0.19048	-0.12633	0.0934	-0.34282	-0.25398	0.41165	-0.41165	0.89765	0.11515	0.01258	0.09968	0.22435	4.43E-06	0.0008	2.22E-08	2.22E-08	brown
EIF4G3	-0.06116	-0.13234	-0.016	0.00615	-0.13599	0.08164	0.00272	0.25534	-0.25534	0.42684	0.08445	0.83544	0.93636	0.07615	0.28845	0.97183	0.00075	0.00075	turquoise
BLACAT1	0.08205	0.02054	0.06195	-0.01495	-0.02009	0.12977	0.18941	-0.51984	0.51984	0.286	0.78979	0.42088	0.84614	0.79425	0.0907	0.01309	3.19E-13	3.19E-13	yellow
SLC6A8	0.05858	-0.07733	-0.02439	-0.04285	0.13835	0.06805	0.03888	-0.44721	0.44721	0.44664	0.31473	0.75149	0.5779	0.07114	0.37652	0.61362	8.68E-10	8.68E-10	grey
NOP14	-0.03849	-0.06774	-0.01338	-0.05441	-0.13987	0.04481	0.05995	-0.0713	0.0713	0.61717	0.37866	0.86213	0.4797	0.06806	0.5606	0.43602	0.35405	0.35405	turquoise
DMXL1	-0.09817	-0.1652	-0.03669	-0.00662	-0.05136	0.02182	-0.09339	0.38983	-0.38983	0.20147	0.03083	0.63375	0.93148	0.50465	0.77697	0.22441	1.36E-07	1.36E-07	turquoise
AARS	0.09755	-0.02834	-0.03077	-0.01966	-0.06619	0.00411	0.06452	0.0271	-0.0271	0.20435	0.71292	0.68949	0.79859	0.38968	0.95748	0.40182	0.72496	0.72496	turquoise

VIPAS39	0.05479	-0.10236	-0.00361	0.02286	-0.03932	0.09398	0.05829	-0.01764	0.01764	0.47659	0.18277	0.96258	0.76661	0.60966	0.22144	0.44889	0.81886	0.81886	turquoise
USP45	-0.07494	-0.09606	-0.0343	0.01876	0.06045	-0.1119	-0.08912	0.278	-0.278	0.32997	0.21139	0.65605	0.80757	0.43219	0.14508	0.2464	0.00023	0.00023	turquoise
CHMP4C	0.12388	0.03698	0.05031	-0.05104	-0.0551	0.14046	0.14225	-0.47816	0.47816	0.10646	0.63107	0.51343	0.50731	0.47409	0.06689	0.06345	3.74E-11	3.74E-11	yellow
FAM83A	0.04454	0.00401	0.05408	0.07727	-0.0235	0.0647	0.18534	-0.29034	0.29034	0.563	0.95848	0.48234	0.31514	0.76026	0.40046	0.01523	0.00012	0.00012	yellow
TATDN1	-0.01901	-0.01728	0.06775	0.03643	-0.08631	0.06575	0.17967	-0.26281	0.26281	0.80512	0.82249	0.37861	0.6362	0.26167	0.39286	0.0187	0.00052	0.00052	turquoise
TMEM231	0.03444	-0.07639	-0.17349	-0.12114	0.10415	-0.1409	-0.14376	0.15171	-0.15171	0.6547	0.32067	0.02325	0.11449	0.1752	0.06603	0.06067	0.04762	0.04762	turquoise
APOBEC3(	-0.08746	0.01377	0.07354	0.09476	-0.21082	0.20271	0.2088	-0.01718	0.01718	0.25536	0.8581	0.33909	0.21766	0.00564	0.00784	0.00613	0.82355	0.82355	black
AKIRIN2	-0.02459	-0.11084	-0.05097	-0.00189	-0.08205	-0.0459	-0.01228	0.20807	-0.20807	0.74958	0.14896	0.50795	0.98045	0.28603	0.5511	0.87333	0.00632	0.00632	turquoise
TBX6	0.03846	-0.00644	0.11087	0.02153	-0.00162	0.16621	0.16884	-0.53346	0.53346	0.61746	0.93334	0.14884	0.77983	0.98318	0.0298	0.02728	5.82E-14	5.82E-14	yellow
NRP1	-0.07447	-0.15251	-0.01698	0.09094	-0.2411	0.06887	-0.05298	0.3964	-0.3964	0.33303	0.04644	0.82558	0.23685	0.00149	0.37076	0.49136	8.00E-08	8.00E-08	blue
FLT3	-0.04228	-0.10122	-0.01876	0.10734	-0.14216	0.10628	-0.04747	0.4428	-0.4428	0.58293	0.18773	0.80757	0.1623	0.06363	0.16652	0.53752	1.32E-09	1.32E-09	blue
RNF126	0.06843	0.11049	-0.07693	-0.0972	0.1968	-0.0387	0.0783	-0.42355	0.42355	0.37385	0.15026	0.31728	0.20598	0.00988	0.6153	0.3087	7.83E-09	7.83E-09	green
ACTRT3	0.01939	-0.04707	0.01597	0.01793	-0.14483	0.18264	0.20775	-0.14297	0.14297	0.80121	0.54098	0.83576	0.81595	0.05876	0.01681	0.0064	0.06213	0.06213	grey
SYNPO	-0.08593	-0.17605	-0.12808	0.03612	-0.13418	0.11201	-0.10157	0.24795	-0.24795	0.26378	0.02126	0.09504	0.63908	0.08017	0.14469	0.18621	0.00108	0.00108	blue
DDB1	-0.05192	-0.1056	-0.05987	-0.06342	-0.13682	-0.05891	0.05865	0.10893	-0.10893	0.50008	0.16926	0.43666	0.40989	0.07436	0.44403	0.44606	0.15612	0.15612	turquoise
RAD54L2	-0.04807	-0.13776	-0.05574	-0.06201	-0.13203	0.04579	-0.0507	0.26165	-0.26165	0.53238	0.07237	0.46899	0.42044	0.08517	0.55206	0.51019	0.00055	0.00055	turquoise
MTLN	0.16681	0.12003	-0.06679	-0.04573	0.14446	-0.06746	0.12068	-0.2849	0.2849	0.02921	0.11789	0.38542	0.55254	0.05941	0.38064	0.11589	0.00016	0.00016	green
ASF1B	-0.05166	0.12961	0.05837	-0.01475	-0.03926	0.10927	0.27846	-0.47808	0.47808	0.50217	0.09111	0.44825	0.84813	0.61015	0.15484	0.00023	3.77E-11	3.77E-11	pink
DPH3	-0.0499	-0.16358	-0.08208	-0.09938	-0.14019	0.01045	-0.0106	0.26093	-0.26093	0.51693	0.03253	0.28583	0.19594	0.06743	0.89205	0.89057	0.00057	0.00057	turquoise
ZC3H10	-0.04998	-0.17164	-0.19271	-0.05111	-0.03017	-0.23194	-0.12877	0.42699	-0.42699	0.51626	0.02479	0.01156	0.50673	0.6953	0.00227	0.09324	5.75E-09	5.75E-09	turquoise
FAM227B	-0.03018	-0.09346	-0.13751	-0.1377	0.13192	-0.20016	-0.23302	0.30732	-0.30732	0.69521	0.22403	0.07289	0.07249	0.08544	0.00867	0.00216	4.34E-05	4.34E-05	turquoise
8-Mar	-0.04861	-0.11937	-0.12983	-0.0486	-0.0801	-0.16503	-0.13488	0.49351	-0.49351	0.52776	0.11993	0.09056	0.5279	0.2977	0.031	0.0786	6.97E-12	6.97E-12	turquoise
MFF	0.03643	-0.06675	-0.08876	0.01687	0.06731	0.01304	0.03897	-0.00203	0.00203	0.6362	0.38569	0.24831	0.82669	0.38171	0.86561	0.61279	0.97893	0.97893	turquoise
MSRB2	0.08869	-0.01017	-0.05219	-0.04824	0.07407	-0.0704	0.05833	-0.1624	0.1624	0.24871	0.89498	0.49785	0.53093	0.33566	0.36021	0.44853	0.03382	0.03382	grey
NLRC3	-0.05177	-0.02976	-0.01259	0.02896	-0.08612	0.00126	-0.06449	0.4299	-0.4299	0.50126	0.69921	0.87021	0.70691	0.26273	0.98697	0.40203	4.41E-09	4.41E-09	blue
ALDH1B1	-0.03072	0.0318	0.06236	0.05915	-0.13152	0.07963	0.07396	0.23758	-0.23758	0.68999	0.67966	0.41782	0.44221	0.0864	0.30054	0.33635	0.00175	0.00175	salmon
FYB1	-0.09441	-0.02964	0.09364	0.06166	-0.25454	0.17939	0.10792	0.31503	-0.31503	0.21937	0.70035	0.22315	0.42304	0.00078	0.01889	0.16002	2.71E-05	2.71E-05	blue
ZNF394	0.02803	-0.15895	0.03299	0.01956	0.07803	0.08812	0.07496	-0.32291	0.32291	0.71589	0.03784	0.66841	0.79954	0.31038	0.25177	0.32988	1.65E-05	1.65E-05	grey
CTCF	0.02187	-0.09756	-0.00415	-0.01199	-0.10071	0.12782	0.01388	0.08961	-0.08961	0.77643	0.20427	0.95707	0.8763	0.18999	0.09571	0.85701	0.24379	0.24379	turquoise
XPNPEP2	-0.04423	0.00013	0.11493	0.06484	0.03013	0.09962	-0.07637	0.3001	-0.3001	0.56568	0.99867	0.13443	0.39945	0.69566	0.19482	0.3208	6.67E-05	6.67E-05	grey
HOMER3	0.01592	#######	-0.05259	0.10457	-0.07184	0.02116	0.23754	-0.07286	0.07286	0.83623	0.99952	0.4945	0.17349	0.35046	0.78352	0.00176	0.3436	0.3436	black
SLC33A1	-0.03834	-0.10472	-0.14932	0.0193	-0.0178	-0.12158	-0.03838	0.15476	-0.15476	0.61862	0.17285	0.05126	0.80214	0.81729	0.11317	0.61825	0.04326	0.04326	turquoise
HRH1	-0.00797	-0.1349	0.14633	0.1427	-0.19076	0.17923	0.26555	-0.23229	0.23229	0.91756	0.07855	0.05616	0.06262	0.01245	0.019	0.00045	0.00223	0.00223	turquoise
ERCC5	0.0447	0.0145	-0.06997	-0.1277	0.12016	-0.02845	0.0211	-0.28671	0.28671	0.56152	0.85074	0.36318	0.09601	0.11747	0.71184	0.7841	0.00014	0.00014	red
TSPAN1	0.03955	0.02624	0.12355	-0.00512	0.07195	0.21779	0.14829	-0.43735	0.43735	0.60754	0.73336	0.10741	0.94696	0.34969	0.00422	0.05292	2.22E-09	2.22E-09	yellow
IRAK4	0.04798	-0.18441	-0.07283	0.00417	0.00884	-0.11074	-0.14947	0.32488	-0.32488	0.53318	0.01575	0.34384	0.9568	0.90865	0.14931	0.05104	1.45E-05	1.45E-05	turquoise
GPR107	-0.0458	-0.15622	-0.09832	-0.02154	-0.16933	-0.0504	0.01052	0.22467	-0.22467	0.55195	0.04131	0.20077	0.77977	0.02683	0.51272	0.89134	0.00313	0.00313	turquoise
MRPS27	-0.00594	0.03466	-0.11841	-0.10878	-0.02562	-0.05016	0.02228	0.03903	-0.03903	0.93852	0.65269	0.12294	0.15672	0.7394	0.51472	0.77234	0.61226	0.61226	turquoise
POLQ	-0.09147	0.07112	0.02545	0.01789	-0.09585	0.04863	0.337	-0.34711	0.34711	0.23409	0.35528	0.74112	0.81637	0.21237	0.52759	6.57E-06	3.30E-06	3.30E-06	pink
DDX58	-0.0344	-0.00417	0.00297	0.10444	-0.23338	0.07727	0.07897	0.10165	-0.10165	0.65513	0.95679	0.96928	0.17401	0.00213	0.31513	0.30458	0.18585	0.18585	tan
SNX22	-0.10784	-0.00534	-0.08364	-0.03671	0.04047	-0.15445	-0.18967	0.24185	-0.24185	0.16034	0.94473	0.27675	0.63363	0.59917	0.04369	0.01297	0.00144	0.00144	blue
ECSCR	-0.12799	-0.01994	-0.07133	0.04658	-0.02432	-0.03854	-0.10258	0.4368	-0.4368	0.09527	0.79575	0.3539	0.54522	0.75216	0.61673	0.18183	2.33E-09	2.33E-09	blue
REX1BD	0.0634	0.02893	-0.09436	-0.09943	0.24322	-0.11199	0.0366	-0.24174	0.24174	0.41002	0.70717	0.21958	0.19571	0.00135	0.14475	0.63458	0.00145	0.00145	green
CYBC1	-0.03045	-0.08942	0.03517	-0.0218	-0.01295	-0.00407	0.07536	0.08151	-0.08151	0.69255	0.24481	0.64786	0.77719	0.86647	0.95792	0.32729	0.28921	0.28921	blue
EIF4ENIF1	-0.063	-0.08782	-0.12518	-0.03742	0.00373	-0.05935	-0.05443	0.18542	-0.18542	0.413	0.25336	0.10282	0.62707	0.96138	0.44062	0.47951	0.01518	0.01518	turquoise
TXNL4B	0.031	-0.10613	-0.07383	-0.01158	-0.05759	0.19579	0.11438	-0.04622	0.04622	0.68733	0.16712	0.33724	0.88051	0.45437	0.01028	0.13633	0.54835	0.54835	turquoise
ICA1L	-0.03032	-0.14757	-0.23239	-0.09102	0.11714	-0.30418	-0.23184	0.45804	-0.45804	0.69386	0.05409	0.00222	0.23642	0.12704	5.24E-05	0.00228	2.99E-10	2.99E-10	brown
CENPB	0.06232	0.0126	-0.14301	-0.06419	0.05525	-0.14437	0.05593	-0.09815	0.09815	0.41809	0.87004	0.06204	0.40421	0.47291	0.05958	0.46751	0.20157	0.20157	grey
ZBTB48	0.05686	-0.11528	-0.11331	-0.08512	0.25143	-0.0794	-0.07934	-0.00963	0.00963	0.46012	0.13325	0.14005	0.26835	0.00091	0.30192	0.30228	0.9005	0.9005	red
EXOC3L1	-0.09097	-0.02269	-0.1492	-0.09539	0.10163	-0.17472	-0.05592	0.19282	-0.19282	0.23668	0.76837	0.05146	0.2146	0.18593	0.02228	0.46754	0.01151	0.01151	grey
ACOX3	-0.02025	0.07736	0.06616	0.10328	-0.09537	0.10624	0.1497	-0.08744	0.08744	0.79267	0.31459	0.38994	0.17887	0.21469	0.16667	0.05067	0.25545	0.25545	grey
PPP2R3C	-0.01748	-0.05939	0.03237	-0.01653	-0.07757	0.00035	0.02454	0.29429	-0.29429	0.82047	0.44035	0.6743	0.8301	0.31322	0.99633	0.75001	9.34E-05	9.34E-05	turquoise
KAT6B	-0.01613	-0.11935	-0.0499	0.01793	-0.05013	-0.07179	-0.1169	0.35455	-0.35455	0.83414	0.11999	0.51688	0.81594	0.51492	0.35079	0.12783	1.96E-06	1.96E-06	turquoise
LAIR1	-0.09489	0.02333	0.0712	0.06764	-0.25098	0.14573	0.08058	0.38177	-0.38177	0.21698	0.762	0.35477	0.37941	0.00093	0.05719	0.29476	2.57E-07	2.57E-07	blue
COL6A2	-0.08119	-0.06138	0.03074	0.06903	-0.0794	0.02985	0.04624	0.253	-0.253	0.29112	0.42515	0.68982	0.36962	0.30195	0.69831	0.54815	0.00084	0.00084	black
SPESP1	0.25109	-0.06875	-0.01708	-0.09547	-0.03338	-0.03933	0.00684	0.2976	-0.2976	0.00092	0.37162	0.8245	0.21419	0.66475	0.60951	0.92929	7.72E-05	7.72E-05	grey
NSFL1C	0.01727	-0.06155	-0.07585	-0.06445	0.01811	-0.08567	0.10158	-0.16714	0.16714	0.82257	0.42386	0.32416	0.40234	0.81411	0.26524	0.18615	0.02889	0.02889	turquoise
STC1	-0.1119	-0.18697	-0.04998	0.03779	-0.08354	0.11921	-0.06712	0.24891	-0.24891	0.1451	0.01434	0.51618	0.6236	0.27732	0.12044	0.3831	0.00103	0.00103	turquoise
JPT2	0.0347	-0.09191	-0.00288	0.01857	-0.12186	0.01952	0.14408	-0.30844	0.30844	0.65227	0.23187	0.97016	0.80954	0.11234	0.79996	0.0601	4.06E-05	4.06E-05	turquoise
LYAR	-0.07401	0.0508	0.0598	-0.01534	-0.13968	0.09915	0.10674	-0.04103	0.04103	0.33605	0.50937	0.43715	0.84217	0.06843	0.19697	0.16465	0.59416	0.59416	turquoise
NFAM1	-0.16051	0.0009	0.03662	0.05745	-0.20708	0.10143	0.03001	0.38079	-0.38079	0.03598	0.99072	0.63446	0.45546	0.00658	0.18682	0.69677	2.78E-07	2.78E-07	blue
ADGRB1	-0.07331	-0.11489	-0.01428	0.07002	0.06818	-0.04551	-0.12327	0.01834	-0.01834	0.34067	0.13458	0.85298	0.36281	0.37559	0.55444	0.10822	0.8118	0.8118	grey
DSN1	-0.03143	0.07337	-0.0271	-0.00747	-0.05417	0.00907	0.26431	-0.20139	0.20139	0.68317	0.34023	0.72493	0.92272	0.48161	0.90631	0.00048	0.00826	0.00826	pink
EGLN2	0.12381	-0.12214	-0.1044	-0.05008	0.0438	-0.09716	0.0131	-0.16747	0.16747	0.10665	0.11152	0.17418	0.51536	0.56944	0.20614	0.86501	0.02857	0.02857	red
ATP5PO	0.09132	0.10942	-0.12889	-0.12296	0.16307	-0.06791	0.10899	-0.22354	0.22354	0.2349	0.15428	0.09294	0.1091	0.03308	0.37747	0.15591	0.00329	0.00329	purple
IL20RA	0.0419	0.0072	-0.08623	-0.0174	0.02051	0.03183	0.05483	-0.07188	0.07188	0.58639	0.92554	0.26211	0.82127	0.79002	0.67939	0.47633	0.35019	0.35019	grey
WNT5B	-0.01949	0.0532	0.02177	0.11365	-0.16676	0.05976	0.22391	-0.0218	0.0218	0.80027	0.48949	0.77742	0.13885	0.02926	0.43751	0.00324	0.77717	0.77717	grey
PPM1L	-0.03441	-0.08401	-0.05225	-0.05512	-0.06339	-0.0308	-0.00881	0.17987	-0.17987	0.65503	0.27465	0.49736	0.474	0.41013	0.68919	0.90894	0.01857	0.01857	turquoise
CRPPA	-0.05435	-0.18648	-0.01253	0.0576	-0.01046	-0.09726	-0.06191	0.22205	-0.22205	0.48014	0.0146	0.87075	0.45424	0.892	0.20568	0.42116	0.00351	0.00351	turquoise
ERAP2	-0.12533	0.04497	0.12333	0.09068	-0.01459	0.1158	0.1129	-0.00215	0.00215	0.10239	0.5592	0.10804	0.23819	0.84976	0.13149	0.1415	0.97776	0.97776	grey
MTHFR	-0.0642	-0.07572	-0.0849	-0.05293	-0.00807	-0.02107	-0.07476	0.20031	-0.20031	0.40416	0.32496	0.26956	0.49177	0.91657	0.78448	0.33113	0.00862	0.00862	turquoise
LDLRAD2	-0.08433	-0.13493	-0.00348	0.06597	-0.00791	0.08204	0.04843	0.16028	-0.16028	0.2728	0.07848	0.96401	0.39131	0.91819	0.28609	0.52932	0.03625	0.03625	blue
TUBB3	-0.03472	-0.13167	-0.01924	0.03502	-0.07934	0.0492	0.05307	-0.05938	0.05938	0.65209	0.08603	0.80276	0.64929	0.30228	0.52281	0.49062	0.44044	0.44044	grey
C1orf109	-0.06004	-0.14086	-0.02007	0.03791	-0.01959	0.03947	-0.09888	0.20736	-0.20736	0.43537	0.06612	0.79444	0.62251	0.79927	0.60822	0.19821	0.0065	0.0065	turquoise
NUP85	-0.03492	-0.10856	-0.04099	-0.02467	0.03846	-0.04338	0.1234	-0.20915	0.20915	0.65028	0.15755	0.59448	0.74872	0.61745	0.57321	0.10784	0.00605	0.00605	grey
TIMMDC1	-0.07053	0.00918	-0.06037	-0.08923	-0.03478	0.02877	0.15384	-0.13218	0.13218	0.35934	0.90515	0.43285	0.24578	0.65157	0.70878	0.04455	0.08483	0.08483	turquoise
SMPX	-0.01415	0.04686	0.05083	0.04022	-0.06891	0.11665	0.01815	0.02146	-0.02146	0.85431	0.54276	0.50911	0.60147	0.3705	0.12865	0.81373	0.7806	0.7806	grey
FAM90A1	0.09296	0.11255	-0.03104	0.04875	-0.01862	-0.01032	0.12171	0.03017	-0.03017	0.22656	0.14275	0.6869	0.52658	0.80902	0.8934	0.11279	0.69531	0.69531	grey
CP	0.03078	-0.10699	0.04198	0.05573	-0.10378	0.08419	0.12926	-0.3033	0.3033	0.68941	0.16367	0.58562	0.46911	0.17677	0.27358	0.092	5.52E-05	5.52E-05	grey
SLC37A4	0.00833	-0.09079	-0.20232	-0.0723	0.14052	-0.17598	-0.07522	0.05676	-0.05676	0.91386	0.23763	0.00796	0.34734	0.06677	0.02132	0.32816	0.46087	0.46087	grey
SMYD2	0.03891	-0.05584	-0.11545	-0.04225	-0.03752	-0.06769	0.09062	-0.17952	0.17952	0.61337	0.46818	0.13268	0.58321	0.62608	0.37903	0.23847	0.0188	0.0188	turquoise
NUP42	-0.04656	-0.1503	-0.11063	0.00172	0.11563	-0.1317	-0.07636	0.10949	-0.10949	0.54538	0.04975	0.14974	0.98217	0.13207	0.08596	0.32088	0.15399	0.15399	turquoise
CLHC1	-0.02481	-0.15567	-0.19338	-0.11355	0.14401	-0.19927	-0.04625	0.0559	-0.0559	0.74735	0.04204	0.01127	0.13921	0.06023	0.00898	0.54803	0.46774	0.46774	red
CENPA	-0.02796	0.08114	0.03603	0.03503	-0.05466	0.09754	0.3584	-0.46486	0.46486	0.7166	0.29142	0.6399	0.64921	0.47765	0.2044	1.49E-06	1.50E-10	1.50E-10	pink
KLHL25	0.06325	-0.14276	-0.05357	-0.07743	0.15631	0.06201	-0.0713	-0.22438	0.22438	0.41114	0.0625	0.48652	0.3141	0.0412	0.42043	0.3541	0.00317	0.00317	grey
CHEK1	-0.07768	0.0689	0.02383	-0.0249	-0.11058	0.03744	0.33284	-0.34514	0.34514	0.31257	0.37052	0.757	0.74653	0.14991	0.62682	8.67E-06	3.78E-06	3.78E-06	pink
TFAP2E	-0.04217	-0.16915	-0.02581	0.07233	-0.0046	0.034	0.01479	-0.09918	0.09918	0.58395	0.02699	0.73754	0.34718	0.95239	0.65889	0.84773	0.19681	0.19681	grey
EXOSC10	-0.03748	-0.05781	0.05114	0.0379	-0.10184	0.12974	0.08186	-0.04209	0.04209	0.62647	0.45262	0.50653	0.62259	0.18505	0.09077	0.28712	0.58466	0.58466	turquoise
TUBB4A	-0.01633	-0.0596	-0.07472	0.17707	-0.10705	0.02144	-0.00192	0.3223	-0.3223	0.83211	0.43876	0.33143	0.02051	0.16344	0.78074	0.98008	1.72E-05	1.72E-05	magenta
TMEM80	0.00411	-0.11329	-0.17105	-0.09927	0.19594	-0.15393	-0.04697	0.0895	-0.0895	0.95741	0.14014	0.02529	0.19641	0.01022	0.04442	0.54184	0.24437	0.24437	grey
TMEM86B	0.0526	-0.10371	-0.23126	-0.18337	0.28118	-0.34158	-0.23345	0.05342	-0.05342	0.49445	0.17705	0.00234	0.01636	0.0002	4.83E-06	0.00212	0.48774	0.48774	grey
RPIA	0.01155	0.09416	0.12511	0.00591	-0.02878	0.19858	0.16764	-0.26737	0.26737	0.88078	0.22056	0.10302	0.93887	0.70867	0.00922	0.0284	0.00041	0.00041	turquoise
GCHFR	0.09033	-0.00802	-0.06682	-0.01866	0.05625	-0.00583	0.04066	-0.26683	0.26683	0.24	0.91705	0.3852	0.80858	0.46495	0.93971	0.59748	0.00042	0.00042	grey
3-Mar	0.01967	-0.06432	-0.09987	-0.15892	0.03481	0.07453	-0.16908	0.06545	-0.06545	0.79841	0.40325	0.19374	0.03788	0.65126	0.33265	0.02706	0.39503	0.39503	grey
LY6E	0.00998	0.04332	0.07585	0.12642	-0.12241	0.14892	0.2909	-0.39177	0.39177	0.8969	0.5737	0.32411	0.09942	0.11073	0.0519	0.00011	1.17E-07	1.17E-07	tan
ECH1	0.13703	-0.12139	0.00243	0.09542	0.01624	0.03951	0.06418	-0.31065	0.31065	0.07391	0.11375	0.97486	0.21441	0.83301	0.60786	0.40432	3.54E-05	3.54E-05	grey
KAT5	-0.04584	-0.05232	-0.01984	-0.00289	-0.00079	0.01201	-0.09299	0.14024	-0.14024	0.55161	0.49676	0.79678	0.97007	0.99185	0.87612	0.22638	0.06733	0.06733	turquoise
JAK2	-0.07166	-0.09059	0.06154	0.06592	-0.06823	0.043	-0.00366	0.32559	-0.32559	0.35164	0.23864	0.42398	0.39166	0.37526	0.57653	0.96209	1.39E-05	1.39E-05	turquoise
CCDC71	-0.15268	-0.06072	-0.14377	-0.12762	0.00353	0.01323	0.01007	0.13639	-0.13639	0.04619	0.43015	0.06066	0.09623	0.96349	0.86365	0.89604	0.07528	0.07528	turquoise
PEX3	-0.11101	-0.01639	#######	0.0193	-0.11296	-0.04104	-0.0816	0.38072	-0.38072	0.14833	0.83146	0.99928	0.80213	0.1413	0.59404	0.2887	2.80E-07	2.80E-07	turquoise
SIT1	-0.10476	0.01261	0.03265	0.03935	-0.1236	0.10468	-0.01335	0.38663	-0.38663	0.17269	0.86995	0.6716	0.60938	0.10727	0.17302	0.86246	1.76E-07	1.76E-07	blue
PCLO	-0.00624	-0.13979	-0.18414	-0.10388	0.11589	-0.267	-0.2029	0.24151	-0.24151	0.93542	0.06821	0.01591	0.17633	0.13118	0.00042	0.00778	0.00146	0.00146	brown
PAK4	0.14706	-0.18438	-0.06857	0.07514	0.07391	-0.05833	0.06908	-0.36447	0.36447	0.05494	0.01577	0.37285	0.32868	0.33667	0.4486	0.36929	9.55E-07	9.55E-07	grey
TNK2	-0.02019	0.00799	0.01455	-0.01564	0.01359	0.09008	0.16609	-0.36331	0.36331	0.79326	0.91743	0.85022	0.83916	0.85996	0.24132	0.02992	1.04E-06	1.04E-06	green
CD300E	-0.21973	-0.04953	0.12636	0.05335	-0.19112	0.09581	0.03914	0.26583	-0.26583	0.00388	0.52	0.09958	0.48826	0.01228	0.21257	0.61123	0.00044	0.00044	blue
NHLRC2	-0.00548	-0.14903	-0.08192	-0.01079	-0.03091	-0.08742	-0.02902	0.13233	-0.13233	0.94331	0.05173	0.28682	0.88862	0.68819	0.25555	0.70638	0.08446	0.08446	turquoise
CLIC1	0.02283	0.11573	0.08538	0.02743	-0.01602	0.17564	0.22468	-0.46996	0.46996	0.76695	0.13174	0.26683	0.72174	0.83526	0.02157	0.00313	8.88E-11	8.88E-11	yellow
EIF3F	0.13707	0.04718	-0.10498	-0.17798	-0.0719	0.00801	0.11245	-0.15032	0.15032	0.07383	0.54004	0.17177	0.01986	0.35002	0.91722	0.14309	0.04972	0.04972	purple
N4BP2	-0.06111	-0.13157	-0.08352	-0.01969	-0.04296	-0.06594	-0.10614	0.42088	-0.42088	0.42721	0.08628	0.27748	0.79826	0.57694	0.39149	0.16708	9.93E-09	9.93E-09	turquoise
PVR	0.12674	-0.22616	-0.10557	-0.09459	0.02557	-0.1508	0.01162	-0.04734	0.04734	0.09857	0.00294	0.16937	0.21847	0.73989	0.04898	0.8801	0.53866	0.53866	grey
PLD6	0.03288	-0.07807	-0.18288	-0.02846	0.08123	-0.22946	-0.09296	0.24508	-0.24508	0.66941	0.31015	0.01666	0.71172	0.29086	0.00254	0.22652	0.00123	0.00123	brown
MTURN	-0.03881	-0.17023	-0.19262	-0.09536	0.0525	-0.21987	-0.24076	0.39835	-0.39835	0.61429	0.02602	0.0116	0.21473	0.49523	0.00386	0.00151	6.82E-08	6.82E-08	brown
GRN	-0.01367	-0.07237	0.00596	0.03368	0.03274	-0.00678	0.07638	-0.03059	0.03059	0.85917	0.34686	0.93833	0.66193	0.67078	0.9299	0.32075	0.6912	0.6912	grey
EBI3	-0.10377	-0.0117	-0.05277	-0.03842	-0.11573	0.06073	0.00118	0.39207	-0.39207	0.17681	0.87924	0.493	0.61782	0.13173	0.43006	0.98777	1.14E-07	1.14E-07	blue
DPH5	-0.07416	-0.07116	-0.12707	-0.07552	0.00584	-0.08719	-0.07865	0.32325	-0.32325	0.33507	0.35501	0.09768	0.32624	0.93958	0.2568	0.30652	1.62E-05	1.62E-05	turquoise
BTN3A2	-0.02677	0.04373	0.0561	0.03241	-0.08982	0.19296	0.07786	0.10188	-0.10188	0.72813	0.5701	0.46616	0.6739	0.24266	0.01145	0.3114	0.18486	0.18486	tan
NSUN2	-0.0157	-0.04068	0.05004	0.01294	-0.01564	-0.04958	0.15233	-0.15769	0.15769	0.83846	0.59733	0.5157	0.86655	0.83908	0.5196	0.0467	0.03941	0.03941	turquoise
FAM120A(	0.02156	-0.10413	-0.02636	0.00585	-0.07224	0.13392	-0.03308	0.0259	-0.0259	0.77952	0.17531	0.73213	0.93952	0.34775	0.08076	0.66753	0.73672	0.73672	turquoise
XPA	0.03691	-0.02331	-0.18115	-0.05916	-0.01063	-0.14998	-0.01254	0.10808	-0.10808	0.63178	0.76218	0.01773	0.4421	0.89026	0.05023	0.87064	0.15941	0.15941	grey
PPM1N	0.04931	0.0501	0.03573	-0.02729	0.0593	0.06938	0.10437	-0.41437	0.41437	0.52184	0.51522	0.64271	0.72308	0.44104	0.36724	0.1743	1.76E-08	1.76E-08	grey
MYO1C	-0.0441	-0.07622	0.0818	0.05741	-0.11293	0.19456	0.12391	-0.05257	0.05257	0.56681	0.32174	0.28752	0.45574	0.14138	0.01077	0.10639	0.49466	0.49466	turquoise
DCAF12	-0.00883	-0.13793	-0.11662	0.00785	-0.03691	-0.01928	-0.01392	0.10066	-0.10066	0.90871	0.07202	0.12875	0.91882	0.63172	0.80233	0.85658	0.19021	0.19021	turquoise
ATAD3B	-0.01759	0.05463	0.06496	0.05724	0.17379	0.07064	0.13511	-0.28361	0.28361	0.81935	0.47789	0.39859	0.45709	0.02301	0.35859	0.07808	0.00017	0.00017	green
ZNF460	0.06238	-0.0915	-0.00186	0.02023	-0.1455	0.18225	0.11082	0.11958	-0.11958	0.41763	0.23393	0.98078	0.7928	0.05759	0.01704	0.14903	0.11928	0.11928	turquoise
TM4SF4	-0.0153	-0.05657	-0.1412	-0.00082	0.18146	-0.05512	-0.2054	0.12933	-0.12933	0.84256	0.46237	0.06545	0.99149	0.01754	0.47394	0.00704	0.09181	0.09181	grey
PTCRA	-0.1987	0.0966	0.09371	0.02297	-0.15076	0.09948	-0.0367	0.26182	-0.26182	0.00918	0.2088	0.2228	0.76551	0.04905	0.19547	0.63365	0.00054	0.00054	blue
ZNF440	0.00835	-0.06617	-0.05997	-0.06127	0.03844	0.1361	0.06197	-0.16888	0.16888	0.91364	0.38983	0.43587	0.42596	0.61771	0.0759	0.42071	0.02724	0.02724	red
CDC42	-0.0908	-0.1166	0.06449	0.0489	-0.13012	0.17195	0.03322	0.0462	-0.0462	0.23755	0.12883	0.40203	0.52533	0.08984	0.02452	0.66624	0.54848	0.54848	turquoise
ZNF777	0.03962	-0.06265	-0.09362	-0.06891	0.03777	-0.04522	0.01147	0.03816	-0.03816	0.60687	0.41559	0.22325	0.37045	0.62381	0.557	0.88168	0.62022	0.62022	grey

SLC31A2	-0.13161	-0.09438	0.06926	0.12967	-0.21032	0.08453	0.11572	0.34184	-0.34184	0.08619	0.2195	0.36808	0.09096	0.00576	0.27169	0.13175	4.74E-06	4.74E-06	blue
EIF4EBP3	0.05761	0.12772	0.01	0.04847	-0.01854	0.22984	0.1773	-0.30166	0.30166	0.45417	0.09596	0.89668	0.52899	0.80976	0.00249	0.02034	6.08E-05	6.08E-05	grey
SLC25A24	-0.11218	-0.0755	0.09395	0.0872	-0.1353	0.20776	0.10769	-0.07723	0.07723	0.14406	0.32636	0.2216	0.25674	0.07766	0.0064	0.16091	0.31538	0.31538	turquoise
TMEM169	-0.02001	-0.07852	-0.27896	-0.09781	-0.06584	-0.22222	0.02962	0.2058	-0.2058	0.79503	0.30736	0.00022	0.20312	0.39225	0.00349	0.70051	0.00693	0.00693	grey
PTER	0.0377	0.04132	0.05461	0.08418	-0.01852	0.16078	0.07013	-0.12984	0.12984	0.62442	0.59159	0.47808	0.27365	0.80997	0.03566	0.36206	0.09053	0.09053	turquoise
GAB2	-0.01502	-0.06601	0.04736	-0.01592	-0.11061	0.09234	-0.07955	0.35976	-0.35976	0.84541	0.39097	0.53844	0.83624	0.14981	0.22967	0.301	1.35E-06	1.35E-06	turquoise
NXT1	0.01587	-0.03312	-0.03722	0.00572	-0.07902	0.01736	0.07674	-0.12505	0.12505	0.83674	0.66716	0.62891	0.94077	0.30428	0.82165	0.31846	0.10317	0.10317	grey
SLC35B4	-0.0561	-0.12616	0.03054	0.02963	-0.14391	0.04111	0.02773	0.36082	-0.36082	0.46609	0.10012	0.69176	0.70045	0.0604	0.59348	0.71882	1.25E-06	1.25E-06	turquoise
WDR3	-0.11221	-0.04312	0.06854	0.07578	-0.13128	0.16825	0.08543	0.00503	-0.00503	0.14395	0.57552	0.37305	0.32455	0.08698	0.02783	0.2666	0.94792	0.94792	turquoise
CRADD	0.0125	-0.10698	-0.10302	-0.11263	0.1628	-0.17304	-0.20475	0.33389	-0.33389	0.87106	0.1637	0.17997	0.14245	0.03338	0.02361	0.00722	8.09E-06	8.09E-06	turquoise
ZHX2	-0.04886	-0.18073	0.02705	0.07822	-0.22147	0.1676	-0.02548	0.19328	-0.19328	0.52564	0.01801	0.72545	0.30921	0.0036	0.02845	0.74082	0.01131	0.01131	turquoise
PRDM11	-0.03996	-0.1506	-0.16491	-0.06988	-0.06443	-0.04924	-0.10259	0.33717	-0.33717	0.60383	0.04928	0.03113	0.36375	0.40244	0.52245	0.1818	6.50E-06	6.50E-06	turquoise
GNA11	0.01055	-0.12388	-0.15285	-0.04771	0.17953	-0.09417	-0.01635	-0.05848	0.05848	0.89106	0.10647	0.04595	0.53552	0.01879	0.22052	0.83188	0.44739	0.44739	green
BTAF1	-0.02754	-0.18954	0.01906	0.04083	0.02767	0.00401	-0.04846	0.13151	-0.13151	0.72071	0.01303	0.80456	0.59596	0.71939	0.95853	0.52906	0.08643	0.08643	turquoise
CCDC136	-0.04839	-0.09113	-0.09301	-0.01669	0.0829	-0.17761	-0.15415	0.24188	-0.24188	0.52967	0.23585	0.22629	0.82847	0.28105	0.02013	0.04411	0.00144	0.00144	grey
ZNF324B	-0.00354	-0.12942	-0.21111	-0.09586	0.08683	-0.15998	-0.05988	0.20851	-0.20851	0.9634	0.09158	0.00558	0.21233	0.2588	0.0366	0.43661	0.0062	0.0062	turquoise
TNKS1BP1	0.00784	-0.09451	0.02573	0.00613	-0.12828	0.1982	0.18243	-0.22107	0.22107	0.91889	0.21887	0.73834	0.93654	0.0945	0.00936	0.01694	0.00366	0.00366	turquoise
L3MBTL4	-0.05708	-0.03988	-0.13418	0.04167	-0.02449	0.01701	-0.0468	0.2368	-0.2368	0.45837	0.60451	0.08018	0.58844	0.75048	0.82523	0.54327	0.00182	0.00182	grey
SEPTIN5	-0.03605	-0.14189	-0.20264	-0.05975	0.07189	-0.32732	-0.22047	0.52861	-0.52861	0.63967	0.06414	0.00786	0.43759	0.35009	1.24E-05	0.00376	1.07E-13	1.07E-13	brown
C3	-0.03471	0.01193	0.00659	0.10326	-0.21225	0.16316	0.16291	0.20571	-0.20571	0.65224	0.87692	0.93186	0.17894	0.00532	0.03299	0.03326	0.00695	0.00695	blue
TCOF1	0.06713	-0.12307	-0.07019	-0.06247	-0.02108	-0.05971	0.07836	-0.10812	0.10812	0.38299	0.1088	0.36162	0.41693	0.78431	0.43787	0.30832	0.15923	0.15923	turquoise
TRIM22	-0.0814	-0.03048	0.0743	0.09686	-0.20172	0.16728	0.10303	0.22969	-0.22969	0.28985	0.69226	0.33412	0.20758	0.00815	0.02876	0.17992	0.00251	0.00251	blue
TMEM26	-0.061	-0.03521	0.05096	0.04402	-0.21052	0.04565	0.11508	0.2114	-0.2114	0.42803	0.64755	0.50804	0.56755	0.00571	0.55327	0.13392	0.00551	0.00551	black
THAP6	-0.06047	-0.12865	-0.09966	-0.09987	0.03195	-0.06561	-0.03261	0.13533	-0.13533	0.4321	0.09354	0.19467	0.1937	0.67823	0.39386	0.67195	0.0776	0.0776	turquoise
DHCR7	0.07663	0.05966	-0.02441	-0.07821	0.0636	0.10855	0.09938	-0.37661	0.37661	0.31916	0.43827	0.75134	0.30929	0.40859	0.15759	0.19592	3.84E-07	3.84E-07	grey
DEF8	-0.07746	-0.04318	-0.10636	-0.0598	0.04147	-0.13254	-0.07902	0.20231	-0.20231	0.31395	0.57499	0.1662	0.43723	0.59021	0.08397	0.30427	0.00796	0.00796	grey
NUDT12	-0.0469	-0.02804	-0.07723	-0.04513	0.16621	-0.12079	-0.01166	-0.01126	0.01126	0.54247	0.71577	0.3154	0.5578	0.0298	0.11557	0.87973	0.88383	0.88383	turquoise
MYL12B	-0.00792	-0.14395	0.0225	-0.02806	-0.01968	0.15303	0.15051	-0.37345	0.37345	0.91812	0.06034	0.77022	0.71563	0.79837	0.04569	0.04942	4.88E-07	4.88E-07	yellow
GLIS1	-0.13303	-0.0412	0.02706	0.02664	-0.03503	0.01252	0.13225	0.1194	-0.1194	0.08282	0.5926	0.72533	0.72947	0.64918	0.87088	0.08466	0.11984	0.11984	black
RAB3A	0.07985	-0.11201	-0.24614	-0.12033	0.20676	-0.34331	-0.19518	0.22457	-0.22457	0.29916	0.14467	0.00117	0.11695	0.00666	4.29E-06	0.01052	0.00315	0.00315	brown
GDA	0.07672	-0.1529	-0.08631	0.01283	-0.00404	-0.00292	0.05608	-0.00395	0.00395	0.31862	0.04588	0.26165	0.86773	0.95818	0.96976	0.46631	0.95912	0.95912	grey
USP34	-0.0706	-0.16143	-0.00725	0.04326	-0.16138	0.07126	-0.00616	0.30749	-0.30749	0.35886	0.03492	0.92506	0.57426	0.03497	0.35437	0.93625	4.29E-05	4.29E-05	turquoise
PLAAT4	0.02158	-0.06015	0.08841	0.11995	-0.13407	0.13902	0.2482	-0.20521	0.20521	0.77932	0.43453	0.25017	0.11813	0.08043	0.06978	0.00106	0.00709	0.00709	tan
LIN54	-0.06235	-0.12249	-0.12522	-0.027	-0.03865	-0.12426	-0.02067	0.19029	-0.19029	0.41789	0.11047	0.1027	0.7259	0.61573	0.10539	0.7884	0.01267	0.01267	turquoise
TAF1C	-0.04577	-0.06166	-0.03141	-0.0145	0.18216	-0.02789	-0.00548	-0.08427	0.08427	0.55222	0.42306	0.68341	0.85067	0.0171	0.71731	0.94333	0.27313	0.27313	red
RHOH	-0.09068	0.01139	0.07911	0.03918	-0.1273	0.10702	-0.0669	0.364	-0.364	0.23821	0.88251	0.30372	0.61089	0.09708	0.16355	0.38461	9.89E-07	9.89E-07	blue
IGF2BP2	0.07737	-0.08133	0.14614	0.06987	-0.11727	0.26883	0.35819	-0.43934	0.43934	0.31448	0.29027	0.05649	0.36382	0.12663	0.00038	1.51E-06	1.84E-09	1.84E-09	yellow
GABARAP	-0.0991	-0.141	-0.13362	-0.08189	-0.00507	-0.20357	-0.15392	0.43614	-0.43614	0.19718	0.06585	0.08145	0.287	0.94751	0.00757	0.04443	2.48E-09	2.48E-09	brown
LMCD1	-0.08948	-0.09257	-0.01622	0.05753	-0.17853	0.02825	-0.071	0.39485	-0.39485	0.24448	0.2285	0.83318	0.45481	0.01947	0.7138	0.3561	9.08E-08	9.08E-08	blue
ACBD3	0.00028	-0.07494	-0.01643	-0.02488	-0.06687	0.12002	0.09199	0.00633	-0.00633	0.99705	0.32996	0.83107	0.7467	0.38487	0.1179	0.23147	0.93447	0.93447	turquoise
MZT1	0.03849	0.02123	-0.04249	-0.0712	-0.01045	-0.10817	0.03427	-0.14589	0.14589	0.61717	0.78287	0.5811	0.35473	0.89207	0.15905	0.65633	0.05692	0.05692	turquoise
RELT	-0.1	-0.08318	0.12293	0.10163	-0.07606	0.16913	0.06795	0.1216	-0.1216	0.19314	0.2794	0.10919	0.18595	0.32277	0.02701	0.37718	0.11312	0.11312	blue
PLA2G10	0.05465	0.09788	0.13282	0.0377	0.04996	0.26137	0.07766	-0.39325	0.39325	0.47778	0.20278	0.0833	0.62449	0.51637	0.00055	0.31269	1.03E-07	1.03E-07	yellow
USP21	0.05054	-0.06317	-0.06403	-0.07865	0.13062	-0.02753	0.00086	-0.19438	0.19438	0.51156	0.41179	0.40537	0.30653	0.0886	0.72081	0.99114	0.01085	0.01085	greenyellow
MAP1LC3I	0.00899	-0.17085	-0.05207	-0.03735	-0.11423	0.01026	-0.00941	0.07947	-0.07947	0.90706	0.02547	0.49883	0.62763	0.13683	0.89404	0.90275	0.3015	0.3015	turquoise
DCTN1	0.03624	-0.03751	-0.04562	-0.02537	-0.1059	0.02467	0.05102	0.144	-0.144	0.63791	0.62618	0.55356	0.74183	0.16804	0.74873	0.5075	0.06023	0.06023	turquoise
ADH1A	0.04296	-0.07834	-0.08405	-0.05024	0.07289	-0.01023	-0.08194	0.17301	-0.17301	0.5769	0.30844	0.27441	0.51403	0.34342	0.8944	0.28671	0.02364	0.02364	grey
USF1	0.07644	0.03716	-0.03818	-0.06796	0.05177	0.07671	0.17961	-0.28197	0.28197	0.32037	0.62939	0.62009	0.37716	0.5013	0.31863	0.01874	0.00019	0.00019	greenyellow
CLSTN2	-0.05603	-0.14773	-0.04217	0.02254	-0.07309	-0.08838	-0.15425	0.61857	-0.61857	0.46668	0.05383	0.58392	0.76982	0.34213	0.25037	0.04397	1.96E-19	1.96E-19	turquoise
ZNF540	-0.008	-0.14467	-0.23034	-0.08686	0.10926	-0.23759	-0.24121	0.47348	-0.47348	0.91731	0.05904	0.00244	0.2586	0.15488	0.00175	0.00148	6.14E-11	6.14E-11	brown
CCDC93	0.00532	-0.17888	-0.03675	0.01975	-0.05209	-0.04185	-0.02328	0.16512	-0.16512	0.94489	0.01924	0.6332	0.79766	0.49865	0.5868	0.76243	0.03091	0.03091	turquoise
MINAR1	-0.10883	-0.14367	-0.0459	0.10073	-0.2326	-0.0006	0.03583	0.39117	-0.39117	0.1565	0.06084	0.55109	0.18988	0.0022	0.9938	0.64178	1.22E-07	1.22E-07	blue
ERI2	-0.06407	-0.01524	0.00751	0.00794	-0.04101	0.13653	0.20468	-0.15097	0.15097	0.40514	0.84314	0.92233	0.91788	0.59435	0.07497	0.00725	0.04873	0.04873	turquoise
TMEM160	0.15893	0.0221	-0.1152	-0.14398	0.13319	-0.12668	0.08797	-0.2804	0.2804	0.03787	0.77415	0.13352	0.06028	0.08246	0.09872	0.25256	0.0002	0.0002	green
AC005041	-0.01094	-0.12755	-0.27054	-0.17907	0.17016	-0.30008	-0.1558	-0.002	0.002	0.88704	0.09642	0.00035	0.01911	0.02607	6.68E-05	0.04187	0.97924	0.97924	grey
NDUFB5	-0.0064	0.02791	-0.09002	-0.07733	-0.03741	0.03107	0.10535	-0.10462	0.10462	0.93384	0.71709	0.24161	0.31477	0.6271	0.68667	0.17028	0.17325	0.17325	turquoise
TMEM259	0.04073	-0.01801	-0.15165	-0.11404	0.25423	-0.17316	-0.02831	-0.22485	0.22485	0.59681	0.81518	0.0477	0.1375	0.00079	0.02352	0.71319	0.00311	0.00311	green
CALM2	0.02019	-0.09664	-0.01809	-0.06089	-0.03524	0.08251	0.11964	-0.18416	0.18416	0.79325	0.20863	0.8143	0.42889	0.64723	0.28331	0.11908	0.0159	0.0159	turquoise
INTS7	0.05853	0.00585	-0.05013	-0.00709	-0.01606	-0.06783	0.10018	-0.01138	0.01138	0.447	0.9395	0.51496	0.92668	0.83488	0.37807	0.19235	0.88261	0.88261	turquoise
CIAPIN1	#######	-0.01582	-0.02801	-0.06039	-0.08891	0.05879	0.1499	-0.13239	0.13239	0.99932	0.83725	0.71609	0.43264	0.24751	0.44499	0.05036	0.08432	0.08432	turquoise
SAFB2	0.02535	-0.1491	-0.2195	-0.13194	0.21396	-0.21761	-0.16934	0.07247	-0.07247	0.74209	0.05162	0.00392	0.0854	0.00495	0.00425	0.02682	0.3462	0.3462	red
F3	0.0056	-0.12318	0.0982	0.06056	-0.04387	0.14337	0.16039	-0.11379	0.11379	0.94206	0.10848	0.20134	0.43141	0.56882	0.06138	0.03612	0.13837	0.13837	turquoise
FAM3B	-0.0107	-0.02185	-0.01234	0.01227	0.10369	0.13225	-0.15585	-0.07636	0.07636	0.88953	0.77671	0.87277	0.87345	0.17713	0.08465	0.0418	0.32088	0.32088	grey
ARRDC3	-0.02881	-0.17246	0.02566	0.10817	-0.03021	0.06179	0.00153	0.12231	-0.12231	0.70832	0.0241	0.739	0.15907	0.69489	0.42209	0.9841	0.11102	0.11102	turquoise
RPL27	0.10545	0.15048	0.00616	-0.00994	-0.06412	0.05436	0.14071	-0.1601	0.1601	0.16985	0.04947	0.93628	0.89728	0.40477	0.4801	0.06641	0.03647	0.03647	purple
UCHL1	-0.04225	-0.10521	-0.11643	0.03791	0.00784	-0.23114	-0.16485	0.5246	-0.5246	0.58318	0.17084	0.12938	0.62253	0.91894	0.00235	0.03119	1.78E-13	1.78E-13	brown
AADAC	-0.08732	0.05853	0.08942	0.1223	-0.00771	0.22167	0.1351	-0.1865	0.1865	0.25613	0.447	0.24479	0.11103	0.92026	0.00357	0.07811	0.01459	0.01459	grey
MRPL40	-0.07022	0.05091	-0.20634	-0.09992	0.05955	-0.22388	-0.00944	-0.12074	0.12074	0.36143	0.50845	0.00678	0.19351	0.43908	0.00324	0.90248	0.11571	0.11571	grey
SH3TC1	-0.07659	-0.1208	-0.15003	-0.08579	0.03822	-0.11456	-0.07379	-0.04125	0.04125	0.31944	0.11552	0.05017	0.26454	0.61964	0.13569	0.33746	0.59215	0.59215	grey
HDAC11	0.06905	-0.03908	-0.1385	-0.05629	0.07804	-0.04656	-0.00697	-0.3143	0.3143	0.36953	0.61182	0.07084	0.46462	0.3103	0.54538	0.92794	2.83E-05	2.83E-05	grey
SH2D7	0.01033	-0.05528	-0.02927	0.1358	0.13365	0.15377	0.09584	0.08135	-0.08135	0.89336	0.47266	0.70389	0.07657	0.08138	0.04464	0.21242	0.29019	0.29019	grey
STX11	-0.12397	-0.1028	0.08202	0.10014	-0.21274	0.19998	0.02456	0.33455	-0.33455	0.10621	0.18089	0.2862	0.19252	0.00521	0.00873	0.74983	7.74E-06	7.74E-06	blue
CPD	0.01749	-0.19688	0.0101	-0.02789	0.07104	0.07511	-0.00173	-0.01378	0.01378	0.82039	0.00985	0.89567	0.71725	0.35581	0.32889	0.98208	0.85799	0.85799	turquoise
CDV3	-0.01474	-0.04058	0.01952	0.0386	-0.22747	0.21477	0.12565	0.13236	-0.13236	0.84829	0.59817	0.79994	0.61622	0.00277	0.00479	0.10152	0.08439	0.08439	turquoise
THRA	0.03069	-0.09879	-0.16458	0.0035	0.14929	-0.14486	-0.17216	0.22745	-0.22745	0.69028	0.19862	0.03147	0.96377	0.05132	0.05871	0.02434	0.00277	0.00277	turquoise
C2orf72	0.08355	-0.04919	-0.11862	0.01638	0.2631	-0.0007	-0.08116	-0.0088	0.0088	0.27726	0.52291	0.12228	0.83159	0.00051	0.99271	0.29128	0.90905	0.90905	grey
SLC4A11	0.01924	0.06535	-0.01553	-0.06787	0.14658	-0.04482	0.14179	-0.40208	0.40208	0.80275	0.39579	0.84026	0.3778	0.05573	0.56046	0.06434	5.01E-08	5.01E-08	grey
BAZ2B	0.03237	-0.12853	0.00684	0.0587	-0.04268	0.11324	0.02289	0.14322	-0.14322	0.67424	0.09386	0.92923	0.44571	0.57938	0.14029	0.76634	0.06166	0.06166	turquoise
MAP6D1	-0.14377	-0.09639	-0.14689	-0.01222	-0.01862	-0.12136	0.01929	0.20036	-0.20036	0.06066	0.2098	0.05522	0.87399	0.80898	0.11384	0.80228	0.0086	0.0086	grey
NBPF15	-0.0682	-0.15491	-0.02374	-0.01206	-0.13108	0.06482	0.03836	0.08218	-0.08218	0.37545	0.04306	0.7579	0.87561	0.08748	0.39963	0.6184	0.28527	0.28527	turquoise
PYM1	0.05987	-0.03843	-0.13762	-0.04958	0.12814	-0.12659	0.00269	-0.29636	0.29636	0.43662	0.61775	0.07266	0.51958	0.09486	0.09897	0.97214	8.29E-05	8.29E-05	grey
PIGK	-0.09655	-0.15875	-0.05619	0.00573	-0.03881	-0.08885	-0.14536	0.38357	-0.38357	0.20901	0.03809	0.4654	0.94071	0.61426	0.24781	0.05784	2.24E-07	2.24E-07	turquoise
LAMC1	-0.06717	-0.14782	0.03354	0.11546	-0.21167	0.17123	0.13001	0.19295	-0.19295	0.38271	0.05368	0.66316	0.13264	0.00545	0.02514	0.09011	0.01146	0.01146	turquoise
TULP4	-0.00984	-0.15387	-0.062	0.01056	-0.09179	-0.09035	-0.09086	0.3285	-0.3285	0.89839	0.0445	0.42047	0.89099	0.23244	0.23991	0.23728	1.15E-05	1.15E-05	turquoise
RNF183	0.00787	0.02101	0.04871	0.01501	0.15726	0.15057	0.01694	-0.47215	0.47215	0.91864	0.78508	0.52693	0.84549	0.03996	0.04933	0.8259	7.06E-11	7.06E-11	yellow
ANKRD22	-0.0662	0.04042	0.17428	0.08855	-0.05568	0.30997	0.23909	-0.40453	0.40453	0.38965	0.59964	0.02262	0.24943	0.46945	3.70E-05	0.00164	4.08E-08	4.08E-08	yellow
GGT5	-0.06613	-0.07976	0.02867	0.02132	-0.20285	0.093	-0.00697	0.25375	-0.25375	0.39011	0.29971	0.70974	0.78198	0.00779	0.22636	0.92795	0.00081	0.00081	black
OPA1	-0.04518	-0.01727	-0.0472	-0.00672	-0.12488	0.07947	0.07074	0.03943	-0.03943	0.5574	0.82256	0.53987	0.93044	0.10366	0.30153	0.35789	0.60859	0.60859	turquoise
YWHAE	-0.05253	-0.12277	-0.1177	-0.13072	0.00747	-0.18065	0.00243	0.08182	-0.08182	0.49506	0.10965	0.12522	0.08835	0.92273	0.01805	0.97479	0.28737	0.28737	turquoise
ZMYM6	-0.07373	-0.13011	-0.00108	0.02046	-0.0433	-0.03474	-0.04858	0.06658	-0.06658	0.33784	0.08986	0.98885	0.7905	0.57386	0.65193	0.52801	0.38695	0.38695	turquoise
RPL13	0.16083	0.13939	-0.02248	-0.10212	-0.04705	0.04618	0.14146	-0.35931	0.35931	0.03561	0.06903	0.77044	0.18383	0.54114	0.54865	0.06496	1.39E-06	1.39E-06	purple
LRRC14	0.00134	-0.06728	-0.14942	-0.11896	0.07431	-0.21414	-0.00014	-0.10882	0.10882	0.98616	0.38194	0.05111	0.12123	0.33404	0.00492	0.99852	0.15656	0.15656	green
C18orf21	-0.12331	-0.06086	-0.12687	0.02801	0.00643	-0.14051	0.01152	0.11089	-0.11089	0.10809	0.4291	0.09821	0.71607	0.93345	0.06679	0.88112	0.14876	0.14876	grey
POLG2	-0.0316	-0.13945	-0.01498	-0.02873	0.08737	-0.03149	0.06726	-0.10654	0.10654	0.6816	0.0689	0.84582	0.70914	0.25583	0.68264	0.38211	0.16547	0.16547	red
ADNP	-0.00729	-0.15426	-0.04395	0.02967	-0.07094	-0.03652	0.02817	0.07909	-0.07909	0.92463	0.04396	0.56814	0.7001	0.35652	0.63536	0.71459	0.30384	0.30384	turquoise
FLAD1	0.07226	0.06282	-0.05077	-0.12909	0.03983	-0.00947	0.17403	-0.3643	0.3643	0.34762	0.41433	0.5096	0.09243	0.60497	0.90219	0.02282	9.66E-07	9.66E-07	greenyellow
CIAO2A	-0.0836	-0.02255	-0.10971	-0.05489	0.06563	-0.06196	-0.03274	-0.07647	0.07647	0.277	0.76975	0.15317	0.4758	0.39376	0.42079	0.67072	0.32017	0.32017	turquoise
CAMK1D	-0.14692	-0.10195	-0.0777	0.0359	-0.17484	0.05103	-0.10565	0.47698	-0.47698	0.05516	0.18456	0.31244	0.64115	0.02218	0.50739	0.16904	4.24E-11	4.24E-11	blue
JDP2	0.02544	-0.09588	-0.06173	0.00788	-0.04468	0.07354	-0.03762	0.22902	-0.22902	0.74122	0.21225	0.4225	0.91849	0.56177	0.33915	0.62518	0.00259	0.00259	blue
KLC2	0.0041	-0.18972	-0.1777	-0.1015	0.14821	-0.29352	-0.14996	0.07214	-0.07214	0.95755	0.01294	0.02006	0.1865	0.05304	9.77E-05	0.05027	0.34844	0.34844	grey
RALGAPB	0.00172	-0.16288	-0.02232	0.02411	-0.03422	-0.01323	0.0106	0.10032	-0.10032	0.98218	0.03329	0.77199	0.75431	0.65684	0.86362	0.89055	0.19172	0.19172	turquoise
SFTPA2	-0.05536	0.00315	0.01026	0.02341	0.05598	0.06333	-0.01339	-0.22777	0.22777	0.47204	0.96735	0.89407	0.76121	0.46712	0.41058	0.86201	0.00273	0.00273	grey
KRT4	0.15321	0.04873	0.02184	0.02958	0.05906	-0.01066	0.16413	-0.13113	0.13113	0.04544	0.52676	0.77677	0.70092	0.44286	0.88998	0.03194	0.08734	0.08734	grey
THAP10	-0.04257	-0.16682	-0.22178	-0.04443	0.13966	-0.21443	-0.07477	0.10195	-0.10195	0.58035	0.0292	0.00355	0.56395	0.06849	0.00486	0.3311	0.18454	0.18454	turquoise
CRYZ	-0.07989	-0.10978	-0.05932	0.03788	0.02353	0.10874	-0.07338	-0.03855	0.03855	0.29897	0.1529	0.4409	0.62278	0.76	0.15684	0.34019	0.61661	0.61661	turquoise
EXOC7	-0.08421	-0.1892	0.00343	0.00482	-0.0257	0.00493	-0.00768	0.1829	-0.1829	0.27352	0.0132	0.96452	0.95009	0.73867	0.94901	0.92054	0.01664	0.01664	turquoise
DHRS2	0.02787	-0.08068	-0.12186	-0.06162	0.01509	-0.23512	-0.12414	0.26384	-0.26384	0.71751	0.29416	0.11235	0.42337	0.84474	0.00196	0.10572	0.00049	0.00049	brown
BPNT1	0.01159	-0.02397	0.01021	-0.03228	0.02244	0.09409	0.17821	-0.3201	0.3201	0.88037	0.75567	0.89456	0.67511	0.77081	0.22092	0.0197	1.97E-05	1.97E-05	turquoise
ANKRD11	-0.02306	-0.13036	-0.06458	-0.01992	-0.0579	0.03538	0.04317	0.12269	-0.12269	0.76465	0.08924	0.40137	0.79595	0.45194	0.64593	0.57506	0.1099	0.1099	turquoise
SERTAD4	-0.1403	-0.14839	-0.00141	0.03441	-0.05503	0.16759	0.10873	-0.0492	0.0492	0.06722	0.05274	0.98537	0.65501	0.47472	0.02845	0.15689	0.52277	0.52277	turquoise
TCHP	0.05452	-0.13053	-0.04824	-0.03769	0.14845	-0.06316	-0.04574	0.26035	-0.26035	0.47883	0.08882	0.53092	0.62453	0.05265	0.41181	0.55244	0.00058	0.00058	turquoise
WWC3	-0.12843	-0.18269	-0.06108	0.01179	-0.17435	0.02979	-0.00537	0.32362	-0.32362	0.09411	0.01678	0.42739	0.87834	0.02256	0.69895	0.9444	1.58E-05	1.58E-05	blue
TCTEX1D2	-0.00533	-0.09219	-0.16605	-0.03853	-0.00721	-0.16185	0.01143	-0.0592	0.0592	0.94483	0.23045	0.02997	0.61684	0.92548	0.03444	0.88201	0.44182	0.44182	grey
HOXB13	0.09715	-0.03739	0.04736	0.01098	0.03712	0.05546	0.09353	-0.17971	0.17971	0.20619	0.62734	0.53846	0.88667	0.62982	0.47127	0.22369	0.01868	0.01868	grey
RNF181	0.14313	0.01667	0.02175	0.01898	-0.09949	0.10246	0.24396	-0.45577	0.45577	0.06183	0.82866	0.77767	0.80533	0.19545	0.18236	0.0013	3.75E-10	3.75E-10	yellow
MLLT10	0.0019	-0.14832	-0.04406	0.02922	-0.01311	-0.00716	-0.0277	0.22725	-0.22725	0.98032	0.05286	0.56719	0.70439	0.86483	0.92595	0.71914	0.0028	0.0028	turquoise
GPM6A	-0.03	0.04412	-0.15232	0.0183	-0.06954	-0.03138	0.02309	0.38489	-0.38489	0.69687	0.56663	0.04672	0.81217	0.36613	0.68369	0.7644	2.02E-07	2.02E-07	brown
CACNA1H	0.02728	-0.14713	-0.22508	-0.11332	0.17144	-0.20209	-0.19158	0.35965	-0.35965	0.72316	0.05482	0.00308	0.14	0.02496	0.00803	0.01207	1.36E-06	1.36E-06	brown
FMR1	-0.00879	-0.2067	-0.02887	0.02483	-0.07672	0.0368	0.03347	0.10056	-0.10056	0.90914	0.00668	0.70776	0.74717	0.3186	0.63275	0.66387	0.19066	0.19066	turquoise
TTLL3	0.00182	-0.1259	-0.04962	-0.05872	0.16328	0.02119	-0.00625	-0.07128	0.07128	0.9812	0.10085	0.51927	0.44551	0.03285	0.78326	0.93532	0.35422	0.35422	red
HEPH	-0.04673	-0.0204	0.14779	0.04605	0.05156	0.22993	0.12573	-0.03374	0.03374	0.54388	0.79111	0.05373	0.54982	0.503	0.00248	0.1013	0.66132	0.66132	turquoise
PCBD2	0.04238	-0.09907	-0.14237	-0.04499	0.13139	-0.15383	-0.0292	-0.13702	0.13702	0.58209	0.19734	0.06323	0.559	0.08672	0.04456	0.70461	0.07393	0.07393	turquoise
SLC39A13	-0.03557	-0.11348	-0.04752	-0.00074	-0.01998	-0.05373	0.01916	0.00504	-0.00504	0.64422	0.13946	0.53708	0.99232	0.79532	0.48518	0.80361	0.94787	0.94787	black
NUDT1	0.01993	0.07624	0.0097	-0.00948	-0.02214	0.06842	0.2134	-0.3454	0.3454	0.79588	0.32161	0.8998	0.90205	0.77373	0.3739	0.00507	3.71E-06	3.71E-06	pink
ISY1	0.01394	0.02478	-0.07787	-0.04008	-0.05545	0.07761	0.17036	-0.2397	0.2397	0.8564	0.7477	0.31139	0.60273	0.47133	0.31297	0.0259	0.00159	0.00159	grey
TPM4	-0.04221	-0.05128	0.07118	0.08223	-0.15931	0.20456	0.15357	-0.05088	0.05088	0.58359	0.50538	0.3549	0.28499	0.03741	0.00728	0.04492	0.50866	0.50866	black
MPZL2	-0.04664	-0.07603	0.04871	0.02765	-0.14647	0.19579	0.22985	-0.29775	0.29775	0.54471	0.32298	0.52697	0.71961	0.05592	0.01028	0.00249	7.65E-05	7.65E-05	turquoise
SPATA18	-0.02861	-0.03881	-0.02497	0.02533	0.07488	-0.00256	-0.05059	0.17941	-0.17941	0.71034	0.61427	0.74581	0.74231	0.33038	0.97352	0.51114	0.01888	0.01888	grey
ECSIT	0.10564	0.13086	-0.24577	-0.09279	0.18106	-0.18991	-0.04596	-0.11965	0.11965	0.16909	0.08801	0.00119	0.2274	0.01779	0.01285	0.55058	0.11905	0.11905	green

SDE2	-0.04414	-0.10888	-0.01741	0.01128	-0.0614	0.11219	0.02365	0.16431	-0.16431	0.56651	0.1563	0.82116	0.88357	0.42497	0.14404	0.75878	0.03176	0.03176	turquoise
MCTP2	0.01785	-0.1092	-0.05875	-0.0384	0.05786	0.0348	-0.04067	0.14415	-0.14415	0.81674	0.1551	0.44533	0.61799	0.4522	0.65139	0.59744	0.05997	0.05997	turquoise
COPZ2	-0.05604	0.00101	0.00287	0.09815	-0.14697	0.11432	0.08373	0.29636	-0.29636	0.4666	0.98955	0.97032	0.20155	0.05508	0.13652	0.27623	8.29E-05	8.29E-05	black
LY6G6C	0.0774	0.14741	0.03975	0.10225	0.01402	0.13918	0.1917	-0.35682	0.35682	0.3143	0.05435	0.60574	0.18325	0.85557	0.06945	0.01201	1.66E-06	1.66E-06	grey
HPX	-0.02142	-0.0898	-0.0833	-0.04057	0.0981	-0.16648	-0.16438	-0.05258	0.05258	0.78095	0.24277	0.27871	0.59829	0.20178	0.02953	0.03168	0.49457	0.49457	grey
NEURL4	-0.04057	-0.16057	-0.13129	-0.09317	0.14011	-0.24676	-0.07904	0.28127	-0.28127	0.59828	0.03591	0.08696	0.2255	0.06759	0.00114	0.30412	0.00019	0.00019	brown
AURKAIP1	-0.00592	0.02389	-0.07015	-0.02448	0.10526	-0.05682	0.12487	-0.3701	0.3701	0.9388	0.7564	0.36189	0.75062	0.17063	0.46044	0.10368	6.29E-07	6.29E-07	green
FAM160A:	0.04422	-0.03522	-0.05156	-0.10243	0.18769	0.12644	0.03235	-0.27878	0.27878	0.56578	0.6474	0.50302	0.18251	0.01396	0.09937	0.6745	0.00022	0.00022	yellow
GRHL1	0.04634	-0.20127	0.12241	0.0495	-0.0006	0.08836	0.16163	-0.3161	0.3161	0.54723	0.0083	0.11071	0.52028	0.99382	0.25047	0.03469	2.53E-05	2.53E-05	grey
CDC6	-0.01821	0.06022	0.04354	0.01358	0.0092	0.0205	0.25992	-0.38183	0.38183	0.81309	0.434	0.57178	0.86004	0.9049	0.79018	0.0006	2.56E-07	2.56E-07	pink
NRBP1	-0.04617	-0.08778	0.01444	0.0423	-0.16033	0.12215	0.16264	0.06182	-0.06182	0.54875	0.2536	0.85133	0.58279	0.03619	0.11148	0.03356	0.42184	0.42184	turquoise
WDR19	-0.06434	-0.2163	-0.18184	-0.08705	-0.0027	-0.17831	-0.12358	0.32587	-0.32587	0.40315	0.00449	0.0173	0.25758	0.97206	0.01963	0.10733	1.37E-05	1.37E-05	turquoise
SIK1	0.0852	-0.06054	-0.12233	0.05053	0.06464	-0.10736	0.01734	0.11783	-0.11783	0.26786	0.43152	0.11096	0.51158	0.40095	0.16223	0.82195	0.12481	0.12481	grey
SERPINB4	-0.06504	0.07065	0.05422	-0.02335	-0.04797	-0.00992	0.10069	-0.26584	0.26584	0.398	0.35852	0.48118	0.76183	0.53326	0.8975	0.19006	0.00044	0.00044	grey
MSMB	-0.00603	0.0068	-0.0244	-0.09757	0.1973	0.05217	-0.0162	-0.16352	0.16352	0.93766	0.9297	0.75142	0.20423	0.00969	0.498	0.83345	0.0326	0.0326	grey
ALDH3A2	-0.00415	-0.0242	-0.11447	0.05884	0.00353	-0.00785	-0.08185	0.11957	-0.11957	0.95701	0.75335	0.13599	0.44458	0.96345	0.91882	0.28719	0.11929	0.11929	grey
SFTPD	-0.00496	-0.03665	-0.07081	0.02567	0.03324	-0.21623	-0.13887	0.0207	-0.0207	0.94871	0.63411	0.35737	0.73896	0.666	0.0045	0.07008	0.78816	0.78816	grey
B3GNT6	0.08913	-0.00647	0.12799	0.04771	0.13514	0.14705	0.01195	-0.2151	0.2151	0.24633	0.93303	0.09526	0.53546	0.07801	0.05495	0.87677	0.00472	0.00472	grey
VTI1A	0.02556	-0.06815	0.04041	0.01557	-0.18056	0.13142	0.16649	-0.1148	0.1148	0.73999	0.37581	0.59979	0.83981	0.01812	0.08665	0.02953	0.13489	0.13489	turquoise
PLA1A	-0.04957	0.04959	-0.16863	-0.09763	-0.09679	0.06825	-0.14904	0.34857	-0.34857	0.51967	0.51947	0.02747	0.20397	0.20791	0.37509	0.05172	2.98E-06	2.98E-06	blue
ZSCAN21	0.01012	-0.20516	-0.16567	-0.02459	0.06098	-0.12968	-0.04787	0.18392	-0.18392	0.89553	0.00711	0.03035	0.74952	0.42818	0.09093	0.53415	0.01604	0.01604	turquoise
CD33	-0.12734	-0.00206	0.11296	0.05862	-0.20533	0.17676	0.11068	0.43959	-0.43959	0.09697	0.97863	0.14129	0.44627	0.00706	0.02074	0.14955	1.80E-09	1.80E-09	blue
EXOSC9	-0.16698	-0.0736	-0.07072	-0.0991	0.04811	-0.09058	-0.0101	-0.00704	0.00704	0.02905	0.3387	0.35803	0.19719	0.5321	0.23871	0.89568	0.92715	0.92715	turquoise
THYN1	0.07198	-0.05601	-0.11924	-0.17624	0.00172	-0.02552	0.10128	-0.14156	0.14156	0.34946	0.46681	0.12033	0.02112	0.98215	0.74041	0.18749	0.06477	0.06477	grey
GEMIN5	-0.04776	-0.13692	-0.05087	0.01208	-0.07697	-0.00131	-0.00304	0.25605	-0.25605	0.53509	0.07415	0.50874	0.8754	0.31704	0.98645	0.96856	0.00072	0.00072	turquoise
ZNF266	-0.0572	-0.01674	-0.13961	-0.08575	0.13768	-0.13817	-0.07969	0.02349	-0.02349	0.45739	0.82796	0.06859	0.26479	0.07254	0.07151	0.30014	0.76037	0.76037	red
MGAM2	0.00275	-0.05528	-0.03759	0.04558	0.05915	0.07937	0.0186	0.07846	-0.07846	0.9715	0.47264	0.62548	0.55386	0.44224	0.3021	0.80922	0.30771	0.30771	grey
DZIP1L	-0.15178	-0.02953	-0.00438	0.07407	0.00904	0.13531	0.13662	0.15554	-0.15554	0.0475	0.7014	0.95465	0.33562	0.90658	0.07765	0.07479	0.04222	0.04222	grey
SLC25A42	0.00339	-0.07456	-0.18664	-0.05415	0.08653	-0.24714	-0.24896	0.41083	-0.41083	0.96489	0.33245	0.01451	0.48179	0.26047	0.00112	0.00103	2.39E-08	2.39E-08	brown
SCML4	-0.04175	-0.0437	-0.02026	0.01202	-0.05197	-0.04287	-0.16526	0.42172	-0.42172	0.58772	0.57033	0.79256	0.87601	0.49962	0.57766	0.03076	9.22E-09	9.22E-09	blue
COL16A1	0.04767	-0.02678	0.09562	0.07586	-0.0417	0.17632	0.06205	0.13781	-0.13781	0.53579	0.72808	0.21346	0.32409	0.58812	0.02106	0.42008	0.07226	0.07226	black
NOD2	-0.05638	-0.03516	0.07175	0.0384	-0.18656	0.08908	0.18049	-0.02977	0.02977	0.46391	0.64803	0.35104	0.61802	0.01456	0.24659	0.01816	0.69913	0.69913	black
SHLD1	0.02886	0.03777	-0.061	-0.05853	0.10206	-0.15211	-0.09572	0.03582	-0.03582	0.70793	0.62382	0.42803	0.44697	0.18407	0.04702	0.21301	0.64186	0.64186	grey
RPS8	0.04862	0.09421	0.04911	-0.06713	-0.11522	0.11671	0.10823	-0.10734	0.10734	0.52768	0.22032	0.52359	0.383	0.13345	0.12848	0.15884	0.16227	0.16227	purple
ARRDC1	0.04934	-0.03599	0.01421	-0.00863	0.06893	0.07841	0.16593	-0.54969	0.54969	0.52164	0.64028	0.85366	0.91077	0.37032	0.30805	0.03008	6.90E-15	6.90E-15	green
KIAA1109	-0.09712	-0.20416	-0.04246	0.01409	-0.04751	0.00867	-0.07956	0.27453	-0.27453	0.20635	0.0074	0.58135	0.85491	0.53716	0.91037	0.30096	0.00028	0.00028	turquoise
ADAM22	0.06924	-0.11168	-0.03747	0.00178	-0.18453	0.03977	-0.11883	0.48103	-0.48103	0.36818	0.14587	0.6266	0.98158	0.01569	0.6055	0.12162	2.75E-11	2.75E-11	blue
VPS33A	0.00544	-0.07684	-0.11122	-0.1182	0.10055	-0.22472	-0.1079	0.24382	-0.24382	0.9437	0.31785	0.14756	0.12363	0.19071	0.00313	0.1601	0.00131	0.00131	turquoise
ATP5F1A	-0.11328	-0.06907	-0.19143	-0.08844	0.10509	-0.1848	-0.11099	0.33423	-0.33423	0.14017	0.3694	0.01214	0.25005	0.17132	0.01553	0.1484	7.90E-06	7.90E-06	turquoise
CCDC89	0.00014	-0.13273	0.02976	0.16265	-0.00115	-0.13011	-0.03332	0.42632	-0.42632	0.99858	0.08352	0.69918	0.03354	0.98813	0.08986	0.6653	6.11E-09	6.11E-09	brown
DICER1	-0.04059	-0.1399	-0.04579	0.0207	-0.0424	0.00181	-0.10162	0.30188	-0.30188	0.59815	0.068	0.55205	0.78816	0.58185	0.98126	0.18601	6.00E-05	6.00E-05	turquoise
RNF215	-0.11181	-0.09053	-0.07146	0.06077	0.00451	-0.03141	0.04126	-0.11625	0.11625	0.1454	0.23894	0.35303	0.42979	0.95328	0.68342	0.59205	0.13	0.13	grey
SH3BGR	-0.07693	-0.07697	-0.085	0.02029	0.18012	-0.10505	-0.22783	0.41035	-0.41035	0.31729	0.31703	0.26898	0.79222	0.0184	0.1715	0.00273	2.49E-08	2.49E-08	grey
RPS15A	0.08496	0.19019	0.0756	-0.00209	-0.07307	0.19423	0.18112	-0.15626	0.15626	0.26922	0.01272	0.32574	0.97839	0.34226	0.01091	0.01775	0.04126	0.04126	purple
CHD8	-0.02138	-0.14337	-0.02593	0.01656	-0.11759	0.02008	0.01909	0.17406	-0.17406	0.78136	0.06139	0.73635	0.82981	0.1256	0.79432	0.80431	0.0228	0.0228	turquoise
CCDC24	0.02335	-0.06637	-0.15695	-0.14065	0.32262	-0.19034	-0.08927	-0.3112	0.3112	0.76177	0.38841	0.04035	0.06652	1.68E-05	0.01265	0.2456	3.43E-05	3.43E-05	green
IFI44	-0.06306	0.02925	0.09145	0.12369	-0.1312	0.16214	0.23595	-0.11298	0.11298	0.41255	0.70416	0.23421	0.10701	0.08717	0.03411	0.00189	0.14122	0.14122	tan
GADD45G	0.04301	0.05866	-0.15479	-0.04868	0.11494	-0.17999	0.06742	-0.3327	0.3327	0.57649	0.44602	0.04324	0.52723	0.1344	0.01849	0.38096	8.75E-06	8.75E-06	green
CLIC6	0.0242	-0.09773	0.02927	0.00868	-0.03264	0.1889	0.0307	0.09102	-0.09102	0.75341	0.20348	0.70388	0.91034	0.67172	0.01335	0.69016	0.23642	0.23642	grey
CAMK1G	-0.02257	-0.07897	-0.06971	0.01881	-0.09962	0.04281	-0.04292	0.38369	-0.38369	0.76947	0.30453	0.36497	0.80705	0.19487	0.57823	0.57727	2.22E-07	2.22E-07	blue
CETN3	-0.04143	-0.10895	-0.14441	-0.03581	0.02977	-0.18472	-0.08203	0.13846	-0.13846	0.59055	0.15605	0.0595	0.64191	0.69908	0.01558	0.28614	0.07092	0.07092	turquoise
LOXL3	-0.07685	-0.05347	0.04952	0.07881	-0.21299	0.10283	0.19354	0.22922	-0.22922	0.31776	0.48734	0.52009	0.30553	0.00516	0.18077	0.0112	0.00256	0.00256	black
FAM229A	-0.00801	-0.07592	-0.10552	-0.08952	0.19411	-0.03767	-0.0158	-0.14599	0.14599	0.91715	0.32367	0.16957	0.24429	0.01096	0.6247	0.8375	0.05674	0.05674	red
PNMT	0.10731	-0.01424	-0.07021	0.02255	0.05086	-0.15301	0.12024	-0.01928	0.01928	0.1624	0.85333	0.36151	0.76967	0.50886	0.04572	0.11724	0.8024	0.8024	grey
TUSC2	0.06177	-0.03883	-0.10381	-0.17751	0.00594	0.01547	-0.00359	-0.11913	0.11913	0.42217	0.61408	0.17663	0.0202	0.93858	0.84086	0.96284	0.12069	0.12069	grey
TPP1	-0.02894	-0.19706	0.03656	-0.00478	-0.17544	0.01818	-0.02114	0.31995	-0.31995	0.7071	0.00978	0.63494	0.95052	0.02172	0.81344	0.78374	1.99E-05	1.99E-05	turquoise
PIP4P1	0.02375	-0.08267	-0.00377	-0.03817	0.03741	-0.04476	-0.10182	-0.08886	0.08886	0.75781	0.28241	0.96099	0.62013	0.62717	0.56106	0.18513	0.24777	0.24777	turquoise
WDR45	0.00615	-0.07207	-0.06477	-0.0561	-0.05168	-0.01897	0.11495	-0.15255	0.15255	0.93636	0.34891	0.39999	0.46613	0.50204	0.80548	0.13437	0.04639	0.04639	grey
CXCL14	-0.0582	0.03285	0.0007	-0.07485	-0.08328	0.1734	0.08356	-0.02083	0.02083	0.4496	0.66975	0.99273	0.33055	0.27886	0.02333	0.27722	0.78686	0.78686	black
MBD6	0.15678	-0.1535	-0.06267	0.01446	0.13823	-0.03321	-0.0176	-0.16636	0.16636	0.04057	0.04502	0.41543	0.85112	0.07139	0.66636	0.81927	0.02965	0.02965	red
EMC9	0.06768	-0.07516	-0.13818	-0.06595	0.2293	-0.22352	-0.01014	-0.35081	0.35081	0.37911	0.32856	0.0715	0.39145	0.00255	0.0033	0.89525	2.55E-06	2.55E-06	green
ENTPD4	-0.0601	-0.12625	0.0498	-0.02759	0.00862	-0.01364	-0.00372	0.14957	-0.14957	0.43486	0.09988	0.51777	0.72014	0.91092	0.8595	0.96146	0.05088	0.05088	turquoise
CCT5	0.02549	-0.03657	0.03463	-0.01913	-0.11935	-0.02676	0.21627	-0.19946	0.19946	0.74073	0.63493	0.65295	0.80383	0.11999	0.72827	0.0045	0.00891	0.00891	turquoise
PCDHGB7	0.01004	-0.12122	0.00077	0.00878	-0.13261	0.10867	0.00759	0.25497	-0.25497	0.89634	0.11427	0.99203	0.90924	0.0838	0.1571	0.9215	0.00076	0.00076	turquoise
SPNS2	0.0942	0.15605	-0.00999	0.0468	0.0164	0.13917	0.00599	-0.13186	0.13186	0.22037	0.04154	0.89682	0.5433	0.83142	0.06946	0.93807	0.08559	0.08559	grey
CLECL1	-0.06812	0.00323	0.04822	0.03749	-0.17038	0.09438	-0.01363	0.37817	-0.37817	0.37603	0.96652	0.53115	0.62636	0.02588	0.21952	0.85961	3.40E-07	3.40E-07	blue
BBX	-0.02808	-0.18678	-0.08533	-0.03471	-0.16656	0.07597	-0.01414	0.20115	-0.20115	0.71543	0.01444	0.26715	0.65217	0.02946	0.32338	0.85434	0.00834	0.00834	turquoise
NME2	0.00374	0.0344	-0.07489	0.00642	0.21066	-0.01454	0.109	-0.31227	0.31227	0.96127	0.65515	0.33032	0.93357	0.00568	0.85025	0.15587	3.21E-05	3.21E-05	green
SCAF11	0.02833	-0.18568	-0.02313	0.0369	-0.04891	0.06278	-0.04599	0.17899	-0.17899	0.71299	0.01504	0.76392	0.63184	0.52525	0.41463	0.55032	0.01916	0.01916	turquoise
FAM240C	-0.07665	0.11258	0.03568	0.12733	-0.09976	0.04416	-0.10366	0.18299	-0.18299	0.31902	0.14262	0.6431	0.09698	0.1942	0.56631	0.17725	0.0166	0.0166	magenta
FANCG	0.04044	-0.08776	-0.08181	-0.04788	-0.01601	-0.06531	0.10206	-0.1284	0.1284	0.59946	0.25371	0.28746	0.53399	0.83531	0.39608	0.18409	0.09419	0.09419	turquoise
UGGT2	-0.03186	-0.10307	-0.01771	-0.0149	-0.08277	-0.04427	0.07603	0.11604	-0.11604	0.67911	0.17976	0.81818	0.84664	0.28183	0.56536	0.32295	0.13068	0.13068	turquoise
GSC	-0.11909	0.00538	0.01672	0.13919	-0.07796	0.12359	0.10199	-0.072	0.072	0.12082	0.94427	0.82817	0.06943	0.31083	0.10729	0.18438	0.34939	0.34939	grey
CCDC47	-0.09977	-0.18046	-0.01849	-0.03303	-0.00507	0.00291	-0.01338	0.10433	-0.10433	0.19419	0.01818	0.81028	0.66803	0.94751	0.96988	0.86213	0.17448	0.17448	turquoise
CCL8	-0.11656	0.00979	0.06492	0.02738	-0.08637	-0.00942	0.04673	0.40673	-0.40673	0.12896	0.89888	0.39886	0.72227	0.26134	0.90263	0.54388	3.38E-08	3.38E-08	blue
RELCH	-0.05559	-0.10045	-0.08041	0.01763	0.18235	-0.0301	-0.07498	0.11607	-0.11607	0.47023	0.19115	0.29578	0.81899	0.01698	0.6959	0.32975	0.1306	0.1306	turquoise
KCNIP2	-0.0459	-0.02068	0.01493	0.02146	0.0945	-0.05046	-0.06926	0.20262	-0.20262	0.55109	0.78839	0.84632	0.78056	0.21893	0.51221	0.36803	0.00786	0.00786	blue
INPP5E	-0.05413	-0.15715	-0.17161	-0.05041	0.06418	-0.20994	-0.07905	0.04416	-0.04416	0.48193	0.0401	0.02481	0.5126	0.40429	0.00585	0.3041	0.56626	0.56626	red
CAPRIN1	0.04684	-0.07577	0.05837	0.00502	-0.16892	0.14196	0.12561	0.00585	-0.00585	0.54292	0.32461	0.44826	0.94802	0.0272	0.064	0.10163	0.93948	0.93948	turquoise
CD8B2	0.00664	0.04435	-0.07158	-0.02231	0.04629	-0.02779	-0.01847	0.1248	-0.1248	0.93132	0.56461	0.35215	0.77207	0.5477	0.71821	0.81051	0.10389	0.10389	grey
KLHDC9	0.08097	-0.05324	-0.2266	-0.16975	0.23098	-0.22079	-0.12625	-0.06473	0.06473	0.29245	0.48916	0.00288	0.02644	0.00237	0.00371	0.09989	0.40028	0.40028	grey
IFT74	0.04135	-0.02831	-0.17125	-0.13803	0.00825	-0.09826	-0.05087	0.19333	-0.19333	0.59125	0.71322	0.02512	0.0718	0.91472	0.20106	0.50875	0.01129	0.01129	turquoise
ERVK3-1	-0.00729	-0.18182	-0.10497	-0.02236	0.05152	-0.03152	-0.034	0.05292	-0.05292	0.92456	0.01731	0.17181	0.77156	0.5034	0.68234	0.65891	0.49185	0.49185	red
COX5B	0.19022	0.09392	-0.0924	-0.148	0.07906	-0.04302	0.15073	-0.49978	0.49978	0.0127	0.22178	0.22936	0.05339	0.30401	0.57641	0.04909	3.42E-12	3.42E-12	green
ZFP82	-0.1239	-0.17717	-0.11639	0.02081	-0.10106	-0.07518	-0.13108	0.57564	-0.57564	0.1064	0.02044	0.12952	0.78709	0.18843	0.32845	0.08748	1.78E-16	1.78E-16	blue
PLXNC1	-0.11931	-0.07852	-0.04082	#######	-0.16962	-0.07457	-0.07348	0.53189	-0.53189	0.12012	0.30732	0.59604	0.9991	0.02656	0.33238	0.33952	7.11E-14	7.11E-14	blue
NUP133	0.0154	-0.04602	-0.07947	-0.07707	-0.08868	0.00018	0.10499	0.10907	-0.10907	0.84151	0.55006	0.30152	0.31636	0.24874	0.99809	0.17175	0.1556	0.1556	turquoise
RFC5	0.01829	-0.01894	0.01073	-0.03941	-0.02414	0.01699	0.12344	-0.16533	0.16533	0.8123	0.80581	0.88923	0.60879	0.75402	0.82547	0.10773	0.0307	0.0307	turquoise
SNRPD2	0.09976	0.01427	-0.0652	-0.10311	0.06334	-0.05117	0.15388	-0.29883	0.29883	0.19422	0.85302	0.39685	0.1796	0.41051	0.50629	0.04448	7.18E-05	7.18E-05	grey
LCAT	-0.0389	-0.11192	-0.00548	0.02513	0.04952	0.01368	0.13094	-0.13635	0.13635	0.61345	0.14501	0.9433	0.74424	0.52009	0.85908	0.08782	0.07537	0.07537	red
GALNT12	0.11834	-0.04464	0.0775	0.06969	0.07118	0.15265	0.07618	-0.35259	0.35259	0.12316	0.56206	0.31371	0.36506	0.35489	0.04624	0.32202	2.25E-06	2.25E-06	grey
SART1	0.11239	-0.00473	-0.03039	-0.01108	-0.03396	-0.03185	0.15154	-0.25526	0.25526	0.1433	0.95104	0.6932	0.8856	0.65928	0.67922	0.04787	0.00075	0.00075	greenyellow
FDX2	0.05243	-0.00269	-0.17852	-0.1172	0.11548	-0.21524	0.02088	-0.07465	0.07465	0.49584	0.97211	0.01948	0.12687	0.13256	0.0047	0.78633	0.33187	0.33187	green
HNRNPA2	-0.01442	-0.12528	-0.0458	-0.01213	-0.00185	0.00016	0.08103	-0.09553	0.09553	0.8515	0.10254	0.55199	0.87484	0.98086	0.99839	0.29208	0.21389	0.21389	turquoise
ASB8	-0.00393	-0.17685	-0.12082	-0.02051	0.06075	-0.15243	-0.19557	0.32105	-0.32105	0.95932	0.02067	0.11546	0.79006	0.42993	0.04656	0.01036	1.86E-05	1.86E-05	turquoise
TNFRSF10	0.03113	0.00073	0.09935	-0.04233	-0.06972	0.0174	0.20324	-0.09119	0.09119	0.68608	0.99249	0.19606	0.58247	0.36489	0.82124	0.00767	0.23554	0.23554	grey
CENPF	-0.0011	0.07747	-0.00942	-0.02927	-0.09106	0.10298	0.29539	-0.37897	0.37897	0.98862	0.3139	0.90264	0.70393	0.2362	0.18013	8.77E-05	3.20E-07	3.20E-07	pink
SEPTIN1	-0.06397	0.00907	-0.04172	-0.02809	-0.06109	-0.01519	-0.06767	0.36144	-0.36144	0.40586	0.90632	0.58797	0.7153	0.42735	0.84365	0.37917	1.19E-06	1.19E-06	blue
RPS14	0.07162	0.08905	-0.01144	-0.02025	0.0055	0.07699	0.06472	-0.18443	0.18443	0.35194	0.24674	0.88192	0.79264	0.94312	0.31688	0.40038	0.01575	0.01575	purple
NFIX	0.00531	-0.03753	-0.04247	0.08242	-0.16332	0.2222	0.11962	0.22965	-0.22965	0.94504	0.626	0.58122	0.28383	0.03281	0.00349	0.11914	0.00251	0.00251	turquoise
DMTN	-0.01202	-0.09693	-0.08446	-0.0941	0.16656	-0.16903	-0.16259	0.03004	-0.03004	0.87599	0.20725	0.27206	0.22088	0.02946	0.0271	0.03361	0.69655	0.69655	grey
BAG1	0.1778	0.00544	-0.13974	-0.11335	0.09752	-0.06782	0.00941	-0.23777	0.23777	0.01999	0.94371	0.06832	0.13989	0.20448	0.37809	0.90276	0.00174	0.00174	grey
TJAP1	-0.03423	-0.11381	-0.07895	-0.01477	0.12218	-0.0589	0.03186	-0.05778	0.05778	0.65669	0.13829	0.30469	0.84793	0.1114	0.44416	0.67913	0.45286	0.45286	red
RETN	-0.01719	-0.02293	0.0864	0.0303	-0.07114	0.11134	0.02755	0.00828	-0.00828	0.82342	0.76599	0.26115	0.69403	0.35515	0.14713	0.72061	0.91438	0.91438	blue
SEMA4B	0.02934	-0.10389	0.01204	0.00354	0.03967	0.067	0.05628	-0.48162	0.48162	0.70324	0.17632	0.87585	0.96335	0.60646	0.3839	0.46469	2.58E-11	2.58E-11	yellow
TMEM170	-0.07629	-0.10334	-0.09566	-0.02182	-0.05783	-0.12811	-0.2144	0.64189	-0.64189	0.32131	0.17862	0.21327	0.77699	0.4525	0.09496	0.00486	3.07E-21	3.07E-21	turquoise
NAA30	0.00785	-0.17364	-0.02488	0.06791	-0.03954	-0.00785	-0.05646	0.15464	-0.15464	0.9188	0.02313	0.74666	0.37746	0.60765	0.91883	0.4633	0.04343	0.04343	turquoise
ENKD1	0.10469	0.02308	-0.19423	-0.08159	0.20576	-0.08284	0.09222	-0.35156	0.35156	0.17298	0.7645	0.01091	0.28876	0.00694	0.28139	0.23026	2.42E-06	2.42E-06	green
CHRDL2	9.34E-06	-0.00791	-0.00135	0.03254	0.00611	0.02918	-0.03685	0.38903	-0.38903	0.9999	0.91819	0.98601	0.67266	0.93679	0.70481	0.63227	1.45E-07	1.45E-07	salmon
GABPA	-0.09634	-0.12774	-0.04021	0.03045	-0.03207	0.01527	-0.06504	0.39142	-0.39142	0.21002	0.0959	0.60157	0.6926	0.67715	0.84284	0.39802	1.20E-07	1.20E-07	turquoise
ADCY4	-0.13879	0.02996	-0.024	0.00288	-0.00123	-0.00057	-0.08785	0.35074	-0.35074	0.07023	0.69732	0.75539	0.97014	0.98723	0.9941	0.2532	2.56E-06	2.56E-06	blue
NFYB	0.03179	-0.13787	-0.04928	0.00932	-0.02956	-0.00827	-0.07588	0.3875	-0.3875	0.67982	0.07213	0.52216	0.90375	0.70111	0.91446	0.32391	1.64E-07	1.64E-07	turquoise
PCDHGB5	-0.18794	-0.27078	0.01971	0.03947	-0.10829	0.07016	0.09122	0.11284	-0.11284	0.01383	0.00034	0.79802	0.60831	0.1586	0.36186	0.23539	0.1417	0.1417	grey
CLASP2	-0.0665	-0.13071	-0.11341	-0.066	-0.08594	-0.05667	-0.11342	0.46488	-0.46488	0.38749	0.08839	0.13969	0.39106	0.2637	0.4616	0.13966	1.50E-10	1.50E-10	turquoise
MRGPRF	-0.08424	-0.05344	0.02929	0.06437	-0.04556	0.05042	0.0414	0.36164	-0.36164	0.27329	0.48758	0.70372	0.40289	0.55404	0.51251	0.5908	1.17E-06	1.17E-06	salmon
FXR2	-0.00709	-0.19694	-0.16312	-0.15188	0.04184	-0.25147	-0.12102	0.34935	-0.34935	0.92671	0.00983	0.03303	0.04736	0.58688	0.00091	0.11485	2.82E-06	2.82E-06	turquoise
LPCAT4	0.03572	0.02106	0.08607	-0.00954	0.09237	0.175	0.1369	-0.52829	0.52829	0.64276	0.78451	0.26302	0.9014	0.22953	0.02206	0.07418	1.12E-13	1.12E-13	yellow
ULK3	-0.03814	-0.06616	-0.09978	-0.1066	0.2498	-0.11483	-0.11791	-0.08565	0.08565	0.62041	0.38992	0.19414	0.16522	0.00098	0.13479	0.12455	0.26533	0.26533	red
ICE2	-0.08065	-0.10822	-0.12527	-0.03645	-0.06763	-0.0496	-0.02891	0.23853	-0.23853	0.29438	0.15885	0.10256	0.63597	0.37943	0.51946	0.70739	0.00168	0.00168	turquoise
DACT3	-0.05434	-0.0518	0.01785	0.1004	-0.14275	0.07598	0.05448	0.32885	-0.32885	0.48029	0.501	0.81674	0.19135	0.06252	0.32332	0.47913	1.13E-05	1.13E-05	salmon
HEPACAM	0.01154	-0.0458	-0.19961	-0.06741	0.19043	-0.11049	-0.13268	0.22182	-0.22182	0.88096	0.55197	0.00886	0.38104	0.0126	0.15025	0.08364	0.00355	0.00355	brown
ALKBH3	-0.1345	-0.04477	-0.04778	-0.02478	-0.11785	-0.06922	0.0958	0.09533	-0.09533	0.07945	0.56091	0.53484	0.74768	0.12476	0.36831	0.21262	0.21488	0.21488	grey
CALHM6	-0.11531	0.05452	-0.04838	-0.00524	-0.18804	0.0779	0.09238	0.31109	-0.31109	0.13314	0.47883	0.52972	0.94579	0.01378	0.3112	0.22944	3.45E-05	3.45E-05	blue
GID8	0.11158	-0.06913	-0.04652	-0.01605	-0.01928	-0.07711	0.10056	-0.24165	0.24165	0.14624	0.36899	0.54573	0.83497	0.80236	0.3161	0.19066	0.00145	0.00145	turquoise
JUP	0.06072	0.00491	0.00382	0.03396	0.03399	0.07124	0.13376	-0.49912	0.49912	0.43017	0.94916	0.96043	0.65928	0.65893	0.35449	0.08114	3.69E-12	3.69E-12	yellow
UAP1	0.00238	-0.02705	0.13354	-0.0074	-0.03706	0.24143	0.1041	-0.08714	0.08714	0.97537	0.72541	0.08163	0.92352	0.6303	0.00147	0.17542	0.25707	0.25707	turquoise
AP5B1	-0.08839	-0.03049	-0.02649	0.01586	-0.02757	0.08418	0.11485	0.01243	-0.01243	0.25031	0.69223	0.73092	0.83683	0.72035	0.27366	0.13469	0.87181	0.87181	grey
DCTPP1	0.05208	0.14015	0.02557	-0.02968	-0.16371	0.1571	0.24594	-0.32846	0.32846	0.49872	0.0675	0.73995	0.70002	0.03239	0.04016	0.00118	1.15E-05	1.15E-05	turquoise
PURB	-0.05167	-0.18635	-0.0193	0.07132	-0.08257	0.03254	0.0577	0.07751	-0.07751	0.50209	0.01467	0.80216	0.35392	0.28297	0.67265	0.45351	0.31362	0.31362	turquoise

POLA2	-0.07805	0.0364	0.03879	-0.03534	-0.11028	0.06684	0.24184	-0.25238	0.25238	0.31026	0.63644	0.6145	0.64636	0.15102	0.38507	0.00144	0.00087	0.00087	pink
RNF220	-0.03327	-0.04523	-0.0943	-0.11025	-0.00099	-0.08665	-0.02447	0.04392	-0.04392	0.66571	0.55691	0.21988	0.15115	0.98975	0.25979	0.75076	0.56838	0.56838	turquoise
CDKN1A	0.05278	-0.05303	0.0833	0.09733	-0.02417	0.12509	0.02752	-0.00829	0.00829	0.49297	0.49094	0.27872	0.20538	0.75363	0.10305	0.72091	0.91426	0.91426	grey
VAMP8	0.07165	0.02499	0.10031	0.04537	-0.12597	0.20928	0.30498	-0.49356	0.49356	0.35174	0.74562	0.19175	0.55572	0.10065	0.00601	4.99E-05	6.93E-12	6.93E-12	yellow
KCNJ3	0.03639	-0.13178	-0.22434	-0.11694	0.24165	-0.29606	-0.20369	0.29882	-0.29882	0.63657	0.08577	0.00318	0.1277	0.00145	8.44E-05	0.00754	7.19E-05	7.19E-05	brown
NRDC	-0.10838	-0.14545	0.01324	-0.01592	-0.10877	0.02746	0.04461	0.10811	-0.10811	0.15826	0.05767	0.86352	0.83625	0.15672	0.72142	0.56237	0.15928	0.15928	turquoise
COPS7A	0.00687	-0.05208	-0.08106	-0.07095	0.09425	-0.11488	-0.05658	-0.0309	0.0309	0.92898	0.49872	0.29189	0.35644	0.22015	0.13462	0.4623	0.68826	0.68826	grey
ACAD9	-0.03419	0.02103	-0.11724	-0.02731	0.02728	-0.00461	0.14322	-0.18035	0.18035	0.65708	0.78484	0.12674	0.72294	0.72325	0.95223	0.06167	0.01825	0.01825	turquoise
NDUFS8	0.05257	0.04605	-0.17653	-0.06328	0.18037	-0.19963	0.09034	-0.36034	0.36034	0.49468	0.54978	0.02091	0.41092	0.01824	0.00885	0.23997	1.29E-06	1.29E-06	green
TBC1D19	0.00163	-0.18209	-0.13944	-0.08154	0.0259	-0.11151	-0.13013	0.34083	-0.34083	0.98313	0.01714	0.06891	0.28905	0.73664	0.14648	0.08981	5.08E-06	5.08E-06	turquoise
ARHGEF2[illegible]	0.11614	-0.0012	-0.08392	-0.08187	0.08927	-0.04223	-0.03267	-0.09219	0.09219	0.13034	0.98753	0.27516	0.2871	0.24558	0.58341	0.67138	0.23042	0.23042	grey
RPL3	0.05232	0.13926	-0.13048	-0.10317	0.06582	-0.13904	-0.14959	0.1333	-0.1333	0.49675	0.06929	0.08893	0.17934	0.39239	0.06973	0.05084	0.08218	0.08218	purple
C12orf76	0.06481	-0.18144	-0.15619	-0.09087	0.14074	-0.32102	-0.13838	0.15269	-0.15269	0.39966	0.01755	0.04135	0.2372	0.06634	1.86E-05	0.07108	0.04618	0.04618	brown
MZB1	-0.17783	0.1003	0.06337	0.00399	-0.09208	0.14735	0.11713	0.23804	-0.23804	0.01997	0.19179	0.41028	0.95867	0.23101	0.05445	0.12709	0.00172	0.00172	blue
SLC2A4	-0.0802	-0.04799	0.00637	0.08047	-0.05433	0.00552	-0.03333	0.27273	-0.27273	0.29707	0.5331	0.93415	0.29542	0.48032	0.94285	0.66514	0.00031	0.00031	salmon
ATP13A1	0.07374	-0.02704	-0.17885	-0.09822	0.2416	-0.22186	-0.10263	-0.03018	0.03018	0.33781	0.72558	0.01926	0.20124	0.00146	0.00354	0.18161	0.6952	0.6952	green
KIAA1211	-0.02784	-0.10699	0.08189	0.02877	0.0273	0.2083	0.05031	0.12825	-0.12825	0.71777	0.16366	0.28699	0.70878	0.72303	0.00626	0.51348	0.09458	0.09458	turquoise
PRMT7	0.05977	0.0539	-0.13693	-0.14141	0.18928	-0.062	0.05551	-0.21447	0.21447	0.43738	0.48381	0.07411	0.06506	0.01316	0.42051	0.47085	0.00485	0.00485	red
RPP25	0.15427	0.10588	0.08317	0.11198	0.02586	0.10836	0.25614	-0.35424	0.35424	0.04395	0.16812	0.27947	0.14478	0.73707	0.15831	0.00072	2.00E-06	2.00E-06	yellow
TMSB4Y	0.82219	-0.03551	-0.09616	-0.02128	0.08173	-0.02973	0.0074	0.04572	-0.04572	3.25E-43	0.64469	0.2109	0.78231	0.28793	0.69949	0.92344	0.55264	0.55264	grey
CORO6	-0.01196	-0.02988	0.01685	0.06927	0.05964	-0.1013	0.03997	0.14895	-0.14895	0.87657	0.69801	0.8269	0.36801	0.43845	0.18737	0.60371	0.05185	0.05185	grey
RAB40B	0.04192	0.03132	-0.05235	-0.06795	0.25051	-0.02038	0.04051	-0.27588	0.27588	0.58619	0.68428	0.49649	0.37722	0.00095	0.79135	0.59886	0.00026	0.00026	grey
TMEM216	-0.00454	-0.05525	-0.02853	-0.03859	-0.07545	0.06721	0.07581	-0.13521	0.13521	0.95297	0.47295	0.71104	0.61628	0.32672	0.3824	0.32439	0.07786	0.07786	turquoise
PAFAH1B[illegible]	0.08703	0.06452	-0.09299	-0.11087	0.06229	-0.14443	0.10764	-0.42728	0.42728	0.25767	0.40181	0.22617	0.14885	0.41831	0.05947	0.16111	5.60E-09	5.60E-09	green
FUBP3	-0.04694	-0.15364	-0.05413	0.0363	-0.04588	-0.08294	-0.04372	0.16709	-0.16709	0.54209	0.04483	0.48197	0.63739	0.55128	0.28082	0.57019	0.02894	0.02894	turquoise
MAL	-0.04529	-0.03115	-0.03651	0.02038	-0.07088	-0.03209	-0.10173	0.38531	-0.38531	0.55637	0.68585	0.63548	0.79135	0.35692	0.67693	0.1855	1.95E-07	1.95E-07	blue
ANGPTL4	-0.04621	-0.12746	0.04086	0.14722	-0.04668	0.01908	0.06172	-0.24158	0.24158	0.54837	0.09665	0.59573	0.05467	0.54435	0.8044	0.42257	0.00146	0.00146	grey
DRG2	-0.05555	-0.0605	-0.07427	-0.02691	-0.01185	-0.12259	-0.06491	0.19709	-0.19709	0.47054	0.43183	0.33435	0.72679	0.87775	0.11018	0.39899	0.00977	0.00977	grey
PLXNA4	-0.00784	-0.03788	-0.00457	0.03458	-0.07467	0.02334	-0.08015	0.44363	-0.44363	0.91892	0.62275	0.95273	0.65342	0.33176	0.76191	0.29736	1.22E-09	1.22E-09	salmon
SLC25A46	-0.03904	-0.11862	-0.0719	-0.00611	0.04652	-0.0709	-0.08175	0.1976	-0.1976	0.61222	0.12229	0.35003	0.93673	0.54573	0.35681	0.28777	0.00958	0.00958	turquoise
MCOLN3	-0.06919	-0.17212	-0.20283	-0.08692	0.10762	-0.29464	-0.19875	0.35674	-0.35674	0.36853	0.02438	0.0078	0.25831	0.16119	9.16E-05	0.00916	1.67E-06	1.67E-06	brown
INCENP	-0.02849	-0.00523	0.04941	0.04093	-0.11282	0.1193	0.22446	-0.17525	0.17525	0.71147	0.94584	0.52104	0.59505	0.14178	0.12014	0.00316	0.02187	0.02187	pink
PDLIM5	-0.0543	-0.14591	0.06593	0.07843	-0.20282	0.20161	0.04962	0.15074	-0.15074	0.48056	0.05687	0.39156	0.30789	0.0078	0.00819	0.51927	0.04907	0.04907	turquoise
CHST2	-0.14018	-0.04545	-0.0362	-0.05948	-0.13236	-0.04194	-0.07804	0.38589	-0.38589	0.06745	0.55501	0.6383	0.43966	0.08439	0.58599	0.31032	1.86E-07	1.86E-07	blue
ZNF506	0.06966	-0.14655	-0.09464	0.00249	0.09314	-0.06458	-0.20906	0.25683	-0.25683	0.36528	0.0558	0.21823	0.97421	0.22566	0.40135	0.00607	0.0007	0.0007	turquoise
ZNF517	-0.02211	-0.08404	-0.1297	-0.07048	0.05921	-0.18751	0.02668	-0.00887	0.00887	0.77411	0.27449	0.09089	0.35968	0.44177	0.01405	0.72905	0.90829	0.90829	green
FKBP4	0.23179	-0.03609	-0.05707	0.00965	0.07686	-0.04731	0.04816	-0.29392	0.29392	0.00228	0.63934	0.45845	0.9003	0.31773	0.53893	0.53167	9.55E-05	9.55E-05	grey
KLRB1	-0.05392	-0.12892	0.02435	0.06337	-0.10729	0.16556	-0.0529	0.39074	-0.39074	0.48363	0.09286	0.75193	0.41024	0.16248	0.03046	0.49198	1.27E-07	1.27E-07	blue
KBTBD6	0.00467	-0.11395	-0.21228	-0.09949	0.00895	-0.26407	-0.13199	0.44275	-0.44275	0.95171	0.1378	0.00531	0.19543	0.90753	0.00048	0.08528	1.33E-09	1.33E-09	brown
RAF1	-0.04794	-0.10683	0.03498	0.01141	-0.11739	0.19757	0.10548	-0.02605	0.02605	0.53352	0.16433	0.64964	0.88225	0.12625	0.00959	0.16974	0.7352	0.7352	turquoise
CDNF	-0.15969	0.00538	-0.07374	-0.02061	0.12432	-0.14017	-0.10786	0.21254	-0.21254	0.03695	0.9443	0.3378	0.78901	0.10521	0.06747	0.16026	0.00526	0.00526	grey
RBIS	0.03138	0.03531	0.00511	-0.00474	-0.02494	-0.00319	0.16956	-0.32401	0.32401	0.68369	0.64662	0.94713	0.95092	0.74612	0.96693	0.02661	1.54E-05	1.54E-05	grey
KCNJ14	0.0784	-0.14091	-0.02568	-0.02662	0.08957	-0.06547	-0.03855	-0.27242	0.27242	0.30811	0.06602	0.73884	0.72964	0.24399	0.39489	0.61666	0.00031	0.00031	grey
TICAM1	0.04928	-0.01547	-0.04541	-0.00214	0.06138	0.05713	0.04703	-0.29158	0.29158	0.52213	0.84081	0.55531	0.97782	0.42518	0.45794	0.54128	0.00011	0.00011	yellow
MTRR	-0.06908	-0.16569	-0.05095	0.00356	-0.01028	-0.04197	-0.02662	0.24666	-0.24666	0.36933	0.03033	0.50808	0.96312	0.89384	0.58574	0.72961	0.00115	0.00115	turquoise
TTC21A	0.03543	-0.15558	-0.26235	-0.13062	0.14444	-0.15577	-0.17064	0.15808	-0.15808	0.64552	0.04216	0.00053	0.08861	0.05945	0.0419	0.02565	0.03892	0.03892	red
GLYR1	0.03461	-0.0959	0.00412	0.00994	0.02577	0.03914	-0.02392	0.06461	-0.06461	0.65312	0.21211	0.95738	0.89729	0.73796	0.61126	0.75612	0.40114	0.40114	turquoise
GOLGB1	0.02081	-0.10429	-0.08559	-0.01797	-0.09234	0.15966	0.09266	0.00446	-0.00446	0.78706	0.17462	0.26567	0.81554	0.22967	0.03698	0.22805	0.95381	0.95381	turquoise
HES2	0.01755	0.06624	0.08109	0.06486	-0.05161	0.03639	0.16527	-0.238	0.238	0.81982	0.38935	0.29175	0.39935	0.50258	0.63657	0.03076	0.00172	0.00172	grey
FNBP1	-0.11614	-0.10038	0.03243	0.05997	-0.14438	0.05103	-0.0711	0.44993	-0.44993	0.13035	0.19143	0.67368	0.43589	0.05956	0.50741	0.35544	6.67E-10	6.67E-10	blue
EMILIN2	-0.08745	-0.03891	0.08367	0.05487	-0.17257	0.20019	0.14767	0.30168	-0.30168	0.25538	0.61336	0.2766	0.47595	0.02401	0.00866	0.05392	6.08E-05	6.08E-05	blue
CAPN12	-0.0303	-0.07809	0.03619	0.05804	0.10511	0.11975	0.13274	-0.35731	0.35731	0.69401	0.30998	0.63842	0.45081	0.17124	0.11875	0.08349	1.61E-06	1.61E-06	yellow
AAR2	-0.08482	-0.031	-0.08729	0.01915	-0.08363	0.00432	0.05428	0.00739	-0.00739	0.27002	0.68736	0.25626	0.80363	0.27685	0.95526	0.4807	0.92359	0.92359	turquoise
ZFX	-0.44062	-0.18631	-0.06418	0.03345	-0.07192	-0.01444	-0.12496	0.3705	-0.3705	1.63E-09	0.0147	0.40427	0.66408	0.3499	0.85135	0.10343	6.10E-07	6.10E-07	turquoise
FABP3	0.0361	-0.06202	0.10138	0.17799	-0.13248	0.15189	0.08773	0.15498	-0.15498	0.63927	0.42036	0.18704	0.01985	0.08412	0.04735	0.25385	0.04296	0.04296	blue
SRPRB	-0.00088	0.00679	-0.16815	-0.00949	-0.00625	-0.09904	0.04644	-0.01702	0.01702	0.99091	0.92979	0.02792	0.90198	0.93531	0.19748	0.54642	0.82511	0.82511	grey
DHX30	0.04671	-0.07097	-0.14878	-0.16993	0.08213	-0.09939	-0.01976	0.05474	-0.05474	0.54412	0.35634	0.05213	0.02628	0.28555	0.1959	0.79751	0.47701	0.47701	grey
TUT4	-0.13724	-0.19224	-0.05546	0.02997	-0.11957	-0.05643	-0.08002	0.43168	-0.43168	0.07345	0.01177	0.47125	0.69719	0.1193	0.46351	0.29814	3.75E-09	3.75E-09	turquoise
LILRB1	-0.12656	-0.00888	0.09779	0.05971	-0.21887	0.11937	0.04558	0.43371	-0.43371	0.09905	0.90823	0.20322	0.43788	0.00403	0.11992	0.55385	3.11E-09	3.11E-09	blue
ACTN4	0.0405	-0.10653	0.09451	0.10382	-0.13327	0.2167	0.1758	-0.25741	0.25741	0.59891	0.16552	0.21885	0.17659	0.08227	0.00441	0.02145	0.00068	0.00068	turquoise
CTXN1	0.06956	-0.08727	-0.1988	0.02207	0.15056	-0.23347	-0.03729	-0.13329	0.13329	0.36597	0.25638	0.00914	0.77445	0.04934	0.00212	0.62825	0.08222	0.08222	green
FGD2	-0.10441	0.02092	0.06081	0.05491	-0.12813	0.12029	0.06024	0.23914	-0.23914	0.17415	0.78589	0.42949	0.47568	0.0949	0.11709	0.43381	0.00163	0.00163	blue
MVK	0.10341	0.04255	-0.13654	-0.18275	0.23807	-0.16295	-0.00897	-0.26647	0.26647	0.1783	0.58058	0.07495	0.01674	0.00172	0.03321	0.9073	0.00043	0.00043	grey
DOCK2	-0.12552	-0.02757	0.09129	0.04934	-0.26174	0.16481	0.03165	0.45985	-0.45985	0.10189	0.72034	0.23502	0.52158	0.00054	0.03123	0.68113	2.50E-10	2.50E-10	blue
TBC1D8	0.08578	-0.18668	-0.09088	0.02291	0.02121	-0.04232	-0.01726	-0.03862	0.03862	0.26461	0.01449	0.23716	0.76616	0.78307	0.58264	0.82275	0.61604	0.61604	grey
COX10	0.03174	-0.05064	-0.05305	-0.08976	-0.00884	-0.07956	-0.02454	0.17328	-0.17328	0.68024	0.51072	0.49074	0.243	0.90864	0.30098	0.75	0.02342	0.02342	turquoise
ZNF284	0.04371	-0.15098	-0.08675	0.00871	0.04987	-0.0088	0.05185	-0.04476	0.04476	0.57026	0.0487	0.25922	0.91002	0.51715	0.90906	0.50061	0.56104	0.56104	red
PEBP1	2.52E-05	-0.14136	-0.17909	-0.09822	0.06147	-0.20937	-0.24857	0.30822	-0.30822	0.99974	0.06514	0.01909	0.20121	0.42447	0.00599	0.00104	4.11E-05	4.11E-05	brown
MAMDC2	-0.0641	-0.12513	-0.06896	0.05884	-0.14713	0.01736	0.01055	0.34057	-0.34057	0.40491	0.10295	0.37016	0.44456	0.05482	0.82169	0.89106	5.17E-06	5.17E-06	blue
EPHX2	-0.00641	0.02001	-0.1171	-0.02923	0.2048	-0.1727	-0.22403	0.35554	-0.35554	0.93364	0.79505	0.12719	0.70429	0.00721	0.0239	0.00322	1.82E-06	1.82E-06	brown
MFSD13A	0.05076	0.11395	-0.02065	-0.06681	0.06686	-0.07616	0.12192	-0.32257	0.32257	0.50968	0.13781	0.78859	0.38525	0.38489	0.32214	0.11217	1.69E-05	1.69E-05	grey
GFRA1	-0.03191	-0.05111	-0.05659	0.00403	-0.0506	0.00779	-0.18965	0.50852	-0.50852	0.67868	0.50677	0.46227	0.95829	0.51102	0.91944	0.01298	1.24E-12	1.24E-12	blue
COA7	-0.07905	0.02851	0.02484	0.04128	-0.10396	0.17267	0.09166	0.15924	-0.15924	0.30405	0.71131	0.74711	0.59189	0.17601	0.02392	0.23314	0.03749	0.03749	turquoise
SPIRE2	0.09986	-0.08769	0.00928	-0.10754	0.26185	-0.02305	-0.03209	-0.445	0.445	0.19378	0.2541	0.90412	0.16151	0.00054	0.76474	0.67692	1.07E-09	1.07E-09	grey
FASTKD3	-0.07297	-0.10815	-0.09574	-0.00066	0.06818	-0.22234	-0.02103	0.16406	-0.16406	0.34287	0.15914	0.21289	0.99314	0.37561	0.00347	0.78487	0.03202	0.03202	turquoise
C4orf36	-0.04036	-0.10228	-0.00411	-0.07172	-0.07213	0.10079	0.13681	-0.05347	0.05347	0.60016	0.18312	0.95741	0.35127	0.3485	0.18963	0.07437	0.48735	0.48735	turquoise
STX18	0.03883	-0.03276	-0.14385	-0.06215	0.1393	-0.02799	-0.03567	-0.15479	0.15479	0.61407	0.67055	0.06051	0.41938	0.0692	0.71633	0.64327	0.04322	0.04322	grey
GPR19	-0.00828	-0.12233	-0.15976	-0.05811	-0.00731	-0.27924	-0.02921	0.1195	-0.1195	0.91442	0.11096	0.03686	0.45026	0.92436	0.00022	0.70454	0.11952	0.11952	grey
H2AFY	-0.00578	-0.12477	-0.12991	-0.10836	0.12303	-0.13029	-0.00498	-0.16812	0.16812	0.94016	0.10394	0.09035	0.1583	0.10889	0.08942	0.94849	0.02795	0.02795	turquoise
ZNF774	0.00357	-0.16545	-0.01951	-0.00177	0.154	0.0503	0.03126	-0.15227	0.15227	0.96302	0.03057	0.80002	0.98165	0.04432	0.51355	0.68479	0.04679	0.04679	grey
ZNF354C	-0.09387	-0.12126	-0.00481	0.10568	-0.17332	0.01047	-0.0385	0.49347	-0.49347	0.22202	0.11415	0.95025	0.16894	0.02339	0.8919	0.6171	7.00E-12	7.00E-12	blue
TFG	0.04915	-0.08633	-0.06029	-0.04707	-0.06492	0.08149	0.14742	-0.24005	0.24005	0.5232	0.26157	0.43346	0.54101	0.39887	0.28936	0.05433	0.00156	0.00156	turquoise
NAP1L2	-0.05418	-0.14386	-0.26753	-0.07844	0.0811	-0.23473	-0.2793	0.5627	-0.5627	0.48151	0.06049	0.0004	0.30786	0.29165	0.002	0.00022	1.15E-15	1.15E-15	brown
SLC1A7	-0.01215	-0.08313	-0.11688	-0.0921	0.04147	0.06106	0.01085	0.26427	-0.26427	0.87464	0.2797	0.12792	0.23087	0.59019	0.42761	0.88795	0.00048	0.00048	grey
MT-CO3	0.00683	-0.05679	-0.14039	-0.09753	0.22597	-0.18129	-0.09385	-0.24222	0.24222	0.92932	0.46065	0.06703	0.20444	0.00296	0.01764	0.22212	0.00141	0.00141	grey
CSTF1	-0.01451	-0.05216	-0.11104	0.00991	-0.01451	-0.09561	0.03415	-0.00057	0.00057	0.85057	0.49806	0.14821	0.89769	0.8506	0.21353	0.65747	0.99413	0.99413	turquoise
TGFB1I1	-0.05529	-0.03819	0.03576	0.05846	-0.14451	0.13351	0.10392	-0.0066	0.0066	0.47256	0.61996	0.64242	0.44754	0.05933	0.0817	0.17619	0.93177	0.93177	black
ABI3BP	-0.07269	-0.0707	0.02607	0.11318	-0.09369	0.16804	0.00381	0.49243	-0.49243	0.34472	0.35816	0.73506	0.14051	0.22289	0.02802	0.96055	7.86E-12	7.86E-12	blue
IL1RN	0.03532	0.08073	0.13085	0.09842	-0.01034	0.26785	0.23	-0.26382	0.26382	0.6465	0.29389	0.08802	0.20031	0.89325	0.0004	0.00248	0.00049	0.00049	yellow
HDDC3	0.03267	0.02288	-0.16153	-0.08048	0.18996	-0.0969	-0.0214	-0.17716	0.17716	0.67141	0.76639	0.0348	0.29536	0.01283	0.2074	0.78115	0.02045	0.02045	grey
HECTD4	-0.00946	-0.19556	-0.07152	0.00253	0.07231	-0.12893	-0.12382	0.28498	-0.28498	0.90225	0.01037	0.3526	0.97375	0.3473	0.09284	0.10665	0.00016	0.00016	turquoise
SYT7	0.03282	-0.10708	-0.2449	-0.11375	0.08972	-0.27194	-0.0821	0.37651	-0.37651	0.67005	0.16332	0.00125	0.1385	0.24323	0.00032	0.28573	3.87E-07	3.87E-07	brown
RUBCNL	-0.08616	-0.02951	0.04787	0.04276	-0.17168	0.03655	0.06476	0.41319	-0.41319	0.2625	0.70161	0.53409	0.5787	0.02475	0.63508	0.40003	1.95E-08	1.95E-08	blue
PSMD1	-0.05394	-0.06577	0.02482	0.00319	-0.09547	0.06499	0.15738	-0.09576	0.09576	0.48353	0.39273	0.74731	0.96696	0.21419	0.39838	0.03981	0.2128	0.2128	turquoise
CDK12	-0.05336	-0.0943	-0.01391	0.017	-0.09953	0.1008	0.09858	0.10257	-0.10257	0.48824	0.21989	0.85666	0.82534	0.19523	0.1896	0.19956	0.1819	0.1819	turquoise
NAPRT	0.01569	0.03501	0.00101	-0.10614	0.04393	0.09747	0.27985	-0.45226	0.45226	0.83862	0.64939	0.98958	0.16706	0.56832	0.2047	0.00021	5.31E-10	5.31E-10	green
EPDR1	-0.11075	-0.11566	-0.02492	0.0098	-0.11287	0.08381	-0.00753	0.0991	-0.0991	0.14927	0.13196	0.74631	0.89882	0.14159	0.27577	0.92215	0.1972	0.1972	black
PARP14	-0.03927	-0.04736	0.04853	0.12341	-0.17011	0.17371	0.19391	-0.07785	0.07785	0.61008	0.53849	0.52846	0.10782	0.02612	0.02308	0.01104	0.31152	0.31152	tan
PRKAR1B	0.02414	-0.04177	-0.01987	-0.05202	0.08736	-0.20393	-0.05973	-0.00369	0.00369	0.75396	0.58748	0.79642	0.49918	0.25591	0.00747	0.43772	0.96176	0.96176	grey
MBNL1	-0.08392	-0.11735	0.00394	0.05185	-0.17073	0.14684	-0.01101	0.30754	-0.30754	0.27515	0.12636	0.95921	0.50064	0.02557	0.0553	0.88633	4.28E-05	4.28E-05	blue
MANBAL	0.07606	-0.06857	-0.10446	-0.05883	0.08407	-0.07545	0.08296	-0.3365	0.3365	0.3228	0.37283	0.17393	0.44464	0.27427	0.32671	0.28073	6.79E-06	6.79E-06	grey
OSBPL2	0.13954	-0.03557	0.00976	-0.05072	0.09752	0.08201	0.08723	-0.36337	0.36337	0.06872	0.64419	0.89915	0.51001	0.20447	0.28625	0.25658	1.04E-06	1.04E-06	grey
MOXD1	-0.03361	-0.06679	0.01008	0.05677	-0.23765	0.20734	0.04495	0.43856	-0.43856	0.66251	0.38542	0.89586	0.46081	0.00175	0.00651	0.55933	1.98E-09	1.98E-09	blue
VAT1	0.00778	-0.21019	-0.06562	0.05969	-0.12689	-0.10735	-0.02541	0.26745	-0.26745	0.91958	0.00579	0.39379	0.438	0.09816	0.16224	0.7415	0.00041	0.00041	blue
CSF3R	-0.12785	-0.01425	0.09766	0.09141	-0.15923	0.12717	0.07846	0.32535	-0.32535	0.09562	0.85328	0.20384	0.23443	0.0375	0.09741	0.30769	1.41E-05	1.41E-05	blue
AKR7A3	0.03175	0.13388	0.06399	0.05249	0.14183	0.24503	0.0027	-0.29485	0.29485	0.68016	0.08086	0.40569	0.49536	0.06425	0.00124	0.972	9.05E-05	9.05E-05	grey
HSPBP1	0.13099	0.08264	-0.06396	-0.09192	0.1706	-0.14257	0.04411	-0.39277	0.39277	0.0877	0.28259	0.07483	0.23181	0.02569	0.06285	0.56676	1.07E-07	1.07E-07	green
LYRM2	-0.09362	-0.05389	-0.0965	-0.04225	-0.02324	-0.1187	-0.06005	0.36674	-0.36674	0.22323	0.48388	0.20929	0.58318	0.76284	0.12204	0.43529	8.07E-07	8.07E-07	turquoise
MRPS35	0.03802	0.03834	-0.01014	-0.05768	0.03094	-0.01696	0.06499	-0.33218	0.33218	0.62156	0.61858	0.89527	0.45364	0.68788	0.82579	0.39838	9.05E-06	9.05E-06	turquoise
C3orf80	-0.095	-0.09608	-0.11218	-0.09106	-0.11633	-0.13576	-0.00616	0.42666	-0.42666	0.21649	0.21126	0.14407	0.23624	0.12973	0.07665	0.93622	5.92E-09	5.92E-09	blue
ADCY6	0.00894	-0.19299	-0.11832	-0.02296	0.16646	-0.08206	-0.00415	0.00758	-0.00758	0.90763	0.01144	0.12325	0.76564	0.02955	0.28597	0.95708	0.92165	0.92165	turquoise
MECR	0.00781	-0.02173	0.00212	-0.06457	-0.08653	0.063	0.12459	-0.25833	0.25833	0.91928	0.7779	0.97801	0.40141	0.26045	0.413	0.10445	0.00065	0.00065	grey
APBA1	0.00128	-0.20253	-0.15746	-0.04734	-0.01295	-0.18736	-0.12867	0.38841	-0.38841	0.98679	0.00789	0.0397	0.53862	0.86651	0.01413	0.09349	1.53E-07	1.53E-07	turquoise
HIRA	-0.04585	-0.02809	-0.22144	-0.09223	0.1686	-0.30076	-0.10239	0.11476	-0.11476	0.5515	0.71535	0.00361	0.23021	0.0275	6.41E-05	0.18265	0.135	0.135	grey
GLIS2	-0.06501	-0.14847	-0.01303	0.10875	-0.15559	0.07696	0.00158	0.29268	-0.29268	0.3982	0.05262	0.86571	0.15683	0.04214	0.31705	0.98362	0.0001	0.0001	black
CGGBP1	-0.07125	-0.18718	-0.01615	0.00987	-0.14582	0.07796	0.01963	0.21165	-0.21165	0.35444	0.01423	0.83398	0.89806	0.05704	0.31078	0.79883	0.00545	0.00545	turquoise
FGB	0.02601	-0.11728	-0.22065	-0.01799	0.09595	-0.14365	-0.1228	-0.02313	0.02313	0.73562	0.12661	0.00373	0.81531	0.21188	0.06086	0.10959	0.76393	0.76393	grey
AMY2A	-0.00413	0.05373	0.07551	0.11513	-0.1149	0.06359	-0.1012	0.16251	-0.16251	0.95728	0.4852	0.32631	0.13377	0.13454	0.40866	0.18782	0.0337	0.0337	magenta
HHLA3	-0.1446	-0.04883	-0.07161	-0.03243	-0.12515	0.01629	0.03169	0.05141	-0.05141	0.05916	0.52589	0.35198	0.67372	0.1029	0.83251	0.68072	0.50428	0.50428	grey
TEX30	-0.05699	-0.07759	-0.09718	-0.07432	0.00025	-0.21347	-0.02508	0.18538	-0.18538	0.45908	0.31312	0.20609	0.33401	0.9974	0.00506	0.7447	0.0152	0.0152	grey
PTCH2	-0.0387	-0.15948	-0.01356	0.0266	0.00402	-0.00458	-0.12234	0.35542	-0.35542	0.6153	0.0372	0.86028	0.72979	0.95836	0.95255	0.11092	1.84E-06	1.84E-06	blue
CLIC5	0.0788	-0.06048	-0.06526	0.04077	-0.12341	0.04544	0.05858	0.07335	-0.07335	0.30563	0.43197	0.39643	0.59648	0.10781	0.55512	0.44662	0.34038	0.34038	grey
SCG3	-0.00034	-0.09496	-0.25943	-0.15445	0.04521	-0.26616	-0.17215	0.4089	-0.4089	0.99646	0.21668	0.00061	0.0437	0.55709	0.00043	0.02436	2.82E-08	2.82E-08	brown
MRM2	-0.08163	-0.08435	-0.08988	-0.0189	-0.06623	-0.05307	0.05653	-0.00955	0.00955	0.28851	0.27269	0.24236	0.80613	0.38943	0.4906	0.46274	0.9013	0.9013	turquoise
TUBG1	-0.10023	-0.04882	-0.08664	-0.05554	0.10949	-0.19085	0.08508	-0.23839	0.23839	0.1921	0.52603	0.25986	0.47058	0.15401	0.01241	0.26852	0.00169	0.00169	pink
TMEM44	0.07214	-0.02091	-0.02627	0.01536	-0.01633	0.08277	0.227	-0.45304	0.45304	0.34842	0.78608	0.73306	0.84199	0.83213	0.28181	0.00283	4.92E-10	4.92E-10	yellow
YJU2	0.00543	0.13283	-0.20662	-0.12249	0.18393	-0.1791	-0.06843	-0.0762	0.0762	0.94385	0.08329	0.0067	0.11047	0.01604	0.01909	0.37386	0.32187	0.32187	green
FNTA	0.02873	-0.03192	-0.0273	0.09254	-0.0698	0.00066	0.14873	-0.07383	0.07383	0.70914	0.67854	0.723	0.22867	0.3643	0.99319	0.0522	0.33719	0.33719	turquoise
U2SURP	-0.06446	-0.1036	-0.05333	-0.02363	-0.02319	0.04891	0.06556	-0.05723	0.05723	0.40222	0.1775	0.48845	0.75901	0.76332	0.52524	0.39427	0.45716	0.45716	turquoise

TNFSF13	-0.16122	-0.02626	0.06328	0.00649	-0.06994	0.23124	0.11937	0.01156	-0.01156	0.03516	0.73319	0.41093	0.93283	0.36336	0.00234	0.11993	0.88073	0.88073	turquoise
LIMK1	0.01906	-0.1606	0.02594	0.11186	-0.19577	0.03673	0.15307	-0.07809	0.07809	0.80461	0.03587	0.73624	0.14524	0.01028	0.63336	0.04563	0.31001	0.31001	black
DCUN1D3	-0.08051	-0.14682	0.00588	0.11089	-0.18094	0.07564	0.03948	0.29232	-0.29232	0.29518	0.05533	0.93919	0.14877	0.01787	0.32548	0.60817	0.0001	0.0001	turquoise
CD300LB	-0.08102	-0.06963	0.05268	0.04435	-0.10334	0.12971	0.00937	0.47634	-0.47634	0.29214	0.36551	0.49376	0.56462	0.17861	0.09086	0.90324	4.54E-11	4.54E-11	blue
PSORS1C2	-0.01032	0.0347	0.02063	-0.0671	0.13385	0.11222	0.09571	-0.37867	0.37867	0.89347	0.65231	0.78887	0.38319	0.08092	0.14393	0.21302	3.27E-07	3.27E-07	yellow
RDM1	-0.0555	-0.01375	-0.03606	-0.02185	0.1018	-0.02414	0.19598	-0.42291	0.42291	0.47094	0.85833	0.63963	0.77667	0.18521	0.754	0.0102	8.29E-09	8.29E-09	pink
B3GALT6	-0.06385	-0.06995	-0.1096	0.02066	-0.06559	-0.11275	0.02364	0.01103	-0.01103	0.40675	0.3633	0.15358	0.78852	0.39403	0.14202	0.75889	0.88614	0.88614	grey
ZNF613	-0.06732	-0.0543	-0.10811	-0.02741	-0.07706	0.02895	-0.01168	0.15848	-0.15848	0.38168	0.48059	0.15928	0.72189	0.31647	0.70698	0.87947	0.03842	0.03842	turquoise
CHKB	-0.02932	0.0667	-0.02036	-0.02546	0.13958	-0.11607	0.03362	-0.14706	0.14706	0.70347	0.38603	0.79156	0.74099	0.06864	0.13059	0.66246	0.05494	0.05494	red
HRAS	0.10262	-0.11449	-0.13661	-0.15448	0.10036	-0.262	-0.0047	-0.27778	0.27778	0.18168	0.13593	0.07479	0.04366	0.19154	0.00054	0.95136	0.00023	0.00023	grey
IL24	-0.09962	0.00521	0.00051	0.00561	-0.12569	0.03608	-0.02098	0.38238	-0.38238	0.19485	0.94604	0.99475	0.94192	0.1014	0.63941	0.78531	2.46E-07	2.46E-07	blue
NTNG2	-0.1464	-0.02801	-0.04844	0.00757	-0.03861	-0.01828	-0.08884	0.36548	-0.36548	0.05605	0.71609	0.52924	0.92172	0.6161	0.81243	0.24787	8.86E-07	8.86E-07	blue
TRIM23	-0.04635	-0.19253	-0.10049	0.00508	-0.07995	-0.15816	-0.10665	0.4437	-0.4437	0.5472	0.01164	0.19096	0.94744	0.29861	0.03883	0.16504	1.22E-09	1.22E-09	turquoise
NBEA	-0.01793	-0.14669	-0.18742	-0.06634	-0.00478	-0.21395	-0.23078	0.48092	-0.48092	0.81593	0.05556	0.0141	0.38867	0.95058	0.00496	0.00239	2.78E-11	2.78E-11	brown
CXorf38	-0.16728	-0.08137	-0.06173	0.05098	-0.11804	0.05192	0.10263	-0.18808	0.18808	0.02876	0.29008	0.42255	0.50786	0.12414	0.50001	0.18161	0.01376	0.01376	turquoise
PSMA7	0.05995	0.01799	0.01258	0.00217	-0.03691	-0.0259	0.19627	-0.27392	0.27392	0.436	0.81538	0.87025	0.97749	0.63169	0.73663	0.01009	0.00029	0.00029	turquoise
ELP6	-0.04856	-0.05222	-0.13635	-0.15711	0.03666	-0.11422	-0.0895	0.13878	-0.13878	0.52823	0.4976	0.07536	0.04015	0.63402	0.13688	0.24439	0.07025	0.07025	grey
MRPL38	0.00432	-0.08331	-0.12224	-0.09754	0.29708	-0.13512	0.00575	-0.1896	0.1896	0.95525	0.27869	0.11122	0.20437	7.95E-05	0.07807	0.94048	0.013	0.013	red
PPWD1	-0.04186	-0.11097	-0.0136	-0.03428	0.03542	-0.05459	-0.05317	0.18181	-0.18181	0.58675	0.14849	0.85982	0.65628	0.64557	0.47823	0.48978	0.01732	0.01732	turquoise
DNAH2	0.0699	0.1344	0.0935	-0.0269	0.04018	0.08857	0.05807	-0.36634	0.36634	0.36361	0.07968	0.22386	0.72686	0.60181	0.24932	0.45063	8.32E-07	8.32E-07	grey
FAIM2	-0.01835	0.06734	-0.11441	-0.11912	-0.01127	-0.07635	-0.07328	0.28079	-0.28079	0.8117	0.38154	0.13623	0.12071	0.88372	0.32095	0.34083	0.0002	0.0002	grey
CCSER2	-0.04138	-0.1114	0.04521	0.06783	-0.223	0.0448	-0.01752	0.38198	-0.38198	0.59104	0.14689	0.55712	0.37802	0.00337	0.56066	0.8201	2.53E-07	2.53E-07	turquoise
LTBP1	-0.09819	-0.09491	0.06546	0.09989	-0.2376	0.24722	0.04126	0.18107	-0.18107	0.20134	0.2169	0.39495	0.19365	0.00175	0.00111	0.59209	0.01779	0.01779	blue
TMEM158	-0.06014	-0.05402	0.0181	0.07749	-0.04535	-0.01692	0.10423	-0.04889	0.04889	0.43455	0.48284	0.81425	0.31373	0.55589	0.82615	0.1749	0.5254	0.5254	black
DCSTAMP	-0.09987	-0.06215	0.07072	0.10061	-0.20751	0.13847	0.10226	0.31534	-0.31534	0.19373	0.41935	0.35799	0.19043	0.00646	0.07089	0.18321	2.66E-05	2.66E-05	blue
BCL9	0.06177	-0.11691	-0.15081	-0.10396	-0.01516	-0.09654	-0.03191	0.17691	-0.17691	0.42221	0.1278	0.04897	0.17601	0.84399	0.20907	0.67868	0.02063	0.02063	turquoise
ETF1	-0.04529	-0.17598	-0.03182	-0.02373	-0.09143	0.02488	0.01633	0.10743	-0.10743	0.55636	0.02131	0.6795	0.75798	0.23433	0.74667	0.83209	0.16194	0.16194	turquoise
CRABP2	0.04625	-0.0188	0.05756	0.10467	-0.06968	0.08359	0.30707	-0.25657	0.25657	0.54801	0.80723	0.45461	0.17306	0.36513	0.27708	4.41E-05	0.00071	0.00071	yellow
MAML1	-0.03028	-0.20291	-0.05922	0.01415	-0.09772	0.07295	0.00594	0.19275	-0.19275	0.69421	0.00777	0.44168	0.85427	0.20354	0.34304	0.93857	0.01155	0.01155	turquoise
TMEM115	-0.02604	-0.11128	-0.10896	-0.09791	0.01187	-0.00024	0.03102	-0.04779	0.04779	0.73528	0.14735	0.15602	0.20267	0.87755	0.99753	0.68711	0.53482	0.53482	grey
BMP5	-0.01528	-0.07397	-0.21095	-0.11783	-0.0512	-0.05099	0.03549	0.36089	-0.36089	0.8428	0.33633	0.00561	0.1248	0.50602	0.50778	0.64489	1.24E-06	1.24E-06	brown
ATPAF1	-0.07977	-0.09064	-0.11766	-0.12503	-0.02016	-0.0807	-0.13121	0.27017	-0.27017	0.29966	0.2384	0.12536	0.10323	0.79355	0.29407	0.08715	0.00035	0.00035	turquoise
KIAA1755	-0.03902	-0.10251	0.02542	0.0972	-0.18546	0.16178	0.12251	0.29912	-0.29912	0.61241	0.18215	0.74134	0.20597	0.01516	0.03452	0.11041	7.06E-05	7.06E-05	blue
FFAR1	-0.01187	-0.06239	-0.20233	-0.08521	0.02396	-0.19873	-0.0747	0.39156	-0.39156	0.87751	0.41754	0.00796	0.26783	0.75573	0.00917	0.33154	1.19E-07	1.19E-07	brown
FCHO2	-0.03251	-0.07092	0.02559	0.07175	-0.08643	0.12151	0.09136	0.06227	-0.06227	0.67294	0.35667	0.73969	0.35102	0.26101	0.11339	0.23467	0.41849	0.41849	turquoise
C1QTNF2	-0.12116	-0.0411	-0.0746	0.09618	-0.07174	0.08681	-0.00195	0.46007	-0.46007	0.11443	0.59351	0.33219	0.21081	0.35112	0.25888	0.97985	2.44E-10	2.44E-10	blue
CDC45	-0.07213	0.16832	0.0261	-0.03895	-0.01005	-0.00407	0.28336	-0.42611	0.42611	0.34849	0.02776	0.7347	0.61296	0.89618	0.95789	0.00017	6.23E-09	6.23E-09	pink
SLC6A20	0.08386	0.05102	0.11337	0.03206	-0.10234	0.30489	0.10285	-0.23246	0.23246	0.2755	0.50756	0.13985	0.67725	0.18287	5.02E-05	0.18069	0.00222	0.00222	yellow
TASOR2	-0.03	-0.1347	-0.00851	0.01353	-0.11117	0.06337	0.04797	0.21848	-0.21848	0.69691	0.079	0.91207	0.86055	0.14773	0.41028	0.53322	0.00409	0.00409	turquoise
FAM122C	-0.02908	-0.22736	-0.08233	0.00292	-0.11231	0.03221	0.08042	-0.0189	0.0189	0.70576	0.00278	0.28441	0.96976	0.14361	0.67578	0.29572	0.80622	0.80622	turquoise
CGB8	0.14597	0.05046	0.03249	0.0344	-0.05701	-0.01657	0.07284	-0.27079	0.27079	0.05678	0.51221	0.67314	0.65507	0.45895	0.82966	0.34373	0.00034	0.00034	grey
WDR43	-0.05147	0.00571	0.0603	0.05829	-0.22044	0.23432	0.20788	-0.03039	0.03039	0.50381	0.94097	0.43338	0.44887	0.00376	0.00204	0.00637	0.6932	0.6932	turquoise
STC2	-0.04156	-0.11837	-0.0392	0.09389	0.01004	-0.11612	-0.21706	0.38229	-0.38229	0.58943	0.12308	0.61068	0.22192	0.89633	0.1304	0.00435	2.47E-07	2.47E-07	grey
TMEM173	-0.027	-0.04313	0.09343	0.0534	-0.1344	0.20326	0.18569	-0.13597	0.13597	0.72591	0.5754	0.22419	0.48788	0.07968	0.00767	0.01504	0.07619	0.07619	turquoise
TMC7	0.08223	0.07391	0.14017	0.03815	0.00722	0.21802	0.1923	-0.45529	0.45529	0.28495	0.33669	0.06746	0.62036	0.92536	0.00418	0.01174	3.94E-10	3.94E-10	yellow
COQ7	-0.04843	-0.01077	-0.18295	-0.12606	0.12443	-0.11454	-0.08534	0.16935	-0.16935	0.52934	0.88883	0.01662	0.1004	0.1049	0.13576	0.26706	0.02681	0.02681	turquoise
SLC31A1	-0.05256	-0.0658	-0.03152	0.04247	-0.0766	0.04904	0.07334	0.15973	-0.15973	0.49474	0.39251	0.68239	0.58129	0.31933	0.52417	0.34042	0.03691	0.03691	turquoise
KIAA1549I	-0.00677	-0.1089	0.01116	-0.01735	-0.21139	-0.10691	0.01141	0.12728	-0.12728	0.92997	0.15624	0.88485	0.82182	0.00551	0.16401	0.88228	0.09713	0.09713	grey
BNIP1	0.04311	-0.11355	-0.15772	-0.13172	0.22934	-0.20396	-0.10216	0.06244	-0.06244	0.57557	0.13921	0.03938	0.08592	0.00255	0.00746	0.18363	0.4172	0.4172	grey
RASSF3	-0.01407	0.00169	0.07846	0.12988	-0.10349	0.27392	0.10288	-0.02326	0.02326	0.85504	0.98254	0.30771	0.09044	0.17798	0.00029	0.18055	0.76265	0.76265	turquoise
PGAP2	0.03626	-0.0314	-0.09749	-0.14525	0.09246	0.01731	0.06558	-0.38857	0.38857	0.63772	0.68346	0.20459	0.05802	0.22908	0.82224	0.39411	1.51E-07	1.51E-07	grey
TIPARP	-0.07375	-0.10625	0.06181	0.13019	-0.1233	0.2239	0.08835	-0.01179	0.01179	0.33776	0.16664	0.42192	0.08966	0.10813	0.00324	0.2505	0.87833	0.87833	turquoise
TMEM263	0.01309	-0.21453	-0.03588	-0.01698	0.05621	-0.08575	-0.09965	0.28474	-0.28474	0.86507	0.00484	0.64127	0.82552	0.46525	0.26476	0.19471	0.00016	0.00016	turquoise
TNFSF4	-0.11159	-0.06608	0.1406	0.05842	-0.22056	0.16812	0.04254	0.23594	-0.23594	0.14621	0.39051	0.06663	0.4479	0.00375	0.02795	0.58064	0.00189	0.00189	black
MISP	0.14609	0.13218	0.03183	-0.02546	0.13173	0.0916	0.13825	-0.50565	0.50565	0.05656	0.08482	0.6794	0.74095	0.08591	0.23345	0.07134	1.74E-12	1.74E-12	yellow
ADSL	-0.09644	0.06679	-0.03178	0.01053	-0.05899	0.00365	0.03763	0.08066	-0.08066	0.20956	0.38539	0.67984	0.89127	0.44344	0.96222	0.62514	0.2943	0.2943	turquoise
SENP8	-0.12582	-0.09484	-0.21001	-0.12469	-0.0152	-0.13976	-0.10017	0.24279	-0.24279	0.10107	0.21723	0.00583	0.10418	0.8436	0.06827	0.19239	0.00138	0.00138	turquoise
HIF1AN	-0.00038	-0.16092	-0.07146	-0.02087	-0.11441	-0.11029	0.03286	0.1634	-0.1634	0.99601	0.0355	0.35297	0.78645	0.13621	0.15099	0.66966	0.03272	0.03272	turquoise
TMEM234	0.00948	-0.09672	0.02639	-0.03246	0.10954	0.04713	0.07062	-0.30672	0.30672	0.90209	0.20821	0.73184	0.67343	0.1538	0.54045	0.35869	4.50E-05	4.50E-05	red
ARHGAP6	0.06027	-0.0119	0.0446	0.04071	-0.04836	0.11709	#######	0.08276	-0.08276	0.43355	0.87724	0.56247	0.59699	0.52994	0.12721	0.99996	0.28184	0.28184	grey
TMEM236	-0.01622	-0.17059	0.02391	0.01943	0.01023	-0.16992	-0.11205	0.41326	-0.41326	0.83321	0.0257	0.75627	0.80084	0.89434	0.02629	0.14453	1.94E-08	1.94E-08	brown
DNAJB11	0.0519	-0.07036	-0.10846	-0.03278	-0.05776	0.03359	0.21016	-0.32712	0.32712	0.50019	0.36049	0.15794	0.67037	0.453	0.66271	0.0058	1.26E-05	1.26E-05	turquoise
MAGEE1	-0.10373	-0.14008	-0.27269	-0.0708	-0.0009	-0.27633	-0.25216	0.55416	-0.55416	0.17698	0.06764	0.00031	0.35748	0.99069	0.00025	0.00088	3.76E-15	3.76E-15	brown
MFSD5	0.15867	-0.02843	-0.05079	0.01684	0.00144	-0.01697	0.09008	-0.26791	0.26791	0.0382	0.71209	0.50944	0.82698	0.98508	0.82565	0.24131	0.0004	0.0004	grey
CPLANE1	-0.0858	-0.19107	-0.11968	0.00956	-0.13871	-0.11996	-0.00675	0.36842	-0.36842	0.2645	0.0123	0.11895	0.90123	0.07041	0.11809	0.93022	7.13E-07	7.13E-07	turquoise
PLA2G4F	0.04826	0.05819	0.04545	0.03486	0.13159	0.13215	0.16327	-0.42502	0.42502	0.53079	0.44969	0.555	0.65082	0.08622	0.0849	0.03287	6.86E-09	6.86E-09	grey
PSMC2	-0.02228	-0.11741	-0.00346	0.00266	-0.03069	-0.0314	0.09041	-0.03519	0.03519	0.7724	0.12618	0.96415	0.97243	0.69025	0.68354	0.23957	0.64772	0.64772	turquoise
NUDCD3	-0.0178	-0.11854	-0.06494	0.02321	-0.05999	0.03068	0.06431	-0.10359	0.10359	0.81722	0.12255	0.39876	0.76321	0.4357	0.69039	0.40334	0.17754	0.17754	turquoise
CARD8	-0.11634	-0.12952	0.01235	0.09003	-0.12393	0.05951	-0.02095	0.40668	-0.40668	0.12968	0.09134	0.87258	0.24157	0.10631	0.43942	0.78565	3.40E-08	3.40E-08	blue
TPX2	0.02217	0.09496	0.068	0.00828	-0.07538	0.1299	0.34744	-0.45844	0.45844	0.77345	0.21666	0.37684	0.91441	0.32715	0.09038	3.23E-06	2.88E-10	2.88E-10	pink
SEPTIN10	-0.03603	-0.07268	0.05866	0.07824	-0.15748	0.17113	0.09905	0.01703	-0.01703	0.63991	0.34484	0.44601	0.30905	0.03968	0.02523	0.19743	0.82504	0.82504	turquoise
CNOT8	-0.014	-0.15025	-0.03068	-0.01958	-0.05729	0.0771	0.02264	0.02297	-0.02297	0.85579	0.04982	0.69033	0.7994	0.45667	0.31618	0.76877	0.76559	0.76559	turquoise
LAMC2	0.02172	0.01726	0.11597	0.06539	-0.11586	0.2435	0.24756	-0.34327	0.34327	0.77797	0.82267	0.13092	0.39547	0.13128	0.00133	0.0011	4.30E-06	4.30E-06	yellow
MCMBP	-0.0471	-0.06738	0.05002	0.01909	-0.16058	0.10306	0.11874	0.03031	-0.03031	0.54068	0.38123	0.51591	0.80425	0.0359	0.17978	0.12192	0.6939	0.6939	turquoise
CNOT11	0.08495	-0.00524	-0.00121	0.03022	0.01394	0.09535	0.1663	-0.3771	0.3771	0.26926	0.94575	0.98743	0.69476	0.85637	0.21478	0.02972	3.70E-07	3.70E-07	turquoise
FAR1	0.00249	-0.04756	0.06305	0.02032	-0.02224	0.03627	0.1331	-0.13851	0.13851	0.97423	0.53674	0.41261	0.79193	0.77279	0.63768	0.08266	0.07082	0.07082	turquoise
ZNF76	-0.07599	-0.06437	-0.15227	-0.15262	0.13963	-0.16664	-0.1657	0.18317	-0.18317	0.32326	0.40293	0.04679	0.04628	0.06855	0.02938	0.03032	0.01649	0.01649	red
SPHK2	-0.01212	0.00286	-0.20796	-0.17534	0.21001	-0.16907	-0.08507	-0.05371	0.05371	0.87502	0.97039	0.00634	0.0218	0.00584	0.02706	0.26862	0.48539	0.48539	green
TLR2	-0.13966	-0.03928	0.05147	0.10172	-0.22521	0.17845	0.15532	0.16781	-0.16781	0.06849	0.60995	0.50378	0.18556	0.00306	0.01953	0.04251	0.02824	0.02824	blue
CLCN3	-0.04302	-0.12472	-0.07425	-0.01471	0.06256	0.05256	-0.08451	0.04936	-0.04936	0.57641	0.1041	0.33446	0.84852	0.41631	0.49475	0.27177	0.52148	0.52148	turquoise
HAMP	0.03516	0.06438	0.04143	0.15213	-0.21118	0.11728	0.15822	0.09623	-0.09623	0.64796	0.40284	0.59052	0.047	0.00556	0.12661	0.03874	0.21053	0.21053	grey
CYP2C18	0.00667	0.0048	0.06183	0.10402	0.12951	0.21538	0.08548	-0.23321	0.23321	0.93101	0.95028	0.42177	0.17574	0.09137	0.00467	0.26631	0.00214	0.00214	yellow
FAM122B	0.00807	-0.15624	-0.0798	-0.07644	0.03796	-0.11426	-0.02784	-0.06181	0.06181	0.91659	0.04128	0.29952	0.32037	0.62202	0.13675	0.71779	0.42188	0.42188	red
BIN2	-0.07983	-0.01605	0.08151	0.05238	-0.18658	0.14503	0.03454	0.39829	-0.39829	0.29934	0.83497	0.28922	0.49628	0.01455	0.0584	0.6538	6.85E-08	6.85E-08	blue
MKNK1	-0.1589	-0.04816	0.06356	0.11828	-0.14625	0.04396	-0.05579	0.40255	-0.40255	0.03791	0.53167	0.40887	0.12339	0.0563	0.56807	0.46856	4.81E-08	4.81E-08	blue
METTL16	-0.06328	-0.18207	-0.187	-0.10596	-0.02833	-0.18027	-0.11441	0.43085	-0.43085	0.41091	0.01716	0.01432	0.1678	0.71296	0.01831	0.1362	4.04E-09	4.04E-09	turquoise
INO80E	0.01878	-0.01373	-0.06553	-0.05914	0.16433	-0.04133	0.0339	-0.22195	0.22195	0.80739	0.8585	0.39449	0.44225	0.03174	0.59142	0.6598	0.00353	0.00353	red
PNPT1	-0.03714	0.00367	-0.01732	0.00752	-0.0368	0.01013	0.0996	-0.08193	0.08193	0.62959	0.962	0.82208	0.92228	0.63273	0.89533	0.19495	0.28676	0.28676	turquoise
ATP8A1	-0.05357	-0.07203	-0.08293	-0.08312	0.13246	-0.1119	-0.2296	0.39353	-0.39353	0.48651	0.34918	0.2809	0.27975	0.08416	0.14507	0.00252	1.01E-07	1.01E-07	brown
DLG2	-0.02975	-0.0898	-0.13076	-0.0106	-0.0525	-0.08445	-0.12967	0.6619	-0.6619	0.69934	0.24278	0.08825	0.89053	0.49523	0.27213	0.09096	6.44E-23	6.44E-23	blue
XRCC1	0.11885	-0.01793	-0.11319	-0.12427	0.06666	-0.08764	0.03913	-0.039	0.039	0.12156	0.81591	0.14049	0.10535	0.38635	0.25436	0.61132	0.61251	0.61251	grey
LRP5	0.0108	-0.04679	0.01639	0.0312	0.07816	0.10549	0.02236	-0.32425	0.32425	0.88849	0.54339	0.8315	0.68538	0.30954	0.16968	0.77163	1.52E-05	1.52E-05	grey
SAPCD1	-0.04688	0.11884	-0.03311	-0.04315	0.25554	-0.10551	-0.01625	-0.23531	0.23531	0.54259	0.12158	0.66724	0.57518	0.00074	0.16963	0.83289	0.00195	0.00195	red
CD163L1	-0.05784	-0.02713	-0.03087	-0.02812	-0.02712	9.77E-05	0.04982	0.16102	-0.16102	0.45236	0.72462	0.6886	0.71507	0.72478	0.99899	0.51753	0.03539	0.03539	grey
ESF1	-0.04397	-0.08883	-0.00932	0.00517	-0.06245	0.02968	0.03216	0.17588	-0.17588	0.56798	0.24795	0.90372	0.94645	0.4171	0.69993	0.67627	0.02139	0.02139	turquoise
AKIP1	-0.00856	-0.04167	-0.12641	-0.07378	-0.05784	-0.12015	0.09213	-0.00799	0.00799	0.91156	0.5884	0.09946	0.33757	0.45236	0.11751	0.23075	0.91736	0.91736	turquoise
OSBP2	-0.00462	-0.03138	-0.30696	-0.06462	0.06904	-0.30338	-0.01239	-0.01557	0.01557	0.95218	0.68371	4.43E-05	0.40106	0.36955	5.49E-05	0.87218	0.83982	0.83982	grey
CXCL2	-0.20448	-0.00384	-0.0037	0.04684	-0.03793	0.15115	0.1112	0.12779	-0.12779	0.0073	0.96024	0.96173	0.54297	0.62237	0.04845	0.14762	0.09579	0.09579	grey
DDOST	-0.03195	-0.07129	0.06955	0.07203	-0.08401	0.086	0.0528	-0.03538	0.03538	0.67829	0.35412	0.36602	0.34917	0.27465	0.26336	0.49284	0.64598	0.64598	turquoise
MAK16	-0.09801	-0.11945	0.01131	0.02964	-0.10472	0.07884	0.03135	0.23468	-0.23468	0.20221	0.11966	0.88326	0.70035	0.17285	0.30538	0.68401	0.002	0.002	turquoise
SHISA3	-0.0796	-0.09948	0.09446	0.11605	-0.08432	0.15914	-0.10765	0.38902	-0.38902	0.30072	0.19548	0.21911	0.13066	0.27285	0.03761	0.16107	1.45E-07	1.45E-07	blue
LPP	-0.07406	-0.03924	0.05131	0.09278	-0.15884	0.2213	0.0835	0.09618	-0.09618	0.3357	0.61032	0.50507	0.22744	0.03798	0.00363	0.27756	0.21079	0.21079	turquoise
NT5DC3	0.11924	-0.19429	-0.07257	-0.05811	0.01473	-0.17233	-0.11454	0.15419	-0.15419	0.12032	0.01088	0.34557	0.45029	0.84833	0.0242	0.13576	0.04406	0.04406	grey
CD69	-0.11069	-0.05476	0.07437	0.0643	-0.15818	0.13658	0.01102	0.40618	-0.40618	0.1495	0.47688	0.33367	0.4034	0.03879	0.07487	0.88622	3.55E-08	3.55E-08	blue
OTUB2	0.0287	0.01658	0.03035	0.01109	0.07971	-0.00786	0.0803	-0.19961	0.19961	0.70945	0.82959	0.6935	0.88557	0.30004	0.91869	0.29647	0.00886	0.00886	grey
FARSA	0.08784	0.04293	-0.12841	-0.05376	0.12051	-0.16344	0.04937	-0.15685	0.15685	0.25326	0.57715	0.09418	0.48497	0.11642	0.03269	0.52133	0.04049	0.04049	green
KDELR2	0.012	-0.09009	0.05001	0.05642	-0.00606	0.09837	0.14266	-0.2341	0.2341	0.87623	0.24125	0.51598	0.46357	0.93728	0.20055	0.06269	0.00206	0.00206	turquoise
FOXO1	-0.06884	-0.11651	-0.04692	-0.00059	-0.26419	0.00734	-0.09257	0.44966	-0.44966	0.37097	0.12912	0.54223	0.99385	0.00048	0.9241	0.22849	6.84E-10	6.84E-10	blue
ZNF445	0.00341	-0.18574	-0.13222	-0.06145	-0.05436	-0.03156	-0.05771	0.35313	-0.35313	0.96464	0.015	0.08474	0.42463	0.48007	0.68199	0.45343	2.16E-06	2.16E-06	turquoise
CRTC2	0.08572	-0.08152	-0.0008	-0.08422	-0.02158	0.07095	0.08109	-0.21827	0.21827	0.26494	0.28913	0.99174	0.27345	0.77938	0.35644	0.2917	0.00413	0.00413	greenyellow
XG	-0.22853	-0.01898	-0.04174	-0.03593	0.08585	-0.01323	0.05535	0.32341	-0.32341	0.00264	0.80534	0.58776	0.64081	0.26423	0.86362	0.47211	1.60E-05	1.60E-05	grey
EIF1AY	0.92236	-0.03146	-0.06988	-0.02791	0.05427	-0.05769	0.01639	0.01906	-0.01906	1.04E-71	0.68296	0.36378	0.71706	0.48086	0.45358	0.83149	0.80458	0.80458	grey
MGAT4A	-0.03569	-0.13968	-0.04826	0.07332	-0.10033	-0.00593	-0.05864	0.49711	-0.49711	0.64302	0.06844	0.53079	0.3406	0.19168	0.93867	0.44612	4.64E-12	4.64E-12	blue
PACC1	-0.00447	-0.08451	-0.04903	-0.01385	-0.11628	-0.07675	0.08568	0.14263	-0.14263	0.95369	0.27177	0.5242	0.85731	0.12989	0.31842	0.2652	0.06276	0.06276	turquoise
ATL1	-0.07778	-0.19706	-0.07419	0.06791	-0.07088	-0.09022	-0.158	0.47146	-0.47146	0.3119	0.00978	0.33483	0.3775	0.35692	0.24059	0.03902	7.59E-11	7.59E-11	turquoise
SIRPA	-0.13465	-0.07089	-0.02703	0.09311	-0.14704	0.00042	0.02367	0.41422	-0.41422	0.07911	0.35683	0.72561	0.2258	0.05497	0.99567	0.75864	1.78E-08	1.78E-08	blue
RPUSD2	-0.11866	-0.0476	-0.18938	-0.09374	-0.06059	-0.1573	0.04106	-0.08969	0.08969	0.12216	0.53641	0.01311	0.22264	0.43116	0.0399	0.59386	0.24337	0.24337	grey
ZNF558	-0.05708	-0.05948	-0.05567	0.07502	0.0236	0.02464	-0.00733	-0.0438	0.0438	0.45836	0.43966	0.46958	0.32943	0.75932	0.749	0.9242	0.56943	0.56943	turquoise
SPARCL1	-0.09413	-0.07993	-0.04756	0.05645	-0.1294	0.05751	-0.07742	0.54759	-0.54759	0.22073	0.2987	0.53675	0.46336	0.09164	0.45495	0.3142	9.16E-15	9.16E-15	blue
NUDT8	0.03408	0.16369	0.08896	0.04052	0.0466	0.16752	0.20703	-0.57182	0.57182	0.65811	0.03241	0.24724	0.59878	0.54506	0.02852	0.00659	3.12E-16	3.12E-16	yellow
CD74	-0.11759	0.06554	0.02788	0.04907	-0.20964	0.18362	0.12441	0.167	-0.167	0.1256	0.39441	0.71741	0.52393	0.00592	0.01622	0.10497	0.02902	0.02902	blue
GKAP1	0.02684	-0.18036	-0.2623	-0.05605	0.18425	-0.22842	-0.24525	0.36146	-0.36146	0.72753	0.01825	0.00053	0.46651	0.01585	0.00266	0.00122	1.19E-06	1.19E-06	brown
SFI1	-0.02182	-0.03492	-0.09522	-0.09152	0.25431	-0.09789	-0.06206	-0.14645	0.14645	0.77698	0.65029	0.21538	0.23385	0.00079	0.20274	0.42	0.05596	0.05596	red
PSMD3	-0.06821	-0.10739	-0.11199	-0.0309	0.1389	-0.12919	0.04675	-0.19028	0.19028	0.37536	0.16211	0.14475	0.68828	0.07001	0.09217	0.54375	0.01267	0.01267	grey
ALDH1A1	0.08258	-0.05912	-0.01275	-0.01416	0.12458	-0.09769	-0.26149	0.29927	-0.29927	0.28291	0.44241	0.86859	0.85418	0.10448	0.20368	0.00055	7.00E-05	7.00E-05	grey
SERINC2	0.06779	-0.02398	0.13008	0.08199	-0.0042	0.27035	0.20136	-0.54559	0.54559	0.37832	0.75551	0.08992	0.28636	0.95648	0.00035	0.00827	1.20E-14	1.20E-14	yellow
ANTKMT	0.08428	0.05324	-0.11293	-0.0725	0.2319	-0.12648	0.09197	-0.33093	0.33093	0.27311	0.48919	0.14138	0.34599	0.00227	0.09927	0.23155	9.83E-06	9.83E-06	green
STOM	-0.04752	-0.15375	-0.02344	0.11319	-0.14777	0.08232	-0.02489	0.34589	-0.34589	0.5371	0.04466	0.76091	0.14046	0.05376	0.28442	0.74658	3.59E-06	3.59E-06	blue
SYNE3	-0.07146	-0.09901	-0.03606	0.02669	-0.12096	0.03868	0.00928	0.51187	-0.51187	0.35297	0.19762	0.63961	0.72897	0.11504	0.61545	0.90407	8.35E-13	8.35E-13	blue

TMEM63B	0.08396	0.01438	-0.03259	-0.02448	0.10107	0.01095	0.1801	-0.34646	0.34646	0.27495	0.8519	0.67217	0.75066	0.18841	0.88699	0.01842	3.45E-06	3.45E-06	grey
DCTN5	0.09017	-0.00231	-0.02025	-0.04435	-0.10401	0.10536	0.17415	-0.1044	0.1044	0.24086	0.97604	0.7926	0.56466	0.1758	0.17021	0.02272	0.17417	0.17417	turquoise
RACK1	0.09825	0.01719	-0.01673	-0.04793	-0.07691	0.07318	0.07167	-0.1551	0.1551	0.20108	0.82346	0.82802	0.53362	0.3174	0.34152	0.35155	0.04281	0.04281	purple
CRISP3	0.00116	-0.00789	0.0289	0.03884	0.04715	0.12699	0.04641	0.07681	-0.07681	0.98795	0.91841	0.7075	0.61397	0.54028	0.0979	0.54667	0.31801	0.31801	grey
TMEM159	0.03673	-0.05866	0.09018	0.0941	-0.09802	0.22201	0.19364	-0.32925	0.32925	0.6334	0.44598	0.24078	0.22088	0.20215	0.00352	0.01116	1.10E-05	1.10E-05	turquoise
PEAR1	-0.0984	-0.04361	-0.01437	0.05439	-0.0111	0.034	0.04782	0.25495	-0.25495	0.20041	0.57119	0.85205	0.47982	0.88545	0.65889	0.53453	0.00076	0.00076	blue
STOX2	-0.12392	-0.27679	-0.10714	0.11361	-0.0764	0.01872	-0.03432	0.26706	-0.26706	0.10635	0.00025	0.1631	0.139	0.32063	0.80804	0.65589	0.00041	0.00041	turquoise
SLC2A13	0.00706	-0.12548	-0.13346	-0.03488	0.11455	-0.12946	-0.1736	0.31694	-0.31694	0.92694	0.10199	0.08183	0.65062	0.13573	0.09149	0.02316	2.41E-05	2.41E-05	turquoise
ZNF316	0.00539	-0.13309	-0.10867	-0.03097	0.1739	-0.09716	-0.0226	-0.09487	0.09487	0.94425	0.08268	0.15714	0.68756	0.02292	0.20616	0.76923	0.21711	0.21711	red
COPS8	0.00671	-0.06481	-0.05018	0.02678	-0.11499	0.02185	0.03311	0.23684	-0.23684	0.93057	0.39968	0.51451	0.72812	0.13424	0.77671	0.66728	0.00182	0.00182	turquoise
ZKSCAN7	-0.16353	-0.17572	-0.16939	-0.02774	-0.02244	-0.12518	-0.06459	0.46749	-0.46749	0.03259	0.02151	0.02677	0.71874	0.77084	0.10281	0.4013	1.15E-10	1.15E-10	turquoise
CCHCR1	0.02345	0.08992	0.02885	0.07779	0.15136	0.09324	0.21458	-0.45625	0.45625	0.76082	0.24219	0.70801	0.31186	0.04814	0.22514	0.00483	3.58E-10	3.58E-10	green
ALG13	-0.18451	-0.16193	-0.07958	-0.05442	0.14444	-0.03474	-0.09095	0.04501	-0.04501	0.0157	0.03434	0.30084	0.47961	0.05946	0.65194	0.23677	0.5588	0.5588	red
NAMPT	-0.05653	-0.13789	0.02822	0.08278	-0.19096	0.11057	0.07384	0.05116	-0.05116	0.46273	0.0721	0.7141	0.28175	0.01235	0.14993	0.33718	0.50635	0.50635	turquoise
RPL26L1	-0.08832	-0.06683	-0.12832	-0.09933	-0.02449	-0.1482	0.07946	-0.10707	0.10707	0.25068	0.38516	0.0944	0.19616	0.75056	0.05306	0.30156	0.16334	0.16334	turquoise
CARF	-0.06479	-0.13724	-0.07303	-0.0139	0.01827	-0.02626	-0.05783	0.28089	-0.28089	0.39985	0.07347	0.34246	0.85685	0.81253	0.73312	0.45245	0.0002	0.0002	red
CEP152	-0.01709	-0.04745	0.01483	0.01912	-0.0788	0.01461	0.1509	-0.17466	0.17466	0.82444	0.53775	0.84732	0.80396	0.3056	0.84959	0.04882	0.02232	0.02232	turquoise
CD244	0.01503	-0.02033	0.07374	-0.01091	-0.07797	0.09665	0.04306	0.28	-0.28	0.84529	0.79184	0.33779	0.88734	0.31074	0.20854	0.57599	0.00021	0.00021	blue
FMOD	0.00067	0.01827	0.11219	0.09396	-0.15053	0.25318	0.10174	0.16812	-0.16812	0.99309	0.81253	0.14402	0.22158	0.04939	0.00083	0.18549	0.02795	0.02795	blue
SAA1	0.00095	0.01752	0.05191	0.11501	-0.10637	0.12132	0.04768	0.08327	-0.08327	0.99011	0.8201	0.50015	0.13414	0.16615	0.11396	0.53578	0.2789	0.2789	grey
AK4	-0.0887	-0.12476	0.09485	0.11045	-0.04265	0.23533	0.13901	-0.29726	0.29726	0.24863	0.10399	0.2172	0.1504	0.57962	0.00195	0.0698	7.87E-05	7.87E-05	turquoise
ZNF655	-0.05541	-0.23258	-0.01993	0.0384	0.07067	-0.04496	0.00539	0.07256	-0.07256	0.47161	0.00221	0.79587	0.61798	0.35838	0.55927	0.94424	0.34559	0.34559	red
MT-CYB	-0.00656	-0.11921	-0.04061	-0.01777	0.19781	-0.10362	-0.16235	-0.06648	0.06648	0.93219	0.12041	0.59795	0.81757	0.0095	0.17744	0.03387	0.38764	0.38764	grey
AHNAK	-0.03518	-0.09416	0.08753	0.09598	-0.19957	0.25294	0.13538	0.01092	-0.01092	0.64779	0.22059	0.25495	0.21176	0.00887	0.00084	0.07749	0.88731	0.88731	turquoise
SLC15A3	-0.0639	0.0282	0.08404	0.0492	-0.2131	0.15942	0.07858	0.16119	-0.16119	0.40635	0.71426	0.27444	0.52283	0.00514	0.03727	0.30695	0.03519	0.03519	blue
CLIP4	-0.11294	-0.16391	-0.04319	0.0431	-0.09242	-0.09705	-0.0187	0.19478	-0.19478	0.14137	0.03218	0.5749	0.57564	0.22925	0.20666	0.80823	0.01068	0.01068	turquoise
STAC3	-0.07707	0.07573	0.03886	-0.00258	-0.07089	0.04472	0.0376	0.14141	-0.14141	0.31637	0.32491	0.61384	0.97327	0.35683	0.56134	0.62541	0.06505	0.06505	blue
C2orf88	-0.08407	0.06569	0.15035	0.07755	0.00745	0.17517	0.02582	-0.10144	0.10144	0.27431	0.3933	0.04966	0.3134	0.92294	0.02193	0.73742	0.18679	0.18679	grey
RHOA	-0.0038	-0.03189	0.0761	-0.01818	-0.14542	0.19445	0.11042	-0.02408	0.02408	0.96063	0.6788	0.32253	0.81338	0.05772	0.01082	0.15052	0.75458	0.75458	turquoise
MYL6	0.08369	-0.09586	-0.05105	-0.00306	0.01834	-0.0731	-0.01846	-0.10666	0.10666	0.27647	0.21232	0.50728	0.96831	0.81183	0.34204	0.81061	0.16498	0.16498	grey
RAB6B	-0.07342	-0.21	-0.18231	0.00576	-0.00118	-0.18199	-0.16382	0.35443	-0.35443	0.3399	0.00584	0.01701	0.94042	0.98781	0.0172	0.03228	1.97E-06	1.97E-06	brown
HECTD3	0.00381	-0.05764	0.07192	0.04671	-0.01237	0.19238	0.13642	-0.14324	0.14324	0.96054	0.45395	0.34989	0.54405	0.87239	0.01171	0.0752	0.06162	0.06162	turquoise
PLPPR4	-0.10196	-0.17689	-0.08065	-0.08755	-0.06534	-0.08594	-0.06902	0.52892	-0.52892	0.18452	0.02064	0.29434	0.25482	0.39586	0.26371	0.3697	1.03E-13	1.03E-13	turquoise
METTL5	0.08165	0.09811	0.02719	0.04299	-0.07008	0.05287	0.14691	-0.38304	0.38304	0.28837	0.20173	0.72411	0.57662	0.36242	0.49221	0.05518	2.33E-07	2.33E-07	turquoise
HRC	-0.15894	-0.15026	-0.1184	0.08962	0.04515	-0.09755	-0.14431	0.48761	-0.48761	0.03785	0.04981	0.12299	0.24372	0.55765	0.20433	0.05969	1.34E-11	1.34E-11	blue
DOCK3	-0.05104	-0.12832	-0.17601	-0.03494	0.01596	-0.22074	-0.13351	0.29105	-0.29105	0.50736	0.0944	0.02129	0.65006	0.8359	0.00372	0.08171	0.00011	0.00011	brown
ATAD5	-0.07273	0.03477	-0.00599	-0.059	0.07893	-0.01565	0.14704	-0.14927	0.14927	0.3445	0.65169	0.93805	0.44336	0.30482	0.83904	0.05496	0.05135	0.05135	pink
GUCA1B	0.02836	-0.10375	-0.046	0.13069	0.00016	-0.05363	0.01689	0.09309	-0.09309	0.71273	0.17687	0.5502	0.08842	0.99836	0.48598	0.8264	0.22588	0.22588	grey
TBL1X	0.00572	-0.18931	-0.17959	-0.0259	-0.03827	-0.24181	-0.21369	0.36055	-0.36055	0.94085	0.01314	0.01876	0.73664	0.61925	0.00144	0.00501	1.27E-06	1.27E-06	turquoise
KIF2A	-0.0527	-0.14197	-0.00581	-0.01313	-0.13435	0.08641	0.03363	0.22235	-0.22235	0.4936	0.064	0.9399	0.86467	0.07978	0.26114	0.66237	0.00347	0.00347	turquoise
PTPN2	0.0147	-0.17245	0.06735	0.0903	-0.07244	0.12016	0.20038	-0.11786	0.11786	0.84869	0.02411	0.38146	0.24018	0.3464	0.11747	0.00859	0.12471	0.12471	turquoise
TMEM184	-0.04641	-0.14635	-0.03879	-0.01489	-0.03576	-0.09132	0.01596	-0.11024	0.11024	0.54664	0.05614	0.61445	0.84669	0.64236	0.23487	0.83591	0.15118	0.15118	grey
SEMA4G	0.03974	0.0782	0.0734	0.03334	0.20324	0.13442	0.13759	-0.33381	0.33381	0.60582	0.30929	0.34003	0.66507	0.00767	0.07963	0.07272	8.13E-06	8.13E-06	yellow
ORMDL1	-0.05724	-0.1125	-0.09069	-0.00722	0.05833	-0.11779	-0.02955	0.03915	-0.03915	0.45707	0.14291	0.23814	0.92536	0.44859	0.12494	0.70122	0.61116	0.61116	red
SPA17	-0.07317	-0.16378	-0.23504	-0.11585	0.10385	-0.25862	-0.0777	-0.06802	0.06802	0.34156	0.03232	0.00197	0.13131	0.17646	0.00064	0.31245	0.37669	0.37669	grey
DOCK8	-0.07649	-0.01575	0.05667	0.04586	-0.18006	0.15742	0.03524	0.40352	-0.40352	0.32005	0.83794	0.46159	0.5514	0.01844	0.03975	0.64726	4.44E-08	4.44E-08	blue
ZIC2	0.06939	0.08803	0.01075	-0.00349	-0.07227	0.01038	0.11223	-0.33759	0.33759	0.36717	0.25225	0.88904	0.96384	0.34758	0.89285	0.14391	6.32E-06	6.32E-06	grey
CEPT1	-0.08973	-0.1526	0.04962	0.0426	-0.06706	0.12283	-0.03154	0.15796	-0.15796	0.24318	0.04631	0.51928	0.58014	0.38352	0.10949	0.6822	0.03907	0.03907	turquoise
RABGGTA	0.04741	0.0099	-0.10102	-0.07205	0.16601	-0.1917	-0.04858	-0.14097	0.14097	0.53801	0.89771	0.18863	0.34899	0.03001	0.01201	0.52803	0.0659	0.0659	grey
POP5	0.04644	-0.00304	-0.08793	-0.12729	0.19895	-0.13234	-0.05596	-0.20797	0.20797	0.54641	0.96849	0.25278	0.09709	0.00909	0.08444	0.46721	0.00634	0.00634	grey
MOB1B	-0.11322	-0.23001	-0.15312	-0.02481	0.04606	-0.26606	-0.18616	0.41978	-0.41978	0.14036	0.00247	0.04556	0.74733	0.54968	0.00044	0.01478	1.09E-08	1.09E-08	turquoise
PLCB1	-0.00335	-0.04285	-0.05518	0.02394	-0.10122	0.03839	-0.14564	0.45364	-0.45364	0.96531	0.57791	0.47345	0.75591	0.18773	0.61813	0.05735	4.63E-10	4.63E-10	turquoise
WDFY4	-0.11219	-0.0351	0.05735	0.01661	-0.19597	0.1239	-0.01783	0.4871	-0.4871	0.14405	0.64856	0.45623	0.82926	0.01021	0.10641	0.81693	1.42E-11	1.42E-11	blue
PRKCG	0.06092	-0.05423	-0.04238	-0.03293	0.21848	-0.06164	-0.18318	0.06031	-0.06031	0.42866	0.48111	0.58204	0.66898	0.00409	0.42319	0.01648	0.4333	0.4333	grey
SCUBE2	-0.02965	0.00037	0.0096	0.07043	-0.18232	0.14805	0.03677	0.38121	-0.38121	0.70026	0.99618	0.90086	0.36001	0.017	0.0533	0.63307	2.69E-07	2.69E-07	blue
FGFBP3	-0.0202	-0.07425	-0.24557	-0.2138	0.21467	-0.3111	-0.14449	0.17941	-0.17941	0.79309	0.33446	0.00121	0.00499	0.00481	3.45E-05	0.05936	0.01888	0.01888	brown
SCAMP5	-0.04902	-0.1742	-0.24121	-0.12236	0.14457	-0.28671	-0.30667	0.39503	-0.39503	0.52428	0.02269	0.00148	0.11087	0.05922	0.00014	4.51E-05	8.95E-08	8.95E-08	brown
TMEM70	-0.0414	0.07579	0.05272	0.0064	-0.04021	-0.02089	-0.09268	0.25984	-0.25984	0.59081	0.32454	0.49348	0.93381	0.60153	0.78627	0.22793	0.0006	0.0006	grey
CCDC134	-0.00523	0.04748	0.02497	0.02641	0.03179	0.00235	0.10331	0.01567	-0.01567	0.94591	0.53743	0.74577	0.73173	0.67981	0.97565	0.17874	0.83881	0.83881	grey
GMDS	0.02505	0.16093	0.05767	-0.06734	0.13435	0.16873	0.08974	-0.36355	0.36355	0.74504	0.03548	0.45369	0.38154	0.07979	0.02738	0.24313	1.02E-06	1.02E-06	grey
AP001931	0.01091	-0.09938	-0.0216	0.04812	0.05714	0.05788	0.10417	-0.12683	0.12683	0.88742	0.19594	0.77911	0.53197	0.45791	0.45205	0.17511	0.09832	0.09832	red
TRAPPC8	-0.14279	-0.13952	-0.02024	0.07011	-0.01923	0.03143	-0.06855	0.32769	-0.32769	0.06246	0.06875	0.79273	0.36218	0.80291	0.68319	0.37296	1.21E-05	1.21E-05	turquoise
BLVRA	-0.03722	-0.06892	0.01247	0.0969	-0.16976	0.02906	0.1292	-0.04466	0.04466	0.62892	0.3704	0.87143	0.20736	0.02643	0.70594	0.09216	0.56189	0.56189	grey
TMBIM4	0.03271	-0.12586	-0.10454	0.00141	0.15373	-0.14381	-0.13956	-0.05925	0.05925	0.67103	0.10094	0.17358	0.98539	0.0447	0.06058	0.06868	0.4414	0.4414	turquoise
CSTF2	0.05341	0.02389	0.01007	-0.01739	-0.09077	0.13203	0.26484	-0.24426	0.24426	0.48785	0.75647	0.89602	0.82137	0.23774	0.08519	0.00046	0.00128	0.00128	turquoise
RGS1	-0.10052	-0.02231	0.02305	0.05543	-0.13556	0.159	0.12315	0.27347	-0.27347	0.19081	0.77208	0.76471	0.47149	0.0771	0.03779	0.10856	0.0003	0.0003	blue
AACS	0.03183	-0.07707	-0.10248	-0.06271	0.21139	-0.19725	-0.15771	-0.02484	0.02484	0.67937	0.31638	0.18227	0.4152	0.00551	0.00971	0.03939	0.74704	0.74704	turquoise
VWA2	-0.05608	-0.06909	0.11452	0.0757	-0.12558	0.24799	0.21364	-0.22213	0.22213	0.46627	0.36925	0.13583	0.32511	0.10173	0.00107	0.00502	0.0035	0.0035	grey
TYW5	0.03038	-0.1226	0.00592	0.08907	0.01283	0.11161	0.08031	-0.11725	0.11725	0.69324	0.11016	0.93876	0.24665	0.86776	0.14614	0.29639	0.12671	0.12671	turquoise
BIN1	-0.00929	-0.08808	-0.08929	0.03649	0.05655	-0.0668	0.04979	0.00313	-0.00313	0.90399	0.25196	0.24547	0.63559	0.46258	0.38533	0.51785	0.96764	0.96764	grey
HSD11B2	0.16385	0.10778	0.08105	0.0516	0.12	0.24353	0.13718	-0.37683	0.37683	0.03224	0.16057	0.29198	0.50266	0.11796	0.00133	0.0736	3.77E-07	3.77E-07	yellow
SIGLEC11	-0.11617	0.07958	0.09518	0.08003	-0.17686	0.10071	-0.00585	0.27806	-0.27806	0.13027	0.30085	0.21558	0.29809	0.02066	0.18998	0.93948	0.00023	0.00023	grey
KIF26B	-0.0736	-0.03248	0.03681	0.07454	-0.22067	0.18286	0.16619	0.13688	-0.13688	0.33874	0.67322	0.63265	0.33255	0.00373	0.01667	0.02983	0.07424	0.07424	black
LYPD1	0.04339	-0.08883	0.01626	0.15347	-0.13006	0.08998	9.55E-05	0.02288	-0.02288	0.57306	0.24792	0.83286	0.04506	0.08998	0.24184	0.99901	0.76644	0.76644	magenta
CDCA2	-0.07408	0.07464	0.08267	-0.01811	-0.10442	0.14999	0.34146	-0.36302	0.36302	0.33558	0.33193	0.28237	0.81411	0.17407	0.05022	4.87E-06	1.06E-06	1.06E-06	pink
ANKRD2	0.02575	-0.10095	-0.0412	0.01376	-0.00076	0.00275	0.01963	-0.03329	0.03329	0.73819	0.18894	0.59266	0.85822	0.99215	0.97154	0.7989	0.66553	0.66553	grey
DNER	-0.01367	-0.10058	-0.17898	-0.01825	-0.02485	-0.25318	-0.07708	0.16421	-0.16421	0.85917	0.19055	0.01917	0.81272	0.747	0.00083	0.3163	0.03186	0.03186	brown
SPPL3	0.0888	-0.15947	-0.05893	-0.00615	0.0377	-0.08872	-0.04811	-0.02087	0.02087	0.24811	0.03722	0.44387	0.93633	0.6245	0.24852	0.53207	0.78642	0.78642	turquoise
TIMM10	0.02407	0.03858	-0.03601	-0.05652	-0.03082	-0.04709	0.18463	-0.18427	0.18427	0.75469	0.61638	0.64004	0.46281	0.68902	0.54077	0.01563	0.01584	0.01584	grey
UPK3A	0.04986	0.01808	-0.03066	-0.11188	0.15164	-0.01523	-0.01922	-0.07231	0.07231	0.51723	0.81439	0.69058	0.14517	0.04771	0.84323	0.80293	0.3473	0.3473	grey
ARF4	-0.03967	-0.08053	0.04107	0.01791	-0.12293	0.20452	0.10455	-0.0502	0.0502	0.60648	0.2951	0.59378	0.81611	0.10919	0.00729	0.17357	0.51437	0.51437	turquoise
AGO2	0.00231	-0.19973	0.00291	0.02275	-0.10927	0.01379	0.0521	-0.05551	0.05551	0.97604	0.00882	0.96988	0.76769	0.15483	0.85792	0.49852	0.4708	0.4708	turquoise
CHRNA10	-0.06845	-0.06156	-0.10416	-0.04259	0.13296	-0.10084	-0.09235	0.07812	-0.07812	0.37372	0.4238	0.17517	0.58023	0.08299	0.1894	0.22962	0.30979	0.30979	grey
UPK3B	0.00373	-0.00956	0.05149	0.00245	-0.02316	0.01353	0.13369	-0.17334	0.17334	0.96135	0.90127	0.50364	0.97465	0.76363	0.86061	0.08129	0.02338	0.02338	grey
CD1B	-0.11331	-0.06938	0.05337	-0.03131	0.00552	0.12599	-0.05652	0.38579	-0.38579	0.14005	0.36722	0.48813	0.68437	0.94284	0.1006	0.46276	1.88E-07	1.88E-07	blue
LPCAT2	-0.04939	-0.03475	0.07392	0.0484	-0.23049	0.22224	0.24691	-0.15246	0.15246	0.52118	0.65188	0.33661	0.52955	0.00242	0.00348	0.00113	0.04652	0.04652	turquoise
TPM1	-0.09307	-0.07192	0.03308	0.03516	-0.11461	0.19992	0.04124	0.07008	-0.07008	0.22598	0.34987	0.66752	0.64804	0.13553	0.00875	0.59223	0.36242	0.36242	black
BAG5	-0.04773	-0.12358	-0.05449	0.00706	-0.03237	0.02957	-0.04098	0.01664	-0.01664	0.53535	0.10731	0.47905	0.927	0.6743	0.70101	0.59462	0.82903	0.82903	turquoise
SGTA	0.00769	-0.00497	-0.20399	-0.09905	0.16962	-0.17432	-0.03601	-0.12652	0.12652	0.92045	0.94852	0.00745	0.1974	0.02657	0.02259	0.64012	0.09914	0.09914	green
UBD	-0.09141	0.05998	0.04623	0.11518	-0.08714	0.19311	0.13856	-0.08613	0.08613	0.23439	0.43583	0.54826	0.13358	0.25707	0.01139	0.07071	0.26268	0.26268	grey
PTOV1	0.08891	-0.07236	-0.18929	-0.13499	0.18125	-0.26425	-0.08238	-0.17246	0.17246	0.2475	0.34694	0.01315	0.07836	0.01767	0.00048	0.28411	0.0241	0.0241	green
BNIP5	0.06115	0.04833	0.16863	0.13186	-0.02198	0.31671	0.03733	-0.19799	0.19799	0.42691	0.53014	0.02747	0.08559	0.77539	2.44E-05	0.62791	0.00944	0.00944	grey
NUP188	-0.05691	-0.1483	-0.03235	-0.03818	-0.13504	-0.03066	0.0601	0.07995	-0.07995	0.45974	0.0529	0.67447	0.62001	0.07824	0.69057	0.43488	0.29861	0.29861	turquoise
LDAH	-0.03243	-0.03349	-0.08565	0.01082	-0.07679	-0.00299	0.16855	0.05072	-0.05072	0.67374	0.66368	0.26536	0.88825	0.31817	0.96908	0.02755	0.51	0.51	turquoise
PGAP1	0.03024	-0.18506	-0.00929	-0.0065	-0.03909	-0.08003	-0.14309	0.37136	-0.37136	0.69457	0.01539	0.90403	0.93272	0.61176	0.2981	0.0619	5.72E-07	5.72E-07	turquoise
PDE4B	-0.05366	-0.07998	0.07565	0.05737	-0.13085	-0.00578	-0.08501	0.55801	-0.55801	0.4858	0.29841	0.32542	0.45611	0.08804	0.94016	0.26893	2.21E-15	2.21E-15	blue
HEATR1	-0.00993	-0.01062	0.04032	0.02411	-0.16304	0.18393	0.14077	-0.01565	0.01565	0.89747	0.89038	0.60057	0.75431	0.03311	0.01603	0.06628	0.83901	0.83901	turquoise
PRKD1	0.01059	-0.14177	-0.02398	0.05837	-0.13792	-0.06167	-0.14097	0.5083	-0.5083	0.89068	0.06436	0.75553	0.44825	0.07203	0.42295	0.06589	1.27E-12	1.27E-12	blue
TPBG	0.05662	-0.06977	0.08494	0.05594	-0.14372	0.23299	0.26416	-0.19963	0.19963	0.46197	0.3645	0.26933	0.46743	0.06075	0.00216	0.00048	0.00885	0.00885	black
PBX4	-0.00311	0.00101	0.13551	0.01706	0.08538	0.09828	0.10837	-0.26607	0.26607	0.96783	0.98955	0.07719	0.82476	0.26687	0.20096	0.15828	0.00044	0.00044	grey
CCDC146	-0.0155	-0.16001	0.00791	0.06431	0.07591	0.08954	-0.08374	-0.00053	0.00053	0.84058	0.03657	0.91822	0.40337	0.32376	0.24415	0.27617	0.9945	0.9945	red
CYCS	0.04349	0.00364	-0.0313	-0.02804	0.01982	-0.00275	0.0557	-0.1705	0.1705	0.57217	0.96236	0.68444	0.71581	0.79689	0.97152	0.46935	0.02578	0.02578	turquoise
HAUS8	0.0266	0.0182	0.01258	0.04251	-0.05989	0.01234	0.12847	-0.22077	0.22077	0.72979	0.81318	0.87025	0.58093	0.43653	0.87272	0.09403	0.00371	0.00371	grey
KCNH8	-0.01905	-0.03928	-0.00369	-0.03819	0.1097	0.10891	-0.18067	-0.1546	0.1546	0.80467	0.61001	0.96176	0.61994	0.1532	0.15621	0.01804	0.04348	0.04348	grey
ZXDA	-0.07447	-0.13785	-0.06689	-0.01374	-0.07629	0.07456	-0.04212	0.23955	-0.23955	0.33305	0.07218	0.38473	0.8584	0.32134	0.33247	0.58441	0.0016	0.0016	turquoise
SMNDC1	#######	-0.14538	0.02404	0.01102	-0.13295	0.07124	0.10376	-0.05921	0.05921	0.99975	0.0578	0.75493	0.88628	0.083	0.35447	0.17685	0.4417	0.4417	turquoise
HDGFL3	-0.08525	-0.13354	-0.19016	-0.09244	-0.12401	-0.17802	-0.10424	0.46493	-0.46493	0.26757	0.08163	0.01273	0.22915	0.10611	0.01984	0.17486	1.49E-10	1.49E-10	turquoise
KCNQ3	0.0486	-0.08079	-0.13213	-0.061	0.0066	-0.05459	-0.09375	0.14934	-0.14934	0.52785	0.29353	0.08494	0.42801	0.93176	0.47826	0.22259	0.05123	0.05123	grey
PPM1B	-0.0266	-0.15948	-0.09936	0.00624	-0.05325	0.03809	-0.03096	0.14318	-0.14318	0.72982	0.0372	0.19602	0.93542	0.4891	0.62089	0.68774	0.06173	0.06173	turquoise
ZNF799	-0.05915	-0.14185	-0.14255	-0.03378	0.1316	-0.04947	-0.1456	0.12858	-0.12858	0.4422	0.06421	0.06291	0.66098	0.0862	0.52051	0.05742	0.09372	0.09372	turquoise
FAM189A	-0.07242	-0.07918	-0.21642	-0.01688	-0.00689	-0.0559	-0.08911	0.37865	-0.37865	0.34655	0.30327	0.00447	0.82652	0.92873	0.46771	0.24643	3.28E-07	3.28E-07	grey
PRLR	0.00148	-0.12348	-0.17753	-0.13099	0.10222	-0.30446	-0.25551	0.33734	-0.33734	0.9847	0.1076	0.02018	0.0877	0.1834	5.15E-05	0.00074	6.43E-06	6.43E-06	brown
ACSM5	-0.06349	0.01948	0.02748	0.0492	-0.16859	0.07311	-0.10725	0.54995	-0.54995	0.40935	0.80033	0.72129	0.52282	0.02751	0.34194	0.16263	6.67E-15	6.67E-15	blue
CCNL1	-0.02526	-0.15681	0.007	0.02421	0.08372	0.09005	0.04188	-0.07946	0.07946	0.74299	0.04055	0.92763	0.75331	0.2763	0.2415	0.58651	0.30158	0.30158	red
WDR62	-0.00671	0.07369	0.02782	-0.02094	0.02091	0.01221	0.21182	-0.37128	0.37128	0.93054	0.33815	0.71793	0.78576	0.78607	0.87408	0.00541	5.75E-07	5.75E-07	pink
SLX4IP	0.0275	-0.10594	-0.17361	-0.01867	0.12541	-0.17149	-0.11408	0.07572	-0.07572	0.72103	0.16787	0.02315	0.80852	0.10218	0.02491	0.13736	0.32493	0.32493	turquoise
SULT2B1	0.08745	0.0473	0.05636	0.05143	0.09768	0.07404	0.13004	-0.37566	0.37566	0.25541	0.53902	0.46405	0.50409	0.20374	0.33586	0.09004	4.13E-07	4.13E-07	yellow
TMEM121	-0.01316	-0.12264	-0.1825	-0.09273	0.10666	-0.2949	-0.23018	0.40848	-0.40848	0.86439	0.11003	0.01689	0.22768	0.16497	9.02E-05	0.00246	2.92E-08	2.92E-08	brown
NOVA2	-0.03102	-0.1179	-0.23325	-0.09275	0.0903	-0.29123	-0.17799	0.50504	-0.50504	0.6871	0.12458	0.00214	0.2276	0.24017	0.00011	0.01985	1.86E-12	1.86E-12	brown
ACKR4	-0.12959	-0.01552	0.11071	0.12535	0.01356	0.20194	0.20651	-0.12095	0.12095	0.09115	0.84036	0.14943	0.10233	0.86027	0.00808	0.00673	0.11507	0.11507	grey
LRRCC1	-0.04727	-0.03348	-0.01668	0.03739	-0.15962	0.13307	0.10159	0.1028	-0.1028	0.53928	0.66373	0.82858	0.62734	0.03703	0.08273	0.18613	0.18092	0.18092	turquoise
APOO	-0.02092	-0.03692	-0.23776	-0.1379	0.11351	-0.39147	-0.08577	0.03211	-0.03211	0.78592	0.63161	0.00174	0.07207	0.13933	1.19E-07	0.26465	0.67675	0.67675	grey
MICOS13	0.0766	0.08895	-0.18053	-0.12249	0.1722	-0.17454	0.0219	-0.27507	0.27507	0.31938	0.24731	0.01813	0.11049	0.02431	0.02242	0.77619	0.00027	0.00027	green
REST	-0.02583	-0.07266	0.09227	0.13166	-0.22164	0.31585	0.21248	0.018	-0.018	0.73735	0.34494	0.23002	0.08607	0.00358	2.57E-05	0.00527	0.81527	0.81527	turquoise
KHK	-0.01331	0.06054	-0.00029	0.08257	0.07659	-0.07005	0.02228	0.04411	-0.04411	0.86279	0.43153	0.99695	0.28296	0.3194	0.36259	0.77238	0.56678	0.56678	grey
IMMP1L	0.05307	-0.10574	-0.17913	-0.111	0.15737	-0.36519	-0.17062	0.14296	-0.14296	0.49058	0.16867	0.01906	0.14838	0.03982	9.06E-07	0.02567	0.06213	0.06213	brown
COA1	-0.02852	-0.14656	0.01586	0.02704	-0.02427	-0.04655	0.0163	-0.06971	0.06971	0.71121	0.05578	0.83684	0.72556	0.75269	0.54548	0.83246	0.36496	0.36496	turquoise
POGLUT2	-0.07263	-0.09112	0.01098	0.03593	-0.20344	0.07773	0.10448	0.36356	-0.36356	0.34515	0.23589	0.88664	0.64082	0.00761	0.31222	0.17384	1.02E-06	1.02E-06	black
TMEM125	0.01228	-0.02269	-0.01666	0.04877	0.20518	0.01954	-0.10006	-0.30656	0.30656	0.87337	0.76834	0.82876	0.52647	0.0071	0.79973	0.19286	4.54E-05	4.54E-05	grey

CFH	-0.07916	-0.1166	-0.06142	0.08781	-0.15698	0.11825	0.12595	0.28115	-0.28115	0.30338	0.12883	0.42487	0.25345	0.04031	0.12347	0.1007	0.0002	0.0002	blue
LRRC70	-0.18326	-0.0876	-0.05696	0.04407	0.01632	0.02171	-0.10045	0.52732	-0.52732	0.01643	0.25455	0.45928	0.56709	0.83219	0.77806	0.19115	1.26E-13	1.26E-13	blue
PHB2	0.01708	0.03216	-0.05214	-0.02851	0.045	-0.06807	0.02034	-0.11439	0.11439	0.82457	0.6763	0.4982	0.71122	0.5589	0.37633	0.79179	0.13629	0.13629	grey
GPR17	-0.01647	-0.04773	-0.08253	-0.0186	0.12704	-0.04817	-0.13722	0.37161	-0.37161	0.83072	0.53531	0.28318	0.80921	0.09775	0.53153	0.07351	5.61E-07	5.61E-07	grey
BTNL9	-0.02962	-0.00276	-0.04829	0.02652	0.08881	0.02797	-0.13319	0.30034	-0.30034	0.70058	0.97138	0.53056	0.73057	0.24803	0.71654	0.08245	6.57E-05	6.57E-05	grey
CCAR1	-0.02742	-0.12253	-0.00499	0.032	-0.01801	0.05702	0.02575	0.0683	-0.0683	0.72188	0.11037	0.94834	0.67777	0.81512	0.4588	0.73812	0.37473	0.37473	turquoise
TMEM245	-0.01442	-0.1799	-0.07453	0.02821	-0.13495	-0.01152	-0.06631	0.22266	-0.22266	0.85147	0.01855	0.33267	0.7142	0.07843	0.88116	0.38886	0.00342	0.00342	turquoise
DPM2	0.04896	-0.01952	-0.14719	-0.0752	0.03264	-0.15513	0.04189	-0.21496	0.21496	0.52483	0.79994	0.05471	0.32831	0.67175	0.04276	0.58643	0.00475	0.00475	grey
SLC5A4	-0.25728	-0.05082	-0.09581	0.06096	0.0433	-0.07749	-0.06746	0.46024	-0.46024	0.00068	0.50916	0.21255	0.42836	0.5739	0.31372	0.38067	2.40E-10	2.40E-10	blue
MLLT6	-0.05359	-0.08799	-0.05645	-0.00633	0.0634	-0.00322	-0.0442	0.08009	-0.08009	0.48638	0.25244	0.46338	0.93447	0.41005	0.96668	0.56592	0.29774	0.29774	red
NCR3LG1	0.10603	-0.03203	-0.04128	-0.03289	-0.0048	0.12721	0.027	0.07936	-0.07936	0.1675	0.67752	0.59188	0.6693	0.95035	0.0973	0.72597	0.30217	0.30217	turquoise
CSNK2A1	0.04208	-0.0822	-0.05447	-0.05947	-0.04345	-0.03386	0.08265	-0.07779	0.07779	0.58471	0.28514	0.47919	0.4397	0.57252	0.66022	0.28248	0.31185	0.31185	turquoise
ATG4B	0.0832	-0.01889	-0.09234	-0.03611	0.19913	-0.07807	0.06262	-0.29277	0.29277	0.27928	0.80625	0.22966	0.63911	0.00903	0.31016	0.41588	0.0001	0.0001	red
VAMP3	-0.03443	-0.10342	0.03919	0.01253	-0.14829	0.15518	0.07805	0.04644	-0.04644	0.65482	0.17828	0.61085	0.87083	0.05291	0.0427	0.31025	0.54639	0.54639	turquoise
NGRN	0.0001	-0.2175	-0.12777	-0.03303	0.06253	-0.129	-0.08329	0.11916	-0.11916	0.99894	0.00427	0.09584	0.66804	0.41655	0.09265	0.2788	0.12058	0.12058	turquoise
ZNF720	0.00109	-0.11378	-0.07323	-0.0108	-0.07038	-0.0189	-0.03182	0.21869	-0.21869	0.98867	0.1384	0.34118	0.88846	0.36034	0.80614	0.6795	0.00406	0.00406	turquoise
ATP6V1D	0.05096	-0.18426	-0.06962	-0.05413	0.03798	-0.09543	-0.0278	-0.08943	0.08943	0.50803	0.01584	0.36556	0.48196	0.62191	0.21438	0.71811	0.24473	0.24473	turquoise
ING5	0.05169	-0.03268	-0.14586	-0.06251	0.16025	-0.16945	-0.04536	0.15858	-0.15858	0.50194	0.6713	0.05697	0.41665	0.03628	0.02672	0.55578	0.03831	0.03831	red
KRTAP5-1	0.10525	-0.01547	-0.02141	-0.10864	0.27449	-0.02095	-0.03308	-0.36368	0.36368	0.17069	0.84087	0.78105	0.15724	0.00028	0.78563	0.66756	1.01E-06	1.01E-06	green
ZNF79	-0.05443	-0.18792	-0.16345	-0.05066	-0.03138	-0.11874	-0.04439	0.37308	-0.37308	0.47953	0.01384	0.03268	0.5105	0.68364	0.12192	0.56427	5.02E-07	5.02E-07	turquoise
DAPK3	0.01485	-0.07835	-0.1277	-0.02403	0.0588	-0.11311	0.00145	-0.07192	0.07192	0.84709	0.30838	0.09601	0.75502	0.44488	0.14075	0.98495	0.3499	0.3499	green
CEP57L1	-0.09369	-0.12608	0.03131	0.04339	-0.1063	0.01256	0.12137	0.23538	-0.23538	0.22289	0.10034	0.68439	0.57308	0.16643	0.87052	0.11379	0.00194	0.00194	turquoise
L3MBTL1	0.02584	-0.13206	-0.08841	-0.07821	0.16485	-0.15865	-0.08751	-0.09547	0.09547	0.73724	0.08511	0.25021	0.30927	0.03119	0.03821	0.25508	0.21419	0.21419	red
SEC61A2	0.03043	-0.16269	-0.11611	-0.08926	0.12953	-0.24002	-0.13957	0.17734	-0.17734	0.69278	0.0335	0.13046	0.24567	0.09132	0.00157	0.06866	0.02032	0.02032	red
PHF20L1	-0.0315	-0.14255	-0.02023	0.05616	-0.08702	0.00544	0.0694	0.13565	-0.13565	0.68257	0.06289	0.79288	0.46563	0.25773	0.94366	0.3671	0.07689	0.07689	turquoise
FXR1	0.02531	-0.15557	-0.07331	0.0006	-0.12595	0.03448	0.11687	-0.0024	0.0024	0.74246	0.04218	0.34063	0.99375	0.10069	0.65437	0.12794	0.97511	0.97511	turquoise
GPR119	-0.06712	-0.15499	-0.1693	-0.13688	0.02623	-0.24764	-0.15051	0.43859	-0.43859	0.38308	0.04296	0.02685	0.07422	0.73344	0.00109	0.04942	1.97E-09	1.97E-09	brown
PLXDC2	-0.04159	-0.09163	0.06663	0.09597	-0.24466	0.18778	0.04967	0.42139	-0.42139	0.58913	0.23329	0.38659	0.21181	0.00126	0.01391	0.51885	9.50E-09	9.50E-09	blue
CARS2	-0.01871	0.08624	-0.10596	-0.05185	0.03506	-0.09631	0.1761	-0.25753	0.25753	0.80813	0.26207	0.16779	0.5006	0.64898	0.21015	0.02122	0.00067	0.00067	red
TMEM50B	0.04317	-0.12837	-0.12362	-0.0831	0.13168	-0.00294	-0.01201	0.09783	-0.09783	0.57502	0.09428	0.1072	0.27986	0.08601	0.96959	0.87615	0.20304	0.20304	turquoise
JAM2	-0.10048	-0.10077	-0.0246	0.10886	-0.13168	0.01204	-0.07574	0.6193	-0.6193	0.191	0.18973	0.74942	0.1564	0.08601	0.87578	0.32485	1.73E-19	1.73E-19	blue
C18orf54	-0.10731	-0.04409	-0.016	0.06583	-0.20813	0.02673	0.19087	0.21961	-0.21961	0.1624	0.56689	0.83542	0.39232	0.0063	0.72852	0.01239	0.0039	0.0039	blue
TUBGCP2	0.07096	-0.05983	-0.12549	-0.08455	0.1053	-0.18537	0.10586	-0.17151	0.17151	0.35642	0.43693	0.10195	0.27158	0.17046	0.01521	0.1682	0.02489	0.02489	green
ZNF30	-0.02362	-0.1389	-0.1824	-0.08726	0.05495	-0.15829	-0.09498	0.21358	-0.21358	0.75906	0.07001	0.01695	0.25643	0.47532	0.03865	0.21658	0.00503	0.00503	turquoise
ZNHIT3	0.00511	-0.09323	-0.13051	-0.03724	0.11982	-0.13845	6.18E-05	-0.10342	0.10342	0.94711	0.22519	0.08886	0.62865	0.11854	0.07092	0.99936	0.17827	0.17827	grey
PGBD2	-0.00858	-0.09794	0.02406	0.01852	0.04348	0.19078	0.0623	0.05519	-0.05519	0.91128	0.20251	0.75472	0.81004	0.57226	0.01244	0.41821	0.47344	0.47344	turquoise
VPS35L	-0.10556	-0.15032	-0.00777	0.07251	-0.18359	0.00382	-0.04424	0.3139	-0.3139	0.16942	0.04971	0.91962	0.34598	0.01623	0.96044	0.56558	2.90E-05	2.90E-05	turquoise
ZNF518A	0.03876	-0.05612	-0.04058	0.00184	0.04332	0.0023	-0.00702	-0.08783	0.08783	0.61473	0.466	0.59825	0.9809	0.5737	0.97622	0.92737	0.25333	0.25333	red
PIK3CA	-0.06554	-0.16306	0.00638	0.0721	-0.23603	0.15079	0.10799	0.22668	-0.22668	0.39438	0.0331	0.93403	0.34867	0.00188	0.049	0.15975	0.00287	0.00287	turquoise
NECTIN3	-0.10484	-0.11703	-0.03092	0.00823	-0.1114	0.11569	0.02588	0.04781	-0.04781	0.17234	0.1274	0.68813	0.91489	0.14689	0.13184	0.73685	0.53464	0.53464	turquoise
USP11	-0.01507	-0.19459	-0.16076	-0.06418	-0.01764	-0.26907	-0.19733	0.33794	-0.33794	0.84486	0.01076	0.03569	0.40431	0.81888	0.00037	0.00968	6.17E-06	6.17E-06	brown
UBR5	-0.03621	-0.10177	0.02379	0.07048	-0.13354	0.0415	0.06725	0.09916	-0.09916	0.6382	0.18535	0.75739	0.35967	0.08164	0.58997	0.38218	0.19695	0.19695	turquoise
METAP1D	0.00916	0.03129	-0.12438	-0.00843	-0.00923	-0.05034	0.03731	0.06745	-0.06745	0.90538	0.68452	0.10504	0.91287	0.90463	0.51322	0.62804	0.3807	0.3807	grey
GIMAP7	-0.14289	-0.0516	-0.04897	0.03383	-0.1008	0.02257	-0.10547	0.54541	-0.54541	0.06226	0.50267	0.52474	0.66049	0.18958	0.76951	0.16977	1.22E-14	1.22E-14	blue
SCNM1	0.0924	-0.00857	-0.04419	-0.08189	-0.0499	0.0236	0.19713	-0.30764	0.30764	0.22935	0.91137	0.56604	0.28697	0.51687	0.75936	0.00976	4.26E-05	4.26E-05	greenyellow
TTR	0.00038	-0.05803	-0.21215	-0.12564	0.06692	-0.18551	-0.13662	0.28775	-0.28775	0.99609	0.45093	0.00534	0.10154	0.38446	0.01513	0.07479	0.00014	0.00014	brown
NR1I2	-0.04737	0.05778	0.10448	0.0513	0.1008	0.18221	0.04496	-0.2925	0.2925	0.53839	0.45283	0.17384	0.50521	0.18958	0.01707	0.55926	0.0001	0.0001	grey
B3GNT10	-0.13905	-0.08043	-0.09698	-0.04471	-0.1514	-0.00966	0.02457	0.1936	-0.1936	0.0697	0.29565	0.20699	0.5615	0.04808	0.9002	0.74969	0.01118	0.01118	turquoise
CROT	-0.00391	-0.15239	-0.00631	0.01667	0.08038	-0.02464	0.03203	0.08599	-0.08599	0.9595	0.04661	0.93468	0.82866	0.29598	0.74909	0.67748	0.26342	0.26342	turquoise
RGS19	-0.06727	0.00533	0.01901	0.10129	-0.07679	0.05962	0.14591	0.06972	-0.06972	0.382	0.94488	0.80509	0.18742	0.31812	0.43859	0.05688	0.36488	0.36488	blue
KLK4	-0.00222	-0.07154	-0.01271	0.10236	-0.05221	-0.06741	0.1002	0.05444	-0.05444	0.97702	0.35245	0.86896	0.18279	0.49763	0.38099	0.19227	0.47946	0.47946	black
ATP5PF	-0.01642	-0.00333	-0.20311	-0.12174	0.16651	-0.21848	-0.06481	0.01066	-0.01066	0.8312	0.96552	0.00771	0.11269	0.02951	0.00409	0.39973	0.88997	0.88997	grey
COX7A2	-0.02824	0.02975	-0.11818	-0.15083	0.09599	-0.12221	0.02729	-0.19731	0.19731	0.71386	0.69931	0.1237	0.04894	0.21169	0.11131	0.72306	0.00969	0.00969	grey
LMBR1L	0.03176	-0.2457	-0.08869	0.00676	0.09867	-0.15048	-0.08613	0.05369	-0.05369	0.68006	0.0012	0.24867	0.93009	0.19915	0.04947	0.26266	0.48554	0.48554	red
GNPNAT1	-0.046	0.04247	0.11338	0.06692	-0.09613	0.19866	0.10067	-0.10787	0.10787	0.55024	0.58127	0.1398	0.38448	0.21105	0.00919	0.19018	0.16022	0.16022	turquoise
HSPB7	-0.09553	-0.04067	0.0239	0.13085	0.0171	0.12496	0.00555	0.29462	-0.29462	0.21389	0.59737	0.75633	0.08803	0.82429	0.10341	0.9426	9.17E-05	9.17E-05	salmon
CHST7	-0.06971	-0.14999	-0.12351	-0.04133	-0.02487	-0.14734	-0.13809	0.36043	-0.36043	0.36492	0.05023	0.10753	0.59148	0.74679	0.05447	0.07169	1.28E-06	1.28E-06	grey
KRT13	0.07599	0.05599	0.09689	0.04928	0.01613	0.04912	0.19872	-0.24713	0.24713	0.32324	0.46702	0.20742	0.52215	0.83411	0.52345	0.00917	0.00112	0.00112	grey
SAA2	0.00423	-0.01608	0.07208	0.03861	-0.0689	0.10868	0.03895	0.02744	-0.02744	0.9562	0.83466	0.34883	0.61611	0.37055	0.15707	0.61299	0.72162	0.72162	grey
TRAF6	-0.01332	-0.16691	0.02339	0.06956	-0.15267	0.13767	-0.0404	0.04332	-0.04332	0.86268	0.02911	0.76141	0.36597	0.0462	0.07256	0.59987	0.57369	0.57369	turquoise
STXBP3	-0.07106	-0.1166	0.04823	0.08202	-0.00468	0.09193	-0.0393	0.12654	-0.12654	0.35569	0.12884	0.53105	0.28622	0.95158	0.23175	0.60981	0.09911	0.09911	turquoise
TXNDC16	-0.03226	-0.09924	-0.06863	-0.04792	0.00184	0.00984	-0.13337	0.3457	-0.3457	0.67534	0.19654	0.37242	0.5337	0.98097	0.89838	0.08204	3.64E-06	3.64E-06	turquoise
NDUFB7	0.06962	0.09089	-0.18328	-0.07686	0.25349	-0.20491	0.01656	-0.31355	0.31355	0.36555	0.23709	0.01642	0.31768	0.00082	0.00718	0.8298	2.97E-05	2.97E-05	green
GNGT2	-0.10758	-0.00358	0.02294	0.02737	-0.21608	0.06441	0.02457	0.41175	-0.41175	0.16135	0.96292	0.7658	0.72233	0.00453	0.40264	0.74974	2.21E-08	2.21E-08	blue
CBFA2T2	0.05627	-0.15676	-0.1106	-0.06185	0.16928	-0.19821	-0.16478	0.1574	-0.1574	0.46475	0.0406	0.14986	0.42164	0.02687	0.00936	0.03126	0.03979	0.03979	turquoise
PRKN	-0.02653	-0.02975	-0.13818	-0.03157	0.05948	-0.15398	-0.18907	0.58174	-0.58174	0.73054	0.69932	0.0715	0.68185	0.43965	0.04434	0.01326	7.19E-17	7.19E-17	brown
UGCG	-0.10925	-0.2212	0.06642	0.09065	-0.17327	0.18308	0.00172	0.15025	-0.15025	0.15491	0.00364	0.38804	0.23836	0.02343	0.01654	0.98216	0.04982	0.04982	turquoise
LAG3	-0.13651	0.09006	0.00935	0.07246	-0.1118	-0.02549	0.10414	0.15622	-0.15622	0.07502	0.24142	0.90337	0.34629	0.14545	0.74066	0.17525	0.04131	0.04131	blue
GDE1	-0.02341	-0.13968	0.08768	0.06984	-0.08313	0.09239	0.12604	-0.1098	0.1098	0.76121	0.06845	0.25415	0.36405	0.27973	0.22942	0.10045	0.15284	0.15284	turquoise
ZNF264	-0.02767	-0.22965	-0.15621	-0.00452	-0.07874	-0.05074	-0.10598	0.26241	-0.26241	0.71939	0.00251	0.04133	0.95326	0.30596	0.50987	0.1677	0.00053	0.00053	turquoise
FERMT3	-0.14164	0.07454	0.13172	-0.04441	-0.08386	0.22918	0.02585	-0.00057	0.00057	0.06461	0.33257	0.08592	0.56407	0.27552	0.00257	0.73715	0.99412	0.99412	grey
TRIM16L	-0.02971	-0.04475	0.06073	0.09461	0.04248	0.02554	0.15089	-0.14116	0.14116	0.69966	0.56109	0.43008	0.21839	0.58121	0.74025	0.04884	0.06552	0.06552	grey
EMP2	-0.0787	-0.03829	-0.03968	0.01832	-0.06956	0.1301	0.23855	-0.23702	0.23702	0.30625	0.61904	0.60635	0.81201	0.36599	0.08987	0.00168	0.0018	0.0018	grey
PEPD	-0.01105	-0.00903	-0.1133	0.01282	0.0399	-0.07245	-0.04888	0.23999	-0.23999	0.88595	0.90666	0.1401	0.86781	0.60437	0.34636	0.52552	0.00157	0.00157	grey
AXL	-0.05892	-0.08169	0.04211	0.09902	-0.25536	0.1632	0.15346	0.13883	-0.13883	0.44394	0.28816	0.58443	0.19758	0.00075	0.03294	0.04507	0.07016	0.07016	black
NOC4L	0.10678	0.05209	-0.08738	-0.07348	0.17574	-0.10673	0.07908	-0.32036	0.32036	0.16452	0.49866	0.25578	0.33954	0.02149	0.1647	0.30386	1.94E-05	1.94E-05	green
FBXO32	-0.00554	-0.11233	0.04617	0.11424	-0.18493	0.1035	0.04327	0.12936	-0.12936	0.94262	0.14354	0.54876	0.13679	0.01546	0.17794	0.57414	0.09173	0.09173	black
UNC5A	-0.00541	-0.01567	-0.2522	-0.08577	0.03631	-0.21121	0.05728	0.07081	-0.07081	0.94399	0.83884	0.00088	0.2647	0.63729	0.00555	0.4568	0.35739	0.35739	grey
ASRGL1	0.01829	-0.07301	-0.02554	0.05183	0.19918	0.11116	-0.05696	0.14326	-0.14326	0.81235	0.34264	0.74017	0.50082	0.00901	0.14779	0.45929	0.06159	0.06159	grey
DDO	0.01789	-0.11178	0.06326	0.09076	0.12504	-0.03918	-0.1594	0.16462	-0.16462	0.81637	0.14552	0.41107	0.23776	0.1032	0.6109	0.0373	0.03143	0.03143	grey
ZNF672	0.0298	-0.06768	-0.19811	-0.14521	0.07333	-0.03737	0.04809	-0.07409	0.07409	0.69879	0.3791	0.00939	0.05809	0.34053	0.62747	0.53226	0.33554	0.33554	grey
EDAR	0.01198	0.08146	0.00016	0.01641	-0.07976	0.12974	0.06583	-0.15859	0.15859	0.87638	0.28951	0.99833	0.8313	0.29975	0.09079	0.39233	0.03829	0.03829	grey
ZNF205	0.11397	-0.01848	-0.1353	-0.05768	0.20591	-0.05344	0.11559	-0.34463	0.34463	0.13772	0.81038	0.07766	0.45361	0.00689	0.48757	0.13219	3.92E-06	3.92E-06	green
DTNBP1	0.09167	0.03324	-0.10466	-0.09012	0.02333	-0.08431	-0.03506	0.15867	-0.15867	0.23307	0.66602	0.1731	0.24109	0.76195	0.2729	0.64893	0.03819	0.03819	grey
BUD23	0.11361	-0.09235	-0.0078	-0.0699	0.22752	-0.18121	-0.03361	-0.38348	0.38348	0.139	0.2296	0.91932	0.36365	0.00276	0.0177	0.66249	2.25E-07	2.25E-07	grey
PSAP	0.01547	-0.09512	0.03954	0.06369	-0.18467	-0.04321	0.00674	0.41819	-0.41819	0.84081	0.21587	0.60765	0.40793	0.01561	0.57469	0.93024	1.26E-08	1.26E-08	blue
POLR3H	-0.09752	-0.02237	-0.18559	-0.04921	0.05476	-0.29186	-0.14133	0.38904	-0.38904	0.20447	0.77151	0.01509	0.5227	0.47685	0.00011	0.06521	1.45E-07	1.45E-07	brown
CTPS1	-0.04547	0.01349	0.01385	0.01771	-0.04878	-0.01692	0.11128	0.02705	-0.02705	0.55479	0.86097	0.85733	0.81818	0.52639	0.82612	0.14733	0.72548	0.72548	turquoise
NGDN	0.03252	-0.0804	-0.03375	0.01222	0.06309	-0.00678	0.03409	-0.15231	0.15231	0.67282	0.29587	0.66122	0.87396	0.41232	0.92982	0.65806	0.04673	0.04673	turquoise
TMEM150	-0.07394	0.11348	0.09235	-0.02682	0.15699	0.12594	0.05902	-0.1373	0.1373	0.33649	0.13946	0.22963	0.72767	0.04031	0.10072	0.44318	0.07333	0.07333	grey
RARG	0.11189	-0.08959	0.0027	0.04931	-0.00588	0.09243	0.17106	-0.32566	0.32566	0.14512	0.2439	0.972	0.52189	0.93912	0.22921	0.02529	1.38E-05	1.38E-05	yellow
OVOL2	0.10746	0.11704	-0.02056	-0.08653	0.17999	0.05086	0.14358	-0.50688	0.50688	0.16183	0.12738	0.78956	0.26042	0.01849	0.50888	0.061	1.50E-12	1.50E-12	yellow
SLC22A5	0.0479	-0.24015	-0.19687	0.03059	0.18936	-0.16897	-0.11818	0.02725	-0.02725	0.53387	0.00156	0.00986	0.69121	0.01312	0.02715	0.12369	0.72345	0.72345	turquoise
PREX1	-0.13174	-0.09428	-0.04114	0.03064	-0.15982	0.02005	-0.10123	0.55905	-0.55905	0.08587	0.21998	0.59316	0.69078	0.03679	0.79463	0.1877	1.92E-15	1.92E-15	blue
LUC7L2	-0.01756	-0.06361	-0.04508	-0.03605	0.0653	-0.09288	-0.0035	-0.04611	0.04611	0.81966	0.40852	0.55827	0.63972	0.3961	0.22696	0.96377	0.54927	0.54927	turquoise
C19orf44	0.11309	-0.22509	-0.22709	-0.01264	0.15559	-0.16587	-0.08504	0.08115	-0.08115	0.14083	0.00308	0.00282	0.8697	0.04214	0.03015	0.26877	0.29135	0.29135	grey
HAT1	-0.08104	-0.04084	-0.00825	0.06079	-0.182	0.07618	0.12874	0.11238	-0.11238	0.29203	0.5959	0.91471	0.42963	0.0172	0.32199	0.09331	0.14334	0.14334	turquoise
CYP2W1	0.05184	0.10358	0.0951	0.04441	0.15641	0.14547	0.02163	-0.2552	0.2552	0.50071	0.17759	0.216	0.56412	0.04106	0.05764	0.77882	0.00076	0.00076	grey
FANCA	-0.05129	0.10684	0.11748	-0.00205	-0.07805	0.13528	0.27919	-0.42574	0.42574	0.50528	0.16425	0.12596	0.97879	0.31026	0.07771	0.00022	6.44E-09	6.44E-09	pink
PPIG	-0.03035	-0.06389	0.00946	-0.00447	-0.0892	0.13244	0.02987	0.11392	-0.11392	0.69358	0.40646	0.90229	0.95374	0.24595	0.08421	0.69817	0.13793	0.13793	turquoise
BYSL	-0.02243	0.04713	-0.04637	-0.02605	-0.09326	-0.00704	0.12846	-0.05073	0.05073	0.77087	0.54043	0.54704	0.73525	0.22506	0.92715	0.09405	0.50991	0.50991	grey
SULF2	-0.02109	-0.0017	0.06387	0.13573	-0.13221	0.15374	0.19409	-0.00175	0.00175	0.78427	0.98237	0.40655	0.07672	0.08475	0.04468	0.01097	0.98185	0.98185	black
LIG1	-0.05184	0.06581	-0.07708	-0.1099	0.07403	-0.06758	0.12447	-0.25319	0.25319	0.50071	0.39242	0.31631	0.15244	0.33592	0.37979	0.10479	0.00083	0.00083	pink
METTL4	0.03006	-0.21118	-0.01293	-0.02045	0.05738	-0.01441	0.01381	0.10029	-0.10029	0.69629	0.00556	0.86673	0.79064	0.45599	0.8516	0.85773	0.19183	0.19183	turquoise
IQCD	0.10195	-0.27957	-0.15237	-0.11404	0.12655	-0.20407	-0.0599	-0.06168	0.06168	0.18455	0.00021	0.04664	0.13748	0.09906	0.00742	0.43645	0.42286	0.42286	grey
SALL2	-0.0482	-0.19675	-0.24875	-0.05363	-0.0108	-0.25692	-0.18119	0.54925	-0.54925	0.53132	0.0099	0.00104	0.48605	0.88848	0.00069	0.01771	7.32E-15	7.32E-15	brown
CRYM	0.00052	-0.12168	0.00406	-0.02951	0.26851	-0.1678	-0.19818	-0.0617	0.0617	0.99461	0.11288	0.95799	0.70164	0.00038	0.02825	0.00937	0.42273	0.42273	grey
ZNF862	-0.05943	-0.18669	-0.13249	-0.0687	0.10799	-0.22429	-0.09794	0.20586	-0.20586	0.44006	0.01449	0.08408	0.37195	0.15974	0.00319	0.20254	0.00691	0.00691	red
MLH1	-0.09422	-0.09266	-0.07499	-0.10446	-0.12562	-0.05502	-0.02255	0.28698	-0.28698	0.22028	0.22804	0.32967	0.17395	0.10159	0.47476	0.76973	0.00014	0.00014	turquoise
MPP3	-0.0015	-0.05737	-0.01293	0.12065	0.20024	0.03145	0.08271	-0.14187	0.14187	0.9845	0.4561	0.86665	0.11599	0.00864	0.68306	0.28218	0.06418	0.06418	grey
ZBTB43	-0.02057	-0.22416	-0.05796	0.04875	-0.07091	0.01924	-0.07275	0.03716	-0.03716	0.78944	0.0032	0.45146	0.52662	0.3567	0.80276	0.34437	0.62939	0.62939	turquoise
SCYL1	0.01719	-0.10043	-0.02368	-0.04085	-0.03568	-0.00405	0.16944	-0.11658	0.11658	0.82338	0.19124	0.75853	0.5958	0.64312	0.9581	0.02673	0.1289	0.1289	turquoise
STAB1	-0.12796	-0.0188	0.01627	0.03964	-0.11132	0.07679	0.11455	0.46917	-0.46917	0.09533	0.80715	0.83276	0.60671	0.14719	0.31815	0.13575	9.63E-11	9.63E-11	blue
RFLNA	-0.04793	0.08635	0.27286	0.10796	0.02507	0.30598	0.16237	-0.2982	0.2982	0.53356	0.26146	0.00031	0.15989	0.74485	4.70E-05	0.03386	7.45E-05	7.45E-05	yellow
SCAMP2	0.00734	-0.02931	-0.01281	-0.02307	0.08227	0.05572	0.07633	-0.20563	0.20563	0.92408	0.70359	0.86792	0.76452	0.28474	0.46912	0.32107	0.00697	0.00697	turquoise
LRR1	0.01998	0.03163	0.01603	0.02858	-0.05721	0.03489	0.14484	-0.24906	0.24906	0.79539	0.68133	0.83516	0.71063	0.45735	0.65053	0.05874	0.00102	0.00102	pink
RERG	-0.05591	-0.17002	-0.028	0.13008	-0.18175	0.14051	-0.0712	0.40716	-0.40716	0.46768	0.0262	0.7162	0.08993	0.01735	0.06679	0.35473	3.27E-08	3.27E-08	blue
GOLT1B	-0.09778	-0.15434	-0.00677	0.01918	-0.1044	-0.00204	0.00871	0.14523	-0.14523	0.20327	0.04384	0.92998	0.80336	0.17416	0.97888	0.91001	0.05807	0.05807	turquoise
TCF7L2	0.0791	-0.0164	0.13593	0.09591	-0.05241	0.21642	0.16132	-0.38423	0.38423	0.30375	0.83138	0.07628	0.21207	0.49601	0.00447	0.03504	2.12E-07	2.12E-07	yellow
SOWAHA	0.02872	-0.11015	-0.15633	-0.11544	0.24202	-0.20552	-0.20312	0.19659	-0.19659	0.70927	0.15152	0.04117	0.1327	0.00143	0.007	0.00771	0.00996	0.00996	brown
NEXN	-0.10898	-0.05259	0.0056	0.06185	-0.22354	0.14999	0.04063	0.46666	-0.46666	0.15594	0.49454	0.94202	0.42158	0.00329	0.05022	0.59775	1.25E-10	1.25E-10	blue
ANKRD52	0.00317	-0.21381	-0.17841	-0.02145	0.02513	-0.19099	-0.1127	0.18471	-0.18471	0.96719	0.00499	0.01956	0.7807	0.74423	0.01234	0.14221	0.01558	0.01558	turquoise
MAP7	0.09274	-0.0482	-0.00346	-0.06002	0.12805	0.00264	-0.04102	-0.1807	0.1807	0.22764	0.53129	0.96422	0.43554	0.0951	0.97265	0.59422	0.01803	0.01803	grey
CCNJL	0.05316	-0.11504	-0.02368	-0.03146	-0.10178	-0.00301	0.10776	-0.25017	0.25017	0.48983	0.13407	0.75857	0.68293	0.1853	0.96887	0.16063	0.00097	0.00097	grey
ESCO1	-0.06177	-0.16087	-0.00129	0.03038	-0.06686	0.137	0.0635	-0.03938	0.03938	0.4222	0.03556	0.98662	0.6933	0.38489	0.07398	0.40931	0.60905	0.60905	turquoise
ICAM2	-0.18522	-0.07539	-0.00166	0.00177	-0.04873	0.07562	-0.06879	0.23112	-0.23112	0.0153	0.32708	0.98286	0.98166	0.5268	0.32562	0.37129	0.00235	0.00235	blue
ANKIB1	-0.05458	-0.1984	0.04498	0.09683	-0.11648	0.09686	0.04511	0.05219	-0.05219	0.47829	0.00929	0.55909	0.20771	0.12922	0.20758	0.55793	0.49784	0.49784	turquoise
MBLAC1	0.04243	-0.08119	-0.16825	-0.12915	0.22329	-0.26234	-0.14032	-0.02391	0.02391	0.58158	0.29114	0.02783	0.09229	0.00333	0.00053	0.06716	0.75624	0.75624	grey
NOP10	-0.04101	-0.0224	0.02707	-0.03416	-0.10815	0.11841	0.17478	-0.3708	0.3708	0.59435	0.77124	0.72529	0.65733	0.15911	0.12296	0.02223	5.96E-07	5.96E-07	grey

SIGLEC15	0.01169	-0.03978	0.06936	0.05988	0.00324	0.04167	0.1709	-0.18635	0.18635	0.87934	0.60542	0.36738	0.43661	0.96649	0.5884	0.02543	0.01467	0.01467	grey
TMEM17	0.01306	-0.1599	-0.225	-0.10964	0.0654	-0.35702	-0.11372	0.2316	-0.2316	0.86539	0.0367	0.00309	0.15344	0.3954	1.64E-06	0.13861	0.0023	0.0023	brown
SETBP1	-0.08046	-0.15733	-0.12663	-0.00964	-0.07704	-0.03825	-0.1876	0.64063	-0.64063	0.29552	0.03987	0.09886	0.90037	0.31659	0.61937	0.01401	3.88E-21	3.88E-21	turquoise
TMEM30B	0.01963	-0.11521	0.05549	0.08515	0.06977	0.15035	0.05071	-0.29187	0.29187	0.79885	0.13347	0.47101	0.26815	0.36452	0.04966	0.51006	0.00011	0.00011	turquoise
N4BP3	0.10662	-0.10119	-0.02656	0.0089	0.06733	-0.00225	0.08168	-0.20978	0.20978	0.16515	0.18787	0.73027	0.90807	0.38156	0.9767	0.28819	0.00589	0.00589	grey
ITPRIPL1	-0.16836	-0.01344	0.04326	0.08139	-0.20557	0.07828	0.07007	0.39478	-0.39478	0.02773	0.86154	0.57422	0.28993	0.00699	0.30882	0.36249	9.13E-08	9.13E-08	blue
UBE4B	-0.10848	-0.15337	0.01564	0.06285	-0.15158	0.11178	0.02732	0.23341	-0.23341	0.15784	0.04521	0.83915	0.41412	0.0478	0.1455	0.72281	0.00212	0.00212	turquoise
CELA3B	0.01497	0.05333	0.06724	0.13223	-0.10247	0.08554	-0.08307	0.12889	-0.12889	0.84593	0.48843	0.3822	0.0847	0.18232	0.26594	0.28006	0.09293	0.09293	magenta
CKMT1A	0.02191	0.06197	-0.04153	-0.10125	0.16949	-0.06622	-0.04639	-0.36462	0.36462	0.7761	0.42072	0.58968	0.18759	0.02668	0.3895	0.54684	9.44E-07	9.44E-07	grey
TMEM33	0.014	-0.06743	-0.10553	-0.04562	0.03974	-0.0784	0.02684	-0.06594	0.06594	0.85579	0.3809	0.16954	0.55355	0.60585	0.30809	0.72745	0.39147	0.39147	turquoise
SEMA5B	-0.19355	-0.05961	-0.0517	0.08058	-0.00389	-0.00163	-0.04982	0.2997	-0.2997	0.0112	0.43868	0.50184	0.29479	0.95977	0.98317	0.51755	6.83E-05	6.83E-05	grey
HOXA4	0.06357	-0.05205	0.06223	0.12242	-0.0701	0.17862	0.12358	0.20102	-0.20102	0.40882	0.49894	0.41878	0.11068	0.36227	0.01942	0.10732	0.00838	0.00838	blue
CRCP	0.04873	-0.10479	-0.00479	-0.05253	0.03139	-0.01987	-0.00852	-0.13241	0.13241	0.52679	0.17255	0.95043	0.49498	0.68359	0.79641	0.91196	0.08427	0.08427	turquoise
EBF3	-0.12819	-0.08799	-0.04774	0.05202	-0.11196	-0.0039	-0.04872	0.61627	-0.61627	0.09475	0.25245	0.5352	0.49923	0.14486	0.9596	0.52687	2.90E-19	2.90E-19	blue
FBRS	0.03372	-0.11799	-0.08838	-0.04701	0.06522	-0.04686	0.00103	-0.11765	0.11765	0.66147	0.12428	0.25034	0.54148	0.39671	0.5428	0.98928	0.12541	0.12541	green
SHQ1	-0.08499	-0.01967	-0.04375	-0.00615	-0.09389	0.10166	0.05343	0.14795	-0.14795	0.26907	0.79848	0.56993	0.93637	0.22192	0.1858	0.48763	0.05346	0.05346	turquoise
WDR5B	-0.09354	-0.21165	-0.18783	-0.08502	0.0596	-0.08423	0.04829	0.02081	-0.02081	0.22364	0.00545	0.01389	0.26888	0.43875	0.27337	0.5305	0.78709	0.78709	turquoise
PCTP	0.01065	-0.10458	-0.03024	0.08987	-0.05403	-0.01825	0.10335	-0.15773	0.15773	0.89008	0.17344	0.69458	0.24244	0.4828	0.8127	0.17857	0.03936	0.03936	grey
SLC38A7	0.02494	-0.0536	-0.06578	-0.02238	-0.10418	0.06933	0.18516	-0.03062	0.03062	0.74608	0.48629	0.39266	0.77139	0.17511	0.36754	0.01533	0.69091	0.69091	turquoise
SMIM26	0.10069	-0.03096	-0.13602	-0.09699	0.10217	-0.17946	0.06609	-0.19494	0.19494	0.19009	0.68773	0.07609	0.20693	0.1836	0.01884	0.39046	0.01062	0.01062	grey
SCCPDH	0.05934	-0.1221	-0.04897	-0.0291	0.04852	0.00839	0.20664	-0.2719	0.2719	0.4407	0.11164	0.52478	0.70552	0.52856	0.91331	0.00669	0.00032	0.00032	grey
SLC9A2	-0.01298	0.04506	0.13814	0.03406	-0.02279	0.20518	0.13698	-0.20038	0.20038	0.86623	0.55838	0.07157	0.65829	0.76731	0.0071	0.07402	0.00859	0.00859	grey
ARID4A	0.00192	-0.14449	0.03032	0.04198	-0.09024	0.13898	0.0225	0.16982	-0.16982	0.98007	0.05935	0.69386	0.58566	0.24049	0.06985	0.77025	0.02638	0.02638	turquoise
OFD1	-0.24371	-0.20259	-0.04651	-0.03373	0.0858	0.01182	-0.02917	-0.17285	0.17285	0.00132	0.00788	0.54578	0.66139	0.2645	0.87803	0.70491	0.02377	0.02377	red
ZNF865	0.11801	-0.03208	-0.10777	-0.05412	0.04871	0.01845	0.0981	-0.29025	0.29025	0.12424	0.67705	0.16059	0.48207	0.52691	0.81066	0.20179	0.00012	0.00012	green
AP3B2	-0.01506	-0.16489	-0.24621	-0.15698	0.15503	-0.33498	-0.2292	0.40423	-0.40423	0.84497	0.03115	0.00117	0.04032	0.04291	7.52E-06	0.00257	4.18E-08	4.18E-08	brown
DCUN1D5	-0.02148	-0.08003	-0.06015	-0.01925	-0.08818	-0.04225	0.24323	-0.06466	0.06466	0.78038	0.29811	0.43452	0.80271	0.25143	0.58325	0.00135	0.40079	0.40079	grey
ARMC6	0.00903	0.07361	-0.21165	-0.12746	0.14096	-0.24482	0.01242	0.05516	-0.05516	0.90666	0.33868	0.00545	0.09665	0.06593	0.00125	0.87186	0.47366	0.47366	green
SNAP25	0.04683	-0.13271	-0.27373	-0.09696	0.17183	-0.29703	-0.24767	0.41654	-0.41654	0.543	0.08357	0.00029	0.20709	0.02462	7.98E-05	0.00109	1.46E-08	1.46E-08	brown
ACLY	-0.02611	-0.15995	-0.13665	-0.06685	0.02399	-0.16031	-0.12812	0.32161	-0.32161	0.73464	0.03664	0.07471	0.38499	0.75543	0.03622	0.09493	1.79E-05	1.79E-05	turquoise
MLEC	0.08248	-0.06791	-0.03577	0.03317	0.05366	-0.0971	0.0276	0.11601	-0.11601	0.28348	0.37748	0.64233	0.6667	0.48575	0.20643	0.72013	0.13078	0.13078	turquoise
GREB1	-0.01367	-0.07558	-0.08864	0.09178	-0.20627	-0.00462	0.0063	0.49723	-0.49723	0.85911	0.32587	0.24894	0.2325	0.0068	0.9522	0.9348	4.58E-12	4.58E-12	blue
KCNAB3	-0.13066	-0.12466	-0.03779	0.00935	0.1083	-0.11286	0.01513	0.1247	-0.1247	0.0885	0.10427	0.62361	0.90342	0.15854	0.14163	0.84429	0.10416	0.10416	grey
RHOT2	0.04819	0.03544	-0.04719	-0.05512	0.20448	-0.0558	0.05365	-0.28723	0.28723	0.53138	0.64541	0.53998	0.47398	0.0073	0.46849	0.48588	0.00014	0.00014	green
ENDOD1	-0.0345	-0.11541	0.00238	0.02173	-0.20967	0.07136	0.17438	-0.11998	0.11998	0.65419	0.13279	0.97539	0.7779	0.00592	0.35365	0.02254	0.11804	0.11804	turquoise
HAPLN1	-0.06111	-0.05716	0.00123	-0.05356	-0.0539	0.02351	0.10756	-0.17883	0.17883	0.42721	0.45775	0.98721	0.48661	0.48381	0.76023	0.16141	0.01927	0.01927	grey
HSP90AB1	0.15555	-0.07774	-0.03291	-0.01211	-0.02017	-0.05668	0.0221	-0.10885	0.10885	0.04221	0.3122	0.66916	0.87512	0.79342	0.46151	0.77421	0.15645	0.15645	turquoise
TMEM185	-0.02629	-0.02512	0.03846	0.11979	-0.19496	0.1446	0.19554	-0.01199	0.01199	0.7329	0.74437	0.6175	0.11862	0.01061	0.05916	0.01038	0.87631	0.87631	turquoise
CACNB2	0.00088	-0.11146	-0.17491	-0.06385	0.15608	-0.28217	-0.28624	0.47559	-0.47559	0.99091	0.14666	0.02213	0.40673	0.0415	0.00018	0.00015	4.91E-11	4.91E-11	brown
GLTP	-0.04818	-0.17467	0.10899	0.07903	-0.07168	0.07164	0.06755	0.03373	-0.03373	0.53145	0.02231	0.15589	0.3042	0.35149	0.35177	0.37999	0.66141	0.66141	turquoise
RPL22	0.04671	0.02413	-0.00356	0.00238	-0.07819	0.16144	0.07832	-0.02787	0.02787	0.54407	0.75405	0.96312	0.97538	0.30939	0.0349	0.3086	0.71743	0.71743	purple
PWWP3B	0.01736	-0.13903	-0.10125	0.0607	-0.10474	0.06528	0.00358	0.34623	-0.34623	0.82174	0.06975	0.18759	0.43031	0.17276	0.39624	0.96291	3.51E-06	3.51E-06	blue
TRIM11	0.15363	0.01585	-0.06155	-0.07747	0.08379	-0.00324	0.19349	-0.48105	0.48105	0.04484	0.83695	0.42386	0.31385	0.2759	0.96647	0.01122	2.74E-11	2.74E-11	greenyellow
TBPL1	-0.0031	-0.12059	-0.08868	-0.06483	0.07209	-0.06035	-0.1094	0.17792	-0.17792	0.96789	0.11616	0.24875	0.39953	0.34875	0.43297	0.15435	0.0199	0.0199	turquoise
METTL21A	-0.05075	-0.06105	0.02721	0.08131	-0.02365	0.09548	0.13721	-0.04799	0.04799	0.50973	0.42767	0.72389	0.29038	0.75878	0.21415	0.07352	0.53309	0.53309	turquoise
ABHD18	-0.06534	-0.16495	-0.01919	-0.03493	0.09731	0.01485	-0.10504	0.07426	-0.07426	0.39581	0.03108	0.80329	0.65019	0.20546	0.84712	0.17153	0.33443	0.33443	turquoise
MADCAM	0.02651	-0.11606	-0.08403	0.02226	-0.01862	-0.02867	-0.04474	0.31483	-0.31483	0.73074	0.13061	0.27453	0.77258	0.80903	0.70976	0.56122	2.74E-05	2.74E-05	blue
ZNF280B	-0.12827	-0.10908	-0.12126	-0.01922	0.00585	-0.21336	-0.11878	0.37393	-0.37393	0.09454	0.15554	0.11412	0.803	0.93945	0.00508	0.12179	4.71E-07	4.71E-07	brown
CBLB	0.0202	-0.15237	0.04253	0.01586	-0.18876	0.18217	0.04742	0.12998	-0.12998	0.79312	0.04665	0.58069	0.83684	0.01342	0.01709	0.53795	0.09018	0.09018	turquoise
GRK6	-0.08721	-0.09531	-0.01566	-0.08014	-0.07573	-0.07534	-0.00559	-0.04579	0.04579	0.25672	0.21495	0.83893	0.29742	0.32491	0.32739	0.94211	0.55208	0.55208	grey
CDH23	-0.07352	-0.04694	0.13486	0.21908	-0.1487	-0.09686	0.01649	0.48234	-0.48234	0.33925	0.54211	0.07864	0.00399	0.05225	0.20758	0.83054	2.39E-11	2.39E-11	blue
ATP6V1B2	-0.04672	-0.07799	-0.02093	-0.00316	-0.06878	-0.09966	-0.03103	0.45828	-0.45828	0.54401	0.31063	0.78589	0.96725	0.37142	0.19465	0.68699	2.92E-10	2.92E-10	turquoise
ERFL	-0.02261	-0.02866	0.03989	-0.10998	0.066	0.03448	-0.03355	-0.2027	0.2027	0.76912	0.70981	0.60447	0.15215	0.39105	0.65439	0.66311	0.00784	0.00784	grey
FIZ1	0.07501	-0.07175	-0.24037	-0.10629	0.16775	-0.15729	-0.09338	-0.07615	0.07615	0.32951	0.35105	0.00154	0.16648	0.02831	0.03992	0.22443	0.32222	0.32222	green
CBWD1	0.06654	-0.02566	-0.06293	-0.06182	-0.00131	-0.09108	0.12094	-0.06765	0.06765	0.38718	0.73901	0.41354	0.42182	0.9864	0.2361	0.11509	0.37934	0.37934	grey
GIT1	0.0115	-0.08092	-0.1399	-0.04629	0.11528	-0.18102	-0.0156	-0.0738	0.0738	0.88128	0.29272	0.06799	0.54772	0.13326	0.01782	0.83952	0.33744	0.33744	grey
ZDHHC11	0.02346	-0.04347	-0.01555	-0.01362	0.29849	-0.07674	-0.07456	-0.22572	0.22572	0.76071	0.57241	0.84005	0.85964	7.33E-05	0.31847	0.33244	0.00299	0.00299	grey
LYPD3	0.0683	-0.05977	0.03309	-0.05317	0.01507	-0.02658	0.09322	-0.17263	0.17263	0.37475	0.4374	0.66748	0.48973	0.84494	0.72999	0.22523	0.02396	0.02396	grey
CHEK2	-0.03234	0.08124	0.0837	0.06001	-0.06089	0.13583	0.26091	-0.22166	0.22166	0.67452	0.29082	0.27642	0.43556	0.42888	0.07649	0.00057	0.00357	0.00357	pink
DVL3	0.0099	-0.19598	-0.07845	0.01261	-0.09069	0.05678	0.07899	0.04945	-0.04945	0.8977	0.0102	0.30776	0.87001	0.23814	0.46069	0.30445	0.52072	0.52072	turquoise
IRF2	-0.07366	-0.08196	0.01675	0.04668	-0.02337	0.12016	-0.02895	0.02649	-0.02649	0.33833	0.28656	0.82789	0.5443	0.76158	0.11749	0.70698	0.73094	0.73094	turquoise
RAI1	0.00178	-0.21781	-0.10411	-0.01648	0.05122	-0.09247	-0.02028	0.03947	-0.03947	0.98152	0.00421	0.17537	0.83059	0.50582	0.229	0.79239	0.6083	0.6083	turquoise
CAPN9	0.00754	0.08285	0.06748	-0.02944	0.08709	0.19023	0.07933	-0.22191	0.22191	0.92204	0.28132	0.38049	0.70231	0.25739	0.0127	0.30236	0.00353	0.00353	yellow
MDM1	0.01888	-0.14289	-0.13617	0.04357	0.11786	-0.13848	-0.16433	0.26633	-0.26633	0.80639	0.06226	0.07575	0.57148	0.12473	0.07087	0.03173	0.00043	0.00043	turquoise
GFM1	-0.05711	-0.00963	-0.07581	-0.00066	-0.08719	0.0626	0.11966	0.06978	-0.06978	0.45814	0.90055	0.32437	0.99315	0.25679	0.41597	0.11902	0.36445	0.36445	turquoise
ARHGEF1(	-0.03366	-0.11533	-0.0833	0.03741	0.21495	-0.06867	-0.156	0.05028	-0.05028	0.66211	0.13306	0.27874	0.62715	0.00475	0.37216	0.0416	0.51366	0.51366	grey
NEK1	-0.06962	-0.12631	-0.08152	-0.01967	-0.09773	-0.05338	-0.1187	0.52278	-0.52278	0.36554	0.09972	0.28916	0.79848	0.20348	0.48803	0.12205	2.22E-13	2.22E-13	turquoise
ITSN1	0.01023	-0.19703	0.03846	0.05737	-0.07451	0.11835	0.04182	0.37494	-0.37494	0.89432	0.00979	0.61749	0.45608	0.33277	0.12315	0.58702	4.36E-07	4.36E-07	blue
IFT27	-0.03461	-0.03597	-0.2291	-0.10953	0.27722	-0.25369	-0.07804	-0.1031	0.1031	0.65312	0.64042	0.00258	0.15385	0.00024	0.00081	0.31028	0.17962	0.17962	grey
ZNF85	-0.03466	-0.0297	-0.07988	-0.02222	-0.08151	-0.09867	-0.0706	0.36281	-0.36281	0.6527	0.69975	0.29902	0.77295	0.28925	0.19916	0.35885	1.08E-06	1.08E-06	grey
LAPTM4A	-0.08214	-0.09267	0.05138	0.09967	-0.11034	0.16273	0.06625	0.10049	-0.10049	0.28549	0.22802	0.50454	0.19462	0.15081	0.03345	0.38931	0.19097	0.19097	turquoise
PPP1R13B	0.075	-0.11734	-0.04905	0.05061	0.21799	0.061	-0.01019	-0.11026	0.11026	0.32957	0.1264	0.52405	0.51092	0.00418	0.42807	0.89481	0.15109	0.15109	grey
GET1	-0.08515	-0.18243	-0.22037	-0.04612	0.21344	-0.28598	-0.20261	0.35122	-0.35122	0.26816	0.01693	0.00378	0.54922	0.00506	0.00015	0.00787	2.48E-06	2.48E-06	turquoise
TRMU	0.02284	0.00509	-0.15722	-0.06625	0.25613	-0.30602	-0.02243	-0.11676	0.11676	0.76683	0.9473	0.04001	0.38931	0.00072	4.69E-05	0.77088	0.12831	0.12831	red
OXSM	-0.04122	-0.10185	-0.07192	-0.09445	0.08092	0.03925	0.12412	-0.30756	0.30756	0.59243	0.18498	0.34987	0.21917	0.29276	0.6103	0.1058	4.28E-05	4.28E-05	grey
COL21A1	-0.12842	-0.09304	-0.03863	0.05514	-0.14266	0.13419	-0.02565	0.47179	-0.47179	0.09416	0.22613	0.61597	0.47384	0.0627	0.08015	0.7391	7.34E-11	7.34E-11	blue
CAPN5	0.06454	0.06253	0.06224	0.03325	0.05531	0.25272	0.17478	-0.36221	0.36221	0.40166	0.41653	0.4187	0.6659	0.47245	0.00085	0.02223	1.13E-06	1.13E-06	yellow
TGFBR1	-0.02522	-0.13812	-0.07045	0.08245	-0.14927	0.04768	-0.03135	0.24468	-0.24468	0.7433	0.07161	0.35987	0.2837	0.05134	0.53575	0.68394	0.00126	0.00126	turquoise
THUMPD3	-0.04072	-0.11739	-0.07771	-0.08856	-0.09722	0.03747	0.10412	-0.01242	0.01242	0.59698	0.12622	0.31239	0.2494	0.20586	0.6266	0.17534	0.87188	0.87188	turquoise
MS4A7	-0.11693	-0.00365	0.05933	0.03984	-0.22722	0.11091	0.01771	0.51984	-0.51984	0.12775	0.96223	0.44082	0.60488	0.0028	0.14871	0.81815	3.19E-13	3.19E-13	blue
BCDIN3D	-0.07835	-0.13379	-0.20335	-0.12696	0.08634	-0.20726	-0.11658	0.19187	-0.19187	0.30842	0.08106	0.00764	0.09797	0.26152	0.00653	0.12891	0.01194	0.01194	grey
LRG1	0.09135	0.05019	0.02562	0.09986	-0.08293	0.25042	0.18013	-0.28024	0.28024	0.23473	0.51448	0.73943	0.19376	0.28086	0.00095	0.0184	0.00021	0.00021	grey
TNRC18	0.06605	-0.02206	-0.03605	0.00329	0.09348	0.07792	0.08839	-0.20091	0.20091	0.39068	0.77453	0.63973	0.96595	0.22396	0.31103	0.25029	0.00842	0.00842	green
DDX50	-0.00585	-0.09827	0.00998	0.00368	-0.1655	0.01839	0.05228	0.18783	-0.18783	0.93951	0.20102	0.89693	0.96195	0.03052	0.81126	0.49708	0.01389	0.01389	turquoise
ABCA10	-0.07929	-0.16539	-0.01234	0.04857	0.08816	0.03354	-0.12628	0.36867	-0.36867	0.30258	0.03063	0.87274	0.52814	0.25152	0.66321	0.09979	7.00E-07	7.00E-07	grey
CLEC14A	-0.12611	-0.06181	-0.10771	0.03223	-0.03783	-0.05066	-0.15149	0.5144	-0.5144	0.10026	0.42194	0.16086	0.67556	0.62325	0.51051	0.04794	6.17E-13	6.17E-13	blue
HPS6	-0.09064	-0.00329	-0.03288	0.01828	0.01786	-0.10404	0.11948	-0.04902	0.04902	0.2384	0.96597	0.66946	0.81241	0.81663	0.17567	0.11959	0.52432	0.52432	grey
HSD17B1(	-0.03066	0.0319	-0.20691	-0.06255	0.06811	-0.22575	0.01048	-0.13215	0.13215	0.69052	0.67874	0.00662	0.41637	0.37607	0.00299	0.8918	0.0849	0.0849	grey
KRT86	0.05588	-0.01078	-0.01814	-0.04903	-0.11406	0.00383	0.04582	-0.24177	0.24177	0.46786	0.88869	0.8138	0.52424	0.13743	0.9603	0.55175	0.00144	0.00144	grey
WIPI2	-0.03363	-0.11191	-0.08109	-0.01715	0.00565	-0.10908	0.03038	-0.02046	0.02046	0.6624	0.14504	0.29173	0.82385	0.94157	0.15555	0.69326	0.79055	0.79055	grey
SNX32	0.11729	-0.06918	0.05392	-0.07291	0.15099	0.0971	0.01564	-0.1574	0.1574	0.12655	0.3686	0.48367	0.34331	0.04869	0.20644	0.83909	0.03979	0.03979	grey
ADGRL4	-0.16945	-0.09841	-0.04536	0.08251	-0.13846	0.00947	-0.08935	0.55045	-0.55045	0.02672	0.20037	0.55578	0.2833	0.07091	0.90215	0.24518	6.23E-15	6.23E-15	blue
CLDN18	-0.07476	0.15971	0.1792	0.04232	0.03491	0.29964	0.04017	-0.25089	0.25089	0.33114	0.03693	0.01902	0.58261	0.65032	6.85E-05	0.60191	0.00093	0.00093	yellow
HTRA4	-0.05495	0.00723	0.123	0.13452	-0.22673	0.14641	0.03831	0.30013	-0.30013	0.47537	0.92526	0.10898	0.0794	0.00286	0.05603	0.61882	6.66E-05	6.66E-05	blue
PMEPA1	0.02988	-0.06475	0.02473	0.0929	-0.19045	0.16827	0.15754	-0.21664	0.21664	0.69802	0.40012	0.7482	0.22684	0.01259	0.02781	0.0396	0.00443	0.00443	yellow
TTC39C	-0.02159	-0.14766	-0.13191	0.03891	-0.02646	0.01996	-0.11825	0.1447	-0.1447	0.77928	0.05394	0.08548	0.61341	0.73123	0.79555	0.12346	0.05899	0.05899	grey
ABCA5	-0.01881	-0.20018	-0.15741	-0.11921	0.24206	-0.25113	-0.24109	0.24711	-0.24711	0.80711	0.00866	0.03976	0.12042	0.00142	0.00092	0.00149	0.00112	0.00112	brown
ITSN2	-0.07406	-0.08401	0.02766	0.04644	-0.11059	0.17489	0.03218	0.1747	-0.1747	0.33572	0.27465	0.71956	0.5464	0.14987	0.02215	0.67604	0.02229	0.02229	turquoise
CLOCK	-0.02724	-0.14798	-0.03589	0.00626	0.00915	-0.03476	-0.00578	0.29232	-0.29232	0.72356	0.05342	0.64117	0.93521	0.90549	0.65176	0.94017	0.0001	0.0001	turquoise
PARP4	0.0188	-0.02949	0.08809	0.01551	-0.13575	0.22942	0.16416	-0.16313	0.16313	0.80717	0.70182	0.2519	0.84048	0.07668	0.00254	0.03191	0.03302	0.03302	turquoise
NLN	-0.06011	0.09089	-0.02316	0.04184	-0.18234	0.1186	0.14442	-0.1678	0.1678	0.43484	0.23709	0.76365	0.58688	0.01699	0.12236	0.05949	0.02825	0.02825	turquoise
MMP2	-0.09072	-0.0067	0.03227	0.0769	-0.18307	0.17793	0.14966	0.23817	-0.23817	0.23796	0.93069	0.67519	0.31748	0.01655	0.0199	0.05073	0.00171	0.00171	black
ARRDC4	-0.0112	-0.25566	-0.08045	-0.02329	0.15761	-0.13056	-0.20735	0.26328	-0.26328	0.88443	0.00074	0.29559	0.76241	0.03951	0.08875	0.0065	0.0005	0.0005	turquoise
CISD3	0.03517	0.06302	-0.09609	-0.06115	0.22388	-0.0646	0.03895	-0.36805	0.36805	0.64795	0.41287	0.21121	0.42687	0.00324	0.40124	0.61304	7.32E-07	7.32E-07	grey
DDX6	-0.06438	-0.1646	-0.0745	-0.03504	-0.1753	0.00984	-0.01471	0.22445	-0.22445	0.40286	0.03145	0.33284	0.64912	0.02183	0.8984	0.84852	0.00316	0.00316	turquoise
RNGTT	-0.09283	-0.06898	0.01276	-0.00339	-0.16418	-0.02957	-0.00981	0.39087	-0.39087	0.22721	0.36999	0.86848	0.96492	0.03189	0.70106	0.89866	1.25E-07	1.25E-07	turquoise
ATF5	-0.02585	-0.02317	-0.10669	0.04101	0.00515	-0.05053	0.04973	-0.03238	0.03238	0.73721	0.76353	0.16486	0.59432	0.94666	0.51158	0.51832	0.67419	0.67419	grey
SLCO4A1	0.07881	0.04671	0.10347	0.0405	0.05608	0.17238	0.23589	-0.49773	0.49773	0.30553	0.54406	0.17808	0.5989	0.46626	0.02417	0.0019	4.33E-12	4.33E-12	yellow
BABAM1	0.07473	-0.00815	-0.05059	-0.01035	0.15596	-0.09889	0.04152	-0.27855	0.27855	0.33137	0.91571	0.51114	0.89314	0.04166	0.19818	0.58979	0.00023	0.00023	green
CES4A	-0.07926	-0.11314	-0.16335	-0.09199	0.18721	-0.23302	-0.15978	0.18573	-0.18573	0.30277	0.14065	0.03278	0.23147	0.01421	0.00216	0.03684	0.01501	0.01501	brown
ZNF681	-0.08275	-0.05367	-0.04463	0.06849	-0.0634	-0.01558	-0.0339	0.22755	-0.22755	0.28192	0.48566	0.56216	0.37342	0.41004	0.83976	0.65981	0.00276	0.00276	grey
P2RY13	-0.1551	-0.02886	-0.00988	-0.02582	-0.15156	0.08554	0.05279	0.49417	-0.49417	0.04281	0.70783	0.89792	0.73747	0.04784	0.26596	0.49285	6.47E-12	6.47E-12	blue
CPOX	-0.04905	-0.09451	-0.03114	-0.03723	-0.0267	0.01128	0.15242	-0.16341	0.16341	0.52404	0.21887	0.68596	0.62878	0.72887	0.88355	0.04657	0.03272	0.03272	turquoise
PLD3	-0.00477	-0.18484	-0.10574	-0.00818	0.01708	-0.2534	-0.14224	0.28839	-0.28839	0.9506	0.01551	0.16868	0.91548	0.82455	0.00083	0.06348	0.00013	0.00013	brown
AARS2	0.04131	0.00333	-0.09103	0.012	0.08833	-0.02525	0.01819	0.04002	-0.04002	0.59161	0.96556	0.23639	0.87619	0.25061	0.74304	0.8133	0.60332	0.60332	turquoise
IFT81	-0.02069	-0.20148	-0.13996	-0.00597	0.1005	-0.19775	-0.0629	0.22751	-0.22751	0.78822	0.00823	0.06788	0.9382	0.1909	0.00953	0.41375	0.00277	0.00277	turquoise
EME1	-0.10332	0.04306	-0.00579	-0.03048	-0.001	-0.00096	0.18492	-0.34478	0.34478	0.17868	0.57602	0.94005	0.69226	0.9896	0.99007	0.01546	3.88E-06	3.88E-06	pink
PGBD5	-0.0722	-0.15873	-0.30244	-0.09553	0.04483	-0.30923	-0.23586	0.44215	-0.44215	0.34804	0.03812	5.81E-05	0.21389	0.56044	3.86E-05	0.0019	1.41E-09	1.41E-09	brown
NLGN4X	-0.02458	-0.13542	-0.15495	-0.07801	-0.09562	-0.12778	-0.18502	0.60985	-0.60985	0.7496	0.0774	0.04301	0.31047	0.2135	0.0958	0.01541	8.51E-19	8.51E-19	brown
AKT2	0.03791	-0.18546	0.02661	0.12877	-0.16061	0.04722	0.04986	-0.01956	0.01956	0.62254	0.01516	0.72974	0.09326	0.03586	0.53971	0.51724	0.79954	0.79954	grey
EP300	-0.06717	-0.10623	-0.00952	0.05881	-0.09222	0.08103	0.00204	0.24927	-0.24927	0.38268	0.16671	0.90166	0.44482	0.23026	0.29207	0.97883	0.00101	0.00101	turquoise
PRKAR2B	-0.0996	-0.14038	-0.1194	0.0976	-0.05601	-0.08997	-0.21061	0.57725	-0.57725	0.19491	0.06705	0.11985	0.2041	0.46684	0.24188	0.00569	1.40E-16	1.40E-16	brown
MN1	0.01759	-0.01364	-0.00493	0.10933	-0.13835	0.13863	0.06492	0.32119	-0.32119	0.81938	0.85945	0.949	0.1546	0.07114	0.07056	0.39888	1.84E-05	1.84E-05	black
DDIAS	-0.06798	0.10194	0.06122	0.01161	-0.14685	0.12358	0.33382	-0.18881	0.18881	0.37697	0.18461	0.42637	0.88021	0.05528	0.10732	8.12E-06	0.01339	0.01339	pink
HCCS	-0.05377	-0.10271	-0.03747	-0.01682	-0.05269	0.02487	0.06916	-0.14757	0.14757	0.48485	0.1813	0.6266	0.82716	0.49373	0.74676	0.36872	0.05409	0.05409	turquoise
SERPING1	-0.0379	-0.03866	-0.00908	0.13636	-0.18742	0.18375	0.04926	0.18953	-0.18953	0.62258	0.61563	0.90618	0.07534	0.0141	0.01614	0.52233	0.01304	0.01304	blue
MTMR11	0.03711	-0.02466	0.05526	-0.05847	0.15648	0.1548	0.01259	-0.36147	0.36147	0.6299	0.74889	0.47287	0.44744	0.04097	0.04322	0.87017	1.19E-06	1.19E-06	yellow
ANKRD18.	0.09079	-0.0694	-0.02559	-0.0594	0.13597	0.0706	0.1056	-0.33908	0.33908	0.23763	0.36705	0.73969	0.44026	0.0762	0.35882	0.16926	5.72E-06	5.72E-06	grey
CMTM8	0.01687	-0.05475	-0.27281	-0.21977	0.12462	-0.20047	-0.1174	-0.02148	0.02148	0.82668	0.47697	0.00031	0.00387	0.10439	0.00857	0.12621	0.7803	0.7803	grey

MOB3B	0.04036	-0.03329	-0.00181	0.02612	-0.12027	0.2301	0.07283	0.09818	-0.09818	0.60023	0.66559	0.98128	0.73447	0.11714	0.00247	0.34385	0.2014	0.2014	turquoise
NEK3	0.0445	0.01051	0.07726	-0.00896	0.09975	0.13138	0.04869	-0.11354	0.11354	0.56329	0.89148	0.31516	0.90741	0.19427	0.08673	0.5271	0.13923	0.13923	grey
LRRC47	-0.0385	-0.07571	-0.09084	0.02903	-0.00046	-0.01466	0.01178	0.09463	-0.09463	0.61708	0.32504	0.23733	0.70626	0.99526	0.8491	0.87848	0.21826	0.21826	turquoise
RNF7	0.03583	-0.05225	-0.03242	-0.01698	-0.0138	0.13115	0.12208	-0.37896	0.37896	0.64174	0.49736	0.67382	0.82551	0.85783	0.08731	0.11168	3.20E-07	3.20E-07	turquoise
GCDH	-0.009	0.08143	-0.22187	-0.19756	0.08622	-0.16153	0.00447	0.08075	-0.08075	0.907	0.28972	0.00354	0.0096	0.26216	0.03481	0.95372	0.29377	0.29377	green
SEMA3B	0.1125	0.05292	0.06744	-0.00428	0.08273	0.13431	0.1469	-0.40254	0.40254	0.14294	0.49179	0.38079	0.95571	0.28202	0.07989	0.05521	4.82E-08	4.82E-08	yellow
AVPI1	0.11742	0.04308	0.00788	0.01549	0.0123	0.05333	0.24533	-0.35101	0.35101	0.12615	0.57585	0.91857	0.84068	0.87312	0.48847	0.00122	2.51E-06	2.51E-06	yellow
POU2F2	-0.1389	0.03001	0.06862	-0.02476	-0.10844	0.07256	-0.05485	0.39313	-0.39313	0.07002	0.69677	0.37252	0.7479	0.15802	0.34563	0.47612	1.04E-07	1.04E-07	blue
CDK4	0.05835	-0.10466	-0.08095	0.03509	-0.01951	-0.07847	0.07322	0.0492	-0.0492	0.44841	0.17311	0.29256	0.64862	0.8001	0.30765	0.34126	0.52281	0.52281	turquoise
RBM45	-0.02817	-0.03167	-0.05334	-0.00483	-0.02451	-0.00396	0.06841	0.01724	-0.01724	0.71456	0.68092	0.48839	0.94998	0.75036	0.95905	0.37396	0.82294	0.82294	turquoise
ARPC1B	-0.01982	-0.03782	0.08643	0.06089	-0.0156	0.16057	0.23677	-0.29327	0.29327	0.79691	0.62333	0.26097	0.42883	0.83951	0.03591	0.00182	9.91E-05	9.91E-05	yellow
ZBTB37	-0.03191	-0.10882	-0.06474	-0.0099	-0.02273	0.05532	-0.01179	0.17706	-0.17706	0.67866	0.15656	0.40018	0.89776	0.76791	0.47238	0.8784	0.02052	0.02052	red
ZCCHC3	-0.02358	-0.19649	-0.19492	-0.08715	0.08456	-0.21862	-0.16555	0.20594	-0.20594	0.75947	0.01	0.01063	0.25705	0.27152	0.00407	0.03047	0.00689	0.00689	turquoise
FMO2	-0.02203	0.04446	0.02481	0.10345	-0.16077	0.13041	-0.03745	0.47112	-0.47112	0.77483	0.56364	0.74737	0.17816	0.03567	0.08911	0.62677	7.86E-11	7.86E-11	blue
GARNL3	-0.02767	-0.09953	-0.10655	0.00919	0.05638	-0.09891	-0.15702	0.43133	-0.43133	0.71937	0.19524	0.16542	0.90504	0.46386	0.19806	0.04027	3.87E-09	3.87E-09	brown
NDC80	-0.01196	0.00553	0.0813	-0.0059	-0.03257	0.11996	0.25288	-0.2764	0.2764	0.87665	0.94277	0.29047	0.93899	0.6724	0.1181	0.00085	0.00025	0.00025	pink
OMP	0.10298	0.05321	0.05063	-0.04875	0.146	0.16015	0.07743	-0.33202	0.33202	0.18015	0.48948	0.51078	0.52658	0.05672	0.03641	0.31409	9.15E-06	9.15E-06	grey
GLI4	0.01924	0.02841	-0.10776	-0.06948	0.19218	-0.09392	0.08243	-0.31235	0.31235	0.80277	0.71224	0.16064	0.36652	0.0118	0.22175	0.28381	3.20E-05	3.20E-05	green
SEC61A1	0.07871	0.00157	-0.09947	2.03E-05	-0.06411	-0.04472	0.09818	-0.05267	0.05267	0.30616	0.98377	0.1955	0.99979	0.40482	0.56138	0.20141	0.49384	0.49384	turquoise
SELENON	-0.1085	-0.1541	-0.03214	0.05974	-0.23652	0.0284	0.0229	0.38984	-0.38984	0.15777	0.04418	0.67647	0.43764	0.00184	0.71237	0.76625	1.36E-07	1.36E-07	blue
NECAP2	-0.06246	-0.03573	0.14729	0.08867	-0.20805	0.28662	0.16021	-0.03766	0.03766	0.41705	0.64265	0.05455	0.2488	0.00632	0.00014	0.03633	0.62484	0.62484	turquoise
STEAP1	0.02334	-0.06528	0.27185	0.13889	-0.09312	0.19564	0.19614	-0.2297	0.2297	0.76187	0.39631	0.00032	0.07004	0.22573	0.01034	0.01014	0.00251	0.00251	turquoise
ZBTB22	-0.07112	-0.02234	-0.08876	-0.03171	0.03607	-0.06623	-0.06095	0.21738	-0.21738	0.35532	0.77179	0.24833	0.68054	0.63956	0.38942	0.42842	0.00429	0.00429	turquoise
BORCS6	0.01203	-0.04986	-0.19308	-0.11239	0.14262	-0.23095	-0.06227	0.00665	-0.00665	0.87586	0.51726	0.0114	0.14331	0.06276	0.00237	0.41846	0.93124	0.93124	green
FGF19	0.0187	-0.02441	-0.06974	0.00939	-0.07825	0.02066	0.15768	-0.01841	0.01841	0.80816	0.75135	0.36473	0.90298	0.30903	0.78858	0.03942	0.81113	0.81113	grey
LILRB5	-0.07979	0.02607	0.0677	-0.01059	-0.13084	0.05941	0.00675	0.51171	-0.51171	0.29955	0.73498	0.37893	0.89069	0.08806	0.44016	0.93019	8.51E-13	8.51E-13	blue
JMJD6	0.03642	-0.2592	-0.05085	-0.03883	0.06475	-0.03776	0.03961	-0.01055	0.01055	0.63628	0.00062	0.50895	0.61408	0.40014	0.6239	0.60704	0.89106	0.89106	grey
SERTAD1	0.02742	-0.23672	0.03603	0.12113	-0.04033	0.16049	0.11215	-0.23077	0.23077	0.72186	0.00183	0.6399	0.11452	0.6005	0.036	0.14417	0.00239	0.00239	grey
GIMAP8	-0.16557	-0.08326	-0.07398	0.02689	-0.14665	-0.00832	-0.0665	0.60855	-0.60855	0.03045	0.27895	0.3362	0.72704	0.05563	0.91404	0.38748	1.06E-18	1.06E-18	blue
LAMP2	-0.05669	-0.20131	-0.09203	0.03739	-0.13433	-0.01361	-0.01635	0.27392	-0.27392	0.46146	0.00828	0.23125	0.62729	0.07983	0.85974	0.83187	0.00029	0.00029	turquoise
UBA1	-0.15573	-0.09475	-0.02708	-0.02424	-0.06348	-0.00456	0.07589	-0.11168	0.11168	0.04196	0.21769	0.72513	0.75303	0.40947	0.9528	0.3239	0.14589	0.14589	turquoise
PGF	-0.07531	-0.17378	-0.01603	0.10245	-0.06638	0.00587	0.00148	0.25576	-0.25576	0.32761	0.02302	0.83519	0.1824	0.38836	0.93928	0.98463	0.00073	0.00073	black
RARA	-0.02744	-0.21262	-0.07667	0.05978	0.01548	0.08618	-0.02538	0.04165	-0.04165	0.72165	0.00524	0.31889	0.43738	0.84076	0.2624	0.74172	0.58859	0.58859	grey
ENTPD7	-0.02215	-0.17042	0.06398	0.07889	-0.12336	0.13484	0.08778	0.22463	-0.22463	0.77371	0.02584	0.40577	0.30506	0.10795	0.07868	0.25362	0.00314	0.00314	turquoise
HCN2	0.01274	-0.12724	-0.14319	-0.01549	0.11719	-0.30216	-0.12865	0.22792	-0.22792	0.86867	0.09723	0.06171	0.84067	0.1269	5.90E-05	0.09354	0.00272	0.00272	brown
METTL7A	-0.04606	-0.18333	-0.1281	0.03284	0.03729	-0.04655	-0.21204	0.47293	-0.47293	0.54966	0.01639	0.09498	0.66981	0.62821	0.54549	0.00537	6.51E-11	6.51E-11	turquoise
AQP3	0.18136	-0.0247	-0.17758	0.05684	0.18557	-0.11187	-0.08972	0.00756	-0.00756	0.0176	0.74843	0.02015	0.46023	0.0151	0.14518	0.24324	0.92179	0.92179	grey
CENPH	-0.05029	0.13679	-0.01376	-0.08323	-0.04266	-0.00933	0.19932	-0.33874	0.33874	0.5136	0.07441	0.85822	0.27913	0.57956	0.90364	0.00896	5.85E-06	5.85E-06	pink
FBN1	-0.06112	-0.05549	0.06024	0.08105	-0.23427	0.17458	0.05565	0.38122	-0.38122	0.42712	0.47096	0.43378	0.29196	0.00204	0.02239	0.46972	2.69E-07	2.69E-07	black
CCL7	-0.12633	0.07556	0.07133	0.03466	-0.12136	0.10404	0.0407	0.03797	-0.03797	0.09966	0.32599	0.35389	0.65266	0.11385	0.17567	0.59714	0.62197	0.62197	grey
NOX1	0.00966	0.02971	0.08296	0.07098	0.11656	0.04311	0.12902	-0.16858	0.16858	0.90022	0.6997	0.28069	0.35624	0.12895	0.57555	0.09261	0.02752	0.02752	grey
METTL18	-0.0019	-0.03836	-0.03656	0.02566	-0.02585	0.06786	0.10427	-0.11155	0.11155	0.98033	0.61838	0.63497	0.73905	0.73721	0.37781	0.17474	0.14635	0.14635	greenyellow
RNF207	0.01945	-0.04104	0.04884	0.02737	0.11561	0.11826	0.158	-0.34194	0.34194	0.80062	0.59403	0.52581	0.72236	0.13212	0.12342	0.03902	4.71E-06	4.71E-06	green
RP2	-0.14461	-0.18022	0.01717	0.07076	-0.07552	0.10112	-0.03085	0.16647	-0.16647	0.05915	0.01833	0.82366	0.35774	0.32623	0.18817	0.68875	0.02954	0.02954	turquoise
PIP4K2C	0.14524	-0.17653	0.00521	0.01434	0.05052	-0.05218	-0.02819	-0.19071	0.19071	0.05805	0.02091	0.94612	0.8523	0.51171	0.49786	0.7144	0.01247	0.01247	turquoise
ZNF787	0.16776	0.10216	-0.05266	-0.04054	0.04464	0.08192	0.12118	-0.4495	0.4495	0.02829	0.18365	0.49395	0.59859	0.56204	0.28679	0.11438	6.95E-10	6.95E-10	green
VEGFA	-0.01711	-0.16045	-0.03828	0.06743	0.13315	-0.01465	-0.01539	-0.22152	0.22152	0.82426	0.03605	0.61915	0.38084	0.08255	0.8492	0.84168	0.00359	0.00359	grey
MAP4K4	-0.01237	-0.08282	0.05165	0.06449	-0.17951	0.11446	0.17424	-0.05872	0.05872	0.87246	0.28149	0.50231	0.40201	0.01881	0.13604	0.02265	0.44556	0.44556	turquoise
TMEM151	0.02025	-0.07127	-0.24783	-0.07486	0.12982	-0.31675	-0.1929	0.19077	-0.19077	0.7926	0.35426	0.00108	0.33052	0.09059	2.43E-05	0.01148	0.01244	0.01244	brown
PKHD1	0.01678	-0.0702	-0.05666	0.13626	-0.06733	0.14845	0.02757	0.14558	-0.14558	0.82757	0.36157	0.46168	0.07557	0.38161	0.05265	0.72034	0.05745	0.05745	magenta
PRRG1	-0.03574	-0.11824	0.0321	0.09919	-0.17124	0.14113	0.11062	-0.06313	0.06313	0.64258	0.12351	0.6768	0.19678	0.02513	0.06559	0.14975	0.41205	0.41205	turquoise
CYP2U1	-0.08943	-0.12711	-0.10957	-0.02832	-0.15704	-0.04965	-0.12864	0.58578	-0.58578	0.24474	0.09758	0.15368	0.71309	0.04024	0.51902	0.09359	3.90E-17	3.90E-17	turquoise
TANC2	-0.00572	-0.2688	-0.04984	0.0223	-0.10147	-0.06513	0.02205	0.17814	-0.17814	0.94079	0.00038	0.51741	0.77218	0.18663	0.39736	0.77471	0.01975	0.01975	turquoise
GSKIP	0.03631	-0.01584	0.05853	0.07108	0.03622	0.10748	0.13166	-0.28234	0.28234	0.63725	0.83704	0.44702	0.35555	0.63811	0.16175	0.08606	0.00018	0.00018	turquoise
P2RY8	-0.05535	-0.04956	-0.03744	-0.0103	-0.14262	0.03109	-0.11612	0.52757	-0.52757	0.47215	0.51979	0.62684	0.89365	0.06276	0.68644	0.13042	1.23E-13	1.23E-13	blue
RIOX1	-0.06042	-0.04799	-0.12132	-0.02398	0.00761	-0.04821	-0.04204	0.05288	-0.05288	0.43246	0.53309	0.11394	0.75552	0.92127	0.53123	0.58506	0.49212	0.49212	turquoise
TNNT2	0.02496	0.00899	0.04188	-0.03782	0.2048	0.08666	0.12086	-0.30261	0.30261	0.74587	0.90705	0.58654	0.62336	0.00721	0.25975	0.11535	5.75E-05	5.75E-05	grey
RPA1	-0.07806	-0.13248	-0.08994	-0.08912	-0.09287	-0.0678	-0.01922	0.3108	-0.3108	0.3102	0.08412	0.24203	0.2464	0.227	0.37827	0.80299	3.51E-05	3.51E-05	turquoise
SLCO1B3	-0.01942	0.05332	0.0204	-0.00786	-0.10104	-0.02096	0.09956	-0.082	0.082	0.80099	0.48852	0.79114	0.91871	0.18852	0.78556	0.1951	0.28632	0.28632	grey
LHPP	0.06641	0.05033	0.07007	-0.04296	0.07341	-0.07637	-0.02023	-0.09034	0.09034	0.38816	0.51329	0.36247	0.57688	0.33998	0.32084	0.79279	0.23998	0.23998	grey
FOXA2	0.08781	-0.05108	-0.13832	-0.12281	0.24869	-0.11592	-0.16785	-0.08965	0.08965	0.25341	0.507	0.0712	0.10954	0.00104	0.1311	0.0282	0.24361	0.24361	grey
ICOS	-0.15679	-0.02446	0.07795	0.06066	-0.17531	0.11743	-0.00044	0.40659	-0.40659	0.04056	0.75086	0.31086	0.43061	0.02182	0.12612	0.99548	3.43E-08	3.43E-08	blue
CCDC34	0.06218	-0.00306	-0.01003	-0.08591	0.10857	0.03448	0.16026	-0.2061	0.2061	0.41913	0.96833	0.89645	0.26387	0.15751	0.65439	0.03627	0.00684	0.00684	grey
ACTL6A	-0.03456	-0.02944	-0.01989	-0.00877	-0.09988	0.12047	0.16393	-0.21933	0.21933	0.65363	0.70226	0.79622	0.90933	0.19367	0.11652	0.03215	0.00395	0.00395	turquoise
PDHB	-0.07766	0.04994	-0.06832	-0.08895	-0.10847	0.00219	-0.04327	0.219	-0.219	0.31266	0.51655	0.37459	0.24731	0.15791	0.97728	0.57417	0.00401	0.00401	turquoise
HLA-B	-0.05964	0.12084	0.07252	0.0527	-0.08783	0.14506	0.17255	-0.09703	0.09703	0.43839	0.11541	0.34588	0.49363	0.25335	0.05835	0.02402	0.20675	0.20675	tan
AC010422	0.02423	0.03667	0.05914	-0.01604	-0.21093	0.03639	0.04628	0.09179	-0.09179	0.75308	0.63396	0.4423	0.83501	0.00562	0.63655	0.54778	0.23246	0.23246	grey
OPLAH	-0.0018	0.15182	0.02056	-0.00845	-0.01112	0.14222	0.25228	-0.41577	0.41577	0.98139	0.04745	0.78954	0.91268	0.88527	0.06351	0.00087	1.56E-08	1.56E-08	yellow
INTU	-0.06945	-0.17905	-0.14419	-0.03344	0.0997	-0.0679	-0.0272	0.13643	-0.13643	0.36673	0.01912	0.05989	0.66418	0.19448	0.37759	0.72394	0.0752	0.0752	turquoise
SNRPA	0.08211	0.03872	-0.05103	-0.09926	0.07252	-0.04294	0.1237	-0.50667	0.50667	0.28565	0.61514	0.50744	0.19645	0.3459	0.57706	0.10697	1.54E-12	1.54E-12	green
HPS4	-0.06454	-0.02594	-0.07865	-0.06372	0.06992	-0.0661	-0.01203	-0.17315	0.17315	0.40167	0.73627	0.30651	0.40769	0.36352	0.39036	0.87595	0.02353	0.02353	red
MRI1	0.0069	-0.05073	-0.05821	-0.03288	0.14661	-0.01256	0.02833	-0.1047	0.1047	0.92865	0.50992	0.44946	0.6694	0.0557	0.87044	0.71304	0.17293	0.17293	red
CES3	-0.00785	0.02074	0.03546	0.04906	0.1451	0.12053	0.01655	-0.26601	0.26601	0.91881	0.78771	0.6452	0.52399	0.05828	0.11634	0.82992	0.00044	0.00044	grey
GPRC5D	0.00686	-0.00238	-0.11055	-0.12093	0.00191	-0.05725	0.18223	-0.33934	0.33934	0.92908	0.97538	0.15003	0.11512	0.98022	0.457	0.01706	5.62E-06	5.62E-06	grey
FAM124A	-0.1107	-0.06021	-0.02899	0.07113	-0.10043	0.01134	-0.10277	0.51557	-0.51557	0.14946	0.43409	0.70664	0.35522	0.19122	0.88301	0.18102	5.36E-13	5.36E-13	blue
FXYD3	0.02936	0.09918	0.06391	-0.0661	0.06987	0.21595	0.17675	-0.54832	0.54832	0.70303	0.19682	0.40631	0.39036	0.36387	0.00456	0.02074	8.30E-15	8.30E-15	yellow
ERCC3	-0.03243	-0.09892	-0.02904	0.00333	-0.07593	-0.01709	0.07701	0.00583	-0.00583	0.67367	0.19804	0.70616	0.9655	0.32361	0.8244	0.31677	0.93966	0.93966	turquoise
ZNF763	-0.00144	-0.04831	-0.16805	-0.02959	0.0796	-0.14481	-0.1679	0.11366	-0.11366	0.98509	0.53031	0.02802	0.70086	0.30069	0.0588	0.02816	0.13884	0.13884	grey
SLC41A3	-0.00432	-0.04031	-0.22723	-0.11126	-0.04734	0.00223	0.04171	-0.00791	0.00791	0.95531	0.60069	0.0028	0.14742	0.53869	0.97686	0.58806	0.91827	0.91827	turquoise
CSTF2T	-0.05517	-0.117	-0.03329	-0.01188	-0.14046	-0.03692	-0.0508	0.33664	-0.33664	0.47355	0.12751	0.6656	0.87742	0.06689	0.63168	0.50933	6.73E-06	6.73E-06	turquoise
IFIH1	-0.00664	0.0022	0.13769	0.1805	-0.16041	0.17764	0.17044	-0.03728	0.03728	0.93134	0.97721	0.07252	0.01815	0.0361	0.0201	0.02583	0.6283	0.6283	tan
ST3GAL1	-0.0866	-0.05283	0.04527	0.05356	-0.26916	0.12716	0.18592	-0.03753	0.03753	0.26006	0.49252	0.55655	0.48662	0.00037	0.09744	0.0149	0.62603	0.62603	turquoise
ZNF607	-0.03482	-0.16558	-0.22444	-0.08419	0.07563	-0.20679	-0.13107	0.27546	-0.27546	0.65114	0.03044	0.00317	0.27359	0.32556	0.00665	0.08748	0.00027	0.00027	turquoise
LRRC19	0.06188	-0.04144	-0.01229	0.04013	0.13292	0.0922	0.09453	0.02954	-0.02954	0.42138	0.59045	0.87321	0.6023	0.08307	0.2304	0.21878	0.70129	0.70129	grey
PLAA	-0.00463	-0.00724	-0.0964	-0.13446	-0.16569	-0.02549	0.05486	0.18586	-0.18586	0.95206	0.92514	0.20975	0.07954	0.03033	0.74068	0.47606	0.01494	0.01494	turquoise
EHD4	-0.11975	-0.10948	-0.0223	0.04349	-0.26682	0.06395	0.07565	0.00483	-0.00483	0.11873	0.15402	0.77217	0.57221	0.00042	0.40599	0.3254	0.95	0.95	turquoise
SLN	-0.12322	0.00711	-0.05947	0.0705	0.0075	-0.04009	-0.01311	0.06267	-0.06267	0.10837	0.92644	0.43975	0.35954	0.92242	0.60265	0.86488	0.41549	0.41549	grey
TNFAIP8L:	-0.0921	-0.12039	0.00318	0.0732	-0.21359	0.12871	-0.01026	0.41165	-0.41165	0.23088	0.11677	0.96711	0.34138	0.00503	0.0934	0.894	2.22E-08	2.22E-08	blue
SEC11C	0.00318	0.00154	-0.25224	-0.12389	0.20737	-0.36008	-0.26267	0.37856	-0.37856	0.96707	0.98401	0.00087	0.10644	0.0065	1.32E-06	0.00052	3.30E-07	3.30E-07	brown
CAPN2	0.04028	-0.05816	0.05993	0.00735	-0.05985	0.16469	0.19602	-0.29425	0.29425	0.60091	0.44991	0.4362	0.92404	0.4368	0.03136	0.01019	9.36E-05	9.36E-05	turquoise
KRCC1	-0.11652	-0.15703	-0.01485	0.00828	-0.02033	0.08637	-0.04765	0.02883	-0.02883	0.1291	0.04026	0.84716	0.91444	0.79187	0.26135	0.53601	0.70821	0.70821	turquoise
RRP7A	-0.04763	0.05743	-0.01857	-0.01561	0.02412	-0.04577	0.07291	-0.21965	0.21965	0.53618	0.45564	0.80951	0.83938	0.7542	0.55221	0.34328	0.00389	0.00389	grey
RSPH3	0.04635	-0.15773	-0.02507	0.04036	-0.03179	-0.01728	0.0966	-0.05057	0.05057	0.54722	0.03937	0.74486	0.60016	0.6798	0.82254	0.20881	0.51127	0.51127	turquoise
EIF6	0.11316	0.05438	0.02573	0.00895	0.03941	0.02663	0.17236	-0.53739	0.53739	0.14059	0.47994	0.73831	0.90752	0.60884	0.72958	0.02418	3.51E-14	3.51E-14	greenyellow
TCEA3	0.10292	0.06778	0.15272	0.18652	-0.05413	0.37076	0.15677	-0.22681	0.22681	0.18038	0.37837	0.04614	0.01458	0.48197	5.98E-07	0.04059	0.00285	0.00285	grey
INSM1	0.04019	-0.08668	-0.24703	-0.15939	0.16812	-0.29852	-0.23024	0.35215	-0.35215	0.60173	0.25964	0.00112	0.03731	0.02795	7.31E-05	0.00245	2.32E-06	2.32E-06	brown
SPINK4	0.03235	0.08815	-0.0015	-0.00935	0.08073	0.07208	0.06204	-0.14579	0.14579	0.67445	0.25159	0.98447	0.90336	0.29388	0.34881	0.42019	0.05709	0.05709	grey
ATXN7L3E	-0.04769	-0.14604	-0.09453	-0.01332	0.06865	-0.03781	-0.12431	0.09276	-0.09276	0.53566	0.05666	0.21878	0.86275	0.3723	0.62343	0.10524	0.22755	0.22755	turquoise
FBXL16	-0.00486	-0.10473	-0.26214	-0.13003	0.1059	-0.30432	-0.17304	0.40823	-0.40823	0.94971	0.17279	0.00053	0.09007	0.16805	5.19E-05	0.02362	2.98E-08	2.98E-08	brown
GATM	0.01325	0.08479	0.06074	0.14039	-0.03059	0.20617	0.00506	0.04959	-0.04959	0.86347	0.27021	0.42999	0.06704	0.69128	0.00682	0.94759	0.51949	0.51949	magenta
LONP2	-0.00038	-0.14452	-0.0078	-0.02217	-0.0679	0.14487	0.0592	-0.05585	0.05585	0.99606	0.05931	0.91936	0.77347	0.37757	0.05869	0.4418	0.46814	0.46814	turquoise
PYCR3	0.02784	0.10683	-0.14066	-0.11829	0.06528	-0.15761	0.14187	-0.4398	0.4398	0.71779	0.1643	0.06651	0.12333	0.39626	0.03952	0.06417	1.76E-09	1.76E-09	green
SEC24B	-0.07981	-0.15784	0.00851	0.02229	-0.11361	0.07764	0.00467	0.14066	-0.14066	0.29942	0.03923	0.91199	0.77228	0.13898	0.31279	0.95166	0.0665	0.0665	turquoise
AMD1	-0.08995	-0.0343	-0.01662	0.0376	-0.11957	0.06891	0.04715	0.22985	-0.22985	0.242	0.65608	0.82921	0.62541	0.1193	0.37048	0.54031	0.00249	0.00249	turquoise
VPS26B	-0.05903	-0.16255	-0.20788	-0.1459	-0.03295	-0.17677	0.01389	0.19728	-0.19728	0.44316	0.03365	0.00637	0.0569	0.66874	0.02073	0.8569	0.0097	0.0097	turquoise
LAP3	-0.12038	0.05557	0.05688	0.06951	-0.14717	0.11345	0.1499	0.16359	-0.16359	0.11681	0.47037	0.45991	0.36633	0.05475	0.13954	0.05036	0.03252	0.03252	blue
NFXL1	0.00207	-0.11739	-0.05733	0.0069	-0.01283	-0.01056	0.07059	0.03284	-0.03284	0.97855	0.12625	0.45641	0.92865	0.86774	0.891	0.35889	0.66978	0.66978	turquoise
SFRP2	-0.02433	0.01866	0.0874	0.12258	-0.19787	0.194	0.06662	0.24867	-0.24867	0.75212	0.80858	0.25565	0.11023	0.00948	0.01101	0.38663	0.00104	0.00104	black
CELF1	0.04167	-0.17527	-0.00656	-0.00634	-0.07522	0.04084	0.02805	-0.06203	0.06203	0.58837	0.02185	0.93211	0.93445	0.32816	0.59582	0.71571	0.42023	0.42023	red
TEX261	-0.01386	-0.1263	-0.13649	-0.01356	-0.10108	-0.08651	-0.06326	0.21716	-0.21716	0.85719	0.09974	0.07506	0.86023	0.18837	0.26057	0.41108	0.00433	0.00433	turquoise
DPH7	0.05092	-0.10696	-0.13462	-0.08487	0.15561	-0.22439	-0.04782	-0.11424	0.11424	0.50836	0.16378	0.07919	0.26971	0.04212	0.00317	0.53453	0.13681	0.13681	red
PPM1H	0.07426	-0.13121	-0.21865	-0.09579	0.17697	-0.13209	-0.1756	0.02578	-0.02578	0.33439	0.08715	0.00406	0.21266	0.02058	0.08504	0.0216	0.73783	0.73783	grey
CCDC121	-0.11923	-0.17308	-0.12099	0.00432	-0.06106	-0.01926	-0.07373	0.36123	-0.36123	0.12036	0.02359	0.11496	0.95523	0.42757	0.80255	0.3379	1.21E-06	1.21E-06	turquoise
KDM5D	0.92239	-0.06516	-0.04703	0.01443	0.03398	-0.02776	0.04012	0.02783	-0.02783	1.01E-71	0.39712	0.54128	0.85141	0.65905	0.71856	0.60239	0.71787	0.71787	grey
TSTA3	0.03938	0.14421	-0.0173	-0.07368	0.09517	0.08144	0.20507	-0.50128	0.50128	0.60905	0.05986	0.8223	0.33823	0.21562	0.28967	0.00713	2.88E-12	2.88E-12	green
MED16	0.02959	0.01535	-0.13616	-0.11049	0.24363	-0.14749	-0.03587	-0.10875	0.10875	0.70082	0.84202	0.07577	0.15026	0.00132	0.05422	0.6414	0.1568	0.1568	green
ACTR1B	0.03588	-0.02855	-0.12401	-0.05776	0.07348	-0.06156	0.00162	-0.13164	0.13164	0.64133	0.71085	0.10609	0.45301	0.33954	0.4238	0.9832	0.08611	0.08611	turquoise
TSN	-0.04985	-0.04095	-0.06016	-0.00819	-0.08203	0.03437	0.09087	0.01732	-0.01732	0.51729	0.59484	0.43444	0.91534	0.28617	0.65541	0.23722	0.82206	0.82206	turquoise
SMDT1	0.07579	0.10631	-0.20583	-0.12788	0.1813	-0.21393	-0.19001	0.07133	-0.07133	0.32448	0.16637	0.00692	0.09555	0.01764	0.00496	0.0128	0.35385	0.35385	grey
TCF12	-0.03918	-0.0692	0.03126	0.09159	-0.15752	0.15898	0.12987	0.16461	-0.16461	0.61086	0.36844	0.68479	0.23351	0.03963	0.03781	0.09045	0.03144	0.03144	turquoise
UQCRC2	0.04505	0.09398	0.03437	-0.03082	-0.04006	0.15924	0.16592	-0.14034	0.14034	0.55847	0.22147	0.65535	0.68902	0.60288	0.03749	0.03009	0.06714	0.06714	turquoise
DEDD2	0.1152	-0.14759	-0.06105	-0.06686	0.17138	-0.03244	0.0071	-0.37851	0.37851	0.1335	0.05406	0.42768	0.3849	0.02501	0.67361	0.92662	3.32E-07	3.32E-07	grey
FAR2	0.0087	0.08951	0.03475	0.05762	-0.01972	0.11079	0.06216	-0.13217	0.13217	0.91006	0.24433	0.65183	0.45411	0.79794	0.14915	0.41925	0.08484	0.08484	grey
ITGB1BP2	-0.08705	-0.06908	0.05487	0.06195	-0.05521	0.11457	0.07545	0.08121	-0.08121	0.25757	0.36928	0.47595	0.42087	0.47322	0.13568	0.32672	0.29099	0.29099	salmon
FEN1	-0.12685	0.14347	-0.05359	-0.04341	-0.07952	-0.07442	0.24225	-0.28307	0.28307	0.09827	0.0612	0.48635	0.57291	0.3012	0.33338	0.00141	0.00018	0.00018	pink
ZDHHC8	-0.05554	-0.08742	-0.12119	-0.01099	0.09976	-0.13244	-0.02416	-0.01287	0.01287	0.4706	0.25554	0.11434	0.88651	0.1942	0.0842	0.75379	0.86734	0.86734	green
RASGRF2	-0.06872	-0.05152	0.01408	0.06813	-0.2261	0.06222	0.00077	0.44894	-0.44894	0.3718	0.50338	0.85494	0.37593	0.00294	0.41882	0.99202	7.34E-10	7.34E-10	blue
QRICH2	0.0018	-0.1741	-0.09594	0.07127	0.18867	-0.15588	-0.02261	0.17009	-0.17009	0.98136	0.02276	0.21194	0.35426	0.01346	0.04176	0.76915	0.02614	0.02614	grey
SRRM3	0.05161	-0.22446	-0.27075	-0.0564	0.08831	-0.28982	-0.18634	0.3478	-0.3478	0.50262	0.00316	0.00034	0.46371	0.25072	0.00012	0.01468	3.15E-06	3.15E-06	brown

ADAM19	-0.10115	-0.05491	0.05678	0.05567	-0.19011	0.10513	0.1017	0.12734	-0.12734	0.18805	0.47562	0.46077	0.46958	0.01275	0.17115	0.18562	0.09698	0.09698	black
SGSH	-0.06449	-0.17313	-0.02087	0.00927	0.04857	0.00586	-0.05556	0.25935	-0.25935	0.40202	0.02354	0.78644	0.90423	0.5281	0.93934	0.47041	0.00061	0.00061	grey
FOXN3	-0.07075	-0.08346	0.06727	0.12107	-0.18654	0.17148	-0.02754	0.45067	-0.45067	0.35784	0.27781	0.382	0.11471	0.01457	0.02493	0.72066	6.20E-10	6.20E-10	turquoise
INSR	0.09491	-0.12492	-0.0965	-0.00397	-0.07974	-0.02316	-0.06892	0.03715	-0.03715	0.21693	0.10354	0.20925	0.95894	0.29984	0.76369	0.37044	0.6295	0.6295	grey
ZMYM3	-0.00846	-0.19754	-0.10296	-0.0323	-0.02301	-0.08166	0.00112	0.03603	-0.03603	0.91256	0.0096	0.18024	0.67495	0.76516	0.28834	0.98836	0.63993	0.63993	turquoise
OBSL1	0.05878	-0.11762	-0.18072	-0.00442	0.13198	-0.18627	-0.03532	-0.00695	0.00695	0.44509	0.1255	0.01801	0.95429	0.0853	0.01472	0.64649	0.92816	0.92816	grey
GAST	0.02408	-0.07075	#######	-0.03429	0.13223	-0.16749	-0.10596	-0.01228	0.01228	0.75457	0.3578	0.99978	0.65612	0.0847	0.02855	0.16778	0.87332	0.87332	grey
CADM1	-0.10657	-0.14167	-0.22351	-0.01894	-0.01767	-0.23955	-0.16557	0.51318	-0.51318	0.16536	0.06456	0.0033	0.8058	0.81857	0.0016	0.03045	7.14E-13	7.14E-13	brown
ORMDL2	0.04315	-0.10164	-0.07055	-0.05724	0.06539	-0.07918	0.05321	-0.42575	0.42575	0.57525	0.18589	0.35914	0.45712	0.39551	0.30325	0.48946	6.43E-09	6.43E-09	grey
ERI1	-0.044	-0.01849	0.12187	0.08152	-0.13056	0.14545	0.21888	-0.00228	0.00228	0.56773	0.81027	0.11232	0.28918	0.08874	0.05767	0.00403	0.97638	0.97638	turquoise
SERF2	-0.02584	0.00583	-0.0339	-0.07607	0.01338	0.00352	0.07662	-0.29641	0.29641	0.73729	0.93972	0.65983	0.32269	0.86208	0.96356	0.3192	8.27E-05	8.27E-05	grey
NUDT22	-0.02111	0.02108	-0.08101	-0.03721	0.13818	-0.05296	0.08983	-0.30623	0.30623	0.78406	0.78438	0.29223	0.62899	0.07149	0.49149	0.24262	4.63E-05	4.63E-05	green
SMC1A	-0.33065	-0.13553	-0.06857	0.01118	-0.10856	0.01297	0.00079	0.2308	-0.2308	1.00E-05	0.07715	0.37284	0.88464	0.15755	0.86633	0.99182	0.00239	0.00239	turquoise
KCTD5	0.08444	-0.00814	0.06694	-0.00022	-0.07759	0.1395	0.12261	-0.32525	0.32525	0.27221	0.91583	0.38437	0.99774	0.31314	0.0688	0.11012	1.42E-05	1.42E-05	turquoise
ZNF707	0.04661	-0.11428	-0.09132	-0.03594	0.0573	-0.12817	0.06892	-0.22268	0.22268	0.54493	0.13668	0.23487	0.64072	0.4566	0.0948	0.37043	0.00342	0.00342	green
GPR65	-0.11696	-0.03025	0.06843	0.06176	-0.20538	0.12168	0.01885	0.47788	-0.47788	0.12764	0.6945	0.37386	0.42228	0.00704	0.11287	0.80672	3.85E-11	3.85E-11	blue
AP4M1	0.02354	-0.07298	-0.11432	-0.07861	0.09265	-0.05109	0.05573	-0.18766	0.18766	0.75986	0.3428	0.13653	0.30676	0.22808	0.50693	0.46909	0.01398	0.01398	grey
RNASE2	-0.07891	-0.00624	0.12946	0.08286	-0.24616	0.16808	0.13043	0.22873	-0.22873	0.30494	0.93539	0.09148	0.28128	0.00117	0.02798	0.08907	0.00262	0.00262	blue
PPY	0.13063	0.06963	-0.0395	0.01781	-0.07124	0.08646	0.00382	0.14613	-0.14613	0.08857	0.36551	0.608	0.8172	0.35448	0.26084	0.96043	0.05651	0.05651	grey
ACAT1	-0.1321	0.01364	-0.18744	-0.03411	-0.08586	-0.19756	-0.07538	0.53564	-0.53564	0.08501	0.8595	0.01409	0.65783	0.26419	0.00959	0.32712	4.40E-14	4.40E-14	brown
LRRC3	-0.10371	-0.04748	-0.00636	0.02325	-0.08741	0.10676	0.13909	0.05034	-0.05034	0.17706	0.53743	0.93422	0.76275	0.25563	0.16459	0.06963	0.51323	0.51323	grey
GPAM	-0.11969	-0.06745	-0.02354	0.11724	-0.03604	-0.013	-0.04666	0.44003	-0.44003	0.11893	0.38072	0.75988	0.12673	0.63981	0.866	0.54454	1.72E-09	1.72E-09	blue
PEX19	0.02676	-0.12976	-0.06878	-0.10854	-0.03829	-0.0141	-0.00169	0.06524	-0.06524	0.72829	0.09072	0.37141	0.15763	0.61904	0.85474	0.98245	0.39658	0.39658	turquoise
PCSK1N	0.07853	-0.09575	-0.29924	-0.1483	0.15686	-0.26055	-0.12685	0.22041	-0.22041	0.30729	0.21284	7.01E-05	0.0529	0.04048	0.00058	0.09827	0.00377	0.00377	brown
FYTTD1	-0.09223	-0.1343	-0.03156	0.03038	-0.19789	0.06388	0.02187	0.30518	-0.30518	0.23021	0.0799	0.682	0.69325	0.00948	0.40654	0.77651	4.93E-05	4.93E-05	turquoise
PFDN2	0.08786	-0.00481	-0.05205	-0.08742	0.05616	-0.03855	0.12306	-0.44866	0.44866	0.25314	0.95022	0.49898	0.25557	0.46566	0.61663	0.10883	7.55E-10	7.55E-10	greenyellow
KALRN	-0.07993	-0.00625	0.08039	0.04017	0.06807	0.22302	0.03369	-0.04438	0.04438	0.29868	0.93536	0.2959	0.60191	0.37633	0.00337	0.66178	0.56438	0.56438	grey
MTMR12	-0.12216	-0.14469	-0.08052	0.07748	-0.15616	0.04564	-0.01271	0.3433	-0.3433	0.11145	0.05901	0.29516	0.31384	0.04139	0.55336	0.86897	4.29E-06	4.29E-06	turquoise
ZNF44	0.0314	-0.12899	-0.13686	-0.07723	0.11038	-0.0512	-0.15776	0.14231	-0.14231	0.68349	0.09267	0.07427	0.31534	0.15066	0.50599	0.03932	0.06335	0.06335	turquoise
NWD1	-0.20765	-0.1723	-0.11823	0.02785	-0.08449	0.05027	0.0231	0.15337	-0.15337	0.00643	0.02423	0.12353	0.71766	0.27189	0.51377	0.76422	0.0452	0.0452	grey
PRUNE1	0.0358	-0.14254	-0.0989	-0.06567	0.05836	-0.1022	-0.0489	0.1544	-0.1544	0.64205	0.06291	0.19811	0.39346	0.44836	0.18347	0.52536	0.04376	0.04376	turquoise
AHCYL1	-0.07994	-0.18926	-0.0784	0.00712	-0.00742	0.0453	-0.07405	0.28802	-0.28802	0.29866	0.01317	0.30809	0.92631	0.92331	0.5563	0.3358	0.00013	0.00013	turquoise
EIF5AL1	-0.03548	0.10772	-0.00883	-0.03046	-0.03643	-0.02467	0.04801	0.07044	-0.07044	0.64501	0.16079	0.90873	0.69249	0.63615	0.74879	0.53291	0.35991	0.35991	grey
DHRS7B	-0.02864	-0.01679	-0.03804	0.0346	0.03858	-0.07585	-0.0664	0.01411	-0.01411	0.70998	0.82742	0.62137	0.65326	0.61643	0.32413	0.3882	0.85463	0.85463	grey
CMTR2	-0.06618	-0.11367	-0.01504	0.04914	-0.03386	0.05785	0.06867	0.05789	-0.05789	0.3898	0.13878	0.84522	0.52327	0.66021	0.4523	0.37215	0.45202	0.45202	turquoise
ARFGEF2	-0.01751	-0.12142	-0.00174	0.04934	-0.01943	-0.00975	-0.03243	0.11418	-0.11418	0.8202	0.11364	0.98199	0.52157	0.80082	0.89927	0.6737	0.13701	0.13701	turquoise
HOOK1	0.00547	-0.11738	-0.14603	-0.08841	0.12585	-0.08687	-0.12811	0.14826	-0.14826	0.94343	0.12627	0.05668	0.25019	0.10097	0.25857	0.09496	0.05296	0.05296	turquoise
FGF1	-0.11516	-0.09448	-0.05803	-0.07752	-0.0269	-0.10737	-0.14577	0.31863	-0.31863	0.13366	0.21898	0.45087	0.31355	0.72695	0.16219	0.05712	2.16E-05	2.16E-05	grey
VMO1	-0.0529	-0.01343	0.0743	-0.02168	0.00943	0.04673	0.12374	0.00186	-0.00186	0.49196	0.86159	0.33417	0.77835	0.90259	0.54388	0.10687	0.98069	0.98069	grey
SLC25A36	-0.0677	-0.16418	-0.07929	0.0158	-0.0321	0.00015	0.00259	0.10199	-0.10199	0.37898	0.03189	0.30258	0.83748	0.67687	0.99842	0.97321	0.18438	0.18438	turquoise
DNAJB6	0.00115	-0.20402	0.07687	0.10325	-0.09876	0.00149	0.04412	0.09363	-0.09363	0.98805	0.00744	0.31767	0.17898	0.19876	0.98455	0.56667	0.22322	0.22322	turquoise
TMEM63A	0.0489	0.072	0.12934	0.08958	-0.04175	0.32103	0.19956	-0.28881	0.28881	0.52537	0.34935	0.0918	0.24395	0.58768	1.86E-05	0.00887	0.00013	0.00013	yellow
PARP10	0.05767	0.0325	-0.01198	0.02581	0.07163	-0.03026	0.1352	-0.2243	0.2243	0.45368	0.673	0.87646	0.73758	0.35183	0.69436	0.07788	0.00319	0.00319	green
AHCTF1	-0.04928	-0.10074	-0.05628	-0.06076	-0.09902	0.05861	0.03121	0.10036	-0.10036	0.5221	0.18984	0.46468	0.42982	0.19758	0.44642	0.68534	0.19155	0.19155	turquoise
ZNF615	-0.05019	-0.17998	-0.04985	0.06401	-0.15113	0.05624	-0.07955	0.32514	-0.32514	0.51444	0.01849	0.51735	0.40556	0.04848	0.46502	0.30099	1.43E-05	1.43E-05	turquoise
CSNK2B	0.07188	0.05235	-0.06669	-0.06582	0.00377	-0.07512	0.10533	-0.36021	0.36021	0.35014	0.4965	0.38613	0.39239	0.96093	0.32885	0.17036	1.30E-06	1.30E-06	grey
CDK2	0.0096	-0.10599	0.05812	0.08386	-0.17046	0.17603	0.20467	-0.22413	0.22413	0.90078	0.16767	0.45018	0.27551	0.02581	0.02128	0.00725	0.00321	0.00321	turquoise
CTSV	0.12684	-0.02698	0.06796	0.08065	-0.11612	0.069	0.23893	-0.34397	0.34397	0.09831	0.72608	0.37715	0.29437	0.13042	0.36986	0.00165	4.10E-06	4.10E-06	grey
INSIG1	0.00221	0.01811	0.00636	-0.00743	0.01581	0.10808	-0.03598	0.07127	-0.07127	0.97709	0.81413	0.93418	0.92313	0.8374	0.1594	0.64039	0.35425	0.35425	grey
MINDY2	-0.05476	-0.19434	0.05404	0.10446	-0.18566	0.13444	0.09264	0.09271	-0.09271	0.47683	0.01086	0.48268	0.17391	0.01505	0.07959	0.22815	0.2278	0.2278	turquoise
NUDT10	-0.00667	-0.04873	0.02082	0.05733	-0.08705	0.03529	-0.09758	0.58027	-0.58027	0.93099	0.52679	0.78697	0.45639	0.25759	0.64674	0.20419	8.97E-17	8.97E-17	blue
NDRG2	-0.02139	-0.06347	-0.09842	-0.03817	0.04141	0.06183	-0.08766	0.1258	-0.1258	0.78124	0.40954	0.20032	0.62017	0.59076	0.42179	0.25427	0.10112	0.10112	grey
NFX1	0.02738	-0.07538	-0.14189	-0.0711	-0.04975	-0.02398	0.01828	0.1121	-0.1121	0.72223	0.32712	0.06414	0.35545	0.51819	0.7556	0.81239	0.14435	0.14435	turquoise
MYO7B	0.10891	0.0439	0.00396	0.02628	0.24581	0.13295	0.04804	-0.12381	0.12381	0.1562	0.56857	0.95903	0.73295	0.00119	0.083	0.53265	0.10666	0.10666	grey
ZNF200	-0.04657	-0.09466	-0.01102	0.04533	-0.06267	0.0376	0.01328	0.13271	-0.13271	0.54529	0.21813	0.88628	0.55604	0.4155	0.62536	0.86314	0.08357	0.08357	turquoise
ELOC	0.03103	0.01705	-0.02552	-0.04408	-0.05723	-0.09155	0.08559	-0.22222	0.22222	0.68701	0.82483	0.74045	0.56699	0.45718	0.23369	0.26565	0.00349	0.00349	turquoise
CEP97	-0.04828	-0.14062	-0.16684	-0.09286	-0.0954	-0.09237	-0.08614	0.36584	-0.36584	0.53058	0.06659	0.02919	0.22703	0.21455	0.22949	0.26262	8.63E-07	8.63E-07	turquoise
GINS3	-0.10189	0.06143	-0.04613	0.01017	-0.10911	0.06885	0.24439	-0.06336	0.06336	0.18482	0.4248	0.54911	0.89502	0.15545	0.37089	0.00128	0.41036	0.41036	pink
DNA2	-0.04847	0.02765	0.01398	-0.01082	0.01636	-0.03041	0.20652	-0.18771	0.18771	0.52899	0.71965	0.85596	0.8883	0.83183	0.69301	0.00673	0.01395	0.01395	pink
PTRH2	0.03867	-0.09233	-0.1261	-0.05381	#######	-0.08517	0.11453	-0.22575	0.22575	0.61558	0.22971	0.10029	0.48458	0.99918	0.26806	0.13581	0.00299	0.00299	grey
NSUN7	0.04525	-0.12716	-0.09768	0.03816	0.15413	0.03355	-0.03409	-0.2388	0.2388	0.55676	0.09744	0.20371	0.62024	0.04414	0.66311	0.658	0.00166	0.00166	grey
NPTXR	-0.0918	-0.26834	-0.15057	0.09322	-0.12366	-0.1811	-0.14271	0.3918	-0.3918	0.2324	0.00039	0.04932	0.22526	0.10708	0.01776	0.0626	1.16E-07	1.16E-07	brown
RFK	0.05462	-0.14034	-0.08818	0.02326	0.04026	-0.04218	-0.16619	0.12026	-0.12026	0.47802	0.06713	0.25141	0.76267	0.60108	0.58389	0.02983	0.11718	0.11718	turquoise
GULP1	0.04142	-0.05283	0.04293	0.06321	-0.16112	0.2106	0.08747	0.01492	-0.01492	0.59063	0.49258	0.57716	0.41146	0.03527	0.0057	0.25527	0.84643	0.84643	turquoise
ARNTL2	-0.01135	-0.07065	0.11176	0.08253	-0.14097	0.11492	0.21515	-0.34331	0.34331	0.88289	0.35851	0.14558	0.28321	0.06591	0.13447	0.00471	4.29E-06	4.29E-06	turquoise
RPS17	0.10621	0.07358	-0.03447	-0.02925	-0.10123	0.03162	0.1206	-0.15308	0.15308	0.16677	0.33889	0.65446	0.70408	0.18768	0.68142	0.11612	0.04563	0.04563	purple
TMED3	0.05404	-0.06649	-0.13894	-0.05263	0.01101	-0.20294	0.06877	-0.29681	0.29681	0.48272	0.38757	0.06993	0.49417	0.88633	0.00777	0.37147	8.08E-05	8.08E-05	grey
IFT46	-0.0141	-0.09384	-0.1769	-0.05227	-0.05175	-0.1167	0.01475	0.09745	-0.09745	0.85477	0.22214	0.02064	0.49715	0.50141	0.12849	0.84813	0.20478	0.20478	grey
PTPN11	-0.04835	-0.14762	-0.04796	0.02044	-0.0478	-0.03235	-0.08135	0.26292	-0.26292	0.52999	0.05401	0.53337	0.79078	0.53475	0.6745	0.29018	0.00051	0.00051	turquoise
RFX2	-0.10492	-0.20922	-0.16708	-0.03534	0.11705	-0.16824	-0.22558	0.28338	-0.28338	0.17204	0.00603	0.02894	0.64632	0.12736	0.02784	0.00301	0.00017	0.00017	brown
SLC50A1	0.10889	0.02938	0.04449	-0.03369	0.05063	0.08273	0.17841	-0.52323	0.52323	0.15626	0.70291	0.56336	0.66175	0.51075	0.28204	0.01956	2.10E-13	2.10E-13	greenyellow
NUDCD1	-0.01458	-0.06534	0.04039	0.05244	-0.21398	0.04436	0.15117	0.00617	-0.00617	0.84993	0.39587	0.59988	0.49574	0.00495	0.56454	0.04842	0.93619	0.93619	turquoise
CS	0.09198	-0.12037	-0.09715	-0.02958	-0.03311	-0.08185	-0.0254	-0.01162	0.01162	0.23148	0.11683	0.20621	0.70098	0.66729	0.28719	0.74156	0.88005	0.88005	turquoise
ZNF12	-0.03281	-0.20407	-0.01273	0.06097	-0.06522	0.03142	-0.00461	0.09194	-0.09194	0.67012	0.00742	0.86876	0.42823	0.39671	0.68333	0.95224	0.23171	0.23171	turquoise
PJA2	-0.02737	-0.19257	-0.0625	0.00762	-0.10583	-0.04143	-0.11182	0.3978	-0.3978	0.7223	0.01162	0.41677	0.92123	0.16831	0.5906	0.14536	7.13E-08	7.13E-08	turquoise
B3GLCT	-0.11014	-0.09308	-0.06719	0.05227	-0.18483	0.02957	0.02066	0.10898	-0.10898	0.15157	0.22595	0.38259	0.49715	0.01552	0.70101	0.78853	0.15594	0.15594	turquoise
RAD1	-0.02759	-0.12645	-0.07847	-0.04892	-0.11783	-0.11626	0.0308	0.23318	-0.23318	0.72019	0.09935	0.30767	0.52518	0.12482	0.12996	0.68925	0.00215	0.00215	turquoise
DNASE1L3	-0.1043	-0.10867	-0.03419	-0.06737	0.02178	-0.00594	-0.13043	0.45893	-0.45893	0.17459	0.15712	0.65706	0.38128	0.77734	0.93854	0.08907	2.74E-10	2.74E-10	blue
SLC22A23	-0.01311	0.04069	0.07511	-0.06281	0.13732	0.15353	0.02347	-0.11141	0.11141	0.86489	0.59725	0.32889	0.41439	0.0733	0.04498	0.76056	0.14687	0.14687	grey
KLHL18	-0.01015	-0.11625	-0.05039	-0.08042	-0.11862	0.11998	-0.01315	0.22285	-0.22285	0.89521	0.13	0.51277	0.29577	0.12228	0.11803	0.86443	0.00339	0.00339	turquoise
FAM91A1	-0.06377	-0.12487	-0.01332	0.02821	-0.0436	-0.00373	0.03023	0.00514	-0.00514	0.40732	0.10368	0.86268	0.71416	0.57127	0.96142	0.69465	0.94677	0.94677	turquoise
UQCRH	0.05871	-0.00461	0.01657	-0.09494	-0.00062	0.01799	0.13118	-0.35606	0.35606	0.4456	0.95225	0.82972	0.21676	0.99358	0.81533	0.08722	1.76E-06	1.76E-06	grey
USP39	0.06302	-0.06846	-0.00357	-0.00973	-0.1138	0.08036	0.16147	-0.19313	0.19313	0.41289	0.37359	0.96301	0.89948	0.13835	0.29612	0.03487	0.01138	0.01138	turquoise
TDRD10	-0.06272	-0.0774	-0.12974	0.02556	-0.00525	-0.08595	-0.11987	0.50854	-0.50854	0.41507	0.31428	0.09078	0.73999	0.94571	0.26365	0.11838	1.24E-12	1.24E-12	blue
GZMH	-0.05914	0.16034	0.08218	0.10617	-0.16309	0.11039	0.07636	0.28378	-0.28378	0.44224	0.03618	0.28528	0.16696	0.03307	0.15062	0.32084	0.00017	0.00017	blue
AP1S3	0.01194	-0.03317	0.05638	0.05289	-0.05329	0.14008	0.17113	-0.27425	0.27425	0.87687	0.66667	0.46389	0.49202	0.48879	0.06765	0.02523	0.00028	0.00028	turquoise
FMO3	-0.02774	0.05787	0.00615	-0.01607	-0.21387	0.084	0.03961	0.34771	-0.34771	0.71871	0.45216	0.93642	0.83479	0.00497	0.27472	0.60697	3.17E-06	3.17E-06	blue
FAAH	0.01883	-0.05983	-0.04232	-0.02943	0.2525	0.00671	-0.06588	-0.32095	0.32095	0.8069	0.43696	0.58259	0.70239	0.00086	0.93059	0.39197	1.87E-05	1.87E-05	grey
PPIL2	-0.05863	-0.03887	-0.08732	-0.03225	0.10042	-0.11016	-0.10028	0.14908	-0.14908	0.44626	0.61373	0.25609	0.67536	0.19125	0.15146	0.19187	0.05164	0.05164	turquoise
ZNF211	-0.03294	-0.21232	-0.04332	-0.02175	0.0209	0.05028	-0.06543	0.01651	-0.01651	0.66886	0.0053	0.5737	0.77771	0.78617	0.51368	0.39521	0.83027	0.83027	red
COMMD2	-0.09624	-0.09818	-0.05862	0.02376	-0.18394	0.11423	0.11976	0.06114	-0.06114	0.2105	0.20139	0.44629	0.75771	0.01603	0.13683	0.11872	0.427	0.427	turquoise
NAGA	-0.08325	-0.02441	9.88E-05	0.06488	-0.14628	0.07394	0.08043	0.06681	-0.06681	0.27901	0.75133	0.99898	0.39919	0.05625	0.33652	0.29568	0.38524	0.38524	turquoise
CCDC110	-0.0709	1.48E-05	-0.15124	0.01323	-0.04015	-0.10779	-0.16042	0.48603	-0.48603	0.35681	0.99985	0.04831	0.86366	0.60213	0.16052	0.03609	1.60E-11	1.60E-11	grey
LMNB2	-0.00497	0.05526	-0.04165	-0.03808	0.00542	-0.00359	0.23742	-0.29036	0.29036	0.94857	0.47283	0.58855	0.62093	0.94395	0.9628	0.00177	0.00012	0.00012	pink
PLCH2	0.0179	-0.05308	0.04143	0.13052	0.08741	-0.02056	0.09583	-0.10836	0.10836	0.81627	0.49054	0.59052	0.08883	0.2556	0.78957	0.21246	0.1583	0.1583	grey
ARL3	0.06827	-0.14195	-0.16556	-0.1272	-0.02046	-0.28495	-0.01074	0.21439	-0.21439	0.37495	0.06402	0.03046	0.09734	0.79055	0.00016	0.88913	0.00487	0.00487	grey
SUPV3L1	-0.01922	-0.00294	-0.06138	-0.04575	-0.13152	-0.07969	0.16876	-0.09267	0.09267	0.80295	0.96954	0.42514	0.5524	0.08641	0.30018	0.02735	0.228	0.228	grey
FBXL19	0.03081	-0.04171	-0.04512	0.02943	0.10416	0.01841	0.20096	-0.36984	0.36984	0.68914	0.58808	0.55789	0.70234	0.17518	0.81111	0.0084	6.41E-07	6.41E-07	green
ULK4	0.058	-0.2395	-0.09645	-0.12895	-0.01615	-0.02772	-0.10604	0.10161	-0.10161	0.45113	0.00161	0.20949	0.09279	0.83389	0.71894	0.16745	0.18604	0.18604	grey
CA3	-0.05105	-0.01794	0.027	0.08301	0.05278	0.06256	-0.04085	0.0988	-0.0988	0.50726	0.81588	0.72595	0.2804	0.49299	0.41631	0.59575	0.19856	0.19856	grey
NSG2	0.04916	-0.16982	-0.17024	-0.06627	0.05837	-0.28472	-0.15217	0.42781	-0.42781	0.52311	0.02638	0.026	0.38911	0.44826	0.00016	0.04694	5.33E-09	5.33E-09	brown
NDUFA11	-0.00672	0.06178	-0.16613	-0.0841	0.23289	-0.22637	0.0226	-0.22347	0.22347	0.93045	0.4221	0.02989	0.27413	0.00217	0.00291	0.76926	0.0033	0.0033	green
RAPGEF4	-0.04178	-0.17607	-0.1923	-0.05912	-0.00887	-0.25384	-0.23111	0.56197	-0.56197	0.5874	0.02125	0.01174	0.44246	0.90828	0.00081	0.00236	1.27E-15	1.27E-15	brown
HOXA11	0.03527	-0.05772	0.12731	0.09214	-0.18044	0.15717	0.15701	-0.1872	0.1872	0.647	0.45335	0.09703	0.23067	0.01819	0.04007	0.04029	0.01422	0.01422	grey
CCT8	-0.00321	-0.02607	-0.07548	-0.05368	0.02155	-0.03397	0.06135	-0.06836	0.06836	0.96676	0.73502	0.32647	0.48559	0.77966	0.65915	0.42536	0.37434	0.37434	turquoise
ZHX3	-0.00035	-0.06	-0.12763	0.01697	-0.05918	-0.18516	-0.08116	0.55514	-0.55514	0.99636	0.43567	0.0962	0.82567	0.44194	0.01533	0.29128	3.29E-15	3.29E-15	turquoise
RGL2	-0.04543	-0.09629	-0.05268	-0.01966	0.13579	-0.06836	0.03599	0.02403	-0.02403	0.55522	0.21025	0.4938	0.7986	0.07659	0.37429	0.64026	0.75504	0.75504	red
TIA1	-0.02527	-0.13946	-0.03443	-0.00499	0.05544	-0.01672	0.02623	-0.00926	0.00926	0.74284	0.06888	0.65479	0.94835	0.47143	0.82815	0.73342	0.90435	0.90435	red
GPX2	0.04611	0.12151	0.07354	0.04462	0.15714	0.18384	0.02061	-0.29664	0.29664	0.54924	0.11337	0.33913	0.56229	0.04011	0.01609	0.789	8.16E-05	8.16E-05	yellow
SUDS3	0.12318	-0.12045	0.03865	0.03194	0.01635	0.05648	0.00325	-0.06489	0.06489	0.10848	0.11659	0.61574	0.67839	0.83195	0.46315	0.9664	0.39912	0.39912	turquoise
NDUFC1	-0.03411	-0.12473	-0.15092	-0.11461	0.24382	-0.15997	-0.1086	-0.29469	0.29469	0.65788	0.10405	0.0488	0.13554	0.00131	0.03661	0.15739	9.13E-05	9.13E-05	grey
TLCD2	0.02224	-0.06393	0.07206	0.04359	0.00584	0.22077	0.24109	-0.2813	0.2813	0.77279	0.40613	0.34893	0.5713	0.93955	0.00371	0.00149	0.00019	0.00019	turquoise
TNFAIP8	-0.10342	-0.06682	0.11376	0.0863	-0.20719	0.25036	0.12953	0.05243	-0.05243	0.17826	0.38522	0.13849	0.26171	0.00655	0.00096	0.09131	0.49586	0.49586	turquoise
SWT1	0.00858	-0.04728	0.04614	0.08286	-0.07356	0.15833	0.11027	0.1355	-0.1355	0.91128	0.5392	0.54903	0.28125	0.33897	0.03862	0.15107	0.07722	0.07722	turquoise
PLPP7	-0.15657	-0.05491	-0.16295	-0.03093	-0.09144	-0.10683	0.00329	0.42543	-0.42543	0.04085	0.47563	0.03322	0.68797	0.23425	0.16432	0.96596	6.62E-09	6.62E-09	blue
CRYBG2	0.03519	-0.06182	0.12289	0.06519	0.0222	0.22154	0.176	-0.46411	0.46411	0.64776	0.42185	0.10932	0.39694	0.7732	0.00359	0.0213	1.62E-10	1.62E-10	yellow
SUN2	-0.02314	-0.07891	-0.01041	0.07862	-0.17393	0.08397	-0.0209	0.07452	-0.07452	0.7639	0.30496	0.89246	0.30674	0.0229	0.27487	0.7861	0.33273	0.33273	turquoise
SLC35A2	0.02509	-0.10149	-0.00998	0.03582	-0.00886	0.03977	0.17119	-0.42008	0.42008	0.74465	0.18655	0.89688	0.64182	0.90844	0.60557	0.02517	1.07E-08	1.07E-08	pink
PDE6B	-0.10594	-0.08311	-0.13668	0.01734	-0.01843	-0.11576	-0.08958	0.37006	-0.37006	0.16789	0.27982	0.07465	0.82185	0.81094	0.13164	0.24393	6.30E-07	6.30E-07	grey
CBX6	-0.09686	-0.17055	-0.17214	-0.02286	-0.06524	-0.23998	-0.19159	0.50805	-0.50805	0.20757	0.02573	0.02437	0.76662	0.3966	0.00157	0.01206	1.31E-12	1.31E-12	brown
ZNF33B	0.07945	-0.01078	-0.06665	-0.01796	-0.02732	-0.06582	0.00739	-0.0455	0.0455	0.30164	0.88873	0.3864	0.81563	0.72281	0.39239	0.92356	0.55458	0.55458	grey
FRG1	-0.01827	-0.06748	-0.06447	-0.06938	0.00934	-0.00618	-0.00016	-0.03448	0.03448	0.81253	0.38051	0.40216	0.36725	0.9035	0.9361	0.99835	0.65437	0.65437	turquoise
FHAD1	0.01119	-0.08957	-0.0711	-0.01509	-0.01606	0.02932	0.07259	-0.03344	0.03344	0.88449	0.24401	0.35545	0.84469	0.83484	0.70339	0.34543	0.66418	0.66418	grey
HIST1H2A	-0.05557	-0.06837	0.12243	0.12226	-0.13889	0.18478	0.27961	-0.2835	0.2835	0.47039	0.37422	0.11067	0.11116	0.07003	0.01554	0.00021	0.00017	0.00017	grey
MYC	-0.16177	0.05757	0.03071	0.09569	-0.1359	0.24195	0.15365	0.10036	-0.10036	0.03453	0.45452	0.69006	0.21315	0.07635	0.00143	0.04481	0.19154	0.19154	grey
MAP3K11	-0.00394	-0.04638	-0.10915	-0.04532	0.09473	-0.01803	0.06504	-0.30642	0.30642	0.95924	0.54693	0.15528	0.55617	0.21779	0.81495	0.39799	4.58E-05	4.58E-05	green
KRT80	0.10246	-0.21192	0.00781	0.09913	-0.15541	0.14309	0.04815	-0.25761	0.25761	0.18238	0.00539	0.91926	0.19705	0.04239	0.06189	0.53169	0.00067	0.00067	yellow
RCC2	-0.05226	-0.00856	0.06255	0.06468	-0.1109	0.19516	0.16384	-0.21267	0.21267	0.49727	0.91155	0.41634	0.40062	0.14875	0.01053	0.03225	0.00523	0.00523	turquoise
TRAPPC6E	0.01749	-0.14653	0.01615	0.07456	-0.05806	0.02402	0.01414	0.0745	-0.0745	0.82036	0.05582	0.83391	0.33246	0.45063	0.75512	0.85436	0.33281	0.33281	turquoise

GAR1	-0.04959	-0.03348	-0.01908	-0.03386	-0.04535	0.06379	0.24741	-0.3092	0.3092	0.51949	0.66375	0.80435	0.66016	0.55584	0.40714	0.0011	3.87E-05	3.87E-05	pink
LAMA4	-0.12272	-0.08707	0.06232	0.06882	-0.20569	0.11283	0.05859	0.2712	-0.2712	0.10982	0.25748	0.41805	0.37108	0.00695	0.14173	0.44656	0.00033	0.00033	blue
DHTKD1	0.02485	-0.01996	0.03787	0.00037	-0.0477	0.03846	0.00747	0.02056	-0.02056	0.74696	0.79555	0.62291	0.99612	0.53555	0.61747	0.92276	0.78955	0.78955	turquoise
CD3D	-0.08369	-0.00291	0.03924	0.03916	-0.14596	0.14576	0.02696	0.28521	-0.28521	0.27645	0.96989	0.61038	0.61109	0.0568	0.05713	0.72634	0.00016	0.00016	blue
ZNF443	0.01608	-0.11629	-0.11611	-0.03866	0.19501	0.00012	-0.06166	0.00074	-0.00074	0.83465	0.12986	0.13047	0.61563	0.01059	0.99879	0.42303	0.9923	0.9923	grey
PSMG2	0.01982	-0.10052	0.05049	0.03024	0.00351	0.06462	0.17745	-0.18358	0.18358	0.79694	0.19083	0.51193	0.69462	0.96367	0.40108	0.02024	0.01624	0.01624	turquoise
CYP27C1	-0.03249	-0.09239	0.05453	0.07542	-0.11356	0.05944	0.12892	-0.17512	0.17512	0.67314	0.22942	0.47874	0.32689	0.13918	0.43999	0.09285	0.02197	0.02197	grey
ZNF784	0.14171	0.03321	-0.1278	-0.08517	0.21799	-0.07973	-0.03731	-0.20891	0.20891	0.06448	0.6663	0.09577	0.26805	0.00418	0.29989	0.62801	0.0061	0.0061	grey
SPATS2	0.00977	-0.2376	-0.07448	0.0174	0.00675	-0.1202	-0.07359	0.17394	-0.17394	0.89903	0.00175	0.33297	0.82133	0.93014	0.11736	0.33878	0.02289	0.02289	turquoise
TDRP	0.01143	-0.16811	-0.18792	0.02264	0.10714	-0.24945	-0.3024	0.51896	-0.51896	0.88208	0.02795	0.01384	0.76885	0.16307	0.001	5.82E-05	3.55E-13	3.55E-13	brown
TRIM9	-0.03082	-0.13304	-0.17292	-0.03398	0.06483	-0.27268	-0.14031	0.4255	-0.4255	0.68907	0.0828	0.02372	0.65904	0.39956	0.00031	0.06719	6.58E-09	6.58E-09	brown
RFTN1	-0.16846	-0.07504	0.04573	0.0118	-0.19808	0.15223	-0.04598	0.34931	-0.34931	0.02763	0.3293	0.55259	0.87823	0.0094	0.04685	0.55037	2.83E-06	2.83E-06	blue
MAP3K8	-0.08845	-0.08333	0.11283	0.08087	-0.18469	0.14396	0.1069	0.21173	-0.21173	0.24999	0.27854	0.14176	0.29301	0.01559	0.06031	0.16402	0.00544	0.00544	blue
PTP4A1	-0.00424	-0.13822	-0.0479	0.07063	-0.0316	0.07298	0.05139	0.07056	-0.07056	0.9561	0.0714	0.53382	0.35864	0.68159	0.34283	0.50442	0.35914	0.35914	turquoise
TMEM237	-0.08516	-0.10592	-0.05	0.02597	-0.14864	-0.0414	0.08422	0.21153	-0.21153	0.2681	0.16796	0.51603	0.73597	0.05234	0.59082	0.27346	0.00548	0.00548	turquoise
SOX15	0.03485	0.09598	-0.11963	-0.05255	0.10304	-0.07336	0.00829	0.08192	-0.08192	0.65094	0.21174	0.11913	0.49486	0.1799	0.34033	0.91426	0.28683	0.28683	grey
CDH5	-0.16419	-0.13463	-0.06561	0.06841	-0.0817	0.0199	-0.14353	0.56025	-0.56025	0.03188	0.07916	0.39391	0.37394	0.28811	0.79613	0.06109	1.62E-15	1.62E-15	blue
MCCC1	-0.04271	-0.08522	-0.0922	-0.01579	-0.0927	0.07993	0.03337	0.1075	-0.1075	0.57912	0.26778	0.2304	0.83763	0.22783	0.29873	0.66482	0.16168	0.16168	grey
GID4	-0.09526	-0.15103	-0.05097	0.04246	-0.11339	-0.03382	-0.01704	0.42369	-0.42369	0.21519	0.04864	0.50797	0.58135	0.13976	0.66052	0.82493	7.74E-09	7.74E-09	turquoise
PRPF4	-0.05589	-0.09674	-0.04606	-0.00321	-0.18995	0.04165	0.09507	0.07517	-0.07517	0.46782	0.20812	0.54971	0.96677	0.01283	0.58858	0.21612	0.32848	0.32848	turquoise
RASAL2	0.01133	-0.12205	0.0132	0.05589	-0.16858	0.12064	0.12983	0.11071	-0.11071	0.88311	0.11178	0.86391	0.46779	0.02751	0.11601	0.09056	0.14945	0.14945	turquoise
SH3D19	-0.03894	-0.04045	0.0545	0.06418	-0.11364	0.26609	0.06726	0.18359	-0.18359	0.61307	0.5994	0.47895	0.4043	0.13889	0.00044	0.38211	0.01623	0.01623	turquoise
CASTOR3	-0.00284	-0.1525	-0.13632	0.058	0.01943	-0.14117	-0.12894	0.3636	-0.3636	0.97061	0.04645	0.07544	0.45114	0.80086	0.06551	0.09281	1.02E-06	1.02E-06	grey
TTLL6	-0.01533	-0.12098	-0.20126	-0.15823	0.27802	-0.24005	-0.16413	0.13541	-0.13541	0.84224	0.11498	0.0083	0.03873	0.00023	0.00156	0.03195	0.07742	0.07742	brown
CDC73	-0.04057	-0.08701	0.01488	0.03272	-0.11108	0.13311	0.09363	0.02966	-0.02966	0.59827	0.25778	0.84682	0.67093	0.14807	0.08265	0.2232	0.70014	0.70014	turquoise
FPGT	-0.05953	-0.07145	-0.02664	0.00818	-0.07376	0.12727	0.07118	0.09501	-0.09501	0.43926	0.35307	0.72945	0.9154	0.33768	0.09714	0.35492	0.21643	0.21643	turquoise
YRDC	-0.09074	-0.04939	-0.03928	-0.04464	-0.04456	-0.06433	-0.06824	0.11045	-0.11045	0.23786	0.5212	0.61002	0.56209	0.56277	0.40323	0.37519	0.15041	0.15041	turquoise
TET3	-0.03234	-0.0987	-0.01001	0.02995	-0.11065	0.1907	0.15148	-0.0965	0.0965	0.67457	0.19901	0.89663	0.69735	0.14965	0.01248	0.04796	0.20927	0.20927	turquoise
SRCIN1	-0.00421	-0.16435	-0.22987	-0.06836	0.25966	-0.32008	-0.24679	0.17098	-0.17098	0.95643	0.03171	0.00249	0.3743	0.0006	1.97E-05	0.00114	0.02536	0.02536	brown
CCT7	0.04734	0.00236	-0.0749	-0.07123	-0.05712	-0.09142	0.14314	-0.16971	0.16971	0.53866	0.97556	0.33023	0.35455	0.45806	0.23435	0.0618	0.02648	0.02648	turquoise
LGR6	0.03634	0.14808	0.03216	0.06565	-0.08413	0.11231	0.2116	-0.22459	0.22459	0.63697	0.05325	0.67627	0.39361	0.27394	0.14361	0.00546	0.00315	0.00315	grey
CCDC120	0.00302	-0.17343	-0.02268	0.04705	0.08298	0.01623	0.08948	-0.44914	0.44914	0.96874	0.0233	0.76847	0.54115	0.28061	0.83316	0.24451	7.20E-10	7.20E-10	yellow
ZBTB17	-0.02384	-0.07136	-0.06699	0.00283	0.06538	0.01695	0.02636	-0.03493	0.03493	0.7569	0.35367	0.38401	0.97074	0.39554	0.82584	0.73217	0.65018	0.65018	green
APBB1	-0.04647	-0.15641	-0.23092	-0.07502	0.008	-0.30593	-0.24957	0.51145	-0.51145	0.54611	0.04106	0.00238	0.32947	0.91731	4.72E-05	0.001	8.77E-13	8.77E-13	brown
RPS19BP1	0.05624	0.09899	-0.09473	-0.01819	0.11013	-0.14139	0.04807	-0.26279	0.26279	0.46498	0.19769	0.2178	0.81336	0.1516	0.0651	0.53241	0.00052	0.00052	grey
HPCA	-0.0328	-0.12632	-0.17409	-0.07593	0.16448	-0.24198	-0.21085	0.30561	-0.30561	0.67015	0.09969	0.02277	0.32363	0.03158	0.00143	0.00564	4.81E-05	4.81E-05	brown
RIC3	0.01806	-0.11648	-0.23955	-0.06814	0.15813	-0.27299	-0.26664	0.42158	-0.42158	0.81462	0.12921	0.0016	0.3759	0.03885	0.0003	0.00042	9.34E-09	9.34E-09	brown
DHX34	0.0547	-0.02269	-0.07459	-0.0313	0.11287	-0.01888	0.09411	-0.24769	0.24769	0.47733	0.76833	0.33228	0.68448	0.14161	0.80633	0.22082	0.00109	0.00109	red
SPINK5	0.04647	0.04408	0.14291	-0.01748	0.16861	0.22975	0.12176	-0.28861	0.28861	0.54617	0.56702	0.06223	0.82047	0.02749	0.0025	0.11264	0.00013	0.00013	grey
PLXNA1	-0.02418	-0.10985	-0.00697	0.0495	-0.15132	0.01294	0.18903	-0.10194	0.10194	0.75356	0.15263	0.92791	0.52023	0.0482	0.86661	0.01328	0.18462	0.18462	black
MTREX	-0.05033	-0.06401	-0.02997	-0.04857	-0.03322	0.02328	0.0311	0.09429	-0.09429	0.5133	0.40556	0.69717	0.52811	0.66622	0.76252	0.68636	0.21996	0.21996	turquoise
XRCC6	-0.06572	0.02031	-0.05747	-0.04461	-0.08316	-0.04556	0.1238	-0.01871	0.01871	0.39309	0.79202	0.45528	0.56232	0.27953	0.55408	0.1067	0.80811	0.80811	turquoise
CREBRF	-0.0444	-0.20508	-0.06171	-0.00019	-0.14751	0.0158	-0.08867	0.30962	-0.30962	0.56415	0.00713	0.42269	0.998	0.05419	0.83744	0.24881	3.78E-05	3.78E-05	turquoise
GPT2	-0.07035	0.12066	0.0285	0.03603	-0.0082	0.05216	0.02568	-0.19182	0.19182	0.36052	0.11594	0.71136	0.63986	0.91518	0.49808	0.73886	0.01196	0.01196	grey
TXNDC5	-0.06889	-0.006	0.11282	0.05146	-0.04973	0.15419	-0.00346	0.36248	-0.36248	0.37064	0.93792	0.14177	0.50389	0.5183	0.04405	0.96415	1.10E-06	1.10E-06	turquoise
DCBLD1	-0.03505	-0.08077	0.0564	0.06524	-0.20182	0.05689	0.1637	0.05704	-0.05704	0.64899	0.29363	0.46374	0.39659	0.00812	0.45985	0.03241	0.45867	0.45867	black
TMEM14C	0.00598	0.07019	0.04203	-0.00786	0.02336	0.06725	0.10994	-0.10916	0.10916	0.93813	0.36162	0.58523	0.9187	0.76165	0.38214	0.15231	0.15526	0.15526	turquoise
TIMP2	-0.04345	-0.10277	0.02448	0.04557	-0.20114	0.06055	0.08013	0.30679	-0.30679	0.57259	0.18103	0.75065	0.55399	0.00834	0.43143	0.29747	4.48E-05	4.48E-05	black
XIAP	-0.01278	-0.15351	-0.06623	-0.01266	-0.05291	0.02823	-0.00892	0.09114	-0.09114	0.86826	0.045	0.38942	0.86941	0.4919	0.71398	0.90782	0.2358	0.2358	turquoise
PDCD10	-0.02179	-0.0251	0.03223	0.0115	-0.12524	0.16328	0.19203	-0.18966	0.18966	0.77728	0.74453	0.67558	0.88132	0.10264	0.03285	0.01186	0.01297	0.01297	turquoise
FCGR3A	-0.10331	0.04797	0.07685	0.05951	-0.25421	0.14881	0.15825	0.21668	-0.21668	0.17873	0.53327	0.31779	0.4394	0.00079	0.05207	0.03871	0.00442	0.00442	blue
ST14	0.06399	0.0182	0.05635	0.06011	-0.02086	0.18725	0.24186	-0.52112	0.52112	0.40569	0.8132	0.46416	0.43482	0.78659	0.01419	0.00144	2.73E-13	2.73E-13	yellow
NFAT5	-0.00234	-0.16846	0.0212	0.06693	-0.14944	0.14151	0.10216	0.16523	-0.16523	0.97575	0.02763	0.78309	0.38443	0.05108	0.06486	0.18365	0.03079	0.03079	turquoise
CNKSR1	0.08024	-0.06432	0.0447	0.01786	0.14601	0.11847	0.09233	-0.49191	0.49191	0.29681	0.40329	0.56154	0.8166	0.05671	0.12276	0.22971	8.34E-12	8.34E-12	yellow
TXNRD3	-0.09831	0.04814	-0.1063	0.0696	-0.19679	-0.01623	0.25727	0.09667	-0.09667	0.20082	0.53181	0.16644	0.36573	0.00989	0.83309	0.00068	0.20847	0.20847	turquoise
C1QTNF7	-0.03591	-0.0686	-0.03972	0.02945	-0.12891	0.18412	0.03283	0.44292	-0.44292	0.64104	0.37264	0.60599	0.70221	0.09288	0.01592	0.66992	1.31E-09	1.31E-09	blue
REEP4	0.03702	0.0244	0.02442	-0.0297	0.11858	-0.10658	0.18432	-0.34396	0.34396	0.6307	0.75143	0.75123	0.69979	0.12242	0.16529	0.01581	4.10E-06	4.10E-06	grey
B9D2	0.01581	-0.07817	-0.1809	-0.04012	0.17988	-0.11887	-0.0904	-0.07268	0.07268	0.83734	0.30948	0.01789	0.60237	0.01856	0.12151	0.23964	0.34483	0.34483	grey
TMEM229	0.03475	-0.09874	-0.09159	-0.06262	0.10775	-0.05149	-0.09054	0.09786	-0.09786	0.65182	0.19883	0.23348	0.41587	0.16069	0.50358	0.23894	0.20289	0.20289	grey
RNF175	-0.16681	-0.05022	0.05377	0.08713	-0.09599	0.06838	-0.03528	0.3264	-0.3264	0.02921	0.5142	0.48489	0.25712	0.2117	0.37421	0.64687	1.32E-05	1.32E-05	blue
RPS21	0.1601	0.17492	0.00785	-0.0375	0.03763	0.00927	0.1547	-0.37593	0.37593	0.03646	0.02212	0.91886	0.62627	0.62513	0.90423	0.04335	4.04E-07	4.04E-07	purple
GRID1	-0.1304	-0.11099	-0.10661	0.04917	-0.15925	0.0218	-0.06344	0.38128	-0.38128	0.08914	0.1484	0.16519	0.52308	0.03748	0.77712	0.40978	2.68E-07	2.68E-07	blue
TRAF7	0.09745	0.02214	0.03348	-0.00211	-0.00349	0.10225	0.09023	-0.1842	0.1842	0.20481	0.77374	0.66376	0.97815	0.96384	0.18324	0.24051	0.01587	0.01587	turquoise
TBC1D7	-0.02463	0.03491	-0.05761	-0.04685	0.0899	-0.06961	0.15471	-0.15562	0.15562	0.7491	0.65036	0.45417	0.54921	0.24229	0.36562	0.04334	0.04211	0.04211	grey
MAMDC4	-0.06243	-0.06317	0.00631	-0.01355	0.20522	-0.0585	0.0157	-0.14604	0.14604	0.41728	0.41177	0.93474	0.86037	0.00709	0.44721	0.83849	0.05665	0.05665	red
ITGB1BP1	-0.0271	-0.06799	-0.00518	0.0227	-0.12641	0.04142	0.13641	0.06386	-0.06386	0.72495	0.37692	0.94641	0.7682	0.09944	0.59067	0.07524	0.40663	0.40663	turquoise
PRPF38A	-0.02577	-0.15358	-0.01259	-0.00794	-0.07155	0.13068	0.083	0.0544	-0.0544	0.73792	0.0449	0.87021	0.91794	0.3524	0.08844	0.28046	0.47979	0.47979	turquoise
PHKG2	0.04825	-0.06543	-0.0854	-0.07924	0.14127	-0.08298	0.07244	-0.38752	0.38752	0.53086	0.39519	0.26675	0.3029	0.06532	0.28056	0.34641	1.64E-07	1.64E-07	red
MAP2K6	-0.06634	0.11264	-0.14788	-0.18635	0.15532	-0.13793	-0.1042	0.10772	-0.10772	0.3886	0.14243	0.05357	0.01467	0.04251	0.072	0.0674	0.16081	0.16081	grey
GPRC5B	-0.04087	-0.11224	0.07066	0.11867	-0.15073	0.27515	0.11058	0.03778	-0.03778	0.59564	0.14386	0.35845	0.12213	0.04908	0.00027	0.14991	0.62373	0.62373	grey
FRMD6	-0.06792	-0.10019	0.06618	0.11426	-0.21298	0.17944	0.16294	0.18508	-0.18508	0.37743	0.19229	0.3898	0.13672	0.00516	0.01885	0.03322	0.01537	0.01537	black
ARFGEF3	0.04526	-0.13794	-0.14217	-0.129	0.14402	-0.2137	-0.21857	0.13544	-0.13544	0.55663	0.07199	0.06361	0.09265	0.06021	0.00501	0.00408	0.07735	0.07735	brown
AKAP5	-0.01896	-0.15054	0.12055	0.04377	-0.07094	0.23004	0.02963	0.14027	-0.14027	0.80558	0.04938	0.11628	0.5697	0.35652	0.00247	0.7005	0.06728	0.06728	turquoise
ATP13A2	-0.0116	-0.05039	0.01466	0.03943	0.08004	-0.0274	0.00515	0.012	-0.012	0.88033	0.51274	0.84911	0.60862	0.29802	0.72202	0.94673	0.87619	0.87619	grey
BTBD10	0.06279	-0.07013	0.07879	-0.01092	-0.06053	0.13817	0.15032	-0.19181	0.19181	0.41461	0.36207	0.3057	0.88729	0.43164	0.07152	0.04971	0.01196	0.01196	turquoise
ZNF354B	-0.00159	-0.16363	-0.06996	5.70E-05	0.01928	-0.00863	0.0244	0.10489	-0.10489	0.9835	0.03248	0.36324	0.99941	0.80239	0.91078	0.75145	0.17215	0.17215	red
PRICKLE4	-0.08743	-0.0066	-0.15096	-0.15587	0.23247	-0.07002	0.05476	-0.13207	0.13207	0.25552	0.93169	0.04874	0.04178	0.00222	0.36279	0.47687	0.08508	0.08508	grey
ZNF267	-0.03926	-0.05301	0.06284	0.10497	-0.0889	0.20815	0.12394	-0.00332	0.00332	0.61021	0.49104	0.41419	0.1718	0.24755	0.0063	0.10629	0.96561	0.96561	turquoise
PSMC3	0.0586	-0.01225	-0.0185	-0.01609	-0.05412	-0.05	0.12136	-0.25502	0.25502	0.44643	0.87362	0.81022	0.83452	0.48207	0.51605	0.11385	0.00076	0.00076	grey
GRK4	-0.08973	-0.23898	-0.17058	-0.06289	-0.00441	-0.28247	-0.2149	0.34538	-0.34538	0.24316	0.00164	0.0257	0.41386	0.95431	0.00018	0.00476	3.72E-06	3.72E-06	brown
LCA5	-0.03647	-0.24519	0.01152	0.11588	-0.1855	0.06153	0.00605	0.38466	-0.38466	0.63583	0.00123	0.88115	0.13123	0.01514	0.42401	0.93738	2.05E-07	2.05E-07	turquoise
PNMA8B	0.07284	-0.05263	-0.15781	-0.03326	-0.04494	-0.17051	-0.11932	0.46621	-0.46621	0.34375	0.49422	0.03927	0.66581	0.55947	0.02577	0.1201	1.31E-10	1.31E-10	brown
RBBP8NL	0.15836	0.12739	-0.04364	0.06093	0.20129	0.05814	0.13351	-0.3893	0.3893	0.03857	0.09685	0.57087	0.42852	0.00829	0.45002	0.0817	1.42E-07	1.42E-07	grey
SH3BP5	-0.14213	-0.13114	0.08947	0.11044	-0.17088	0.1331	-0.02782	0.47621	-0.47621	0.06369	0.08732	0.24453	0.15043	0.02544	0.08266	0.71798	4.61E-11	4.61E-11	blue
GPR161	-0.08184	-0.04467	0.06096	0.06853	-0.29917	0.17789	0.10212	0.26501	-0.26501	0.28724	0.56186	0.42837	0.37315	7.04E-05	0.01993	0.18381	0.00046	0.00046	blue
AKAP3	-0.10204	-0.00913	-0.0924	-0.11414	-0.00588	-0.06503	-0.03931	0.25273	-0.25273	0.18416	0.90562	0.22935	0.13715	0.93919	0.39806	0.60968	0.00085	0.00085	grey
GPR180	-0.02613	-0.14927	-0.0541	-0.04139	-0.04099	-0.17027	0.00632	0.22481	-0.22481	0.73446	0.05135	0.48218	0.59094	0.59456	0.02598	0.93457	0.00311	0.00311	turquoise
MYL9	-0.09349	-0.00862	0.02187	0.06543	-0.14561	0.10908	0.08108	0.19657	-0.19657	0.22387	0.91085	0.77644	0.39522	0.0574	0.15555	0.29178	0.00997	0.00997	salmon
PHLDB2	-0.10142	-0.21069	-0.0772	0.02817	-0.16783	-0.07858	-0.08405	0.48337	-0.48337	0.18688	0.00567	0.31559	0.71451	0.02822	0.307	0.27439	2.14E-11	2.14E-11	blue
NAT1	-0.04636	-0.02346	0.15796	0.0787	0.05679	0.16388	0.06696	-0.11616	0.11616	0.54712	0.76074	0.03908	0.30624	0.46067	0.03221	0.38423	0.13029	0.13029	grey
BAIAP2	0.01266	-0.14991	-0.14089	-0.1495	0.10799	-0.12222	0.11746	-0.23827	0.23827	0.86948	0.05035	0.06605	0.05098	0.15974	0.11128	0.12601	0.0017	0.0017	grey
ITGAM	-0.11261	-0.00745	0.13138	0.09332	-0.1544	0.2215	0.12404	0.21902	-0.21902	0.14253	0.92299	0.08674	0.22476	0.04376	0.0036	0.10602	0.004	0.004	blue
WHAMM	0.01294	-0.19607	-0.06756	0.02441	0.01473	-0.01081	-0.02132	-0.07752	0.07752	0.86658	0.01017	0.37995	0.75136	0.84837	0.88836	0.78191	0.31353	0.31353	turquoise
ATG10	0.00202	-0.05758	-0.11813	-0.04159	-0.07012	-0.19158	-0.02537	0.0703	-0.0703	0.97907	0.4544	0.12384	0.58909	0.36214	0.01207	0.74191	0.36086	0.36086	turquoise
LTO1	0.02777	-0.05716	-0.12695	-0.06667	0.06344	-0.07129	0.01745	-0.23167	0.23167	0.71846	0.45776	0.09799	0.38629	0.40976	0.35414	0.82083	0.0023	0.0023	red
TES	-0.01755	-0.09644	0.11908	0.1391	-0.17357	0.20652	0.12269	-0.2457	0.2457	0.81978	0.20958	0.12084	0.06961	0.02318	0.00673	0.1099	0.0012	0.0012	turquoise
GXYLT1	0.02483	-0.2964	-0.07656	-0.03051	-0.013	-0.10286	-0.11423	0.28668	-0.28668	0.74715	8.27E-05	0.31958	0.69196	0.866	0.18065	0.13685	0.00014	0.00014	turquoise
SLC35G2	-0.01008	-0.14785	-0.15073	0.00469	-0.01887	-0.23635	-0.16964	0.30563	-0.30563	0.89594	0.05362	0.04909	0.9515	0.80652	0.00186	0.02655	4.80E-05	4.80E-05	brown
RSPO1	-0.00144	0.01174	0.0169	0.0457	-0.05891	0.0816	-0.08217	0.45954	-0.45954	0.98505	0.87891	0.82635	0.55281	0.44404	0.28871	0.28534	2.57E-10	2.57E-10	salmon
RAP1B	0.00466	-0.15995	0.07326	0.15217	-0.06734	0.0825	-0.00082	0.07843	-0.07843	0.9518	0.03665	0.341	0.04693	0.38151	0.28337	0.99151	0.30791	0.30791	turquoise
TDO2	-0.1845	-0.1	-0.01482	-0.02251	-0.21919	-0.01624	0.07953	0.39339	-0.39339	0.01571	0.19316	0.84742	0.77013	0.00397	0.83303	0.30115	1.02E-07	1.02E-07	blue
MMP7	0.00268	0.01943	0.0965	0.10666	-0.12532	0.25193	0.13628	-0.19453	0.19453	0.97221	0.80087	0.20928	0.165	0.10243	0.00089	0.07552	0.01079	0.01079	yellow
GSTM2	-0.08404	-0.13152	-0.10481	-0.0575	0.06077	-0.1538	-0.15532	0.38313	-0.38313	0.27445	0.08641	0.17249	0.45505	0.4298	0.0446	0.04251	2.31E-07	2.31E-07	brown
FAM192A	0.04692	-0.04998	-0.04542	-0.08065	-0.04547	0.08996	0.13972	-0.25593	0.25593	0.54225	0.51621	0.55527	0.29436	0.55483	0.24196	0.06837	0.00073	0.00073	turquoise
PAK1IP1	-0.07424	0.0301	0.03583	0.02955	-0.06203	0.06988	0.07688	0.13765	-0.13765	0.33455	0.69598	0.64174	0.70122	0.42024	0.36376	0.31755	0.0726	0.0726	turquoise
CLEC2D	-0.08023	-0.10759	0.06107	0.0932	-0.0841	0.06789	0.03125	0.18946	-0.18946	0.29686	0.16132	0.42751	0.22534	0.27411	0.37764	0.6849	0.01307	0.01307	blue
ADCK5	0.08081	0.0537	-0.01202	-0.03264	0.21341	-0.06724	0.12415	-0.44669	0.44669	0.29337	0.48545	0.87596	0.67173	0.00507	0.38219	0.10569	9.12E-10	9.12E-10	green
TCF21	-0.05659	-0.07893	-0.07101	0.03327	-0.15159	0.14082	0.01328	0.42559	-0.42559	0.46223	0.30481	0.35607	0.66579	0.0478	0.06619	0.86308	6.52E-09	6.52E-09	blue
CRELD2	0.04542	0.12649	-0.18723	-0.06993	0.12847	-0.33569	-0.03444	0.03498	-0.03498	0.55525	0.09924	0.0142	0.36346	0.09401	7.17E-06	0.65471	0.64965	0.64965	grey
LRRK2	-0.12759	-0.02626	-0.02279	0.0836	-0.14413	0.10411	0.00309	0.48344	-0.48344	0.09631	0.73318	0.76737	0.27699	0.06001	0.17538	0.96802	2.12E-11	2.12E-11	blue
CDS1	0.01906	-0.09951	0.01006	-0.05826	0.0864	0.06203	0.03787	-0.3938	0.3938	0.80458	0.19533	0.89611	0.44912	0.26118	0.42026	0.62293	9.89E-08	9.89E-08	turquoise
CLDN11	-0.00291	-0.15487	0.00213	0.04973	-0.21106	0.08308	0.0308	0.34405	-0.34405	0.96985	0.04312	0.97794	0.51834	0.00559	0.28001	0.68919	4.08E-06	4.08E-06	blue
BHLHB9	0.00168	-0.17516	-0.20836	-0.04434	-0.02689	-0.15948	-0.04645	0.44568	-0.44568	0.98257	0.02194	0.00624	0.56475	0.72697	0.0372	0.54634	1.01E-09	1.01E-09	turquoise
ATG16L1	0.09781	-0.04212	0.0391	0.01116	-0.00372	0.14071	0.17945	-0.29609	0.29609	0.20312	0.58439	0.61165	0.88482	0.96152	0.0664	0.01885	8.42E-05	8.42E-05	turquoise
COPS3	-0.07484	-0.08745	-0.09245	-0.05906	-0.00649	-0.19893	-0.06584	0.3798	-0.3798	0.33064	0.25538	0.2291	0.44293	0.93285	0.0091	0.39222	3.00E-07	3.00E-07	turquoise
SPC24	0.0167	0.13256	-0.01679	-0.06038	0.0003	0.03718	0.32568	-0.53725	0.53725	0.82834	0.08393	0.8275	0.43276	0.9969	0.62926	1.38E-05	3.57E-14	3.57E-14	pink
UXT	0.0429	-0.01702	-0.07964	-0.08326	-0.01242	-0.10258	0.07441	-0.25119	0.25119	0.57741	0.82515	0.30048	0.27897	0.87192	0.18187	0.33339	0.00092	0.00092	purple
ALDH2	-0.03484	0.07955	0.11034	0.0313	0.12993	0.17641	-0.03626	-0.02178	0.02178	0.65097	0.30102	0.15082	0.68446	0.09031	0.021	0.63773	0.77733	0.77733	grey
NDUFS1	-0.04821	-0.00358	-0.05321	0.00935	-0.05229	0.03492	0.04708	0.0538	-0.0538	0.53117	0.96296	0.48945	0.90335	0.49695	0.65026	0.54088	0.48461	0.48461	turquoise
EVC2	-0.02584	-0.12227	-0.0345	0.0943	-0.19581	0.1523	0.07237	0.44583	-0.44583	0.73725	0.11122	0.65419	0.2199	0.01027	0.04675	0.34685	9.91E-10	9.91E-10	blue
RPL37A	0.1555	0.16238	-0.02226	-0.00344	-0.01749	0.00535	0.10446	-0.20174	0.20174	0.04227	0.03384	0.77257	0.96439	0.82037	0.94462	0.17392	0.00814	0.00814	purple
NR2C2AP	0.1394	0.08557	-0.06683	-0.0743	0.03392	-0.03621	0.15166	-0.42277	0.42277	0.069	0.26581	0.38516	0.33413	0.65958	0.63826	0.04768	8.40E-09	8.40E-09	grey
CA5B	-0.23079	-0.19046	-0.0599	0.03629	-0.10516	0.04532	-0.1691	0.44913	-0.44913	0.00239	0.01259	0.43642	0.63749	0.17106	0.5561	0.02704	7.21E-10	7.21E-10	turquoise
COG2	0.05027	0.00595	-0.19142	-0.15433	0.11054	-0.03245	0.06693	-0.09532	0.09532	0.51375	0.93843	0.01214	0.04386	0.15007	0.67352	0.3844	0.21493	0.21493	turquoise
UBE2Q2	-0.05025	-0.12405	-0.05492	0.02049	-0.09238	0.00912	0.00783	0.25146	-0.25146	0.51395	0.10599	0.47555	0.79026	0.22948	0.9058	0.91909	0.00091	0.00091	turquoise
TPRG1	-0.04506	-0.03083	0.14725	0.10887	-0.0769	0.23713	0.05995	-0.01061	0.01061	0.55844	0.68897	0.05462	0.15634	0.31746	0.00179	0.43604	0.89047	0.89047	grey
SENP7	-0.12863	-0.15179	-0.10141	-0.00895	-0.03767	0.01138	-0.10167	0.33064	-0.33064	0.0936	0.04749	0.18689	0.90753	0.62469	0.88252	0.18577	1.00E-05	1.00E-05	turquoise
THOC5	-0.03895	0.01797	-0.05694	-0.01357	-0.18687	-0.01802	0.16721	0.08059	-0.08059	0.61296	0.81554	0.45947	0.86017	0.01439	0.81508	0.02882	0.2947	0.2947	turquoise
TMED8	-0.02048	-0.14918	-0.10925	-0.0621	-0.00653	-0.20237	-0.21947	0.40394	-0.40394	0.79035	0.05149	0.1549	0.41974	0.93246	0.00794	0.00392	4.29E-08	4.29E-08	turquoise

CSPP1	-0.02236	-0.0814	-0.06191	0.06884	-0.04289	-0.00295	-0.00466	0.09599	-0.09599	0.77164	0.28989	0.42114	0.371	0.57754	0.96948	0.95176	0.21172	0.21172	turquoise
TMTC2	0.04194	-0.12338	0.10927	0.09106	-0.0632	0.21842	0.11214	-0.0791	0.0791	0.586	0.10789	0.15482	0.23624	0.41152	0.0041	0.14422	0.30375	0.30375	turquoise
POLE	-0.01032	0.02651	0.05059	0.01688	0.02673	0.04642	0.14215	-0.2347	0.2347	0.89342	0.73067	0.51112	0.82651	0.72851	0.54656	0.06365	0.002	0.002	pink
LIPT1	0.00111	-0.00645	-0.04553	-0.07818	-0.0225	0.06796	0.02771	0.04732	-0.04732	0.98853	0.93322	0.5543	0.30947	0.77016	0.37714	0.71899	0.53883	0.53883	turquoise
SYNC	-0.07291	-0.0261	0.0866	0.12957	-0.20536	0.13863	0.09943	0.38283	-0.38283	0.34326	0.73467	0.26008	0.09121	0.00705	0.07056	0.19569	2.37E-07	2.37E-07	blue
TUSC3	-0.01656	-0.16213	-0.20654	-0.08572	0.04585	-0.26432	-0.21638	0.39136	-0.39136	0.82977	0.03412	0.00672	0.26497	0.55148	0.00048	0.00448	1.20E-07	1.20E-07	brown
NMI	0.01027	0.04073	0.11136	0.15547	-0.16675	0.20108	0.22115	-0.25212	0.25212	0.8939	0.59689	0.14704	0.0423	0.02927	0.00836	0.00365	0.00088	0.00088	tan
C14orf132	-0.04854	-0.17764	-0.16105	-0.01358	0.01594	-0.20617	-0.1649	0.55744	-0.55744	0.52843	0.0201	0.03535	0.86006	0.83603	0.00682	0.03114	2.39E-15	2.39E-15	brown
IARS2	-0.07237	-0.03106	-0.00136	-0.02412	-0.08973	0.09685	0.1956	-0.13295	0.13295	0.34685	0.68671	0.98593	0.75414	0.24315	0.20759	0.01035	0.08301	0.08301	turquoise
MAST3	-0.01941	-0.0974	-0.14747	-0.11303	0.1064	-0.26747	-0.24673	0.35159	-0.35159	0.80103	0.20506	0.05425	0.14102	0.16602	0.00041	0.00114	2.41E-06	2.41E-06	brown
WNT2B	-0.1007	-0.20613	-0.091	-0.07329	0.06681	-0.01146	-0.12832	0.36877	-0.36877	0.19001	0.00683	0.23654	0.34075	0.38526	0.88175	0.0944	6.94E-07	6.94E-07	turquoise
ACAP3	2.57E-05	-0.03261	-0.15867	-0.04742	0.19427	-0.20504	-0.04365	-0.0079	0.0079	0.99973	0.67196	0.03819	0.53792	0.01089	0.00714	0.57079	0.91833	0.91833	red
SLC16A13	-0.05527	-0.1324	-0.03539	-0.00672	0.07686	-0.0476	0.05901	-0.2207	0.2207	0.47275	0.08431	0.64589	0.93054	0.31768	0.53642	0.44327	0.00372	0.00372	grey
NSA2	0.03186	-0.01847	-0.04104	-0.0242	-0.05413	-0.00566	-0.02215	0.1225	-0.1225	0.6791	0.81051	0.59403	0.75336	0.48191	0.9414	0.77368	0.11046	0.11046	turquoise
SMARCB1	-0.04385	-0.02886	-0.12577	-0.0816	0.03342	-0.17812	-0.05483	0.04905	-0.04905	0.56902	0.70792	0.1012	0.28869	0.66433	0.01977	0.47631	0.52408	0.52408	grey
SLC25A39	0.03725	0.08856	-0.07155	-0.07024	0.20562	-0.11323	0.10075	-0.48643	0.48643	0.62864	0.24937	0.3524	0.36128	0.00697	0.14032	0.1898	1.53E-11	1.53E-11	green
ZNF675	-0.01486	-0.07234	0.00627	0.06503	-0.01925	0.04636	0.00587	0.15266	-0.15266	0.84706	0.34711	0.93515	0.39809	0.80269	0.54708	0.93929	0.04623	0.04623	turquoise
SNX17	-0.08531	-0.03554	-0.0798	-0.02638	-0.11399	0.01044	0.05672	0.13736	-0.13736	0.26723	0.64446	0.29952	0.73194	0.13768	0.89219	0.46117	0.07321	0.07321	turquoise
PIK3C3	-0.1326	-0.13795	-0.10783	-0.00157	-0.07369	-0.0762	-0.01289	0.41957	-0.41957	0.08383	0.07197	0.16036	0.98371	0.33811	0.3219	0.86709	1.12E-08	1.12E-08	turquoise
CYTL1	-0.04899	-0.0946	0.04583	0.03561	-0.14285	0.05795	-0.05391	0.46544	-0.46544	0.52459	0.21842	0.5517	0.64384	0.06234	0.45156	0.4837	1.42E-10	1.42E-10	blue
POLDIP3	0.0298	-0.06787	-0.14325	-0.02092	-0.08193	-0.07121	-0.01298	0.11648	-0.11648	0.6988	0.37774	0.06159	0.78595	0.28676	0.3547	0.86623	0.12924	0.12924	turquoise
IGF1	-0.05923	-0.01337	0.02256	0.04624	-0.08025	0.07269	-0.08824	0.60142	-0.60142	0.4416	0.86221	0.76966	0.54817	0.29679	0.34474	0.25112	3.37E-18	3.37E-18	blue
FAM189B	0.04649	-0.01327	-0.02978	-0.04878	0.04918	-0.02897	0.19392	-0.3508	0.3508	0.54597	0.86322	0.69898	0.52633	0.52293	0.70682	0.01104	2.55E-06	2.55E-06	greenyellow
RPLP2	0.17146	0.1366	-0.0033	-0.07246	0.0039	0.05731	0.15123	-0.36978	0.36978	0.02494	0.07483	0.96587	0.34629	0.95958	0.45655	0.04832	6.44E-07	6.44E-07	purple
PLA2G1B	0.01674	0.05511	0.03767	0.12608	-0.08322	0.06749	-0.10121	0.13529	-0.13529	0.828	0.47405	0.62468	0.10033	0.27921	0.38042	0.1878	0.07768	0.07768	magenta
LAMB2	-0.01159	-0.08182	0.09807	0.14034	-0.15552	0.22587	0.1722	0.1091	-0.1091	0.88039	0.28738	0.20192	0.06713	0.04223	0.00297	0.02432	0.15547	0.15547	black
ORC3	-0.13254	-0.04575	-0.05263	-0.04305	-0.1099	-0.02605	0.01468	0.26816	-0.26816	0.08396	0.55236	0.4942	0.57606	0.15245	0.73524	0.84882	0.00039	0.00039	turquoise
CAMK2N2	-0.03046	-0.01901	-0.29656	-0.12397	0.07779	-0.32235	-0.19564	0.32493	-0.32493	0.69245	0.80509	8.20E-05	0.1062	0.31185	1.71E-05	0.01034	1.45E-05	1.45E-05	brown
OASL	0.03262	0.12689	0.19612	0.09494	-0.08455	0.24843	0.22502	-0.41017	0.41017	0.67188	0.09816	0.01014	0.21676	0.27156	0.00105	0.00309	2.53E-08	2.53E-08	yellow
LACTB	-0.07164	-0.05428	0.12193	0.06515	-0.13041	0.19697	0.10582	-0.03039	0.03039	0.35179	0.48071	0.11214	0.39721	0.08912	0.00982	0.16836	0.69315	0.69315	turquoise
TULP3	0.0089	-0.15145	0.00826	0.05885	-0.07609	0.0294	-0.00526	0.10978	-0.10978	0.908	0.04801	0.91459	0.44453	0.32261	0.70269	0.94553	0.15288	0.15288	turquoise
WDR89	-0.10335	-0.1166	-0.06278	0.0175	0.00424	-0.0003	-0.0166	0.09785	-0.09785	0.17857	0.12882	0.41462	0.8203	0.95615	0.99687	0.82939	0.20294	0.20294	turquoise
FAM160B2	0.00576	-0.01175	0.04238	-0.00239	0.12525	-0.0521	0.07337	-0.10676	0.10676	0.94043	0.87882	0.58204	0.97526	0.10263	0.4986	0.34027	0.16458	0.16458	red
CNTRL	-0.09929	-0.13847	-0.04959	-0.04244	-0.12577	0.04204	-0.0522	0.26535	-0.26535	0.19633	0.07088	0.5195	0.58153	0.1012	0.58513	0.49771	0.00045	0.00045	turquoise
SMAGP	0.13135	0.06501	0.09251	0.05068	0.02335	0.23441	0.23631	-0.46499	0.46499	0.08681	0.39823	0.22882	0.51037	0.76176	0.00203	0.00186	1.48E-10	1.48E-10	yellow
HSD17B8	0.06973	0.21713	-0.05406	-0.02732	0.19964	-0.06715	0.01142	-0.36834	0.36834	0.36478	0.00434	0.48248	0.72277	0.00885	0.38284	0.88216	7.17E-07	7.17E-07	grey
FAM136A	-0.00966	0.01783	0.02388	0.03494	-0.08403	0.07327	0.20738	-0.48479	0.48479	0.90017	0.81699	0.75657	0.65009	0.2745	0.34093	0.0065	1.83E-11	1.83E-11	pink
HCST	-0.14388	0.05579	-0.00519	0.02605	-0.05477	0.05301	0.0329	0.26041	-0.26041	0.06045	0.46857	0.94625	0.73523	0.47678	0.49104	0.66927	0.00058	0.00058	blue
TSPAN12	-0.04404	-0.07154	-0.18463	-0.08545	0.20339	-0.08806	-0.19276	0.22926	-0.22926	0.56734	0.35243	0.01563	0.26647	0.00763	0.25207	0.01154	0.00256	0.00256	turquoise
RPLP1	0.09154	0.08322	-0.03569	-0.04709	0.06131	0.04112	0.11866	-0.35712	0.35712	0.23375	0.27916	0.64302	0.54079	0.42569	0.59332	0.12216	1.63E-06	1.63E-06	purple
SKI	-0.08733	-0.13889	0.01275	0.0818	-0.19119	0.13229	0.01507	0.23351	-0.23351	0.25602	0.07003	0.86854	0.28748	0.01225	0.08455	0.84488	0.00211	0.00211	turquoise
GUF1	0.04577	1.33E-05	-0.15938	-0.149	0.0313	-0.09754	0.01583	0.00329	-0.00329	0.55219	0.99986	0.03732	0.05177	0.68445	0.20438	0.83714	0.96592	0.96592	turquoise
GPRC5C	0.1262	-0.04159	-0.05251	-0.06374	0.24504	-0.04272	-0.04321	-0.43056	0.43056	0.10001	0.58908	0.49518	0.40756	0.00124	0.57908	0.57466	4.15E-09	4.15E-09	grey
RCN2	-0.0949	-0.16344	-0.06142	-0.00019	-0.07284	0.01038	-0.00312	0.08636	-0.08636	0.21695	0.03269	0.42486	0.99804	0.34378	0.89286	0.96772	0.26138	0.26138	turquoise
SLC16A4	0.10688	-0.06352	0.01386	0.07078	0.05945	0.14209	0.09117	-0.03777	0.03777	0.16412	0.40914	0.85723	0.35761	0.43989	0.06375	0.23563	0.62384	0.62384	turquoise
HDAC4	0.02763	-0.07659	-0.13201	-0.03913	0.01617	-0.10476	-0.08947	0.38918	-0.38918	0.71983	0.31942	0.08522	0.61135	0.8337	0.1727	0.24451	1.43E-07	1.43E-07	turquoise
ZNF561	-0.05389	-0.14454	-0.18069	-0.04832	-0.0071	-0.04521	-0.08206	0.21187	-0.21187	0.48391	0.05928	0.01803	0.53025	0.9266	0.55707	0.28596	0.0054	0.0054	turquoise
ASCL2	-0.04158	0.0181	-0.12061	-0.05021	0.04711	-0.09138	0.0636	0.17351	-0.17351	0.58921	0.81422	0.1161	0.51427	0.54065	0.23459	0.4086	0.02324	0.02324	grey
SNX11	-0.03063	-0.11697	-0.10787	-0.05861	0.01148	-0.13731	-0.12645	0.06909	-0.06909	0.69083	0.12762	0.16023	0.4464	0.8815	0.07331	0.09933	0.36923	0.36923	grey
TADA1	-0.03045	-0.06663	-0.07815	-0.07901	0.09259	-0.05257	-0.07188	0.07197	-0.07197	0.69261	0.38657	0.30961	0.30434	0.22842	0.49467	0.35018	0.34959	0.34959	turquoise
ZBTB40	-0.05751	-0.15007	-0.0915	-0.01925	-0.02375	-0.07459	-0.155	0.4475	-0.4475	0.45497	0.0501	0.23397	0.80264	0.75784	0.33224	0.04295	8.44E-10	8.44E-10	turquoise
TMEM107	-0.08665	-0.12861	-0.21443	-0.08108	0.04379	-0.24934	-0.08214	0.17248	-0.17248	0.25978	0.09366	0.00486	0.2918	0.56958	0.00101	0.28548	0.02408	0.02408	grey
FBXL3	-0.0752	-0.10874	-0.01743	0.04446	-0.12065	0.02855	-0.05946	0.32474	-0.32474	0.32831	0.15686	0.82096	0.56364	0.11598	0.71091	0.43979	1.47E-05	1.47E-05	turquoise
FITM2	0.00173	-0.10961	-0.23975	-0.02878	0.09149	-0.29314	-0.13642	0.42094	-0.42094	0.98213	0.15356	0.00159	0.70868	0.234	9.98E-05	0.07522	9.88E-09	9.88E-09	brown
SPTLC3	-0.00436	0.00653	-0.03823	0.07205	-0.06775	0.13508	0.24639	0.034	-0.034	0.95485	0.93245	0.61956	0.34904	0.37861	0.07814	0.00116	0.65892	0.65892	turquoise
SNN	-0.12944	-0.0973	-0.10715	-0.03572	-0.17252	-0.00966	-0.03452	0.34565	-0.34565	0.09154	0.2055	0.16305	0.64275	0.02405	0.90019	0.654	3.65E-06	3.65E-06	blue
WBP1L	-0.0447	-0.06265	0.05553	0.09969	-0.22877	0.15033	0.12013	0.29502	-0.29502	0.56154	0.41565	0.47069	0.19455	0.00262	0.0497	0.11756	8.96E-05	8.96E-05	blue
ARC	-0.04907	-0.1592	-0.11423	0.086	0.00763	-0.03245	-0.11861	0.3081	-0.3081	0.52388	0.03754	0.13685	0.26341	0.92114	0.67353	0.12231	4.14E-05	4.14E-05	grey
MAGI1	-0.00424	-0.07462	-0.08588	0.02476	0.03829	0.15369	-0.05667	0.05407	-0.05407	0.95611	0.33205	0.26403	0.74783	0.61905	0.04475	0.46162	0.48246	0.48246	turquoise
TEC	-0.06077	-0.11969	0.16398	0.1596	-0.13293	0.19246	0.18764	-0.08293	0.08293	0.42981	0.11894	0.0321	0.03705	0.08305	0.01167	0.01399	0.28086	0.28086	turquoise
ARPC5	-0.00361	-0.00967	0.00496	-0.02307	-0.06136	0.0753	0.16127	-0.14835	0.14835	0.96265	0.90007	0.94868	0.76457	0.42533	0.32765	0.0351	0.05281	0.05281	greenyellow
FHDC1	-0.04192	-0.12092	-0.06386	-0.18857	0.12664	-0.05164	-0.09334	-0.18434	0.18434	0.58617	0.11516	0.40662	0.01351	0.09885	0.50233	0.22462	0.0158	0.0158	grey
ASPA	-0.11316	-0.0993	-0.05224	0.05628	-0.02446	-0.00623	-0.12169	0.55476	-0.55476	0.14056	0.19627	0.49742	0.4647	0.75083	0.93552	0.11284	3.46E-15	3.46E-15	blue
LRATD2	-0.00325	-0.09806	-0.12609	-0.04894	0.03987	-0.01272	0.02616	-0.22748	0.22748	0.96635	0.20195	0.10031	0.52499	0.60461	0.86883	0.73409	0.00277	0.00277	turquoise
LINGO1	-0.0937	-0.0532	-0.2019	-0.01562	0.03886	-0.1601	-0.03845	0.2228	-0.2228	0.22284	0.48955	0.00809	0.83932	0.61382	0.03646	0.61758	0.0034	0.0034	grey
SFXN1	-0.07333	-0.22296	-0.09071	-0.02652	0.06636	-0.14196	0.02231	-0.04754	0.04754	0.34049	0.00338	0.23804	0.73065	0.38852	0.064	0.77211	0.53692	0.53692	turquoise
BCHE	0.01843	-0.10494	-0.20481	-0.06053	-0.11491	-0.03488	-0.07452	0.56848	-0.56848	0.81086	0.17194	0.00721	0.43159	0.1345	0.65058	0.33273	5.04E-16	5.04E-16	blue
ZFP3	-0.1431	-0.12994	-0.16857	-0.0881	0.10065	-0.17706	-0.19318	0.41823	-0.41823	0.06188	0.09029	0.02753	0.25187	0.19023	0.02052	0.01136	1.26E-08	1.26E-08	turquoise
GRAMD1B	-0.08449	0.06093	-0.20833	-0.08851	0.14463	-0.0789	-0.11261	0.11761	-0.11761	0.2719	0.42855	0.00625	0.24965	0.05912	0.30498	0.14254	0.12551	0.12551	grey
CIC	-0.03417	-0.1297	-0.1097	-0.02513	-0.02324	-0.00052	-0.0093	0.15468	-0.15468	0.6573	0.09088	0.15319	0.74423	0.76283	0.99464	0.90388	0.04337	0.04337	turquoise
CEP19	-0.12695	-0.19394	-0.12011	0.02683	-0.05748	-0.03263	-0.00172	0.41674	-0.41674	0.098	0.01103	0.11763	0.72756	0.45523	0.67177	0.9822	1.43E-08	1.43E-08	turquoise
CLDN7	0.16637	-0.00183	-0.01006	-0.05677	0.11187	0.05603	0.1294	-0.53417	0.53417	0.02965	0.98106	0.89615	0.46079	0.14519	0.46665	0.09163	5.31E-14	5.31E-14	yellow
ZNRF1	0.08795	-0.00984	0.07018	-0.02116	0.00914	0.00533	0.21618	-0.36179	0.36179	0.25268	0.8984	0.36168	0.78354	0.9056	0.94481	0.00451	1.16E-06	1.16E-06	grey
AC068580	0.00061	-0.04145	-0.0531	-0.08406	-0.01941	0.02273	0.1918	-0.32982	0.32982	0.99369	0.59034	0.49035	0.27434	0.80104	0.76795	0.01197	1.06E-05	1.06E-05	grey
LCP2	-0.13901	-0.06279	0.05768	0.0573	-0.25557	0.15403	0.03386	0.44632	-0.44632	0.0698	0.41456	0.45364	0.4566	0.00074	0.04428	0.66016	9.45E-10	9.45E-10	blue
LDLRAD4	-0.01339	-0.11982	-0.09493	0.0359	-0.13054	0.11127	-0.11661	0.43275	-0.43275	0.86203	0.11854	0.2168	0.64113	0.08879	0.14737	0.12878	3.40E-09	3.40E-09	blue
DBF4	-0.07874	-0.0517	0.09645	0.06329	-0.07106	0.12641	0.10996	-0.17649	0.17649	0.30597	0.50183	0.20953	0.41087	0.35567	0.09944	0.15222	0.02094	0.02094	pink
LONRF1	-0.05829	-0.15389	0.00656	0.06158	-0.0737	0.05173	-0.13346	0.34182	-0.34182	0.44885	0.04447	0.93211	0.42364	0.33807	0.50163	0.08182	4.75E-06	4.75E-06	turquoise
TADA3	0.01845	-0.07107	-0.07112	-0.1133	-0.07106	0.06079	0.11628	-0.15867	0.15867	0.81075	0.35563	0.35526	0.14008	0.35571	0.42963	0.12988	0.03819	0.03819	grey
ORAI2	-0.05067	-0.2014	-0.15199	-0.04494	-0.06427	-0.22207	-0.15415	0.39391	-0.39391	0.51044	0.00826	0.0472	0.55947	0.40366	0.00351	0.04412	9.80E-08	9.80E-08	turquoise
PHF7	-0.11895	-0.01547	-0.147	-0.11372	-0.00516	-0.02528	-0.04839	0.25508	-0.25508	0.12124	0.84089	0.05503	0.1386	0.94661	0.74274	0.52964	0.00076	0.00076	turquoise
HGD	0.03114	-0.03253	-0.17497	-0.05693	0.23587	-0.06683	-0.05024	-0.13307	0.13307	0.68598	0.67279	0.02208	0.45957	0.0019	0.3851	0.51402	0.08272	0.08272	grey
GNL3L	-0.05112	-0.11785	-0.02174	0.03367	-0.17471	0.10446	0.10964	0.21179	-0.21179	0.50665	0.12476	0.77777	0.66202	0.02229	0.17392	0.15341	0.00542	0.00542	turquoise
MPC2	0.0175	-0.11178	-0.22646	-0.19133	0.31252	-0.09665	-0.11245	-0.01188	0.01188	0.8203	0.1455	0.0029	0.01218	3.16E-05	0.20858	0.14309	0.87746	0.87746	grey
TLDC2	0.0961	-0.03771	-0.0664	-0.04938	0.29671	-0.02763	-0.02912	-0.14432	0.14432	0.21118	0.62434	0.38821	0.52129	8.13E-05	0.71984	0.70536	0.05967	0.05967	grey
MAP2K4	-0.12719	-0.25932	-0.05859	0.0147	-0.06244	-0.11251	-0.11958	0.41871	-0.41871	0.09735	0.00061	0.44651	0.84862	0.41718	0.1429	0.11929	1.20E-08	1.20E-08	turquoise
GATC	0.04547	-0.17304	-0.00445	0.0241	-0.036	-0.00956	0.01154	0.08849	-0.08849	0.55481	0.02362	0.95394	0.75436	0.64022	0.90125	0.88096	0.24974	0.24974	turquoise
LIG4	-0.06495	-0.18095	-0.08892	-0.04868	0.0433	-0.16163	-0.11405	0.30006	-0.30006	0.39865	0.01786	0.24747	0.5272	0.57392	0.03469	0.13747	6.68E-05	6.68E-05	turquoise
LTV1	-0.07517	0.03799	0.04819	0.01326	-0.05421	0.07151	0.03349	0.22944	-0.22944	0.32849	0.62183	0.53134	0.8633	0.48128	0.35267	0.6637	0.00254	0.00254	turquoise
PRDM8	-0.03236	-0.05745	0.08582	-0.03618	-0.0635	0.19185	0.17543	-0.1696	0.1696	0.67432	0.45548	0.26439	0.63846	0.40932	0.01195	0.02173	0.02658	0.02658	turquoise
ILRUN	0.02659	-0.08501	0.02985	0.03641	-0.12468	0.08053	0.03611	-0.05371	0.05371	0.72988	0.26894	0.69834	0.63638	0.10421	0.29507	0.63914	0.48533	0.48533	turquoise
ZNF337	0.00535	-0.16914	-0.0542	0.01697	0.08346	-0.04465	-0.0244	0.074	-0.074	0.94463	0.027	0.48135	0.82568	0.27782	0.56203	0.75141	0.33612	0.33612	red
NBEAL1	-0.024	-0.15024	-0.00403	0.05755	-0.1324	0.18553	0.14033	0.11387	-0.11387	0.75533	0.04984	0.95827	0.45468	0.08429	0.01512	0.06716	0.13807	0.13807	turquoise
ZNF738	0.01915	-0.0539	-0.04638	0.02058	-0.05449	-0.01493	-0.03731	0.13143	-0.13143	0.80364	0.48378	0.54695	0.78931	0.47901	0.84636	0.62802	0.08661	0.08661	turquoise
KCNMB1	-0.12777	0.05018	-0.00692	0.03382	-0.03934	0.09997	0.04965	0.21269	-0.21269	0.09583	0.51457	0.92845	0.66055	0.60945	0.1933	0.51896	0.00522	0.00522	salmon
BLNK	-0.03442	0.04061	0.13171	0.06492	0.05984	0.27526	0.06438	-0.12436	0.12436	0.65496	0.5979	0.08594	0.39887	0.43689	0.00027	0.40285	0.10511	0.10511	grey
EVC	-0.07881	-0.06441	0.00081	0.10552	-0.22947	0.18076	0.07814	0.42734	-0.42734	0.30556	0.40261	0.9916	0.16959	0.00254	0.01799	0.30968	5.57E-09	5.57E-09	blue
SEPTIN2	-0.03709	-0.0799	0.00859	0.08922	-0.14234	0.13182	0.01679	0.18136	-0.18136	0.63006	0.29887	0.91118	0.24586	0.06329	0.08569	0.82741	0.0176	0.0176	turquoise
APOC1	-0.05376	0.03137	0.11772	0.1376	-0.20693	0.15064	0.07074	0.09319	-0.09319	0.48499	0.68376	0.12516	0.07269	0.00662	0.04923	0.35787	0.2254	0.2254	blue
TMEM168	-0.04209	-0.10826	-0.02978	-0.03503	0.11156	0.06522	-0.10407	0.09906	-0.09906	0.58463	0.1587	0.69897	0.64926	0.14633	0.3967	0.17555	0.1974	0.1974	grey
YIF1B	0.00659	-0.08498	-0.06067	0.03431	-0.05097	-0.08418	0.12476	-0.00607	0.00607	0.9318	0.26914	0.43052	0.656	0.50791	0.27366	0.10398	0.93723	0.93723	grey
FAN1	-0.06545	-0.10579	-0.08175	-0.01953	-0.00786	-0.02506	-0.02789	0.22207	-0.22207	0.39505	0.16849	0.28783	0.79985	0.91871	0.74493	0.71728	0.00351	0.00351	turquoise
EFNB3	-0.00659	-0.16622	-0.23558	-0.15897	0.09832	-0.36846	-0.24811	0.44314	-0.44314	0.93185	0.02979	0.00192	0.03783	0.20076	7.10E-07	0.00107	1.28E-09	1.28E-09	brown
TM6SF1	-0.12572	-0.07453	0.0394	0.08046	-0.25698	0.13265	0.02263	0.53572	-0.53572	0.10133	0.33263	0.6089	0.29547	0.00069	0.0837	0.76888	4.35E-14	4.35E-14	blue
PRKCQ	-0.06666	-0.0426	-0.03793	0.05303	-0.1771	0.06497	0.0017	0.37542	-0.37542	0.38634	0.58011	0.62234	0.49091	0.02049	0.39852	0.98237	4.20E-07	4.20E-07	blue
SGPP2	0.0082	-0.05837	0.01789	0.08001	0.0314	0.119	0.02946	-0.11432	0.11432	0.91526	0.44824	0.81639	0.29822	0.68348	0.1211	0.70206	0.13654	0.13654	turquoise
AZIN2	-0.02669	-0.12112	-0.0771	0.03082	-0.04199	-0.13462	-0.09021	0.36708	-0.36708	0.72895	0.11454	0.31621	0.68904	0.58557	0.07917	0.24062	7.88E-07	7.88E-07	blue
LARGE2	-0.0599	0.08136	-0.01616	-0.06428	-0.04841	-0.04507	-0.08174	-0.06932	0.06932	0.43641	0.29011	0.83379	0.4036	0.52954	0.55832	0.28784	0.36766	0.36766	grey
SH3BP4	-0.10644	0.00665	0.07188	0.11661	-0.19679	0.3098	0.15606	-0.07107	0.07107	0.16586	0.93117	0.35016	0.1288	0.00989	3.73E-05	0.04152	0.35562	0.35562	turquoise
PIGP	-0.03015	-0.12012	-0.17303	-0.14649	0.2298	-0.23977	-0.19047	0.20269	-0.20269	0.6955	0.11759	0.02363	0.05589	0.0025	0.00159	0.01258	0.00784	0.00784	grey
TSEN34	0.0984	-0.07754	-0.08636	-0.052	-0.02214	-0.0478	0.08291	-0.26335	0.26335	0.2004	0.31343	0.2614	0.49941	0.77382	0.53472	0.281	0.0005	0.0005	grey
SP1	0.06309	-0.11664	0.04411	0.05344	-0.11768	0.19	0.11435	-0.11898	0.11898	0.41233	0.12869	0.56671	0.48757	0.12531	0.01281	0.13641	0.12114	0.12114	turquoise
EIF4E	-0.1277	-0.19412	-0.06868	0.00559	0.0056	-0.04071	-0.03592	0.11544	-0.11544	0.09603	0.01096	0.37209	0.94214	0.94204	0.597	0.64095	0.13271	0.13271	turquoise
DYNLRB1	0.05411	-0.07257	-0.09344	-0.07504	0.08071	-0.18315	0.01495	-0.28378	0.28378	0.48209	0.34553	0.22415	0.32936	0.294	0.0165	0.84613	0.00017	0.00017	grey
FRAT1	-0.1022	-0.02731	-0.10659	-0.04191	0.02154	-0.00898	0.01728	0.08356	-0.08356	0.18348	0.72293	0.16526	0.58622	0.77977	0.90717	0.82246	0.27724	0.27724	grey
CAPS	-0.00459	-0.13273	-0.05535	-0.05713	0.04351	-0.07049	0.04348	-0.41866	0.41866	0.95249	0.08353	0.47214	0.45795	0.57199	0.35956	0.57227	1.21E-08	1.21E-08	grey
ATP11C	-0.116	-0.08212	0.0333	0.0878	-0.24579	0.17364	0.15137	0.29445	-0.29445	0.13083	0.28564	0.6655	0.25349	0.00119	0.02313	0.04812	9.26E-05	9.26E-05	blue
CBX7	-0.04474	-0.05072	-0.20891	-0.09472	0.04564	-0.19368	-0.26854	0.45979	-0.45979	0.56123	0.51001	0.0061	0.21784	0.55335	0.01114	0.00038	2.51E-10	2.51E-10	brown
TMPPE	0.03878	-0.03325	0.06668	-0.00628	-0.14916	0.21981	0.18637	-0.06518	0.06518	0.61457	0.66596	0.38624	0.93499	0.05152	0.00387	0.01466	0.39698	0.39698	turquoise
KCTD12	-0.01616	-0.10122	-0.015	0.06647	-0.05744	-0.06941	-0.12524	0.53564	-0.53564	0.83388	0.18772	0.84566	0.38771	0.45553	0.36703	0.10264	4.40E-14	4.40E-14	turquoise
DIAPH3	-0.04588	0.0616	0.03366	-0.02585	-0.15753	0.1071	0.29868	-0.24957	0.24957	0.55126	0.42348	0.66203	0.73713	0.03961	0.16325	7.25E-05	0.001	0.001	pink
SLFN11	-0.12045	-0.07891	0.08424	0.0888	-0.13894	0.17606	0.04993	0.40338	-0.40338	0.11658	0.30493	0.27334	0.24808	0.06994	0.02126	0.51662	4.49E-08	4.49E-08	blue
SART3	0.03649	-0.14695	-0.02707	-0.00686	0.01263	-0.04081	-0.06939	0.11891	-0.11891	0.6356	0.05512	0.72523	0.92906	0.86978	0.59614	0.36716	0.12138	0.12138	turquoise
VPREB3	-0.06834	0.02957	0.01879	-0.02485	-0.05801	0.0156	-0.08835	0.26132	-0.26132	0.37445	0.70105	0.80733	0.74695	0.45102	0.83948	0.25053	0.00056	0.00056	blue
CYBA	-0.07289	-0.00066	0.0359	0.05094	-0.11515	0.16402	0.25377	-0.28064	0.28064	0.34341	0.99316	0.64113	0.50822	0.13368	0.03207	0.00081	0.0002	0.0002	yellow
CCDC84	-0.03296	-0.07658	-0.1008	-0.04754	0.15343	-0.11622	-0.01011	-0.20717	0.20717	0.66868	0.31949	0.18958	0.53694	0.04512	0.13009	0.89555	0.00655	0.00655	red
RUFY2	-0.04597	-0.17124	-0.03276	0.02766	-0.06223	-0.04623	0.00723	0.23736	-0.23736	0.55044	0.02513	0.67056	0.71949	0.41877	0.54823	0.92518	0.00177	0.00177	turquoise
IFRD1	-0.0054	-0.19083	0.08078	0.08837	0.04785	-0.01842	-0.15301	0.05934	-0.05934	0.94414	0.01242	0.29358	0.25039	0.53427	0.81105	0.04572	0.44076	0.44076	grey
PLK1	0.02161	0.10943	0.06109	0.01857	-0.00519	0.09982	0.31952	-0.53789	0.53789	0.77908	0.15421	0.42736	0.80952	0.94634	0.19394	2.05E-05	3.29E-14	3.29E-14	pink

MBOAT4	0.03483	-0.08872	-0.21353	0.02309	0.03741	-0.16132	-0.1138	0.39505	-0.39505	0.65106	0.24855	0.00504	0.76438	0.62716	0.03504	0.13832	8.93E-08	8.93E-08	magenta
AKR1C4	0.01603	0.00191	-0.11507	-0.11453	0.18414	-0.07746	-0.13387	-0.00278	0.00278	0.83514	0.98017	0.13395	0.1358	0.01591	0.31392	0.08088	0.97118	0.97118	grey
STAG2	-0.06076	-0.17013	-0.01897	0.02221	-0.11291	0.05943	-0.01803	0.1943	-0.1943	0.42984	0.02611	0.80547	0.77306	0.14145	0.44007	0.81496	0.01088	0.01088	turquoise
PDX1	0.0556	0.02261	-0.20289	-0.10986	0.17912	-0.01008	-0.09347	0.02277	-0.02277	0.47009	0.76907	0.00778	0.1526	0.01907	0.89593	0.22399	0.76752	0.76752	grey
CHMP4B	0.07785	-0.07957	0.02367	0.05007	0.07881	0.047	0.16844	-0.42982	0.42982	0.31147	0.30091	0.75861	0.5155	0.30555	0.54155	0.02765	4.44E-09	4.44E-09	green
IL20RB	0.12317	-0.03444	0.04067	0.1508	-0.00085	0.01015	0.2122	-0.27046	0.27046	0.10849	0.65472	0.59736	0.04899	0.99116	0.89521	0.00533	0.00035	0.00035	grey
SLC12A6	-0.05759	-0.178	0.02725	0.01224	-0.1551	0.10315	0.07048	0.12653	-0.12653	0.45432	0.01985	0.72351	0.87375	0.0428	0.17943	0.35966	0.09912	0.09912	turquoise
EIF4G2	0.00781	-0.1723	-0.02641	-0.00245	-0.06783	0.00366	-0.00191	0.16845	-0.16845	0.91929	0.02423	0.73174	0.97467	0.37803	0.96215	0.98018	0.02764	0.02764	turquoise
ISYNA1	-0.0036	-0.18674	-0.16731	0.01143	0.10823	-0.22798	-0.14507	0.08823	-0.08823	0.96269	0.01446	0.02872	0.88206	0.15881	0.00271	0.05834	0.25115	0.25115	grey
MBD1	-0.0623	-0.19203	-0.11436	-0.02354	0.09747	-0.12781	-0.0996	0.24597	-0.24597	0.41825	0.01186	0.13638	0.75992	0.20468	0.09573	0.19495	0.00118	0.00118	turquoise
SH3BP2	-0.02105	-0.09859	0.02994	0.03933	-0.038	0.08024	0.18114	-0.25088	0.25088	0.78465	0.19952	0.69743	0.60957	0.62169	0.29684	0.01774	0.00093	0.00093	turquoise
C20orf202	-0.00861	0.05564	-0.16896	-0.00609	0.2207	-0.10167	-0.06119	0.01954	-0.01954	0.91105	0.46978	0.02716	0.93704	0.00372	0.18575	0.42656	0.79975	0.79975	grey
HERPUD1	-0.03752	-0.03354	-0.05007	-0.00642	-0.04423	0.07525	-0.08421	0.38827	-0.38827	0.62613	0.66318	0.5155	0.93363	0.56565	0.32796	0.27349	1.54E-07	1.54E-07	grey
PELI3	-0.03719	-0.13671	-0.21243	-0.11895	0.05141	-0.35676	-0.15447	0.35803	-0.35803	0.62916	0.07458	0.00528	0.12123	0.50424	1.67E-06	0.04367	1.53E-06	1.53E-06	brown
EMP3	-0.06817	0.03824	0.09191	0.10489	-0.13441	0.17697	0.16031	-0.07796	0.07796	0.37563	0.61952	0.23183	0.17217	0.07966	0.02059	0.03622	0.3108	0.3108	black
EXOSC1	0.12138	0.00265	0.05603	-0.04736	-0.05804	-0.051	0.16259	-0.34323	0.34323	0.11377	0.97254	0.46666	0.53847	0.45081	0.50766	0.03361	4.31E-06	4.31E-06	greenyellow
HPDL	0.01259	0.21239	0.16894	0.03683	0.076	0.22162	0.29006	-0.43508	0.43508	0.87014	0.00529	0.02718	0.63247	0.3232	0.00358	0.00012	2.74E-09	2.74E-09	grey
CD180	-0.13517	-0.03758	0.07132	0.05057	-0.22242	0.13672	0.03116	0.39636	-0.39636	0.07795	0.62555	0.35392	0.51128	0.00346	0.07457	0.6858	8.03E-08	8.03E-08	blue
MIF4GD	-0.04262	-0.1621	-0.09748	-0.05679	0.01783	-0.09985	-0.06994	0.08391	-0.08391	0.57989	0.03416	0.20468	0.46062	0.81694	0.1938	0.36332	0.27522	0.27522	grey
RPAP2	-0.09917	-0.15128	-0.05198	0.01072	-0.09835	0.03749	0.03794	0.2524	-0.2524	0.19689	0.04825	0.49956	0.88937	0.20065	0.62637	0.62227	0.00087	0.00087	turquoise
RAB7A	0.00356	-0.07293	-0.07823	-0.0361	-0.09266	0.09567	0.11973	-0.02742	0.02742	0.96313	0.34319	0.30911	0.63924	0.22806	0.21325	0.11881	0.72184	0.72184	turquoise
GALNT2	-0.00503	-0.0999	0.03135	0.05695	-0.19467	0.03818	0.11774	0.06237	-0.06237	0.9479	0.19358	0.68399	0.4594	0.01073	0.62002	0.12511	0.41773	0.41773	turquoise
SIRT7	0.08722	-0.02069	0.08836	-0.04457	0.18604	0.07112	0.11034	-0.53214	0.53214	0.25668	0.78829	0.25048	0.5627	0.01484	0.35528	0.1508	6.88E-14	6.88E-14	grey
TSHZ3	-0.07764	-0.10114	-0.00295	0.05541	-0.20069	0.12018	0.05979	0.39175	-0.39175	0.31283	0.18808	0.96949	0.47166	0.00849	0.11742	0.43727	1.17E-07	1.17E-07	blue
SKIV2L	0.04527	0.00241	-0.03612	-0.03325	0.1063	-0.1012	0.10245	-0.11005	0.11005	0.55659	0.97503	0.63905	0.6659	0.16643	0.18784	0.18241	0.1519	0.1519	grey
HCAR1	0.10103	-0.05934	0.12342	-0.01271	-0.11674	0.12693	0.22517	-0.29239	0.29239	0.18856	0.44069	0.10777	0.86899	0.12838	0.09805	0.00307	0.0001	0.0001	grey
DTX4	0.05789	0.0246	0.00857	0.00288	-0.06365	0.09882	0.21274	-0.29239	0.29239	0.45198	0.74942	0.91138	0.97019	0.4082	0.19846	0.00521	0.0001	0.0001	yellow
MXD4	-0.1006	-0.06713	-0.24124	-0.0869	0.0606	-0.24487	-0.1242	0.29404	-0.29404	0.19048	0.38303	0.00148	0.25839	0.43109	0.00125	0.10555	9.48E-05	9.48E-05	brown
SNX2	-0.05905	-0.08066	-0.02667	-0.02146	-0.1358	0.02035	-0.02184	0.38709	-0.38709	0.44295	0.29429	0.72915	0.78055	0.07656	0.79164	0.7768	1.69E-07	1.69E-07	turquoise
CASP7	0.1153	-0.05692	0.10196	0.05451	-0.01379	0.12615	0.14385	-0.28016	0.28016	0.13317	0.45959	0.18453	0.47891	0.85797	0.10017	0.06051	0.00021	0.00021	turquoise
CIR1	-0.0407	-0.02172	-0.02146	0.00427	-0.06666	0.09473	0.0686	-0.04808	0.04808	0.5971	0.77799	0.78056	0.95579	0.38633	0.21776	0.37262	0.53234	0.53234	turquoise
OGG1	-0.03927	-0.10034	0.00343	-0.0788	-0.02747	0.13376	0.08503	0.04469	-0.04469	0.61005	0.19162	0.96452	0.30562	0.72138	0.08112	0.26882	0.56169	0.56169	turquoise
HP	0.08858	-0.00786	0.10817	-0.0325	-0.08234	0.13692	-0.0205	0.0697	-0.0697	0.24927	0.91875	0.15905	0.67305	0.28434	0.07414	0.79009	0.365	0.365	grey
FAM122A	-0.06122	-0.17619	-0.11414	-0.01735	0.01141	-0.09048	-0.24882	0.39133	-0.39133	0.42633	0.02116	0.13716	0.82179	0.88227	0.23924	0.00103	1.21E-07	1.21E-07	turquoise
XKR9	0.06472	-0.03668	0.1306	0.01103	0.05373	0.1984	0.11543	-0.35859	0.35859	0.40038	0.63383	0.08865	0.88614	0.48519	0.00929	0.13273	1.46E-06	1.46E-06	grey
WDR46	0.01515	0.0477	-0.05488	-0.01301	0.02519	-0.03067	0.18978	-0.05581	0.05581	0.84407	0.53553	0.47591	0.86592	0.74365	0.69051	0.01291	0.46843	0.46843	greenyellow
LRRC31	0.00173	0.03721	0.13259	0.03912	0.09919	0.20861	0.08042	-0.27645	0.27645	0.98206	0.62898	0.08384	0.61146	0.1968	0.00618	0.29571	0.00025	0.00025	grey
MRC2	-0.08392	-0.09109	0.08205	0.13123	-0.16895	0.14832	0.1169	0.19885	-0.19885	0.27518	0.23605	0.28605	0.0871	0.02717	0.05287	0.12784	0.00913	0.00913	black
PARP11	-0.01278	-0.03383	-0.07485	-0.00903	-0.06656	-0.08156	-0.15651	0.3687	-0.3687	0.86819	0.66042	0.33054	0.90665	0.38703	0.2889	0.04092	6.98E-07	6.98E-07	turquoise
PGS1	-0.01545	-0.18643	-0.01681	-0.04147	0.06699	-0.10398	0.00156	0.21113	-0.21113	0.84103	0.01463	0.82722	0.59019	0.38399	0.17592	0.98389	0.00557	0.00557	red
ARHGAP1	-0.03751	-0.09855	-0.0091	0.01812	-0.09257	0.05284	-0.01532	0.17815	-0.17815	0.62618	0.19973	0.90596	0.81405	0.22853	0.4925	0.84237	0.01974	0.01974	turquoise
C7orf61	0.08866	-0.2027	-0.02358	0.1095	0.03952	-0.01623	0.12681	-0.2339	0.2339	0.24883	0.00784	0.75948	0.15398	0.60779	0.83315	0.09838	0.00208	0.00208	grey
HLA-DRB1	-0.13643	0.08555	0.01179	0.05459	-0.209	0.1408	0.16538	0.20192	-0.20192	0.07519	0.26592	0.87834	0.4782	0.00608	0.06623	0.03065	0.00809	0.00809	blue
FAM78A	-0.12022	-0.00855	0.01201	0.04949	-0.17218	0.03211	-0.01279	0.47639	-0.47639	0.11728	0.91166	0.87616	0.52033	0.02433	0.67673	0.8681	4.52E-11	4.52E-11	blue
A1CF	0.07519	-0.12735	-0.21446	-0.11376	0.25957	-0.2562	-0.18703	0.23567	-0.23567	0.32835	0.09695	0.00485	0.13846	0.00061	0.00072	0.0143	0.00192	0.00192	brown
EMC8	0.0548	-0.01845	-0.05674	-0.05575	0.0064	-0.00196	0.14455	-0.34422	0.34422	0.4765	0.81075	0.46101	0.46891	0.93381	0.97972	0.05925	4.03E-06	4.03E-06	turquoise
SUSD5	-0.12104	-0.04657	-0.02346	0.06876	-0.1393	0.09617	-0.00384	0.45534	-0.45534	0.1148	0.54532	0.76074	0.37155	0.0692	0.21085	0.9603	3.91E-10	3.91E-10	blue
TPK1	-0.03934	-0.05371	0.08832	0.03614	-0.05604	-0.0389	-0.05096	0.15026	-0.15026	0.60943	0.48533	0.25066	0.6389	0.4666	0.61344	0.50802	0.04981	0.04981	grey
OPRL1	-0.12605	-0.11174	-0.01204	0.07155	-0.09939	-0.0036	-0.07258	0.35383	-0.35383	0.10043	0.14566	0.87575	0.35235	0.19589	0.96273	0.34548	2.06E-06	2.06E-06	blue
MRPS10	0.03067	0.0205	0.01161	0.02521	-0.12834	0.0488	0.2076	-0.14366	0.14366	0.69046	0.79017	0.88015	0.74343	0.09435	0.5262	0.00644	0.06085	0.06085	pink
FKBP5	-0.03982	-0.10408	-0.06517	0.0031	-0.18408	-0.07366	-0.05929	0.29571	-0.29571	0.60505	0.17551	0.3971	0.96789	0.01594	0.33833	0.44113	8.61E-05	8.61E-05	blue
VIP	-0.01353	-0.11219	-0.04455	0.07237	-0.03504	0.02852	-0.0375	0.31992	-0.31992	0.86061	0.14404	0.56289	0.34687	0.64913	0.7112	0.6263	1.99E-05	1.99E-05	grey
L1CAM	-0.08564	-0.08945	0.02242	0.04798	-0.02043	-0.04673	0.01132	0.12659	-0.12659	0.2654	0.24463	0.771	0.53316	0.7908	0.54394	0.88317	0.09897	0.09897	grey
UBE3C	-0.06274	-0.09637	0.03285	0.03811	-0.12453	0.00806	0.05816	0.07909	-0.07909	0.41498	0.2099	0.66973	0.62066	0.10463	0.91667	0.44991	0.30384	0.30384	turquoise
C5orf63	0.15288	-0.00114	-0.21523	-0.1948	0.16502	-0.12079	-0.05252	-0.13768	0.13768	0.0459	0.98819	0.0047	0.01067	0.03101	0.11555	0.49508	0.07253	0.07253	grey
SYTL3	-0.08818	-0.13015	0.03055	0.07436	-0.24218	0.08531	0.08306	0.2016	-0.2016	0.25141	0.08976	0.6916	0.33378	0.00142	0.26727	0.28009	0.00819	0.00819	blue
TYW1B	-0.04316	-0.09868	-0.00266	0.02567	-0.04531	-0.01967	-0.15019	0.22686	-0.22686	0.57514	0.19911	0.97245	0.73897	0.55618	0.79847	0.04991	0.00285	0.00285	grey
GEN1	-0.10094	0.04501	-0.01782	-0.09232	0.00285	-0.07821	0.15161	-0.11249	0.11249	0.18898	0.5588	0.81706	0.22976	0.97052	0.30924	0.04776	0.14296	0.14296	grey
RSPRY1	-0.05404	-0.1819	-0.04335	-0.02496	-0.07864	0.11115	0.03596	0.04517	-0.04517	0.48266	0.01726	0.57345	0.74593	0.30658	0.14781	0.64053	0.55748	0.55748	turquoise
SNX25	-0.0573	-0.24429	-0.11588	-0.05948	-0.00555	-0.1047	-0.20752	0.33247	-0.33247	0.45664	0.00128	0.13121	0.43962	0.9426	0.17293	0.00646	8.88E-06	8.88E-06	turquoise
LRSAM1	-0.00527	-0.07573	-0.11326	-0.01457	0.07781	-0.09114	-0.02867	-0.05448	0.05448	0.94549	0.32491	0.14021	0.84997	0.31173	0.23579	0.70976	0.47915	0.47915	green
GALK1	0.04657	0.03119	0.0356	0.04966	0.04671	0.15259	0.21715	-0.27404	0.27404	0.5453	0.68552	0.64385	0.51888	0.54409	0.04633	0.00433	0.00029	0.00029	grey
SNRPD1	-0.03124	-0.0197	0.01257	-0.06227	-0.05785	0.07257	0.1827	-0.32941	0.32941	0.68499	0.79811	0.87038	0.41845	0.4523	0.34558	0.01677	1.09E-05	1.09E-05	grey
FCSK	0.08729	0.02486	-0.05726	-0.0593	0.29916	0.00823	0.03476	-0.24061	0.24061	0.25628	0.7469	0.45697	0.44104	7.05E-05	0.91489	0.65175	0.00152	0.00152	red
MPND	0.09124	0.13582	-0.02579	-0.07815	0.10975	-0.04016	0.04528	-0.30368	0.30368	0.23528	0.07652	0.73773	0.30965	0.153	0.60198	0.55645	5.40E-05	5.40E-05	green
KRT14	0.07878	0.08707	0.07487	0.00989	-0.06063	0.00488	0.1924	-0.1797	0.1797	0.30575	0.25746	0.33045	0.89786	0.43085	0.94951	0.0117	0.01868	0.01868	grey
CASP3	-0.11748	-0.08957	0.01057	0.02873	0.03621	0.1324	0.09472	-0.06727	0.06727	0.12595	0.24399	0.89084	0.7091	0.63822	0.0843	0.21781	0.38202	0.38202	turquoise
COMT	-0.00393	-0.00482	-0.01952	0.05855	0.04066	-0.04444	0.05787	-0.30114	0.30114	0.95935	0.95016	0.79997	0.44681	0.59745	0.56388	0.45214	6.27E-05	6.27E-05	grey
AMDHD2	-0.00216	0.00873	-0.07654	-0.04144	0.05585	-0.0323	0.1525	-0.08193	0.08193	0.97759	0.90977	0.31973	0.59048	0.46815	0.67495	0.04645	0.28674	0.28674	green
SF1	-0.01842	-0.16464	-0.10357	-0.04524	0.00248	-0.01004	-0.02564	0.14324	-0.14324	0.81104	0.03141	0.17765	0.55684	0.97431	0.89629	0.7392	0.06162	0.06162	turquoise
TTI2	-0.02446	-0.08994	-0.0546	-0.05103	0.03056	0.0113	0.03642	0.09213	-0.09213	0.75087	0.24206	0.47814	0.50744	0.69156	0.88338	0.63626	0.23074	0.23074	turquoise
HAUS7	-0.00075	0.01323	-0.08388	-0.02416	0.06095	-0.05574	0.13938	-0.11351	0.11351	0.99227	0.86368	0.27539	0.75378	0.42843	0.46902	0.06904	0.13933	0.13933	grey
C11orf96	-0.04268	-0.11431	0.00533	0.02166	-0.01549	0.05851	-0.03957	0.27079	-0.27079	0.57942	0.13658	0.94486	0.77858	0.84068	0.44718	0.60736	0.00034	0.00034	blue
EPHB1	-0.12873	-0.01801	-0.07854	-0.13376	-0.05324	-0.0039	-0.0931	0.4024	-0.4024	0.09335	0.81514	0.3072	0.08114	0.48922	0.95963	0.22582	4.87E-08	4.87E-08	grey
PAPSS1	-0.1125	-0.09736	0.06804	0.00224	-0.11681	0.09806	0.00413	0.15574	-0.15574	0.14291	0.2052	0.37659	0.97678	0.12815	0.20196	0.95729	0.04194	0.04194	turquoise
NUS1	-0.09879	-0.08263	0.02829	0.02558	-0.11462	0.02485	0.01378	0.26781	-0.26781	0.19863	0.28261	0.71336	0.73984	0.13548	0.74697	0.858	0.0004	0.0004	turquoise
ERAP1	-0.00912	-0.113	0.08326	0.05957	-0.06238	0.09434	0.02475	0.11883	-0.11883	0.90577	0.14115	0.27896	0.43898	0.41764	0.21967	0.74801	0.12161	0.12161	turquoise
CCDC186	-0.00603	-0.18264	-0.00963	0.02642	-0.03986	-0.01027	-0.0344	0.10033	-0.10033	0.93758	0.0168	0.90049	0.73163	0.60468	0.89394	0.65508	0.19167	0.19167	turquoise
TMEM88	-0.14701	-0.06265	-0.1274	0.00843	0.15088	-0.11144	-0.24162	0.48138	-0.48138	0.05501	0.41565	0.0968	0.91284	0.04886	0.14674	0.00145	2.65E-11	2.65E-11	blue
ARHGEF39	0.0161	-0.00244	0.02388	-0.05079	0.14569	0.01841	0.12764	-0.41355	0.41355	0.83448	0.97472	0.75653	0.50947	0.05726	0.81107	0.09617	1.89E-08	1.89E-08	grey
NOTUM	-0.03858	0.03278	0.06134	0.09257	-0.00291	0.12511	0.11834	-0.04807	0.04807	0.61639	0.67038	0.42544	0.22848	0.96989	0.103	0.12319	0.5324	0.5324	grey
MAFA	-0.00873	0.01715	-0.16164	0.01439	-0.00461	-0.0355	0.0914	0.20294	-0.20294	0.90975	0.82386	0.03468	0.85181	0.95231	0.64481	0.23448	0.00776	0.00776	brown
RHOBTB3	0.0432	-0.2005	-0.03685	0.0872	-0.10853	-0.08996	-0.06795	0.40765	-0.40765	0.57482	0.00855	0.63233	0.25677	0.15765	0.24193	0.3772	3.13E-08	3.13E-08	turquoise
FRZB	0.04207	-0.01283	-0.02378	-0.00322	-0.02522	0.12998	-0.12962	0.41128	-0.41128	0.58482	0.86771	0.75758	0.9667	0.74333	0.09018	0.09109	2.30E-08	2.30E-08	blue
MYO1E	0.00336	-0.07675	0.05913	0.07062	-0.16304	0.2267	0.15995	-0.07205	0.07205	0.96526	0.31843	0.44239	0.35873	0.03312	0.00287	0.03664	0.34901	0.34901	turquoise
STAG1	-0.0976	-0.10308	-0.00764	0.05369	-0.19024	0.11155	0.03675	0.26797	-0.26797	0.20408	0.17972	0.921	0.48556	0.01269	0.14635	0.63322	0.00039	0.00039	turquoise
PRR5L	0.10748	-0.0024	0.08522	0.12015	-0.09988	0.17314	0.21008	-0.12024	0.12024	0.16175	0.97514	0.26777	0.11752	0.19369	0.02354	0.00582	0.11724	0.11724	grey
THOC7	0.0256	0.04171	-0.0177	-0.09942	-0.06152	0.09863	0.10169	-0.17758	0.17758	0.73966	0.58808	0.81825	0.19576	0.4241	0.19932	0.18569	0.02014	0.02014	turquoise
AEBP2	-0.05372	-0.16902	-0.02922	0.01378	-0.01474	0.01452	0.00165	0.06046	-0.06046	0.48529	0.02711	0.70443	0.85803	0.84827	0.85053	0.98294	0.43213	0.43213	turquoise
SLC25A4	-0.0315	-0.07545	-0.22586	-0.1255	0.19358	-0.24536	-0.25769	0.21062	-0.21062	0.68257	0.32667	0.00297	0.10192	0.01119	0.00122	0.00067	0.00569	0.00569	brown
MRFAP1	-0.07677	-0.12668	-0.15314	-0.05174	0.01433	-0.12433	-0.04266	0.11268	-0.11268	0.31829	0.09873	0.04554	0.50153	0.85248	0.10519	0.57955	0.1423	0.1423	turquoise
SLC2A3	-0.09858	-0.15537	0.09983	0.13467	-0.13009	0.14027	0.06711	0.17831	-0.17831	0.19959	0.04244	0.19392	0.07906	0.08991	0.06727	0.38312	0.01963	0.01963	black
ZNF471	-0.07081	-0.25038	-0.17693	0.01712	-0.0203	-0.06211	-0.12932	0.50928	-0.50928	0.35741	0.00096	0.02061	0.82414	0.79215	0.41968	0.09185	1.13E-12	1.13E-12	brown
TRIOBP	-0.00571	0.03761	0.00864	0.00823	-0.01067	0.15685	0.09837	-0.28669	0.28669	0.94088	0.62526	0.91065	0.91491	0.88981	0.04048	0.20052	0.00014	0.00014	yellow
EPS8L3	0.00264	0.07076	0.10852	0.06605	0.13002	0.27051	0.07679	-0.34555	0.34555	0.97265	0.35772	0.15768	0.39074	0.09008	0.00035	0.31818	3.68E-06	3.68E-06	yellow
PPP4R3A	0.00204	-0.11646	0.01991	0.10726	-0.0132	0.07411	-0.02543	0.05504	-0.05504	0.97888	0.12929	0.79601	0.16259	0.86393	0.33541	0.74132	0.47463	0.47463	turquoise
S100A13	0.05722	0.01869	0.00499	-0.0457	-0.07355	0.17753	0.26549	-0.38715	0.38715	0.45724	0.80826	0.94837	0.55279	0.33907	0.02018	0.00045	1.69E-07	1.69E-07	yellow
NRSN2	-0.01847	-0.18826	-0.23876	-0.07115	-0.03027	-0.34041	-0.18334	0.45034	-0.45034	0.81055	0.01367	0.00166	0.35506	0.69432	5.22E-06	0.01638	6.41E-10	6.41E-10	brown
FBLL1	0.10452	-0.11584	-0.2285	-0.10979	0.18956	-0.3001	-0.17982	0.27674	-0.27674	0.17366	0.13135	0.00265	0.15285	0.01302	6.67E-05	0.0186	0.00025	0.00025	brown
TAF6L	-0.04805	-0.02082	-0.14152	-0.10975	0.14018	-0.20018	-0.03037	0.13224	-0.13224	0.5326	0.78691	0.06484	0.15302	0.06744	0.00866	0.69334	0.08467	0.08467	grey
ALG8	0.02094	-0.10864	-0.00543	-0.01255	-0.06114	-0.03323	0.17024	-0.23012	0.23012	0.7857	0.15722	0.94381	0.87059	0.42694	0.66608	0.02601	0.00246	0.00246	turquoise
SYPL2	-0.04439	-0.12251	0.02371	0.0911	-0.20797	0.12004	-0.001	0.46362	-0.46362	0.56431	0.11042	0.75825	0.23599	0.00634	0.11784	0.98964	1.70E-10	1.70E-10	blue
PIK3IP1	-0.01575	-0.00375	0.02586	0.0112	-0.12942	0.10413	-0.03347	0.11709	-0.11709	0.83804	0.96115	0.73709	0.88443	0.0916	0.17529	0.66389	0.1272	0.1272	turquoise
GLUL	-0.0361	-0.03801	0.13934	0.12472	-0.22368	0.22841	0.1378	0.10101	-0.10101	0.63925	0.62156	0.06912	0.10409	0.00327	0.00266	0.07229	0.18866	0.18866	turquoise
CGRRF1	-0.04293	-0.18073	-0.13474	-0.05805	0.05197	-0.229	-0.22153	0.28896	-0.28896	0.57714	0.018	0.07891	0.45074	0.49964	0.00259	0.00359	0.00013	0.00013	turquoise
PLXND1	-0.14975	-0.07172	-0.08727	0.02536	-0.11666	-0.0435	0.05769	0.33694	-0.33694	0.05059	0.35121	0.25637	0.74199	0.12862	0.57213	0.45357	6.60E-06	6.60E-06	blue
GOLGA8B	-0.07229	-0.00699	-0.04128	-0.02	0.12211	-0.14783	-0.06867	-0.0345	0.0345	0.34745	0.92771	0.59191	0.79516	0.11161	0.05365	0.37218	0.65419	0.65419	red
NEK9	-0.03522	-0.1422	-0.05758	0.04584	-0.03059	-0.02326	-0.12104	0.32488	-0.32488	0.64743	0.06354	0.45445	0.55164	0.69122	0.76267	0.11481	1.45E-05	1.45E-05	turquoise
EGR3	-0.08064	-0.14349	0.0577	0.02072	0.00399	0.08179	-0.05409	0.44019	-0.44019	0.29442	0.06117	0.4535	0.7879	0.95867	0.28755	0.48231	1.70E-09	1.70E-09	grey
DNAJC14	-0.05437	-0.20897	-0.09999	0.01657	-0.02003	-0.07178	-0.07547	0.20735	-0.20735	0.48004	0.00609	0.19317	0.8297	0.79486	0.35081	0.32657	0.0065	0.0065	turquoise
RNF144A	-0.01222	-0.11106	0.04603	0.11324	-0.27339	0.10192	0.04569	0.37849	-0.37849	0.87392	0.14813	0.54992	0.14031	0.0003	0.1847	0.55287	3.32E-07	3.32E-07	blue
PHLDA3	0.06334	0.00762	0.06399	0.08191	-0.09877	0.10468	0.21031	-0.20564	0.20564	0.41052	0.92117	0.40568	0.28688	0.19868	0.17301	0.00576	0.00697	0.00697	grey
ADAMTS9	-0.1164	-0.12761	0.00891	0.03119	-0.18516	0.17781	-0.0685	0.25848	-0.25848	0.1295	0.09626	0.90793	0.68553	0.01533	0.01999	0.37332	0.00064	0.00064	blue
CNTN3	-0.11184	-0.09111	-0.21797	-0.01859	0.05428	-0.16247	-0.0756	0.21007	-0.21007	0.14531	0.23595	0.00418	0.80933	0.48073	0.03374	0.32574	0.00582	0.00582	grey
ING3	-0.092	-0.091	-0.06413	-0.04464	-0.01026	-0.09368	-0.1123	0.4148	-0.4148	0.23138	0.23655	0.40463	0.56208	0.89401	0.22294	0.14363	1.69E-08	1.69E-08	turquoise
TESC	0.02814	0.1452	-0.03878	-0.05191	0.10641	0.12417	0.00268	-0.28106	0.28106	0.71488	0.05812	0.61458	0.50011	0.16599	0.10565	0.97226	0.0002	0.0002	grey
XPNPEP3	-0.02817	-0.03637	-0.15978	-0.0397	0.07358	-0.20185	0.01691	0.12644	-0.12644	0.71453	0.63678	0.03684	0.60614	0.33887	0.00811	0.82628	0.09938	0.09938	turquoise
ACTC1	-0.06545	-0.00164	-0.12022	0.05356	0.16211	-0.17064	-0.02547	0.27249	-0.27249	0.39504	0.98301	0.1173	0.48657	0.03415	0.02565	0.74088	0.00031	0.00031	grey
TUBE1	-0.00162	-0.08111	-0.03357	0.00116	0.14956	-0.09822	-0.10128	0.25453	-0.25453	0.98319	0.29159	0.66294	0.98795	0.05089	0.20125	0.1875	0.00078	0.00078	turquoise
RBM23	-0.00691	-0.15761	-0.13425	-0.04876	0.03441	-0.15249	-0.11181	0.26358	-0.26358	0.92853	0.03952	0.08001	0.52656	0.65499	0.04648	0.14539	0.0005	0.0005	turquoise
CAMSAP2	-0.0558	-0.17196	-0.07213	0.01937	-0.18583	0.04163	0.09816	0.20585	-0.20585	0.46856	0.02451	0.34849	0.80142	0.01496	0.58881	0.2015	0.00691	0.00691	turquoise
HSBP1L1	0.03	-0.03733	-0.03156	0.02293	0.14919	0.05474	0.16993	-0.44825	0.44825	0.69687	0.62787	0.68197	0.76599	0.05147	0.47704	0.02629	7.85E-10	7.85E-10	grey
PLAT	0.0012	-0.21178	0.05836	0.08317	-0.14538	0.15961	0.15587	-0.12258	0.12258	0.98752	0.00542	0.44834	0.2795	0.0578	0.03705	0.04177	0.11023	0.11023	turquoise
CFI	-0.02534	-0.06894	0.0198	0.10443	-0.11083	0.22198	0.10437	0.06119	-0.06119	0.74212	0.3703	0.79709	0.17404	0.14898	0.00352	0.17429	0.42662	0.42662	blue
ARL6	0.00317	-0.16361	-0.21064	-0.10887	-0.05518	-0.10621	-0.10729	0.32683	-0.32683	0.96718	0.0325	0.00569	0.15636	0.47352	0.16679	0.16249	1.28E-05	1.28E-05	turquoise
MFSD6	0.07555	-0.07465	-0.04559	-0.04366	0.05897	-0.00135	-0.04633	-0.07686	0.07686	0.32603	0.33186	0.55377	0.57074	0.44356	0.98603	0.54734	0.31769	0.31769	turquoise
FAM126B	-0.02318	-0.14636	0.02773	0.06092	-0.01832	0.08262	0.06924	-0.06852	0.06852	0.76346	0.05611	0.71887	0.42865	0.81199	0.28267	0.36821	0.3732	0.3732	turquoise
DNAJC10	-0.00084	-0.04556	-0.00045	0.04286	-0.00706	0.05632	-0.01174	0.03896	-0.03896	0.99127	0.55405	0.99534	0.57778	0.927	0.4644	0.87891	0.6129	0.6129	turquoise
PPP6C	-0.08917	-0.14489	-0.1055	-0.01833	-0.11393	-0.06431	-0.12649	0.28061	-0.28061	0.24612	0.05865	0.16966	0.81188	0.13789	0.40334	0.09923	0.0002	0.0002	turquoise

SULT1A1	-0.09101	-0.03702	-0.06973	-0.04618	0.0937	-0.02045	0.11677	0.00044	-0.00044	0.23645	0.63069	0.36482	0.54867	0.22287	0.79064	0.12827	0.99548	0.99548	grey
RPRD1B	0.05392	-0.14515	-0.03058	0.00462	-0.05616	-0.02787	0.02227	0.01431	-0.01431	0.48368	0.05819	0.69135	0.9522	0.46562	0.71743	0.77248	0.85268	0.85268	turquoise
N6AMT1	0.08579	-0.14132	-0.25128	-0.13507	0.11015	-0.16992	0.01087	0.16875	-0.16875	0.26457	0.06523	0.00092	0.07817	0.15151	0.02629	0.8878	0.02736	0.02736	turquoise
DCHS1	-0.10269	-0.12861	0.00122	0.04876	-0.13667	0.03253	-0.02127	0.46901	-0.46901	0.18136	0.09367	0.98739	0.52652	0.07468	0.67277	0.78243	9.79E-11	9.79E-11	blue
EGFL6	-0.1355	0.01267	0.08766	0.14978	-0.07334	-0.08466	0.08014	0.1445	-0.1445	0.07722	0.86933	0.25422	0.05055	0.34047	0.27091	0.2974	0.05934	0.05934	black
GPS1	0.00588	-0.07636	-0.09428	-0.08659	0.19529	-0.21889	0.00959	-0.11518	0.11518	0.9392	0.32085	0.21998	0.26012	0.01047	0.00402	0.90093	0.1336	0.1336	grey
OPTN	0.08853	-0.05464	0.01885	-0.01666	-0.09428	0.07634	0.09607	-0.11702	0.11702	0.24954	0.47784	0.80672	0.82878	0.21997	0.32102	0.2113	0.12745	0.12745	turquoise
C3orf38	-0.09135	-0.10589	0.03098	0.02771	-0.1983	0.16347	0.1256	0.06302	-0.06302	0.23474	0.16807	0.68748	0.71898	0.00932	0.03265	0.10165	0.41289	0.41289	turquoise
S100A4	0.0243	-0.04171	0.08401	0.02022	-0.1536	0.13102	0.21629	-0.23226	0.23226	0.7524	0.58806	0.27465	0.79289	0.04488	0.08762	0.00449	0.00224	0.00224	grey
SPTSSB	-0.10777	-0.28242	-0.05997	-0.03743	0.07472	-0.07503	0.00487	-0.10458	0.10458	0.16062	0.00018	0.43592	0.62694	0.33142	0.32941	0.94963	0.17345	0.17345	grey
INKA2	-0.15783	-0.08506	-0.15508	0.00303	-0.00175	-0.21248	-0.17578	0.53963	-0.53963	0.03923	0.26865	0.04283	0.96862	0.9819	0.00527	0.02147	2.62E-14	2.62E-14	brown
ZNF346	-0.00671	-0.21525	-0.19757	-0.06107	-0.03402	-0.17236	-0.14491	0.30314	-0.30314	0.93061	0.00469	0.00959	0.42748	0.65868	0.02418	0.05863	5.57E-05	5.57E-05	turquoise
HYAL3	-0.004	-0.09423	-0.11819	-0.05056	0.0428	-0.02411	0.05281	-0.22726	0.22726	0.95863	0.22024	0.12365	0.51131	0.57833	0.75428	0.49271	0.0028	0.0028	grey
STX16-NF	-0.03683	0.02016	0.00163	0.01487	0.06012	-0.0493	-0.01871	0.13447	-0.13447	0.6325	0.79351	0.98308	0.84689	0.43471	0.52191	0.80811	0.07952	0.07952	red
RBBP6	-0.08083	-0.12224	-0.05551	0.03811	-0.08031	0.06562	-0.00369	0.18492	-0.18492	0.29329	0.11122	0.47081	0.62073	0.29641	0.39383	0.96178	0.01547	0.01547	turquoise
AIFM3	0.04234	-0.00408	-0.01912	0.03243	0.32731	-0.13699	-0.01361	-0.16537	0.16537	0.58243	0.95779	0.80397	0.67372	1.24E-05	0.07399	0.85981	0.03066	0.03066	grey
PSME4	-0.05039	-0.09259	0.01917	0.01783	-0.1373	0.16331	0.17755	-0.04655	0.04655	0.51276	0.22843	0.8035	0.81696	0.07334	0.03282	0.02017	0.54546	0.54546	turquoise
HSPA2	0.01678	-0.07876	0.01134	0.03082	0.0722	0.07346	-0.042	0.10092	-0.10092	0.82761	0.30584	0.88292	0.68905	0.34799	0.33963	0.58545	0.18907	0.18907	grey
SBK1	-0.03383	-0.0162	-0.09683	0.05716	-0.01721	-0.00805	-0.03941	0.07914	-0.07914	0.66052	0.83346	0.2077	0.45769	0.82322	0.9168	0.60883	0.3035	0.3035	grey
PDLIM1	0.00276	-0.05822	0.03545	0.04016	-0.07715	0.19625	0.16124	-0.21173	0.21173	0.9714	0.4494	0.64527	0.60196	0.31585	0.0101	0.03513	0.00544	0.00544	yellow
OTUD6B	0.02305	-0.07115	-0.00046	0.01791	-0.08661	-0.00168	0.11682	-0.001	0.001	0.76479	0.35512	0.9952	0.81613	0.26001	0.98261	0.12811	0.98966	0.98966	turquoise
MTPN	-0.01597	-0.09667	0.05101	0.04233	-0.12824	0.0594	0.03797	0.08593	-0.08593	0.83573	0.20848	0.50764	0.58254	0.09461	0.44026	0.62201	0.26376	0.26376	turquoise
CPPED1	-0.06763	-0.01927	-0.0275	-0.01105	-0.11945	0.02934	-0.04924	0.24663	-0.24663	0.37944	0.80245	0.72101	0.88598	0.11967	0.70329	0.52246	0.00115	0.00115	blue
PPP1R3F	0.00243	-0.21958	-0.19174	-0.06673	0.07997	-0.31957	-0.21961	0.28609	-0.28609	0.97487	0.00391	0.012	0.38582	0.29845	2.04E-05	0.0039	0.00015	0.00015	brown
ZNF396	-0.08283	-0.09455	-0.21318	-0.05516	0.15213	-0.20855	-0.12664	0.2179	-0.2179	0.28145	0.21867	0.00512	0.47362	0.04699	0.00619	0.09883	0.0042	0.0042	brown
BAAT	0.02981	-0.0319	-0.0986	0.0234	0.05121	0.07071	0.05007	0.07426	-0.07426	0.69869	0.67869	0.19946	0.76129	0.5059	0.35809	0.51547	0.33441	0.33441	grey
UBE2F	0.09577	-0.09248	-0.06847	0.02737	-0.01211	-0.11017	0.01472	0.10134	-0.10134	0.21276	0.22897	0.37356	0.72237	0.87507	0.15146	0.84843	0.18722	0.18722	grey
FKBP14	-0.06913	-0.10258	0.07314	0.09873	-0.17084	0.15118	0.08276	0.30998	-0.30998	0.36899	0.18184	0.34179	0.1989	0.02547	0.04841	0.28188	3.69E-05	3.69E-05	black
ZNF195	0.02852	-0.12672	-0.05985	-0.08715	0.10165	0.00093	-0.00296	-0.15733	0.15733	0.71115	0.09861	0.43682	0.25705	0.18587	0.99032	0.96932	0.03987	0.03987	red
BICDL1	0.0214	-0.14109	-0.0854	-0.11425	0.25417	-0.2025	-0.23924	-0.08219	0.08219	0.78112	0.06567	0.26673	0.13677	0.00079	0.0079	0.00162	0.28521	0.28521	grey
B4GALT5	0.0098	-0.13828	0.0838	0.08472	-0.15375	0.1536	0.17799	-0.14185	0.14185	0.89881	0.07128	0.27586	0.27059	0.04467	0.04488	0.01986	0.06422	0.06422	turquoise
NPR2	-0.07079	-0.1965	0.03094	0.03627	-0.12543	0.12431	0.02013	0.28858	-0.28858	0.35752	0.01	0.68786	0.63771	0.10213	0.10523	0.79384	0.00013	0.00013	blue
FAM104B	-0.05897	-0.13785	-0.18417	-0.03464	0.1296	-0.25907	-0.1235	0.28548	-0.28548	0.44361	0.07218	0.0159	0.65285	0.09115	0.00062	0.10755	0.00015	0.00015	brown
DYNLT3	-0.0374	-0.19349	0.00147	0.01812	-0.058	0.00251	-0.0022	-0.00877	0.00877	0.62721	0.01122	0.98481	0.81403	0.45115	0.97398	0.97726	0.90934	0.90934	turquoise
ADPGK	-0.05084	-0.09071	0.02503	0.00811	-0.11027	0.11551	0.10187	-0.00509	0.00509	0.509	0.23805	0.74526	0.91619	0.15106	0.13248	0.18491	0.94734	0.94734	turquoise
TPPP	-0.04227	-0.04431	-0.1539	-0.05596	0.18738	-0.16162	-0.16018	0.29311	-0.29311	0.58303	0.56497	0.04446	0.46725	0.01412	0.0347	0.03636	1.00E-04	1.00E-04	brown
INSYN1	-0.01311	-0.11309	-0.2168	-0.09947	0.02373	-0.26575	-0.19853	0.5475	-0.5475	0.86483	0.14084	0.0044	0.19553	0.75807	0.00044	0.00924	9.26E-15	9.26E-15	brown
UBASH3A	-0.09676	0.00532	0.02688	0.02774	-0.08114	0.10369	-0.06453	0.34446	-0.34446	0.20805	0.94498	0.72711	0.71878	0.29142	0.17715	0.40174	3.96E-06	3.96E-06	blue
SLC35C1	0.04411	0.01193	0.01321	-0.01207	0.00252	0.13937	0.15694	-0.27302	0.27302	0.56675	0.87688	0.86386	0.87553	0.97392	0.06907	0.04037	0.0003	0.0003	turquoise
MXRA7	-0.03415	-0.12584	-0.07559	-0.00592	-0.12726	-0.13148	-0.12511	0.51886	-0.51886	0.65742	0.101	0.32579	0.93872	0.09717	0.0865	0.10301	3.60E-13	3.60E-13	turquoise
NDUFA6	-0.07534	0.07549	-0.12734	-0.03094	0.01964	-0.14008	-0.00183	-0.08358	0.08358	0.3274	0.32644	0.09696	0.68785	0.7987	0.06765	0.9811	0.27712	0.27712	grey
FBXO36	-0.09393	-0.14532	-0.08382	-0.00456	-0.08882	-0.02548	-0.09437	0.14877	-0.14877	0.2217	0.05791	0.27575	0.95282	0.24802	0.74079	0.21956	0.05214	0.05214	turquoise
CD47	-0.05252	-0.07882	-0.10156	-0.03396	-0.19027	0.12051	0.1403	-0.11858	0.11858	0.49511	0.30547	0.18625	0.65923	0.01268	0.11641	0.06721	0.12241	0.12241	turquoise
MATN2	-0.05556	-0.13226	-0.04686	0.06309	0.09252	0.10034	-0.0936	0.22233	-0.22233	0.4704	0.08462	0.54277	0.41236	0.22877	0.19165	0.22334	0.00347	0.00347	grey
DLX5	0.08527	-0.06674	0.14611	0.10138	-0.12722	0.08236	0.09749	-0.03016	0.03016	0.26748	0.38578	0.05655	0.18704	0.0973	0.28418	0.20461	0.69538	0.69538	grey
ZNF606	-0.04791	-0.13634	-0.11908	0.00099	-0.02464	-0.05243	-0.09787	0.2428	-0.2428	0.53379	0.07538	0.12085	0.98978	0.74901	0.49583	0.20283	0.00138	0.00138	turquoise
NDUFA12	0.07103	-0.00555	0.01719	-0.03383	0.08951	-0.06049	0.01998	-0.24601	0.24601	0.35593	0.94256	0.82337	0.66047	0.2443	0.43191	0.7953	0.00118	0.00118	turquoise
CUL7	-0.00729	-0.09971	-0.09155	-0.0812	0.06517	-0.11871	0.02818	0.1054	-0.1054	0.92462	0.19443	0.23371	0.29107	0.39704	0.122	0.71447	0.17005	0.17005	turquoise
IL21R	-0.11532	0.00923	0.09366	0.02477	-0.22297	0.15701	0.07734	0.38	-0.38	0.13312	0.90465	0.22303	0.74776	0.00337	0.04028	0.31467	2.95E-07	2.95E-07	blue
DNAJC19	-0.03577	-0.02614	-0.18674	-0.06669	0.1189	-0.11215	0.03156	-0.15073	0.15073	0.64228	0.73434	0.01446	0.38613	0.12141	0.14417	0.68192	0.04909	0.04909	grey
SPOUT1	0.05281	-0.04471	-0.1174	-0.08878	0.02018	-0.11405	0.02805	-0.06595	0.06595	0.49273	0.56148	0.12619	0.2482	0.79333	0.13745	0.71573	0.39142	0.39142	grey
PLIN5	0.00787	0.0091	-0.03132	0.12329	-0.06076	0.04368	0.04564	-0.06459	0.06459	0.91863	0.90596	0.68431	0.10816	0.42984	0.57055	0.55334	0.4013	0.4013	magenta
PITPNM2	-0.02739	-0.24976	-0.11841	0.04359	0.01799	-0.20008	-0.09548	0.27808	-0.27808	0.72212	0.00099	0.12295	0.57137	0.81531	0.0087	0.21412	0.00023	0.00023	grey
RABL6	0.04031	-0.03335	-0.16393	-0.14524	0.08336	-0.20239	0.04701	-0.19681	0.19681	0.60067	0.66496	0.03216	0.05805	0.27836	0.00794	0.54147	0.00988	0.00988	green
WBP4	-0.05628	-0.16598	-0.09153	-0.06791	-0.0368	-0.12546	-0.13525	0.31416	-0.31416	0.46473	0.03004	0.2338	0.37746	0.63273	0.10204	0.07778	2.86E-05	2.86E-05	turquoise
TXNRD2	-0.02855	0.0485	-0.15275	-0.09673	0.18781	-0.19985	-0.02733	-0.1319	0.1319	0.71092	0.52871	0.0461	0.20818	0.0139	0.00877	0.7227	0.08548	0.08548	green
ITPRID2	-0.08649	-0.05087	0.09382	0.1201	-0.08024	0.24725	0.11681	-0.06659	0.06659	0.26064	0.50874	0.22226	0.11765	0.29684	0.00111	0.12813	0.38681	0.38681	turquoise
ANXA7	-0.00625	-0.11604	-0.01167	-0.02423	-0.07	-0.09422	-0.07025	0.07961	-0.07961	0.93534	0.13068	0.87957	0.75306	0.36295	0.2203	0.36124	0.30064	0.30064	turquoise
MAB21L4	0.01457	-0.02854	-0.02443	0.05217	0.18245	-0.10486	-0.07149	-0.14864	0.14864	0.84997	0.71096	0.75107	0.498	0.01692	0.17228	0.35279	0.05235	0.05235	grey
ANKRD27	0.07519	-0.10161	-0.01966	0.04656	-0.12946	0.05454	0.15955	-0.09503	0.09503	0.32839	0.18603	0.79857	0.54541	0.09148	0.47865	0.03712	0.21634	0.21634	turquoise
VPS26C	-0.05465	-0.10865	-0.00117	0.031	-0.02754	0.12667	0.11156	0.11454	-0.11454	0.47772	0.15719	0.98792	0.6873	0.72066	0.09876	0.14631	0.13578	0.13578	turquoise
AAMP	0.11268	-0.0197	-0.01783	-0.01487	0.05489	0.01678	0.11147	-0.33765	0.33765	0.14226	0.79819	0.81696	0.84694	0.47581	0.82752	0.14664	6.29E-06	6.29E-06	turquoise
TRPC4AP	-0.00193	-0.10382	0.00705	-0.03989	-0.00967	-0.03098	0.04506	-0.10096	0.10096	0.98003	0.17661	0.92704	0.60446	0.90013	0.68752	0.5584	0.1889	0.1889	turquoise
IL4R	-0.03595	-0.00677	0.11381	0.09126	-0.15716	0.27404	0.15485	-0.14831	0.14831	0.6406	0.92997	0.13831	0.23519	0.04009	0.00029	0.04315	0.05287	0.05287	turquoise
TMEM123	-0.01848	-0.07796	0.11514	0.12007	-0.17121	0.26385	0.18624	-0.21435	0.21435	0.81045	0.31078	0.1337	0.11775	0.02515	0.00049	0.01473	0.00488	0.00488	turquoise
RCBTB1	-0.0246	-0.13878	-0.16795	-0.06991	0.02333	-0.14268	-0.15994	0.38802	-0.38802	0.74944	0.07025	0.02811	0.36355	0.76201	0.06265	0.03665	1.57E-07	1.57E-07	turquoise
BIRC7	0.05496	0.04231	0.09941	-0.00064	-0.06301	0.13132	0.12758	-0.28455	0.28455	0.47523	0.58267	0.1958	0.99333	0.41297	0.08689	0.09634	0.00016	0.00016	grey
HIST1H2A	0.01317	-0.11808	0.05371	-0.03424	-0.01338	0.05304	0.09954	-0.12941	0.12941	0.86424	0.124	0.48533	0.6566	0.86212	0.49084	0.19521	0.09162	0.09162	grey
COL18A1	-0.05338	-0.06345	-0.06634	0.07353	-0.08764	0.06725	0.06379	0.11354	-0.11354	0.48807	0.40969	0.38863	0.33922	0.25436	0.38212	0.4072	0.13925	0.13925	black
GABPB2	0.0518	-0.07335	-0.06734	-0.08916	0.04091	0.0538	-0.01244	0.04129	-0.04129	0.50108	0.34035	0.38149	0.24616	0.5952	0.48463	0.8717	0.59181	0.59181	turquoise
DYNLRB2	-0.1385	-0.20061	-0.26129	-0.0337	0.15791	-0.19211	-0.13461	0.1516	-0.1516	0.07082	0.00852	0.00056	0.66173	0.03913	0.01183	0.07919	0.04778	0.04778	grey
INAFM1	0.13508	-0.05539	-0.07149	-0.0275	-0.02346	0.03406	0.11484	-0.08493	0.08493	0.07815	0.47178	0.3528	0.72104	0.76072	0.65835	0.13473	0.2694	0.2694	grey
KCNAB2	-0.05525	0.00621	-0.01022	0.0093	-0.11323	0.05642	-0.0405	0.38586	-0.38586	0.47291	0.93573	0.89443	0.9039	0.14031	0.46355	0.59893	1.87E-07	1.87E-07	blue
EXOC3L4	0.03285	0.04147	0.08309	0.05564	0.18166	0.19396	0.15062	-0.38445	0.38445	0.66975	0.59018	0.27994	0.4698	0.01741	0.01103	0.04925	2.09E-07	2.09E-07	yellow
ETNK1	0.01509	-0.05111	-0.01567	-0.02729	0.18116	0.01018	-0.13334	-0.06058	0.06058	0.84465	0.50676	0.83877	0.72309	0.01773	0.8949	0.08211	0.43124	0.43124	turquoise
PTCH1	-0.00908	-0.16193	-0.1007	-0.0504	-0.08519	0.05205	-0.02526	0.26856	-0.26856	0.90621	0.03434	0.19004	0.51272	0.26794	0.49901	0.74299	0.00038	0.00038	turquoise
RASGEF1A	-0.08854	-0.12297	0.01937	0.01734	-0.13059	-0.09958	-0.04967	0.09912	-0.09912	0.24951	0.10908	0.80145	0.82191	0.08867	0.19503	0.51885	0.1971	0.1971	grey
ASB7	-0.07765	-0.13444	-0.07377	0.04293	-0.11632	0.01041	0.04601	0.03921	-0.03921	0.31275	0.07959	0.33761	0.57714	0.12975	0.89253	0.55016	0.61059	0.61059	turquoise
ITPR2	-0.09588	-0.08797	0.06482	0.08664	-0.26771	0.21937	0.04935	0.44096	-0.44096	0.21223	0.25255	0.39958	0.25986	0.0004	0.00394	0.52157	1.58E-09	1.58E-09	blue
STX1B	-0.06865	-0.07491	-0.02498	0.04529	-0.10951	0.04408	0.09094	0.13146	-0.13146	0.3723	0.33018	0.74569	0.55643	0.15394	0.56696	0.23683	0.08656	0.08656	grey
NASP	-0.11831	-0.10488	0.00595	0.00621	-0.06797	0.04086	0.17548	-0.07694	0.07694	0.12327	0.1722	0.93847	0.93574	0.37707	0.59571	0.02169	0.31723	0.31723	turquoise
SLC4A1AP	-0.00456	-0.08873	-0.05268	-0.02131	-0.09641	-0.01087	0.10158	0.05961	-0.05961	0.95281	0.24846	0.49381	0.78208	0.2097	0.88777	0.18617	0.43863	0.43863	turquoise
DVL2	0.02467	-0.19059	-0.07199	-0.04851	-0.00227	-0.09628	0.02197	0.25309	-0.25309	0.74879	0.01253	0.3494	0.52864	0.97651	0.21033	0.77549	0.00084	0.00084	turquoise
VPS13D	-0.07538	-0.15376	0.01025	0.03834	-0.13621	0.14153	-0.01343	0.23763	-0.23763	0.32715	0.04465	0.89415	0.61856	0.07566	0.06483	0.86161	0.00175	0.00175	turquoise
PUS7	-0.03092	-0.02511	0.02516	0.04764	-0.03934	-0.00259	0.13638	-0.05982	0.05982	0.68809	0.74439	0.74396	0.53612	0.60945	0.97323	0.07529	0.437	0.437	turquoise
PDE3A	-0.09892	-0.09945	-0.08439	0.08153	-0.16421	0.05088	-0.09106	0.55918	-0.55918	0.19804	0.1956	0.27245	0.28908	0.03186	0.5087	0.23622	1.88E-15	1.88E-15	blue
TMEM69	-0.14997	-0.03017	0.02934	-0.01329	-0.06027	0.03678	0.03516	-0.00121	0.00121	0.05024	0.6953	0.70322	0.86298	0.43356	0.63297	0.64796	0.98752	0.98752	turquoise
MGAT5	-0.00664	0.04025	0.01099	0.00359	-0.17253	0.10288	0.09713	0.13508	-0.13508	0.9313	0.60115	0.88651	0.96285	0.02404	0.18058	0.20627	0.07814	0.07814	turquoise
GATA6	-0.0144	0.01817	0.07265	0.04364	-0.01689	0.29543	0.07967	-0.06831	0.06831	0.85175	0.81352	0.34501	0.5709	0.82649	8.75E-05	0.30026	0.37467	0.37467	yellow
SNRPD3	-0.05272	-0.0248	-0.0829	-0.07913	-0.02411	-0.01522	0.13526	-0.29224	0.29224	0.49345	0.74744	0.28104	0.30357	0.75423	0.84341	0.07774	0.00011	0.00011	turquoise
ZNF670	-0.17569	-0.06811	-0.07333	-0.02694	-0.1193	0.14891	0.03809	0.15265	-0.15265	0.02153	0.37606	0.34053	0.72652	0.12013	0.05192	0.62084	0.04624	0.04624	turquoise
HSPB8	-0.07071	-0.09954	-0.06335	0.10538	-0.10747	0.03633	-0.12345	0.45865	-0.45865	0.35808	0.1952	0.41045	0.17013	0.16179	0.63707	0.10769	2.81E-10	2.81E-10	blue
SLC6A9	0.15315	-0.01414	-0.06182	0.0897	-0.05818	-0.00647	0.09347	0.00728	-0.00728	0.04552	0.85431	0.42181	0.24329	0.44972	0.93305	0.22398	0.92467	0.92467	grey
RAB20	-0.0313	0.00228	0.01522	0.07501	-0.02686	0.10815	0.12853	-0.22104	0.22104	0.6845	0.97643	0.84342	0.32953	0.72732	0.15914	0.09386	0.00367	0.00367	grey
DTNB	-0.01222	-0.05175	-0.08212	-0.00548	-0.09265	-0.08934	0.17261	-0.04131	0.04131	0.87394	0.50143	0.28559	0.94325	0.22812	0.24521	0.02397	0.59168	0.59168	grey
NNMT	-0.08164	-0.05043	-0.08663	0.10301	-0.20371	0.07046	0.10728	0.10183	-0.10183	0.28843	0.51245	0.25988	0.17999	0.00753	0.35978	0.16255	0.18507	0.18507	black
RING1	0.00579	0.01108	-0.11364	-0.02502	0.17007	-0.16418	-0.0195	0.04105	-0.04105	0.94008	0.8856	0.13888	0.74527	0.02616	0.03189	0.80011	0.59399	0.59399	grey
TCTE3	0.01758	-0.07381	-0.07602	-0.11752	0.18384	-0.15849	-0.05345	-0.05619	0.05619	0.81944	0.33735	0.32306	0.12583	0.01609	0.03842	0.48747	0.4654	0.4654	red
LEO1	-0.00065	-0.12051	-0.0597	-0.08478	0.0119	-0.06442	0.01502	-0.03401	0.03401	0.99329	0.11642	0.43794	0.27026	0.87719	0.4025	0.84544	0.65875	0.65875	turquoise
LMNA	0.04251	-0.00924	0.0245	0.02223	-0.02338	0.25032	0.2163	-0.26269	0.26269	0.58091	0.90458	0.75046	0.77284	0.76144	0.00096	0.00449	0.00052	0.00052	yellow
GTSE1	-0.06687	0.12571	0.00033	-0.00014	0.01053	0.00654	0.2675	-0.38054	0.38054	0.38487	0.10135	0.99657	0.9986	0.89128	0.9323	0.0004	2.83E-07	2.83E-07	pink
CYB5D2	-0.07546	-0.16158	-0.2329	-0.14848	0.13378	-0.3369	-0.23388	0.3706	-0.3706	0.32664	0.03474	0.00217	0.05261	0.08108	6.62E-06	0.00208	6.05E-07	6.05E-07	brown
YIPF2	-0.00515	-0.0726	-0.15739	-0.05962	0.16735	-0.17481	-0.06381	-0.08722	0.08722	0.94675	0.34537	0.0398	0.43855	0.02869	0.0222	0.407	0.25664	0.25664	grey
POLE2	0.01026	0.11656	0.05586	-0.04866	0.05401	0.04001	0.23659	-0.37906	0.37906	0.89405	0.12894	0.46803	0.52735	0.48295	0.60339	0.00184	3.18E-07	3.18E-07	pink
POLR2A	-0.02506	-0.13801	0.01335	-0.02	-0.11225	0.05976	0.01741	0.27107	-0.27107	0.7449	0.07185	0.86246	0.79513	0.14383	0.43753	0.82117	0.00034	0.00034	turquoise
TRIM59	-0.04437	-0.02291	0.00817	0.06201	-0.13686	0.0407	0.14908	-0.07376	0.07376	0.56441	0.76619	0.91553	0.42042	0.07426	0.59716	0.05165	0.3377	0.3377	black
ATP5F1D	0.09769	0.12169	-0.17066	-0.11702	0.20776	-0.16979	0.03291	-0.32783	0.32783	0.20368	0.11286	0.02564	0.12745	0.0064	0.02641	0.6691	1.20E-05	1.20E-05	green
ASB9	-0.01811	-0.03787	-0.18429	0.01127	-0.03726	-0.03717	-0.00155	0.27629	-0.27629	0.81417	0.62291	0.01583	0.88373	0.62847	0.62932	0.98391	0.00025	0.00025	brown
PDCD2L	0.08716	0.04759	-0.08341	-0.08623	0.12002	-0.11689	0.04295	-0.23064	0.23064	0.25698	0.53653	0.27807	0.26208	0.11791	0.12786	0.577	0.00241	0.00241	grey
DNAJC11	-0.07806	-0.07624	0.02556	0.02149	-0.0759	0.11474	0.00136	0.1387	-0.1387	0.3102	0.32164	0.74	0.78024	0.32381	0.13508	0.98588	0.07042	0.07042	turquoise
NAGS	-0.05425	-0.01192	0.00624	0.08153	0.03781	0.16112	0.21876	-0.37568	0.37568	0.48094	0.87704	0.93544	0.28912	0.62343	0.03527	0.00405	4.12E-07	4.12E-07	grey
PPP1R14A	-0.07938	0.00852	-0.06716	-0.0198	-0.00014	0.10993	0.05274	0.13827	-0.13827	0.30206	0.91197	0.38276	0.79712	0.99857	0.15234	0.49328	0.0713	0.0713	grey
CX3CL1	-0.11632	-0.20568	-0.05787	0.04443	-0.21869	0.00218	0.03154	-0.0097	0.0097	0.12975	0.00696	0.45218	0.56397	0.00406	0.97738	0.6822	0.89976	0.89976	grey
STARD4	0.00184	-0.05472	-0.0606	-0.07934	0.11091	0.03923	-0.056	-0.0323	0.0323	0.9809	0.47717	0.43105	0.30231	0.14869	0.61048	0.46693	0.67492	0.67492	turquoise
ACKR2	-0.04759	-0.19852	0.03276	-0.01545	-0.01997	0.05109	-0.13266	0.26412	-0.26412	0.53654	0.00924	0.67057	0.84101	0.79544	0.50693	0.08369	0.00048	0.00048	grey
GZF1	0.02992	-0.18907	-0.12598	-0.07231	-0.0104	-0.11348	-0.08994	0.30826	-0.30826	0.69768	0.01326	0.10063	0.34728	0.89262	0.13946	0.24205	4.10E-05	4.10E-05	turquoise
RILPL2	-0.01764	-0.05986	0.01907	-0.02756	-0.09622	0.13279	0.03443	0.10502	-0.10502	0.8189	0.43675	0.80449	0.7205	0.21058	0.08338	0.65487	0.17161	0.17161	blue
TBXAS1	0.04509	0.06213	0.1104	-0.01829	-0.01662	0.21629	0.17338	0.03238	-0.03238	0.55814	0.41949	0.15057	0.81229	0.82913	0.00449	0.02334	0.67415	0.67415	grey
HPS1	-0.05048	-0.05529	0.10307	0.09554	-0.05598	0.02211	0.10297	-0.14108	0.14108	0.51201	0.47262	0.17977	0.21386	0.4671	0.77411	0.18016	0.06569	0.06569	grey
MUC5B	0.06658	0.10915	0.11133	0.01936	0.10453	0.18024	0.12167	-0.1853	0.1853	0.38695	0.15527	0.14715	0.80159	0.17364	0.01832	0.1129	0.01525	0.01525	grey
METRNL	0.00181	-0.02818	0.0976	0.11438	-0.10677	0.23038	0.27164	-0.17455	0.17455	0.98124	0.71448	0.20411	0.13633	0.16455	0.00243	0.00033	0.02241	0.02241	grey
SIGLEC7	-0.09083	-0.0429	0.11403	0.09155	-0.24856	0.18777	0.06963	0.38512	-0.38512	0.23741	0.57744	0.13753	0.23372	0.00105	0.01392	0.3655	1.98E-07	1.98E-07	blue
RANGRF	-0.08019	-0.09833	-0.14971	-0.12476	0.01505	-0.19914	0.00232	0.13087	-0.13087	0.29715	0.20074	0.05066	0.10399	0.84509	0.00902	0.97597	0.08798	0.08798	grey
UBE2D2	0.00437	-0.17246	-0.10502	-0.069	-0.00518	-0.07989	-0.05159	-0.01042	0.01042	0.95481	0.0241	0.17164	0.36989	0.94634	0.29895	0.50279	0.89235	0.89235	turquoise
KMT2A	-0.07042	-0.1881	-0.07021	-0.04921	-0.16652	0.04439	0.00678	0.2154	-0.2154	0.36006	0.01375	0.36152	0.52271	0.0295	0.56424	0.92989	0.00466	0.00466	turquoise
CFDP1	0.14757	-0.10556	-0.06378	-0.07768	0.06369	0.02912	0.13383	-0.26534	0.26534	0.05409	0.1694	0.40721	0.31255	0.40793	0.7054	0.08097	0.00045	0.00045	turquoise
FZD6	-0.10092	-0.11949	-0.01264	0.08451	-0.18695	0.06108	0.12265	-0.01914	0.01914	0.18906	0.11956	0.8697	0.27178	0.01435	0.42739	0.11002	0.80372	0.80372	turquoise
ANKRD35	0.08514	-0.04221	0.03202	0.03509	-0.07708	0.1166	0.1037	0.19762	-0.19762	0.26822	0.58362	0.67755	0.64866	0.31633	0.12881	0.17709	0.00957	0.00957	black
IMPAD1	-0.02811	-0.0899	-0.09947	-0.02517	-0.11594	-0.08404	-0.04043	0.1738	-0.1738	0.7151	0.24228	0.19554	0.74385	0.13101	0.27444	0.59956	0.023	0.023	turquoise

ZNF256	-0.09483	-0.11891	-0.09878	0.06711	-0.0416	0.02369	-0.11028	0.33614	-0.33614	0.21731	0.12138	0.19866	0.38314	0.58906	0.75844	0.15102	6.96E-06	6.96E-06	grey
COMMD7	0.105	-0.04193	-0.00821	0.03531	-0.00849	0.03922	0.11616	-0.31934	0.31934	0.1717	0.58611	0.91512	0.64659	0.91225	0.61055	0.1303	2.07E-05	2.07E-05	grey
PBRM1	-0.04142	-0.08082	-0.01838	0.00666	-0.14028	0.09541	-0.00914	0.3033	-0.3033	0.59068	0.29336	0.81145	0.93112	0.06725	0.2145	0.90552	5.52E-05	5.52E-05	turquoise
IGSF5	0.14947	-0.15677	-0.07913	0.07624	0.03856	-0.07017	-0.02497	-0.02121	0.02121	0.05103	0.04059	0.30359	0.32166	0.61658	0.36178	0.74578	0.78309	0.78309	grey
CSKMT	0.18177	-0.01951	0.02539	-0.10947	0.13908	-0.0623	0.11337	-0.23678	0.23678	0.01734	0.80005	0.74164	0.15407	0.06965	0.4182	0.13985	0.00182	0.00182	grey
SMC3	-0.02039	-0.14507	0.01533	0.00173	-0.14442	0.01149	0.00264	0.18364	-0.18364	0.7912	0.05834	0.84221	0.98204	0.05949	0.88138	0.9727	0.01621	0.01621	turquoise
RIPK2	-0.01529	-0.03523	0.07074	0.0965	-0.20884	0.20445	0.23074	-0.17423	0.17423	0.84266	0.64734	0.35786	0.20928	0.00612	0.00731	0.0024	0.02266	0.02266	turquoise
GMEB2	0.00488	-0.0303	-0.1267	-0.04045	0.09415	-0.10973	0.01747	-0.20122	0.20122	0.94952	0.69397	0.09867	0.59941	0.22065	0.1531	0.82063	0.00832	0.00832	green
AP3D1	0.05866	-0.0539	-0.14778	-0.06554	0.12224	-0.18078	-0.06382	-0.00633	0.00633	0.44602	0.48383	0.05374	0.3944	0.11121	0.01797	0.40693	0.93454	0.93454	green
TADA2A	-0.04322	-0.1064	-0.14546	-0.06052	0.20605	-0.14806	-0.04837	0.10421	-0.10421	0.57464	0.16601	0.05765	0.4317	0.00685	0.05329	0.52987	0.17497	0.17497	turquoise
GAB3	-0.11284	-0.06415	-0.0168	0.04176	-0.16054	0.06472	-0.0015	0.51074	-0.51074	0.14172	0.4045	0.82732	0.58762	0.03595	0.40037	0.98448	9.55E-13	9.55E-13	blue
LILRA6	-0.0998	0.0467	0.1392	0.07467	-0.19752	0.11659	0.07948	0.32134	-0.32134	0.19405	0.54416	0.0694	0.3317	0.00961	0.12885	0.30142	1.82E-05	1.82E-05	blue
CSRNP2	0.0058	-0.27412	-0.08412	-0.00274	-0.04283	-0.06562	-0.12516	0.30393	-0.30393	0.93998	0.00029	0.27401	0.97158	0.57805	0.39384	0.10287	5.31E-05	5.31E-05	turquoise
KCNQ1	-0.00339	0.04542	0.07823	0.09628	-0.07541	0.28613	0.15201	-0.21328	0.21328	0.96485	0.55529	0.30912	0.21033	0.32694	0.00015	0.04717	0.0051	0.0051	grey
HEATR5A	-0.04425	-0.12119	0.04643	0.08355	-0.05713	0.08869	0.03363	0.2106	-0.2106	0.5655	0.11433	0.54652	0.27727	0.45795	0.24867	0.66231	0.00569	0.00569	turquoise
RND3	-0.05752	-0.12206	0.05059	0.11172	-0.16607	0.12365	0.16127	0.00553	-0.00553	0.45494	0.11174	0.51111	0.14574	0.02994	0.10713	0.0351	0.94281	0.94281	turquoise
MGST1	0.00879	0.08399	0.08	0.1092	-0.06386	0.15812	0.25712	-0.28305	0.28305	0.90918	0.27476	0.29826	0.1551	0.40668	0.03887	0.00069	0.00018	0.00018	yellow
RUNX2	0.03048	-0.01532	0.11539	0.07278	-0.21658	0.16248	0.19457	0.08347	-0.08347	0.6923	0.8424	0.13288	0.34417	0.00444	0.03373	0.01077	0.27777	0.27777	black
ALDH4A1	-0.06049	0.0693	-0.14764	-0.00014	0.00417	-0.06569	-0.02753	0.09083	-0.09083	0.43193	0.36777	0.05398	0.99859	0.95687	0.39333	0.72078	0.23741	0.23741	grey
RPS12	0.14057	0.15715	0.04339	-0.02808	-0.06824	0.09297	0.11142	-0.05983	0.05983	0.06668	0.0401	0.5731	0.71547	0.37516	0.22649	0.14682	0.43692	0.43692	purple
RAB32	-0.0798	-0.02079	0.0328	0.09603	-0.23354	0.17531	0.16183	0.09458	-0.09458	0.2995	0.78724	0.67021	0.21149	0.00211	0.02182	0.03446	0.2185	0.2185	black
NID1	-0.07902	-0.11323	-0.01187	0.03988	-0.19619	0.11055	0.02431	0.34135	-0.34135	0.30425	0.14033	0.87749	0.60457	0.01012	0.15004	0.75226	4.90E-06	4.90E-06	black
METTL23	-0.06455	-0.12563	-0.05099	-0.1048	0.11223	-0.06641	0.02697	-0.11731	0.11731	0.40162	0.10157	0.50773	0.17252	0.14388	0.38812	0.72622	0.12651	0.12651	grey
CD164L2	-0.02378	-0.14657	0.13154	0.11679	0.04052	0.00867	0.08921	-0.34388	0.34388	0.75754	0.05576	0.08637	0.12821	0.59877	0.91036	0.24591	4.12E-06	4.12E-06	grey
HNF4G	0.04004	0.0299	-0.00901	0.01767	0.08869	0.17578	0.04925	-0.1852	0.1852	0.60312	0.69789	0.90686	0.81853	0.24871	0.02147	0.52237	0.01531	0.01531	yellow
HADH	-0.09034	0.01166	-0.15064	-0.03522	-0.02006	0.11775	0.07451	0.19342	-0.19342	0.23998	0.87965	0.04922	0.64745	0.79457	0.12506	0.33279	0.01125	0.01125	grey
ZNF547	-0.02615	-0.25941	-0.2166	-0.03711	0.08881	-0.22168	-0.19137	0.11707	-0.11707	0.73427	0.00061	0.00443	0.62993	0.24804	0.00357	0.01217	0.12728	0.12728	grey
COL14A1	-0.07141	-0.08565	0.00515	0.07306	-0.14366	0.12278	-0.05302	0.59525	-0.59525	0.35336	0.26533	0.94668	0.34231	0.06086	0.10964	0.49101	9.00E-18	9.00E-18	blue
DDRGK1	0.11984	0.01828	-0.11971	-0.04883	0.24172	-0.25684	-0.00429	-0.17518	0.17518	0.11846	0.81245	0.11888	0.52595	0.00145	0.0007	0.95563	0.02192	0.02192	grey
UTP14A	0.04981	0.00946	0.02341	0.02114	-0.16195	0.20468	0.24736	-0.17803	0.17803	0.51762	0.90229	0.7612	0.78375	0.03432	0.00724	0.00111	0.01982	0.01982	turquoise
ZDHHC18	-0.08719	-0.19943	0.08827	0.06779	-0.17999	0.0914	0.12495	0.11897	-0.11897	0.25684	0.00892	0.25097	0.37831	0.01849	0.23447	0.10345	0.12117	0.12117	turquoise
HS2ST1	-0.04764	-0.14718	-0.01565	0.02454	-0.18441	0.00522	-0.00704	0.19144	-0.19144	0.5361	0.05474	0.83902	0.75003	0.01575	0.94597	0.92714	0.01213	0.01213	turquoise
FTL	-0.03987	0.06392	0.08975	0.08307	-0.15115	0.13892	0.0733	0.09752	-0.09752	0.60467	0.40619	0.24304	0.28006	0.04845	0.06998	0.3407	0.20447	0.20447	blue
LRRC42	-0.06501	-0.11164	-0.01334	0.00724	-0.11605	0.02334	0.16491	-0.10212	0.10212	0.39821	0.14602	0.86247	0.92514	0.13066	0.7619	0.03112	0.18381	0.18381	turquoise
CD302	-0.06213	-0.12876	0.02775	0.06317	-0.16838	0.10683	-0.0098	0.54041	-0.54041	0.41952	0.09326	0.71864	0.41179	0.0277	0.16431	0.89876	2.37E-14	2.37E-14	blue
YTHDF3	-0.03911	-0.10121	-0.04018	0.04674	-0.08989	-0.00487	-0.01581	0.20403	-0.20403	0.61158	0.18779	0.60181	0.54381	0.24234	0.94955	0.83735	0.00744	0.00744	turquoise
MB	0.11681	-0.08473	-0.0023	0.05556	0.00245	0.01685	0.05064	-0.24063	0.24063	0.12812	0.27052	0.97615	0.47044	0.9746	0.82681	0.51068	0.00152	0.00152	grey
RPL31	0.15397	0.1612	0.03984	-0.01768	-0.08501	0.09982	0.1452	-0.20968	0.20968	0.04436	0.03518	0.60492	0.81847	0.26893	0.19394	0.05812	0.00591	0.00591	purple
KCNJ8	-0.08838	-0.08938	-0.13019	-0.01051	-0.01334	-0.21556	-0.24347	0.60486	-0.60486	0.25033	0.24503	0.08965	0.89144	0.86256	0.00463	0.00133	1.93E-18	1.93E-18	brown
DFFA	-0.07501	-0.14534	0.00716	0.01809	-0.14594	0.11018	0.0846	0.13486	-0.13486	0.32951	0.05787	0.92591	0.81435	0.05682	0.15141	0.27128	0.07865	0.07865	turquoise
HLA-DMA	-0.13018	0.12516	0.04255	0.09165	-0.2298	0.20271	0.20225	0.13507	-0.13507	0.08969	0.10288	0.58052	0.2332	0.0025	0.00784	0.00798	0.07817	0.07817	blue
ALDH9A1	-0.09813	-0.05231	-0.08461	-0.09625	0.09034	-0.04285	-0.10737	0.23195	-0.23195	0.20165	0.49679	0.2712	0.21044	0.23998	0.5779	0.16219	0.00227	0.00227	turquoise
RAB30	-0.04178	-0.12447	0.06937	0.05351	-0.13279	0.10281	-0.12575	0.4144	-0.4144	0.58743	0.10481	0.36732	0.487	0.08339	0.18087	0.10124	1.75E-08	1.75E-08	blue
C1orf216	-0.08389	-0.18074	-0.05212	0.04142	-0.19049	-0.05501	0.01746	0.37416	-0.37416	0.27532	0.018	0.4984	0.59061	0.01257	0.47481	0.82067	4.63E-07	4.63E-07	blue
PM20D1	0.01303	0.04389	0.11304	0.07385	-0.0883	0.02825	-0.13931	0.24241	-0.24241	0.86565	0.56871	0.14099	0.33707	0.25081	0.7138	0.06917	0.0014	0.0014	magenta
PUS1	0.08402	0.11145	0.00771	-0.07305	0.11733	0.02619	0.10851	-0.32404	0.32404	0.27455	0.1467	0.92024	0.34235	0.12644	0.73386	0.15774	1.54E-05	1.54E-05	grey
SSNA1	0.05735	-0.00196	-0.1032	-0.03229	0.01075	-0.08199	0.08152	-0.36808	0.36808	0.45626	0.97966	0.17923	0.67506	0.88901	0.28637	0.28917	7.31E-07	7.31E-07	grey
CLEC12A	-0.07126	0.0246	0.13834	0.08323	-0.18551	0.20912	0.10195	0.26999	-0.26999	0.35432	0.74947	0.07117	0.27914	0.01513	0.00605	0.18455	0.00036	0.00036	blue
ZNF461	-0.08279	-0.19569	-0.08085	0.02295	-0.0598	-0.0542	-0.09351	0.26383	-0.26383	0.28168	0.01032	0.29314	0.76579	0.43716	0.48137	0.2238	0.00049	0.00049	turquoise
PTPRN2	0.07958	-0.02507	-0.18649	-0.16845	0.2023	-0.27709	-0.23636	0.2367	-0.2367	0.30082	0.74486	0.0146	0.02764	0.00797	0.00024	0.00186	0.00183	0.00183	brown
C19orf47	0.0219	-0.18098	-0.02681	0.08553	-0.04617	-0.01814	0.10091	-0.1206	0.1206	0.77616	0.01784	0.72776	0.26601	0.54874	0.81387	0.18911	0.11612	0.11612	grey
COL6A3	-0.07687	-0.06902	0.0516	0.09561	-0.19808	0.15856	0.10984	0.24458	-0.24458	0.31766	0.36969	0.50268	0.2135	0.00941	0.03832	0.15268	0.00126	0.00126	black
UTP15	-0.05401	-0.06427	-0.07411	-0.04914	-0.07014	-0.0159	0.01742	0.11938	-0.11938	0.48294	0.40363	0.3354	0.52327	0.36196	0.83648	0.82109	0.11988	0.11988	turquoise
WDFY1	0.00104	-0.05364	0.07797	0.11206	-0.13702	0.17074	0.11935	0.01296	-0.01296	0.98918	0.48596	0.31076	0.14451	0.07393	0.02556	0.11998	0.86644	0.86644	turquoise
ATP1A3	0.03737	-0.13185	-0.15593	-0.05392	0.11881	-0.31884	-0.28476	0.33536	-0.33536	0.62753	0.0856	0.04169	0.48363	0.12168	2.14E-05	0.00016	7.33E-06	7.33E-06	brown
CXorf21	-0.14435	-0.03201	0.04121	0.03126	-0.17685	0.13882	0.02591	0.48462	-0.48462	0.05961	0.67772	0.59253	0.68485	0.02067	0.07018	0.73662	1.86E-11	1.86E-11	blue
SMIM10	-0.0883	-0.0536	-0.0425	0.07439	-0.27125	0.07525	0.0801	0.32252	-0.32252	0.25078	0.48624	0.58097	0.33353	0.00033	0.32795	0.29765	1.69E-05	1.69E-05	blue
FOPNL	-0.04836	-0.05019	-0.02482	#######	-0.10842	0.01055	0.01394	0.07421	-0.07421	0.52992	0.51441	0.74728	0.99973	0.15809	0.89108	0.85635	0.33472	0.33472	turquoise
ZFP69	-0.08238	-0.0495	0.02526	0.08046	-0.13147	0.06283	0.05534	0.22369	-0.22369	0.28409	0.52023	0.74295	0.29552	0.08652	0.41428	0.47222	0.00327	0.00327	turquoise
SULT1C2	0.08292	0.08992	0.14481	0.06506	0.08006	0.18168	0.07075	-0.30146	0.30146	0.28092	0.24216	0.0588	0.39785	0.29789	0.0174	0.35778	6.16E-05	6.16E-05	yellow
DST	-0.01804	-0.23702	-0.05345	0.03534	-0.07662	0.02474	-0.04035	0.21204	-0.21204	0.8148	0.0018	0.4875	0.64631	0.31922	0.74804	0.60028	0.00537	0.00537	turquoise
ANGPT2	-0.09718	-0.12481	-0.07759	0.00572	-0.05777	-0.10203	-0.02528	0.07828	-0.07828	0.20607	0.10384	0.31311	0.94086	0.45296	0.1842	0.7428	0.3088	0.3088	black
DNHD1	-0.02989	-0.14313	-0.02948	0.00905	0.06318	-0.01905	-0.03672	0.05929	-0.05929	0.69797	0.06182	0.70189	0.90645	0.41168	0.80465	0.63348	0.4411	0.4411	red
CHI3L1	-0.08462	0.10562	0.09283	0.17342	-0.10732	0.12673	0.09936	0.17258	-0.17258	0.27118	0.16916	0.22719	0.0233	0.16236	0.0986	0.19601	0.024	0.024	blue
KLHDC10	-0.02278	-0.20855	-0.12977	-0.02972	0.02492	-0.20071	-0.11646	0.26351	-0.26351	0.76742	0.00619	0.09071	0.69956	0.74632	0.00848	0.12928	0.0005	0.0005	turquoise
LRP6	-0.04678	-0.16528	-0.069	0.00029	-0.04363	-0.01633	-0.04007	0.27716	-0.27716	0.54345	0.03075	0.36983	0.99702	0.57098	0.83214	0.60284	0.00024	0.00024	turquoise
RBM3	-0.05652	0.10069	-0.04304	-0.04538	-0.14915	0.04687	0.10244	-0.10254	0.10254	0.46276	0.19006	0.57618	0.55562	0.05154	0.54268	0.18244	0.18201	0.18201	turquoise
XPR1	0.04084	-0.15417	-0.0897	-0.02059	-0.10967	0.01549	0.0118	0.11789	-0.11789	0.59584	0.04409	0.24333	0.78922	0.1533	0.84066	0.8783	0.12461	0.12461	turquoise
PIP5K1A	-0.06013	-0.23954	-0.04726	-0.04626	-0.0667	-0.00484	-0.00703	0.06337	-0.06337	0.43467	0.0016	0.53931	0.54798	0.38604	0.94992	0.92726	0.4103	0.4103	turquoise
NAA35	-0.01187	-0.07551	-0.09867	-0.03208	-0.11033	0.01561	0.08681	0.01217	-0.01217	0.87757	0.32633	0.19917	0.67704	0.15083	0.83946	0.25891	0.87445	0.87445	turquoise
ZNF287	-0.1087	-0.14748	-0.12788	-0.03562	-0.0481	-0.18369	-0.1363	0.50079	-0.50079	0.157	0.05424	0.09555	0.64373	0.53215	0.01618	0.07547	3.05E-12	3.05E-12	turquoise
PTPN22	-0.12125	0.0135	0.09332	0.08029	-0.14975	0.14498	0.09106	0.1936	-0.1936	0.11416	0.86093	0.22472	0.29654	0.05059	0.0585	0.23624	0.01118	0.01118	blue
ALAD	-0.02577	-0.09994	#######	0.07517	-0.02938	0.12096	-0.06265	0.15444	-0.15444	0.73799	0.1934	0.99952	0.32848	0.7029	0.11504	0.4156	0.0437	0.0437	turquoise
ACTG1	-0.00456	-0.15134	-0.05253	-0.06159	-0.0545	0.05152	0.087	-0.1762	0.1762	0.95276	0.04816	0.49506	0.42356	0.47896	0.50337	0.25786	0.02115	0.02115	grey
TMBIM1	0.07298	-0.00245	0.12551	0.0695	-0.12215	0.22152	0.19958	-0.34758	0.34758	0.3428	0.97465	0.1019	0.36638	0.11149	0.00359	0.00887	3.19E-06	3.19E-06	yellow
SLITRK4	0.02924	0.01047	0.02983	0.05211	-0.25797	0.11075	0.01721	0.34737	-0.34737	0.70417	0.89194	0.69858	0.49846	0.00066	0.14927	0.82319	3.24E-06	3.24E-06	blue
FDX1	-0.00498	-0.05526	-0.06343	0.01496	-0.00164	0.00597	-0.02172	-0.10647	0.10647	0.9485	0.47287	0.40984	0.846	0.98299	0.93823	0.77799	0.16573	0.16573	turquoise
FAM171A1	-0.12871	0.01179	-0.12074	0.03972	-0.10221	0.12517	-0.06345	0.24927	-0.24927	0.09339	0.87831	0.1157	0.60597	0.18344	0.10285	0.40967	0.00101	0.00101	grey
FAHD2A	0.01469	0.07399	0.01441	0.03769	0.12073	-0.00358	0.0841	-0.08026	0.08026	0.84879	0.33616	0.8516	0.62458	0.11574	0.96297	0.27412	0.29673	0.29673	grey
ADORA1	-0.00133	-0.05651	-0.07712	0.06388	0.00792	0.0234	-0.02576	0.15391	-0.15391	0.98623	0.46286	0.31606	0.4065	0.91809	0.76126	0.73802	0.04444	0.04444	magenta
COA6	0.0282	0.07962	-0.03058	-0.05633	0.14158	-0.00702	0.15771	-0.38213	0.38213	0.7143	0.30057	0.69137	0.46431	0.06472	0.92744	0.03939	2.50E-07	2.50E-07	greenyellow
GLI2	-0.03478	-0.06014	0.02867	0.08418	-0.20403	0.17782	0.13716	0.19503	-0.19503	0.65152	0.43458	0.70969	0.27364	0.00743	0.01997	0.07362	0.01058	0.01058	black
ST3GAL4	0.00073	-0.08413	-0.00947	0.01259	-0.11299	0.05092	0.15431	-0.04493	0.04493	0.99245	0.27394	0.90213	0.8702	0.14119	0.50835	0.04389	0.55958	0.55958	grey
DGKZ	0.01534	-0.06447	-0.11106	-0.11152	0.09719	-0.2267	-0.09733	-0.05065	0.05065	0.84216	0.40216	0.14813	0.14644	0.20603	0.00287	0.20534	0.51059	0.51059	green
TXLNA	-0.04985	-0.23576	-0.09717	-0.02885	-0.03475	-0.07462	-0.07149	0.27456	-0.27456	0.51734	0.00191	0.20612	0.70798	0.65184	0.33204	0.35276	0.00028	0.00028	turquoise
RLIM	-0.07552	-0.17297	-0.07989	-0.01421	-0.11968	0.06428	-0.01429	0.1313	-0.1313	0.32622	0.02368	0.29894	0.85369	0.11896	0.40357	0.85286	0.08693	0.08693	turquoise
SLC10A6	-0.17207	-0.14684	-0.12887	0.02313	-0.0082	-0.05675	-0.13771	0.53048	-0.53048	0.02442	0.0553	0.09298	0.76393	0.91522	0.46096	0.07246	8.49E-14	8.49E-14	blue
ARF6	-0.00213	-0.02712	0.06378	0.06913	-0.06145	0.20331	0.11891	-0.21096	0.21096	0.97793	0.72477	0.40721	0.36894	0.4246	0.00765	0.12139	0.00561	0.00561	turquoise
ABHD17A	0.01242	-0.00426	-0.13155	-0.08063	0.24662	-0.12855	-0.01905	-0.0962	0.0962	0.87189	0.95591	0.08632	0.29451	0.00115	0.09382	0.80463	0.21071	0.21071	green
DPM3	-0.02209	-0.00699	-0.14319	-0.1674	0.22108	-0.18325	-0.06054	-0.13551	0.13551	0.77424	0.92775	0.06171	0.02864	0.00366	0.01643	0.4315	0.0772	0.0772	green
B4GALNT1	-0.18	-0.06634	0.01691	0.05805	-0.10429	-0.02277	0.07047	0.37024	-0.37024	0.01848	0.38861	0.82626	0.45073	0.17462	0.76754	0.35969	6.22E-07	6.22E-07	black
AC008750	-0.07801	-0.00944	-0.11259	-0.04467	0.2149	-0.19901	0.01586	-0.04836	0.04836	0.31053	0.90244	0.1426	0.56182	0.00476	0.00907	0.83684	0.52996	0.52996	grey
JPH2	-0.0954	0.08943	0.02636	0.077	-0.04206	0.07168	0.12829	0.10997	-0.10997	0.21454	0.24473	0.73214	0.31682	0.58492	0.35152	0.09448	0.15221	0.15221	salmon
NADK2	-0.04211	-0.02294	0.0327	0.14637	-0.13916	0.11623	0.1151	0.2047	-0.2047	0.58447	0.76589	0.67113	0.05609	0.06948	0.13006	0.13386	0.00724	0.00724	turquoise
CDC5L	-0.01011	-0.07235	-0.0273	0.02405	-0.06595	0.04045	0.0163	0.21821	-0.21821	0.89555	0.347	0.72297	0.75483	0.39145	0.59936	0.83244	0.00414	0.00414	turquoise
RNF38	-0.04266	-0.16466	-0.05627	0.01534	-0.08677	0.04374	-0.10551	0.3074	-0.3074	0.57957	0.03139	0.46478	0.8422	0.25911	0.57002	0.16962	4.32E-05	4.32E-05	turquoise
ZBTB2	-0.11418	-0.05249	0.08443	0.10546	-0.19748	0.18678	0.05028	0.22703	-0.22703	0.13702	0.49531	0.27221	0.16983	0.00963	0.01444	0.51369	0.00282	0.00282	turquoise
WT1	-0.10508	-0.06502	0.06129	0.12138	-0.15997	0.09072	-0.01302	0.51784	-0.51784	0.17138	0.39814	0.42585	0.11378	0.03661	0.23797	0.86577	4.07E-13	4.07E-13	blue
FAM149B1	-0.0188	-0.09384	0.07733	0.08392	-0.10146	0.10383	0.16193	-0.01498	0.01498	0.80716	0.22217	0.31476	0.27518	0.18669	0.17656	0.03435	0.84582	0.84582	turquoise
MGME1	-0.03732	0.0549	-0.04531	-0.01578	-0.07622	0.01789	0.23816	-0.16834	0.16834	0.62798	0.47576	0.55625	0.83765	0.32174	0.8163	0.00171	0.02774	0.02774	pink
FP565260	-0.04858	-0.03732	-0.04519	0.06342	-0.02675	0.07698	-0.00232	0.04187	-0.04187	0.52808	0.62797	0.5573	0.4099	0.72834	0.31697	0.97596	0.5866	0.5866	grey
ACVR1B	0.04106	-0.09375	0.02829	0.13371	0.0414	0.13491	-0.11391	-0.0546	0.0546	0.59392	0.22262	0.71341	0.08125	0.59085	0.07853	0.13796	0.47817	0.47817	turquoise
RBM25	-0.03736	-0.13489	0.01008	0.03491	0.06127	0.03818	0.01061	0.03064	-0.03064	0.62754	0.07858	0.89587	0.65031	0.42601	0.62007	0.89049	0.69076	0.69076	red
IGFL2	-0.07385	0.0724	0.0778	0.02555	-0.12381	0.10572	0.11232	-0.10605	0.10605	0.33708	0.34667	0.31184	0.74012	0.10666	0.16877	0.14356	0.16744	0.16744	black
RHOBTB1	-0.03911	-0.16985	0.0117	0.05908	-0.11029	0.12935	0.09627	0.15629	-0.15629	0.61157	0.02636	0.87926	0.4427	0.151	0.09176	0.21035	0.04122	0.04122	blue
NENF	0.04001	-0.1017	-0.23826	-0.12303	0.10735	-0.33971	-0.10198	0.05772	-0.05772	0.60336	0.18566	0.0017	0.10892	0.16226	5.48E-06	0.18442	0.45335	0.45335	grey
ARID5B	-0.1336	-0.14386	-0.01042	0.05529	-0.22474	0.09853	0.00795	0.42705	-0.42705	0.08151	0.06049	0.89245	0.47261	0.00312	0.19981	0.91779	5.72E-09	5.72E-09	blue
NEURL1B	-0.08957	-0.1802	-0.20367	0.04873	-0.06623	-0.17782	-0.15683	0.33898	-0.33898	0.24401	0.01835	0.00754	0.5268	0.3894	0.01998	0.04052	5.75E-06	5.75E-06	turquoise
CXCR2	-0.14289	-0.05048	0.0941	0.0147	-0.09958	0.11603	0.0538	0.31735	-0.31735	0.06227	0.51202	0.22088	0.8487	0.19501	0.13074	0.48463	2.34E-05	2.34E-05	blue
PCCA	0.05049	0.05642	-0.09869	0.01672	0.00087	0.03238	-0.03622	0.10503	-0.10503	0.51194	0.46356	0.19908	0.82817	0.99095	0.67418	0.63812	0.17159	0.17159	grey
PPP1CA	#######	0.03561	0.05818	0.01918	-0.08432	0.09388	0.22467	-0.43456	0.43456	0.99934	0.64379	0.44973	0.8034	0.27289	0.22197	0.00313	2.87E-09	2.87E-09	yellow
UBAP2	0.00574	-0.09153	-0.09287	-0.06496	-0.027	-0.04096	-0.01113	-0.0569	0.0569	0.94063	0.23382	0.227	0.39862	0.72592	0.59477	0.88512	0.45981	0.45981	turquoise
RFC3	-0.08092	0.11162	-0.09622	-0.10589	-0.09222	-0.02998	0.1003	0.01782	-0.01782	0.29273	0.1461	0.21059	0.16806	0.23027	0.6971	0.19181	0.81705	0.81705	turquoise
IL17RE	0.06963	0.01876	0.06312	0.01266	0.10047	0.16751	0.12114	-0.45938	0.45938	0.3655	0.80759	0.41212	0.86949	0.19104	0.02853	0.11449	2.62E-10	2.62E-10	yellow
SESN2	0.08853	-0.28065	-0.07909	-0.00671	0.04698	-0.1059	-0.13588	0.24236	-0.24236	0.24956	0.0002	0.30381	0.93056	0.54173	0.16805	0.07639	0.0014	0.0014	grey
INO80B	0.08198	0.02429	0.00533	-0.05459	0.14915	0.00351	0.13487	-0.39433	0.39433	0.28642	0.75247	0.94487	0.47824	0.05153	0.9637	0.07861	9.47E-08	9.47E-08	red
FUT11	-0.07176	-0.26623	0.01186	0.04658	-0.16803	0.0303	0.01998	0.14945	-0.14945	0.35098	0.00043	0.87763	0.54516	0.02804	0.69402	0.79535	0.05106	0.05106	turquoise
RNFT1	-0.14481	-0.22481	-0.08485	0.0091	0.01097	-0.03671	-0.10597	0.2898	-0.2898	0.05879	0.00311	0.26986	0.90601	0.88679	0.63358	0.16776	0.00012	0.00012	turquoise
ADA2	-0.08423	-0.00261	#######	0.02694	-0.13969	0.00023	-0.10107	0.47266	-0.47266	0.27338	0.97299	0.99995	0.72654	0.06843	0.99766	0.18839	6.69E-11	6.69E-11	blue
ATP6V1A	-0.04059	-0.1143	-0.07205	-0.02229	-0.14708	0.01012	0.06048	0.19775	-0.19775	0.59809	0.13661	0.349	0.77227	0.0549	0.8955	0.43201	0.00953	0.00953	turquoise
MRVI1	-0.06976	-0.06434	0.01731	0.02393	-0.14656	0.14804	0.05071	0.28612	-0.28612	0.36459	0.40314	0.82224	0.75606	0.05578	0.05332	0.5101	0.00015	0.00015	blue
ASXL1	-0.06845	-0.15889	-0.02404	0.04111	-0.10938	0.02608	0.03742	0.18607	-0.18607	0.37366	0.03791	0.75501	0.59347	0.15442	0.73489	0.62705	0.01482	0.01482	turquoise
RBPMS	-0.00038	-0.09511	0.02702	0.11211	-0.0972	0.20513	0.11234	-0.05565	0.05565	0.99604	0.21593	0.7257	0.14432	0.20597	0.00712	0.1435	0.46973	0.46973	black
ANP32B	0.11279	0.02714	-0.03001	-0.03332	-0.13435	0.08696	0.16925	-0.27293	0.27293	0.1419	0.72459	0.69681	0.66529	0.07979	0.25805	0.0269	0.0003	0.0003	turquoise
GHDC	0.04396	0.01349	-0.10216	-0.08787	0.05168	-0.00423	-0.02902	-0.22302	0.22302	0.56806	0.86098	0.18364	0.25309	0.50199	0.9562	0.70637	0.00337	0.00337	grey
ARSG	0.00221	-0.18225	-0.22981	-0.14963	0.06279	-0.31581	-0.16434	0.43738	-0.43738	0.97708	0.01704	0.0025	0.05078	0.41457	2.58E-05	0.03173	2.21E-09	2.21E-09	brown
ZNF281	-0.08353	-0.18018	0.00183	0.05098	-0.19436	0.05371	0.06354	0.21657	-0.21657	0.27741	0.01836	0.98108	0.50783	0.01085	0.48537	0.409	0.00444	0.00444	turquoise
BCLAF3	0.01862	-0.14545	-0.04969	0.03838	0.02106	0.05925	0.02044	-0.04587	0.04587	0.809	0.05768	0.51862	0.61822	0.78453	0.4414	0.79072	0.55136	0.55136	turquoise
NFIB	-0.05948	-0.09476	0.00013	0.10296	-0.23406	0.20464	0.1244	0.25359	-0.25359	0.43963	0.21766	0.99864	0.18021	0.00206	0.00726	0.105	0.00082	0.00082	blue

CNEP1R1	-0.08972	-0.24433	0.0435	0.08559	-0.07231	0.0308	-0.02402	0.19911	-0.19911	0.2432	0.00128	0.57214	0.26568	0.34732	0.68924	0.75515	0.00903	0.00903	turquoise
DPY19L2	-0.11863	-0.12166	-0.24991	-0.04146	0.13856	-0.30582	-0.18198	0.35424	-0.35424	0.12227	0.11295	0.00098	0.59033	0.0707	4.75E-05	0.01721	2.00E-06	2.00E-06	brown
ZNF493	-0.05543	-0.10376	-0.11754	-0.02654	0.0981	-0.04405	-0.14089	0.29612	-0.29612	0.4715	0.17685	0.12574	0.73041	0.20179	0.56726	0.06606	8.41E-05	8.41E-05	turquoise
NMNAT1	-0.02879	-0.09702	0.01986	0.04459	-0.00486	0.11723	0.0354	0.23804	-0.23804	0.70852	0.2068	0.79655	0.56253	0.94967	0.12676	0.64572	0.00172	0.00172	turquoise
DHRS4	0.04472	0.01305	-0.08231	0.06155	0.19736	-0.13883	-0.08051	-0.07481	0.07481	0.56141	0.86553	0.28452	0.42389	0.00967	0.07015	0.29522	0.33083	0.33083	grey
RNF111	-0.02389	-0.1061	-0.01983	0.03833	-0.10243	0.05459	0.02158	0.18997	-0.18997	0.7564	0.02991	0.79684	0.61865	0.1825	0.47826	0.77938	0.01282	0.01282	turquoise
TTC31	0.03498	-0.0285	0.04597	0.0129	-0.01216	0.12642	0.13278	-0.14187	0.14187	0.6497	0.71133	0.5505	0.86703	0.87456	0.09943	0.08341	0.06418	0.06418	red
CD40LG	-0.08548	-0.03494	0.0248	0.01794	-0.10823	0.1507	-0.05558	0.38261	-0.38261	0.26632	0.65003	0.74746	0.81581	0.15883	0.04914	0.39414	2.41E-07	2.41E-07	blue
MPG	0.00633	0.05589	0.01351	-0.0231	0.08918	0.04592	0.11674	-0.36389	0.36389	0.93447	0.46783	0.86081	0.76428	0.24608	0.55091	0.12836	9.96E-07	9.96E-07	green
PPIL1	-0.07728	0.0175	-0.00031	0.00482	-0.0698	0.04633	0.13779	0.0508	-0.0508	0.31504	0.82032	0.99682	0.95012	0.36432	0.54734	0.07231	0.50931	0.50931	turquoise
SLC12A2	-0.05435	-0.10602	0.05616	0.08285	-0.00446	0.22216	0.00885	-0.10266	0.10266	0.48021	0.16754	0.46562	0.28136	0.95386	0.0035	0.90854	0.18151	0.18151	turquoise
TMEM120	0.09998	-0.14428	-0.04041	-0.08379	0.1507	-0.20461	0.03378	-0.09356	0.09356	0.19323	0.05974	0.59976	0.27591	0.04913	0.00726	0.66097	0.22353	0.22353	grey
ZNF383	-0.05003	-0.15273	-0.09763	-0.00605	-0.03884	-0.03518	-0.09577	0.31507	-0.31507	0.5158	0.04613	0.20397	0.93743	0.61398	0.64783	0.21274	2.70E-05	2.70E-05	turquoise
PLD4	-0.0708	-0.02527	-0.07764	-0.04344	-0.10696	0.09986	-0.0025	0.35885	-0.35885	0.35745	0.74283	0.31279	0.57263	0.16379	0.19375	0.97412	1.44E-06	1.44E-06	blue
HINT2	0.03625	0.0093	-0.17622	-0.1966	0.12885	-0.17884	-0.11013	-0.09525	0.09525	0.63782	0.9039	0.02113	0.00996	0.09303	0.01927	0.1516	0.21526	0.21526	grey
PRKCH	-0.14212	-0.1373	0.03818	0.12716	-0.21993	0.11992	-0.01089	0.39543	-0.39543	0.0637	0.07334	0.62009	0.09744	0.00385	0.11821	0.88755	8.66E-08	8.66E-08	blue
ARF1	0.0293	-0.02251	-0.08123	-0.06781	0.06848	-0.02928	0.05603	-0.30833	0.30833	0.70368	0.77014	0.29089	0.37818	0.37346	0.7038	0.4667	4.08E-05	4.08E-05	greenyellow
PATL2	-0.11638	0.04922	0.0257	-0.01787	-0.0052	0.04894	0.01091	0.19026	-0.19026	0.12957	0.52264	0.73865	0.81655	0.94616	0.52499	0.88739	0.01268	0.01268	blue
ACACB	-0.02134	-0.08953	-0.15524	-0.01964	0.0848	-0.17795	-0.24301	0.49139	-0.49139	0.78172	0.24423	0.04262	0.79877	0.27014	0.01988	0.00136	8.83E-12	8.83E-12	brown
SHISA4	0.03211	-0.09848	-0.04742	0.01002	-0.1662	-0.0379	0.02142	0.17872	-0.17872	0.67675	0.20005	0.53793	0.8965	0.02982	0.62265	0.78091	0.01934	0.01934	black
PAN2	0.0244	-0.12278	-0.09949	-0.04685	0.21505	-0.13389	-0.13683	0.08712	-0.08712	0.7514	0.10962	0.19542	0.54289	0.00473	0.08084	0.07434	0.25721	0.25721	red
CTSG	-0.0099	-0.04521	-0.01172	0.06216	-0.13727	0.00749	-0.14837	0.50568	-0.50568	0.89769	0.55707	0.87903	0.41929	0.0734	0.92254	0.05278	1.73E-12	1.73E-12	blue
TRAFD1	0.02329	-0.13423	0.02716	0.03056	-0.03551	0.04676	0.08574	0.02849	-0.02849	0.76235	0.08007	0.72441	0.69155	0.64476	0.54361	0.26485	0.71142	0.71142	turquoise
ZNF608	0.01753	-0.17665	-0.1061	0.01949	-0.0702	-0.04587	-0.05577	0.07216	-0.07216	0.82	0.02081	0.16721	0.8003	0.36157	0.55134	0.46873	0.34831	0.34831	turquoise
APBB3	#######	-0.11049	-0.10304	-0.10907	0.164	-0.18365	-0.11752	0.10744	-0.10744	0.99952	0.15025	0.1799	0.1556	0.03208	0.0162	0.12583	0.16189	0.16189	red
DES	-0.06715	0.00357	0.02234	0.04205	0.10765	0.02399	0.06087	0.1796	-0.1796	0.38284	0.96305	0.77178	0.58503	0.16108	0.75548	0.42904	0.01875	0.01875	salmon
GGT1	-0.02402	-0.04977	-0.07878	0.11474	-0.03675	0.08438	0.08892	0.02598	-0.02598	0.75512	0.51801	0.30576	0.13508	0.63326	0.27254	0.24744	0.73588	0.73588	magenta
FAM117A	-0.00044	-0.05675	-0.19862	-0.09959	0.15447	-0.24656	-0.27061	0.41341	-0.41341	0.99549	0.461	0.00921	0.19497	0.04367	0.00115	0.00034	1.91E-08	1.91E-08	brown
BMP4	0.06785	0.03362	0.11681	0.10707	-0.05736	0.21238	0.18774	-0.29577	0.29577	0.37791	0.66244	0.12813	0.16337	0.45619	0.00529	0.01394	8.58E-05	8.58E-05	yellow
PRSS3	0.11704	0.09843	0.12028	0.1168	-0.05206	0.19267	0.04269	-0.05599	0.05599	0.12738	0.20025	0.11712	0.12818	0.49891	0.01158	0.57932	0.46697	0.46697	magenta
TRAPPC3	-0.03982	-0.12908	-0.00495	-0.00496	-0.00255	-0.00065	0.01246	-0.05055	0.05055	0.60512	0.09245	0.94877	0.94868	0.97361	0.99328	0.87147	0.5114	0.5114	turquoise
XRCC5	-0.01024	-0.00402	-0.00224	0.00859	-0.1256	0.11824	0.14763	-0.0775	0.0775	0.89424	0.95836	0.97684	0.91126	0.10165	0.12351	0.05399	0.31366	0.31366	turquoise
PNMA1	-0.04232	-0.18403	-0.12664	-0.05444	0.01436	-0.16536	-0.10014	0.11302	-0.11302	0.5826	0.01597	0.09884	0.47944	0.85209	0.03067	0.19251	0.14106	0.14106	turquoise
SZT2	-0.10869	-0.18572	-0.01519	0.01421	-0.00814	0.01752	-0.07968	0.21178	-0.21178	0.15706	0.01502	0.84366	0.85368	0.9158	0.82008	0.30023	0.00542	0.00542	turquoise
SLC4A3	-0.02577	-0.1284	-0.18134	-0.03776	0.03698	-0.31724	-0.10243	0.09149	-0.09149	0.73799	0.0942	0.01762	0.62388	0.6311	2.36E-05	0.1825	0.23399	0.23399	grey
TUBB2A	-0.07819	-0.08523	-0.01299	0.01412	0.07854	0.24595	0.04418	-0.018	0.018	0.30937	0.26769	0.86606	0.85454	0.30721	0.00118	0.56609	0.81527	0.81527	grey
RELN	-0.09895	-0.1316	-0.22696	-0.04645	0.06919	-0.11862	-0.15722	0.45661	-0.45661	0.19787	0.08621	0.00283	0.54634	0.36851	0.12228	0.04001	3.45E-10	3.45E-10	grey
MRPS28	-0.0372	0.07637	-0.07133	0.0107	0.06607	-0.16364	-0.01778	0.01212	-0.01212	0.62905	0.32079	0.35385	0.88958	0.39054	0.03247	0.8175	0.87497	0.87497	grey
ZNF783	0.01382	-0.01507	0.00481	-0.01484	-0.03886	0.03483	0.1742	-0.04575	0.04575	0.85759	0.84495	0.95018	0.84723	0.61385	0.65106	0.02269	0.55237	0.55237	grey
POF1B	-0.03204	0.02965	0.12274	0.11892	-0.01507	0.31546	0.14501	-0.27575	0.27575	0.67737	0.70024	0.10974	0.12134	0.84488	2.64E-05	0.05844	0.00026	0.00026	yellow
BPTF	-0.02325	-0.199	-0.09883	-0.02811	-0.0278	-0.01933	-0.0594	0.33881	-0.33881	0.76281	0.00907	0.19844	0.71516	0.71817	0.80189	0.44026	5.82E-06	5.82E-06	turquoise
AGRP	0.01067	0.01325	0.08525	0.09177	-0.00611	0.07887	0.00866	-0.00938	0.00938	0.88979	0.86344	0.26756	0.23258	0.93679	0.30518	0.91052	0.90309	0.90309	grey
GEMIN6	-0.08986	-0.03538	-0.07588	0.03639	-0.08858	0.07861	0.21379	-0.23062	0.23062	0.24247	0.64591	0.32395	0.63654	0.2493	0.30678	0.00499	0.00241	0.00241	grey
IGSF8	0.1042	-0.03615	-0.05952	-0.04409	0.12759	-0.01579	0.04549	-0.36242	0.36242	0.17503	0.63876	0.43938	0.56691	0.0963	0.83755	0.55463	1.11E-06	1.11E-06	greenyellow
FCGR2A	-0.11995	-0.00255	0.09695	0.04773	-0.26218	0.18307	0.12254	0.34439	-0.34439	0.11813	0.97355	0.20717	0.53532	0.00053	0.01655	0.11034	3.98E-06	3.98E-06	blue
FOXQ1	-0.0126	0.07853	0.15805	0.0161	-0.03066	0.30907	0.2271	-0.41936	0.41936	0.87011	0.30729	0.03896	0.83449	0.69061	3.90E-05	0.00282	1.14E-08	1.14E-08	yellow
UBA7	0.01306	0.05256	0.09585	0.03337	-0.04078	0.22825	0.07536	-0.09952	0.09952	0.86536	0.49474	0.21235	0.66477	0.59645	0.00268	0.32726	0.19532	0.19532	tan
SDCCAG8	-0.05918	-0.09265	-0.08935	-0.06925	-0.00531	-0.00226	-0.01022	0.10161	-0.10161	0.44195	0.22811	0.24516	0.36814	0.945	0.97661	0.8945	0.18605	0.18605	turquoise
ACTA2	-0.12796	-0.03572	0.05148	0.03121	-0.1766	0.16307	0.05338	0.22775	-0.22775	0.09533	0.64273	0.50366	0.68529	0.02085	0.03309	0.48806	0.00274	0.00274	salmon
SYNPO2	-0.06601	-0.04646	-0.00533	0.03047	-0.07703	0.08663	-0.03736	0.45863	-0.45863	0.39102	0.54627	0.94487	0.69243	0.31666	0.25989	0.62759	2.82E-10	2.82E-10	salmon
ANGPT1	-0.13866	-0.06284	-0.02122	0.09044	-0.1228	0.13861	0.03171	0.54665	-0.54665	0.07051	0.41424	0.78294	0.23945	0.10958	0.07061	0.68051	1.04E-14	1.04E-14	blue
ADH1B	-0.05997	-0.00076	-0.05214	0.0823	-0.0087	0.06531	-0.11546	0.52691	-0.52691	0.43591	0.99213	0.49821	0.28457	0.91004	0.39603	0.13264	1.33E-13	1.33E-13	blue
SNAI2	-0.03912	-0.04901	0.02823	0.11596	-0.25724	0.06881	0.15583	0.10325	-0.10325	0.61145	0.52437	0.71401	0.13094	0.00068	0.37117	0.04183	0.17901	0.17901	black
LPL	-0.02549	-0.06323	0.01601	-0.03211	-0.00294	0.06276	-0.14742	0.49953	-0.49953	0.7407	0.41134	0.83539	0.67671	0.96953	0.41481	0.05434	3.52E-12	3.52E-12	blue
GCC1	0.00868	-0.09362	-0.03569	0.05428	-0.06506	-0.01905	0.03927	0.09609	-0.09609	0.91031	0.22323	0.64308	0.48076	0.39785	0.8047	0.61004	0.21124	0.21124	turquoise
FAM221A	0.00317	0.01369	0.00805	-0.0034	0.21176	0.11338	-0.02452	-0.19155	0.19155	0.96713	0.85898	0.91679	0.96483	0.00543	0.1398	0.75025	0.01208	0.01208	grey
RMDN1	-0.01956	0.03785	-0.00125	0.03457	-0.08714	-0.01058	0.10961	-0.00656	0.00656	0.79955	0.62308	0.98709	0.65347	0.25709	0.89077	0.15354	0.93217	0.93217	turquoise
GEMIN4	-0.08851	-0.01776	-0.06124	-0.05398	-0.00381	-0.00045	0.09192	0.19373	-0.19373	0.24965	0.81764	0.42622	0.48318	0.96058	0.99534	0.23179	0.01112	0.01112	turquoise
HES1	0.06283	-0.02484	0.07508	0.06059	0.07344	0.26397	0.24667	-0.38196	0.38196	0.41424	0.74706	0.32907	0.43116	0.33978	0.00049	0.00114	2.54E-07	2.54E-07	yellow
ENAM	-0.01741	-0.08713	-0.23564	-0.06012	0.13375	-0.20918	-0.17337	0.38665	-0.38665	0.82118	0.25717	0.00192	0.43474	0.08115	0.00604	0.02335	1.75E-07	1.75E-07	brown
POC1B	0.03045	0.02316	0.03452	0.0245	0.0555	0.17674	0.04172	-0.22815	0.22815	0.69254	0.76363	0.654	0.75042	0.47096	0.02075	0.58794	0.00269	0.00269	turquoise
HDDC2	-0.01384	0.04158	-0.0737	-0.0289	0.04492	-0.19131	-0.05223	0.17769	-0.17769	0.85744	0.58923	0.33805	0.7075	0.55961	0.01219	0.49748	0.02007	0.02007	grey
HDAC3	0.01146	-0.16647	-0.11121	-0.08856	-0.02273	-0.06814	0.0092	0.02182	-0.02182	0.88171	0.02955	0.14761	0.24938	0.76796	0.37587	0.90498	0.77693	0.77693	turquoise
ORMDL3	-0.00539	-0.1142	-0.03727	0.00137	0.14326	-0.05174	-0.07898	-0.06952	0.06952	0.94422	0.13694	0.62844	0.96988	0.06159	0.50156	0.3045	0.36623	0.36623	grey
SYT11	-0.08127	-0.1337	-0.16006	-0.17329	-0.14335	-0.13982	-0.14567	0.54885	-0.54885	0.29065	0.08126	0.03651	0.02341	0.06142	0.06816	0.0573	7.73E-15	7.73E-15	brown
PRIMA1	-0.07784	-0.0342	-0.1149	0.06569	-0.01485	-0.0952	-0.08592	0.40504	-0.40504	0.31154	0.65702	0.13455	0.39331	0.84713	0.21551	0.26381	3.90E-08	3.90E-08	salmon
ZNF154	-0.03979	-0.13664	-0.07838	0.04694	-0.06699	-0.02798	-0.05598	0.52602	-0.52602	0.60531	0.07475	0.30823	0.54207	0.38397	0.71637	0.46706	1.49E-13	1.49E-13	blue
RAB11FIP2	-0.05075	-0.06414	-0.05588	0.03044	-0.07565	0.01523	0.00057	0.44755	-0.44755	0.50976	0.40459	0.46785	0.6927	0.32541	0.84331	0.99406	8.40E-10	8.40E-10	turquoise
COPB2	-0.05041	-0.09239	-0.02515	-0.0082	-0.1113	0.11945	0.08484	0.01986	-0.01986	0.51262	0.22944	0.74404	0.91522	0.14725	0.11966	0.2699	0.79656	0.79656	turquoise
C22orf42	0.00602	-0.03538	-0.15316	-0.12117	0.10051	-0.33856	-0.19135	0.38665	-0.38665	0.9377	0.64591	0.04551	0.1144	0.19088	5.92E-06	0.01217	1.75E-07	1.75E-07	brown
LEMD3	-0.02716	-0.17829	-0.05962	0.06897	-0.00953	-0.06055	-0.09042	0.20222	-0.20222	0.72438	0.01964	0.4386	0.37004	0.90156	0.43146	0.23953	0.00799	0.00799	turquoise
MYH11	-0.11065	-0.04515	0.02372	0.04454	-0.01981	0.11521	0.0287	0.24223	-0.24223	0.14966	0.55758	0.75809	0.56295	0.79709	0.13349	0.70946	0.00141	0.00141	salmon
DOK7	-0.04353	0.07067	-0.0384	-0.03613	-0.02817	0.09093	0.12284	-0.28233	0.28233	0.57187	0.35838	0.61799	0.63897	0.71454	0.23691	0.10945	0.00018	0.00018	grey
ISCA2	0.02543	-0.04355	-0.12968	-0.06575	0.03333	-0.16267	0.02534	-0.27168	0.27168	0.74129	0.57164	0.09092	0.3929	0.66522	0.03352	0.74218	0.00033	0.00033	grey
ZNF280D	-0.02234	-0.04355	-0.10135	-0.01559	-0.01847	-0.01406	-0.03664	0.18324	-0.18324	0.77178	0.57169	0.18716	0.83965	0.81049	0.85518	0.63419	0.01644	0.01644	turquoise
CAD	0.00131	0.03299	-0.01407	0.06017	-0.13691	0.05037	0.22093	0.03591	-0.03591	0.98639	0.66844	0.85512	0.43436	0.07417	0.51292	0.00369	0.64098	0.64098	turquoise
HIST1H2BJ	-0.04201	-0.08462	0.13422	0.08131	-0.12895	0.09836	0.23114	-0.2802	0.2802	0.58539	0.27117	0.08008	0.29038	0.09277	0.2006	0.00235	0.00021	0.00021	grey
STAC	-0.04952	-0.09247	0.03885	0.08559	-0.0962	0.09022	0.11396	0.05081	-0.05081	0.52008	0.22902	0.61395	0.26566	0.21068	0.24058	0.13778	0.50929	0.50929	grey
ACSM1	-0.03016	-0.14194	-0.13029	-0.0906	0.09763	0.06913	-0.02904	-0.0794	0.0794	0.69533	0.06405	0.0894	0.23862	0.20397	0.36897	0.70614	0.30192	0.30192	grey
ADGRE3	-0.08269	-0.03857	0.0618	0.04427	-0.04117	0.12795	0.01456	0.27639	-0.27639	0.28227	0.61651	0.42196	0.56534	0.5929	0.09537	0.85004	0.00025	0.00025	blue
NUDT17	-0.05864	-0.07627	-0.05448	-0.04016	0.09775	-0.08445	-0.00791	-0.0307	0.0307	0.44615	0.32146	0.4791	0.602	0.20339	0.27211	0.91825	0.69015	0.69015	grey
ZWINT	-0.04205	0.08161	0.05594	-0.01102	-0.02501	0.04044	0.29282	-0.48694	0.48694	0.58499	0.28865	0.46741	0.88626	0.74543	0.59949	0.0001	1.45E-11	1.45E-11	pink
DOCK4	-0.07799	-0.14384	-0.00052	0.09637	-0.18064	0.00342	0.02795	0.52139	-0.52139	0.31062	0.06054	0.99466	0.20987	0.01806	0.96461	0.71667	2.64E-13	2.64E-13	blue
COG5	0.02469	-0.09374	0.08314	-0.01322	-0.10255	0.12421	0.06258	0.15373	-0.15373	0.74853	0.22264	0.27965	0.86376	0.18197	0.10552	0.41612	0.04469	0.04469	turquoise
BCAP31	0.05415	-0.12497	-0.03197	-0.06259	-0.00414	-0.08727	0.09048	-0.45332	0.45332	0.4818	0.10341	0.67808	0.41604	0.95709	0.25638	0.23923	4.78E-10	4.78E-10	grey
UBC	0.00028	-0.15179	0.01963	0.00848	-0.04367	0.10013	0.05645	-0.1907	0.1907	0.99715	0.0475	0.79881	0.91236	0.57059	0.19256	0.46331	0.01247	0.01247	turquoise
CDPF1	-0.06639	0.07154	-0.19402	-0.05404	0.01641	-0.22457	0.02945	0.0143	-0.0143	0.38829	0.35247	0.011	0.48271	0.83127	0.00315	0.70223	0.85274	0.85274	grey
H1FX	0.0152	-0.06819	-0.20045	-0.13536	0.17643	-0.14766	0.03247	-0.22043	0.22043	0.84359	0.37552	0.00857	0.07752	0.02098	0.05394	0.67335	0.00377	0.00377	green
FNIP1	0.03226	-0.22481	-0.09578	0.00157	-0.09171	-0.01819	-0.00596	0.18548	-0.18548	0.67534	0.00312	0.21272	0.98379	0.23285	0.81336	0.93829	0.01515	0.01515	turquoise
PCCB	-0.0584	0.13735	-0.23636	-0.15529	0.09123	-0.15714	0.09811	0.06664	-0.06664	0.448	0.07323	0.00186	0.04255	0.23533	0.04012	0.20174	0.38646	0.38646	grey
WASHC2C	0.02069	-0.11349	0.01773	0.01554	-0.13166	0.00921	0.05402	0.22922	-0.22922	0.78822	0.13943	0.81796	0.84012	0.08607	0.90485	0.48281	0.00256	0.00256	turquoise
DNAJC8	-0.07249	-0.14322	0.00939	0.01137	-0.13416	0.06091	0.00834	0.19773	-0.19773	0.34606	0.06165	0.90293	0.88269	0.08022	0.42868	0.91384	0.00953	0.00953	turquoise
P4HA3	-0.01293	-0.03691	0.09096	0.09531	-0.19702	0.13298	0.09001	0.27088	-0.27088	0.86669	0.6317	0.23672	0.21496	0.0098	0.08293	0.24167	0.00034	0.00034	black
GSTP1	0.03989	0.06679	0.08733	0.08203	-0.10128	0.26627	0.3121	-0.45861	0.45861	0.60442	0.38542	0.25605	0.28612	0.18746	0.00043	3.25E-05	2.83E-10	2.83E-10	yellow
ZNF285	-0.00346	-0.09008	-0.06432	-0.02479	-0.06775	-0.00524	-0.04037	0.07674	-0.07674	0.96417	0.2413	0.40326	0.74759	0.37859	0.94577	0.60011	0.31844	0.31844	grey
ZNF524	0.10222	0.14221	-0.11909	-0.11213	0.14271	0.00143	0.09155	-0.42934	0.42934	0.1834	0.06354	0.12082	0.14425	0.06259	0.98515	0.23368	4.65E-09	4.65E-09	green
SDC2	-0.10585	-0.04855	0.00494	0.03172	-0.15314	-0.08964	-0.12588	0.52339	-0.52339	0.16825	0.52833	0.94884	0.68041	0.04553	0.24362	0.1009	2.06E-13	2.06E-13	blue
TNFRSF1B	-0.09855	-0.02152	0.08333	0.13049	-0.20515	0.22889	0.14513	0.30179	-0.30179	0.19971	0.77991	0.27853	0.08892	0.00711	0.0026	0.05824	6.04E-05	6.04E-05	blue
NAXE	0.0909	0.02202	-0.12503	-0.11804	0.14634	-0.22073	0.05166	-0.2831	0.2831	0.23707	0.77495	0.10324	0.12413	0.05614	0.00372	0.50218	0.00018	0.00018	greenyellow
BMT2	-0.04077	-0.22535	0.01819	0.1047	-0.08413	-0.01808	-0.02479	0.25119	-0.25119	0.59648	0.00304	0.8133	0.17292	0.27396	0.81446	0.74753	0.00092	0.00092	turquoise
NSUN6	0.04091	-0.11478	-0.15174	-0.06837	0.19837	-0.08092	-0.10238	0.05447	-0.05447	0.59521	0.13496	0.04756	0.37426	0.0093	0.29272	0.18271	0.47922	0.47922	red
LIN52	-0.016	-0.12088	-0.11895	-0.05986	-0.01447	-0.13867	-0.13749	0.33156	-0.33156	0.83547	0.11527	0.12125	0.43676	0.85103	0.07049	0.07294	9.43E-06	9.43E-06	turquoise
KYNU	0.04484	0.03313	0.02857	0.03991	-0.16431	0.12967	0.27806	-0.12929	0.12929	0.56029	0.6671	0.7107	0.60429	0.03175	0.09096	0.00023	0.09191	0.09191	black
TMPRSS6	-0.0341	-0.10209	-0.27088	-0.16338	0.20576	-0.32249	-0.26105	0.31145	-0.31145	0.6579	0.18397	0.00034	0.03274	0.00694	1.70E-05	0.00056	3.38E-05	3.38E-05	brown
FBXL4	-0.04019	-0.08747	-0.0733	0.00565	-0.01996	-0.05389	-0.07444	0.34932	-0.34932	0.60177	0.25526	0.34073	0.94149	0.79549	0.48391	0.33324	2.83E-06	2.83E-06	turquoise
TIMM23B	-0.08621	-0.09496	0.07152	0.03172	-0.05497	0.02053	-0.00737	0.10766	-0.10766	0.26222	0.21668	0.35261	0.68049	0.47521	0.78984	0.92376	0.16103	0.16103	turquoise
PMF1-BGLAP	0.05026	-0.01877	-0.19768	-0.0591	0.21803	-0.16339	-0.04008	0.01259	-0.01259	0.51389	0.80748	0.00955	0.44257	0.00417	0.03274	0.60274	0.87016	0.87016	green
MSLN	0.03422	0.1328	0.10504	0.08572	-0.00164	0.25466	0.26959	-0.35142	0.35142	0.65684	0.08337	0.17154	0.26498	0.98298	0.00078	0.00036	2.44E-06	2.44E-06	yellow
CEP135	-0.09136	-0.07058	0.05108	0.04927	-0.05905	0.10635	0.07668	0.22895	-0.22895	0.23466	0.35895	0.50705	0.52223	0.44294	0.16624	0.31882	0.00259	0.00259	turquoise
DHFR	-0.10323	0.0264	0.02371	-0.01168	0.00823	0.07588	0.18309	-0.13529	0.13529	0.17907	0.73179	0.75817	0.87951	0.91493	0.32394	0.01653	0.07769	0.07769	pink
ACSL5	0.0077	0.06989	0.15842	0.08623	-0.03986	0.29325	0.2063	-0.36499	0.36499	0.92036	0.36373	0.0385	0.26213	0.60476	9.92E-05	0.00679	9.19E-07	9.19E-07	yellow
GPR27	-0.04129	-0.09969	-0.22974	-0.12063	0.02796	-0.15974	-0.19228	0.33943	-0.33943	0.59179	0.19454	0.0025	0.11604	0.71656	0.03688	0.01175	5.58E-06	5.58E-06	brown
CITED1	0.11387	-0.0287	-0.01103	0.00825	-0.04539	-0.06355	-0.06874	-0.20724	0.20724	0.13808	0.70938	0.88618	0.91467	0.55556	0.4089	0.37167	0.00653	0.00653	grey
MRS2	-0.08976	-0.01	-0.04115	-0.01156	0.12144	-0.10946	-0.02369	0.2121	-0.2121	0.24301	0.8967	0.59304	0.88068	0.1136	0.15411	0.75839	0.00535	0.00535	turquoise
COL9A1	-0.02993	-0.17769	-0.02126	0.01792	0.05142	0.13881	-0.00698	-0.06876	0.06876	0.69755	0.02007	0.78255	0.81605	0.50422	0.0702	0.92775	0.37155	0.37155	grey
NR1H2	0.04051	0.02242	-0.05928	-0.02944	0.05395	0.09616	0.09817	-0.23885	0.23885	0.59882	0.77105	0.44118	0.70228	0.4834	0.21089	0.20147	0.00165	0.00165	green
SELENOM	-0.07727	0.03657	-0.07516	-0.01159	0.04196	-0.00674	0.04845	0.08124	-0.08124	0.31511	0.63487	0.32858	0.88039	0.58583	0.93031	0.52919	0.29081	0.29081	grey
RBP4	0.0739	0.05116	-0.00031	0.02953	0.00077	0.14574	0.01393	0.07694	-0.07694	0.33675	0.50637	0.99674	0.70145	0.99198	0.05718	0.85646	0.31723	0.31723	grey
NTMT1	-0.00945	0.00944	0.02211	-0.01138	-0.09893	0.16526	0.15861	-0.23868	0.23868	0.90238	0.90245	0.77407	0.88253	0.19797	0.03077	0.03826	0.00167	0.00167	grey
CLCN1	0.05575	0.08671	0.13373	-0.00747	0.10134	0.15034	0.14683	-0.30506	0.30506	0.46888	0.25944	0.0812	0.92274	0.18722	0.04968	0.05532	4.97E-05	4.97E-05	grey
LYSMD1	0.08702	-0.13696	-0.14017	-0.10902	0.02521	-0.12453	0.0374	-0.00624	0.00624	0.25776	0.07406	0.06747	0.15577	0.74346	0.10463	0.62717	0.93544	0.93544	turquoise
ZNF808	0.01236	-0.08799	-0.05614	-0.00629	0.00922	0.07992	-0.03965	-0.00063	0.00063	0.87252	0.25244	0.46582	0.93496	0.90478	0.29875	0.60666	0.9935	0.9935	red
NELFCD	0.06634	-0.09059	-0.07911	-0.02558	0.10451	-0.1582	0.03315	-0.18925	0.18925	0.38861	0.23829	0.30369	0.73983	0.17372	0.03877	0.66684	0.01317	0.01317	grey
HMGB1	0.02042	-0.0106	-0.05293	-0.03989	-0.04702	-0.00022	0.03741	-0.0666	0.0666	0.79096	0.8906	0.49175	0.60445	0.5414	0.99776	0.62711	0.38678	0.38678	turquoise
CCNF	-0.00702	0.06121	0.04792	0.00879	-0.03763	0.04784	0.27859	-0.36102	0.36102	0.92736	0.42646	0.5337	0.90917	0.62511	0.53437	0.00022	1.23E-06	1.23E-06	pink
THSD1	-0.16937	-0.09371	-0.07365	0.02338	-0.0466	-0.09178	-0.05375	0.4361	-0.4361	0.02679	0.22278	0.33839	0.76148	0.54505	0.23254	0.48502	2.49E-09	2.49E-09	blue
CD82	-0.04647	-0.04411	0.05899	-0.00048	-0.03846	0.04868	0.19159	-0.21797	0.21797	0.54615	0.56676	0.44347	0.99501	0.6175	0.52722	0.01207	0.00418	0.00418	turquoise
KDM5C	-0.43384	-0.19407	-0.07394	-0.03133	0.06073	-0.03925	-0.05579	0.11322	-0.11322	3.07E-09	0.01098	0.3365	0.68417	0.43007	0.61026	0.46863	0.14035	0.14035	turquoise
ZSCAN30	-0.06447	-0.25772	-0.15496	-0.02312	0.12709	-0.17263	-0.13791	0.36405	-0.36405	0.40215	0.00067	0.043	0.76404	0.09764	0.02395	0.07205	9.85E-07	9.85E-07	turquoise

FAM111B	-0.14324	0.12137	0.05105	0.0042	-0.06627	0.12591	0.27483	-0.26208	0.26208	0.06162	0.11381	0.50728	0.95654	0.38917	0.1008	0.00028	0.00053	0.00053	pink
UBQLN1	0.01334	-0.14516	-0.07617	-0.02255	-0.13051	-0.01356	0.00829	0.08603	-0.08603	0.86247	0.05817	0.3221	0.7697	0.08888	0.86029	0.91434	0.26323	0.26323	turquoise
FNDC10	-0.02973	-0.01936	0.00719	0.03705	-0.04989	0.00641	0.13007	-0.00945	0.00945	0.69953	0.80155	0.92564	0.63041	0.51701	0.93367	0.08997	0.90241	0.90241	grey
ZC2HC1A	-0.02513	-0.08468	0.01148	0.06127	-0.17226	-0.00651	-0.00511	0.12116	-0.12116	0.74425	0.27082	0.88152	0.42598	0.02426	0.93267	0.94713	0.11442	0.11442	turquoise
GNPTG	-0.03078	-0.05036	-0.01273	-0.01184	0.19126	-0.1759	-0.05718	0.06559	-0.06559	0.68941	0.51302	0.86872	0.87789	0.01221	0.02138	0.45758	0.39404	0.39404	grey
TCP11L1	-0.0718	-0.18243	-0.03292	0.00164	-0.17417	0.0222	0.06271	0.34523	-0.34523	0.35069	0.01693	0.66905	0.98303	0.02271	0.77322	0.41518	3.76E-06	3.76E-06	blue
ACTL6B	0.01289	-0.11776	-0.27153	-0.17316	0.14924	-0.3744	-0.25143	0.35574	-0.35574	0.86711	0.12503	0.00033	0.02352	0.0514	4.54E-07	0.00091	1.80E-06	1.80E-06	brown
DEPDC1B	-0.17565	0.03439	0.01158	-0.02238	0.01602	0.09351	0.09739	-0.35035	0.35035	0.02156	0.65519	0.88051	0.77138	0.83522	0.22378	3.97E-05	2.63E-06	2.63E-06	pink
GRID2IP	0.00175	-0.06133	-0.01027	0.05152	0.06928	0.11696	0.09545	-0.39341	0.39341	0.9819	0.42554	0.8939	0.50339	0.36791	0.12763	0.21428	1.02E-07	1.02E-07	grey
ATXN2L	0.05923	-0.07341	0.05916	0.00952	0.00781	0.06003	0.08896	-0.17339	0.17339	0.44158	0.34001	0.44214	0.9016	0.9192	0.43542	0.24725	0.02334	0.02334	turquoise
VPS35	-0.02067	-0.07891	0.06895	0.01683	-0.14566	0.19255	0.12929	-0.05292	0.05292	0.78841	0.30491	0.3702	0.82708	0.05731	0.01163	0.09192	0.49178	0.49178	turquoise
CEP68	-0.14943	-0.06244	-0.08022	0.07016	-0.15222	0.06853	-0.05132	0.49945	-0.49945	0.05109	0.41719	0.29693	0.36183	0.04687	0.37312	0.50506	3.56E-12	3.56E-12	blue
GJB2	-0.04259	0.05554	0.08957	0.04671	-0.15915	0.19457	0.2414	-0.207	0.207	0.58019	0.47056	0.24401	0.54411	0.03761	0.01077	0.00147	0.0066	0.0066	black
TCF3	-0.01001	0.05525	0.03563	0.01401	0.01073	0.1324	0.17255	-0.22858	0.22858	0.89657	0.47291	0.64363	0.85564	0.88919	0.08429	0.02402	0.00264	0.00264	grey
TLK1	0.04528	-0.09543	0.00116	0.05583	-0.10354	0.0497	0.00233	0.12526	-0.12526	0.55644	0.2144	0.98803	0.46831	0.17777	0.51854	0.97586	0.1026	0.1026	turquoise
DHPS	0.06897	-0.06804	-0.19062	-0.1608	0.20049	-0.26024	-0.09674	-0.16712	0.16712	0.3701	0.3766	0.01251	0.03564	0.00856	0.00059	0.20812	0.02891	0.02891	green
EVA1B	-0.03545	-0.1008	-0.01508	0.01386	0.01555	-0.01974	0.0888	-0.11491	0.11491	0.6453	0.18959	0.84477	0.85718	0.84004	0.79771	0.24811	0.13451	0.13451	green
PPARD	-0.0525	-0.12299	0.08189	0.08546	-0.10093	0.14581	0.07102	-0.052	0.052	0.49527	0.10901	0.28694	0.26641	0.18899	0.05705	0.35596	0.49942	0.49942	turquoise
SCARF1	-0.19905	-0.1277	-0.03102	0.04807	0.02187	-0.03183	-0.04882	0.43642	-0.43642	0.00905	0.09601	0.68708	0.53239	0.77644	0.67944	0.52601	2.42E-09	2.42E-09	blue
CYP4F3	0.03268	-0.06828	-0.02054	0.05491	0.19877	-0.15103	-0.02612	-0.07981	0.07981	0.67136	0.37486	0.78978	0.47567	0.00916	0.04863	0.73453	0.29942	0.29942	grey
ZCRB1	0.0425	-0.19877	-0.09623	-0.03187	0.04597	-0.11552	-0.08859	0.01625	-0.01625	0.58099	0.00916	0.21053	0.67897	0.55044	0.13242	0.24921	0.83296	0.83296	turquoise
PIH1D1	0.05788	0.04446	-0.14487	-0.10701	0.09653	-0.05868	0.09738	-0.38331	0.38331	0.4521	0.56364	0.05869	0.1636	0.2091	0.44584	0.20514	2.28E-07	2.28E-07	grey
KDM8	-0.06562	-0.05557	-0.19288	-0.07422	0.062	-0.09629	-0.18753	0.4109	-0.4109	0.3938	0.47039	0.01149	0.33468	0.42047	0.21027	0.01405	2.37E-08	2.37E-08	turquoise
SMARCD2	0.04672	-0.07758	-0.02812	-0.04702	-0.01507	0.09909	0.17428	-0.17835	0.17835	0.54399	0.31317	0.71507	0.54143	0.8449	0.19723	0.02262	0.0196	0.0196	grey
ZNF276	-0.01617	-0.04245	-0.01583	-0.05267	0.11242	0.07348	0.04081	-0.16984	0.16984	0.83375	0.58148	0.83719	0.49384	0.1432	0.3395	0.5961	0.02637	0.02637	red
ING4	-0.01191	-0.1263	-0.08899	-0.06964	0.04381	-0.08101	-0.04104	-0.06819	0.06819	0.8771	0.09975	0.24708	0.3654	0.56943	0.29222	0.59402	0.3755	0.3755	grey
CCL19	-0.11043	-0.01748	-0.02193	0.00598	-0.06691	0.10049	-0.02991	0.36131	-0.36131	0.15049	0.82044	0.77586	0.93817	0.38458	0.19096	0.69775	1.20E-06	1.20E-06	blue
MAVS	-0.03366	-0.09483	-0.16005	-0.07341	-0.02848	-0.20923	0.00162	0.20854	-0.20854	0.66205	0.21728	0.03652	0.33997	0.71159	0.00602	0.98317	0.0062	0.0062	turquoise
HPS3	-0.019	-0.15498	-0.01385	-0.00919	-0.12455	0.11889	0.09899	-0.01029	0.01029	0.80514	0.04297	0.85734	0.90506	0.10458	0.12144	0.1977	0.89373	0.89373	turquoise
RBBP8	-0.01053	-0.04848	0.08078	0.12504	-0.22194	0.26128	0.15751	-0.14641	0.14641	0.89128	0.52889	0.29355	0.1032	0.00353	0.00056	0.03964	0.05603	0.05603	turquoise
CALCOCO	-0.00188	-0.15084	-0.09815	-0.05452	0.07107	-0.10531	-0.08871	0.14903	-0.14903	0.98051	0.04892	0.20153	0.47882	0.35563	0.17045	0.24855	0.05172	0.05172	turquoise
FBP2	-0.07984	0.05476	0.19169	0.07985	0.00188	0.32365	-0.01643	-0.09214	0.09214	0.29926	0.47684	0.01202	0.29917	0.98053	1.57E-05	0.83108	0.23067	0.23067	grey
TRIM45	-0.01141	-0.10952	-0.05484	0.0766	0.07473	0.04311	0.06284	-0.19148	0.19148	0.88223	0.15387	0.47625	0.31936	0.33135	0.5756	0.41424	0.01211	0.01211	grey
FARSB	0.04863	0.14032	0.02964	0.01764	0.03422	0.04725	0.17115	-0.33922	0.33922	0.5276	0.06717	0.70033	0.81889	0.65685	0.53941	0.02521	5.66E-06	5.66E-06	turquoise
EPHA4	-0.0045	-0.10913	0.01876	0.0167	-0.11462	0.08799	-0.06873	-0.02996	0.02996	0.95346	0.15535	0.80763	0.82837	0.13549	0.25247	0.37171	0.69729	0.69729	turquoise
CIPC	0.04171	-0.13126	-0.07096	0.06206	0.01081	-0.00931	-0.05681	0.12816	-0.12816	0.58802	0.08702	0.35636	0.42002	0.88844	0.90386	0.46046	0.09481	0.09481	turquoise
EIF2AK1	0.03646	-0.0888	0.01944	-0.00122	0.00884	0.01453	0.08356	-0.32418	0.32418	0.63593	0.24808	0.80077	0.9874	0.9086	0.85034	0.27722	1.52E-05	1.52E-05	turquoise
TCEAL5	0.03883	-0.17932	-0.22721	-0.14985	0.13685	-0.30537	-0.26671	0.47386	-0.47386	0.61408	0.01894	0.0028	0.05044	0.07429	4.88E-05	0.00042	5.90E-11	5.90E-11	brown
CORO1B	0.08836	0.07216	0.02792	-0.01865	0.07671	0.13017	0.20081	-0.43759	0.43759	0.25044	0.34832	0.71702	0.80874	0.31863	0.08971	0.00845	2.17E-09	2.17E-09	yellow
STIP1	0.11484	-0.16084	-0.02009	-0.02173	-0.10846	-0.01749	0.16281	-0.18661	0.18661	0.13473	0.0356	0.79422	0.77785	0.15792	0.82039	0.03337	0.01453	0.01453	turquoise
ZNF831	-0.07012	-0.04719	0.10142	0.08534	-0.08388	-0.0998	-0.16576	0.50725	-0.50725	0.36211	0.53996	0.18687	0.26708	0.27539	0.19404	0.03026	1.44E-12	1.44E-12	blue
LCOR	0.03573	-0.18086	0.02963	0.01967	0.00475	0.06542	0.03701	-0.12129	0.12129	0.64272	0.01792	0.70041	0.79844	0.9508	0.39526	0.63081	0.11403	0.11403	turquoise
MRE11	-0.01106	-0.0416	-0.01927	-0.00157	-0.14917	0.09151	0.19197	0.02818	-0.02818	0.88582	0.58904	0.80249	0.98374	0.0515	0.23391	0.01189	0.71446	0.71446	turquoise
BICC1	-0.09312	-0.12778	-0.0041	0.15098	-0.2637	0.17737	0.09042	0.24033	-0.24033	0.22572	0.0958	0.9576	0.04871	0.00049	0.02029	0.23954	0.00154	0.00154	blue
CYP4F12	0.05393	0.03623	0.12479	0.11582	0.17957	0.1093	0.19051	-0.33556	0.33556	0.48355	0.638	0.1039	0.13141	0.01877	0.15473	0.01257	7.24E-06	7.24E-06	grey
CASP8	-0.01773	-0.00476	0.10639	0.12849	-0.14031	0.27712	0.21943	-0.21338	0.21338	0.818	0.95068	0.16605	0.09397	0.06718	0.00024	0.00393	0.00508	0.00508	turquoise
KARS	0.02093	0.03578	0.01624	-0.03652	-0.13057	0.09775	0.20491	-0.3584	0.3584	0.78586	0.64218	0.83304	0.63534	0.08871	0.20342	0.00718	1.49E-06	1.49E-06	turquoise
RCOR2	0.13263	0.02197	-0.13797	-0.12149	0.13916	-0.10145	0.16509	-0.32535	0.32535	0.08375	0.77545	0.07193	0.11345	0.06949	0.18673	0.03095	1.41E-05	1.41E-05	grey
TGOLN2	0.05125	-0.08693	-0.05146	-0.06248	-0.13377	0.0987	0.05921	0.10804	-0.10804	0.50556	0.25823	0.50386	0.41685	0.08112	0.19902	0.44176	0.15957	0.15957	turquoise
DIRAS2	0.04036	-0.15487	-0.17848	-0.0314	0.11793	-0.18401	-0.17041	0.25439	-0.25439	0.60021	0.04312	0.01951	0.68349	0.12448	0.01599	0.02586	0.00079	0.00079	brown
PSRC1	-0.01359	0.02379	0.04288	-0.0081	0.00709	0.01649	0.23805	-0.35399	0.35399	0.85996	0.7574	0.5776	0.91623	0.92672	0.83053	0.00172	2.04E-06	2.04E-06	pink
FUT10	-0.08248	-0.07541	0.04261	0.05419	-0.02787	0.17321	0.09204	0.09024	-0.09024	0.28349	0.32695	0.58	0.48145	0.71743	0.02348	0.23121	0.24047	0.24047	turquoise
CYGB	-0.04767	-0.13472	-0.04458	0.01787	-0.13991	0.03868	-0.09014	0.32932	-0.32932	0.53578	0.07896	0.56259	0.81658	0.06799	0.6155	0.241	1.09E-05	1.09E-05	blue
CTLA4	-0.13814	-0.02047	0.07052	0.06636	-0.15703	0.11857	0.07412	0.34934	-0.34934	0.07157	0.79047	0.35941	0.38848	0.04026	0.12244	0.33532	2.83E-06	2.83E-06	blue
PHC3	-0.01554	-0.14326	-0.03005	0.04796	-0.18066	0.15625	0.10367	0.17259	-0.17259	0.84009	0.06158	0.6964	0.53334	0.01805	0.04127	0.1772	0.02399	0.02399	turquoise
P4HA2	0.07739	-0.17136	0.02491	0.09214	-0.02319	-0.12837	-0.04185	-0.05921	0.05921	0.31437	0.02502	0.74643	0.2307	0.76339	0.09428	0.5868	0.44177	0.44177	grey
HIVEP1	-0.05755	-0.11894	0.00569	0.06159	-0.14747	0.15848	-0.00665	0.3536	-0.3536	0.4547	0.12127	0.94116	0.42358	0.05425	0.03842	0.93122	2.09E-06	2.09E-06	turquoise
DAPK2	-0.01296	-0.2291	0.08513	0.13907	0.05988	-0.01846	-0.00158	0.03647	-0.03647	0.86644	0.00258	0.26827	0.06966	0.43657	0.81059	0.98359	0.63577	0.63577	grey
AQP1	-0.08904	0.01313	-0.09108	0.14567	-0.10104	0.1175	-0.05317	0.34254	-0.34254	0.24684	0.86469	0.2361	0.05729	0.18851	0.1259	0.4898	4.52E-06	4.52E-06	magenta
ANKRD37	-0.02416	-0.19356	-0.10118	0.05091	0.21538	-0.08022	-0.09678	-0.16348	0.16348	0.7538	0.01119	0.1879	0.50841	0.00467	0.29692	0.20793	0.03264	0.03264	grey
PPP1R11	0.09977	-0.02853	0.00309	-0.02671	0.00011	0.12245	0.04547	-0.26607	0.26607	0.19418	0.71106	0.96797	0.72877	0.99889	0.11061	0.55485	0.00044	0.00044	turquoise
STK10	-0.02963	-0.06912	0.08095	0.00659	-0.1375	0.07452	0.0065	0.15926	-0.15926	0.70041	0.36899	0.29256	0.93178	0.07292	0.33272	0.93278	0.03747	0.03747	turquoise
SLC16A7	0.03631	-0.15532	-0.05149	0.12465	-0.11464	0.0682	-0.00105	0.19041	-0.19041	0.63726	0.04251	0.50362	0.10429	0.13544	0.37541	0.98909	0.01261	0.01261	grey
CASC3	0.0048	-0.15999	-0.12821	-0.05378	-0.00792	-0.04288	-0.04309	0.25017	-0.25017	0.95034	0.03659	0.0947	0.48482	0.91809	0.57757	0.57579	0.00097	0.00097	turquoise
NPHP1	0.02893	-0.14056	-0.23315	-0.07026	0.08012	-0.21827	-0.12641	0.19083	-0.19083	0.7072	0.06671	0.00215	0.36117	0.29758	0.00413	0.09945	0.01241	0.01241	turquoise
GPATCH2	0.03888	-0.12117	-0.08568	-0.03015	-0.0185	0.11762	0.11067	-0.06479	0.06479	0.61365	0.11439	0.26517	0.69547	0.81017	0.12549	0.14958	0.39983	0.39983	turquoise
SLC44A4	0.10537	0.1062	0.1038	0.03241	0.08487	0.26412	0.05409	-0.32491	0.32491	0.1702	0.16681	0.17668	0.67385	0.26974	0.00048	0.48229	1.45E-05	1.45E-05	yellow
TMX1	-0.05454	-0.07687	0.03005	0.10915	-0.07498	-0.0078	0.02975	0.10697	-0.10697	0.47866	0.31766	0.69643	0.1553	0.32974	0.91936	0.69931	0.16377	0.16377	turquoise
ZNF239	0.00601	0.04737	0.07979	0.05028	-0.08704	0.19564	0.26962	-0.11314	0.11314	0.93779	0.53842	0.29956	0.5137	0.25764	0.01033	0.00036	0.14066	0.14066	turquoise
BICD2	-0.06376	-0.16068	-0.04336	0.05062	-0.21666	-0.05503	0.04596	0.37666	-0.37666	0.40742	0.03578	0.57335	0.51083	0.00442	0.47467	0.55058	3.82E-07	3.82E-07	blue
MBD4	-0.04677	-0.06	-0.06699	-0.0752	-0.03621	0.00855	0.00791	-0.00198	0.00198	0.54352	0.43562	0.38402	0.32832	0.63823	0.91166	0.9182	0.97951	0.97951	turquoise
CEBPZ	-0.03241	-0.06911	0.01664	0.01164	-0.09133	0.14582	0.13041	0.03102	-0.03102	0.67389	0.36908	0.82899	0.8799	0.23483	0.05704	0.08911	0.68709	0.68709	turquoise
PAX9	0.07903	-0.03413	0.00531	0.0795	-0.06711	0.01518	0.12141	-0.31055	0.31055	0.30422	0.65762	0.94502	0.3013	0.38316	0.84377	0.11369	3.57E-05	3.57E-05	grey
ATG101	0.1128	-0.17162	-0.10228	-0.0429	0.113	-0.12016	-0.07525	-0.0468	0.0468	0.14186	0.0248	0.18312	0.57742	0.14114	0.11748	0.32799	0.54333	0.54333	grey
SLC25A25	0.01697	-0.08383	-0.03004	-0.03332	0.05155	0.06513	-0.14173	0.12737	-0.12737	0.82562	0.27566	0.69649	0.66524	0.5031	0.39737	0.06444	0.0969	0.0969	grey
AK6	-0.03569	-0.0734	-0.14993	-0.02019	0.06689	-0.17305	-0.09199	0.09922	-0.09922	0.64307	0.34005	0.05031	0.79322	0.3847	0.02361	0.23146	0.19666	0.19666	turquoise
ETFRF1	-0.03669	-0.06277	-0.11216	-0.04637	0.0677	-0.09158	-0.24676	0.22845	-0.22845	0.63381	0.41469	0.14415	0.54702	0.37899	0.23356	0.00114	0.00265	0.00265	turquoise
ZBTB1	-0.0616	-0.14703	0.04763	0.07836	-0.04961	0.12729	-0.03655	0.13546	-0.13546	0.42353	0.05498	0.53615	0.30832	0.51932	0.09711	0.63503	0.07731	0.07731	turquoise
ELOA	-0.05896	-0.17141	-0.05996	0.00872	-0.08344	-0.00587	-0.1184	0.31406	-0.31406	0.44364	0.02498	0.436	0.90991	0.27793	0.93923	0.12299	2.88E-05	2.88E-05	turquoise
MRPS9	0.07101	0.12967	-0.10807	-0.06238	-0.07787	-0.06271	0.13296	-0.13296	0.13296	0.35605	0.09096	0.15944	0.41766	0.31134	0.41514	0.08298	0.08299	0.08299	turquoise
POLD4	0.05847	0.04134	0.0911	0.03698	0.05558	0.19562	0.1622	-0.50662	0.50662	0.44747	0.59139	0.236	0.63111	0.47027	0.01034	0.03404	1.55E-12	1.55E-12	yellow
NFATC1	-0.16154	-0.12343	-0.05107	0.03034	-0.07616	0.0598	-0.13543	0.40567	-0.40567	0.03479	0.10776	0.50706	0.69367	0.32213	0.43715	0.07737	3.70E-08	3.70E-08	blue
TRMT61B	-0.03324	-0.04241	-0.03134	0.01224	-0.07655	0.06443	0.05327	0.09804	-0.09804	0.66604	0.58177	0.68409	0.87379	0.31966	0.40249	0.48894	0.20204	0.20204	turquoise
ZNF706	0.02786	0.0256	-0.00585	-0.05201	0.07103	0.01356	0.0509	-0.30592	0.30592	0.7176	0.73958	0.93949	0.49934	0.3559	0.86026	0.50849	4.72E-05	4.72E-05	turquoise
PRDM5	-0.02181	-0.0638	-0.14159	-0.01423	0.04821	0.04868	0.05979	0.37494	-0.37494	0.77709	0.40713	0.06471	0.85346	0.53123	0.5272	0.43725	4.36E-07	4.36E-07	turquoise
CLIC4	-0.11096	-0.0623	0.07856	0.10649	-0.24191	0.18018	0.05317	0.32032	-0.32032	0.14852	0.41826	0.30708	0.16566	0.00143	0.01836	0.48976	1.95E-05	1.95E-05	blue
HINT1	0.09266	-0.01476	-0.18085	-0.10568	0.14322	-0.25014	-0.06842	-0.1473	0.1473	0.22806	0.84802	0.01793	0.16893	0.06166	0.00097	0.37394	0.05453	0.05453	grey
FCRL3	-0.09849	0.01041	0.03402	0.01551	-0.10468	0.05365	-0.07456	0.38205	-0.38205	0.19998	0.89248	0.65869	0.84047	0.17303	0.48585	0.33246	2.52E-07	2.52E-07	blue
UCN	0.04668	-0.06331	-0.09797	-0.04276	0.17472	-0.03529	0.07349	-0.36399	0.36399	0.54433	0.4107	0.20237	0.57866	0.02228	0.6468	0.33945	9.89E-07	9.89E-07	red
SYTL5	0.06187	-0.05641	0.0541	-0.02312	-0.04353	0.16051	0.15979	-0.27026	0.27026	0.42143	0.46368	0.48222	0.76408	0.57189	0.03598	0.03683	0.00035	0.00035	grey
TCEAL9	-0.02281	-0.16813	-0.0219	0.0264	-0.08237	0.05947	0.04094	0.0676	-0.0676	0.76716	0.02794	0.77614	0.73175	0.28417	0.43974	0.59495	0.3797	0.3797	turquoise
PHF23	-0.06101	-0.20258	-0.06559	-0.0173	-0.06592	-0.07345	0.00998	0.21277	-0.21277	0.42796	0.00788	0.39402	0.82234	0.39163	0.33973	0.89689	0.00521	0.00521	grey
HAUS2	-0.07709	-0.12796	0.00148	0.00608	-0.19502	0.05224	0.03732	0.04988	-0.04988	0.31626	0.09535	0.98468	0.9371	0.01059	0.49737	0.62798	0.5171	0.5171	turquoise
RPUSD1	0.02148	0.09996	-0.06344	-0.06696	0.10102	-0.05871	0.15387	-0.30997	0.30997	0.78032	0.19331	0.40972	0.38424	0.18864	0.4456	0.0445	3.70E-05	3.70E-05	green
NOSTRIN	0.0269	0.04673	-0.01861	0.10341	0.10236	0.14169	0.00621	-0.00588	0.00588	0.72692	0.54388	0.80909	0.17832	0.1828	0.06452	0.93571	0.93915	0.93915	grey
ABHD8	-0.02399	-0.15077	-0.24148	-0.09282	0.12063	-0.22122	-0.08924	0.1017	-0.1017	0.75547	0.04903	0.00146	0.22727	0.11605	0.00364	0.24576	0.18564	0.18564	green
SLAIN1	0.0174	-0.12407	-0.09749	-0.06707	0.1324	0.00439	-0.23857	0.12143	-0.12143	0.82133	0.10593	0.20462	0.38346	0.0843	0.95453	0.00168	0.11363	0.11363	grey
TMEM131	0.02713	-0.06858	0.02628	0.04452	-0.07345	0.07437	0.04136	0.17493	-0.17493	0.72467	0.37276	0.73292	0.56312	0.3397	0.33368	0.59117	0.02212	0.02212	turquoise
PDHA1	-0.00758	-0.01521	-0.09415	-0.01866	0.00845	-0.05995	0.11075	-0.18103	0.18103	0.92157	0.8435	0.22062	0.80856	0.91264	0.43602	0.14928	0.01781	0.01781	grey
ZMAT1	-0.11401	-0.16869	-0.18681	-0.05037	0.12752	-0.13414	-0.20751	0.35704	-0.35704	0.1376	0.02742	0.01442	0.51293	0.0965	0.08025	0.00646	1.64E-06	1.64E-06	brown
ATP7B	-0.06064	-0.15231	0.01979	0.0235	0.1179	0.06049	-0.04824	0.00301	-0.00301	0.43074	0.04674	0.79727	0.76032	0.1246	0.43188	0.53097	0.96879	0.96879	grey
PELP1	0.02591	-0.06696	-0.16664	-0.16162	0.12753	-0.28904	-0.07053	0.11606	-0.11606	0.73655	0.38421	0.02938	0.03469	0.09647	0.00013	0.35932	0.13062	0.13062	grey
XRCC2	-0.11968	0.11238	0.02012	-0.01968	-0.05245	-0.00962	0.31465	-0.27389	0.27389	0.11895	0.14337	0.79391	0.79839	0.49566	0.90064	2.77E-05	0.00029	0.00029	pink
GNG3	0.06223	-0.07563	-0.09642	-0.00636	0.00134	-0.02104	-0.08825	0.2411	-0.2411	0.41873	0.32551	0.20965	0.93422	0.98608	0.78473	0.25104	0.00149	0.00149	grey
MAGT1	-0.0246	-0.16181	0.01512	0.10362	-0.07833	0.07478	0.06016	-0.01623	0.01623	0.74946	0.03449	0.84442	0.17745	0.30851	0.33102	0.43445	0.83313	0.83313	turquoise
SDF2L1	0.05245	0.04766	-0.2045	-0.1242	0.20712	-0.2829	-0.05516	-0.08669	0.08669	0.4957	0.53589	0.0073	0.10555	0.00657	0.00018	0.47367	0.25956	0.25956	grey
CFAP53	-0.02902	-0.18321	-0.21125	-0.06588	0.19632	-0.28305	-0.19513	0.18673	-0.18673	0.70636	0.01646	0.00555	0.39196	0.01007	0.00018	0.01054	0.01447	0.01447	brown
OXSR1	0.02331	-0.14563	0.07241	0.00508	-0.15963	0.21239	0.14658	-0.16075	0.16075	0.76222	0.05736	0.3466	0.94741	0.03702	0.00529	0.05575	0.0357	0.0357	turquoise
COQ10B	0.03004	-0.12865	0.04494	0.02224	-0.03992	0.14604	0.01817	-0.05394	0.05394	0.69653	0.09355	0.55948	0.77279	0.60418	0.05666	0.8135	0.48353	0.48353	turquoise
DYSF	-0.15682	-0.05319	-0.01135	0.07868	-0.12428	0.00373	0.00996	0.40505	-0.40505	0.04053	0.48957	0.88291	0.30635	0.10534	0.96142	0.89718	3.90E-08	3.90E-08	blue
ZBTB33	-0.0756	-0.22097	-0.07928	0.00689	-0.17298	0.0093	-0.01599	0.22075	-0.22075	0.32573	0.00368	0.30266	0.92874	0.02367	0.90389	0.83558	0.00371	0.00371	turquoise
ADGRF4	0.11477	-0.08044	0.01308	0.0679	-0.04829	0.14594	0.12675	-0.20394	0.20394	0.13499	0.29562	0.86516	0.37755	0.53053	0.05682	0.09854	0.00746	0.00746	turquoise
NPC1	0.02154	-0.18076	0.10082	0.10410	-0.16037	0.14204	0.07558	-0.06021	0.06021	0.77971	0.01798	0.18948	0.06567	0.03615	0.06385	0.32589	0.43402	0.43402	turquoise
MED21	0.04749	-0.14377	-0.03047	-0.00238	-0.08378	0.01438	-0.01757	-0.0356	0.0356	0.53738	0.06066	0.69238	0.97531	0.27597	0.85192	0.8196	0.64386	0.64386	turquoise
ATG12	0.04975	-0.17271	-0.03947	0.01403	-0.03847	-0.02592	0.02739	-0.02084	0.02084	0.51818	0.02389	0.60823	0.85545	0.61736	0.73646	0.72214	0.78678	0.78678	turquoise
PRSS36	0.03298	0.0292	0.11748	0.04249	-0.03546	0.17808	0.16553	-0.13744	0.13744	0.66847	0.70458	0.12594	0.58105	0.64523	0.01979	0.03049	0.07305	0.07305	grey
MAP3K3	-0.10754	-0.13768	-0.04663	0.02358	-0.16722	0.00518	-0.08972	0.53819	-0.53819	0.1615	0.07253	0.54478	0.75954	0.02881	0.94637	0.2432	3.16E-14	3.16E-14	blue
HSF2	-0.04611	-0.0584	-0.10908	-0.02719	-0.03475	-0.16508	-0.11435	0.44041	-0.44041	0.54923	0.44802	0.15555	0.72404	0.65183	0.03096	0.13643	1.66E-09	1.66E-09	turquoise
FAM124B	-0.09103	-0.08015	-0.05697	0.11644	-0.11574	0.12019	-0.01763	0.44689	-0.44689	0.23639	0.29739	0.45923	0.12936	0.13169	0.11739	0.81897	8.95E-10	8.95E-10	blue
GLCCI1	-0.06853	-0.1112	-0.0572	-0.02445	-0.02319	-0.07754	-0.11671	0.44234	-0.44234	0.3731	0.14763	0.45738	0.75089	0.76336	0.31342	0.12847	1.38E-09	1.38E-09	turquoise
STXBP6	0.11196	-0.09328	-0.06866	0.07551	0.04537	0.15878	-0.04467	0.06712	-0.06712	0.14485	0.22493	0.37222	0.32628	0.55571	0.03806	0.56179	0.3831	0.3831	grey
PAGR1	-0.07456	-0.0409	-0.12589	0.00219	0.20228	-0.11312	-0.00854	-0.0453	0.0453	0.33246	0.59537	0.10087	0.97734	0.00797	0.14072	0.91174	0.5563	0.5563	green
KLHL42	-0.06325	-0.12965	-0.1207	-0.00035	-0.02312	-0.22641	-0.17605	0.43932	-0.43932	0.41115	0.09102	0.11582	0.99641	0.76403	0.0029	0.02126	1.84E-09	1.84E-09	turquoise
ZADH2	-0.04275	-0.13635	-0.19644	-0.08489	0.14805	-0.13053	-0.14869	0.21786	-0.21786	0.57879	0.07537	0.01002	0.26961	0.0533	0.08882	0.05227	0.0042	0.0042	turquoise
FAM117B	0.03505	-0.03102	-0.05592	0.04344	-0.0341	0.07948	-0.01936	0.07203	-0.07203	0.64906	0.6871	0.46753	0.57267	0.65793	0.30109	0.80158	0.34917	0.34917	turquoise
PREPL	0.00874	-0.2038	-0.19604	-0.05672	0.01779	-0.11319	-0.14275	0.36813	-0.36813	0.90966	0.0075	0.01018	0.46121	0.81734	0.14048	0.06252	7.28E-07	7.28E-07	turquoise
ZKSCAN2	-0.0955	-0.18283	-0.18663	-0.04389	-0.0526	-0.21239	-0.10144	0.43542	-0.43542	0.21404	0.01669	0.01452	0.56866	0.49442	0.00529	0.18677	2.65E-09	2.65E-09	turquoise
GNPAT	0.03376	0.0153	-0.04525	-0.06891	-0.0342	0.085	0.13693	-0.20562	0.20562	0.66114	0.84253	0.55674	0.37047	0.65698	0.26898	0.07411	0.00698	0.00698	turquoise
INHA	-0.06037	-0.01449	-0.0301	-0.02753	0.05532	-0.037	-0.0819	-0.04367	0.04367	0.4328	0.85078	0.69596	0.7208	0.47237	0.63095	0.28694	0.57063	0.57063	grey

ADGRG7	-0.14834	0.0638	0.06145	0.0692	0.05501	0.06998	0.05918	-0.16584	0.16584	0.05283	0.4071	0.42463	0.36844	0.47482	0.36308	0.44194	0.03017	0.03017	grey
RSF1	-0.02998	-0.20115	-0.04168	0.01291	-0.14604	0.05226	0.03294	0.15072	-0.15072	0.69708	0.00834	0.58829	0.86695	0.05665	0.49721	0.66886	0.04909	0.04909	turquoise
PNMA8A	-0.01485	-0.15861	-0.21421	-0.07417	-0.00717	-0.23053	-0.16727	0.54496	-0.54496	0.84711	0.03826	0.0049	0.33496	0.92582	0.00242	0.02876	1.30E-14	1.30E-14	brown
SPAG4	-0.04962	-0.0758	0.07636	0.09505	0.01585	0.09471	0.12339	-0.41365	0.41365	0.51921	0.32447	0.32085	0.21622	0.83698	0.21787	0.10786	1.87E-08	1.87E-08	grey
MSX1	0.03381	-0.02618	-0.07624	0.06657	-0.08492	0.00686	0.11706	0.27284	-0.27284	0.66064	0.73398	0.32167	0.387	0.26948	0.929	0.12731	0.00031	0.00031	grey
GIMAP5	-0.13468	-0.03834	-0.02578	0.04272	-0.10132	0.03068	-0.05963	0.52949	-0.52949	0.07905	0.61862	0.73782	0.57903	0.18732	0.69039	0.43851	9.62E-14	9.62E-14	blue
ATP7A	-0.02996	-0.18152	0.00625	0.04987	-0.09367	0.10157	0.0295	0.17848	-0.17848	0.69731	0.0175	0.93532	0.51718	0.223	0.1862	0.70175	0.01951	0.01951	turquoise
COPS2	-0.07501	-0.08535	-0.03576	-0.00874	-0.1416	0.04989	0.00945	0.05374	-0.05374	0.3295	0.26702	0.64236	0.90965	0.0647	0.51693	0.90236	0.48515	0.48515	turquoise
NDFIP1	0.01623	-0.18837	-0.21704	-0.10039	0.0745	-0.21762	-0.17659	0.23993	-0.23993	0.83316	0.01361	0.00435	0.1914	0.33284	0.00425	0.02086	0.00157	0.00157	turquoise
MMP14	-0.03676	-0.04673	0.07521	0.09109	-0.15679	0.20574	0.18454	-0.05568	0.05568	0.63313	0.54392	0.32825	0.23608	0.04056	0.00694	0.01568	0.46949	0.46949	black
CAB39L	-0.01138	-0.14986	-0.05154	0.02382	-0.11988	-0.07431	-0.05649	0.3709	-0.3709	0.88257	0.05043	0.50319	0.75713	0.11834	0.33407	0.46304	5.92E-07	5.92E-07	turquoise
KDM5A	0.03445	-0.0887	-0.00654	0.04691	-0.11883	0.09795	-0.00579	0.10206	-0.10206	0.65464	0.24862	0.9323	0.54238	0.12162	0.20248	0.94011	0.18407	0.18407	turquoise
OCRL	0.01371	-0.2453	-0.22163	-0.11894	0.06688	-0.22797	-0.10141	0.23498	-0.23498	0.85879	0.00122	0.00358	0.12128	0.38475	0.00271	0.18691	0.00198	0.00198	turquoise
ZNF322	0.00901	-0.16435	-0.02492	#######	-0.05123	0.0514	0.04468	0.29953	-0.29953	0.90693	0.03172	0.74625	0.99988	0.50576	0.50436	0.56177	6.89E-05	6.89E-05	turquoise
RAB25	0.07899	0.0485	0.0738	-0.02956	0.0579	0.1698	0.14079	-0.52533	0.52533	0.30447	0.52871	0.33744	0.70116	0.4519	0.0264	0.06626	1.62E-13	1.62E-13	yellow
MAP6	-0.02443	-0.15173	-0.24444	-0.14061	0.02731	-0.29186	-0.28527	0.48385	-0.48385	0.75112	0.04758	0.00127	0.0666	0.72295	0.00011	0.00016	2.03E-11	2.03E-11	brown
GABRB3	0.03865	-0.10182	-0.19143	-0.0832	0.16448	-0.21805	-0.22045	0.33103	-0.33103	0.61578	0.18513	0.01214	0.2793	0.03157	0.00417	0.00376	9.76E-06	9.76E-06	brown
CYP51A1	0.04821	-0.07524	0.0407	0.05154	0.09876	0.11913	-0.01227	-0.06397	0.06397	0.53121	0.32807	0.59717	0.50321	0.19876	0.12068	0.8734	0.40585	0.40585	grey
PLEKHA3	-0.02637	-0.14512	-0.03888	0.06083	-0.09725	0.01765	-0.04244	0.17609	-0.17609	0.73206	0.05825	0.61365	0.4293	0.20572	0.81882	0.58157	0.02124	0.02124	turquoise
CD81	-0.11128	-0.09145	-0.09879	0.01694	-0.16023	-0.09946	-0.03482	0.38077	-0.38077	0.14732	0.23423	0.1986	0.82592	0.0363	0.19558	0.65116	2.78E-07	2.78E-07	blue
LRAT	0.00174	-0.04412	-0.25759	-0.13922	0.10368	-0.227	-0.13142	0.02361	-0.02361	0.98199	0.56668	0.00067	0.06936	0.17718	0.00283	0.08664	0.75919	0.75919	grey
C12orf65	-0.01016	-0.21206	-0.14191	-0.0538	0.078	-0.21173	-0.12856	0.15548	-0.15548	0.89509	0.00536	0.0641	0.48462	0.31055	0.00544	0.09377	0.0423	0.0423	turquoise
PAFAH2	-0.0185	-0.14332	0.04379	0.02553	0.00901	0.1781	0.02932	-0.04244	0.04244	0.81021	0.06148	0.56958	0.74027	0.90694	0.01978	0.70347	0.58155	0.58155	turquoise
HS3ST3B1	0.01522	-0.12142	0.12837	0.00155	-0.16245	-0.0645	-0.05835	0.29758	-0.29758	0.84337	0.11366	0.09427	0.984	0.03376	0.40194	0.4484	7.73E-05	7.73E-05	turquoise
CHI3L2	0.03286	0.00096	0.09171	-0.00472	-0.11335	0.121	-0.08038	0.23512	-0.23512	0.66966	0.99003	0.23289	0.95119	0.1399	0.11492	0.29601	0.00196	0.00196	blue
HMBOX1	-0.06389	-0.15518	-0.03232	0.01826	-0.01635	-0.00974	-0.09205	0.22303	-0.22303	0.40641	0.0427	0.67477	0.81262	0.83187	0.89941	0.23113	0.00337	0.00337	turquoise
ZNF585B	-0.03332	-0.14294	-0.08741	-0.00185	-0.02908	-0.02407	-0.10177	0.28874	-0.28874	0.66524	0.06218	0.25562	0.98079	0.70576	0.75463	0.18532	0.00013	0.00013	turquoise
C19orf48	0.18748	0.23883	-0.00713	-0.05998	0.00445	0.09122	0.24315	-0.47908	0.47908	0.01407	0.00166	0.92624	0.43582	0.95396	0.23538	0.00135	3.39E-11	3.39E-11	purple
RBKS	-0.08035	-0.15704	-0.1273	0.0076	0.00162	-0.04896	-0.02791	0.09397	-0.09397	0.29617	0.04024	0.09709	0.9214	0.98321	0.52484	0.71704	0.22153	0.22153	grey
LEMD1	0.01792	0.02009	0.07125	-0.04607	-0.07957	0.09137	0.16562	-0.40518	0.40518	0.81607	0.7942	0.35438	0.54963	0.30092	0.2346	0.0304	3.86E-08	3.86E-08	yellow
RNASEH2/	0.03338	0.14592	-0.06788	-0.05713	0.0257	-0.08306	0.24122	-0.44941	0.44941	0.66469	0.05686	0.37772	0.45794	0.73862	0.2801	0.00148	7.01E-10	7.01E-10	pink
ABLIM1	0.10952	-0.04656	-0.02989	-0.00625	0.05474	0.01778	-0.06909	0.02913	-0.02913	0.15387	0.54538	0.69797	0.93536	0.47699	0.81746	0.36926	0.70526	0.70526	turquoise
RRM2	-0.07129	0.08647	0.07432	0.00676	-0.03113	0.14839	0.32024	-0.3753	0.3753	0.35413	0.26079	0.33403	0.93006	0.68603	0.05275	1.96E-05	4.24E-07	4.24E-07	pink
DDHD1	-0.11365	-0.10862	-0.02458	0.04439	-0.04679	-0.07249	-0.12835	0.39097	-0.39097	0.13884	0.15733	0.74961	0.56426	0.54335	0.34607	0.09433	1.24E-07	1.24E-07	turquoise
PINLYP	-0.03103	-0.03611	-0.08902	-0.03693	-0.03929	-0.02584	0.08349	0.38878	-0.38878	0.68705	0.63914	0.24691	0.63152	0.60994	0.73727	0.27764	1.48E-07	1.48E-07	grey
LSM7	0.04176	0.06627	-0.13454	-0.10433	0.25551	-0.13924	0.0517	-0.33634	0.33634	0.58763	0.3891	0.07935	0.17445	0.00074	0.06932	0.5019	6.87E-06	6.87E-06	green
HARBI1	-0.02673	-0.08062	-0.03621	-0.06338	-0.042	0.03679	0.13408	-0.02393	0.02393	0.72853	0.29454	0.63825	0.41021	0.58541	0.63287	0.08039	0.75607	0.75607	turquoise
MOB2	0.09777	-0.03474	-0.08688	-0.08425	0.07932	-0.01662	0.04501	-0.25584	0.25584	0.20333	0.65196	0.25852	0.27324	0.30245	0.8292	0.55888	0.00073	0.00073	grey
ANXA8L1	0.0364	-0.00816	0.08775	0.07765	-0.09323	0.05544	0.19092	-0.29069	0.29069	0.63649	0.91568	0.25376	0.31274	0.22517	0.4714	0.01237	0.00011	0.00011	grey
MAPKAP1	-0.04581	-0.0343	-0.01767	0.05525	-0.16934	0.08399	0.08418	0.10619	-0.10619	0.55187	0.65602	0.81855	0.47296	0.02682	0.27476	0.27366	0.16688	0.16688	turquoise
RPS6KB2	-0.05872	0.0304	-0.03335	-0.03176	0.07019	-0.02252	0.07572	-0.29115	0.29115	0.44552	0.69307	0.66496	0.68011	0.36162	0.77001	0.32493	0.00011	0.00011	green
VAT1L	-0.10358	-0.13667	-0.12678	-0.01406	-0.01469	-0.12683	-0.1244	0.50544	-0.50544	0.17761	0.07467	0.09846	0.85514	0.84874	0.09833	0.10499	1.78E-12	1.78E-12	brown
EIF4EBP2	0.03793	-0.07727	0.0363	0.05685	-0.19337	0.18695	0.12608	0.08681	-0.08681	0.62238	0.31515	0.63742	0.46014	0.01127	0.01435	0.10035	0.25888	0.25888	turquoise
WDR12	-0.00851	0.0197	0.01003	-0.0012	-0.08643	0.09742	0.15132	-0.01245	0.01245	0.91208	0.79815	0.89644	0.98759	0.26097	0.20495	0.0482	0.87158	0.87158	turquoise
AGBL5	0.02621	0.00128	-0.0808	0.03291	-0.0253	0.02407	0.13243	-0.11656	0.11656	0.7336	0.98676	0.29346	0.66916	0.74259	0.75468	0.08422	0.12897	0.12897	grey
CCDC159	0.01652	-0.02093	-0.1596	-0.19026	0.23423	-0.12952	-0.18468	-0.00868	0.00868	0.83017	0.78585	0.03705	0.01268	0.00205	0.09135	0.0156	0.91028	0.91028	red
ERGIC2	-0.01982	-0.09766	0.05644	0.08349	-0.16385	0.04789	0.07797	0.00883	-0.00883	0.7969	0.20381	0.46344	0.2776	0.03224	0.53395	0.31072	0.90871	0.90871	turquoise
TMEM154	-0.04467	0.07852	0.12889	-0.00599	-0.16264	0.19998	0.08511	-0.18871	0.18871	0.56185	0.30731	0.09293	0.938	0.03355	0.00873	0.26839	0.01344	0.01344	grey
ACADM	-0.1561	-0.10436	-0.03858	-0.01877	0.01611	0.00741	-0.16092	0.40685	-0.40685	0.04147	0.17434	0.61636	0.80746	0.83437	0.92336	0.03551	3.35E-08	3.35E-08	turquoise
KIF3C	-0.0661	-0.16211	-0.08634	0.03171	-0.15176	-0.14771	-0.08971	0.41509	-0.41509	0.39033	0.03415	0.26148	0.68055	0.04755	0.05385	0.24328	1.65E-08	1.65E-08	turquoise
C12orf75	-0.081	-0.07643	-0.01322	0.03666	0.17383	-0.0525	-0.18411	-0.0515	0.0515	0.29227	0.32042	0.86378	0.63409	0.02298	0.49526	0.01593	0.50351	0.50351	grey
SH3GLB1	-0.05067	-0.14158	0.03203	0.0521	-0.16161	0.11346	-0.0007	0.08964	-0.08964	0.51044	0.06473	0.67753	0.49854	0.03471	0.13953	0.99276	0.24362	0.24362	turquoise
CRABP1	0.04994	0.02054	0.05153	0.09022	-0.03277	0.00956	0.12503	0.13664	-0.13664	0.51652	0.78973	0.50327	0.24061	0.67051	0.90126	0.10323	0.07473	0.07473	grey
TIMD4	-0.17789	-0.01197	0.08084	-0.00455	-0.10955	0.09413	-0.05965	0.29307	-0.29307	0.01993	0.87655	0.29322	0.9529	0.15378	0.22075	0.43832	0.0001	0.0001	blue
SREK1	-0.01058	-0.14824	-0.08766	-0.02169	0.0642	-0.04559	-0.06811	0.17034	-0.17034	0.8908	0.05299	0.25427	0.77823	0.40413	0.55375	0.37609	0.02592	0.02592	turquoise
FBXL13	0.01764	-0.15012	0.03343	0.06501	-0.02683	0.05175	0.08554	-0.09568	0.09568	0.8189	0.05002	0.66427	0.39821	0.7276	0.50146	0.26594	0.21316	0.21316	grey
MST1	-0.05574	-0.06063	-0.13544	-0.12998	0.30439	-0.17651	-0.11771	-0.13374	0.13374	0.46897	0.43083	0.07735	0.09018	5.17E-05	0.02092	0.12519	0.08117	0.08117	red
PTPRN	-0.00695	-0.12492	-0.25928	-0.12472	0.07713	-0.32008	-0.17177	0.40341	-0.40341	0.92812	0.10353	0.00062	0.1041	0.31602	1.98E-05	0.02467	4.48E-08	4.48E-08	brown
CCL21	-0.19881	-0.00851	-0.06235	0.04391	-0.03741	0.07258	-0.01958	0.32245	-0.32245	0.00914	0.91205	0.41789	0.56853	0.62714	0.34551	0.79931	1.70E-05	1.70E-05	blue
PDZD3	0.03826	-0.00469	0.12884	0.05967	0.24309	0.10458	0.05428	-0.22062	0.22062	0.61927	0.95142	0.09308	0.43822	0.00136	0.17344	0.48077	0.00374	0.00374	grey
SLC16A10	-0.10252	-0.10324	0.13109	0.12873	-0.23222	0.10635	-0.04171	0.38076	-0.38076	0.18212	0.17904	0.08745	0.09336	0.00224	0.16624	0.58809	2.79E-07	2.79E-07	blue
FAM207A	0.08548	0.04268	-0.09373	-0.07062	0.16767	-0.06595	0.18174	-0.33608	0.33608	0.26628	0.57943	0.22272	0.35871	0.02838	0.39145	0.01736	6.99E-06	6.99E-06	green
DNAJB13	-0.05569	-0.02334	-0.01934	0.13107	0.19508	0.11082	0.03665	-0.30517	0.30517	0.46939	0.76191	0.80176	0.0875	0.01056	0.14902	0.63411	4.94E-05	4.94E-05	grey
HAUS3	-0.11947	-0.12596	-0.09309	0.01245	-0.03482	-0.00527	0.00427	0.13944	-0.13944	0.11963	0.10066	0.22591	0.8716	0.65121	0.94545	0.95575	0.06893	0.06893	turquoise
CDC42EP5	0.13006	0.09872	0.10002	-0.00975	0.09822	0.27026	0.23997	-0.4647	0.4647	0.08999	0.19893	0.19307	0.89924	0.20121	0.00035	0.00157	1.53E-10	1.53E-10	yellow
PACS1	0.01393	-0.12236	0.04403	0.0691	-0.22069	0.15804	0.04539	0.00218	-0.00218	0.85646	0.11088	0.56741	0.36917	0.00373	0.03898	0.55551	0.97745	0.97745	turquoise
MTA2	-0.01864	-0.08884	0.0274	-0.00253	-0.08082	0.07521	0.12312	-0.12121	0.12121	0.80875	0.24789	0.72205	0.97384	0.29336	0.32825	0.10864	0.1143	0.1143	turquoise
ZNF138	-0.00829	-0.08289	0.05587	0.03936	0.05958	0.06412	0.09171	-0.11121	0.11121	0.91433	0.28109	0.46799	0.60929	0.43891	0.40475	0.23286	0.14759	0.14759	grey
DENND11	-0.06341	-0.07374	-0.07211	-0.10676	0.04404	-0.03959	-0.08319	0.13657	-0.13657	0.40999	0.33781	0.34864	0.1646	0.56731	0.60713	0.27935	0.07489	0.07489	turquoise
TMEM140	-0.00339	-0.12886	-0.02938	0.10638	-0.16204	0.08716	-0.00078	0.18672	-0.18672	0.96492	0.09302	0.7029	0.1661	0.03423	0.25698	0.99197	0.01447	0.01447	blue
ATP6AP1L	-0.03525	-0.0505	-0.1748	-0.03372	-0.01717	-0.18448	0.03782	-0.04094	0.04094	0.64713	0.51185	0.02222	0.66153	0.8236	0.01571	0.62337	0.59497	0.59497	grey
PFN4	-0.03659	-0.15678	-0.14746	0.03465	0.00112	-0.14068	0.00772	-0.06182	0.06182	0.63473	0.04058	0.05427	0.65274	0.98845	0.06647	0.92016	0.42183	0.42183	grey
NEDD4	-0.08279	-0.13921	0.00826	0.12797	-0.23173	0.11326	0.12326	0.22098	-0.22098	0.2817	0.06938	0.91456	0.09531	0.00229	0.14023	0.10826	0.00368	0.00368	turquoise
PTPRR	0.04783	-0.07116	0.07876	0.1283	0.02531	0.13558	0.06617	-0.33543	0.33543	0.53447	0.35501	0.30584	0.09445	0.74243	0.07704	0.38985	7.30E-06	7.30E-06	grey
VSTM2L	0.03369	-0.1411	0.03371	0.00316	-0.00084	-0.04094	-0.01989	-0.19939	0.19939	0.66181	0.06565	0.66156	0.96728	0.99126	0.59492	0.79621	0.00893	0.00893	grey
RNF121	0.04096	-0.16644	-0.0896	0.00126	-0.13729	0.01785	0.10374	-0.01525	0.01525	0.59478	0.02958	0.24384	0.98695	0.07335	0.81671	0.17691	0.8431	0.8431	turquoise
DHX9	-0.00142	-0.07724	-0.00989	-0.01535	-0.09721	0.05247	0.057	0.08443	-0.08443	0.98526	0.31532	0.89789	0.8421	0.2059	0.49555	0.45896	0.27224	0.27224	turquoise
CACNG6	-0.02623	-0.05831	0.01148	0.033	0.16216	0.02187	-0.0618	-0.14373	0.14373	0.7334	0.44873	0.88154	0.66835	0.03409	0.77643	0.42201	0.06073	0.06073	grey
DPP8	-0.08772	-0.08014	-0.05111	0.01564	-0.06031	0.01355	-0.0452	0.21971	-0.21971	0.2539	0.29745	0.5068	0.83915	0.43332	0.86038	0.5572	0.00388	0.00388	turquoise
SDSL	0.07094	-0.0347	-0.18096	-0.1135	0.19973	-0.28293	-0.15449	0.14229	-0.14229	0.35649	0.65231	0.01786	0.13937	0.00882	0.00018	0.04363	0.06339	0.06339	grey
ATN1	0.03119	-0.17221	-0.07045	-0.02174	0.06714	-0.09001	-0.10385	0.1074	-0.1074	0.68553	0.02431	0.35987	0.77778	0.38291	0.24171	0.17645	0.16208	0.16208	turquoise
EVI2B	-0.14484	-0.05167	0.10375	0.06955	-0.22134	0.17939	0.04037	0.36412	-0.36412	0.05875	0.50211	0.17687	0.36606	0.00362	0.01889	0.60006	9.79E-07	9.79E-07	blue
LYRM9	-0.06182	-0.04887	-0.24059	-0.13254	0.09556	-0.27528	-0.23949	0.3958	-0.3958	0.42187	0.52561	0.00153	0.08396	0.21377	0.00027	0.00161	8.40E-08	8.40E-08	brown
ZNF419	-0.00434	-0.1519	-0.2023	-0.04409	0.03994	-0.12391	-0.08285	0.0397	-0.0397	0.95511	0.04733	0.00797	0.5669	0.60397	0.10637	0.28135	0.60614	0.60614	turquoise
OSTC	-0.057	-0.01026	0.08869	0.08198	-0.08506	0.07633	0.09863	-0.03134	0.03134	0.45901	0.89408	0.24871	0.28647	0.26864	0.32105	0.19935	0.68404	0.68404	turquoise
RAB6A	-0.03107	-0.14792	0.04512	0.06788	-0.1789	0.12389	0.06708	-0.01473	0.01473	0.68661	0.05352	0.55787	0.37769	0.01922	0.10644	0.38338	0.84837	0.84837	turquoise
CLDN2	-0.03904	0.00618	0.09008	0.1044	0.0056	0.2883	0.1035	-0.03774	0.03774	0.61219	0.93604	0.24131	0.17418	0.94202	0.00013	0.17794	0.62408	0.62408	grey
CLDN3	0.10913	-0.11687	-0.09657	0.06631	0.16484	-0.04814	0.02221	-0.1385	0.1385	0.15537	0.12795	0.20896	0.38889	0.0312	0.53176	0.77313	0.07084	0.07084	grey
TAF4	0.08833	-0.06447	-0.01997	0.06655	-0.00292	0.02309	0.11168	-0.19804	0.19804	0.25061	0.40215	0.79548	0.38716	0.9698	0.76432	0.14588	0.00942	0.00942	turquoise
CHST3	-0.01779	-0.13056	-0.0337	0.10673	-0.26937	0.09125	0.06671	0.21356	-0.21356	0.81733	0.08874	0.66168	0.16471	0.00037	0.23523	0.38596	0.00504	0.00504	black
SARS2	0.06067	0.00394	-0.06264	0.00971	0.11016	-0.06639	0.12074	-0.27767	0.27767	0.43055	0.95921	0.41568	0.89972	0.15147	0.3883	0.11569	0.00024	0.00024	red
SMARCA1	-0.07317	-0.27781	-0.19115	-0.05439	-0.066	-0.17778	-0.09667	0.41372	-0.41372	0.3416	0.00023	0.01226	0.47983	0.39108	0.02	0.20849	1.86E-08	1.86E-08	turquoise
RNF157	0.0288	-0.14836	-0.16716	-0.00977	0.04501	-0.24283	-0.14187	0.2899	-0.2899	0.7085	0.05281	0.02887	0.89912	0.55885	0.00137	0.06417	0.00012	0.00012	brown
INS	0.0188	0.0765	-0.07059	0.04649	-0.00198	0.01205	0.08678	0.11443	-0.11443	0.80722	0.31999	0.35893	0.54597	0.97953	0.87569	0.25904	0.13616	0.13616	grey
FERMT1	0.06886	0.20035	0.14915	0.04303	-0.08943	0.20233	0.24905	-0.35453	0.35453	0.37083	0.00861	0.05153	0.57628	0.24473	0.00796	0.00102	1.96E-06	1.96E-06	yellow
CBARP	-0.04858	-0.18035	-0.16281	-0.05996	0.0407	-0.27017	-0.08385	0.34744	-0.34744	0.52807	0.01825	0.03337	0.43598	0.59709	0.00035	0.27556	3.23E-06	3.23E-06	brown
SCUBE3	0.05969	0.02463	0.09544	0.01824	-0.20158	0.10902	0.02813	0.1202	-0.1202	0.43803	0.74913	0.21434	0.81278	0.0082	0.1558	0.71497	0.11735	0.11735	black
MYO9B	-0.03344	-0.05746	0.07685	0.08461	-0.17219	0.12666	0.10052	0.18323	-0.18323	0.66413	0.45538	0.31774	0.27123	0.02432	0.09877	0.19084	0.01645	0.01645	black
MLXIP	0.05564	-0.12301	-0.06867	0.01903	0.01651	-0.10534	-0.12582	0.28434	-0.28434	0.46979	0.10897	0.37214	0.80483	0.83032	0.17031	0.10106	0.00016	0.00016	turquoise
LSM10	-0.08838	-0.06597	-0.07323	-0.0576	0.00709	-0.07006	-0.09987	0.21829	-0.21829	0.25034	0.39131	0.34116	0.4543	0.92672	0.36253	0.19372	0.00413	0.00413	turquoise
CHRNB2	-0.03683	-0.13028	-0.26404	-0.15483	0.10702	-0.32798	-0.23927	0.44874	-0.44874	0.63251	0.08944	0.00048	0.04317	0.16354	1.19E-05	0.00162	7.48E-10	7.48E-10	brown
GSDMA	0.00758	0.043	0.00316	-0.02452	-0.01512	0.06938	0.07454	-0.06034	0.06034	0.92164	0.57652	0.96725	0.75023	0.84443	0.36725	0.33259	0.43305	0.43305	grey
ECHDC2	0.01448	-0.12046	-0.14258	-0.08245	0.25419	-0.08505	-0.15604	-0.07878	0.07878	0.8509	0.11655	0.06284	0.28367	0.00079	0.26873	0.04154	0.30573	0.30573	red
GLT8D1	0.01054	-0.13034	-0.10619	-0.08478	-0.01577	-0.0887	-0.04592	0.21991	-0.21991	0.8912	0.08928	0.16687	0.27023	0.83783	0.24861	0.55089	0.00385	0.00385	turquoise
SAMHD1	-0.11583	-0.0006	0.03147	0.09408	-0.21882	0.11403	0.02466	0.52082	-0.52082	0.13138	0.99375	0.68279	0.22096	0.00403	0.13754	0.7489	2.83E-13	2.83E-13	blue
CFD	-0.00096	0.04647	-0.00428	0.05993	0.0461	0.01586	0.00449	0.29151	-0.29151	0.99008	0.54618	0.95565	0.43622	0.54935	0.83684	0.95354	0.00011	0.00011	grey
APCDD1L	-0.06784	-0.01024	0.02996	0.12135	-0.12329	0.08773	0.05789	-0.01174	0.01174	0.37795	0.89424	0.69727	0.11387	0.10816	0.25387	0.452	0.87887	0.87887	grey
STX6	-0.01337	-0.03072	0.06741	0.06825	-0.10109	0.19594	0.2277	-0.1503	0.1503	0.86223	0.68998	0.38103	0.37512	0.18831	0.01022	0.00274	0.04975	0.04975	turquoise
HMOX2	-0.05109	0.0235	-0.01146	0.02604	-0.0561	0.08053	0.09725	-0.13074	0.13074	0.50695	0.76028	0.88173	0.73531	0.46611	0.2951	0.20573	0.08829	0.08829	turquoise
EGR2	-0.0674	-0.15651	0.02694	0.0578	-0.16011	0.14371	0.01872	0.37423	-0.37423	0.38109	0.04094	0.72654	0.45267	0.03645	0.06077	0.80798	4.60E-07	4.60E-07	blue
TTYH1	0.04359	-0.05829	-0.23066	-0.03011	-0.00897	-0.09487	-0.10943	0.08053	-0.08053	0.57134	0.4489	0.0024	0.69582	0.90735	0.21711	0.15422	0.29506	0.29506	magenta
CDKN1C	-0.00757	-0.04396	-0.08079	0.00667	-0.00758	0.00378	-0.049	0.09543	-0.09543	0.92175	0.56803	0.29352	0.931	0.9216	0.9609	0.52446	0.21437	0.21437	grey
AGPAT5	-0.03988	0.01304	-0.01538	-0.02518	0.01553	-0.0651	0.07803	0.03632	-0.03632	0.60451	0.86556	0.84171	0.74377	0.84024	0.39759	0.31039	0.63724	0.63724	turquoise
TCAP	0.03946	-0.1288	-0.12252	0.01948	0.24423	-0.29748	-0.17162	0.07575	-0.07575	0.60838	0.09318	0.11039	0.80039	0.00129	7.77E-05	0.0248	0.32474	0.32474	grey
FEZ1	-0.05067	-0.0764	-0.02793	0.04961	-0.16246	0.05366	0.0096	0.4345	-0.4345	0.51044	0.32064	0.71693	0.51936	0.03376	0.48576	0.90085	2.89E-09	2.89E-09	blue
NAT10	0.09529	-0.0317	0.06453	-0.00329	-0.15244	0.12768	0.17666	-0.22103	0.22103	0.21506	0.68063	0.40174	0.96591	0.04655	0.09607	0.02081	0.00367	0.00367	turquoise
PPARGC1/	0.05482	-0.01345	-0.07315	0.02994	0.02762	0.07836	-0.13357	0.08145	-0.08145	0.47636	0.86144	0.34167	0.6975	0.71985	0.30833	0.08158	0.28959	0.28959	grey
FAM3D	0.09387	0.10882	0.16399	0.02483	0.14982	0.26554	0.11208	-0.25052	0.25052	0.222	0.15656	0.03209	0.74722	0.05049	0.00045	0.14444	0.00095	0.00095	yellow
SPR	0.01634	0.0399	-0.02721	0.05325	0.05692	-0.01402	0.09138	-0.45833	0.45833	0.83204	0.60436	0.72391	0.48915	0.45963	0.85554	0.23457	2.91E-10	2.91E-10	grey
GGA3	-0.03512	-0.11912	-0.00832	-0.07574	0.07875	-0.0221	-0.00792	-0.05264	0.05264	0.64839	0.12072	0.91404	0.3248	0.30593	0.77413	0.9181	0.49414	0.49414	red
ADAM12	-0.06541	-0.03151	0.07417	0.10892	-0.22763	0.12301	0.09413	0.302	-0.302	0.39536	0.68244	0.33496	0.15616	0.00275	0.10896	0.22071	5.96E-05	5.96E-05	black
XKR6	-0.00785	0.02304	-0.0474	-0.05916	0.17926	0.07785	0.07708	-0.07957	0.07957	0.91881	0.76487	0.5381	0.44214	0.01898	0.3115	0.31631	0.30092	0.30092	grey
USP54	0.05285	-0.08463	0.13366	0.07743	-0.05917	0.13102	0.05863	-0.19655	0.19655	0.49236	0.2711	0.08135	0.31414	0.44207	0.08761	0.44625	0.00998	0.00998	turquoise
DCTD	-0.0987	-0.04776	-0.06373	-0.01719	-0.01683	0.03327	-0.01604	-0.02211	0.02211	0.19903	0.53502	0.4076	0.82337	0.82708	0.66571	0.83505	0.77408	0.77408	turquoise
TUT7	-0.01927	-0.12075	-0.00277	0.0694	-0.22177	0.18668	0.1495	-0.03473	0.03473	0.80246	0.11567	0.97134	0.36705	0.00356	0.01449	0.05098	0.65202	0.65202	turquoise
PLEKHF2	-0.01133	-0.10426	0.0607	0.039	-0.12969	0.02925	-0.05278	-0.01686	0.01686	0.88303	0.17475	0.43027	0.61254	0.09092	0.70408	0.49294	0.8268	0.8268	turquoise
GPATCH3	-0.03144	-0.14083	-0.05463	0.00902	-0.00874	-0.02108	-0.01701	0.1593	-0.1593	0.68312	0.06618	0.47789	0.90678	0.90965	0.7843	0.82523	0.03742	0.03742	grey
ACD	0.04938	-0.03834	-0.13739	-0.12247	0.07521	-0.1443	0.00526	-0.20704	0.20704	0.5213	0.61862	0.07315	0.11055	0.32823	0.0597	0.94554	0.00659	0.00659	green
PLEKHA6	0.10564	-0.01163	-0.05823	-0.08775	0.21928	0.01079	-0.06417	-0.2211	0.2211	0.16908	0.87995	0.44934	0.25376	0.00396	0.88856	0.40436	0.00366	0.00366	grey

NPHS1	-0.06047	0.06348	-0.0896	-0.0444	-0.00635	-0.11082	-0.21842	0.44054	-0.44054	0.43205	0.40943	0.24384	0.56418	0.93428	0.14904	0.0041	1.64E-09	1.64E-09	grey
PCM1	-0.03132	-0.11758	-0.04613	0.00098	-0.05424	0.00856	-0.01782	0.31935	-0.31935	0.68429	0.12563	0.54913	0.98987	0.48109	0.91154	0.81705	2.07E-05	2.07E-05	turquoise
POP4	0.03335	-0.14893	-0.04704	0.04609	-0.14444	-0.05925	0.11835	-0.12151	0.12151	0.66499	0.05189	0.54119	0.54947	0.05945	0.44139	0.12315	0.11338	0.11338	turquoise
DMAP1	-0.00795	-0.07665	-0.13783	-0.10507	0.1857	-0.21011	-0.1393	0.03335	-0.03335	0.91784	0.31906	0.07222	0.17141	0.01503	0.00581	0.06921	0.66499	0.66499	red
MUC1	0.08113	0.10489	0.11759	0.02483	-0.03173	0.31053	0.19882	-0.46343	0.46343	0.29149	0.17214	0.12559	0.74715	0.68031	3.57E-05	0.00914	1.74E-10	1.74E-10	yellow
AQP9	-0.16314	0.02034	0.13633	0.08864	-0.17373	0.15457	0.1419	0.20848	-0.20848	0.033	0.79176	0.07541	0.24897	0.02306	0.04354	0.06412	0.00621	0.00621	blue
ST8SIA3	-0.04234	-0.10998	-0.26327	-0.08053	0.12923	-0.24571	-0.18802	0.35545	-0.35545	0.58239	0.15216	0.0005	0.29509	0.09207	0.0012	0.01379	1.84E-06	1.84E-06	brown
C17orf113	0.05467	-0.07534	-0.13058	0.00936	0.17971	-0.10279	-0.06224	-0.05893	0.05893	0.47762	0.32737	0.08869	0.90325	0.01867	0.18094	0.41865	0.44388	0.44388	grey
ASIC3	-0.04344	-0.15861	-0.209	-0.08343	0.28316	-0.31956	-0.2151	0.01013	-0.01013	0.57261	0.03826	0.00608	0.27798	0.00017	2.04E-05	0.00472	0.89533	0.89533	grey
SLC30A6	-0.0295	-0.08839	0.02428	0.06593	-0.16185	0.15347	0.2	0.04511	-0.04511	0.70173	0.25029	0.7526	0.39159	0.03444	0.04506	0.00872	0.558	0.558	turquoise
HNRNPA1	-0.00069	-0.04487	-0.20268	-0.09729	0.0389	-0.16103	-0.15383	0.07471	-0.07471	0.9929	0.5601	0.00785	0.20556	0.61342	0.03537	0.04456	0.33148	0.33148	red
NACAD	-0.01005	-0.1585	-0.17325	-0.15877	0.00714	-0.30511	-0.21977	0.33355	-0.33355	0.89625	0.0384	0.02344	0.03807	0.92615	4.95E-05	0.00388	8.27E-06	8.27E-06	brown
SF3B2	0.05077	-0.05398	-0.04153	-0.06246	-0.07483	-0.00819	0.10669	-0.09495	0.09495	0.50959	0.48314	0.58965	0.41702	0.3307	0.91531	0.16485	0.2167	0.2167	turquoise
EAPP	0.0208	-0.07692	-0.03358	-0.00438	-0.01338	0.04341	-0.01138	-0.04864	0.04864	0.78713	0.31731	0.66281	0.95469	0.86207	0.5729	0.88259	0.52751	0.52751	turquoise
REXO4	0.07691	-0.09945	-0.06119	-0.00656	-0.13591	-0.02032	0.10681	-0.09515	0.09515	0.31742	0.19561	0.42663	0.93218	0.07632	0.79198	0.16438	0.21574	0.21574	turquoise
KCTD20	-0.08924	-0.14447	0.05392	0.06081	-0.1716	0.12384	0.02681	0.43277	-0.43277	0.24574	0.0594	0.48368	0.4295	0.02482	0.10657	0.72775	3.39E-09	3.39E-09	blue
MUS81	0.051	-0.07774	-0.00899	-0.0412	0.08283	-0.00282	0.0598	-0.24497	0.24497	0.5077	0.31219	0.90707	0.59263	0.28145	0.97077	0.43716	0.00124	0.00124	red
RTN4RL1	-0.03709	-0.1448	-0.24589	-0.05213	0.1043	-0.26577	-0.20433	0.41156	-0.41156	0.63005	0.05882	0.00119	0.49831	0.17459	0.00044	0.00735	2.24E-08	2.24E-08	brown
GPN1	0.01235	-0.06512	0.02472	0.03734	-0.16256	0.13649	0.23897	-0.16604	0.16604	0.87261	0.39745	0.74822	0.62775	0.03364	0.07505	0.00165	0.02998	0.02998	turquoise
ZNF770	-0.10957	-0.15097	-0.01908	0.04151	-0.15621	0.09764	0.02416	0.20533	-0.20533	0.15368	0.04871	0.80436	0.58984	0.04132	0.20389	0.75382	0.00706	0.00706	turquoise
NAV1	-0.04231	-0.14243	-0.05542	0.02496	-0.0608	-0.12122	-0.04065	0.28714	-0.28714	0.5827	0.06313	0.47156	0.74592	0.42958	0.11427	0.59761	0.00014	0.00014	turquoise
NFKBIZ	-0.04003	-0.15453	-0.00375	0.00922	-0.01373	0.10693	0.06142	-0.02076	0.02076	0.60317	0.04358	0.96118	0.90471	0.85855	0.1639	0.42483	0.78755	0.78755	red
ZNF780B	0.01789	-0.26866	-0.12948	0.01719	0.06047	-0.13061	-0.15168	0.1888	-0.1888	0.81636	0.00038	0.09143	0.82337	0.43207	0.08861	0.04765	0.0134	0.0134	turquoise
OTUD4	-0.10693	-0.17124	-0.04029	0.03095	-0.13262	0.06488	0.00608	0.13216	-0.13216	0.16391	0.02513	0.60079	0.68778	0.08378	0.39916	0.93707	0.08487	0.08487	turquoise
CEP128	-0.07881	-0.01499	0.0692	0.04551	-0.18677	0.10697	0.09123	0.18233	-0.18233	0.30553	0.8457	0.36844	0.55451	0.01445	0.16374	0.23536	0.017	0.017	turquoise
ACAP2	-0.07364	-0.12718	-0.01674	0.01093	-0.11408	0.07	0.03454	0.23051	-0.23051	0.33848	0.09738	0.82794	0.88714	0.13736	0.36298	0.65382	0.00242	0.00242	turquoise
WDFY2	-0.04166	-0.125	-0.13246	-0.04864	-0.07744	-0.20763	-0.10797	0.2774	-0.2774	0.58852	0.10332	0.08417	0.52752	0.31409	0.00643	0.15984	0.00024	0.00024	turquoise
TMEM246	0.10738	-0.01211	-0.11741	-0.1232	0.1266	-0.02828	-0.07961	-0.13465	0.13465	0.16214	0.8751	0.12618	0.10841	0.09893	0.71348	0.30063	0.07912	0.07912	grey
FAM83B	0.03619	-0.02876	0.13199	0.13467	-0.07898	0.22922	0.24184	-0.23742	0.23742	0.63839	0.70885	0.08527	0.07906	0.30452	0.00256	0.00144	0.00177	0.00177	turquoise
CRYL1	0.01666	-0.00268	-0.04977	-0.1308	0.1071	0.06035	-0.02648	-0.0732	0.0732	0.82874	0.97224	0.51795	0.08815	0.16324	0.433	0.73101	0.34138	0.34138	grey
ARID5A	-0.11622	-0.04905	0.04702	0.10162	-0.14127	0.1326	0.04776	0.25173	-0.25173	0.13009	0.52407	0.54139	0.18598	0.06531	0.08384	0.53505	0.0009	0.0009	blue
PANO1	0.05832	-0.08876	-0.13569	-0.04336	0.03002	-0.22547	-0.08542	0.17126	-0.17126	0.44866	0.24833	0.0768	0.57337	0.69675	0.00303	0.26665	0.02511	0.02511	grey
DAGLB	0.00603	-0.18978	0.01332	0.08086	-0.05565	0.0774	0.1136	-0.1583	0.1583	0.93757	0.01291	0.86271	0.2931	0.46974	0.31429	0.13903	0.03865	0.03865	grey
DNAJC1	0.05209	-0.01295	-0.01203	-0.04254	0.08415	-0.01147	-0.03768	-0.1935	0.1935	0.49863	0.86645	0.87594	0.5806	0.27383	0.88166	0.62461	0.01122	0.01122	grey
RPS15	0.13822	0.12341	-0.06412	-0.08282	0.12445	-0.07318	0.06426	-0.299	0.299	0.07141	0.10782	0.40471	0.28154	0.10486	0.34152	0.4037	7.11E-05	7.11E-05	purple
FAM184A	-0.06884	-0.12213	-0.14405	-0.04473	0.05349	-0.25094	-0.24845	0.55069	-0.55069	0.37097	0.11155	0.06016	0.56131	0.48717	0.00093	0.00105	6.03E-15	6.03E-15	brown
FZD5	0.02938	-0.02385	-0.03757	0.06146	0.03885	0.19916	0.04123	-0.27037	0.27037	0.70283	0.7568	0.62567	0.42458	0.61388	0.00901	0.59239	0.00035	0.00035	yellow
RFNG	0.00373	-0.06515	-0.0503	-0.06607	0.15292	-0.10051	0.06006	-0.17574	0.17574	0.9614	0.39724	0.51354	0.3906	0.04585	0.19085	0.43521	0.02149	0.02149	red
IL17RA	-0.06894	-0.07975	-0.0179	0.06385	-0.18511	0.05156	0.07504	0.23739	-0.23739	0.37025	0.29977	0.81622	0.40673	0.01535	0.50304	0.32932	0.00177	0.00177	blue
CD163	-0.07361	0.01069	0.0756	0.05673	-0.26226	0.13953	0.13315	0.38109	-0.38109	0.33869	0.88964	0.32573	0.46115	0.00053	0.06873	0.08255	2.72E-07	2.72E-07	blue
ZNF630	0.05328	-0.20145	-0.09892	-0.11934	0.06296	-0.04506	-0.08518	-0.0183	0.0183	0.48889	0.00824	0.19804	0.12001	0.41331	0.55844	0.26797	0.81223	0.81223	turquoise
REG4	0.07046	0.09792	0.19242	0.08193	0.08635	0.27703	0.06552	-0.30015	0.30015	0.35976	0.20259	0.01169	0.28671	0.26145	0.00024	0.39453	6.65E-05	6.65E-05	grey
NFKB2	-0.02903	0.00095	0.09716	0.01348	-0.04189	0.20968	0.20914	-0.27266	0.27266	0.70621	0.99018	0.20615	0.86114	0.58646	0.00591	0.00605	0.00031	0.00031	yellow
KLLN	-0.05681	-0.18377	-0.11018	-0.08417	-0.05845	-0.15339	-0.05014	0.37134	-0.37134	0.46052	0.01613	0.15138	0.27374	0.44763	0.04517	0.51486	5.73E-07	5.73E-07	turquoise
BACH1	-0.04177	-0.13262	0.04457	0.09498	-0.11514	0.20402	0.12857	0.09966	-0.09966	0.58751	0.08377	0.56272	0.21656	0.13373	0.00744	0.09376	0.19467	0.19467	turquoise
CSTA	0.07902	0.09738	0.05014	0.01551	-0.07656	0.05984	0.13151	-0.14758	0.14758	0.30425	0.20511	0.51485	0.8404	0.31964	0.43689	0.08643	0.05407	0.05407	grey
TAB1	-0.04848	0.05186	-0.10918	-0.02046	0.01556	-0.04395	-0.00727	0.04848	-0.04848	0.52889	0.50053	0.15518	0.79053	0.83996	0.56819	0.9248	0.52893	0.52893	turquoise
GPC1	0.02396	-0.04994	-0.05239	0.03822	-0.11399	0.04455	0.2236	-0.1201	0.1201	0.75576	0.51659	0.49613	0.61965	0.13767	0.5629	0.00328	0.11765	0.11765	black
SYT17	0.12459	-0.17002	-0.06055	0.03187	0.03786	-0.15462	-0.04763	-0.08714	0.08714	0.10445	0.0262	0.43144	0.67904	0.62296	0.04346	0.5362	0.25707	0.25707	grey
YARS2	-0.04406	-0.00916	0.01647	-0.01057	-0.05377	0.03827	0.10449	-0.26529	0.26529	0.56719	0.90537	0.83066	0.89087	0.48488	0.61923	0.17382	0.00045	0.00045	turquoise
CARMIL2	-0.10485	-0.05207	-0.06765	-0.07572	-0.04274	-0.05445	-0.12662	0.35891	-0.35891	0.17233	0.49882	0.3793	0.32494	0.57886	0.47937	0.0989	1.43E-06	1.43E-06	blue
FBXO25	-0.02776	-0.02527	-0.16127	-0.08122	-0.04026	-0.10636	-0.00429	0.34354	-0.34354	0.71857	0.7429	0.0351	0.29092	0.60109	0.16619	0.95559	4.22E-06	4.22E-06	turquoise
ANPEP	-0.04751	-0.04609	0.03078	0.08916	0.00216	0.1102	0.00514	0.07261	-0.07261	0.53718	0.54946	0.68944	0.24618	0.97762	0.15131	0.9468	0.34529	0.34529	magenta
CLDN4	0.14307	-0.04574	0.10261	0.0338	0.00922	0.2028	0.17565	-0.50005	0.50005	0.06194	0.55246	0.18172	0.66072	0.90476	0.00781	0.02156	3.32E-12	3.32E-12	yellow
C12orf45	0.12119	0.16841	0.17765	0.07271	-0.01208	0.15725	0.16813	-0.28368	0.28368	0.11435	0.02768	0.0201	0.3446	0.87543	0.03997	0.02794	0.00017	0.00017	purple
RASSF2	-0.11986	-0.06988	0.01307	0.06227	-0.20159	0.11767	-0.00488	0.51158	-0.51158	0.11841	0.36375	0.86525	0.41847	0.00819	0.12533	0.94952	8.64E-13	8.64E-13	blue
SLC18B1	-0.08911	-0.07026	-0.03469	0.05141	0.09067	-0.00973	-0.08508	0.16839	-0.16839	0.24647	0.36116	0.65242	0.50431	0.23823	0.89944	0.26857	0.02769	0.02769	turquoise
DHCR24	0.02169	0.05258	0.1132	0.06001	-0.12268	0.29348	0.24965	-0.28669	0.28669	0.77824	0.49461	0.14042	0.43554	0.10992	9.79E-05	0.00099	0.00014	0.00014	yellow
PGK1	-0.02453	-0.13103	0.00366	0.00412	-0.05392	0.0667	0.14816	-0.32297	0.32297	0.75014	0.08758	0.9621	0.95731	0.48364	0.38604	0.05312	1.64E-05	1.64E-05	turquoise
DDX19B	-0.01391	0.01619	-0.02572	-0.08049	-0.05186	0.058	0.12371	-0.19814	0.19814	0.8567	0.83355	0.73841	0.29533	0.50056	0.45116	0.10694	0.00938	0.00938	turquoise
EIF1B	0.02102	-0.21219	-0.13938	-0.14411	-0.04006	-0.00286	-0.10876	0.19565	-0.19565	0.78491	0.00533	0.06904	0.06005	0.60295	0.97042	0.15677	0.01033	0.01033	turquoise
POLR2J	0.08858	-0.00859	-0.0932	-0.09224	0.21265	-0.13552	0.03678	-0.41944	0.41944	0.24929	0.91125	0.22534	0.23016	0.00523	0.07717	0.63291	1.13E-08	1.13E-08	green
SYT4	-0.01828	-0.14145	-0.25072	-0.12639	0.08425	-0.3001	-0.21391	0.44067	-0.44067	0.81238	0.06498	0.00094	0.09951	0.27326	6.67E-05	0.00496	1.62E-09	1.62E-09	brown
ERICH5	-0.00313	-0.08065	-0.10134	0.06407	0.01303	0.09276	-0.04104	0.00376	-0.00376	0.96764	0.29435	0.18722	0.40507	0.86567	0.22756	0.59404	0.96112	0.96112	magenta
SCRIB	0.06368	0.00945	-0.04185	-0.02882	0.12573	-0.05892	0.15154	-0.4018	0.4018	0.40799	0.90234	0.58682	0.70828	0.10131	0.44394	0.04787	5.12E-08	5.12E-08	green
CYTH2	0.1234	-0.07242	-0.04779	-0.05991	0.10458	0.01379	0.04005	-0.36574	0.36574	0.10783	0.34656	0.53478	0.43637	0.17342	0.85792	0.603	8.70E-07	8.70E-07	red
D2HGDH	0.0613	0.04647	-0.05177	-0.04733	0.23489	-0.03498	0.14113	-0.2882	0.2882	0.42578	0.54611	0.50129	0.53873	0.00199	0.64969	0.06559	0.00013	0.00013	green
BCL3	0.06327	-0.02114	0.08196	0.05437	0.01535	0.26499	0.12692	-0.23988	0.23988	0.41104	0.78374	0.28657	0.48003	0.84209	0.00046	0.09808	0.00158	0.00158	yellow
EXOC4	0.05685	-0.09419	0.04737	0.03378	-0.10102	0.08525	0.13868	-0.04491	0.04491	0.4602	0.22044	0.53836	0.66097	0.1886	0.26761	0.07046	0.55976	0.55976	turquoise
SMIM31	0.07589	-0.01477	0.1008	0.06465	0.06778	0.16963	0.01082	-0.29089	0.29089	0.32388	0.84792	0.18957	0.40087	0.37839	0.02656	0.88832	0.00011	0.00011	grey
GPR20	0.01335	0.05028	0.07578	0.01805	0.11186	0.087	0.06062	-0.2142	0.2142	0.86239	0.51373	0.32459	0.81476	0.14524	0.25788	0.43094	0.0049	0.0049	grey
NDC1	-0.08569	0.03393	0.06659	0.0528	-0.13233	0.12811	0.23762	-0.14978	0.14978	0.26514	0.6595	0.38683	0.49277	0.08446	0.09496	0.00175	0.05054	0.05054	pink
MAP1LC3A	0.01928	-0.08814	-0.2007	-0.2496	0.09184	-0.21753	-0.14762	0.10386	-0.10386	0.80237	0.25164	0.00849	0.00099	0.23223	0.00426	0.05401	0.17644	0.17644	grey
NDUFAF6	0.03403	0.02434	-0.12059	-0.12335	0.01116	-0.25158	-0.04713	0.04107	-0.04107	0.65856	0.75201	0.11616	0.10797	0.88486	0.0009	0.54046	0.59376	0.59376	grey
PABPC1L	0.02436	-0.1103	-0.06378	-0.01927	0.1927	-0.09623	0.01607	-0.26506	0.26506	0.75182	0.15096	0.40724	0.80249	0.01157	0.21054	0.8348	0.00046	0.00046	red
PSMD11	-0.1555	-0.16221	-0.0764	0.00073	-0.02042	-0.02534	0.09611	-0.08169	0.08169	0.04227	0.03403	0.3206	0.99243	0.79095	0.74216	0.2111	0.28816	0.28816	turquoise
C11orf58	-0.02513	-0.16877	-0.044	-0.03085	-0.09088	-0.01179	-0.0399	0.2049	-0.2049	0.7442	0.02734	0.5677	0.68872	0.23716	0.87838	0.60432	0.00718	0.00718	turquoise
CES2	-0.03413	0.05697	0.0511	0.01147	0.09766	0.16755	0.07182	-0.18937	0.18937	0.65769	0.45919	0.50683	0.8816	0.20381	0.02849	0.35057	0.01311	0.01311	grey
SLC9A1	-0.00536	-0.0546	0.12678	0.10316	-0.05425	0.28075	0.13537	-0.21135	0.21135	0.94456	0.47811	0.09845	0.17936	0.48095	0.0002	0.07751	0.00552	0.00552	yellow
RENBP	-0.0632	0.04231	-0.06512	-0.03568	-0.15005	0.05617	0.06569	0.23983	-0.23983	0.4115	0.58266	0.39742	0.64311	0.05012	0.46558	0.39329	0.00158	0.00158	blue
IFT80	-0.06925	-0.15463	-0.15331	-0.03489	0.0422	-0.10433	-0.06487	0.13795	-0.13795	0.36813	0.04344	0.0453	0.65056	0.58371	0.17447	0.39924	0.07197	0.07197	turquoise
CPA4	0.02176	-0.0319	-0.0103	0.00437	-0.25218	0.00783	0.1938	0.09399	-0.09399	0.77756	0.67873	0.89358	0.95472	0.00088	0.91908	0.01109	0.22143	0.22143	grey
DLG4	-0.027	-0.22522	-0.16548	-0.08054	0.03729	-0.30841	-0.19734	0.45864	-0.45864	0.72588	0.00306	0.03055	0.295	0.62825	4.06E-05	0.00968	2.82E-10	2.82E-10	brown
TMEM98	0.01199	-0.04131	0.09639	0.08188	0.03824	0.10391	0.00316	0.1458	-0.1458	0.87633	0.59165	0.2098	0.28706	0.61946	0.17621	0.96733	0.05707	0.05707	grey
B4GALT6	-0.10073	-0.09856	-0.13494	-0.09243	0.13066	-0.19275	-0.21737	0.36017	-0.36017	0.18991	0.19968	0.07846	0.22919	0.0885	0.01154	0.00429	1.31E-06	1.31E-06	turquoise
PALD1	-0.13938	-0.04142	-0.04458	0.06355	-0.1986	0.0469	-0.00651	0.4357	-0.4357	0.06904	0.59061	0.5626	0.40894	0.00922	0.54245	0.93266	2.58E-09	2.58E-09	blue
UFSP2	-0.05381	-0.09074	-0.09735	-0.06368	-0.01799	0.02672	-0.06645	0.29494	-0.29494	0.48457	0.2379	0.20528	0.40796	0.81538	0.72863	0.38786	9.00E-05	9.00E-05	turquoise
ENC1	0.0053	0.02764	0.0574	0.05865	-0.18645	0.25811	0.14939	0.01186	-0.01186	0.94515	0.71972	0.45584	0.44609	0.01462	0.00065	0.05115	0.87764	0.87764	turquoise
FAM114A1	-0.04452	-0.14045	0.04826	0.0695	-0.11473	0.12489	0.17726	-0.05514	0.05514	0.56315	0.06691	0.53074	0.36636	0.13513	0.10362	0.02038	0.47384	0.47384	turquoise
APAF1	-0.01752	-0.16847	0.03319	0.00905	0.00544	0.10476	-0.00964	0.16521	-0.16521	0.8201	0.02762	0.66646	0.90652	0.94368	0.1727	0.90045	0.03082	0.03082	turquoise
SLC34A2	0.03578	-0.03487	-0.01052	0.13463	-0.05354	0.14008	0.10092	-0.109	0.109	0.64217	0.65066	0.89135	0.07916	0.48673	0.06764	0.18908	0.15587	0.15587	grey
C2orf68	-0.006	-0.1778	-0.07985	-0.08053	0.00623	0.05086	0.09498	-0.19595	0.19595	0.9379	0.01999	0.29917	0.29509	0.9355	0.50883	0.21655	0.01021	0.01021	red
FAM110C	0.1069	-0.06897	-0.01728	0.03591	0.10113	0.18281	0.09031	-0.37573	0.37573	0.16403	0.37006	0.82254	0.64101	0.18815	0.0167	0.2401	4.11E-07	4.11E-07	grey
TTBK2	-0.09598	-0.19712	-0.1312	-0.043	-0.14527	-0.08702	-0.10164	0.42867	-0.42867	0.21173	0.00976	0.08717	0.57659	0.05799	0.25776	0.18591	4.94E-09	4.94E-09	turquoise
SACM1L	-0.0666	-0.12969	-0.07976	-0.10055	-0.04381	-0.03685	-0.07645	0.32044	-0.32044	0.38678	0.09091	0.29974	0.19069	0.56941	0.63233	0.32028	1.93E-05	1.93E-05	turquoise
MAML2	-0.11128	-0.1366	0.01725	0.06106	-0.26523	0.12974	0.13044	0.09514	-0.09514	0.14733	0.07483	0.82285	0.42759	0.00046	0.09079	0.08905	0.21581	0.21581	turquoise
COCH	-0.10555	-0.00648	-0.06796	-0.02261	0.00383	-0.02989	-0.09603	0.15969	-0.15969	0.16944	0.93297	0.37713	0.76909	0.96033	0.69793	0.21149	0.03695	0.03695	grey
NDP	0.0077	-0.11003	0.01156	0.02663	-0.14166	0.04516	-0.02192	-0.05423	0.05423	0.92041	0.15195	0.88068	0.72954	0.06457	0.55749	0.77601	0.48115	0.48115	grey
LRRC37A3	-0.02733	-0.28476	-0.15442	-0.02105	0.12366	-0.30312	-0.22781	0.26383	-0.26383	0.72274	0.00016	0.04374	0.78461	0.1071	5.58E-05	0.00273	0.00049	0.00049	brown
HSF4	0.0518	-0.03441	0.00517	-0.01223	0.27596	-0.05475	0.02803	-0.32083	0.32083	0.50101	0.65503	0.94648	0.87386	0.00026	0.47692	0.71589	1.88E-05	1.88E-05	red
DOCK5	-0.02954	-0.07079	0.12173	0.04124	-0.07779	0.26069	0.24679	-0.25225	0.25225	0.70134	0.35751	0.11271	0.59225	0.31189	0.00057	0.00114	0.00087	0.00087	turquoise
CCDC163	0.07325	-0.10077	0.01011	0.10752	0.03434	0.08172	0.05624	0.01657	-0.01657	0.34101	0.1897	0.8956	0.16157	0.65568	0.288	0.465	0.82965	0.82965	grey
DIP2B	0.04352	-0.1899	-0.0209	-0.01651	0.0359	-0.0449	-0.11967	0.07992	-0.07992	0.57197	0.01286	0.78609	0.83033	0.6411	0.55979	0.11899	0.29875	0.29875	turquoise
ELMO2	-0.01197	-0.12899	-0.06138	0.00773	-0.06733	-0.08463	-0.12589	0.39697	-0.39697	0.87654	0.09268	0.42518	0.92003	0.38156	0.27109	0.10086	7.63E-08	7.63E-08	turquoise
FCMR	-0.06106	-0.01993	0.05762	0.02724	-0.13501	0.09149	-0.04881	0.38832	-0.38832	0.42759	0.79582	0.45413	0.72359	0.07831	0.23398	0.52607	1.54E-07	1.54E-07	blue
SFXN4	0.10149	0.13401	-0.04832	-0.04917	0.07129	-0.11362	0.14489	-0.33562	0.33562	0.18655	0.08056	0.5303	0.52303	0.35418	0.13898	0.05865	7.21E-06	7.21E-06	grey
CPNE2	-0.19557	-0.14133	0.01487	0.13764	-0.14404	0.09355	0.05139	0.21485	-0.21485	0.01036	0.0652	0.84697	0.07261	0.06016	0.2236	0.50446	0.00477	0.00477	black
WIPF1	-0.10257	-0.04689	0.09318	0.08195	-0.23581	0.16942	0.0409	0.39915	-0.39915	0.18187	0.54254	0.22542	0.28664	0.0019	0.02674	0.59529	6.38E-08	6.38E-08	blue
TIRAP	-0.08943	-0.14549	-0.11555	-0.062	-0.03497	#######	-0.00675	0.16802	-0.16802	0.24476	0.05761	0.13233	0.4205	0.64978	0.99999	0.93016	0.02804	0.02804	turquoise
CKLF	-0.00299	-0.03707	0.06872	-0.00359	-0.05884	0.08008	0.26047	-0.41271	0.41271	0.969	0.6303	0.37179	0.96279	0.44461	0.2978	0.00058	2.03E-08	2.03E-08	yellow
CCDC184	0.00901	-0.16641	-0.16088	-0.05285	0.0503	-0.24264	-0.2436	0.32979	-0.32979	0.90686	0.0296	0.03555	0.49238	0.51354	0.00139	0.00132	1.06E-05	1.06E-05	brown
PNRC2	-0.03991	-0.16864	0.05787	0.04667	-0.08931	0.21283	-0.02681	0.14853	-0.14853	0.60423	0.02746	0.45218	0.5444	0.24539	0.00519	0.72779	0.05253	0.05253	turquoise
POLRMT	0.05433	0.02058	-0.16674	-0.10892	0.2459	-0.15707	0.02958	-0.20728	0.20728	0.48032	0.78937	0.02928	0.15615	0.00119	0.0402	0.7009	0.00652	0.00652	green
ARHGAP5	0.04536	-0.18577	0.00588	0.08621	-0.04711	0.09175	-0.01269	-0.03381	0.03381	0.55577	0.01499	0.93915	0.26222	0.54064	0.23267	0.86918	0.66068	0.66068	turquoise
PPP1R14C	-0.0237	-0.12104	-0.10242	-0.0776	-0.02967	-0.21112	-0.03901	-0.08369	0.08369	0.75836	0.11481	0.18255	0.31308	0.70006	0.00558	0.61244	0.27645	0.27645	grey
ATF2	-0.03533	-0.11114	-0.0283	0.0862	-0.09026	0.03319	-0.04093	0.17404	-0.17404	0.64645	0.14785	0.71332	0.26226	0.24036	0.66651	0.59507	0.02282	0.02282	turquoise
MKRN1	0.05944	-0.12863	-0.01124	-0.04039	0.01954	-0.06002	0.00154	-0.16173	0.16173	0.43993	0.0936	0.88402	0.59993	0.79973	0.43548	0.98401	0.03458	0.03458	turquoise
LRRC45	-0.02621	0.01405	-0.07812	-0.0127	0.17778	-0.0951	0.15004	-0.34881	0.34881	0.73366	0.85526	0.30982	0.86907	0.02	0.216	0.05014	2.93E-06	2.93E-06	green
CLN8	-0.05355	-0.15048	-0.04878	0.02092	0.08336	-0.10062	-0.03355	0.3308	-0.3308	0.48668	0.04947	0.52634	0.78597	0.27835	0.1904	0.66311	9.91E-06	9.91E-06	turquoise
LNX2	0.14931	-0.04835	-0.05724	0.02464	-0.03783	0.12308	0.04133	-0.04876	0.04876	0.05129	0.53001	0.45707	0.74905	0.62321	0.10876	0.59142	0.52653	0.52653	turquoise
FGF2	-0.14431	-0.08495	0.00295	0.0549	-0.18142	0.07381	0.08383	0.23048	-0.23048	0.05968	0.26928	0.96946	0.47577	0.01756	0.33735	0.27568	0.00242	0.00242	turquoise
XPO5	0.03313	-0.02092	-0.05107	0.01636	-0.10764	-0.05692	0.13746	0.03382	-0.03382	0.66712	0.78596	0.50706	0.83181	0.1611	0.45961	0.07299	0.66053	0.66053	turquoise
SORBS3	-0.01079	-0.06956	-0.06403	-0.0091	0.00425	-0.07437	0.01791	0.21353	-0.21353	0.88864	0.36597	0.40541	0.90595	0.95604	0.33366	0.81619	0.00504	0.00504	grey
NTF3	-0.2268	-0.03925	0.04064	0.10133	0.08294	0.08758	0.07045	0.20968	-0.20968	0.00285	0.61025	0.59771	0.18725	0.2808	0.25467	0.35985	0.00592	0.00592	black
ELF4	0.0325	-0.13493	0.11311	0.05753	-0.08856	0.23029	0.14554	-0.14801	0.14801	0.67303	0.07848	0.14074	0.45479	0.24937	0.00244	0.05752	0.05336	0.05336	turquoise
SMCO3	-0.02889	-0.08379	-0.09628	-0.14012	-0.1045	0.02737	-0.12031	0.41623	-0.41623	0.70763	0.27588	0.2103	0.06757	0.17375	0.72229	0.11701	1.50E-08	1.50E-08	grey
PKM	0.02489	-0.08015	-0.01192	0.02256	-0.04235	-0.02332	0.18655	-0.36872	0.36872	0.74659	0.29736	0.877	0.76961	0.58233	0.76203	0.01457	6.97E-07	6.97E-07	turquoise
ELP2	-0.09749	-0.16629	-0.1167	-0.0256	-0.0382	-0.09978	-0.03918	0.35041	-0.35041	0.20459	0.02973	0.12851	0.73963	0.61988	0.19413	0.61094	2.62E-06	2.62E-06	turquoise
RARS2	-0.05354	-0.06152	0.04705	-0.00335	-0.14052	0.06425	0.06249	0.24894	-0.24894	0.48671	0.42411	0.54117	0.96532	0.06678	0.40378	0.41685	0.00103	0.00103	turquoise

SPATA5	-0.08333	-0.08046	0.00891	-0.0611	-0.16139	0.06388	0.11297	-0.01406	0.01406	0.27856	0.29553	0.90796	0.42725	0.03496	0.40647	0.14124	0.85516	0.85516	turquoise
CST4	0.04067	-0.03705	0.01133	0.02124	0.03264	0.15874	0.12205	-0.28809	0.28809	0.59739	0.6304	0.88307	0.7828	0.67171	0.03811	0.11178	0.00013	0.00013	grey
AMTN	0.03139	-0.09855	-0.06517	0.03624	-0.212	0.07173	-0.02977	0.20608	-0.20608	0.68357	0.19972	0.3971	0.63796	0.00537	0.3512	0.6991	0.00685	0.00685	grey
CD177	0.11088	-0.05777	0.08733	0.00175	-0.21484	0.12766	0.04798	0.02639	-0.02639	0.1488	0.45297	0.25606	0.98191	0.00478	0.09613	0.53314	0.73188	0.73188	grey
RPGRIP1L	0.01254	-0.12424	-0.10099	-0.0565	-0.08056	-0.00128	0.07102	0.21387	-0.21387	0.8707	0.10543	0.18876	0.46297	0.2949	0.98676	0.35597	0.00497	0.00497	turquoise
LILRB2	-0.15469	0.01338	0.08899	0.02193	-0.21539	0.11038	0.06523	0.44917	-0.44917	0.04336	0.86211	0.24709	0.77589	0.00467	0.15065	0.39667	7.18E-10	7.18E-10	blue
UQCRHL	0.07353	-0.00555	-0.02778	-0.11429	0.01879	0.00666	0.07852	-0.21896	0.21896	0.33916	0.94252	0.71834	0.13663	0.80724	0.93112	0.30734	0.00401	0.00401	grey
FADS3	-0.04286	-0.07847	-0.07591	0.03968	-0.02339	-0.1731	-0.16682	0.18594	-0.18594	0.5778	0.30763	0.32371	0.60636	0.76136	0.02357	0.0292	0.0149	0.0149	grey
UBE2QL1	-0.02596	-0.16094	-0.13403	-0.01132	0.06545	-0.29633	-0.16008	0.45333	-0.45333	0.73607	0.03548	0.08051	0.8832	0.39508	8.31E-05	0.03649	4.78E-10	4.78E-10	brown
ARMC7	0.00905	-0.11853	-0.09726	-0.01427	0.11652	-0.01641	0.15458	-0.18288	0.18288	0.90651	0.12259	0.20571	0.85302	0.12909	0.83129	0.04352	0.01666	0.01666	grey
CCDC157	-0.1006	-0.08936	-0.27074	-0.05381	0.17402	-0.31684	-0.15913	0.06875	-0.06875	0.19047	0.2451	0.00034	0.48454	0.02283	2.42E-05	0.03762	0.37162	0.37162	grey
ENPP5	-0.0507	-0.1918	-0.22552	-0.01985	-0.04036	-0.18627	-0.18323	0.28662	-0.28662	0.51022	0.01197	0.00302	0.79667	0.60019	0.01472	0.01645	0.00014	0.00014	brown
IGFBP3	-0.009	-0.14539	0.0126	0.09509	-0.14672	0.18166	0.11964	-0.08415	0.08415	0.90697	0.05777	0.87011	0.21605	0.0555	0.01741	0.11909	0.27384	0.27384	black
REEP6	0.08669	0.08181	-0.0729	-0.06437	0.28733	0.06199	0.04557	-0.4167	0.4167	0.25954	0.28744	0.34337	0.40289	0.00014	0.42058	0.55397	1.44E-08	1.44E-08	grey
PIN4	-0.04753	-0.06622	-0.16711	-0.11127	0.01664	-0.13576	0.05463	-0.22487	0.22487	0.53698	0.38952	0.02892	0.14739	0.829	0.07665	0.47791	0.00311	0.00311	grey
SLC25A35	-0.15755	-0.12316	-0.16318	-0.11883	0.07203	-0.08591	-0.07922	0.12051	-0.12051	0.03959	0.10853	0.03297	0.12162	0.34919	0.26387	0.30302	0.11641	0.11641	grey
DNASE2B	-0.10081	-0.01049	-0.03603	-0.03195	-0.04811	-0.04979	-0.12586	0.27528	-0.27528	0.18956	0.89165	0.63989	0.67822	0.5321	0.51784	0.10095	0.00027	0.00027	grey
ZFP41	0.0504	-0.08519	-0.22025	-0.0927	0.10054	-0.29854	0.01004	-0.14038	0.14038	0.5127	0.26794	0.0038	0.22784	0.19075	7.31E-05	0.89628	0.06705	0.06705	grey
STAMBPL1	0.01384	-0.09488	0.14807	-0.01098	-0.00967	0.15185	0.07502	-0.12842	0.12842	0.85746	0.21705	0.05327	0.88668	0.90013	0.04741	0.32948	0.09414	0.09414	turquoise
THAP9	-0.15605	-0.16837	-0.17342	-0.083	0.0683	-0.09915	-0.14285	0.20444	-0.20444	0.04153	0.02771	0.0233	0.28049	0.37476	0.19699	0.06234	0.00731	0.00731	turquoise
ARHGEF7	-0.01037	-0.1166	-0.03204	0.03245	0.02992	-0.04356	-0.04117	0.11618	-0.11618	0.89291	0.12881	0.67737	0.67351	0.69767	0.5716	0.59293	0.13022	0.13022	turquoise
CD48	-0.0923	-0.0091	0.05821	0.04425	-0.15693	0.14162	-0.0455	0.39514	-0.39514	0.22989	0.90595	0.44948	0.56555	0.04038	0.06466	0.55455	8.87E-08	8.87E-08	blue
DMRTA1	-0.0022	0.07732	-0.03731	0.06284	-0.05897	0.19222	0.09614	-0.10393	0.10393	0.97722	0.3148	0.62806	0.41417	0.44356	0.01178	0.211	0.17612	0.17612	grey
GZMA	-0.08445	0.0556	0.12903	0.14111	-0.17135	0.11416	0.04482	0.28475	-0.28475	0.27213	0.47012	0.09257	0.06564	0.02504	0.13709	0.56052	0.00016	0.00016	blue
KCNIP1	-0.01635	-0.12477	-0.06273	-0.06092	-0.00375	-0.15786	-0.14143	0.43824	-0.43824	0.83192	0.10396	0.41504	0.42863	0.9612	0.0392	0.06502	2.04E-09	2.04E-09	brown
MIS12	-0.07384	-0.20502	-0.15229	-0.04732	0.06167	-0.14691	-0.10774	0.34779	-0.34779	0.33717	0.00715	0.04676	0.53884	0.42294	0.05518	0.16072	3.15E-06	3.15E-06	turquoise
FUCA1	-0.05556	-0.12841	0.08941	0.02676	-0.04896	0.16035	0.02053	-0.00829	0.00829	0.47046	0.09418	0.24483	0.72825	0.5248	0.03617	0.78979	0.91432	0.91432	turquoise
ADCY5	0.01312	-0.07924	-0.09061	0.08893	-0.02569	0.061	-0.05499	0.38095	-0.38095	0.86476	0.30288	0.23853	0.24742	0.73877	0.42801	0.47501	2.74E-07	2.74E-07	grey
NCAPG2	-0.10241	-0.01669	0.06706	-0.01839	-0.09692	0.04759	0.21314	-0.18736	0.18736	0.18258	0.82845	0.38347	0.81127	0.20729	0.53653	0.00513	0.01413	0.01413	pink
ZNF467	0.02391	-0.13257	-0.25283	-0.07945	0.15346	-0.34415	-0.22406	0.22744	-0.22744	0.75623	0.08391	0.00085	0.30164	0.04508	4.05E-06	0.00322	0.00277	0.00277	brown
ALKBH1	0.02323	-0.1558	-0.12808	-0.00445	0.05641	-0.05304	-0.11447	0.14111	-0.14111	0.76301	0.04186	0.09503	0.95398	0.46364	0.49082	0.13602	0.06562	0.06562	turquoise
PRPF31	0.04766	0.05106	-0.13187	-0.06186	0.06599	-0.09284	0.09917	-0.26081	0.26081	0.53593	0.50715	0.08557	0.42156	0.39112	0.22715	0.19687	0.00057	0.00057	green
NAA50	-0.01567	-0.02955	0.01047	0.01003	-0.18356	0.16865	0.19052	-0.05893	0.05893	0.83876	0.70126	0.89192	0.89644	0.01625	0.02745	0.01256	0.44388	0.44388	turquoise
MRPS18A	0.09583	0.10545	-0.04415	-0.00743	0.0855	0.03057	0.0896	-0.30558	0.30558	0.21248	0.16986	0.56642	0.92319	0.26621	0.69143	0.24384	4.82E-05	4.82E-05	grey
CLDN5	-0.02092	-0.09372	-0.08861	0.05757	0.03028	-0.03665	-0.10743	0.27261	-0.27261	0.78594	0.22276	0.24911	0.45453	0.69418	0.63415	0.16195	0.00031	0.00031	blue
PLCXD2	0.07695	-0.06046	-0.3636	-0.23706	0.10281	-0.30392	-0.22366	0.10757	-0.10757	0.31711	0.43214	1.02E-06	0.0018	0.18084	5.32E-05	0.00328	0.1614	0.1614	brown
TFEB	0.01091	-0.0173	-0.12754	-0.03435	-0.00726	-0.09762	-0.05952	0.15985	-0.15985	0.88743	0.82231	0.09645	0.65558	0.92493	0.20401	0.43931	0.03676	0.03676	grey
CXCL8	-0.16909	-0.11564	0.0312	0.00885	-0.15094	0.17057	0.07027	0.06818	-0.06818	0.02705	0.13204	0.68537	0.90856	0.04876	0.02571	0.36108	0.3756	0.3756	black
SLC25A45	-0.07151	-0.0715	0.00101	-0.06883	-0.03308	0.02729	-0.16924	0.25413	-0.25413	0.35266	0.35272	0.98959	0.37102	0.6675	0.72314	0.02691	0.0008	0.0008	blue
FEM1B	-0.07143	-0.18404	-0.10395	-0.00406	-0.10092	-0.02099	-0.05229	0.29738	-0.29738	0.35318	0.01597	0.17607	0.95799	0.18906	0.78529	0.497	7.82E-05	7.82E-05	turquoise
RAB11FIP4	0.04634	0.00397	-0.0541	-0.06078	0.12603	0.12212	0.01029	-0.21272	0.21272	0.54731	0.95895	0.48216	0.42972	0.10049	0.11158	0.89372	0.00522	0.00522	grey
CENPK	-0.04846	0.07999	0.05406	0.01792	-0.00374	0.0456	0.2401	-0.38896	0.38896	0.52911	0.29832	0.48249	0.81608	0.96133	0.5537	0.00156	1.46E-07	1.46E-07	pink
HNF4A	0.08245	0.03335	-0.01539	-0.00274	0.26686	0.05075	-0.11624	-0.17196	0.17196	0.28368	0.66495	0.84166	0.9716	0.00042	0.50981	0.13003	0.02452	0.02452	grey
ZEB2	-0.11861	-0.11374	0.01737	0.10774	-0.21416	0.07371	0.02727	0.53542	-0.53542	0.12231	0.13855	0.82156	0.16074	0.00491	0.33801	0.72334	4.52E-14	4.52E-14	blue
LCN12	0.00807	-0.07755	-0.18565	-0.15441	0.24553	-0.14852	-0.09454	-0.04151	0.04151	0.91652	0.31334	0.01505	0.04375	0.00121	0.05254	0.21872	0.58981	0.58981	grey
PRR13	0.14256	-0.02579	0.04641	-0.00736	0.08114	0.10911	0.055	-0.4045	0.4045	0.06288	0.73775	0.54668	0.92389	0.29144	0.15545	0.47495	4.09E-08	4.09E-08	yellow
SCNN1D	-0.09729	-0.08702	-0.11603	0.00594	0.23369	-0.10144	-0.08325	0.02556	-0.02556	0.20556	0.25776	0.13073	0.93849	0.0021	0.18676	0.27902	0.73999	0.73999	red
SNU13	-0.02704	-0.01598	-0.17225	-0.11406	0.09652	-0.2658	-0.07563	-0.06193	0.06193	0.72551	0.83565	0.02427	0.13741	0.20919	0.00044	0.32557	0.421	0.421	grey
CCDC126	-0.0529	-0.10436	-0.0537	0.01863	0.01832	-0.01508	-0.14469	0.33763	-0.33763	0.49196	0.17436	0.48545	0.80886	0.81201	0.84477	0.05901	6.30E-06	6.30E-06	turquoise
STAP1	-0.09465	-0.01716	0.09972	0.03853	-0.10142	0.08267	-0.12106	0.32651	-0.32651	0.2182	0.82372	0.19439	0.61682	0.18687	0.28238	0.11473	1.31E-05	1.31E-05	blue
USPL1	-0.02182	-0.22663	-0.08924	-0.02825	-0.00985	-0.07889	-0.11195	0.1671	-0.1671	0.77692	0.00288	0.24575	0.71374	0.89825	0.30504	0.1449	0.02893	0.02893	red
DMKN	0.05624	-0.00988	-0.09389	0.02088	-0.03128	-0.04275	0.15788	-0.35545	0.35545	0.46503	0.89794	0.22193	0.7863	0.68462	0.57874	0.03918	1.83E-06	1.83E-06	grey
GDPD2	0.01113	0.04105	0.15071	-0.01487	0.16687	0.10642	0.09605	-0.33403	0.33403	0.88517	0.59396	0.04912	0.84691	0.02915	0.16595	0.2114	8.01E-06	8.01E-06	grey
THAP7	-0.05701	-0.0106	-0.24858	-0.12417	0.22457	-0.29407	-0.1015	0.0876	-0.0876	0.45893	0.8906	0.00104	0.10565	0.00315	9.46E-05	0.18651	0.25459	0.25459	green
DNAJC13	-0.03316	-0.06156	0.01653	0.05468	-0.19979	0.19451	0.20698	0.06932	-0.06932	0.66679	0.42381	0.83013	0.47753	0.00879	0.0108	0.0066	0.36762	0.36762	turquoise
TMEM68	0.00081	0.00343	-0.09923	-0.04685	0.06786	-0.20684	-0.00885	-0.10511	0.10511	0.99159	0.96447	0.1966	0.54284	0.37786	0.00664	0.90854	0.17125	0.17125	turquoise
MRPL51	0.04013	-0.02169	-0.0609	-0.01543	0.05316	-0.1506	0.04087	-0.2208	0.2208	0.60224	0.77823	0.42878	0.84125	0.48983	0.04928	0.59557	0.00371	0.00371	grey
LRRC49	-0.03399	-0.22309	-0.21905	-0.15746	0.00528	-0.20892	-0.2233	0.35893	-0.35893	0.65899	0.00336	0.004	0.0397	0.94533	0.0061	0.00333	1.43E-06	1.43E-06	brown
PCDHGB6	-0.13716	-0.26294	-0.00702	0.02961	-0.13866	0.07056	0.06832	0.43966	-0.43966	0.07363	0.00051	0.92743	0.70065	0.07051	0.35909	0.37463	1.78E-09	1.78E-09	blue
C4orf48	0.07766	-0.05664	-0.16515	-0.04277	0.19775	-0.20562	-0.01278	-0.24638	0.24638	0.31271	0.46184	0.03088	0.57864	0.00952	0.00698	0.86822	0.00116	0.00116	green
ANGEL2	-0.06584	-0.10332	-0.10048	-0.03929	0.03371	-0.03966	0.00303	0.12522	-0.12522	0.39221	0.17869	0.19101	0.6099	0.66155	0.60657	0.96864	0.10269	0.10269	turquoise
PDK2	0.03409	-0.04808	-0.08425	-0.03352	0.09263	0.03718	-0.12944	0.15813	-0.15813	0.65799	0.53229	0.27324	0.66342	0.22822	0.62926	0.09153	0.03886	0.03886	grey
PLPP3	-0.14475	-0.15711	0.02046	0.1137	-0.12842	0.00837	-0.10426	0.60968	-0.60968	0.0589	0.04015	0.79055	0.13868	0.09415	0.91352	0.17474	8.76E-19	8.76E-19	blue
MYMX	-0.04185	0.09755	0.0825	0.14316	-0.06642	0.20357	-0.07261	0.23438	-0.23438	0.58679	0.20432	0.28339	0.06177	0.38808	0.00757	0.34527	0.00203	0.00203	magenta
POLR2L	0.10712	0.00346	-0.08217	-0.12092	0.0972	-0.07227	0.09823	-0.24296	0.24296	0.16316	0.96422	0.28532	0.11517	0.20597	0.34754	0.20116	0.00137	0.00137	grey
FBLN2	-0.06507	0.01798	0.05265	0.05753	-0.19523	0.14131	0.05156	0.39505	-0.39505	0.39781	0.81545	0.49402	0.45482	0.0105	0.06524	0.50305	8.93E-08	8.93E-08	black
IFRD2	0.03089	0.10697	0.05005	-0.0769	0.02176	0.17055	0.13752	-0.39744	0.39744	0.68832	0.16376	0.51559	0.31745	0.77756	0.02573	0.07288	7.35E-08	7.35E-08	grey
MED13L	-0.04609	-0.13806	0.01667	0.05702	-0.14325	0.06458	-0.01613	0.37234	-0.37234	0.54944	0.07174	0.82867	0.45886	0.0616	0.40139	0.83411	5.31E-07	5.31E-07	turquoise
PROCA1	-0.03136	-0.19038	-0.08866	-0.06641	0.11962	-0.08532	-0.05415	-0.01936	0.01936	0.68384	0.01263	0.24883	0.38814	0.11915	0.26722	0.48183	0.80153	0.80153	red
NMNAT3	0.01808	0.05438	-0.18164	-0.09445	0.08936	-0.08692	-0.09445	0.25673	-0.25673	0.81444	0.47992	0.01743	0.21914	0.24511	0.25829	0.21914	0.0007	0.0007	grey
RFC1	-0.10058	-0.10843	-0.11703	-0.01613	-0.17888	-0.03182	0.01672	0.23759	-0.23759	0.19056	0.15806	0.12741	0.83412	0.01924	0.67947	0.82813	0.00175	0.00175	turquoise
TNIK	0.04435	-0.07385	0.08543	0.05246	-0.06017	0.21317	0.05103	-0.14698	0.14698	0.56463	0.3371	0.26657	0.49559	0.43433	0.00512	0.50742	0.05506	0.05506	turquoise
RB1	-0.03056	-0.04643	-0.01219	0.00185	-0.07815	0.07009	-0.01352	0.22753	-0.22753	0.69153	0.54651	0.87431	0.98082	0.30962	0.36231	0.86065	0.00276	0.00276	turquoise
SCG2	0.02833	-0.14016	-0.2594	-0.15895	0.04746	-0.29759	-0.19794	0.50445	-0.50445	0.71303	0.06749	0.00061	0.03784	0.53761	7.72E-05	0.00945	2.00E-12	2.00E-12	brown
SPOPL	0.01244	-0.11308	0.03999	0.06461	-0.09766	0.08913	-0.00394	0.08635	-0.08635	0.87167	0.14088	0.6035	0.40114	0.20383	0.24632	0.95923	0.26144	0.26144	turquoise
ANXA8	0.04442	-0.04491	0.10991	0.07495	-0.11681	0.08109	0.19959	-0.27097	0.27097	0.56399	0.55975	0.15241	0.32994	0.12815	0.2917	0.00887	0.00034	0.00034	grey
EIF2B4	0.03639	-0.08997	-0.04233	0.01299	-0.05895	0.01344	0.16551	-0.09403	0.09403	0.63659	0.24191	0.58253	0.86611	0.44374	0.86154	0.03052	0.22124	0.22124	turquoise
ITGA3	0.02225	-0.09503	0.06072	0.03974	-0.05505	0.10293	0.21676	-0.42735	0.42735	0.77268	0.21633	0.43012	0.6058	0.4745	0.18037	0.0044	5.56E-09	5.56E-09	yellow
SNTB2	-0.05429	-0.1394	0.0571	0.09134	-0.21984	0.16959	0.13024	0.22223	-0.22223	0.48069	0.06901	0.45824	0.23478	0.00386	0.02659	0.08952	0.00349	0.00349	turquoise
PNPLA2	0.15297	-0.01285	0.02976	-0.10213	0.16322	0.11612	0.09811	-0.40461	0.40461	0.04577	0.86756	0.69917	0.18376	0.03292	0.13043	0.20173	4.05E-08	4.05E-08	green
ARHGEF4	0.15353	0.102	-0.04541	0.02747	-0.02473	-0.15085	0.04514	0.0289	-0.0289	0.04498	0.18434	0.55531	0.72136	0.74816	0.04891	0.55772	0.70752	0.70752	grey
PRKD3	-0.07303	-0.12208	0.01228	0.03783	-0.20139	0.12706	0.06138	0.39583	-0.39583	0.34249	0.11169	0.8734	0.62329	0.00826	0.09771	0.42516	8.39E-08	8.39E-08	blue
PRKAB2	0.11085	-0.06191	0.0051	0.011	-0.06247	0.09956	0.07761	0.07216	-0.07216	0.14892	0.42118	0.94717	0.88648	0.41696	0.1951	0.31299	0.34828	0.34828	turquoise
SAMD12	0.07923	-0.08229	0.00129	0.02516	-0.01538	0.10101	0.08534	-0.18947	0.18947	0.30297	0.28462	0.98667	0.74397	0.84179	0.18868	0.26709	0.01307	0.01307	turquoise
PRSS2	0.08604	0.09669	0.13022	0.106	-0.07192	0.13607	-0.06998	0.02967	-0.02967	0.26318	0.20835	0.08959	0.16763	0.34988	0.07597	0.36308	0.70009	0.70009	magenta
CD28	-0.09373	-0.0486	0.05938	0.0425	-0.18218	0.1217	-0.04254	0.51392	-0.51392	0.22271	0.52785	0.44039	0.58098	0.01709	0.11281	0.58064	6.53E-13	6.53E-13	blue
ZNF543	-0.0259	-0.20046	-0.13126	-0.0054	-0.0463	0.00311	-0.03334	0.28247	-0.28247	0.73665	0.00857	0.08704	0.94412	0.54764	0.96777	0.66506	0.00018	0.00018	turquoise
NVL	0.10258	0.00255	-0.03643	-0.02265	0.00731	-0.01759	0.05997	-0.06109	0.06109	0.18183	0.97357	0.63617	0.76868	0.9244	0.81939	0.43589	0.42738	0.42738	turquoise
MAN1A1	-0.08004	-0.14898	-0.05116	0.03433	-0.04616	-0.13653	-0.19558	0.57177	-0.57177	0.29806	0.05181	0.50633	0.65578	0.54883	0.07498	0.01036	3.14E-16	3.14E-16	turquoise
TRIM38	-0.01732	-0.02599	0.0669	0.10513	-0.11513	0.2279	0.17188	0.00299	-0.00299	0.8221	0.73575	0.38464	0.17118	0.13375	0.00272	0.02458	0.96903	0.96903	turquoise
SHMT2	0.09334	-0.02178	-0.02353	-0.0315	-0.06161	-0.02857	0.06079	-0.21929	0.21929	0.22465	0.77736	0.76	0.68258	0.42341	0.71073	0.42964	0.00395	0.00395	grey
TMEM14B	-0.0976	0.06377	0.02523	0.00239	0.06657	0.02172	0.03764	-0.08479	0.08479	0.20409	0.40732	0.74322	0.9753	0.38702	0.77794	0.62498	0.2702	0.2702	grey
DRD1	-0.09821	-0.08359	-0.05409	0.00408	0.01964	0.01568	-0.05672	0.02849	-0.02849	0.20126	0.27703	0.48229	0.95776	0.79872	0.8387	0.46121	0.71145	0.71145	grey
TMEM170	0.00153	-0.19305	0.06244	0.03006	0.00522	0.14627	0.016	-0.08075	0.08075	0.9842	0.01141	0.41722	0.69634	0.94595	0.05627	0.83551	0.29377	0.29377	turquoise
TK1	-0.00595	0.103	0.08988	0.01381	-0.07826	0.14191	0.35356	-0.5121	0.5121	0.93842	0.18006	0.24235	0.85776	0.30897	0.06409	2.10E-06	8.12E-13	8.12E-13	pink
SP100	-0.01518	-0.04705	0.10159	0.13708	-0.19211	0.29344	0.18235	-0.12361	0.12361	0.84383	0.54117	0.18613	0.0738	0.01183	9.81E-05	0.01698	0.10724	0.10724	turquoise
ZNF605	-0.00759	-0.16317	-0.07639	-0.07082	0.04675	-0.05313	-0.05008	0.19537	-0.19537	0.9215	0.03298	0.32065	0.35735	0.54369	0.49011	0.51537	0.01045	0.01045	turquoise
VARS	0.03962	0.13877	-0.06316	-0.07181	0.05619	-0.07854	0.12029	-0.25284	0.25284	0.60686	0.07027	0.41183	0.35061	0.46541	0.30724	0.11708	0.00085	0.00085	grey
ZFR	-0.02353	-0.16101	-0.05831	0.02285	-0.07661	-0.02532	-0.02651	0.26852	-0.26852	0.75999	0.03539	0.4487	0.76672	0.31928	0.74234	0.73067	0.00038	0.00038	turquoise
TMEM199	-0.08359	-0.12113	-0.19498	-0.10493	0.12341	-0.26627	-0.13323	0.07154	-0.07154	0.27705	0.11453	0.0106	0.17197	0.10782	0.00043	0.08236	0.35246	0.35246	turquoise
ZBTB16	-0.02708	-0.10187	-0.08238	0.04548	-0.16488	-0.0185	-0.11764	0.40813	-0.40813	0.7251	0.1849	0.28412	0.55474	0.03115	0.81023	0.12544	3.01E-08	3.01E-08	blue
GUK1	0.00524	0.12641	-0.06521	-0.03204	0.04073	0.00125	0.19169	-0.42634	0.42634	0.94581	0.09944	0.39677	0.67736	0.59683	0.98708	0.01202	6.10E-09	6.10E-09	green
DDX5	-0.09088	-0.21807	-0.05979	-0.07783	0.07216	-0.08649	-0.10775	0.26798	-0.26798	0.23714	0.00417	0.43725	0.31161	0.34828	0.26068	0.1607	0.00039	0.00039	red
ATG7	-0.07223	-0.00485	0.06455	-0.00351	-0.27473	0.20098	0.28597	0.0101	-0.0101	0.34783	0.9498	0.40158	0.96371	0.00028	0.00839	0.00015	0.8957	0.8957	turquoise
GIPR	0.10061	-0.18016	-0.18711	-0.1428	0.26475	-0.25075	-0.15197	-0.00293	0.00293	0.19044	0.01838	0.01426	0.06243	0.00047	0.00094	0.04723	0.96969	0.96969	brown
HSPB2	-0.01343	-0.02627	-0.01836	0.03846	-0.05398	-0.00722	-0.08238	0.30824	-0.30824	0.86159	0.73309	0.81158	0.61748	0.4832	0.92538	0.28411	4.10E-05	4.10E-05	blue
MSI1	-0.02562	-0.18519	-0.24611	-0.09812	0.15509	-0.36592	-0.24945	0.4387	-0.4387	0.73942	0.01531	0.00118	0.20169	0.04282	8.58E-07	0.001	1.95E-09	1.95E-09	brown
TNFRSF12	-0.00206	-0.11787	0.0609	0.05892	-0.13992	0.23692	0.19145	-0.31937	0.31937	0.97866	0.12468	0.42878	0.44395	0.06796	0.00181	0.01213	2.07E-05	2.07E-05	yellow
TBC1D8B	-0.05114	-0.14334	0.0352	0.04145	-0.02993	0.15016	0.0723	0.07963	-0.07963	0.50648	0.06143	0.64761	0.59041	0.69757	0.04996	0.34734	0.30052	0.30052	turquoise
DERL2	-0.08438	-0.15154	-0.18358	-0.06462	0.00917	-0.24735	-0.08228	0.2702	-0.2702	0.27253	0.04787	0.01624	0.40111	0.90529	0.00111	0.2847	0.00035	0.00035	grey
BTF3	0.08597	0.06363	-0.02668	-0.07979	-0.06088	0.10446	0.11714	-0.26749	0.26749	0.26357	0.40833	0.72903	0.29953	0.42892	0.17391	0.12705	0.0004	0.0004	turquoise
JAZF1	-0.08089	-0.21745	-0.10657	0.05399	-0.03181	-0.14546	-0.25489	0.45394	-0.45394	0.29293	0.00428	0.16533	0.48313	0.67956	0.05766	0.00077	4.50E-10	4.50E-10	turquoise
NEURL3	0.05095	0.00509	-0.02583	0.16178	-0.03102	0.15463	0.07386	0.04523	-0.04523	0.50807	0.94735	0.73737	0.03452	0.68716	0.04345	0.33699	0.55693	0.55693	magenta
CBLC	0.14973	0.0859	0.04205	0.00136	0.15042	0.14241	0.21994	-0.55511	0.55511	0.05062	0.26393	0.58498	0.98594	0.04955	0.06315	0.00385	3.30E-15	3.30E-15	yellow
SNX3	-0.07255	-0.05669	0.07627	0.06753	-0.1584	0.053	0.00818	0.31706	-0.31706	0.34565	0.46144	0.32146	0.38017	0.03852	0.49118	0.91549	2.39E-05	2.39E-05	blue
CYP2C9	-0.07349	0.00396	0.07696	0.04963	0.24466	0.15417	0.05882	-0.24652	0.24652	0.33945	0.959	0.31707	0.51915	0.00126	0.04408	0.44474	0.00115	0.00115	grey
NMB	0.1396	-0.05585	-0.05867	-0.07634	-0.00173	-0.0589	0.05013	-0.33568	0.33568	0.06859	0.46811	0.44592	0.32101	0.98211	0.44413	0.51497	7.18E-06	7.18E-06	grey
ZNF81	-0.01813	-0.15508	-0.08722	-0.01315	-0.1023	0.03039	-0.00521	0.27341	-0.27341	0.81393	0.04284	0.25666	0.8645	0.18303	0.69317	0.94611	0.0003	0.0003	turquoise
RASIP1	-0.08302	-0.02588	-0.10972	0.03691	0.01363	-0.07667	-0.10416	0.47292	-0.47292	0.28033	0.73682	0.15314	0.63171	0.85957	0.3189	0.17518	6.51E-11	6.51E-11	blue
FBXL17	-0.00546	-0.17233	-0.16941	-0.09099	0.02114	-0.16474	-0.14689	0.3836	-0.3836	0.94346	0.0242	0.02675	0.23658	0.78377	0.03131	0.05522	2.23E-07	2.23E-07	turquoise
USP47	-0.04931	-0.18396	-0.02809	-0.00674	-0.0633	0.02044	0.0083	0.22291	-0.22291	0.52184	0.01602	0.71531	0.93025	0.41079	0.79075	0.91416	0.00338	0.00338	turquoise
MYO18A	0.01539	-0.07563	-0.00727	0.04025	0.08323	0.04714	-0.02133	0.09612	-0.09612	0.84163	0.32554	0.92483	0.60123	0.27914	0.54037	0.78187	0.2111	0.2111	turquoise
PSMD10	-0.04472	-0.21055	-0.07577	-0.03257	-0.00942	-0.07516	-0.02326	0.00119	-0.00119	0.56134	0.00571	0.32465	0.67237	0.90269	0.32858	0.76267	0.98766	0.98766	turquoise
MRM1	0.01969	-0.004	-0.17979	-0.03871	0.244	-0.21957	0.00437	-0.14922	0.14922	0.79824	0.95855	0.01862	0.61523	0.0013	0.00391	0.95479	0.05143	0.05143	grey
MBIP	-0.02531	-0.22057	0.03045	0.09698	0.04545	0.0824	-0.06401	-0.01316	0.01316	0.74245	0.00374	0.69259	0.207	0.55501	0.28395	0.40557	0.86433	0.86433	turquoise
NCAPD2	-0.02966	-0.04063	-0.00141	-0.00392	-0.02788	0.0173	0.11403	-0.14632	0.14632	0.70015	0.59775	0.98541	0.95938	0.71737	0.82229	0.13752	0.05619	0.05619	pink
NUP54	-0.09937	-0.08368	0.06013	0.03622	-0.06614	0.13534	0.08833	-0.0212	0.0212	0.19598	0.27651	0.43463	0.63816	0.3901	0.07756	0.25061	0.78318	0.78318	turquoise
MUC5AC	0.08938	0.06921	0.16309	0.02309	0.10637	0.26318	0.15358	-0.37819	0.37819	0.24499	0.36837	0.03306	0.76436	0.16616	0.00051	0.04491	3.40E-07	3.40E-07	yellow
CASP9	-0.06628	-0.12878	0.06018	0.0333	-0.11748	0.24995	-0.08204	0.08376	-0.08376	0.38909	0.09322	0.43431	0.66543	0.12594	0.00098	0.28607	0.27609	0.27609	grey

CCND2	-0.03609	-0.04645	-0.08411	-0.12411	0.04531	-0.14758	-0.32096	0.44137	-0.44137	0.63937	0.54632	0.27406	0.10581	0.5562	0.05406	1.87E-05	1.52E-09	1.52E-09	grey
PSMB10	0.02248	0.13026	0.03021	-0.00193	0.04027	0.17372	0.25586	-0.40321	0.40321	0.77036	0.08947	0.69487	0.97998	0.60104	0.02306	0.00073	4.55E-08	4.55E-08	tan
CENPJ	0.03458	0.01354	-0.04749	-0.03012	-0.07661	-0.0367	0.12781	-0.02063	0.02063	0.65346	0.86043	0.5374	0.69575	0.31932	0.63366	0.09574	0.78879	0.78879	turquoise
LRATD1	0.07299	-0.079	0.0195	0.03822	0.12702	0.04277	-0.00947	-0.03092	0.03092	0.34277	0.3044	0.80016	0.61965	0.09783	0.57862	0.90215	0.68804	0.68804	grey
MSS51	0.0022	-0.12828	-0.06806	-0.02121	0.09137	-0.1524	-0.06593	-0.0017	0.0017	0.97717	0.09451	0.37645	0.78306	0.23463	0.0466	0.39155	0.98243	0.98243	red
PGM2L1	0.00769	-0.14739	0.1171	0.10719	-0.20877	0.20671	0.10218	-0.13219	0.13219	0.92049	0.05438	0.12717	0.16289	0.00614	0.00668	0.18356	0.0848	0.0848	turquoise
GLRX5	-0.00902	-0.08688	-0.09248	0.00982	0.03985	-0.06774	0.04091	-0.16139	0.16139	0.90676	0.2585	0.22894	0.89859	0.60454	0.37868	0.59524	0.03497	0.03497	grey
KAT14	-0.00549	-0.02412	-0.19929	-0.0586	0.16706	-0.21911	-0.10291	0.23498	-0.23498	0.94323	0.75419	0.00897	0.44648	0.02897	0.00399	0.18042	0.00198	0.00198	turquoise
SLC25A43	0.03986	-0.10915	0.09681	0.15029	-0.15753	0.11821	0.15518	-0.16211	0.16211	0.60476	0.15529	0.2078	0.04976	0.03961	0.12359	0.0427	0.03414	0.03414	turquoise
KMT5B	-0.02067	-0.13341	-0.07409	-0.02817	-0.00706	0.04019	-0.02905	0.04289	-0.04289	0.78847	0.08193	0.33548	0.71454	0.92702	0.60169	0.70607	0.57757	0.57757	turquoise
RNF32	0.00982	-0.08536	0.01755	0.00427	0.10846	-0.07114	0.03546	-0.16908	0.16908	0.89852	0.26699	0.81983	0.95578	0.15792	0.35516	0.64524	0.02706	0.02706	grey
TLE3	0.0536	-0.0991	0.03052	0.04867	-0.05492	0.11667	0.09174	-0.12582	0.12582	0.48625	0.1972	0.69189	0.52732	0.47554	0.12859	0.23274	0.10106	0.10106	grey
ACSF2	-0.04475	-0.11944	0.05038	0.10476	-0.10713	0.21577	0.20493	-0.25082	0.25082	0.56114	0.1197	0.51289	0.17268	0.16312	0.00459	0.00717	0.00094	0.00094	yellow
RTN3	0.03067	-0.14833	-0.02203	0.04332	-0.11715	-0.00593	0.04398	-0.12477	0.12477	0.6905	0.05285	0.77489	0.57375	0.12703	0.93867	0.56785	0.10395	0.10395	turquoise
GTPBP10	-0.00969	-0.16516	-0.01117	-0.00666	0.06244	0.00208	0.00399	-0.08567	0.08567	0.89987	0.03087	0.88471	0.9311	0.41715	0.97847	0.95867	0.26525	0.26525	turquoise
BMP2K	-0.1157	-0.02798	0.08984	0.04697	-0.22036	0.13177	0.06567	0.40642	-0.40642	0.13182	0.71645	0.2426	0.54188	0.00378	0.08579	0.39347	3.48E-08	3.48E-08	blue
CLIP3	-0.02515	-0.1704	-0.1471	-0.07012	0.00591	-0.25014	-0.22634	0.52327	-0.52327	0.744	0.02586	0.05486	0.36214	0.9388	0.00097	0.00291	2.09E-13	2.09E-13	brown
RTL5	0.0089	-0.20264	-0.20229	-0.08775	-0.0014	-0.23304	-0.20875	0.56794	-0.56794	0.90804	0.00786	0.00797	0.25378	0.9855	0.00216	0.00614	5.46E-16	5.46E-16	brown
EDEM1	-0.04459	-0.13088	0.05192	-0.02909	-0.21082	0.13556	0.06677	0.11613	-0.11613	0.56255	0.08797	0.50006	0.70567	0.00564	0.07708	0.38553	0.13039	0.13039	turquoise
CLNS1A	0.0514	-0.01163	-0.00393	-0.07778	-0.12408	0.10684	0.19626	-0.2888	0.2888	0.50437	0.87996	0.95934	0.31195	0.10588	0.16427	0.01009	0.00013	0.00013	turquoise
ATG9A	0.05821	-0.12438	-0.10553	0.02733	0.04211	-0.06988	0.01675	-0.06591	0.06591	0.44949	0.10504	0.16954	0.72272	0.58447	0.36378	0.8279	0.39172	0.39172	turquoise
INPP5J	-0.00978	0.06306	-0.20443	-0.08035	0.25301	-0.10424	-0.11597	-0.09105	0.09105	0.89901	0.4126	0.00732	0.29618	0.00084	0.17486	0.13093	0.23627	0.23627	grey
BCAP29	-0.05253	-0.16445	0.03364	0.02464	-0.12556	-0.01516	0.03198	0.15636	-0.15636	0.49502	0.03161	0.66228	0.7491	0.10176	0.84394	0.67794	0.04113	0.04113	turquoise
MRPL53	-0.06527	-0.07256	-0.16116	-0.13682	0.19645	-0.23542	-0.01318	-0.105	0.105	0.39634	0.34561	0.03522	0.07436	0.01002	0.00194	0.86411	0.17172	0.17172	red
PRR29	-0.10227	-0.22934	-0.10857	-0.00271	0.0262	-0.15624	-0.16013	0.17343	-0.17343	0.18317	0.00255	0.1575	0.97198	0.73378	0.04129	0.03643	0.0233	0.0233	grey
VPS39	-0.07218	-0.16848	-0.09499	-0.03301	-0.11786	-0.04317	-0.04892	0.21746	-0.21746	0.34813	0.02761	0.21652	0.66819	0.12472	0.57504	0.52515	0.00428	0.00428	turquoise
KCTD11	-0.10539	-0.23464	0.00261	0.06726	-0.03922	0.03017	0.0989	-0.0935	0.0935	0.1701	0.00201	0.97299	0.38209	0.61054	0.69522	0.1981	0.22387	0.22387	turquoise
WDR74	0.03093	0.04474	-0.01416	-0.00213	-0.05776	0.0293	0.17874	-0.36352	0.36352	0.68794	0.56122	0.8542	0.97799	0.45303	0.70366	0.01933	1.02E-06	1.02E-06	grey
UIMC1	0.00093	-0.17064	-0.05916	-0.05119	-0.05571	0.03362	-0.01851	0.03144	-0.03144	0.99037	0.02565	0.44209	0.50608	0.46927	0.66243	0.81008	0.68312	0.68312	turquoise
MAFB	-0.06188	-0.17121	-0.08159	0.06927	-0.06093	-0.19875	-0.12033	0.47589	-0.47589	0.42138	0.02516	0.28873	0.36795	0.42853	0.00916	0.11697	4.76E-11	4.76E-11	brown
CAMLG	0.0313	-0.13397	-0.12342	-0.04219	0.00998	-0.12029	-0.04493	0.09545	-0.09545	0.68442	0.08066	0.10779	0.58377	0.89694	0.11709	0.55957	0.21427	0.21427	turquoise
NHSL2	-0.09792	-0.13929	-0.11343	-0.02771	-0.14239	-0.00533	-0.03126	0.61816	-0.61816	0.20261	0.06922	0.13963	0.71898	0.0632	0.94487	0.68483	2.11E-19	2.11E-19	blue
HSCB	-0.08967	0.0191	-0.12009	-0.03736	-0.0498	-0.04334	0.11054	0.01807	-0.01807	0.24347	0.80419	0.1177	0.62754	0.51773	0.57353	0.15007	0.81457	0.81457	grey
ABCC10	-0.01585	0.02788	-0.04072	-0.02396	0.06643	-0.00883	0.05953	-0.08032	0.08032	0.83699	0.71737	0.59697	0.75576	0.38797	0.90873	0.43927	0.29637	0.29637	red
CD200R1	-0.11056	-0.07017	0.0683	0.00748	-0.14729	0.12473	0.00059	0.48959	-0.48959	0.15	0.36178	0.37473	0.92263	0.05454	0.10406	0.99387	1.08E-11	1.08E-11	blue
PIP5KL1	-0.04072	-0.16081	0.03633	0.04676	-0.04849	-0.05178	0.02844	-0.29103	0.29103	0.59694	0.03563	0.63712	0.54369	0.5288	0.50118	0.71197	0.00011	0.00011	grey
HNRNPK	-0.01933	-0.0943	-0.04362	-0.0009	-0.14017	0.0378	0.03653	0.0326	-0.0326	0.80189	0.21991	0.57105	0.99072	0.06747	0.6235	0.63525	0.67208	0.67208	turquoise
GMPR	0.01557	0.03126	-0.07023	0.0913	0.05834	0.00167	-0.08835	0.07598	-0.07598	0.83979	0.68482	0.3614	0.23496	0.44851	0.98271	0.2505	0.3233	0.3233	grey
COMMD6	0.04673	0.04215	-0.05284	-0.0833	-0.03663	-0.00299	0.12566	-0.12086	0.12086	0.54391	0.58413	0.49243	0.27874	0.6343	0.96906	0.10149	0.11533	0.11533	purple
KLHL15	-0.0513	-0.16	-0.13362	-0.04743	-0.09303	-0.0516	-0.13881	0.44983	-0.44983	0.50519	0.03658	0.08145	0.53789	0.22621	0.50271	0.07019	6.73E-10	6.73E-10	turquoise
TBC1D22A	-0.04208	0.067	-0.08391	0.01197	0.00091	-0.13384	0.00666	-0.03679	0.03679	0.58471	0.38389	0.27521	0.87649	0.99057	0.08096	0.93113	0.63281	0.63281	grey
SOX13	0.14743	-0.08042	-0.01429	0.00622	0.00057	-0.00542	0.13052	-0.23666	0.23666	0.05431	0.29576	0.85285	0.93568	0.99412	0.9439	0.08884	0.00183	0.00183	grey
SLU7	0.01547	-0.19562	-0.10476	-0.07673	-0.05705	-0.03485	-0.01847	0.15184	-0.15184	0.84087	0.01034	0.1727	0.31855	0.45874	0.65089	0.8105	0.04742	0.04742	turquoise
NAA38	-0.03524	-0.0812	-0.22508	-0.12879	0.24812	-0.34706	-0.06863	-0.00434	0.00434	0.64725	0.29106	0.00308	0.09318	0.00107	3.31E-06	0.37246	0.95504	0.95504	green
PDZD2	-0.11813	-0.14172	-0.08649	-0.06284	-0.08623	-0.2207	-0.10706	0.42867	-0.42867	0.12384	0.06446	0.26065	0.4142	0.26213	0.00372	0.16341	4.93E-09	4.93E-09	turquoise
PIK3C2B	0.08461	-0.00666	0.07802	0.03985	0.05848	0.15585	0.09143	-0.21538	0.21538	0.27123	0.93114	0.31046	0.60479	0.44742	0.04179	0.23431	0.00467	0.00467	turquoise
C17orf80	-0.08298	-0.17602	-0.16569	-0.04104	0.04516	-0.15748	-0.07062	0.35095	-0.35095	0.28056	0.02129	0.03033	0.5941	0.55756	0.03968	0.35869	2.52E-06	2.52E-06	turquoise
SGF29	0.11016	0.04769	-0.10366	0.00323	0.01513	-0.10754	-0.03885	-0.00482	0.00482	0.15147	0.53567	0.17727	0.96661	0.84429	0.1615	0.61393	0.95012	0.95012	grey
SIRPG	-0.09442	0.00244	0.04374	0.04929	-0.18867	0.12784	0.0228	0.351	-0.351	0.2193	0.97476	0.57	0.52205	0.01346	0.09564	0.76725	2.51E-06	2.51E-06	blue
HNRNPUL	0.03142	-0.05698	-0.05214	-0.02621	-0.08626	-0.03243	0.10966	-0.12909	0.12909	0.68333	0.45915	0.49826	0.73361	0.26194	0.67374	0.15334	0.09242	0.09242	turquoise
SOX5	-0.06577	-0.08532	-0.1424	-0.09805	-0.06617	-0.1773	-0.19023	0.55334	-0.55334	0.39271	0.26719	0.06318	0.20202	0.38989	0.02035	0.0127	4.21E-15	4.21E-15	brown
UBE2A	-0.04316	-0.20739	-0.02086	0.01823	-0.06575	-0.01305	0.04815	-0.04483	0.04483	0.57511	0.0065	0.78654	0.81289	0.39289	0.86549	0.5317	0.56043	0.56043	turquoise
FGFR1OP	-0.05197	-0.13587	-0.09459	-0.05461	0.16404	-0.17592	-0.0604	0.06754	-0.06754	0.49966	0.0764	0.21849	0.47803	0.03204	0.02136	0.43258	0.38006	0.38006	red
ZNF841	0.06408	-0.13356	-0.03127	-0.01853	-0.01658	0.05613	0.03639	-0.08724	0.08724	0.40505	0.0816	0.68475	0.80987	0.82963	0.4659	0.63653	0.25652	0.25652	red
PDE4DIP	0.01716	-0.12797	-0.14802	-0.08733	-0.05828	-0.21674	0.01722	0.30346	-0.30346	0.82369	0.09533	0.05336	0.25604	0.44892	0.00441	0.82311	5.47E-05	5.47E-05	turquoise
RNF8	0.03256	-0.09181	-0.06722	0.00064	0.01815	-0.04209	0.07926	0.03648	-0.03648	0.67247	0.23235	0.38236	0.99342	0.81375	0.58463	0.30281	0.63569	0.63569	turquoise
FAM131A	-0.05704	-0.28058	-0.18136	-0.1075	0.13515	-0.16724	-0.15907	0.11126	-0.11126	0.45864	0.0002	0.0176	0.16166	0.07799	0.02879	0.0377	0.14742	0.14742	grey
RETREG3	0.00738	-0.18301	-0.16109	-0.02978	0.00051	-0.30764	-0.03431	-0.08572	0.08572	0.92363	0.01658	0.0353	0.69897	0.99473	4.26E-05	0.65598	0.26494	0.26494	grey
LMO3	-0.04193	0.07167	-0.11351	-0.02818	-0.0921	-0.0242	-0.14857	0.52912	-0.52912	0.58609	0.35161	0.13935	0.71443	0.2309	0.75341	0.05246	1.01E-13	1.01E-13	grey
ATP10D	-0.06004	-0.1436	-0.04426	0.03901	-0.14834	0.07228	-0.01057	0.42272	-0.42272	0.43534	0.06096	0.5654	0.61241	0.05283	0.34746	0.89083	8.44E-09	8.44E-09	turquoise
RXYLT1	0.02231	-0.12572	-0.23017	-0.06515	0.15127	-0.29554	-0.2256	0.27716	-0.27716	0.77207	0.10132	0.00246	0.39725	0.04827	8.70E-05	0.00301	0.00024	0.00024	turquoise
GSTM4	-0.08722	0.08339	0.04372	0.01809	0.03417	0.20635	0.07002	0.02172	-0.02172	0.25667	0.27823	0.57018	0.81437	0.65725	0.00677	0.36283	0.77795	0.77795	grey
TMEM253	-0.02726	0.01669	0.05297	0.07258	0.12401	0.14957	0.10531	-0.27544	0.27544	0.72339	0.82851	0.49142	0.34549	0.10611	0.05088	0.17045	0.00027	0.00027	grey
TBRG4	0.03433	-0.00081	0.018	-0.01417	0.05899	0.03794	0.14965	-0.45735	0.45735	0.65575	0.99164	0.81523	0.85403	0.4434	0.62223	0.05074	3.21E-10	3.21E-10	grey
RNPC3	-0.00458	-0.08489	-0.06644	-0.03934	0.06424	0.03327	-0.00029	0.03043	-0.03043	0.9526	0.26964	0.38795	0.60944	0.40384	0.66576	0.99701	0.69281	0.69281	red
TTC14	-0.02849	-0.12144	-0.07019	-0.03908	0.06533	0.05049	0.03998	-0.00562	0.00562	0.71142	0.11358	0.36165	0.61179	0.39592	0.51195	0.6036	0.9418	0.9418	red
FBXL12	0.01799	-0.0851	-0.04586	-0.00886	0.09968	0.0744	-0.01105	-0.15385	0.15385	0.81531	0.26841	0.55139	0.90845	0.19457	0.33347	0.8859	0.04453	0.04453	grey
BEX2	-0.0224	-0.06466	-0.32489	-0.19676	0.12506	-0.3061	-0.26743	0.29077	-0.29077	0.77119	0.4008	1.45E-05	0.0099	0.10315	4.67E-05	0.00041	0.00011	0.00011	brown
THEM4	0.03488	0.02083	-0.11493	-0.15622	0.06149	-0.11166	-0.05032	0.16005	-0.16005	0.65059	0.78686	0.13445	0.04132	0.42432	0.14595	0.51333	0.03652	0.03652	grey
MTHFD2L	-0.07198	-0.13476	-0.18491	-0.13426	0.19423	-0.27731	-0.14876	0.16451	-0.16451	0.34947	0.07887	0.01547	0.07998	0.01091	0.00024	0.05215	0.03155	0.03155	grey
NUAK1	-0.01557	-0.13127	-0.02748	0.00827	-0.16318	0.00074	0.05946	0.35132	-0.35132	0.83987	0.08702	0.72129	0.91456	0.03296	0.99235	0.43977	2.46E-06	2.46E-06	blue
CA12	-0.05018	0.0786	0.1063	0.12022	-0.03605	0.16013	0.23876	-0.28895	0.28895	0.51456	0.30687	0.16643	0.1173	0.63966	0.03642	0.00166	0.00013	0.00013	grey
TGFBRAP1	-0.02123	-0.05481	-0.01883	0.00733	-0.14046	0.03196	0.09812	0.24641	-0.24641	0.78281	0.47648	0.80685	0.92424	0.06689	0.67821	0.20171	0.00116	0.00116	turquoise
ADRB2	-0.10248	-0.1155	-0.06518	0.00867	-0.14744	-0.01407	-0.033	0.40797	-0.40797	0.18227	0.13251	0.39702	0.91036	0.0543	0.8551	0.66828	3.05E-08	3.05E-08	blue
AHSA1	0.09768	-0.10349	-0.02434	0.04796	0.07946	-0.05294	0.04955	-0.24395	0.24395	0.2037	0.17797	0.75196	0.53332	0.30154	0.49162	0.51986	0.0013	0.0013	turquoise
PSMD5	-0.10499	-0.07772	0.04602	0.08572	-0.19197	0.12264	0.11038	0.09344	-0.09344	0.17175	0.31233	0.55005	0.26493	0.01189	0.11005	0.15067	0.22412	0.22412	turquoise
VIL1	0.0944	0.01825	0.07627	0.05281	0.24464	0.04818	-0.07938	-0.11787	0.11787	0.21939	0.8127	0.32144	0.49275	0.00126	0.53144	0.30205	0.12467	0.12467	grey
MAMLD1	-0.06188	-0.20731	-0.16745	-0.0316	0.06887	-0.25562	-0.28659	0.47697	-0.47697	0.42141	0.00652	0.02859	0.68155	0.37073	0.00074	0.00014	4.25E-11	4.25E-11	brown
FGGY	0.04259	0.00545	-0.02771	0.02391	0.02843	0.0601	0.12635	0.12853	-0.12853	0.58016	0.9436	0.71901	0.75627	0.71199	0.43489	0.0996	0.09387	0.09387	grey
TOR1AIP2	0.07739	-0.15892	-0.13128	-0.06163	-0.00861	-0.02557	0.06303	-0.175	0.175	0.31439	0.03788	0.08698	0.42326	0.91102	0.73994	0.41282	0.02206	0.02206	turquoise
TTC8	-0.00622	-0.15112	-0.13587	-0.03556	0.12545	-0.2402	-0.14782	0.27167	-0.27167	0.93565	0.0485	0.0764	0.6443	0.10207	0.00155	0.05368	0.00033	0.00033	turquoise
QPCTL	0.05849	-0.0918	0.00941	-0.03968	0.12128	-0.0247	0.13939	-0.38593	0.38593	0.44733	0.23241	0.90277	0.60639	0.11409	0.74847	0.06902	1.86E-07	1.86E-07	grey
SOX4	0.04201	-0.09864	0.00535	0.0445	-0.16164	0.20171	0.14667	-0.16169	0.16169	0.58539	0.19931	0.94466	0.56327	0.03467	0.00815	0.05558	0.03462	0.03462	turquoise
MOK	0.03479	-0.07491	0.02031	0.02238	0.15007	-0.02021	0.05223	-0.33152	0.33152	0.65148	0.33018	0.79204	0.77143	0.0501	0.79308	0.49745	9.45E-06	9.45E-06	grey
IGFALS	0.04009	0.07013	-0.1588	-0.03587	0.15385	-0.06758	-0.08563	0.05269	-0.05269	0.60264	0.36204	0.03803	0.64136	0.04454	0.37984	0.26546	0.49367	0.49367	grey
C1orf210	0.06342	-0.05872	-0.05532	-0.06708	0.23008	0.01897	-0.11838	-0.26403	0.26403	0.40987	0.44555	0.47234	0.38338	0.00247	0.80543	0.12307	0.00048	0.00048	grey
MGLL	-0.01472	0.00619	0.11638	0.07111	-0.00598	0.2521	0.10635	-0.30957	0.30957	0.84849	0.93591	0.12956	0.35537	0.93817	0.00088	0.16621	3.79E-05	3.79E-05	turquoise
PDCD6IP	-0.04233	-0.07741	0.07692	-0.0128	-0.11693	0.19439	0.11856	-0.10313	0.10313	0.5825	0.31423	0.31734	0.868	0.12775	0.01084	0.12247	0.17952	0.17952	turquoise
ATP1B2	-0.01695	-0.10563	-0.24585	-0.07258	0.04676	-0.24339	-0.20385	0.52993	-0.52993	0.82579	0.16914	0.00119	0.34549	0.54362	0.00134	0.00749	9.11E-14	9.11E-14	brown
ARHGAP1	-0.11253	-0.04847	0.10932	0.09619	-0.16665	0.27145	0.10348	0.13497	-0.13497	0.14281	0.52897	0.15464	0.21073	0.02937	0.00033	0.17801	0.0784	0.0784	turquoise
APOBEC1	0.07867	0.1267	0.10646	0.047	0.10109	0.17112	0.01556	-0.40029	0.40029	0.3064	0.09867	0.16579	0.54155	0.18829	0.02524	0.83989	5.81E-08	5.81E-08	yellow
UBE2E1	-0.04947	-0.08268	-0.00489	-0.0256	-0.1958	0.13375	0.12134	-0.04723	0.04723	0.52049	0.28236	0.94942	0.7396	0.01027	0.08115	0.11388	0.53961	0.53961	turquoise
PATL1	-0.02153	-0.05241	0.0547	0.06121	-0.18377	0.18354	0.17025	-0.15012	0.15012	0.77985	0.49604	0.47733	0.42641	0.01613	0.01626	0.026	0.05002	0.05002	turquoise
PTPRC	-0.13425	-0.06118	0.08428	0.05647	-0.22845	0.16876	0.0268	0.45489	-0.45489	0.08001	0.4267	0.27311	0.4632	0.00265	0.02735	0.72789	4.10E-10	4.10E-10	blue
PIGM	-0.03321	-0.06317	-0.11617	-0.10242	-0.00539	0.02112	0.0732	0.02329	-0.02329	0.66633	0.41172	0.13027	0.18252	0.94425	0.7839	0.34135	0.76238	0.76238	turquoise
CIP2A	-0.07604	-0.01325	0.03893	-0.01412	-0.14367	0.13412	0.23227	-0.156	0.156	0.32289	0.8634	0.61321	0.85458	0.06084	0.08031	0.00224	0.04159	0.04159	pink
DCAF4	0.02987	0.03392	-0.0214	0.06061	-0.065	0.04819	0.13093	-0.20999	0.20999	0.69811	0.65959	0.78116	0.43102	0.39829	0.53138	0.08783	0.00584	0.00584	grey
UBE2M	0.13671	-0.06724	-0.15492	-0.1157	0.09653	-0.19933	0.01213	-0.25718	0.25718	0.07459	0.38219	0.04305	0.13183	0.20913	0.00896	0.87489	0.00068	0.00068	green
PTGIS	-0.11154	-0.08223	0.07147	0.1207	-0.18726	0.19276	0.06535	0.34633	-0.34633	0.14639	0.28495	0.35294	0.11584	0.01419	0.01154	0.39575	3.48E-06	3.48E-06	blue
FBXO42	-0.07733	-0.16905	-0.01797	0.0435	-0.09209	0.0871	-0.0483	0.26188	-0.26188	0.31477	0.02708	0.8155	0.57216	0.23093	0.2573	0.53045	0.00054	0.00054	turquoise
GTF2IRD2	0.04396	-0.13182	-0.10012	-0.07131	0.0959	-0.10581	-0.14928	0.09479	-0.09479	0.56806	0.08568	0.19262	0.35398	0.21213	0.1684	0.05134	0.21749	0.21749	grey
ARHGEF26	-0.00097	-0.09794	-0.21408	-0.04003	0.00329	-0.15159	-0.18787	0.51028	-0.51028	0.98995	0.20251	0.00493	0.60322	0.96592	0.04779	0.01387	1.01E-12	1.01E-12	brown
EIF3H	0.08903	0.07041	0.03788	-0.03504	-0.15488	0.08438	0.17227	-0.2035	0.2035	0.24689	0.3601	0.62279	0.64916	0.04311	0.27252	0.02425	0.00759	0.00759	turquoise
ALG1L2	0.0124	-0.04911	0.07678	0.10012	-0.00652	0.07422	0.14018	-0.38847	0.38847	0.87215	0.52359	0.31824	0.1926	0.93259	0.33464	0.06744	1.52E-07	1.52E-07	grey
RRP12	0.10696	-0.06673	-0.01658	-0.01453	0.01317	-0.11846	0.06668	-0.21189	0.21189	0.16378	0.38584	0.82954	0.85035	0.86422	0.12279	0.38624	0.0054	0.0054	grey
TELO2	0.04826	0.07262	-0.01467	-0.05152	0.18103	-0.03465	0.14591	-0.3124	0.3124	0.53078	0.34519	0.84898	0.50336	0.01781	0.6528	0.05688	3.19E-05	3.19E-05	green
TXLNG	-0.39345	-0.18319	-0.12251	-0.07392	-0.01813	-0.06762	-0.08853	0.16263	-0.16263	1.02E-07	0.01647	0.11042	0.33662	0.81397	0.37952	0.24953	0.03357	0.03357	turquoise
CNIH4	0.04567	-0.11416	-0.04542	-0.02247	-0.07714	0.00712	0.16609	-0.33939	0.33939	0.55305	0.13708	0.5553	0.7705	0.31595	0.92637	0.02993	5.60E-06	5.60E-06	greenyellow
IRF6	0.09438	-0.0098	0.0499	-0.06294	0.03831	0.11435	0.09016	-0.44582	0.44582	0.21949	0.89878	0.51691	0.41343	0.61887	0.13641	0.2409	9.93E-10	9.93E-10	yellow
AMPD2	-0.04702	-0.11129	-0.1267	-0.04734	0.09283	-0.12921	-0.03177	0.25423	-0.25423	0.54139	0.14729	0.09867	0.53865	0.22719	0.09212	0.67995	0.00079	0.00079	turquoise
SF3B6	0.02377	-0.0149	-0.01267	-0.0268	-0.05485	0.06417	0.2357	-0.38966	0.38966	0.75762	0.84667	0.86941	0.72791	0.47615	0.40441	0.00191	1.38E-07	1.38E-07	turquoise
SAA2-SAA4	-0.05614	-0.0016	0.01342	0.05594	-0.06889	0.05861	-0.02883	0.11318	-0.11318	0.46577	0.98347	0.86175	0.46739	0.37065	0.44638	0.70816	0.14051	0.14051	grey
HMG20A	-0.06458	-0.15687	-0.02601	0.04937	-0.09384	0.0981	0.02837	0.27113	-0.27113	0.4014	0.04046	0.7356	0.52137	0.22214	0.2018	0.7126	0.00033	0.00033	turquoise
EEF1A2	0.04125	-0.13023	-0.25736	-0.11244	0.10005	-0.29089	-0.13508	0.10888	-0.10888	0.59219	0.08957	0.00068	0.14312	0.19294	0.00011	0.07814	0.1563	0.1563	brown
FAM171B	-0.05646	-0.21764	-0.14073	0.00895	-0.12298	-0.21307	-0.06309	0.55499	-0.55499	0.46326	0.00424	0.06637	0.90751	0.10905	0.00514	0.41232	3.36E-15	3.36E-15	turquoise
ACSS3	-0.00288	-0.12598	-0.01645	0.06656	-0.25495	0.13744	-0.04003	0.51015	-0.51015	0.97014	0.10061	0.83085	0.38707	0.00076	0.07304	0.60322	1.02E-12	1.02E-12	blue
SYT12	0.01547	-0.11243	-0.03924	0.06445	-0.07795	0.07745	0.13686	-0.3063	0.3063	0.84079	0.14317	0.6104	0.40229	0.31086	0.31397	0.07427	4.61E-05	4.61E-05	grey
ELK3	-0.09046	-0.05047	0.04121	0.09202	-0.19148	0.15213	0.05061	0.19583	-0.19583	0.23931	0.51207	0.59256	0.23131	0.01211	0.047	0.51094	0.01026	0.01026	black
QSER1	-0.02885	-0.1798	-0.02657	0.03184	-0.21879	0.08908	0.10451	0.14581	-0.14581	0.70802	0.01862	0.73017	0.67927	0.00404	0.24659	0.17373	0.05706	0.05706	turquoise
EME2	-0.06879	-0.05535	0.01771	-0.01952	0.2233	-0.11364	-0.04419	-0.16947	0.16947	0.37129	0.47209	0.81816	0.79994	0.00333	0.13889	0.56605	0.0267	0.0267	red
KLHDC4	0.01776	0.03183	-0.13144	-0.13041	0.20247	-0.18058	0.00611	-0.25396	0.25396	0.81765	0.67944	0.0866	0.08912	0.00791	0.0181	0.93677	0.0008	0.0008	red
UBR7	-0.04681	-0.10093	-0.05805	0.05083	-0.03576	-0.05795	-0.09217	0.24628	-0.24628	0.54322	0.18901	0.45072	0.50906	0.64242	0.45153	0.23054	0.00117	0.00117	turquoise
TBC1D10A	-0.04345	-0.09276	-0.05476	0.01264	-0.02622	0.00972	0.05733	-0.06692	0.06692	0.5726	0.22753	0.47683	0.86966	0.73354	0.89956	0.45638	0.38451	0.38451	grey
STAP2	0.10596	0.07482	0.03378	0.0095	0.13391	0.09363	0.17339	-0.54125	0.54125	0.16781	0.33074	0.66094	0.90182	0.08078	0.22318	0.02333	2.12E-14	2.12E-14	yellow
PTGDR2	0.01891	-0.08462	-0.22151	-0.13106	0.17505	-0.25814	-0.12952	0.29585	-0.29585	0.80605	0.27115	0.00359	0.08751	0.02202	0.00065	0.09133	8.54E-05	8.54E-05	brown
ADAMTS1	-0.01965	-0.09874	-0.00548	0.16923	-0.09591	0.11613	0.01267	0.23656	-0.23656	0.79869	0.19886	0.94326	0.02692	0.21209	0.13038	0.86933	0.00184	0.00184	blue
TRIAP1	0.00648	-0.0084	0.01625	0.02754	-0.02852	0.0003	0.07091	-0.10147	0.10147	0.933	0.91313	0.83295	0.72071	0.71113	0.99691	0.35671	0.18664	0.18664	turquoise
SLC46A3	-0.02772	-0.10183	-0.04063	-0.01509	-0.18646	0.08263	-0.0137	0.3033	-0.3033	0.71891	0.18508	0.59779	0.8447	0.01461	0.28261	0.85881	5.52E-05	5.52E-05	blue
ACOT13	-0.09745	-0.03192	-0.09693	-0.03663	0.14773	-0.09118	-0.05883	0.14865	-0.14865	0.20482	0.67857	0.20726	0.63433	0.05382	0.23559	0.44466	0.05233	0.05233	turquoise

CLPTM1L	0.02218	-0.09045	-0.07587	-0.0481	0.13155	-0.22481	-0.0029	-0.13881	0.13881	0.77335	0.23937	0.32401	0.53218	0.08633	0.00311	0.96996	0.0702	0.0702	grey
RCC1L	0.0441	-0.10956	-0.08082	-0.03619	0.09623	-0.15558	-0.00074	-0.00617	0.00617	0.56683	0.15375	0.29332	0.63839	0.21055	0.04216	0.99235	0.93613	0.93613	grey
FAM50A	0.02562	-0.14979	-0.0885	-0.09999	0.00865	-0.13476	0.08881	-0.22582	0.22582	0.73946	0.05053	0.24973	0.1932	0.91059	0.07887	0.24803	0.00298	0.00298	grey
PCYT1B	-0.05665	-0.10969	-0.14661	0.00664	-0.07992	-0.04192	-0.12009	0.48314	-0.48314	0.46173	0.15324	0.05568	0.93135	0.29877	0.58619	0.11768	2.19E-11	2.19E-11	grey
DEFB131B	-0.00873	-0.10692	0.04325	-0.06238	-0.07489	0.05567	0.1038	0.06017	-0.06017	0.90977	0.16394	0.57435	0.41767	0.33031	0.46956	0.17666	0.43435	0.43435	turquoise
G3BP2	-0.07053	-0.13181	-0.02034	-0.03514	-0.0512	0.05178	-0.02515	0.15497	-0.15497	0.35928	0.08571	0.79176	0.64821	0.50604	0.50123	0.74407	0.04298	0.04298	turquoise
LONRF3	0.06096	-0.12828	0.0619	0.12399	-0.0451	0.30744	0.14608	-0.22294	0.22294	0.42834	0.0945	0.4212	0.10617	0.55807	4.31E-05	0.05659	0.00338	0.00338	turquoise
ERBIN	-0.0206	-0.09738	0.0049	0.04866	-0.11996	0.12919	0.02372	0.00324	-0.00324	0.78913	0.20512	0.94933	0.5274	0.1181	0.09216	0.75811	0.96649	0.96649	turquoise
SLC28A3	0.04544	0.05741	-0.04141	0.07775	-0.01738	0.15261	0.10027	-0.10071	0.10071	0.55507	0.45574	0.59077	0.3121	0.82153	0.0463	0.19193	0.18996	0.18996	grey
EML3	-0.08233	-0.06066	0.00174	-0.01284	-0.05739	0.0261	0.05537	-0.00671	0.00671	0.28438	0.43062	0.98193	0.86765	0.4559	0.73469	0.47193	0.93062	0.93062	grey
GABRE	0.03454	-0.0384	0.06773	0.0729	0.05474	0.1014	0.10363	-0.27334	0.27334	0.65383	0.61807	0.37873	0.34336	0.47701	0.18695	0.1774	0.0003	0.0003	grey
EID3	-0.0618	-0.15733	-0.05838	0.01537	-0.03623	-0.19729	-0.16995	0.56285	-0.56285	0.42198	0.03988	0.44815	0.84188	0.63805	0.0097	0.02626	1.12E-15	1.12E-15	brown
PTCD2	-0.02849	-0.07472	-0.14036	-0.08693	-0.01588	-0.10915	0.00098	0.22176	-0.22176	0.71142	0.33138	0.06708	0.25823	0.83664	0.1553	0.98985	0.00356	0.00356	turquoise
UCP3	-0.06852	-0.16146	-0.15003	0.0279	0.01218	-0.10842	-0.13519	0.17101	-0.17101	0.37318	0.03488	0.05016	0.71721	0.87435	0.15808	0.07791	0.02533	0.02533	red
CD300LF	-0.09724	-0.01952	0.09955	0.08901	-0.19954	0.20449	0.12037	0.23201	-0.23201	0.20579	0.79991	0.19518	0.24699	0.00888	0.0073	0.11683	0.00226	0.00226	blue
CCSER1	0.00886	-0.12586	0.09943	-0.00188	-0.03609	0.1953	0.05839	-0.18529	0.18529	0.90839	0.10095	0.19571	0.98057	0.63929	0.01047	0.44808	0.01525	0.01525	turquoise
SCARA5	-0.13134	-0.00688	-0.01119	0.07657	-0.02138	0.04317	-0.0871	0.57184	-0.57184	0.08683	0.92881	0.88447	0.31956	0.78138	0.57505	0.25731	3.11E-16	3.11E-16	blue
HERPUD2	-0.01873	-0.12303	-0.00571	0.01863	-0.05954	0.09065	0.03323	0.09465	-0.09465	0.80788	0.10891	0.94086	0.80893	0.43921	0.23832	0.6661	0.21815	0.21815	turquoise
IPO11	-0.08212	-0.06866	-0.02378	0.02516	-0.13311	0.05951	0.05067	0.29561	-0.29561	0.28564	0.3722	0.75749	0.74393	0.08262	0.43943	0.51048	8.66E-05	8.66E-05	turquoise
TIAL1	0.05401	-0.12371	-0.01733	-0.04493	0.02294	-0.08643	0.06636	-0.09651	0.09651	0.48289	0.10696	0.82199	0.55951	0.76583	0.26099	0.38852	0.2092	0.2092	turquoise
INVS	-0.04738	-0.18885	-0.13631	0.06651	-0.11577	-0.08063	-0.03561	0.35788	-0.35788	0.53832	0.01337	0.07545	0.3874	0.1316	0.29449	0.64378	1.54E-06	1.54E-06	turquoise
SCARA3	-0.05098	-0.04649	-0.01094	0.06975	-0.18624	0.14064	0.09942	0.35586	-0.35586	0.50781	0.54601	0.88706	0.36468	0.01473	0.06655	0.19575	1.78E-06	1.78E-06	blue
INO80D	-0.00275	-0.11522	0.01389	0.06143	-0.03458	0.10579	-0.01722	0.01415	-0.01415	0.97151	0.13346	0.85688	0.42477	0.65342	0.16847	0.82308	0.85428	0.85428	turquoise
STAT2	0.01419	-0.11515	-0.00351	0.06108	-0.13153	0.03436	0.11074	0.13264	-0.13264	0.85383	0.13367	0.9637	0.42741	0.08638	0.6555	0.14931	0.08374	0.08374	turquoise
RPP14	-0.0939	-0.04048	-0.11352	-0.10426	#######	0.01559	-0.13488	0.23619	-0.23619	0.22185	0.59915	0.13931	0.17475	0.99927	0.83964	0.0786	0.00187	0.00187	turquoise
HIST2H3P	-0.02857	-0.033	0.00405	0.10461	-0.14927	0.13377	0.1804	0.0184	-0.0184	0.71072	0.66833	0.95811	0.17331	0.05134	0.08111	0.01822	0.81117	0.81117	grey
ANKRD10	0.03573	-0.10095	0.00216	-0.01015	0.08181	0.02842	0.04822	-0.26473	0.26473	0.6427	0.18894	0.97762	0.89519	0.28743	0.71218	0.53112	0.00047	0.00047	red
CDK5R2	0.00144	-0.10587	-0.29337	-0.16744	0.125	-0.3532	-0.22897	0.35546	-0.35546	0.98511	0.16817	9.85E-05	0.0286	0.10332	2.15E-06	0.00259	1.83E-06	1.83E-06	brown
C16orf46	0.07006	-0.17335	-0.1053	0.05055	-0.02502	0.00276	0.11397	0.04804	-0.04804	0.36253	0.02337	0.17048	0.51146	0.74535	0.97146	0.13773	0.53268	0.53268	turquoise
TRMT10B	-0.00115	-0.13765	-0.19953	-0.11587	0.11433	-0.13217	-0.14171	0.16106	-0.16106	0.98805	0.07259	0.00888	0.13127	0.13648	0.08485	0.06448	0.03534	0.03534	turquoise
ELOB	0.10158	0.11165	-0.06462	-0.10948	0.07164	-0.0093	0.15057	-0.42366	0.42366	0.18617	0.14598	0.40106	0.15404	0.35181	0.90394	0.04933	7.75E-09	7.75E-09	grey
SPTLC2	0.03663	-0.055	0.05736	0.05876	-0.03125	0.17057	0.1331	-0.16783	0.16783	0.63435	0.4749	0.45616	0.4452	0.68491	0.02571	0.08265	0.02823	0.02823	turquoise
UBP1	-0.03949	-0.13494	-0.00811	-0.00181	-0.14761	0.12429	0.08565	0.18031	-0.18031	0.60811	0.07846	0.91611	0.98123	0.05403	0.1053	0.26533	0.01828	0.01828	turquoise
CD7	-0.07142	-0.0235	-0.03547	0.05133	-0.12013	0.03926	0.04387	0.23328	-0.23328	0.35327	0.76034	0.64513	0.5049	0.11758	0.61014	0.56885	0.00214	0.00214	blue
P2RX7	-0.06909	-0.01801	0.03781	0.12476	-0.18096	0.11228	0.05067	0.48161	-0.48161	0.36922	0.81514	0.62346	0.10399	0.01785	0.14371	0.51042	2.58E-11	2.58E-11	blue
PLEK	-0.13316	-0.00316	0.08347	0.06041	-0.17189	0.16338	0.05656	0.46041	-0.46041	0.08253	0.96731	0.27777	0.43253	0.02458	0.03275	0.46246	2.36E-10	2.36E-10	blue
CLUH	0.02576	0.07104	-0.13599	-0.03506	0.14757	-0.11095	0.1415	-0.14718	0.14718	0.73809	0.35587	0.07614	0.64892	0.05408	0.14856	0.06488	0.05473	0.05473	grey
STRAP	-0.03458	-0.14214	-0.05688	-0.02891	-0.03181	-0.1325	-0.01727	0.12002	-0.12002	0.65344	0.06366	0.45992	0.70738	0.67963	0.08406	0.82261	0.11792	0.11792	turquoise
COG1	0.00475	-0.19039	-0.15593	-0.13109	0.13169	-0.24055	-0.18902	0.11531	-0.11531	0.95087	0.01262	0.04169	0.08744	0.08599	0.00153	0.01329	0.13315	0.13315	turquoise
MINPP1	-0.05103	-0.19335	-0.12675	0.01313	0.0729	-0.20337	-0.03232	0.16709	-0.16709	0.5074	0.01128	0.09854	0.86467	0.34335	0.00763	0.67477	0.02894	0.02894	turquoise
IMPACT	-0.09943	-0.05589	-0.05604	0.07036	-0.11705	0.10103	0.01285	0.13403	-0.13403	0.19572	0.46781	0.46658	0.36049	0.12733	0.18857	0.86757	0.08051	0.08051	turquoise
AMBP	0.05462	-0.01885	-0.07229	0.11981	0.01631	0.07243	-0.06622	-0.02137	0.02137	0.47801	0.80663	0.34741	0.11856	0.83232	0.34646	0.38952	0.78142	0.78142	magenta
HAX1	0.11837	-0.09357	-0.12923	-0.18593	0.1075	-0.15846	-0.12131	-0.10869	0.10869	0.12308	0.22348	0.09206	0.0149	0.16167	0.03846	0.11399	0.15703	0.15703	greenyellow
LBX2	0.12188	-0.01475	0.05283	-0.00387	0.04443	0.10165	0.27362	-0.50996	0.50996	0.11228	0.8482	0.49253	0.95989	0.56395	0.18588	0.00029	1.05E-12	1.05E-12	green
RCAN2	-0.12573	-0.16591	-0.22112	-0.06288	0.01199	-0.24859	-0.20706	0.61055	-0.61055	0.1013	0.03011	0.00366	0.41394	0.87629	0.00104	0.00658	7.58E-19	7.58E-19	brown
NDUFB8	0.07303	0.03508	-0.08478	-0.11367	0.09486	-0.27234	0.00712	-0.17816	0.17816	0.34251	0.64874	0.27025	0.13879	0.21716	0.00031	0.92633	0.01973	0.01973	grey
TMEM211	0.07477	0.042	0.03804	-0.0356	0.051	-0.03722	-0.0777	-0.10851	0.10851	0.33111	0.58549	0.62134	0.64392	0.50767	0.62886	0.31245	0.15775	0.15775	grey
GGA2	-0.08017	-0.13445	-0.05544	-0.03373	-0.1236	-0.0951	-0.15617	0.45407	-0.45407	0.29726	0.07955	0.47138	0.66141	0.10728	0.21598	0.04137	4.44E-10	4.44E-10	blue
MMP25	-0.15361	0.01433	0.06539	0.00302	-0.13191	0.09821	0.11131	0.22833	-0.22833	0.04486	0.85247	0.39547	0.96875	0.08546	0.20129	0.14724	0.00267	0.00267	blue
AKR7A2	-0.01162	-0.04101	-0.04204	-0.11147	0.09564	0.02404	-0.02459	0.02514	-0.02514	0.88008	0.59435	0.58513	0.14663	0.21337	0.75495	0.74954	0.74411	0.74411	grey
MTCP1	-0.05046	-0.24553	-0.11554	-0.01844	0.06941	-0.00295	-0.05018	-0.17434	0.17434	0.51221	0.00121	0.13236	0.81083	0.367	0.96948	0.51458	0.02257	0.02257	red
RNF31	-0.01801	-0.09911	0.003	0.02524	-0.03392	0.03494	-0.00199	0.12402	-0.12402	0.8151	0.19716	0.96889	0.74314	0.65962	0.65006	0.97936	0.10608	0.10608	turquoise
GLRX	0.0527	0.01722	-0.00799	-0.04226	0.11547	0.00529	0.06328	0.03433	-0.03433	0.49364	0.82315	0.91744	0.58317	0.13259	0.94522	0.41097	0.65572	0.65572	grey
TSC22D2	-0.04504	-0.25478	0.05174	0.07599	-0.13562	0.18381	0.03057	0.04059	-0.04059	0.55856	0.00077	0.50156	0.32321	0.07696	0.01611	0.69148	0.59813	0.59813	turquoise
IKBIP	-0.08581	-0.07058	0.06585	0.07961	-0.18184	0.00959	0.02782	0.27698	-0.27698	0.26444	0.35894	0.39215	0.30067	0.0173	0.90091	0.71799	0.00025	0.00025	black
EXOC6	0.05075	-0.01231	0.01472	-0.02679	0.07087	-0.02881	-0.01323	0.08947	-0.08947	0.50979	0.87307	0.84846	0.72797	0.357	0.70836	0.8636	0.24455	0.24455	grey
FGF12	-0.03932	-0.07201	-0.24218	-0.10391	-0.02539	-0.23863	-0.18414	0.46784	-0.46784	0.60962	0.34926	0.00142	0.17623	0.74164	0.00167	0.01591	1.11E-10	1.11E-10	brown
CSDC2	-0.09247	-0.00284	-0.07512	0.02848	-0.10596	0.02343	-0.05272	0.3222	-0.3222	0.22901	0.97059	0.32879	0.71154	0.16777	0.76102	0.49345	1.73E-05	1.73E-05	grey
RAB26	0.08307	0.01551	-0.29931	-0.20256	0.24508	-0.29884	-0.15403	0.19595	-0.19595	0.28008	0.84045	6.98E-05	0.00788	0.00123	7.18E-05	0.04428	0.01021	0.01021	brown
CAVIN3	-0.06574	0.02428	0.00177	-0.00389	-0.01029	0.04501	0.11082	-0.16317	0.16317	0.39297	0.75264	0.98164	0.95974	0.89374	0.55883	0.14904	0.03297	0.03297	black
RAB3B	0.00978	-0.17669	-0.185	-0.22358	0.14017	-0.23471	-0.14546	0.31744	-0.31744	0.899	0.02079	0.01542	0.00329	0.06747	0.002	0.05765	2.33E-05	2.33E-05	turquoise
HAVCR2	-0.10122	-0.01364	0.08361	0.09711	-0.28649	0.18331	0.1096	0.30967	-0.30967	0.18774	0.85943	0.27694	0.20637	0.00015	0.0164	0.1536	3.76E-05	3.76E-05	blue
P2RY10	-0.11095	-0.05657	0.08306	0.05593	-0.18867	0.15369	-0.02562	0.45447	-0.45447	0.14853	0.4624	0.28012	0.46745	0.01346	0.04475	0.73942	4.27E-10	4.27E-10	blue
ASPM	-0.01367	0.05554	0.02922	-0.01454	-0.11736	0.11669	0.34057	-0.35733	0.35733	0.85911	0.47056	0.70437	0.85027	0.12634	0.12852	5.17E-06	1.60E-06	1.60E-06	pink
IRAK2	-0.02153	-0.12659	0.16008	0.06403	-0.24354	0.26403	0.23414	-0.28578	0.28578	0.77983	0.09898	0.03648	0.4054	0.00133	0.00048	0.00205	0.00015	0.00015	turquoise
SSR1	-0.00062	-0.05119	-0.01341	0.00971	-0.04154	-0.12339	-0.08536	0.33306	-0.33306	0.99359	0.50613	0.86183	0.89966	0.58954	0.10787	0.26695	8.54E-06	8.54E-06	turquoise
MCUR1	-0.01886	-0.05344	-0.00885	0.01213	-0.07607	-0.01113	0.01722	0.14025	-0.14025	0.80654	0.48754	0.9085	0.87488	0.3227	0.88511	0.82314	0.06731	0.06731	turquoise
DBI	0.00023	0.05304	-0.00281	0.01901	-0.06669	0.16183	0.18426	-0.19719	0.19719	0.99757	0.49082	0.97087	0.80504	0.3861	0.03446	0.01584	0.00973	0.00973	grey
TDP2	-0.00488	-0.07375	-0.09451	-0.06311	0.13974	-0.11603	-0.08444	0.19335	-0.19335	0.94948	0.33774	0.21884	0.41217	0.06833	0.13072	0.27216	0.01128	0.01128	turquoise
TTC4	0.02499	-0.15852	0.01312	-0.01062	-0.09416	0.06887	0.09282	0.12888	-0.12888	0.74559	0.03838	0.86471	0.8904	0.22056	0.37078	0.22722	0.09296	0.09296	turquoise
KIAA0319I	-0.02831	-0.16732	-0.02903	-0.00027	0.09023	0.08055	-0.06035	-0.10366	0.10366	0.71317	0.02871	0.70621	0.99721	0.24056	0.29495	0.43297	0.17725	0.17725	turquoise
TTC26	-0.04118	-0.11319	-0.10992	-0.0072	-0.06869	-0.14268	-0.02946	0.22171	-0.22171	0.59283	0.14047	0.15238	0.92555	0.37203	0.06266	0.7021	0.00356	0.00356	turquoise
ZC3HAV1	-0.01675	-0.11588	-0.00062	0.02733	-0.15805	-0.01057	-0.00423	0.25532	-0.25532	0.8279	0.13123	0.99356	0.72269	0.03896	0.89087	0.95617	0.00075	0.00075	turquoise
FBXO8	-0.0805	-0.10499	-0.10383	-0.02554	0.06297	0.01619	-0.16272	0.20273	-0.20273	0.29523	0.17174	0.17654	0.74023	0.41323	0.83349	0.03347	0.00783	0.00783	turquoise
TSSK4	0.00838	-0.15505	-0.03659	0.0643	0.00892	0.02145	0.07142	0.10859	-0.10859	0.9134	0.04288	0.63468	0.40339	0.90784	0.78061	0.35326	0.15742	0.15742	red
TNFSF8	-0.12806	-0.01054	0.08297	0.06116	-0.22391	0.13896	-0.00558	0.48044	-0.48044	0.09509	0.89118	0.28062	0.42683	0.00324	0.0699	0.94231	2.93E-11	2.93E-11	blue
ADD2	-0.17733	-0.07434	-0.10368	0.05122	-0.08041	-0.0591	-0.09118	0.56316	-0.56316	0.02033	0.33386	0.17719	0.50586	0.29582	0.44256	0.23561	1.08E-15	1.08E-15	blue
SERPINB5	0.02009	0.0774	0.13793	0.043	-0.03374	0.24112	0.29128	-0.41984	0.41984	0.79428	0.3143	0.072	0.57653	0.66131	0.00149	0.00011	1.09E-08	1.09E-08	yellow
ETV7	0.06435	0.10574	0.08049	0.07557	-0.04836	0.19384	0.21046	-0.30126	0.30126	0.40307	0.16868	0.2953	0.32594	0.52994	0.01108	0.00573	6.23E-05	6.23E-05	tan
TRIR	0.10041	0.0229	-0.13435	-0.09264	0.04413	-0.12447	0.04011	-0.25324	0.25324	0.19131	0.76623	0.07979	0.22815	0.56658	0.10481	0.60242	0.00083	0.00083	green
EIF2B3	-0.06787	-0.03265	-0.06174	-0.08212	-0.11029	-0.05827	0.07032	0.20565	-0.20565	0.37777	0.67163	0.42244	0.28559	0.15099	0.44899	0.36073	0.00697	0.00697	turquoise
ZFP64	0.06038	0.00285	-0.05696	0.05051	-0.04415	0.06021	0.11569	-0.07717	0.07717	0.43277	0.97047	0.45933	0.51177	0.56641	0.43405	0.13187	0.31576	0.31576	turquoise
MED29	0.03397	-0.28964	-0.09191	0.06081	-0.11586	-0.05903	-0.0409	0.02548	-0.02548	0.65913	0.00012	0.23185	0.42944	0.13128	0.4431	0.59529	0.74078	0.74078	grey
TASOR	-0.02991	-0.08985	-0.01476	-0.00162	-0.13859	0.09295	-0.02027	0.26257	-0.26257	0.69779	0.24251	0.84803	0.9832	0.07065	0.22661	0.79242	0.00052	0.00052	turquoise
STX1A	0.13666	-0.14056	-0.0709	-0.08319	0.22793	-0.12701	-0.09813	-0.29358	0.29358	0.07469	0.06671	0.35676	0.27936	0.00272	0.09785	0.20163	9.73E-05	9.73E-05	grey
GPR142	-0.05628	-0.08381	-0.15049	-0.08021	0.11774	-0.30873	-0.21944	0.45696	-0.45696	0.46473	0.27578	0.04945	0.29699	0.1251	3.99E-05	0.00393	3.33E-10	3.33E-10	brown
VWDE	0.01351	-0.08015	-0.24217	-0.10351	0.04989	-0.128	-0.03647	0.19279	-0.19279	0.8608	0.29736	0.00142	0.17791	0.51702	0.09523	0.63585	0.01153	0.01153	brown
NIPAL3	-0.04828	-0.05968	-0.04858	0.00747	-0.09144	0.09283	-0.03614	0.11502	-0.11502	0.53059	0.4381	0.52803	0.92277	0.23424	0.2272	0.63888	0.13412	0.13412	turquoise
PRIM1	-0.01458	-0.00691	-0.06026	-0.0566	-0.01958	-0.05204	0.13795	0.01321	-0.01321	0.84989	0.92856	0.43364	0.46212	0.79936	0.49908	0.07197	0.86384	0.86384	turquoise
RNF10	0.15753	-0.17464	0.00301	0.01686	0.06879	-0.01782	-0.06981	-0.05444	0.05444	0.03961	0.02234	0.96887	0.82679	0.37129	0.81702	0.36423	0.47947	0.47947	turquoise
GHITM	-0.02526	-0.06291	-0.04667	-0.03943	-0.07938	-0.08569	0.04137	-0.01587	0.01587	0.74293	0.41366	0.54444	0.60866	0.30206	0.26512	0.59112	0.83678	0.83678	turquoise
GYG2	-0.2844	-0.16853	-0.15801	-0.12723	0.0328	-0.1538	-0.12935	0.35585	-0.35585	0.00016	0.02757	0.039	0.09725	0.67016	0.0446	0.09178	1.78E-06	1.78E-06	grey
INTS11	-0.04436	-0.0481	-0.03223	-0.01922	0.09348	0.03522	0.02396	-0.2687	0.2687	0.5645	0.53211	0.67557	0.80295	0.22394	0.64741	0.75571	0.00038	0.00038	green
CUTA	0.0695	-0.00845	-0.2009	-0.16915	0.173	-0.27	-0.02676	-0.05663	0.05663	0.36636	0.91267	0.00842	0.02699	0.02365	0.00036	0.7283	0.4619	0.4619	grey
COPRS	-0.02397	-0.11514	-0.26148	-0.08734	0.05615	-0.32894	-0.05581	0.14048	-0.14048	0.75567	0.13372	0.00055	0.25599	0.46573	1.12E-05	0.46844	0.06686	0.06686	grey
ZXDB	-0.11335	-0.1622	0.01068	0.02161	-0.12414	0.10747	-0.05892	0.00026	-0.00026	0.1399	0.03404	0.88976	0.77903	0.10572	0.16179	0.44395	0.99728	0.99728	turquoise
HLA-DQB	-0.09348	0.00944	0.0041	0.05787	-0.13657	0.12225	0.03852	0.19008	-0.19008	0.22394	0.90249	0.95753	0.45216	0.07489	0.11118	0.61697	0.01277	0.01277	blue
SYNJ2	-0.07623	-0.19801	0.00157	0.01555	-0.05261	-0.01339	-0.01303	0.20761	-0.20761	0.32172	0.00943	0.98375	0.84007	0.49439	0.86201	0.86568	0.00644	0.00644	turquoise
PET100	0.0769	0.01084	-0.17946	-0.1532	0.14747	-0.24943	-0.03799	-0.25799	0.25799	0.31745	0.88811	0.01885	0.04545	0.05426	0.001	0.62178	0.00066	0.00066	grey
SUCLG2	0.02355	0.1164	0.07725	0.00457	-0.06577	0.31341	0.17527	-0.21507	0.21507	0.75976	0.1295	0.31528	0.95265	0.39274	2.99E-05	0.02185	0.00473	0.00473	turquoise
GSPT2	-0.12041	-0.11571	-0.07113	0.01178	-0.06658	-0.15033	-0.05304	0.06824	-0.06824	0.11672	0.13179	0.35525	0.87847	0.38692	0.0497	0.49085	0.37517	0.37517	grey
GPR18	-0.07918	-0.03667	0.04946	-0.01319	-0.07717	0.05746	-0.1009	0.36652	-0.36652	0.30327	0.63393	0.52064	0.86402	0.31575	0.45541	0.18913	8.21E-07	8.21E-07	blue
CPVL	-0.05907	0.00525	0.01503	0.04935	-0.19619	0.16995	0.07626	0.36036	-0.36036	0.44284	0.94571	0.84532	0.52151	0.01012	0.02626	0.32149	1.29E-06	1.29E-06	blue
PAQR4	0.00229	0.04559	0.02288	0.02846	-0.01136	0.16986	0.17049	-0.4103	0.4103	0.97628	0.55378	0.76639	0.71179	0.88279	0.02635	0.02579	2.50E-08	2.50E-08	yellow
EDNRB	-0.13593	-0.09165	-0.11493	0.01538	-0.07162	-0.01124	-0.17719	0.61145	-0.61145	0.07627	0.2332	0.13444	0.84172	0.3519	0.88396	0.02043	6.54E-19	6.54E-19	blue
KIAA0895I	-0.02929	-0.1423	-0.16082	-0.09247	0.30596	-0.23893	-0.11387	0.03095	-0.03095	0.70377	0.06337	0.03562	0.22901	4.71E-05	0.00165	0.13809	0.68784	0.68784	red
STMN2	-0.01334	-0.0198	-0.09873	-0.07413	-0.07666	-0.03812	-0.06232	0.28708	-0.28708	0.86257	0.79718	0.19889	0.33526	0.31899	0.62058	0.41808	0.00014	0.00014	grey
ABCF3	-0.03204	-0.07169	-0.14893	-0.10964	-0.04772	-0.09096	0.0855	0.00363	-0.00363	0.67741	0.35145	0.05188	0.15344	0.53542	0.23672	0.26618	0.96245	0.96245	turquoise
ZFP30	-0.09901	-0.1465	-0.12526	0.04664	-0.02934	-0.11103	-0.06618	0.34346	-0.34346	0.19759	0.05587	0.10259	0.54466	0.70321	0.14825	0.38982	4.24E-06	4.24E-06	turquoise
DISP1	-0.00359	-0.06476	-0.11541	-0.05239	0.0481	-0.03337	-0.04269	0.24599	-0.24599	0.9628	0.40004	0.13281	0.49618	0.53212	0.66479	0.57929	0.00118	0.00118	grey
ZMYND12	-0.00387	0.03657	-0.03866	-0.06749	0.09391	-0.02659	-0.12554	0.14482	-0.14482	0.95998	0.63488	0.61568	0.38046	0.22183	0.72989	0.10181	0.05877	0.05877	grey
TSC1	-0.032	-0.1699	-0.08957	-0.0391	-0.00927	-0.08619	-0.02256	0.16762	-0.16762	0.67782	0.02631	0.24399	0.6116	0.90419	0.2623	0.76962	0.02842	0.02842	turquoise
ATP5IF1	0.05202	-0.03756	-0.05356	-0.07744	0.214	-0.05911	-0.03515	-0.28963	0.28963	0.49924	0.62576	0.48655	0.31408	0.00495	0.44249	0.64804	0.00012	0.00012	grey
CCNT1	0.03711	-0.23246	-0.06441	0.01647	0.01813	-0.04139	-0.13082	0.17599	-0.17599	0.62991	0.00222	0.40262	0.83075	0.81392	0.59089	0.08811	0.02131	0.02131	turquoise
CAMK2N1	0.06918	-0.1149	-0.02442	-0.05501	0.01213	0.05062	0.07369	-0.3473	0.3473	0.36858	0.13453	0.75125	0.47483	0.87485	0.51087	0.3381	3.26E-06	3.26E-06	grey
ARL5A	-0.08093	-0.05878	-0.04026	0.02395	-0.12506	0.02845	-0.01956	0.33222	-0.33222	0.29271	0.44503	0.60112	0.75588	0.10315	0.71181	0.79956	9.03E-06	9.03E-06	turquoise
SMCHD1	-0.00681	-0.189	0.07933	0.03343	-0.08066	0.13889	-0.00913	0.20471	-0.20471	0.92953	0.0133	0.30233	0.6642	0.2943	0.07003	0.90566	0.00723	0.00723	turquoise
LHFPL6	-0.08405	-0.08298	0.0234	0.0829	-0.1839	0.08593	-0.0543	0.5482	-0.5482	0.27442	0.28059	0.76124	0.28106	0.01605	0.26377	0.48054	8.43E-15	8.43E-15	blue
SMPDL3A	-0.06247	-0.06188	0.00418	-0.01541	0.11921	-0.11186	-0.15416	0.12833	-0.12833	0.41694	0.42142	0.95669	0.84146	0.12042	0.14524	0.04409	0.09437	0.09437	grey
NIBAN1	-0.11049	-0.01006	0.08481	0.10668	-0.2761	0.20588	0.04794	0.3524	-0.3524	0.15023	0.89614	0.27008	0.16491	0.00026	0.0069	0.53352	2.28E-06	2.28E-06	blue
COPS5	0.03152	0.03386	0.0546	-0.01081	-0.05658	-0.02064	0.12678	-0.24067	0.24067	0.68233	0.66017	0.47816	0.88842	0.46235	0.78876	0.09847	0.00152	0.00152	turquoise
CHKA	0.05933	-0.12479	-0.02866	0.02654	-0.01846	-0.04221	0.13451	-0.18155	0.18155	0.44084	0.10389	0.70983	0.73039	0.81058	0.58362	0.07943	0.01748	0.01748	grey
RYBP	-0.01316	-0.20199	-0.02684	0.00487	-0.08591	0.09374	-0.07672	0.24009	-0.24009	0.86435	0.00806	0.72752	0.94961	0.26389	0.22263	0.31857	0.00156	0.00156	turquoise
ZDHHC13	0.10179	-0.10801	-0.01944	-0.06841	0.04764	-0.02103	-0.02008	-0.2571	0.2571	0.18524	0.15966	0.80076	0.37399	0.53608	0.78481	0.79438	0.00069	0.00069	turquoise
RCL1	-0.04918	-0.00589	0.08653	0.06179	-0.21926	0.12465	0.06199	0.1641	-0.1641	0.523	0.93901	0.26042	0.42205	0.00396	0.1043	0.42052	0.03198	0.03198	turquoise
BAK1	0.04131	0.0155	0.06998	0.05141	-0.01249	0.14135	0.20984	-0.35276	0.35276	0.59166	0.8405	0.36305	0.50429	0.87115	0.06517	0.00588	2.22E-06	2.22E-06	yellow
NUP35	0.01075	0.05369	-0.04482	0.07901	-0.02336	0.00244	0.07187	-0.06977	0.06977	0.88907	0.4855	0.56054	0.30433	0.7617	0.9747	0.35023	0.36452	0.36452	turquoise
ZNF577	-0.04192	-0.06128	-0.02226	0.12155	0.02587	0.09997	0.0704	0.16322	-0.16322	0.58613	0.4259	0.77257	0.11327	0.73697	0.19329	0.36019	0.03292	0.03292	red
FAM228B	-0.0525	-0.15611	-0.22087	-0.06256	0.00711	-0.17975	-0.15697	0.42333	-0.42333	0.49526	0.04146	0.0037	0.41628	0.92644	0.01865	0.04033	7.99E-09	7.99E-09	brown

PUF60	0.0756	0.04972	-0.07201	-0.07361	-0.02724	-0.08719	0.14576	-0.26803	0.26803	0.3257	0.51843	0.34928	0.33868	0.72362	0.25679	0.05714	0.00039	0.00039	green
FYB2	-0.1042	-0.09851	-0.17589	-0.06822	0.11991	-0.32976	-0.19811	0.36069	-0.36069	0.17501	0.19989	0.02138	0.37533	0.11826	1.06E-05	0.00939	1.26E-06	1.26E-06	brown
HNRNPA3	0.00549	-0.04719	-0.00931	-0.0147	-0.05562	0.0438	0.07264	-0.04424	0.04424	0.94316	0.53992	0.90381	0.84867	0.46996	0.56943	0.3451	0.56562	0.56562	turquoise
PPEF1	-0.03116	0.0351	-0.01877	0.00838	-0.20188	0.00208	0.05488	0.07052	-0.07052	0.68582	0.64854	0.80745	0.91339	0.0081	0.97845	0.47592	0.35936	0.35936	black
UBE2L6	-0.00357	0.00208	0.05648	0.09798	-0.1974	0.16347	0.16988	-0.03761	0.03761	0.96301	0.97849	0.46313	0.20235	0.00966	0.03265	0.02633	0.62532	0.62532	tan
MYO5C	0.03692	-0.04356	0.09385	0.07516	-0.01164	0.14734	0.05574	-0.24784	0.24784	0.63164	0.57155	0.22208	0.32857	0.87993	0.05447	0.46901	0.00108	0.00108	turquoise
CDC20	-0.01018	0.09377	0.073	-0.00573	-0.02791	0.12247	0.33016	-0.48718	0.48718	0.8949	0.22251	0.34268	0.94067	0.71705	0.11055	1.03E-05	1.41E-11	1.41E-11	pink
PLEKHG1	-0.0916	-0.10125	0.07064	0.07787	-0.17062	0.21312	0.04698	0.22162	-0.22162	0.23342	0.18762	0.35853	0.31137	0.02567	0.00513	0.54172	0.00358	0.00358	turquoise
PARP9	-0.04507	-0.01317	-0.10117	0.06901	-0.12548	0.01782	0.1343	#######	9.58E-05	0.55831	0.86428	0.18794	0.36981	0.10199	0.81704	0.07991	0.99901	0.99901	tan
CINP	0.03487	0.05923	-0.13488	-0.02028	0.06997	-0.1415	0.04894	-0.13041	0.13041	0.65067	0.44162	0.0786	0.79233	0.36318	0.06488	0.52503	0.0891	0.0891	grey
KLHL29	-0.05447	-0.12771	-0.15975	-0.07401	-0.00047	-0.12137	-0.09459	0.3118	-0.3118	0.47917	0.09598	0.03687	0.33601	0.99515	0.11381	0.21847	3.31E-05	3.31E-05	turquoise
TIAF1	0.05806	-0.14392	-0.01003	0.03935	0.16894	-0.03907	-0.0251	0.03684	-0.03684	0.45066	0.06038	0.89644	0.60939	0.02718	0.61192	0.74453	0.63242	0.63242	red
PDE1A	-0.0274	-0.08752	-0.0188	0.07474	-0.1648	0.10321	-0.0983	0.54581	-0.54581	0.72204	0.25501	0.80716	0.33125	0.03124	0.17916	0.20087	1.16E-14	1.16E-14	blue
HEXA	-0.07246	-0.16031	-0.04427	0.02774	-0.03793	-0.11898	-0.02346	0.14481	-0.14481	0.34631	0.03622	0.56536	0.71872	0.62238	0.12116	0.76072	0.05879	0.05879	grey
HSPA13	-0.09288	-0.18291	-0.08684	-0.00747	-0.06164	-0.14759	-0.11797	0.43373	-0.43373	0.22696	0.01664	0.25874	0.92275	0.4232	0.05406	0.12438	3.10E-09	3.10E-09	turquoise
S1PR1	-0.12893	-0.12327	-0.06164	0.06576	-0.11037	0.03847	-0.15855	0.57191	-0.57191	0.09284	0.10821	0.42318	0.39278	0.15071	0.61735	0.03833	3.08E-16	3.08E-16	blue
HERC1	-0.10407	-0.17069	-0.09872	-0.04116	-0.07342	-0.05722	-0.12801	0.53262	-0.53262	0.17555	0.02561	0.19892	0.59299	0.33992	0.45729	0.0952	6.47E-14	6.47E-14	turquoise
SYBU	0.01434	-0.09265	-0.1124	0.08415	0.02855	-0.01958	-0.19216	0.14404	-0.14404	0.85233	0.22809	0.14329	0.27385	0.71085	0.79932	0.01181	0.06017	0.06017	grey
KIAA0100	-0.0905	-0.11999	-0.07143	-0.0488	-0.07103	-0.09281	0.00628	0.22847	-0.22847	0.2391	0.11799	0.35321	0.52616	0.3559	0.22728	0.93507	0.00265	0.00265	turquoise
ANKRD50	-0.06083	-0.16868	0.02793	0.10704	-0.21084	0.17029	0.13262	0.10161	-0.10161	0.42934	0.02743	0.71685	0.16348	0.00564	0.02596	0.08378	0.18606	0.18606	turquoise
DNM1	0.01641	-0.13897	-0.18616	-0.08255	0.10086	-0.28979	-0.15063	0.35188	-0.35188	0.83129	0.06987	0.01477	0.28311	0.1893	0.00012	0.04923	2.36E-06	2.36E-06	brown
XKR8	-0.07649	-0.25662	-0.07171	0.07657	-0.02614	-0.0799	-0.17148	0.23052	-0.23052	0.32004	0.0007	0.35134	0.31953	0.73437	0.29887	0.02493	0.00242	0.00242	grey
SSC5D	-0.01209	-0.08652	-0.01808	0.05572	-0.1269	0.07337	0.00942	0.31348	-0.31348	0.87527	0.26051	0.8144	0.46913	0.09813	0.34023	0.90268	2.98E-05	2.98E-05	black
C7orf31	-0.07162	-0.08616	-0.06535	0.05172	-0.05947	-0.03991	-0.17014	0.23898	-0.23898	0.35192	0.26248	0.3958	0.50172	0.43971	0.60428	0.02609	0.00164	0.00164	turquoise
PNRC1	-0.12423	-0.16845	0.01473	0.11627	-0.08742	0.01549	-0.0963	0.29357	-0.29357	0.10549	0.02764	0.84837	0.12992	0.25554	0.84067	0.21022	9.74E-05	9.74E-05	blue
MGAT2	0.00216	-0.04678	-0.00816	0.03162	0.13407	0.07335	0.09477	-0.04745	0.04745	0.97763	0.54347	0.91567	0.68136	0.08041	0.34035	0.21758	0.53772	0.53772	grey
P2RY11	0.01055	-0.04155	-0.16184	-0.10341	0.09058	-0.10069	-0.06134	0.02107	-0.02107	0.89108	0.58952	0.03445	0.17831	0.23873	0.19005	0.42545	0.78449	0.78449	green
RNF145	-0.08067	-0.21755	-0.06924	-0.04809	-0.03052	-0.06113	-0.05384	0.14119	-0.14119	0.29422	0.00426	0.36821	0.53219	0.69195	0.42705	0.48431	0.06547	0.06547	turquoise
MID1	-0.06294	-0.18397	-0.06415	0.08073	-0.11364	0.10498	-0.03649	0.18948	-0.18948	0.41346	0.01601	0.4045	0.29387	0.13889	0.17177	0.63563	0.01306	0.01306	turquoise
ZNF181	-0.09062	-0.18791	-0.20236	-0.04057	0.00912	-0.17485	-0.18425	0.462	-0.462	0.23849	0.01385	0.00795	0.59831	0.90574	0.02217	0.01585	2.01E-10	2.01E-10	turquoise
ZNF77	-0.02064	0.00605	-0.22144	-0.13878	0.11325	-0.19383	-0.08111	0.15994	-0.15994	0.78869	0.93741	0.00361	0.07026	0.14025	0.01108	0.29159	0.03665	0.03665	grey
DUSP22	0.01995	-0.00643	0.00536	0.02569	-0.09195	0.12261	0.02593	0.23795	-0.23795	0.79565	0.93349	0.94457	0.73871	0.23164	0.11013	0.73639	0.00173	0.00173	blue
STON2	0.0659	-0.11935	-0.05033	-0.03133	0.0686	-0.11711	-0.03065	-0.09176	0.09176	0.39178	0.11998	0.51324	0.68419	0.37267	0.12714	0.6907	0.23262	0.23262	grey
MDH2	0.13762	-0.00272	-0.04565	-0.08786	0.09663	-0.13421	0.12371	-0.31562	0.31562	0.07265	0.97179	0.55328	0.25316	0.20867	0.0801	0.10694	2.61E-05	2.61E-05	grey
C21orf91	-0.13341	-0.12157	-0.02747	0.06772	-0.17781	0.09773	0.06665	0.39094	-0.39094	0.08193	0.11319	0.72136	0.37883	0.01998	0.2035	0.38641	1.25E-07	1.25E-07	blue
ZSWIM6	-0.04806	-0.21632	0.03596	0.06719	-0.00953	0.14487	0.01039	0.05865	-0.05865	0.53251	0.00449	0.64058	0.38257	0.90156	0.05869	0.89268	0.44607	0.44607	turquoise
PEX11G	0.0066	0.03882	-0.16104	-0.07312	0.1861	-0.03575	0.144	-0.30954	0.30954	0.93171	0.61421	0.03536	0.34191	0.01481	0.6425	0.06025	3.79E-05	3.79E-05	green
KCNH2	0.05348	-0.08517	-0.3131	-0.14919	0.12212	-0.35812	-0.19928	0.27542	-0.27542	0.48725	0.26807	3.05E-05	0.05147	0.11158	1.52E-06	0.00897	0.00027	0.00027	brown
PPIP5K2	-0.09782	-0.13896	-0.0208	0.0169	-0.05332	0.01525	-0.00194	0.27229	-0.27229	0.20306	0.06989	0.78718	0.82639	0.4885	0.84305	0.97994	0.00031	0.00031	turquoise
KCNS1	0.00223	-0.16664	0.02937	0.04475	-0.03135	0.10199	0.09645	-0.00222	0.00222	0.97687	0.02938	0.70295	0.56114	0.68398	0.18439	0.20951	0.97702	0.97702	grey
CHD1	-0.06642	-0.16775	-0.00042	0.05247	-0.11245	0.11492	0.01794	0.17309	-0.17309	0.38803	0.0283	0.99564	0.49551	0.14311	0.13447	0.81582	0.02358	0.02358	turquoise
LRRC40	-0.14163	-0.12181	0.04089	0.06867	-0.14119	0.05927	0.0261	0.24409	-0.24409	0.06463	0.11248	0.59537	0.37216	0.06548	0.44129	0.73475	0.00129	0.00129	turquoise
CHSY3	-0.03554	-0.01339	0.02706	0.05458	-0.21165	0.1243	0.06178	0.35947	-0.35947	0.64449	0.86196	0.72535	0.47827	0.00545	0.10527	0.42217	1.37E-06	1.37E-06	blue
C5orf22	-0.04294	-0.07564	0.00017	-0.0076	-0.15447	0.06339	0.15613	0.0136	-0.0136	0.57705	0.32546	0.99823	0.9214	0.04366	0.41015	0.04143	0.85987	0.85987	turquoise
CDK19	-0.05887	-0.12981	0.00097	0.04573	-0.15886	0.05876	0.08193	0.21845	-0.21845	0.44433	0.09061	0.98993	0.5526	0.03796	0.44519	0.28672	0.0041	0.0041	turquoise
GIPC3	-0.10508	-0.18635	-0.06052	0.02168	-0.14813	0.01346	-0.12132	0.37675	-0.37675	0.17139	0.01467	0.43169	0.77838	0.05317	0.86126	0.11394	3.80E-07	3.80E-07	blue
EGFL7	-0.09541	-0.09097	-0.19481	-0.01249	0.16127	-0.22061	-0.13909	0.32904	-0.32904	0.21448	0.23671	0.01067	0.87122	0.0351	0.00374	0.06962	1.11E-05	1.11E-05	grey
NIPSNAP3	-0.08663	-0.11056	-0.05809	0.00422	-0.05492	-0.054	-0.05692	0.13755	-0.13755	0.25989	0.15001	0.45044	0.95629	0.47558	0.483	0.45959	0.07281	0.07281	turquoise
HAGH	0.07213	-0.09193	-0.16084	-0.19122	0.13379	-0.25485	-0.22028	0.04786	-0.04786	0.34849	0.23174	0.03559	0.01223	0.08107	0.00077	0.00379	0.53421	0.53421	grey
AZGP1	0.14629	0.12877	0.0811	0.11134	0.04853	0.19433	-0.03011	-0.19114	0.19114	0.05623	0.09325	0.29167	0.14713	0.52847	0.01087	0.69589	0.01227	0.01227	grey
BICRAL	-0.03788	-0.11193	-0.08268	-0.00449	-0.10041	0.01727	-0.08095	0.35412	-0.35412	0.62283	0.14496	0.28236	0.95349	0.19131	0.82263	0.29259	2.02E-06	2.02E-06	turquoise
KIF13B	0.01963	-0.05391	0.07111	0.05441	-0.04944	0.15968	0.13048	-0.18761	0.18761	0.79887	0.48375	0.35538	0.4797	0.52079	0.03696	0.08893	0.01401	0.01401	turquoise
PRAM1	-0.07953	-0.01393	0.04643	0.08419	-0.10856	0.12301	0.03764	0.2597	-0.2597	0.30111	0.85647	0.54645	0.27363	0.15753	0.10896	0.62501	0.0006	0.0006	blue
RAD52	0.08465	-0.11645	-0.13513	-0.01257	0.20392	-0.08711	-0.11942	-0.00663	0.00663	0.27098	0.12932	0.07804	0.87034	0.00747	0.25726	0.11976	0.93146	0.93146	red
MAX	0.05359	-0.12774	-0.00612	0.05294	0.00061	0.07015	0.06298	-0.02546	0.02546	0.48634	0.09591	0.93664	0.49161	0.99369	0.36192	0.41318	0.74095	0.74095	turquoise
RNASE7	0.07769	-0.00038	-0.04645	0.02702	-0.0168	0.03919	0.12088	-0.03158	0.03158	0.31252	0.99605	0.54636	0.72576	0.82736	0.6108	0.11529	0.68179	0.68179	grey
MYRF	0.00602	0.00022	-0.0537	0.02591	0.15552	0.06434	-0.00692	-0.25211	0.25211	0.9377	0.99769	0.48541	0.73655	0.04224	0.40314	0.92843	0.00088	0.00088	yellow
ANKMY2	-0.01553	-0.16474	0.02403	0.04856	0.01296	0.08733	0.06611	-0.09327	0.09327	0.8402	0.0313	0.75503	0.52827	0.8664	0.25606	0.39032	0.22498	0.22498	turquoise
FOXO6	-0.02906	-0.17526	-0.15368	0.07204	0.10517	-0.06129	-0.16846	0.05565	-0.05565	0.70596	0.02186	0.04476	0.3491	0.17102	0.42582	0.02763	0.46969	0.46969	grey
MPP7	0.04973	0.06605	0.04511	0.15	-0.0082	0.03864	-0.00541	0.12278	-0.12278	0.51835	0.39071	0.55793	0.05021	0.91525	0.61583	0.94401	0.10965	0.10965	grey
KAT2A	0.0272	-0.01766	-0.0525	-0.02191	0.24354	-0.16855	-0.01255	-0.24386	0.24386	0.724	0.81869	0.4953	0.77605	0.00133	0.02754	0.87057	0.00131	0.00131	red
AK2	-0.00886	-0.07114	0.04822	0.00046	-0.03363	0.03064	0.09569	-0.20811	0.20811	0.90839	0.35517	0.53115	0.99527	0.66236	0.69079	0.21315	0.00631	0.00631	turquoise
PLXNA3	0.01135	-0.2233	-0.02962	-0.05746	0.05935	-0.06039	0.0572	-0.27079	0.27079	0.88288	0.00333	0.70054	0.45539	0.44068	0.43268	0.45741	0.00034	0.00034	red
CCT6A	0.00839	-0.08307	0.00684	0.01552	-0.06583	-0.02724	0.12826	-0.20765	0.20765	0.91331	0.28005	0.92922	0.84031	0.39231	0.72358	0.09457	0.00643	0.00643	turquoise
TOLLIP	0.04662	-0.13628	-0.24755	-0.14834	0.18418	-0.29796	-0.17687	0.04742	-0.04742	0.54483	0.07551	0.0011	0.05284	0.01589	7.56E-05	0.02066	0.53792	0.53792	grey
ERMN	-0.12739	0.03455	0.02849	0.03092	-0.14835	0.01532	0.06709	0.08874	-0.08874	0.09684	0.65369	0.71146	0.68805	0.05282	0.84237	0.38328	0.24843	0.24843	black
PSCA	0.02331	0.02227	0.11874	0.03934	-0.04668	0.20984	0.21765	-0.2279	0.2279	0.76214	0.77253	0.12189	0.60945	0.54432	0.00588	0.00424	0.00272	0.00272	yellow
TCEA2	-0.02243	-0.11276	-0.19883	-0.01856	0.06642	-0.2591	-0.11283	0.10235	-0.10235	0.77094	0.14198	0.00913	0.80963	0.38806	0.00062	0.14176	0.18285	0.18285	grey
STK3	-0.00473	-0.1117	0.00249	0.10435	-0.17441	0.11956	0.18775	-0.08182	0.08182	0.95103	0.1458	0.97421	0.17439	0.02252	0.11935	0.01393	0.28739	0.28739	turquoise
SLC12A7	-0.00646	-0.12189	-0.08397	0.00369	-0.03863	0.01905	0.02427	-0.12285	0.12285	0.9332	0.11224	0.27487	0.96182	0.61591	0.80467	0.7527	0.10942	0.10942	grey
SLC29A4	-0.04091	-0.12186	-0.25249	-0.08845	0.13422	-0.30871	-0.2222	0.41242	-0.41242	0.59522	0.11233	0.00086	0.24998	0.08009	3.99E-05	0.00349	2.08E-08	2.08E-08	brown
IFNGR2	-0.02111	-0.12267	0.02529	-0.05327	-0.00266	0.15885	0.12677	-0.19206	0.19206	0.78405	0.10995	0.74265	0.48899	0.9725	0.03796	0.09849	0.01185	0.01185	turquoise
AL121753	0.05591	-0.18614	0.04758	0.02252	-0.07176	0.08044	0.07533	-0.02456	0.02456	0.46761	0.01478	0.5366	0.77002	0.35095	0.29565	0.32747	0.74986	0.74986	grey
TMEM250	0.05778	-0.06771	-0.0818	-0.01079	-0.06879	-0.02089	0.15525	-0.24877	0.24877	0.45289	0.3789	0.28748	0.88865	0.37133	0.78619	0.0426	0.00103	0.00103	grey
USP6NL	-0.01241	-0.01943	0.10306	0.02773	-0.10988	0.13255	0.04405	-0.08898	0.08898	0.87204	0.80086	0.17981	0.71881	0.15253	0.08394	0.56727	0.24712	0.24712	turquoise
UTY	0.87392	-0.05639	-0.06499	-0.00277	0.04933	-0.00228	0.01751	0.07327	-0.07327	7.87E-55	0.46384	0.39838	0.97132	0.52173	0.97636	0.82018	0.34091	0.34091	grey
C9orf116	-0.01074	-0.08952	-0.2164	-0.04036	0.11127	-0.19694	-0.04193	-0.13859	0.13859	0.88913	0.24426	0.00447	0.60021	0.14738	0.00983	0.58605	0.07064	0.07064	grey
MGAT4B	0.06935	0.01392	0.04211	-0.01402	-0.02555	0.16698	0.23705	-0.47518	0.47518	0.36743	0.85663	0.58446	0.85559	0.74007	0.02905	0.0018	5.13E-11	5.13E-11	yellow
EDA2R	-0.06957	-0.05394	-0.08382	0.02781	-0.09206	-0.15795	-0.01001	0.51544	-0.51544	0.36593	0.48347	0.27575	0.71805	0.23108	0.03909	0.8966	5.44E-13	5.44E-13	turquoise
SAMD9L	-0.04858	-0.00336	0.14021	0.16007	-0.19616	0.25151	0.21406	-0.01583	0.01583	0.52803	0.96526	0.06738	0.0365	0.01013	0.00091	0.00493	0.83716	0.83716	tan
WSCD2	-0.09122	-0.0309	-0.11693	-0.02599	-0.0727	0.0103	0.05211	0.25904	-0.25904	0.23538	0.68823	0.12774	0.73581	0.34467	0.89359	0.49847	0.00062	0.00062	brown
MCM6	-0.1046	0.08125	0.06125	0.03906	-0.19154	0.09613	0.24659	-0.03915	0.03915	0.17336	0.29079	0.42615	0.61201	0.01209	0.21105	0.00115	0.61116	0.61116	pink
NDUFB3	0.06573	0.02226	-0.00904	-0.05871	0.04288	0.01748	0.15668	-0.30928	0.30928	0.393	0.77255	0.90662	0.4456	0.57764	0.82046	0.04071	3.85E-05	3.85E-05	turquoise
DVL1	-0.00413	0.0262	0.01012	0.02534	0.06638	0.03958	0.12865	-0.4078	0.4078	0.95721	0.73373	0.89549	0.74212	0.38833	0.6073	0.09354	3.09E-08	3.09E-08	green
SH3RF2	-0.01307	0.024	0.12422	0.07933	-0.0126	0.24948	0.20786	-0.38585	0.38585	0.86532	0.75535	0.1055	0.30234	0.87007	0.001	0.00637	1.87E-07	1.87E-07	yellow
KCNJ2	-0.01175	-0.09324	-0.01166	0.03322	0.10306	-0.24961	-0.14823	0.35947	-0.35947	0.87877	0.22516	0.87969	0.6662	0.1798	0.00099	0.05301	1.38E-06	1.38E-06	grey
AC092718	0.01337	-0.13473	-0.10118	0.04118	-0.07046	0.06303	0.14537	0.0324	-0.0324	0.86218	0.07893	0.18791	0.59282	0.3598	0.41282	0.05781	0.67394	0.67394	grey
MPPED2	-0.04416	-0.11481	-0.17118	0.00501	0.01944	-0.24063	-0.17927	0.51295	-0.51295	0.56626	0.13486	0.02518	0.94816	0.8008	0.00152	0.01897	7.34E-13	7.34E-13	brown
GALNS	-0.04248	-0.09176	0.04147	0.05954	-0.08129	0.00457	0.06569	0.12534	-0.12534	0.58115	0.23261	0.59024	0.43917	0.29056	0.95265	0.39329	0.10238	0.10238	black
GORAB	-0.03212	-0.05286	-0.01832	0.01401	0.0259	0.11414	0.01367	0.02033	-0.02033	0.67665	0.49235	0.81203	0.85565	0.73663	0.13714	0.85917	0.79182	0.79182	turquoise
GAS2L1	-0.07478	-0.03609	-0.13783	-0.01466	-0.01531	-0.01015	0.0707	-0.0699	0.0699	0.33105	0.6393	0.07223	0.84905	0.84247	0.89521	0.35813	0.36363	0.36363	grey
C4BPB	0.12325	0.0907	0.15119	0.06018	0.07614	0.21522	0.03084	-0.45286	0.45286	0.10828	0.23807	0.04839	0.43424	0.32227	0.0047	0.68882	5.00E-10	5.00E-10	yellow
AUP1	0.04615	0.04676	-0.00998	-0.0134	0.04245	0.06599	0.16902	-0.40578	0.40578	0.54892	0.54364	0.89689	0.86189	0.58148	0.39113	0.02711	3.67E-08	3.67E-08	yellow
NFATC2	-0.13123	-0.18684	-0.05253	0.059	-0.07762	0.12109	-0.11394	0.45288	-0.45288	0.08711	0.01441	0.49499	0.44332	0.31291	0.11464	0.13784	4.99E-10	4.99E-10	blue
MCM7	0.03749	0.05075	0.00655	-0.06486	-0.02252	0.02212	0.25251	-0.36225	0.36225	0.62643	0.50974	0.93226	0.39934	0.77003	0.77396	0.00086	1.12E-06	1.12E-06	pink
ATP5MF	0.05526	-0.06349	-0.03276	-0.05968	0.07714	-0.08592	0.1496	-0.41927	0.41927	0.47282	0.40934	0.67056	0.43813	0.31593	0.26386	0.05083	1.15E-08	1.15E-08	grey
TCAF1	-0.05604	-0.11952	-0.07488	-0.06217	-0.05631	-0.069	-0.05291	0.26606	-0.26606	0.46657	0.11947	0.33038	0.41924	0.46447	0.36989	0.49192	0.00044	0.00044	turquoise
CCDC50	-0.09698	-0.13498	-0.06592	0.03455	-0.18423	0.08166	0.03677	0.158	-0.158	0.20702	0.07837	0.39163	0.6537	0.01586	0.28834	0.63301	0.03902	0.03902	blue
TTI1	0.04798	-0.1429	-0.07651	-0.02906	-0.05512	-0.08079	0.07798	-0.0189	0.0189	0.53314	0.06224	0.31993	0.70595	0.47394	0.29353	0.31071	0.80613	0.80613	turquoise
SLAMF1	-0.12646	-0.02543	0.04595	0.05365	-0.15004	0.1344	-0.03138	0.4288	-0.4288	0.09931	0.74126	0.55063	0.48587	0.05014	0.07967	0.68365	4.88E-09	4.88E-09	blue
MAPK8IP3	0.02566	-0.08778	-0.08637	-0.08248	0.26902	-0.14902	-0.04559	-0.1413	0.1413	0.73907	0.25362	0.26136	0.2835	0.00037	0.05174	0.55381	0.06527	0.06527	red
DTNA	-0.03249	-0.12862	-0.24032	-0.06533	0.07057	-0.24118	-0.29508	0.52064	-0.52064	0.67316	0.09362	0.00155	0.39594	0.35904	0.00148	8.93E-05	2.89E-13	2.89E-13	brown
MALSU1	0.04007	-0.06093	-0.13397	-0.02921	0.05019	-0.06592	0.13639	-0.20988	0.20988	0.60283	0.42852	0.08065	0.70448	0.51447	0.39168	0.07528	0.00587	0.00587	grey
MBNL2	-0.0377	-0.13453	-0.01047	0.04436	-0.0649	0.0077	-0.10646	0.19947	-0.19947	0.62442	0.07938	0.89191	0.56452	0.39903	0.9204	0.16577	0.00891	0.00891	turquoise
NME7	0.07819	-0.09608	0.00079	-0.00149	-0.10321	0.07211	0.06477	-0.05648	0.05648	0.30938	0.21128	0.9918	0.98454	0.17915	0.34859	0.39997	0.46314	0.46314	turquoise
RND1	0.03186	-0.1056	-0.04286	0.04092	0.1587	0.14303	-0.13587	-0.11608	0.11608	0.67912	0.16926	0.5778	0.59515	0.03815	0.062	0.07641	0.13055	0.13055	grey
HHAT	-0.02624	-0.19564	-0.06541	0.00585	-0.03858	0.13566	0.06342	0.02092	-0.02092	0.7333	0.01033	0.39531	0.93943	0.61634	0.07687	0.40992	0.78593	0.78593	turquoise
TFAP4	0.16316	0.12922	-0.06669	0.02789	0.05899	0.03454	0.13439	-0.3457	0.3457	0.03299	0.09209	0.38616	0.71731	0.44345	0.65378	0.07971	3.64E-06	3.64E-06	grey
ZNF280C	-0.10703	-0.10137	-0.05058	0.0324	-0.06372	0.00888	-0.00215	0.23361	-0.23361	0.16351	0.1871	0.51119	0.67397	0.40769	0.90825	0.97777	0.0021	0.0021	turquoise
MAPRE2	-0.05339	-0.13769	-0.05755	-0.02219	0.04399	-0.07405	-0.21488	0.43868	-0.43868	0.48796	0.07252	0.45468	0.77332	0.56776	0.33575	0.00477	1.96E-09	1.96E-09	turquoise
LSM2	0.05378	0.09671	-0.07289	-0.07446	-0.03053	-0.03873	0.18026	-0.1903	0.1903	0.48482	0.20826	0.34344	0.33307	0.69177	0.61497	0.01831	0.01266	0.01266	grey
TXNDC15	-0.0752	-0.16878	-0.13255	-0.04167	0.02387	-0.21905	-0.20907	0.46229	-0.46229	0.32832	0.02733	0.08394	0.58837	0.75661	0.004	0.00606	1.95E-10	1.95E-10	turquoise
TRAIP	0.06141	0.10886	0.05512	-0.07949	-0.0455	0.09136	0.23784	-0.40973	0.40973	0.42493	0.15641	0.474	0.30136	0.55454	0.23467	0.00173	2.62E-08	2.62E-08	pink
OSBPL7	0.07777	0.00697	0.03634	-0.01844	0.2359	0.10736	0.04123	-0.37483	0.37483	0.31197	0.92791	0.63699	0.81076	0.0019	0.16223	0.59231	4.40E-07	4.40E-07	grey
UPK2	0.02165	-0.10331	-0.06377	-0.02326	-0.04292	-0.03441	0.15057	-0.21525	0.21525	0.77865	0.17875	0.40732	0.76266	0.57723	0.65499	0.04933	0.00469	0.00469	grey
DNAJC4	0.01121	0.01866	-0.04331	-0.08423	0.04124	-0.02106	0.05621	-0.17859	0.17859	0.88431	0.80863	0.57376	0.27339	0.59229	0.78454	0.46523	0.01944	0.01944	green
SPACA9	-0.00773	-0.11393	-0.22047	-0.06954	0.02965	-0.21204	-0.05074	0.16216	-0.16216	0.92003	0.13786	0.00376	0.3661	0.70029	0.00537	0.50982	0.03409	0.03409	grey
LRRC37A2	0.04898	-0.26984	-0.01648	0.05755	0.10537	0.06414	-0.02851	0.09432	-0.09432	0.52462	0.00036	0.83059	0.45468	0.17019	0.40459	0.7113	0.21978	0.21978	grey
AARSD1	-0.00544	-0.08467	-0.14483	-0.09489	0.23214	-0.17461	-0.07503	-0.06446	0.06446	0.94375	0.2709	0.05877	0.21701	0.00225	0.02237	0.32937	0.40227	0.40227	red
GPSM1	-0.05818	-0.17182	-0.20056	0.03886	-0.0186	-0.21148	0.01357	0.26187	-0.26187	0.44971	0.02464	0.00853	0.61379	0.80917	0.00549	0.86012	0.00054	0.00054	grey
QSOX1	0.09591	-0.00901	0.08484	0.05412	-0.05819	0.19477	0.28831	-0.42614	0.42614	0.21206	0.90688	0.26989	0.48203	0.44968	0.01069	0.00013	6.21E-09	6.21E-09	yellow
BABAM2	0.0245	-0.0145	-0.03552	-0.01111	-0.13365	0.05988	0.14864	-0.0041	0.0041	0.75047	0.85073	0.64462	0.8853	0.08138	0.43656	0.05235	0.95753	0.95753	turquoise
SNRNP200	-0.02984	-0.09048	-0.02496	0.00193	-0.11112	0.07687	0.08754	0.08584	-0.08584	0.69839	0.23921	0.74587	0.98	0.14794	0.31763	0.2549	0.26425	0.26425	turquoise
KIF4A	-0.03741	0.03865	0.03022	-0.01786	-0.08481	0.07158	0.3133	-0.41039	0.41039	0.62712	0.61577	0.69476	0.81663	0.27005	0.35218	3.01E-05	2.48E-08	2.48E-08	pink
FLT4	-0.17062	-0.05467	-0.0954	0.04287	-0.00677	-0.06983	-0.09193	0.47854	-0.47854	0.02567	0.47758	0.21455	0.57769	0.93001	0.36412	0.23177	3.59E-11	3.59E-11	blue
ZBED5	-0.01705	-0.11128	-0.08198	-0.08163	0.06436	-0.06918	0.00764	0.0652	-0.0652	0.82487	0.14733	0.28644	0.28854	0.40301	0.36864	0.92103	0.39685	0.39685	red
HOXB6	-0.016	0.03774	0.00476	0.09455	0.07455	0.10327	0.17699	-0.33314	0.33314	0.83548	0.62411	0.9507	0.21866	0.33249	0.17892	0.02057	8.50E-06	8.50E-06	yellow
SLC9A3R1	0.04914	-0.10108	-0.13281	-0.03969	0.12576	-0.09768	0.07767	-0.21517	0.21517	0.52328	0.18836	0.08333	0.60628	0.10123	0.20371	0.3126	0.00471	0.00471	grey
HNRNPUL	0.02643	-0.10023	-0.05405	-0.00318	-0.11318	0.05363	0.0349	0.03521	-0.03521	0.73144	0.19212	0.48258	0.96711	0.14049	0.486	0.65045	0.64749	0.64749	turquoise

NOLC1	-0.01774	0.02475	0.05209	-0.00478	-0.11271	0.08178	0.18775	-0.06299	0.06299	0.81784	0.74801	0.49864	0.95057	0.14216	0.28761	0.01393	0.41307	0.41307	turquoise
PGM2	-0.05945	-0.07907	0.03509	0.07402	-0.15741	0.24539	0.20541	-0.02062	0.02062	0.43988	0.30396	0.64868	0.33594	0.03976	0.00122	0.00704	0.78898	0.78898	turquoise
SYTL1	0.07654	0.01224	0.03878	-0.01498	0.17284	-0.01103	0.08757	-0.45053	0.45053	0.31974	0.87375	0.61454	0.84579	0.02378	0.88612	0.25476	6.29E-10	6.29E-10	green
AKT3	-0.09998	-0.15291	-0.10493	-0.01691	-0.1991	-0.06388	-0.13832	0.53028	-0.53028	0.19323	0.04586	0.17197	0.82627	0.00904	0.40654	0.0712	8.72E-14	8.72E-14	blue
ACOT7	-0.01353	-0.02274	0.01844	-0.08374	0.00735	-0.0574	0.1176	-0.17732	0.17732	0.86056	0.76784	0.81077	0.27621	0.92399	0.45588	0.12556	0.02033	0.02033	grey
TACC3	-0.04651	0.09043	-0.02826	-0.02974	0.01155	0.00389	0.1906	-0.30488	0.30488	0.54584	0.23948	0.71368	0.69943	0.88085	0.9597	0.01252	5.02E-05	5.02E-05	pink
INTS3	-0.05025	-0.17006	-0.04661	-0.06359	0.08289	-0.02352	-0.10494	0.11876	-0.11876	0.51392	0.02616	0.54492	0.40866	0.28108	0.76012	0.17193	0.12185	0.12185	red
WWOX	-0.04216	-0.04681	-0.18578	-0.05906	0.06286	-0.20013	-0.16616	0.39448	-0.39448	0.58406	0.54322	0.01498	0.44288	0.41406	0.00868	0.02986	9.36E-08	9.36E-08	turquoise
TMEM187	-0.07377	-0.04571	-0.11473	-0.02419	0.04713	-0.04953	0.13416	-0.12275	0.12275	0.3376	0.55275	0.13512	0.7535	0.54044	0.52002	0.08021	0.10973	0.10973	grey
ERMP1	0.10642	-0.12361	-0.00867	-0.03425	-0.07353	0.04355	0.12696	-0.08094	0.08094	0.16593	0.10724	0.91034	0.65649	0.33919	0.57167	0.09797	0.29264	0.29264	turquoise
SPATA25	0.00569	-0.15323	-0.10544	0.01701	0.19007	-0.12606	-0.01797	0.04127	-0.04127	0.94114	0.0454	0.1699	0.82519	0.01278	0.10039	0.81559	0.59197	0.59197	red
PANX1	-0.0885	-0.1287	0.05003	0.08358	-0.24291	0.17433	0.14611	0.05966	-0.05966	0.24971	0.09341	0.51583	0.27711	0.00137	0.02258	0.05654	0.43826	0.43826	turquoise
FBXO31	-0.01803	-0.1283	-0.1322	-0.04435	-0.00881	-0.12439	-0.13116	0.33072	-0.33072	0.81492	0.09445	0.08478	0.56459	0.90899	0.10502	0.08727	9.96E-06	9.96E-06	turquoise
ARNT2	-0.04885	-0.13915	-0.19748	-0.16979	0.05241	-0.26625	-0.22252	0.50523	-0.50523	0.52576	0.0695	0.00963	0.02641	0.49599	0.00043	0.00344	1.83E-12	1.83E-12	brown
SP4	-0.1026	-0.17238	-0.14035	-0.04152	-0.11097	-0.03525	-0.1002	0.3617	-0.3617	0.18176	0.02416	0.0671	0.58976	0.14847	0.64717	0.19223	1.17E-06	1.17E-06	turquoise
FAT1	-0.07385	-0.06284	0.03661	0.03205	-0.12524	0.20895	0.13123	-0.12164	0.12164	0.33712	0.41422	0.63447	0.67727	0.10266	0.00609	0.0871	0.11301	0.11301	turquoise
CARMIL1	0.03814	0.06267	0.07873	0.05469	0.04756	0.21484	0.09648	-0.16652	0.16652	0.62042	0.41548	0.30606	0.47745	0.53679	0.00478	0.20938	0.0295	0.0295	turquoise
MILR1	-0.10395	-0.07396	0.10593	0.05651	-0.20625	0.19086	0.20126	0.16876	-0.16876	0.17606	0.33634	0.1679	0.4629	0.0068	0.0124	0.0083	0.02735	0.02735	blue
CCDC9	0.14948	0.02156	0.03922	-0.01399	-0.09158	0.18307	0.2093	-0.18818	0.18818	0.05102	0.77956	0.61056	0.85592	0.23353	0.01655	0.00601	0.01371	0.01371	grey
MICALL2	0.0265	-0.02748	0.0402	-0.00072	0.14238	0.11936	0.21272	-0.45776	0.45776	0.73077	0.72127	0.60166	0.99256	0.06322	0.11996	0.00522	3.08E-10	3.08E-10	green
DDX23	0.0922	-0.16727	-0.05064	-0.00115	0.07224	-0.04546	-0.02339	-0.09228	0.09228	0.23038	0.02877	0.51072	0.9881	0.34775	0.55492	0.76138	0.22995	0.22995	turquoise
TLE6	0.08901	-0.11103	-0.14667	-0.11751	0.24711	-0.15272	-0.0324	-0.14134	0.14134	0.24696	0.14827	0.05558	0.12585	0.00112	0.04613	0.67402	0.06518	0.06518	grey
BBC3	0.12047	0.05946	0.009	0.0237	0.04667	0.11687	0.14147	-0.49744	0.49744	0.11653	0.43977	0.90703	0.75829	0.54441	0.12794	0.06495	4.47E-12	4.47E-12	green
EIF1	0.09032	-0.17446	-0.11605	-0.08944	0.12108	-0.19396	-0.1356	-0.00164	0.00164	0.24008	0.02248	0.13066	0.24467	0.11468	0.01102	0.07699	0.98307	0.98307	grey
IL4I1	-0.1584	0.08098	0.05572	0.07771	-0.11204	0.10838	0.12202	0.06046	-0.06046	0.03852	0.29235	0.46918	0.31236	0.14459	0.15823	0.11187	0.43212	0.43212	blue
KIFC3	0.0398	-0.15232	-0.13828	0.05725	-0.00653	-0.12068	0.01981	0.0537	-0.0537	0.60523	0.04672	0.07129	0.45702	0.93244	0.1159	0.79708	0.48544	0.48544	grey
RPS23	0.10259	0.11571	-0.02884	-0.0386	0.00909	-0.03395	-0.05256	0.04598	-0.04598	0.1818	0.13179	0.7081	0.61616	0.90607	0.65936	0.49478	0.55035	0.55035	purple
DDX55	0.01642	-0.12298	-0.03908	-0.04302	0.09903	-0.01655	0.01217	-0.0608	0.0608	0.83121	0.10905	0.61179	0.57634	0.1975	0.82986	0.87447	0.42957	0.42957	turquoise
PLAUR	0.00347	0.04028	0.13105	0.06912	-0.14817	0.27547	0.24252	-0.2446	0.2446	0.96404	0.60096	0.08754	0.369	0.05311	0.00027	0.00139	0.00126	0.00126	yellow
ZNF212	0.00225	-0.15348	-0.08237	-0.04864	0.04997	-0.16673	0.03814	-0.06994	0.06994	0.97668	0.04506	0.28414	0.52753	0.51629	0.02929	0.62037	0.36335	0.36335	grey
NYNRIN	-0.01789	-0.1955	-0.00627	0.03619	-0.02476	0.10941	0.05454	-0.05166	0.05166	0.81633	0.01039	0.93517	0.63844	0.74791	0.15429	0.47866	0.50222	0.50222	turquoise
OPN1SW	-0.18289	-0.13888	-0.00959	0.00337	-0.05751	-0.06042	0.02198	0.16605	-0.16605	0.01666	0.07005	0.90092	0.96513	0.45499	0.43244	0.77533	0.02996	0.02996	black
ZNF415	-0.07165	-0.15502	-0.04543	0.11201	-0.04733	0.00579	-0.09449	0.43719	-0.43719	0.35173	0.04291	0.55518	0.14469	0.53869	0.9401	0.21893	2.25E-09	2.25E-09	turquoise
CEACAM4	-0.10271	-0.08558	0.03516	0.02108	-0.2255	0.12501	0.0836	0.25986	-0.25986	0.18131	0.26573	0.64803	0.78433	0.00302	0.10329	0.27701	0.0006	0.0006	blue
GAS2L3	0.00029	-0.14	-0.03068	0.02749	0.10403	0.05156	0.1365	-0.1037	0.1037	0.99698	0.06781	0.6904	0.72119	0.17573	0.50302	0.07505	0.17712	0.17712	grey
SLFN5	-0.0375	-0.01984	0.06893	0.10342	-0.17846	0.19566	0.18002	0.09942	-0.09942	0.6263	0.79678	0.37033	0.17828	0.01952	0.01033	0.01847	0.19573	0.19573	turquoise
PLCB3	0.04856	0.0358	0.12952	0.03305	0.01849	0.24168	0.29311	-0.46686	0.46686	0.52821	0.64199	0.09134	0.66785	0.81029	0.00145	1.00E-04	1.22E-10	1.22E-10	yellow
ZNF837	-0.01142	-0.06513	-0.24062	-0.13719	0.2248	-0.20793	-0.10947	-0.09695	0.09695	0.88212	0.39735	0.00152	0.07356	0.00312	0.00635	0.15409	0.20715	0.20715	green
PTN	-0.12862	-0.04571	-0.14016	-0.0052	-0.09486	-0.02154	-0.01055	0.38585	-0.38585	0.09364	0.55274	0.06749	0.94623	0.21714	0.77972	0.89102	1.87E-07	1.87E-07	grey
TSNARE1	0.0687	0.05004	-0.05899	-0.02221	0.09903	-0.05862	0.00964	-0.26514	0.26514	0.37198	0.51568	0.44344	0.77313	0.19754	0.44633	0.9004	0.00046	0.00046	green
SELENBP1	0.08023	0.05342	0.02916	-0.00851	0.17879	0.10357	0.08883	-0.2232	0.2232	0.29691	0.48775	0.70501	0.912	0.0193	0.17766	0.24795	0.00334	0.00334	yellow
GABARAP	0.013	-0.25247	-0.09082	0.01046	-0.09668	-0.04426	-0.13199	0.30651	-0.30651	0.86594	0.00086	0.23747	0.89204	0.2084	0.56538	0.08528	4.56E-05	4.56E-05	turquoise
CD58	-0.05484	-0.05985	0.06635	0.07328	-0.03903	0.15337	0.15385	-0.31746	0.31746	0.47621	0.43679	0.38857	0.34082	0.61227	0.04521	0.04453	2.33E-05	2.33E-05	yellow
HSPA1A	0.18532	-0.13624	0.14036	0.04679	0.01195	0.17605	0.05993	-0.23658	0.23658	0.01524	0.0756	0.06709	0.5434	0.87673	0.02126	0.43618	0.00184	0.00184	grey
SIMC1	-0.02158	-0.20528	-0.06958	-0.09056	0.11764	-0.05097	-0.06526	0.03267	-0.03267	0.77936	0.00707	0.36581	0.23882	0.12544	0.50796	0.39642	0.67141	0.67141	turquoise
UBE2Q1	0.09092	-0.07436	-0.04803	-0.10201	0.01244	-0.00438	0.03722	-0.11037	0.11037	0.23696	0.33376	0.53277	0.18431	0.87173	0.95462	0.62883	0.1507	0.1507	greenyellow
QARS	0.0578	0.04438	0.03788	-0.09365	-0.06236	0.18708	0.20558	-0.2745	0.2745	0.45271	0.56438	0.62282	0.22312	0.41776	0.01428	0.00699	0.00028	0.00028	purple
ASNSD1	-0.05427	-0.03023	-0.05935	0.04965	-0.12431	0.00171	0.0052	0.28325	-0.28325	0.4808	0.69466	0.44062	0.51898	0.10525	0.98228	0.94616	0.00017	0.00017	turquoise
POLD2	0.06457	0.0522	-0.1451	-0.08965	0.15872	-0.15186	0.08248	-0.34799	0.34799	0.40146	0.49773	0.05829	0.24361	0.03813	0.04739	0.28352	3.10E-06	3.10E-06	grey
MVB12A	0.06379	0.11675	-0.04729	0.00349	0.06042	0.13931	0.20701	-0.37768	0.37768	0.40716	0.12833	0.53906	0.96384	0.43244	0.06919	0.00659	3.54E-07	3.54E-07	yellow
IGF2BP3	0.07022	-0.09773	0.12524	0.12837	-0.15197	0.22747	0.15966	-0.22107	0.22107	0.36146	0.2035	0.10265	0.09428	0.04723	0.00277	0.03699	0.00366	0.00366	grey
SPOCK3	0.04671	-0.15945	-0.05539	-0.05592	0.06046	-0.3334	-0.19414	0.28598	-0.28598	0.54403	0.03724	0.47178	0.46757	0.43216	8.35E-06	0.01095	0.00015	0.00015	brown
SMIM1	-0.00489	-0.0955	-0.03998	0.08689	-0.01432	0.04827	0.08941	-0.23017	0.23017	0.94944	0.21407	0.60363	0.25844	0.85256	0.53069	0.24483	0.00246	0.00246	grey
PSME2	0.06184	0.05779	0.07346	0.06833	-0.01345	0.01274	0.21111	-0.44436	0.44436	0.42166	0.45278	0.33965	0.37454	0.86135	0.86862	0.00558	1.14E-09	1.14E-09	grey
ADPRHL1	-0.03452	0.00943	-0.11812	-0.09007	0.0635	0.0002	0.18681	-0.28324	0.28324	0.65403	0.90256	0.12389	0.24136	0.40932	0.99792	0.01443	0.00017	0.00017	grey
SLC4A8	-0.02876	-0.10183	-0.22171	-0.18242	0.0799	-0.31706	-0.2376	0.44956	-0.44956	0.70882	0.18506	0.00356	0.01694	0.29887	2.39E-05	0.00175	6.91E-10	6.91E-10	brown
EHD1	-0.05902	-0.06749	0.11421	0.06168	-0.11629	0.20396	0.19943	-0.23028	0.23028	0.44317	0.38048	0.1369	0.42287	0.12985	0.00746	0.00892	0.00245	0.00245	yellow
LYNX1	-0.18527	0.05432	-0.10344	0.02451	-0.02713	-0.09272	-0.01616	0.07123	-0.07123	0.01526	0.48044	0.1782	0.75035	0.72467	0.22775	0.83387	0.35454	0.35454	grey
RAPGEF5	-0.03182	-0.09313	0.01979	0.1011	-0.13981	0.25822	0.03606	0.19537	-0.19537	0.67949	0.2257	0.7972	0.18828	0.06818	0.00065	0.63964	0.01044	0.01044	turquoise
GRAP	-0.12757	3.50E-05	0.02197	0.05681	-0.06922	0.02288	-0.07703	0.39431	-0.39431	0.09637	0.99964	0.77549	0.46047	0.36833	0.7664	0.31666	9.49E-08	9.49E-08	blue
SLC2A11	-0.08497	-0.07432	-0.22578	-0.10429	0.15824	-0.32893	-0.10203	0.32249	-0.32249	0.26919	0.33399	0.00299	0.17465	0.03872	1.12E-05	0.18422	1.70E-05	1.70E-05	brown
TSG101	0.07162	-0.06161	-0.00031	-0.09293	-0.04108	0.04657	0.07692	-0.25506	0.25506	0.3519	0.42341	0.99678	0.2267	0.59374	0.5453	0.31734	0.00076	0.00076	turquoise
NDUFA13	0.07869	0.05222	-0.18023	-0.14345	0.23571	-0.26391	-0.0552	-0.21612	0.21612	0.30631	0.49761	0.01833	0.06124	0.00191	0.00049	0.47333	0.00452	0.00452	green
NAGPA	0.00933	0.04357	-0.02209	-0.03394	0.11497	-0.08255	0.01336	-0.03854	0.03854	0.90356	0.5715	0.7743	0.65945	0.13428	0.28311	0.86234	0.6167	0.6167	green
PRPS1	-0.03822	-0.15098	-0.10684	-0.04291	-0.06091	-0.21111	-0.16703	0.32765	-0.32765	0.61967	0.04871	0.16428	0.57737	0.42869	0.00558	0.029	1.22E-05	1.22E-05	turquoise
TMEM219	0.10955	0.05694	-0.01467	-0.03787	0.00611	0.05045	0.15982	-0.42349	0.42349	0.15377	0.4595	0.84899	0.62287	0.93677	0.51225	0.03679	7.88E-09	7.88E-09	grey
GPR89A	0.10875	0.02795	-0.05405	-0.10923	0.0684	-0.10618	0.17514	-0.34133	0.34133	0.15681	0.71671	0.48258	0.15497	0.37403	0.16693	0.02196	4.91E-06	4.91E-06	greenyellow
FNDC3A	-0.00834	-0.13782	-0.12688	-0.03707	0.03816	-0.16647	-0.20669	0.40819	-0.40819	0.91378	0.07224	0.09819	0.6303	0.62019	0.02955	0.00668	2.99E-08	2.99E-08	turquoise
CARD16	-0.07058	-0.00801	0.07007	0.06738	-0.14432	0.23466	0.15315	0.0083	-0.0083	0.35895	0.91721	0.36247	0.38119	0.05967	0.00201	0.04552	0.91419	0.91419	blue
AKAP8L	0.09679	-0.10893	-0.11292	-0.04652	0.21857	-0.12753	-0.03649	-0.11223	0.11223	0.20789	0.15611	0.14144	0.54569	0.00408	0.09645	0.63561	0.14388	0.14388	red
KRT23	-0.02137	-0.18049	-0.02557	-0.08775	-0.0417	0.0369	-0.01604	-0.03139	0.03139	0.78142	0.01816	0.73995	0.25376	0.58813	0.63179	0.83506	0.68362	0.68362	grey
CMTM2	-0.11839	-0.08843	0.1178	0.01176	-0.00545	0.04637	0.07043	-0.05418	0.05418	0.12301	0.25006	0.1249	0.87863	0.9436	0.54703	0.35997	0.48155	0.48155	grey
IMMT	0.00133	0.0039	-0.02223	-0.04891	-0.12745	0.02794	0.12257	-0.04088	0.04088	0.9862	0.95957	0.77292	0.52529	0.09667	0.71683	0.11026	0.59553	0.59553	turquoise
MT-ND4L	-0.0459	-0.17469	-0.03253	0.0188	0.14352	-0.00641	-0.1083	-0.08305	0.08305	0.55105	0.0223	0.67277	0.80718	0.0611	0.93366	0.15854	0.28019	0.28019	grey
LRCH4	0.02238	-0.10284	-0.0107	-0.02951	0.10818	0.00595	-0.07098	-0.00516	0.00516	0.77136	0.18074	0.88956	0.70158	0.159	0.93846	0.35627	0.94661	0.94661	red
PRR11	0.0234	0.01982	0.0459	-0.00924	-0.14663	0.10032	0.34124	-0.26924	0.26924	0.76132	0.79691	0.55109	0.90448	0.05566	0.19172	4.94E-06	0.00037	0.00037	pink
NDUFA8	-0.01447	-0.03982	-0.14639	-0.05471	0.0386	-0.18072	0.02232	-0.11681	0.11681	0.85103	0.60507	0.05606	0.47728	0.61622	0.01801	0.77198	0.12812	0.12812	grey
GPNMB	-0.10753	-0.01688	0.09295	0.13334	-0.26922	0.19382	0.06152	0.38158	-0.38158	0.16154	0.82655	0.22659	0.08209	0.00037	0.01109	0.42408	2.61E-07	2.61E-07	blue
TMEM200	-0.02483	-0.15816	0.0741	0.02702	-0.17716	0.03522	-0.06046	0.36741	-0.36741	0.74719	0.03882	0.33546	0.7257	0.02045	0.64742	0.43217	7.68E-07	7.68E-07	blue
TMTC1	-0.0449	-0.12769	-0.06879	0.03076	-0.10985	-0.05186	-0.13958	0.55307	-0.55307	0.55979	0.09604	0.37132	0.68959	0.15265	0.50053	0.06864	4.36E-15	4.36E-15	turquoise
TTF2	-0.0847	-0.05408	0.09629	0.08197	-0.08926	0.16359	0.13168	-0.11341	0.11341	0.27067	0.48237	0.21026	0.28653	0.24565	0.03253	0.08601	0.13969	0.13969	turquoise
ADAT2	0.00634	0.10598	-0.0186	-0.01012	0.03759	-0.06988	0.08517	-0.13489	0.13489	0.93437	0.16772	0.8092	0.89547	0.62548	0.36378	0.26806	0.07857	0.07857	red
ECD	-0.00661	-0.06008	0.0183	-0.00241	-0.139	0.05256	0.10801	0.01489	-0.01489	0.93166	0.43508	0.8122	0.97507	0.06982	0.49474	0.15968	0.84672	0.84672	turquoise
IFIT2	-0.03414	-0.01029	0.05336	0.1284	-0.24891	0.13737	0.17996	#######	9.95E-05	0.65757	0.89379	0.4882	0.0942	0.00103	0.07319	0.01851	0.99897	0.99897	tan
HOMER1	-0.02729	-0.17575	-0.00924	0.12363	-0.1428	0.02257	0.02131	0.31962	-0.31962	0.72309	0.02149	0.90449	0.10719	0.06242	0.76955	0.78205	2.03E-05	2.03E-05	turquoise
SRSF1	-0.06387	-0.10658	-0.01572	-0.03015	-0.04599	0.05833	0.09333	-0.07127	0.07127	0.40655	0.16532	0.83825	0.69545	0.55034	0.44854	0.22471	0.35428	0.35428	turquoise
ISL1	0.06997	-0.07746	-0.22473	-0.16644	0.12716	-0.27711	-0.18784	0.30579	-0.30579	0.36314	0.31392	0.00313	0.02957	0.09746	0.00024	0.01389	4.76E-05	4.76E-05	brown
SLC44A1	0.04135	-0.02459	0.09179	0.06042	-0.12718	0.23694	0.16534	-0.16093	0.16093	0.59126	0.74958	0.23248	0.43245	0.09739	0.00181	0.03069	0.03549	0.03549	turquoise
MOV10	-0.06491	-0.11715	0.0219	0.02627	-0.00971	0.07915	0.14696	-0.21372	0.21372	0.39898	0.12703	0.77619	0.73308	0.89972	0.30344	0.05511	0.00501	0.00501	grey
RRS1	0.04201	0.17913	0.05829	0.03401	-0.11448	0.14004	0.17736	-0.21645	0.21645	0.58538	0.01906	0.44887	0.65877	0.13597	0.06772	0.0203	0.00446	0.00446	grey
CREBBP	0.05289	-0.09517	0.01816	0.0802	-0.10448	0.13637	0.01279	0.23188	-0.23188	0.49204	0.21565	0.81365	0.29705	0.17385	0.07531	0.86818	0.00228	0.00228	turquoise
GPR37	-0.01112	-0.12341	-0.09783	0.10568	-0.01524	-0.11297	-0.09763	0.06458	-0.06458	0.88519	0.10781	0.20304	0.16893	0.84314	0.14126	0.20395	0.40136	0.40136	grey
TMEM37	0.01389	0.03734	-0.10058	0.08359	0.07661	0.06247	-0.08254	0.00224	-0.00224	0.85691	0.62781	0.19058	0.27707	0.31932	0.41698	0.28312	0.97681	0.97681	grey
DDAH1	-0.05386	-0.05559	0.09651	0.10601	0.08507	0.14115	0.0069	0.02022	-0.02022	0.48415	0.4702	0.20924	0.16759	0.26863	0.06556	0.92865	0.79292	0.79292	turquoise
LDHD	-0.0105	0.15069	0.12878	0.0582	0.11928	0.24938	0.08766	-0.35131	0.35131	0.89156	0.04914	0.09321	0.44955	0.12022	0.001	0.25425	2.46E-06	2.46E-06	grey
SYPL1	0.05541	-0.05071	0.08013	0.01611	-0.04408	0.16876	0.09131	-0.2259	0.2259	0.47166	0.51012	0.29751	0.83436	0.56704	0.02735	0.23493	0.00297	0.00297	turquoise
CCNI	0.04284	-0.14176	0.02858	0.03729	-0.13897	0.0202	-0.02697	0.16249	-0.16249	0.57794	0.06439	0.7106	0.6282	0.06988	0.79316	0.72618	0.03372	0.03372	turquoise
UMAD1	0.02869	-0.07337	0.03596	0.04409	-0.05222	0.08864	-0.05545	0.19154	-0.19154	0.70953	0.34026	0.64055	0.56695	0.49761	0.24896	0.4713	0.01208	0.01208	turquoise
SLC22A18	0.11465	0.01498	0.01585	-0.02352	0.14865	0.15689	0.14419	-0.53081	0.53081	0.1354	0.84579	0.83701	0.76006	0.05234	0.04044	0.05989	8.15E-14	8.15E-14	yellow
AL121845	0.00688	-0.13428	-0.10077	-0.02078	0.22391	-0.15159	-0.06291	-0.05067	0.05067	0.92883	0.07995	0.1897	0.78735	0.00324	0.04779	0.41371	0.51042	0.51042	red
ZNF691	-0.05166	-0.12366	-0.17168	-0.08409	0.05419	-0.03172	-0.00594	0.07526	-0.07526	0.50217	0.10708	0.02476	0.2742	0.48143	0.68048	0.93854	0.32791	0.32791	grey
CHST10	-0.07535	-0.09162	-0.27241	-0.04303	-0.08972	-0.24343	-0.08362	0.54664	-0.54664	0.32734	0.23334	0.00031	0.57632	0.24321	0.00133	0.27686	1.04E-14	1.04E-14	brown
SUFU	-0.02821	-0.08266	-0.0028	0.02349	-0.18046	0.05276	0.09042	0.22917	-0.22917	0.71414	0.28247	0.97098	0.76037	0.01818	0.49309	0.23955	0.00257	0.00257	turquoise
CLASP1	-0.06117	-0.19415	-0.06206	0.05466	-0.16371	-0.01151	-0.02834	0.30578	-0.30578	0.42674	0.01094	0.42005	0.47767	0.03239	0.8812	0.71289	4.76E-05	4.76E-05	turquoise
C1QTNF6	-0.04725	-0.04599	-0.00349	0.08916	-0.11834	-0.00366	0.12509	0.1279	-0.1279	0.5394	0.55028	0.96381	0.24616	0.12317	0.96209	0.10307	0.0955	0.0955	black
IER2	0.05518	0.02458	0.0489	-0.05645	0.11487	0.13352	0.16161	-0.21789	0.21789	0.47346	0.74961	0.52533	0.46337	0.13464	0.08168	0.03471	0.0042	0.0042	grey
PHOSPHC	-0.19312	-0.05367	-0.03339	-0.01932	-0.0251	0.01787	-0.00553	0.42533	-0.42533	0.01138	0.48568	0.66463	0.802	0.74449	0.81655	0.94275	6.68E-09	6.68E-09	blue
TASP1	0.00496	-0.1136	-0.07829	-0.04196	0.00026	-0.03799	-0.02692	0.17066	-0.17066	0.9487	0.13902	0.30874	0.58582	0.99734	0.62177	0.72668	0.02564	0.02564	turquoise
PTHLH	-0.02016	-0.03408	0.0547	0.0934	-0.07794	0.06002	0.06291	-0.01388	0.01388	0.7935	0.65814	0.47733	0.22432	0.31091	0.43551	0.41372	0.85697	0.85697	black
C2CD2L	0.11713	-0.10262	-0.17824	-0.19877	0.17343	-0.22343	-0.07608	0.00609	-0.00609	0.12708	0.18167	0.01968	0.00915	0.0233	0.00331	0.32266	0.93704	0.93704	grey
POLR3C	0.00586	0.01286	-0.019	-0.01212	-0.10954	0.14356	0.20359	-0.01471	0.01471	0.93933	0.86746	0.80518	0.87494	0.15381	0.06104	0.00757	0.8486	0.8486	turquoise
C2CD4D	0.08447	0.04969	-0.03218	-0.13936	0.22748	0.01031	0.04007	-0.32861	0.32861	0.27202	0.51867	0.67605	0.06908	0.00277	0.89357	0.6028	1.14E-05	1.14E-05	grey
GPATCH8	-0.06134	-0.20399	-0.03312	-0.01095	-0.03505	0.04536	-0.04821	0.16063	-0.16063	0.42548	0.00745	0.66713	0.88696	0.64907	0.55576	0.53123	0.03583	0.03583	turquoise
IFNGR1	-0.16607	-0.07769	0.11433	0.12348	-0.1615	0.15977	0.01489	0.23828	-0.23828	0.02995	0.31247	0.13649	0.10762	0.03484	0.03685	0.84673	0.0017	0.0017	blue
ACTN1	-0.05125	-0.05182	0.07155	0.04176	-0.11854	0.12353	0.09262	0.05134	-0.05134	0.50562	0.5009	0.35236	0.58763	0.12254	0.10747	0.22825	0.50485	0.50485	turquoise
ZNF527	-0.09314	-0.11422	-0.10735	0.00621	0.00284	-0.07607	-0.10605	0.42785	-0.42785	0.22562	0.13686	0.16225	0.93577	0.97061	0.32275	0.16741	5.32E-09	5.32E-09	turquoise
ZNF354A	-0.05659	-0.19508	0.00785	0.04225	-0.07054	-0.03334	0.01011	0.18818	-0.18818	0.46223	0.01056	0.91882	0.58323	0.35926	0.66513	0.89553	0.01371	0.01371	turquoise
INPP5F	0.006	-0.20544	-0.09222	-0.00128	-0.06818	-0.15114	0.02801	0.21387	-0.21387	0.93795	0.00703	0.23026	0.9867	0.37562	0.04846	0.71611	0.00497	0.00497	turquoise
CIAO1	-0.03639	-0.07035	-0.02339	0.02835	-0.07079	0.03791	0.10242	0.06108	-0.06108	0.63657	0.36051	0.76139	0.7128	0.35752	0.62249	0.18254	0.42743	0.42743	turquoise
SLC37A1	0.01764	-0.06811	-0.08104	-0.05158	0.2925	-0.02241	-0.02462	-0.22031	0.22031	0.81887	0.37609	0.29203	0.50285	0.0001	0.77108	0.74921	0.00379	0.00379	grey
EIF2B5	0.0318	-0.05146	-0.13296	-0.0825	0.03594	-0.10214	0.12691	-0.20156	0.20156	0.67971	0.50382	0.08299	0.28338	0.64071	0.18372	0.0981	0.0082	0.0082	turquoise
DGKA	0.00512	-0.03289	0.12176	0.05088	-0.05426	0.1278	0.09759	-0.1779	0.1779	0.94699	0.66936	0.11265	0.50864	0.48094	0.09576	0.20414	0.01992	0.01992	turquoise
RUBCN	-0.00602	-0.1462	-0.04425	-0.02519	-0.1779	0.00272	-0.00428	0.20915	-0.20915	0.93768	0.05638	0.56552	0.74363	0.01992	0.97179	0.95565	0.00604	0.00604	turquoise
KMO	-0.17838	-0.06499	0.11766	0.07752	-0.24576	0.07437	-0.04293	0.36134	-0.36134	0.01958	0.3984	0.12536	0.31353	0.0012	0.33369	0.57721	1.20E-06	1.20E-06	blue
TMEM144	0.00993	0.02394	0.08753	0.06497	-0.01615	0.32211	0.18459	-0.23234	0.23234	0.89742	0.75595	0.25496	0.39852	0.83392	1.74E-05	0.01565	0.00223	0.00223	yellow
MBD2	-0.08189	-0.01978	0.00892	0.03539	-0.01966	0.09206	0.01687	0.09098	-0.09098	0.28695	0.79735	0.90779	0.64584	0.79852	0.23111	0.82665	0.23663	0.23663	turquoise
MRPL24	0.07199	0.09737	-0.08779	-0.13079	0.05451	-0.04076	0.16214	-0.35305	0.35305	0.34943	0.20515	0.25354	0.08819	0.47887	0.59655	0.03411	2.18E-06	2.18E-06	greenyellow
SEMA4C	-0.09264	-0.13296	-0.04642	0.05995	-0.09713	0.03507	0.06604	0.07381	-0.07381	0.22816	0.08297	0.54659	0.43605	0.20629	0.6488	0.39079	0.33734	0.33734	turquoise
AGFG2	0.1931	-0.07321	-0.17332	-0.0584	0.24295	-0.15401	-0.07266	-0.1436	0.1436	0.01139	0.34131	0.02339	0.44801	0.00137	0.04431	0.34496	0.06097	0.06097	grey

ANKRD40	-0.0482	-0.19041	-0.03656	0.04805	-0.16044	0.04716	-0.06152	0.24518	-0.24518	0.53128	0.01261	0.635	0.53255	0.03606	0.54024	0.42407	0.00123	0.00123	turquoise
CMTM6	-0.09411	-0.16919	0.09687	0.02398	-0.12788	0.17355	0.09597	-0.12646	0.12646	0.22081	0.02696	0.20753	0.75557	0.09556	0.0232	0.2118	0.09933	0.09933	turquoise
EIF3B	0.01005	0.04195	0.00619	0.02825	-0.03204	-0.00899	0.19072	-0.3377	0.3377	0.89618	0.58588	0.93592	0.71375	0.67737	0.9071	0.01247	6.27E-06	6.27E-06	pink
ZNF721	-0.04252	-0.14139	-0.09839	-0.01391	0.08529	-0.10153	-0.10059	0.09494	-0.09494	0.58077	0.06509	0.20044	0.85672	0.26737	0.18637	0.19051	0.21675	0.21675	turquoise
NPIPA5	0.00794	0.00382	-0.03993	0.12886	-0.04705	-0.047	-0.00657	-0.03803	0.03803	0.91788	0.96041	0.60413	0.09301	0.54114	0.54158	0.93205	0.62142	0.62142	grey
ZMYND15	-0.04358	0.0209	0.04725	-0.02051	0.11927	0.13389	0.06946	-0.02334	0.02334	0.57138	0.78618	0.53943	0.79003	0.12023	0.08082	0.36668	0.76187	0.76187	grey
RGS7	0.03418	-0.04936	-0.24535	-0.20847	0.17912	-0.30986	-0.24183	0.29573	-0.29573	0.6572	0.52145	0.00122	0.00621	0.01907	3.72E-05	0.00144	8.60E-05	8.60E-05	brown
OAF	-0.03352	0.02257	0.04334	0.09159	-0.08643	0.20094	0.10952	0.13843	-0.13843	0.66335	0.76954	0.57351	0.23349	0.261	0.00841	0.15389	0.07097	0.07097	blue
LGI4	-0.04576	-0.01231	-0.08655	0.03179	-0.06621	-0.08466	-0.05767	0.32131	-0.32131	0.55228	0.87306	0.26031	0.67977	0.38956	0.27091	0.45369	1.83E-05	1.83E-05	grey
CDX2	0.10009	-0.00022	-0.00879	0.09835	0.20463	0.13542	0.02977	-0.18248	0.18248	0.19276	0.99767	0.90912	0.20065	0.00726	0.0774	0.6991	0.0169	0.0169	grey
PDP1	-0.08376	-0.12265	0.06942	0.05984	-0.23723	0.15868	0.15982	-0.05644	0.05644	0.27609	0.11	0.36695	0.43685	0.00178	0.03818	0.0368	0.46342	0.46342	turquoise
C11orf71	-0.1508	-0.16519	-0.18042	-0.12789	0.09889	-0.19457	-0.1131	0.06787	-0.06787	0.04898	0.03084	0.01821	0.09551	0.19816	0.01077	0.14081	0.37773	0.37773	grey
DHX35	0.07333	0.0184	-0.04654	-0.01097	0.03115	-0.03304	-0.00523	0.07747	-0.07747	0.34054	0.8112	0.54553	0.88676	0.68591	0.66792	0.94584	0.31386	0.31386	turquoise
NFE2	0.09309	0.01476	0.07349	0.06045	-0.02388	0.07331	0.14816	-0.10273	0.10273	0.2259	0.84805	0.33943	0.43223	0.75657	0.34064	0.05312	0.18119	0.18119	grey
THBS4	0.00317	-0.04867	0.03131	0.09523	-0.07315	0.08283	-0.15332	0.47915	-0.47915	0.96715	0.52732	0.68432	0.21534	0.34171	0.28146	0.04528	3.36E-11	3.36E-11	blue
FH	0.02112	0.0833	-0.18142	-0.19763	0.08051	-0.09843	0.15758	-0.17995	0.17995	0.78392	0.27875	0.01756	0.00957	0.29518	0.20026	0.03955	0.01852	0.01852	greenyellow
PFKM	0.02003	-0.11819	-0.17278	-0.07564	0.00735	-0.21096	-0.11954	0.27226	-0.27226	0.79481	0.12365	0.02383	0.32545	0.92395	0.00561	0.11939	0.00032	0.00032	turquoise
POLD1	-0.00422	0.13389	-0.01009	-0.0451	0.02583	0.01904	0.25353	-0.29193	0.29193	0.95627	0.08084	0.89582	0.55803	0.7374	0.80481	0.00082	0.00011	0.00011	green
UBB	-0.01299	-0.1014	0.02023	0.04527	-0.00152	-0.0768	-0.02576	0.05795	-0.05795	0.86611	0.18693	0.79282	0.55653	0.98425	0.31811	0.73805	0.45154	0.45154	grey
SLC30A1	-0.004	-0.10473	0.06768	0.01479	-0.19481	0.14814	0.12639	0.05801	-0.05801	0.95859	0.17282	0.37911	0.84772	0.01067	0.05315	0.09952	0.45107	0.45107	turquoise
GSPT1	0.021	-0.03225	0.0845	0.05982	-0.18087	0.24409	0.10302	-0.06657	0.06657	0.78516	0.67545	0.27184	0.437	0.01792	0.00129	0.17999	0.387	0.387	turquoise
MBTPS2	-0.07719	-0.1842	-0.11249	-0.02262	-0.12938	-0.10506	0.01504	0.26068	-0.26068	0.31561	0.01588	0.14295	0.76902	0.0917	0.17143	0.84517	0.00057	0.00057	turquoise
USP8	-0.0596	-0.12333	-0.03732	-0.02609	-0.09201	0.03314	-0.02239	0.21807	-0.21807	0.43873	0.10803	0.62796	0.73482	0.23133	0.66701	0.77126	0.00417	0.00417	turquoise
PRORP	-0.02112	-0.07665	0.02457	0.08765	-0.05116	0.06254	0.05964	0.19388	-0.19388	0.78395	0.31903	0.74974	0.25428	0.50639	0.41643	0.43844	0.01106	0.01106	turquoise
BTBD9	-0.03357	-0.13629	-0.09674	-0.05948	-0.02881	-0.05946	-0.11739	0.30619	-0.30619	0.66288	0.07549	0.20815	0.43967	0.70832	0.43984	0.12623	4.65E-05	4.65E-05	turquoise
LENG1	0.06494	-0.00805	-0.17135	-0.10997	0.12266	-0.10775	-0.02732	-0.07656	0.07656	0.39871	0.91682	0.02503	0.15217	0.10999	0.1607	0.7228	0.3196	0.3196	green
CCDC32	-0.0978	-0.16971	-0.02204	-0.01541	-0.11022	0.08032	0.00618	0.07963	-0.07963	0.20316	0.02648	0.7748	0.84141	0.15127	0.29635	0.93611	0.3005	0.3005	turquoise
ZW10	-0.04462	-0.03822	0.06678	0.00091	-0.20615	0.17724	0.22149	-0.0724	0.0724	0.56228	0.61964	0.38547	0.99055	0.00683	0.02039	0.0036	0.34671	0.34671	turquoise
LXN	-0.04764	-0.07738	0.09281	0.03889	-0.1443	0.17781	0.02426	0.30158	-0.30158	0.53605	0.31444	0.2273	0.61355	0.05971	0.01998	0.7528	6.11E-05	6.11E-05	blue
COX4I2	-0.11336	0.06	-0.12978	0.01866	0.04188	-0.2095	-0.11064	0.38315	-0.38315	0.13987	0.43562	0.09069	0.80855	0.5865	0.00596	0.14967	2.31E-07	2.31E-07	blue
SST	0.02502	0.00307	-0.15928	-0.066	0.02245	-0.04338	-0.09889	0.19017	-0.19017	0.74532	0.96817	0.03744	0.39104	0.77074	0.57317	0.19816	0.01273	0.01273	brown
TCEAL4	0.01341	-0.16285	-0.15865	-0.03284	-0.07085	-0.12336	-0.07924	0.32451	-0.32451	0.86182	0.03332	0.03821	0.66983	0.35711	0.10796	0.30292	1.49E-05	1.49E-05	turquoise
PIWIL4	-0.00335	-0.03118	-0.04591	0.06795	0.07186	0.17549	0.08047	-0.16486	0.16486	0.96534	0.68555	0.55101	0.37717	0.35033	0.02168	0.29546	0.03118	0.03118	grey
SMAD5	-0.01126	-0.22854	-0.12171	-0.02808	0.02778	-0.09388	-0.13493	0.27164	-0.27164	0.8838	0.00264	0.11279	0.71539	0.71831	0.22194	0.07848	0.00033	0.00033	turquoise
PBXIP1	0.05095	-0.12824	-0.14913	-0.1094	0.00511	-0.14153	-0.1593	0.19192	-0.19192	0.50811	0.09469	0.05157	0.15435	0.94716	0.06483	0.03741	0.01191	0.01191	grey
DNAAF3	0.09644	-0.07998	-0.03711	0.09312	0.0787	0.04637	0.0741	-0.38138	0.38138	0.20956	0.29838	0.6299	0.22574	0.30623	0.54697	0.33542	2.65E-07	2.65E-07	grey
TMOD3	-0.03858	-0.11201	0.02894	0.07346	-0.15398	0.17571	0.16513	-0.16307	0.16307	0.61642	0.14469	0.70712	0.33963	0.04435	0.02152	0.0309	0.03308	0.03308	turquoise
SLC51A	-0.08045	-0.17563	-0.00757	0.00064	0.03373	0.04978	0.01191	0.0678	-0.0678	0.29557	0.02158	0.92171	0.99338	0.66142	0.51787	0.87716	0.37824	0.37824	grey
ZNF414	0.03919	-0.00622	-0.22205	-0.13834	0.2467	-0.24065	-0.12485	-0.05387	0.05387	0.61083	0.93561	0.00351	0.07117	0.00114	0.00152	0.10373	0.48406	0.48406	green
MYO1B	-0.05408	-0.18776	-0.10878	0.06121	-0.11949	-0.08064	-0.0544	0.40052	-0.40052	0.48233	0.01393	0.1567	0.42641	0.11956	0.29439	0.47973	5.70E-08	5.70E-08	turquoise
SNX16	-0.05322	-0.1632	-0.00235	0.09484	-0.06352	0.058	-0.0372	0.274	-0.274	0.48934	0.03294	0.97566	0.21723	0.40918	0.45117	0.62905	0.00029	0.00029	turquoise
FAM20B	0.00068	-0.12219	-0.06252	-0.11373	-0.08261	0.02984	0.04081	0.15213	-0.15213	0.99293	0.11138	0.41656	0.13858	0.28273	0.6984	0.59617	0.04699	0.04699	turquoise
TFDP2	-0.02694	-0.15983	-0.1689	0.01786	-0.18662	0.08621	0.12798	0.29503	-0.29503	0.72653	0.03678	0.02722	0.81667	0.01452	0.26223	0.09529	8.96E-05	8.96E-05	turquoise
TSR2	-0.01115	-0.15128	-0.17582	-0.06493	-0.0196	-0.15836	-0.06692	-0.00234	0.00234	0.88491	0.04826	0.02144	0.39882	0.79911	0.03857	0.38449	0.9758	0.9758	grey
QSOX2	-0.01452	-0.14192	-0.19091	-0.09692	-0.02058	-0.31906	-0.14968	0.31486	-0.31486	0.8505	0.06407	0.01238	0.20729	0.78932	2.11E-05	0.05071	2.74E-05	2.74E-05	turquoise
STMN3	-0.13192	-0.14481	-0.11433	-0.00066	-0.04691	-0.2846	-0.10694	0.31455	-0.31455	0.08545	0.05879	0.13649	0.99318	0.54238	0.00016	0.16387	2.79E-05	2.79E-05	brown
NCF4	-0.18014	0.02505	-0.01849	0.04675	-0.07394	0.09655	0.04519	0.31539	-0.31539	0.01839	0.74498	0.81025	0.54375	0.3365	0.20903	0.55728	2.65E-05	2.65E-05	blue
SLC25A6	-0.10176	-0.05745	-0.11702	0.02378	0.07011	-0.01585	0.01829	0.04813	-0.04813	0.1854	0.45547	0.12744	0.75756	0.36221	0.83702	0.81232	0.53185	0.53185	grey
ODF3B	-0.003	0.06408	-0.00666	0.0781	0.0821	0.00967	0.13652	-0.29005	0.29005	0.96894	0.40503	0.93111	0.30992	0.28575	0.90015	0.07499	0.00012	0.00012	red
TRPS1	-0.11254	-0.04149	0.02923	0.10946	-0.34717	0.11884	0.19375	0.2186	-0.2186	0.14277	0.58999	0.70427	0.15413	3.29E-06	0.12158	0.01111	0.00407	0.00407	blue
ZNF341	0.03624	-0.2314	0.04223	-0.00087	0.061	0.03349	0.11935	-0.32531	0.32531	0.63794	0.00233	0.58344	0.99098	0.428	0.66372	0.12	1.42E-05	1.42E-05	grey
DUSP2	-0.00671	-0.00337	0.06505	0.03772	-0.05255	0.13431	0.0775	0.18923	-0.18923	0.93062	0.96506	0.39794	0.6243	0.49483	0.07987	0.31365	0.01318	0.01318	blue
KIF17	-0.16077	-0.0423	-0.03648	0.03391	-0.04366	-0.06884	-0.03304	0.43126	-0.43126	0.03567	0.5828	0.63571	0.6597	0.57074	0.371	0.66791	3.90E-09	3.90E-09	grey
ARPP19	-0.05956	-0.17634	-0.08421	-0.0284	-0.10232	-0.06312	-0.11334	0.1719	-0.1719	0.43902	0.02104	0.27351	0.71232	0.18295	0.4121	0.13996	0.02457	0.02457	turquoise
IAPP	-0.0655	-0.05488	-0.12266	0.01482	-0.04666	-0.07636	-0.01852	0.31188	-0.31188	0.39471	0.47586	0.10999	0.84748	0.54451	0.32085	0.81005	3.29E-05	3.29E-05	brown
GGT7	0.07914	-0.16501	-0.07001	0.02399	0.16493	-0.04554	-0.0369	-0.04524	0.04524	0.30353	0.03103	0.36284	0.75547	0.03111	0.5542	0.63186	0.55688	0.55688	grey
SNX19	-0.05428	-0.14616	-0.08641	-0.02809	-0.12039	-0.02649	0.06296	0.23439	-0.23439	0.48074	0.05645	0.2611	0.71532	0.11678	0.73091	0.41328	0.00203	0.00203	turquoise
NACA	0.14931	-0.01667	-0.04455	-0.04743	-0.06463	0.06549	0.06917	-0.21454	0.21454	0.05129	0.82867	0.56284	0.53785	0.40098	0.39479	0.36866	0.00483	0.00483	purple
TM9SF2	0.04668	-0.07641	-0.03602	-0.03848	0.00361	-0.0793	0.01191	0.05656	-0.05656	0.54429	0.32054	0.63998	0.61731	0.96266	0.30253	0.87711	0.46251	0.46251	turquoise
ACTR10	0.01131	-0.10856	-0.0289	0.01676	0.02421	-0.10503	-0.0819	0.11171	-0.11171	0.88324	0.15753	0.70751	0.82779	0.75333	0.17157	0.2869	0.14576	0.14576	turquoise
NDUFB9	0.07108	0.01753	-0.09512	-0.08422	0.08052	-0.19914	0.07623	-0.34691	0.34691	0.35558	0.81996	0.21588	0.27346	0.29513	0.00902	0.32173	3.35E-06	3.35E-06	grey
POLR3B	0.03764	-0.02506	-0.07771	0.00643	0.05543	-0.11101	-0.05002	0.27793	-0.27793	0.62499	0.74488	0.31237	0.93344	0.4715	0.14834	0.5159	0.00023	0.00023	turquoise
ABRACL	-0.02478	0.05094	0.06157	7.46E-05	-0.15664	0.15987	0.1731	-0.16485	0.16485	0.74772	0.50822	0.42374	0.99923	0.03038	0.03673	0.02357	0.03119	0.03119	grey
DDTL	0.07861	-0.05776	-0.05928	-0.02252	0.21453	0.01591	-0.05685	-0.22827	0.22827	0.30675	0.453	0.44123	0.76999	0.00484	0.83635	0.46017	0.00267	0.00267	red
STK40	0.00808	-0.27766	0.08802	0.07282	0.00152	0.08456	-0.11917	0.13014	-0.13014	0.91649	0.00024	0.25228	0.34386	0.98425	0.2715	0.12054	0.08978	0.08978	grey
SURF1	0.01604	-0.07671	-0.2023	-0.08547	0.04458	-0.22196	-0.06155	-0.12462	0.12462	0.83502	0.31865	0.00797	0.26634	0.56257	0.00353	0.42386	0.10438	0.10438	grey
GLIPR2	-0.09394	0.02698	0.08494	0.12414	-0.17564	0.08554	-0.00113	0.39756	-0.39756	0.22166	0.72613	0.26932	0.10572	0.02157	0.26596	0.98827	7.28E-08	7.28E-08	blue
TCN1	0.10804	0.00846	0.13781	0.10451	0.02106	0.14729	0.15602	-0.22005	0.22005	0.15957	0.91257	0.07226	0.17371	0.78454	0.05455	0.04158	0.00383	0.00383	yellow
BTC	0.00508	-0.06536	-0.05111	-0.01091	0.17083	-0.04824	-0.10728	-0.00972	0.00972	0.94742	0.39569	0.50676	0.88741	0.02549	0.53093	0.16251	0.89957	0.89957	grey
MXD3	0.00039	0.03628	-0.04372	-0.03126	0.13731	-0.00223	0.21253	-0.43683	0.43683	0.99599	0.6376	0.57021	0.68486	0.07331	0.97686	0.00526	2.33E-09	2.33E-09	green
CCDC88C	-0.02957	0.03071	0.18633	0.11734	-0.09354	0.29237	0.15603	-0.18039	0.18039	0.701	0.69005	0.01468	0.12639	0.22367	0.0001	0.04156	0.01823	0.01823	turquoise
APPBP2	-0.08025	-0.23019	-0.13487	0.0041	-0.01513	-0.11293	-0.10622	0.42559	-0.42559	0.29679	0.00245	0.07861	0.95758	0.84429	0.14138	0.16674	6.52E-09	6.52E-09	turquoise
PCSK9	0.04445	0.18089	0.06805	-0.03315	0.13824	0.18976	0.06623	-0.29863	0.29863	0.56373	0.0179	0.37652	0.66688	0.07138	0.01292	0.38945	7.27E-05	7.27E-05	grey
FUS	0.04903	-0.03079	-0.03879	-0.09889	0.02285	-0.06652	0.04424	-0.02389	0.02389	0.52421	0.6893	0.61451	0.19816	0.76671	0.38733	0.5656	0.75644	0.75644	turquoise
CENPX	0.00872	0.01455	0.00134	0.00124	0.16957	-0.04741	0.18796	-0.45988	0.45988	0.90991	0.85023	0.98617	0.98711	0.02661	0.53804	0.01382	2.49E-10	2.49E-10	green
TTC7A	-0.00046	-0.01457	0.09382	0.07494	-0.20447	0.19747	0.25628	-0.25677	0.25677	0.99521	0.84999	0.22228	0.32996	0.00731	0.00963	0.00072	0.0007	0.0007	turquoise
MFSD10	-0.0176	-0.0487	-0.01383	-0.02673	0.05379	0.0378	0.14984	-0.485	0.485	0.81927	0.52705	0.85755	0.7286	0.48468	0.62357	0.05045	1.79E-11	1.79E-11	green
ARID1B	-0.03922	-0.11406	0.03395	0.05896	-0.14468	0.06301	0.00589	0.30534	-0.30534	0.61054	0.13741	0.65935	0.44363	0.05902	0.41292	0.9391	4.89E-05	4.89E-05	turquoise
SNRPB	0.07395	0.07664	-0.02347	-0.0438	0.03375	-0.0539	0.15764	-0.41621	0.41621	0.33643	0.31911	0.76059	0.56949	0.6612	0.48383	0.03948	1.50E-08	1.50E-08	pink
BATF	-0.00304	-0.00139	0.14039	0.077	-0.13999	0.23747	0.22908	-0.37074	0.37074	0.96857	0.98558	0.06704	0.3168	0.06783	0.00176	0.00258	5.99E-07	5.99E-07	yellow
GPR135	-0.04804	-0.20037	-0.16451	-0.03936	0.07671	-0.23073	-0.22475	0.45098	-0.45098	0.53269	0.0086	0.03154	0.60927	0.31862	0.0024	0.00312	6.02E-10	6.02E-10	brown
MUL1	-0.05031	-0.1251	-0.01613	0.07856	-0.13006	0.05343	0.06052	0.07996	-0.07996	0.51343	0.10303	0.8341	0.30711	0.08997	0.48761	0.43167	0.2985	0.2985	turquoise
MBNL3	-0.02981	0.04118	0.05799	0.02457	-0.09032	0.24284	0.0724	0.03137	-0.03137	0.69875	0.59281	0.45119	0.74976	0.24009	0.00137	0.34668	0.68376	0.68376	turquoise
IKBKG	-0.03862	-0.15577	-0.00309	0.00536	-0.08139	0.07087	0.06509	0.15532	-0.15532	0.61604	0.0419	0.968	0.94451	0.28992	0.35699	0.39768	0.0425	0.0425	turquoise
TLR3	-0.03561	-0.03263	0.05102	0.15307	-0.11748	0.26014	0.14557	-0.10122	0.10122	0.6438	0.67181	0.50651	0.04564	0.12593	0.00059	0.05748	0.18774	0.18774	turquoise
CUL3	-0.05017	-0.0934	0.02789	0.03989	-0.04642	0.11273	0.02303	0.07615	-0.07615	0.51462	0.22435	0.71729	0.60448	0.5466	0.1421	0.76499	0.32223	0.32223	turquoise
SPSB3	0.01106	-0.01832	-0.08458	-0.00586	0.2041	-0.02792	0.00326	-0.21074	0.21074	0.88585	0.81201	0.27139	0.93941	0.00741	0.71696	0.96622	0.00566	0.00566	green
ZNF677	-0.09975	-0.18846	-0.09266	0.07576	-0.12886	0.0701	0.03555	0.47392	-0.47392	0.19426	0.01357	0.22806	0.32473	0.09302	0.36223	0.64441	5.86E-11	5.86E-11	blue
PLEK2	0.02979	0.06421	0.16918	0.05559	-0.05823	0.32004	0.24178	-0.51973	0.51973	0.6989	0.40408	0.02696	0.47018	0.44932	1.98E-05	0.00144	3.23E-13	3.23E-13	yellow
ANKRD13	0.01858	-0.1437	-0.15241	-0.02166	0.12238	-0.21987	-0.13364	0.15388	-0.15388	0.80935	0.06079	0.04658	0.77859	0.11081	0.00386	0.08141	0.04449	0.04449	grey
SETD4	0.04815	-0.06625	-0.15633	-0.09441	0.27105	-0.0848	0.0489	-0.17615	0.17615	0.53168	0.38929	0.04116	0.21935	0.00034	0.27012	0.52536	0.02119	0.02119	red
PPP1CB	-0.03338	-0.10029	-0.06528	0.06787	-0.1004	0.06274	0.02921	0.04754	-0.04754	0.66473	0.19187	0.39624	0.37779	0.19137	0.41494	0.70454	0.5369	0.5369	turquoise
ANKS6	0.05863	-0.05584	0.01823	0.04732	-0.00791	0.07913	0.15716	-0.26574	0.26574	0.44622	0.4682	0.81289	0.5388	0.91821	0.30359	0.04009	0.00044	0.00044	grey
YWHAH	-0.08369	-0.02775	-0.02652	-0.014	-0.04354	0.08095	-0.01406	-0.02185	0.02185	0.27646	0.71863	0.73065	0.85575	0.57176	0.29258	0.85514	0.7767	0.7767	turquoise
SRM	-0.0592	0.10063	0.02773	0.05667	-0.13484	0.04855	0.13464	0.07975	-0.07975	0.44178	0.19035	0.71884	0.46163	0.07868	0.52834	0.07913	0.29979	0.29979	grey
CIART	0.11128	0.01273	0.00792	-0.01435	-0.02727	-0.0694	-0.02279	-0.17934	0.17934	0.14733	0.86873	0.91807	0.85224	0.72331	0.3671	0.76736	0.01892	0.01892	grey
PYROXD2	0.02546	-0.03828	0.12622	0.13329	0.0283	-0.02759	0.08698	-0.16005	0.16005	0.74099	0.61915	0.09996	0.08222	0.71334	0.72022	0.25795	0.03652	0.03652	grey
KLKB1	-0.0793	-0.11805	-0.2727	-0.04841	0.19334	-0.25057	-0.18484	0.28877	-0.28877	0.30255	0.12409	0.00031	0.5295	0.01129	0.00095	0.01551	0.00013	0.00013	brown
BRI3	0.07451	-0.07368	0.03203	#######	-0.0534	0.15669	0.25919	-0.32752	0.32752	0.33274	0.33817	0.67751	0.99943	0.48791	0.0407	0.00062	1.23E-05	1.23E-05	yellow
CIB1	0.06111	-0.03948	0.01492	-0.00782	0.06593	0.07696	0.1686	-0.56159	0.56159	0.42722	0.60819	0.84641	0.91919	0.39161	0.31709	0.0275	1.34E-15	1.34E-15	yellow
MBP	-0.05311	-0.14945	-0.12982	-0.01171	0.00085	-0.10875	-0.16781	0.33794	-0.33794	0.49023	0.05106	0.09059	0.87918	0.99123	0.1568	0.02825	6.17E-06	6.17E-06	turquoise
RAB37	-0.01357	-0.1032	-0.0854	-0.02465	0.00649	-0.01531	-0.04102	0.25076	-0.25076	0.8602	0.1792	0.26673	0.74897	0.93287	0.8425	0.59424	0.00094	0.00094	blue
EAF1	-0.00921	-0.19581	-0.06951	-0.10802	-0.071	0.04724	-0.07462	0.10982	-0.10982	0.90483	0.01027	0.36632	0.15964	0.35611	0.53947	0.33206	0.15274	0.15274	turquoise
PABPC5	-0.05649	-0.16063	-0.13899	-0.00924	-0.05837	-0.09293	-0.10106	0.60237	-0.60237	0.46299	0.03584	0.06983	0.90453	0.44824	0.22668	0.18846	2.89E-18	2.89E-18	brown
CRACR2A	0.04365	0.08499	0.02117	0.00687	-0.02334	0.11766	0.01461	-0.03637	0.03637	0.57082	0.26908	0.78344	0.9289	0.76188	0.12536	0.84962	0.63671	0.63671	grey
CYB561D1	-0.05884	-0.17127	-0.17935	-0.10292	-0.00795	-0.23836	-0.15114	0.4015	-0.4015	0.44462	0.02511	0.01891	0.18039	0.91784	0.00169	0.04846	5.25E-08	5.25E-08	turquoise
CREBZF	-0.02458	-0.15937	-0.04019	0.01018	0.05612	0.00505	0.02586	-0.01101	0.01101	0.74964	0.03734	0.60169	0.8949	0.46595	0.94775	0.7371	0.88639	0.88639	red
EEF1E1	-0.01907	0.07496	-0.06659	-0.10013	0.08403	-0.16439	0.03284	-0.04158	0.04158	0.80448	0.32983	0.38682	0.19255	0.27454	0.03167	0.66982	0.58923	0.58923	grey
ZDBF2	-0.1246	-0.20606	-0.10873	0.05963	-0.09195	-0.09107	-0.09352	0.45702	-0.45702	0.10444	0.00685	0.15687	0.4385	0.23165	0.23617	0.22373	3.31E-10	3.31E-10	turquoise
RINT1	-0.04621	-0.13268	0.00645	0.01716	0.06096	-0.06351	0.00051	0.1326	-0.1326	0.54836	0.08364	0.93329	0.82375	0.42837	0.40923	0.99471	0.08384	0.08384	turquoise
AP3M1	-0.00397	-0.12544	-0.04182	0.0105	-0.1008	-0.03478	0.01132	0.21525	-0.21525	0.95885	0.1021	0.58707	0.8916	0.18958	0.65155	0.88322	0.00469	0.00469	turquoise
FBXL18	-0.00561	-0.08212	-0.05096	-0.05941	-0.02735	-0.03334	0.11813	0.09097	-0.09097	0.94193	0.28562	0.50798	0.4402	0.72256	0.66511	0.12385	0.23669	0.23669	turquoise
TMEM238	0.07754	0.04428	0.15089	0.05671	0.1454	0.1721	0.05652	-0.27545	0.27545	0.31342	0.56528	0.04884	0.46129	0.05776	0.0244	0.46278	0.00027	0.00027	yellow
AMY2B	-0.05739	0.05159	0.0546	0.1039	-0.07333	0.02515	-0.17083	0.17139	-0.17139	0.45591	0.50275	0.47816	0.17626	0.34051	0.74399	0.02548	0.025	0.025	magenta
GDF10	-0.03585	0.01472	0.04953	0.13513	-0.11348	0.10293	-0.13908	0.44577	-0.44577	0.64159	0.84842	0.52002	0.07804	0.13945	0.18034	0.06964	9.98E-10	9.98E-10	grey
DHRS12	0.00715	0.03215	-0.1212	-0.03099	0.05409	-0.07947	-0.09041	0.04065	-0.04065	0.92601	0.67637	0.11431	0.68745	0.48225	0.30151	0.23959	0.59757	0.59757	grey
RNF139	-0.1181	-0.16659	0.01432	0.09897	-0.13158	0.00635	0.05476	0.08665	-0.08665	0.12394	0.02943	0.85258	0.19779	0.08626	0.93428	0.47682	0.2598	0.2598	turquoise
HKDC1	0.08553	0.07694	0.07147	0.1003	0.01038	0.2244	0.09653	-0.36498	0.36498	0.26602	0.31722	0.35291	0.19182	0.89285	0.00317	0.20911	9.20E-07	9.20E-07	yellow
SNTA1	-0.0508	-0.05996	-0.13982	0.08905	0.03954	-0.291	-0.13169	0.24697	-0.24697	0.50938	0.43597	0.06816	0.24677	0.60765	0.00011	0.08598	0.00113	0.00113	brown
FN1	-0.07518	-0.08782	0.08268	0.1278	-0.18175	0.13699	0.07457	0.10296	-0.10296	0.32841	0.25339	0.28231	0.09575	0.01736	0.07399	0.33235	0.18024	0.18024	black
CCDC125	0.04202	-0.08012	-0.1346	-0.06333	0.13845	-0.04839	0.05068	-0.125	0.125	0.58524	0.29758	0.07923	0.41053	0.07094	0.52964	0.51038	0.1033	0.1033	grey
ATP2C2	0.04968	0.07838	0.06694	0.00656	0.14697	0.06923	0.12871	-0.36683	0.36683	0.51878	0.30822	0.38436	0.93218	0.05508	0.36823	0.0934	8.02E-07	8.02E-07	grey
ANKFN1	0.08553	-0.01547	0.00539	0.05758	-0.00809	0.08368	0.08701	-0.25362	0.25362	0.266	0.84088	0.94417	0.45445	0.91637	0.27656	0.25782	0.00082	0.00082	grey
TM2D2	0.0019	-0.17739	0.0299	0.114	-0.04165	0.00701	0.14366	-0.03019	0.03019	0.98031	0.02028	0.69786	0.13762	0.58863	0.9275	0.06085	0.69504	0.69504	turquoise
GBP4	-0.09605	-0.00017	0.06146	0.08421	-0.2506	0.1409	0.1882	0.06898	-0.06898	0.21143	0.99819	0.42453	0.27347	0.00095	0.06604	0.0137	0.36999	0.36999	blue
CRISPLD2	-0.04191	-0.0699	0.02432	0.07488	-0.19844	0.16711	0.02268	0.41309	-0.41309	0.58627	0.36363	0.75219	0.33038	0.00927	0.02892	0.7684	1.97E-08	1.97E-08	blue
MSL3	-0.16216	-0.14293	0.00334	0.06035	-0.1412	0.04071	0.03369	0.20495	-0.20495	0.03408	0.0622	0.9654	0.43297	0.06546	0.59702	0.66176	0.00717	0.00717	blue
INTS6L	-0.09908	-0.11792	-0.02974	0.04603	0.02541	0.02682	0.00051	0.17214	-0.17214	0.19727	0.12453	0.69939	0.54995	0.74144	0.7277	0.99467	0.02436	0.02436	red
LRTOMT	0.03002	-0.18234	-0.16939	-0.11196	0.09976	-0.12014	-0.07975	-0.0617	0.0617	0.69668	0.01699	0.02677	0.14485	0.1942	0.11754	0.29977	0.42272	0.42272	grey

FAM163A	-0.02481	-0.05151	-0.13129	-0.15949	0.19237	-0.29958	-0.31409	0.35708	-0.35708	0.74737	0.50347	0.08697	0.03719	0.01171	6.88E-05	2.87E-05	1.63E-06	1.63E-06	brown
CCDC170	-0.07507	-0.07666	0.01162	-0.00039	-0.1536	0.08776	0.03302	0.32647	-0.32647	0.32912	0.31898	0.88012	0.99598	0.04488	0.25371	0.66813	1.31E-05	1.31E-05	blue
MS4A2	-0.08734	-0.11664	0.02777	0.02214	-0.14494	0.10951	-0.02675	0.46294	-0.46294	0.25599	0.12868	0.71842	0.77379	0.05857	0.15392	0.72838	1.83E-10	1.83E-10	blue
SMCO4	0.08193	-0.03912	0.05368	0.03145	0.06484	0.1054	0.13566	-0.13317	0.13317	0.28674	0.61143	0.48563	0.68302	0.39946	0.17005	0.07688	0.0825	0.0825	grey
MYH7B	-0.00795	-0.05025	0.01166	0.03586	0.22104	-0.05715	-0.00731	-0.1317	0.1317	0.91785	0.51392	0.87974	0.64146	0.00367	0.45782	0.92444	0.08596	0.08596	red
CMAS	-0.00071	-0.094	-0.05923	-0.07548	0.11567	-0.1663	-0.10752	-0.13084	0.13084	0.99268	0.22137	0.44157	0.32651	0.13193	0.02972	0.1616	0.08805	0.08805	turquoise
ARL10	-0.02291	-0.18043	-0.02605	0.07895	-0.1261	-0.0502	-0.04588	0.43637	-0.43637	0.76616	0.0182	0.7352	0.30467	0.10029	0.51435	0.55129	2.43E-09	2.43E-09	blue
RHOJ	-0.1298	-0.10721	-0.06518	0.07971	-0.16258	0.04189	-0.10625	0.51109	-0.51109	0.09064	0.1628	0.39699	0.30002	0.03362	0.58646	0.16662	9.16E-13	9.16E-13	blue
ZNF589	0.01189	-0.08595	-0.07665	-0.11846	-0.04266	0.02915	-0.00834	0.21916	-0.21916	0.8773	0.26368	0.31904	0.12279	0.57953	0.70507	0.9138	0.00398	0.00398	red
RPS20	0.08668	0.13679	-0.06191	-0.04468	-0.04431	-0.08693	0.09243	-0.22	0.22	0.25961	0.07441	0.42117	0.5617	0.56498	0.25824	0.2292	0.00384	0.00384	purple
ERLIN1	0.09647	-0.05531	0.09474	0.03361	-0.11973	0.14531	0.2494	-0.20481	0.20481	0.20943	0.47246	0.21775	0.66255	0.11882	0.05791	0.001	0.00721	0.00721	turquoise
ZNF610	-0.00338	-0.10562	-0.08663	0.04022	-0.01698	0.03374	-0.03126	0.30492	-0.30492	0.96499	0.16917	0.25988	0.60148	0.82551	0.66134	0.68485	5.01E-05	5.01E-05	grey
HCN3	0.08582	-0.17358	-0.13871	-0.15199	0.26759	-0.24956	-0.11684	-0.07069	0.07069	0.26438	0.02318	0.0704	0.04721	0.0004	0.001	0.12804	0.35824	0.35824	red
CACTIN	0.02008	-0.03516	-0.13735	-0.0809	0.17751	-0.07731	0.00539	-0.11715	0.11715	0.79429	0.64798	0.07323	0.29288	0.02019	0.31485	0.9442	0.12701	0.12701	green
UGT2B7	0.05544	0.04758	0.07488	0.05909	0.16665	0.26754	0.00737	-0.14208	0.14208	0.47143	0.53663	0.3304	0.44267	0.02937	0.0004	0.9238	0.06378	0.06378	grey
ANXA10	-0.07596	0.09227	0.19737	0.04339	0.03613	0.30837	0.07254	-0.3049	0.3049	0.3234	0.23004	0.00967	0.5731	0.63897	4.07E-05	0.34577	5.02E-05	5.02E-05	yellow
SPRED2	-0.02476	-0.19673	0.05183	0.06355	-0.11151	0.20086	0.03362	-0.00248	0.00248	0.74789	0.00991	0.50079	0.40896	0.14649	0.00843	0.66245	0.97427	0.97427	turquoise
ASB16	-0.02001	-0.14093	-0.17979	-0.14435	0.19365	-0.33413	-0.23848	0.31515	-0.31515	0.79504	0.06597	0.01862	0.0596	0.01116	7.96E-06	0.00168	2.69E-05	2.69E-05	brown
TIMM44	0.04861	0.08183	-0.19183	-0.0331	0.26235	-0.14699	-0.04457	-0.12471	0.12471	0.52783	0.28732	0.01195	0.66733	0.00053	0.05505	0.56267	0.10413	0.10413	green
SBNO1	0.00107	-0.16835	-0.02612	-0.01671	-0.06239	-0.01998	-0.00526	0.25197	-0.25197	0.98888	0.02773	0.73454	0.82829	0.41754	0.79533	0.94554	0.00089	0.00089	turquoise
SLC16A9	-0.01141	-0.12268	-0.10766	-0.0033	0.12389	-0.11415	0.01837	0.21721	-0.21721	0.88222	0.10992	0.16105	0.96581	0.10643	0.13713	0.8115	0.00432	0.00432	brown
CHMP4A	0.0532	-0.06396	-0.00415	0.00408	0.09154	-0.04313	-0.01743	-0.17143	0.17143	0.48952	0.4059	0.95704	0.95773	0.23373	0.57539	0.821	0.02497	0.02497	red
WDR55	0.04848	-0.15897	0.02597	-0.02864	0.02031	0.11637	0.11505	-0.23278	0.23278	0.52894	0.03782	0.73596	0.70996	0.79199	0.12958	0.13402	0.00218	0.00218	turquoise
CTSW	-0.10476	0.12056	0.04281	0.07017	-0.15582	0.06464	0.11206	0.2343	-0.2343	0.1727	0.11624	0.5782	0.36179	0.04184	0.40095	0.14452	0.00204	0.00204	blue
ZNF70	-0.09297	-0.12763	-0.12058	0.05139	-0.00485	-0.07537	0.03325	0.33877	-0.33877	0.22648	0.09621	0.1162	0.50441	0.94976	0.3272	0.6659	5.84E-06	5.84E-06	turquoise
WNT2	-0.10612	-0.03479	0.18154	0.11378	-0.16238	0.26285	0.04793	0.09706	-0.09706	0.16714	0.65145	0.01749	0.13841	0.03385	0.00051	0.5336	0.20662	0.20662	black
FANCF	-0.0323	-0.04997	-0.11723	-0.03478	0.06934	-0.02537	-0.04901	-0.14113	0.14113	0.67492	0.51628	0.12677	0.65158	0.36749	0.74185	0.52443	0.0656	0.0656	turquoise
INCA1	-0.11181	-0.19599	-0.11896	-0.05541	0.08978	-0.1422	-0.06657	0.27882	-0.27882	0.1454	0.0102	0.12122	0.47165	0.2429	0.06356	0.38698	0.00022	0.00022	turquoise
AXIN1	0.08111	-0.01931	0.03784	0.01823	0.07393	0.12364	0.10648	-0.3476	0.3476	0.29161	0.80207	0.62314	0.81294	0.33656	0.10716	0.16571	3.19E-06	3.19E-06	green
DIABLO	0.01448	-0.20091	-0.04297	-0.02407	0.06843	-0.11077	-0.06016	0.03304	-0.03304	0.85087	0.00842	0.57681	0.75465	0.37385	0.14919	0.43445	0.66792	0.66792	turquoise
GBP1	-0.11769	0.01305	0.05187	0.10904	-0.25611	0.14858	0.19753	0.13063	-0.13063	0.12527	0.86546	0.50044	0.15573	0.00072	0.05244	0.00961	0.08857	0.08857	blue
BOLA2-SM	0.00156	-0.03696	0.01414	0.02005	-0.08905	-0.02407	0.05515	-0.11117	0.11117	0.98381	0.63131	0.85439	0.79462	0.24676	0.75465	0.47376	0.14775	0.14775	turquoise
TOP3A	-0.09277	-0.13381	-0.08903	-0.02719	-0.05579	-0.14339	-0.0426	0.28684	-0.28684	0.22748	0.08102	0.2469	0.72407	0.46862	0.06135	0.58008	0.00014	0.00014	turquoise
ARHGAP4	-0.04797	0.03766	-0.01254	0.02544	-0.11081	0.03971	0.11731	0.09215	-0.09215	0.53327	0.62485	0.8707	0.74122	0.14906	0.60612	0.1265	0.23062	0.23062	blue
CCDC59	-0.01986	-0.03663	-0.00075	-0.04951	0.03206	0.04049	-0.04234	-0.06087	0.06087	0.79653	0.63434	0.9922	0.52018	0.67725	0.59901	0.58242	0.42905	0.42905	turquoise
TNNI3	-0.07187	-0.12746	0.01374	0.1409	0.07491	0.00034	0.03883	-0.31656	0.31656	0.35025	0.09666	0.85843	0.06603	0.33016	0.99651	0.6141	2.46E-05	2.46E-05	grey
PA2G4	0.0734	-0.02297	-0.0588	-0.04371	-0.01589	-0.06654	0.09753	-0.30404	0.30404	0.34007	0.76554	0.44492	0.57027	0.83658	0.38717	0.20445	5.28E-05	5.28E-05	turquoise
SND1	0.04988	0.03237	-0.02781	0.02496	-0.03338	-0.20313	-0.04273	0.04729	-0.04729	0.51704	0.67431	0.71807	0.74587	0.66468	0.00771	0.57897	0.53909	0.53909	turquoise
AC009779	0.02804	-0.13433	-0.15031	-0.06955	0.27581	-0.14208	-0.17625	-0.04036	0.04036	0.71584	0.07983	0.04973	0.36607	0.00026	0.06377	0.02112	0.60022	0.60022	red
SSR4	-0.01111	0.00047	-0.18156	-0.14598	0.17559	-0.34475	-0.14898	0.07647	-0.07647	0.88532	0.99511	0.01747	0.05676	0.02161	3.88E-06	0.05181	0.3202	0.3202	turquoise
GNG12	-0.06883	-0.1154	0.0654	0.07561	-0.1517	0.254	0.06219	0.11843	-0.11843	0.37106	0.13283	0.39541	0.32569	0.04764	0.0008	0.41908	0.1229	0.1229	turquoise
USP48	-0.04141	-0.14514	0.05309	0.03657	-0.1349	0.13783	0.02869	0.07911	-0.07911	0.59073	0.05821	0.49045	0.63486	0.07855	0.07222	0.70953	0.30369	0.30369	turquoise
ZNF418	-0.0526	-0.1751	-0.08277	0.03601	-0.06968	-0.02947	-0.02737	0.43067	-0.43067	0.49445	0.02199	0.28178	0.64009	0.36514	0.70198	0.72229	4.11E-09	4.11E-09	blue
PIAS4	0.01698	-0.07528	-0.28598	-0.15293	0.2217	-0.20794	-0.07034	0.00166	-0.00166	0.82558	0.32782	0.00015	0.04583	0.00357	0.00635	0.36058	0.98284	0.98284	green
PARS2	-0.06212	-0.0952	-0.04739	0.01062	-0.09583	0.00478	0.10436	0.07594	-0.07594	0.41962	0.2155	0.53818	0.89036	0.21244	0.95048	0.17435	0.32356	0.32356	turquoise
PTK7	-0.02306	-0.06998	0.00483	0.06637	-0.15699	-0.00076	0.11403	0.09965	-0.09965	0.76464	0.36306	0.95	0.38839	0.04031	0.99217	0.13752	0.19472	0.19472	black
PRR14	0.07388	-0.01229	-0.0873	-0.01407	0.07454	-0.01765	0.04709	-0.22452	0.22452	0.33688	0.87325	0.2562	0.85511	0.33257	0.81878	0.54081	0.00316	0.00316	green
FRYL	-0.03732	-0.1545	0.00876	0.04446	#######	0.0562	0.02692	0.0632	-0.0632	0.62796	0.04363	0.90945	0.56367	0.99909	0.46531	0.72675	0.41153	0.41153	turquoise
SCO1	-0.10039	-0.12903	-0.10237	-0.06712	-0.02183	-0.09451	-0.0038	0.32628	-0.32628	0.19141	0.09257	0.18273	0.3831	0.7769	0.21883	0.96071	1.33E-05	1.33E-05	turquoise
PCDHGC3	-0.07318	-0.22383	0.01895	0.09162	-0.18587	0.07923	0.0911	0.33417	-0.33417	0.3415	0.00325	0.80569	0.23335	0.01493	0.30298	0.23601	7.94E-06	7.94E-06	blue
VAMP2	-0.01608	-0.15715	-0.22152	-0.20145	0.00849	-0.25823	-0.20688	0.45017	-0.45017	0.83467	0.0401	0.00359	0.00824	0.91228	0.00065	0.00663	6.51E-10	6.51E-10	brown
GREB1L	0.05663	-0.17164	0.08229	0.10839	-0.10805	0.06626	0.11903	-0.29973	0.29973	0.46189	0.02479	0.28459	0.15822	0.15954	0.38924	0.12098	6.82E-05	6.82E-05	grey
FZD8	-0.0202	-0.17926	-0.08101	0.02947	-0.15185	0.08516	-0.10592	0.232	-0.232	0.79311	0.01898	0.29219	0.70196	0.04741	0.26807	0.16794	0.00226	0.00226	blue
OSBPL1A	-0.03806	-0.13879	0.06237	0.14259	-0.2362	0.15304	0.02733	0.18382	-0.18382	0.62119	0.07023	0.41774	0.06283	0.00187	0.04568	0.72268	0.0161	0.0161	turquoise
MAN1B1	-0.01912	-0.10668	-0.09732	-0.01744	-0.07037	-0.10606	0.08198	-0.02694	0.02694	0.80392	0.16491	0.20543	0.82091	0.36042	0.1674	0.28642	0.72655	0.72655	grey
APOBEC3I	-0.06931	-0.09241	-0.12881	0.01153	-0.04945	-0.0957	-0.03813	0.17024	-0.17024	0.36768	0.2293	0.09314	0.88102	0.52068	0.21311	0.62053	0.02601	0.02601	blue
NXNL2	0.0083	-0.12213	-0.04905	0.05235	-0.12102	0.04464	0.15216	-0.35927	0.35927	0.91418	0.11154	0.52409	0.49647	0.11485	0.56211	0.04695	1.40E-06	1.40E-06	grey
GALNT7	0.02103	-0.02299	0.12269	0.09801	-0.00177	0.26544	0.1261	-0.24887	0.24887	0.78485	0.76536	0.10988	0.20219	0.98167	0.00045	0.1003	0.00103	0.00103	turquoise
EDN1	-0.21158	-0.12147	0.0165	0.10896	-0.13478	0.17736	0.02257	0.00551	-0.00551	0.00547	0.11351	0.83037	0.15603	0.07882	0.0203	0.76947	0.94301	0.94301	black
DCAF11	0.07075	-0.09664	-0.08855	-0.04087	-0.01111	-0.05471	-0.03121	0.09721	-0.09721	0.35784	0.2086	0.24944	0.59557	0.88533	0.47729	0.68529	0.20593	0.20593	turquoise
CHAC1	0.04199	0.12898	-0.01775	-0.04678	0.06977	0.00588	0.02002	-0.13257	0.13257	0.58552	0.0927	0.81777	0.54344	0.36453	0.93915	0.79491	0.0839	0.0839	grey
ADIPOR1	0.06145	-0.0522	0.02608	-0.07209	-0.10944	0.06506	0.12598	-0.22706	0.22706	0.4246	0.49776	0.73491	0.34874	0.15417	0.39785	0.10061	0.00282	0.00282	turquoise
PLK4	-0.1064	0.02414	0.07883	0.03437	-0.06412	0.05775	0.26125	-0.34781	0.34781	0.16602	0.75396	0.30544	0.6554	0.40474	0.45312	0.00056	3.14E-06	3.14E-06	pink
PXN	0.12362	-0.11783	0.02072	0.02382	0.04532	-0.01272	0.06021	-0.24174	0.24174	0.10721	0.1248	0.78796	0.75713	0.55618	0.8688	0.43408	0.00145	0.00145	turquoise
TUBA1A	-0.11222	-0.09946	-0.12957	-0.02182	-0.07424	-0.13773	-0.17426	0.43238	-0.43238	0.14393	0.19558	0.0912	0.77694	0.33455	0.07243	0.02264	3.51E-09	3.51E-09	blue
C7orf50	0.02962	-0.04855	-0.14318	-0.06249	0.16876	-0.17805	-0.04819	-0.17302	0.17302	0.70053	0.52831	0.06172	0.41682	0.02735	0.01981	0.53134	0.02363	0.02363	green
SCAF4	0.00291	-0.12749	-0.03757	-0.02618	0.08517	0.02915	-0.02778	0.09571	-0.09571	0.96983	0.09656	0.62563	0.73394	0.26804	0.70513	0.71835	0.21302	0.21302	turquoise
PPP2CA	-0.03989	-0.17286	-0.0466	-0.01416	-0.0237	-0.00832	-0.05211	0.0027	-0.0027	0.60446	0.02377	0.54505	0.8542	0.75835	0.91396	0.49851	0.97203	0.97203	turquoise
PDSS2	-0.07538	-0.00051	-0.07775	-0.00459	-0.04562	-0.07191	0.02121	0.23277	-0.23277	0.32712	0.99469	0.31215	0.95253	0.55354	0.34998	0.78308	0.00219	0.00219	turquoise
CKAP4	0.07516	-0.04556	0.01881	0.01423	-0.02344	0.01987	0.11916	0.08432	-0.08432	0.32857	0.55403	0.80708	0.85342	0.76087	0.79645	0.12057	0.27287	0.27287	black
BHLHA15	-0.07196	0.09073	0.04862	0.11799	-0.03557	-0.02867	-0.10454	0.2038	-0.2038	0.34964	0.23792	0.52768	0.12431	0.64417	0.70974	0.17361	0.0075	0.0075	magenta
GSE1	-0.02309	-0.17469	-0.15643	-0.05132	0.14731	-0.14378	-0.19865	0.14206	-0.14206	0.76435	0.0223	0.04104	0.505	0.05451	0.06064	0.0092	0.06382	0.06382	turquoise
TMUB1	0.06225	0.00099	-0.04251	-0.04097	0.17267	-0.10583	0.07522	-0.47601	0.47601	0.41861	0.98976	0.58092	0.59471	0.02392	0.16832	0.32819	4.70E-11	4.70E-11	green
DYNLL1	0.10012	-0.15081	-0.0682	-0.04544	0.08409	-0.06321	0.00998	-0.18563	0.18563	0.19259	0.04896	0.37542	0.55511	0.27416	0.41148	0.89692	0.01507	0.01507	grey
UVRAG	-0.06633	-0.09373	0.0111	0.01581	-0.13367	0.11616	0.00188	0.17997	-0.17997	0.38868	0.22271	0.88542	0.83737	0.08134	0.13029	0.9805	0.0185	0.0185	turquoise
IGF2	-0.12184	-0.12765	0.0741	0.08657	-0.02285	0.11482	0.00876	0.17053	-0.17053	0.11241	0.09615	0.33544	0.26024	0.76677	0.1348	0.9095	0.02575	0.02575	black
SLC5A1	0.01604	-0.04662	0.0279	0.09721	0.09848	0.16982	0.0469	-0.06056	0.06056	0.83503	0.54481	0.71718	0.20594	0.20001	0.02638	0.54241	0.43137	0.43137	grey
THTPA	-0.063	-0.18261	-0.12505	-0.02754	0.07123	-0.11339	-0.14042	0.36283	-0.36283	0.41302	0.01682	0.10318	0.72072	0.35455	0.13976	0.06697	1.08E-06	1.08E-06	turquoise
HEATR3	-0.11394	-0.04742	-0.0608	-0.03658	-0.00883	-0.12595	-0.01038	0.22308	-0.22308	0.13783	0.53795	0.42956	0.6348	0.90874	0.10069	0.89277	0.00336	0.00336	turquoise
TRIM27	0.11016	-0.02075	-0.06999	-0.05428	0.11428	-0.04649	0.06088	-0.10802	0.10802	0.15146	0.78766	0.36301	0.4807	0.13665	0.54594	0.42891	0.15963	0.15963	grey
TOM1L2	-0.04653	-0.13462	-0.19075	-0.0676	0.11667	-0.30911	-0.16338	0.34053	-0.34053	0.54564	0.07918	0.01245	0.37964	0.1286	3.89E-05	0.03274	5.18E-06	5.18E-06	brown
GNRH1	0.03605	-0.06812	0.01519	0.0419	0.12166	0.01774	0.0772	-0.29218	0.29218	0.63972	0.37602	0.84372	0.58632	0.11293	0.81787	0.31556	0.00011	0.00011	red
CSRP1	-0.03714	0.00784	-0.02693	-0.00762	-0.05761	0.06401	-0.01116	0.10285	-0.10285	0.62964	0.91889	0.72658	0.92118	0.45416	0.40552	0.88479	0.18068	0.18068	salmon
ARHGAP2	-0.12399	-0.04189	0.0562	0.05804	-0.19974	0.09986	-0.03791	0.43707	-0.43707	0.10615	0.58648	0.46532	0.45083	0.00881	0.19379	0.62248	2.28E-09	2.28E-09	blue
ZNF765	0.03781	-0.12782	0.01342	0.01581	-0.09545	0.15095	0.04513	0.06575	-0.06575	0.62348	0.09571	0.8617	0.83742	0.2143	0.04876	0.55779	0.39291	0.39291	turquoise
ETV5	-0.10406	-0.08594	-0.04873	0.04877	-0.30983	0.12471	0.25156	0.09544	-0.09544	0.1756	0.26373	0.52677	0.52646	3.73E-05	0.10412	0.0009	0.21432	0.21432	grey
PNISR	-0.04073	-0.12237	-0.02938	0.0041	0.07406	-0.02423	-0.06024	0.16583	-0.16583	0.59684	0.11083	0.7029	0.9576	0.33568	0.75306	0.43383	0.03019	0.03019	red
NAA15	-0.10886	-0.08365	0.03825	-0.00571	-0.11851	0.14108	0.10522	0.02734	-0.02734	0.15639	0.27669	0.61939	0.94092	0.12264	0.06569	0.17078	0.72267	0.72267	turquoise
EGFR	0.00145	-0.13584	0.07552	0.13362	-0.24977	0.25511	0.24297	-0.11941	0.11941	0.98495	0.07647	0.32627	0.08145	0.00099	0.00076	0.00136	0.11981	0.11981	turquoise
SETD1B	0.04474	-0.13922	-0.06862	-0.00561	0.0576	-0.06703	-0.08672	0.17749	-0.17749	0.56118	0.06936	0.37251	0.94191	0.45427	0.38373	0.25939	0.02021	0.02021	turquoise
GRAMD2B	0.01033	-0.18716	-0.05637	-0.08623	-0.03962	-0.04531	-0.02169	-0.04787	0.04787	0.89338	0.01424	0.46397	0.26214	0.60693	0.55621	0.77827	0.53408	0.53408	turquoise
PCDHGA9	0.05418	-0.09117	0.03931	-0.04214	-0.19675	0.1016	0.06023	0.33303	-0.33303	0.48154	0.23566	0.60975	0.58424	0.0099	0.18609	0.43387	8.56E-06	8.56E-06	blue
TMEM71	-0.06775	-0.15038	0.03907	0.04757	-0.19247	0.08216	-0.00138	0.24177	-0.24177	0.37859	0.04962	0.61187	0.53666	0.01167	0.28541	0.98571	0.00144	0.00144	blue
RGS12	-0.05645	-0.10932	-0.06256	-0.00504	0.04364	-0.06934	0.02851	-0.1398	0.1398	0.46333	0.15464	0.4163	0.94782	0.57084	0.3675	0.71123	0.06821	0.06821	grey
TRIL	-0.06487	-0.11839	0.01277	0.03692	-0.09393	0.02463	0.03073	0.44047	-0.44047	0.39924	0.12303	0.86837	0.63163	0.22173	0.74917	0.68987	1.65E-09	1.65E-09	blue
ADSSL1	0.05329	-0.13266	-0.08961	-0.03347	0.17707	-0.17599	-0.15919	-0.10643	0.10643	0.48881	0.0837	0.24381	0.66387	0.02052	0.0213	0.03755	0.16592	0.16592	grey
BAMBI	0.04847	-0.14878	-0.15926	-0.00144	0.14606	-0.17198	-0.12757	0.06018	-0.06018	0.52896	0.05213	0.03747	0.98512	0.05662	0.0245	0.09635	0.43424	0.43424	grey
TXK	-0.12043	-0.09163	0.02692	0.07728	-0.1316	0.07225	-0.05413	0.33272	-0.33272	0.11664	0.23328	0.72675	0.31504	0.08621	0.34766	0.48197	8.74E-06	8.74E-06	blue
DUSP5	-0.07402	-0.18024	-0.00353	0.0211	-0.16871	0.04048	0.01454	0.04938	-0.04938	0.33598	0.01832	0.96348	0.78419	0.0274	0.59909	0.85024	0.52126	0.52126	black
ZNF511	0.06257	0.00458	-0.04707	-0.01373	0.20357	-0.14575	0.06884	-0.4595	0.4595	0.41623	0.95261	0.54098	0.85855	0.00757	0.05716	0.37096	2.59E-10	2.59E-10	green
UBN2	0.01663	-0.2235	-0.11926	-0.00342	0.04948	-0.12936	-0.0702	0.15602	-0.15602	0.82906	0.0033	0.12028	0.96463	0.52043	0.09174	0.36156	0.04157	0.04157	turquoise
IL23A	-0.03311	-0.00488	0.10713	0.06424	-0.0387	0.14203	-0.00387	-0.17973	0.17973	0.6673	0.9495	0.16313	0.40383	0.61531	0.06387	0.9599	0.01866	0.01866	grey
HMBS	0.03948	0.19304	-0.02451	-0.10304	0.0291	-0.04655	0.26603	-0.42489	0.42489	0.60816	0.01142	0.75032	0.17987	0.70552	0.54545	0.00044	6.94E-09	6.94E-09	pink
LIPM	-0.07259	0.0538	0.16312	0.0492	0.04698	0.18606	0.15615	-0.40833	0.40833	0.34543	0.48467	0.03303	0.52281	0.54178	0.01483	0.0414	2.96E-08	2.96E-08	yellow
G6PC3	0.00855	-0.097	-0.20203	-0.12388	0.28191	-0.3564	-0.18956	0.04221	-0.04221	0.91158	0.20691	0.00805	0.10645	0.00019	1.71E-06	0.01302	0.5836	0.5836	grey
TBRG1	-0.01814	-0.08125	0.00321	-0.05163	0.00063	0.10113	0.08411	-0.12541	0.12541	0.81384	0.29076	0.96679	0.50243	0.99347	0.18814	0.27409	0.10218	0.10218	red
ADPRM	0.01635	-0.07515	-0.04833	-0.05455	-0.06948	0.01568	-0.03016	0.2377	-0.2377	0.83194	0.32863	0.53016	0.47859	0.36654	0.83871	0.6954	0.00175	0.00175	blue
RTL6	-0.1256	-0.09559	-0.12829	0.05297	-0.06769	-0.08055	-0.04971	0.36337	-0.36337	0.10167	0.21362	0.09448	0.4914	0.37905	0.29497	0.51852	1.04E-06	1.04E-06	turquoise
NME5	0.06392	-0.16356	-0.228	-0.08537	0.2268	-0.28026	-0.2794	0.29459	-0.29459	0.4062	0.03256	0.00271	0.26689	0.00285	0.00021	0.00021	9.18E-05	9.18E-05	brown
CLPSL1	-0.10251	0.06869	0.07525	0.08467	0.01172	0.09568	-0.05855	0.09992	-0.09992	0.18213	0.37204	0.32796	0.2709	0.87908	0.21316	0.44688	0.1935	0.1935	magenta
HM13	0.13871	0.0379	-0.06616	0.01259	0.06554	-0.16098	0.05143	-0.2491	0.2491	0.07041	0.6226	0.38991	0.87023	0.3944	0.03543	0.50412	0.00102	0.00102	grey
C15orf48	-0.00236	-0.05094	0.05829	-0.01839	-0.00195	0.02839	0.05137	-0.27201	0.27201	0.97558	0.50817	0.44888	0.81132	0.9798	0.7124	0.50457	0.00032	0.00032	grey
NAA25	-0.02809	-0.09864	0.04158	0.03231	-0.00412	4.30E-05	0.00803	0.07106	-0.07106	0.7153	0.19928	0.5892	0.67486	0.95732	0.99955	0.91702	0.35569	0.35569	turquoise
NEIL3	-0.08439	0.14382	0.07204	0.01065	-0.02972	0.05237	0.26848	-0.36587	0.36587	0.27249	0.06057	0.34908	0.89003	0.69957	0.49631	0.00038	8.61E-07	8.61E-07	pink
FAAH2	0.02907	-0.03757	-0.09981	-0.02379	0.06336	-0.10984	-0.10171	-0.26242	0.26242	0.70585	0.62561	0.19397	0.75741	0.41033	0.15268	0.18561	0.00053	0.00053	grey
FES	-0.10973	-0.09383	-0.01833	0.10173	-0.05531	0.11642	0.03537	0.23331	-0.23331	0.15311	0.22222	0.81187	0.18552	0.47247	0.12941	0.64606	0.00213	0.00213	blue
PPP1R16B	-0.11903	-0.08417	-0.02392	0.01264	-0.12258	-0.01963	-0.09375	0.52407	-0.52407	0.12099	0.2737	0.75614	0.86972	0.11022	0.79881	0.2226	1.90E-13	1.90E-13	blue
FBXO9	-0.04482	-0.11007	-0.1388	-0.02779	0.12365	-0.19461	-0.1582	0.35091	-0.35091	0.56049	0.15182	0.07022	0.71822	0.10712	0.01075	0.03878	2.53E-06	2.53E-06	turquoise
ICK	0.05603	-0.02462	0.11252	0.06669	-0.09622	0.23438	0.08905	0.08637	-0.08637	0.46671	0.74922	0.14284	0.38612	0.2106	0.00203	0.24674	0.26132	0.26132	turquoise
RANBP1	-0.07245	0.06127	-0.07911	-0.09289	-0.0139	-0.07259	0.18615	-0.2768	0.2768	0.34637	0.42602	0.30368	0.22692	0.85682	0.34539	0.01478	0.00025	0.00025	grey
EXTL3	-0.11251	-0.12916	-0.14348	-0.02884	-0.12287	-0.18551	-0.01993	0.35461	-0.35461	0.14287	0.09226	0.06118	0.70809	0.10938	0.01513	0.79581	1.95E-06	1.95E-06	turquoise
CMBL	0.03039	-0.02295	0.09753	0.02437	0.19573	0.09302	0.05997	-0.25869	0.25869	0.69312	0.76577	0.20444	0.75173	0.0103	0.22625	0.43591	0.00063	0.00063	grey
PIK3CG	-0.13228	-0.06611	0.05562	0.02779	-0.22046	0.20685	0.05592	0.44455	-0.44455	0.08459	0.39031	0.46994	0.71828	0.00376	0.00664	0.46752	1.12E-09	1.12E-09	blue
SYTL2	0.01525	-0.04841	0.14774	0.00847	-0.01867	0.29499	0.17435	-0.13531	0.13531	0.84304	0.52947	0.05381	0.91244	0.80854	8.97E-05	0.02257	0.07764	0.07764	turquoise
OAT	-0.09477	-0.09917	0.02031	-0.04043	0.02111	-0.02645	-0.00732	0.11661	-0.11661	0.21757	0.19687	0.79202	0.59956	0.78406	0.73131	0.92427	0.1288	0.1288	turquoise
SLC23A3	0.05469	-0.04663	0.14064	0.07237	0.11465	0.12162	0.1369	-0.40379	0.40379	0.47746	0.54481	0.06655	0.34686	0.13539	0.11305	0.07418	4.34E-08	4.34E-08	yellow
MOGAT1	0.0271	0.02218	-0.07857	0.02901	0.15174	0.08172	0.07873	-0.0394	0.0394	0.72496	0.77336	0.30702	0.70648	0.04757	0.288	0.30605	0.60893	0.60893	grey
CLK4	-0.0366	-0.24658	-0.06238	-0.03238	0.09636	-0.02459	-0.05653	0.24838	-0.24838	0.63465	0.00115	0.41761	0.67415	0.20992	0.74955	0.46272	0.00105	0.00105	red
ITGBL1	-0.03227	-0.05963	0.05945	0.07182	-0.22828	0.23469	0.07921	0.32476	-0.32476	0.67523	0.43849	0.43985	0.35057	0.00267	0.002	0.30307	1.47E-05	1.47E-05	black

TFF3	0.05184	0.03879	0.034	-0.02632	0.26684	0.04446	-0.06879	-0.27215	0.27215	0.50068	0.6145	0.65884	0.73259	0.00042	0.56371	0.37134	0.00032	0.00032	grey
ZNF343	-0.01846	-0.08286	-0.10104	-0.02247	0.03193	-0.03077	0.03149	0.10669	-0.10669	0.81057	0.28131	0.18852	0.77055	0.67842	0.6895	0.6826	0.16487	0.16487	turquoise
COA4	-0.01987	-0.00554	-0.07179	-0.052	-0.00708	0.02659	0.19585	-0.41244	0.41244	0.79643	0.94263	0.35076	0.49937	0.92674	0.72988	0.01025	2.08E-08	2.08E-08	greenyellow
RAP1A	-0.08536	-0.09544	0.08274	0.08417	-0.11418	0.13952	0.00289	0.21474	-0.21474	0.26697	0.21433	0.28201	0.27374	0.137	0.06875	0.97012	0.00479	0.00479	blue
JKAMP	-0.01533	-0.14729	-0.0407	-0.00591	0.02685	-0.11317	-0.02371	0.06975	-0.06975	0.84227	0.05455	0.59715	0.93885	0.72743	0.14053	0.75817	0.36467	0.36467	turquoise
LMO4	-0.07327	0.00753	0.1148	0.11865	0.02012	0.24861	0.10496	-0.12785	0.12785	0.34087	0.92217	0.13488	0.12219	0.79395	0.00104	0.17187	0.09563	0.09563	turquoise
TRIM31	0.06041	0.15081	0.1233	0.09187	0.11624	0.21592	0.15792	-0.4325	0.4325	0.43253	0.04897	0.10813	0.23203	0.13	0.00456	0.03912	3.48E-09	3.48E-09	yellow
NAP1L1	0.03465	-0.12959	-0.02387	-0.06362	-0.07899	-0.02625	-0.10718	0.29215	-0.29215	0.65277	0.09116	0.75661	0.4084	0.30444	0.73322	0.16293	0.00011	0.00011	turquoise
ZFP14	0.04377	-0.17537	-0.15903	-0.03711	0.02086	-0.08202	-0.1486	0.3039	-0.3039	0.56974	0.02178	0.03775	0.62985	0.78656	0.2862	0.05242	5.33E-05	5.33E-05	turquoise
SLC24A3	0.06676	-0.03261	-0.01477	0.01727	-0.06812	-0.05398	-0.05061	0.29628	-0.29628	0.38567	0.67204	0.84797	0.82263	0.37598	0.48314	0.51094	8.33E-05	8.33E-05	turquoise
ZNF639	-0.10355	-0.06779	-0.11163	0.0401	-0.11497	-0.00849	0.02841	0.16593	-0.16593	0.17773	0.3783	0.14608	0.60254	0.13429	0.91227	0.71219	0.03009	0.03009	turquoise
PQBP1	0.13154	-0.04754	-0.13503	-0.03511	0.11181	-0.23218	0.02968	-0.213	0.213	0.08634	0.53695	0.07826	0.6485	0.14542	0.00225	0.69993	0.00516	0.00516	grey
CTTN	0.0225	-0.0451	0.04559	0.03479	-0.04303	0.16363	0.21448	-0.36836	0.36836	0.77023	0.5581	0.55381	0.65146	0.57632	0.03247	0.00485	7.16E-07	7.16E-07	yellow
ENKUR	-0.05279	-0.24115	-0.03161	0.03593	0.03932	-0.11052	-0.13645	-0.10438	0.10438	0.49286	0.00149	0.68146	0.64084	0.60959	0.15014	0.07516	0.17424	0.17424	grey
IFT88	0.01581	-0.13456	-0.15619	-0.13304	0.13587	-0.15741	-0.09243	0.10726	-0.10726	0.83735	0.07932	0.04135	0.08279	0.07641	0.03977	0.22919	0.16261	0.16261	turquoise
THAP5	-0.02105	-0.18328	-0.10097	-0.03556	-0.00586	-0.08462	-0.13314	0.29326	-0.29326	0.78468	0.01642	0.18883	0.64427	0.9394	0.27114	0.08257	9.91E-05	9.91E-05	turquoise
BCAM	-0.03391	-0.12034	-0.24178	-0.079	0.0815	-0.29223	-0.08978	0.20737	-0.20737	0.65973	0.11693	0.00144	0.30439	0.28925	0.00011	0.24288	0.0065	0.0065	brown
TP53BP2	0.01955	-0.13397	0.02625	0.02498	-0.1115	0.20509	0.06961	-0.06676	0.06676	0.79965	0.08065	0.73328	0.74568	0.14653	0.00713	0.3656	0.38563	0.38563	turquoise
ZFAND6	0.0045	-0.12354	-0.06709	-0.01944	0.00709	0.02319	0.02213	-0.05155	0.05155	0.95346	0.10744	0.38327	0.80077	0.92671	0.76334	0.77385	0.5031	0.5031	turquoise
PDE7A	-0.0829	-0.08753	0.08938	0.13102	-0.16042	0.13619	0.08546	0.11916	-0.11916	0.28104	0.25496	0.24503	0.08763	0.03608	0.0757	0.26638	0.12057	0.12057	blue
SMAD4	-0.02967	-0.13738	-0.16102	0.02065	0.04948	-0.1419	-0.08307	0.4829	-0.4829	0.70008	0.07316	0.03539	0.7886	0.5204	0.06412	0.28006	2.25E-11	2.25E-11	turquoise
BBIP1	-0.05722	-0.1954	-0.14658	-0.04401	0.03175	-0.18926	-0.22506	0.31242	-0.31242	0.45722	0.01043	0.05573	0.56759	0.68013	0.01317	0.00308	3.18E-05	3.18E-05	turquoise
NCOA7	-0.11268	-0.09548	0.0211	0.05783	-0.06456	0.12276	-0.04086	0.14854	-0.14854	0.14229	0.21416	0.78411	0.45246	0.40148	0.10971	0.59566	0.05251	0.05251	turquoise
TMSB4X	-0.03947	-0.06965	0.03402	0.04231	-0.12146	0.20994	0.15166	-0.16566	0.16566	0.60826	0.36535	0.65866	0.58269	0.11353	0.00585	0.0477	0.03036	0.03036	black
ARF3	0.0633	-0.22758	-0.1135	-0.03446	-0.02454	-0.14812	-0.11059	0.06654	-0.06654	0.41082	0.00276	0.13938	0.65458	0.75001	0.05319	0.14989	0.38722	0.38722	turquoise
LZTS1	-0.02179	-0.10336	-0.08461	-0.00368	-0.20005	0.02939	-0.00116	0.44863	-0.44863	0.77729	0.17853	0.2712	0.96193	0.0087	0.70274	0.988	7.56E-10	7.56E-10	blue
SAT1	-0.09535	-0.01588	0.05012	0.02745	-0.0917	0.15775	0.20895	-0.23901	0.23901	0.21475	0.83668	0.515	0.72156	0.23295	0.03933	0.00609	0.00164	0.00164	yellow
SMC6	-0.02146	-0.06024	0.02817	-0.01411	-0.09017	0.03154	0.07514	0.1557	-0.1557	0.78058	0.43383	0.71454	0.85464	0.24083	0.68219	0.32871	0.042	0.042	turquoise
PDCL3	0.02385	0.06077	0.00803	0.02028	-0.10481	0.01001	0.06824	0.01925	-0.01925	0.75687	0.42981	0.91701	0.7923	0.17248	0.89657	0.37517	0.80269	0.80269	turquoise
TEDC1	0.06155	0.06402	-0.02722	-0.00477	0.24518	-0.04638	0.09588	-0.42388	0.42388	0.42387	0.40544	0.72375	0.95059	0.00123	0.54692	0.21223	7.60E-09	7.60E-09	green
SERINC1	-0.08269	-0.17494	-0.06655	-0.00117	-0.07677	-0.08029	-0.16013	0.44498	-0.44498	0.28228	0.0221	0.38711	0.98786	0.31827	0.29654	0.03643	1.08E-09	1.08E-09	turquoise
HIC1	-0.05428	-0.12842	-0.04648	0.05633	-0.08876	0.05689	0.03603	0.3357	-0.3357	0.48076	0.09415	0.54607	0.4643	0.2483	0.45988	0.6399	7.17E-06	7.17E-06	black
CACNB3	0.09827	-0.18172	0.00201	0.02872	0.0649	-0.00287	0.04731	-0.33678	0.33678	0.20102	0.01737	0.97922	0.70922	0.39902	0.9703	0.53893	6.67E-06	6.67E-06	grey
CLIC2	-0.14948	-0.05868	0.01181	0.09715	-0.16376	0.13049	-0.02807	0.48757	-0.48757	0.05101	0.44585	0.87813	0.2062	0.03234	0.08892	0.71552	1.35E-11	1.35E-11	blue
PIK3C2A	-0.08262	-0.17104	-0.00138	0.05664	-0.05746	0.12068	0.0231	0.22101	-0.22101	0.28267	0.02531	0.98574	0.46182	0.45534	0.11589	0.76423	0.00367	0.00367	turquoise
CRACR2B	0.08905	0.07071	0.0754	-0.03575	0.23006	0.12264	0.0891	-0.50673	0.50673	0.24676	0.35806	0.32701	0.6425	0.00247	0.11004	0.2465	1.53E-12	1.53E-12	brown
TIGD7	-0.04246	-0.19704	-0.11989	0.02547	-0.08677	-0.01222	-0.06238	0.36064	-0.36064	0.58134	0.00979	0.1183	0.74084	0.25914	0.874	0.41765	1.26E-06	1.26E-06	turquoise
LPAR5	0.01771	0.11573	0.12338	0.03552	0.04656	0.25183	0.18577	-0.34679	0.34679	0.81819	0.13173	0.1079	0.64464	0.54538	0.00089	0.01499	3.37E-06	3.37E-06	yellow
LRRC2	-0.04813	-0.08975	-0.0906	-0.02528	0.03992	-0.03778	-0.16913	0.5544	-0.5544	0.53192	0.24308	0.23858	0.74273	0.60417	0.6237	0.02701	3.64E-15	3.64E-15	blue
ELFN2	0.01273	-0.19436	-0.03526	0.05616	-0.00973	-0.2329	-0.07655	0.00123	-0.00123	0.86875	0.01086	0.64703	0.46562	0.89944	0.00217	0.31964	0.98727	0.98727	grey
HNRNPH1	-0.00348	-0.17319	-0.04822	-0.02075	0.08129	-0.04199	-0.06048	0.11011	-0.11011	0.96398	0.0235	0.5311	0.78762	0.29054	0.58558	0.43199	0.15167	0.15167	red
NTN1	0.01732	-0.07825	0.10782	0.16598	-0.07085	-0.01543	0.05637	0.12716	-0.12716	0.8221	0.30903	0.16042	0.03004	0.35715	0.84126	0.46399	0.09743	0.09743	grey
DGKG	-0.07412	-0.05043	-0.14881	0.02681	-0.00838	-0.08305	-0.04846	0.17408	-0.17408	0.33532	0.51242	0.05208	0.72777	0.91339	0.28017	0.52904	0.02278	0.02278	grey
VPS41	-0.05956	-0.18823	-0.04731	0.00065	-0.06993	-0.02717	-0.04027	0.23599	-0.23599	0.439	0.01368	0.53893	0.99323	0.3634	0.72424	0.60105	0.00189	0.00189	turquoise
PRAC2	0.21849	0.11274	-0.01044	-0.03265	0.03662	0.06882	0.23374	-0.33455	0.33455	0.00409	0.14208	0.89218	0.67157	0.63443	0.37108	0.00209	7.74E-06	7.74E-06	grey
ZNF614	-0.10149	-0.18094	-0.11838	-0.00876	-0.11197	-0.03037	-0.11993	0.37413	-0.37413	0.18657	0.01787	0.12305	0.90945	0.14483	0.69336	0.11818	4.64E-07	4.64E-07	turquoise
ALDOB	0.06475	-0.05071	0.10834	0.08437	0.12133	0.21942	-0.02977	-0.06893	0.06893	0.4001	0.5101	0.15841	0.27255	0.11392	0.00393	0.69909	0.37037	0.37037	grey
MON1B	-0.03316	-0.1083	-0.06467	-0.05413	-0.05019	0.06897	0.05062	0.02786	-0.02786	0.66676	0.15854	0.40069	0.48198	0.51448	0.37006	0.51089	0.71757	0.71757	turquoise
MNDA	-0.13006	0.04535	0.07281	0.02044	-0.22672	0.15694	0.10282	0.42364	-0.42364	0.08998	0.55587	0.34396	0.7907	0.00286	0.04037	0.18083	7.77E-09	7.77E-09	blue
NLRP6	-0.08246	-0.05655	0.11791	0.07081	0.01431	0.12575	0.06151	0.0569	-0.0569	0.28363	0.46254	0.12457	0.35742	0.85263	0.10124	0.42414	0.45977	0.45977	grey
CDK20	0.04588	-0.15395	-0.2576	-0.03106	0.10248	-0.2033	-0.07404	0.18517	-0.18517	0.55125	0.04438	0.00067	0.68676	0.18226	0.00766	0.33582	0.01532	0.01532	brown
ARHGAP2	-0.14686	-0.23136	0.00851	0.13889	-0.21278	0.12715	0.06194	0.24014	-0.24014	0.05526	0.00233	0.91207	0.07003	0.0052	0.09747	0.42091	0.00156	0.00156	turquoise
NPHP3	-0.05516	-0.12582	-0.04617	0.02334	0.03258	0.01557	-0.03097	0.08207	-0.08207	0.47368	0.10105	0.54872	0.76191	0.67228	0.83984	0.68757	0.28592	0.28592	red
SLC19A1	0.02786	0.06714	-0.0489	0.04039	0.04991	-0.00998	0.20579	-0.31935	0.31935	0.71753	0.38294	0.52534	0.59993	0.51677	0.89696	0.00693	2.07E-05	2.07E-05	grey
SPTY2D1	-0.02736	-0.13478	0.04668	0.05002	-0.13151	0.19805	0.1115	0.04968	-0.04968	0.72243	0.07883	0.54435	0.51592	0.08643	0.00942	0.14654	0.51878	0.51878	turquoise
SUGT1	0.12382	-0.09205	-0.07293	-0.07128	-0.00878	-0.143	0.02553	-0.07058	0.07058	0.10663	0.23115	0.34314	0.35421	0.90924	0.06207	0.74031	0.35899	0.35899	turquoise
EIF2B1	0.03221	-0.06132	0.02567	-0.05642	0.02646	0.00174	0.05814	-0.00415	0.00415	0.67582	0.42563	0.73891	0.46361	0.73122	0.98199	0.45004	0.95701	0.95701	turquoise
SNX27	0.05623	-0.12915	-0.06367	-0.06593	-0.02079	0.04834	0.07071	-0.00277	0.00277	0.46513	0.09229	0.40806	0.3916	0.78723	0.5301	0.35806	0.97137	0.97137	turquoise
SPATA6	-0.04203	-0.17031	-0.03265	0.02413	0.03126	0.09227	-0.10671	0.15494	-0.15494	0.58516	0.02594	0.67166	0.75406	0.6848	0.23004	0.16477	0.04302	0.04302	turquoise
FCRLA	-0.09846	0.01495	0.04097	0.00018	-0.0807	0.00973	-0.11868	0.32486	-0.32486	0.20012	0.84608	0.59472	0.99817	0.29403	0.89951	0.12211	1.46E-05	1.46E-05	blue
CRYBG1	-0.09884	-0.0493	0.11365	0.09753	-0.2019	0.27115	0.22665	-0.06516	0.06516	0.1984	0.52191	0.13885	0.20441	0.00809	0.00033	0.00287	0.39715	0.39715	turquoise
CD68	-0.08375	0.10651	0.14193	0.11795	-0.08726	0.16081	0.12613	0.01332	-0.01332	0.27612	0.1656	0.06406	0.12443	0.25641	0.03563	0.10022	0.86276	0.86276	blue
YPEL5	-0.02467	-0.18315	-0.01502	0.04802	-0.11937	0.06691	-0.00945	0.05783	-0.05783	0.74871	0.0165	0.84536	0.53279	0.11994	0.38458	0.90235	0.45248	0.45248	turquoise
UBXN2B	0.00826	-0.0941	-0.01319	0.05429	-0.21496	0.12302	0.09633	0.03831	-0.03831	0.91458	0.22086	0.86406	0.48069	0.00475	0.10895	0.21009	0.61882	0.61882	turquoise
PTGES	0.03968	0.01617	0.00106	0.01275	-0.15351	0.08555	0.32206	-0.3079	0.3079	0.60635	0.83377	0.98898	0.86854	0.04501	0.26591	1.74E-05	4.19E-05	4.19E-05	yellow
ANAPC15	-0.00432	-0.00785	-0.13272	-0.21135	0.08601	-0.17003	-0.04421	-0.01973	0.01973	0.95529	0.91886	0.08354	0.00552	0.26335	0.0262	0.56584	0.79783	0.79783	grey
MKI67	0.00264	0.103	0.05051	-0.02371	-0.07941	0.12961	0.3275	-0.41912	0.41912	0.97266	0.18007	0.5118	0.75825	0.30188	0.0911	1.23E-05	1.16E-08	1.16E-08	pink
SLC25A32	-0.10583	-0.04191	-0.0232	0.00456	-0.08474	-0.06046	-0.03497	0.29229	-0.29229	0.16831	0.58622	0.76331	0.95277	0.27048	0.43217	0.64976	0.0001	0.0001	turquoise
CCDC127	-0.00562	-0.21208	-0.11778	-0.01743	0.01784	-0.19949	0.06458	0.09787	-0.09787	0.9418	0.00536	0.12497	0.821	0.81681	0.0089	0.40139	0.20282	0.20282	turquoise
HS6ST1	0.00885	-0.12228	-0.13023	0.10383	-0.1129	-0.04069	0.0687	0.30549	-0.30549	0.90849	0.11109	0.08955	0.17657	0.14149	0.59721	0.37197	4.84E-05	4.84E-05	magenta
RECQL5	-0.0416	-0.15061	-0.12187	-0.09951	0.22639	-0.15312	0.00964	-0.23636	0.23636	0.58905	0.04926	0.11231	0.19535	0.00291	0.04556	0.90043	0.00186	0.00186	red
NKG7	-0.08528	0.09754	0.0522	0.09094	-0.18069	0.06862	0.08319	0.28243	-0.28243	0.26742	0.20439	0.49773	0.23683	0.01803	0.37249	0.27936	0.00018	0.00018	blue
CTSD	0.05425	-0.02761	0.03264	0.06244	-0.09101	0.03668	0.20163	-0.1913	0.1913	0.481	0.71999	0.67175	0.41722	0.23647	0.63384	0.00818	0.0122	0.0122	grey
CCND1	0.0233	0.06776	0.00341	0.02293	-0.12077	0.16795	0.22663	-0.30771	0.30771	0.76228	0.37856	0.96472	0.76597	0.11561	0.02811	0.00288	4.24E-05	4.24E-05	grey
FAM131B	-0.01522	-0.09953	0.00997	0.00539	0.01045	0.00279	-0.00051	0.08826	-0.08826	0.84333	0.19524	0.89706	0.94424	0.89212	0.97112	0.9947	0.25101	0.25101	grey
FOXC2	-0.14431	-0.22583	0.06385	0.07074	-0.06081	0.07051	0.08153	-0.0216	0.0216	0.05969	0.00298	0.40673	0.35788	0.42945	0.35944	0.28908	0.77915	0.77915	grey
C18orf25	-0.08442	-0.19525	-0.07312	0.00804	-0.01196	-0.01581	-0.04184	0.23963	-0.23963	0.27231	0.01049	0.34188	0.91687	0.87659	0.83742	0.58691	0.0016	0.0016	turquoise
SH3RF1	-0.01028	-0.0802	0.00417	0.00748	0.07224	0.14356	-0.00256	-0.25288	0.25288	0.8938	0.29706	0.95684	0.92262	0.34777	0.06104	0.97346	0.00085	0.00085	turquoise
HSPA6	0.09401	-0.12546	0.10632	0.09174	0.00861	0.18923	0.03393	-0.12912	0.12912	0.22131	0.10205	0.16636	0.2327	0.91103	0.01318	0.65954	0.09236	0.09236	grey
GPAA1	0.06099	-0.00935	-0.17647	-0.06708	0.06939	-0.18001	0.0804	-0.214	0.214	0.42812	0.90338	0.02095	0.38338	0.36717	0.01848	0.29589	0.00495	0.00495	green
GPR84	-0.12151	0.01626	0.09113	0.08645	-0.19152	0.17443	0.18734	0.28592	-0.28592	0.11338	0.83283	0.23585	0.26089	0.01209	0.0225	0.01414	0.00015	0.00015	blue
GALNT11	-0.1475	-0.15314	-0.12167	-0.02017	-0.02827	-0.11322	-0.14804	0.47986	-0.47986	0.05421	0.04554	0.1129	0.79343	0.71363	0.14035	0.05331	3.12E-11	3.12E-11	turquoise
CCR4	-0.12617	-0.08122	0.07337	0.09412	-0.18476	0.16789	-0.03687	0.49149	-0.49149	0.10011	0.29097	0.34027	0.22077	0.01555	0.02816	0.63208	8.74E-12	8.74E-12	blue
ACP6	0.1034	0.04726	0.08038	-0.07618	0.13695	0.04816	0.09206	-0.48442	0.48442	0.17838	0.53938	0.29597	0.322	0.07407	0.53165	0.23107	1.90E-11	1.90E-11	grey
TRIM69	-0.05654	-0.07592	-0.03894	0.02325	-0.10555	0.00965	0.03223	0.1297	-0.1297	0.4626	0.32365	0.61305	0.76278	0.16943	0.90034	0.67559	0.09088	0.09088	tan
ZBED3	-0.10931	-0.07518	-0.23582	-0.0125	-0.03114	-0.11208	-0.16169	0.54879	-0.54879	0.15468	0.32844	0.0019	0.87108	0.68598	0.14444	0.03462	7.80E-15	7.80E-15	turquoise
PLXNB1	0.05107	-0.08205	-0.10899	-0.07847	0.23208	-0.08661	-0.02178	-0.2049	0.2049	0.50707	0.28605	0.1559	0.30762	0.00226	0.25998	0.77737	0.00718	0.00718	grey
MPHOSPH	-0.08233	-0.06041	-0.04598	-0.01373	-0.11887	-0.01971	0.08422	-0.0818	0.0818	0.28438	0.43249	0.55038	0.85857	0.1215	0.79809	0.27341	0.28751	0.28751	turquoise
CD276	-0.02598	-0.11812	-0.03386	0.05579	-0.1364	0.03731	0.12457	0.0206	-0.0206	0.73585	0.12387	0.66021	0.46862	0.07526	0.628	0.10451	0.78918	0.78918	black
CPSF7	-0.04376	-0.12758	-0.06734	-0.06028	-0.03828	-0.01956	0.00962	0.11993	-0.11993	0.5698	0.09633	0.38151	0.43348	0.61912	0.79957	0.90059	0.11818	0.11818	turquoise
FBXL6	0.02909	0.08321	-0.07188	-0.07948	0.13309	-0.09118	0.11691	-0.48042	0.48042	0.70568	0.27925	0.35013	0.30143	0.08268	0.23562	0.12781	2.94E-11	2.94E-11	green
JMY	-0.01976	-0.16435	-0.13404	0.01541	-0.09581	-0.07453	-0.11123	0.45887	-0.45887	0.79759	0.03171	0.0805	0.84144	0.21254	0.33265	0.14751	2.75E-10	2.75E-10	turquoise
CPSF4	0.06186	-0.1078	0.00819	0.00437	0.09139	-0.09123	0.18112	-0.21522	0.21522	0.42156	0.16049	0.91534	0.95481	0.23453	0.23535	0.01776	0.0047	0.0047	grey
ZNF217	0.02104	-0.09779	0.09586	0.12795	-0.14959	0.15913	0.1114	-0.0971	0.0971	0.7847	0.2032	0.21233	0.09538	0.05084	0.03762	0.14691	0.20642	0.20642	turquoise
CRYZL1	-0.05125	-0.0696	-0.02484	-0.01194	0.07818	-0.0126	-0.04614	0.13652	-0.13652	0.5056	0.36567	0.74705	0.8768	0.30947	0.87008	0.549	0.075	0.075	turquoise
STRIP2	-0.00257	0.08308	-0.00401	-0.01821	0.05224	-0.07767	0.098	-0.2577	0.2577	0.9734	0.27997	0.95851	0.81311	0.49744	0.3126	0.20223	0.00067	0.00067	grey
UBR1	-0.07645	-0.1314	-0.0061	0.02345	-0.16	0.07247	-0.01323	0.19991	-0.19991	0.32033	0.08668	0.93691	0.76081	0.03658	0.34621	0.86368	0.00875	0.00875	turquoise
GSAP	-0.0348	-0.12243	-0.0067	-0.08891	0.18853	-0.06917	-0.09189	0.00563	-0.00563	0.6514	0.11067	0.93069	0.2475	0.01353	0.36869	0.23198	0.94172	0.94172	red
TRIM32	-0.02782	-0.12681	-0.02683	0.06822	-0.24474	0.1072	0.09394	0.16157	-0.16157	0.71792	0.09838	0.72755	0.37532	0.00125	0.16283	0.22168	0.03475	0.03475	turquoise
CCDC8	-0.02246	-0.07893	0.023	0.12391	-0.21064	0.09434	0.04205	0.39913	-0.39913	0.77057	0.30482	0.76524	0.10638	0.00569	0.21968	0.58497	6.39E-08	6.39E-08	black
AL365205	-0.01772	-0.01289	-0.01111	0.04134	0.16247	0.08113	0.12607	-0.17839	0.17839	0.81811	0.86706	0.88536	0.59139	0.03374	0.2915	0.10037	0.01957	0.01957	grey
CCDC167	-0.01882	0.09051	-0.08472	-0.02426	0.00034	-0.00859	0.10271	-0.1474	0.1474	0.807	0.23905	0.27057	0.75278	0.99646	0.91117	0.1813	0.05437	0.05437	grey
OXCT1	-0.082	-0.07797	0.00402	-0.07727	-0.07335	-0.01812	-0.12279	0.32943	-0.32943	0.28631	0.31072	0.95837	0.31511	0.34039	0.81398	0.10959	1.08E-05	1.08E-05	turquoise
MSRA	0.08226	0.083	0.05195	0.02547	0.01166	0.1044	0.14512	-0.12871	0.12871	0.28478	0.28048	0.49982	0.74092	0.87972	0.17418	0.05826	0.0934	0.0934	grey
RHOQ	-0.07014	-0.10675	-0.00582	0.10033	-0.21602	0.03764	-0.04399	0.41391	-0.41391	0.36198	0.16463	0.93982	0.19167	0.00454	0.62503	0.5678	1.83E-08	1.83E-08	blue
SLFN13	-0.08945	-0.04179	0.02619	0.0833	0.02227	0.18045	0.1947	-0.25167	0.25167	0.24463	0.58735	0.73382	0.27872	0.77247	0.01818	0.01072	0.0009	0.0009	yellow
ARHGEF1!	-0.12084	-0.05438	-0.06113	0.08068	-0.05462	-0.04544	-0.1376	0.60508	-0.60508	0.11541	0.47993	0.42708	0.29417	0.47797	0.55512	0.0727	1.86E-18	1.86E-18	blue
DMWD	0.04842	-0.10577	-0.09217	-0.07911	0.01554	-0.02151	0.09325	0.06	-0.06	0.52946	0.16854	0.23051	0.30371	0.84014	0.78008	0.2251	0.43564	0.43564	grey
CELSR1	-0.03681	0.04958	0.01942	0.04191	-0.06906	0.03705	0.16115	-0.30131	0.30131	0.63262	0.51955	0.80097	0.58625	0.36943	0.63044	0.03524	6.21E-05	6.21E-05	turquoise
ANAPC5	0.02136	-0.06828	-0.04361	-0.06069	0.131	-0.12354	-0.04752	0.12752	-0.12752	0.78155	0.37486	0.57114	0.43041	0.08766	0.10743	0.53712	0.09649	0.09649	turquoise
DRG1	-0.07961	-0.00906	-0.11913	-0.01806	-0.11531	-0.05081	0.12754	-0.0064	0.0064	0.30063	0.90639	0.12067	0.81468	0.13314	0.50928	0.09644	0.93381	0.93381	grey
ABRAXAS1	-0.04667	-0.01766	0.0471	0.00862	-0.15241	0.07047	-0.02404	0.268	-0.268	0.54439	0.81866	0.54071	0.91092	0.04658	0.35969	0.75497	0.00039	0.00039	turquoise
CFAP20	0.00176	-0.11721	-0.08383	-0.11407	0.00474	-0.04407	-0.04878	0.01368	-0.01368	0.98177	0.12681	0.27568	0.13741	0.95095	0.56707	0.52635	0.85908	0.85908	turquoise
ATG4D	0.08112	-0.05994	-0.16165	-0.12912	0.22793	-0.15946	-0.17068	-0.18406	0.18406	0.29153	0.43611	0.03466	0.09236	0.00272	0.03723	0.02562	0.01596	0.01596	green
SPDYE3	-0.02366	-0.02854	-0.00946	-0.0464	-0.02031	0.05616	0.08475	-0.08503	0.08503	0.75872	0.71095	0.90228	0.54677	0.79208	0.46562	0.27042	0.26884	0.26884	grey
SAYSD1	-0.04777	-0.00118	-0.16054	0.01004	0.16597	-0.23868	-0.1523	0.23089	-0.23089	0.53495	0.98774	0.03595	0.89634	0.03004	0.00167	0.04674	0.00238	0.00238	grey
ABLIM3	0.07073	-0.07752	-0.02806	0.05059	-0.10832	0.07386	0.11244	-0.25243	0.25243	0.35798	0.31357	0.71559	0.51111	0.15846	0.33704	0.14313	0.00087	0.00087	turquoise
NUB1	-0.02362	-0.0612	0.00933	-0.00926	-0.10917	0.02588	0.12482	-0.0126	0.0126	0.75908	0.42648	0.90362	0.9043	0.1552	0.73683	0.10382	0.87012	0.87012	turquoise
ZSCAN25	-0.07913	-0.22619	-0.04954	0.04333	0.03006	-0.02001	-0.04595	0.21827	-0.21827	0.30359	0.00293	0.51991	0.57365	0.69632	0.79503	0.55064	0.00413	0.00413	turquoise
BAX	0.02877	0.06276	-0.00896	-0.05218	0.04716	-0.05477	0.13259	-0.26607	0.26607	0.70871	0.41482	0.90742	0.49789	0.54019	0.47681	0.08386	0.00044	0.00044	grey
MSR1	-0.09674	0.02739	0.09602	0.06823	-0.25531	0.19229	0.11124	0.36684	-0.36684	0.20811	0.72218	0.21154	0.3752	0.00075	0.01175	0.14747	8.02E-07	8.02E-07	blue
WNK1	0.00995	-0.06935	0.00137	0.04479	-0.08271	0.02494	-0.07566	0.2139	-0.2139	0.89722	0.36742	0.98579	0.56081	0.28215	0.7461	0.32537	0.00497	0.00497	turquoise
RNF224	0.00682	-0.01215	-0.16968	-0.10231	0.10217	-0.09855	-0.00103	0.00684	-0.00684	0.92944	0.87465	0.0265	0.18301	0.1836	0.19971	0.98932	0.92929	0.92929	grey
MEAK7	0.03251	-0.09744	0.05244	0.04651	-0.10642	0.08272	0.2522	-0.28965	0.28965	0.67293	0.20485	0.49575	0.54577	0.16594	0.2821	0.00088	0.00012	0.00012	turquoise
POLR1B	-0.06876	0.01554	-0.03258	0.01622	-0.13043	0.05189	0.05205	0.15275	-0.15275	0.37152	0.84017	0.67228	0.83326	0.08906	0.50031	0.49897	0.04609	0.04609	turquoise
SLC35E2B	-0.09559	-0.12644	-0.08552	-0.00738	-0.02736	0.02123	-0.13222	0.31074	-0.31074	0.2136	0.09938	0.26604	0.92365	0.72245	0.78282	0.08473	3.53E-05	3.53E-05	turquoise
ZFP2	-0.07532	-0.10382	-0.1909	-0.05075	0.11082	-0.24398	-0.24009	0.44876	-0.44876	0.32752	0.17661	0.01238	0.5098	0.14904	0.0013	0.00156	7.47E-10	7.47E-10	brown
NKX6-1	0.0476	-0.12852	-0.21261	-0.04162	0.06028	-0.16112	-0.11362	0.30347	-0.30347	0.53638	0.09388	0.00524	0.58887	0.4335	0.03527	0.13897	5.46E-05	5.46E-05	brown
DNAJC15	0.02535	0.07607	-0.03642	-0.01285	0.03538	-0.05343	-0.01131	-0.0344	0.0344	0.74207	0.32273	0.63625	0.86757	0.64591	0.48768	0.8833	0.65514	0.65514	grey

LAIR2	-0.13692	0.11661	0.00438	-0.06635	-0.0196	0.13842	-0.02122	0.01707	-0.01707	0.07413	0.12879	0.95461	0.38856	0.79913	0.07099	0.78291	0.82467	0.82467 grey
PPP1R3C	0.0304	-0.23938	-0.04416	0.18779	0.0066	0.0218	-0.0395	0.0586	-0.0586	0.69309	0.00161	0.56629	0.01391	0.93172	0.77712	0.60803	0.44649	0.44649 grey
SLC27A1	0.0078	-0.02618	-0.06363	-0.00553	0.0104	0.03672	0.16689	-0.25348	0.25348	0.91934	0.73391	0.40837	0.94274	0.89256	0.63352	0.02913	0.00082	0.00082 grey
TM7SF3	-0.02061	-0.04536	0.0555	0.10425	-0.0639	0.07155	0.0891	-0.00906	0.00906	0.78907	0.55582	0.47095	0.17478	0.40633	0.35239	0.2465	0.9064	0.9064 turquoise
CEACAM7	0.04849	-0.0214	-0.06934	-0.03009	0.06382	0.16049	0.03766	-0.019	0.019	0.52881	0.78114	0.36751	0.69602	0.40695	0.036	0.62481	0.8052	0.8052 grey
GSS	0.02483	-0.0179	0.01971	-0.00791	0.14536	-0.00283	0.16482	-0.39683	0.39683	0.74715	0.8162	0.79802	0.91822	0.05783	0.97073	0.03122	7.73E-08	7.73E-08 grey
DOK3	-0.16445	-0.04759	0.05821	0.03273	-0.15719	0.08024	-0.01812	0.33885	-0.33885	0.0316	0.53651	0.4495	0.67081	0.04004	0.29685	0.81403	5.80E-06	5.80E-06 blue
SMIM27	0.00927	-0.05446	-0.22642	-0.159	0.03568	-0.26364	-0.20585	0.22869	-0.22869	0.90426	0.47924	0.0029	0.03779	0.64314	0.00049	0.00691	0.00263	0.00263 grey
COMMD3	0.01373	-0.00139	-0.08078	-0.17674	0.03708	0.00566	0.02525	-0.1986	0.1986	0.85859	0.98565	0.29358	0.02075	0.63019	0.94141	0.74304	0.00921	0.00921 grey
HLA-DPA1	-0.12845	0.03837	-0.02053	0.03734	-0.2447	0.16204	0.09608	0.35568	-0.35568	0.09406	0.61831	0.78984	0.62772	0.00126	0.03422	0.21127	1.81E-06	1.81E-06 blue
FNBP4	-0.00463	-0.14126	0.01126	-0.01102	0.00471	0.10222	0.01778	-0.04617	0.04617	0.95205	0.06535	0.88377	0.8862	0.95121	0.18341	0.81746	0.54873	0.54873 red
GPX8	-0.04524	-0.04655	0.06609	0.07203	-0.18985	0.22189	0.12246	0.11661	-0.11661	0.55679	0.54543	0.39044	0.34915	0.01288	0.00354	0.11058	0.12879	0.12879 black
STX3	0.07503	-0.11805	-0.0416	-0.05833	0.03657	0.04523	0.10061	-0.17274	0.17274	0.32943	0.1241	0.58904	0.44855	0.63484	0.5569	0.19041	0.02386	0.02386 turquoise
CFAP44	-0.01955	-0.13163	-0.12362	-0.02768	0.18098	-0.02744	-0.06987	-0.05996	0.05996	0.79963	0.08613	0.10722	0.71934	0.01784	0.7216	0.36387	0.43593	0.43593 red
GAPVD1	-0.07535	-0.1863	-0.07522	0.00619	-0.15922	-0.00223	0.00865	0.31152	-0.31152	0.32734	0.0147	0.32818	0.93599	0.03752	0.97691	0.91056	3.36E-05	3.36E-05 turquoise
TRAM1L1	-0.07327	-0.0452	-0.27131	-0.09623	0.00557	-0.25489	-0.19203	0.49108	-0.49108	0.34088	0.55719	0.00033	0.21056	0.94234	0.00077	0.01186	9.15E-12	9.15E-12 brown
MDM4	0.03759	-0.1287	-0.0764	-0.04642	0.12821	-0.01961	-0.06614	-0.00841	0.00841	0.62545	0.09341	0.32064	0.54659	0.09469	0.799	0.39009	0.91308	0.91308 red
MAP2	-0.02883	-0.17767	-0.21168	-0.05626	-0.07777	-0.2378	-0.23194	0.45133	-0.45133	0.70814	0.02008	0.00545	0.46482	0.31198	0.00174	0.00227	5.81E-10	5.81E-10 brown
ZNF8	-0.05112	-0.18371	-0.15406	-0.0086	-0.02506	-0.08277	-0.11009	0.26011	-0.26011	0.50667	0.01617	0.04424	0.91106	0.74487	0.28181	0.15173	0.00059	0.00059 turquoise
SRPX2	-0.01882	-0.07964	0.0599	0.09063	-0.22279	0.15839	0.12668	0.15773	-0.15773	0.80701	0.30048	0.43642	0.23846	0.0034	0.03853	0.09871	0.03937	0.03937 black
CDC25C	0.00125	0.0457	0.02133	-0.03277	-0.02836	0.10363	0.35197	-0.53151	0.53151	0.98703	0.55279	0.78182	0.6705	0.71274	0.17741	2.35E-06	7.45E-14	7.45E-14 pink
STK32B	-0.09718	-0.09445	-0.05338	0.01367	-0.06843	-0.12881	-0.17548	0.44843	-0.44843	0.20608	0.21917	0.48803	0.85914	0.37386	0.09315	0.02169	7.71E-10	7.71E-10 grey
TRAP1	0.05253	0.07419	-0.05547	-0.12717	0.12538	0.00164	0.03497	-0.19234	0.19234	0.49504	0.33487	0.47112	0.09742	0.10228	0.98303	0.64978	0.01173	0.01173 turquoise
NPY	-0.02387	-0.01055	-0.14179	0.0191	-0.02634	-0.15713	-0.02568	0.27234	-0.27234	0.75665	0.89104	0.06433	0.80421	0.73235	0.04012	0.73882	0.00031	0.00031 grey
FCGR1A	-0.07564	0.08283	0.07014	0.07173	-0.26519	0.12624	0.14232	0.30369	-0.30369	0.32544	0.28145	0.36198	0.35121	0.00046	0.09991	0.06333	5.39E-05	5.39E-05 blue
ZNF84	-0.00645	-0.18381	-0.08127	-0.01934	0.10758	-0.1027	-0.12858	0.19386	-0.19386	0.93324	0.0161	0.29063	0.80177	0.16135	0.18133	0.09372	0.01107	0.01107 turquoise
BCKDHA	-0.05593	-0.13011	-0.12465	0.02069	0.0856	-0.0896	-0.06703	0.12197	-0.12197	0.46745	0.08987	0.1043	0.7882	0.2656	0.24383	0.38368	0.11201	0.11201 grey
COBL	0.04928	-0.06841	-0.00245	0.11625	0.09502	-0.01667	-0.1657	-0.02093	0.02093	0.52216	0.37399	0.97464	0.13	0.21638	0.8287	0.03032	0.78588	0.78588 grey
MCRS1	0.1091	-0.03464	-0.12005	-0.09628	0.154	-0.14577	-0.06364	-0.15928	0.15928	0.15548	0.65284	0.11781	0.21033	0.04432	0.05713	0.40827	0.03744	0.03744 turquoise
NTN4	0.03462	-0.15041	0.08017	0.12524	-0.05141	0.17121	0.06296	-0.12926	0.12926	0.65308	0.04958	0.29725	0.10264	0.50428	0.02515	0.4133	0.09199	0.09199 turquoise
PADI2	-0.05013	-0.03375	0.14427	0.1755	-0.1786	0.24274	0.00686	0.15739	-0.15739	0.51492	0.66119	0.05975	0.02168	0.01943	0.00138	0.92904	0.0398	0.0398 blue
ST3GAL5	0.04188	-0.12335	-0.05081	-0.04087	-0.04783	-0.05032	-0.03078	0.31819	-0.31819	0.58657	0.10798	0.50925	0.59558	0.53443	0.51335	0.68943	2.22E-05	2.22E-05 blue
SAMD15	0.0158	-0.20848	-0.21042	-0.0316	0.07678	-0.22655	-0.12454	0.22037	-0.22037	0.8375	0.00621	0.00574	0.68155	0.3182	0.00289	0.1046	0.00378	0.00378 turquoise
MOSPD3	0.12318	-0.02244	-0.0088	-0.01965	0.08732	-0.00731	0.122	-0.41546	0.41546	0.10848	0.77076	0.90909	0.79865	0.2561	0.92435	0.11192	1.60E-08	1.60E-08 green
KLHL6	-0.14418	-0.02664	0.09067	0.04336	-0.23466	0.1533	-0.00148	0.40776	-0.40776	0.05992	0.72944	0.23822	0.5734	0.00201	0.04531	0.9847	3.10E-08	3.10E-08 blue
BASP1	-0.12037	-0.07145	0.02401	0.02448	-0.18053	0.1309	0.02991	0.32696	-0.32696	0.11683	0.35309	0.75528	0.75063	0.01813	0.08792	0.69779	1.27E-05	1.27E-05 black
MAD2L1	-0.09433	0.05914	-0.00424	-0.05906	-0.00781	-0.00483	0.2017	-0.35278	0.35278	0.21972	0.44224	0.95609	0.44287	0.91929	0.94996	0.00816	2.22E-06	2.22E-06 pink
BCR	-0.03018	-0.04256	-0.07782	-0.04234	0.00011	-0.1046	0.04956	-0.22922	0.22922	0.69514	0.58044	0.31166	0.5824	0.99882	0.17334	0.51973	0.00256	0.00256 grey
XYLB	0.07563	0.09485	0.10154	0.01687	-0.11206	0.2419	0.26702	-0.29718	0.29718	0.32555	0.21719	0.18635	0.82663	0.14449	0.00143	0.00041	7.91E-05	7.91E-05 grey
TFAP2A	0.06017	0.01819	0.05965	0.00159	-0.04058	0.13805	0.22074	-0.42023	0.42023	0.43433	0.81333	0.43836	0.98349	0.59823	0.07177	0.00372	1.05E-08	1.05E-08 yellow
CALR	0.09454	-0.05958	-0.07559	-0.04182	-0.00275	-0.0785	0.12343	-0.18968	0.18968	0.21873	0.43886	0.32581	0.58703	0.97148	0.30748	0.10776	0.01296	0.01296 turquoise
AFF4	-0.04418	-0.22697	-0.04852	0.02457	-0.08384	0.03416	-0.06117	0.23662	-0.23662	0.56615	0.00283	0.5286	0.74977	0.27562	0.65741	0.42677	0.00183	0.00183 turquoise
ZNF10	-0.04805	-0.18804	-0.15038	-0.10791	0.16017	-0.19731	-0.21701	0.37316	-0.37316	0.53255	0.01378	0.04962	0.16005	0.03638	0.00969	0.00436	4.99E-07	4.99E-07 brown
GAPDH	0.07529	-0.03072	0.02083	-0.00581	0.08096	-0.04336	0.10147	-0.4637	0.4637	0.32769	0.69003	0.78688	0.93989	0.29251	0.57334	0.18666	1.69E-10	1.69E-10 pink
OAZ2	-0.12069	-0.15932	-0.21751	-0.05613	-0.02748	-0.22715	-0.14513	0.45268	-0.45268	0.11584	0.03739	0.00427	0.46593	0.72122	0.00281	0.05823	5.09E-10	5.09E-10 brown
NME1	-0.04115	0.02336	-0.06898	-0.09097	0.02321	-0.15458	0.11134	-0.33188	0.33188	0.59308	0.76165	0.37003	0.23668	0.76312	0.04351	0.1471	9.23E-06	9.23E-06 grey
GPBP1L1	-0.03179	-0.11884	0.02471	0.02782	-0.07484	0.1482	0.06803	-0.04938	0.04938	0.67982	0.12161	0.74837	0.71795	0.33063	0.05307	0.37664	0.52124	0.52124 turquoise
ESPL1	0.02377	0.06039	0.07903	0.01421	0.00689	0.10107	0.23498	-0.42678	0.42678	0.75758	0.43266	0.30421	0.85369	0.92869	0.18839	0.00198	5.86E-09	5.86E-09 pink
KATNAL2	-0.12986	-0.18499	-0.15948	0.05224	0.08175	-0.19648	-0.17619	0.4027	-0.4027	0.09048	0.01543	0.03721	0.4974	0.28782	0.01	0.02116	4.75E-08	4.75E-08 brown
IK	0.04641	-0.17508	-0.0949	-0.0982	-0.0166	-0.03338	0.00238	-0.00754	0.00754	0.54667	0.022	0.21695	0.20134	0.8294	0.66467	0.97532	0.92206	0.92206 turquoise
CPEB2	0.00254	-0.10166	-0.03457	0.07082	0.01544	0.02878	-0.02861	0.20768	-0.20768	0.97366	0.18583	0.65356	0.3573	0.84115	0.70867	0.71025	0.00642	0.00642 turquoise
PRKAB1	0.07186	-0.0466	0.046	-0.01266	0.07242	0.09757	0.00482	-0.23133	0.23133	0.35032	0.54504	0.55022	0.86946	0.34655	0.20426	0.95016	0.00233	0.00233 grey
C12orf57	0.06442	-0.03017	-0.17767	-0.09469	0.19512	-0.20261	-0.19035	0.07266	-0.07266	0.40253	0.69524	0.02008	0.21799	0.01054	0.00787	0.01264	0.34496	0.34496 grey
ZBTB11	-0.01094	-0.14284	-0.04106	-0.01453	-0.11198	0.12299	0.01274	0.19732	-0.19732	0.88704	0.06237	0.59391	0.8504	0.1448	0.10902	0.86863	0.00968	0.00968 turquoise
ARIH2OS	-0.01887	-0.04711	-0.22513	-0.23423	0.1753	-0.18505	-0.13789	0.13683	-0.13683	0.80647	0.54065	0.00307	0.00205	0.02183	0.01539	0.0721	0.07433	0.07433 grey
CAPNS1	0.07247	-0.07928	-0.00392	-0.0087	-0.05276	0.04704	0.10068	-0.34557	0.34557	0.34619	0.30269	0.95946	0.91012	0.49311	0.54121	0.19011	3.67E-06	3.67E-06 grey
ITFG2	0.03599	-0.01082	-0.07294	-0.0481	0.07682	-0.06816	-0.14001	0.1306	-0.1306	0.64028	0.8883	0.34309	0.53215	0.31793	0.37572	0.06779	0.08864	0.08864 turquoise
SLC35B3	-0.02916	0.01625	-0.0124	0.04316	0.09327	0.09542	0.05525	0.02776	-0.02776	0.705	0.83288	0.87217	0.57512	0.22499	0.21446	0.47294	0.71857	0.71857 turquoise
MT2A	-0.11171	-0.00366	-0.01435	0.08576	-0.21222	0.00946	0.06413	0.09503	-0.09503	0.14578	0.9621	0.85223	0.2647	0.00533	0.90226	0.40466	0.21634	0.21634 grey
FIGN	-0.05733	-0.15693	-0.08644	0.06507	-0.14211	-0.0623	-0.01382	0.47245	-0.47245	0.45637	0.04038	0.26096	0.39782	0.06372	0.41825	0.85762	6.85E-11	6.85E-11 turquoise
GET4	0.05039	-0.07978	0.02839	-0.02346	0.1472	0.04965	0.07259	-0.36921	0.36921	0.51275	0.29961	0.71242	0.7607	0.0547	0.51903	0.3454	6.72E-07	6.72E-07 green
DDX46	-0.04588	-0.14212	-0.05659	-0.06472	-0.06615	0.01766	0.01409	0.14061	-0.14061	0.55129	0.06371	0.46225	0.40038	0.39003	0.81867	0.85482	0.06659	0.06659 turquoise
CAPN6	-0.01602	0.01282	-0.05881	0.0436	0.06202	0.16323	-0.07521	0.16485	-0.16485	0.8353	0.86784	0.44484	0.5712	0.4203	0.03291	0.32823	0.03119	0.03119 grey
RABGAP1	-0.04597	-0.11283	-0.04042	0.04358	-0.15226	0.03374	0.06165	0.17073	-0.17073	0.55044	0.14175	0.59967	0.57144	0.0468	0.66128	0.42315	0.02558	0.02558 turquoise
MED14	-0.12339	-0.16471	-0.01498	-0.02072	-0.05292	0.05314	0.0391	0.05288	-0.05288	0.10786	0.03134	0.84582	0.78797	0.49185	0.49005	0.61166	0.49217	0.49217 turquoise
ENTR1	0.00989	0.00014	-0.07555	-0.00932	-0.07147	-0.01675	0.1769	-0.127	0.127	0.89785	0.99857	0.32602	0.90374	0.35293	0.8279	0.02063	0.09788	0.09788 grey
ST5	-0.01523	-0.11432	-0.03529	-0.03504	-0.12659	0.10756	0.09724	0.03391	-0.03391	0.84327	0.13652	0.64681	0.64913	0.09897	0.16142	0.20577	0.65968	0.65968 turquoise
KREMEN2	0.00077	-0.01237	0.04195	-0.02893	0.06484	0.02504	0.04046	-0.38145	0.38145	0.99203	0.87242	0.58587	0.70716	0.39945	0.74508	0.59932	2.64E-07	2.64E-07 grey
E2F6	-0.08084	-0.22728	-0.10559	0.01438	0.01248	-0.04687	-0.02301	0.20523	-0.20523	0.29325	0.00279	0.16931	0.85189	0.87133	0.54269	0.76512	0.00709	0.00709 turquoise
PEX1	-0.05304	-0.15318	-0.03976	0.01827	0.10658	-0.0221	-0.08011	0.07986	-0.07986	0.4908	0.04548	0.60561	0.8125	0.16532	0.77417	0.29764	0.29914	0.29914 turquoise
PPAT	-0.07879	-0.02284	0.05918	0.05326	-0.14893	0.06844	0.17049	0.05331	-0.05331	0.30566	0.76686	0.44194	0.48904	0.05189	0.37374	0.02578	0.48866	0.48866 turquoise
NAALADL1	-0.10295	-0.05552	-0.00061	0.01858	-0.12561	0.06011	-0.06051	0.39649	-0.39649	0.18029	0.47076	0.99372	0.80941	0.10164	0.43481	0.43178	7.95E-08	7.95E-08 blue
TMPRSS5	-0.12778	0.04462	-0.05993	-0.05133	0.20058	-0.07816	0.04868	-0.18804	0.18804	0.0958	0.56226	0.43623	0.50498	0.00853	0.30956	0.52717	0.01378	0.01378 grey
ARL6IP5	-0.06938	-0.08638	0.01569	0.05361	-0.17758	0.14233	0.05643	0.14813	-0.14813	0.36722	0.26127	0.83864	0.48618	0.02015	0.06332	0.46353	0.05318	0.05318 turquoise
PLD1	0.03095	-0.09715	-0.00535	0.07171	-0.04655	0.20763	0.15646	0.01199	-0.01199	0.68782	0.2062	0.94466	0.35128	0.54543	0.00643	0.04099	0.87632	0.87632 grey
PACRG	-0.012	-0.14256	-0.192	-0.04877	0.13233	-0.3116	-0.28323	0.31141	-0.31141	0.87624	0.06287	0.01188	0.52648	0.08447	3.35E-05	0.00017	3.39E-05	3.39E-05 brown
IER5L	0.08586	-0.03833	-0.06143	-0.02038	0.00736	0.0817	0.23291	-0.25516	0.25516	0.26418	0.61867	0.42475	0.79135	0.92393	0.28807	0.00217	0.00076	0.00076 green
SLC16A2	-0.14042	-0.22678	0.01411	0.05782	-0.19092	0.10209	0.02883	0.48216	-0.48216	0.06698	0.00286	0.8547	0.45254	0.01237	0.18394	0.70818	2.44E-11	2.44E-11 blue
SYDE2	0.00552	-0.1624	0.06771	0.10961	-0.00822	0.02319	-0.05808	0.24476	-0.24476	0.94286	0.03382	0.37888	0.15355	0.91504	0.76332	0.45049	0.00125	0.00125 turquoise
RASL11B	-0.07243	-0.08695	-0.04607	0.05893	-0.21211	0.17667	0.05358	0.31369	-0.31369	0.34651	0.25813	0.54958	0.4439	0.00535	0.02081	0.48639	2.94E-05	2.94E-05 blue
HELLS	-0.06955	0.03809	0.03107	-0.01013	-0.03376	0.01674	0.25251	-0.39278	0.39278	0.36604	0.62087	0.68661	0.8954	0.66116	0.82793	0.00086	1.07E-07	1.07E-07 pink
F13A1	-0.10156	-0.01158	0.07515	0.05013	-0.15938	0.16109	0.1129	0.45174	-0.45174	0.18625	0.88052	0.32865	0.51496	0.03732	0.03531	0.14148	5.59E-10	5.59E-10 blue
OSGIN1	0.06763	-0.01277	0.05177	0.05249	0.00126	0.17811	0.1623	-0.36277	0.36277	0.37942	0.86834	0.50127	0.49532	0.98696	0.01977	0.03393	1.08E-06	1.08E-06 yellow
HIST1H4I	0.02043	-0.02418	0.13565	0.125	-0.08808	0.11489	0.21538	-0.33586	0.33586	0.79083	0.75357	0.07689	0.10332	0.25195	0.13457	0.00467	7.09E-06	7.09E-06 grey
TMEM59L	0.07561	-0.1815	-0.2262	-0.14798	0.0638	-0.40795	-0.19936	0.39717	-0.39717	0.32567	0.01751	0.00293	0.05342	0.4071	3.05E-08	0.00895	7.51E-08	7.51E-08 brown
HIST1H4H	-0.05248	-0.0385	0.08143	0.15474	-0.08671	0.14056	0.16043	-0.17427	0.17427	0.49545	0.61707	0.28971	0.0433	0.25943	0.0667	0.03608	0.02263	0.02263 grey
PM20D2	-0.04664	-0.10321	-0.12759	0.02386	0.00574	-0.09915	-0.10292	0.26876	-0.26876	0.54471	0.17916	0.09632	0.75674	0.94065	0.19697	0.18038	0.00038	0.00038 turquoise
MED4	-0.06261	-0.04034	-0.07387	-0.04558	0.01801	-0.0886	-0.03701	0.01897	-0.01897	0.41593	0.6004	0.33693	0.55391	0.81518	0.24915	0.63081	0.80548	0.80548 turquoise
GPHA2	-0.08448	0.07535	0.05539	0.12671	-0.16762	0.03222	-0.13243	0.21792	-0.21792	0.27195	0.32736	0.47176	0.09866	0.02842	0.6757	0.08423	0.00419	0.00419 magenta
CDKN1B	0.0024	-0.09066	-0.0548	0.00216	-0.00611	-0.03651	-0.00952	0.215	-0.215	0.9751	0.23827	0.47654	0.97761	0.93681	0.63543	0.90165	0.00474	0.00474 turquoise
KCTD21	-0.03235	-0.19412	0.02267	0.05755	-0.17746	0.05046	0.06828	0.02636	-0.02636	0.67444	0.01096	0.76847	0.45469	0.02023	0.51216	0.37491	0.73213	0.73213 turquoise
CCDC71L	-0.07476	-0.14779	0.08151	0.08666	-0.18657	0.1226	0.00033	0.1969	-0.1969	0.33116	0.05373	0.28922	0.25973	0.01455	0.11014	0.99658	0.00984	0.00984 blue
POGZ	-0.00018	-0.18758	-0.11859	-0.07667	0.06929	-0.01913	-0.04132	0.01911	-0.01911	0.99815	0.01402	0.12238	0.31889	0.36786	0.80383	0.59157	0.80408	0.80408 turquoise
YWHAQ	-0.07732	-0.11533	-0.0542	0.0132	-0.13962	-0.04454	0.02451	0.16997	-0.16997	0.3148	0.13307	0.48136	0.86392	0.06856	0.56294	0.75032	0.02624	0.02624 turquoise
FCGBP	-0.02345	0.0897	0.00334	0.05955	0.15532	0.06549	0.07677	-0.02157	0.02157	0.76084	0.24333	0.96543	0.43912	0.0425	0.39472	0.31828	0.77949	0.77949 grey
ZP3	0.12405	0.0769	0.04764	0.06754	-0.04232	0.03344	0.15597	-0.40362	0.40362	0.10599	0.31744	0.5361	0.38007	0.58261	0.66416	0.04164	4.40E-08	4.40E-08 grey
SCARF2	-0.04057	-0.07044	0.00639	0.06036	-0.11141	0.12758	0.08482	0.15271	-0.15271	0.59828	0.3599	0.93392	0.43286	0.14688	0.09633	0.27004	0.04615	0.04615 black
ZNF136	-0.06453	-0.12312	-0.14136	-0.0127	-0.00433	-0.06215	-0.16525	0.35504	-0.35504	0.40171	0.10864	0.06514	0.86903	0.95523	0.41933	0.03078	1.89E-06	1.89E-06 turquoise
ZNF526	0.00136	-0.11042	-0.11694	-0.02818	-0.03012	0.00816	0.12384	-0.09466	0.09466	0.9859	0.15051	0.1277	0.71449	0.6957	0.91564	0.10657	0.21812	0.21812 turquoise
KIT	-0.04135	-0.16769	0.01828	0.1046	-0.14304	0.15578	-0.09492	0.39038	-0.39038	0.59127	0.02836	0.81243	0.17336	0.06199	0.0419	0.21687	1.30E-07	1.30E-07 blue
TMEM9	0.05405	-0.08927	-0.17972	-0.03221	0.08772	-0.25097	-0.03996	-0.12885	0.12885	0.48259	0.24559	0.01867	0.67583	0.25391	0.00093	0.60386	0.09304	0.09304 grey
CCNT2	-0.00875	-0.13239	-0.02171	0.06954	0.02034	0.01882	-0.03014	0.02852	-0.02852	0.90958	0.08432	0.77802	0.36614	0.79178	0.807	0.69555	0.71112	0.71112 red
REV1	-0.02072	-0.13673	-0.04994	-0.01514	-0.04996	0.05741	0.03055	0.14456	-0.14456	0.78792	0.07456	0.5166	0.84422	0.51637	0.45575	0.6916	0.05924	0.05924 red
ERN1	-0.07924	-0.17878	0.00475	0.04258	-0.01286	-0.05063	-0.22045	0.36946	-0.36946	0.30293	0.0193	0.95088	0.58024	0.86744	0.51079	0.00376	6.59E-07	6.59E-07 turquoise
TNFRSF11	0.05201	-0.0953	0.00931	0.07484	0.0354	0.12733	0.04995	-0.02013	0.02013	0.4993	0.21501	0.90384	0.33064	0.64578	0.09701	0.51649	0.79383	0.79383 grey
FABP6	0.18923	0.06628	-0.06462	0.00909	0.0762	0.07	0.1071	-0.31543	0.31543	0.01318	0.38907	0.40107	0.90605	0.32191	0.36295	0.16324	2.64E-05	2.64E-05 grey
GPX7	-0.04898	-0.07556	-0.01098	0.07895	-0.09805	-0.08175	-0.07056	0.33328	-0.33328	0.52467	0.32601	0.88664	0.30466	0.20202	0.2878	0.35909	8.42E-06	8.42E-06 blue
HNRNPF	0.01878	-0.0209	0.10133	0.03798	-0.13636	0.22987	0.19528	-0.23587	0.23587	0.80737	0.78617	0.18724	0.6219	0.07534	0.00249	0.01048	0.0019	0.0019 turquoise
CCDC6	0.03668	-0.10786	0.04004	0.08223	-0.18761	0.11609	0.1357	-0.16091	0.16091	0.63382	0.16027	0.60309	0.28497	0.014	0.13053	0.07678	0.03551	0.03551 turquoise
GOLGA4	0.03887	-0.13698	0.00574	-0.06224	-0.03871	0.0919	0.03549	0.09	-0.09	0.61375	0.07401	0.9406	0.41871	0.61521	0.23189	0.64492	0.24173	0.24173 turquoise
SLC25A34	-0.09402	-0.13441	-0.20178	-0.02717	0.1254	-0.18204	-0.06998	0.42638	-0.42638	0.22127	0.07965	0.00813	0.72428	0.1022	0.01718	0.3631	6.07E-09	6.07E-09 brown
ATP6V1G1	-0.02273	-0.14368	-0.11771	-0.05564	0.00998	-0.15576	-0.1564	0.11647	-0.11647	0.76789	0.06082	0.12521	0.46983	0.89695	0.04191	0.04108	0.12926	0.12926 turquoise
MRPL35	0.01	-0.00382	-0.06616	-0.04793	0.03494	-0.03524	0.06119	0.00682	-0.00682	0.89669	0.96043	0.3899	0.53357	0.65003	0.64727	0.42661	0.92943	0.92943 turquoise
RIBC1	-0.08128	-0.18842	-0.18752	-0.0227	0.16872	-0.20194	-0.19475	0.06089	-0.06089	0.29056	0.01359	0.01405	0.76821	0.02739	0.00808	0.0107	0.42886	0.42886 grey
CTPS2	-0.09674	-0.13166	-0.09199	-0.00729	0.00012	0.00557	0.04504	-0.18279	0.18279	0.20813	0.08606	0.23145	0.92461	0.99878	0.94238	0.55862	0.01671	0.01671 turquoise
FAHD1	0.00923	0.03087	0.00376	-0.03274	-0.04046	0.0994	0.10415	-0.25931	0.25931	0.90465	0.68855	0.9611	0.67072	0.59929	0.19582	0.1752	0.00062	0.00062 grey
UROD	-0.0527	-0.05361	-0.0679	-0.01208	-0.04672	-0.09484	0.01143	0.16505	-0.16505	0.49366	0.48616	0.37758	0.8754	0.54395	0.21726	0.88206	0.03098	0.03098 turquoise
CORO2B	-0.07505	-0.19585	-0.29024	-0.12978	-0.04997	-0.14309	-0.188	0.57719	-0.57719	0.32925	0.01025	0.00012	0.09069	0.51628	0.06189	0.0138	1.42E-16	1.42E-16 brown
SEMA3E	0.05668	-0.17792	-0.16767	-0.00654	-0.0146	0.01461	0.01808	0.25891	-0.25891	0.46152	0.0199	0.02838	0.93233	0.8497	0.84958	0.81446	0.00063	0.00063 grey
NPEPPS	-0.05266	-0.23246	-0.03292	0.04569	0.01163	-0.00015	0.05765	-0.05134	0.05134	0.49398	0.00222	0.66907	0.55288	0.88	0.99843	0.45384	0.50488	0.50488 turquoise
ZDHHC1	0.07707	-0.10063	-0.18071	0.00154	0.22083	-0.11825	-0.09365	0.00432	-0.00432	0.3164	0.19033	0.01802	0.98403	0.0037	0.12346	0.22309	0.95528	0.95528 grey
AKAP10	-0.06673	-0.14796	-0.0385	0.00057	-0.08337	-0.05193	-0.04232	0.28483	-0.28483	0.38585	0.05345	0.61712	0.99414	0.27831	0.5	0.58255	0.00016	0.00016 turquoise
AGAP2	-0.09015	-0.05971	-0.09653	-0.0118	-0.06841	-0.08471	-0.06612	0.33489	-0.33489	0.24094	0.43792	0.20914	0.8783	0.374	0.27064	0.39018	7.57E-06	7.57E-06 blue
KCNC3	-0.04332	-0.26713	-0.17986	0.00639	0.072	-0.24416	-0.15442	0.23141	-0.23141	0.57375	0.00041	0.01858	0.93388	0.34939	0.00129	0.04374	0.00232	0.00232 grey
CCDC174	0.0699	-0.07294	-0.02166	-0.09377	0.02958	0.1568	0.06113	-0.18473	0.18473	0.36363	0.34307	0.77857	0.22248	0.70098	0.04055	0.42703	0.01557	0.01557 turquoise
CAPZA2	-0.05592	-0.10909	0.0367	0.05634	-0.02565	0.04176	0.00696	0.10485	-0.10485	0.46759	0.15551	0.63369	0.46421	0.73913	0.58758	0.92797	0.17233	0.17233 turquoise
BFSP1	0.05052	-0.00403	0.01174	0.0253	0.00835	-0.02443	0.03845	-0.15384	0.15384	0.51169	0.95826	0.87886	0.74254	0.91365	0.75111	0.61757	0.04454	0.04454 grey
ANKRD9	0.11894	-0.05438	0.1067	0.09366	0.09625	0.12981	0.11564	-0.33007	0.33007	0.12128	0.47989	0.16482	0.22307	0.21047	0.0906	0.13203	1.04E-05	1.04E-05 grey
COL15A1	-0.12538	-0.05711	-0.05036	0.04627	-0.2321	0.12543	0.00456	0.40334	-0.40334	0.10226	0.45816	0.51307	0.54789	0.00225	0.10211	0.95276	4.51E-08	4.51E-08 blue

EPM2A	-0.06715	-0.04574	-0.10077	-0.0119	0.01377	-0.15974	-0.19292	0.55593	-0.55593	0.38283	0.55251	0.18974	0.87728	0.85809	0.03689	0.01147	2.95E-15	2.95E-15	brown
HSPA9	-0.02647	-0.13548	-0.08792	-0.06084	-0.00259	-0.09547	-0.0098	0.026	-0.026	0.73107	0.07727	0.25286	0.42924	0.97313	0.21422	0.89878	0.73566	0.73566	turquoise
DONSON	-0.00507	-0.14272	-0.09025	-0.0723	0.142	-0.05623	0.11633	-0.07274	0.07274	0.94754	0.06258	0.24043	0.34736	0.06392	0.46511	0.12974	0.34441	0.34441	turquoise
RECK	-0.09139	-0.11782	-0.00869	0.06952	-0.18236	0.04996	-0.07038	0.62058	-0.62058	0.23449	0.12483	0.91022	0.36624	0.01698	0.5164	0.36036	1.39E-19	1.39E-19	blue
AVPR1A	-0.22932	-0.17015	-0.05615	0.12083	-0.14654	0.07802	-0.05107	0.45271	-0.45271	0.00255	0.02608	0.46572	0.11543	0.0558	0.31045	0.50712	5.08E-10	5.08E-10	blue
SELENOS	0.01615	-0.11738	-0.01366	-0.00336	0.02079	0.02512	0.01527	-0.20761	0.20761	0.83392	0.12626	0.8592	0.96525	0.78723	0.74428	0.84284	0.00644	0.00644	grey
GLO1	-0.0895	-0.05267	-0.00173	0.02969	-0.08074	0.06446	0.08743	0.02121	-0.02121	0.2444	0.49383	0.98207	0.69986	0.29383	0.40225	0.2555	0.78308	0.78308	turquoise
GRIN2D	0.0609	-0.03904	0.07925	0.07943	-0.20105	0.08171	0.14563	-0.11437	0.11437	0.42881	0.61221	0.30283	0.30173	0.00837	0.28802	0.05737	0.13636	0.13636	grey
TSKU	-0.00692	-0.03944	0.0871	0.09497	-0.19354	0.24015	0.27393	-0.12248	0.12248	0.92839	0.60851	0.2573	0.21662	0.0112	0.00156	0.00029	0.11051	0.11051	black
ITGAE	-0.0435	-0.09216	-0.03034	0.04163	-0.05131	-0.15642	0.00992	0.29767	-0.29767	0.57212	0.23057	0.69363	0.58876	0.50511	0.04105	0.89755	7.68E-05	7.68E-05	blue
SHE	-0.10964	-0.10181	-0.0998	0.0397	-0.10637	0.00436	-0.11083	0.62256	-0.62256	0.15343	0.18517	0.19404	0.60616	0.16615	0.95491	0.149	9.88E-20	9.88E-20	blue
MRPL39	-0.05581	0.02329	-0.02304	-0.04953	0.04571	0.01063	0.0022	0.02057	-0.02057	0.46842	0.76234	0.76488	0.51997	0.55272	0.8902	0.9772	0.78944	0.78944	turquoise
DTX3	0.01878	-0.15682	-0.20845	-0.05248	0.11962	-0.29237	-0.24247	0.31664	-0.31664	0.80735	0.04053	0.00622	0.49545	0.11913	0.0001	0.0014	2.45E-05	2.45E-05	brown
TMEM186	0.06839	0.01983	-0.12764	-0.10387	0.0322	-0.05466	0.15275	-0.17385	0.17385	0.37414	0.79687	0.09618	0.17638	0.67587	0.47765	0.0461	0.02296	0.02296	turquoise
SFMBT2	-0.09637	-0.04345	-0.07905	0.02787	-0.12588	-0.0402	-0.08022	0.54842	-0.54842	0.2099	0.57258	0.30407	0.71751	0.1009	0.60164	0.29694	8.19E-15	8.19E-15	blue
PCDHB4	0.00373	-0.08463	-0.07072	-0.00236	0.03196	-0.1914	-0.18634	0.51835	-0.51835	0.96143	0.27111	0.35801	0.97553	0.67813	0.01215	0.01468	3.82E-13	3.82E-13	brown
ZNF846	-0.03511	-0.14673	-0.10456	-0.03038	0.08635	-0.10233	-0.04479	0.06992	-0.06992	0.64843	0.05549	0.1735	0.69321	0.26144	0.18291	0.56075	0.36347	0.36347	red
PF4V1	-0.06295	-0.13159	-0.04796	-0.04654	-0.04129	0.0286	0.07327	-0.01531	0.01531	0.41335	0.08622	0.53333	0.54554	0.59184	0.71041	0.34089	0.84243	0.84243	grey
SOD1	0.02618	-0.0896	-0.07301	-0.04561	0.1819	-0.07775	0.03533	-0.18176	0.18176	0.73393	0.24387	0.34262	0.55357	0.01726	0.3121	0.64644	0.01735	0.01735	grey
ZCCHC10	-0.03738	-0.17793	-0.08169	-0.00652	0.04921	-0.02235	-0.12798	0.13442	-0.13442	0.6274	0.01989	0.28816	0.93255	0.52273	0.77173	0.09527	0.07962	0.07962	turquoise
KRT10	-0.03243	-0.14154	-0.13201	-0.06068	0.19936	-0.31166	-0.13113	0.04843	-0.04843	0.67366	0.06481	0.08522	0.43047	0.00895	3.33E-05	0.08734	0.52931	0.52931	grey
MANF	0.05676	-0.04052	-0.09629	-0.0337	-0.07536	-0.03052	0.12687	-0.2573	0.2573	0.46087	0.59872	0.21028	0.66174	0.3273	0.6919	0.09822	0.00068	0.00068	grey
SPP1	0.00694	-0.03445	-0.02334	0.12059	-0.12756	0.17136	0.07077	-0.02698	0.02698	0.9282	0.65461	0.76189	0.11615	0.09639	0.02502	0.35767	0.72616	0.72616	grey
SCG5	0.00295	-0.1349	-0.24804	-0.16413	0.04225	-0.30089	-0.21334	0.41245	-0.41245	0.96942	0.07855	0.00107	0.03194	0.58324	6.36E-05	0.00508	2.08E-08	2.08E-08	brown
MYEF2	-0.07318	-0.21364	-0.23144	-0.08052	0.05228	-0.22609	-0.20853	0.34319	-0.34319	0.34149	0.00502	0.00232	0.29514	0.49705	0.00294	0.0062	4.32E-06	4.32E-06	brown
RDH10	0.04403	0.05101	0.10261	0.01141	-0.01574	0.18593	0.01746	-0.02127	0.02127	0.5674	0.50761	0.18172	0.88228	0.83807	0.0149	0.82073	0.78247	0.78247	turquoise
AGO3	-0.06846	-0.27628	-0.06484	0.02584	-0.08929	-0.03882	-0.06468	0.40048	-0.40048	0.37362	0.00025	0.39951	0.73729	0.24549	0.61421	0.40065	5.72E-08	5.72E-08	turquoise
SCGN	0.009	-0.0312	-0.25953	-0.12979	0.09402	-0.22349	-0.15909	0.36208	-0.36208	0.90701	0.68545	0.00061	0.09066	0.22128	0.0033	0.03768	1.14E-06	1.14E-06	brown
XPOT	0.01554	-0.11355	-0.02325	0.03766	-0.05937	-0.03872	0.02671	0.09913	-0.09913	0.84008	0.13922	0.76282	0.6248	0.44048	0.61509	0.72875	0.19704	0.19704	turquoise
SRSF11	-0.09126	-0.18135	-0.00889	0.00679	0.0621	0.01855	-0.01277	0.08667	-0.08667	0.23521	0.01761	0.90809	0.92978	0.4197	0.80966	0.86834	0.25966	0.25966	red
HNRNPH3	0.02854	-0.19398	-0.03738	0.01059	-0.00605	-0.01857	-0.03246	0.12625	-0.12625	0.71101	0.01101	0.62742	0.89069	0.93737	0.80948	0.67343	0.09989	0.09989	turquoise
TTC9C	-0.011	-0.12305	0.00164	0.03164	-0.08635	0.06888	0.18077	-0.18584	0.18584	0.88642	0.10886	0.98298	0.68119	0.26141	0.37073	0.01798	0.01495	0.01495	turquoise
DAPP1	-0.11915	-0.0384	0.16832	0.09028	-0.16023	0.26245	0.19469	-0.1071	0.1071	0.1206	0.61807	0.02776	0.24029	0.03631	0.00052	0.01072	0.16324	0.16324	turquoise
RRP36	-0.0092	-0.00749	-0.04886	-0.03976	-0.11806	-0.07451	0.12522	-0.03198	0.03198	0.90489	0.92259	0.52571	0.60559	0.12406	0.33275	0.1027	0.67796	0.67796	grey
UGT8	-0.06308	-0.00282	0.08003	-0.0033	0.05631	0.08019	0.02668	-0.14384	0.14384	0.41245	0.97084	0.29812	0.96581	0.46445	0.29712	0.72907	0.06053	0.06053	grey
TPSG1	0.06831	-0.01075	-0.12334	-0.03557	0.25401	-0.15413	-0.18865	0.04592	-0.04592	0.37467	0.88903	0.10802	0.64421	0.0008	0.04414	0.01347	0.55094	0.55094	grey
NKAIN4	0.02183	-0.0386	0.06479	0.05838	-0.02995	0.04597	0.10385	-0.13964	0.13964	0.77688	0.61623	0.39985	0.44815	0.69737	0.55046	0.17646	0.06852	0.06852	grey
PCSK1	-0.03572	-0.08326	-0.26794	-0.1333	0.07797	-0.17802	-0.14064	0.42152	-0.42152	0.64277	0.27895	0.0004	0.08219	0.31078	0.01983	0.06655	9.39E-09	9.39E-09	brown
AQP6	0.09358	-0.02519	0.10078	0.06035	0.05451	0.06187	0.1344	-0.17385	0.17385	0.22343	0.74367	0.18969	0.433	0.47887	0.42149	0.07967	0.02296	0.02296	grey
PROSER1	0.07767	-0.05241	0.01712	-0.00854	-0.10404	0.09973	0.0379	-0.05982	0.05982	0.31264	0.496	0.8241	0.91172	0.17566	0.19435	0.62263	0.43707	0.43707	turquoise
VPS18	-0.06608	-0.17727	-0.13738	-0.06165	-0.08574	-0.08902	0.02454	0.1331	-0.1331	0.39051	0.02036	0.07317	0.4231	0.26484	0.24691	0.75	0.08266	0.08266	grey
CAMK1	-0.12426	-0.17199	-0.08336	0.02649	-0.0269	0.01389	-0.06479	0.51948	-0.51948	0.10538	0.02449	0.27835	0.73092	0.72687	0.85686	0.39982	3.33E-13	3.33E-13	blue
STK25	0.06811	-0.04748	-0.07244	-0.00658	0.10296	-0.0914	0.02649	-0.12167	0.12167	0.3761	0.53746	0.34643	0.93189	0.18022	0.23446	0.73092	0.11289	0.11289	red
RDH13	0.18479	0.0389	-0.01133	-0.00938	0.17286	0.08348	0.16385	-0.43086	0.43086	0.01554	0.61346	0.88307	0.90313	0.02377	0.27766	0.03224	4.04E-09	4.04E-09	yellow
WDYHV1	0.07968	-0.05167	-0.09669	-0.04578	-0.08776	-0.1416	0.08069	-0.07407	0.07407	0.30022	0.50214	0.20839	0.55214	0.2537	0.06469	0.29414	0.33563	0.33563	grey
NUDT2	-0.06762	-0.02944	-0.16577	-0.14164	0.07712	-0.09973	0.02351	-0.06873	0.06873	0.37953	0.70228	0.03025	0.06462	0.3161	0.19436	0.76019	0.37171	0.37171	grey
MAP4K1	-0.08953	-0.01883	0.03668	0.03666	-0.1439	0.10041	-0.00696	0.38028	-0.38028	0.24421	0.80685	0.63387	0.63401	0.06042	0.1913	0.92796	2.89E-07	2.89E-07	blue
TPR	-0.02846	-0.10794	0.01625	-0.03487	-0.0835	0.15321	0.11795	0.01703	-0.01703	0.71178	0.15996	0.83289	0.65067	0.27756	0.04544	0.12442	0.82506	0.82506	turquoise
GYS1	-0.01204	-0.01773	-0.00508	0.05438	-0.0623	0.08087	0.12903	-0.23803	0.23803	0.87584	0.818	0.94747	0.47991	0.41824	0.29302	0.09259	0.00172	0.00172	turquoise
GFI1	-0.03982	-0.02672	0.12285	0.10948	-0.14646	0.1308	0.09371	0.13961	-0.13961	0.6051	0.72865	0.10943	0.15402	0.05594	0.08816	0.22278	0.06857	0.06857	grey
PTGER2	-0.02187	-0.06553	0.10207	-0.00544	-0.15857	0.24136	0.0873	0.09296	-0.09296	0.77649	0.39445	0.18406	0.94366	0.03831	0.00147	0.25618	0.22652	0.22652	turquoise
MCF2L	0.05193	-0.11056	-0.2149	-0.06209	0.15867	-0.18501	-0.2422	0.29541	-0.29541	0.49996	0.15	0.00476	0.41977	0.03819	0.01541	0.00142	8.76E-05	8.76E-05	brown
EIF4E2	0.01354	-0.01129	-0.02421	0.0162	-0.03658	0.10757	0.22245	-0.22566	0.22566	0.86049	0.88352	0.75329	0.8334	0.63482	0.16137	0.00345	0.003	0.003	turquoise
RANBP17	-0.04466	-0.27539	-0.07931	0.11726	0.03456	-0.08396	-0.01852	0.05938	-0.05938	0.5619	0.00027	0.3025	0.12666	0.65359	0.27495	0.80997	0.44044	0.44044	grey
KRBOX1	-0.00967	-0.0306	0.06408	0.00048	-0.12774	0.0012	0.05477	-0.12061	0.12061	0.90009	0.69118	0.40501	0.99506	0.09591	0.98752	0.47679	0.11609	0.11609	grey
CPLX1	-0.05438	-0.12606	-0.21899	-0.09255	0.22562	-0.33369	-0.16461	0.06866	-0.06866	0.4799	0.10039	0.00401	0.22862	0.00301	8.19E-06	0.03144	0.37224	0.37224	grey
VEPH1	0.04241	-0.01909	-0.19152	0.05731	-0.13403	-0.01466	0.10043	0.13541	-0.13541	0.58178	0.8043	0.01209	0.45654	0.08051	0.84903	0.19121	0.07742	0.07742	magenta
PRXL2B	0.00436	-0.00245	0.05916	0.05586	0.09713	0.15943	0.17104	-0.4265	0.4265	0.95483	0.97467	0.4421	0.46806	0.2063	0.03726	0.0253	6.01E-09	6.01E-09	yellow
CHSY1	-0.13948	-0.14406	0.06564	0.10238	-0.21893	0.13194	0.03243	0.343	-0.343	0.06885	0.06012	0.39366	0.18271	0.00402	0.08539	0.67373	4.38E-06	4.38E-06	blue
SEMA6D	-0.14461	-0.21585	-0.0961	0.00645	-0.00134	-0.16618	-0.16178	0.55956	-0.55956	0.05915	0.00458	0.21117	0.93328	0.98607	0.02983	0.03451	1.78E-15	1.78E-15	turquoise
FIGNL1	-0.10627	-0.03576	0.02423	-0.01959	-0.05379	0.05421	0.12264	-0.00678	0.00678	0.16656	0.64237	0.7531	0.79921	0.48469	0.48127	0.11003	0.92984	0.92984	turquoise
CD2	-0.10345	-0.02561	0.05148	0.09548	-0.19066	0.15906	0.03709	0.35456	-0.35456	0.17813	0.73949	0.50371	0.21416	0.01249	0.03771	0.63007	1.96E-06	1.96E-06	blue
RALYL	0.03825	-0.05715	-0.31153	-0.12876	0.17979	-0.19488	-0.06274	0.29985	-0.29985	0.61942	0.45782	3.36E-05	0.09327	0.01862	0.01064	0.41498	6.76E-05	6.76E-05	grey
HECW2	-0.12611	-0.13882	-0.13132	-0.01649	-0.04139	-0.15205	-0.16733	0.54412	-0.54412	0.10026	0.07017	0.08688	0.83051	0.59092	0.04711	0.0287	1.45E-14	1.45E-14	turquoise
BBS4	-0.07454	-0.1891	-0.10971	-0.04492	0.08902	-0.05764	-0.07771	0.24387	-0.24387	0.33257	0.01325	0.15318	0.55965	0.24694	0.45396	0.31239	0.00131	0.00131	turquoise
NT5C	0.10216	0.05029	-0.01317	-0.02261	0.15767	-0.0023	0.17841	-0.56663	0.56663	0.18364	0.51359	0.86422	0.76909	0.03944	0.9762	0.01956	6.58E-16	6.58E-16	green
KCNQ4	-0.0043	0.08136	0.04268	0.08786	0.01598	0.12416	0.10127	-0.21754	0.21754	0.9555	0.29014	0.57944	0.25316	0.83565	0.10567	0.18754	0.00426	0.00426	grey
ST8SIA4	-0.11511	-0.11718	0.00701	0.07608	-0.19252	0.0601	-0.03026	0.50574	-0.50574	0.13381	0.12693	0.9275	0.32267	0.01165	0.43486	0.6944	1.72E-12	1.72E-12	blue
MKRN2	-0.03338	-0.13913	0.03413	-0.04612	-0.15311	0.15897	0.04314	0.11307	-0.11307	0.66474	0.06955	0.65761	0.54917	0.04558	0.03782	0.57527	0.1409	0.1409	turquoise
PRMT2	-0.02697	-0.1656	-0.11252	-0.01582	0.10349	-0.12902	-0.11738	0.27788	-0.27788	0.72623	0.03042	0.14284	0.83727	0.17796	0.09262	0.12627	0.00023	0.00023	turquoise
ANKRD13	0.02316	-0.0946	-0.09211	-0.07323	0.07046	-0.14703	0.07539	-0.1863	0.1863	0.76363	0.21844	0.23084	0.34116	0.35982	0.05499	0.32709	0.0147	0.0147	red
MYCN	0.08562	0.00016	-0.00926	-0.06432	0.11507	-0.0812	-0.08311	0.03068	-0.03068	0.26554	0.99839	0.90433	0.4033	0.13396	0.29107	0.27981	0.69038	0.69038	grey
POP1	-0.11698	-0.07008	-0.10633	-0.02982	-0.11381	-0.06541	0.13102	-0.01006	0.01006	0.12759	0.36237	0.16629	0.69865	0.1383	0.39531	0.08761	0.89615	0.89615	turquoise
PHLDB3	0.14176	-0.13346	-0.06534	-0.0962	0.19621	-0.18299	-0.07215	0.02413	-0.02413	0.06438	0.08183	0.39586	0.21071	0.01011	0.01659	0.34832	0.75404	0.75404	grey
PHKB	-0.0184	-0.07603	-0.02296	-0.00404	-0.00803	0.08487	0.04297	0.04403	-0.04403	0.81117	0.32298	0.7656	0.95816	0.91693	0.26974	0.57685	0.56743	0.56743	turquoise
FGR	-0.11734	-0.02732	0.06423	0.13128	-0.155	0.10489	0.02217	0.34161	-0.34161	0.12641	0.72284	0.40394	0.08698	0.04295	0.17216	0.77347	4.82E-06	4.82E-06	blue
MAP3K7C	-0.05268	-0.08972	-0.0818	-0.05885	0.02797	-0.0846	-0.17122	0.37111	-0.37111	0.49375	0.24321	0.28748	0.44456	0.71648	0.27128	0.02515	5.83E-07	5.83E-07	turquoise
MYO7A	-0.12435	-0.12995	0.13394	0.15279	-0.02866	0.07327	0.03252	0.02232	-0.02232	0.10513	0.09025	0.08073	0.04604	0.70983	0.34092	0.67283	0.77197	0.77197	grey
VWF	-0.03367	-0.0356	0.00817	0.00349	-0.1215	0.01402	-0.11475	0.44414	-0.44414	0.66193	0.64386	0.91555	0.96387	0.11341	0.85561	0.13505	1.17E-09	1.17E-09	blue
SDF2	-0.02174	-0.12218	-0.11641	-0.0319	0.04968	-0.1028	-0.01637	-0.07208	0.07208	0.77775	0.1114	0.12947	0.67877	0.51876	0.18091	0.83169	0.34882	0.34882	grey
RASL11A	0.01432	-0.17055	0.01087	0.06824	0.03822	0.06236	-0.08094	0.18903	-0.18903	0.85253	0.02573	0.88774	0.37517	0.61969	0.41782	0.29262	0.01328	0.01328	blue
ST8SIA2	0.01234	-0.0049	0.07921	0.0559	-0.09342	0.07864	0.16646	0.04132	-0.04132	0.8727	0.94929	0.3031	0.46768	0.22425	0.3066	0.02956	0.59159	0.59159	black
UGT1A10	0.05903	-0.05905	0.11591	0.07411	0.01673	0.21929	0.22614	-0.34674	0.34674	0.44315	0.44299	0.13113	0.33539	0.82804	0.00396	0.00294	3.39E-06	3.39E-06	yellow
CDC7	-0.18086	-0.00222	-0.03954	-0.02492	-0.08024	-0.03864	0.18572	0.02279	-0.02279	0.01792	0.97701	0.60764	0.74629	0.29684	0.6158	0.01502	0.76735	0.76735	pink
SNIP1	-0.0987	-0.22584	-0.03204	0.00782	-0.06601	0.0267	-0.06416	0.16743	-0.16743	0.19901	0.00298	0.67739	0.9191	0.39101	0.72886	0.40446	0.02861	0.02861	turquoise
PDZK1	0.02658	-0.15476	-0.25843	-0.06662	0.16861	-0.21894	-0.13224	0.29714	-0.29714	0.73003	0.04327	0.00064	0.38665	0.02749	0.00402	0.08467	7.93E-05	7.93E-05	brown
CXorf56	0.01205	-0.19897	-0.14218	-0.04604	-0.00999	-0.04739	0.03497	-0.002	0.002	0.87567	0.00908	0.06359	0.54989	0.89678	0.53818	0.64979	0.9793	0.9793	turquoise
MAP4K3	-0.01785	-0.16374	0.02992	0.04805	-0.03354	0.1467	0.05729	0.00458	-0.00458	0.81678	0.03236	0.69764	0.53261	0.66318	0.05554	0.45673	0.95262	0.95262	turquoise
RAD51C	0.02689	-0.17433	-0.15116	-0.08333	-0.00975	-0.22948	-0.08374	0.26072	-0.26072	0.72703	0.02258	0.04844	0.27855	0.89926	0.00253	0.2762	0.00057	0.00057	turquoise
USP14	-0.0813	-0.22357	0.00871	0.00223	-0.02426	-0.00603	0.02479	0.0667	-0.0667	0.29047	0.00329	0.90998	0.97694	0.75283	0.93757	0.74761	0.38603	0.38603	turquoise
VSTM4	-0.03011	-0.1022	-0.01231	0.0608	-0.18555	0.02769	-0.02046	0.45178	-0.45178	0.69587	0.18346	0.873	0.42953	0.01511	0.71918	0.79057	5.56E-10	5.56E-10	blue
PARP8	0.01823	-0.09578	0.01243	0.0131	-0.05707	0.11258	-0.01159	0.09357	-0.09357	0.8129	0.21271	0.87179	0.86494	0.4584	0.14263	0.88043	0.2235	0.2235	turquoise
PPP2R3B	0.01393	-0.11539	-0.12752	-0.10755	0.19601	-0.25547	-0.01626	0.0114	-0.0114	0.85652	0.13286	0.09649	0.16149	0.01019	0.00075	0.83279	0.88236	0.88236	red
TRIM52	0.03545	-0.17476	-0.15643	-0.07001	0.12806	-0.13662	-0.06989	0.06879	-0.06879	0.6453	0.02225	0.04103	0.36288	0.09507	0.07479	0.36372	0.37135	0.37135	red
PALM	-0.00501	-0.08157	-0.06769	0.04946	-0.021	-0.08237	-0.12703	0.47963	-0.47963	0.9481	0.28887	0.37903	0.52058	0.78511	0.28413	0.0978	3.20E-11	3.20E-11	blue
EBP	0.10022	0.04545	-0.02347	-0.07408	0.08002	0.01211	0.14948	-0.64014	0.64014	0.19216	0.55497	0.76061	0.33558	0.29819	0.87508	0.05101	4.25E-21	4.25E-21	pink
HERC3	-0.06788	-0.13944	-0.01575	0.00304	-0.09729	0.0812	0.00446	0.09948	-0.09948	0.37771	0.06891	0.83797	0.96852	0.20555	0.29104	0.95379	0.1955	0.1955	turquoise
BLM	-0.07066	0.09598	0.03049	-0.02857	-0.08802	0.02228	0.17172	-0.16257	0.16257	0.35843	0.21173	0.69224	0.71067	0.25231	0.77236	0.02472	0.03363	0.03363	pink
NOM1	-0.04097	-0.0952	0.05204	0.01696	-0.09491	0.02921	0.09254	0.00064	-0.00064	0.59472	0.2155	0.49906	0.82577	0.2169	0.70451	0.22868	0.99333	0.99333	turquoise
EED	0.04932	-0.04956	-0.00579	0.01126	-0.02525	0.0415	0.114	-0.1168	0.1168	0.52179	0.51979	0.94008	0.88376	0.74301	0.58989	0.13765	0.12815	0.12815	turquoise
NLRC4	-0.13122	-0.03072	0.0584	0.05257	-0.17147	0.10077	0.03663	0.41729	-0.41729	0.08714	0.68997	0.44802	0.49467	0.02494	0.1897	0.63436	1.36E-08	1.36E-08	blue
CHST12	-0.09459	-0.1271	-0.09618	0.03336	0.05815	-0.16891	-0.0107	0.24182	-0.24182	0.21848	0.09761	0.2108	0.66485	0.44994	0.02721	0.88957	0.00144	0.00144	grey
HIST1H2A	0.05802	-0.0609	0.07249	0.05734	-0.04906	0.09761	0.20172	-0.24087	0.24087	0.45098	0.42881	0.34609	0.45629	0.52402	0.20403	0.00815	0.00151	0.00151	grey
MACF1	-0.08512	-0.21502	-0.01442	0.02631	-0.19194	0.09094	-0.04439	0.33055	-0.33055	0.26832	0.00474	0.85152	0.73266	0.01191	0.23682	0.56425	1.01E-05	1.01E-05	turquoise
CDC26	0.02862	-0.06709	-0.10983	-0.01703	-0.00974	0.01678	0.02253	-0.04879	0.04879	0.71017	0.38325	0.15272	0.82499	0.89937	0.82756	0.76991	0.52627	0.52627	turquoise
MSANTD2	-0.04601	-0.19468	-0.01666	0.02782	0.05275	0.03743	0.00487	-0.05436	0.05436	0.55017	0.01072	0.82881	0.71792	0.49321	0.62691	0.94964	0.48006	0.48006	red
BTN2A1	-0.0196	-0.1628	-0.0072	-0.02827	0.06301	0.03205	-0.04107	0.11506	-0.11506	0.79912	0.03338	0.9255	0.71356	0.41292	0.6773	0.59375	0.13398	0.13398	turquoise
RABIF	-0.01265	-0.07872	-0.00082	0.03173	-0.04933	0.06015	0.11666	-0.12969	0.12969	0.86961	0.30611	0.99155	0.68037	0.52166	0.43453	0.12861	0.0909	0.0909	greenyellow
PSMD6	-0.06857	0.05405	0.02506	-0.03163	-0.14202	0.10061	-0.04793	0.09153	-0.09153	0.37287	0.48256	0.74496	0.68131	0.06389	0.19044	0.53362	0.23381	0.23381	grey
ZNF236	-0.09584	-0.17815	-0.08387	0.03399	-0.02343	-0.04317	-0.06373	0.3563	-0.3563	0.2124	0.01975	0.27543	0.65893	0.76102	0.57508	0.40763	1.73E-06	1.73E-06	turquoise
PLGRKT	-0.02228	0.12215	0.00423	-0.02074	-0.03971	0.08623	0.17947	-0.05778	0.05778	0.77242	0.11148	0.95622	0.78776	0.60609	0.26213	0.01884	0.45285	0.45285	grey
BRMS1	0.06199	0.06957	-0.04232	-0.01411	-0.01114	-0.0435	0.21077	-0.37782	0.37782	0.42056	0.36588	0.58262	0.8547	0.88507	0.57211	0.00566	3.50E-07	3.50E-07	greenyellow
ARMCX1	-0.07035	-0.14725	-0.15425	0.03561	0.0129	-0.19887	-0.16247	0.50879	-0.50879	0.36055	0.05461	0.04397	0.64382	0.86698	0.00912	0.03374	1.20E-12	1.20E-12	brown
KRT16	0.0115	0.01948	0.09344	0.02444	-0.05325	0.11618	0.17422	-0.33906	0.33906	0.88133	0.8004	0.22416	0.75106	0.48909	0.1302	0.02267	5.72E-06	5.72E-06	yellow
KIAA0895	-0.0615	-0.24614	-0.14993	-0.03414	0.13687	-0.13134	-0.15377	-0.01416	0.01416	0.42426	0.00117	0.05031	0.65752	0.07425	0.08683	0.04464	0.85412	0.85412	turquoise
BDKRB1	-0.11333	-0.16434	0.05158	0.13882	0.0191	0.11041	0.00494	0.16633	-0.16633	0.13999	0.03173	0.50285	0.07017	0.80421	0.15054	0.94883	0.02969	0.02969	grey
TAMM41	-0.01613	-0.03979	0.0288	-0.07874	-0.07584	0.10398	0.14677	-0.15993	0.15993	0.83412	0.60534	0.70843	0.306	0.32418	0.17592	0.05542	0.03667	0.03667	grey
DNAJB2	0.15704	-0.14671	-0.20103	-0.04019	0.115	-0.21347	-0.06251	-0.17289	0.17289	0.04024	0.05551	0.00838	0.60176	0.13418	0.00506	0.41669	0.02374	0.02374	grey
SYMPK	0.12227	-0.06243	-0.12836	-0.13171	0.14891	-0.10528	0.08096	-0.15051	0.15051	0.11112	0.41725	0.09429	0.08593	0.05192	0.17054	0.29252	0.04942	0.04942	grey
TRIM5	0.02111	-0.07467	0.02632	0.10943	-0.17316	0.25584	0.25215	-0.08221	0.08221	0.78402	0.33173	0.73254	0.15423	0.02352	0.00073	0.00088	0.2851	0.2851	turquoise
TBC1D17	0.03677	-0.0197	-0.15755	-0.10167	0.11183	-0.10192	-0.04721	-0.02417	0.02417	0.63303	0.79813	0.03959	0.18578	0.14533	0.18467	0.53977	0.75368	0.75368	red
LILRA1	-0.06916	-0.0978	-0.00385	0.00473	-0.16537	0.06556	0.04753	0.52015	-0.52015	0.36875	0.20319	0.96012	0.95109	0.03065	0.39427	0.53705	3.07E-13	3.07E-13	blue
PDZD8	0.03768	-0.10408	-0.05016	0.03958	-0.02913	0.11855	0.06135	-0.03174	0.03174	0.62468	0.1755	0.51472	0.60726	0.70523	0.12252	0.42536	0.68024	0.68024	turquoise
ITLN1	-0.05448	-0.05168	0.07208	0.07609	0.20687	0.16712	0.00032	-0.12748	0.12748	0.47915	0.50203	0.34883	0.32257	0.00663	0.02891	0.9967	0.0966	0.0966	grey
CCNE1	-0.01286	-0.06037	-0.0229	0.03106	-0.12688	0.03223	0.23875	-0.31724	0.31724	0.86745	0.4328	0.76625	0.68675	0.09819	0.67558	0.00166	2.36E-05	2.36E-05	pink
DGKH	0.05797	-0.15396	-0.00582	0.04451	-0.07179	0.09898	0.10115	-0.05288	0.05288	0.45138	0.04438	0.9398	0.56322	0.35075	0.19776	0.18803	0.49215	0.49215	turquoise
CTNNBIP1	0.00969	-0.08062	-0.01628	-0.0863	-0.03619	0.01119	0.01426	-0.1919	0.1919	0.89989	0.29451	0.83261	0.26173	0.63837	0.88447	0.8531	0.01192	0.01192	grey
ALS2	0.0029	-0.17264	-0.06524	0.02571	0.01257	0.04319	0.05226	0.02313	-0.02313	0.97	0.02395	0.39658	0.7385	0.87038	0.57489	0.49728	0.76394	0.76394	turquoise
ACTR8	-0.03857	-0.10776	-0.06299	-0.08835	-0.15145	0.05913	0.04872	0.18451	-0.18451	0.61649	0.16067	0.4131	0.25053	0.048	0.44233	0.52684	0.0157	0.0157	turquoise

OTX1	0.03714	0.01454	-0.02989	0.01385	0.03201	0.01535	0.2432	-0.58043	0.58043	0.6296	0.85026	0.69794	0.85736	0.67766	0.84207	0.00135	8.75E-17	8.75E-17	pink
STT3A	0.00425	-0.10791	-0.02968	-0.00663	-0.08308	-0.01442	0.11715	-0.08434	0.08434	0.95597	0.16008	0.69997	0.93142	0.28	0.85154	0.12701	0.27275	0.27275	turquoise
TLN2	-0.07034	-0.00033	0.11899	0.09707	-0.11115	0.16006	0.15752	0.1074	-0.1074	0.36061	0.99655	0.12113	0.20659	0.14781	0.03651	0.03963	0.16205	0.16205	turquoise
PRKACA	-0.0264	-0.16808	-0.19549	-0.04255	-0.01245	-0.21812	-0.17905	0.35078	-0.35078	0.73177	0.02798	0.01039	0.58058	0.87159	0.00416	0.01912	2.56E-06	2.56E-06	turquoise
MRPS2	0.00258	0.01995	-0.15794	-0.10049	-0.03246	-0.10444	0.10004	-0.2224	0.2224	0.9733	0.79559	0.0391	0.19097	0.67346	0.174	0.19296	0.00346	0.00346	green
NUBP1	-0.06072	0.02106	-0.09022	-0.03209	-0.09749	0.01585	-0.05169	0.15022	-0.15022	0.43016	0.78457	0.2406	0.67695	0.20462	0.83696	0.50195	0.04987	0.04987	turquoise
GHRL	-0.00231	-0.03404	-0.1194	-0.05098	0.0207	-0.00649	-0.02634	0.27543	-0.27543	0.97603	0.65848	0.11984	0.50781	0.78813	0.93283	0.73241	0.00027	0.00027	grey
IKZF2	-0.00807	-0.13057	0.04357	0.07812	-0.12066	0.10356	0.16182	-0.07241	0.07241	0.91659	0.08872	0.57153	0.30979	0.11596	0.1777	0.03448	0.34662	0.34662	turquoise
LYPLA2	0.02258	-0.01058	0.01493	-0.00059	-0.00222	0.11294	0.15981	-0.41022	0.41022	0.76946	0.89081	0.84629	0.9939	0.97701	0.14137	0.03681	2.52E-08	2.52E-08	green
XPNPEP1	0.07176	0.02233	0.11454	0.02362	-0.00554	0.17044	0.1971	-0.27564	0.27564	0.35094	0.77192	0.13577	0.75909	0.94271	0.02583	0.00977	0.00026	0.00026	turquoise
ARMT1	-0.10717	-0.06553	0.0269	0.01822	-0.05016	0.02341	0.00661	0.2074	-0.2074	0.16296	0.39443	0.72693	0.81299	0.51473	0.76123	0.93158	0.00649	0.00649	turquoise
CHMP5	-0.01269	-0.07243	-0.07718	-0.06503	0.00444	-0.06558	0.01167	-0.09308	0.09308	0.86912	0.34649	0.31572	0.39809	0.95399	0.39413	0.87963	0.22594	0.22594	turquoise
LUZP1	-0.06964	-0.14942	0.04938	0.07011	-0.16684	0.23736	0.10539	0.19822	-0.19822	0.36539	0.05111	0.52125	0.36219	0.02918	0.00177	0.17009	0.00935	0.00935	turquoise
QRICH1	-0.05846	-0.11418	-0.025	-0.07244	-0.07567	0.08759	0.01268	0.09306	-0.09306	0.44757	0.137	0.74554	0.34639	0.32529	0.25462	0.86925	0.22604	0.22604	turquoise
GLUD1	0.06694	-0.03274	0.01402	-0.01063	-0.09257	0.03218	0.17193	-0.16751	0.16751	0.38432	0.6708	0.85554	0.89028	0.22852	0.67609	0.02454	0.02853	0.02853	turquoise
CD247	-0.07646	-0.03115	0.05424	0.06915	-0.16034	0.1072	0.01217	0.35981	-0.35981	0.32025	0.68592	0.48105	0.36884	0.03618	0.16283	0.87445	1.34E-06	1.34E-06	blue
SUN1	-0.00636	-0.12313	0.03702	-0.00453	-0.0119	0.04632	0.02344	-0.23031	0.23031	0.93424	0.10862	0.63073	0.95315	0.87726	0.54743	0.76087	0.00244	0.00244	turquoise
CBLL1	-0.01074	-0.15279	-0.02075	-0.00131	-0.07362	0.03457	-0.02753	0.13017	-0.13017	0.88916	0.04603	0.78768	0.98642	0.33861	0.65353	0.72079	0.0897	0.0897	turquoise
C16orf87	0.00554	-0.08936	-0.00023	0.03376	-0.11285	0.06247	0.08709	0.04671	-0.04671	0.94264	0.24511	0.99761	0.66113	0.14168	0.41695	0.25739	0.5441	0.5441	turquoise
HJURP	0.01811	0.06353	0.04016	0.03204	-0.05523	0.09403	0.3144	-0.4861	0.4861	0.81414	0.40912	0.60199	0.67737	0.47311	0.22121	2.82E-05	1.58E-11	1.58E-11	pink
IVD	-0.08882	-0.0566	-0.18834	-0.1164	0.04986	-0.11998	-0.05355	-0.03581	0.03581	0.24801	0.46216	0.01363	0.12947	0.51719	0.11804	0.48665	0.64196	0.64196	grey
SMC4	-0.07078	-0.07317	0.03392	0.02685	-0.15085	0.14232	0.22452	-0.04429	0.04429	0.35759	0.34154	0.65963	0.72742	0.04891	0.06332	0.00316	0.56514	0.56514	turquoise
RPL12	0.14146	0.14001	0.01476	-0.04456	-0.14071	0.11128	0.11307	-0.26428	0.26428	0.06496	0.06778	0.84807	0.56274	0.0664	0.14734	0.14089	0.00048	0.00048	purple
HNRNPC	0.01731	-0.08529	-0.01435	-0.02929	-0.05847	0.06796	0.04563	-0.09489	0.09489	0.82219	0.26738	0.85225	0.70372	0.4475	0.37712	0.55347	0.21698	0.21698	turquoise
GTPBP3	-0.00364	0.01044	-0.11598	-0.06745	0.20747	-0.09263	0.06434	-0.28927	0.28927	0.96232	0.89221	0.13089	0.38075	0.00647	0.22819	0.40315	0.00012	0.00012	red
ZNF646	-0.0092	-0.11522	-0.07359	0.00753	-0.03776	0.03509	0.07477	0.04116	-0.04116	0.90491	0.13344	0.33882	0.92208	0.62391	0.64867	0.3311	0.59295	0.59295	turquoise
CCDC65	0.04459	-0.20687	-0.20278	0.01466	0.18625	-0.31	-0.24745	0.28153	-0.28153	0.56252	0.00663	0.00782	0.84909	0.01473	3.69E-05	0.0011	0.00019	0.00019	brown
MPZL1	-0.0336	-0.09148	0.06496	0.0182	-0.05466	0.22555	0.13609	-0.03616	0.03616	0.66265	0.23406	0.39861	0.81318	0.47768	0.00302	0.07593	0.63871	0.63871	turquoise
FBXL8	0.02473	0.04652	-0.01958	-0.09089	0.20655	0.05232	0.11876	-0.40808	0.40808	0.74812	0.54572	0.79936	0.2371	0.00672	0.49671	0.12184	3.02E-08	3.02E-08	green
CFAP97	-0.1056	-0.07972	-0.03738	0.0418	-0.12396	0.12285	0.00105	0.23875	-0.23875	0.16926	0.3	0.62737	0.58721	0.10624	0.10944	0.98909	0.00166	0.00166	turquoise
FOXF2	0.0203	-0.01111	0.01205	0.03542	-0.15417	0.18254	0.14023	0.02884	-0.02884	0.79209	0.88529	0.87569	0.64556	0.04408	0.01686	0.06734	0.7081	0.7081	black
PPARG	0.00132	-0.05827	0.24187	0.08771	-0.05622	0.31067	0.21574	-0.45882	0.45882	0.98633	0.44905	0.00144	0.25396	0.46521	3.54E-05	0.0046	2.77E-10	2.77E-10	yellow
SFRP4	-0.03449	-0.11648	0.09921	0.14499	-0.18532	0.26391	0.04419	0.38744	-0.38744	0.65425	0.12921	0.19672	0.05847	0.01524	0.00049	0.56605	1.65E-07	1.65E-07	blue
TOMM40L	-0.04654	-0.05451	-0.06625	-0.0562	-0.06577	-0.02609	0.10715	0.07381	-0.07381	0.54556	0.47888	0.3893	0.4653	0.39272	0.73483	0.16303	0.33737	0.33737	turquoise
LAS1L	-0.05308	-0.04773	-0.14671	-0.06768	-0.02827	-0.07611	0.12733	-0.1531	0.1531	0.49048	0.53527	0.05551	0.37914	0.71358	0.32245	0.097	0.04559	0.04559	grey
FAM167B	-0.14936	-0.11133	-0.07226	-0.04117	0.05126	-0.07304	-0.08632	0.28751	-0.28751	0.05121	0.14717	0.34761	0.59287	0.50551	0.34239	0.26161	0.00014	0.00014	grey
ZNF347	-0.09231	-0.14802	-0.09703	0.0589	-0.06829	0.02369	-0.11891	0.33073	-0.33073	0.22985	0.05335	0.20675	0.44416	0.37483	0.75843	0.12136	9.96E-06	9.96E-06	turquoise
ADAM28	-0.07194	-0.02613	0.11812	0.02705	-0.00632	0.24426	0.10942	-0.22709	0.22709	0.34977	0.7344	0.1239	0.72546	0.9346	0.00128	0.15428	0.00282	0.00282	yellow
NRF1	-0.00096	-0.186	-0.10747	0.02499	0.07822	-0.23559	-0.10487	0.18972	-0.18972	0.99006	0.01486	0.16178	0.74557	0.30921	0.00192	0.17222	0.01294	0.01294	turquoise
LRRC15	-0.06611	0.02093	0.06105	0.03789	-0.15259	0.08841	0.10303	0.14267	-0.14267	0.39032	0.78587	0.42762	0.62269	0.04632	0.25021	0.17991	0.06268	0.06268	black
ROMO1	0.08417	-0.02865	-0.12915	-0.07659	0.22785	-0.21217	0.04816	-0.4433	0.4433	0.27372	0.70992	0.09228	0.3194	0.00272	0.00534	0.53162	1.26E-09	1.26E-09	green
WWP2	0.05954	0.00035	0.0274	-0.02807	-0.04965	0.19781	0.09959	-0.06402	0.06402	0.43923	0.99637	0.72204	0.71552	0.519	0.0095	0.19498	0.40544	0.40544	turquoise
B4GALNT	0.03429	-0.06663	-0.03153	0.01565	0.07642	-0.00317	-0.00969	-0.26657	0.26657	0.65615	0.38655	0.68228	0.83899	0.3205	0.96723	0.89993	0.00042	0.00042	grey
LRP10	0.06927	-0.07844	0.09905	0.01609	-0.04197	0.18545	0.11367	-0.22671	0.22671	0.368	0.30785	0.19741	0.83454	0.5857	0.01516	0.13879	0.00287	0.00287	turquoise
SOWAHB	-0.03955	-0.13354	0.06679	0.00979	0.02944	0.18715	0.07672	-0.28923	0.28923	0.60756	0.08163	0.38541	0.89889	0.70226	0.01424	0.31857	0.00012	0.00012	grey
USF2	0.08394	-0.09341	-0.17169	-0.0952	-0.00855	-0.13433	-0.01049	0.1579	-0.1579	0.27502	0.22428	0.02475	0.21551	0.9116	0.07984	0.8917	0.03914	0.03914	grey
PRRC1	-0.0109	-0.13307	-0.02167	0.01813	-0.07306	0.06233	-0.01492	0.12705	-0.12705	0.88744	0.08272	0.77849	0.81389	0.3423	0.41801	0.84644	0.09774	0.09774	turquoise
SYNDIG1	-0.032	-0.11936	-0.02163	0.06753	-0.06834	-0.09453	-0.08474	0.28624	-0.28624	0.67781	0.11994	0.77884	0.38014	0.37447	0.21878	0.27047	0.00015	0.00015	black
STYK1	0.03682	0.01125	0.09152	0.08786	0.06117	0.16577	0.21789	-0.45689	0.45689	0.63261	0.88393	0.23384	0.25315	0.42674	0.03024	0.0042	3.36E-10	3.36E-10	yellow
COX7A2L	0.07362	-0.05213	-0.10919	-0.08166	-0.05281	-0.03079	0.05609	-0.10096	0.10096	0.33859	0.49832	0.15513	0.28836	0.49275	0.68932	0.46624	0.18888	0.18888	turquoise
ZNF22	-0.1002	-0.11647	-0.0187	0.04164	-0.14397	-0.08383	-0.10148	0.32667	-0.32667	0.19225	0.12927	0.80814	0.58867	0.06029	0.27568	0.18659	1.30E-05	1.30E-05	turquoise
CD80	-0.07025	-0.01698	0.12716	0.0494	-0.20801	0.21283	0.10534	0.25265	-0.25265	0.36125	0.82555	0.09744	0.5211	0.00633	0.00519	0.17029	0.00086	0.00086	blue
FCHSD2	-0.1025	-0.08088	0.00529	0.01112	-0.20754	0.11468	-0.04105	0.35488	-0.35488	0.1822	0.29295	0.94528	0.88523	0.00645	0.1353	0.59394	1.91E-06	1.91E-06	blue
TRIM36	0.04721	-0.16539	0.07199	0.11548	0.11743	-0.06268	-0.12705	-0.10689	0.10689	0.53973	0.03064	0.3494	0.13257	0.1261	0.41538	0.09774	0.16406	0.16406	grey
CNPPD1	0.09222	0.04219	0.0733	0.02689	-0.08047	0.19792	0.08052	-0.2177	0.2177	0.2303	0.58374	0.34073	0.72705	0.29545	0.00946	0.29514	0.00423	0.00423	turquoise
CDC42EP	0.05622	-0.08858	0.04839	0.04096	-0.04271	0.10733	0.08494	-0.12986	0.12986	0.46513	0.24928	0.52968	0.59479	0.57914	0.16235	0.26931	0.09047	0.09047	grey
MYBL2	0.03224	0.15782	0.1107	0.06705	-0.09388	0.15174	0.31323	-0.4106	0.4106	0.67548	0.03925	0.14947	0.3836	0.22194	0.04757	3.03E-05	2.44E-08	2.44E-08	pink
EZR	0.0192	0.01889	0.14971	0.06847	-0.08307	0.22134	0.23683	-0.41019	0.41019	0.80321	0.80632	0.05065	0.37358	0.28005	0.00362	0.00182	2.52E-08	2.52E-08	yellow
ARHGEF6	-0.12849	-0.06021	0.03084	0.05572	-0.23027	0.14119	-0.0262	0.5567	-0.5567	0.09397	0.43407	0.68888	0.46916	0.00245	0.06547	0.73375	2.65E-15	2.65E-15	blue
DIMT1	-0.07936	-0.08569	-0.04162	-0.01755	-0.05913	-0.01239	-0.08201	0.27294	-0.27294	0.3022	0.26513	0.58886	0.81977	0.44234	0.87217	0.28627	0.0003	0.0003	turquoise
CTTNBP2N	-0.07832	-0.16506	0.03548	0.08615	-0.13251	0.20766	0.08962	0.08552	-0.08552	0.30855	0.03097	0.64502	0.26254	0.08404	0.00642	0.24374	0.26609	0.26609	turquoise
FTSJ3	-0.02629	-0.14703	-0.02768	-0.01368	-0.1055	0.03273	0.14677	0.01933	-0.01933	0.73285	0.05499	0.71929	0.85909	0.16964	0.67087	0.05542	0.80187	0.80187	grey
C1QTNF1	-0.05454	0.00938	0.08614	0.07343	0.11354	0.10247	0.08668	-0.3724	0.3724	0.47866	0.9031	0.2626	0.33982	0.13925	0.18231	0.25962	5.29E-07	5.29E-07	yellow
PKP3	0.18375	0.07366	0.03704	-0.03024	0.09237	0.09839	0.19845	-0.65676	0.65676	0.01614	0.33832	0.63056	0.69459	0.22953	0.20044	0.00927	1.79E-22	1.79E-22	yellow
CNIH3	-0.06178	-0.18307	-0.08043	0.02841	-0.02051	-0.17208	-0.05712	0.17203	-0.17203	0.4221	0.01655	0.2957	0.71219	0.79007	0.02442	0.45801	0.02446	0.02446	grey
C4orf33	-0.10793	-0.03086	-0.01637	-0.02334	0.07719	0.12312	0.06469	-0.12949	0.12949	0.15999	0.68868	0.8317	0.76192	0.31563	0.10864	0.40059	0.0914	0.0914	turquoise
NIPA1	-0.01695	-0.19387	-0.06155	-0.02054	-0.02864	-0.0372	0.01243	-0.12258	0.12258	0.82584	0.01106	0.42385	0.78976	0.71001	0.6291	0.87181	0.11022	0.11022	turquoise
MED19	0.05461	-0.13035	-0.11023	-0.1051	-0.013	-0.04404	0.12748	-0.19215	0.19215	0.4781	0.08925	0.15123	0.17129	0.86596	0.56739	0.0966	0.01181	0.01181	grey
ARMC8	-0.08436	-0.12286	-0.06124	-0.00624	-0.09885	0.08078	0.08174	0.14782	-0.14782	0.27263	0.1094	0.42618	0.93542	0.19831	0.29355	0.28783	0.05368	0.05368	turquoise
ATP5MPL	0.12203	-0.01464	-0.13189	-0.08219	0.19413	-0.21933	0.02092	-0.30563	0.30563	0.11184	0.84922	0.08552	0.28519	0.01095	0.00395	0.78597	4.80E-05	4.80E-05	grey
PHAX	-0.07799	-0.18034	-0.10772	-0.05137	-0.0451	-0.04973	-0.08945	0.25892	-0.25892	0.3106	0.01826	0.1608	0.50457	0.5581	0.51828	0.24466	0.00063	0.00063	turquoise
MTUS1	-0.02956	-0.08172	-0.03393	0.0531	-0.07092	0.10715	-0.00363	0.09667	-0.09667	0.70115	0.28799	0.65956	0.49037	0.35662	0.16304	0.96244	0.20846	0.20846	turquoise
ZSWIM8	-0.04385	-0.20349	-0.03824	0.0087	0.02506	-0.06422	-0.07551	0.22935	-0.22935	0.56902	0.0076	0.61953	0.91006	0.74496	0.40398	0.32633	0.00255	0.00255	turquoise
KLHL8	-0.09391	-0.11202	-0.10334	0.01105	-0.05304	-0.05555	-0.08185	0.47357	-0.47357	0.22182	0.14464	0.17862	0.88597	0.49082	0.47051	0.2872	6.08E-11	6.08E-11	turquoise
TUBB6	-0.19071	-0.10743	-0.03031	0.08589	-0.10764	0.09455	0.05269	0.25542	-0.25542	0.01247	0.16194	0.69388	0.26402	0.16111	0.21865	0.49368	0.00075	0.00075	black
RIOK3	0.05336	-0.02573	0.08453	0.11191	-0.09227	0.16471	0.11699	-0.30773	0.30773	0.48821	0.73834	0.27164	0.14503	0.23004	0.03134	0.12755	4.23E-05	4.23E-05	turquoise
SMN2	-0.04974	0.04176	-0.03953	0.07552	-0.09535	0.0629	-0.0581	-0.08157	0.08157	0.51824	0.58763	0.60772	0.32625	0.21478	0.41374	0.45037	0.28886	0.28886	grey
ARHGEF5	0.15101	-0.02376	0.04715	-0.08247	0.04959	0.09917	0.11242	-0.53798	0.53798	0.04865	0.75775	0.54027	0.28356	0.51953	0.19688	0.14323	3.25E-14	3.25E-14	yellow
POLR2K	0.01715	-0.03345	-0.10065	-0.04115	-0.04447	-0.16495	-0.01785	-0.07246	0.07246	0.82385	0.66403	0.19025	0.59304	0.56359	0.03109	0.81675	0.34625	0.34625	turquoise
ALOX15B	-0.00705	0.04314	0.10889	0.11247	-0.22566	0.12852	0.16103	0.12959	-0.12959	0.92707	0.57534	0.15629	0.14302	0.003	0.09387	0.03537	0.09115	0.09115	black
PERP	0.05743	-0.00471	0.08298	0.04864	-0.06958	0.13189	0.12176	-0.41877	0.41877	0.45559	0.95129	0.28056	0.52758	0.36586	0.08551	0.11263	1.20E-08	1.20E-08	turquoise
WDR6	-0.00681	-0.1006	-0.10074	-0.14968	0.03759	-0.10031	-0.14841	0.18731	-0.18731	0.92956	0.19049	0.18984	0.0507	0.6255	0.19174	0.05272	0.01416	0.01416	turquoise
PHRF1	0.0281	-0.14602	-0.0759	-0.04813	0.0793	-0.02095	0.01692	-0.15646	0.15646	0.71524	0.0567	0.32382	0.53186	0.30251	0.78566	0.82615	0.04099	0.04099	grey
QTRT1	0.07142	0.07245	-0.15228	-0.08377	0.28832	-0.18889	-0.06376	-0.17035	0.17035	0.35329	0.34633	0.04678	0.27602	0.00013	0.01335	0.40742	0.02591	0.02591	green
MEI1	-0.17788	0.11804	0.03197	0.01042	-0.03471	0.07715	-0.05202	0.19359	-0.19359	0.01993	0.12415	0.67806	0.89245	0.65218	0.31587	0.49925	0.01118	0.01118	blue
LTA	-0.08514	0.02888	0.06179	-0.01808	-0.11524	0.09263	-0.04571	0.3391	-0.3391	0.26821	0.70771	0.42206	0.81446	0.13337	0.22818	0.55273	5.71E-06	5.71E-06	blue
LIMS1	-0.05684	-0.09155	0.07148	0.09898	-0.23256	0.19919	0.14816	0.07283	-0.07283	0.46024	0.23367	0.35284	0.19776	0.00221	0.00901	0.05312	0.34379	0.34379	black
RAB5IF	0.03016	0.07367	0.02305	0.03746	0.03326	0.02402	0.18035	-0.44094	0.44094	0.69538	0.3383	0.7648	0.62667	0.66585	0.7552	0.01825	1.58E-09	1.58E-09	pink
MEOX2	-0.09239	0.00572	0.0463	0.07812	-0.20139	0.10346	0.02651	0.50455	-0.50455	0.22941	0.94082	0.54764	0.30981	0.00826	0.17812	0.73068	1.98E-12	1.98E-12	blue
INSIG2	-0.01704	-0.09142	0.01386	0.10711	-0.13746	0.12741	0.08961	-0.19046	0.19046	0.82498	0.23434	0.85723	0.1632	0.073	0.0968	0.24381	0.01259	0.01259	turquoise
DENND1B	0.06307	-0.05006	0.05307	0.09361	-0.02531	0.17725	0.13873	-0.07731	0.07731	0.41246	0.51559	0.4906	0.22332	0.74248	0.02038	0.07036	0.31489	0.31489	turquoise
DECR1	-0.03291	0.06409	-0.00614	-0.04786	-0.06932	-0.01115	0.15817	-0.07226	0.07226	0.66916	0.40495	0.93649	0.53421	0.36763	0.88487	0.03881	0.34762	0.34762	grey
HOGA1	0.03196	-0.05534	-0.1875	0.05186	0.04829	-0.09673	-0.11892	0.16237	-0.16237	0.67817	0.47217	0.01406	0.50057	0.53055	0.20818	0.12133	0.03386	0.03386	magenta
RAB2A	-0.02618	-0.0349	-0.09216	-0.06406	2.75E-05	-0.13456	-0.05854	0.02043	-0.02043	0.73395	0.65041	0.23059	0.40516	0.99971	0.07931	0.44692	0.79083	0.79083	turquoise
FRMD4A	-0.16216	-0.05686	-0.04873	0.09684	-0.21533	0.10324	0.08349	0.46573	-0.46573	0.03409	0.4601	0.52676	0.20768	0.00468	0.17905	0.27763	1.37E-10	1.37E-10	blue
TRMT13	-0.06268	-0.12729	-0.0248	0.00399	0.06024	0.04603	-0.01011	0.12735	-0.12735	0.41543	0.0971	0.74751	0.95865	0.43381	0.54995	0.89557	0.09695	0.09695	red
EIF4EBP1	0.08409	0.19776	0.09965	0.1163	-0.07341	0.09841	0.2389	-0.35998	0.35998	0.2742	0.00952	0.1947	0.12984	0.33997	0.20037	0.00165	1.33E-06	1.33E-06	grey
NICN1	-0.00195	-0.09708	-0.17303	-0.11946	0.01038	-0.10274	-0.05279	0.22177	-0.22177	0.97977	0.20652	0.02363	0.11965	0.89284	0.18114	0.49287	0.00356	0.00356	red
SRPK1	-0.02231	0.02486	0.12913	0.04353	-0.06106	0.19987	0.16852	-0.27048	0.27048	0.7721	0.74688	0.09232	0.57189	0.42756	0.00877	0.02757	0.00035	0.00035	turquoise
CLCN5	0.00337	-0.13653	-0.16143	-0.08373	0.11093	-0.15429	-0.09504	0.31534	-0.31534	0.96506	0.07498	0.03492	0.27627	0.1486	0.04392	0.21627	2.66E-05	2.66E-05	turquoise
GPAT2	0.11449	-0.03149	0.01015	0.01012	-0.01732	0.05907	-0.01247	0.16314	-0.16314	0.13596	0.68261	0.89516	0.89547	0.82213	0.44283	0.87143	0.03301	0.03301	grey
MLF2	0.04709	-0.05767	-0.10127	-0.05856	0.17934	-0.17186	-0.04412	-0.22481	0.22481	0.54081	0.45369	0.18753	0.4468	0.01892	0.0246	0.56669	0.00311	0.00311	grey
TMEM39B	0.03173	-0.11395	-0.01452	0.0029	0.05137	-0.0707	-0.09033	0.09784	-0.09784	0.68038	0.13782	0.85045	0.96994	0.50464	0.35816	0.24002	0.20299	0.20299	turquoise
UBL3	0.04197	-0.14403	-0.10615	-0.00702	0.00809	-0.07925	-0.15122	0.3297	-0.3297	0.58575	0.06018	0.16704	0.92743	0.91634	0.30285	0.04834	1.06E-05	1.06E-05	turquoise
AMACR	-0.04176	-0.01724	-0.16465	-0.06521	0.00914	-0.21229	-0.01999	0.27836	-0.27836	0.58764	0.82289	0.0314	0.3968	0.90559	0.00531	0.7952	0.00023	0.00023	turquoise
TEAD2	-0.11571	-0.0512	-0.00091	0.12153	-0.26988	0.16675	0.07193	0.2066	-0.2066	0.13178	0.50604	0.99053	0.11331	0.00036	0.02927	0.34983	0.00671	0.00671	blue
ZFHX4	-0.01376	-0.08052	-0.0013	0.07448	-0.26258	0.10907	0.076	0.43619	-0.43619	0.85823	0.29513	0.98655	0.33294	0.00052	0.1556	0.32319	2.47E-09	2.47E-09	blue
CPNE5	-0.16142	0.08031	0.06071	-0.01064	-0.06075	0.14436	-0.05802	0.24251	-0.24251	0.03492	0.29639	0.43027	0.89018	0.42997	0.05959	0.45099	0.00139	0.00139	blue
PPP1R1C	0.02739	-0.05874	0.00151	0.03712	-0.09882	0.00648	0.06763	-0.29542	0.29542	0.72217	0.44536	0.98437	0.62978	0.19848	0.93297	0.37947	8.76E-05	8.76E-05	grey
KHDRBS3	0.03372	0.07682	-0.05076	-0.04361	-0.01221	-0.11285	-0.05151	0.19391	-0.19391	0.66153	0.31798	0.50969	0.57117	0.87405	0.14169	0.50346	0.01104	0.01104	grey
PDCL	-0.07683	-0.11843	-0.06326	0.00388	-0.14801	0.01866	-0.0181	0.29395	-0.29395	0.31788	0.12288	0.41108	0.9598	0.05336	0.8086	0.81427	9.53E-05	9.53E-05	turquoise
LIMS2	-0.07683	0.00583	-0.02076	0.03875	-0.01249	0.02535	-0.03363	0.27072	-0.27072	0.31792	0.93966	0.78756	0.61487	0.87122	0.74208	0.6624	0.00034	0.00034	salmon
SAMD4A	-0.06986	-0.20482	-0.01488	0.11016	-0.13151	0.03122	-0.02141	0.25824	-0.25824	0.3639	0.0072	0.84683	0.1515	0.08642	0.68524	0.78106	0.00065	0.00065	blue
DNAJA3	0.07987	0.00597	-0.01833	0.00263	-0.01004	0.00813	0.10634	-0.12648	0.12648	0.29908	0.93818	0.81192	0.97282	0.89634	0.91598	0.16626	0.09927	0.09927	turquoise
SPTBN4	0.01847	-0.13511	-0.30086	-0.14713	0.16772	-0.3479	-0.24657	0.40709	-0.40709	0.81051	0.07808	6.38E-05	0.05481	0.02833	3.12E-06	0.00115	3.29E-08	3.29E-08	brown
FNIP2	-0.07198	-0.10758	-0.00926	0.0434	0.02033	0.08416	-0.07953	0.24987	-0.24987	0.34951	0.16134	0.90433	0.57301	0.79186	0.2738	0.30114	0.00098	0.00098	turquoise
PMM2	-0.06266	-0.04578	0.07184	0.03472	-0.09843	0.18107	0.09174	-0.11053	0.11053	0.41552	0.55214	0.35045	0.65207	0.20028	0.01779	0.23271	0.15009	0.15009	turquoise
H2AFZ	-0.07621	-0.01853	-0.04391	-0.09438	-0.01707	-0.05208	0.1802	-0.32321	0.32321	0.32182	0.80993	0.56846	0.21948	0.82464	0.49869	0.01835	1.62E-05	1.62E-05	pink
ACBD6	0.08126	0.02976	-0.15779	-0.09634	0.03619	-0.08962	0.14325	-0.20834	0.20834	0.2907	0.69922	0.03928	0.21002	0.63839	0.24376	0.06161	0.00625	0.00625	greenyellow
C3orf14	-0.05317	-0.08422	-0.19266	-0.13918	0.05125	-0.28545	-0.28835	0.36477	-0.36477	0.48973	0.27342	0.01158	0.06945	0.50561	0.00015	0.00013	9.34E-07	9.34E-07	brown
INTS8	0.04398	-0.05107	0.02478	0.03992	-0.09361	0.07108	0.17418	-0.08503	0.08503	0.56793	0.50709	0.74767	0.60421	0.22332	0.3556	0.0227	0.26882	0.26882	turquoise
IMPA1	0.02107	-0.11525	-0.00765	0.00828	0.01572	-0.04762	-0.03074	0.08259	-0.08259	0.78441	0.13335	0.92089	0.91436	0.83828	0.53624	0.68977	0.28288	0.28288	turquoise
BMS1	0.0408	-0.09133	0.07275	-0.02574	-0.12513	0.06888	0.1298	-0.14458	0.14458	0.59623	0.23483	0.34438	0.73821	0.10296	0.3707	0.09063	0.0592	0.0592	turquoise
MS4A4A	-0.09903	0.02119	0.08706	0.05735	-0.23279	0.14155	0.05639	0.44406	-0.44406	0.19753	0.78321	0.25752	0.45624	0.00218	0.06479	0.46382	1.17E-09	1.17E-09	blue
CRYAB	0.035	-0.03803	-0.05281	0.08137	-0.07545	0.02831	-0.1262	0.32979	-0.32979	0.64945	0.6214	0.49275	0.29007	0.3267	0.7132	0.10003	1.06E-05	1.06E-05	blue
USB1	0.04635	-0.09066	0.01172	0.00245	-0.14085	0.10189	0.21649	-0.20999	0.20999	0.54716	0.2383	0.87904	0.97468	0.06614	0.1848	0.00445	0.00584	0.00584	turquoise
STEAP4	-0.00942	-0.11113	0.00343	0.06564	-0.0908	0.07685	-0.07035	0.35501	-0.35501	0.9027	0.14789	0.96453	0.39367	0.23756	0.31775	0.36053	1.89E-06	1.89E-06	blue
S100P	0.0216	0.13537	0.22259	0.08139	-0.07369	0.35292	0.30524	-0.44375	0.44375	0.77915	0.0775	0.00343	0.28994	0.33815	2.20E-06	4.92E-05	1.21E-09	1.21E-09	yellow
DCLK2	-0.04235	-0.15077	-0.13059	-0.00505	-0.03499	-0.17697	-0.15343	0.56624	-0.56624	0.58237	0.04902	0.08868	0.9477	0.64956	0.02059	0.04512	6.95E-16	6.95E-16	brown

SLITRK6	0.00697	-0.09207	-0.16621	-0.21563	-0.07781	-0.07349	0.04676	0.11061	-0.11061	0.9279	0.23102	0.0298	0.00462	0.31176	0.33946	0.54364	0.14979	0.14979	grey
SOBP	-0.05353	-0.1231	-0.22466	-0.02198	0.00547	-0.26562	-0.13422	0.54045	-0.54045	0.48681	0.10871	0.00314	0.77538	0.94334	0.00045	0.08008	2.36E-14	2.36E-14	brown
HNRNPA1	0.01378	0.12382	-0.01812	-0.06688	0.00107	0.04656	0.06627	-0.05434	0.05434	0.858	0.10664	0.81399	0.38479	0.98895	0.54534	0.38912	0.48024	0.48024	grey
SPDEF	0.06271	0.10788	0.16766	0.07706	0.06831	0.26304	0.14866	-0.39924	0.39924	0.4152	0.16018	0.02839	0.31645	0.37468	0.00051	0.05231	6.33E-08	6.33E-08	yellow
RPL35	0.06685	0.13526	-0.02694	-0.01214	-0.10385	0.0098	0.14527	-0.27775	0.27775	0.38497	0.07776	0.7265	0.8748	0.17645	0.89881	0.05798	0.00024	0.00024	purple
MAPKAPK	-0.04421	-0.02581	0.01239	-0.02705	-0.19561	0.06664	0.16499	-0.11321	0.11321	0.56584	0.73759	0.87226	0.7254	0.01035	0.38651	0.03105	0.14042	0.14042	grey
RAB12	-0.04886	-0.21201	-0.0612	-0.00231	-0.05337	-0.12602	0.00223	0.16838	-0.16838	0.52568	0.00537	0.42655	0.97609	0.48817	0.10051	0.97693	0.0277	0.0277	turquoise
NEB	-0.11259	-0.06031	-0.13621	-0.0503	0.0606	-0.03553	-0.07594	0.0726	-0.0726	0.14259	0.4333	0.07567	0.51352	0.43104	0.64451	0.32354	0.34533	0.34533	grey
ETV3	-0.01781	-0.14318	0.03063	0.02019	-0.06636	0.19368	0.08162	-0.00101	0.00101	0.81711	0.06174	0.69087	0.79322	0.38852	0.01114	0.28858	0.98953	0.98953	turquoise
NGF	-0.09172	-0.09603	-0.03094	0.06936	-0.03454	-0.03669	-0.02093	0.45723	-0.45723	0.23281	0.21153	0.68786	0.36735	0.65383	0.63377	0.78588	3.25E-10	3.25E-10	blue
GSDMD	-0.00752	0.03611	-0.09475	-0.01365	0.01275	0.02715	0.2125	-0.39162	0.39162	0.92221	0.63919	0.2177	0.85939	0.86857	0.7245	0.00527	1.18E-07	1.18E-07	green
PSEN1	0.02104	-0.11663	0.07647	0.10285	-0.07491	0.20589	0.15221	-0.23817	0.23817	0.78475	0.12873	0.32018	0.18069	0.33017	0.0069	0.04688	0.00171	0.00171	turquoise
DOLK	-0.06357	-0.06743	-0.1627	-0.06078	-0.07825	-0.1489	0.03945	0.1693	-0.1693	0.40881	0.3809	0.03349	0.42969	0.30903	0.05194	0.60848	0.02686	0.02686	turquoise
RB1CC1	-0.04739	-0.11774	-0.05631	0.01022	-0.19345	-0.02654	0.04467	0.16611	-0.16611	0.53824	0.1251	0.46445	0.89442	0.01124	0.73042	0.5618	0.02991	0.02991	turquoise
VIPR2	-0.00832	0.06791	-0.00124	0.05788	-0.12146	0.05096	-0.11835	0.4668	-0.4668	0.914	0.37749	0.98715	0.4521	0.11353	0.50805	0.12313	1.23E-10	1.23E-10	grey
ZNF711	-0.06289	-0.12039	-0.11344	-0.0142	0.16675	-0.03709	-0.13682	0.10992	-0.10992	0.41386	0.11677	0.13958	0.85379	0.02927	0.6301	0.07436	0.15237	0.15237	grey
BBS10	-0.08586	-0.16678	-0.0877	-0.00703	-0.03095	-0.08927	-0.09715	0.34629	-0.34629	0.26418	0.02924	0.25401	0.92729	0.68776	0.2456	0.2062	3.49E-06	3.49E-06	turquoise
HMCN1	-0.03457	-0.0618	0.04121	0.07435	-0.24943	0.16795	0.08697	0.40556	-0.40556	0.65353	0.42197	0.59257	0.33378	0.001	0.02811	0.25801	3.74E-08	3.74E-08	blue
CDH24	-0.04517	-0.06914	-0.06289	-0.016	0.02769	-0.0592	0.07175	-0.04381	0.04381	0.55746	0.36891	0.41385	0.83546	0.71925	0.44179	0.35107	0.56941	0.56941	grey
FOXI1	-0.0708	-0.06828	0.03514	0.05655	0.01833	0.1207	0.02222	-0.09011	0.09011	0.35744	0.37485	0.64814	0.46253	0.81193	0.11583	0.77299	0.24117	0.24117	grey
EXOG	-0.07584	-0.1559	-0.17831	-0.13804	0.05324	-0.15842	-0.11624	0.34854	-0.34854	0.32417	0.04173	0.01963	0.07179	0.48918	0.0385	0.13003	2.99E-06	2.99E-06	turquoise
FBXO46	-0.00151	-0.09882	-0.08712	-0.09231	0.15289	-0.01202	0.03508	-0.26048	0.26048	0.98434	0.1985	0.25721	0.22981	0.04589	0.87597	0.64878	0.00058	0.00058	green
TRAT1	-0.09486	-0.01106	0.07436	0.08181	-0.12215	0.13841	-0.04429	0.43857	-0.43857	0.21715	0.88584	0.33373	0.28743	0.11149	0.07101	0.56514	1.98E-09	1.98E-09	blue
COLEC11	0.0216	-0.04929	-0.09252	-0.03903	-0.03161	0.118	-0.06464	0.33894	-0.33894	0.77911	0.52207	0.22876	0.61226	0.68146	0.12426	0.40096	5.77E-06	5.77E-06	grey
GSTK1	0.05719	0.04019	0.13191	-0.04133	0.07399	0.09362	0.13097	-0.39005	0.39005	0.45747	0.60173	0.08546	0.59147	0.33615	0.22324	0.08773	1.34E-07	1.34E-07	yellow
SLC9A6	-0.02362	-0.20364	-0.09785	-0.02758	-0.05987	-0.05161	-0.07459	0.17495	-0.17495	0.75907	0.00755	0.20292	0.72027	0.43662	0.50265	0.33227	0.0221	0.0221	turquoise
ACIN1	0.02325	-0.16411	-0.05178	-0.00871	0.1148	-0.0286	-0.02228	-0.03646	0.03646	0.76273	0.03197	0.50118	0.90995	0.13487	0.71037	0.77241	0.6359	0.6359	red
PYGB	0.10688	0.03474	0.11377	0.02124	-0.01116	0.10905	0.20238	-0.41938	0.41938	0.16411	0.65188	0.13843	0.78272	0.88485	0.15567	0.00794	1.13E-08	1.13E-08	grey
SORT1	-0.0311	-0.19754	-0.08552	-0.03484	-0.04985	0.03011	-0.04487	0.17799	-0.17799	0.68639	0.0096	0.26605	0.65096	0.51734	0.69583	0.56004	0.01986	0.01986	turquoise
ASNS	0.14487	0.01679	-0.08398	-0.04799	0.17603	-0.18183	-0.14848	-0.08008	0.08008	0.05869	0.82743	0.27478	0.53307	0.02128	0.0173	0.0526	0.2978	0.2978	grey
FPGS	0.06327	0.06605	0.0294	-0.03214	-0.11978	0.12995	0.17283	-0.36285	0.36285	0.41099	0.39068	0.7027	0.67646	0.11865	0.09026	0.02379	1.08E-06	1.08E-06	grey
GADD45G	0.05714	-0.07354	-0.26147	-0.05847	0.14569	-0.22224	-0.16849	0.12185	-0.12185	0.45789	0.33913	0.00055	0.4475	0.05726	0.00348	0.0276	0.11237	0.11237	brown
IFIT3	-0.03084	0.05205	0.07112	0.11726	-0.19561	0.14774	0.20136	-0.05511	0.05511	0.68883	0.49896	0.35529	0.12666	0.01035	0.0538	0.00827	0.47402	0.47402	tan
CCDC68	-0.0792	-0.03725	0.00104	-0.00535	0.06524	0.11837	0.1168	-0.27212	0.27212	0.30315	0.6286	0.98928	0.94461	0.39658	0.12308	0.12817	0.00032	0.00032	turquoise
PARK7	-0.03186	-0.04861	-0.05989	-0.02607	-0.03083	-0.06129	-0.00416	0.04167	-0.04167	0.67908	0.52777	0.4365	0.73505	0.68896	0.42585	0.95688	0.58843	0.58843	turquoise
SRRM1	-0.04107	-0.15043	0.03945	0.01224	-0.07889	0.19064	0.00909	0.00963	-0.00963	0.59383	0.04955	0.60841	0.87374	0.30505	0.0125	0.90611	0.90054	0.90054	turquoise
PDCD1	-0.07573	0.01687	-0.00039	0.06781	-0.10234	0.09139	0.01505	0.29773	-0.29773	0.32488	0.82666	0.99592	0.37815	0.18287	0.2345	0.84511	7.66E-05	7.66E-05	blue
KLF3	-0.00792	-0.01727	0.08156	0.07148	-0.07165	0.26525	0.14814	-0.16141	0.16141	0.91808	0.82262	0.28891	0.35288	0.35173	0.00045	0.05316	0.03494	0.03494	turquoise
GASK1B	-0.11033	-0.06234	0.02336	0.0851	-0.21733	0.09909	0.0212	0.43609	-0.43609	0.15084	0.41791	0.76165	0.26843	0.0043	0.19726	0.78316	2.49E-09	2.49E-09	blue
SLC13A5	-0.01172	-0.05661	0.09452	0.10479	-0.00995	0.0782	0.12896	-0.13403	0.13403	0.87904	0.46211	0.21883	0.17258	0.89725	0.30929	0.09275	0.0805	0.0805	grey
NKIRAS2	-0.03367	-0.14746	-0.04124	0.01346	-0.08077	0.04186	0.05365	-0.11686	0.11686	0.66197	0.05426	0.5923	0.86131	0.29363	0.58668	0.48585	0.12798	0.12798	turquoise
CASR	-0.03034	-0.07762	-0.10503	-0.01649	0.10637	-0.01754	-0.07956	0.2083	-0.2083	0.69368	0.31294	0.17156	0.83049	0.16616	0.81985	0.30097	0.00626	0.00626	grey
PTGFRN	-0.00995	-0.05328	0.04546	0.10304	-0.22387	0.24133	0.17562	0.08744	-0.08744	0.89722	0.4889	0.55494	0.17987	0.00325	0.00147	0.02159	0.25543	0.25543	black
RNF4	-0.08732	-0.16754	-0.02515	0.02457	-0.10144	0.03194	-0.04536	0.16274	-0.16274	0.25611	0.0285	0.74402	0.74972	0.18679	0.67838	0.55575	0.03345	0.03345	turquoise
GPR132	-0.08462	0.0385	0.08703	0.14072	-0.08193	0.14226	0.07979	0.02501	-0.02501	0.27116	0.61709	0.2577	0.0664	0.28674	0.06345	0.29953	0.74536	0.74536	blue
NFKB1	-0.08577	-0.13931	0.05329	0.04573	-0.09733	0.20361	0.02442	0.16021	-0.16021	0.26469	0.06917	0.48879	0.55254	0.20538	0.00756	0.7512	0.03633	0.03633	turquoise
LOXL2	-0.11245	-0.05303	0.02848	0.04819	-0.11112	0.08126	0.11619	-0.05964	0.05964	0.14309	0.49088	0.71156	0.53136	0.14791	0.29073	0.13017	0.4384	0.4384	black
DNAJC12	0.08546	-0.04063	-0.20213	-0.15124	0.20975	-0.24117	-0.24132	0.25602	-0.25602	0.26643	0.59777	0.00802	0.04831	0.0059	0.00148	0.00147	0.00073	0.00073	brown
IFT43	0.07654	-0.09782	-0.17094	-0.06235	0.21668	-0.21148	-0.06423	-0.24991	0.24991	0.31976	0.20307	0.02539	0.41788	0.00442	0.00549	0.40395	0.00098	0.00098	grey
ZBTB41	-0.00029	-0.15836	-0.07981	0.00797	-0.06025	-0.01336	0.01827	0.14891	-0.14891	0.99704	0.03858	0.29943	0.91761	0.43376	0.86232	0.8125	0.05191	0.05191	turquoise
SRA1	-0.0179	-0.06449	-0.13823	-0.08728	0.10998	-0.14033	0.03672	-0.19999	0.19999	0.81621	0.40203	0.07139	0.25634	0.15215	0.06714	0.63347	0.00873	0.00873	grey
SRSF8	-0.06673	-0.05778	-0.05909	-0.02428	-0.02845	0.01163	-0.10449	0.16228	-0.16228	0.38586	0.45284	0.44266	0.75258	0.71186	0.87997	0.1738	0.03396	0.03396	turquoise
ADRA1B	-0.10812	-0.1251	-0.07133	0.05383	-0.06501	-0.06038	0.06739	-0.07325	0.07325	0.15924	0.10303	0.3539	0.48435	0.39824	0.43271	0.38115	0.34102	0.34102	grey
SYCE3	-0.04857	0.11789	-0.14843	-0.00618	0.13506	-0.16516	0.06056	-0.27216	0.27216	0.52818	0.12463	0.05268	0.93605	0.0782	0.03087	0.43139	0.00032	0.00032	grey
BTG1	-0.00778	-0.04705	-0.05589	0.01782	-0.00993	-0.0857	-0.21619	0.26818	-0.26818	0.91956	0.54113	0.46777	0.8171	0.89744	0.26505	0.00451	0.00039	0.00039	blue
BVES	-0.06317	-0.02858	0.01153	0.07117	-0.039	-0.16	-0.11136	0.46603	-0.46603	0.41175	0.7106	0.88104	0.35494	0.61254	0.03658	0.14705	1.33E-10	1.33E-10	turquoise
LMO1	0.05681	-0.07691	-0.1862	-0.13674	0.05932	-0.27231	-0.10573	0.30687	-0.30687	0.46053	0.31736	0.01475	0.07452	0.44085	0.00031	0.16873	4.46E-05	4.46E-05	brown
PSMD13	0.01779	-0.04095	-0.00514	-0.06148	-0.03033	-0.01168	0.17413	-0.36835	0.36835	0.81737	0.59488	0.94685	0.42439	0.69376	0.87945	0.02274	7.16E-07	7.16E-07	grey
AKT1	0.0067	-0.06897	0.02702	0.09908	0.01528	0.03534	0.02692	-0.06858	0.06858	0.93074	0.37004	0.7257	0.19727	0.84277	0.64635	0.72672	0.37275	0.37275	turquoise
PDE11A	0.00198	0.01323	-0.05483	0.0564	0.02262	0.17122	0.02711	-0.14197	0.14197	0.97951	0.86366	0.47632	0.4637	0.76903	0.02515	0.72487	0.06399	0.06399	grey
SUCLG1	0.06671	0.10012	-0.12883	-0.11092	0.02574	-0.02886	0.10645	-0.15342	0.15342	0.386	0.19262	0.09308	0.14864	0.73822	0.70789	0.16582	0.04513	0.04513	grey
MX2	-0.02663	0.00244	0.10773	0.10723	-0.10174	0.15979	0.08117	-0.06107	0.06107	0.72953	0.97474	0.16077	0.16273	0.18548	0.03683	0.29126	0.42752	0.42752	tan
C5orf38	-0.01559	-0.11908	-0.23373	-0.14589	0.12983	-0.32612	-0.24089	0.26264	-0.26264	0.83963	0.12084	0.00209	0.05691	0.09056	1.34E-05	0.0015	0.00052	0.00052	brown
EPN3	0.06111	0.03687	0.09164	0.03978	0.02562	0.1995	0.24373	-0.42566	0.42566	0.42718	0.63207	0.23326	0.60548	0.73944	0.0089	0.00132	6.48E-09	6.48E-09	yellow
ATOH8	0.00254	-0.09408	-0.09065	0.0346	-0.03827	-0.11196	-0.06084	0.33172	-0.33172	0.97365	0.22098	0.23833	0.6532	0.61924	0.14485	0.42925	9.33E-06	9.33E-06	grey
DAAM1	0.02232	-0.15689	0.00987	0.05668	-0.12016	-0.05164	-0.09422	0.22469	-0.22469	0.77204	0.04044	0.89803	0.46155	0.11749	0.50233	0.22028	0.00313	0.00313	turquoise
NEIL1	-0.01097	-0.08393	-0.08596	-0.09063	0.25797	-0.08705	-0.1005	-0.12695	0.12695	0.88674	0.27512	0.26363	0.23845	0.00066	0.25759	0.19093	0.09799	0.09799	red
C16orf86	-0.0591	0.02284	-0.10923	-0.12909	-0.00968	-0.09001	-0.01651	0.16391	-0.16391	0.44262	0.76685	0.155	0.09242	0.89997	0.2417	0.83029	0.03218	0.03218	grey
GUSB	-0.00549	-0.07432	0.00943	-0.00524	0.15854	-0.06993	-0.01666	-0.03704	0.03704	0.94321	0.334	0.90255	0.94573	0.03835	0.36341	0.82873	0.63052	0.63052	grey
CDKN2AIF	0.01544	-0.13256	-0.23417	-0.15105	0.11296	-0.33399	-0.19192	0.12801	-0.12801	0.84112	0.08391	0.00205	0.04861	0.14127	8.03E-06	0.01191	0.09521	0.09521	brown
STAT6	0.04476	-0.05227	0.09646	0.13063	-0.05323	0.24738	0.12906	-0.2025	0.2025	0.56107	0.49713	0.20947	0.08857	0.4893	0.00111	0.09251	0.0079	0.0079	turquoise
HINFP	0.06495	-0.15672	-0.08935	-0.07976	-0.03293	-0.03793	0.06872	-0.20785	0.20785	0.39864	0.04066	0.24519	0.29975	0.669	0.62234	0.37179	0.00637	0.00637	grey
GABARAP	#######	-0.19861	-0.1926	-0.19293	0.0627	-0.16692	-0.09849	0.13484	-0.13484	0.99965	0.00921	0.01161	0.01146	0.41523	0.0291	0.19997	0.07868	0.07868	turquoise
ZNHIT2	0.00273	0.00347	-0.18299	-0.09392	0.22363	-0.16659	0.07507	-0.3127	0.3127	0.97169	0.96403	0.01659	0.22177	0.00328	0.02943	0.32916	3.13E-05	3.13E-05	green
CCL3L1	-0.09845	-0.05366	-0.07893	-0.01915	0.05159	-0.10406	-0.11993	0.29459	-0.29459	0.20018	0.48575	0.3048	0.80364	0.50279	0.1756	0.1182	9.18E-05	9.18E-05	grey
COA5	0.0155	-0.03445	-0.16246	-0.08288	0.06767	-0.15895	0.04666	-0.10509	0.10509	0.84055	0.65468	0.03375	0.28117	0.37917	0.03784	0.54454	0.17134	0.17134	grey
CLGN	-0.01304	-0.09952	-0.23628	-0.1009	0.05884	-0.3166	-0.2049	0.42696	-0.42696	0.86561	0.19532	0.00186	0.18914	0.44462	2.46E-05	0.00718	5.76E-09	5.76E-09	brown
JHY	-0.09249	-0.2058	-0.13742	-0.01465	-0.16416	-0.06459	-0.05877	0.20652	-0.20652	0.22889	0.00693	0.07309	0.8492	0.03191	0.40128	0.44514	0.00673	0.00673	turquoise
TLL1	0.01844	-0.12067	-0.01418	0.11751	-0.15397	0.08088	-0.06362	0.50497	-0.50497	0.81082	0.11592	0.854	0.12585	0.04437	0.29297	0.40845	1.88E-12	1.88E-12	blue
HOXB4	-0.04264	-0.08903	-0.01598	-0.02138	-0.02922	0.05441	0.0793	0.13734	-0.13734	0.57973	0.24689	0.83564	0.78134	0.70436	0.47972	0.30256	0.07325	0.07325	grey
HSPB6	-0.02534	-0.00058	-0.01838	0.05664	-0.06218	0.12331	0.00575	0.49462	-0.49462	0.74212	0.99401	0.8114	0.46185	0.41915	0.1081	0.94048	6.15E-12	6.15E-12	blue
CERS6	0.06541	-0.09338	0.06466	0.05701	-0.13327	0.11103	0.12698	-0.00606	0.00606	0.39533	0.22442	0.40077	0.4589	0.08226	0.14826	0.09793	0.93733	0.93733	turquoise
PHKA2	-0.09077	-0.17702	-0.21099	-0.08824	0.23682	-0.15521	-0.08343	-0.02992	0.02992	0.23773	0.02055	0.0056	0.2511	0.00182	0.04266	0.278	0.69768	0.69768	grey
HYKK	0.09168	-0.05549	-0.10231	-0.12431	0.11774	-0.00504	0.04355	-0.08368	0.08368	0.23303	0.47097	0.18302	0.10524	0.12511	0.94783	0.57171	0.27652	0.27652	grey
IL18RAP	-0.06946	-0.08115	-0.01573	-0.00676	-0.12244	0.03891	-0.03989	0.37324	-0.37324	0.36664	0.29134	0.83821	0.93012	0.11063	0.61335	0.6045	4.96E-07	4.96E-07	blue
PRDM2	-0.07821	-0.1585	0.01212	0.09359	-0.17307	0.14558	-0.03391	0.36225	-0.36225	0.30923	0.0384	0.87495	0.22338	0.02359	0.05744	0.65974	1.12E-06	1.12E-06	blue
TBC1D30	0.082	0.03079	-0.09964	-0.01193	0.22341	-0.08416	-0.11542	-0.10493	0.10493	0.28632	0.68936	0.19478	0.87688	0.00331	0.2738	0.13275	0.17201	0.17201	grey
TRMT2A	-0.03852	0.03332	-0.11385	-0.1061	0.19041	-0.12808	0.01691	-0.20427	0.20427	0.61693	0.66531	0.13815	0.16724	0.01261	0.09504	0.8262	0.00736	0.00736	red
SREK1IP1	-0.04733	-0.06859	-0.07841	-0.1044	0.00186	-0.13458	-0.08688	0.12087	-0.12087	0.53872	0.37274	0.30804	0.17418	0.98075	0.07926	0.25853	0.11531	0.11531	turquoise
SCAMP4	0.03351	-0.13506	-0.18369	-0.06076	0.14327	-0.2666	-0.08369	-0.09226	0.09226	0.66351	0.07819	0.01618	0.42987	0.06156	0.00042	0.2765	0.23005	0.23005	green
TRIP11	-0.01037	-0.13781	-0.06743	0.07114	-0.08624	0.03463	-0.03163	0.30199	-0.30199	0.89296	0.07226	0.38089	0.35514	0.26208	0.65295	0.68131	5.97E-05	5.97E-05	turquoise
FAM107B	-0.09193	-0.00114	0.07953	0.06069	-0.03943	0.16559	-0.03635	-0.02098	0.02098	0.23174	0.98822	0.30116	0.43042	0.60863	0.03043	0.63696	0.78531	0.78531	turquoise
TDG	0.01703	-0.11197	0.00805	-0.00102	0.0677	-0.03769	-0.01781	0.11068	-0.11068	0.82506	0.14482	0.91674	0.98948	0.37899	0.62451	0.81719	0.14953	0.14953	turquoise
NOS3	-0.10713	-0.00637	-0.00522	0.04253	-0.08076	0.07791	-0.08174	0.33758	-0.33758	0.16311	0.93411	0.946	0.58075	0.2937	0.31113	0.28787	6.32E-06	6.32E-06	blue
ABL1	-0.10549	-0.157	-0.04796	0.06104	-0.11675	0.00874	-0.0808	0.40943	-0.40943	0.16971	0.04029	0.53337	0.42776	0.12834	0.90967	0.29344	2.69E-08	2.69E-08	turquoise
VPS37D	0.08998	-0.15967	-0.23817	-0.01617	0.28816	-0.23408	-0.16347	0.00042	-0.00042	0.24183	0.03697	0.00171	0.83375	0.00013	0.00206	0.03265	0.99567	0.99567	grey
LRIG3	0.00717	-0.07214	-0.03352	0.05711	0.00328	0.07722	0.03458	-0.01688	0.01688	0.9258	0.34841	0.66341	0.45809	0.96609	0.31545	0.65338	0.82657	0.82657	turquoise
FAM111A	-0.03828	-0.03792	-0.00903	0.01888	-0.08435	0.17766	0.21678	-0.18826	0.18826	0.61915	0.62242	0.90671	0.80638	0.27269	0.02009	0.0044	0.01367	0.01367	turquoise
FHL2	0.04514	-0.00204	0.14431	0.05952	-0.10306	0.25412	0.22611	-0.35879	0.35879	0.55767	0.97892	0.05969	0.43931	0.17981	0.0008	0.00294	1.44E-06	1.44E-06	yellow
NFS1	0.04622	-0.09321	-0.15385	-0.04038	0.13744	-0.16152	-0.00515	-0.04583	0.04583	0.5483	0.22528	0.04453	0.60004	0.07304	0.03481	0.94667	0.55169	0.55169	turquoise
HIST1H3E	0.09583	-0.17747	0.14139	-0.07381	0.00183	0.16632	0.12171	-0.10386	0.10386	0.21244	0.02022	0.06508	0.33737	0.98105	0.0297	0.11279	0.17641	0.17641	grey
ZNF121	-0.03568	-0.07728	-0.08184	-0.042	0.09819	-0.0386	-0.01042	-0.30377	0.30377	0.64313	0.31503	0.28729	0.58542	0.20136	0.61619	0.89239	5.37E-05	5.37E-05	grey
BORA	0.03344	0.02188	-0.00557	-0.01948	-0.02617	0.05808	0.1839	-0.28375	0.28375	0.66413	0.7764	0.9424	0.8004	0.734	0.45054	0.01605	0.00017	0.00017	pink
DTD1	0.0644	-0.03016	-0.22322	-0.15626	0.07743	-0.33326	-0.06148	-0.04983	0.04983	0.40269	0.69538	0.00334	0.04126	0.31413	8.43E-06	0.42437	0.51748	0.51748	grey
ZNF836	0.05387	-0.10004	-0.12931	-0.04098	-0.05752	0.05722	-0.05067	0.05187	-0.05187	0.4841	0.19294	0.09186	0.59464	0.4549	0.45727	0.51041	0.5005	0.5005	turquoise
CENPV	0.02308	0.02582	0.03691	-0.03989	0.13719	-0.02923	0.10052	-0.2947	0.2947	0.76449	0.73748	0.63172	0.60446	0.07357	0.70432	0.19082	9.13E-05	9.13E-05	grey
URGCP	-0.02597	-0.20965	-0.10794	-0.01643	0.11758	-0.12893	-0.07257	-0.08249	0.08249	0.73602	0.00592	0.15995	0.83111	0.12562	0.09284	0.34555	0.28344	0.28344	grey
MEN1	-0.017	-0.08709	-0.03113	0.03268	-0.02678	-0.03986	0.14278	-0.17768	0.17768	0.82537	0.25734	0.68605	0.6713	0.72808	0.60468	0.06248	0.02008	0.02008	grey
CKS2	0.02898	0.03392	0.00309	-0.03826	-0.08929	0.09002	0.29176	-0.48166	0.48166	0.70668	0.65966	0.96799	0.61929	0.24548	0.24166	0.00011	2.57E-11	2.57E-11	pink
MITD1	0.03717	0.01649	0.08474	0.04941	-0.05479	0.12605	0.17466	-0.19899	0.19899	0.62934	0.83049	0.27049	0.52103	0.47664	0.10044	0.02233	0.00908	0.00908	turquoise
SIL1	0.08711	-0.04865	-0.13716	-0.06892	0.22797	-0.30959	-0.12152	0.0028	-0.0028	0.25728	0.52747	0.07363	0.37042	0.00271	3.78E-05	0.11335	0.97099	0.97099	grey
SRR	-0.20644	-0.15312	-0.20959	-0.05364	-0.02993	-0.20938	-0.15012	0.51348	-0.51348	0.00675	0.04556	0.00594	0.48595	0.69756	0.00599	0.05002	6.89E-13	6.89E-13	brown
SERPINA1	0.02021	-0.0727	-0.01836	0.09247	0.14036	-0.09751	-0.12238	-0.11187	0.11187	0.79303	0.34469	0.81166	0.22903	0.06709	0.2045	0.11082	0.1452	0.1452	grey
PPRC1	0.00165	-0.04795	0.04719	0.01994	-0.12881	0.14136	0.18422	-0.05089	0.05089	0.98296	0.53344	0.53991	0.79569	0.09315	0.06514	0.01586	0.50862	0.50862	turquoise
HECTD2	-0.07626	-0.21318	-0.08424	0.07024	-0.0473	-0.10505	-0.08169	0.54183	-0.54183	0.32154	0.00512	0.27329	0.3613	0.539	0.17149	0.28814	1.97E-14	1.97E-14	turquoise
MTRF1L	-0.03572	-0.16876	-0.00771	0.04971	-0.15757	-0.00054	0.00338	0.24508	-0.24508	0.64276	0.02735	0.92032	0.51846	0.03956	0.99441	0.96503	0.00123	0.00123	turquoise
NANS	0.13804	0.04282	0.07683	0.03606	-0.02422	0.12345	0.23332	-0.36902	0.36902	0.07178	0.57815	0.31788	0.63961	0.75323	0.10769	0.00213	6.82E-07	6.82E-07	pink
MFSD1	-0.09042	-0.0785	-0.01146	0.0452	-0.19516	0.11373	0.0855	0.33355	-0.33355	0.23953	0.30745	0.88178	0.5572	0.01053	0.13858	0.26619	8.27E-06	8.27E-06	blue
SMU1	-0.02264	-0.10722	-0.14911	-0.05834	-0.09636	-0.08837	-0.07444	0.21062	-0.21062	0.76884	0.16275	0.0516	0.44851	0.20994	0.25041	0.33321	0.00569	0.00569	turquoise
KCTD13	0.04082	-0.12596	-0.12581	-0.10581	0.21466	-0.14958	-0.09866	-0.12042	0.12042	0.59604	0.10066	0.10109	0.1684	0.00481	0.05087	0.1992	0.11668	0.11668	red
MMGT1	-0.01014	-0.15084	-0.0637	-0.02918	-0.15218	0.01501	-0.00175	0.22737	-0.22737	0.89529	0.04891	0.4078	0.70478	0.04691	0.84551	0.98184	0.00278	0.00278	turquoise
DUOXA2	-0.06426	0.01161	0.11108	0.04968	0.14878	0.18353	0.06513	-0.26556	0.26556	0.40372	0.88019	0.14807	0.51878	0.05212	0.01627	0.39738	0.00045	0.00045	grey
MTHFSD	-0.01765	0.00637	-0.12145	-0.08452	0.08706	-0.00698	0.05511	-0.22201	0.22201	0.81872	0.93408	0.11356	0.27172	0.25751	0.92783	0.47404	0.00352	0.00352	grey
SEMA6C	-0.05432	-0.14765	-0.1177	0.02785	0.07312	-0.18722	-0.01022	0.35881	-0.35881	0.48042	0.05396	0.12522	0.71767	0.34188	0.01421	0.89443	1.44E-06	1.44E-06	grey
ZSWIM4	-0.03151	-0.22779	-0.0517	0.05065	-0.04533	0.0016	0.03163	-0.10307	0.10307	0.68246	0.00273	0.5019	0.51057	0.55601	0.98341	0.68126	0.17974	0.17974	grey
DCP1B	0.06262	-0.01463	-0.11356	-0.00426	0.08026	-0.16767	-0.16681	0.12901	-0.12901	0.41584	0.84933	0.13915	0.95589	0.29669	0.02837	0.02921	0.09263	0.09263	turquoise
L3MBTL3	-0.03808	-0.06488	0.03088	0.05857	-0.17443	0.13869	0.02983	0.14932	-0.14932	0.62096	0.39918	0.68849	0.4467	0.0225	0.07045	0.69848	0.05127	0.05127	turquoise
PPIH	0.01308	0.07332	0.00318	-0.03652	-0.11064	0.07835	0.15635	-0.28565	0.28565	0.86513	0.34057	0.96709	0.63533	0.14968	0.30841	0.04115	0.00015	0.00015	pink
MAGOH	-0.05848	-0.02688	0.04586	-0.01286	0.00029	0.04108	0.07454	-0.21372	0.21372	0.4474	0.72714	0.55142	0.86746	0.99695	0.59366	0.3326	0.005	0.005	turquoise

BCOR	-0.03059	-0.17034	-0.09342	-0.01102	0.03633	-0.0135	-0.1212	0.17914	-0.17914	0.69128	0.02591	0.22423	0.88629	0.63714	0.8609	0.11431	0.01906	0.01906	turquoise
BPIFB1	-0.02184	0.05116	0.08274	-0.00128	0.02432	0.15244	0.13821	-0.19222	0.19222	0.7768	0.50632	0.282	0.98674	0.75222	0.04655	0.07143	0.01178	0.01178	grey
CLDN15	-0.09264	-0.07204	-0.05633	0.01224	0.21356	-0.0456	-0.07127	0.08424	-0.08424	0.22814	0.34906	0.46431	0.87377	0.00504	0.55369	0.35426	0.2733	0.2733	grey
CHCHD2	0.03739	-0.01486	-0.06643	-0.03207	0.0993	-0.089	0.05966	-0.41721	0.41721	0.62733	0.84703	0.38799	0.67714	0.19628	0.24702	0.43827	1.37E-08	1.37E-08	grey
LGALS9	0.00932	0.10679	0.06586	0.0848	-0.06734	0.21159	0.24457	-0.25855	0.25855	0.90366	0.16447	0.39209	0.27014	0.38154	0.00547	0.00127	0.00064	0.00064	yellow
CNOT10	-0.00142	-0.07233	-0.04453	-0.1038	-0.11373	0.07867	0.14949	-0.02809	0.02809	0.98532	0.34713	0.56308	0.17668	0.13859	0.30644	0.05101	0.71533	0.71533	turquoise
PYGM	-0.11187	-0.00274	-0.00855	0.10334	-0.01126	0.01045	-0.06778	0.41171	-0.41171	0.1452	0.9716	0.9116	0.17861	0.88376	0.8921	0.37838	2.21E-08	2.21E-08	salmon
VIM	-0.12565	-0.08381	0.0422	0.09149	-0.14288	0.0856	-0.06468	0.31922	-0.31922	0.10151	0.27578	0.58365	0.23399	0.06229	0.26563	0.4006	2.08E-05	2.08E-05	black
LRRFIP1	-0.02146	-0.05566	0.03742	0.03278	-0.05313	0.08047	0.03949	-0.0799	0.0799	0.78057	0.46961	0.62706	0.67035	0.49012	0.29543	0.60812	0.29891	0.29891	turquoise
KIF2C	-0.03716	0.0728	0.06115	-0.04775	-0.08016	0.11593	0.32157	-0.42052	0.42052	0.62946	0.344	0.42686	0.53515	0.29729	0.13107	1.80E-05	1.03E-08	1.03E-08	pink
TEX10	-0.01879	-0.07682	-0.076	-0.03277	-0.15883	0.01849	0.04174	0.16455	-0.16455	0.80725	0.31797	0.32314	0.67044	0.03799	0.8103	0.58775	0.0315	0.0315	turquoise
FMO4	-0.04718	-0.03656	0.0201	0.00614	-0.14489	0.27404	0.14194	0.07495	-0.07495	0.54005	0.63494	0.79409	0.93649	0.05865	0.00029	0.06405	0.3299	0.3299	turquoise
HES4	0.03689	-0.04021	-0.00921	0.03534	0.0379	0.02047	0.15133	-0.27522	0.27522	0.63192	0.60158	0.90485	0.64629	0.62264	0.79044	0.04818	0.00027	0.00027	green
OXR1	-0.05749	-0.07897	0.03798	0.05225	-0.17331	0.19123	0.1644	-0.00854	0.00854	0.45515	0.30459	0.62192	0.49731	0.02339	0.01223	0.03166	0.91173	0.91173	turquoise
ZNF764	-0.05741	-0.14403	-0.13246	-0.06141	0.11104	-0.04881	-0.06228	0.01246	-0.01246	0.45577	0.06019	0.08415	0.42495	0.14823	0.52606	0.41838	0.8715	0.8715	red
ZNF571	-0.03542	-0.14006	-0.0748	0.00937	0.03787	-0.03841	-0.13585	0.27837	-0.27837	0.64559	0.06769	0.33086	0.90317	0.62287	0.61791	0.07644	0.00023	0.00023	turquoise
PPP2R2C	-0.18884	-0.07279	-0.13526	0.01172	0.02895	-0.25678	0.03071	0.00463	-0.00463	0.01338	0.34405	0.07776	0.87904	0.70701	0.0007	0.69009	0.95203	0.95203	grey
LSG1	0.01672	-0.01911	-0.03957	-0.05519	-0.08059	0.08356	0.12736	-0.15798	0.15798	0.82821	0.80411	0.6074	0.47337	0.29469	0.27723	0.09691	0.03904	0.03904	turquoise
PCLAF	-0.05291	0.11755	0.02748	-0.0052	0.01612	0.06577	0.30936	-0.43977	0.43977	0.49189	0.12571	0.72127	0.94623	0.8342	0.39274	3.83E-05	1.77E-09	1.77E-09	pink
RNF213	-0.05179	-0.12367	0.03749	0.07784	-0.16358	0.12253	0.14878	0.04258	-0.04258	0.50115	0.10706	0.62634	0.31153	0.03253	0.11036	0.05213	0.5803	0.5803	turquoise
FAM32A	0.01323	-0.04883	-0.06589	-0.00225	0.00289	-0.01376	0.08213	-0.17557	0.17557	0.86369	0.5259	0.39186	0.9767	0.97008	0.85825	0.28553	0.02162	0.02162	turquoise
ADM5	-0.0168	-0.00168	-0.08667	0.07558	0.15324	-0.13276	-0.01458	0.18874	-0.18874	0.82738	0.98257	0.2597	0.32589	0.04539	0.08345	0.84983	0.01342	0.01342	grey
STAT5A	-0.1024	0.01073	0.01161	0.01711	-0.1494	0.11	0.06066	0.25649	-0.25649	0.18263	0.88925	0.88023	0.82421	0.05114	0.15209	0.43061	0.00071	0.00071	blue
REC8	-0.02401	-0.11016	-0.17777	-0.07664	0.23998	-0.29993	-0.14749	0.1142	-0.1142	0.75528	0.15149	0.02001	0.31908	0.00157	6.74E-05	0.05421	0.13694	0.13694	grey
MTHFS	0.00352	-0.10661	-0.04446	0.04886	0.20936	-0.1111	-0.03634	-0.22016	0.22016	0.96356	0.16519	0.56367	0.52566	0.00599	0.14799	0.63697	0.00381	0.00381	grey
ARTN	-0.01978	-0.04494	-0.0808	-0.08793	-0.03273	-0.11146	0.07397	-0.03267	0.03267	0.79733	0.55941	0.29349	0.25278	0.67084	0.14668	0.33631	0.67145	0.67145	grey
KIAA1522	0.06081	-0.0254	0.10814	0.0764	-0.04313	0.2216	0.2063	-0.46225	0.46225	0.42948	0.74159	0.15917	0.32064	0.57539	0.00358	0.00679	1.96E-10	1.96E-10	yellow
CD93	-0.14713	-0.12792	-0.02336	0.07483	-0.17794	0.08318	-0.05675	0.53759	-0.53759	0.05482	0.09543	0.76167	0.33073	0.01989	0.27943	0.46096	3.42E-14	3.42E-14	blue
C11orf68	0.00172	-0.04184	-0.03014	0.01358	-0.11233	0.09666	0.13929	-0.09466	0.09466	0.98219	0.5869	0.69559	0.86005	0.14353	0.2085	0.06922	0.2181	0.2181	grey
ATG9B	0.11837	0.00581	-0.0091	-0.00379	0.15365	-0.12058	0.01499	-0.31179	0.31179	0.12308	0.93989	0.90592	0.96078	0.04481	0.11619	0.84568	3.31E-05	3.31E-05	grey
B9D1	0.02871	-0.07113	-0.22834	-0.14244	0.23394	-0.39283	-0.18303	0.15767	-0.15767	0.70931	0.35522	0.00267	0.06309	0.00207	1.07E-07	0.01657	0.03944	0.03944	brown
AFG3L2	-0.01141	-0.09262	-0.00066	0.0203	0.01567	0.0611	0.11024	-0.03205	0.03205	0.88229	0.22828	0.99312	0.7921	0.83878	0.42731	0.15119	0.67729	0.67729	turquoise
ZMPSTE24	-0.01012	-0.10349	-0.01066	0.06962	-0.07271	0.06137	-0.02176	0.13079	-0.13079	0.89544	0.178	0.88993	0.36558	0.34459	0.42519	0.77754	0.08817	0.08817	turquoise
STK39	0.06201	0.02152	0.13085	0.07404	-0.05343	0.20626	0.1584	-0.38729	0.38729	0.42042	0.77998	0.08803	0.33582	0.48766	0.0068	0.03853	1.67E-07	1.67E-07	yellow
REP15	0.08199	0.04836	0.06249	-0.01816	0.12862	0.1558	0.06871	-0.19374	0.19374	0.28636	0.52993	0.41681	0.81364	0.09363	0.04186	0.37187	0.01112	0.01112	grey
MT-ND2	-0.02115	-0.08604	-0.02222	-0.03689	0.18985	-0.00777	-0.10642	-0.13557	0.13557	0.78365	0.26317	0.77295	0.63192	0.01288	0.91965	0.16595	0.07707	0.07707	grey
ATXN10	-0.08052	-0.06109	-0.16979	0.00474	0.04437	-0.25819	-0.07529	0.31258	-0.31258	0.29516	0.42735	0.02641	0.95094	0.56445	0.00065	0.32775	3.15E-05	3.15E-05	turquoise
TAGAP	-0.09675	-0.02979	0.08283	0.06283	-0.1976	0.14539	0.02722	0.42855	-0.42855	0.20809	0.69895	0.28145	0.41427	0.00958	0.05777	0.7238	4.99E-09	4.99E-09	blue
SCNN1A	0.14085	0.0183	0.15014	0.09032	-0.08445	0.27042	0.21199	-0.46212	0.46212	0.06613	0.81225	0.04999	0.24007	0.2721	0.00035	0.00538	1.98E-10	1.98E-10	yellow
THOC2	-0.03238	-0.14304	-0.01798	-0.00463	-0.047	0.04971	0.0103	0.08858	-0.08858	0.67416	0.062	0.81543	0.95209	0.54157	0.51852	0.89359	0.24925	0.24925	turquoise
AP2A1	0.02019	-0.02965	-0.04758	-0.03601	-0.01586	0.02781	0.07561	-0.09161	0.09161	0.79329	0.70025	0.5366	0.64003	0.83686	0.71808	0.32564	0.23337	0.23337	grey
PEX11B	0.01277	-0.02436	-0.03871	-0.07692	-0.00831	0.01571	0.12698	-0.25704	0.25704	0.86839	0.75183	0.61519	0.31732	0.91411	0.83841	0.09792	0.00069	0.00069	turquoise
NARS	-0.07676	-0.10654	-0.08538	-0.00012	0.09265	-0.05504	-0.04443	0.20834	-0.20834	0.31831	0.16545	0.26686	0.99873	0.22808	0.47459	0.56393	0.00625	0.00625	turquoise
DPY30	-0.01166	-0.04268	0.02324	0.01147	-0.08191	0.03079	0.17379	-0.32947	0.32947	0.8797	0.57942	0.76287	0.88163	0.28688	0.68928	0.02301	1.08E-05	1.08E-05	turquoise
RESF1	-0.02189	-0.08243	0.14122	0.06464	-0.10613	0.22958	0.08314	-0.07536	0.07536	0.77628	0.28382	0.06542	0.40095	0.1671	0.00252	0.27964	0.3273	0.3273	turquoise
TAF5L	-0.04704	-0.0506	-0.05636	-0.04945	-0.05625	0.10763	0.08253	-0.01934	0.01934	0.5412	0.51105	0.46404	0.52072	0.46492	0.16116	0.28321	0.80177	0.80177	turquoise
SNX18	-0.04836	-0.1783	-0.03825	-0.00283	-0.15553	0.04021	-0.04272	0.3896	-0.3896	0.5299	0.01964	0.6194	0.9707	0.04222	0.60155	0.57906	1.39E-07	1.39E-07	blue
C19orf57	-0.09274	0.02983	0.05027	0.13838	-0.02184	0.12495	0.24353	-0.16524	0.16524	0.22762	0.69851	0.51377	0.07109	0.77673	0.10345	0.00133	0.03079	0.03079	grey
PYURF	-0.12349	0.06164	-0.02302	-0.01594	-0.02354	-0.13945	-0.1503	0.17404	-0.17404	0.10759	0.42316	0.76506	0.83605	0.75992	0.06891	0.04975	0.02281	0.02281	grey
AGTPBP1	-0.08594	-0.00394	-0.04073	-0.01295	-0.17658	0.03124	-0.04771	0.33319	-0.33319	0.2637	0.95917	0.59688	0.86651	0.02087	0.68504	0.53548	8.47E-06	8.47E-06	turquoise
FANCI	-0.0532	0.01552	0.04074	0.00039	-0.0376	0.05148	0.26116	-0.32462	0.32462	0.4895	0.84036	0.59672	0.99592	0.62533	0.50371	0.00056	1.48E-05	1.48E-05	pink
MRPL11	0.06549	0.10186	-0.02556	-0.02628	-0.06123	0.05287	0.2928	-0.4114	0.4114	0.39472	0.18496	0.73997	0.73299	0.4263	0.49225	0.0001	2.27E-08	2.27E-08	grey
GCNT1	0.03984	-0.12856	0.06384	-0.01828	-0.03633	0.00596	-0.0178	-0.00903	0.00903	0.60494	0.09379	0.40679	0.81238	0.63714	0.9383	0.81722	0.90671	0.90671	grey
KLHL30	0.17981	0.16202	-0.06702	0.08022	0.10976	0.07628	0.1918	-0.29901	0.29901	0.01861	0.03425	0.38378	0.29695	0.15299	0.32141	0.01197	7.11E-05	7.11E-05	grey
CRK	-0.11052	-0.14038	-0.01671	0.03998	-0.10631	0.06433	0.072	0.25183	-0.25183	0.15012	0.06705	0.82827	0.60359	0.16639	0.40322	0.34937	0.00089	0.00089	turquoise
NEDD9	-0.10704	-0.14048	-0.02897	-0.02072	-0.01628	0.18632	-0.13814	0.21305	-0.21305	0.16347	0.06686	0.70681	0.7879	0.83267	0.01469	0.07158	0.00515	0.00515	turquoise
GJC3	0.077	0.0454	-0.05389	-0.01819	0.14525	-0.03271	-0.12491	-0.03151	0.03151	0.31683	0.55545	0.48394	0.81337	0.05802	0.67109	0.10358	0.6824	0.6824	grey
PRR15L	0.0214	0.02325	-0.00317	0.01825	0.1471	0.21823	0.02745	-0.33141	0.33141	0.78118	0.7628	0.96721	0.81272	0.05486	0.00414	0.72153	9.52E-06	9.52E-06	yellow
ABCD3	-0.04135	-0.14246	-0.00067	0.01217	0.133	-0.00831	-0.0528	0.06986	-0.06986	0.59123	0.06306	0.99306	0.87451	0.08288	0.91414	0.49282	0.36388	0.36388	turquoise
PPP1R9A	0.01122	-0.19186	-0.08033	0.00387	0.1618	-0.06794	-0.08661	0.04833	-0.04833	0.88425	0.01194	0.29628	0.95992	0.03449	0.37726	0.25999	0.53022	0.53022	grey
COX7B	0.08616	0.00908	-0.13096	-0.13767	0.16812	-0.08647	0.1055	-0.40639	0.40639	0.26252	0.9062	0.08776	0.07255	0.02795	0.26078	0.16965	3.49E-08	3.49E-08	grey
PRKAA1	-0.066	-0.0879	0.02348	0.00352	-0.06069	0.13739	0.05658	-0.03136	0.03136	0.39111	0.25293	0.76048	0.96355	0.4304	0.07315	0.46235	0.68389	0.68389	turquoise
ANXA2	-0.00609	0.02838	0.11821	0.09084	-0.09499	0.24191	0.25325	-0.42245	0.42245	0.93702	0.71257	0.12359	0.23737	0.21652	0.00143	0.00083	8.64E-09	8.64E-09	yellow
COL9A3	-0.00804	0.02458	0.02279	0.04641	0.01861	0.14001	0.05554	-0.0346	0.0346	0.91689	0.74963	0.76729	0.54669	0.8091	0.06778	0.47058	0.65326	0.65326	grey
NAA80	-0.06854	0.02615	-0.04884	-0.1054	0.03218	0.01835	0.02301	-0.21445	0.21445	0.37304	0.73426	0.52589	0.17005	0.67611	0.81167	0.7651	0.00485	0.00485	grey
TLCD5	0.00423	-0.2271	-0.10397	-0.00643	-0.03896	-0.05906	-0.11056	0.21359	-0.21359	0.95618	0.00282	0.17596	0.93345	0.61293	0.44286	0.14997	0.00503	0.00503	turquoise
MON2	-0.01099	-0.20442	-0.12012	-0.01789	0.07168	-0.1129	-0.1229	0.27592	-0.27592	0.88654	0.00732	0.11762	0.8164	0.35151	0.14149	0.10928	0.00026	0.00026	turquoise
CLDN9	0.04739	-0.22171	-0.14994	-0.06268	0.13574	-0.15361	-0.12142	-0.13208	0.13208	0.53825	0.00357	0.05029	0.41538	0.07668	0.04486	0.11365	0.08506	0.08506	grey
PEAK1	-0.07624	-0.14597	0.03336	0.08515	-0.16505	0.16131	0.06912	0.37161	-0.37161	0.32164	0.05677	0.6649	0.26818	0.03098	0.03505	0.369	5.61E-07	5.61E-07	turquoise
UNC13A	-0.02835	-0.15816	-0.2468	-0.1194	0.11502	-0.31656	-0.2696	0.44719	-0.44719	0.71282	0.03882	0.00114	0.11984	0.13411	2.46E-05	0.00036	8.70E-10	8.70E-10	brown
NCKAP5L	-0.03983	-0.18835	-0.08263	0.03533	-0.06437	-0.05328	-0.08488	0.30377	-0.30377	0.60499	0.01362	0.28261	0.64642	0.4029	0.48887	0.26969	5.37E-05	5.37E-05	blue
NBL1	-0.00286	0.01359	0.00737	0.02604	-0.03613	0.09516	0.11208	-0.08205	0.08205	0.97042	0.85994	0.92379	0.73533	0.63893	0.21571	0.14444	0.28605	0.28605	black
FRRS1	0.00752	-0.1337	0.08373	0.08377	-0.1233	0.2486	0.21886	-0.0573	0.0573	0.92221	0.08126	0.27626	0.276	0.10811	0.00104	0.00403	0.45666	0.45666	turquoise
RIF1	-0.01479	-0.12746	-0.00699	0.07341	-0.20353	0.09868	0.09361	0.12729	-0.12729	0.84776	0.09665	0.92772	0.34001	0.00759	0.19913	0.22332	0.09709	0.09709	turquoise
QTRT2	-0.03571	-0.08293	-0.02575	-0.00668	-0.13261	0.1582	0.09039	-0.04105	0.04105	0.64289	0.28087	0.73818	0.93094	0.08381	0.03877	0.23969	0.59399	0.59399	turquoise
GBA2	0.06594	-0.12734	-0.07855	-0.11069	0.2105	-0.06775	-0.05682	-0.01656	0.01656	0.39148	0.09696	0.30714	0.14951	0.00572	0.3786	0.46041	0.82975	0.82975	grey
GNAS	0.08364	-0.12713	-0.16455	-0.08541	0.09198	-0.32375	-0.24102	0.23703	-0.23703	0.27676	0.09751	0.0315	0.26666	0.2315	1.56E-05	0.0015	0.0018	0.0018	brown
TM9SF4	0.05217	-0.07785	0.01996	0.01484	0.02975	0.00295	0.03346	-0.16429	0.16429	0.49799	0.31152	0.79552	0.84719	0.69927	0.96944	0.66399	0.03177	0.03177	turquoise
LBP	-0.00154	-0.00907	-0.00151	0.02909	-0.00642	0.00427	-0.11298	0.30535	-0.30535	0.98407	0.90624	0.98432	0.70567	0.93361	0.95584	0.14123	4.88E-05	4.88E-05	blue
FCHO1	0.03399	-0.005	0.10616	0.09342	-0.02542	0.11499	0.09752	-0.198	0.198	0.65892	0.9482	0.167	0.22425	0.74134	0.13422	0.20449	0.00943	0.00943	grey
INMT	-0.031	-0.05591	-0.00319	0.04004	-0.15057	0.16449	-0.06519	0.43163	-0.43163	0.68728	0.46766	0.96694	0.60306	0.04933	0.03157	0.39694	3.76E-09	3.76E-09	blue
MPZL3	0.02706	-0.11187	-0.02908	-0.02459	-0.03956	0.14261	0.11999	-0.31831	0.31831	0.72532	0.14518	0.70575	0.74951	0.60743	0.06279	0.11801	2.21E-05	2.21E-05	turquoise
ACTR3C	0.06324	-0.13598	0.06391	0.10234	0.06306	-0.05496	0.02501	-0.04115	0.04115	0.41121	0.07616	0.4063	0.18288	0.41254	0.47524	0.74537	0.59306	0.59306	grey
ATP8A2	0.03305	-0.12577	-0.2374	-0.18873	0.04648	-0.29523	-0.22178	0.43481	-0.43481	0.66783	0.10118	0.00177	0.01343	0.54605	8.85E-05	0.00355	2.81E-09	2.81E-09	brown
LST1	-0.11993	-0.01562	0.04156	0.03377	-0.183	0.13224	0.06105	0.32784	-0.32784	0.11818	0.83934	0.58939	0.661	0.01659	0.08469	0.42767	1.20E-05	1.20E-05	blue
CHUK	0.00674	-0.12056	-0.0136	-0.00501	-0.04236	-0.11947	0.03364	0.24667	-0.24667	0.93026	0.11624	0.85983	0.94817	0.58228	0.11962	0.66226	0.00114	0.00114	turquoise
ADGRF1	0.04866	-0.01307	0.04166	-0.0283	-0.04788	0.12912	0.19069	-0.30395	0.30395	0.52737	0.86525	0.58846	0.7133	0.53401	0.09236	0.01248	5.31E-05	5.31E-05	yellow
ICE1	-0.06841	-0.18082	-0.05505	0.03755	-0.13474	-0.02625	0.01327	0.30388	-0.30388	0.37399	0.01795	0.47456	0.62582	0.07892	0.73323	0.86326	5.33E-05	5.33E-05	turquoise
NUMBL	-0.0159	-0.23366	-0.05086	0.02553	-0.07658	0.00076	0.00917	0.2223	-0.2223	0.83649	0.0021	0.50881	0.7403	0.31951	0.99212	0.90523	0.00347	0.00347	black
DEGS2	0.06936	0.07429	0.03271	0.04784	0.26663	0.12021	0.02596	-0.28964	0.28964	0.36737	0.33418	0.67108	0.53433	0.00042	0.11732	0.73611	0.00012	0.00012	grey
CCDC97	0.04976	-0.10717	-0.10388	-0.02164	-0.02736	0.00377	-0.0489	0.08884	-0.08884	0.51804	0.16297	0.17636	0.77872	0.72243	0.961	0.52532	0.2479	0.2479	turquoise
C2orf69	-0.09777	-0.12086	0.01218	0.09372	-0.07051	0.02626	-0.02857	0.20548	-0.20548	0.20332	0.11533	0.8744	0.22275	0.35942	0.73312	0.71065	0.00701	0.00701	turquoise
CLK1	0.00894	-0.20363	-0.00997	0.02642	0.15764	0.02305	-0.05891	0.02449	-0.02449	0.90758	0.00755	0.89703	0.7316	0.03948	0.76472	0.44403	0.75056	0.75056	red
PTPRF	0.06765	-0.09519	0.02868	0.0348	-0.00074	0.07127	0.07283	-0.25683	0.25683	0.37929	0.21556	0.70966	0.65139	0.9923	0.35425	0.34381	0.0007	0.0007	turquoise
FCRL5	-0.17871	0.04244	0.04427	-0.02766	-0.14171	0.04277	-0.00516	0.36444	-0.36444	0.01936	0.58155	0.56534	0.71947	0.06448	0.57856	0.94658	9.57E-07	9.57E-07	blue
RFT1	-0.05595	-0.02012	-0.07341	-0.01624	-0.12812	0.09432	0.08703	-0.04963	0.04963	0.46735	0.79392	0.33996	0.833	0.09492	0.21978	0.2577	0.51919	0.51919	turquoise
SETD6	0.00292	-0.01704	-0.13126	-0.07763	0.13506	-0.09783	0.06415	-0.10143	0.10143	0.96978	0.82496	0.08704	0.31288	0.07819	0.20302	0.4045	0.18683	0.18683	red
KBTBD4	-0.07265	-0.12125	-0.08214	-0.04129	-0.07721	-0.01158	-0.06108	0.27375	-0.27375	0.34501	0.11415	0.28549	0.59179	0.31553	0.88051	0.42739	0.00029	0.00029	turquoise
IL3RA	-0.00386	-0.05256	-0.09802	-0.03701	0.00617	-0.03531	-0.04403	0.41231	-0.41231	0.96008	0.49478	0.20215	0.63078	0.93618	0.64663	0.56746	2.10E-08	2.10E-08	blue
HTRA1	-0.03912	-0.08262	-0.04245	0.05048	-0.14897	0.00672	0.01319	0.24531	-0.24531	0.61144	0.28269	0.58146	0.51204	0.05182	0.93046	0.86409	0.00122	0.00122	black
RGL3	0.00576	-0.14041	-0.19176	-0.00446	0.17346	-0.12144	-0.03838	-0.16852	0.16852	0.94044	0.06699	0.01198	0.9538	0.02328	0.11358	0.61818	0.02758	0.02758	grey
LMLN	-0.03236	-0.15692	-0.1201	-0.04886	-0.05305	0.0102	0.06233	0.12242	-0.12242	0.67439	0.04039	0.11766	0.5257	0.49076	0.89463	0.41803	0.11068	0.11068	turquoise
MRPS15	-0.00011	-0.02849	-0.04687	-0.0615	0.03602	-0.08265	0.07556	-0.28313	0.28313	0.99889	0.7115	0.54265	0.42428	0.64002	0.28252	0.32596	0.00018	0.00018	grey
SPEF1	0.03122	-0.09883	-0.1857	-0.00834	0.16855	-0.24029	-0.13095	-0.0312	0.0312	0.68526	0.19843	0.01503	0.91381	0.02754	0.00155	0.08779	0.68543	0.68543	grey
HILPDA	-0.01494	-0.18093	0.0849	0.11834	0.00607	0.10534	0.08845	-0.1967	0.1967	0.84623	0.01788	0.26958	0.12317	0.93719	0.17029	0.24995	0.00992	0.00992	grey
TCTN2	0.0232	-0.21062	-0.21076	-0.16036	0.06703	-0.19698	-0.08378	0.0648	-0.0648	0.76326	0.00569	0.00566	0.03616	0.38373	0.00981	0.27594	0.39978	0.39978	turquoise
GNLY	-0.12795	0.18244	0.09307	0.06042	-0.08045	0.04995	0.12408	0.00513	-0.00513	0.09535	0.01693	0.226	0.43245	0.29557	0.51648	0.10589	0.94688	0.94688	grey
SPATA24	0.11052	-0.15885	-0.17056	-0.06237	0.16519	-0.28035	0.04068	-0.1098	0.1098	0.15015	0.03796	0.02572	0.41772	0.03084	0.0002	0.59728	0.15282	0.15282	grey
SLC26A6	0.04932	0.00794	0.15357	-0.02987	0.16277	0.17363	0.12017	-0.34369	0.34369	0.52174	0.91791	0.04493	0.69815	0.03341	0.02314	0.11744	4.18E-06	4.18E-06	grey
MSL2	-0.05097	-0.14782	-0.10917	-0.01513	-0.09914	0.07172	-0.0453	0.22633	-0.22633	0.50792	0.05368	0.15519	0.84426	0.19703	0.35122	0.5563	0.00291	0.00291	turquoise
TGM2	-0.03073	-0.05369	0.02426	0.05313	-0.24294	0.15748	0.27944	-0.12707	0.12707	0.68993	0.48553	0.75284	0.4901	0.00137	0.03968	0.00021	0.0977	0.0977	turquoise
PROX1	0.03249	-0.03395	-0.22543	-0.03284	0.10071	-0.171	-0.17252	0.28426	-0.28426	0.6731	0.6593	0.00303	0.66985	0.18999	0.02534	0.02405	0.00016	0.00016	grey
YPEL4	-0.00884	-0.08613	0.03422	0.12627	-0.07352	0.0781	0.05405	0.22562	-0.22562	0.90862	0.26266	0.65676	0.09982	0.33925	0.30995	0.48259	0.00301	0.00301	blue
PRDX5	0.03427	-0.00112	-0.06971	-0.03701	0.05485	-0.07272	0.12827	-0.36699	0.36699	0.65631	0.98837	0.36492	0.63084	0.47613	0.34454	0.09454	7.93E-07	7.93E-07	grey
TAF1D	0.07346	0.03281	0.07237	0.00338	0.00112	0.10299	0.20223	-0.36349	0.36349	0.33967	0.67007	0.3469	0.96497	0.98841	0.1801	0.00799	1.03E-06	1.03E-06	grey
PER1	-0.10002	-0.1762	-0.07631	0.04879	-0.09625	-0.03677	-0.12466	0.26752	-0.26752	0.19304	0.02115	0.32122	0.52625	0.21045	0.633	0.10427	0.0004	0.0004	grey
ZBED2	0.02649	-0.04757	0.00722	-0.00183	-0.13306	-0.00773	0.21384	-0.16019	0.16019	0.73091	0.53666	0.92534	0.981	0.08274	0.92006	0.00498	0.03636	0.03636	grey
MAN2A2	-0.05712	-0.17425	-0.09063	0.00641	0.09452	-0.11156	-0.17266	0.35366	-0.35366	0.45806	0.02265	0.23842	0.93366	0.21881	0.14631	0.02393	2.09E-06	2.09E-06	turquoise
MT-CO1	-0.08492	-0.12222	-0.11773	-0.01273	0.27253	-0.1211	-0.16006	-0.13944	0.13944	0.26945	0.11128	0.12513	0.86872	0.00031	0.11461	0.03651	0.06893	0.06893	grey
DECR2	0.04776	0.16694	0.05243	0.02624	0.1744	0.04817	0.12507	-0.40374	0.40374	0.53502	0.02909	0.49583	0.73336	0.02252	0.53156	0.10312	4.36E-08	4.36E-08	green
CRNKL1	0.09145	-0.05476	-0.08096	-0.03197	0.01964	-0.08814	0.06368	0.06284	-0.06284	0.23423	0.47683	0.29249	0.67808	0.79878	0.25164	0.40795	0.41421	0.41421	turquoise
WDR53	-0.04405	-0.07383	-0.07983	-0.05736	-0.14477	0.09114	0.1964	-0.08133	0.08133	0.56727	0.33721	0.29931	0.45619	0.05887	0.23583	0.01004	0.29026	0.29026	turquoise
MIB1	-0.05543	-0.18448	0.00325	0.03271	-0.13693	0.07932	0.01077	0.21004	-0.21004	0.47148	0.01572	0.96636	0.67101	0.07412	0.30241	0.88882	0.00583	0.00583	turquoise
LAMB3	0.06707	0.06344	0.15378	0.05694	-0.11286	0.29728	0.31081	-0.43931	0.43931	0.38341	0.40978	0.04462	0.45949	0.14162	7.86E-05	3.51E-05	1.84E-09	1.84E-09	yellow
SNX13	-0.06853	-0.17632	-0.04467	0.0496	0.01732	-0.02932	-0.0312	0.25872	-0.25872	0.37312	0.02106	0.56183	0.51945	0.8221	0.70344	0.68539	0.00063	0.00063	turquoise
GSTA4	-0.02286	-0.05454	-0.17812	-0.07613	0.07208	-0.25333	-0.28211	0.59221	-0.59221	0.76664	0.47862	0.01977	0.32233	0.3488	0.00083	0.00019	1.45E-17	1.45E-17	brown
FAM160B1	-0.05194	-0.18811	-0.01137	0.05185	-0.14491	-0.04854	-0.02267	0.38135	-0.38135	0.49984	0.01375	0.88262	0.50059	0.05862	0.52841	0.76856	2.66E-07	2.66E-07	turquoise
HHIPL1	-0.04735	-0.02328	0.01428	0.08276	-0.14491	0.07179	0.03734	0.31176	-0.31176	0.53854	0.7625	0.85296	0.28187	0.05862	0.35079	0.62774	3.31E-05	3.31E-05	black
HLA-G	-0.02985	0.10705	-0.03019	0.03616	-0.03471	0.07634	0.14262	-0.14283	0.14283	0.69836	0.16342	0.69505	0.63865	0.65225	0.32097	0.06276	0.06237	0.06237	grey

DDX42	-0.0498	-0.20026	-0.00033	-0.04223	-0.0764	0.00125	0.02186	0.04941	-0.04941	0.51774	0.00864	0.99659	0.58336	0.32065	0.98701	0.77657	0.52103	0.52103	turquoise
B4GAT1	-0.03535	-0.10399	-0.24297	-0.0819	0.12573	-0.31489	-0.14892	0.39015	-0.39015	0.64624	0.17588	0.00136	0.28694	0.10131	2.73E-05	0.0519	1.33E-07	1.33E-07	brown
KLHDC7B	-0.02682	-0.04133	0.15462	0.0935	-0.14249	0.15455	0.17973	-0.21639	0.21639	0.72767	0.59144	0.04347	0.22384	0.06301	0.04355	0.01866	0.00447	0.00447	grey
EXOSC5	0.09413	0.14008	-0.02241	-0.01801	-0.04983	0.06829	0.2436	-0.45365	0.45365	0.22073	0.06765	0.77105	0.81517	0.5175	0.3748	0.00132	4.63E-10	4.63E-10	purple
IL18	-0.04792	-0.01812	0.0698	0.03032	-0.10537	0.25856	0.26319	-0.27224	0.27224	0.53369	0.81408	0.36432	0.69381	0.17017	0.00064	0.00051	0.00032	0.00032	yellow
ZYG11B	-0.08953	-0.14609	-0.05313	0.03193	-0.15196	-0.00255	-0.02514	0.41173	-0.41173	0.24421	0.05658	0.4901	0.67848	0.04724	0.97361	0.74409	2.21E-08	2.21E-08	turquoise
POFUT2	-0.05512	-0.2071	-0.08844	-0.01804	0.18873	-0.09374	-0.03016	0.0267	-0.0267	0.47399	0.00657	0.25001	0.8148	0.01343	0.22266	0.69536	0.72887	0.72887	red
AC011448	0.09563	-0.0687	-0.18283	-0.07529	0.12968	-0.16728	-0.00795	-0.21347	0.21347	0.21341	0.37192	0.01669	0.32775	0.09093	0.02875	0.91784	0.00506	0.00506	red
TAB2	-0.04214	-0.12632	0.05968	0.06461	-0.21532	0.12246	0.04579	0.24485	-0.24485	0.58419	0.09969	0.43809	0.40117	0.00468	0.11058	0.55202	0.00125	0.00125	turquoise
YBX3	0.00412	-0.01147	0.10701	0.15006	-0.21422	0.27036	0.22827	-0.19194	0.19194	0.95737	0.88169	0.1636	0.05012	0.0049	0.00035	0.00267	0.0119	0.0119	black
IL7	-0.03056	-0.01414	0.02462	-0.00511	-0.08895	0.17664	0.0607	-0.1033	0.1033	0.69154	0.85438	0.74925	0.94708	0.24731	0.02082	0.43032	0.17879	0.17879	turquoise
H2AFJ	-0.03499	-0.07016	-0.01542	0.05834	0.07798	-0.09633	0.13335	-0.29445	0.29445	0.64962	0.36185	0.84138	0.44847	0.31069	0.21007	0.08208	9.26E-05	9.26E-05	grey
ARFGAP3	0.01685	-0.10205	-0.00455	0.04831	0.00165	0.00824	-0.14874	0.03837	-0.03837	0.82681	0.18411	0.95289	0.53038	0.98293	0.91479	0.05218	0.61833	0.61833	turquoise
AC012651	-0.04973	-0.02876	-0.02883	0.03858	0.07508	0.04721	-0.0063	0.09558	-0.09558	0.51834	0.70886	0.70814	0.61636	0.32907	0.53974	0.93485	0.21364	0.21364	red
COPS7B	0.04281	-0.10708	-0.05042	-0.00101	0.02611	0.00743	0.09867	-0.31228	0.31228	0.57822	0.16332	0.51252	0.98955	0.73466	0.92314	0.19916	3.21E-05	3.21E-05	grey
PCBP4	-0.01867	0.03631	-0.16926	-0.12154	0.03916	-0.09571	-0.00481	-0.19615	0.19615	0.80852	0.63732	0.02689	0.11329	0.61108	0.21304	0.95019	0.01013	0.01013	grey
SPOCD1	-0.06049	-0.00995	0.07266	0.12286	-0.06289	0.02611	0.1781	-0.18056	0.18056	0.43192	0.89724	0.34493	0.10939	0.41386	0.73461	0.01978	0.01811	0.01811	black
DIRAS3	0.01964	-0.08784	-0.17914	-0.0267	0.0263	-0.1605	-0.15866	0.44288	-0.44288	0.79879	0.25329	0.01906	0.72887	0.73279	0.036	0.0382	1.31E-09	1.31E-09	brown
SOWAHC	0.09247	-0.02229	0.06589	0.06725	-0.10864	0.13885	0.1107	-0.13076	0.13076	0.22899	0.77233	0.39184	0.38211	0.15723	0.07012	0.14945	0.08825	0.08825	turquoise
SCAPER	-0.06224	-0.1931	-0.09913	-0.04044	-0.07522	-0.07433	-0.10811	0.40521	-0.40521	0.4187	0.01139	0.19706	0.59948	0.32815	0.33394	0.15927	3.85E-08	3.85E-08	turquoise
NFKBIB	0.13084	-0.15753	0.01063	0.06436	-0.0537	0.10205	0.1729	-0.33387	0.33387	0.08806	0.03962	0.8902	0.40295	0.48548	0.18414	0.02373	8.10E-06	8.10E-06	grey
MID2	-0.0149	-0.24157	-0.05695	0.01633	-0.14893	0.00534	-0.03537	0.4187	-0.4187	0.84658	0.00146	0.45937	0.83216	0.05189	0.94469	0.646	1.20E-08	1.20E-08	turquoise
RIDA	-0.05082	-0.01578	-0.09273	0.08518	0.09131	-0.11239	0.04628	-0.10526	0.10526	0.50921	0.83769	0.22771	0.26797	0.23492	0.14333	0.54778	0.17064	0.17064	grey
NOXA1	0.05881	0.01957	-0.08242	-0.04075	0.26878	0.03275	0.04793	-0.41657	0.41657	0.44483	0.79947	0.28383	0.59671	0.00038	0.67062	0.53359	1.45E-08	1.45E-08	green
DUS2	-0.05237	0.06593	0.0039	-0.00925	-0.03193	0.03541	0.16789	-0.17124	0.17124	0.49633	0.39161	0.95966	0.90438	0.67845	0.64562	0.02816	0.02513	0.02513	turquoise
ADAMTSL	-0.04434	-0.09239	-0.04105	0.03496	-0.1225	0.0311	-0.15002	0.61187	-0.61187	0.56476	0.22941	0.59396	0.64985	0.11046	0.68636	0.05018	6.09E-19	6.09E-19	turquoise
OAZ1	0.05703	0.04057	-0.13513	-0.10239	0.16023	-0.22207	-0.04702	-0.10107	0.10107	0.45877	0.59834	0.07804	0.18266	0.03631	0.00336	0.54144	0.18841	0.18841	green
HIST1H2B(	0.0053	-0.14228	0.10053	0.02472	-0.06871	0.12254	0.08675	-0.02175	0.02175	0.9452	0.06341	0.19078	0.74826	0.37185	0.11032	0.25922	0.77763	0.77763	grey
AIFM2	-0.0583	-0.06233	-0.05759	-0.06613	-0.0962	0.03615	0.15287	-0.25892	0.25892	0.4488	0.41802	0.45435	0.39012	0.2107	0.63877	0.04593	0.00063	0.00063	turquoise
NBPF26	0.04454	-0.12288	0.05821	0.15236	-0.07946	0.14355	0.05348	0.20095	-0.20095	0.56295	0.10934	0.44947	0.04665	0.30154	0.06105	0.4872	0.0084	0.0084	turquoise
BRICD5	0.07267	-0.14787	-0.13147	-0.07995	0.25434	-0.19914	-0.13969	-0.10858	0.10858	0.34488	0.0536	0.08653	0.29859	0.00079	0.00902	0.06843	0.15746	0.15746	red
WSB2	-0.01042	-0.19197	-0.04476	-0.05827	0.03774	-0.09367	-0.10229	0.09475	-0.09475	0.89236	0.01189	0.56107	0.44905	0.6241	0.223	0.1831	0.21767	0.21767	turquoise
NMT2	-0.06694	-0.11282	-0.01181	0.11666	-0.14945	0.1427	0.05431	0.09673	-0.09673	0.38434	0.1418	0.87818	0.12863	0.05107	0.06262	0.4805	0.20818	0.20818	turquoise
FAM234B	0.01767	-0.21323	-0.09513	0.04642	-0.0736	-0.20864	-0.17696	0.35409	-0.35409	0.81855	0.00511	0.21586	0.54657	0.33872	0.00617	0.02059	2.02E-06	2.02E-06	brown
SYCP2	-0.08219	-0.10821	-0.00268	0.14746	-0.0083	0.03836	0.08368	0.04777	-0.04777	0.2852	0.15888	0.97226	0.05427	0.91417	0.61843	0.27654	0.53498	0.53498	grey
FTCDNL1	-0.00872	0.12716	-0.0249	0.05376	-0.11113	0.12133	0.01528	0.1068	-0.1068	0.90988	0.09745	0.74653	0.48496	0.14787	0.11391	0.84276	0.16443	0.16443	grey
ZNHIT1	0.0905	-0.01701	-0.0654	-0.03226	0.23553	-0.10301	0.09039	-0.44491	0.44491	0.23911	0.8252	0.3954	0.67535	0.00193	0.18	0.2397	1.08E-09	1.08E-09	green
SHF	0.01928	0.00138	0.01085	-0.06264	0.04507	0.02287	0.07584	-0.13265	0.13265	0.80235	0.98571	0.88796	0.41572	0.55834	0.76656	0.32418	0.08371	0.08371	grey
PTPRJ	0.04366	-0.18562	-0.12566	-0.11936	0.05461	-0.05962	-0.08264	0.08549	-0.08549	0.57073	0.01507	0.1015	0.11997	0.47811	0.4386	0.28256	0.26625	0.26625	turquoise
BLOC1S2	-0.02592	0.01783	0.01706	0.03282	-0.07102	-0.0485	0.01013	0.0394	-0.0394	0.73651	0.81696	0.82475	0.67001	0.35599	0.52872	0.89542	0.60889	0.60889	turquoise
SIGLEC12	-0.06359	0.00056	0.0765	0.01316	-0.12939	-0.01855	0.11966	0.05817	-0.05817	0.40864	0.99415	0.31999	0.8644	0.09167	0.80967	0.11904	0.44978	0.44978	grey
TLN1	-0.02434	-0.0833	0.0347	0.0306	-0.13744	0.10237	0.02644	0.28436	-0.28436	0.75196	0.27872	0.65229	0.69119	0.07304	0.18276	0.73138	0.00016	0.00016	turquoise
TTC21B	-0.03222	-0.16211	-0.06695	0.04437	-0.09167	-0.06159	-0.01986	0.34467	-0.34467	0.6757	0.03415	0.38428	0.56443	0.23309	0.4236	0.79655	3.91E-06	3.91E-06	turquoise
CPXM1	-0.04259	-0.02709	0.0169	0.09763	-0.11939	0.0515	0.07928	0.32994	-0.32994	0.58019	0.72508	0.82631	0.20395	0.11987	0.50352	0.30267	1.05E-05	1.05E-05	blue
ARL13B	-0.08683	-0.15789	-0.09126	-0.01215	-0.14587	0.06722	-0.06251	0.34235	-0.34235	0.25877	0.03917	0.23519	0.87468	0.05695	0.38239	0.41663	4.58E-06	4.58E-06	turquoise
PDPK1	-0.02106	-0.08468	-0.04128	-0.01222	-0.04168	-0.00785	-0.07349	0.20023	-0.20023	0.7845	0.27082	0.5919	0.87397	0.58836	0.91886	0.33946	0.00864	0.00864	turquoise
NPRL2	0.05235	0.04505	-0.20606	-0.22945	0.13207	-0.03031	0.01412	-0.19375	0.19375	0.49653	0.55847	0.00685	0.00254	0.08508	0.69389	0.85453	0.01111	0.01111	grey
TLR7	-0.07869	-0.00036	0.04823	0.02161	-0.26073	0.16141	0.04963	0.47831	-0.47831	0.30628	0.99627	0.53107	0.77908	0.00057	0.03494	0.51912	3.68E-11	3.68E-11	blue
ANGPTL7	-0.07809	-0.05944	-0.08074	0.08895	-0.07142	-0.07891	-0.00615	0.41852	-0.41852	0.30998	0.43993	0.29384	0.24729	0.35327	0.30495	0.93641	1.22E-08	1.22E-08	blue
P2RX1	-0.12562	0.10491	0.09459	0.03872	-0.10147	0.13774	-0.12494	0.34825	-0.34825	0.1016	0.17206	0.21845	0.6151	0.18664	0.07241	0.10348	3.05E-06	3.05E-06	magenta
CAVIN2	-0.13583	-0.09975	-0.01872	0.07831	-0.05152	0.07472	-0.13101	0.54067	-0.54067	0.07649	0.19426	0.80799	0.30863	0.50332	0.33142	0.08763	2.29E-14	2.29E-14	blue
OTUD7B	0.07055	-0.15661	-0.00174	0.01693	-0.08192	0.16008	0.12872	-0.0765	0.0765	0.35915	0.04081	0.98201	0.82604	0.28682	0.03649	0.09338	0.31996	0.31996	turquoise
HBS1L	-0.11864	-0.07589	0.01951	0.0159	-0.12792	0.00309	0.02186	0.25626	-0.25626	0.12222	0.32388	0.8001	0.83647	0.09545	0.96799	0.77659	0.00072	0.00072	turquoise
ZNF302	-0.10835	-0.16374	-0.11678	-0.02517	0.03452	-0.09193	-0.10175	0.24409	-0.24409	0.15836	0.03236	0.12823	0.74379	0.65397	0.23176	0.18543	0.00129	0.00129	turquoise
TMX2	0.0133	-0.07621	-0.04328	-0.0644	-0.10115	-0.0234	0.13351	-0.16918	0.16918	0.86288	0.32184	0.57406	0.40266	0.18803	0.76132	0.08171	0.02697	0.02697	turquoise
WARS2	-0.0439	0.02425	0.09076	0.07253	0.07147	0.18024	0.02781	-0.03538	0.03538	0.5686	0.75292	0.2378	0.34581	0.35295	0.01832	0.71809	0.64594	0.64594	turquoise
PTAR1	-0.03023	-0.15943	-0.06483	0.03151	-0.09631	0.0681	-0.0074	0.21201	-0.21201	0.69469	0.03726	0.39958	0.6824	0.21018	0.37612	0.9235	0.00537	0.00537	turquoise
STXBP4	0.02587	-0.22244	-0.15273	0.04805	-0.01772	-0.14151	-0.14982	0.43676	-0.43676	0.73696	0.00345	0.04612	0.53259	0.81803	0.06486	0.05048	2.34E-09	2.34E-09	turquoise
C5orf30	0.00278	-0.00786	-0.02879	-0.0342	0.01776	0.08018	0.04653	-0.1489	0.1489	0.97116	0.91878	0.70861	0.65697	0.81771	0.29719	0.54563	0.05193	0.05193	turquoise
UBAP1L	-0.03328	-0.14217	-0.14096	-0.06164	0.09439	-0.12889	-0.17208	0.2062	-0.2062	0.66569	0.06361	0.06593	0.42321	0.21943	0.09294	0.02442	0.00681	0.00681	red
MRRF	0.0059	0.00108	-0.0792	-0.09901	-0.03977	0.05826	0.13713	-0.2944	0.2944	0.93896	0.98878	0.30318	0.19761	0.60556	0.44914	0.07368	9.29E-05	9.29E-05	turquoise
CYYR1	-0.15216	-0.09739	-0.11068	0.057	-0.10621	-0.00951	-0.12625	0.59242	-0.59242	0.04695	0.20507	0.14954	0.45897	0.16677	0.90174	0.09987	1.40E-17	1.40E-17	blue
DCBLD2	-0.0619	-0.1273	-0.01171	0.03346	-0.2023	0.07207	0.1241	-0.04204	0.04204	0.42127	0.09707	0.87923	0.66392	0.00797	0.34891	0.10584	0.58508	0.58508	black
BCL2L15	0.03058	0.05712	0.14199	0.0942	0.02195	0.28781	0.13604	-0.33692	0.33692	0.69137	0.45801	0.06396	0.22036	0.77564	0.00014	0.07603	6.61E-06	6.61E-06	yellow
ZNF428	0.04976	-0.09647	-0.19097	-0.13903	0.15073	-0.26852	-0.08135	0.10905	-0.10905	0.51808	0.20942	0.01235	0.06975	0.04908	0.00038	0.29017	0.15566	0.15566	grey
CNTD2	0.13377	0.00479	-0.01778	0.08367	0.07466	-0.04301	-0.06073	-0.20139	0.20139	0.08111	0.95037	0.81748	0.27658	0.33183	0.57646	0.43006	0.00826	0.00826	grey
CEP72	0.09056	0.11421	0.04653	-0.03302	0.03929	0.00595	0.19312	-0.38861	0.38861	0.23879	0.13691	0.54566	0.66809	0.60986	0.93845	0.01138	1.50E-07	1.50E-07	grey
FBXW8	0.03104	-0.11575	-0.06221	-0.01558	0.01795	-0.09588	-0.12892	0.22661	-0.22661	0.68697	0.13164	0.41888	0.8397	0.81578	0.21222	0.09286	0.00288	0.00288	turquoise
ARHGEF1:	-0.02925	-0.12451	-0.05288	0.02581	-0.08994	0.08534	0.01614	0.11527	-0.11527	0.70412	0.10468	0.49217	0.73757	0.24205	0.26709	0.83405	0.13327	0.13327	turquoise
GNB1L	-0.03558	-0.05506	0.01894	0.10627	-0.02534	-0.01371	0.18687	-0.21345	0.21345	0.64408	0.47446	0.80576	0.16655	0.74218	0.8587	0.01439	0.00506	0.00506	green
TMIE	-0.01514	-0.1627	-0.1763	-0.09846	-0.01769	-0.2351	-0.08654	0.00928	-0.00928	0.84422	0.03349	0.02107	0.20014	0.81833	0.00197	0.26037	0.90412	0.90412	grey
CASP8AP2	-0.10697	-0.07347	0.00553	0.03481	-0.14362	0.08466	0.04256	0.31803	-0.31803	0.16377	0.33959	0.94277	0.65129	0.06092	0.27094	0.58044	2.25E-05	2.25E-05	turquoise
PPIB	-0.07446	-0.03605	-0.09068	-0.0161	0.02721	-0.13527	-0.01658	-0.07213	0.07213	0.33308	0.63971	0.2382	0.83449	0.72385	0.07772	0.82959	0.34848	0.34848	grey
DHRS11	0.01399	-0.06697	-0.04823	-0.126	0.28562	-0.07792	-0.00023	-0.27416	0.27416	0.85586	0.38416	0.53104	0.10055	0.00015	0.31104	0.99758	0.00028	0.00028	grey
B3GNT9	-0.10315	-0.144	-0.09525	-0.01506	-0.02081	-0.03461	0.00595	0.29678	-0.29678	0.17942	0.06025	0.21526	0.84502	0.78703	0.65317	0.93846	8.09E-05	8.09E-05	blue
SLC9A4	-0.03424	0.04146	0.10274	0.0322	-0.03177	0.1714	0.05246	-0.07275	0.07275	0.65659	0.59032	0.18117	0.67591	0.67993	0.02499	0.49558	0.34436	0.34436	grey
LSM11	0.02101	-0.16802	0.00108	0.05554	-0.14056	0.04354	0.02119	0.23589	-0.23589	0.785	0.02804	0.98877	0.4706	0.06669	0.57179	0.78326	0.0019	0.0019	turquoise
TRMT12	-0.03535	-0.13404	-0.07662	-0.04897	-0.01712	-0.03988	0.12768	-0.05321	0.05321	0.64625	0.08048	0.31924	0.52472	0.82413	0.60457	0.09608	0.48945	0.48945	turquoise
NOL7	0.02649	0.01871	-0.05352	-0.11704	0.08113	-0.06673	0.03863	-0.07435	0.07435	0.73087	0.80809	0.48692	0.12738	0.29149	0.38585	0.61589	0.3338	0.3338	grey
SWAP70	-0.07102	-0.14354	0.0875	0.07896	-0.21224	0.23645	0.04788	0.27338	-0.27338	0.35601	0.06107	0.25513	0.30461	0.00532	0.00185	0.53404	0.0003	0.0003	turquoise
PPM1D	-0.10839	-0.21878	-0.17478	0.00591	0.06291	-0.09304	-0.15426	0.40589	-0.40589	0.15821	0.00404	0.02223	0.93882	0.41367	0.22615	0.04396	3.64E-08	3.64E-08	turquoise
TSPAN11	-0.08562	-0.07731	-0.11744	-0.00574	-0.04805	-0.09225	-0.05597	0.53537	-0.53537	0.26548	0.31488	0.12607	0.9406	0.53258	0.23014	0.46714	4.55E-14	4.55E-14	blue
STK32C	0.04458	-0.02567	-0.11185	-0.06879	0.07198	-0.25009	0.08223	0.00724	-0.00724	0.5626	0.73896	0.14528	0.37135	0.34949	0.00097	0.28494	0.92515	0.92515	grey
MRPL28	0.03318	0.03329	-0.07503	-0.05354	0.09504	-0.10878	0.14389	-0.36904	0.36904	0.66657	0.66558	0.32939	0.48675	0.21628	0.15669	0.06044	6.80E-07	6.80E-07	green
NINJ2	0.00122	0.02964	0.07707	0.07896	-0.21411	0.21482	0.07212	0.12903	-0.12903	0.98732	0.70034	0.31635	0.30462	0.00492	0.00478	0.34855	0.09259	0.09259	black
TMEM54	0.10699	0.0097	0.05083	-0.00581	0.13624	0.08302	0.15298	-0.46931	0.46931	0.16368	0.89981	0.50908	0.93988	0.0756	0.28036	0.04577	9.50E-11	9.50E-11	yellow
LAD1	0.08382	0.10689	0.0596	-0.01607	-0.03377	0.26812	0.23279	-0.49983	0.49983	0.27574	0.16409	0.43874	0.8348	0.66106	0.00039	0.00218	3.40E-12	3.40E-12	yellow
NRG4	0.02512	-0.02631	0.09072	0.08989	-0.08553	0.15631	0.00741	-0.04084	0.04084	0.74436	0.73271	0.238	0.24232	0.26599	0.0412	0.92334	0.5959	0.5959	grey
LRRC59	-0.04776	-0.09969	0.05762	0.05307	-0.08398	0.17161	0.09825	-0.16361	0.16361	0.53509	0.19453	0.45413	0.49061	0.27479	0.02481	0.20109	0.0325	0.0325	pink
NUP205	-0.03669	-0.07023	0.01957	-0.00724	-0.10941	-0.04969	0.05903	0.08769	-0.08769	0.63382	0.36136	0.79942	0.92509	0.15429	0.5187	0.44314	0.25408	0.25408	turquoise
SLC39A7	0.04042	-0.05754	-0.07173	0.00036	0.10486	-0.06314	0.10849	-0.02807	0.02807	0.59961	0.45472	0.35115	0.99627	0.17227	0.41196	0.15783	0.71557	0.71557	grey
YLPM1	0.01703	-0.14114	-0.08362	-0.01844	-0.09265	-0.0143	-0.01709	0.26779	-0.26779	0.82503	0.06558	0.27686	0.81079	0.22808	0.85276	0.82442	0.0004	0.0004	turquoise
TRHDE	-0.05541	-0.00156	0.0911	0.03147	-0.07627	0.06355	-0.00044	0.07471	-0.07471	0.47162	0.98388	0.23601	0.68282	0.32145	0.40894	0.9954	0.33146	0.33146	grey
NRAS	-0.11069	-0.07068	0.08563	0.10773	-0.17076	0.2185	0.14392	0.00313	-0.00313	0.14949	0.35826	0.26546	0.16077	0.02555	0.00409	0.06038	0.96762	0.96762	turquoise
RFC4	-0.00973	0.05254	-0.01809	-0.02613	-0.0576	0.01567	0.27778	-0.34339	0.34339	0.89952	0.49491	0.81438	0.73441	0.45431	0.83882	0.00023	4.26E-06	4.26E-06	pink
CA9	-0.08163	0.04161	-0.00959	0.04197	0.1013	0.06157	0.03154	-0.47951	0.47951	0.28851	0.58898	0.90094	0.58575	0.18737	0.42375	0.68218	3.24E-11	3.24E-11	grey
FMNL3	-0.08207	-0.10558	-0.00669	0.04364	-0.21345	0.07822	0.0042	0.44703	-0.44703	0.28589	0.17018	0.93082	0.57084	0.00506	0.30922	0.95653	8.84E-10	8.84E-10	blue
ADGRG2	-0.06365	-0.07041	-0.1421	-0.0748	0.17803	-0.26837	-0.20019	0.32496	-0.32496	0.40822	0.36015	0.06374	0.33091	0.01982	0.00039	0.00866	1.45E-05	1.45E-05	brown
MRPL15	#######	0.13139	-0.02937	-0.01313	-0.15679	-0.01624	0.19041	-0.28277	0.28277	0.99962	0.08671	0.70294	0.86464	0.04057	0.83301	0.01261	0.00018	0.00018	turquoise
OGFOD3	-0.0478	-0.14331	-0.14178	-0.08616	0.12909	-0.3056	-0.07065	0.15224	-0.15224	0.53472	0.06149	0.06434	0.2625	0.09242	4.81E-05	0.35849	0.04683	0.04683	brown
ZSWIM9	0.06551	-0.06379	-0.17396	-0.01375	0.07786	-0.13986	0.02325	-0.01816	0.01816	0.3946	0.40717	0.02288	0.8583	0.31141	0.06808	0.76274	0.81366	0.81366	grey
IL1B	-0.17375	-0.01798	-0.02107	-0.01079	-0.06261	0.11845	0.07214	0.20032	-0.20032	0.02304	0.8154	0.78448	0.88864	0.41588	0.12281	0.34843	0.00861	0.00861	grey
SLCO3A1	-0.09504	-0.06896	-0.02092	0.11587	-0.2337	0.07622	0.05161	0.04332	-0.04332	0.21629	0.37015	0.78598	0.13126	0.0021	0.32176	0.50259	0.57374	0.57374	turquoise
PRRC2C	-0.02173	-0.09996	0.04458	0.00137	-0.12697	0.21448	0.10299	0.01924	-0.01924	0.7779	0.19333	0.56262	0.98586	0.09795	0.00485	0.1801	0.80278	0.80278	turquoise
ADH5	-0.05888	-0.07485	0.02279	0.04669	-0.08951	0.11492	0.08603	0.10847	-0.10847	0.44428	0.33056	0.76732	0.54422	0.24435	0.13445	0.26324	0.15788	0.15788	turquoise
TNFAIP6	-0.12855	-0.0161	0.07507	0.0953	-0.25422	0.08363	0.06634	0.23742	-0.23742	0.09381	0.83446	0.32916	0.215	0.00079	0.27681	0.38867	0.00177	0.00177	black
ACSL1	-0.06962	-0.11917	-0.04731	-0.0082	-0.08728	0.02351	-0.08118	0.29558	-0.29558	0.36553	0.12056	0.53894	0.91528	0.25631	0.7602	0.29116	8.68E-05	8.68E-05	turquoise
ZNF747	-0.08763	-0.10308	-0.16356	-0.08288	0.00923	-0.10193	-0.09346	0.13018	-0.13018	0.25441	0.17973	0.03255	0.28118	0.90459	0.18464	0.22405	0.08968	0.08968	grey
KLHL36	-0.06751	-0.1462	-0.13203	-0.0535	-0.04315	0.00049	-0.06537	0.15232	-0.15232	0.3803	0.05638	0.08517	0.48706	0.57518	0.99488	0.39561	0.04672	0.04672	turquoise
COL4A5	0.00768	-0.06238	-0.11632	0.00214	-0.06647	0.05956	0.06455	0.18908	-0.18908	0.92063	0.41765	0.12974	0.97788	0.38773	0.43902	0.40156	0.01325	0.01325	blue
MOCS1	-0.09946	-0.11706	-0.11486	0.06924	-0.08044	-0.06008	-0.17901	0.54039	-0.54039	0.19557	0.12733	0.13468	0.36821	0.29563	0.43505	0.01915	2.37E-14	2.37E-14	blue
FOXA1	0.11091	-0.11043	0.01364	0.0276	0.11102	0.07547	0.04427	-0.30179	0.30179	0.1487	0.15047	0.85951	0.72008	0.1483	0.32659	0.56534	6.03E-05	6.03E-05	grey
PLEKHF1	-0.03395	-0.07941	0.03097	0.15323	-0.18208	0.13694	0.20201	-0.01311	0.01311	0.6593	0.30186	0.68758	0.0454	0.01715	0.0741	0.00806	0.86484	0.86484	black
PSMF1	0.05668	0.01076	-0.05955	-0.02061	-0.00467	-0.10858	0.03645	-0.0499	0.0499	0.4615	0.88893	0.4391	0.789	0.95164	0.15745	0.63595	0.5169	0.5169	grey
B3GALT4	-0.06391	0.03629	-0.10262	-0.04796	0.08165	-0.03677	0.02959	-0.25469	0.25469	0.40632	0.63748	0.18168	0.53337	0.28841	0.63302	0.70082	0.00077	0.00077	grey
SELENOI	0.00087	-0.0862	-0.07728	-0.05069	0.04416	-0.07782	0.03873	-0.0353	0.0353	0.99094	0.26227	0.31505	0.51024	0.56629	0.31167	0.61499	0.64672	0.64672	turquoise
MTCH1	0.03865	-0.09384	-0.01788	0.00293	0.04824	-0.07375	-0.04271	0.08039	-0.08039	0.61573	0.22218	0.81648	0.96966	0.53097	0.33777	0.57917	0.29592	0.29592	turquoise
DPYD	-0.11469	-0.06502	0.05669	0.12895	-0.23884	0.18593	0.14482	0.15278	-0.15278	0.13524	0.39817	0.46144	0.09279	0.00165	0.0149	0.05878	0.04604	0.04604	turquoise
ELOF1	0.03579	-0.05098	-0.21736	-0.07903	0.08443	-0.21385	-0.07866	-0.07824	0.07824	0.64215	0.50785	0.00429	0.30421	0.27226	0.00498	0.30648	0.30908	0.30908	grey
CXCR3	-0.07812	0.03429	0.08351	0.10749	0.00483	0.12135	0.0259	0.0903	-0.0903	0.30984	0.65611	0.27752	0.16172	0.95003	0.11386	0.73668	0.24019	0.24019	blue
ZNF112	0.00892	-0.14849	-0.12997	-0.00485	0.04097	-0.07776	-0.00728	0.16028	-0.16028	0.90782	0.05258	0.09021	0.94982	0.5947	0.31207	0.92466	0.03624	0.03624	turquoise
PITHD1	0.04515	-0.12504	-0.01128	-0.03113	-0.02693	0.02268	-0.05355	0.07194	-0.07194	0.55759	0.10321	0.88358	0.68606	0.72664	0.76838	0.48666	0.34977	0.34977	turquoise
AGBL2	-0.0502	-0.08275	0.03393	0.04478	0.09455	-0.00922	0.03462	-0.26106	0.26106	0.51437	0.28193	0.65948	0.56087	0.21867	0.90473	0.65305	0.00056	0.00056	red
GPR137	0.00744	-0.17082	-0.13195	-0.04122	0.12034	-0.10759	-0.04912	-0.06594	0.06594	0.92305	0.02549	0.08537	0.59241	0.11692	0.1613	0.52343	0.39153	0.39153	grey
MTMR6	-0.07943	-0.1106	-0.06666	-0.04255	-0.10306	-0.07598	-0.11286	0.40843	-0.40843	0.30178	0.14984	0.38636	0.58059	0.1798	0.32327	0.14162	2.93E-08	2.93E-08	turquoise
TSPO2	0.07103	0.04931	0.03937	0.04237	0.203	-0.03089	-0.03345	-0.19852	0.19852	0.35591	0.52187	0.60915	0.58219	0.00775	0.68834	0.66408	0.00924	0.00924	grey
TNFRSF10	-0.02497	0.03235	0.17677	0.06188	-0.14581	0.33245	0.23252	-0.27623	0.27623	0.7458	0.67448	0.02073	0.42138	0.05705	8.89E-06	0.00221	0.00026	0.00026	yellow
PTDSS1	0.03486	0.00095	#######	0.03703	-0.19716	0.06147	0.13388	-0.00574	0.00574	0.65082	0.99018	0.99911	0.63065	0.00975	0.42447	0.08085	0.94064	0.94064	turquoise
AGR2	0.03585	0.08366	0.14346	0.02167	0.05062	0.28974	0.13633	-0.40203	0.40203	0.64157	0.27667	0.06121	0.77848	0.51088	0.00012	0.0754	5.02E-08	5.02E-08	yellow

ERLEC1	-0.05986	-0.14966	-0.11779	-0.05615	-0.01281	-0.12918	-0.04863	0.27458	-0.27458	0.43671	0.05073	0.12494	0.46575	0.86795	0.09219	0.52761	0.00028	0.00028	turquoise
AGRN	-0.00443	-0.02077	0.00295	0.10654	-0.04949	0.15642	0.21954	-0.34408	0.34408	0.95418	0.78743	0.96949	0.16546	0.52039	0.04105	0.00391	4.07E-06	4.07E-06	yellow
FXYD1	-0.05934	-0.04336	-0.07609	0.02316	-0.02771	-0.05668	-0.09146	0.45972	-0.45972	0.4407	0.5734	0.32257	0.76368	0.71907	0.46153	0.23414	2.53E-10	2.53E-10	grey
PPP2R5A	-0.00065	-0.03949	-0.02121	-0.02871	0.06675	0.16757	0.04296	-0.19777	0.19777	0.99328	0.60811	0.78302	0.70934	0.38568	0.02848	0.57687	0.00952	0.00952	turquoise
CDC14B	0.01309	-0.0896	-0.02371	0.03801	-0.06873	0.14171	0.0909	0.04686	-0.04686	0.8651	0.24385	0.75818	0.62161	0.37172	0.06448	0.23703	0.54282	0.54282	turquoise
GALT	-0.0111	-0.06536	-0.20258	-0.15935	0.13959	-0.14972	-0.0793	0.00592	-0.00592	0.88542	0.3957	0.00788	0.03736	0.06862	0.05064	0.30255	0.93878	0.93878	grey
ARMCX6	0.01482	-0.17518	-0.03353	0.01608	-0.04429	0.08561	0.0411	0.02167	-0.02167	0.84745	0.02192	0.66327	0.83462	0.56516	0.26556	0.59348	0.77846	0.77846	turquoise
PHACTR4	-0.04407	-0.13659	0.01113	0.06308	-0.10305	0.17656	-0.00477	0.12991	-0.12991	0.56711	0.07485	0.88517	0.41244	0.17986	0.02088	0.95064	0.09035	0.09035	turquoise
ANOS1	-0.10699	-0.06503	0.1154	0.11594	-0.30104	0.16757	0.10695	0.24386	-0.24386	0.16366	0.39809	0.13284	0.13101	6.31E-05	0.02847	0.16383	0.00131	0.00131	black
SCNN1B	0.09296	-0.12811	0.06824	0.11377	-0.03436	-0.14755	-0.11277	0.13201	-0.13201	0.22657	0.09494	0.37515	0.13844	0.65547	0.05412	0.14196	0.08523	0.08523	grey
CLN5	0.04189	-0.05702	-0.07624	-0.0691	-0.06583	-0.0618	-0.01814	0.19796	-0.19796	0.58644	0.4588	0.32165	0.36915	0.3923	0.42196	0.8138	0.00945	0.00945	turquoise
ELF1	-0.03692	-0.11803	0.04479	0.04355	-0.10149	0.20355	-0.0593	0.12558	-0.12558	0.63167	0.12419	0.56079	0.57167	0.18658	0.00758	0.44104	0.10171	0.10171	turquoise
TSFM	0.03451	-0.06639	-0.10955	-0.01103	0.0984	-0.2021	-0.00773	-0.04354	0.04354	0.65413	0.38827	0.15379	0.88611	0.20039	0.00803	0.92005	0.57179	0.57179	turquoise
KANSL2	0.04572	-0.20521	-0.08774	0.0286	0.07058	-0.0913	-0.14746	0.14723	-0.14723	0.55267	0.00709	0.2538	0.7104	0.35896	0.23499	0.05426	0.05465	0.05465	turquoise
TRABD	0.04348	0.17458	-0.0418	-0.02087	0.12741	-0.04138	0.1527	-0.36867	0.36867	0.5723	0.02238	0.58722	0.78642	0.09677	0.59098	0.04616	6.99E-07	6.99E-07	green
ZDHHC16	0.03006	-0.14585	-0.00428	-0.00612	-0.03758	-0.02881	0.19689	-0.34026	0.34026	0.69631	0.05699	0.95574	0.93668	0.62557	0.70839	0.00985	5.28E-06	5.28E-06	grey
ZFPM2	-0.08428	-0.08119	0.07858	0.09746	-0.29085	0.18977	0.07523	0.42826	-0.42826	0.27307	0.29111	0.30696	0.20477	0.00011	0.01292	0.3281	5.12E-09	5.12E-09	blue
RAB11B	0.03203	-0.0688	-0.17315	-0.1011	0.15481	-0.16699	-0.02273	-0.18538	0.18538	0.67747	0.37128	0.02353	0.18828	0.04321	0.02903	0.7679	0.01521	0.01521	green
USP38	-0.08323	-0.17522	-0.05281	0.00342	-0.04258	0.01563	-0.05804	0.20117	-0.20117	0.27912	0.02189	0.49275	0.96461	0.58027	0.83926	0.45085	0.00833	0.00833	turquoise
MBTD1	-0.12202	-0.17001	-0.09186	0.00265	0.02847	0.00781	-0.03166	0.08656	-0.08656	0.11187	0.02621	0.23209	0.97255	0.71162	0.9193	0.68103	0.26026	0.26026	turquoise
COPZ1	0.13946	-0.08303	-0.12917	-0.069	0.09409	-0.18384	-0.02226	-0.24566	0.24566	0.06889	0.28032	0.09221	0.36986	0.22091	0.01609	0.77261	0.0012	0.0012	turquoise
FAM214B	0.08529	-0.08622	0.05909	-0.00424	-0.05816	0.14709	0.09418	-0.1912	0.1912	0.26735	0.26219	0.44269	0.95611	0.44988	0.05489	0.2205	0.01224	0.01224	grey
FITM1	-0.04839	0.0238	-0.03591	0.13065	-0.02966	-0.02358	-0.1354	0.16539	-0.16539	0.52967	0.75731	0.64104	0.08851	0.70021	0.75947	0.07744	0.03063	0.03063	magenta
PRRG2	0.07122	-0.01219	-0.10229	-0.11867	0.13758	-0.09898	0.0194	-0.38865	0.38865	0.35462	0.87428	0.18309	0.12213	0.07274	0.19773	0.8012	1.50E-07	1.50E-07	grey
DPEP1	0.06518	0.03339	0.12508	0.04047	-0.1192	0.0847	-0.09333	0.29631	-0.29631	0.39701	0.66461	0.1031	0.59924	0.12045	0.27071	0.22468	8.32E-05	8.32E-05	magenta
HSD17B2	0.05737	-0.00625	0.06071	0.08916	0.00922	0.28287	0.1183	-0.26548	0.26548	0.45605	0.93539	0.43025	0.24616	0.90478	0.00018	0.12331	0.00045	0.00045	yellow
KCNK5	0.04713	0.00942	0.11003	0.19789	0.04857	0.28698	0.1491	-0.19044	0.19044	0.54048	0.90264	0.15195	0.00947	0.52811	0.00014	0.05162	0.0126	0.0126	yellow
SARS	-0.00101	-0.08582	-0.07719	-0.02706	-0.09637	-0.01926	-0.00424	0.14964	-0.14964	0.98952	0.2644	0.31562	0.72535	0.2099	0.80252	0.95607	0.05077	0.05077	turquoise
MYL5	-0.10802	0.01703	-0.12751	-0.10413	0.16857	-0.01531	0.02004	-0.17953	0.17953	0.15963	0.82505	0.09652	0.17531	0.02753	0.84244	0.79478	0.0188	0.0188	red
PTPN6	-0.01033	-0.02364	0.04311	-0.04479	0.0688	-0.0373	-0.09564	0.09243	-0.09243	0.89334	0.75887	0.57561	0.5608	0.37123	0.62817	0.21337	0.22921	0.22921	blue
HTATIP2	0.10618	-0.0359	0.08188	0.03702	-0.0371	0.10488	0.20429	-0.48136	0.48136	0.1669	0.64106	0.28705	0.63071	0.63002	0.17219	0.00736	2.65E-11	2.65E-11	turquoise
PTH1R	-0.05538	-0.03941	-0.08303	0.05278	-0.04287	-0.02504	0.00645	0.33097	-0.33097	0.47184	0.60885	0.28029	0.493	0.57772	0.74509	0.93328	9.80E-06	9.80E-06	blue
MEFV	-0.15539	-0.02456	0.11598	0.04684	-0.17328	0.09539	0.07014	0.34182	-0.34182	0.04241	0.74981	0.13087	0.54297	0.02342	0.21457	0.36197	4.75E-06	4.75E-06	blue
RPP30	0.03921	-0.00848	-0.01394	-0.01272	-0.12901	-0.07402	0.19	-0.07127	0.07127	0.61062	0.91232	0.85641	0.86881	0.09262	0.33598	0.01281	0.35425	0.35425	turquoise
FBXO21	-0.00823	-0.11695	-0.13537	-0.0163	0.02	-0.07396	-0.17103	0.26558	-0.26558	0.9149	0.12767	0.07751	0.83238	0.79509	0.33634	0.02531	0.00045	0.00045	turquoise
RASSF5	-0.02674	-0.08454	0.03788	0.0586	-0.19064	0.13304	0.11565	0.15971	-0.15971	0.72848	0.2716	0.62282	0.44642	0.0125	0.0828	0.132	0.03693	0.03693	blue
RPS6KL1	-0.01182	-0.02204	-0.13636	-0.14661	0.28977	-0.2361	-0.19006	-0.05988	0.05988	0.878	0.77477	0.07534	0.0557	0.00012	0.00188	0.01278	0.43657	0.43657	grey
AFAP1	-0.06273	-0.10889	0.09909	0.06535	-0.15693	0.20209	0.111	0.03813	-0.03813	0.41501	0.15627	0.19726	0.3958	0.04038	0.00803	0.14835	0.62053	0.62053	turquoise
NECAB2	-0.00998	-0.08824	-0.23918	-0.13205	0.07516	-0.35614	-0.14225	0.41895	-0.41895	0.8969	0.2511	0.00163	0.08513	0.32857	1.75E-06	0.06346	1.18E-08	1.18E-08	brown
TRDMT1	-0.02644	-0.07733	-0.03974	0.03923	-0.09083	-0.07999	-0.12154	0.37176	-0.37176	0.73136	0.31477	0.6058	0.61041	0.23743	0.29836	0.1133	5.55E-07	5.55E-07	turquoise
ZNF141	-0.05526	-0.17304	-0.07154	0.02211	-0.0518	-0.02609	-0.09302	0.26181	-0.26181	0.47283	0.02362	0.35244	0.77403	0.501	0.73485	0.22626	0.00054	0.00054	turquoise
SLC39A3	0.02918	0.06071	-0.22117	-0.14898	0.17797	-0.26302	-0.03376	-0.08123	0.08123	0.70476	0.43027	0.00365	0.05181	0.01987	0.00051	0.66112	0.29086	0.29086	green
TRMT1	0.06529	0.06721	-0.07216	-0.03716	0.19956	-0.12683	0.01919	-0.23882	0.23882	0.39624	0.3824	0.34827	0.62946	0.00887	0.09831	0.80328	0.00166	0.00166	red
KLF2	-0.05382	0.07194	0.05551	0.01728	0.0385	0.10655	0.03092	-0.01845	0.01845	0.4845	0.34974	0.47081	0.82252	0.61712	0.16543	0.68811	0.8107	0.8107	grey
RAB2B	-0.06851	-0.20335	-0.04514	0.04171	-0.01459	-0.06386	-0.11925	0.25147	-0.25147	0.37324	0.00764	0.5577	0.58808	0.84978	0.40665	0.12032	0.00091	0.00091	turquoise
DYRK1B	0.00473	-0.227	-0.18651	-0.00787	0.04379	-0.24829	-0.11787	-0.06033	0.06033	0.95107	0.00283	0.01458	0.91864	0.56953	0.00106	0.12469	0.43314	0.43314	grey
KNL1	-0.05004	0.04502	0.04425	0.01302	-0.09481	0.11139	0.29967	-0.387	0.387	0.51568	0.55878	0.56551	0.86575	0.21738	0.14694	6.84E-05	1.71E-07	1.71E-07	pink
CEP44	-0.03859	-0.10014	-0.0913	-0.05324	0.09021	-0.03673	-0.07462	0.03689	-0.03689	0.61633	0.19253	0.23496	0.48918	0.24064	0.63336	0.33208	0.63189	0.63189	turquoise
CCNA2	-0.07997	0.01929	0.048	-0.03092	-0.01192	0.10593	0.28423	-0.43658	0.43658	0.29846	0.8023	0.53296	0.68812	0.87708	0.16792	0.00016	2.38E-09	2.38E-09	pink
PCDHB7	-0.06414	0.00807	-0.1595	0.05142	-0.10972	-0.0649	-0.01487	0.44299	-0.44299	0.40462	0.91653	0.03718	0.50417	0.15311	0.39903	0.84696	1.30E-09	1.30E-09	blue
JCAD	-0.08546	-0.17374	-0.04178	0.11605	-0.25556	0.06302	-0.02714	0.47574	-0.47574	0.26642	0.02305	0.58745	0.13067	0.00074	0.41284	0.72456	4.84E-11	4.84E-11	blue
TRUB2	-0.03698	-0.01671	-0.13811	-0.0955	-0.08128	-0.11335	0.0811	-0.05275	0.05275	0.63111	0.82829	0.07164	0.21407	0.29062	0.13992	0.29167	0.49324	0.49324	turquoise
WIPF3	0.01878	-0.13324	-0.12867	-0.01051	0.03916	-0.10464	-0.26712	0.3154	-0.3154	0.8074	0.08234	0.09349	0.89148	0.61107	0.17318	0.00041	2.65E-05	2.65E-05	brown
TMEM256	-0.07021	-0.01492	-0.06773	-0.06753	0.10376	-0.08667	0.10918	-0.17716	0.17716	0.36147	0.84638	0.37875	0.38018	0.17683	0.25967	0.15519	0.02045	0.02045	grey
AJUBA	-0.01402	-0.01428	0.00699	0.13578	-0.15372	0.17164	0.26175	-0.19141	0.19141	0.85554	0.85295	0.92768	0.07661	0.04471	0.02479	0.00054	0.01215	0.01215	turquoise
SHOC2	-0.01009	-0.17525	0.007	0.02209	-0.14472	0.03079	-0.01007	0.18079	-0.18079	0.89579	0.02187	0.92757	0.7743	0.05895	0.68929	0.896	0.01796	0.01796	turquoise
FGFR3	-0.04217	0.01659	-0.06697	-0.00762	0.16844	0.02797	0.01439	-0.06401	0.06401	0.58395	0.82944	0.38412	0.92116	0.02765	0.71649	0.85185	0.40552	0.40552	grey
SREBF1	-0.00127	-0.126	-0.11987	-0.10863	0.18084	-0.20577	-0.07773	-0.04219	0.04219	0.98685	0.10055	0.11838	0.15729	0.01794	0.00693	0.31224	0.58374	0.58374	grey
UHRF1	-0.08159	0.118	0.03419	0.01603	-0.08749	0.07183	0.29643	-0.33933	0.33933	0.28875	0.12427	0.65711	0.83516	0.25516	0.35048	8.26E-05	5.62E-06	5.62E-06	pink
GZMM	-0.06351	0.05431	0.00028	0.07653	-0.06087	0.05724	-0.03021	0.40084	-0.40084	0.40925	0.48051	0.9971	0.3198	0.42905	0.45711	0.69491	5.55E-08	5.55E-08	blue
EGFL8	-0.01416	-0.06627	-0.11344	-0.01553	0.22801	-0.11872	-0.04958	-0.04159	0.04159	0.85418	0.38917	0.1396	0.84026	0.00271	0.12198	0.51963	0.58915	0.58915	red
FLNC	-0.02925	0.01842	0.04118	0.06562	-0.06578	0.10532	0.102	0.16761	-0.16761	0.70411	0.811	0.59284	0.3938	0.39265	0.17039	0.18433	0.02843	0.02843	salmon
C1QBP	-0.06603	0.00854	-0.10395	-0.15425	0.04219	-0.2159	0.01476	0.09193	-0.09193	0.39085	0.91175	0.17605	0.04397	0.5838	0.00457	0.84809	0.23176	0.23176	grey
RPA2	-0.01699	-0.10599	-0.01175	-0.02472	-0.15583	0.06831	0.01768	0.22913	-0.22913	0.82544	0.16767	0.87875	0.7483	0.04182	0.37471	0.81848	0.00257	0.00257	turquoise
FKBP11	0.00983	0.07585	-0.02409	0.05599	0.15187	-0.24549	-0.16571	0.22892	-0.22892	0.89846	0.32416	0.75445	0.46701	0.04737	0.00121	0.03031	0.0026	0.0026	grey
TMEM106	-0.01444	-0.05963	0.17579	0.1183	-0.17514	0.27195	0.24496	0.03923	-0.03923	0.85129	0.4385	0.02146	0.12331	0.02195	0.00032	0.00124	0.61042	0.61042	turquoise
TMED4	-0.00525	-0.22403	-0.11289	-0.06857	0.10457	-0.21008	-0.06155	-0.09084	0.09084	0.94567	0.00322	0.14153	0.37288	0.17347	0.00582	0.4239	0.23737	0.23737	turquoise
BOD1L1	-0.07685	-0.15288	-0.0266	0.03181	-0.12392	0.08557	0.00305	0.26555	-0.26555	0.31774	0.04591	0.72985	0.67954	0.10635	0.26582	0.96846	0.00045	0.00045	turquoise
MAOA	0.05512	-0.127	-0.04354	0.02208	0.04806	0.01156	-0.09189	-0.0209	0.0209	0.47394	0.09787	0.57178	0.77435	0.53248	0.88075	0.23196	0.78612	0.78612	grey
SLC9A3	-0.10749	0.03779	0.10272	-0.05335	0.13292	0.07335	-0.03642	-0.1339	0.1339	0.1617	0.62361	0.18124	0.4883	0.08308	0.34039	0.63624	0.08082	0.08082	grey
PPP1R37	0.13325	-0.10305	-0.15545	-0.11544	0.25826	-0.16342	-0.02074	-0.22788	0.22788	0.08231	0.17985	0.04234	0.13272	0.00065	0.03271	0.78773	0.00272	0.00272	green
RPL10	0.09446	0.05625	0.08243	0.03919	-0.17438	0.20437	0.12256	-0.15061	0.15061	0.21912	0.46494	0.2838	0.61077	0.02255	0.00734	0.11027	0.04927	0.04927	purple
PRR4	0.01409	-0.01762	-0.02527	0.00621	0.09982	-0.04106	-0.00658	-0.02694	0.02694	0.85485	0.81903	0.74281	0.93576	0.19397	0.59389	0.93197	0.72652	0.72652	grey
KDM1A	0.02608	-0.11428	-0.00934	0.02738	-0.06903	0.04846	0.12305	-0.09081	0.09081	0.73491	0.13668	0.90352	0.72224	0.36963	0.52904	0.10886	0.23751	0.23751	turquoise
CUX1	0.11435	-0.01352	0.01962	-0.00116	-0.01252	0.03418	0.06388	-0.06028	0.06028	0.13643	0.86065	0.79893	0.98794	0.87087	0.65714	0.40651	0.43351	0.43351	turquoise
HIST1H2B	0.01355	-0.12576	0.08189	0.0596	-0.07305	0.10792	0.07171	-0.18434	0.18434	0.86042	0.10122	0.28699	0.43871	0.34238	0.16001	0.35133	0.0158	0.0158	grey
ATXN2	0.08076	-0.20516	-0.07722	-0.02079	0.08534	-0.11479	-0.02642	0.08081	-0.08081	0.29367	0.00711	0.31542	0.78727	0.2671	0.13493	0.73159	0.2934	0.2934	turquoise
SMIM12	-0.06293	-0.07939	0.00107	-0.03869	-0.07603	0.04883	0.00586	0.0554	-0.0554	0.41352	0.30201	0.98889	0.61535	0.32295	0.52597	0.93936	0.47173	0.47173	turquoise
IGFBPL1	-0.09232	-0.14927	-0.15409	-0.03179	0.03992	-0.27535	-0.12209	0.43619	-0.43619	0.22975	0.05135	0.04419	0.6798	0.60414	0.00027	0.11167	2.47E-09	2.47E-09	brown
RPS13	0.12513	0.14475	0.04157	-0.05468	-0.07669	0.09421	0.14517	-0.19895	0.19895	0.10296	0.0589	0.58932	0.40064	0.31879	0.22031	0.05816	0.00909	0.00909	purple
FKBP1C	-0.11036	0.12087	0.03142	0.00594	-0.06319	-0.00444	0.02924	0.12968	-0.12968	0.15075	0.11532	0.68326	0.93851	0.4116	0.95403	0.7042	0.09093	0.09093	grey
TRIM39	-0.04471	-0.08221	-0.16392	-0.05641	0.0898	-0.16543	-0.15308	0.20714	-0.20714	0.56149	0.28507	0.03217	0.46367	0.24278	0.0306	0.04563	0.00656	0.00656	turquoise
SUV39H2	-0.04647	-0.01872	-0.03601	-0.00929	-0.09224	0.01337	0.17643	0.01661	-0.01661	0.54616	0.80799	0.64008	0.90399	0.23015	0.86222	0.02098	0.82925	0.82925	turquoise
CUL5	-0.08136	-0.13704	-0.07243	0.0398	-0.08304	0.01073	0.03801	0.24071	-0.24071	0.29009	0.07389	0.34645	0.6053	0.28022	0.88921	0.62161	0.00152	0.00152	turquoise
C11orf91	-0.01256	-0.05272	0.06673	-0.00396	-0.07801	0.1141	0.05039	-0.2829	0.2829	0.87051	0.4935	0.38581	0.95901	0.31048	0.1373	0.5128	0.00018	0.00018	grey
ZFAT	0.03177	-0.03792	-0.04337	0.01478	-0.19143	0.04177	0.07098	0.09583	-0.09583	0.68001	0.62244	0.57328	0.84789	0.01214	0.58753	0.35626	0.21248	0.21248	turquoise
DRD4	0.08671	0.00631	-0.0557	-0.02523	0.13695	0.03599	0.05621	-0.34774	0.34774	0.25947	0.93476	0.46933	0.74327	0.07407	0.64029	0.46523	3.16E-06	3.16E-06	green
CHAF1B	-0.09723	0.08625	-0.19228	-0.01076	0.03908	-0.06704	0.19441	-0.15684	0.15684	0.20584	0.26199	0.01175	0.88894	0.61182	0.38365	0.01084	0.04051	0.04051	grey
EXT2	-0.03059	-0.17307	0.02685	0.07824	-0.21509	0.07299	0.11884	0.07236	-0.07236	0.69126	0.02359	0.72739	0.30909	0.00472	0.34275	0.12159	0.34698	0.34698	turquoise
CTSS	-0.00962	0.03984	0.08465	0.05918	-0.08936	0.26762	0.18226	-0.02172	0.02172	0.90067	0.60486	0.271	0.44199	0.2451	0.0004	0.01704	0.77794	0.77794	turquoise
DNAL1	-0.00527	-0.17234	-0.16867	-0.02095	0.05363	-0.14592	-0.11363	0.28008	-0.28008	0.94542	0.0242	0.02743	0.78562	0.48602	0.05686	0.13894	0.00021	0.00021	turquoise
ENOSF1	0.00987	-0.16609	-0.05384	-0.03273	0.31056	-0.06146	-0.00067	-0.13065	0.13065	0.89809	0.02993	0.48427	0.67085	3.57E-05	0.42457	0.99301	0.08853	0.08853	red
ZFP36L2	-0.04142	-0.01923	0.08418	0.05188	-0.12244	0.29985	0.16192	-0.03802	0.03802	0.59067	0.80291	0.27363	0.50035	0.11062	6.77E-05	0.03436	0.62152	0.62152	turquoise
JRK	0.07242	-0.11516	-0.0734	-0.0726	0.0459	-0.17558	0.00145	-0.07839	0.07839	0.34658	0.13364	0.34003	0.34535	0.55108	0.02162	0.98498	0.30813	0.30813	red
B3GNT7	0.04645	0.08191	0.14328	-0.03496	-0.00114	0.23373	0.09295	-0.38126	0.38126	0.54632	0.28687	0.06155	0.64989	0.98822	0.00209	0.22658	2.68E-07	2.68E-07	yellow
BCL10	-0.02352	-0.10815	0.13139	0.07526	-0.00414	0.26229	0.11185	-0.27506	0.27506	0.76013	0.15913	0.08672	0.32795	0.95717	0.00053	0.14527	0.00027	0.00027	turquoise
ISG20	-0.02632	0.03612	0.00704	0.03047	0.00201	0.07591	0.04837	-0.19836	0.19836	0.73258	0.6391	0.92722	0.69236	0.9792	0.32377	0.52984	0.0093	0.0093	grey
EMC6	0.02254	-0.07389	-0.11112	-0.09113	0.22541	-0.13028	0.05571	-0.17101	0.17101	0.76983	0.33681	0.14793	0.23587	0.00303	0.08943	0.4692	0.02533	0.02533	green
WDR48	-0.05219	-0.18993	-0.08681	-0.10905	-0.15979	0.03651	0.01263	0.23132	-0.23132	0.49783	0.01284	0.25889	0.15567	0.03683	0.63547	0.86978	0.00233	0.00233	turquoise
NPC1L1	-0.02004	-0.05501	-0.08007	-0.06006	0.34893	-0.01544	-0.24127	-0.01703	0.01703	0.79475	0.47483	0.29787	0.43523	2.91E-06	0.84113	0.00148	0.82508	0.82508	grey
MYO5A	-0.09574	-0.13058	-0.06992	0.03145	-0.20992	-0.09897	-0.08255	0.56305	-0.56305	0.21288	0.0887	0.36351	0.68303	0.00586	0.19779	0.2831	1.09E-15	1.09E-15	blue
DCLRE1C	0.0612	0.00431	0.0825	0.01375	-0.09863	0.12434	0.09971	-0.09883	0.09883	0.42653	0.9554	0.28338	0.85838	0.19936	0.10516	0.19446	0.19842	0.19842	turquoise
RPL36	0.138	0.17388	-0.03979	-0.062	0.04724	-0.01023	0.12136	-0.32387	0.32387	0.07186	0.02294	0.60538	0.42051	0.5395	0.8944	0.11385	1.55E-05	1.55E-05	purple
EIF1AD	0.01203	-0.22258	-0.05584	-0.03109	-0.13514	0.04959	0.09147	-0.13501	0.13501	0.8759	0.00343	0.46821	0.68649	0.07801	0.51947	0.23411	0.0783	0.0783	turquoise
ABCC5	-0.10183	-0.15383	-0.11333	-0.07363	0.05976	-0.12341	-0.03974	0.17653	-0.17653	0.18507	0.04456	0.13996	0.33851	0.43753	0.10781	0.60577	0.02091	0.02091	turquoise
AKIRIN1	-0.06358	-0.15849	-0.02419	-0.01812	0.0148	0.00669	-0.14237	0.3056	-0.3056	0.40873	0.03841	0.75345	0.81408	0.84767	0.93075	0.06324	4.81E-05	4.81E-05	turquoise
TERF1	-0.0283	-0.05328	-0.01838	-0.03191	-0.09543	0.03224	-0.01027	0.18249	-0.18249	0.71327	0.48885	0.81141	0.67861	0.21437	0.67553	0.8939	0.0169	0.0169	turquoise
OLFML1	-0.09776	-0.06466	0.02956	0.05793	-0.22928	0.10402	-0.00102	0.45087	-0.45087	0.20334	0.4008	0.70113	0.45168	0.00256	0.17577	0.98944	6.08E-10	6.08E-10	blue
FAM49A	-0.12252	-0.06217	0.07228	0.06349	-0.2785	0.15049	0.04494	0.46158	-0.46158	0.1104	0.41918	0.34746	0.40941	0.00023	0.04945	0.55946	2.10E-10	2.10E-10	blue
CERK	-0.12789	-0.18538	-0.17417	-0.03224	-0.02209	-0.21839	-0.20453	0.49621	-0.49621	0.09552	0.01521	0.02271	0.67551	0.77432	0.00411	0.00729	5.15E-12	5.15E-12	brown
ARHGDIG	-0.05076	0.05843	#######	0.0181	0.02623	-0.02704	-0.09187	0.1872	-0.1872	0.50971	0.44779	0.99983	0.81427	0.73341	0.72555	0.23206	0.01422	0.01422	magenta
ANKZF1	0.11047	-0.08568	-0.05387	-0.04329	0.19267	-0.02175	0.03803	-0.37993	0.37993	0.15034	0.26517	0.48409	0.574	0.01158	0.77764	0.62139	2.97E-07	2.97E-07	red
PPM1K	-0.0736	-0.12365	-0.12987	-0.02041	-0.06978	-0.17339	-0.21293	0.58876	-0.58876	0.3387	0.10713	0.09047	0.79104	0.36446	0.02333	0.00517	2.47E-17	2.47E-17	brown
SENP5	0.00116	-0.20592	-0.1006	-0.0775	-0.04337	-0.02243	0.04326	0.03763	-0.03763	0.98798	0.00689	0.19046	0.31369	0.57331	0.77094	0.57423	0.6251	0.6251	turquoise
ADAM23	-0.10718	-0.22508	-0.04963	0.04011	-0.1148	-0.05319	-0.07069	0.53306	-0.53306	0.16291	0.00308	0.51915	0.60248	0.13488	0.48964	0.35819	6.12E-14	6.12E-14	blue
ACOT1	-0.0269	-0.04383	0.07927	0.11361	0.00873	0.03007	-0.08191	0.05521	-0.05521	0.72693	0.56919	0.3027	0.13901	0.9098	0.69627	0.28686	0.47326	0.47326	grey
POLA1	-0.08076	-0.05313	-0.04969	-0.02529	-0.1214	-0.00366	0.08611	0.14499	-0.14499	0.29371	0.49008	0.51867	0.74264	0.1137	0.96209	0.26279	0.05848	0.05848	turquoise
LOXL4	-0.039	-0.18699	-0.07242	0.11817	-0.1133	-0.15733	0.03115	0.07078	-0.07078	0.61257	0.01433	0.34656	0.12372	0.14007	0.03988	0.68584	0.35758	0.35758	grey
TMEM167	-0.0308	-0.13117	-0.00152	0.03015	-0.08785	0.07425	-0.03198	0.19592	-0.19592	0.68924	0.08725	0.98431	0.6955	0.25322	0.33444	0.67795	0.01023	0.01023	turquoise
HPCAL1	0.07428	0.0216	0.07804	0.09496	0.03049	0.13516	0.16044	-0.47148	0.47148	0.33429	0.77911	0.31033	0.21668	0.69217	0.07798	0.03606	7.57E-11	7.57E-11	yellow
IRF3	0.11089	0.00449	-0.01104	-0.05157	0.07844	0.07607	0.13054	-0.38123	0.38123	0.14876	0.9535	0.88604	0.5029	0.30781	0.32274	0.0888	2.69E-07	2.69E-07	red
ITPR3	-0.00719	0.05023	0.07347	0.057	-0.07461	0.26857	0.18985	-0.2775	0.2775	0.92568	0.51413	0.33957	0.45896	0.3321	0.00038	0.01288	0.00024	0.00024	turquoise
MRPL58	0.01922	-0.03311	-0.16318	-0.09471	0.06806	-0.16451	0.11684	-0.28096	0.28096	0.80298	0.66728	0.03297	0.21786	0.37644	0.03155	0.12803	0.0002	0.0002	grey
PRDM4	0.00474	-0.14554	-0.03883	-0.02852	-0.03766	-0.06235	-0.04596	0.24948	-0.24948	0.95098	0.05753	0.6141	0.71114	0.6248	0.41785	0.55056	0.001	0.001	turquoise
MUC15	0.00313	0.02481	0.05441	0.21692	-0.14088	0.14768	-0.01229	0.08189	-0.08189	0.96755	0.74736	0.47972	0.00437	0.06607	0.05391	0.87328	0.28697	0.28697	magenta
GLTPD2	-0.0741	-0.17333	-0.09537	-0.13622	0.27056	-0.19491	-0.17003	0.06992	-0.06992	0.33547	0.02338	0.21469	0.07564	0.00034	0.01063	0.02619	0.36349	0.36349	grey
SMUG1	0.11547	-0.01826	-0.0787	0.01068	0.19031	-0.10228	0.0482	-0.43907	0.43907	0.13259	0.81257	0.30624	0.88977	0.01266	0.18312	0.53132	1.89E-09	1.89E-09	grey
PCDH7	-0.06192	-0.10808	0.02544	0.06307	-0.2766	0.17541	0.19289	0.03749	-0.03749	0.42111	0.15938	0.74118	0.41248	0.00025	0.02175	0.01148	0.62638	0.62638	black
ASPRV1	0.05951	0.02583	-0.13039	-0.08246	-0.03529	0.03407	0.00867	0.26639	-0.26639	0.43939	0.73735	0.08915	0.28362	0.64679	0.65819	0.91039	0.00043	0.00043	grey

ROGDI	0.06001	-0.10175	-0.18543	-0.1301	0.22459	-0.28701	-0.1746	-0.00902	0.00902	0.43555	0.18541	0.01518	0.08989	0.00315	0.00014	0.02237	0.90681	0.90681	grey
MRM3	-0.11791	0.01969	-0.15524	-0.0634	-0.02335	-0.14518	-0.03076	0.18197	-0.18197	0.12455	0.79827	0.04262	0.41004	0.76181	0.05815	0.68957	0.01722	0.01722	grey
TRIP13	-0.04854	0.04701	0.0065	0.03749	-0.03078	-0.01957	0.25318	-0.3433	0.3433	0.52837	0.54149	0.9328	0.62639	0.68946	0.7995	0.00083	4.29E-06	4.29E-06	pink
C1orf131	0.02254	0.04867	-0.03858	-0.05393	0.10218	0.06683	0.07187	-0.13879	0.13879	0.76978	0.52732	0.61642	0.48358	0.18356	0.38516	0.3502	0.07024	0.07024	turquoise
OCLN	0.05107	-0.06471	0.02044	0.06822	0.01185	0.19328	0.04052	-0.25881	0.25881	0.50707	0.40041	0.7907	0.3753	0.87775	0.01131	0.59879	0.00063	0.00063	turquoise
LRRFIP2	0.00254	-0.17215	-0.01376	-0.02877	-0.07444	0.10872	0.08223	-0.05435	0.05435	0.97367	0.02435	0.85823	0.70873	0.33324	0.15695	0.28497	0.48017	0.48017	turquoise
SEL1L	-0.03626	-0.03357	0.04327	0.11268	-0.18654	0.11528	-0.062	0.30293	-0.30293	0.63773	0.66295	0.57419	0.14228	0.01457	0.13325	0.42047	5.64E-05	5.64E-05	turquoise
ZNF746	-0.01383	-0.17915	-0.0888	-0.03468	0.04855	-0.1763	-0.08367	0.20238	-0.20238	0.85756	0.01905	0.24807	0.65247	0.52829	0.02108	0.27659	0.00794	0.00794	turquoise
DUSP6	-0.08484	-0.08651	-0.00218	-0.01893	-0.05842	0.07762	0.11123	-0.26697	0.26697	0.26992	0.26058	0.97745	0.80586	0.44784	0.31294	0.14753	0.00042	0.00042	grey
PRR14L	-0.05005	-0.14338	-0.1202	-0.03462	-0.0252	-0.03864	-0.02731	0.17163	-0.17163	0.5156	0.06136	0.11736	0.65304	0.74355	0.6158	0.7229	0.02479	0.02479	turquoise
SLC38A5	0.04928	0.06851	0.20341	0.12669	-0.12662	0.18401	0.26	-0.26643	0.26643	0.52208	0.37325	0.00762	0.0987	0.09888	0.01599	0.00059	0.00043	0.00043	grey
SLC38A11	0.06121	-0.07326	-0.16627	0.02062	0.09769	-0.07043	-0.14332	0.28494	-0.28494	0.42642	0.34099	0.02974	0.78893	0.20369	0.36001	0.06147	0.00016	0.00016	grey
SLC25A10	0.09159	0.0815	0.01855	0.00263	0.19604	0.01955	0.22103	-0.57474	0.57474	0.23347	0.28927	0.80969	0.97272	0.01018	0.79968	0.00367	2.03E-16	2.03E-16	green
EEF2K	-0.02627	0.0115	0.01382	0.10397	-0.08822	0.1791	0.05879	-0.00196	0.00196	0.73308	0.88128	0.85763	0.17594	0.2512	0.01909	0.44495	0.97971	0.97971	turquoise
ARL9	0.10947	0.16201	-0.0573	-0.03192	0.11357	0.01095	0.0941	-0.09664	0.09664	0.15406	0.03426	0.45665	0.67851	0.13915	0.88694	0.22085	0.20859	0.20859	grey
SLITRK3	0.09582	-0.013	-0.0002	0.00262	-0.15214	0.11644	0.0498	0.28717	-0.28717	0.21249	0.86601	0.99791	0.97284	0.04699	0.12937	0.51775	0.00014	0.00014	blue
LYN	-0.0929	0.07721	0.04635	0.01093	-0.13345	0.18173	0.13237	0.00836	-0.00836	0.22687	0.31553	0.54719	0.88714	0.08184	0.01737	0.08437	0.91363	0.91363	turquoise
PITPNC1	0.00805	-0.16827	-0.07838	-0.01341	-0.02222	-0.17748	-0.08181	0.27477	-0.27477	0.91678	0.02781	0.30819	0.86177	0.77297	0.02022	0.28745	0.00028	0.00028	turquoise
CABCOCC	-0.0985	-0.15343	-0.20053	-0.11165	0.02108	-0.10247	-0.17654	0.24895	-0.24895	0.19993	0.04512	0.00854	0.14598	0.78434	0.18231	0.0209	0.00103	0.00103	grey
PLCB4	-0.00118	-0.04672	-0.03171	-0.05342	0.16159	-0.05191	-0.10772	0.18801	-0.18801	0.98781	0.54396	0.68059	0.48774	0.03473	0.5001	0.16079	0.0138	0.0138	grey
C2orf15	0.05417	-0.06567	-0.18538	-0.1027	0.09204	-0.16077	-0.09345	-0.1159	0.1159	0.48165	0.39342	0.01521	0.18133	0.23121	0.03567	0.22408	0.13116	0.13116	grey
VTN	-0.00707	-0.10561	-0.29309	-0.06219	0.15306	-0.21882	-0.26202	0.21831	-0.21831	0.92691	0.16922	0.0001	0.41908	0.04565	0.00403	0.00054	0.00412	0.00412	magenta
TAF6	0.02755	-0.1673	-0.02105	-0.00568	0.02472	-0.0059	-0.04605	0.03929	-0.03929	0.72055	0.02874	0.78461	0.94119	0.74822	0.93895	0.54977	0.60994	0.60994	turquoise
PNKD	0.04761	-0.04399	0.05588	0.03952	0.07522	0.14249	0.16024	-0.31974	0.31974	0.53629	0.56782	0.46784	0.6078	0.32816	0.06302	0.0363	2.02E-05	2.02E-05	yellow
SMIM30	-0.06096	-0.10907	-0.04504	-0.00984	0.0566	-0.0714	-0.04303	0.04545	-0.04545	0.42832	0.1556	0.55862	0.89837	0.46217	0.35339	0.57626	0.55504	0.55504	turquoise
MAP3K5	-0.03916	-0.06133	0.10885	-0.00235	-0.14241	0.22379	0.05929	-0.01624	0.01624	0.61112	0.42552	0.15642	0.9757	0.06316	0.00326	0.44111	0.83304	0.83304	turquoise
SPATA33	0.03272	-0.01028	-0.15988	-0.12792	0.11104	-0.15839	0.01265	-0.18676	0.18676	0.67097	0.89389	0.03672	0.09545	0.14823	0.03853	0.86958	0.01445	0.01445	grey
MTHFD2	0.02315	0.04005	0.11665	0.09535	-0.18106	0.18257	0.14474	-0.01207	0.01207	0.76375	0.60296	0.12866	0.21476	0.01779	0.01685	0.05891	0.87545	0.87545	pink
RSBN1L	0.00246	-0.1771	-0.01562	0.0426	0.09811	0.01239	0.02122	0.04043	-0.04043	0.97449	0.02049	0.83933	0.58007	0.20175	0.87221	0.78297	0.59953	0.59953	turquoise
SLC25A13	0.00983	-0.14866	-0.00059	-0.02566	0.07488	0.06576	0.13893	-0.06891	0.06891	0.89849	0.05231	0.99394	0.73904	0.33038	0.39283	0.06996	0.37052	0.37052	turquoise
ANP32A	0.03083	-0.03482	-0.07747	-0.04953	-0.00874	-0.03429	0.06272	-0.18139	0.18139	0.68896	0.65117	0.3139	0.51999	0.90969	0.65617	0.41508	0.01758	0.01758	turquoise
DCAF10	-0.06932	-0.11761	-0.18165	-0.0615	-0.10732	-0.0783	-0.03554	0.24689	-0.24689	0.36766	0.12554	0.01741	0.42423	0.16238	0.30868	0.64449	0.00113	0.00113	turquoise
GPR82	-0.03824	-0.04385	0.04922	-0.06766	-0.16581	0.10601	0.07238	0.31001	-0.31001	0.61949	0.56899	0.52261	0.37928	0.0302	0.1676	0.34684	3.69E-05	3.69E-05	blue
ZBTB7B	0.02305	-0.0352	0.01818	-0.03501	0.05215	0.162	0.10516	-0.34324	0.34324	0.76475	0.64762	0.81341	0.64938	0.49814	0.03427	0.17103	4.31E-06	4.31E-06	turquoise
CXorf40B	-0.01071	-0.15909	-0.09831	-0.08007	0.0597	-0.07111	0.05726	0.00422	-0.00422	0.88947	0.03767	0.20083	0.29783	0.43793	0.35536	0.45697	0.95633	0.95633	grey
UBOX5	0.0196	-0.03977	-0.2314	-0.10352	0.11014	-0.31112	-0.08071	0.15244	-0.15244	0.79913	0.60555	0.00233	0.17787	0.15156	3.45E-05	0.29398	0.04654	0.04654	grey
AP2B1	-0.07109	-0.16106	-0.05857	-0.00818	-0.00368	-0.00255	0.0484	-0.05605	0.05605	0.3555	0.03535	0.44669	0.9154	0.96192	0.97362	0.52954	0.46656	0.46656	turquoise
KIAA0355	-0.05904	-0.225	-0.05732	0.02228	-0.15316	0.05441	-0.08133	0.46287	-0.46287	0.44307	0.00309	0.45651	0.77239	0.04551	0.47972	0.29028	1.84E-10	1.84E-10	turquoise
ZNF575	0.08734	0.04301	-0.12734	-0.06805	0.1368	-0.04281	-0.05151	0.11981	-0.11981	0.25598	0.57644	0.09696	0.37651	0.07439	0.57825	0.50348	0.11857	0.11857	grey
ZNFX1	0.03779	-0.10813	0.01584	0.08472	-0.1561	0.10539	0.13045	-0.04158	0.04158	0.62359	0.15919	0.8371	0.27058	0.04147	0.17011	0.08902	0.58925	0.58925	tan
FAS	-0.10572	-0.12798	0.16477	0.10075	-0.20945	0.21738	0.19229	0.10633	-0.10633	0.16875	0.09529	0.03127	0.1898	0.00597	0.00429	0.01175	0.16631	0.16631	turquoise
SRP9	-0.01786	-0.03427	-0.12959	-0.0752	0.05621	-0.0752	0.0089	-0.07573	0.07573	0.81666	0.65635	0.09115	0.32828	0.46526	0.3283	0.90805	0.32489	0.32489	turquoise
WDR88	-0.12861	-0.20447	-0.17844	-0.09001	0.12203	-0.19125	-0.07649	0.16637	-0.16637	0.09366	0.00731	0.01954	0.24167	0.11183	0.01222	0.32004	0.02964	0.02964	grey
NT5E	-0.00827	-0.05544	0.08956	0.08241	-0.21266	0.12841	0.25196	-0.21692	0.21692	0.91453	0.4714	0.24404	0.28389	0.00523	0.09418	0.00089	0.00438	0.00438	turquoise
NRARP	-0.01413	-0.04755	0.05244	0.0887	0.03955	0.17647	0.08251	-0.12824	0.12824	0.85442	0.53689	0.49574	0.24866	0.6075	0.02095	0.28333	0.0946	0.0946	grey
TESPA1	-0.07875	-0.07519	0.05956	0.03998	-0.16375	0.10923	-0.06607	0.44715	-0.44715	0.30593	0.3284	0.43902	0.60365	0.03235	0.15496	0.39059	8.73E-10	8.73E-10	blue
CELF2	-0.11748	-0.08688	-0.01096	0.06049	-0.18113	-0.00809	-0.07303	0.58974	-0.58974	0.12595	0.25853	0.8869	0.43189	0.01775	0.91639	0.34248	2.13E-17	2.13E-17	blue
SNAP47	0.01076	-0.01297	-0.15705	-0.09768	-0.01947	-0.11726	0.0865	0.12927	-0.12927	0.88887	0.8663	0.04023	0.20372	0.80041	0.12665	0.26063	0.09196	0.09196	turquoise
PIGR	0.01548	-0.03043	0.12545	0.05973	0.03027	0.30414	0.05119	-0.1243	0.1243	0.84069	0.69274	0.10208	0.4377	0.69434	5.25E-05	0.50614	0.10527	0.10527	yellow
ITGA7	-0.10649	-0.05667	-0.13776	0.00308	0.11097	-0.12367	-0.17757	0.47509	-0.47509	0.16566	0.4616	0.07238	0.96809	0.14849	0.10707	0.02015	5.18E-11	5.18E-11	salmon
COL6A1	-0.12488	-0.05414	0.04162	0.0918	-0.06879	0.03199	0.0587	0.2612	-0.2612	0.10365	0.48184	0.58884	0.23244	0.37134	0.67784	0.4457	0.00056	0.00056	black
TTC39B	0.03511	-0.05588	-0.02773	-0.07296	0.02617	0.04644	-0.00584	0.11701	-0.11701	0.64848	0.46786	0.71884	0.34298	0.73403	0.54636	0.93952	0.12747	0.12747	turquoise
IGSF9	0.04352	0.00605	0.12389	0.01341	0.00482	0.19062	0.22245	-0.47885	0.47885	0.57191	0.9374	0.10644	0.86185	0.9501	0.01251	0.00345	3.48E-11	3.48E-11	yellow
PEX11A	-0.06423	-0.18601	-0.09688	0.03392	0.05357	0.06445	0.1049	-0.0963	0.0963	0.40396	0.01485	0.20745	0.65962	0.48647	0.40234	0.1721	0.21022	0.21022	grey
GPS2	-0.04767	-0.11281	-0.09072	-0.10024	0.22141	-0.20706	-0.13366	0.14995	-0.14995	0.5358	0.1418	0.23797	0.19208	0.00361	0.00658	0.08137	0.05028	0.05028	red
TBL3	0.07714	0.07694	-0.03796	-0.08228	0.08656	0.02526	0.17438	-0.37315	0.37315	0.31591	0.31717	0.62207	0.28465	0.26026	0.74292	0.02254	4.99E-07	4.99E-07	green
A4GNT	-0.11112	0.05478	0.0412	0.05882	0.06436	0.20861	-0.00132	-0.0178	0.0178	0.14794	0.47667	0.59263	0.44477	0.40299	0.00618	0.98631	0.81728	0.81728	grey
CDC42BP(	0.05738	-0.1205	-0.05703	-0.06079	0.14016	-0.02019	0.08814	-0.37681	0.37681	0.45597	0.11642	0.45878	0.42963	0.06748	0.79326	0.25167	3.78E-07	3.78E-07	grey
CDH3	0.01585	0.03294	0.20257	0.0805	-0.15716	0.26997	0.22027	-0.37768	0.37768	0.83701	0.66887	0.00788	0.29524	0.04009	0.00036	0.00379	3.53E-07	3.53E-07	yellow
PDF	0.02118	0.11099	-0.08277	-0.04157	0.12045	0.00431	0.23474	-0.34337	0.34337	0.78335	0.1484	0.28181	0.58931	0.11659	0.95538	0.002	4.27E-06	4.27E-06	green
SEMA4D	-0.05861	-0.11089	-0.07665	-0.01659	-0.0271	0.02371	-0.11318	0.23991	-0.23991	0.44638	0.14879	0.31907	0.8295	0.72493	0.75826	0.14051	0.00157	0.00157	blue
DTYMK	0.0224	0.11446	-0.04347	-0.01638	0.02796	0.00662	0.28453	-0.43926	0.43926	0.7712	0.13605	0.57237	0.83162	0.7166	0.9315	0.00016	1.85E-09	1.85E-09	pink
CDC16	0.04857	-0.0295	-0.08946	-0.07677	0.02516	-0.10278	0.01877	-0.08423	0.08423	0.52812	0.70173	0.24457	0.31831	0.74392	0.181	0.80748	0.27337	0.27337	red
RAMAC	-0.07912	-0.12206	-0.09477	-0.07087	0.02057	-0.01217	-0.03841	-0.07798	0.07798	0.30363	0.11175	0.2176	0.35698	0.78947	0.87452	0.61798	0.31067	0.31067	turquoise
FCHSD1	0.05814	-0.14457	0.01442	-0.04086	0.0207	-0.01372	0.00048	-0.14594	0.14594	0.45004	0.05922	0.85154	0.59571	0.78809	0.85865	0.99502	0.05682	0.05682	red
BIRC3	-0.12218	-0.07956	0.04506	0.12555	-0.19998	0.23428	0.17197	-0.09437	0.09437	0.11139	0.30095	0.55839	0.1018	0.00873	0.00204	0.02451	0.21953	0.21953	turquoise
TRIB3	0.20058	-0.02479	-0.15348	-0.05344	-0.00355	-0.20673	0.01589	-0.2244	0.2244	0.00853	0.74761	0.04505	0.48758	0.96322	0.00667	0.83659	0.00317	0.00317	grey
PAX8	0.12439	0.02597	0.05692	-0.00427	-0.05928	-0.02793	0.11847	-0.07236	0.07236	0.10501	0.73597	0.4596	0.95583	0.44117	0.7169	0.12276	0.34694	0.34694	grey
HIRIP3	0.03096	-0.05593	-0.20498	-0.09695	0.07706	-0.19494	-0.08637	0.17203	-0.17203	0.68774	0.46746	0.00716	0.20715	0.31647	0.01062	0.26134	0.02446	0.02446	turquoise
CCS	0.00932	-0.02877	-0.0334	0.02845	0.09286	-0.07021	0.115	-0.21157	0.21157	0.90374	0.70876	0.66448	0.71186	0.22705	0.36147	0.13421	0.00547	0.00547	red
URM1	0.05294	0.00452	-0.08039	-0.0567	-0.08626	-0.05077	0.06255	-0.22878	0.22878	0.49165	0.95318	0.29591	0.46139	0.26192	0.50959	0.41639	0.00261	0.00261	grey
ZNF3	0.05459	-0.12718	-0.08815	-0.06451	0.07665	-0.03857	0.01882	-0.08724	0.08724	0.47822	0.0974	0.25157	0.40187	0.31905	0.61649	0.80702	0.25654	0.25654	turquoise
MTFR1L	-0.05239	-0.17922	-0.0963	-0.0448	-0.01553	-0.03711	-0.14649	0.2867	-0.2867	0.49616	0.019	0.21024	0.5607	0.84021	0.62987	0.05589	0.00014	0.00014	turquoise
PHF5A	-0.04637	0.04642	-0.00663	-0.0022	-0.04419	0.03839	0.13693	-0.15502	0.15502	0.54702	0.54662	0.9314	0.97718	0.56607	0.61812	0.07412	0.04292	0.04292	turquoise
ANKRD24	0.01947	0.01149	-0.16496	-0.06231	0.26354	-0.0253	-0.03504	-0.15862	0.15862	0.80047	0.8814	0.03107	0.41815	0.0005	0.74259	0.64909	0.03825	0.03825	grey
CCNG2	-0.01507	-0.1594	0.05277	0.04752	-0.09989	0.07141	0.09364	-0.10502	0.10502	0.84486	0.0373	0.49305	0.53712	0.19361	0.35332	0.22315	0.17163	0.17163	turquoise
ZDHHC15	-0.05281	-0.16296	-0.14257	-0.01152	-0.05578	-0.13816	-0.21767	0.67816	-0.67816	0.49273	0.03321	0.06286	0.84538	0.46865	0.07153	0.00424	2.23E-24	2.23E-24	brown
DET1	-0.03266	-0.09913	-0.10052	-0.11799	-0.00286	0.03742	0.05496	0.22352	-0.22352	0.67152	0.19708	0.19083	0.12429	0.97036	0.62707	0.47526	0.00329	0.00329	turquoise
COX6B2	0.06134	-0.02936	0.04987	-0.00475	-0.01737	0.07703	0.13367	-0.36554	0.36554	0.42547	0.70306	0.51718	0.95086	0.82164	0.3166	0.08134	8.82E-07	8.82E-07	grey
PGC	-0.1038	0.10481	0.17041	0.04849	-0.06053	0.30603	0.03699	-0.05409	0.05409	0.17666	0.1725	0.02586	0.52886	0.43159	4.69E-05	0.63103	0.48227	0.48227	grey
FAM83E	0.03902	0.09653	0.10604	-0.0072	0.12941	0.24841	0.10983	-0.47772	0.47772	0.61236	0.20914	0.16746	0.92556	0.09161	0.00105	0.15273	3.92E-11	3.92E-11	yellow
LLGL2	0.07594	-0.06035	-0.10742	-0.08762	0.34454	-0.09472	-0.07785	-0.36551	0.36551	0.32358	0.43296	0.16198	0.25446	3.94E-06	0.21781	0.3115	8.85E-07	8.85E-07	grey
KCNS3	-0.04612	-0.05786	-0.13283	0.02891	-0.14756	0.10826	0.00585	0.0076	-0.0076	0.54919	0.45225	0.08328	0.70744	0.0541	0.15871	0.93949	0.92138	0.92138	black
SRSF6	0.01386	-0.06434	-0.09804	-0.01051	0.13672	-0.10834	-0.03569	0.03741	-0.03741	0.85724	0.40311	0.20205	0.89153	0.07457	0.15841	0.64301	0.62716	0.62716	red
BACE1	-0.09521	-0.14791	-0.08324	0.02808	-0.17142	-0.10655	-0.12271	0.61424	-0.61424	0.21545	0.05354	0.27907	0.71548	0.02498	0.16543	0.10984	4.10E-19	4.10E-19	turquoise
CPT1C	-0.06176	-0.17222	-0.18781	-0.08751	0.04576	-0.26051	-0.1876	0.55156	-0.55156	0.42227	0.0243	0.0139	0.25508	0.55227	0.00058	0.01401	5.36E-15	5.36E-15	brown
PPP3CC	-0.12201	-0.03192	-0.0206	-0.00088	-0.1194	-0.02117	-0.06254	0.35475	-0.35475	0.11191	0.67856	0.78917	0.99086	0.11984	0.78344	0.41641	1.93E-06	1.93E-06	blue
DRP2	-0.01044	-0.15632	-0.06326	-0.04387	-0.21961	0.06772	0.06328	0.14961	-0.14961	0.89215	0.04118	0.41107	0.56888	0.0039	0.37881	0.41093	0.05081	0.05081	grey
TGFB1	-0.00555	-0.11329	0.03855	0.09376	-0.15205	0.05211	0.08158	0.0528	-0.0528	0.94257	0.14012	0.61665	0.22256	0.04711	0.49852	0.28882	0.49283	0.49283	black
DLK1	-0.11579	0.07683	-0.16311	-0.0918	-0.00918	-0.08948	-0.06496	0.28635	-0.28635	0.13151	0.3179	0.03304	0.23242	0.90519	0.24447	0.39861	0.00015	0.00015	brown
HDGFL2	0.09773	-0.0338	-0.19249	-0.10463	0.14816	-0.18553	0.04649	-0.20352	0.20352	0.20351	0.66072	0.01166	0.17324	0.05313	0.01512	0.54595	0.00759	0.00759	green
RHOD	0.04973	0.07101	0.04213	0.06068	-0.09802	0.18715	0.27141	-0.41903	0.41903	0.51832	0.35602	0.58425	0.43046	0.20213	0.01424	0.00033	1.17E-08	1.17E-08	yellow
RHPN2	0.0365	-0.02964	0.09222	0.10484	-0.05703	0.25928	0.16227	-0.34936	0.34936	0.63553	0.70037	0.23029	0.17234	0.45875	0.00062	0.03396	2.82E-06	2.82E-06	yellow
CCP110	-0.01339	-0.14472	-0.03897	-0.01716	-0.05513	0.07344	0.05833	0.16168	-0.16168	0.86197	0.05895	0.61285	0.82369	0.47391	0.33979	0.44859	0.03463	0.03463	turquoise
RPL27A	0.16374	0.12856	-0.00788	-0.09209	-0.0161	0.08602	0.17497	-0.35877	0.35877	0.03235	0.09378	0.91856	0.23095	0.83444	0.2633	0.02208	1.45E-06	1.45E-06	purple
ELOVL1	0.01109	-0.04052	0.11667	0.06176	-0.06321	0.19702	0.15917	-0.33385	0.33385	0.88553	0.59876	0.12858	0.42226	0.41146	0.0098	0.03758	8.11E-06	8.11E-06	turquoise
ARSI	-0.08681	-0.01129	0.04108	0.10676	-0.2033	0.01953	0.15118	0.15529	-0.15529	0.25888	0.88347	0.59373	0.1646	0.00766	0.79988	0.04841	0.04255	0.04255	black
FAM102B	-0.09457	-0.12359	0.06844	0.09178	-0.1306	0.2287	0.03947	0.23697	-0.23697	0.21855	0.10729	0.37374	0.2325	0.08864	0.00262	0.60823	0.0018	0.0018	turquoise
DHX38	-0.04426	-0.06056	0.00339	0.01149	-0.05045	0.07417	0.08607	-0.00035	0.00035	0.56543	0.4314	0.96485	0.88141	0.51224	0.33496	0.26302	0.99638	0.99638	turquoise
PRKD2	0.12505	-0.03666	-0.027	-0.00183	-0.01432	0.05668	0.13782	-0.34427	0.34427	0.10317	0.63403	0.72596	0.98106	0.85249	0.46155	0.07225	4.02E-06	4.02E-06	red
NSD1	-0.00608	-0.17336	-0.10831	-0.00825	-0.08262	-0.04909	-0.02197	0.28711	-0.28711	0.93706	0.02336	0.15851	0.91476	0.2827	0.52369	0.77544	0.00014	0.00014	turquoise
GPR39	-0.01077	0.02703	-0.00042	0.06244	-0.08791	0.10469	0.24753	-0.36766	0.36766	0.88885	0.72561	0.9957	0.41722	0.25288	0.17298	0.0011	7.54E-07	7.54E-07	yellow
PLS1	-0.01001	-0.00283	0.05941	0.02252	0.0625	0.21144	0.06571	-0.29266	0.29266	0.89657	0.97067	0.44019	0.77004	0.41673	0.0055	0.39316	0.0001	0.0001	yellow
CSE1L	-0.01281	0.04867	-0.00396	0.00676	-0.11807	-0.02529	0.2183	-0.07632	0.07632	0.86795	0.5273	0.95903	0.93003	0.12404	0.74266	0.00413	0.32114	0.32114	turquoise
UBE2D1	0.01217	-0.12758	0.05739	0.09399	-0.11229	0.01568	0.04386	0.10075	-0.10075	0.87445	0.09633	0.45595	0.22143	0.14368	0.8387	0.56897	0.18979	0.18979	turquoise
TMEM87A	-0.05814	-0.06984	0.03946	0.04249	-0.18146	0.15413	0.15803	-0.14564	0.14564	0.45008	0.36408	0.60833	0.58109	0.01754	0.04414	0.03898	0.05734	0.05734	turquoise
HMGA2	0.09	-0.08132	0.11018	0.06205	-0.08576	0.07696	0.21283	-0.33114	0.33114	0.24172	0.29034	0.1514	0.4201	0.26471	0.31705	0.00519	9.69E-06	9.69E-06	grey
TMEM141	0.06868	0.04885	-0.01598	-0.11303	0.13596	0.00252	0.10161	-0.52229	0.52229	0.37209	0.52573	0.8357	0.14103	0.07621	0.97393	0.18605	2.36E-13	2.36E-13	grey
C16orf54	-0.02719	-0.11155	0.03334	-0.01887	0.00311	-0.11233	-0.08013	0.25793	-0.25793	0.72406	0.14636	0.66513	0.80643	0.96775	0.14352	0.29749	0.00066	0.00066	grey
CRIP2	-0.01362	-0.11061	-0.12868	-0.03464	0.0952	-0.10086	-0.08459	-0.06786	0.06786	0.85962	0.14979	0.09349	0.65286	0.2155	0.18931	0.27134	0.37781	0.37781	grey
ZNF251	0.04661	-0.1031	-0.04446	-0.01617	-0.00865	-0.10839	-0.01024	0.02304	-0.02304	0.54493	0.17962	0.56368	0.83376	0.91062	0.15821	0.8942	0.76483	0.76483	red
CHCHD4	0.04893	0.01935	-0.07238	-0.10778	-0.12782	0.08358	0.15057	-0.18758	0.18758	0.52508	0.80164	0.34684	0.16056	0.09572	0.27711	0.04932	0.01402	0.01402	turquoise
CCDC78	0.07147	0.041	-0.07075	-0.05836	0.22457	-0.20547	-0.03495	-0.19948	0.19948	0.35291	0.59446	0.35783	0.44835	0.00315	0.00702	0.64994	0.0089	0.0089	red
PEX10	-0.13333	-0.06082	-0.01766	0.0032	-0.00279	0.00995	-0.02037	-0.09101	0.09101	0.08211	0.42938	0.81864	0.96682	0.97113	0.89726	0.79143	0.23649	0.23649	grey
NDST2	-0.01997	-0.14933	0.02393	-0.02937	-0.06054	-0.02971	-0.00632	0.34129	-0.34129	0.79545	0.05125	0.75602	0.70296	0.43151	0.69971	0.93462	4.92E-06	4.92E-06	turquoise
RGS16	-0.00098	-0.06192	0.06249	0.05578	-0.1251	0.12684	0.05301	0.22222	-0.22222	0.9898	0.4211	0.41684	0.46869	0.10304	0.0983	0.49109	0.00349	0.00349	black
E2F8	-0.00104	0.11607	0.11575	0.04143	-0.00828	0.15209	0.30205	-0.33561	0.33561	0.98926	0.13059	0.13167	0.59058	0.91437	0.04706	5.95E-05	7.21E-06	7.21E-06	pink
NDUFAB1	0.04592	0.0648	-0.08338	-0.13918	0.03896	-0.09198	0.13074	-0.31486	0.31486	0.55095	0.39975	0.27827	0.06945	0.6129	0.23148	0.08829	2.74E-05	2.74E-05	grey
FRA10AC1	0.04928	-0.0464	-0.0032	0.03153	-0.00763	-0.20232	0.0033	0.09087	-0.09087	0.52208	0.54677	0.9669	0.68222	0.92113	0.00796	0.96587	0.23722	0.23722	grey
ZNF426	-0.10324	-0.1744	-0.14804	-0.01496	-0.13164	-0.05227	0.00035	0.27332	-0.27332	0.17902	0.02253	0.05331	0.84604	0.08612	0.49717	0.9964	0.0003	0.0003	turquoise
E2F2	-0.10461	0.11435	0.1125	-0.02048	-0.02605	0.15693	0.20639	-0.184	0.184	0.17329	0.13642	0.14291	0.79035	0.73519	0.04038	0.00676	0.01599	0.01599	pink
CASK	-0.07297	-0.12643	0.04846	0.10265	-0.06085	0.12858	0.05117	-0.09285	0.09285	0.34291	0.09938	0.52904	0.18154	0.42921	0.09374	0.50623	0.22708	0.22708	turquoise
PI16	-0.0975	-0.03076	-0.03675	0.06729	-0.01228	0.00988	-0.09844	0.45766	-0.45766	0.20458	0.68962	0.63325	0.38185	0.87338	0.89792	0.20023	3.11E-10	3.11E-10	blue
VRK2	-0.12911	-0.03536	0.04014	0.08894	-0.1427	0.08069	0.09729	0.05067	-0.05067	0.09238	0.64615	0.60217	0.24736	0.06261	0.29412	0.20557	0.51042	0.51042	turquoise
TAOK1	-0.07459	-0.17578	-0.05605	0.03886	-0.09261	-0.02495	-0.0537	0.26431	-0.26431	0.33223	0.02146	0.46649	0.6138	0.22832	0.74602	0.48542	0.00048	0.00048	turquoise
ATRNL1	-0.04881	-0.09325	-0.2233	-0.14608	0.07377	-0.30815	-0.18001	0.48536	-0.48536	0.52608	0.22509	0.00333	0.0566	0.33763	4.13E-05	0.01847	1.72E-11	1.72E-11	brown
LRRN4	0.0105	-0.06125	-0.05927	0.16892	-0.08826	0.03976	0.04304	0.0675	-0.0675	0.89157	0.42614	0.44124	0.0272	0.25102	0.60566	0.57622	0.38039	0.38039	magenta
SNAI1	-0.12111	-0.11676	0.03201	0.13101	-0.19391	0.13052	0.00605	0.3889	-0.3889	0.11458	0.12829	0.67768	0.08764	0.01105	0.08885	0.93738	1.47E-07	1.47E-07	blue
TOMM7	0.08436	-0.01401	-0.14254	-0.0417	0.00605	-0.11654	0.06334	-0.003	0.003	0.27263	0.85568	0.06292	0.58812	0.93742	0.12904	0.41047	0.9689	0.9689	purple

HOXA13	0.13733	0.02138	0.16375	0.14522	-0.00437	0.24511	0.2122	-0.24447	0.24447	0.07327	0.78132	0.03235	0.05807	0.95479	0.00123	0.00533	0.00127	0.00127	grey
DOCK6	0.0741	-0.04705	-0.01778	0.00249	0.09216	0.08452	0.09589	-0.25033	0.25033	0.33544	0.54112	0.81747	0.97426	0.23058	0.27175	0.21218	0.00096	0.00096	grey
TMEM132	-0.00742	-0.08162	-0.03203	-0.00862	-0.15221	-0.07047	0.08367	-0.0758	0.0758	0.92323	0.28856	0.67754	0.91086	0.04687	0.35972	0.27656	0.32442	0.32442	grey
PHF10	-0.06806	-0.23377	-0.00917	0.01948	-0.02475	-0.03252	-0.1161	0.36171	-0.36171	0.37644	0.00209	0.90522	0.80039	0.74801	0.67281	0.13048	1.17E-06	1.17E-06	turquoise
KIF11	-0.02282	0.08879	0.06205	0.00177	-0.08821	0.09621	0.33878	-0.3858	0.3858	0.76703	0.24817	0.42009	0.98169	0.25125	0.21064	5.83E-06	1.88E-07	1.88E-07	pink
INTS6	0.02007	-0.1051	-0.02405	-0.01042	-0.11718	0.05419	0.03506	0.15622	-0.15622	0.79442	0.17127	0.75491	0.89237	0.12693	0.48143	0.64892	0.04132	0.04132	turquoise
CCDC28A	0.00733	-0.07265	-0.10086	-0.09463	-0.00235	-0.07567	-0.05206	0.20626	-0.20626	0.92424	0.34499	0.18931	0.21826	0.97565	0.3253	0.49886	0.0068	0.0068	grey
LOXL1	-0.05957	-0.04968	-0.03451	-0.0116	-0.11415	0.0696	0.11545	0.16882	-0.16882	0.43898	0.51873	0.65413	0.88027	0.1371	0.36569	0.13267	0.02729	0.02729	black
EPS8L2	0.15017	-0.05303	-0.00241	-0.0656	0.16929	0.00726	0.11782	-0.54686	0.54686	0.04994	0.49091	0.975	0.39395	0.02687	0.92489	0.12484	1.01E-14	1.01E-14	yellow
SMIM29	-0.00334	-0.00302	-0.15672	-0.0872	0.01973	-0.18169	-0.06923	-0.05506	0.05506	0.96537	0.96872	0.04066	0.25676	0.79782	0.01739	0.36827	0.47444	0.47444	grey
PMM1	0.02302	-0.15346	-0.22443	-0.07147	0.08998	-0.30575	-0.15455	0.23622	-0.23622	0.76503	0.04507	0.00317	0.35294	0.24185	4.77E-05	0.04356	0.00187	0.00187	grey
WDR81	-0.06411	-0.08076	-0.08215	-0.04054	-0.10066	-0.05562	0.02948	0.41099	-0.41099	0.4048	0.2937	0.28544	0.59856	0.1902	0.46999	0.70194	2.35E-08	2.35E-08	blue
ZNF843	0.00962	-0.07951	-0.15447	-0.00844	-0.04668	-0.15276	-0.08752	0.64013	-0.64013	0.90057	0.30127	0.04367	0.91275	0.5443	0.04607	0.255	4.25E-21	4.25E-21	brown
ESYT3	0.10935	0.05186	-0.05639	-0.05411	-0.02066	0.13519	0.11217	-0.23453	0.23453	0.15453	0.50057	0.46383	0.48212	0.78857	0.07791	0.1441	0.00202	0.00202	grey
COL11A1	-0.01573	-0.01624	0.09492	0.11004	-0.21227	0.18662	0.15089	0.04641	-0.04641	0.83815	0.83306	0.21687	0.15192	0.00532	0.01452	0.04884	0.54664	0.54664	black
PRMT1	0.05115	0.02211	-0.08635	-0.1044	0.05275	-0.10895	0.03723	-0.24946	0.24946	0.50647	0.77409	0.26144	0.17417	0.49318	0.15604	0.62877	0.001	0.001	grey
TERF2	0.02532	-0.06697	-0.02163	-0.02997	-0.13333	0.10339	0.08601	0.09505	-0.09505	0.74241	0.38412	0.7789	0.69721	0.08213	0.17839	0.26334	0.21623	0.21623	turquoise
ATP5ME	0.04837	0.02192	-0.18775	-0.12113	0.13304	-0.16601	0.01525	-0.25647	0.25647	0.52983	0.776	0.01393	0.11453	0.0828	0.03	0.84308	0.00071	0.00071	grey
CREB3L1	0.07623	0.0685	0.21187	0.07254	-0.01809	0.36079	0.18536	-0.31448	0.31448	0.32171	0.37333	0.0054	0.34572	0.81429	1.25E-06	0.01522	2.80E-05	2.80E-05	yellow
CCNG1	0.00125	-0.00604	0.10252	0.05978	-0.0784	0.17574	0.07552	0.06758	-0.06758	0.9871	0.93747	0.18208	0.43734	0.30809	0.0215	0.32624	0.3798	0.3798	turquoise
MAP3K1	-0.03775	-0.1172	0.03842	0.13747	-0.14797	0.15902	0.06663	0.0947	-0.0947	0.62395	0.12685	0.61789	0.07297	0.05343	0.03776	0.38659	0.21791	0.21791	turquoise
NR2F2	0.00769	-0.08864	-0.03916	0.06785	-0.11475	0.12284	0.0858	0.28203	-0.28203	0.92045	0.24894	0.61107	0.37788	0.13504	0.10945	0.26452	0.00019	0.00019	blue
PEX5	0.04334	-0.10827	-0.12401	-0.06962	0.14028	-0.20612	-0.1723	0.14748	-0.14748	0.57354	0.15868	0.10609	0.36557	0.06725	0.00684	0.02423	0.05423	0.05423	turquoise
COL4A4	0.00798	-0.05109	-0.1346	0.02566	-0.07141	0.04292	-0.02529	0.33925	-0.33925	0.91753	0.50696	0.07923	0.73903	0.35333	0.57722	0.7427	5.65E-06	5.65E-06	blue
KLRG1	-0.07751	-0.02957	-0.00604	0.02835	-0.11365	0.01401	-0.09506	0.55642	-0.55642	0.31361	0.70107	0.93749	0.71283	0.13887	0.85568	0.21619	2.76E-15	2.76E-15	blue
S1PR5	-0.01963	-0.15836	0.06478	0.03771	-0.21017	-0.02927	0.05994	0.1206	-0.1206	0.79882	0.03858	0.39994	0.62435	0.0058	0.70394	0.43614	0.11612	0.11612	grey
FSTL1	-0.11339	-0.09238	0.05463	0.08962	-0.22762	0.15594	0.05183	0.32767	-0.32767	0.13976	0.22947	0.47792	0.24376	0.00275	0.04168	0.5008	1.22E-05	1.22E-05	black
DNAJC27	-0.08039	-0.21268	-0.16061	-0.04108	-0.03299	-0.07619	-0.14843	0.47292	-0.47292	0.29594	0.00523	0.03586	0.59369	0.66835	0.32199	0.05269	6.52E-11	6.52E-11	turquoise
ACOT11	-0.0234	-0.1043	0.05142	-0.0058	0.09937	0.10648	0.13862	-0.40972	0.40972	0.76132	0.17459	0.50416	0.93997	0.19597	0.16572	0.07059	2.63E-08	2.63E-08	grey
ZNF134	-0.04075	-0.18494	-0.08312	-0.04715	-0.12238	0.03887	-0.08806	0.23831	-0.23831	0.59669	0.01545	0.27979	0.54031	0.1108	0.61371	0.2521	0.0017	0.0017	turquoise
MPST	-0.01747	0.11359	0.01268	0.04833	0.12908	0.18575	0.22352	-0.51701	0.51701	0.82061	0.13908	0.86921	0.53018	0.09246	0.015	0.0033	4.50E-13	4.50E-13	yellow
OSMR	-0.11875	-0.11902	0.07642	0.11042	-0.25154	0.22746	0.20182	0.04668	-0.04668	0.12188	0.12101	0.32049	0.15051	0.0009	0.00277	0.00812	0.54437	0.54437	turquoise
LTB4R	-0.06498	-0.07585	-0.12158	-0.05567	0.2116	-0.15207	-0.07371	0.01024	-0.01024	0.39844	0.3241	0.11318	0.46954	0.00547	0.04708	0.33803	0.89426	0.89426	red
DUSP14	-0.11801	-0.19876	0.01945	0.12583	-0.13462	-0.02246	0.02457	0.08085	-0.08085	0.12425	0.00916	0.80068	0.10103	0.07919	0.77059	0.74977	0.29319	0.29319	black
PCDHB12	-0.07792	-0.15885	-0.04657	-0.01484	-0.09328	-0.0753	-0.06061	0.32446	-0.32446	0.31107	0.03797	0.54528	0.84724	0.22495	0.32766	0.43101	1.50E-05	1.50E-05	turquoise
TMEM60	-0.00254	-0.24558	-0.06393	-0.01737	-0.00224	-0.12342	0.00649	0.10545	-0.10545	0.97367	0.00121	0.40614	0.82163	0.97681	0.10779	0.93287	0.16985	0.16985	grey
POLL	0.04756	0.01406	-0.06867	-0.06279	0.18634	-0.14005	0.10179	-0.23496	0.23496	0.5368	0.8552	0.37213	0.41458	0.01468	0.06769	0.18523	0.00198	0.00198	red
SPRR1B	0.01023	0.14981	-0.01082	-0.08155	0.10463	-0.01752	0.109	-0.18053	0.18053	0.89435	0.05051	0.88827	0.289	0.17321	0.82007	0.15587	0.01813	0.01813	grey
ASPHD2	0.00151	0.04471	0.04639	-0.089	0.01688	0.10301	0.06859	-0.34326	0.34326	0.98441	0.5615	0.54685	0.24702	0.82657	0.17999	0.37272	4.30E-06	4.30E-06	grey
ZMAT5	-0.1131	-0.08964	-0.12285	0.00368	-0.01368	-0.12572	0.03629	0.01374	-0.01374	0.1408	0.24366	0.10943	0.96187	0.85909	0.10133	0.63745	0.85844	0.85844	grey
MARK4	0.07862	-0.13549	-0.14293	-0.04771	0.02293	-0.11432	0.04129	0.01586	-0.01586	0.3067	0.07723	0.06219	0.53549	0.76597	0.13653	0.59177	0.83688	0.83688	grey
TYRP1	-0.01389	-0.14388	0.01495	-0.0615	0.01248	0.12722	0.00411	-0.03983	0.03983	0.85687	0.06045	0.84608	0.42428	0.87127	0.09727	0.9575	0.60499	0.60499	grey
ZNF438	-0.04413	-0.04723	0.08954	0.0991	-0.18804	0.23422	0.17597	0.02957	-0.02957	0.56655	0.53956	0.24417	0.1972	0.01378	0.00205	0.02132	0.701	0.701	turquoise
DKK3	-0.04281	-0.09784	-0.03572	-0.01955	-0.14373	0.07916	-0.04017	0.2882	-0.2882	0.57827	0.20297	0.64276	0.79962	0.06072	0.30337	0.60191	0.00013	0.00013	blue
NPL	-0.0798	0.08416	0.07686	0.07805	-0.20186	0.15996	0.13918	0.3352	-0.3352	0.29951	0.27378	0.31774	0.31025	0.00811	0.03663	0.06944	7.41E-06	7.41E-06	blue
AGK	-0.0396	-0.0499	0.0042	-0.01547	0.00637	-0.12729	-0.01747	0.01146	-0.01146	0.60706	0.51693	0.95649	0.84081	0.93407	0.09709	0.82055	0.88171	0.88171	turquoise
CCDC86	0.00076	-0.08924	-0.07089	-0.03586	-0.01773	-0.19714	-0.02572	0.05753	-0.05753	0.99214	0.24576	0.35683	0.64145	0.81794	0.00975	0.73847	0.45481	0.45481	grey
LRRC57	-0.04431	-0.16628	-0.03913	-0.02519	-0.1	0.02882	0.07004	-0.10633	0.10633	0.56499	0.02973	0.6114	0.74366	0.19313	0.70823	0.36264	0.16629	0.16629	turquoise
FAM168A	-0.02487	-0.14298	-0.03344	0.05471	-0.22843	0.05999	0.00194	0.41862	-0.41862	0.74682	0.06211	0.6641	0.47725	0.00266	0.43573	0.97988	1.21E-08	1.21E-08	blue
ARRB1	-0.0567	-0.12674	0.07115	0.12132	-0.081	0.17802	0.0951	0.1991	-0.1991	0.46136	0.09856	0.35511	0.11395	0.29227	0.01983	0.21598	0.00904	0.00904	grey
TAF1B	-0.07947	-0.07748	0.05988	0.00157	-0.15607	0.15944	0.09151	0.0321	-0.0321	0.30148	0.31383	0.43656	0.98373	0.04151	0.03726	0.23389	0.67683	0.67683	turquoise
PLEKHH3	0.0061	-0.10674	-0.13267	0.03017	0.16999	-0.05549	0.05002	-0.19448	0.19448	0.9369	0.16465	0.08367	0.69522	0.02623	0.47099	0.51584	0.01081	0.01081	green
NKX3-1	-0.09408	-0.14546	0.08209	0.05201	-0.14477	0.04832	0.1186	-0.07481	0.07481	0.22097	0.05765	0.28578	0.4993	0.05886	0.53024	0.12234	0.33081	0.33081	grey
ZBED6	0.01789	-0.11988	0.00694	0.05034	-0.16248	0.12492	0.10219	0.22057	-0.22057	0.8164	0.11835	0.92823	0.51316	0.03374	0.10353	0.18351	0.00374	0.00374	turquoise
CDC14A	-0.1125	-0.18325	-0.02949	0.06725	-0.13707	0.07672	-0.05853	0.39691	-0.39691	0.14291	0.01644	0.70179	0.38212	0.07383	0.31857	0.44702	7.67E-08	7.67E-08	blue
SGK2	-0.04137	-0.08642	-0.02252	0.00939	0.19355	0.00423	-0.04478	-0.22561	0.22561	0.59114	0.26104	0.77004	0.90297	0.0112	0.95616	0.56086	0.00301	0.00301	grey
MAPK8IP2	0.04478	-0.07869	-0.28664	-0.13571	0.13178	-0.35682	-0.23781	0.32595	-0.32595	0.56089	0.3063	0.00014	0.07676	0.08579	1.66E-06	0.00174	1.36E-05	1.36E-05	brown
FBLIM1	-0.06792	-0.09174	-0.00799	-0.01585	-0.14917	0.21345	0.12567	-0.04895	0.04895	0.37741	0.23273	0.91741	0.83702	0.0515	0.00506	0.10147	0.52488	0.52488	black
NXN	-0.11275	-0.08039	-0.02779	0.04564	-0.15805	-0.05702	0.1252	0.34484	-0.34484	0.14205	0.29594	0.71828	0.55336	0.03896	0.4588	0.10276	3.86E-06	3.86E-06	black
NOTCH3	-0.13359	-0.14277	0.01992	0.11389	-0.17881	0.08636	0.04773	0.19364	-0.19364	0.08153	0.06249	0.79596	0.138	0.01929	0.26138	0.53527	0.01116	0.01116	black
GEMIN8	-0.25918	-0.08525	-0.1743	-0.00715	0.11979	-0.06011	-0.05855	-0.10575	0.10575	0.00062	0.26759	0.0226	0.92604	0.11861	0.4348	0.44682	0.16866	0.16866	grey
SERPINB2	-0.05343	0.09372	0.02593	0.00937	0.06368	0.06242	0.09132	0.11221	-0.11221	0.48761	0.22274	0.73641	0.90316	0.40797	0.41736	0.23488	0.14398	0.14398	grey
COL5A2	-0.0678	-0.04557	0.04062	0.06082	-0.19391	0.16391	0.07932	0.20924	-0.20924	0.37827	0.55397	0.59789	0.42942	0.01105	0.03218	0.3024	0.00602	0.00602	black
ADRM1	0.07061	-0.0013	0.00199	-0.01015	0.0747	-0.05619	0.16064	-0.40575	0.40575	0.35876	0.98657	0.97944	0.89518	0.33151	0.46538	0.03583	3.68E-08	3.68E-08	green
RNF167	0.01253	-0.09173	-0.16242	-0.11944	0.08544	-0.26756	-0.12539	0.23363	-0.23363	0.87079	0.23275	0.0338	0.11969	0.2665	0.0004	0.10225	0.0021	0.0021	grey
NR0B1	-0.00978	-0.10308	-0.27465	-0.14802	0.14478	-0.19479	-0.16873	0.25915	-0.25915	0.89899	0.1797	0.00028	0.05335	0.05884	0.01068	0.02738	0.00062	0.00062	brown
SNRNP27	-0.0556	-0.1852	-0.05706	-0.07501	-0.07369	0.01646	-0.01102	0.08472	-0.08472	0.47009	0.0153	0.45853	0.32951	0.33813	0.83085	0.88625	0.27058	0.27058	turquoise
CPSF6	0.07496	-0.1565	-0.04616	0.01387	0.05748	-0.01061	-0.0454	-0.00695	0.00695	0.32984	0.04094	0.54884	0.85711	0.45521	0.89049	0.55544	0.92811	0.92811	turquoise
ATP13A4	0.03419	-0.00663	-0.01551	0.09524	0.05911	0.16412	-0.02247	0.13062	-0.13062	0.65709	0.93142	0.84039	0.2153	0.44249	0.03195	0.77055	0.08859	0.08859	magenta
PPM1M	-0.08209	-0.03344	0.05326	0.09181	-0.19614	0.12516	0.05121	0.32347	-0.32347	0.28581	0.6641	0.48906	0.23235	0.01014	0.10286	0.50593	1.59E-05	1.59E-05	blue
EIF3E	-0.0162	0.06403	0.07711	0.02605	-0.15568	0.12066	0.07346	-0.02679	0.02679	0.83343	0.40539	0.31615	0.73523	0.04202	0.11593	0.33967	0.72801	0.72801	turquoise
EPM2AIP1	-0.0538	-0.20095	-0.17542	-0.14714	-0.03339	-0.0915	-0.11035	0.3026	-0.3026	0.48461	0.0084	0.02174	0.05479	0.6646	0.23395	0.15075	5.75E-05	5.75E-05	turquoise
FLOT2	-0.05442	-0.10295	-0.00106	0.00081	-0.04325	0.12083	0.04871	-0.2256	0.2256	0.47959	0.18027	0.98898	0.99159	0.57436	0.11542	0.52691	0.00301	0.00301	grey
MED25	0.09989	0.03442	-0.08077	-0.10938	0.21179	-0.13464	0.00339	-0.29991	0.29991	0.19363	0.65492	0.29363	0.15443	0.00542	0.07913	0.96491	6.74E-05	6.74E-05	green
SOS1	-0.05733	-0.14963	-0.0268	0.05554	-0.12925	0.09908	0.0662	0.23288	-0.23288	0.45638	0.05078	0.72792	0.47056	0.09202	0.19731	0.38965	0.00218	0.00218	turquoise
CTC1	-0.07332	-0.18368	-0.10205	-0.07774	0.0497	-0.1196	-0.16283	0.29566	-0.29566	0.34059	0.01618	0.18414	0.31218	0.51858	0.1192	0.03335	8.63E-05	8.63E-05	turquoise
CZIB	-0.0788	-0.07879	0.00101	0.00922	-0.0129	0.02579	0.01957	0.14942	-0.14942	0.30559	0.30568	0.98957	0.90473	0.86705	0.73772	0.79942	0.05111	0.05111	turquoise
CIITA	-0.13504	0.04691	0.00034	0.03995	-0.18829	0.11688	0.097	0.28416	-0.28416	0.07823	0.54234	0.99643	0.60393	0.01366	0.1279	0.2069	0.00017	0.00017	blue
SCN1B	-0.05142	-0.03331	-0.1999	-0.13843	-0.00712	-0.07788	-0.03817	0.37948	-0.37948	0.50423	0.66538	0.00876	0.07097	0.92638	0.31128	0.6201	3.08E-07	3.08E-07	grey
HNRNPLL	-0.01679	-0.1265	0.03456	0.01354	-0.13464	0.11099	0.09903	0.11996	-0.11996	0.82748	0.0992	0.65363	0.86043	0.07914	0.14841	0.19754	0.11809	0.11809	turquoise
SPATA6L	0.08772	-0.03044	-0.0534	-0.04368	0.1825	0.07349	0.01535	-0.30304	0.30304	0.25393	0.69265	0.48785	0.57051	0.01689	0.33946	0.84208	5.61E-05	5.61E-05	grey
ANO7	0.06443	0.06809	0.12321	0.03432	0.07741	0.2053	0.04305	-0.30373	0.30373	0.40246	0.37619	0.10838	0.65591	0.31426	0.00707	0.5761	5.38E-05	5.38E-05	grey
AC007906	-0.03402	0.00273	0.10846	0.17236	-0.05907	0.27554	0.18493	-0.1441	0.1441	0.65867	0.97176	0.15793	0.02418	0.4428	0.00026	0.01546	0.06007	0.06007	grey
FMC1	-0.01382	-0.01662	-0.23401	-0.14675	0.23248	-0.33825	-0.18098	0.13188	-0.13188	0.85764	0.82918	0.00207	0.05546	0.00222	6.04E-06	0.01784	0.08554	0.08554	brown
EIF2AK3	-0.04607	-0.12733	-0.00094	0.02659	-0.07844	0.09579	-0.09713	0.27114	-0.27114	0.54965	0.097	0.99023	0.7299	0.30785	0.21265	0.20632	0.00033	0.00033	turquoise
NEFL	0.11346	-0.08556	-0.09853	0.0246	-0.00119	-0.01869	-0.17569	0.35182	-0.35182	0.13952	0.26583	0.1998	0.74947	0.98768	0.80826	0.02153	2.37E-06	2.37E-06	grey
COL3A1	-0.06468	-0.06235	0.07433	0.10257	-0.16999	0.17429	0.08418	0.21172	-0.21172	0.40068	0.41787	0.33395	0.18191	0.02623	0.02262	0.27367	0.00544	0.00544	black
DUSP18	-0.05391	-0.06769	-0.06715	-0.0094	0.08288	-0.02457	-0.08178	-0.00142	0.00142	0.48376	0.37901	0.38286	0.90285	0.28117	0.74971	0.28762	0.9853	0.9853	turquoise
DNAH5	0.04684	-0.20318	-0.16115	-0.05121	-0.08096	-0.16492	-0.00663	0.05985	-0.05985	0.54292	0.00769	0.03524	0.50595	0.29249	0.03111	0.93137	0.43678	0.43678	turquoise
ZNF142	0.01596	-0.11506	-0.1093	0.00075	0.0084	-0.05311	-0.043	0.13376	-0.13376	0.83586	0.13397	0.15471	0.99226	0.91317	0.49025	0.57659	0.08114	0.08114	turquoise
VTI1B	0.00601	-0.10041	0.05046	0.08581	-0.05915	0.03068	0.02077	0.03786	-0.03786	0.93777	0.19131	0.5122	0.26444	0.4422	0.69038	0.78747	0.62299	0.62299	turquoise
HSPH1	0.18412	-0.19651	-0.00247	0.01414	0.02914	0.04902	0.0275	-0.09165	0.09165	0.01593	0.00999	0.97442	0.8544	0.7052	0.52428	0.72108	0.2332	0.2332	turquoise
BDKRB2	-0.19787	-0.15635	-0.05862	0.06969	-0.0487	0.12815	-0.03293	0.2285	-0.2285	0.00948	0.04113	0.44633	0.36507	0.52706	0.09485	0.66894	0.00265	0.00265	blue
CXCL17	0.08636	0.19178	0.2011	0.03087	0.00489	0.22263	0.1562	-0.23475	0.23475	0.26136	0.01198	0.00835	0.68853	0.94941	0.00343	0.04133	0.002	0.002	grey
SRP54	-0.03625	-0.11881	-0.01578	0.02743	-0.02233	0.00508	0.00607	0.05449	-0.05449	0.63782	0.12169	0.83766	0.72177	0.77188	0.94739	0.93718	0.47904	0.47904	turquoise
LRRC8A	0.05353	-0.02448	0.0581	-0.00711	-0.15842	0.15005	0.18687	-0.28619	0.28619	0.48681	0.75058	0.45033	0.92646	0.0385	0.05013	0.01439	0.00015	0.00015	yellow
PALM3	0.07607	-0.08417	0.03901	0.19442	0.11804	0.18742	0.17108	-0.2168	0.2168	0.32273	0.27373	0.61245	0.01083	0.12415	0.0141	0.02527	0.0044	0.0044	grey
TGIF2	-0.0275	0.06186	0.01897	0.1243	-0.19563	0.16968	0.26117	-0.11209	0.11209	0.72108	0.42151	0.80547	0.10528	0.01034	0.02651	0.00056	0.14441	0.14441	grey
PTRHD1	0.05421	0.10552	-0.08866	-0.08208	0.16597	-0.05782	0.11105	-0.24022	0.24022	0.48127	0.16956	0.24883	0.28587	0.03005	0.45256	0.14819	0.00155	0.00155	grey
FRAS1	-0.0483	-0.11795	-0.09023	0.057	-0.02616	0.12133	0.06404	0.11063	-0.11063	0.53047	0.12443	0.24053	0.45899	0.73409	0.11391	0.40534	0.14973	0.14973	magenta
ZNF562	-0.01504	-0.13829	-0.09316	-0.07701	-0.01703	0.00983	-0.02636	-0.00237	0.00237	0.84524	0.07127	0.22554	0.31678	0.82505	0.8985	0.7322	0.97546	0.97546	turquoise
SEC11A	0.0018	-0.14828	-0.04982	0.00379	-0.10015	0.01791	0.067	-0.01199	0.01199	0.98137	0.05293	0.51754	0.96075	0.19245	0.81619	0.38392	0.87628	0.87628	turquoise
ARL5B	0.04142	-0.17492	0.08301	0.09003	-0.03146	0.13958	0.04969	0.00619	-0.00619	0.59067	0.02212	0.28039	0.2416	0.6829	0.06864	0.51862	0.93595	0.93595	turquoise
MITF	-0.02096	-0.09977	0.0617	0.13946	-0.14421	0.22429	0.13872	0.17168	-0.17168	0.78551	0.19417	0.42276	0.06887	0.05986	0.00319	0.07039	0.02475	0.02475	blue
NELFA	0.01507	-0.04116	-0.07558	-0.0024	0.17286	-0.03252	0.01313	-0.33015	0.33015	0.84485	0.59303	0.32588	0.9751	0.02377	0.67281	0.86466	1.03E-05	1.03E-05	green
PPP1R35	0.08328	-0.0227	-0.03312	-0.06188	0.20313	-0.05034	0.0965	-0.51031	0.51031	0.27884	0.76818	0.66714	0.42135	0.00771	0.51319	0.20925	1.00E-12	1.00E-12	green
SRPK2	-0.00057	-0.1828	-0.0597	-0.01294	-0.04799	-0.05018	-0.06474	0.27414	-0.27414	0.99411	0.01671	0.43793	0.86664	0.53304	0.51453	0.40018	0.00029	0.00029	turquoise
TBC1D10E	0.04498	-0.05718	-0.10248	-0.03282	-0.00628	-0.04762	0.10504	-0.19306	0.19306	0.55907	0.4576	0.18228	0.67004	0.93504	0.53628	0.17152	0.01141	0.01141	green
DPH2	-0.06082	-0.02167	0.00021	-0.05598	-0.07868	0.07286	0.07912	-0.08577	0.08577	0.42942	0.77848	0.99784	0.46705	0.30634	0.34363	0.30365	0.2647	0.2647	turquoise
C1orf53	0.10462	0.08088	-0.03005	-0.00711	0.23359	-0.07045	0.10162	-0.46523	0.46523	0.17325	0.29298	0.69643	0.92647	0.00211	0.35988	0.18598	1.45E-10	1.45E-10	grey
PNPO	0.03395	-0.04128	-0.13134	-0.03907	0.09442	-0.0788	-0.06487	-0.01764	0.01764	0.65934	0.59193	0.08683	0.61193	0.21928	0.30559	0.39925	0.81884	0.81884	turquoise
PSMD14	-0.08504	-0.00851	-0.00377	0.04376	-0.0662	-0.0272	0.11155	-0.16698	0.16698	0.26879	0.91199	0.96101	0.56984	0.38965	0.72402	0.14634	0.02905	0.02905	turquoise
PSMC4	0.04088	-0.14029	0.01655	0.07642	-0.04351	-0.00916	0.09874	-0.2069	0.2069	0.59554	0.06723	0.82991	0.32049	0.572	0.90532	0.19885	0.00662	0.00662	grey
ZFAND3	0.03805	-0.13163	-0.03387	0.02692	-0.00112	-0.0232	-0.05477	0.22516	-0.22516	0.62124	0.08613	0.66006	0.72676	0.98837	0.76329	0.47679	0.00307	0.00307	turquoise
JARID2	-0.05877	-0.07786	-0.04171	-0.02858	-0.11466	0.00404	0.02026	0.21471	-0.21471	0.44513	0.31145	0.58809	0.71056	0.13537	0.95814	0.7925	0.0048	0.0048	turquoise
SERGEF	0.08732	-0.0484	-0.27744	-0.21206	0.17814	-0.30276	-0.1899	-0.03288	0.03288	0.25613	0.52962	0.00024	0.00536	0.01975	5.70E-05	0.01286	0.6694	0.6694	grey
CABYR	0.07087	-0.17832	-0.06541	0.10051	-0.11048	0.01997	-0.01607	0.10857	-0.10857	0.35697	0.01963	0.39533	0.19088	0.15028	0.79548	0.83477	0.15752	0.15752	grey
WDR60	-0.02402	-0.22551	-0.03568	0.04765	0.11944	-0.10305	-0.02449	0.03089	-0.03089	0.75514	0.00302	0.64317	0.536	0.11971	0.17983	0.75056	0.68834	0.68834	turquoise
TNFSF14	-0.03589	-0.12525	-0.08025	0.03125	-0.0539	0.04801	-0.02451	0.22426	-0.22426	0.64123	0.10262	0.29677	0.68488	0.48381	0.53289	0.75036	0.00319	0.00319	blue
LYPLAL1	-0.10505	0.00376	-0.10169	-0.01575	-0.0066	-0.04916	0.06182	0.03976	-0.03976	0.1715	0.96108	0.18568	0.83798	0.9317	0.52317	0.42187	0.6056	0.6056	grey
AASS	-0.05036	-0.12829	0.04027	0.05109	-0.16163	0.15908	0.014	0.33478	-0.33478	0.51301	0.09448	0.60101	0.50689	0.03469	0.03769	0.8558	7.62E-06	7.62E-06	blue
ELF3	0.09433	0.03189	0.06299	0.04227	0.09247	0.24685	0.09551	-0.47575	0.47575	0.21976	0.6788	0.4131	0.58306	0.22902	0.00113	0.21401	4.84E-11	4.84E-11	yellow
CPEB3	0.02549	-0.08993	-0.15599	-0.07906	0.04962	-0.15497	-0.12831	0.31439	-0.31439	0.74066	0.24209	0.04162	0.30399	0.51927	0.04298	0.09442	2.82E-05	2.82E-05	turquoise
ADAMTS1	-0.03721	-0.06577	0.06499	0.08475	-0.26874	0.17082	0.13071	0.13891	-0.13891	0.62901	0.39272	0.39839	0.27044	0.00038	0.02549	0.08836	0.06999	0.06999	black
COMMD1	0.00703	-0.139	-0.04548	0.01868	0.03692	-0.10313	-0.17246	0.27583	-0.27583	0.92729	0.06981	0.55477	0.80842	0.63161	0.17949	0.02409	0.00026	0.00026	turquoise
TMC8	-0.0921	-0.01535	0.07466	0.03117	-0.08843	0.12529	0.01767	0.23507	-0.23507	0.23087	0.84202	0.3318	0.68567	0.25009	0.10252	0.81856	0.00197	0.00197	blue
RHPN1	0.03404	-0.05389	-0.10364	-0.07483	0.24003	-0.19798	0.00963	-0.36639	0.36639	0.65847	0.48391	0.17736	0.33068	0.00157	0.00944	0.9005	8.29E-07	8.29E-07	green
JOSD1	-0.02691	-0.0873	0.04363	0.09347	-0.13385	0.21168	0.11925	-0.05541	0.05541	0.72683	0.25621	0.57102	0.224	0.08093	0.00545	0.1203	0.4716	0.4716	turquoise
ITIH3	-0.15047	-0.02338	-0.02255	-0.00136	-0.0238	0.17993	-0.09871	0.30259	-0.30259	0.04947	0.76145	0.76973	0.98593	0.75731	0.01853	0.19896	5.76E-05	5.76E-05	grey

FAM193B	0.02114	-0.11652	-0.08238	-0.04564	0.23625	-0.12327	-0.02937	-0.15626	0.15626	0.7837	0.1291	0.28409	0.55333	0.00187	0.1082	0.703	0.04126	0.04126	red
ANKRD28	-0.0978	-0.13255	0.0469	0.02667	-0.19382	0.14748	0.13312	0.11429	-0.11429	0.20318	0.08395	0.54239	0.72911	0.01108	0.05423	0.08261	0.13664	0.13664	turquoise
MRPL46	0.00211	0.01582	-0.17706	-0.09646	0.18689	-0.1507	0.0684	-0.18961	0.18961	0.97815	0.83733	0.02052	0.20946	0.01438	0.04913	0.37402	0.013	0.013	grey
CEACAM6	0.05737	0.04043	0.13379	0.06461	-0.02762	0.2657	0.11522	-0.21288	0.21288	0.45611	0.59957	0.08107	0.40112	0.71991	0.00044	0.13344	0.00518	0.00518	yellow
HEXIM1	0.01134	-0.2187	-0.05345	0.05884	0.07923	0.03464	0.01244	-0.13039	0.13039	0.88293	0.00406	0.48749	0.4446	0.30297	0.65285	0.87169	0.08917	0.08917	turquoise
SPCS3	-0.09177	-0.00511	0.05564	0.06649	-0.06459	0.04212	-0.09278	0.13761	-0.13761	0.23254	0.94711	0.46976	0.38754	0.40132	0.58434	0.22744	0.07267	0.07267	turquoise
ISG15	-0.01188	0.07145	0.04942	0.1049	-0.08835	0.09057	0.19148	-0.25105	0.25105	0.87745	0.35305	0.52097	0.17209	0.25053	0.23876	0.01211	0.00093	0.00093	tan
TMF1	-0.05202	-0.18785	-0.00903	-0.01335	-0.08132	0.10169	-0.002	0.13824	-0.13824	0.49923	0.01388	0.90667	0.86244	0.29036	0.18567	0.97931	0.07136	0.07136	turquoise
RORA	-0.08552	-0.17264	-0.10516	0.01474	-0.17351	0.00234	-0.11067	0.4644	-0.4644	0.26606	0.02394	0.17103	0.84823	0.02323	0.97578	0.14957	1.57E-10	1.57E-10	blue
PCDHB3	-0.0101	-0.14202	-0.0409	0.07261	-0.08635	-0.12036	-0.06035	0.42344	-0.42344	0.89572	0.06389	0.59529	0.34526	0.26146	0.11688	0.43296	7.91E-09	7.91E-09	grey
TBP	-0.07988	-0.07987	-0.03761	-0.0149	-0.0152	-0.03265	-0.01213	0.19249	-0.19249	0.29901	0.29904	0.62524	0.84661	0.84354	0.67161	0.87486	0.01166	0.01166	turquoise
CLCN2	-0.00408	-0.00038	-0.14848	-0.07066	0.11805	-0.03062	0.10382	-0.23838	0.23838	0.95772	0.99605	0.05261	0.35842	0.12412	0.69094	0.17659	0.00169	0.00169	grey
EXO1	-0.03002	0.1286	0.01183	-0.02258	-0.04021	0.08419	0.3493	-0.36849	0.36849	0.69671	0.09367	0.87793	0.76942	0.6016	0.2736	2.83E-06	7.09E-07	7.09E-07	pink
PITPNA	-0.03354	-0.17954	-0.08731	-0.09157	0.01189	-0.17766	-0.11438	0.35694	-0.35694	0.66316	0.01879	0.25614	0.23358	0.87734	0.02009	0.13633	1.65E-06	1.65E-06	turquoise
MPHOSPH	-0.0711	-0.08782	-0.02284	-0.0047	-0.09787	0.05637	0.06665	0.02602	-0.02602	0.35545	0.2534	0.76682	0.95136	0.20282	0.46395	0.38639	0.73545	0.73545	turquoise
CACNA2D	-0.03049	-0.02101	-0.25724	-0.14956	0.06399	-0.19099	-0.15989	0.40481	-0.40481	0.69219	0.78499	0.00068	0.05089	0.40568	0.01234	0.03671	3.98E-08	3.98E-08	brown
GTF2IRD2	-0.0006	-0.10121	-0.08012	-0.02642	0.13102	-0.05393	-0.17141	0.09918	-0.09918	0.99382	0.18777	0.29756	0.73161	0.08762	0.4836	0.02499	0.19684	0.19684	grey
MAPK12	-0.10266	-0.05105	-0.18259	-0.04292	0.12664	-0.37165	-0.15608	0.32241	-0.32241	0.18152	0.50723	0.01683	0.57723	0.09882	5.59E-07	0.04149	1.70E-05	1.70E-05	brown
CHTF8	-0.00538	-0.1034	-0.04588	-0.04804	-0.1275	0.14123	0.12813	0.06769	-0.06769	0.94428	0.17835	0.55124	0.53263	0.09655	0.06541	0.0949	0.37905	0.37905	turquoise
IDH3A	-0.0831	-0.15849	-0.03443	0.01388	-0.06927	-0.04159	-0.05934	0.27117	-0.27117	0.2799	0.03841	0.65481	0.857	0.36795	0.58913	0.4407	0.00033	0.00033	turquoise
PITRM1	0.05271	-0.16998	-0.0253	-0.11195	-0.01253	-0.09746	-0.00802	0.0132	-0.0132	0.49357	0.02624	0.74255	0.14491	0.87079	0.20477	0.91707	0.86397	0.86397	turquoise
THEMIS2	-0.10367	-0.01658	0.10919	0.05557	-0.2282	0.1598	0.05027	0.28573	-0.28573	0.17723	0.82954	0.15514	0.47038	0.00268	0.03682	0.51376	0.00015	0.00015	blue
SPOCK1	-0.0282	-0.03676	-0.03265	0.0618	-0.22745	0.10125	0.05455	0.22545	-0.22545	0.71431	0.63313	0.67161	0.42199	0.00277	0.1876	0.47856	0.00303	0.00303	black
FAM25A	0.06926	0.10444	0.04921	-0.0141	0.10728	-0.0557	0.14619	-0.34187	0.34187	0.36805	0.174	0.52275	0.85481	0.16255	0.46932	0.05639	4.73E-06	4.73E-06	grey
RALGAPA	0.00861	-0.15708	-0.05495	0.02055	0.03572	-0.06296	-0.13984	0.29277	-0.29277	0.91098	0.04019	0.47534	0.78965	0.64274	0.41333	0.06812	0.0001	0.0001	turquoise
RECQL4	-0.01366	0.09072	-0.05707	-0.06182	0.10191	-0.14284	0.20922	-0.46723	0.46723	0.85928	0.23798	0.45846	0.42183	0.18475	0.06236	0.00603	1.18E-10	1.18E-10	green
POLR2B	-0.12202	-0.12017	-0.0211	0.01656	-0.0337	0.00762	0.03496	0.20232	-0.20232	0.11185	0.11746	0.78415	0.82978	0.66166	0.92118	0.64988	0.00796	0.00796	turquoise
PTPA	0.07806	-0.13128	-0.13517	-0.03208	-0.08033	-0.11442	0.03663	-0.11397	0.11397	0.31016	0.08697	0.07794	0.677	0.2963	0.13619	0.63432	0.13774	0.13774	grey
ALPK3	0.01922	-0.20308	-0.15522	-0.01694	0.01778	-0.18117	-0.09901	0.33287	-0.33287	0.80298	0.00772	0.04265	0.82597	0.81741	0.01772	0.19761	8.65E-06	8.65E-06	brown
MYL6B	0.03363	-0.1484	-0.19635	-0.08695	0.04664	-0.32111	-0.05611	0.17126	-0.17126	0.66239	0.05273	0.01006	0.25813	0.54468	1.85E-05	0.46604	0.02511	0.02511	brown
MYO19	-0.03564	-0.04334	0.00117	0.00709	0.16063	-0.01458	0.16067	-0.38002	0.38002	0.64348	0.5735	0.98793	0.92664	0.03584	0.8499	0.03579	2.95E-07	2.95E-07	pink
ZNF761	0.02684	-0.17471	-0.07041	-0.02041	0.06937	-0.01237	-0.09475	0.04411	-0.04411	0.7275	0.02228	0.36014	0.791	0.36728	0.87239	0.21769	0.56675	0.56675	turquoise
ZNF253	-0.04444	-0.07141	-0.1318	0.02252	0.05907	-0.09773	-0.21369	0.34764	-0.34764	0.5638	0.35337	0.08573	0.77005	0.44285	0.2035	0.00501	3.18E-06	3.18E-06	turquoise
SMG1	-0.00132	-0.12452	0.03995	0.06167	-0.12207	0.18353	0.09787	0.07564	-0.07564	0.98636	0.10465	0.60392	0.42298	0.11171	0.01627	0.20285	0.32549	0.32549	turquoise
KCNK7	-0.01022	-0.04512	0.04809	-0.04338	0.19211	-0.05433	-0.03027	-0.32402	0.32402	0.89446	0.55787	0.53227	0.57319	0.01183	0.48029	0.69431	1.54E-05	1.54E-05	grey
PIFO	0.0095	-0.17803	-0.18103	0.00171	0.04411	-0.12935	-0.12215	0.19562	-0.19562	0.90185	0.01982	0.01781	0.98229	0.56674	0.09176	0.11149	0.01034	0.01034	grey
ELP5	-0.0747	-0.16147	-0.16984	-0.13284	0.10224	-0.23126	-0.08489	0.26342	-0.26342	0.33157	0.03487	0.02637	0.08326	0.1833	0.00234	0.26963	0.0005	0.0005	grey
KDM2B	-0.07026	-0.12238	-0.00965	0.04716	-0.05981	0.024	-0.02028	0.3121	-0.3121	0.36115	0.1108	0.90028	0.5402	0.43715	0.75535	0.79233	3.24E-05	3.24E-05	turquoise
ADI1	0.0551	0.05107	0.06159	0.02433	-0.0485	0.20492	0.11609	-0.10681	0.10681	0.47412	0.50714	0.42357	0.75207	0.52873	0.00718	0.13052	0.16437	0.16437	turquoise
MPV17L2	0.06139	0.07254	-0.11776	-0.09378	0.08387	-0.16425	-0.08298	-0.07163	0.07163	0.42507	0.34576	0.12503	0.22247	0.27541	0.03182	0.28059	0.35184	0.35184	grey
KCNA3	-0.13511	-0.03911	-0.02561	-0.03313	-0.15315	0.03819	-0.05604	0.54984	-0.54984	0.07808	0.61158	0.73948	0.66708	0.04552	0.61993	0.46664	6.76E-15	6.76E-15	blue
EPHB2	-0.04372	-0.07061	0.05981	0.16167	-0.21494	0.14108	0.21897	0.04905	-0.04905	0.57017	0.35874	0.43711	0.03464	0.00476	0.0657	0.00401	0.52407	0.52407	grey
RPL5	-0.0082	0.00074	-0.04164	-0.03719	-0.10772	0.01686	0.00073	0.17632	-0.17632	0.91522	0.99232	0.58863	0.62911	0.1608	0.82674	0.99248	0.02106	0.02106	purple
SMOC2	-0.11507	-0.05438	-0.03002	-0.01471	-0.0783	0.14992	-0.02692	0.40979	-0.40979	0.13397	0.47992	0.69675	0.84852	0.30869	0.05033	0.72671	2.61E-08	2.61E-08	blue
IL13RA1	-0.02591	-0.21017	0.01491	0.04256	-0.14363	0.12396	0.05546	0.02202	-0.02202	0.73658	0.0058	0.84648	0.58045	0.06091	0.10623	0.4712	0.77497	0.77497	turquoise
ARVCF	-0.07777	-0.10979	-0.24145	-0.10528	0.22205	-0.31088	-0.18754	0.17121	-0.17121	0.31197	0.15287	0.00147	0.17055	0.00351	3.50E-05	0.01404	0.02516	0.02516	brown
SIPA1L3	-0.0033	-0.20282	-0.14057	-0.04366	0.08889	-0.07239	-0.07131	-0.00418	0.00418	0.96584	0.0078	0.06669	0.57074	0.24761	0.34672	0.35403	0.95668	0.95668	turquoise
B4GALT2	0.02285	0.0255	0.03593	0.03639	-0.05408	0.02055	0.11209	-0.15994	0.15994	0.76671	0.74059	0.6408	0.63654	0.48235	0.78967	0.1444	0.03666	0.03666	grey
AP1S1	0.13991	0.00524	-0.06807	-0.04688	0.117	-0.10104	0.1311	-0.44606	0.44606	0.06797	0.94572	0.37637	0.54265	0.12751	0.18851	0.08743	9.70E-10	9.70E-10	grey
MED17	-0.05517	-0.15445	-0.05968	-0.03067	-0.10214	0.08448	0.05065	0.10593	-0.10593	0.47353	0.0437	0.43815	0.6905	0.18372	0.27198	0.51059	0.16789	0.16789	turquoise
EEF1B2	0.05822	0.14466	0.06743	-0.01162	-0.15507	0.21743	0.14916	-0.16009	0.16009	0.44941	0.05905	0.38088	0.88009	0.04285	0.00428	0.05152	0.03647	0.03647	purple
SMIM3	-0.09322	-0.1273	0.09207	0.10751	-0.1966	0.1979	0.0927	0.13684	-0.13684	0.22522	0.09708	0.23103	0.16164	0.00996	0.00947	0.22783	0.07432	0.07432	black
WDSUB1	-0.02939	-0.02057	-0.08972	0.02664	-0.03384	-0.00799	-0.02151	0.1295	-0.1295	0.7028	0.78947	0.24319	0.72949	0.66035	0.91742	0.78003	0.09138	0.09138	turquoise
RPL18	0.13391	0.18952	-0.02021	-0.07607	0.0519	0.00957	0.05485	-0.37008	0.37008	0.0808	0.01304	0.79308	0.32269	0.50019	0.90112	0.47612	6.30E-07	6.30E-07	purple
UNC119	0.01023	-0.17244	-0.13674	-0.10338	0.15518	-0.23345	-0.13348	0.08295	-0.08295	0.89437	0.02412	0.07453	0.17845	0.04269	0.00212	0.08178	0.28074	0.28074	grey
HDAC8	-0.03801	-0.21328	-0.15617	-0.09985	-0.00523	-0.08951	0.04757	-0.14215	0.14215	0.62165	0.0051	0.04137	0.19383	0.94588	0.24435	0.53668	0.06365	0.06365	turquoise
VAV1	-0.00011	-0.05395	0.04387	0.04681	-0.19924	0.11624	0.14539	0.08691	-0.08691	0.99887	0.48338	0.56886	0.54322	0.00899	0.13003	0.05779	0.25834	0.25834	blue
TM7SF2	-0.00552	-0.01595	-0.11852	-0.09107	0.18719	-0.14016	-0.08712	-0.28618	0.28618	0.94283	0.83599	0.12262	0.23618	0.01422	0.06748	0.2572	0.00015	0.00015	grey
PCK2	0.07127	-0.02357	0.05897	0.07192	0.14904	-0.02297	-0.04707	-0.20249	0.20249	0.35431	0.75958	0.4436	0.34988	0.05171	0.76552	0.54095	0.00791	0.00791	grey
MAT2A	-0.04217	-0.08032	0.0137	0.01796	0.02919	0.15279	-0.0293	-0.01128	0.01128	0.58393	0.29635	0.85881	0.81568	0.70472	0.04603	0.70365	0.88359	0.88359	red
ADAP1	0.0717	0.12429	0.04815	-0.02088	0.13673	0.14169	0.1845	-0.52954	0.52954	0.35135	0.10531	0.53172	0.78631	0.07455	0.06451	0.01571	9.57E-14	9.57E-14	yellow
LRRC28	-0.07391	-0.12012	-0.06585	0.03277	-0.01267	-0.00755	-0.00374	0.14874	-0.14874	0.3367	0.11761	0.39214	0.67049	0.86931	0.92191	0.96123	0.05218	0.05218	turquoise
EFNA3	0.03933	0.05492	0.05752	-0.01902	0.09523	0.03429	0.14066	-0.46334	0.46334	0.60954	0.47558	0.4549	0.80496	0.21536	0.65619	0.06651	1.75E-10	1.75E-10	grey
SLC7A4	0.06093	0.05642	-0.02235	-0.01348	0.27595	-0.13525	-0.09885	-0.21234	0.21234	0.42857	0.46356	0.77174	0.86105	0.00026	0.07777	0.19836	0.0053	0.0053	grey
TOB2	-0.03809	-0.10739	0.00199	0.09017	-0.07745	0.19676	0.03692	0.00489	-0.00489	0.62088	0.16209	0.97941	0.24084	0.31399	0.0099	0.63165	0.94939	0.94939	turquoise
GABPB1	-0.08381	-0.13186	-0.12071	-0.0726	-0.11155	-0.10605	-0.06536	0.16981	-0.16981	0.27579	0.08559	0.11578	0.34534	0.14635	0.16742	0.39567	0.02639	0.02639	turquoise
CLCN6	0.00973	-0.14502	-0.06804	-0.00794	-0.03164	-0.00726	-0.07429	0.2788	-0.2788	0.89945	0.05843	0.37656	0.91792	0.68125	0.92494	0.3342	0.00022	0.00022	turquoise
NAPSA	-0.00075	-0.13837	-0.0455	0.07335	0.15432	-0.06828	-0.09369	0.08235	-0.08235	0.99224	0.07111	0.5546	0.34035	0.04387	0.37486	0.22292	0.28428	0.28428	grey
EXPH5	0.07787	-0.06047	0.03997	0.03706	-0.0538	0.16816	0.1835	-0.31339	0.31339	0.31136	0.43208	0.60377	0.63038	0.48461	0.02791	0.01629	3.00E-05	3.00E-05	turquoise
TP53I13	-0.01086	-0.12833	-0.14599	-0.05341	0.27082	-0.29792	-0.14776	-0.02806	0.02806	0.88786	0.09439	0.05674	0.48784	0.00034	7.57E-05	0.05377	0.71563	0.71563	green
SAMD3	0.0021	-0.05591	0.02211	0.04684	-0.08184	-0.09885	-0.10551	0.51049	-0.51049	0.97824	0.46764	0.7741	0.54293	0.28727	0.19834	0.16962	9.83E-13	9.83E-13	blue
RIN2	-0.02607	-0.12272	-0.09713	-0.00032	-0.0535	-0.14962	0.0456	0.28695	-0.28695	0.73503	0.1098	0.20631	0.9967	0.48711	0.05079	0.55368	0.00014	0.00014	turquoise
VAPB	0.02037	-0.11604	-0.10439	-0.03952	0.01079	-0.20145	-0.07368	0.17789	-0.17789	0.79144	0.13069	0.17421	0.60777	0.88863	0.00824	0.33817	0.01993	0.01993	turquoise
ABCD1	-0.12725	-0.20252	-0.00426	0.03665	-0.0754	0.06409	0.01842	-0.06344	0.06344	0.09721	0.0079	0.95592	0.63412	0.327	0.40495	0.81102	0.40975	0.40975	grey
ATL3	-0.00802	-0.03274	0.04916	0.09242	-0.19939	0.1971	0.14142	0.04491	-0.04491	0.91709	0.67079	0.52316	0.22926	0.00893	0.00977	0.06504	0.55974	0.55974	turquoise
VEGFB	0.02444	-0.12166	-0.09549	-0.01839	-0.04384	-0.08022	-0.06157	0.21773	-0.21773	0.75101	0.11293	0.21411	0.8113	0.56911	0.29696	0.42374	0.00423	0.00423	grey
PKIA	-0.04592	-0.02096	-0.0304	0.00583	-0.11369	-0.09541	-0.11787	0.30697	-0.30697	0.55091	0.78555	0.69305	0.93971	0.13871	0.21448	0.12469	4.43E-05	4.43E-05	grey
RUVBL1	-0.00072	0.06943	-0.13147	-0.10358	-0.07224	0.01024	0.22344	-0.25235	0.25235	0.99259	0.36687	0.08653	0.17761	0.34774	0.89427	0.00331	0.00087	0.00087	turquoise
HMGXB4	-0.07369	-0.04946	-0.0329	0.02338	-0.1343	0.09029	0.07464	0.03536	-0.03536	0.33812	0.52061	0.66924	0.76151	0.0799	0.24021	0.3319	0.64615	0.64615	turquoise
CCDC88A	-0.11457	-0.08937	0.06336	0.07325	-0.21139	0.0989	0.0624	0.30882	-0.30882	0.13567	0.24506	0.41031	0.34102	0.00551	0.1981	0.4175	3.96E-05	3.96E-05	blue
TAP2	-0.08523	0.13201	0.01363	0.04977	-0.15212	0.0171	0.20945	-0.14374	0.14374	0.2677	0.08522	0.85954	0.51799	0.04701	0.82434	0.00597	0.06071	0.06071	tan
TMEM268	-0.01635	-0.06343	-0.03267	0.02719	-0.11263	-0.03242	-0.03097	0.41313	-0.41313	0.83194	0.40979	0.6714	0.7241	0.14247	0.67382	0.6876	1.96E-08	1.96E-08	turquoise
SPOP	-0.03325	-0.11274	-0.09477	-0.00254	0.00166	-0.07855	-0.12759	0.37498	-0.37498	0.66595	0.14208	0.21757	0.97369	0.98276	0.30717	0.09631	4.35E-07	4.35E-07	turquoise
CSNK2A2	0.10292	-0.09031	-0.01296	-0.03643	-0.12128	0.11282	0.11455	-0.11512	0.11512	0.18037	0.2401	0.86642	0.63621	0.11406	0.14178	0.13575	0.13379	0.13379	turquoise
JAK1	-0.08356	-0.14886	0.06143	0.05719	-0.20273	0.19704	-0.00858	0.29586	-0.29586	0.27722	0.05199	0.42476	0.45748	0.00783	0.00979	0.91127	8.53E-05	8.53E-05	turquoise
QPRT	-0.00573	-0.06443	-0.05114	0.10491	-0.0188	0.04031	0.03624	0.17237	-0.17237	0.94074	0.40246	0.5065	0.17207	0.80716	0.60062	0.63797	0.02417	0.02417	grey
HECA	-0.02821	-0.09049	0.10531	0.07089	-0.22997	0.21847	0.03	0.21997	-0.21997	0.71421	0.23917	0.17044	0.35687	0.00248	0.0041	0.69691	0.00384	0.00384	turquoise
RAB3C	-0.01748	-0.13275	-0.21938	-0.10973	0.04771	-0.24056	-0.25112	0.54099	-0.54099	0.82049	0.08347	0.00394	0.15308	0.53549	0.00153	0.00092	2.20E-14	2.20E-14	brown
RNF123	-0.03521	-0.12982	-0.05159	-0.0885	0.00865	0.00236	-0.09043	0.14783	-0.14783	0.6475	0.09059	0.50278	0.24971	0.91063	0.97556	0.2395	0.05366	0.05366	turquoise
SLC25A23	0.10137	-0.03797	-0.07668	0.01176	0.04796	0.0413	-0.04403	-0.12729	0.12729	0.1871	0.62198	0.31886	0.87872	0.53337	0.59176	0.56741	0.09709	0.09709	grey
INTS2	-0.10866	-0.1271	-0.10702	-0.01909	-0.01306	-0.0961	-0.01889	0.25292	-0.25292	0.15717	0.09761	0.16355	0.80432	0.86534	0.21116	0.80624	0.00085	0.00085	turquoise
PXK	-0.04336	-0.0867	-0.06931	-0.05139	-0.15728	0.00055	-0.07091	0.49952	-0.49952	0.57338	0.25948	0.36771	0.50441	0.03994	0.99435	0.3567	3.53E-12	3.53E-12	blue
PCBP2	0.10172	-0.1178	-0.1194	0.01661	0.08636	-0.20094	-0.15832	0.03596	-0.03596	0.18558	0.12491	0.11983	0.82933	0.2614	0.00841	0.03862	0.64056	0.64056	turquoise
RIBC2	-0.08377	-0.02383	-0.11561	-0.00859	0.03263	-0.11404	0.13199	-0.16313	0.16313	0.27603	0.757	0.13212	0.91123	0.67176	0.13749	0.08527	0.03302	0.03302	grey
ATRAID	0.02487	-0.01845	-0.0657	-0.01248	-0.00069	-0.03648	0.1144	-0.11141	0.11141	0.74676	0.81071	0.39325	0.87132	0.99289	0.63574	0.13625	0.14685	0.14685	grey
PAPSS2	-0.0755	-0.24176	-0.0725	0.02059	-0.01659	-0.14503	-0.13023	0.43833	-0.43833	0.32639	0.00144	0.34602	0.7892	0.82949	0.05841	0.08956	2.02E-09	2.02E-09	turquoise
ANO6	-0.01302	-0.12687	0.08508	0.19129	-0.18171	0.21947	0.1282	0.06724	-0.06724	0.86578	0.0982	0.26854	0.0122	0.01738	0.00393	0.09471	0.38223	0.38223	turquoise
ZNF420	-0.05607	-0.17424	-0.10292	0.00534	-0.0392	-0.07112	-0.07172	0.36682	-0.36682	0.46635	0.02265	0.1804	0.94473	0.61068	0.35527	0.35127	8.03E-07	8.03E-07	turquoise
HIBADH	-0.05489	-0.05984	-0.01309	0.09049	-0.01951	0.00906	0.0195	-0.01991	0.01991	0.47585	0.4369	0.86509	0.23918	0.80003	0.90641	0.8001	0.79605	0.79605	turquoise
NCOA5	0.00448	-0.14638	-0.15378	-0.00666	0.04971	-0.17895	-0.13046	0.33857	-0.33857	0.95362	0.05608	0.04463	0.93112	0.5185	0.01919	0.089	5.92E-06	5.92E-06	turquoise
FAM189A	-0.14646	-0.00612	-0.00563	0.06407	0.07019	0.14805	-0.05421	-0.04791	0.04791	0.05595	0.93669	0.9417	0.40514	0.36162	0.05331	0.48128	0.53378	0.53378	grey
SHFL	0.03288	0.02076	-0.05017	0.04153	-0.02682	0.05297	0.14087	-0.10952	0.10952	0.6694	0.78749	0.51463	0.5897	0.72766	0.49144	0.06609	0.15387	0.15387	tan
PPP2R3A	-0.02446	-0.1259	-0.03704	0.09764	-0.16041	0.11442	0.14329	0.13249	-0.13249	0.75082	0.10083	0.6305	0.20391	0.03609	0.13618	0.06153	0.08408	0.08408	turquoise
SLAMF9	-0.02358	0.01936	0.07224	0.03342	-0.03457	0.06794	0.12761	-0.23365	0.23365	0.75951	0.80155	0.34777	0.66432	0.65353	0.37724	0.09627	0.0021	0.0021	grey
FOXN2	-0.03448	-0.13691	0.03442	0.07554	-0.18485	0.12587	0.09999	0.09146	-0.09146	0.65433	0.07416	0.65488	0.32613	0.0155	0.10091	0.19318	0.23418	0.23418	turquoise
KRT8	0.1674	0.04443	0.02187	0.04239	0.12362	0.10976	0.1294	-0.52942	0.52942	0.02864	0.56391	0.77647	0.58199	0.1072	0.15298	0.09164	9.71E-14	9.71E-14	yellow
ERI3	-0.04162	-0.08882	-0.14232	-0.0997	-0.00051	-0.12661	-0.00057	0.0687	-0.0687	0.5889	0.24798	0.06332	0.1945	0.9947	0.09892	0.99409	0.37196	0.37196	grey
MRPL34	0.0407	0.02179	-0.21136	-0.1003	0.30997	-0.19711	-0.10224	-0.09586	0.09586	0.59713	0.77726	0.00552	0.19179	3.70E-05	0.00977	0.1833	0.21231	0.21231	green
CRLS1	0.02339	0.05345	0.02086	0.07547	0.06795	0.07826	0.13494	-0.2951	0.2951	0.76134	0.4875	0.78657	0.32654	0.37723	0.30896	0.07847	8.92E-05	8.92E-05	grey
TUFM	0.11113	0.11618	-0.05214	-0.04174	0.05589	-0.05098	0.18222	-0.4032	0.4032	0.14787	0.13021	0.49825	0.58778	0.46783	0.50785	0.01707	4.56E-08	4.56E-08	grey
TMEM135	-0.06024	-0.1191	0.01359	-0.00756	-0.06129	0.05858	-0.01732	0.05338	-0.05338	0.43384	0.12076	0.85996	0.92179	0.42587	0.4466	0.82207	0.48803	0.48803	turquoise
MORC3	-0.04355	-0.15272	0.01554	0.05968	-0.02124	0.09188	0.02074	0.18223	-0.18223	0.57166	0.04614	0.84013	0.43808	0.78278	0.23198	0.78778	0.01706	0.01706	turquoise
ADRA2A	-0.04117	0.03427	0.04823	0.07977	-0.0271	0.1344	0.01568	0.09339	-0.09339	0.59292	0.65629	0.531	0.29969	0.72499	0.07968	0.8387	0.22437	0.22437	grey
CYBB	-0.16406	0.02862	0.11352	0.04591	-0.26404	0.19553	0.09275	0.37927	-0.37927	0.03202	0.71022	0.13932	0.55097	0.00048	0.01038	0.22761	3.13E-07	3.13E-07	blue
LNP1	-0.08637	-0.13752	-0.23744	-0.14876	0.01	-0.21433	-0.07208	0.47905	-0.47905	0.26136	0.07286	0.00177	0.05215	0.89668	0.00488	0.3488	3.40E-11	3.40E-11	brown
C4orf3	-0.08878	-0.19763	-0.04202	0.02735	-0.09739	0.06692	-0.01024	0.16842	-0.16842	0.24823	0.00957	0.58529	0.72251	0.20507	0.3845	0.89425	0.02766	0.02766	turquoise
SMARCA2	-0.00464	-0.12851	-0.12105	-0.01266	-0.0307	-0.08196	-0.13803	0.50332	-0.50332	0.95199	0.09392	0.11477	0.86942	0.69014	0.28656	0.0718	2.28E-12	2.28E-12	turquoise
TNIP1	-0.00334	-0.14632	-0.01684	-0.01396	-0.08241	0.1681	0.13336	-0.28078	0.28078	0.96544	0.05617	0.82695	0.85617	0.28391	0.02797	0.08206	0.0002	0.0002	turquoise
UBTF	0.02743	-0.0968	-0.10039	-0.01467	0.03953	-0.05965	-0.06596	0.26191	-0.26191	0.7217	0.20786	0.19141	0.84896	0.60773	0.43837	0.39135	0.00054	0.00054	turquoise
G3BP1	-0.01701	-0.10874	0.00442	0.03735	-0.14017	0.10844	0.07085	0.06547	-0.06547	0.82522	0.15686	0.95422	0.62765	0.06747	0.158	0.35713	0.39488	0.39488	turquoise
SH3PXD2E	-0.04749	-0.11951	0.02498	0.11614	-0.23162	0.10098	0.08044	0.2945	-0.2945	0.53734	0.11948	0.74567	0.13037	0.0023	0.18881	0.29559	9.23E-05	9.23E-05	black
BBS7	-0.13269	-0.14639	-0.08195	-0.03668	-0.05168	-0.021	-0.03562	0.24107	-0.24107	0.08361	0.05606	0.28659	0.63389	0.50204	0.78517	0.64375	0.00149	0.00149	turquoise
ACSL4	-0.0646	-0.20304	0.00413	0.07799	-0.16734	0.14282	0.04376	0.16779	-0.16779	0.40121	0.00774	0.95723	0.31059	0.02869	0.0624	0.56984	0.02827	0.02827	turquoise
ZNF106	-0.10091	-0.17197	-0.01632	0.03828	-0.16544	0.09519	-0.00277	0.41226	-0.41226	0.18909	0.02451	0.83221	0.61916	0.03058	0.21553	0.97129	2.11E-08	2.11E-08	turquoise
EEPD1	0.03758	-0.11141	-0.03862	0.05929	-0.04672	0.16311	0.04009	-0.14448	0.14448	0.62553	0.14684	0.61598	0.44115	0.54397	0.03304	0.60266	0.05937	0.05937	grey
ETV4	0.05758	0.05904	0.06264	-0.01882	0.04493	0.16649	0.21376	-0.51084	0.51084	0.4544	0.44304	0.41568	0.80699	0.55957	0.02953	0.005	9.44E-13	9.44E-13	grey
EDN2	0.02435	-0.12651	-0.05044	0.13731	-0.05614	0.03131	0.03595	-0.21248	0.21248	0.75186	0.09917	0.51232	0.07331	0.46583	0.68435	0.64064	0.00527	0.00527	grey
RDH14	-0.07868	-0.06032	-0.07447	0.00933	-0.09567	0.0419	0.09474	-0.00254	0.00254	0.30633	0.43324	0.33305	0.90361	0.21325	0.58636	0.21773	0.97373	0.97373	turquoise
UBAC2	0.01886	-0.0282	-0.01622	0.0218	-0.047	0.00813	0.20728	-0.18651	0.18651	0.80661	0.71426	0.83325	0.7772	0.5416	0.91597	0.00652	0.01458	0.01458	turquoise

SQSTM1	0.13622	-0.12726	-0.0108	-0.02281	-0.0102	0.1659	0.04236	-0.13046	0.13046	0.07565	0.09718	0.88852	0.76718	0.89462	0.03012	0.58221	0.089	0.089	turquoise
TAPBPL	0.0772	-0.00891	-0.00439	0.02557	0.07889	-0.03956	-0.09176	-0.00874	0.00874	0.31558	0.9079	0.95459	0.73987	0.30506	0.60748	0.23262	0.90962	0.90962	grey
KAT2B	-0.04216	-0.17019	-0.05534	0.01449	-0.10038	0.04204	-0.10206	0.49388	-0.49388	0.58402	0.02605	0.47216	0.85075	0.19146	0.58512	0.18408	6.69E-12	6.69E-12	turquoise
C11orf45	-0.1013	-0.13266	-0.07674	0.09429	-0.15351	0.03328	0.17149	0.00336	-0.00336	0.18738	0.08369	0.31847	0.21995	0.04501	0.66561	0.02491	0.96516	0.96516	grey
PRELP	-0.03485	-0.04879	0.02882	0.02962	-0.17111	0.22952	-0.00092	0.31276	-0.31276	0.6509	0.52629	0.7083	0.70057	0.02524	0.00253	0.99044	3.12E-05	3.12E-05	blue
NSD3	0.04226	-0.03268	0.03445	0.04246	-0.01452	0.10655	0.11501	-0.00319	0.00319	0.58312	0.67136	0.65462	0.58136	0.85048	0.16541	0.13418	0.96693	0.96693	turquoise
LDLRAD1	0.01395	-0.1234	-0.14344	-0.02217	0.17975	-0.22197	-0.22187	-0.01488	0.01488	0.85626	0.10785	0.06125	0.7735	0.01865	0.00353	0.00354	0.84686	0.84686	grey
TOM1	-0.02976	-0.05476	-0.11736	0.00384	-0.02537	-0.05471	0.05159	-0.0319	0.0319	0.69916	0.47684	0.12633	0.96028	0.74186	0.47727	0.50282	0.67871	0.67871	grey
TRPM4	0.05245	0.10698	0.13107	0.03876	0.08218	0.26756	0.19923	-0.42453	0.42453	0.49568	0.16373	0.08751	0.61472	0.28527	0.0004	0.00899	7.17E-09	7.17E-09	yellow
SUMF2	-0.00451	-0.1734	0.01634	0.06114	0.06341	-0.11893	-0.07485	-0.07384	0.07384	0.95327	0.02332	0.83205	0.42699	0.40999	0.1213	0.33058	0.33715	0.33715	grey
PCDHGB2	-0.01889	-0.1399	0.02631	0.05488	-0.19418	0.1287	0.05968	-0.01429	0.01429	0.80631	0.068	0.73268	0.47592	0.01093	0.09343	0.43813	0.85283	0.85283	grey
NECTIN1	0.06491	-0.00127	-0.00599	-0.07932	-0.02766	-0.06594	0.19099	-0.19109	0.19109	0.39894	0.9868	0.93805	0.30239	0.71952	0.39153	0.01234	0.01229	0.01229	grey
ATXN7L2	-0.04342	-0.01429	-0.12917	-0.07115	0.19712	-0.13253	-0.10306	0.15754	-0.15754	0.5728	0.85283	0.09222	0.35507	0.00976	0.08399	0.1798	0.0396	0.0396	red
SCAP	0.07102	-0.05067	-0.12982	-0.08913	0.09632	-0.00462	-0.01003	-0.01627	0.01627	0.35596	0.51042	0.09059	0.24636	0.21012	0.95215	0.89638	0.83273	0.83273	grey
TMEM53	0.00605	-0.03003	-0.07798	-0.12077	0.09727	-0.00251	-0.02493	-0.25256	0.25256	0.93744	0.69658	0.31071	0.11562	0.20563	0.97397	0.74623	0.00086	0.00086	grey
KDM7A	-0.01432	-0.14166	0.05843	-0.01549	0.02827	0.08128	-0.00136	0.07527	-0.07527	0.85257	0.06458	0.4478	0.84068	0.71355	0.29059	0.98595	0.32785	0.32785	turquoise
CD1E	-0.093	-0.13016	-0.01149	-0.03168	-0.05629	0.13799	-0.02076	0.39583	-0.39583	0.22632	0.08974	0.88146	0.68085	0.46458	0.07189	0.78751	8.38E-08	8.38E-08	blue
CD34	-0.16356	-0.1077	-0.06931	0.04151	-0.09338	-0.01066	-0.13386	0.58142	-0.58142	0.03256	0.16089	0.36772	0.58985	0.22446	0.88999	0.08089	7.54E-17	7.54E-17	blue
SLC11A2	-0.01744	-0.18997	-0.07243	0.02796	0.04343	-0.04542	-0.09232	0.10483	-0.10483	0.82093	0.01282	0.3465	0.71662	0.57277	0.55529	0.22976	0.17238	0.17238	turquoise
FOXO3B	-0.08148	-0.08158	0.00635	0.07564	-0.16395	0.1411	0.05648	0.41258	-0.41258	0.28943	0.28882	0.93432	0.32549	0.03213	0.06565	0.46307	2.05E-08	2.05E-08	turquoise
NMNAT2	0.02406	-0.14505	-0.12237	-0.04627	-0.03562	-0.17656	-0.11012	0.21007	-0.21007	0.75474	0.05837	0.11084	0.54789	0.64373	0.02089	0.15162	0.00582	0.00582	brown
PANK4	-0.08296	-0.13649	-0.07829	0.01265	0.07321	-0.05576	-0.09734	0.17644	-0.17644	0.2807	0.07506	0.30879	0.8696	0.34127	0.46881	0.20533	0.02098	0.02098	turquoise
PLPP5	-0.00435	0.03996	-0.0669	-0.04633	0.19904	-0.20129	-0.13868	0.11374	-0.11374	0.95496	0.60384	0.3846	0.54735	0.00906	0.00829	0.07047	0.13855	0.13855	grey
EZH2	-0.03453	0.08748	0.08571	-0.01507	0.03033	0.08047	0.23316	-0.42247	0.42247	0.6539	0.25525	0.26502	0.84489	0.69376	0.29544	0.00215	8.62E-09	8.62E-09	pink
SKIL	-0.01597	-0.1202	0.05099	0.09644	-0.17849	0.22498	0.0805	0.06741	-0.06741	0.83579	0.11735	0.50778	0.20957	0.01951	0.00309	0.29526	0.381	0.381	turquoise
CWF19L2	0.00696	-0.02796	0.04565	0.00899	-0.22063	0.16262	0.10143	0.17713	-0.17713	0.92799	0.7166	0.55326	0.90705	0.00373	0.03358	0.18684	0.02047	0.02047	turquoise
HMG20B	0.0776	0.06981	0.02218	0.02075	0.07343	0.041	0.15872	-0.41211	0.41211	0.31306	0.36423	0.77342	0.78767	0.33983	0.59444	0.03813	2.14E-08	2.14E-08	green
TMEM189	0.07236	-0.01744	0.04026	0.03145	-0.04391	0.08754	0.17225	-0.39931	0.39931	0.34696	0.82086	0.60111	0.68304	0.56854	0.2549	0.02427	6.30E-08	6.30E-08	pink
COG8	-0.03961	-0.09529	-0.17695	-0.11284	0.0569	-0.138	-0.00932	0.17723	-0.17723	0.607	0.21507	0.0206	0.1417	0.4598	0.07186	0.90371	0.02039	0.02039	turquoise
DNTTIP1	0.1097	-0.03296	0.11763	0.0572	0.00639	0.08523	0.08257	-0.47483	0.47483	0.15319	0.66865	0.12547	0.45745	0.93391	0.26769	0.28295	5.33E-11	5.33E-11	grey
ZNF597	-0.02121	-0.1409	-0.01931	0.00404	-0.11276	0.12638	-0.0366	0.13559	-0.13559	0.78302	0.06604	0.8021	0.95819	0.14199	0.09952	0.63465	0.07702	0.07702	turquoise
JRKL	0.01457	-0.15779	-0.00358	0.04896	-0.0843	0.11376	0.14569	-0.14294	0.14294	0.85002	0.03929	0.96291	0.5248	0.27298	0.13849	0.05726	0.06217	0.06217	turquoise
PRUNE2	0.0285	-0.0263	-0.1197	-0.02936	0.06104	-0.14712	-0.12006	0.40302	-0.40302	0.71137	0.73272	0.11891	0.70307	0.42769	0.05484	0.11779	4.63E-08	4.63E-08	brown
UNC50	-0.0053	-0.09195	-0.09637	-0.02595	0.00645	-0.0842	0.01185	0.09517	-0.09517	0.94513	0.23163	0.20989	0.73614	0.93322	0.27356	0.8777	0.21562	0.21562	turquoise
TFF1	0.00337	0.11408	0.18329	0.03832	0.15548	0.24777	0.07997	-0.35616	0.35616	0.96513	0.13737	0.01641	0.61874	0.04229	0.00109	0.29844	1.74E-06	1.74E-06	yellow
NINJ1	-0.09758	-0.08573	0.08817	0.16713	-0.20557	0.15847	0.13638	0.06898	-0.06898	0.20417	0.26489	0.2515	0.0289	0.00699	0.03844	0.07529	0.37003	0.37003	black
ACVR2A	0.06727	-0.10128	0.01987	0.04815	-0.07768	0.15571	-0.04793	0.23895	-0.23895	0.38199	0.18749	0.79644	0.5317	0.31254	0.04199	0.53362	0.00165	0.00165	turquoise
CUTC	-0.05834	-0.02862	-0.02059	0.00161	-0.04899	-0.0497	0.05637	0.23061	-0.23061	0.44848	0.71021	0.78925	0.98337	0.52457	0.51862	0.46396	0.00241	0.00241	blue
ZNF57	0.09598	0.05518	-0.02125	-0.02823	0.10523	0.08668	-0.01674	-0.30185	0.30185	0.21173	0.47346	0.78264	0.714	0.17075	0.25959	0.82794	6.01E-05	6.01E-05	grey
GDF11	-0.02721	-0.21122	-0.15328	-0.03877	-0.01797	-0.29424	-0.17668	0.47471	-0.47471	0.72391	0.00555	0.04534	0.61468	0.81555	9.37E-05	0.0208	5.39E-11	5.39E-11	brown
EMC2	0.00197	-0.06267	0.02788	0.06103	-0.08892	-0.04297	0.04225	-0.03698	0.03698	0.97955	0.41549	0.71732	0.42782	0.24746	0.57683	0.58322	0.63109	0.63109	turquoise
CITED4	0.09493	0.02599	0.05778	0.09848	-0.05589	0.14618	0.15504	-0.33195	0.33195	0.21679	0.73577	0.45289	0.20005	0.46784	0.05642	0.04289	9.19E-06	9.19E-06	grey
SCP2	-0.09277	-0.08016	0.05425	0.03057	0.07209	0.08277	-0.08824	0.03937	-0.03937	0.22752	0.29733	0.48097	0.6914	0.34877	0.28178	0.25109	0.60921	0.60921	turquoise
SCAF1	0.1071	-0.04185	-0.12289	-0.11152	0.1211	-0.0756	0.05901	-0.27875	0.27875	0.16323	0.58676	0.10932	0.14645	0.1146	0.32571	0.44326	0.00022	0.00022	green
DTX2	0.05569	-0.00966	0.09159	0.03531	0.08738	0.11782	0.22988	-0.48765	0.48765	0.46937	0.90024	0.23349	0.64663	0.25576	0.12485	0.00249	1.34E-11	1.34E-11	green
CPNE7	0.20264	0.07124	-0.00701	-0.04473	0.01406	0.0903	0.15247	-0.23483	0.23483	0.00786	0.35451	0.92744	0.56129	0.85516	0.24014	0.0465	0.00199	0.00199	grey
C6orf136	0.12605	0.02021	-0.08151	-0.11779	0.25045	-0.16372	-0.03149	-0.30355	0.30355	0.10043	0.793	0.28922	0.12493	0.00095	0.03238	0.68263	5.44E-05	5.44E-05	grey
BAIAP2L1	0.06247	-0.07371	0.12805	0.02112	0.07961	0.18079	0.14784	-0.55916	0.55916	0.41698	0.33803	0.09512	0.78391	0.30063	0.01796	0.05364	1.88E-15	1.88E-15	yellow
LMTK3	0.10623	-0.11527	-0.10701	-0.01676	0.12043	-0.09662	0.0166	-0.3251	0.3251	0.16669	0.13326	0.1636	0.82777	0.11663	0.20868	0.82939	1.43E-05	1.43E-05	grey
PABPC1	0.0661	0.03858	0.10413	0.01457	-0.12942	0.18643	0.21505	-0.26375	0.26375	0.39034	0.61636	0.17529	0.85001	0.0916	0.01463	0.00473	0.00049	0.00049	turquoise
KLF6	-0.05002	-0.11973	0.06634	0.06363	-0.17728	0.2129	0.08144	0.01642	-0.01642	0.51585	0.11882	0.38866	0.40836	0.02036	0.00518	0.28966	0.8312	0.8312	turquoise
KCNN4	0.02009	0.06457	0.17159	0.03956	-0.05434	0.2803	0.33105	-0.45144	0.45144	0.79428	0.40148	0.02483	0.60744	0.48027	0.0002	9.75E-06	5.75E-10	5.75E-10	yellow
CDK7	0.07028	-0.01374	0.00591	-0.04541	-0.04552	0.03095	0.16139	-0.34441	0.34441	0.36103	0.85844	0.93884	0.55533	0.55436	0.68781	0.03496	3.98E-06	3.98E-06	turquoise
JAG1	-0.05135	-0.03377	0.0274	0.0594	-0.16071	0.17315	0.14263	-0.0214	0.0214	0.5048	0.66106	0.72199	0.44027	0.03574	0.02353	0.06274	0.78117	0.78117	turquoise
MAP3K2	-0.02226	-0.1602	0.00733	0.06162	-0.13556	0.10974	0.00341	0.20359	-0.20359	0.7726	0.03634	0.92422	0.42331	0.07709	0.15304	0.96468	0.00757	0.00757	turquoise
MAD2L2	-0.01005	-0.04014	-0.03762	0.04844	-0.11164	-0.04791	0.0915	0.06561	-0.06561	0.89625	0.60221	0.62523	0.52925	0.14604	0.53376	0.23397	0.39389	0.39389	grey
PNN	0.04012	-0.15074	-0.043	-0.02845	0.09514	0.00879	0.00172	-0.06002	0.06002	0.60233	0.04908	0.57658	0.71183	0.21578	0.90918	0.98219	0.43547	0.43547	red
PPP5C	0.07079	-0.11721	-0.20937	-0.06436	0.15519	-0.16676	-0.00366	-0.01539	0.01539	0.35753	0.12683	0.00599	0.40297	0.04269	0.02926	0.96206	0.84162	0.84162	grey
HIBCH	-0.01715	0.09323	0.07224	0.04784	0.02558	0.12459	0.07037	-0.1358	0.1358	0.82383	0.22521	0.34777	0.53441	0.73985	0.10445	0.36044	0.07657	0.07657	grey
RAPGEF2	-0.096	-0.18782	-0.05335	0.03753	-0.08401	0.04556	-0.0611	0.30791	-0.30791	0.21164	0.01389	0.48826	0.62598	0.27465	0.55401	0.42731	4.19E-05	4.19E-05	turquoise
ZNF454	-0.00619	-0.19512	-0.07638	0.07081	-0.06662	-0.05897	-0.08767	0.5321	-0.5321	0.93597	0.01054	0.32075	0.35738	0.38661	0.44359	0.25418	6.91E-14	6.91E-14	turquoise
RNF216	-0.08116	-0.14115	-0.0883	0.0176	-0.03421	-0.15096	-0.10808	0.3681	-0.3681	0.29133	0.06555	0.25081	0.81928	0.6569	0.04873	0.15939	7.30E-07	7.30E-07	turquoise
DYRK3	-0.11814	-0.21941	-0.10274	0.07525	-0.0709	0.0423	-0.09826	0.40472	-0.40472	0.12381	0.00394	0.18116	0.328	0.35682	0.58274	0.20102	4.01E-08	4.01E-08	turquoise
SYVN1	0.04915	-0.15977	-0.051	-0.0115	-0.00908	-0.10407	-0.08999	-0.04628	0.04628	0.52319	0.03685	0.50767	0.88132	0.90618	0.17556	0.2418	0.54777	0.54777	turquoise
MLLT3	0.00634	0.02248	-0.17716	-0.05732	0.06162	0.00599	-0.0363	0.09943	-0.09943	0.9344	0.77042	0.02045	0.45649	0.42332	0.93803	0.63741	0.19572	0.19572	turquoise
ARGLU1	0.00243	-0.10652	-0.05909	-0.08722	0.18074	-0.08215	-0.01158	-0.07664	0.07664	0.97485	0.16554	0.44265	0.25663	0.01799	0.28542	0.88046	0.31907	0.31907	red
P3H2	0.04501	-0.01036	0.16007	0.07875	-0.12152	0.21762	0.11577	-0.19175	0.19175	0.55888	0.89304	0.03649	0.30591	0.11335	0.00425	0.13159	0.01199	0.01199	grey
GPATCH1	0.06792	-0.1661	-0.00826	0.07171	0.01821	0.0065	0.04644	-0.1523	0.1523	0.37741	0.02991	0.91463	0.35132	0.81316	0.93277	0.54645	0.04674	0.04674	turquoise
RWDD4	-0.10222	-0.11789	-0.09269	-0.05243	0.04896	-0.07595	-0.0829	0.08979	-0.08979	0.18338	0.12462	0.22791	0.49581	0.52481	0.32351	0.28103	0.24284	0.24284	turquoise
SULT1A2	0.02161	-0.11603	-0.09249	-0.00082	0.09317	-0.02457	0.02202	-0.0996	0.0996	0.77902	0.1307	0.2289	0.99153	0.22551	0.74969	0.77495	0.19493	0.19493	grey
RORC	0.11214	0.11472	-0.07208	-0.00643	0.18653	0.10635	-0.07051	-0.12712	0.12712	0.1442	0.13514	0.3488	0.93345	0.01457	0.16623	0.35942	0.09755	0.09755	grey
C1GALT1(	0.01446	-0.08771	-0.00635	0.01835	-0.14802	0.01695	0.0668	0.14884	-0.14884	0.85105	0.254	0.9343	0.81171	0.05335	0.82583	0.38536	0.05202	0.05202	grey
SEC14L2	-0.07969	-0.04962	0.00113	0.01336	-0.10035	0.05662	0.17905	-0.31414	0.31414	0.30015	0.51921	0.98833	0.86228	0.19157	0.46203	0.01912	2.86E-05	2.86E-05	yellow
ZNF559	-0.15645	-0.12361	-0.05679	-0.00194	-0.04634	-0.13062	-0.1187	0.43656	-0.43656	0.04101	0.10724	0.46066	0.97995	0.54727	0.08859	0.12203	2.38E-09	2.38E-09	turquoise
RRAD	-0.0798	-0.07273	0.03802	0.11698	-0.02895	0.16293	0.11584	-0.10511	0.10511	0.29952	0.34447	0.62154	0.12758	0.70701	0.03323	0.13134	0.17124	0.17124	grey
IDUA	0.00451	-0.01777	-0.09451	-0.04107	0.23783	-0.07077	0.03328	-0.27021	0.27021	0.95331	0.81751	0.21885	0.59376	0.00173	0.35766	0.66562	0.00035	0.00035	green
H2AFX	0.00835	0.01419	-0.06374	-0.07325	0.07769	-0.03593	0.235	-0.4824	0.4824	0.91369	0.85387	0.40751	0.34103	0.31247	0.64084	0.00198	2.37E-11	2.37E-11	green
RPL7L1	-0.03312	-0.06071	-0.08584	-0.00277	-0.04737	-0.06093	-0.01152	0.20746	-0.20746	0.66716	0.43027	0.2643	0.97136	0.53837	0.42858	0.88108	0.00648	0.00648	turquoise
HAPLN3	-0.06734	0.08883	0.06925	0.09064	-0.13973	0.18001	0.14661	-0.05445	0.05445	0.38151	0.24791	0.36814	0.23839	0.06835	0.01848	0.05569	0.47933	0.47933	black
RPS27L	-0.00768	0.04391	0.0169	-0.01227	0.0503	0.03861	0.04306	-0.17715	0.17715	0.92056	0.56852	0.82637	0.8734	0.51351	0.61614	0.57598	0.02045	0.02045	grey
TUFT1	0.1576	-0.08719	0.04405	-0.00856	0.02533	0.13818	0.09183	-0.43489	0.43489	0.03953	0.25683	0.5673	0.91155	0.74227	0.0715	0.23228	2.79E-09	2.79E-09	yellow
SDK2	0.03991	-0.06507	-0.06283	-0.03034	0.01535	-0.13221	-0.23226	0.27016	-0.27016	0.60429	0.39781	0.4143	0.69361	0.84202	0.08476	0.00224	0.00035	0.00035	grey
RAB41	-0.03501	-0.29628	-0.18005	-0.07367	-0.02617	-0.19892	-0.05391	0.04345	-0.04345	0.64944	8.33E-05	0.01845	0.3383	0.73402	0.0091	0.48371	0.57255	0.57255	grey
SERPINA6	0.05025	-0.05485	-0.15817	0.04547	0.09571	-0.16251	-0.18588	0.18075	-0.18075	0.51396	0.47611	0.0388	0.55486	0.21302	0.0337	0.01493	0.01799	0.01799	magenta
DDA1	0.03843	-0.08119	-0.04253	-0.04716	0.13936	-0.04723	0.01413	-0.33588	0.33588	0.61771	0.29113	0.58068	0.54018	0.06907	0.53956	0.85443	7.08E-06	7.08E-06	green
TCTN3	-0.00107	-0.14757	-0.02568	-0.03111	-0.16839	-0.05734	0.04775	0.25916	-0.25916	0.98893	0.0541	0.73888	0.68629	0.0277	0.45629	0.53509	0.00062	0.00062	turquoise
LAT2	-0.13816	-0.09413	-0.07749	-0.03414	-0.13043	-0.04828	-0.088	0.40536	-0.40536	0.07154	0.22074	0.31376	0.65754	0.08905	0.53064	0.25242	3.80E-08	3.80E-08	blue
MYLK2	-0.11328	0.12194	0.01569	0.10871	-0.17027	0.0505	-0.01952	0.16366	-0.16366	0.14015	0.11209	0.83857	0.15698	0.02598	0.51183	0.79998	0.03245	0.03245	magenta
PIWIL2	-0.03924	0.04923	0.09099	0.07092	-0.0084	0.28992	-0.00173	0.02393	-0.02393	0.6104	0.52258	0.2366	0.35665	0.91313	0.00012	0.98211	0.75608	0.75608	grey
EFTUD2	-0.03934	-0.05167	0.02638	0.01024	-0.06434	0.12277	0.2199	-0.20058	0.20058	0.60941	0.50215	0.73195	0.8942	0.40315	0.10966	0.00385	0.00853	0.00853	greenyellow
NOL4L	0.07898	-0.17869	-0.01598	-0.01908	0.09893	0.04321	0.05991	-0.23965	0.23965	0.30449	0.01937	0.83571	0.80442	0.19797	0.5747	0.43638	0.00159	0.00159	turquoise
MBOAT7	0.19906	-0.16978	-0.08285	-0.01312	0.12018	-0.09419	0.07849	-0.28506	0.28506	0.00905	0.02642	0.28133	0.8648	0.11743	0.22041	0.3075	0.00016	0.00016	grey
HDHD3	0.08119	0.04085	0.00257	0.01498	0.18723	0.11441	0.08776	-0.43717	0.43717	0.29112	0.59577	0.97334	0.84579	0.0142	0.13622	0.25369	2.25E-09	2.25E-09	yellow
BSDC1	-0.03748	-0.23901	-0.05285	-0.05461	-0.04738	0.03365	-0.07503	0.06975	-0.06975	0.62649	0.00164	0.49242	0.47804	0.53833	0.66212	0.32942	0.36464	0.36464	turquoise
LSM3	0.01085	0.02532	-0.08898	-0.15927	-0.08524	-0.02778	0.16869	-0.12336	0.12336	0.88796	0.7424	0.24714	0.03746	0.26763	0.71838	0.02741	0.10796	0.10796	grey
PLAC8L1	-0.10675	-0.11981	-0.13945	0.04482	0.168	-0.18584	-0.12068	-0.01972	0.01972	0.16464	0.11856	0.06891	0.56053	0.02806	0.01495	0.11588	0.79792	0.79792	grey
MTDH	-0.0075	-0.03393	0.02442	0.06207	-0.15555	0.09153	0.10763	0.04549	-0.04549	0.9224	0.65951	0.75118	0.41998	0.0422	0.23379	0.16116	0.55466	0.55466	turquoise
STX12	-0.09015	-0.16048	0.00057	0.03659	-0.13716	0.07748	-0.07053	0.36232	-0.36232	0.24094	0.03601	0.99413	0.63465	0.07363	0.31381	0.35928	1.12E-06	1.12E-06	turquoise
SF3A1	0.01203	-0.10322	-0.03549	-0.01175	-0.14423	0.04202	-0.03376	0.25957	-0.25957	0.87592	0.17912	0.64493	0.87875	0.05983	0.58525	0.66114	0.00061	0.00061	turquoise
TP53RK	0.02003	-0.04499	-0.05874	-0.00967	-0.04508	-0.01933	0.07535	0.04406	-0.04406	0.79489	0.55905	0.4454	0.90006	0.55824	0.80187	0.32734	0.56718	0.56718	turquoise
STAM2	-0.00524	-0.12339	0.01564	0.07821	-0.06765	0.06617	-0.01816	0.14781	-0.14781	0.94582	0.10788	0.83914	0.30923	0.37933	0.38989	0.81362	0.05369	0.05369	turquoise
BFAR	0.00972	0.00263	0.09048	0.02959	-0.13922	0.22221	0.1254	-0.26745	0.26745	0.89964	0.97275	0.23921	0.70087	0.06936	0.00349	0.10221	0.00041	0.00041	turquoise
ERCC6	0.01343	-0.20058	0.01462	0.01501	-0.08244	0.01993	0.0463	0.15936	-0.15936	0.86163	0.00853	0.84952	0.84551	0.28375	0.79587	0.54762	0.03734	0.03734	turquoise
FUT1	-0.07985	-0.02907	-0.0634	-0.0232	-0.09598	-0.11007	-0.14722	0.15426	-0.15426	0.2992	0.70583	0.41006	0.76323	0.21176	0.1518	0.05466	0.04396	0.04396	grey
RBM39	0.05791	-0.1597	-0.04038	0.00542	0.0908	-0.02247	0.01069	-0.04357	0.04357	0.45187	0.03693	0.60005	0.94396	0.23758	0.77051	0.88961	0.57153	0.57153	red
CSF2RA	-0.11563	-0.0392	0.02784	0.09009	-0.19777	0.17423	0.08726	0.39187	-0.39187	0.13207	0.61074	0.71772	0.24125	0.00952	0.02266	0.25646	1.16E-07	1.16E-07	blue
SHISA6	-0.00629	-0.08404	-0.0686	0.01926	-0.21865	0.01409	0.12394	0.34783	-0.34783	0.93492	0.27448	0.37262	0.80261	0.00406	0.85491	0.10629	3.14E-06	3.14E-06	blue
EXOSC4	0.03165	0.06875	-0.12569	-0.07326	0.06179	-0.17656	0.18914	-0.36859	0.36859	0.68107	0.37159	0.10142	0.34095	0.42207	0.02089	0.01323	7.04E-07	7.04E-07	green
ZBTB7C	0.06432	0.114	0.06378	0.01835	0.06321	0.21421	0.14497	-0.1689	0.1689	0.40328	0.13764	0.40722	0.81173	0.41146	0.0049	0.05851	0.02722	0.02722	yellow
RWDD2B	-0.10029	-0.02024	-0.09583	0.03058	0.00849	-0.09549	-0.00719	0.15092	-0.15092	0.19184	0.79279	0.21248	0.69136	0.91227	0.21412	0.92568	0.04879	0.04879	turquoise
KCNH3	-0.04494	-0.17422	-0.28129	-0.15724	0.21026	-0.34047	-0.25531	0.26842	-0.26842	0.55948	0.02267	0.00019	0.03999	0.00578	5.20E-06	0.00075	0.00039	0.00039	brown
MAT2B	0.00524	-0.0763	0.05154	0.02979	-0.09725	0.17525	0.05826	-0.03859	0.03859	0.94574	0.32126	0.50323	0.6989	0.20574	0.02187	0.44913	0.61629	0.61629	turquoise
JUNB	-0.01971	-0.03166	-0.04166	0.01438	0.10298	0.07939	0.10327	-0.10953	0.10953	0.79805	0.68105	0.58849	0.85192	0.18013	0.30197	0.1789	0.15385	0.15385	grey
MROH1	0.05281	0.00566	-0.07273	-0.09839	0.07885	-0.10397	0.08255	-0.35365	0.35365	0.49268	0.94141	0.34451	0.20046	0.30527	0.17597	0.28311	2.09E-06	2.09E-06	green
ASPSCR1	0.03891	0.00325	-0.15206	-0.10868	0.2849	-0.23952	0.01026	-0.08177	0.08177	0.61333	0.96637	0.04709	0.15707	0.00016	0.0016	0.89407	0.28771	0.28771	green
CPTP	-0.03055	0.02704	0.0453	0.05215	0.00719	0.15595	0.14815	-0.43966	0.43966	0.69165	0.72554	0.55631	0.49812	0.92566	0.04167	0.05315	1.78E-09	1.78E-09	green
FOLH1	0.03131	-0.06764	0.00411	-0.03854	0.06321	0.03562	-0.05266	0.03365	-0.03365	0.68436	0.37937	0.95746	0.61673	0.41147	0.64367	0.49397	0.6622	0.6622	grey
DIPK2B	-0.24146	-0.07598	-0.06669	0.05257	-0.09348	-0.00075	-0.11036	0.56125	-0.56125	0.00146	0.32327	0.38611	0.49469	0.22396	0.9922	0.15074	1.41E-15	1.41E-15	blue
MRPS21	-0.05262	0.0223	-0.04569	-0.10942	-0.00083	-0.05503	0.12696	-0.22095	0.22095	0.49429	0.77224	0.5529	0.15427	0.99145	0.4747	0.09796	0.00368	0.00368	grey
NUBP2	-0.00481	0.04744	-0.12889	-0.05395	0.17916	-0.10873	0.06038	-0.2467	0.2467	0.95022	0.53778	0.09294	0.48344	0.01904	0.15689	0.43273	0.00114	0.00114	green
EHF	0.0342	-0.07368	0.05746	0.04812	-0.04421	0.15424	0.07701	-0.16202	0.16202	0.65695	0.33821	0.45534	0.53199	0.56582	0.04398	0.31674	0.03425	0.03425	turquoise
ANKRD13(	-0.14029	-0.14173	-0.0167	0.03521	-0.08658	0.03692	-0.01847	0.31173	-0.31173	0.06722	0.06445	0.8284	0.64752	0.26014	0.63161	0.8105	3.32E-05	3.32E-05	turquoise
AASDHPP	-0.08865	-0.13245	-0.09057	0.00871	-0.12689	-0.09322	-0.06646	0.28113	-0.28113	0.2489	0.08418	0.23874	0.90999	0.09818	0.22526	0.38776	0.0002	0.0002	turquoise
MAPK15	0.02854	-0.04186	0.00474	0.05579	0.10706	0.0148	0.23815	-0.39171	0.39171	0.71097	0.58673	0.95093	0.46863	0.1634	0.84768	0.00171	1.17E-07	1.17E-07	grey
ECHS1	0.08479	0.05354	-0.02383	-0.02975	0.07293	-0.14519	0.16101	-0.36974	0.36974	0.27018	0.48675	0.75707	0.69934	0.34312	0.05812	0.0354	6.46E-07	6.46E-07	grey
CEP76	-0.04911	-0.16078	-0.03914	-0.05793	0.0368	-0.03678	0.03719	0.17683	-0.17683	0.52355	0.03566	0.61125	0.45165	0.63277	0.6329	0.62918	0.02069	0.02069	turquoise
GGT6	-0.00278	0.01409	0.10648	0.10067	0.02162	0.28864	0.05473	-0.17446	0.17446	0.97125	0.8549	0.16569	0.19017	0.77897	0.00013	0.47713	0.02248	0.02248	grey
EDEM2	0.02468	-0.0815	0.04256	0.11579	-0.02005	-0.0308	0.07557	-0.13829	0.13829	0.74866	0.28926	0.58049	0.13151	0.79466	0.68922	0.32594	0.07126	0.07126	grey
KRT6A	0.06814	0.10505	0.07737	0.05978	-0.05034	0.02154	0.1386	-0.24049	0.24049	0.37586	0.17151	0.31447	0.43738	0.5132	0.77972	0.07063	0.00153	0.00153	grey

POLE4	0.00898	0.12901	0.03537	0.07419	-0.16266	0.19136	0.30058	-0.25752	0.25752	0.90716	0.09263	0.64601	0.33484	0.03353	0.01217	6.48E-05	0.00067	0.00067	yellow
SEC63	-0.07435	-0.08264	0.00652	0.03668	-0.12036	-0.02509	-0.08676	0.39774	-0.39774	0.33383	0.28255	0.93254	0.63388	0.11688	0.74462	0.25917	7.17E-08	7.17E-08	turquoise
CD209	-0.12557	0.03146	0.05903	-0.03485	-0.13514	0.00883	0.02204	0.51011	-0.51011	0.10173	0.68293	0.4431	0.65094	0.07802	0.90871	0.77475	1.03E-12	1.03E-12	blue
CTRC	0.00953	0.04113	0.06987	0.15155	-0.10003	0.07925	-0.07804	0.13666	-0.13666	0.90151	0.59324	0.36381	0.04786	0.19299	0.30285	0.31028	0.0747	0.0747	magenta
SLC2A4RG	0.13358	0.05754	-0.04405	0.01408	0.01787	-0.05133	0.18478	-0.41766	0.41766	0.08155	0.45478	0.56727	0.85494	0.81657	0.50492	0.01554	1.32E-08	1.32E-08	green
SPRR1A	-0.0133	0.1338	-0.02584	-0.06344	0.17307	0.0603	0.07638	-0.16316	0.16316	0.86297	0.08105	0.73724	0.40974	0.02359	0.43337	0.32076	0.03299	0.03299	grey
DUSP26	-0.01718	-0.0794	-0.27127	-0.14291	0.14049	-0.30314	-0.22101	0.41442	-0.41442	0.82349	0.30191	0.00033	0.06224	0.06683	5.57E-05	0.00367	1.75E-08	1.75E-08	brown
TRO	-0.02323	-0.18168	-0.15504	-0.00013	0.04234	-0.23728	-0.17578	0.52486	-0.52486	0.76295	0.0174	0.04289	0.99861	0.58239	0.00178	0.02146	1.72E-13	1.72E-13	brown
SEMA6A	-0.05377	-0.19451	-0.17522	0.05824	-0.03051	0.01905	-0.0992	0.32553	-0.32553	0.48484	0.01079	0.02189	0.44923	0.69201	0.80466	0.19673	1.40E-05	1.40E-05	grey
IGSF1	-0.05804	-0.07032	-0.1492	-0.03804	0.06902	-0.29395	-0.17447	0.28887	-0.28887	0.45079	0.36072	0.05145	0.62134	0.36969	9.53E-05	0.02247	0.00013	0.00013	brown
RBM27	-0.00858	-0.13281	-0.05176	-0.01576	-0.03447	0.01082	-0.0475	0.05524	-0.05524	0.91132	0.08332	0.50137	0.83792	0.65441	0.88834	0.53725	0.47297	0.47297	turquoise
FER	-0.02747	-0.16687	-0.07518	0.01962	-0.19833	0.04784	0.02151	0.28078	-0.28078	0.72135	0.02915	0.3284	0.79898	0.00931	0.53437	0.78009	0.0002	0.0002	turquoise
CD8A	-0.05813	0.01015	0.00918	0.11352	-0.19343	0.09408	0.03632	0.41195	-0.41195	0.45013	0.89517	0.90511	0.13929	0.01125	0.22096	0.63716	2.17E-08	2.17E-08	blue
CCDC183	0.00287	-0.10954	-0.15346	-0.09569	0.27449	-0.20624	-0.1555	-0.09445	0.09445	0.97032	0.15379	0.04507	0.21314	0.00028	0.0068	0.04227	0.21917	0.21917	grey
RPL23	0.05242	0.04463	-0.08841	-0.02452	-0.02546	0.00951	0.10041	-0.11585	0.11585	0.49591	0.56216	0.25017	0.75023	0.74096	0.90171	0.19132	0.13133	0.13133	purple
PRELID3A	0.07522	-0.07545	0.0011	0.05772	0.05342	0.0145	0.16024	-0.0967	0.0967	0.32815	0.3267	0.98862	0.45331	0.48776	0.85069	0.0363	0.20831	0.20831	grey
DNAJC18	-0.0862	-0.20132	-0.18757	-0.07181	0.00036	-0.24052	-0.19685	0.56229	-0.56229	0.26228	0.00828	0.01403	0.35061	0.99631	0.00153	0.00986	1.22E-15	1.22E-15	brown
PIK3R4	-0.0011	-0.0454	-0.05137	-0.03088	-0.11708	0.06611	0.08723	0.16784	-0.16784	0.98856	0.55543	0.5046	0.68849	0.12726	0.39028	0.25657	0.02822	0.02822	turquoise
SPARC	-0.04533	-0.07886	0.02407	0.06381	-0.16054	0.13255	0.00742	0.29447	-0.29447	0.55607	0.30521	0.75466	0.40701	0.03594	0.08395	0.92323	9.25E-05	9.25E-05	black
ENPP2	0.00432	-0.20867	-0.1383	-0.08314	-0.00792	-0.15461	-0.17021	0.43935	-0.43935	0.95533	0.00616	0.07125	0.27963	0.91814	0.04347	0.02603	1.84E-09	1.84E-09	brown
PPP1R18	-0.0387	-0.06394	0.08769	0.09449	-0.203	0.2134	0.11543	0.05591	-0.05591	0.61532	0.40604	0.25408	0.21896	0.00775	0.00507	0.13275	0.46765	0.46765	black
PPP1R12C	0.06161	-0.04132	-0.11175	-0.02013	0.14601	-0.09398	-0.06166	0.02864	-0.02864	0.4234	0.59157	0.14561	0.7938	0.05671	0.22148	0.42305	0.71005	0.71005	red
SPPL2A	-0.07417	-0.10463	0.00956	0.04804	-0.09336	0.10625	0.06226	0.08895	-0.08895	0.33498	0.17322	0.9012	0.53262	0.22454	0.16664	0.41854	0.24731	0.24731	turquoise
WDR82	-0.06138	-0.10439	-0.02136	-0.02509	-0.15779	0.1109	-0.02611	0.27385	-0.27385	0.42517	0.1742	0.78157	0.74461	0.03929	0.14873	0.73461	0.00029	0.00029	turquoise
POLDIP2	-0.06678	-0.01071	-0.10084	-0.02511	0.03859	-0.10466	0.09492	-0.22399	0.22399	0.38545	0.88939	0.1894	0.7444	0.6163	0.17311	0.21684	0.00323	0.00323	grey
COQ6	-0.03101	-0.1024	-0.13642	-0.09666	0.15869	-0.17084	-0.06516	-0.0013	0.0013	0.68723	0.18263	0.07521	0.20851	0.03816	0.02548	0.39716	0.98652	0.98652	turquoise
GUCY2C	0.02839	-0.01789	-0.00876	0.0369	0.17694	-0.01659	-0.09652	-0.05713	0.05713	0.71244	0.81637	0.90945	0.63187	0.02061	0.82951	0.20919	0.458	0.458	grey
PODNL1	-0.00182	0.01107	0.04503	0.07198	-0.09722	0.14306	0.13047	0.10055	-0.10055	0.98118	0.88569	0.55868	0.34952	0.20589	0.06195	0.08895	0.19067	0.19067	black
CNTD1	-0.02389	-0.03089	-0.1298	-0.07921	0.27785	-0.16219	-0.16998	-0.0398	0.0398	0.75645	0.6884	0.09064	0.30307	0.00023	0.03405	0.02624	0.60528	0.60528	grey
ZNF772	-0.04389	-0.21066	-0.15007	-0.03911	-0.05057	-0.0828	-0.13009	0.26176	-0.26176	0.56867	0.00568	0.0501	0.61156	0.51124	0.28164	0.08991	0.00054	0.00054	turquoise
SF3A3	0.01189	-0.0837	-0.02947	-0.02005	-0.1627	0.03908	0.0362	0.16349	-0.16349	0.87729	0.27643	0.70199	0.79469	0.03349	0.61185	0.63833	0.03263	0.03263	turquoise
RAB33A	-0.01084	-0.14522	-0.08865	-0.01778	-0.07032	-0.08749	-0.0727	0.36143	-0.36143	0.88807	0.05808	0.24888	0.81743	0.36074	0.25517	0.34468	1.19E-06	1.19E-06	blue
PHYHIP	-0.04189	-0.09504	-0.06934	-0.05399	-0.07887	0.00208	-0.04376	0.24481	-0.24481	0.58648	0.21629	0.36749	0.48311	0.30519	0.97851	0.56986	0.00125	0.00125	blue
ZNF296	0.13607	0.07654	-0.14913	-0.05414	0.19722	-0.14602	0.07761	-0.30897	0.30897	0.07598	0.3197	0.05156	0.48185	0.00972	0.0567	0.31299	3.93E-05	3.93E-05	green
MAP1B	-0.02194	-0.19359	-0.14466	-0.0489	-0.13481	-0.16236	-0.1877	0.5509	-0.5509	0.77576	0.01118	0.05907	0.52537	0.07875	0.03387	0.01395	5.86E-15	5.86E-15	brown
CSGALNA(	-0.08049	-0.14167	0.07965	0.07684	-0.20253	0.15351	0.03079	0.33032	-0.33032	0.29534	0.06455	0.30041	0.31784	0.00789	0.045	0.68935	1.02E-05	1.02E-05	blue
GMPPB	0.02711	-0.05201	-0.0775	-0.1421	0.16163	-0.08869	-0.073	-0.03989	0.03989	0.72483	0.49926	0.31366	0.06373	0.03468	0.24869	0.34269	0.60448	0.60448	grey
FXYD5	0.05656	-0.02935	0.05884	-0.0281	-0.12219	0.11836	0.22872	-0.2781	0.2781	0.46246	0.70313	0.44463	0.71519	0.11137	0.1231	0.00262	0.00023	0.00023	yellow
CDK10	0.06046	0.00248	-0.09877	-0.08078	0.30318	-0.11315	-0.00418	-0.30701	0.30701	0.43215	0.97431	0.19869	0.29357	5.56E-05	0.1406	0.95668	4.42E-05	4.42E-05	red
BEST1	-0.11601	-0.06717	-0.00319	0.03305	-0.17183	0.02505	-0.01872	0.56449	-0.56449	0.13079	0.38273	0.96698	0.66785	0.02463	0.74502	0.80801	8.92E-16	8.92E-16	blue
TMEM79	0.09179	-0.08338	-0.06135	-0.06559	0.08289	-0.02656	0.06628	-0.34504	0.34504	0.23246	0.27825	0.42541	0.39401	0.28113	0.73024	0.38907	3.81E-06	3.81E-06	grey
GBE1	-0.10877	-0.14656	-0.04793	-0.0077	-0.10494	-0.0071	0.02605	0.14456	-0.14456	0.15674	0.05578	0.53357	0.92039	0.17196	0.92653	0.73516	0.05924	0.05924	turquoise
SERP2	0.06044	-0.11114	-0.2176	-0.22595	0.13964	-0.31807	-0.2728	0.24256	-0.24256	0.43228	0.14785	0.00425	0.00296	0.06851	2.24E-05	0.00031	0.00139	0.00139	brown
INTS9	-0.01979	0.03045	0.01018	-0.00396	-0.05244	-0.01458	0.06017	0.16424	-0.16424	0.79727	0.69259	0.8949	0.95897	0.49572	0.84992	0.43436	0.03183	0.03183	turquoise
ACTR6	#######	-0.12708	-0.06873	-0.05518	0.06805	-0.13812	-0.12461	0.18614	-0.18614	0.99914	0.09767	0.37176	0.4735	0.37648	0.07161	0.1044	0.01479	0.01479	turquoise
IGSF22	0.01708	-0.07131	-0.16337	-0.14774	0.15827	-0.22981	-0.07408	-0.02849	0.02849	0.82457	0.354	0.03276	0.05381	0.03868	0.0025	0.33555	0.71146	0.71146	grey
PROM2	0.01315	-0.01432	0.01621	0.05858	-0.04623	0.10751	0.16189	-0.26251	0.26251	0.86448	0.85251	0.83335	0.44663	0.54821	0.16163	0.0344	0.00052	0.00052	yellow
DEFB1	0.02019	0.03195	-0.01309	0.08249	-0.08651	0.1308	0.06109	-0.20719	0.20719	0.7932	0.67827	0.86504	0.28345	0.26056	0.08815	0.42732	0.00655	0.00655	grey
RPRD1A	-0.06487	-0.15365	-0.12511	0.00407	0.05031	-0.13755	-0.10763	0.2612	-0.2612	0.39924	0.04482	0.10302	0.9579	0.51341	0.07281	0.16117	0.00056	0.00056	turquoise
EPHA2	0.03121	-0.00646	0.15376	0.0569	-0.05227	0.29403	0.22381	-0.39166	0.39166	0.68536	0.93321	0.04466	0.45982	0.49714	9.49E-05	0.00325	1.18E-07	1.18E-07	yellow
ZNF629	-0.01239	-0.07932	-0.1665	-0.04648	-0.04318	-0.11586	0.08045	-0.06459	0.06459	0.87225	0.30242	0.02951	0.54603	0.57493	0.1313	0.29555	0.40126	0.40126	grey
MADD	0.05862	-0.19194	-0.08802	-0.08126	0.02527	-0.15524	-0.06709	0.15096	-0.15096	0.44631	0.01191	0.25229	0.29069	0.74284	0.04262	0.38332	0.04874	0.04874	turquoise
PROB1	0.04424	-0.19032	-0.12131	-0.01639	0.06069	-0.1059	0.15447	-0.05098	0.05098	0.56559	0.01266	0.11397	0.83153	0.43035	0.16803	0.04367	0.50789	0.50789	grey
PADI3	0.09865	-0.05835	0.09541	0.09296	0.06471	0.06766	0.11894	-0.1387	0.1387	0.19924	0.44839	0.21448	0.22652	0.40039	0.37923	0.12126	0.07042	0.07042	grey
STEAP2	0.01654	-0.19623	0.10669	0.06054	0.04882	0.03045	0.06084	-0.03624	0.03624	0.83	0.0101	0.16486	0.4315	0.52603	0.69254	0.42928	0.63798	0.63798	turquoise
IRF2BP1	0.07932	-0.0761	-0.23793	-0.16037	0.25419	-0.23635	-0.12638	0.02042	-0.02042	0.30244	0.32251	0.00173	0.03614	0.00079	0.00186	0.09953	0.79097	0.79097	green
ASPH	0.05785	-0.00014	0.10948	0.06627	-0.14092	0.16225	0.20026	-0.25151	0.25151	0.45228	0.99852	0.15403	0.38911	0.06599	0.03398	0.00863	0.00091	0.00091	turquoise
TDRKH	0.05419	-0.12839	-0.06261	-0.14494	-0.03108	-0.07976	0.04352	-0.00339	0.00339	0.4815	0.09421	0.41594	0.05856	0.68657	0.29971	0.57196	0.96491	0.96491	turquoise
UBTD2	-0.06854	-0.17479	0.00712	0.10125	-0.12525	0.10288	0.00922	0.23214	-0.23214	0.37309	0.02222	0.92631	0.18761	0.10262	0.18058	0.90469	0.00225	0.00225	turquoise
ZNF404	-0.08926	-0.14801	-0.07593	-0.01089	0.1317	-0.0862	-0.07088	0.0917	-0.0917	0.24564	0.05336	0.3236	0.88756	0.08597	0.26226	0.35691	0.23291	0.23291	red
S100A14	0.06753	0.132	0.10532	0.01081	0.01243	0.23478	0.25969	-0.45833	0.45833	0.38018	0.08525	0.17038	0.88839	0.87184	0.002	0.0006	2.91E-10	2.91E-10	yellow
SEC23IP	-0.02546	-0.09605	0.05871	0.04316	-0.07112	0.12757	0.08814	0.03944	-0.03944	0.74095	0.2114	0.44564	0.57511	0.35526	0.09637	0.25163	0.60856	0.60856	turquoise
ZNF185	-0.01592	-0.05135	0.11962	0.05268	-0.15073	0.15381	0.23513	-0.27695	0.27695	0.83628	0.50475	0.11916	0.49377	0.04908	0.04459	0.00196	0.00025	0.00025	turquoise
IGFBP7	-0.09965	-0.12553	-0.00373	0.02188	-0.15825	0.14311	-0.0579	0.39026	-0.39026	0.19472	0.10184	0.96143	0.77642	0.03871	0.06186	0.45193	1.32E-07	1.32E-07	blue
VOPP1	-0.00533	-0.1467	0.01392	0.00637	-0.04642	-0.10481	-0.01398	0.01824	-0.01824	0.94486	0.05554	0.85664	0.93405	0.54658	0.17246	0.85603	0.81283	0.81283	turquoise
MRPL49	-0.0382	-0.1226	-0.0016	-0.00265	-0.12214	0.03982	0.07614	-0.11102	0.11102	0.6199	0.11016	0.98346	0.97253	0.11152	0.60505	0.32228	0.14829	0.14829	turquoise
MYB	0.05143	0.09609	0.16271	0.06801	0.02953	0.16875	0.19671	-0.15968	0.15968	0.50412	0.21124	0.03348	0.3768	0.70145	0.02736	0.00992	0.03696	0.03696	grey
OAS3	-0.00777	0.01111	0.12176	0.13308	-0.18035	0.1752	0.22068	-0.14134	0.14134	0.91968	0.88534	0.11265	0.0827	0.01825	0.0219	0.00373	0.0652	0.0652	tan
CFAP300	0.05778	-0.21108	-0.05649	0.04024	0.00907	-0.11789	0.00081	-0.02813	0.02813	0.45288	0.00558	0.46301	0.60126	0.9063	0.12461	0.99157	0.71498	0.71498	grey
DKK4	-0.0249	0.06224	0.16912	0.0147	-0.07602	0.14121	0.08772	-0.28207	0.28207	0.74645	0.41867	0.02701	0.8487	0.32305	0.06545	0.25393	0.00019	0.00019	grey
PIK3AP1	-0.01203	-0.06193	-0.05868	0.05396	-0.15541	0.13035	0.05185	0.15566	-0.15566	0.87589	0.42104	0.44585	0.48334	0.04239	0.08926	0.50063	0.04205	0.04205	grey
HLTF	-0.0662	-0.12643	-0.15185	-0.08422	-0.0701	-0.11756	0.00516	0.29012	-0.29012	0.38965	0.09941	0.04741	0.27346	0.36226	0.12568	0.94661	0.00012	0.00012	turquoise
DENND2A	-0.07015	-0.12428	-0.16285	-0.07292	0.06078	-0.26584	-0.09572	0.49206	-0.49206	0.36194	0.10532	0.03332	0.34324	0.42972	0.00044	0.21297	8.20E-12	8.20E-12	brown
STAT4	-0.0612	-0.10441	-0.0831	-0.0173	-0.09821	-0.10585	-0.13623	0.53766	-0.53766	0.42651	0.17415	0.27988	0.82234	0.20129	0.16825	0.07561	3.39E-14	3.39E-14	blue
CYP2S1	0.01425	0.09057	0.19285	0.06765	-0.02191	0.31174	0.1735	-0.36117	0.36117	0.85329	0.23875	0.0115	0.37934	0.77607	3.32E-05	0.02324	1.22E-06	1.22E-06	yellow
FXYD2	0.02256	-0.03625	-0.22165	-0.00401	0.13115	-0.14176	-0.1459	0.24609	-0.24609	0.76966	0.63784	0.00357	0.95851	0.0873	0.06439	0.05689	0.00118	0.00118	magenta
MORN4	0.07533	-0.16045	-0.223	-0.13269	0.0258	-0.35086	-0.08296	0.26628	-0.26628	0.32749	0.03605	0.00337	0.0836	0.7377	2.54E-06	0.28071	0.00043	0.00043	brown
FCN3	-0.09999	-0.0474	-0.05449	0.07214	-0.05936	-0.224	-0.048	0.42141	-0.42141	0.19318	0.53815	0.47902	0.34842	0.44055	0.00323	0.533	9.47E-09	9.47E-09	grey
C15orf62	0.10684	-0.06949	-0.00353	-0.01536	-0.03058	0.00967	0.13031	-0.34688	0.34688	0.16426	0.36648	0.9634	0.84196	0.69136	0.90013	0.08935	3.35E-06	3.35E-06	grey
STX7	-0.04648	-0.08615	0.0348	0.01285	-0.10081	0.13444	-0.00799	0.20137	-0.20137	0.54603	0.26255	0.65134	0.8675	0.18954	0.07958	0.91734	0.00827	0.00827	blue
SNRPA1	-0.03104	0.03195	-0.00441	-0.03982	-0.01494	0.05465	0.17685	-0.3599	0.3599	0.68697	0.67828	0.95435	0.60509	0.84623	0.47771	0.02067	1.33E-06	1.33E-06	pink
ZBTB24	-0.03101	-0.072	-0.05228	-0.05423	-0.129	0.00247	-0.01689	0.40066	-0.40066	0.6872	0.34937	0.49707	0.48113	0.09265	0.97443	0.82641	5.63E-08	5.63E-08	turquoise
UCKL1	0.10238	-0.03711	-0.07286	-0.07312	0.20932	-0.15492	0.01631	-0.28922	0.28922	0.18269	0.62985	0.3436	0.34188	0.006	0.04306	0.83234	0.00012	0.00012	red
DENND6A	-0.04352	-0.1056	-0.02976	-0.04846	-0.14125	0.09993	0.05651	0.08674	-0.08674	0.57197	0.16924	0.69925	0.52904	0.06536	0.19344	0.46286	0.2593	0.2593	turquoise
MED10	0.01149	-0.07378	-0.02464	0.01941	0.08257	-0.13086	0.02042	-0.14252	0.14252	0.8814	0.33753	0.74906	0.80109	0.28299	0.08801	0.79099	0.06296	0.06296	grey
GRHL2	0.07928	0.03533	0.08933	0.09481	-0.06883	0.24651	0.23501	-0.37624	0.37624	0.30269	0.64637	0.24526	0.21738	0.37106	0.00115	0.00197	3.95E-07	3.95E-07	yellow
WSB1	-0.08153	-0.16942	0.04095	0.06885	0.12276	0.03554	-0.02727	0.02736	-0.02736	0.28908	0.02674	0.59486	0.3709	0.10969	0.64445	0.72332	0.72238	0.72238	red
ACO2	-0.05137	0.03049	-0.03303	-0.00751	-0.01663	0.03696	-0.04823	0.0977	-0.0977	0.50463	0.69217	0.668	0.92237	0.82911	0.63125	0.53106	0.20361	0.20361	turquoise
OAS2	-0.02202	0.07888	0.17996	0.15091	-0.19292	0.22733	0.22458	-0.10564	0.10564	0.77496	0.30509	0.01851	0.04881	0.01147	0.00279	0.00315	0.16908	0.16908	tan
SIGLEC9	-0.10766	-0.00603	0.10046	0.07201	-0.2538	0.1561	0.11441	0.39242	-0.39242	0.16103	0.93758	0.19107	0.3493	0.00081	0.04147	0.13621	1.11E-07	1.11E-07	blue
DDX27	0.10352	-0.05785	0.02892	0.04984	-0.04138	0.06199	0.15193	-0.26174	0.26174	0.17784	0.4523	0.70729	0.5174	0.59104	0.42056	0.04729	0.00054	0.00054	turquoise
SEC62	-0.07488	-0.09024	-0.03428	-0.00551	-0.12095	0.06824	-0.02278	0.2738	-0.2738	0.33034	0.24046	0.65621	0.94296	0.11507	0.37514	0.7674	0.00029	0.00029	turquoise
FBXL20	-0.01266	-0.1974	-0.15501	-0.0064	0.09016	-0.12739	-0.07445	0.11496	-0.11496	0.86948	0.00965	0.04292	0.93376	0.24091	0.09685	0.33319	0.13434	0.13434	turquoise
CAPN8	0.06915	0.0779	0.17575	0.03559	0.12189	0.32277	0.15719	-0.44565	0.44565	0.36884	0.3112	0.02149	0.64399	0.11225	1.67E-05	0.04005	1.01E-09	1.01E-09	yellow
MVD	0.05965	0.09206	-0.03702	-0.08326	0.1451	0.09851	0.14303	-0.41809	0.41809	0.43834	0.23108	0.63075	0.27898	0.05829	0.19989	0.062	1.27E-08	1.27E-08	green
RHCG	0.05414	-0.0586	0.04912	-0.01924	-0.1313	-0.03483	0.1532	-0.12505	0.12505	0.48186	0.44646	0.52349	0.80274	0.08694	0.6511	0.04545	0.10317	0.10317	grey
DDX49	0.10677	0.06563	-0.19845	-0.09206	0.09988	-0.08665	0.10632	-0.23613	0.23613	0.16456	0.39372	0.00927	0.23108	0.19366	0.2598	0.16633	0.00188	0.00188	green
NMU	0.08447	0.05584	0.13532	0.04637	-0.01639	0.15603	0.23517	-0.40364	0.40364	0.27203	0.46816	0.07761	0.547	0.83146	0.04156	0.00196	4.39E-08	4.39E-08	yellow
SERP1	0.06973	0.02433	-0.00095	-0.00344	-0.11815	0.17441	0.1136	-0.12217	0.12217	0.36483	0.75214	0.99016	0.96435	0.12378	0.02252	0.13905	0.11141	0.11141	turquoise
JSRP1	0.00502	-0.05818	-0.11029	-0.04934	0.18239	-0.08515	-0.04959	-0.05963	0.05963	0.94807	0.44969	0.151	0.52162	0.01696	0.26815	0.51948	0.43852	0.43852	grey
CAVIN1	-0.12516	-0.0688	0.03926	0.10267	-0.1591	0.11481	0.02406	0.27952	-0.27952	0.10287	0.37125	0.61014	0.18147	0.03766	0.13483	0.75478	0.00021	0.00021	black
STRBP	-0.01654	-0.03446	-0.06049	-0.04298	-0.10271	-0.00976	-0.07239	0.02841	-0.02841	0.82999	0.65454	0.43191	0.57672	0.1813	0.89921	0.34674	0.71225	0.71225	turquoise
IQANK1	0.06186	0.03223	0.00609	-0.01873	0.03131	0.05652	0.21182	-0.4765	0.4765	0.42151	0.67563	0.93703	0.80791	0.68433	0.46282	0.00542	4.46E-11	4.46E-11	green
CFAP36	0.07699	-0.18574	-0.18345	-0.10533	0.13404	-0.14267	-0.11879	0.05273	-0.05273	0.31687	0.015	0.01632	0.17037	0.08049	0.06268	0.12176	0.49333	0.49333	turquoise
TOR1B	0.00744	-0.20208	0.01526	0.09384	-0.06884	0.01407	0.02162	0.03088	-0.03088	0.9231	0.00803	0.84299	0.22214	0.37096	0.85509	0.77898	0.68849	0.68849	turquoise
MYOF	-0.0263	-0.06737	0.15397	0.11476	-0.20617	0.27691	0.26923	-0.14653	0.14653	0.73274	0.38127	0.04436	0.13501	0.00682	0.00025	0.00037	0.05582	0.05582	turquoise
LYPD6B	0.01588	0.00684	-0.06846	0.08989	0.05909	0.07055	0.06025	-0.15203	0.15203	0.83663	0.92925	0.37362	0.24234	0.44263	0.3592	0.43377	0.04715	0.04715	grey
RBM4B	0.01296	-0.17556	-0.15427	-0.07737	-0.00855	-0.09567	-0.05852	0.18092	-0.18092	0.86636	0.02163	0.04394	0.31448	0.91163	0.21323	0.44708	0.01788	0.01788	turquoise
AMIGO1	-0.05676	-0.10642	-0.20022	-0.08969	0.06362	-0.13119	-0.17873	0.4587	-0.4587	0.46085	0.16595	0.00865	0.24337	0.40841	0.0872	0.01934	2.80E-10	2.80E-10	brown
RNF141	0.05905	-0.04783	0.03587	0.0167	-0.05257	0.07959	0.12905	-0.13754	0.13754	0.44295	0.53445	0.64141	0.82836	0.49472	0.3008	0.09253	0.07282	0.07282	turquoise
GNG5	-0.01174	-0.0532	0.05623	-0.02224	-0.00637	0.19022	0.16103	-0.38426	0.38426	0.87887	0.4895	0.46513	0.77276	0.93406	0.0127	0.03537	2.12E-07	2.12E-07	yellow
GSTA2	-0.01065	0.01991	0.07294	0.10518	-0.04593	0.11708	-0.05594	0.0708	-0.0708	0.89009	0.79606	0.34311	0.17098	0.55079	0.12724	0.46737	0.35746	0.35746	magenta
WASHC1	0.05279	-0.06473	-0.0435	-0.12521	0.08075	-0.12667	-0.04949	-0.21574	0.21574	0.49286	0.40029	0.57211	0.10274	0.29376	0.09875	0.52032	0.0046	0.0046	grey
LGALS4	0.09022	0.043	0.14059	0.11013	0.05803	0.28722	0.09624	-0.25169	0.25169	0.2406	0.57653	0.06665	0.15158	0.45092	0.00014	0.21049	0.0009	0.0009	yellow
ETV1	-0.0206	-0.1125	-0.16182	-0.12278	-0.00641	-0.08165	-0.04644	0.29677	-0.29677	0.78917	0.14291	0.03447	0.10963	0.93367	0.28837	0.54643	8.10E-05	8.10E-05	turquoise
CHCHD6	0.0683	0.07248	-0.2589	-0.08754	0.08729	-0.19521	0.03491	-0.07254	0.07254	0.37476	0.34612	0.00063	0.25492	0.25628	0.01051	0.65032	0.34577	0.34577	grey
MAGEH1	-0.12743	-0.11623	-0.21297	-0.05903	0.01463	-0.23728	-0.20785	0.61394	-0.61394	0.09674	0.04041	0.00516	0.4431	0.84937	0.00178	0.00637	4.30E-19	4.30E-19	brown
FGFRL1	-0.03106	0.07281	0.09304	0.0642	-0.10878	0.27625	0.22861	-0.33099	0.33099	0.68676	0.34398	0.22617	0.40417	0.15671	0.00025	0.00263	9.79E-06	9.79E-06	yellow
NAT9	0.05544	-0.10803	-0.07898	-0.10569	0.18567	-0.10833	0.02392	-0.2202	0.2202	0.47138	0.15958	0.30449	0.16887	0.01505	0.15845	0.75611	0.0038	0.0038	grey
ZKSCAN1	0.06451	-0.12191	-0.13621	-0.08388	0.14577	-0.13647	-0.12433	0.14528	-0.14528	0.40189	0.1122	0.07568	0.2754	0.05711	0.0751	0.1052	0.05798	0.05798	turquoise
TMEM42	0.00245	-0.08485	-0.17115	-0.1518	0.0195	-0.10574	0.0809	-0.15726	0.15726	0.97461	0.26984	0.02521	0.04748	0.80018	0.16867	0.29287	0.03996	0.03996	grey
YY2	-0.09022	-0.21459	-0.02481	-0.01644	-0.17387	0.00035	0.03548	0.26363	-0.26363	0.2406	0.00482	0.74737	0.83103	0.02295	0.99638	0.645	0.00049	0.00049	turquoise
TBK1	-0.07101	-0.18434	-0.04734	0.02415	0.00987	-0.04753	-0.06374	0.12098	-0.12098	0.35607	0.01579	0.53864	0.75392	0.89801	0.53703	0.40756	0.11497	0.11497	turquoise
PLA2G2D	-0.07588	0.03581	0.05195	0.00735	-0.10024	0.05346	-0.00653	0.36961	-0.36961	0.32396	0.64191	0.49978	0.92398	0.19206	0.48737	0.93245	6.52E-07	6.52E-07	blue
REM1	-0.12242	-0.03127	0.01982	0.0027	-0.08762	0.02079	-0.11093	0.50524	-0.50524	0.11069	0.68477	0.7969	0.97207	0.25445	0.78724	0.1486	1.82E-12	1.82E-12	blue
OSBPL3	-0.00577	-0.01522	0.13247	0.10143	-0.13056	0.25025	0.24048	-0.3438	0.3438	0.94029	0.84338	0.08414	0.18681	0.08874	0.00096	0.00153	4.15E-06	4.15E-06	yellow
IQSEC2	-0.0705	-0.16109	0.02907	0.07784	-0.02083	0.08401	0.03893	0.04773	-0.04773	0.35951	0.03531	0.70584	0.31158	0.78687	0.27463	0.61316	0.53534	0.53534	turquoise
FAM193A	-0.06798	-0.20088	-0.14021	-0.01443	0.02469	-0.08325	-0.12875	0.25476	-0.25476	0.37697	0.00843	0.06739	0.85137	0.74855	0.27902	0.09329	0.00077	0.00077	turquoise
RAB28	-0.04289	-0.12232	-0.13109	-0.05234	-0.00216	-0.10286	-0.06812	0.27057	-0.27057	0.57751	0.11099	0.08744	0.49654	0.97759	0.18065	0.376	0.00034	0.00034	turquoise

AIMP2	0.00545	0.01023	-0.03181	-0.02757	-0.03253	0.04601	0.17968	-0.2664	0.2664	0.94361	0.89433	0.67962	0.72037	0.67276	0.55011	0.0187	0.00043	0.00043	turquoise
RAB43	-0.05062	-0.01614	0.00938	0.01466	-0.05211	0.13868	0.15609	-0.10761	0.10761	0.51082	0.83404	0.90309	0.84907	0.4985	0.07046	0.04149	0.16121	0.16121	grey
HMGN2	-0.0449	-0.117	-0.09331	-0.06582	0.03197	-0.07435	-0.09656	0.19873	-0.19873	0.55983	0.12752	0.22481	0.3924	0.67804	0.33379	0.20896	0.00917	0.00917	turquoise
ZNF682	-0.03031	-0.07995	-0.16648	-0.05109	0.04774	-0.11452	-0.1812	0.24881	-0.24881	0.69389	0.29859	0.02953	0.50691	0.53523	0.13585	0.0177	0.00103	0.00103	grey
EPB41L1	0.10391	-0.07989	0.06336	0.09114	-0.03113	0.12957	0.14866	-0.28656	0.28656	0.17623	0.29896	0.41038	0.23582	0.68605	0.0912	0.05231	0.00014	0.00014	turquoise
TSPAN15	0.02676	0.01139	0.10448	0.0868	0.04478	0.24209	0.18492	-0.48599	0.48599	0.72827	0.88245	0.17386	0.25896	0.56083	0.00142	0.01546	1.60E-11	1.60E-11	yellow
CEBPZOS	-0.00714	-0.11027	-0.10543	-0.07285	0.03807	0.02335	0.07139	-0.10756	0.10756	0.92617	0.15107	0.16994	0.3437	0.62109	0.76174	0.35346	0.16144	0.16144	turquoise
CES1	0.03068	-0.03554	-0.00519	0.0868	0.00915	0.09003	0.03082	0.20138	-0.20138	0.69034	0.64442	0.94625	0.25896	0.9054	0.24159	0.68904	0.00826	0.00826	grey
SMAD2	-0.09114	-0.16024	-0.08391	0.02721	-0.06201	-0.01218	-0.02974	0.33472	-0.33472	0.23578	0.03629	0.2752	0.72384	0.42043	0.87437	0.69938	7.65E-06	7.65E-06	turquoise
GRIK5	-0.01906	-0.13019	-0.29954	-0.12196	0.12931	-0.34178	-0.26952	0.43742	-0.43742	0.80456	0.08966	6.89E-05	0.11204	0.09186	4.76E-06	0.00036	2.20E-09	2.20E-09	brown
LY6H	0.15585	-0.07117	-0.13387	-0.04914	0.17804	-0.363	-0.17815	0.26117	-0.26117	0.04179	0.35496	0.08087	0.52328	0.01982	1.06E-06	0.01974	0.00056	0.00056	brown
KLHL7	-0.11773	-0.17369	-0.05102	0.05515	-0.15465	0.00582	-0.00113	0.21695	-0.21695	0.12514	0.02309	0.50754	0.47375	0.04342	0.93975	0.98833	0.00437	0.00437	turquoise
PDE8B	0.03301	-0.13758	-0.05964	0.09134	-0.0425	-0.1827	-0.14679	0.45553	-0.45553	0.66818	0.07274	0.43843	0.23476	0.58099	0.01677	0.05538	3.84E-10	3.84E-10	brown
MCTS2P	-0.01455	-0.17088	-0.03705	-0.09793	0.00103	-0.04146	0.01332	0.17117	-0.17117	0.85016	0.02544	0.63048	0.20256	0.98934	0.59031	0.86269	0.02519	0.02519	turquoise
IRX3	-0.05033	-0.0589	-0.05594	0.0953	-0.07225	-0.02269	0.14959	-0.20588	0.20588	0.51326	0.4441	0.4674	0.21501	0.34767	0.76828	0.05084	0.0069	0.0069	grey
PGR	0.00488	-0.09185	-0.11077	-0.03955	-0.07273	-0.171	-0.13379	0.57742	-0.57742	0.94944	0.23216	0.1492	0.60756	0.34449	0.02534	0.08107	1.37E-16	1.37E-16	brown
DBP	0.15203	0.05639	-0.11449	-0.10073	0.08545	-0.09145	-0.00811	-0.17803	0.17803	0.04714	0.4638	0.13594	0.18989	0.26648	0.23421	0.91619	0.01982	0.01982	grey
MSMO1	0.00715	0.05013	0.00283	-0.04154	0.05222	0.14757	0.02811	-0.32925	0.32925	0.92606	0.51494	0.97069	0.58961	0.49759	0.05409	0.71514	1.10E-05	1.10E-05	yellow
FGF7	-0.0841	-0.05676	0.08535	0.05568	-0.14027	0.13528	-0.02633	0.51	-0.51	0.27412	0.46089	0.26703	0.46948	0.06728	0.0777	0.73248	1.04E-12	1.04E-12	blue
ZC3H6	-0.02325	-0.1223	-0.09784	-0.05056	-0.06606	-0.08203	-0.07969	0.36359	-0.36359	0.76281	0.11103	0.20298	0.51136	0.39061	0.28617	0.30018	1.02E-06	1.02E-06	turquoise
KCNK3	-0.0624	-0.06974	-0.20531	-0.08975	0.06545	-0.23848	-0.27548	0.48214	-0.48214	0.41745	0.36471	0.00706	0.24304	0.39503	0.00168	0.00027	2.44E-11	2.44E-11	brown
DENND2D	0.03337	-0.0965	0.0616	0.05769	-0.05043	0.17671	0.02008	-0.14858	0.14858	0.66482	0.20927	0.42349	0.45357	0.51244	0.02077	0.79438	0.05244	0.05244	turquoise
RASGRF1	-0.04539	-0.14714	-0.17228	-0.11249	-0.03658	-0.20632	-0.06627	0.36164	-0.36164	0.5555	0.05481	0.02425	0.14295	0.63478	0.00678	0.38917	1.17E-06	1.17E-06	brown
RGN	-0.01359	-0.06406	-0.16886	0.02758	0.02763	-0.16726	-0.16955	0.37309	-0.37309	0.85997	0.40516	0.02725	0.72031	0.71982	0.02877	0.02663	5.02E-07	5.02E-07	magenta
DUSP1	-0.10675	-0.13262	0.00387	0.01596	-0.05558	0.02779	-0.03905	0.40912	-0.40912	0.16463	0.08379	0.95992	0.83584	0.47029	0.71826	0.61209	2.76E-08	2.76E-08	blue
ARHGAP3	-0.01828	-0.1302	-0.14711	-0.00056	-0.09073	-0.13881	-0.01433	0.30217	-0.30217	0.81244	0.08963	0.05485	0.99423	0.23791	0.07019	0.85241	5.90E-05	5.90E-05	turquoise
TMEM147	0.13191	-0.00752	-0.09098	-0.09608	0.03701	-0.19791	0.0501	-0.22278	0.22278	0.08546	0.92222	0.23663	0.21128	0.6308	0.00946	0.51518	0.0034	0.0034	grey
ZNF628	0.07388	0.053	-0.13211	-0.08746	0.13157	-0.09005	0.05843	-0.18832	0.18832	0.33692	0.49119	0.08498	0.25536	0.08629	0.24146	0.44777	0.01364	0.01364	green
RIC1	-0.08173	-0.13049	0.04597	0.06332	-0.21799	0.12164	0.08475	0.35326	-0.35326	0.28791	0.08892	0.55051	0.41067	0.00418	0.11298	0.27042	2.15E-06	2.15E-06	blue
F5	0.1108	-0.09879	-0.03922	-0.03297	0.1461	-0.00679	-0.06336	-0.03749	0.03749	0.14909	0.19863	0.61053	0.66855	0.05655	0.92979	0.41034	0.62635	0.62635	grey
CPA1	0.02314	0.05093	0.06189	0.13988	-0.11743	0.09208	-0.07375	0.12978	-0.12978	0.76383	0.50823	0.42133	0.06804	0.1261	0.23098	0.33774	0.09068	0.09068	magenta
ZNF626	-0.14013	-0.12579	-0.15048	-0.01451	-0.0989	-0.00883	-0.09585	0.47244	-0.47244	0.06755	0.10115	0.04947	0.85061	0.19811	0.90877	0.21236	6.85E-11	6.85E-11	blue
RBM15	-0.09683	-0.10035	0.04857	0.02295	-0.12638	0.16992	0.01051	-0.09651	0.09651	0.20772	0.19157	0.5281	0.76569	0.09954	0.02629	0.89146	0.20922	0.20922	turquoise
SLCO2A1	-0.13059	-0.14801	-0.04251	0.02725	-0.08136	0.04643	-0.11933	0.4133	-0.4133	0.08868	0.05337	0.58087	0.72348	0.29014	0.54647	0.12007	1.93E-08	1.93E-08	blue
TBC1D9B	-0.03756	-0.17615	-0.04589	0.00283	-0.06591	0.00381	0.00674	0.15615	-0.15615	0.62578	0.02119	0.55115	0.97068	0.39175	0.96052	0.93033	0.04141	0.04141	turquoise
NIPSNAP3	-0.05111	-0.14814	-0.15859	-0.07363	-0.04463	-0.1301	-0.20433	0.52672	-0.52672	0.50674	0.05316	0.03829	0.33854	0.56214	0.08988	0.00735	1.36E-13	1.36E-13	brown
SCGB2A1	-0.06806	-0.03992	-0.19824	-0.14438	0.1638	-0.12966	-0.16879	0.19312	-0.19312	0.37644	0.60421	0.00934	0.05955	0.03229	0.09098	0.02733	0.01138	0.01138	brown
SMCO2	0.01736	-0.01735	0.04304	0.00745	-0.08156	0.06916	0.19707	-0.39965	0.39965	0.82174	0.82178	0.5762	0.92291	0.2889	0.36873	0.00978	6.12E-08	6.12E-08	grey
ALPL	-0.09888	0.00085	-0.05481	-0.00599	-0.09351	-0.04179	0.00189	0.37023	-0.37023	0.19821	0.99117	0.47647	0.938	0.22378	0.58732	0.98044	6.22E-07	6.22E-07	blue
FAM155B	-0.07483	-0.06977	-0.28432	-0.10852	0.20564	-0.3515	-0.32429	0.24913	-0.24913	0.33069	0.36451	0.00016	0.1577	0.00697	2.43E-06	1.51E-05	0.00102	0.00102	brown
ADAR	0.00202	-0.08521	-0.04721	-0.03198	-0.13938	0.03914	0.10608	0.08876	-0.08876	0.97906	0.26783	0.53974	0.67795	0.06903	0.61131	0.1673	0.24832	0.24832	turquoise
MRPL30	0.07385	0.03118	-0.03673	0.0102	-0.07423	0.09028	0.18706	-0.12073	0.12073	0.3371	0.68564	0.63343	0.89461	0.33458	0.24029	0.01429	0.11572	0.11572	turquoise
ZNF7	0.03612	-0.0765	-0.05702	-0.05712	-0.01462	-0.05245	0.09864	-0.18117	0.18117	0.63902	0.31997	0.45882	0.45805	0.84947	0.49567	0.19932	0.01772	0.01772	turquoise
HBEGF	-0.0639	-0.06349	0.0602	-0.01309	-0.00658	0.21671	0.15359	0.02788	-0.02788	0.40634	0.4094	0.43414	0.86508	0.93194	0.00441	0.0449	0.71738	0.71738	grey
PRAP1	-0.03733	0.08459	-0.04194	0.07532	0.13731	0.1083	0.08779	-0.19006	0.19006	0.62788	0.27133	0.58598	0.32755	0.07331	0.15854	0.25353	0.01278	0.01278	grey
IFT22	0.04096	-0.17163	-0.13501	0.0356	0.10164	-0.18664	-0.05613	0.04842	-0.04842	0.59481	0.02479	0.07831	0.6439	0.18588	0.01451	0.46587	0.52939	0.52939	brown
SETD1A	-0.00342	-0.06146	-0.10042	0.02137	-0.00447	-0.02268	0.04711	-0.03616	0.03616	0.96455	0.42458	0.19127	0.7815	0.95377	0.76837	0.54061	0.63864	0.63864	turquoise
TRPV4	-0.03787	-0.11543	0.06765	0.05175	-0.09194	0.17502	0.14148	-0.14254	0.14254	0.62291	0.13273	0.37932	0.50144	0.23173	0.02205	0.06493	0.06291	0.06291	yellow
PSMB2	-0.06104	-0.11267	-0.02082	-0.03702	-0.03967	-0.00883	0.08102	-0.18929	0.18929	0.42776	0.1423	0.78692	0.6307	0.60649	0.90878	0.29214	0.01315	0.01315	turquoise
OSCAR	-0.09747	0.04245	0.14447	0.15198	-0.15099	0.2032	0.15926	0.07109	-0.07109	0.20472	0.58142	0.0594	0.04721	0.04869	0.00769	0.03747	0.3555	0.3555	blue
TSPAN13	0.07365	-0.08275	-0.03235	0.02315	0.18884	-0.02169	-0.09614	-0.15201	0.15201	0.3384	0.28195	0.67444	0.76375	0.01337	0.77827	0.21096	0.04717	0.04717	grey
FAM78B	-0.05803	-0.10108	-0.13508	-0.10931	-0.12149	-0.04759	-0.06949	0.45037	-0.45037	0.45094	0.18833	0.07815	0.15469	0.11344	0.53653	0.36643	6.38E-10	6.38E-10	turquoise
TFAP2C	0.00988	-0.05829	0.10653	0.14519	-0.10697	0.1024	0.13703	-0.09449	0.09449	0.89795	0.44889	0.16552	0.05813	0.16376	0.1826	0.0739	0.21898	0.21898	grey
SMIM24	-0.02823	-0.04524	-0.20507	-0.11643	0.2698	-0.05058	-0.17248	-0.07629	0.07629	0.71394	0.55688	0.00713	0.12938	0.00036	0.51119	0.02408	0.32132	0.32132	grey
RHBDL1	0.08627	0.07329	-0.09479	-0.08817	0.27034	-0.2128	-0.03784	-0.26834	0.26834	0.2619	0.34076	0.21748	0.25148	0.00035	0.0052	0.62317	0.00039	0.00039	green
CREB1	-0.04581	-0.12486	-0.01389	0.03964	-0.14913	0.08075	0.04676	0.17644	-0.17644	0.5519	0.1037	0.85693	0.6067	0.05157	0.29374	0.5436	0.02097	0.02097	turquoise
PRDX1	-0.06699	0.02279	0.0832	0.04195	-0.09885	0.18903	0.20102	-0.23389	0.23389	0.38402	0.76736	0.27932	0.58591	0.19836	0.01328	0.00838	0.00208	0.00208	turquoise
CDR2	-0.01608	-0.15189	0.06285	0.06969	-0.12586	0.21534	0.02906	0.03752	-0.03752	0.83469	0.04735	0.41412	0.36507	0.10094	0.00468	0.70592	0.62613	0.62613	turquoise
PASK	-0.06056	-0.04318	-0.06763	-0.06759	0.11505	-0.06571	-0.09703	0.12001	-0.12001	0.43141	0.57497	0.37946	0.37971	0.13402	0.39316	0.20678	0.11795	0.11795	red
ZNF696	0.05245	-0.03316	-0.17097	-0.08541	0.05361	-0.17216	0.03683	-0.08035	0.08035	0.4957	0.66683	0.02536	0.26669	0.48617	0.02434	0.63247	0.29618	0.29618	green
ZNF232	-0.05434	-0.14322	-0.22469	-0.14624	0.09069	-0.1797	-0.12895	0.14344	-0.14344	0.48028	0.06166	0.00313	0.05631	0.23815	0.01868	0.09278	0.06126	0.06126	grey
C1orf198	-0.00706	-0.03771	-0.00778	0.01006	-0.15592	0.2007	0.10268	0.01547	-0.01547	0.92697	0.6244	0.91956	0.89607	0.0417	0.00849	0.18141	0.84085	0.84085	turquoise
STMN1	-0.07672	-0.13253	-0.09261	-0.09263	-0.03747	-0.09351	-0.01963	-0.05145	0.05145	0.31861	0.08399	0.2283	0.2282	0.62661	0.22378	0.79887	0.50397	0.50397	grey
PIDD1	-0.02369	-0.04133	-0.11602	-0.08584	0.22493	-0.125	0.02984	-0.25227	0.25227	0.7584	0.59144	0.13075	0.2643	0.0031	0.10333	0.69847	0.00087	0.00087	red
MRPS30	0.08743	-0.00625	-0.07234	-0.0976	-0.05751	-0.02997	0.13579	-0.16593	0.16593	0.25549	0.93535	0.3471	0.2041	0.45501	0.69721	0.07657	0.03009	0.03009	turquoise
TOE1	-0.09827	-0.03253	0.00822	-0.05659	-0.00931	0.06289	-0.0158	0.06736	-0.06736	0.20097	0.67272	0.915	0.46224	0.90383	0.41381	0.83753	0.38136	0.38136	turquoise
PCYT2	0.03962	0.08248	-0.00948	-0.07091	0.12762	0.02072	0.16236	-0.45354	0.45354	0.6069	0.28349	0.90204	0.35675	0.09622	0.78793	0.03387	4.68E-10	4.68E-10	grey
SLC35D1	-0.00349	-0.12691	0.11484	0.05953	-0.00197	0.14443	0.10345	0.1004	-0.1004	0.96385	0.09811	0.13473	0.43925	0.97964	0.05946	0.17816	0.19137	0.19137	turquoise
ABCF1	0.00997	-0.03928	0.04214	0.02531	-0.06149	0.05141	0.14246	0.0305	-0.0305	0.89702	0.61002	0.58417	0.7425	0.42429	0.50425	0.06307	0.69212	0.69212	turquoise
PRPF4B	-0.04652	-0.07718	-0.02247	-0.00351	-0.01465	0.08401	0.01265	0.26895	-0.26895	0.5457	0.31572	0.77054	0.96366	0.84919	0.27462	0.86954	0.00038	0.00038	turquoise
MKLN1	-0.01388	-0.16144	0.03952	0.05794	-0.12002	0.10405	0.05081	0.15327	-0.15327	0.85704	0.03491	0.60781	0.4516	0.1179	0.17562	0.5093	0.04535	0.04535	turquoise
SETD3	0.01515	-0.07603	-0.01786	0.07203	-0.02511	0.04056	-0.01592	-0.01647	0.01647	0.84408	0.323	0.81665	0.34918	0.74444	0.59838	0.83628	0.83067	0.83067	turquoise
PABPC4	0.02995	0.06849	0.08825	0.07006	-0.231	0.17312	0.02484	0.12345	-0.12345	0.69739	0.37341	0.25103	0.36251	0.00237	0.02355	0.74713	0.10771	0.10771	grey
KCNMB4	0.02688	-0.17458	-0.04152	0.07419	-0.15405	-0.11122	0.02275	0.13442	-0.13442	0.72709	0.02238	0.58978	0.33484	0.04425	0.14756	0.76774	0.07963	0.07963	black
ENY2	-0.05142	-0.11012	-0.0857	0.00248	-0.09204	-0.12326	0.04528	-0.03224	0.03224	0.50415	0.15163	0.26505	0.97428	0.23118	0.10824	0.55646	0.67552	0.67552	turquoise
MTIF3	0.1124	0.00939	-0.16762	-0.14591	0.11913	-0.1105	-0.02837	-0.16084	0.16084	0.14328	0.90297	0.02843	0.05687	0.12069	0.15021	0.71261	0.03559	0.03559	grey
CLYBL	0.05414	0.14017	-0.07401	-0.10094	0.09526	-0.05558	-0.0014	-0.12162	0.12162	0.48185	0.06747	0.33603	0.18896	0.21522	0.47025	0.98551	0.11306	0.11306	grey
TBL2	0.01899	-0.12097	0.01011	0.00645	0.02929	-0.01715	0.04546	-0.26977	0.26977	0.80529	0.11502	0.89554	0.93322	0.70371	0.8238	0.55488	0.00036	0.00036	grey
PARM1	0.06746	-0.1518	-0.09488	-0.07588	0.15682	-0.22985	-0.27889	0.35064	-0.35064	0.38066	0.04748	0.21706	0.3239	0.04053	0.00249	0.00022	2.58E-06	2.58E-06	brown
SLC5A6	0.00751	0.00287	0.03404	0.05073	-0.02337	0.03127	0.24586	-0.1212	0.1212	0.92238	0.9703	0.65852	0.50991	0.76162	0.68477	0.00119	0.11432	0.11432	turquoise
CTNND2	0.01879	-0.09611	-0.15063	0.07529	0.02473	-0.20055	-0.16581	0.38731	-0.38731	0.80727	0.21111	0.04923	0.32774	0.74817	0.00854	0.03021	1.66E-07	1.66E-07	magenta
GCSH	0.03032	-0.05328	-0.04579	-0.02437	-0.07099	-0.03116	0.18281	-0.1685	0.1685	0.69379	0.48891	0.55201	0.75168	0.35619	0.68577	0.0167	0.0276	0.0276	turquoise
ZFAND5	-0.09174	-0.19751	-0.06857	0.03766	-0.10714	-0.03628	-0.13833	0.26644	-0.26644	0.23274	0.00962	0.37288	0.62481	0.16307	0.63756	0.07118	0.00043	0.00043	turquoise
TMEM165	-0.06415	-0.09009	0.06787	0.04342	0.05997	0.08894	0.11152	-0.14622	0.14622	0.40454	0.24127	0.37779	0.57287	0.4359	0.24737	0.14647	0.05634	0.05634	turquoise
NEO1	-0.01773	-0.17242	-0.13648	-0.03688	-0.01525	-0.00035	-0.13298	0.26949	-0.26949	0.818	0.02413	0.07508	0.63199	0.84303	0.99639	0.08294	0.00036	0.00036	turquoise
ZNF776	-0.00318	-0.16577	-0.13219	0.00263	-0.04257	-0.0381	-0.11055	0.22631	-0.22631	0.96705	0.03025	0.08479	0.97276	0.5804	0.62078	0.15004	0.00292	0.00292	turquoise
TMEM230	-0.03567	-0.0608	-0.06983	0.00484	0.01848	-0.11238	-0.05295	0.15062	-0.15062	0.64323	0.42958	0.36409	0.94993	0.81036	0.14337	0.49158	0.04925	0.04925	turquoise
ACOT2	0.07053	-0.09017	-0.0154	0.04736	0.07487	0.01934	-0.02443	0.01264	-0.01264	0.35934	0.24083	0.84153	0.53851	0.33043	0.80179	0.7511	0.86969	0.86969	turquoise
ZDHHC17	-0.04846	-0.17357	-0.07822	0.00193	0.03047	-0.15314	-0.17117	0.38728	-0.38728	0.52909	0.02318	0.30917	0.97997	0.69241	0.04553	0.02519	1.67E-07	1.67E-07	turquoise
ACSM3	0.04646	0.00829	-0.06002	0.0969	-0.05206	0.10549	-0.00362	0.0571	-0.0571	0.54624	0.91429	0.43553	0.20739	0.49889	0.16971	0.96248	0.45821	0.45821	magenta
ZMYM5	0.06185	-0.10722	0.00568	-0.03722	-0.00625	0.08552	-0.05321	-0.01031	0.01031	0.42163	0.16275	0.94121	0.6289	0.93531	0.26606	0.48944	0.89356	0.89356	turquoise
SLC26A9	0.03829	0.14861	0.1099	-0.02585	0.11352	0.1472	0.06791	-0.27833	0.27833	0.61908	0.0524	0.15245	0.73712	0.1393	0.0547	0.37748	0.00023	0.00023	grey
PDGFRA	-0.06801	-0.10468	0.03838	0.08393	-0.22604	0.1959	0.10244	0.41597	-0.41597	0.37677	0.17302	0.61819	0.27512	0.00295	0.01023	0.18245	1.53E-08	1.53E-08	blue
RABEP2	0.08483	0.04174	-0.07394	-0.07714	0.18596	-0.0389	0.0318	-0.19765	0.19765	0.26998	0.58781	0.33652	0.31592	0.01488	0.61342	0.67973	0.00956	0.00956	green
AGL	-0.10763	-0.08887	0.01426	0.04897	-0.1435	0.07726	-0.00114	0.25428	-0.25428	0.16115	0.24773	0.85314	0.52478	0.06115	0.31518	0.9882	0.00079	0.00079	turquoise
ABCA6	-0.04669	-0.12475	0.03006	0.04766	-0.21905	0.12449	-0.01122	0.54061	-0.54061	0.54423	0.10401	0.69636	0.53589	0.004	0.10474	0.8842	2.31E-14	2.31E-14	blue
ABHD2	-0.02447	-0.04193	0.05154	0.02401	-0.05554	0.19417	0.03622	-0.0998	0.0998	0.75071	0.58609	0.50321	0.7553	0.47058	0.01093	0.63814	0.19402	0.19402	turquoise
MTMR3	-0.04868	-0.18422	-0.18143	-0.07619	0.02674	-0.23429	-0.15512	0.30939	-0.30939	0.52722	0.01586	0.01756	0.32194	0.72846	0.00204	0.04278	3.83E-05	3.83E-05	turquoise
H1F0	0.05703	0.01539	0.01553	0.00613	-0.06681	0.07512	0.19755	-0.41008	0.41008	0.45873	0.8416	0.84021	0.93658	0.38528	0.32883	0.0096	2.55E-08	2.55E-08	yellow
SLC25A1	0.06841	-0.0126	-0.15431	-0.10371	0.20868	-0.14927	0.00715	-0.37849	0.37849	0.374	0.87004	0.04389	0.17707	0.00616	0.05134	0.92601	3.32E-07	3.32E-07	green
GRAMD4	-0.04765	-0.05001	-0.09992	-0.01285	0.17311	-0.13892	-0.15504	0.02062	-0.02062	0.53602	0.51593	0.19349	0.86749	0.02356	0.06998	0.04289	0.78897	0.78897	grey
CCN1	-0.0716	-0.09361	0.01929	0.00225	-0.15385	0.07739	0.00089	0.38875	-0.38875	0.35205	0.22328	0.80223	0.97672	0.04453	0.31435	0.99083	1.49E-07	1.49E-07	blue
LAMC3	-0.05575	0.04293	-0.08589	0.00035	-0.09287	0.00101	0.08559	0.30083	-0.30083	0.46894	0.5772	0.26398	0.9964	0.22701	0.98959	0.26569	6.39E-05	6.39E-05	grey
TMEM178	-0.03247	-0.16121	-0.15821	-0.13276	0.09478	-0.29496	-0.2122	0.40581	-0.40581	0.67335	0.03516	0.03876	0.08345	0.21756	8.99E-05	0.00533	3.66E-08	3.66E-08	brown
SLC3A2	0.09228	-0.12879	-0.07471	-0.03178	0.09764	-0.17298	-0.07622	-0.01703	0.01703	0.22996	0.09319	0.33148	0.67987	0.20389	0.02367	0.32174	0.82503	0.82503	grey
TP53I3	-0.03023	-0.01522	0.07004	0.09844	-0.06502	0.18148	0.20622	-0.27413	0.27413	0.69465	0.84337	0.36265	0.20022	0.39815	0.01752	0.00681	0.00029	0.00029	yellow
COL4A1	-0.09885	-0.12497	-0.00661	0.02833	-0.21326	0.1091	0.00253	0.25447	-0.25447	0.19834	0.10339	0.93165	0.71304	0.0051	0.15549	0.97376	0.00078	0.00078	black
CDYL	-0.00799	-0.08623	-0.009	-0.07046	-0.05129	0.05318	0.03327	0.08515	-0.08515	0.91734	0.26208	0.90695	0.3598	0.50525	0.48967	0.66574	0.26817	0.26817	turquoise
ACVRL1	-0.11867	-0.08242	-0.02566	0.0292	-0.11048	0.03254	-0.06092	0.49256	-0.49256	0.12214	0.28384	0.73905	0.70463	0.15029	0.67262	0.42864	7.75E-12	7.75E-12	blue
BMX	-0.06553	-0.07888	-0.00132	0.16931	0.03553	0.21418	0.02142	0.2309	-0.2309	0.3945	0.30511	0.98629	0.02684	0.64456	0.00491	0.78099	0.00238	0.00238	grey
PPFIBP1	-0.08396	-0.11725	0.05603	0.09529	-0.18752	0.16226	0.16759	0.10425	-0.10425	0.27495	0.12671	0.4667	0.21509	0.01405	0.03398	0.02846	0.1748	0.1748	turquoise
GCSAM	-0.00167	-0.01203	0.0916	-0.01385	-0.01578	0.17825	-0.00487	-0.04533	0.04533	0.98272	0.87591	0.23342	0.85734	0.83765	0.01967	0.94959	0.55608	0.55608	blue
KIAA1143	-0.03102	-0.0932	-0.03358	-0.05493	-0.1028	0.0812	-0.02821	0.18436	-0.18436	0.68711	0.22535	0.66283	0.47551	0.18089	0.29106	0.71415	0.01579	0.01579	turquoise
DR1	-0.03514	-0.13368	0.06082	0.03748	-0.08906	0.15853	0.0216	0.09466	-0.09466	0.64822	0.08131	0.42942	0.62646	0.24671	0.03837	0.77918	0.21812	0.21812	turquoise
UBR2	-0.00884	-0.0911	0.00029	0.04884	-0.07306	0.06239	0.00689	0.20823	-0.20823	0.9086	0.23601	0.99701	0.52585	0.34231	0.41756	0.92878	0.00628	0.00628	turquoise
CHTF18	0.02486	0.07321	-0.02564	-0.04242	0.18475	-0.036	0.16188	-0.39506	0.39506	0.74684	0.34131	0.73919	0.58174	0.01556	0.64017	0.03441	8.92E-08	8.92E-08	green
RAD50	-0.02414	-0.10751	-0.04949	-0.01251	-0.08707	-0.00577	0.04795	0.09138	-0.09138	0.75399	0.16162	0.52036	0.87099	0.25747	0.94032	0.53345	0.23457	0.23457	turquoise
RPS9	0.12706	0.19645	-0.02629	-0.03888	-0.01321	-0.01651	0.07602	-0.15092	0.15092	0.09771	0.01002	0.73284	0.61365	0.86385	0.83029	0.32304	0.0488	0.0488	purple
ILF2	-0.02062	-0.04276	-0.0417	-0.09501	-0.05977	0.03134	0.15467	-0.08399	0.08399	0.78891	0.57866	0.58815	0.21642	0.43745	0.68408	0.0434	0.27474	0.27474	greenyellow
CDC40	-0.08411	-0.08377	0.0064	-0.00154	-0.10611	0.01165	-0.06018	0.42409	-0.42409	0.27406	0.27601	0.93383	0.98401	0.16719	0.87976	0.4343	7.47E-09	7.47E-09	turquoise
FAM81B	0.01456	-0.20043	-0.02325	0.07985	0.07295	-0.09748	-0.05589	-0.05297	0.05297	0.85013	0.00858	0.76278	0.29918	0.34303	0.20466	0.46782	0.4914	0.4914	grey
ST6GAL1	-0.15026	0.02104	-0.04787	0.00471	-0.18483	0.10225	0.06086	0.20957	-0.20957	0.04981	0.78474	0.53407	0.95124	0.01552	0.18327	0.42912	0.00594	0.00594	blue
TUBA4A	0.07016	0.05533	-0.04465	-0.04608	0.1137	-0.04825	0.06216	-0.40766	0.40766	0.36184	0.47226	0.56204	0.54949	0.13869	0.53088	0.4193	3.13E-08	3.13E-08	grey
AATF	0.05505	-0.01058	-0.01723	0.00174	0.06326	-0.00903	0.16573	-0.33667	0.33667	0.47452	0.89071	0.823	0.98199	0.41109	0.90672	0.03029	6.72E-06	6.72E-06	greenyellow
RALGPS2	0.05193	-0.13866	0.04204	-0.02853	-0.03614	0.00772	0.03732	-0.14777	0.14777	0.49996	0.0705	0.58513	0.71112	0.63885	0.92022	0.62792	0.05376	0.05376	turquoise
CDK17	-0.08885	-0.16554	0.01243	0.08576	-0.09212	0.00297	-0.07029	0.33501	-0.33501	0.24781	0.03048	0.87178	0.26471	0.23077	0.9692	0.36095	7.50E-06	7.50E-06	turquoise
CCDC152	-0.0158	-0.02657	-0.13328	-0.06848	-0.05612	-0.08222	-0.04189	0.18451	-0.18451	0.83751	0.73009	0.08223	0.37349	0.46593	0.28501	0.58641	0.0157	0.0157	grey
ZNF768	0.11619	-0.04465	-0.11951	-0.04158	0.14023	0.00359	0.12095	-0.38536	0.38536	0.13018	0.562	0.11948	0.58925	0.06735	0.96279	0.11506	1.94E-07	1.94E-07	green
PARG	-0.01038	-0.13642	-0.02059	-0.00177	-0.10997	-0.05587	0.05991	0.07564	-0.07564	0.89278	0.07521	0.78923	0.98163	0.15218	0.46799	0.43638	0.32545	0.32545	turquoise

MIER1	-0.06531	-0.17277	0.06259	0.06882	-0.11403	0.15483	0.0091	0.17115	-0.17115	0.39608	0.02384	0.41608	0.37111	0.13754	0.04318	0.90595	0.02521	0.02521	turquoise
SLC25A22	0.04156	-0.00042	-0.07485	-0.03034	0.09078	-0.12787	0.0503	-0.26505	0.26505	0.58939	0.99566	0.33059	0.69368	0.23767	0.09558	0.51355	0.00046	0.00046	grey
SIGLEC1	-0.13335	0.05096	0.12372	0.09264	-0.2058	0.13839	0.1404	0.28486	-0.28486	0.08208	0.50799	0.10692	0.22813	0.00693	0.07105	0.06701	0.00016	0.00016	blue
WDR59	0.04985	-0.10213	-0.05559	-0.0523	0.1547	-0.13209	-0.04648	-0.05941	0.05941	0.51729	0.18377	0.47018	0.49691	0.04335	0.08503	0.54605	0.44021	0.44021	red
ZNF444	0.11758	-0.03085	-0.14422	-0.09944	0.15571	0.01993	0.02502	-0.31482	0.31482	0.12564	0.68871	0.05985	0.19566	0.04199	0.79582	0.74527	2.74E-05	2.74E-05	red
MRNIP	0.03012	-0.19389	-0.1065	-0.04265	0.19131	-0.11585	-0.12336	0.01435	-0.01435	0.69572	0.01105	0.16564	0.5797	0.01219	0.13134	0.10796	0.85225	0.85225	red
SRSF12	-0.00355	-0.20749	-0.1644	0.01413	0.04319	-0.31591	-0.24689	0.51169	-0.51169	0.96322	0.00647	0.03166	0.85446	0.57485	2.56E-05	0.00113	8.53E-13	8.53E-13	brown
POM121	-0.0305	-0.17927	-0.00418	0.00316	0.01085	-0.03149	-0.13366	0.207	-0.207	0.69215	0.01897	0.95673	0.96728	0.888	0.68261	0.08136	0.0066	0.0066	turquoise
AIP	0.04021	0.02684	-0.05059	0.00721	0.00048	-0.04063	0.06372	-0.15187	0.15187	0.60153	0.72745	0.5111	0.92548	0.99503	0.59773	0.40769	0.04738	0.04738	grey
PPP1R1A	-0.0374	-0.09384	-0.21791	-0.10201	0.08386	-0.2752	-0.27563	0.38765	-0.38765	0.62717	0.22213	0.00419	0.18429	0.27548	0.00027	0.00026	1.62E-07	1.62E-07	brown
ABCB1	0.02108	-0.18464	-0.12493	0.01963	0.05455	-0.1016	-0.21455	0.44896	-0.44896	0.7843	0.01562	0.10351	0.79884	0.47859	0.18606	0.00483	7.33E-10	7.33E-10	brown
HTT	-0.15553	-0.0909	-0.1249	-0.01191	-0.08759	-0.1457	-0.03674	0.25529	-0.25529	0.04223	0.23706	0.10359	0.87713	0.25463	0.05724	0.63334	0.00075	0.00075	turquoise
EIF4A3	-0.02606	-0.11589	0.0704	0.01897	-0.07068	0.07438	0.16104	-0.05918	0.05918	0.73514	0.1312	0.3602	0.80547	0.35827	0.33359	0.03537	0.44198	0.44198	turquoise
CDC42BPE	0.02182	-0.12565	-0.05944	0.01744	0.05763	-0.02594	-0.00481	-0.00926	0.00926	0.77698	0.10152	0.43999	0.82089	0.45404	0.73628	0.95016	0.90429	0.90429	turquoise
IDH3G	0.04435	-0.02076	-0.09333	-0.10522	0.05951	-0.07581	0.08738	-0.23962	0.23962	0.56461	0.78757	0.22471	0.17081	0.43945	0.32438	0.25578	0.0016	0.0016	grey
C19orf25	0.06423	-0.01773	-0.21921	-0.11661	0.21616	-0.20597	-0.03816	-0.14659	0.14659	0.40393	0.81796	0.00397	0.12878	0.00452	0.00688	0.62021	0.05572	0.05572	green
TOMM5	-0.1007	0.00275	-0.14477	-0.06894	0.09389	-0.14965	-0.0426	-0.05873	0.05873	0.19001	0.97147	0.05887	0.37025	0.2219	0.05076	0.58009	0.44544	0.44544	grey
GLCE	-0.00727	-0.13081	-0.01819	0.01547	0.0086	0.09426	0.03794	0.0906	-0.0906	0.92479	0.08813	0.81332	0.84088	0.91108	0.22007	0.62224	0.23859	0.23859	turquoise
EIF2D	-0.01208	0.03566	-0.05735	-0.06295	0.0347	0.08498	0.07054	-0.06395	0.06395	0.87537	0.64333	0.45624	0.41336	0.65225	0.2691	0.35924	0.40602	0.40602	turquoise
RPL8	0.16277	0.16822	0.04439	-0.04119	-0.05557	-0.01229	0.1676	-0.42623	0.42623	0.03341	0.02785	0.5643	0.59272	0.47033	0.87324	0.02845	6.16E-09	6.16E-09	purple
CLPTM1	0.07358	-0.17315	-0.09453	-0.03142	0.12693	-0.14978	-0.02577	-0.08217	0.08217	0.33883	0.02353	0.21876	0.68329	0.09805	0.05054	0.73796	0.28531	0.28531	grey
PKNOX2	-0.09877	-0.09307	-0.03301	0.12272	-0.16957	0.0636	-0.03781	0.4904	-0.4904	0.19872	0.22601	0.66822	0.10981	0.02661	0.40855	0.62343	9.87E-12	9.87E-12	blue
PGAM5	0.07596	-0.03418	0.00888	-0.04535	0.03745	-0.01762	0.04005	-0.1694	0.1694	0.32341	0.65719	0.90818	0.55585	0.62674	0.81912	0.60298	0.02676	0.02676	turquoise
SLC30A8	0.00045	-0.04641	-0.17394	-0.07653	-0.02338	-0.1372	-0.04257	0.34531	-0.34531	0.99538	0.54663	0.02289	0.31979	0.76147	0.07355	0.5804	3.74E-06	3.74E-06	brown
DTL	-0.05961	0.12551	0.00847	-0.01401	-0.10412	0.10184	0.34623	-0.34252	0.34252	0.43864	0.10191	0.91241	0.8557	0.17532	0.18505	3.51E-06	4.53E-06	4.53E-06	pink
FBXW7	-0.04728	-0.20179	-0.01678	-0.00591	0.09544	-0.08129	-0.15419	0.2104	-0.2104	0.53913	0.00813	0.82759	0.93886	0.21432	0.29053	0.04405	0.00574	0.00574	turquoise
C19orf71	-0.02867	-0.06712	-0.09554	-0.05777	0.16958	-0.13479	-0.09098	0.10426	-0.10426	0.70971	0.38309	0.21387	0.45292	0.0266	0.07879	0.23666	0.17477	0.17477	grey
SYS1	0.06652	-0.14003	-0.01951	0.08247	-0.07352	0.02	-0.02408	0.04698	-0.04698	0.38735	0.06775	0.80001	0.28356	0.33928	0.79515	0.75454	0.54179	0.54179	turquoise
KCNK1	0.04606	0.00707	0.09587	-0.00791	-0.00505	0.21118	0.19279	-0.43669	0.43669	0.5497	0.92684	0.21229	0.91821	0.9477	0.00556	0.01153	2.36E-09	2.36E-09	yellow
ILK	-0.07028	-0.11276	0.00619	0.0349	-0.11133	0.10605	0.01915	0.21976	-0.21976	0.36099	0.14201	0.93591	0.65045	0.14716	0.16742	0.80365	0.00388	0.00388	turquoise
COX15	0.02318	-0.06177	-0.04151	-0.06513	-0.00036	-0.01381	0.10259	-0.12959	0.12959	0.76342	0.42219	0.58985	0.3974	0.99623	0.85773	0.1818	0.09116	0.09116	turquoise
PSMB4	-0.00499	0.01911	-0.06436	-0.12436	0.02601	-0.07761	0.13951	-0.3166	0.3166	0.94837	0.80403	0.403	0.10511	0.73557	0.313	0.06878	2.46E-05	2.46E-05	greenyellow
PILRB	0.05983	-0.06009	-0.03251	-0.05287	0.22827	-0.04991	-0.09561	-0.08864	0.08864	0.43697	0.43498	0.67295	0.4922	0.00267	0.5168	0.21354	0.24893	0.24893	red
SCIMP	-0.13008	-0.01037	0.06196	-0.00087	-0.23574	0.10302	-0.02638	0.47452	-0.47452	0.08993	0.89289	0.4208	0.99101	0.00191	0.17996	0.73202	5.50E-11	5.50E-11	blue
TIPRL	-0.02618	-0.08594	-0.02459	-0.05229	-0.02254	0.02481	0.06628	-0.02405	0.02405	0.73389	0.26372	0.74953	0.49702	0.76981	0.7474	0.38904	0.75484	0.75484	turquoise
MTSS1	-0.04313	-0.14261	-0.02777	0.01421	0.04283	-0.07559	-0.05408	0.19335	-0.19335	0.57537	0.06279	0.71845	0.85368	0.57805	0.32578	0.48239	0.01128	0.01128	grey
ADA	-0.05836	-0.00221	0.02974	0.10215	-0.12901	0.09773	0.19466	-0.02369	0.02369	0.44835	0.97716	0.69944	0.18368	0.09264	0.2035	0.01073	0.75842	0.75842	black
KANK2	-0.06509	-0.10351	-0.00592	0.09724	-0.17235	0.14236	0.07669	0.29418	-0.29418	0.39767	0.17791	0.93873	0.2058	0.02419	0.06326	0.3188	9.41E-05	9.41E-05	blue
IFI27	0.00217	0.0892	0.19455	0.15228	-0.03308	0.21217	0.18239	-0.35177	0.35177	0.97757	0.24596	0.01078	0.04677	0.66753	0.00534	0.01696	2.38E-06	2.38E-06	yellow
XK	0.15533	0.00834	0.08549	0.04074	0.05624	0.10525	0.04257	-0.27037	0.27037	0.04249	0.91382	0.26621	0.59678	0.46498	0.17067	0.58038	0.00035	0.00035	grey
GCG	-0.04484	-0.03513	-0.08762	-0.02928	-0.06985	-0.0316	0.0183	0.18036	-0.18036	0.56029	0.64831	0.25446	0.70381	0.36397	0.6816	0.81226	0.01825	0.01825	brown
CALHM5	-0.06029	-0.06335	0.09585	0.10054	-0.2591	0.1284	0.04267	0.41331	-0.41331	0.43342	0.4104	0.21236	0.19075	0.00062	0.0942	0.57947	1.93E-08	1.93E-08	blue
ATP1B1	0.05693	-0.10919	0.07688	0.02964	0.12678	0.17806	-0.02731	-0.27557	0.27557	0.45952	0.15512	0.31756	0.7004	0.09846	0.0198	0.72296	0.00026	0.00026	grey
ERAL1	0.01436	-0.0605	-0.06781	-0.0785	0.0888	-0.13186	0.01397	-0.19348	0.19348	0.85211	0.43184	0.37821	0.30747	0.24812	0.08559	0.85609	0.01123	0.01123	grey
ANXA6	-0.05329	-0.07764	-0.05863	-0.01781	-0.11018	-0.1071	-0.14525	0.53643	-0.53643	0.48879	0.31278	0.4462	0.81713	0.15141	0.16324	0.05802	3.97E-14	3.97E-14	blue
C4orf47	-0.0315	-0.20673	-0.09253	0.0252	0.01702	-0.04694	-0.07398	-0.09651	0.09651	0.68252	0.00667	0.22873	0.74356	0.8251	0.54211	0.3362	0.20921	0.20921	grey
PPP1R3E	0.00938	-0.10656	-0.17037	-0.14834	0.18942	-0.28679	-0.20295	0.22088	-0.22088	0.90307	0.16537	0.02589	0.05284	0.01309	0.00014	0.00776	0.00369	0.00369	brown
NOL6	0.02696	0.01043	-0.07241	-0.0201	-0.0223	-0.00419	0.01773	0.0249	-0.0249	0.72635	0.89226	0.34661	0.79416	0.77218	0.95667	0.81791	0.7465	0.7465	turquoise
LLGL1	-0.13206	-0.12976	0.02123	0.06514	-0.12078	-0.1077	0.01579	0.2263	-0.2263	0.0851	0.09074	0.78281	0.39729	0.11559	0.16087	0.83759	0.00292	0.00292	turquoise
HEMK1	-0.04219	-0.03052	-0.08199	-0.15398	0.03279	0.07143	0.04797	-0.07169	0.07169	0.58374	0.69195	0.28639	0.04435	0.67028	0.35317	0.53327	0.35143	0.35143	grey
ITGB8	0.01147	-0.23268	0.03071	0.15028	-0.07748	0.05111	0.05569	-0.09101	0.09101	0.8816	0.0022	0.69007	0.04978	0.31382	0.50675	0.4694	0.23645	0.23645	turquoise
IGSF3	-0.00915	-0.10552	0.04952	0.11721	-0.1203	0.16357	0.0913	-0.17073	0.17073	0.90549	0.16959	0.52012	0.12682	0.11703	0.03254	0.23497	0.02557	0.02557	turquoise
SLC43A1	-0.01133	0.11874	-0.06152	-0.01227	0.01443	0.03772	-0.0528	0.17661	-0.17661	0.88303	0.12191	0.42407	0.87347	0.85144	0.62429	0.49279	0.02085	0.02085	magenta
CLDND1	-0.03257	-0.17967	-0.05098	-0.01104	-0.10211	-0.00934	-0.03621	0.09902	-0.09902	0.67233	0.0187	0.50786	0.88604	0.18388	0.90345	0.63822	0.19758	0.19758	turquoise
WDR24	-0.01652	0.00721	-0.19489	-0.07091	0.21531	-0.18816	0.01023	-0.1051	0.1051	0.83021	0.9254	0.01064	0.35675	0.00468	0.01372	0.89439	0.17127	0.17127	green
COQ5	0.04488	-0.07002	-0.06057	-0.03193	0.11096	-0.06333	0.00431	-0.09893	0.09893	0.55996	0.36281	0.43131	0.67842	0.1485	0.41055	0.95541	0.19797	0.19797	turquoise
VPS28	0.06315	0.04475	-0.09088	-0.13636	0.08608	-0.1507	0.08105	-0.36183	0.36183	0.41193	0.56109	0.23716	0.07535	0.26296	0.04913	0.29197	1.16E-06	1.16E-06	green
TEX9	0.02032	-0.11392	-0.08916	-0.05711	0.01284	-0.03219	-0.11608	0.02695	-0.02695	0.79196	0.1379	0.2462	0.45815	0.86763	0.67599	0.13057	0.7264	0.7264	grey
RNF39	0.07304	0.0134	0.08696	0.01154	0.00868	0.19577	0.13897	-0.4955	0.4955	0.34244	0.86186	0.25809	0.8809	0.91029	0.01028	0.06988	5.57E-12	5.57E-12	yellow
KIAA0232	-0.05221	-0.17699	-0.18231	-0.0922	-0.02186	-0.09481	-0.08233	0.25135	-0.25135	0.49761	0.02057	0.017	0.23038	0.77654	0.21738	0.28438	0.00091	0.00091	turquoise
CHRNA3	-0.01141	-0.07115	-0.02322	0.0347	-0.01741	-0.02515	-0.11966	0.32986	-0.32986	0.88225	0.35508	0.76307	0.65226	0.82117	0.74404	0.11902	1.05E-05	1.05E-05	grey
ZNF226	0.00534	-0.1344	-0.1209	-0.10181	0.11392	-0.11173	-0.05807	0.00088	-0.00088	0.94472	0.07967	0.1152	0.18516	0.1379	0.1457	0.45058	0.99091	0.99091	red
KIAA2026	-0.0276	-0.12452	-0.05653	-0.02688	-0.0973	0.0329	-0.02327	0.31877	-0.31877	0.72009	0.10465	0.46268	0.72707	0.2055	0.66928	0.76261	2.15E-05	2.15E-05	turquoise
RCAN1	-0.03592	-0.15467	-0.03022	0.0871	-0.08356	0.04188	-0.04114	0.45248	-0.45248	0.64093	0.04339	0.69478	0.25732	0.27721	0.58654	0.59316	5.19E-10	5.19E-10	blue
REXO5	-0.07205	-0.02453	-0.08242	-0.08133	0.06079	-0.21008	0.04381	0.06192	-0.06192	0.34906	0.75009	0.28386	0.29028	0.42966	0.00582	0.56939	0.42108	0.42108	grey
AEN	0.01258	-0.02086	-0.10487	-0.07442	0.01692	-0.1831	-0.08959	0.15694	-0.15694	0.87025	0.78654	0.17222	0.33337	0.82615	0.01653	0.2439	0.04038	0.04038	grey
EIF3K	0.17328	-0.04747	-0.05612	-0.01314	-0.05065	-0.00782	0.12782	-0.1681	0.1681	0.02342	0.53754	0.466	0.86454	0.51056	0.91917	0.09571	0.02797	0.02797	purple
ZNF91	0.01635	-0.13096	-0.14892	-0.02679	0.15229	-0.08882	-0.18858	0.16894	-0.16894	0.83194	0.08776	0.05191	0.72803	0.04677	0.24799	0.01351	0.02718	0.02718	turquoise
TOPBP1	-0.0643	-0.05113	-0.03537	-0.01417	-0.1309	0.09425	0.16558	0.00054	-0.00054	0.4034	0.50656	0.64602	0.85405	0.08792	0.22015	0.03044	0.99436	0.99436	turquoise
ZNF202	-0.03599	-0.12933	-0.03923	0.00355	-0.05215	0.08253	0.06956	0.02348	-0.02348	0.64024	0.09183	0.61049	0.9632	0.49813	0.28321	0.36598	0.76046	0.76046	turquoise
GMFB	-0.01653	-0.10599	-0.0082	0.01507	-0.03649	-0.01196	-0.03804	0.11219	-0.11219	0.83008	0.16768	0.9152	0.84491	0.63558	0.87659	0.62137	0.14404	0.14404	turquoise
RERE	-0.03838	-0.15672	-0.05098	0.03631	-0.10497	0.1265	-0.03875	0.26	-0.26	0.61825	0.04066	0.50789	0.63726	0.17184	0.09922	0.6148	0.00059	0.00059	turquoise
AK1	0.03605	0.05008	-0.1424	-0.05942	0.13079	-0.16664	0.04106	-0.08902	0.08902	0.63974	0.51534	0.06318	0.44009	0.08818	0.02938	0.59391	0.24693	0.24693	grey
AK5	-0.04813	-0.05575	-0.09738	-0.10243	0.03591	-0.1984	-0.07815	0.24745	-0.24745	0.53188	0.46889	0.20513	0.18248	0.64103	0.00929	0.30961	0.0011	0.0011	grey
GNB1	-0.07535	-0.1378	0.03433	0.04054	-0.14639	0.0867	0.00474	0.11884	-0.11884	0.32734	0.07227	0.65577	0.59856	0.05605	0.25948	0.95093	0.1216	0.1216	turquoise
EPG5	-0.06758	-0.22556	-0.12435	-0.007	-0.01359	-0.1378	-0.09716	0.42843	-0.42843	0.37983	0.00301	0.10513	0.92762	0.85995	0.07229	0.20616	5.05E-09	5.05E-09	turquoise
WNT11	-0.01696	0.02538	0.04343	0.09605	-0.09568	0.013	0.02125	0.01506	-0.01506	0.82572	0.74175	0.5727	0.21142	0.21318	0.86597	0.78265	0.84497	0.84497	grey
C11orf74	-0.06424	-0.10471	-0.13409	-0.00867	-0.13435	0.02279	0.07438	0.03696	-0.03696	0.40388	0.17289	0.08039	0.91044	0.07979	0.76733	0.33364	0.63131	0.63131	grey
MYO9A	-0.07235	-0.18965	-0.03895	0.03441	-0.18788	0.05606	0.01031	0.2868	-0.2868	0.347	0.01297	0.613	0.655	0.01386	0.46645	0.89353	0.00014	0.00014	turquoise
EMILIN1	-0.08261	-0.05526	0.02864	0.0531	-0.11591	0.16563	0.09949	0.22141	-0.22141	0.28276	0.47282	0.71001	0.49031	0.13111	0.03039	0.19545	0.00361	0.00361	black
ATP6V1B1	-0.08784	0.08239	-0.03267	0.06119	0.14096	-0.0706	0.07937	-0.27223	0.27223	0.25327	0.28404	0.67138	0.42657	0.06592	0.35884	0.30209	0.00032	0.00032	grey
MCFD2	-0.03161	-0.13098	0.02067	0.07317	-0.26057	0.02636	0.11361	0.21923	-0.21923	0.68147	0.08772	0.78839	0.34159	0.00058	0.73216	0.13901	0.00397	0.00397	turquoise
OGA	0.05525	-0.12447	0.00147	-0.00598	-0.06129	0.0248	0.05349	-0.04095	0.04095	0.47289	0.1048	0.98482	0.93817	0.42581	0.74752	0.48713	0.59486	0.59486	red
CD36	-0.07903	-0.09028	0.03288	0.14072	-0.06309	-0.04204	-0.13243	0.52504	-0.52504	0.30421	0.24025	0.66945	0.06639	0.41231	0.58511	0.08424	1.68E-13	1.68E-13	blue
SCIN	0.05684	-0.07878	0.00551	-0.06238	0.06715	0.00982	-0.10119	-0.05661	0.05661	0.46024	0.30576	0.94296	0.41767	0.38285	0.89862	0.18786	0.46204	0.46204	grey
DYNC1H1	0.02362	-0.14332	-0.03121	0.00985	-0.09563	0.0483	0.02812	0.1419	-0.1419	0.75914	0.06148	0.68527	0.89825	0.21343	0.53041	0.71503	0.06411	0.06411	turquoise
MSRB3	-0.11096	-0.05552	0.02102	0.06622	-0.18184	0.14373	-0.00024	0.40699	-0.40699	0.14853	0.47073	0.78491	0.38951	0.0173	0.06073	0.99752	3.31E-08	3.31E-08	blue
ZNF74	-0.02044	-0.0827	-0.14245	0.0032	0.0393	-0.13588	-0.01317	0.18409	-0.18409	0.79075	0.28222	0.06309	0.96689	0.60981	0.07639	0.86429	0.01594	0.01594	grey
RIOK2	-0.03371	-0.1292	-0.04805	-0.02912	-0.04028	-0.02398	-0.00343	0.2033	-0.2033	0.6616	0.09214	0.53261	0.70541	0.60088	0.75555	0.96453	0.00766	0.00766	turquoise
PPIL6	0.04298	-0.20549	-0.09958	0.02634	0.10449	-0.17591	-0.13878	0.15246	-0.15246	0.57677	0.00701	0.19504	0.73233	0.1738	0.02137	0.07026	0.04651	0.04651	grey
HLCS	-0.01513	-0.13398	-0.15589	-0.07806	0.07162	-0.11739	-0.01449	0.08124	-0.08124	0.8443	0.08064	0.04175	0.31021	0.3519	0.12622	0.85076	0.29085	0.29085	turquoise
TENT5C	-0.0773	-0.07603	-0.12359	-0.05313	-0.00439	-0.12468	-0.20153	0.52922	-0.52922	0.31495	0.32296	0.1073	0.49006	0.95456	0.10421	0.00821	9.96E-14	9.96E-14	brown
ANLN	-0.03025	0.02364	0.06377	0.04035	-0.1145	0.11772	0.30345	-0.36903	0.36903	0.69447	0.7589	0.40728	0.60025	0.1359	0.12516	5.47E-05	6.81E-07	6.81E-07	pink
SYK	-0.05571	0.06523	0.07939	0.0664	-0.197	0.30745	0.20269	-0.00324	0.00324	0.46921	0.3966	0.30198	0.38819	0.00981	4.30E-05	0.00784	0.9664	0.9664	blue
CENPN	0.01624	0.00457	0.01853	-0.03113	-0.13575	0.07915	0.25755	-0.25086	0.25086	0.833	0.95267	0.80988	0.68609	0.07668	0.30344	0.00067	0.00093	0.00093	pink
C1orf226	-0.00573	-0.0129	-0.016	-0.01057	0.07024	0.04206	0.01718	-0.27449	0.27449	0.94066	0.86702	0.83551	0.89085	0.36129	0.58489	0.82351	0.00028	0.00028	grey
CRMP1	-0.04194	-0.15095	-0.24424	-0.12963	0.07254	-0.29231	-0.24812	0.4779	-0.4779	0.58597	0.04875	0.00129	0.09105	0.34573	0.0001	0.00107	3.85E-11	3.85E-11	brown
SLC8B1	0.00586	-0.03212	0.15229	0.12426	-0.13885	0.19988	0.18256	-0.14472	0.14472	0.93936	0.67668	0.04676	0.10538	0.07012	0.00877	0.01685	0.05896	0.05896	black
ARHGAP2	0.00897	-0.06812	0.07059	0.02333	0.00667	0.2496	0.15391	-0.20824	0.20824	0.90734	0.37599	0.3589	0.76195	0.93102	0.00099	0.04444	0.00627	0.00627	yellow
PLXNB3	-0.08269	-0.04777	-0.02527	0.01128	0.14749	-0.04743	0.09983	-0.15304	0.15304	0.2823	0.535	0.7429	0.88356	0.05423	0.53785	0.19391	0.04567	0.04567	grey
VPS11	-0.0572	-0.06298	-0.07684	-0.09604	-0.10927	0.03137	0.0223	0.17258	-0.17258	0.4574	0.41316	0.31785	0.21147	0.15484	0.68379	0.77215	0.024	0.024	turquoise
TMEM102	-0.05152	0.00273	-0.04819	-0.04096	-0.00334	0.13812	0.20163	-0.34754	0.34754	0.50338	0.97178	0.53136	0.59479	0.96546	0.07162	0.00818	3.20E-06	3.20E-06	grey
TMEM121	-0.0505	-0.10776	-0.17477	-0.00144	0.12346	-0.22317	-0.03496	0.10199	-0.10199	0.51183	0.16065	0.02223	0.98506	0.10767	0.00335	0.6499	0.1844	0.1844	green
KSR1	-0.09466	-0.08148	-0.01202	0.06712	0.00133	-0.02881	-0.15114	0.52393	-0.52393	0.21812	0.28939	0.87597	0.38305	0.9862	0.70832	0.04846	1.93E-13	1.93E-13	blue
BTG3	-0.05084	-0.18441	-0.11642	-0.06394	0.01386	-0.06651	-0.00902	0.17457	-0.17457	0.50902	0.01575	0.12943	0.40608	0.85722	0.38738	0.90676	0.0224	0.0224	turquoise
KIF5B	0.01253	-0.08879	0.07156	0.0528	-0.05915	0.10184	0.04565	0.05778	-0.05778	0.87075	0.24814	0.3523	0.49278	0.44222	0.18506	0.5533	0.45282	0.45282	turquoise
PRRC2B	-0.04508	-0.17469	-0.05282	0.01545	-0.16492	0.02944	-0.0827	0.36307	-0.36307	0.55819	0.0223	0.49259	0.84107	0.03112	0.70227	0.2822	1.06E-06	1.06E-06	turquoise
PSMC3IP	-0.09703	-0.10953	-0.1197	-0.02938	0.0489	-0.16071	0.09053	-0.08396	0.08396	0.20679	0.15385	0.11891	0.70282	0.52535	0.03575	0.23896	0.27492	0.27492	grey
OR2A7	0.02844	-0.13412	0.07407	-0.02068	0.03603	0.15748	-0.02174	-0.18745	0.18745	0.71191	0.08032	0.33563	0.78838	0.63988	0.03968	0.77777	0.01409	0.01409	grey
GCAT	-0.01093	0.19917	-0.09402	-0.06511	-0.00091	-0.05382	0.04843	-0.03029	0.03029	0.88717	0.00901	0.22126	0.39753	0.99062	0.48449	0.52934	0.69409	0.69409	grey
SLTM	-0.04654	-0.14361	-0.06402	-0.04638	-0.0176	0.00351	-0.04585	0.11488	-0.11488	0.54551	0.06095	0.40545	0.54693	0.81927	0.96369	0.55156	0.1346	0.1346	turquoise
COG7	0.05893	-0.05514	-0.07266	-0.10508	0.16573	-0.03469	0.00376	-0.22905	0.22905	0.44391	0.47383	0.34498	0.17138	0.03029	0.65239	0.96106	0.00258	0.00258	grey
UBE2C	0.0452	0.11107	0.06218	0.00225	-0.03761	0.09256	0.31826	-0.55258	0.55258	0.55722	0.14812	0.41912	0.97675	0.6253	0.22855	2.21E-05	4.66E-15	4.66E-15	pink
GTF3C3	-0.0147	-0.0353	0.01654	0.02902	-0.04367	0.03736	0.10057	-0.0696	0.0696	0.84868	0.64669	0.82998	0.70636	0.57065	0.62758	0.19062	0.3657	0.3657	turquoise
UHRF2	-0.00823	-0.09099	0.00036	-0.00947	-0.12892	0.06471	0.07044	0.1305	-0.1305	0.91491	0.23656	0.99632	0.9022	0.09287	0.40044	0.35994	0.08889	0.08889	turquoise
GNB5	4.46E-05	-0.2149	-0.19691	-0.08504	-0.0863	-0.17371	-0.19046	0.26882	-0.26882	0.99954	0.00476	0.00984	0.26878	0.2617	0.02307	0.01259	0.00038	0.00038	turquoise
SELPLG	-0.06506	0.07815	0.12217	0.04517	-0.10734	0.26569	0.16401	0.01736	-0.01736	0.39786	0.30963	0.11141	0.55743	0.16231	0.00044	0.03208	0.82167	0.82167	blue
XRN2	-0.06418	-0.12766	0.01679	0.00343	-0.07187	0.06157	0.05535	0.12928	-0.12928	0.4043	0.09612	0.82741	0.96449	0.35023	0.42375	0.47211	0.09195	0.09195	turquoise
ZNF773	-0.06394	-0.15884	-0.09461	-0.02957	-0.07429	0.00384	-0.07327	0.19538	-0.19538	0.40603	0.03798	0.21839	0.70104	0.33422	0.96027	0.34093	0.01044	0.01044	turquoise
ANKRD54	-0.03365	-0.08814	-0.28486	-0.13801	0.14735	-0.29162	-0.16574	0.0243	-0.0243	0.66212	0.25163	0.00016	0.07185	0.05445	0.00011	0.03028	0.75242	0.75242	grey
POLR1E	0.02213	0.03467	-0.0938	-0.02541	-0.15962	0.05604	0.06536	0.18594	-0.18594	0.77389	0.65255	0.22236	0.74147	0.03703	0.46659	0.39568	0.0149	0.0149	grey
EIF5A2	0.0578	-0.04462	0.07734	0.04002	-0.23126	0.21123	0.19303	0.05634	-0.05634	0.45268	0.56224	0.31466	0.60324	0.00234	0.00555	0.01142	0.46418	0.46418	turquoise
MCEMP1	-0.06699	-0.00605	0.14714	0.0678	-0.09958	0.15751	#######	0.08498	-0.08498	0.38396	0.93742	0.0548	0.37826	0.19503	0.03965	0.99971	0.26912	0.26912	blue
ARHGEF3	-0.03027	0.044	-0.03307	0.04122	-0.15245	0.09668	0.00892	0.27233	-0.27233	0.69427	0.56768	0.66762	0.59244	0.04652	0.20843	0.90779	0.00031	0.00031	turquoise
MARS2	-0.05502	0.03356	0.01861	0.04924	-0.0283	0.12638	-0.02128	0.06429	-0.06429	0.47478	0.66301	0.8091	0.52248	0.71328	0.09954	0.78231	0.40348	0.40348	turquoise
NPM3	0.08245	0.12024	0.0499	0.00597	-0.01005	-0.0487	0.28121	-0.36847	0.36847	0.2837	0.11724	0.51685	0.93825	0.89623	0.52702	0.00019	7.10E-07	7.10E-07	purple
WWP1	-0.05554	-0.07644	0.06322	0.11049	-0.12044	0.20775	0.06747	-0.04148	0.04148	0.4706	0.32039	0.41136	0.15026	0.11662	0.0064	0.38058	0.59013	0.59013	turquoise
RBM33	0.01081	-0.12677	-0.0435	-0.00981	0.06016	-0.0611	-0.01766	-0.06617	0.06617	0.88839	0.0985	0.57215	0.89868	0.4344	0.42724	0.81871	0.38986	0.38986	red
CDCA5	-0.02963	0.0844	0.06305	-0.01281	-0.12024	0.09436	0.35624	-0.45111	0.45111	0.70043	0.27243	0.41265	0.86796	0.11722	0.2196	1.73E-06	5.94E-10	5.94E-10	pink

NCBP2	-0.03979	-0.08029	-0.06474	-0.01466	-0.11622	0.05726	0.15237	-0.08164	0.08164	0.60537	0.29655	0.4002	0.84911	0.13009	0.45695	0.04664	0.28844	0.28844	turquoise
ELMO3	0.11016	0.12542	-0.06357	-0.1176	0.1275	0.13589	0.18808	-0.62607	0.62607	0.15147	0.10215	0.40879	0.12556	0.09655	0.07636	0.01376	5.36E-20	5.36E-20	yellow
TTYH2	-0.0592	-0.08142	-0.0186	0.06093	-0.1111	-0.01317	-0.07087	0.58133	-0.58133	0.44178	0.28977	0.80916	0.42857	0.148	0.86424	0.35701	7.65E-17	7.65E-17	blue
NCCRP1	-0.0422	-0.13667	0.06319	0.15473	-0.03777	0.10493	0.09162	-0.04233	0.04233	0.58363	0.07468	0.41158	0.04331	0.6238	0.17198	0.23331	0.58255	0.58255	grey
STX2	-0.07821	-0.15169	-0.09374	0.03698	-0.06532	-0.12305	-0.13127	0.35527	-0.35527	0.30923	0.04764	0.22266	0.63106	0.39598	0.10884	0.087	1.86E-06	1.86E-06	turquoise
UTP23	-0.05189	-0.10311	-0.01806	0.01695	-0.14948	-0.01122	0.00862	0.15112	-0.15112	0.50029	0.17961	0.81468	0.82587	0.05101	0.88416	0.91095	0.04849	0.04849	turquoise
PAXBP1	-0.02989	-0.1668	-0.11142	0.01989	0.19989	-0.07311	-0.06393	0.14588	-0.14588	0.698	0.02922	0.14683	0.79627	0.00876	0.34197	0.40615	0.05693	0.05693	red
HIST1H2B	0.00477	-0.06084	0.11841	0.09375	-0.01467	-0.00128	0.13057	-0.19668	0.19668	0.95065	0.42923	0.12297	0.22261	0.84898	0.98674	0.08871	0.00993	0.00993	grey
SPSB1	-0.04957	-0.124	0.00325	0.08438	-0.24685	0.19545	0.0325	0.18958	-0.18958	0.5197	0.10611	0.9663	0.27255	0.00113	0.01041	0.67305	0.01301	0.01301	black
ARHGAP3	0.07676	-0.14923	-0.11757	-0.02148	0.14878	-0.24633	-0.03132	-0.13681	0.13681	0.31831	0.05141	0.12566	0.78031	0.05213	0.00116	0.68429	0.07438	0.07438	grey
CACNA2D	0.02351	-0.10078	-0.28857	-0.19087	0.13583	-0.2971	-0.22743	0.42957	-0.42957	0.7602	0.18966	0.00013	0.01239	0.07649	7.95E-05	0.00278	4.54E-09	4.54E-09	brown
TMEM35A	-0.01675	-0.02435	-0.0131	0.06999	-0.16009	0.07793	0.08948	0.31685	-0.31685	0.82786	0.75187	0.86499	0.363	0.03648	0.31097	0.24449	2.42E-05	2.42E-05	salmon
CSF1R	-0.10112	-0.03994	0.02071	0.02371	-0.22022	0.14516	0.09963	0.46683	-0.46683	0.18817	0.60396	0.78802	0.75824	0.0038	0.05818	0.19479	1.23E-10	1.23E-10	blue
VPS51	-0.01565	0.04039	-0.09034	-0.0591	0.02766	-0.04728	-0.00447	-0.01616	0.01616	0.83897	0.59996	0.23997	0.44255	0.71948	0.53913	0.95372	0.83383	0.83383	grey
SNPH	-0.03004	-0.14439	-0.24146	-0.08993	0.10268	-0.3442	-0.25913	0.49421	-0.49421	0.69655	0.05953	0.00146	0.24211	0.18142	4.03E-06	0.00062	6.44E-12	6.44E-12	brown
ABRAXAS	-0.06461	-0.07093	0.00571	0.02845	-0.09739	0.04292	0.10597	0.03804	-0.03804	0.40117	0.35659	0.9409	0.71183	0.20509	0.57725	0.16777	0.62134	0.62134	turquoise
TMEM74B	0.00885	-0.11787	-0.22472	-0.15927	0.29307	-0.28809	-0.16543	0.03964	-0.03964	0.90853	0.12468	0.00313	0.03745	0.0001	0.00013	0.03059	0.6067	0.6067	grey
NFU1	-0.01863	-0.04587	-0.08697	-0.02947	-0.01273	-0.18519	-0.06414	0.0415	-0.0415	0.80885	0.55131	0.25801	0.70204	0.86878	0.01531	0.40461	0.58993	0.58993	turquoise
COA3	-0.02583	-0.07266	-0.25075	-0.09583	0.22965	-0.2948	-0.11062	-0.12042	0.12042	0.73736	0.34492	0.00094	0.21248	0.00252	9.08E-05	0.14977	0.11668	0.11668	grey
KLHL17	-0.04233	-0.0038	-0.0365	-0.04329	0.21204	-0.06279	0.07772	-0.25985	0.25985	0.58248	0.9607	0.63554	0.574	0.00537	0.41461	0.31229	0.0006	0.0006	green
CHADL	-0.11632	0.05504	-0.20385	-0.04388	0.20818	-0.18743	-0.12898	0.04664	-0.04664	0.12974	0.47462	0.00749	0.56879	0.00629	0.0141	0.0927	0.54468	0.54468	grey
GATAD2A	0.02702	-0.03537	-0.06583	-0.0046	0.01808	0.05286	0.11478	-0.17491	0.17491	0.7257	0.64606	0.39227	0.95242	0.81439	0.49227	0.13494	0.02213	0.02213	turquoise
BCORL1	0.06048	-0.31501	-0.09603	0.0904	-0.15059	0.03493	0.11305	0.02305	-0.02305	0.432	2.71E-05	0.21153	0.23967	0.0493	0.65013	0.14096	0.76479	0.76479	turquoise
ZBTB8A	-0.09139	-0.10583	-0.02577	0.0774	-0.15897	0.1026	0.05116	0.33631	-0.33631	0.2345	0.16833	0.73794	0.31432	0.03783	0.18176	0.50633	6.89E-06	6.89E-06	blue
SLC1A4	-0.0166	-0.07149	-0.11099	-0.05293	-0.06671	-0.20256	-0.24307	0.42506	-0.42506	0.82936	0.35277	0.14838	0.49174	0.38597	0.00788	0.00136	6.84E-09	6.84E-09	brown
SORD	-0.03789	0.05833	0.06549	-0.00117	0.03173	0.07139	0.08498	-0.32933	0.32933	0.62268	0.44856	0.39478	0.98791	0.68039	0.35345	0.26909	1.09E-05	1.09E-05	grey
RFX8	-0.03782	0.03334	-0.00954	0.02232	-0.12913	-0.10292	0.11638	-0.09683	0.09683	0.62339	0.66507	0.9014	0.77195	0.09233	0.18038	0.12955	0.20769	0.20769	grey
NKAPD1	-0.09542	-0.13942	-0.05901	0.00172	-0.10666	0.0554	0.06947	0.05271	-0.05271	0.21445	0.06897	0.4433	0.98221	0.16499	0.47169	0.36659	0.49353	0.49353	turquoise
TMEM209	-0.03188	-0.11409	-0.09038	-0.04823	-0.01665	-0.19841	-0.05061	0.19074	-0.19074	0.67889	0.13734	0.23977	0.53101	0.82888	0.00928	0.51096	0.01246	0.01246	turquoise
SLC17A7	-0.05464	-0.13614	0.00813	0.10507	-0.12031	0.0195	0.07975	0.47916	-0.47916	0.47781	0.07582	0.91593	0.17142	0.11701	0.80019	0.2998	3.36E-11	3.36E-11	blue
ZNF320	-0.0011	-0.10646	-0.06929	0.00346	0.05252	0.057	-0.05882	0.06747	-0.06747	0.9886	0.16577	0.36786	0.96414	0.49513	0.45896	0.44474	0.38059	0.38059	turquoise
DERA	-0.02734	-0.12187	0.00338	0.01494	-0.07121	-0.0008	0.08947	-0.19804	0.19804	0.72265	0.1123	0.965	0.84623	0.35467	0.99166	0.24454	0.00942	0.00942	turquoise
EIF3C	0.07795	0.01752	0.01772	0.01294	-0.05393	0.13403	0.12141	-0.01671	0.01671	0.3109	0.82007	0.8181	0.8666	0.48354	0.08052	0.11369	0.82825	0.82825	turquoise
INA	-0.00139	-0.13202	-0.25851	-0.11875	0.05761	-0.314	-0.1864	0.47667	-0.47667	0.98564	0.08521	0.00064	0.12189	0.45421	2.89E-05	0.01464	4.38E-11	4.38E-11	brown
PACSIN1	-0.0101	-0.0529	-0.2208	-0.08455	0.05635	-0.27486	-0.30512	0.43538	-0.43538	0.89565	0.49197	0.00371	0.27155	0.46417	0.00027	4.95E-05	2.66E-09	2.66E-09	brown
MRPL19	-0.06385	0.00117	-0.04139	-0.05098	-0.11049	0.08253	0.14664	-0.01109	0.01109	0.40673	0.98788	0.5909	0.50781	0.15026	0.28322	0.05564	0.88551	0.88551	turquoise
BACH2	-0.0937	-0.10932	-0.00234	-0.00885	-0.22006	-0.04984	-0.1473	0.51717	-0.51717	0.22285	0.15462	0.97581	0.90851	0.00383	0.51741	0.05453	4.42E-13	4.42E-13	blue
ZNF34	-0.02796	-0.15219	-0.15612	-0.06488	0.04459	-0.27973	-0.14608	0.23164	-0.23164	0.71661	0.04691	0.04144	0.39918	0.56255	0.00021	0.05659	0.0023	0.0023	brown
FDCSP	-0.0546	0.01306	-0.0636	-0.07484	-0.01829	0.04666	-0.07083	0.2087	-0.2087	0.47815	0.86538	0.4086	0.33066	0.81228	0.54453	0.35728	0.00616	0.00616	blue
MRAS	-0.09677	-0.05649	-0.05627	0.04444	-0.22034	0.07673	0.04934	0.46695	-0.46695	0.20799	0.46301	0.46474	0.56388	0.00378	0.31853	0.5216	1.21E-10	1.21E-10	blue
CLDN23	-0.07432	-0.01248	0.07899	-0.00082	0.05131	0.24639	0.17765	-0.28052	0.28052	0.33399	0.87128	0.30447	0.9915	0.50514	0.00116	0.0201	0.0002	0.0002	yellow
EFNA2	0.01529	0.07291	0.04169	0.00633	0.22695	0.15451	0.09828	-0.34558	0.34558	0.8427	0.34327	0.58823	0.93454	0.00283	0.04362	0.20094	3.67E-06	3.67E-06	yellow
ZSCAN29	-0.10377	-0.14138	-0.09318	0.01301	-0.11228	0.00062	-0.00743	0.10868	-0.10868	0.1768	0.06511	0.22542	0.86593	0.14371	0.99358	0.92317	0.15708	0.15708	turquoise
DGUOK	0.04625	-0.02062	-0.06472	-0.09523	0.08585	-0.06106	0.10829	-0.40947	0.40947	0.54804	0.78894	0.40035	0.21534	0.26423	0.42761	0.15859	2.68E-08	2.68E-08	green
LILRA4	-0.09587	0.06245	0.08606	0.0665	-0.22893	0.1051	-0.04788	0.33112	-0.33112	0.21229	0.41709	0.26303	0.38746	0.0026	0.17131	0.53405	9.71E-06	9.71E-06	blue
GLRB	-0.0042	-0.12814	-0.18067	0.01811	-0.06767	-0.14347	-0.17709	0.39781	-0.39781	0.95656	0.09487	0.01804	0.81418	0.37915	0.0612	0.0205	7.13E-08	7.13E-08	brown
EIF5	0.0244	-0.09488	-0.011	0.05742	0.09003	-0.03394	-0.08223	0.11656	-0.11656	0.75141	0.21707	0.8865	0.45569	0.24161	0.65942	0.28497	0.12895	0.12895	turquoise
ARL4A	-0.06214	-0.08186	0.01908	0.06572	-0.06211	0.09508	-0.00608	-0.20005	0.20005	0.41943	0.28714	0.80434	0.3931	0.41966	0.21607	0.93706	0.0087	0.0087	turquoise
ZNF845	-0.04147	-0.07669	0.02026	0.05933	-0.18647	0.19842	0.0607	0.11391	-0.11391	0.59016	0.3188	0.79251	0.44081	0.01461	0.00928	0.43031	0.13795	0.13795	turquoise
RBM12B	-0.00699	-0.16942	-0.03195	0.03666	-0.0979	0.04077	0.069	0.08529	-0.08529	0.92772	0.02674	0.67824	0.63403	0.20271	0.59652	0.36989	0.26738	0.26738	turquoise
HSD17B3	-0.03295	-0.03648	-0.20199	-0.04431	0.19866	-0.2197	-0.20406	0.04297	-0.04297	0.66875	0.63571	0.00807	0.56501	0.00919	0.00389	0.00743	0.5768	0.5768	grey
TBKBP1	0.06616	-0.09529	-0.23791	0.02934	0.08014	-0.24282	-0.11833	0.35713	-0.35713	0.38993	0.21507	0.00173	0.70322	0.29745	0.00137	0.12319	1.63E-06	1.63E-06	brown
SOX11	-0.06574	-0.03311	0.03406	0.07278	-0.04514	0.03356	0.12676	-0.01738	0.01738	0.39294	0.6673	0.65834	0.34412	0.55772	0.66301	0.09851	0.82154	0.82154	black
DSCC1	-0.03864	0.12115	-0.02725	0.02588	-0.12544	0.04133	0.28972	-0.14296	0.14296	0.61584	0.11445	0.72346	0.73691	0.10209	0.59142	0.00012	0.06213	0.06213	pink
MAGI3	-0.02911	-0.07037	0.07443	0.09857	-0.05613	0.21824	0.02726	0.06668	-0.06668	0.70542	0.36039	0.33327	0.1996	0.46585	0.00414	0.72344	0.38623	0.38623	turquoise
RCBTB2	-0.11843	-0.07846	0.03851	0.10079	-0.10586	0.05737	-0.03614	0.43406	-0.43406	0.1229	0.30773	0.617	0.18962	0.16819	0.45611	0.63885	3.01E-09	3.01E-09	blue
RND2	-0.02098	-0.22477	-0.3159	-0.14794	0.12421	-0.38684	-0.22985	0.35341	-0.35341	0.78529	0.00312	2.57E-05	0.05348	0.10552	1.73E-07	0.00249	2.12E-06	2.12E-06	brown
C21orf62	-0.04151	-0.04864	-0.1536	0.04563	-0.07029	0.00458	-0.04143	0.28914	-0.28914	0.58985	0.52753	0.04488	0.55346	0.36097	0.95258	0.5906	0.00013	0.00013	magenta
MT-ND3	-0.0677	-0.07621	-0.04825	-0.01112	0.17171	-0.04531	-0.14384	-0.07304	0.07304	0.37899	0.32183	0.5309	0.88524	0.02473	0.55619	0.06053	0.3424	0.3424	grey
KIF7	-0.0614	-0.13184	0.0006	0.10106	-0.09141	0.05327	0.175	0.09465	-0.09465	0.42497	0.08563	0.9938	0.18845	0.23439	0.48897	0.02206	0.21817	0.21817	black
PRPS2	-0.09429	-0.00466	0.0174	0.0831	-0.18686	0.23646	0.1752	-0.18121	0.18121	0.21992	0.95174	0.82129	0.27988	0.0144	0.00185	0.02191	0.0177	0.0177	turquoise
COX4I1	0.13448	0.10327	-0.1146	-0.15142	0.1089	-0.05894	0.06173	-0.44428	0.44428	0.07949	0.17892	0.13556	0.04804	0.15625	0.44383	0.42253	1.15E-09	1.15E-09	grey
BHLHE40	-0.03712	-0.07693	0.09625	0.07811	-0.05162	0.27586	0.07719	-0.18678	0.18678	0.62983	0.31724	0.21044	0.30987	0.50254	0.00026	0.31564	0.01444	0.01444	turquoise
LDLRAP1	-0.00603	-0.09004	-0.1598	-0.09435	-0.00495	-0.08854	-0.05085	0.26673	-0.26673	0.9376	0.24155	0.03681	0.21966	0.94877	0.2495	0.50897	0.00042	0.00042	grey
NSD2	-0.07273	-0.05612	-0.07461	-0.03544	0.02529	-0.04117	0.06161	-0.18812	0.18812	0.3445	0.46598	0.33213	0.64536	0.74263	0.59286	0.42342	0.01374	0.01374	turquoise
RBM42	0.11582	-0.0356	-0.07436	-0.04394	0.07279	-0.04463	0.15047	-0.37182	0.37182	0.13141	0.64394	0.33374	0.56826	0.3441	0.56215	0.04949	5.52E-07	5.52E-07	green
VSTM5	0.0027	-0.00241	0.03934	-0.0151	0.09553	0.18449	0.12508	-0.27552	0.27552	0.97204	0.97499	0.60947	0.84459	0.2139	0.01571	0.1031	0.00027	0.00027	grey
A4GALT	-0.01766	-0.02913	0.00782	0.09861	-0.06432	-0.03789	0.06508	-0.04731	0.04731	0.81865	0.70524	0.91912	0.19945	0.40331	0.62269	0.39774	0.53891	0.53891	grey
CENPW	0.02333	0.0936	0.05107	0.00717	-0.10211	0.05943	0.26618	-0.34998	0.34998	0.76201	0.22337	0.50708	0.9258	0.18386	0.44003	0.00043	2.70E-06	2.70E-06	pink
MTM1	-0.07173	-0.14956	0.02511	0.03071	-0.04267	0.10002	0.00809	0.14681	-0.14681	0.35116	0.05089	0.74445	0.69007	0.57951	0.19307	0.91631	0.05535	0.05535	turquoise
ELF2	-0.08835	-0.21474	-0.06029	0.00534	-0.0396	0.02757	-0.07098	0.26596	-0.26596	0.2505	0.00479	0.43343	0.94475	0.60712	0.72035	0.35628	0.00044	0.00044	turquoise
TMIGD2	-0.06393	-0.04497	0.01262	0.00706	-0.091	0.07393	-0.02981	0.14857	-0.14857	0.40615	0.55922	0.86987	0.92695	0.23654	0.33657	0.69869	0.05247	0.05247	blue
NDUFAF1	-0.1529	-0.03999	-0.14302	-0.05909	-0.06606	-0.15167	-0.06667	0.17801	-0.17801	0.04587	0.60354	0.06203	0.44262	0.39064	0.04767	0.38628	0.01984	0.01984	grey
GDAP2	-0.03674	-0.13333	0.08737	0.14726	-0.12648	0.20108	0.06656	-0.05661	0.05661	0.63329	0.08212	0.25581	0.05461	0.09926	0.00836	0.3871	0.46205	0.46205	turquoise
GLS	0.04762	-0.21603	-0.01182	0.10429	-0.01558	-0.16707	-0.12061	0.29481	-0.29481	0.53628	0.00454	0.87802	0.17463	0.83967	0.02895	0.11609	9.07E-05	9.07E-05	turquoise
ADGRG1	0.13281	-0.08208	-0.04384	-0.01594	-0.00372	0.03384	0.02426	-0.31584	0.31584	0.08334	0.28583	0.56911	0.83606	0.96144	0.66035	0.75284	2.58E-05	2.58E-05	grey
NDUFA1	0.07592	-0.09509	-0.15449	-0.15154	0.12841	-0.1589	-0.03778	-0.24768	0.24768	0.32368	0.21605	0.04364	0.04787	0.09417	0.03791	0.62371	0.00109	0.00109	grey
SVIL	-0.04181	-0.11663	0.05371	0.11444	-0.1803	0.20466	0.08192	0.19704	-0.19704	0.58719	0.12874	0.48534	0.13613	0.01829	0.00725	0.28682	0.00979	0.00979	turquoise
CLCN4	-0.17612	-0.21891	-0.18176	-0.07851	-0.03039	-0.21332	-0.14691	0.37988	-0.37988	0.02121	0.00402	0.01735	0.3074	0.69311	0.00509	0.05519	2.98E-07	2.98E-07	brown
FLCN	-0.0196	-0.24387	-0.12255	0.01005	0.02643	-0.094	-0.0292	0.36047	-0.36047	0.79915	0.00131	0.1103	0.89616	0.73152	0.22137	0.70456	1.28E-06	1.28E-06	turquoise
MARCKSL	-0.02406	-0.02541	0.08428	-0.01774	0.09121	0.23533	0.07336	-0.3916	0.3916	0.7548	0.7415	0.27309	0.81788	0.23546	0.00195	0.34032	1.18E-07	1.18E-07	yellow
PRR5	-0.032	0.08984	0.00947	0.08136	0.16758	-0.04058	0.05216	-0.15404	0.15404	0.67775	0.2426	0.90213	0.2901	0.02847	0.59823	0.49805	0.04427	0.04427	grey
CLASRP	0.10862	-0.05703	-0.09641	-0.0916	0.21198	-0.0907	-0.00688	-0.2008	0.2008	0.15731	0.45876	0.20968	0.23343	0.00538	0.23808	0.92886	0.00845	0.00845	red
SMARCD3	0.05762	-0.18396	-0.25076	-0.02691	0.01651	-0.20338	-0.14889	0.36162	-0.36162	0.45408	0.01602	0.00094	0.72676	0.83028	0.00763	0.05195	1.18E-06	1.18E-06	brown
GPD1	-0.02244	-0.04511	-0.08466	-0.02596	0.21807	-0.10095	-0.15999	0.31191	-0.31191	0.77081	0.55796	0.27094	0.73614	0.00417	0.18894	0.0366	3.28E-05	3.28E-05	grey
UBE2O	-0.03418	-0.23417	-0.10976	-0.07171	0.12092	-0.23311	-0.12247	0.20254	-0.20254	0.65721	0.00205	0.15296	0.35134	0.11514	0.00215	0.11053	0.00789	0.00789	turquoise
KDR	-0.15148	-0.16092	-0.12709	0.00471	-0.09783	-0.08144	-0.14077	0.60444	-0.60444	0.04796	0.0355	0.09763	0.95129	0.20303	0.28965	0.06628	2.07E-18	2.07E-18	blue
GRIPAP1	0.05376	-0.17384	-0.12211	-0.07496	0.13267	-0.15995	-0.07002	-0.07233	0.07233	0.48497	0.02297	0.11161	0.32984	0.08366	0.03664	0.36283	0.34718	0.34718	red
FGL2	-0.10175	-0.10399	0.01891	0.05208	-0.16432	0.13514	0.03209	0.47993	-0.47993	0.18541	0.17587	0.80607	0.49871	0.03174	0.07802	0.67697	3.10E-11	3.10E-11	blue
SRGN	-0.14771	-0.09181	0.09721	0.08297	-0.22287	0.15114	0.03591	0.3976	-0.3976	0.05385	0.23234	0.20593	0.28066	0.00339	0.04847	0.64102	7.25E-08	7.25E-08	blue
C1orf74	-0.06945	-0.04307	-0.00959	-0.07379	-0.09133	-0.02228	0.06837	0.01231	-0.01231	0.36676	0.57591	0.90095	0.33745	0.23482	0.77236	0.37424	0.873	0.873	turquoise
GSTCD	-0.09402	-0.1578	-0.06279	0.00769	-0.03747	-0.02956	0.05091	0.07084	-0.07084	0.22127	0.03927	0.41454	0.9205	0.62659	0.70109	0.50843	0.35723	0.35723	turquoise
LIMD1	0.00402	-0.11364	-0.03757	0.02909	-0.09254	0.12206	0.04976	0.0783	-0.0783	0.9584	0.13887	0.62563	0.70561	0.22866	0.11176	0.51804	0.30871	0.30871	turquoise
CAPN7	-0.03716	-0.16383	-0.0638	-0.05882	-0.06465	0.0272	-0.02173	0.30983	-0.30983	0.62947	0.03226	0.40707	0.44476	0.40085	0.72399	0.77788	3.73E-05	3.73E-05	turquoise
EFNA1	0.07921	-0.02459	-0.03657	0.01154	0.16416	0.0954	0.07405	-0.49477	0.49477	0.30312	0.74956	0.63488	0.88094	0.03192	0.21453	0.33577	6.05E-12	6.05E-12	yellow
PDE12	-0.04999	-0.09666	-0.0656	-0.04376	-0.03172	0.08196	-0.03377	0.22668	-0.22668	0.51614	0.20851	0.39392	0.5698	0.6804	0.28657	0.66101	0.00287	0.00287	turquoise
FAM162A	0.06825	0.06524	-0.02298	-0.09403	0.05128	0.03341	0.0072	-0.44923	0.44923	0.37508	0.39658	0.76549	0.22122	0.50535	0.66441	0.9255	7.13E-10	7.13E-10	grey
SDR42E1	0.03892	-0.12128	-0.13736	-0.04567	-0.05944	0.07964	0.06655	-0.14904	0.14904	0.61332	0.11408	0.0732	0.55311	0.43998	0.30044	0.38713	0.0517	0.0517	turquoise
PLPP4	-0.02383	-0.03642	0.05149	0.08159	-0.17324	0.13433	0.09186	-0.03066	0.03066	0.75702	0.6363	0.50361	0.28874	0.02345	0.07983	0.2321	0.69057	0.69057	black
INHBB	-0.05506	-0.16833	0.03882	0.13966	-0.17281	0.1172	0.02857	0.12641	-0.12641	0.47445	0.02775	0.61419	0.06848	0.02381	0.12686	0.71072	0.09945	0.09945	black
SH3GL2	-0.03567	-0.10712	-0.26212	-0.13855	0.03391	-0.23387	-0.14331	0.46041	-0.46041	0.64324	0.16316	0.00053	0.07073	0.65976	0.00208	0.06149	2.36E-10	2.36E-10	brown
LSM4	0.1191	0.06328	-0.05858	-0.12056	0.15261	-0.1319	0.13088	-0.49217	0.49217	0.12079	0.41091	0.44661	0.11625	0.04629	0.0855	0.08796	8.10E-12	8.10E-12	green
INIP	-0.01908	-0.167	-0.07156	-0.01772	-0.08842	0.00956	-0.06161	0.27308	-0.27308	0.80433	0.02903	0.35234	0.81807	0.25012	0.90125	0.42345	0.0003	0.0003	turquoise
ZCCHC9	-0.01227	-0.06705	-0.01612	-0.00757	0.01623	0.02273	0.01732	-0.05228	0.05228	0.87347	0.38356	0.83427	0.92171	0.83316	0.76788	0.82205	0.49705	0.49705	turquoise
CXCR6	-0.10765	-0.03473	0.00388	0.08009	-0.19332	0.10474	0.02477	0.42112	-0.42112	0.16108	0.652	0.95984	0.29776	0.0113	0.17278	0.74773	9.72E-09	9.72E-09	blue
CTNND1	0.05291	-0.12178	0.06385	0.03488	-0.07357	0.2293	0.14686	-0.20817	0.20817	0.49192	0.11257	0.40672	0.65064	0.33895	0.00255	0.05527	0.00629	0.00629	turquoise
UBALD2	0.05708	-0.02631	-0.0519	-0.08186	0.01726	-0.00853	0.1657	-0.5729	0.5729	0.45835	0.73271	0.50019	0.28716	0.8227	0.91182	0.03032	2.66E-16	2.66E-16	green
TIMM8A	-0.03461	-0.01296	-0.01536	0.03649	-0.12496	0.12985	0.29117	-0.16972	0.16972	0.65317	0.86636	0.84196	0.63563	0.10341	0.09051	0.00011	0.02647	0.02647	turquoise
RAB10	-0.00384	-0.04222	-0.00058	0.06751	-0.06257	0.07616	0.13152	-0.0858	0.0858	0.96024	0.58348	0.99396	0.38031	0.41624	0.32215	0.0864	0.26453	0.26453	turquoise
GDPD5	-0.09069	-0.05482	0.03973	0.12523	-0.17333	0.05846	0.1043	0.04861	-0.04861	0.23814	0.47635	0.60594	0.10267	0.02338	0.44758	0.17457	0.52781	0.52781	grey
CKMT2	-0.11531	0.1522	-0.0202	0.05463	-0.04447	0.04112	-0.08936	0.2411	-0.2411	0.13313	0.04689	0.7931	0.4779	0.5636	0.59338	0.2451	0.00149	0.00149	grey
CYB5B	0.09304	0.04779	0.04468	0.02222	-0.07608	0.1525	0.2436	-0.30583	0.30583	0.22616	0.53482	0.56171	0.77303	0.32263	0.04646	0.00132	4.74E-05	4.74E-05	turquoise
ADM	0.00531	-0.15557	0.02927	0.09162	0.09055	0.07151	0.05974	-0.40432	0.40432	0.94509	0.04218	0.70388	0.23335	0.23888	0.35264	0.43764	4.15E-08	4.15E-08	grey
ABHD6	0.005	-0.10078	-0.06131	-0.10642	0.00192	-0.0678	-0.18633	0.2557	-0.2557	0.94825	0.18967	0.42571	0.16594	0.98016	0.37829	0.01468	0.00074	0.00074	turquoise
CD9	0.03603	-0.01132	0.12381	0.11654	-0.08008	0.22977	0.14406	-0.24816	0.24816	0.63992	0.88315	0.10667	0.12901	0.29781	0.0025	0.06014	0.00107	0.00107	grey
RAVER1	-0.04522	-0.07644	-0.10333	-0.04148	0.16966	-0.03906	-0.0586	-0.00942	0.00942	0.55697	0.32037	0.17865	0.59013	0.02653	0.61198	0.4465	0.90271	0.90271	grey
FAM167A	-0.07244	-0.14579	-0.17795	-0.0883	0.11461	-0.27499	-0.24155	0.35276	-0.35276	0.34644	0.05709	0.01988	0.2508	0.13553	0.00027	0.00146	2.22E-06	2.22E-06	brown
ZNF326	-0.01102	-0.2617	-0.04501	0.00132	0.12303	-0.06088	-0.09153	0.11868	-0.11868	0.88624	0.00055	0.55882	0.98628	0.1089	0.42893	0.23378	0.12209	0.12209	red
FOXD2	0.007	-0.00357	0.02367	-0.01648	0.08786	0.09497	0.04979	-0.30813	0.30813	0.92756	0.96299	0.75863	0.83058	0.25318	0.2166	0.51783	4.13E-05	4.13E-05	grey
ZBED9	-0.13048	-0.09962	-0.23527	-0.06878	0.05789	-0.16355	-0.04951	0.25219	-0.25219	0.08895	0.19486	0.00195	0.3714	0.45202	0.03256	0.5202	0.00088	0.00088	grey
OLFML2A	-0.12002	-0.1223	-0.10252	-0.01951	-0.11219	-0.12497	-0.03814	0.24195	-0.24195	0.1179	0.11105	0.18209	0.80006	0.14405	0.10339	0.62037	0.00143	0.00143	blue
ARMC5	-0.0004	-0.04402	-0.19909	-0.0946	0.1947	-0.20518	-0.07828	0.12317	-0.12317	0.99583	0.56755	0.00904	0.21843	0.01072	0.0071	0.30883	0.10852	0.10852	green
CAPRIN2	0.00579	-0.04765	0.00786	0.05798	0.03419	-0.03492	0.11384	-0.23144	0.23144	0.94011	0.53597	0.9187	0.45128	0.65709	0.65026	0.13818	0.00232	0.00232	red
C12orf4	-0.02748	-0.06724	-0.07047	-0.00392	-0.01571	-0.06206	-0.01864	0.33393	-0.33393	0.72123	0.38221	0.35971	0.95943	0.8384	0.42006	0.8088	8.06E-06	8.06E-06	turquoise
ZNF93	-0.02902	-0.05972	0.02089	0.08059	-0.11832	0.02835	-0.01173	0.26618	-0.26618	0.70629	0.43781	0.78625	0.29475	0.12324	0.71284	0.879	0.00043	0.00043	grey
MRPL14	0.04716	0.13484	-0.02022	-0.01522	0.01405	0.01815	0.27091	-0.46793	0.46793	0.54017	0.07868	0.79293	0.84337	0.85524	0.81376	0.00034	1.10E-10	1.10E-10	greenyellow
ELFN1	-0.14726	-0.06415	-0.16241	-0.00727	-0.01716	-0.14765	-0.02007	0.34279	-0.34279	0.05461	0.40454	0.03381	0.9248	0.82376	0.05396	0.79441	4.44E-06	4.44E-06	grey
DOK4	0.04629	0.01436	0.03376	0.01613	0.06446	0.16357	0.14296	-0.4128	0.4128	0.54768	0.85217	0.66115	0.83411	0.40227	0.03255	0.06214	2.01E-08	2.01E-08	yellow
ZNF525	0.0328	-0.05592	0.02059	0.01624	-0.06255	0.14225	0.0408	-0.04869	0.04869	0.67017	0.46758	0.78919	0.83299	0.41639	0.06345	0.59618	0.52711	0.52711	turquoise

ZC3HAV1l	0.00824	-0.17682	-0.01613	-0.00373	0.00589	-0.03378	0.14861	-0.00865	0.00865	0.91477	0.02069	0.83416	0.9614	0.93911	0.66093	0.0524	0.91063	0.91063	turquoise
VAMP4	-0.09025	-0.07949	-0.12657	-0.06803	0.07612	-0.10722	-0.13698	0.20411	-0.20411	0.24041	0.30139	0.09902	0.37665	0.32237	0.16276	0.07401	0.00741	0.00741	turquoise
VTCN1	-0.02634	-0.08174	0.00458	0.14634	-0.14807	0.13683	0.03484	0.0125	-0.0125	0.73234	0.28787	0.95262	0.05615	0.05327	0.07434	0.65098	0.87111	0.87111	magenta
RNF103	0.05551	-0.03852	0.01447	-0.0209	0.06472	0.17545	0.00616	-0.21546	0.21546	0.4708	0.61689	0.85101	0.78609	0.40033	0.02171	0.93626	0.00465	0.00465	grey
RIMS3	0.05946	0.02332	-0.01479	0.06853	0.01614	-0.07931	-0.08542	0.14344	-0.14344	0.43981	0.76212	0.84771	0.37311	0.83401	0.30245	0.26661	0.06125	0.06125	grey
ZNF554	-0.05942	-0.06609	-0.20942	-0.10633	0.22155	-0.23204	-0.24407	0.17924	-0.17924	0.4401	0.3904	0.00598	0.1663	0.00359	0.00226	0.0013	0.01899	0.01899	brown
CHST11	-0.08437	-0.06369	0.11648	0.11605	-0.267	0.06756	0.12848	0.18611	-0.18611	0.27257	0.40792	0.12923	0.13065	0.00042	0.37998	0.09398	0.0148	0.0148	black
RBMS1	-0.11623	-0.12305	0.04172	0.079	-0.21924	0.14562	0.04607	0.14996	-0.14996	0.13006	0.10886	0.58795	0.30441	0.00396	0.05738	0.54959	0.05027	0.05027	black
DAG1	-0.01812	-0.0532	0.05913	-0.02249	-0.0411	0.20499	-0.00227	-0.08864	0.08864	0.81401	0.48951	0.44239	0.77033	0.59355	0.00715	0.97653	0.24894	0.24894	turquoise
ARPIN	-0.09893	-0.13468	0.02582	0.06851	-0.16776	0.14758	0.05223	0.22491	-0.22491	0.19797	0.07904	0.73747	0.3733	0.02829	0.05407	0.49748	0.0031	0.0031	turquoise
FBXO17	-0.02639	-0.21553	-0.12499	0.05545	-0.07564	-0.14525	-0.07518	0.41273	-0.41273	0.73188	0.00464	0.10333	0.47132	0.32547	0.05802	0.32844	2.03E-08	2.03E-08	brown
GDAP1	0.00496	-0.03375	-0.21007	-0.11179	0.05984	-0.26619	-0.2489	0.38539	-0.38539	0.94864	0.66125	0.00582	0.14547	0.43691	0.00043	0.00103	1.94E-07	1.94E-07	brown
CDCA4	-0.02846	0.06253	0.06314	0.07289	-0.11263	0.07858	0.25113	-0.33769	0.33769	0.71174	0.41651	0.412	0.34344	0.14247	0.30695	0.00092	6.28E-06	6.28E-06	pink
RDX	-0.12674	-0.14767	-0.09201	0.05814	-0.18281	-0.05633	-0.03548	0.37055	-0.37055	0.09857	0.05392	0.23135	0.45007	0.0167	0.46427	0.64506	6.08E-07	6.08E-07	blue
CTSH	0.0625	-0.02162	0.06001	0.15954	-0.13564	0.21927	0.14086	-0.14107	0.14107	0.41671	0.77897	0.43557	0.03713	0.0769	0.00396	0.06611	0.06571	0.06571	yellow
FAM180A	-0.06037	-0.10589	0.03162	0.07752	-0.17473	0.13754	0.09144	0.49665	-0.49665	0.43286	0.16808	0.68137	0.31358	0.02227	0.07283	0.23425	4.89E-12	4.89E-12	blue
GPT	-0.04927	0.10792	-0.01933	-0.06781	0.17913	0.09998	0.02747	-0.30455	0.30455	0.52223	0.16001	0.8019	0.37816	0.01906	0.19324	0.72139	5.12E-05	5.12E-05	grey
FLRT3	0.0911	-0.07188	0.08036	0.16803	-0.05907	0.04802	0.12406	-0.15665	0.15665	0.236	0.35018	0.29613	0.02804	0.4428	0.53286	0.10597	0.04075	0.04075	grey
COPA	-0.02995	-0.09995	-0.01715	-0.03586	-0.06495	0.10232	0.06798	0.01907	-0.01907	0.69738	0.19337	0.82377	0.64151	0.39868	0.18296	0.37702	0.80449	0.80449	turquoise
FRMD4B	-0.05172	-0.11961	0.07703	0.11161	-0.04898	0.21704	0.07828	0.01272	-0.01272	0.50167	0.11917	0.31662	0.14614	0.52465	0.00435	0.3088	0.86883	0.86883	turquoise
CEP250	0.01448	-0.15153	-0.06426	0.0005	0.01365	-0.11318	-0.02353	0.11526	-0.11526	0.85089	0.04788	0.40373	0.99484	0.85935	0.14052	0.76001	0.1333	0.1333	turquoise
PPFIA2	0.02846	-0.14592	0.0416	0.06299	-0.2482	0.17109	0.12705	0.37393	-0.37393	0.7117	0.05687	0.58901	0.41309	0.00106	0.02526	0.09774	4.71E-07	4.71E-07	blue
GK5	0.01358	-0.10256	0.01341	0.02525	0.07324	0.1364	0.06203	-0.12861	0.12861	0.86008	0.18193	0.86177	0.74303	0.3411	0.07525	0.42028	0.09366	0.09366	turquoise
NTS	-0.0038	-0.04325	-0.08399	-0.19899	0.02009	-0.05604	-0.11947	0.20062	-0.20062	0.96071	0.57436	0.27472	0.00908	0.79419	0.46659	0.1196	0.00851	0.00851	grey
TIGD3	0.00111	-0.15301	-0.24328	-0.11066	0.13369	-0.33129	-0.15986	0.02114	-0.02114	0.98848	0.04572	0.00134	0.14962	0.08129	9.60E-06	0.03675	0.78378	0.78378	grey
IL22RA1	0.07624	0.1093	0.15869	0.16672	0.01322	0.28426	0.03251	-0.16687	0.16687	0.32162	0.1547	0.03817	0.0293	0.86375	0.00017	0.67298	0.02915	0.02915	grey
RAB13	0.016	-0.02921	0.04316	0.00939	-0.01949	0.15042	0.18996	-0.14833	0.14833	0.83546	0.70454	0.57513	0.90298	0.80026	0.04957	0.01283	0.05285	0.05285	greenyellow
CYB5R3	-0.0641	-0.07396	-0.09504	0.01269	-0.01459	-0.00919	-0.12271	0.17116	-0.17116	0.40491	0.33637	0.21626	0.86911	0.84974	0.90504	0.10984	0.0252	0.0252	black
PELO	-0.00185	-0.16367	-0.04769	-0.05089	-0.05455	-0.02271	-0.03266	0.11069	-0.11069	0.98088	0.03243	0.53567	0.50859	0.47856	0.76808	0.67147	0.1495	0.1495	turquoise
CEP57	-0.06516	-0.10268	-0.00943	-0.01064	-0.03302	0.07706	0.07496	-0.00559	0.00559	0.39717	0.18144	0.90254	0.89014	0.66813	0.31642	0.32985	0.94214	0.94214	turquoise
SRP14	-0.08201	-0.13227	-0.09764	-0.07128	-0.05786	-0.06319	-0.076	-0.00315	0.00315	0.28625	0.08461	0.2039	0.35423	0.45222	0.41162	0.32314	0.96741	0.96741	turquoise
THSD7A	-0.02743	-0.07913	-0.08928	0.03339	-0.19024	0.05761	0.03755	0.51518	-0.51518	0.72176	0.30361	0.24555	0.66462	0.01269	0.45418	0.62584	5.61E-13	5.61E-13	blue
CPA6	-0.14014	0.05177	0.12526	0.12061	-0.02565	0.24863	-0.04909	-0.05961	0.05961	0.06753	0.5013	0.1026	0.1161	0.73914	0.00104	0.52369	0.43866	0.43866	grey
SNRPB2	-0.04118	-0.04921	-0.06513	-0.03273	-0.00685	-0.07381	0.11721	-0.06979	0.06979	0.59277	0.52274	0.39738	0.67081	0.9291	0.33733	0.12684	0.36436	0.36436	turquoise
ARSA	-0.07385	0.04338	0.02085	0.06446	0.10363	-0.14649	-0.05741	0.00706	-0.00706	0.33707	0.57315	0.7866	0.40226	0.17739	0.0559	0.45577	0.92696	0.92696	grey
RARRES2	-0.12391	0.02301	0.07205	0.12327	-0.19659	0.16976	0.148	0.18303	-0.18303	0.10639	0.76519	0.34903	0.10821	0.00996	0.02643	0.05338	0.01657	0.01657	black
CABLES2	0.00408	-0.07323	-0.00085	0.00114	0.00428	0.09511	0.10385	-0.18466	0.18466	0.95775	0.34117	0.99115	0.98824	0.95567	0.21594	0.17645	0.01561	0.01561	grey
SLC37A2	0.00457	-0.04305	0.08599	0.06193	-0.29968	0.13431	0.2099	0.17632	-0.17632	0.95269	0.57611	0.26344	0.42101	6.84E-05	0.07989	0.00586	0.02106	0.02106	blue
FAM126A	-0.11386	-0.13791	-0.01161	0.07957	-0.13874	0.01262	-0.04557	0.47297	-0.47297	0.13812	0.07205	0.88023	0.30089	0.07033	0.8699	0.55397	6.48E-11	6.48E-11	blue
ZMYM1	-0.0752	-0.17424	0.01733	0.03527	-0.06503	0.05396	0.0661	0.08512	-0.08512	0.3283	0.02265	0.82198	0.64701	0.3981	0.48331	0.39038	0.26834	0.26834	turquoise
RAMP1	-0.00624	0.00593	-0.02723	0.05372	0.05723	0.12464	-0.05145	-0.07991	0.07991	0.9354	0.93861	0.72369	0.48525	0.45722	0.10433	0.50395	0.29883	0.29883	grey
ETFDH	-0.06608	-0.10837	-0.04778	-0.02671	0.15135	-0.10896	-0.12822	0.16329	-0.16329	0.39053	0.15826	0.53485	0.72878	0.04814	0.15601	0.09466	0.03284	0.03284	turquoise
PSD	0.01373	-0.13313	-0.19704	-0.07968	0.19896	-0.28825	-0.21774	0.35869	-0.35869	0.85856	0.08259	0.00979	0.3002	0.00909	0.00013	0.00422	1.45E-06	1.45E-06	brown
TMA16	-0.08474	0.01549	0.02414	-0.01947	-0.03525	0.11784	0.13395	-0.11298	0.11298	0.27045	0.8406	0.75393	0.80049	0.6472	0.12478	0.0807	0.14122	0.14122	turquoise
DIDO1	0.05378	-0.12481	-0.0702	-0.04938	0.032	-0.03285	0.00934	0.00902	-0.00902	0.48481	0.10383	0.36157	0.5213	0.67775	0.66972	0.90345	0.90677	0.90677	red
FEV	-0.01289	-0.10459	-0.27621	-0.22207	0.08892	-0.31322	-0.18437	0.33037	-0.33037	0.86711	0.17339	0.00026	0.00351	0.24743	3.03E-05	0.01578	1.02E-05	1.02E-05	brown
KCND3	-0.09105	-0.09384	-0.12643	-0.00258	-0.03022	0.00269	-0.15259	0.47257	-0.47257	0.23628	0.22214	0.09939	0.97332	0.69481	0.9721	0.04633	6.76E-11	6.76E-11	brown
SUOX	-0.01142	-0.15787	-0.1734	-0.09009	0.17668	-0.27445	-0.23011	0.27743	-0.27743	0.8822	0.03918	0.02332	0.24127	0.0208	0.00028	0.00246	0.00024	0.00024	turquoise
FAM43B	-0.09111	-0.11777	-0.16053	0.05273	-0.11935	-0.03845	-0.05859	0.36004	-0.36004	0.23595	0.12501	0.03595	0.49335	0.11999	0.61755	0.44653	1.32E-06	1.32E-06	blue
NR3C1	-0.10473	-0.17592	-0.03342	0.06469	-0.2009	0.02021	-0.04781	0.38382	-0.38382	0.17282	0.02136	0.6643	0.40056	0.00842	0.79303	0.53465	2.19E-07	2.19E-07	blue
CCN3	0.03521	-0.06614	-0.0446	0.14708	-0.04922	-0.00752	-0.11041	0.35338	-0.35338	0.64749	0.39009	0.56242	0.0549	0.52259	0.92227	0.15056	2.13E-06	2.13E-06	blue
FILIP1	-0.12027	-0.05644	-0.00671	0.05594	-0.18958	0.07981	-0.06141	0.5869	-0.5869	0.11715	0.46342	0.9306	0.46744	0.01301	0.29943	0.42491	3.29E-17	3.29E-17	blue
ATRX	-0.0534	-0.12261	-0.03875	0.00542	-0.15174	0.05762	-0.02375	0.27883	-0.27883	0.48788	0.11014	0.61479	0.94392	0.04757	0.45414	0.75781	0.00022	0.00022	turquoise
SCAF8	-0.06513	-0.08264	0.00061	0.02444	-0.13483	0.0695	-0.02107	0.33699	-0.33699	0.39737	0.28256	0.99367	0.75101	0.0787	0.36641	0.78446	6.58E-06	6.58E-06	turquoise
ABHD12	0.16503	-0.11821	-0.07072	0.06399	0.0757	-0.21736	-0.05162	-0.15328	0.15328	0.031	0.12359	0.35803	0.40571	0.32509	0.00429	0.50251	0.04534	0.04534	grey
PYGL	-0.02887	-0.08628	0.09237	0.20189	-0.12059	0.05237	0.12722	0.04865	-0.04865	0.70782	0.26183	0.22949	0.0081	0.11615	0.49636	0.09727	0.52747	0.52747	black
CACYBP	0.10752	-0.07847	0.00692	-0.05184	0.01085	0.01212	0.11157	-0.203	0.203	0.16157	0.30763	0.92841	0.50074	0.88802	0.87499	0.14628	0.00775	0.00775	greenyellow
MPP6	0.08397	-0.08611	-0.14926	0.01183	0.00325	-0.16519	-0.02572	0.39679	-0.39679	0.27487	0.2628	0.05136	0.87797	0.96638	0.03084	0.73846	7.75E-08	7.75E-08	brown
SNCAIP	-0.02133	-0.22117	-0.10954	0.07119	-0.08756	0.02165	0.01699	0.2012	-0.2012	0.78184	0.00365	0.15382	0.35484	0.25477	0.77866	0.82543	0.00832	0.00832	grey
LIMA1	-0.01647	-0.08723	0.16176	0.06611	-0.01229	0.23207	0.10892	-0.10559	0.10559	0.8307	0.25659	0.03454	0.39026	0.87323	0.00226	0.15617	0.1693	0.1693	turquoise
LIMK2	-0.12165	-0.03712	0.00603	-0.00593	-0.08685	0.10642	0.07382	0.01756	-0.01756	0.11296	0.62981	0.93758	0.93869	0.25868	0.16592	0.33729	0.81966	0.81966	turquoise
PHF8	0.03271	-0.2095	-0.06824	0.00631	-0.05901	-0.01834	-0.04954	0.15768	-0.15768	0.671	0.00596	0.37519	0.93467	0.44332	0.8118	0.51992	0.03943	0.03943	turquoise
DNAJC7	0.01759	-0.16568	-0.14938	-0.05257	-0.00614	-0.19145	-0.01282	-0.04212	0.04212	0.81941	0.03034	0.05118	0.49467	0.93648	0.01213	0.86781	0.58435	0.58435	turquoise
RGCC	-0.13389	-0.177	0.01008	0.01491	-0.04689	0.04471	-0.06745	0.31423	-0.31423	0.08083	0.02056	0.89587	0.84652	0.54253	0.56146	0.38075	2.85E-05	2.85E-05	grey
DDX39A	0.04756	0.09318	-0.0598	-0.06203	0.06327	-0.08483	0.09981	-0.2873	0.2873	0.53673	0.22545	0.43719	0.42029	0.41103	0.26999	0.19399	0.00014	0.00014	grey
RABGAP1l	0.00705	-0.0696	-0.00138	-0.03282	-0.04842	0.12208	0.05254	-0.06364	0.06364	0.92706	0.36571	0.98575	0.67004	0.52945	0.11169	0.49492	0.40828	0.40828	turquoise
METTL9	-0.00767	-0.0657	0.04498	0.01283	-0.18502	0.09265	0.06165	-0.03931	0.03931	0.92073	0.39324	0.55909	0.86775	0.01541	0.2281	0.42315	0.6097	0.6097	turquoise
UBA6	-0.04625	-0.12055	0.04385	0.01789	-0.0729	0.03091	0.1232	-0.17189	0.17189	0.54806	0.11627	0.56905	0.81637	0.34338	0.68815	0.10841	0.02457	0.02457	turquoise
FUZ	-0.01369	0.02854	-0.08601	-0.07326	0.05711	-0.05979	-0.02603	-0.17543	0.17543	0.85895	0.71099	0.26333	0.34095	0.45813	0.4373	0.73537	0.02173	0.02173	grey
ATP6V1G2	-0.06643	-0.15269	-0.20016	-0.13536	0.04958	-0.28426	-0.21512	0.61582	-0.61582	0.38802	0.04618	0.00867	0.07753	0.5196	0.00016	0.00472	3.14E-19	3.14E-19	brown
C11orf95	-0.12419	-0.14689	-0.07895	0.07056	-0.13857	-0.01872	-0.03936	0.58534	-0.58534	0.10557	0.05522	0.30469	0.35911	0.07069	0.80803	0.60926	4.17E-17	4.17E-17	blue
ZBTB38	-0.07104	-0.1479	-0.01403	0.06977	-0.17689	0.11569	0.02257	0.23945	-0.23945	0.35584	0.05354	0.85553	0.36452	0.02064	0.13187	0.76953	0.00161	0.00161	turquoise
SMAD7	-0.11339	-0.19327	-0.05143	0.05541	-0.0988	0.01237	-0.07165	0.36855	-0.36855	0.13976	0.01132	0.50407	0.47167	0.19858	0.87247	0.35169	7.06E-07	7.06E-07	turquoise
NR1D1	0.12346	-0.06012	0.06167	-0.02116	0.00347	-0.00299	0.02777	-0.3167	0.3167	0.10765	0.43477	0.42299	0.78356	0.96412	0.96906	0.71841	2.44E-05	2.44E-05	grey
RTL8B	-0.09501	-0.11566	-0.08966	0.06432	-0.16742	-0.06697	0.00461	0.28527	-0.28527	0.21642	0.13197	0.24354	0.4033	0.02861	0.38411	0.95227	0.00016	0.00016	blue
ZHX1	-0.00665	-0.1237	-0.05343	0.06128	-0.08202	-0.01805	0.00056	0.28944	-0.28944	0.93126	0.10697	0.48764	0.42594	0.2862	0.81478	0.99418	0.00012	0.00012	turquoise
CCDC40	-0.00162	-0.22275	-0.17268	-0.01909	0.17095	-0.16588	-0.10653	0.15414	-0.15414	0.98323	0.00341	0.02391	0.80431	0.02538	0.03013	0.16552	0.04413	0.04413	grey
KLK6	0.02786	0.03739	0.0125	0.00969	-0.04776	0.0625	0.12359	-0.1733	0.1733	0.71753	0.62729	0.87106	0.8999	0.53504	0.41676	0.10729	0.0234	0.0234	grey
ASB13	-0.05104	0.00294	-0.13454	-0.12796	-0.02914	-0.11954	0.08031	0.16174	-0.16174	0.50736	0.96954	0.07935	0.09535	0.70516	0.11941	0.29639	0.03456	0.03456	grey
PTGER4	-0.09202	-0.13357	0.12682	-0.00113	-0.0706	0.16428	-0.01155	0.34994	-0.34994	0.23128	0.08157	0.09835	0.98827	0.35883	0.03179	0.88083	2.71E-06	2.71E-06	blue
LSMEM1	-0.01998	-0.14447	-0.06555	0.12015	0.18512	-0.11409	-0.01092	-0.18674	0.18674	0.7953	0.05941	0.39435	0.1175	0.01535	0.13732	0.88727	0.01446	0.01446	grey
KPNA2	-0.04559	-0.04888	-0.02645	-0.06503	-0.0733	-0.044	0.17378	-0.11296	0.11296	0.5538	0.52554	0.73133	0.39806	0.34072	0.56769	0.02302	0.14129	0.14129	pink
RSBN1	-0.08251	-0.16196	-0.02376	0.03004	-0.07059	0.06447	-0.1562	0.32184	-0.32184	0.2833	0.03431	0.75775	0.69647	0.35887	0.40217	0.04133	1.77E-05	1.77E-05	turquoise
API5	-0.05922	-0.10157	-0.01786	-0.02853	-0.13885	0.01936	0.03008	0.15435	-0.15435	0.44169	0.18622	0.81667	0.71104	0.07012	0.80153	0.69612	0.04383	0.04383	turquoise
RRM2B	-0.08114	-0.11235	-0.06482	0.02369	-0.14741	-0.14431	-0.11045	0.32676	-0.32676	0.29141	0.14346	0.3996	0.75846	0.05435	0.05968	0.15039	1.29E-05	1.29E-05	turquoise
LRBA	-0.06841	-0.10259	-0.06752	-0.05657	-0.00729	-0.00409	-0.0943	0.11021	-0.11021	0.374	0.18178	0.38026	0.46237	0.92462	0.95762	0.21987	0.1513	0.1513	turquoise
ARL6IP1	-0.03087	-0.04415	0.10778	0.03756	-0.01528	0.18487	0.09978	-0.3618	0.3618	0.68853	0.56636	0.16055	0.62578	0.84276	0.01549	0.19413	1.16E-06	1.16E-06	turquoise
EEA1	-0.04868	-0.15295	-0.01866	0.03361	-0.11138	0.00309	-0.0299	0.39313	-0.39313	0.52717	0.04581	0.80861	0.66251	0.14697	0.96801	0.69785	1.04E-07	1.04E-07	turquoise
AC093323	0.06734	-0.10881	0.04177	0.05423	-0.0251	-0.0607	0.08995	-0.06691	0.06691	0.38153	0.15657	0.58754	0.48115	0.74453	0.43032	0.24202	0.38454	0.38454	grey
TENM4	0.03297	-0.08095	0.00905	-0.01954	-0.1956	-0.06434	-0.00784	0.41654	-0.41654	0.66861	0.29257	0.90646	0.79973	0.01035	0.4031	0.91893	1.46E-08	1.46E-08	blue
PDGFRB	-0.07945	-0.1089	0.02277	0.0523	-0.21167	0.14847	0.02635	0.2814	-0.2814	0.30162	0.15625	0.76752	0.49694	0.00545	0.05263	0.73232	0.00019	0.00019	black
APOBR	-0.04446	0.00816	0.10677	0.04788	-0.08484	0.18891	0.07978	0.18963	-0.18963	0.56368	0.91559	0.16453	0.53403	0.26993	0.01334	0.2996	0.01299	0.01299	blue
SUPT6H	-0.01429	-0.18555	-0.04124	-0.02553	-0.01111	-0.02468	0.00851	0.11834	-0.11834	0.8528	0.01511	0.59225	0.74031	0.88528	0.74866	0.91206	0.12318	0.12318	turquoise
CYTH1	-0.0569	-0.09528	0.11983	0.03765	-0.0172	0.13648	0.08379	-0.03836	0.03836	0.45978	0.2151	0.11849	0.62495	0.82332	0.07509	0.27592	0.6184	0.6184	grey
TERF2IP	-0.01088	-0.19481	-0.19597	-0.12699	0.03162	-0.25123	-0.24506	0.36641	-0.36641	0.88766	0.01067	0.0102	0.09789	0.68143	0.00092	0.00124	8.28E-07	8.28E-07	brown
ZIK1	-0.15003	-0.21073	-0.12451	-0.01494	-0.10491	-0.05581	-0.13927	0.57115	-0.57115	0.05015	0.00566	0.10468	0.84624	0.17209	0.46848	0.06927	3.43E-16	3.43E-16	blue
PNMA2	0.04558	-0.16876	-0.112	-0.04429	0.02172	-0.18108	-0.16338	0.27415	-0.27415	0.55384	0.02735	0.1447	0.56515	0.77794	0.01778	0.03275	0.00029	0.00029	brown
RBMX	-0.0088	-0.07407	-0.07164	-0.07341	-0.05995	0.07298	0.04202	0.08559	-0.08559	0.90906	0.33567	0.35178	0.33996	0.43605	0.34284	0.58531	0.26565	0.26565	turquoise
PLAC9	-0.01456	-0.04128	-0.06685	0.02561	0.00155	-0.01959	-0.1213	0.35087	-0.35087	0.85007	0.59194	0.38496	0.73952	0.98391	0.79927	0.114	2.54E-06	2.54E-06	blue
MDH1	-0.02393	-0.00469	-0.08752	-0.07668	0.00092	-0.08546	0.00971	-0.0148	0.0148	0.75601	0.95147	0.25502	0.31881	0.99047	0.26642	0.89967	0.84764	0.84764	turquoise
BRCA1	-0.05179	0.12324	-0.15831	-0.09173	-0.03792	-0.06532	0.18952	-0.05344	0.05344	0.50115	0.1083	0.03863	0.23278	0.62241	0.39598	0.01304	0.48757	0.48757	pink
KLF5	0.07801	0.08508	0.12617	0.05323	-0.01656	0.26351	0.26808	-0.45745	0.45745	0.31052	0.26856	0.1001	0.48926	0.82975	0.0005	0.00039	3.18E-10	3.18E-10	yellow
SH3GL1	-0.0014	-0.06129	-0.03627	0.01319	-0.03074	0.04326	0.13803	-0.12451	0.12451	0.98547	0.42584	0.63767	0.8641	0.68976	0.57427	0.07181	0.10469	0.10469	black
ICAM3	-0.11759	-0.05225	0.0628	0.03589	-0.1542	0.12078	-0.05705	0.4177	-0.4177	0.1256	0.4973	0.41452	0.64119	0.04404	0.11559	0.45862	1.32E-08	1.32E-08	blue
THOC6	0.05578	0.03113	0.01199	0.03291	0.07634	0.01748	0.14049	-0.34326	0.34326	0.46867	0.68606	0.87629	0.66913	0.32102	0.82047	0.06684	4.30E-06	4.30E-06	green
VANGL1	-0.06956	-0.06495	0.06686	0.10801	-0.19741	0.19886	0.16319	-0.13254	0.13254	0.36595	0.39871	0.3849	0.15967	0.00965	0.00912	0.03295	0.08396	0.08396	turquoise
RPL11	0.09282	0.09357	-0.00058	-0.04438	-0.10483	0.15358	0.07273	-0.07005	0.07005	0.22722	0.22351	0.99399	0.56435	0.1724	0.0449	0.3445	0.36261	0.36261	purple
NFE4	-0.0613	0.03393	0.1111	0.09002	-0.05843	0.04749	0.12804	-0.1796	0.1796	0.42578	0.65955	0.148	0.24162	0.44777	0.53736	0.09513	0.01875	0.01875	grey
DENND10	0.03241	-0.08411	0.05743	0.02583	-0.127	0.03731	0.21628	-0.09585	0.09585	0.67387	0.27407	0.45563	0.73735	0.09786	0.62806	0.00449	0.21235	0.21235	turquoise
IL11	-0.13671	-0.03289	-0.04446	0.05339	-0.05267	-0.00528	0.18878	-0.02068	0.02068	0.07459	0.66935	0.56367	0.48799	0.49388	0.94539	0.01341	0.7883	0.7883	grey
SLC25A40	-0.03616	-0.14476	0.00828	0.01114	-0.0167	0.06302	-0.01638	0.07553	-0.07553	0.63865	0.05888	0.91444	0.88497	0.82841	0.41285	0.83163	0.3262	0.3262	turquoise
HBP1	-0.04128	-0.16057	0.01645	0.04255	-0.14894	0.12299	0.06527	0.17243	-0.17243	0.5919	0.03591	0.83094	0.58053	0.05187	0.10904	0.39633	0.02412	0.02412	turquoise
ARHGEF18	0.08268	-0.1169	-0.00406	0.01864	-0.01593	0.07763	0.02682	-0.12402	0.12402	0.28233	0.12784	0.95799	0.80875	0.83618	0.31289	0.72765	0.10606	0.10606	turquoise
EPHB3	0.03199	0.21119	0.02951	0.04495	0.04795	0.18132	0.20135	-0.1737	0.1737	0.67792	0.00556	0.70164	0.5594	0.53343	0.01762	0.00827	0.02308	0.02308	grey
LDB1	-0.04761	-0.12002	-0.02515	-0.02182	-0.03582	-0.04383	-0.03728	0.31048	-0.31048	0.53637	0.11791	0.74401	0.77697	0.64183	0.56918	0.62829	3.58E-05	3.58E-05	turquoise
SLC23A2	-0.03627	-0.12112	-0.19558	-0.1007	0.07644	-0.25077	-0.18931	0.46552	-0.46552	0.63768	0.11457	0.01036	0.19004	0.32038	0.00094	0.01315	1.40E-10	1.40E-10	turquoise
IDO1	-0.02308	0.04694	-0.02158	0.00032	-0.17006	0.04022	0.13844	0.06068	-0.06068	0.76441	0.54213	0.77939	0.99668	0.02617	0.60149	0.07095	0.43048	0.43048	blue
MT-CO2	0.02118	-0.02679	-0.05721	-0.1211	0.26339	-0.1193	-0.09014	-0.28028	0.28028	0.78339	0.72801	0.45735	0.11463	0.0005	0.12014	0.24099	0.0002	0.0002	grey
R3HCC1L	0.07346	-0.10941	0.00757	0.01536	-0.05913	0.07794	0.14745	-0.07967	0.07967	0.33962	0.1543	0.92169	0.84194	0.44238	0.31094	0.05429	0.30027	0.30027	turquoise
COL23A1	-0.17321	-0.02525	-0.03376	0.06907	-0.0216	0.03306	0.00536	0.37827	-0.37827	0.02348	0.74309	0.66115	0.36939	0.77914	0.6677	0.94454	3.38E-07	3.38E-07	blue
YAF2	-0.01924	-0.18151	-0.06449	-0.02472	-0.01845	-0.08854	-0.1245	0.30042	-0.30042	0.80274	0.0175	0.402	0.74822	0.81072	0.24952	0.1047	6.54E-05	6.54E-05	turquoise
MAP4	0.01925	-0.0663	0.02882	0.03983	-0.19162	0.18424	0.08582	0.13786	-0.13786	0.80267	0.38896	0.70831	0.60499	0.01205	0.01586	0.26438	0.07216	0.07216	turquoise
GABBR1	0.02119	-0.08737	-0.13491	-0.04986	0.1641	-0.2275	-0.1091	0.12866	-0.12866	0.78322	0.25584	0.07852	0.51722	0.03198	0.00277	0.15546	0.09353	0.09353	red
CCDC25	-0.0905	0.00988	0.03492	-0.00467	-0.03384	0.00044	0.0241	0.22141	-0.22141	0.23912	0.89794	0.65028	0.95163	0.66034	0.99547	0.7544	0.00361	0.00361	turquoise
PSMD2	-0.06817	-0.07351	-0.09702	-0.04119	-0.16057	-0.02951	0.22525	-0.0626	0.0626	0.37568	0.33935	0.2068	0.59272	0.0359	0.70164	0.00306	0.41596	0.41596	turquoise
ITGAX	-0.12158	-0.02293	0.07442	0.09551	-0.1545	0.12522	0.06422	0.25077	-0.25077	0.11317	0.76597	0.33333	0.21399	0.04363	0.1027	0.404	0.00094	0.00094	blue
PEG10	-0.0061	-0.20396	-0.15605	-0.04655	-0.03647	-0.19675	-0.05038	0.32032	-0.32032	0.93686	0.00746	0.04153	0.54548	0.63577	0.0099	0.51284	1.95E-05	1.95E-05	brown
CYB5R4	-0.10132	-0.0466	-0.04238	-0.01966	-0.10507	0.02396	-0.03838	0.32363	-0.32363	0.1873	0.54502	0.58206	0.79854	0.17139	0.75577	0.61825	1.58E-05	1.58E-05	turquoise
KIF13A	-0.09312	-0.08715	0.00138	0.02294	-0.03427	0.04544	0.00684	0.31574	-0.31574	0.22574	0.25704	0.98575	0.76582	0.65631	0.55512	0.9293	2.59E-05	2.59E-05	turquoise

SRP72	-0.0711	-0.03039	0.04391	0.04252	-0.10875	0.04405	0.13253	0.19733	-0.19733	0.35546	0.69319	0.5685	0.58079	0.1568	0.56729	0.08399	0.00968	0.00968	turquoise
TRPM7	-0.08297	-0.1411	-0.05413	0.011	-0.09926	0.02705	-0.02901	0.25988	-0.25988	0.28064	0.06566	0.48191	0.88645	0.19649	0.72545	0.70642	0.0006	0.0006	turquoise
CAMP	-0.10443	0.04636	0.11262	0.06366	-0.06759	0.13611	-0.05363	0.14981	-0.14981	0.17406	0.5471	0.14249	0.40815	0.37973	0.07588	0.48603	0.05049	0.05049	blue
CEP63	0.03599	-0.12961	-0.17354	-0.1112	-0.05928	0.01422	-0.00554	0.21957	-0.21957	0.64026	0.09112	0.02321	0.14762	0.44117	0.85353	0.94263	0.00391	0.00391	turquoise
MCC	-0.05064	-0.16573	-0.03584	0.06503	-0.22596	-0.08702	-0.02974	0.47395	-0.47395	0.51056	0.03029	0.64163	0.39809	0.00296	0.25773	0.69936	5.85E-11	5.85E-11	blue
REN	0.13015	-0.11953	-0.01742	-0.01424	-0.04267	-0.01868	0.01234	-0.03243	0.03243	0.08977	0.11943	0.82113	0.85334	0.57951	0.80843	0.8727	0.67367	0.67367	grey
APOBEC3I	0.03159	-0.02381	0.05365	0.06452	-0.07604	0.09341	0.05978	0.00143	-0.00143	0.68164	0.75719	0.48588	0.40183	0.32289	0.22428	0.43733	0.98524	0.98524	blue
AMT	-0.02857	-0.03865	-0.07594	-0.09581	0.03298	0.06917	0.0488	-0.02785	0.02785	0.71066	0.61574	0.32358	0.21258	0.66849	0.36868	0.52617	0.71763	0.71763	grey
ABO	-0.07008	0.04863	0.10384	0.0435	-0.01547	0.23615	0.17121	-0.34971	0.34971	0.36239	0.52761	0.1765	0.57211	0.84084	0.00187	0.02516	2.75E-06	2.75E-06	yellow
UFD1	-0.05397	-0.03599	-0.08949	-0.05262	0.00668	-0.17214	0.04239	-0.07818	0.07818	0.48322	0.64025	0.24442	0.49427	0.93088	0.02436	0.58194	0.30945	0.30945	grey
GPRIN2	-0.05546	0.03946	-0.02338	0.15321	0.11809	0.13433	0.1251	-0.29886	0.29886	0.47124	0.60838	0.76149	0.04544	0.12399	0.07982	0.10304	7.17E-05	7.17E-05	grey
CYB561A3	-0.13795	-0.04499	-0.00316	-0.00754	-0.14505	0.02166	-0.03348	0.35208	-0.35208	0.07197	0.55903	0.96725	0.922	0.05837	0.77854	0.66375	2.33E-06	2.33E-06	blue
MXRA5	-0.13235	-0.07509	0.10334	0.12848	-0.26275	0.23596	0.18994	0.06725	-0.06725	0.08441	0.32904	0.17862	0.09399	0.00052	0.00189	0.01284	0.38214	0.38214	black
ITGAV	-0.05056	-0.15453	0.06833	0.1386	-0.24055	0.21091	0.13813	0.15769	-0.15769	0.51135	0.04359	0.37453	0.07064	0.00153	0.00562	0.0716	0.03941	0.03941	turquoise
SDC1	-0.0155	0.05294	0.0684	0.0256	-0.03061	0.21578	0.25067	-0.35299	0.35299	0.84057	0.49169	0.37402	0.73957	0.69103	0.00459	0.00094	2.19E-06	2.19E-06	yellow
KAT6A	-0.02614	-0.1498	-0.05158	0.06231	-0.13102	0.03719	0.02508	0.27512	-0.27512	0.73429	0.05052	0.50284	0.41817	0.08761	0.62918	0.74471	0.00027	0.00027	turquoise
RAET1G	0.07195	0.0566	0.02225	0.08236	-0.1351	-0.09007	0.14677	-0.12702	0.12702	0.34969	0.46214	0.77265	0.28422	0.07811	0.24135	0.05542	0.09782	0.09782	grey
ONECUT3	-0.02479	0.09305	0.09333	-0.01292	-0.01185	0.15954	0.0784	-0.36279	0.36279	0.74753	0.2261	0.2247	0.8668	0.8777	0.03713	0.30811	1.08E-06	1.08E-06	grey
TMEM38A	0.05915	-0.02117	0.00116	0.00089	0.0651	0.08059	0.05358	-0.37919	0.37919	0.44222	0.78345	0.98803	0.9908	0.39757	0.29475	0.48644	3.15E-07	3.15E-07	grey
FSD1L	-0.03769	-0.1832	-0.19998	-0.02844	0.00677	-0.25398	-0.18721	0.33057	-0.33057	0.62459	0.01646	0.00873	0.71198	0.93002	0.0008	0.01421	1.01E-05	1.01E-05	brown
HDAC9	-0.03128	-0.09853	0.00372	0.0228	-0.17444	0.04681	0.03986	0.24455	-0.24455	0.68464	0.19981	0.96148	0.76724	0.02249	0.54318	0.60473	0.00127	0.00127	turquoise
KIAA0319	0.02754	-0.148	-0.26127	-0.14319	0.09271	-0.30748	-0.18283	0.40635	-0.40635	0.72064	0.05339	0.00056	0.06172	0.22778	4.30E-05	0.01669	3.50E-08	3.50E-08	brown
CD55	0.02927	-0.07071	0.15355	0.02612	0.01099	0.17501	0.008	-0.30115	0.30115	0.70396	0.35809	0.04496	0.73455	0.88659	0.02205	0.91732	6.27E-05	6.27E-05	grey
PLEKHG2	-0.07431	-0.23029	-0.00612	0.09836	-0.26341	0.06766	0.10831	0.05338	-0.05338	0.33406	0.00244	0.93672	0.2006	0.0005	0.37921	0.1585	0.48804	0.48804	black
ZNF680	-0.02805	-0.10465	-0.00063	0.08538	-0.07527	0.05441	0.10088	0.02449	-0.02449	0.71574	0.17315	0.99347	0.26686	0.32782	0.47971	0.18922	0.7505	0.7505	turquoise
USP5	0.02185	-0.04381	-0.09435	-0.04066	0.07559	-0.19253	-0.02214	0.00519	-0.00519	0.7767	0.5694	0.21963	0.59749	0.32582	0.01164	0.77376	0.94629	0.94629	grey
RITA1	0.06469	-0.02169	-0.1198	-0.05521	0.11002	-0.20613	-0.04938	-0.03529	0.03529	0.40054	0.7783	0.1186	0.47328	0.15202	0.00683	0.52123	0.64682	0.64682	turquoise
IZUMO4	-0.03425	-0.00283	-0.17763	-0.19713	0.16975	-0.18723	-0.11363	-0.14955	0.14955	0.65654	0.97067	0.02011	0.00976	0.02645	0.0142	0.13891	0.05091	0.05091	grey
PRKCD	0.07308	-0.01836	0.05874	-0.04129	0.06381	0.17204	0.02531	-0.242	0.242	0.34219	0.81157	0.44539	0.59181	0.40699	0.02445	0.74244	0.00143	0.00143	grey
RTKN	0.02168	-0.02348	-0.09303	-0.07049	0.11123	-0.01946	0.11381	-0.42801	0.42801	0.77832	0.76048	0.22617	0.35961	0.14751	0.80055	0.1383	5.24E-09	5.24E-09	yellow
YES1	-0.02576	-0.20035	0.0572	0.04833	-0.01344	0.18748	0.11646	-0.10218	0.10218	0.73807	0.00861	0.45742	0.53014	0.86154	0.01407	0.12931	0.18356	0.18356	turquoise
BEX4	-0.05839	-0.14581	-0.22121	-0.07202	0.10861	-0.24091	-0.27255	0.52434	-0.52434	0.44812	0.05705	0.00364	0.34925	0.15737	0.0015	0.00031	1.83E-13	1.83E-13	brown
SLC41A2	-0.05115	-0.11901	0.07868	0.14399	-0.01394	0.13648	0.02065	-0.03994	0.03994	0.50647	0.12105	0.30638	0.06026	0.8564	0.07508	0.78865	0.60399	0.60399	turquoise
LMBRD2	-0.05279	-0.15407	-0.1216	-0.03965	-0.01527	-0.13176	-0.07812	0.30422	-0.30422	0.4929	0.04422	0.11312	0.60662	0.84285	0.08581	0.30979	5.23E-05	5.23E-05	turquoise
CCL20	-0.13072	0.01437	0.0289	-0.00248	-0.09433	0.17462	0.18026	-0.22756	0.22756	0.08834	0.85206	0.70748	0.97429	0.21972	0.02236	0.01831	0.00276	0.00276	grey
ZNF429	-0.08124	-0.16558	-0.17568	-0.08188	0.01125	-0.13681	-0.18559	0.41654	-0.41654	0.29084	0.03043	0.02154	0.28704	0.88384	0.07438	0.01509	1.46E-08	1.46E-08	turquoise
FAM110A	0.03128	-0.01165	-0.04591	-0.0388	0.02814	-0.02971	0.23657	-0.45999	0.45999	0.6846	0.87982	0.551	0.61434	0.71488	0.69973	0.00184	2.46E-10	2.46E-10	green
KLC3	0.12886	-0.04971	-0.12196	0.01738	0.0035	-0.15626	0.1121	-0.16928	0.16928	0.09302	0.51852	0.11204	0.82147	0.96379	0.04125	0.14437	0.02687	0.02687	grey
RPL22L1	0.05241	0.10116	-0.04064	-0.08382	-0.0428	0.0231	0.20235	-0.28652	0.28652	0.49604	0.18799	0.59769	0.27573	0.57832	0.76428	0.00795	0.00015	0.00015	purple
MTRES1	0.0086	0.04413	-0.08609	-0.05626	0.07633	-0.07558	0.05818	-0.00212	0.00212	0.91108	0.5666	0.2629	0.46487	0.32109	0.32586	0.44976	0.97808	0.97808	grey
PCYOX1	-0.06235	-0.19376	-0.09097	0.01294	-0.14919	-0.00307	-0.01967	0.29847	-0.29847	0.41785	0.01111	0.23667	0.86658	0.05147	0.96817	0.79844	7.33E-05	7.33E-05	turquoise
PDCD1LG2	-0.16986	0.04064	0.10825	0.08899	-0.26014	0.15545	0.14189	0.39754	-0.39754	0.02634	0.59763	0.15874	0.2471	0.00059	0.04234	0.06414	7.29E-08	7.29E-08	blue
HNRNPA0	0.00784	-0.16943	-0.0943	-0.05321	0.0666	-0.04197	-0.09364	0.07151	-0.07151	0.91894	0.02674	0.21991	0.48944	0.38679	0.5857	0.22315	0.35263	0.35263	turquoise
LAX1	-0.16998	#######	0.042	0.02818	-0.16719	0.11181	0.02197	0.45998	-0.45998	0.02624	0.99899	0.58545	0.7145	0.02884	0.14539	0.77547	2.46E-10	2.46E-10	blue
RADIL	-0.11598	-0.02876	-0.14871	-0.04062	0.09522	-0.1972	-0.20817	0.44143	-0.44143	0.13089	0.70882	0.05223	0.59786	0.21541	0.00973	0.00629	1.51E-09	1.51E-09	grey
WFDC2	0.03766	-0.05719	-0.00328	-0.02659	0.01397	0.038	0.06326	-0.43935	0.43935	0.6248	0.45749	0.96601	0.7299	0.85612	0.62167	0.4111	1.84E-09	1.84E-09	grey
RNF113A	0.11107	-0.13229	-0.09847	-0.03251	0.02574	-0.01541	0.04776	-0.27048	0.27048	0.14811	0.08456	0.2001	0.6729	0.73828	0.84148	0.53506	0.00035	0.00035	grey
CTTNBP2	-0.10956	-0.19343	-0.1544	0.03106	0.01524	-0.18225	-0.19937	0.51646	-0.51646	0.15372	0.01125	0.04376	0.68679	0.8432	0.01704	0.00894	4.81E-13	4.81E-13	brown
ZBP1	-0.00716	0.02135	0.10157	0.11106	-0.15864	0.11238	0.10516	0.01517	-0.01517	0.92599	0.78162	0.18619	0.14815	0.03823	0.14335	0.17105	0.84389	0.84389	tan
TMEM200	-0.10394	-0.15054	0.04903	0.14107	-0.16536	0.14846	0.11239	0.19482	-0.19482	0.17611	0.04937	0.52423	0.0657	0.03066	0.05264	0.1433	0.01067	0.01067	black
ZEB1	-0.09284	-0.14619	-0.02198	0.0695	-0.19147	0.09528	0.01426	0.44825	-0.44825	0.22714	0.0564	0.7754	0.36637	0.01212	0.21512	0.85315	7.85E-10	7.85E-10	blue
SOCS3	-0.13678	-0.09705	0.04617	0.08016	-0.13093	0.18478	0.07261	0.29893	-0.29893	0.07443	0.20665	0.54875	0.2973	0.08783	0.01554	0.34526	7.14E-05	7.14E-05	blue
JPH3	-0.01996	-0.13371	-0.3347	-0.14895	0.15709	-0.29278	-0.20695	0.33047	-0.33047	0.79553	0.08126	7.67E-06	0.05186	0.04018	0.0001	0.00661	1.01E-05	1.01E-05	brown
SMARCE1	-0.13816	-0.1434	-0.09929	-0.02192	0.04678	-0.14771	-0.14935	0.33571	-0.33571	0.07154	0.06132	0.19635	0.77593	0.54348	0.05386	0.05122	7.17E-06	7.17E-06	turquoise
C17orf100	0.03168	-0.11908	-0.24747	-0.18061	0.1867	-0.3012	-0.09572	0.07191	-0.07191	0.68083	0.12083	0.0011	0.01808	0.01449	6.25E-05	0.21299	0.34998	0.34998	brown
KLHL23	0.04889	-0.01339	0.02425	0.00208	0.06044	0.05876	-0.05012	-0.05656	0.05656	0.52544	0.86203	0.75289	0.97847	0.4323	0.44523	0.515	0.4625	0.4625	grey
R3HDM1	-0.03374	-0.04056	-0.05692	0.0489	-0.13317	-0.04784	0.04836	0.20597	-0.20597	0.66133	0.5984	0.45959	0.52531	0.08249	0.53435	0.52991	0.00688	0.00688	turquoise
EGFLAM	-0.13561	-0.01121	-0.08281	-0.03946	-0.03756	-0.15015	-0.08552	0.39452	-0.39452	0.07698	0.8843	0.28158	0.60835	0.62575	0.04998	0.26609	9.33E-08	9.33E-08	grey
CHPF	-0.01203	-0.00687	-0.01681	0.08886	0.06055	-0.03229	0.08402	-0.08051	0.08051	0.87594	0.92893	0.82728	0.24777	0.43144	0.67499	0.27461	0.2952	0.2952	grey
RWDD3	-0.11002	-0.07948	-0.0914	-0.00817	0.11937	-0.05021	-0.11171	0.09486	-0.09486	0.152	0.30146	0.23448	0.91549	0.11992	0.51426	0.14578	0.21717	0.21717	grey
ELL	-0.01261	-0.14494	-0.01757	0.03284	0.01647	0.02128	-0.00188	0.16576	-0.16576	0.86997	0.05857	0.8196	0.66985	0.83069	0.78236	0.9805	0.03025	0.03025	grey
ZBED1	7.93E-05	-0.0614	-0.03802	0.05413	-0.14289	0.11117	0.04965	0.25416	-0.25416	0.99918	0.42497	0.62147	0.48194	0.06227	0.14775	0.51899	0.0008	0.0008	black
C1orf162	-0.09311	0.01	0.01969	0.08059	-0.19758	0.08957	0.03709	0.40398	-0.40398	0.22577	0.89674	0.79827	0.29471	0.00959	0.24401	0.63004	4.27E-08	4.27E-08	blue
FIP1L1	-0.00911	-0.11453	-0.00245	-0.02336	-0.04614	0.10883	0.11774	0.02423	-0.02423	0.90583	0.1358	0.97459	0.76167	0.549	0.15652	0.1251	0.75311	0.75311	turquoise
MRPL36	-0.00697	-0.02947	-0.08336	-0.05814	0.03747	-0.15123	0.16696	-0.18534	0.18534	0.92793	0.702	0.27836	0.45006	0.62654	0.04833	0.02906	0.01523	0.01523	grey
ZNF175	-0.05194	-0.12391	-0.13063	-0.00266	0.0384	-0.11956	-0.10693	0.24289	-0.24289	0.49986	0.10638	0.08858	0.9724	0.61799	0.11933	0.16391	0.00137	0.00137	turquoise
AHRR	-0.03613	0.00593	0.08242	-0.00177	0.04065	-0.01345	0.08065	0.08197	-0.08197	0.63897	0.93865	0.28387	0.9817	0.59754	0.86144	0.29435	0.28652	0.28652	grey
C5orf15	-0.02715	-0.20681	-0.04549	0.00907	-0.03706	-0.05088	-0.01399	0.11288	-0.11288	0.72447	0.00665	0.55462	0.90632	0.63036	0.50871	0.85591	0.14158	0.14158	turquoise
RBM11	-0.06001	-0.06491	-0.17237	-0.07172	0.14783	-0.21657	-0.25804	0.40782	-0.40782	0.43559	0.39898	0.02417	0.35125	0.05366	0.00444	0.00066	3.09E-08	3.09E-08	brown
CDKN2AIP	-0.08669	-0.14279	-0.00519	-0.02099	0.12456	0.10067	-0.13185	0.03997	-0.03997	0.25956	0.06246	0.94626	0.78527	0.10454	0.19016	0.08561	0.60373	0.60373	turquoise
GRIK3	0.00699	-0.09727	-0.13426	-0.00515	-0.19235	-0.05625	-0.07836	0.43398	-0.43398	0.9277	0.20564	0.07998	0.94667	0.01172	0.46492	0.3083	3.03E-09	3.03E-09	blue
PIGQ	0.0526	0.00996	-0.14032	-0.05446	0.17827	-0.11692	0.01577	-0.20928	0.20928	0.49448	0.89715	0.06718	0.47931	0.01966	0.12776	0.83775	0.00601	0.00601	green
ZNF793	-0.06641	-0.10448	-0.16324	-0.00266	0.03248	-0.08721	-0.06659	0.40455	-0.40455	0.38815	0.17386	0.0329	0.97248	0.67322	0.2567	0.38682	4.07E-08	4.07E-08	brown
S100A10	0.0502	0.0087	0.11308	0.02621	-0.10357	0.26458	0.22867	-0.48126	0.48126	0.51439	0.91012	0.14085	0.73367	0.17766	0.00047	0.00263	2.68E-11	2.68E-11	yellow
CYP2R1	0.00924	0.01371	0.03759	0.02422	0.05618	0.11808	0.0906	-0.22335	0.22335	0.90451	0.8587	0.62545	0.75323	0.46553	0.12399	0.23862	0.00332	0.00332	grey
FAM220A	-0.04684	-0.16247	0.03117	0.07059	-0.12999	0.0872	0.05496	0.02334	-0.02334	0.54298	0.03374	0.68572	0.35889	0.09016	0.25676	0.47528	0.76188	0.76188	turquoise
ZSCAN26	-0.02181	-0.11419	-0.11362	-0.02193	0.05998	-0.08238	-0.12454	0.39458	-0.39458	0.77711	0.13697	0.13897	0.77588	0.43579	0.28408	0.1046	9.28E-08	9.28E-08	turquoise
WWC2	-0.10156	-0.18192	-0.02781	0.09264	-0.14905	0.1132	0.05588	0.202	-0.202	0.18625	0.01725	0.71802	0.22814	0.0517	0.14043	0.46787	0.00806	0.00806	turquoise
IL10	-0.02284	0.03876	0.05434	0.03385	-0.16877	0.06619	0.17332	0.4784	-0.4784	0.76683	0.61475	0.48024	0.66025	0.02734	0.38972	0.02339	3.65E-11	3.65E-11	blue
ACRBP	-0.00606	0.0269	-0.02914	0.03828	0.09374	-0.02554	-0.06546	0.27696	-0.27696	0.93728	0.72689	0.70496	0.61911	0.22264	0.74018	0.39498	0.00025	0.00025	grey
UBE2B	0.04229	-0.17924	-0.03563	-0.04636	-0.01353	-0.02055	-0.07712	-0.02637	0.02637	0.58288	0.01899	0.6436	0.54712	0.86062	0.78967	0.31604	0.73207	0.73207	turquoise
SPAST	0.00089	-0.13684	-0.04885	0.00205	-0.06959	-0.00738	0.05178	0.22303	-0.22303	0.99082	0.0743	0.52578	0.97874	0.36577	0.92368	0.50121	0.00337	0.00337	turquoise
ARMH3	-0.01661	-0.07198	-0.03507	-0.04549	-0.05589	-0.05705	0.06363	0.11506	-0.11506	0.82924	0.34949	0.64879	0.55463	0.46783	0.45859	0.40837	0.13398	0.13398	turquoise
MFAP1	-0.07554	-0.20191	-0.09756	-0.03045	-0.10927	-0.00047	0.07857	0.10173	-0.10173	0.32614	0.00809	0.20428	0.69256	0.15485	0.99517	0.30703	0.1855	0.1855	turquoise
ZNF687	0.00861	-0.07977	-0.11336	-0.14035	-0.02316	-0.0065	-0.01569	-0.01714	0.01714	0.91105	0.29968	0.13986	0.06711	0.76363	0.93274	0.83857	0.82394	0.82394	turquoise
GNAI3	-0.11195	-0.10491	0.07206	0.09665	-0.17774	0.1851	0.13645	0.03127	-0.03127	0.1449	0.17207	0.34897	0.20856	0.02003	0.01536	0.07514	0.68478	0.68478	turquoise
PHLPP1	-0.007	-0.14495	-0.03118	0.11741	0.0755	0.06598	0.0048	0.0899	-0.0899	0.92764	0.05854	0.68557	0.12616	0.3264	0.39122	0.95029	0.24225	0.24225	grey
JADE2	0.01231	-0.16394	-0.15117	-0.07105	-0.07129	-0.1329	-0.13162	0.1691	-0.1691	0.87302	0.03214	0.04842	0.35579	0.35411	0.08312	0.08617	0.02703	0.02703	turquoise
KIAA1586	-0.13613	-0.10483	-0.14424	-0.03586	-0.15083	-0.09822	-0.06765	0.52508	-0.52508	0.07584	0.1724	0.05982	0.64148	0.04894	0.20121	0.37933	1.67E-13	1.67E-13	blue
INPP1	0.08036	0.0037	0.01475	0.00691	0.0853	0.13834	0.02665	-0.28711	0.28711	0.29613	0.96166	0.84811	0.9285	0.26733	0.07116	0.72932	0.00014	0.00014	grey
RAB8B	-0.09037	-0.16521	0.04151	0.05606	-0.14779	0.15155	-0.01996	0.30218	-0.30218	0.23982	0.03082	0.58987	0.46645	0.05373	0.04786	0.79551	5.90E-05	5.90E-05	blue
PLXNB2	0.01938	0.01888	-0.10126	0.0199	0.03834	-0.07694	0.13725	-0.17685	0.17685	0.80134	0.80642	0.18757	0.79613	0.61856	0.31722	0.07343	0.02067	0.02067	grey
TBX3	-0.0402	-0.14995	0.04765	0.13787	-0.08435	0.04715	0.17391	0.26342	-0.26342	0.60167	0.05028	0.53603	0.07213	0.27272	0.54028	0.02291	0.0005	0.0005	blue
STX10	0.09841	0.00153	-0.06291	-0.03537	0.13431	-0.06144	0.09698	-0.3232	0.3232	0.20034	0.98417	0.41365	0.64605	0.07989	0.42468	0.20699	1.62E-05	1.62E-05	green
TMED5	-0.06261	-0.15778	0.08436	0.10531	-0.1097	0.01244	-0.04464	0.21586	-0.21586	0.41588	0.0393	0.27264	0.17044	0.15319	0.87173	0.56205	0.00458	0.00458	turquoise
LRRC66	0.02282	0.03211	0.07922	0.04333	0.17263	0.14884	0.04148	-0.2278	0.2278	0.767	0.67678	0.30303	0.5736	0.02396	0.05203	0.59013	0.00273	0.00273	grey
TFPI	0.01831	-0.14833	0.08561	0.08702	-0.17887	0.25747	0.11374	-0.15139	0.15139	0.81215	0.05285	0.26557	0.25773	0.01924	0.00067	0.13853	0.0481	0.0481	turquoise
UNKL	-0.05458	-0.10791	-0.0329	-0.01444	0.04951	-0.02656	0.0538	0.12746	-0.12746	0.47833	0.16005	0.66928	0.85134	0.52019	0.73019	0.48459	0.09664	0.09664	red
SPATA13	-0.00533	-0.09546	-0.04445	0.02995	-0.02564	0.05671	-0.17974	0.23884	-0.23884	0.94487	0.21426	0.56373	0.69739	0.73923	0.46125	0.01866	0.00166	0.00166	turquoise
XPO1	-0.0268	-0.13573	0.0098	0.03295	-0.08951	0.07957	0.1141	-0.02977	0.02977	0.7279	0.07671	0.89881	0.66875	0.24433	0.30089	0.13728	0.69913	0.69913	turquoise
SPIB	-0.02351	-0.0082	0.02901	0.01507	0.0311	0.19709	-0.00331	0.11611	-0.11611	0.76024	0.91521	0.70639	0.84489	0.68635	0.00977	0.96577	0.13044	0.13044	blue
KIF5C	-0.03309	-0.14706	-0.26142	-0.14989	0.01575	-0.31215	-0.1923	0.53111	-0.53111	0.66745	0.05493	0.00055	0.05038	0.83799	3.23E-05	0.01174	7.85E-14	7.85E-14	brown
CNKSR3	-0.00327	-0.24824	-0.07626	0.05161	-0.06376	-0.05173	-0.20435	0.30484	-0.30484	0.96618	0.00106	0.3215	0.50264	0.40735	0.50166	0.00734	5.04E-05	5.04E-05	turquoise
FAM210A	-0.10539	-0.23002	0.02207	0.11121	-0.06004	0.07441	0.12163	-0.0392	0.0392	0.17009	0.00247	0.77449	0.1476	0.43534	0.33343	0.11302	0.6107	0.6107	turquoise
COL4A3	-0.00407	-0.04793	-0.11394	-0.01787	0.00515	0.03446	-0.07039	0.24963	-0.24963	0.9579	0.53364	0.13783	0.81653	0.94668	0.65454	0.36028	0.00099	0.00099	blue
ZNF480	-0.0118	0.00565	0.02175	0.06843	-0.06156	0.10899	0.02368	-0.05514	0.05514	0.87831	0.94154	0.77766	0.37387	0.42376	0.15592	0.75854	0.4738	0.4738	turquoise
PRDX4	-0.04464	0.01399	-0.07289	0.02139	0.01263	-0.21489	0.02917	0.08834	-0.08834	0.56208	0.85588	0.34341	0.78128	0.86973	0.00476	0.70484	0.25054	0.25054	grey
ZNF582	-0.0948	-0.18404	-0.15514	-0.02445	0.04178	-0.18034	-0.26932	0.55892	-0.55892	0.21743	0.01597	0.04275	0.75088	0.58739	0.01826	0.00037	1.95E-15	1.95E-15	brown
TOX4	0.02527	-0.12686	0.02061	0.04672	-0.12947	0.09114	0.04418	-0.02057	0.02057	0.74286	0.09824	0.78904	0.54398	0.09145	0.23579	0.56616	0.78945	0.78945	turquoise
ZRANB2	-0.04912	-0.13904	-0.02358	0.00469	0.02807	0.0095	0.02976	0.06849	-0.06849	0.52351	0.06973	0.75952	0.95141	0.71555	0.90185	0.69918	0.3734	0.3734	red
CNN1	-0.10032	-0.0116	0.0142	0.05628	-0.05857	0.10327	0.01311	0.25442	-0.25442	0.19169	0.88031	0.85373	0.4647	0.44673	0.17891	0.8649	0.00078	0.00078	salmon
ZBTB45	0.04555	-0.03304	-0.17853	-0.05633	0.05983	-0.03686	0.04985	-0.17372	0.17372	0.55415	0.66793	0.01947	0.46427	0.43696	0.63217	0.51729	0.02307	0.02307	green
SLC9A3R2	0.07715	-0.03836	-0.03961	-0.05774	0.15556	-0.01617	0.10595	-0.29415	0.29415	0.31589	0.61842	0.60702	0.45316	0.04219	0.83369	0.16782	9.42E-05	9.42E-05	green
OBSCN	-0.05602	-0.05905	-0.00359	0.15524	-0.09488	-0.03822	0.15727	-0.0882	0.0882	0.46677	0.44296	0.96284	0.04261	0.21706	0.61969	0.03994	0.25132	0.25132	grey
RSPO3	-0.10397	-0.00292	-0.0057	0.07893	-0.13984	0.08051	0.01231	0.49243	-0.49243	0.17596	0.96979	0.94099	0.30483	0.06812	0.2952	0.87307	7.87E-12	7.87E-12	salmon
DNAJC17	-0.0224	-0.06231	-0.06216	-0.04379	-0.00391	-0.02333	0.10261	-0.27858	0.27858	0.77124	0.41818	0.41926	0.56953	0.9595	0.76199	0.18172	0.00022	0.00022	grey
CFP	-0.12586	-0.06064	-0.0308	-0.00594	-0.0647	0.0189	-0.05432	0.41705	-0.41705	0.10093	0.43073	0.6892	0.93854	0.40046	0.80615	0.48042	1.39E-08	1.39E-08	blue
SMC2	-0.08065	-0.03609	-0.024	-0.04499	-0.14667	0.02078	0.08521	0.08469	-0.08469	0.29433	0.6393	0.75534	0.55906	0.05558	0.78731	0.26781	0.27075	0.27075	turquoise
PEX2	-0.00492	0.03498	0.00492	-0.00342	-0.01357	0.04598	-0.01294	-0.00503	0.00503	0.94904	0.64967	0.94903	0.96464	0.86018	0.55042	0.86656	0.94796	0.94796	turquoise
NOL9	-0.08492	-0.11829	-0.06572	-0.00122	-0.05629	-0.03773	-0.01056	0.31561	-0.31561	0.26948	0.12333	0.3931	0.98736	0.46464	0.62421	0.89097	2.61E-05	2.61E-05	turquoise
MELK	-0.04538	0.08852	0.02674	-0.0294	-0.08786	0.08358	0.30111	-0.39082	0.39082	0.55564	0.24962	0.72843	0.70264	0.25316	0.27713	6.28E-05	1.26E-07	1.26E-07	pink
KLHDC2	-0.00514	-0.0937	-0.04943	-0.03229	0.14576	-0.03745	-0.11258	0.11751	-0.11751	0.94677	0.22285	0.52082	0.67504	0.05715	0.62675	0.14263	0.12584	0.12584	turquoise
DCLRE1A	-0.00875	-0.08568	-0.0771	-0.03084	0.04506	-0.09479	0.01098	0.08143	-0.08143	0.9096	0.26517	0.31618	0.68883	0.55837	0.21751	0.88661	0.28968	0.28968	turquoise
FBXW2	-0.06027	-0.12188	-0.05153	-0.00948	-0.15159	0.04124	0.0146	0.28942	-0.28942	0.43363	0.11227	0.50324	0.90205	0.0478	0.5923	0.8497	0.00012	0.00012	turquoise
GCLM	-0.04034	-0.20934	-0.06752	0.02821	-0.03727	-0.13341	-0.06493	0.24263	-0.24263	0.60034	0.006	0.3802	0.71412	0.62843	0.08193	0.39979	0.00139	0.00139	turquoise
INPP5D	-0.12502	-0.02746	-0.02013	-0.02115	-0.116	0.02263	0.02809	0.29743	-0.29743	0.10327	0.72145	0.79386	0.78361	0.1308	0.76887	0.71538	7.79E-05	7.79E-05	blue
PRPH	0.03579	-0.06704	-0.17509	-0.03447	0.03356	-0.19505	-0.13578	0.37327	-0.37327	0.64212	0.38362	0.02199	0.6545	0.66306	0.01057	0.07661	4.95E-07	4.95E-07	brown
EIPR1	0.01206	0.03147	-0.04305	-0.07686	-0.02371	-0.06685	0.05645	-0.24543	0.24543	0.87557	0.68284	0.57615	0.31768	0.7582	0.38497	0.46333	0.00121	0.00121	turquoise
TTC39A	0.07047	-0.00272	-0.04322	-0.02896	0.21963	-0.01679	-0.01896	-0.11457	0.11457	0.35973	0.97185	0.57457	0.70694	0.0039	0.82743	0.80558	0.13566	0.13566	grey

CDHR2	0.03793	0.04535	-0.03658	-0.01921	0.19081	0.11537	0.0632	-0.22705	0.22705	0.62233	0.55584	0.63483	0.80309	0.01242	0.13294	0.4115	0.00282	0.00282	yellow
KIAA1549	-0.05431	-0.10881	-0.10671	-0.04985	0.07216	-0.16915	-0.07016	0.15833	-0.15833	0.48051	0.15659	0.16477	0.51731	0.34832	0.02699	0.36186	0.03861	0.03861	turquoise
SMG5	0.06676	-0.09783	-0.05321	-0.0481	-0.0036	-0.017	0.16793	-0.25642	0.25642	0.38565	0.20301	0.48945	0.53215	0.96276	0.82532	0.02813	0.00071	0.00071	greenyellow
C3orf18	-0.05728	-0.09903	-0.22882	-0.121	-0.07295	-0.2074	-0.14016	0.51385	-0.51385	0.4568	0.19753	0.00261	0.11492	0.34302	0.00649	0.06748	6.59E-13	6.59E-13	brown
SYNE1	-0.07169	-0.15262	-0.12604	0.02177	-0.12709	-0.05887	-0.16895	0.68298	-0.68298	0.35145	0.04628	0.10045	0.77744	0.09764	0.44438	0.02718	7.86E-25	7.86E-25	turquoise
PTGS1	-0.11458	-0.06797	0.03139	0.08797	-0.12441	0.0698	-0.01574	0.39716	-0.39716	0.13563	0.37703	0.68361	0.25258	0.10495	0.36434	0.83812	7.52E-08	7.52E-08	blue
TMEM18	0.00239	0.08278	-0.0386	-0.05698	-0.08779	0.00986	0.04733	-0.03346	0.03346	0.97524	0.28176	0.61621	0.45915	0.25354	0.8981	0.5387	0.664	0.664	turquoise
TNRC6A	-0.04915	-0.201	-0.1165	-0.00686	-0.01255	-0.06272	-0.05487	0.24255	-0.24255	0.52319	0.00839	0.12915	0.92905	0.87054	0.4151	0.47597	0.00139	0.00139	turquoise
RPGR	-0.08254	-0.20441	-0.09554	-0.02185	0.01916	-0.1013	-0.16553	0.35386	-0.35386	0.28313	0.00732	0.21386	0.77664	0.80361	0.18737	0.03049	2.05E-06	2.05E-06	turquoise
NUDT19	0.01228	-0.06993	-0.03531	0.02588	-0.09089	-0.09546	0.02737	-0.09008	0.09008	0.87337	0.3634	0.64659	0.73686	0.23711	0.21425	0.72236	0.24132	0.24132	grey
TRIM21	-0.01507	-0.01637	0.0528	0.03826	-0.15551	0.21643	0.17583	-0.19762	0.19762	0.84488	0.83172	0.49278	0.61929	0.04225	0.00447	0.02143	0.00957	0.00957	tan
SFXN3	0.02243	-0.06864	0.06164	0.08443	-0.08607	0.13351	0.18687	-0.25552	0.25552	0.77087	0.37237	0.42318	0.27222	0.26298	0.08171	0.01439	0.00074	0.00074	turquoise
HTRA2	-0.05891	-0.0794	-0.02278	0.04967	-0.12542	0.02372	0.07454	-0.00798	0.00798	0.44403	0.3019	0.76742	0.51884	0.10214	0.75816	0.33259	0.91748	0.91748	turquoise
PDGFD	-0.04815	-0.13444	-0.09106	0.07489	-0.12538	0.08265	-0.10521	0.38425	-0.38425	0.5317	0.07959	0.23621	0.33031	0.10225	0.2825	0.17082	2.12E-07	2.12E-07	blue
HAS3	0.01177	-0.09667	0.08334	-0.0131	0.04373	0.1007	0.16969	-0.29081	0.29081	0.87853	0.20846	0.27848	0.86493	0.57007	0.19003	0.0265	0.00011	0.00011	grey
ZNF398	0.01037	-0.18028	-0.07124	-0.04827	-0.00974	-0.03849	0.01339	0.13541	-0.13541	0.89287	0.0183	0.35447	0.53073	0.89935	0.61719	0.86196	0.07742	0.07742	turquoise
GSK3B	-0.01104	-0.08368	-0.11443	-0.0699	-0.08385	0.00685	0.06601	0.05518	-0.05518	0.88609	0.27652	0.13615	0.36361	0.27554	0.92913	0.39101	0.47349	0.47349	turquoise
PDS5B	0.0218	-0.13538	-0.07951	-0.0236	-0.13558	-0.06328	-0.02114	0.30227	-0.30227	0.77716	0.07749	0.30126	0.7593	0.07704	0.41095	0.78372	5.87E-05	5.87E-05	turquoise
REXO2	-0.02509	-0.03896	0.06497	0.06639	-0.21216	0.12585	0.23338	-0.1238	0.1238	0.74459	0.61289	0.39852	0.3883	0.00534	0.10099	0.00213	0.10668	0.10668	turquoise
FOXD3	0.00899	-0.11134	-0.16635	0.03903	0.02897	-0.16523	-0.09721	0.28587	-0.28587	0.90709	0.14711	0.02967	0.61229	0.70679	0.0308	0.2059	0.00015	0.00015	grey
CLSTN3	-0.09071	-0.22538	-0.15518	-0.06972	0.0874	-0.25332	-0.26544	0.37078	-0.37078	0.23802	0.00304	0.0427	0.36485	0.25568	0.00083	0.00045	5.97E-07	5.97E-07	brown
CCDC191	-0.05688	-0.15381	-0.16632	-0.1249	0.1047	-0.05096	-0.03237	-0.06838	0.06838	0.45993	0.04458	0.0297	0.10359	0.17292	0.50799	0.6743	0.3742	0.3742	red
ERGIC1	0.07259	-0.0754	0.06837	0.0279	-0.12277	0.22581	0.11895	-0.15417	0.15417	0.34544	0.32698	0.37422	0.71718	0.10965	0.00298	0.12124	0.04408	0.04408	turquoise
GCN1	0.05188	-0.08085	0.02543	-0.02544	0.01881	-0.01574	0.06133	0.018	-0.018	0.50036	0.29314	0.74132	0.74122	0.80709	0.8381	0.42554	0.8152	0.8152	turquoise
TCERG1	-0.03293	-0.11615	-0.03521	-0.03308	0.06225	-0.00346	0.00157	-0.14124	0.14124	0.669	0.13033	0.64749	0.66754	0.41862	0.96414	0.98379	0.06538	0.06538	red
C19orf33	0.08981	0.08074	0.1424	0.06989	-0.05956	0.26098	0.31623	-0.518	0.518	0.24276	0.29383	0.06318	0.36372	0.43905	0.00057	2.51E-05	3.99E-13	3.99E-13	yellow
GMFG	-0.13239	-0.01508	0.03512	0.04743	-0.18489	0.04874	0.00139	0.36388	-0.36388	0.08433	0.84484	0.64836	0.53791	0.01548	0.52672	0.98563	9.97E-07	9.97E-07	blue
DIPK2A	0.00328	-0.14218	-0.03571	0.09261	-0.12424	0.19034	0.00381	0.21132	-0.21132	0.96602	0.0636	0.64285	0.22829	0.10545	0.01265	0.9606	0.00553	0.00553	turquoise
RALY	0.07508	-0.00054	0.00829	0.03275	0.06202	0.08085	0.18201	-0.43513	0.43513	0.32909	0.99438	0.91428	0.67065	0.42037	0.29313	0.01719	2.73E-09	2.73E-09	green
LUM	-0.08456	-0.05396	0.06839	0.06864	-0.19502	0.2229	0.04864	0.28582	-0.28582	0.27152	0.48332	0.37411	0.37239	0.01059	0.00339	0.52751	0.00015	0.00015	black
SLC7A2	-0.09671	-0.15777	-0.14055	-0.06023	-0.03819	-0.1719	-0.17359	0.53111	-0.53111	0.20827	0.03931	0.06672	0.43393	0.61995	0.02457	0.02317	7.84E-14	7.84E-14	brown
HOXC9	0.09772	-0.00907	0.06587	0.02718	-0.03565	0.11273	0.17019	-0.29689	0.29689	0.20356	0.90626	0.39201	0.72415	0.6434	0.14209	0.02605	8.04E-05	8.04E-05	grey
WDR61	-0.0171	-0.07385	-0.18707	-0.10184	0.10186	-0.14735	-0.07437	0.09159	-0.09159	0.82431	0.3371	0.01429	0.18503	0.18493	0.05445	0.33369	0.23348	0.23348	grey
PRPF38B	-0.07288	-0.16725	0.00475	0.02329	-0.02391	0.13113	-0.02037	0.16284	-0.16284	0.34347	0.02878	0.95089	0.76233	0.75624	0.08735	0.79139	0.03334	0.03334	turquoise
MNX1	0.1381	0.02327	-0.14999	-0.04936	0.23702	-0.06493	-0.04138	-0.13159	0.13159	0.07166	0.76255	0.05023	0.52148	0.0018	0.39882	0.59104	0.08624	0.08624	grey
WDR33	-0.00174	-0.08806	-0.05943	0.01765	-0.06718	0.05577	0.05234	0.03623	-0.03623	0.98199	0.25206	0.44006	0.81877	0.38264	0.46874	0.49656	0.63808	0.63808	turquoise
RIPK4	0.03819	-0.05857	0.01398	0.05709	0.06248	0.03303	0.07578	-0.32826	0.32826	0.61998	0.44672	0.85598	0.45826	0.41692	0.66803	0.32456	1.17E-05	1.17E-05	grey
DNM2	0.06639	-0.0306	0.04625	0.04295	0.10128	0.14859	0.12587	-0.34209	0.34209	0.38828	0.69116	0.54802	0.57702	0.18747	0.05243	0.10092	4.66E-06	4.66E-06	grey
FAM241B	-0.03813	-0.13453	-0.17251	-0.08148	0.09568	-0.23111	-0.0525	-0.27423	0.27423	0.62052	0.07939	0.02405	0.28938	0.21318	0.00236	0.49525	0.00028	0.00028	grey
HACD2	-0.01691	-0.11647	-0.06915	0.01892	-0.04599	0.01215	0.13589	-0.07102	0.07102	0.82621	0.12925	0.36879	0.80599	0.55031	0.8747	0.07635	0.35596	0.35596	turquoise
CHRD	-0.07648	-0.05494	-0.02522	0.11458	-0.13255	0.12821	0.02585	0.42409	-0.42409	0.32012	0.47537	0.74331	0.13563	0.08395	0.09469	0.73715	7.46E-09	7.46E-09	blue
CFAP70	-0.03533	-0.20954	-0.2164	-0.0969	0.20981	-0.27991	-0.1998	0.16431	-0.16431	0.64643	0.00595	0.00447	0.20736	0.00588	0.00021	0.00879	0.03175	0.03175	brown
GRASP	-0.05931	-0.14825	-0.08334	0.04017	0.00596	-0.08467	-0.12258	0.46706	-0.46706	0.44099	0.05297	0.27852	0.60192	0.93836	0.27085	0.11023	1.20E-10	1.20E-10	blue
NETO2	0.00623	-0.15389	0.0464	0.09605	-0.18091	0.01095	0.0617	0.04581	-0.04581	0.9355	0.04447	0.54679	0.21143	0.01789	0.88701	0.42272	0.55186	0.55186	grey
APH1B	-0.08442	-0.17976	-0.19023	-0.07114	-0.00763	-0.20072	-0.21454	0.37577	-0.37577	0.27228	0.01864	0.0127	0.35516	0.92109	0.00848	0.00484	4.09E-07	4.09E-07	brown
DCTN2	0.08573	-0.18706	-0.12094	-0.01684	0.08081	-0.1734	-0.05318	-0.02192	0.02192	0.26491	0.01429	0.11509	0.82699	0.29342	0.02332	0.48965	0.77595	0.77595	turquoise
AMPD3	0.00404	-0.14336	-0.06586	-0.05753	-0.09091	-0.10939	-0.01138	0.13423	-0.13423	0.95821	0.0614	0.3921	0.4548	0.237	0.15437	0.88259	0.08005	0.08005	turquoise
U2AF2	0.0325	-0.1096	-0.04134	-0.05856	0.00282	0.00855	-0.00024	-0.13972	0.13972	0.67307	0.15358	0.59133	0.44677	0.97083	0.91165	0.99748	0.06835	0.06835	turquoise
MAPK8	-0.01267	-0.24539	-0.03325	-0.02249	-0.10563	-0.08053	-0.04178	0.14076	-0.14076	0.86934	0.00122	0.66596	0.77027	0.16913	0.29506	0.58743	0.0663	0.0663	turquoise
HNRNPH2	-0.09004	-0.15443	-0.10022	-0.0213	-0.06043	-0.00326	-0.01878	0.17286	-0.17286	0.24155	0.04372	0.19217	0.78211	0.43234	0.96625	0.80743	0.02377	0.02377	turquoise
DHRS7	0.03438	-0.11312	-0.01061	0.01577	0.07364	0.00244	-0.02288	-0.20684	0.20684	0.65526	0.14072	0.89046	0.83776	0.33847	0.97476	0.76641	0.00664	0.00664	grey
TRIM15	0.03319	0.07518	0.07574	0.01969	0.15126	0.21364	0.13186	-0.46285	0.46285	0.66647	0.32842	0.32482	0.79826	0.04829	0.00502	0.08558	1.84E-10	1.84E-10	yellow
APLF	-0.1375	0.02733	0.06852	0.11758	-0.12612	0.1665	0.09231	0.1217	-0.1217	0.07292	0.72271	0.3732	0.12563	0.10024	0.02951	0.22982	0.11283	0.11283	turquoise
GSTM1	-0.08237	0.06839	0.05786	0.01283	-0.03533	0.03452	-0.02253	0.02769	-0.02769	0.28416	0.37408	0.45221	0.86771	0.64638	0.65396	0.76992	0.71918	0.71918	grey
ADAMTS1	-0.0996	-0.12231	-0.02571	0.11517	-0.06417	0.05802	-0.05157	0.49959	-0.49959	0.19496	0.111	0.73854	0.13362	0.40436	0.45095	0.50294	3.50E-12	3.50E-12	blue
LINGO3	-0.06453	-0.04434	-0.0461	0.00389	-0.04515	-0.05068	-0.10955	0.33184	-0.33184	0.40173	0.56475	0.54937	0.95971	0.55759	0.51032	0.15378	9.26E-06	9.26E-06	blue
SNTB1	-0.07452	0.0848	0.05568	0.07038	-0.25063	0.1371	0.19114	-0.08387	0.08387	0.33273	0.27013	0.46948	0.36035	0.00095	0.07376	0.01227	0.27545	0.27545	grey
SFR1	0.0368	0.03952	0.11552	-0.03408	-0.01119	0.11176	0.19101	-0.3356	0.3356	0.63275	0.60779	0.13244	0.6581	0.88452	0.14558	0.01233	7.22E-06	7.22E-06	turquoise
HIC2	-0.10415	-0.22183	-0.11251	0.01695	0.10584	-0.15267	-0.11798	0.23116	-0.23116	0.1752	0.00355	0.14287	0.82587	0.16827	0.04621	0.12432	0.00235	0.00235	grey
KCNK16	-0.02992	-0.06236	-0.207	-0.14542	0.05708	-0.21314	-0.14678	0.33957	-0.33957	0.69766	0.41778	0.0066	0.05772	0.45832	0.00513	0.05541	5.53E-06	5.53E-06	brown
CGNL1	-0.00028	-0.07747	0.01413	0.04676	-0.18842	0.18741	0.02402	0.4564	-0.4564	0.99713	0.31389	0.85448	0.5436	0.01359	0.01411	0.75518	3.52E-10	3.52E-10	blue
SVBP	-0.02059	-0.02786	-0.04917	-0.04854	-0.06246	-0.0292	-0.08524	0.13439	-0.13439	0.78923	0.71758	0.52303	0.52844	0.41706	0.7046	0.26763	0.07969	0.07969	turquoise
DNAJC2	0.09692	-0.10066	0.01957	-0.0543	0.04146	0.10462	0.10439	-0.25899	0.25899	0.20727	0.1902	0.79943	0.48057	0.59031	0.17328	0.17422	0.00063	0.00063	turquoise
OGFOD1	-0.03778	-0.08381	-0.0233	-0.00533	-0.19669	0.13909	0.15737	1.80E-05	#######	0.62371	0.27579	0.76228	0.94482	0.00992	0.06963	0.03982	0.99981	0.99981	turquoise
CDHR5	0.0757	-0.03726	0.04178	0.0202	0.16492	0.1528	0.02894	-0.34398	0.34398	0.32506	0.62855	0.58741	0.79313	0.03112	0.04602	0.7071	4.09E-06	4.09E-06	yellow
WDR73	-0.05553	-0.17795	-0.15733	-0.0764	0.11438	-0.08787	-0.0189	0.07098	-0.07098	0.47071	0.01988	0.03987	0.3206	0.13632	0.2531	0.80617	0.35623	0.35623	turquoise
PPM1A	-0.01552	-0.14609	-0.04464	-0.00732	-0.00689	-0.03093	-0.07574	0.21296	-0.21296	0.84033	0.05657	0.5621	0.92427	0.92871	0.68796	0.32482	0.00517	0.00517	turquoise
CALCRL	-0.18779	-0.07981	-0.05941	0.09584	-0.13291	0.04477	-0.08894	0.57533	-0.57533	0.01391	0.29943	0.4402	0.2124	0.08311	0.56094	0.24735	1.86E-16	1.86E-16	blue
NFIC	-0.05785	-0.13644	-0.15211	0.03867	-0.14214	-0.0554	-0.06634	0.3725	-0.3725	0.45234	0.07516	0.04703	0.61554	0.06366	0.47171	0.38864	5.24E-07	5.24E-07	turquoise
TRAK1	-0.06303	-0.09007	0.02726	-0.07944	0.05488	0.14873	0.05863	-0.25472	0.25472	0.41275	0.24137	0.72335	0.30171	0.47589	0.0522	0.44622	0.00077	0.00077	turquoise
PRSS23	-0.05138	-0.06723	0.00095	0.05484	-0.20621	0.11778	0.05712	0.2765	-0.2765	0.5045	0.38229	0.99018	0.47623	0.00681	0.12497	0.45808	0.00025	0.00025	black
KIF26A	-0.20587	-0.02607	-0.11711	0.12523	0.02245	-0.12526	-0.03732	0.53619	-0.53619	0.0069	0.73506	0.12716	0.10267	0.77073	0.1026	0.62796	4.09E-14	4.09E-14	grey
TIGD5	-0.05799	-0.07455	-0.21665	-0.07622	0.0152	-0.24679	-0.06243	-0.09283	0.09283	0.45122	0.33254	0.00442	0.32178	0.84355	0.00114	0.41725	0.2272	0.2272	grey
TATDN2	-0.0085	-0.09556	-0.14278	-0.14535	-0.09133	-0.10955	0.03891	0.09711	-0.09711	0.91217	0.21374	0.06247	0.05785	0.23484	0.15377	0.61336	0.2064	0.2064	grey
ZFP92	0.02051	-0.14997	-0.15162	0.0177	-0.13414	-0.1338	-0.10255	0.50335	-0.50335	0.79008	0.05025	0.04775	0.81825	0.08027	0.08104	0.18199	2.27E-12	2.27E-12	blue
ZNF786	-0.0629	-0.15986	-0.09798	-0.02135	0.12452	-0.15125	-0.08875	0.15682	-0.15682	0.41377	0.03675	0.20231	0.7817	0.10467	0.0483	0.24839	0.04052	0.04052	turquoise
CBX8	0.05694	-0.14328	-0.12108	-0.00838	0.29213	-0.22627	-0.04886	-0.13769	0.13769	0.45946	0.06155	0.11468	0.91333	0.00011	0.00292	0.52565	0.07252	0.07252	grey
SPINT2	0.16339	-0.12208	-0.03228	-0.02171	0.09716	-0.09446	0.02186	-0.36368	0.36368	0.03274	0.1117	0.6751	0.77806	0.20614	0.2191	0.77652	1.01E-06	1.01E-06	grey
TRAPPC9	0.06103	-0.09617	-0.11249	-0.09657	0.0265	-0.19245	-0.09752	-0.0159	0.0159	0.4278	0.21085	0.14294	0.20895	0.73083	0.01168	0.20445	0.83645	0.83645	grey
C1orf127	-0.04579	0.02479	-0.2737	-0.12266	0.05966	-0.17799	-0.09513	0.38051	-0.38051	0.55204	0.74752	0.00029	0.10997	0.43824	0.01985	0.21583	2.84E-07	2.84E-07	brown
PRIMPOL	-0.14999	-0.08178	-0.1387	-0.0619	0.03815	-0.05692	-0.06965	0.18553	-0.18553	0.05022	0.2876	0.07042	0.42125	0.62032	0.45966	0.36534	0.01512	0.01512	turquoise
CAPN10	0.07354	-0.04117	-0.0815	-0.0463	0.24301	-0.06883	0.00398	-0.2478	0.2478	0.33912	0.59285	0.2893	0.54761	0.00136	0.37106	0.95882	0.00108	0.00108	red
ABCC3	0.0105	0.01478	0.11488	0.08143	-0.03572	0.31685	0.19455	-0.35126	0.35126	0.89156	0.84786	0.13461	0.28971	0.64279	2.42E-05	0.01078	2.47E-06	2.47E-06	yellow
APCS	0.07823	-0.02938	-0.05613	0.03767	0.03823	0.12545	0.00163	-0.1179	0.1179	0.30915	0.70283	0.46591	0.62477	0.61955	0.10208	0.98316	0.12459	0.12459	magenta
MIGA1	-0.07717	-0.15496	-0.04273	-0.01721	-0.03865	-0.02228	-0.0506	0.22252	-0.22252	0.31576	0.043	0.57898	0.82321	0.61574	0.77239	0.51098	0.00344	0.00344	turquoise
LIPH	0.04881	0.02009	0.16581	0.09152	-0.03508	0.24212	0.18176	-0.37182	0.37182	0.52609	0.79427	0.03021	0.23383	0.64872	0.00142	0.01735	5.52E-07	5.52E-07	yellow
POMK	-0.06786	-0.08561	-0.02489	0.0077	-0.11636	0.1127	0.13183	0.18896	-0.18896	0.37782	0.26558	0.74661	0.92035	0.12961	0.14221	0.08567	0.01332	0.01332	turquoise
LATS1	-0.06668	-0.12762	-0.01446	0.04181	-0.12069	0.03449	-0.04674	0.35932	-0.35932	0.3862	0.09623	0.85114	0.58713	0.11586	0.65426	0.54386	1.39E-06	1.39E-06	turquoise
ATP5F1C	0.0826	0.07319	-0.00235	-0.03036	0.00728	-0.01237	0.12461	-0.33587	0.33587	0.28281	0.34145	0.97571	0.69345	0.92469	0.8724	0.10441	7.09E-06	7.09E-06	grey
FOXM1	-0.00439	0.06582	0.04362	0.01593	-0.06829	0.06085	0.22973	-0.39003	0.39003	0.95451	0.39239	0.57108	0.83617	0.37482	0.4292	0.00251	1.34E-07	1.34E-07	pink
CHCHD3	0.01118	-0.02057	0.03163	0.02074	-0.02439	-0.00717	0.06272	-0.11166	0.11166	0.88457	0.78946	0.68133	0.78777	0.7515	0.92589	0.41513	0.14597	0.14597	turquoise
F2R	-0.07389	-0.16704	0.00984	0.03066	-0.24504	0.14182	0.01044	0.31584	-0.31584	0.33682	0.02898	0.8984	0.69057	0.00124	0.06428	0.89224	2.58E-05	2.58E-05	blue
CHMP2A	0.14899	0.02738	-0.10395	-0.13616	0.07831	-0.01384	0.09976	-0.46821	0.46821	0.05179	0.72226	0.17604	0.07578	0.30863	0.85743	0.19423	1.06E-10	1.06E-10	grey
AURKA	0.02221	0.06235	0.04006	0.03826	-0.04249	0.03451	0.27295	-0.419	0.419	0.77308	0.41788	0.60287	0.6193	0.58104	0.65409	0.0003	1.17E-08	1.17E-08	pink
PSMB7	0.02274	-0.02435	-0.11983	-0.04405	-0.14843	-0.09642	0.18117	-0.1765	0.1765	0.76785	0.7519	0.1185	0.56729	0.05268	0.20965	0.01772	0.02093	0.02093	grey
NUFIP2	-0.02453	-0.18146	-0.03249	0.0499	-0.07807	0.09156	0.03654	0.00303	-0.00303	0.75009	0.01753	0.67311	0.51692	0.31012	0.23363	0.63513	0.96862	0.96862	turquoise
EREG	0.00345	-0.03637	0.03505	-0.02268	-0.11768	0.09626	0.23105	-0.26514	0.26514	0.96423	0.63672	0.649	0.76845	0.12531	0.2104	0.00236	0.00046	0.00046	grey
LUC7L3	-0.03098	-0.16955	-0.10963	-0.08279	0.15898	-0.10462	-0.08979	0.01177	-0.01177	0.68748	0.02662	0.15347	0.2817	0.0378	0.17327	0.24286	0.8786	0.8786	red
CDK5R1	-0.07282	-0.14045	-0.19387	-0.07807	0.00789	-0.3093	-0.15945	0.29991	-0.29991	0.3439	0.06692	0.01106	0.3101	0.91845	3.85E-05	0.03724	6.74E-05	6.74E-05	brown
DMAC2	0.05152	-0.07045	-0.10251	-0.04363	0.01675	-0.05037	0.07124	-0.15577	0.15577	0.50339	0.35984	0.18214	0.57093	0.8279	0.51295	0.35447	0.04191	0.04191	grey
TMEM9B	0.11192	-0.05158	-0.06284	-0.11962	0.04597	0.01992	0.01752	-0.15649	0.15649	0.14501	0.50284	0.41417	0.11915	0.55044	0.79592	0.82003	0.04096	0.04096	turquoise
IL13RA2	-0.01819	-0.1374	-0.17312	-0.13742	0.03461	-0.28831	-0.08554	0.27296	-0.27296	0.8133	0.07312	0.02355	0.07309	0.65318	0.00013	0.26596	0.0003	0.0003	brown
TAOK3	0.04345	-0.07349	0.10301	0.06687	-0.0364	0.13012	0.07361	0.0392	-0.0392	0.57258	0.33948	0.18	0.38484	0.63649	0.08984	0.33865	0.61068	0.61068	turquoise
TTC30B	-0.10775	-0.1461	-0.12383	0.041	-0.0004	-0.06184	0.02286	0.03557	-0.03557	0.1607	0.05656	0.10661	0.59444	0.9959	0.4217	0.76661	0.64415	0.64415	turquoise
SAP18	0.06784	-0.0316	-0.11377	-0.15415	0.06023	-0.10516	-0.0726	-0.17985	0.17985	0.37794	0.68159	0.13844	0.04411	0.43393	0.17105	0.34536	0.01858	0.01858	grey
SOD2	-0.04234	-0.02937	0.02335	0.11578	-0.24451	0.12289	0.0312	0.20095	-0.20095	0.58239	0.703	0.76177	0.13154	0.00127	0.10931	0.68544	0.0084	0.0084	blue
BRIX1	-0.01589	-0.03219	0.00056	-0.05053	-0.09355	-0.0181	0.13854	-0.19462	0.19462	0.83659	0.67595	0.99422	0.51159	0.22357	0.81424	0.07075	0.01075	0.01075	turquoise
ZNF165	0.06837	0.01362	-0.0164	-0.03408	0.07164	0.13874	0.04743	-0.35596	0.35596	0.37427	0.8597	0.83141	0.65815	0.35178	0.07035	0.53784	1.77E-06	1.77E-06	grey
HSD11B1	-0.01253	0.02136	0.06343	0.07193	-0.2407	0.18226	0.03881	0.3817	-0.3817	0.87079	0.78157	0.40979	0.34986	0.00152	0.01704	0.61429	2.59E-07	2.59E-07	blue
ATIC	0.12388	0.11399	0.04151	0.0075	-0.06788	0.15416	0.274	-0.39708	0.39708	0.10645	0.13766	0.58982	0.92245	0.37768	0.0441	0.00029	7.57E-08	7.57E-08	turquoise
CCL23	-0.09141	-0.05749	0.02093	-0.01229	0.04125	0.05187	-0.04531	0.32268	-0.32268	0.23442	0.45517	0.78587	0.8732	0.5922	0.5005	0.55622	1.67E-05	1.67E-05	blue
MSN	-0.14523	-0.06442	0.06095	0.08634	-0.25372	0.17982	0.08099	0.17959	-0.17959	0.05806	0.40251	0.42843	0.26148	0.00081	0.0186	0.29234	0.01876	0.01876	black
PCBD1	0.11483	0.03274	-0.04095	-0.02902	0.14049	-0.18448	-0.10663	-0.26293	0.26293	0.13479	0.67073	0.59484	0.70633	0.06684	0.01571	0.16512	0.00051	0.00051	grey
PCDHGA6	-0.00336	-0.21014	-0.05165	0.01719	-0.03675	-0.02767	-0.09057	0.25529	-0.25529	0.96525	0.0058	0.50231	0.82343	0.63325	0.7194	0.23878	0.00075	0.00075	turquoise
SLC29A3	0.06673	-0.03806	0.13045	0.15454	-0.14569	0.11369	0.19201	-0.03385	0.03385	0.38588	0.62113	0.08902	0.04358	0.05725	0.13871	0.01187	0.66025	0.66025	grey
NDUFS6	0.12195	0.04819	-0.1402	-0.1054	0.12872	-0.26391	0.12521	-0.31076	0.31076	0.11207	0.53141	0.06741	0.17008	0.09338	0.00049	0.10272	3.52E-05	3.52E-05	green
SERINC5	-0.0057	-0.04781	0.0375	0.0892	-0.07701	0.19201	0.07151	0.03922	-0.03922	0.94103	0.53462	0.62631	0.24599	0.31675	0.01187	0.35266	0.6105	0.6105	turquoise
ASH2L	0.02492	0.07227	0.0739	0.0442	-0.07749	0.06621	0.16209	0.12437	-0.12437	0.74634	0.34753	0.33673	0.56597	0.31374	0.38957	0.03417	0.10509	0.10509	turquoise
NELFE	0.08275	0.04952	-0.12078	-0.11017	0.03262	-0.11759	0.1287	-0.29745	0.29745	0.28195	0.52008	0.11558	0.15143	0.67188	0.12558	0.09343	7.79E-05	7.79E-05	greenyellow
ZDHHC9	-0.00519	-0.08502	0.05308	0.05051	-0.11969	0.20785	0.24682	-0.31958	0.31958	0.94628	0.26891	0.49051	0.51173	0.11892	0.00637	0.00114	2.04E-05	2.04E-05	turquoise
SHTN1	-0.00143	-0.13987	-0.13591	-0.00856	0.01497	-0.08096	-0.04961	0.06884	-0.06884	0.98518	0.06806	0.07633	0.91154	0.84591	0.29251	0.51932	0.37097	0.37097	turquoise
BCL2L14	0.05768	0.11656	0.21797	0.10945	0.03769	0.29321	0.20007	-0.45711	0.45711	0.45366	0.12896	0.00418	0.15413	0.62454	9.94E-05	0.0087	3.29E-10	3.29E-10	yellow
HPF1	-0.15195	-0.02352	-0.19774	-0.09751	-0.00528	-0.16637	-0.04412	0.24434	-0.24434	0.04725	0.76007	0.00953	0.20454	0.94536	0.02965	0.56661	0.00128	0.00128	turquoise
VAX2	-0.23549	0.05018	-0.16627	0.00894	-0.02654	-0.1409	-0.07116	-0.07264	0.07264	0.00193	0.51454	0.02974	0.90762	0.73045	0.06604	0.355	0.34511	0.34511	grey
DLGAP4	0.03891	-0.13827	-0.09061	0.04879	0.04599	-0.10752	0.00243	-0.0264	0.0264	0.61339	0.0713	0.23855	0.52629	0.55028	0.1616	0.97486	0.73181	0.73181	grey
CYP24A1	-0.02268	-0.09437	0.07356	0.02569	-0.22222	0.06496	0.23995	-0.19842	0.19842	0.76842	0.21956	0.33897	0.73872	0.00349	0.39857	0.00157	0.00928	0.00928	grey
CYSLTR1	-0.1097	-0.02875	0.01584	0.06232	-0.18591	0.05971	-0.0007	0.46135	-0.46135	0.15321	0.70897	0.83706	0.41812	0.01491	0.43792	0.99279	2.15E-10	2.15E-10	blue
CXorf65	-0.11324	0.0579	0.05439	-0.03848	0.05782	0.02051	0.03444	0.01941	-0.01941	0.14028	0.45189	0.47982	0.61734	0.45252	0.79004	0.65473	0.80107	0.80107	blue
IMPDH2	0.14385	0.12958	0.093	-0.07355	-0.11588	0.21985	0.25916	-0.23041	0.23041	0.06051	0.09118	0.22634	0.33905	0.13124	0.00386	0.00062	0.00243	0.00243	purple

TST	-0.08253	0.09605	0.00191	0.05932	0.14086	0.18332	0.16886	-0.44381	0.44381	0.28322	0.21141	0.98022	0.44088	0.06611	0.0164	0.02726	1.20E-09	1.20E-09	yellow
CNR1	-0.03344	-0.1142	-0.10073	0.04716	-0.06241	-0.01363	-0.13049	0.47114	-0.47114	0.66416	0.13696	0.18988	0.54016	0.41741	0.85956	0.08891	7.85E-11	7.85E-11	blue
STYXL1	0.07861	-0.08638	0.04908	0.05301	-0.04642	0.03385	0.20203	-0.39687	0.39687	0.30679	0.26127	0.52384	0.49109	0.54656	0.66032	0.00805	7.70E-08	7.70E-08	grey
ABCB8	-0.00341	-0.04734	-0.11343	-0.06949	0.22939	-0.21824	-0.05319	-0.07485	0.07485	0.9647	0.53869	0.13964	0.36643	0.00254	0.00414	0.48962	0.33058	0.33058	green
DLG3	0.01411	-0.06506	-0.0509	-0.06078	-0.00796	0.11254	0.07762	-0.38629	0.38629	0.85468	0.39784	0.50852	0.42974	0.91768	0.14278	0.31295	1.80E-07	1.80E-07	grey
GREM2	0.00024	-0.05961	0.01697	0.06176	-0.06752	0.07864	0.00171	0.25253	-0.25253	0.99751	0.43865	0.82567	0.4223	0.38025	0.30661	0.9823	0.00086	0.00086	salmon
TMEM127	0.05097	-0.04392	-0.00369	0.06851	-0.20011	0.13004	0.13351	-0.05879	0.05879	0.50792	0.56844	0.96176	0.37325	0.00869	0.09003	0.08171	0.44498	0.44498	turquoise
TYMS	-0.12915	-0.01324	0.05342	-0.05278	0.07358	0.05955	0.24316	-0.31882	0.31882	0.09226	0.86356	0.48771	0.49296	0.33884	0.4391	0.00135	2.14E-05	2.14E-05	pink
BRSK2	-0.06058	0.02842	-0.11056	0.02568	0.03714	-0.21281	-0.2352	0.35712	-0.35712	0.43121	0.71217	0.14998	0.73883	0.62965	0.0052	0.00196	1.63E-06	1.63E-06	yellow
ADAMTS1	-0.03274	-0.02953	0.03	0.10244	-0.11385	0.05607	0.17462	-0.0216	0.0216	0.6708	0.7014	0.69689	0.18243	0.13816	0.46635	0.02236	0.77917	0.77917	black
COL5A3	-0.09837	0.03521	-0.01465	0.04238	-0.09988	-0.07068	0.08172	0.2201	-0.2201	0.20055	0.6475	0.84915	0.58208	0.19369	0.35828	0.28799	0.00382	0.00382	black
FBXO30	-0.10491	-0.11161	-0.00232	0.06243	-0.12233	0.0031	-0.07271	0.44473	-0.44473	0.17205	0.14614	0.97599	0.41729	0.11096	0.96787	0.34462	1.10E-09	1.10E-09	turquoise
GLDN	0.02957	0.05761	0.0737	0.10751	-0.14211	0.12578	0.06167	0.14497	-0.14497	0.70102	0.45416	0.33809	0.16161	0.06373	0.10115	0.42293	0.05852	0.05852	grey
RNF122	-0.13138	-0.1049	0.03372	0.17447	-0.11566	0.06036	-0.00719	0.34789	-0.34789	0.08675	0.17212	0.6615	0.02247	0.13195	0.43291	0.9256	3.13E-06	3.13E-06	blue
INTS12	-0.15592	-0.19137	-0.05989	0.00094	-0.0328	0.05352	0.04773	-0.01469	0.01469	0.04171	0.01217	0.43648	0.99026	0.67019	0.48694	0.53531	0.84873	0.84873	turquoise
PDXP	-0.0297	-0.08734	-0.19596	-0.10961	0.23608	-0.28395	-0.16298	0.14028	-0.14028	0.69979	0.256	0.01021	0.15352	0.00188	0.00017	0.03318	0.06725	0.06725	grey
FAM92A	0.01681	-0.13807	-0.10213	-0.03949	-0.00986	-0.25125	-0.13958	0.28675	-0.28675	0.82728	0.07172	0.18378	0.60809	0.89812	0.00092	0.06863	0.00014	0.00014	turquoise
MALL	0.03753	-0.04585	0.05649	0.08204	-0.07821	0.1433	0.23632	-0.33217	0.33217	0.626	0.55148	0.46306	0.28609	0.30924	0.0615	0.00186	9.06E-06	9.06E-06	turquoise
SGPL1	0.02253	-0.16602	0.07611	0.10726	-0.20316	-0.00953	0.04769	0.0194	-0.0194	0.7699	0.03	0.32247	0.1626	0.0077	0.9015	0.53568	0.80117	0.80117	turquoise
SERPINA5	0.04943	-0.03037	-0.01609	0.09998	-0.11761	0.13415	0.01007	0.03383	-0.03383	0.52089	0.69338	0.83452	0.19324	0.12553	0.08025	0.89601	0.66045	0.66045	magenta
GPKOW	0.0525	-0.07577	-0.06289	-0.03547	-0.01206	-0.06417	0.1228	-0.18751	0.18751	0.49527	0.32461	0.41386	0.64514	0.87558	0.40439	0.10959	0.01405	0.01405	turquoise
ADGRA2	-0.05669	-0.13239	-0.02174	0.04446	-0.14141	0.06954	-0.04899	0.50152	-0.50152	0.46145	0.08433	0.77776	0.5637	0.06505	0.36614	0.52458	2.80E-12	2.80E-12	blue
PFKFB4	-0.05892	-0.14658	0.03962	0.03917	0.0661	-0.03323	0.00536	-0.16752	0.16752	0.44395	0.05574	0.60686	0.61102	0.39037	0.66611	0.94453	0.02852	0.02852	grey
SAMD13	-0.06182	0.01082	0.02145	0.0254	0.05937	0.11095	0.03718	-0.15521	0.15521	0.42183	0.88835	0.78069	0.74158	0.44052	0.14855	0.62924	0.04266	0.04266	grey
ZNF385D	-0.12131	-0.09859	-0.0732	0.03065	-0.06646	-0.03045	-0.04851	0.49478	-0.49478	0.11399	0.19952	0.34136	0.69066	0.3878	0.69259	0.52866	6.05E-12	6.05E-12	blue
ZNF839	-0.04897	-0.13662	-0.09631	-0.07583	0.16048	-0.09918	-0.15046	0.13097	-0.13097	0.52477	0.07479	0.21016	0.32423	0.03602	0.19683	0.0495	0.08775	0.08775	turquoise
TFE3	-0.05067	-0.22272	-0.03954	0.035	-0.12868	0.0437	0.00305	0.14877	-0.14877	0.5104	0.00341	0.60765	0.64947	0.09348	0.57035	0.96844	0.05214	0.05214	turquoise
COPE	0.0803	0.06129	-0.18343	-0.0742	0.26997	-0.26896	-0.00496	-0.19483	0.19483	0.29647	0.42583	0.01633	0.33482	0.00036	0.00037	0.94867	0.01066	0.01066	green
MGAT1	-0.0682	-0.10348	-0.01742	0.05375	-0.10318	0.00202	0.03562	0.32975	-0.32975	0.37545	0.17801	0.8211	0.48501	0.1793	0.97912	0.6437	1.06E-05	1.06E-05	blue
LRRC36	0.09755	0.01197	-0.09179	0.04174	0.18853	-0.10083	-0.10328	0.11889	-0.11889	0.20435	0.87656	0.23246	0.58783	0.01353	0.18947	0.17887	0.12143	0.12143	grey
EIF2S1	-0.04432	-0.03906	0.08328	0.06084	-0.15923	0.1647	0.17288	-0.04486	0.04486	0.56487	0.61203	0.27883	0.42925	0.03751	0.03134	0.02375	0.56015	0.56015	turquoise
ACADS	0.04394	0.03876	0.02467	0.00808	0.19693	0.09822	0.0838	-0.31401	0.31401	0.56826	0.61475	0.74876	0.9165	0.00983	0.20123	0.27584	2.88E-05	2.88E-05	grey
SUPT5H	0.14681	-0.2334	-0.01321	0.04028	-0.08209	-0.01012	0.04867	-0.09921	0.09921	0.05535	0.00212	0.86383	0.60094	0.28578	0.89552	0.52729	0.19669	0.19669	grey
NISCH	0.01534	-0.12645	-0.13844	-0.08927	0.04027	-0.14358	-0.14371	0.38566	-0.38566	0.84219	0.09934	0.07095	0.24561	0.601	0.061	0.06076	1.90E-07	1.90E-07	brown
SYNGR4	-0.01513	-0.06372	-0.20238	-0.12615	0.13837	-0.25616	-0.13452	0.22963	-0.22963	0.84425	0.40772	0.00794	0.10016	0.07109	0.00072	0.0794	0.00252	0.00252	brown
LIG3	-0.05939	-0.07768	-0.037	0.00297	0.05296	-0.04282	0.00199	0.07102	-0.07102	0.44035	0.31255	0.63087	0.96922	0.49152	0.57815	0.97941	0.35594	0.35594	turquoise
DPF2	-0.00473	-0.19262	-0.06027	-0.01711	-0.15059	0.00095	0.03343	0.05547	-0.05547	0.95105	0.0116	0.43356	0.82422	0.04929	0.99011	0.6642	0.47119	0.47119	turquoise
HNRNPM	0.02141	-0.03348	-0.1105	-0.10244	0.04245	-0.08377	0.06282	0.01259	-0.01259	0.7811	0.66374	0.15021	0.18242	0.58145	0.27604	0.41433	0.8702	0.8702	grey
CCT2	0.0749	-0.13097	-0.01892	0.05205	0.03744	-0.04522	-0.0108	-0.08655	0.08655	0.33023	0.08774	0.80602	0.49901	0.62685	0.55697	0.88847	0.26036	0.26036	turquoise
IFITM10	0.03472	-0.0235	-0.04242	-0.0316	-0.13786	0.04347	0.25478	-0.30665	0.30665	0.65213	0.76028	0.5817	0.68164	0.07216	0.57236	0.00077	4.52E-05	4.52E-05	grey
LAMA3	0.00437	0.03432	0.13317	0.08855	-0.11485	0.20383	0.20202	-0.3298	0.3298	0.95481	0.65586	0.08251	0.24947	0.13471	0.0075	0.00805	1.06E-05	1.06E-05	turquoise
TMEM161	0.01561	-0.05531	-0.01134	0.00363	0.03745	0.01049	0.0091	-0.04995	0.04995	0.8394	0.47243	0.88298	0.96245	0.62677	0.89166	0.90597	0.51648	0.51648	turquoise
PIK3CD	-0.14753	-0.03184	0.06377	0.06419	-0.19636	0.11565	0.04008	0.37248	-0.37248	0.05415	0.67929	0.40732	0.40426	0.01005	0.132	0.60273	5.25E-07	5.25E-07	blue
TXN2	-0.07954	0.11042	-0.14467	-0.09393	0.09327	-0.07294	0.05513	-0.11634	0.11634	0.30106	0.15049	0.05904	0.22173	0.22501	0.34309	0.47388	0.1297	0.1297	grey
TCF25	0.03659	0.01096	-0.06363	-0.06384	0.05565	-0.04456	0.09728	-0.05929	0.05929	0.63472	0.88687	0.40838	0.40677	0.4697	0.56279	0.20559	0.44109	0.44109	grey
MAP2K3	0.01329	-0.07024	0.0285	0.042	-0.04781	0.08267	0.03822	-0.23416	0.23416	0.86303	0.36128	0.71135	0.58548	0.53462	0.28239	0.61971	0.00205	0.00205	grey
CHST1	-0.07074	-0.09584	-0.14226	0.02149	-0.09336	-0.0259	-0.00956	0.26884	-0.26884	0.3579	0.21239	0.06344	0.78021	0.22455	0.7367	0.90123	0.00038	0.00038	black
CHPF2	-0.01686	-0.0864	-0.03478	-0.05189	0.00568	-0.16285	0.07282	0.02839	-0.02839	0.82676	0.26119	0.65154	0.50032	0.94127	0.03332	0.34391	0.71245	0.71245	grey
UCP2	-0.09298	-0.03384	0.00264	-0.04739	0.09815	-0.07936	-0.18404	0.16645	-0.16645	0.22647	0.66035	0.97266	0.53818	0.20153	0.3022	0.01597	0.02956	0.02956	grey
PTGER3	-0.00769	-0.04562	0.00763	0.10726	-0.12862	0.03436	-0.09095	0.63896	-0.63896	0.9205	0.55353	0.92115	0.16262	0.09362	0.65549	0.2368	5.28E-21	5.28E-21	blue
GOLGA5	0.02034	-0.08774	-0.01274	0.07007	-0.02022	0.04036	0.02324	-0.0068	0.0068	0.79178	0.25383	0.86869	0.36247	0.79297	0.60023	0.76285	0.92968	0.92968	turquoise
HPD	0.02201	-0.05925	0.07753	0.08817	-0.0388	0.05635	-0.10315	0.24415	-0.24415	0.77507	0.44139	0.31348	0.2515	0.61442	0.46416	0.17943	0.00129	0.00129	grey
PDLIM2	-0.02052	-0.02586	0.128	0.1013	0.00773	0.00339	0.06601	0.13416	-0.13416	0.78996	0.73704	0.09522	0.1874	0.92006	0.96487	0.39102	0.08022	0.08022	black
ARPC2	0.01086	0.00033	0.06012	0.0183	-0.13063	0.16925	0.18543	-0.08786	0.08786	0.88793	0.99653	0.43471	0.8122	0.08858	0.0269	0.01518	0.25315	0.25315	turquoise
HNF1B	0.01637	0.02815	-0.01149	0.10501	0.04329	0.211	0.0443	-0.10016	0.10016	0.83166	0.71477	0.88146	0.17167	0.57402	0.0056	0.56511	0.19241	0.19241	yellow
ITPRIP	-0.06834	-0.11888	0.09552	0.07709	-0.22107	0.15181	0.06275	0.23811	-0.23811	0.37444	0.12146	0.21393	0.31628	0.00366	0.04746	0.41487	0.00171	0.00171	black
PDCD2	0.00879	0.05222	-0.03097	-0.06649	-0.04167	-0.04703	-0.03302	0.10276	-0.10276	0.90919	0.49761	0.68765	0.38758	0.5884	0.54132	0.66808	0.18107	0.18107	grey
KXD1	0.05865	-0.00994	-0.03257	-0.04253	0.02785	-0.01013	0.08668	-0.17243	0.17243	0.4461	0.89731	0.67239	0.58072	0.71771	0.89537	0.25962	0.02412	0.02412	grey
ASTE1	-0.01903	-0.06868	-0.11077	-0.02951	-0.04512	0.14746	0.07149	0.13822	-0.13822	0.80485	0.37209	0.14921	0.70163	0.55792	0.05426	0.35279	0.07141	0.07141	turquoise
TCIM	-0.01483	-0.15119	0.03306	0.08217	0.05007	0.14186	0.00775	-0.0024	0.0024	0.84729	0.04839	0.66776	0.28532	0.51549	0.0642	0.91988	0.97516	0.97516	grey
BTBD7	-0.03389	-0.12418	-0.00869	0.11759	-0.09845	0.0932	0.00575	0.12833	-0.12833	0.65987	0.10561	0.91023	0.12559	0.20017	0.22535	0.94046	0.09437	0.09437	turquoise
FRMD3	0.08279	-0.06688	-0.03529	0.04698	-0.0843	0.07889	0.11543	0.14733	-0.14733	0.2817	0.3848	0.64678	0.54172	0.27296	0.30505	0.13273	0.05448	0.05448	grey
PHGR1	0.07819	0.09041	0.00643	-0.06176	0.24319	0.06998	0.0122	-0.28118	0.28118	0.30938	0.23961	0.93348	0.42228	0.00135	0.36306	0.87418	0.0002	0.0002	grey
NSRP1	-0.13586	-0.14109	-0.04007	-0.03488	-0.02365	0.00147	-0.0363	0.18605	-0.18605	0.07643	0.06568	0.60282	0.65063	0.75884	0.98475	0.63735	0.01484	0.01484	turquoise
EMCN	-0.20771	-0.07187	-0.07775	0.05666	-0.05643	-0.03315	-0.18983	0.63765	-0.63765	0.00641	0.35022	0.31214	0.46165	0.4635	0.66684	0.01289	6.72E-21	6.72E-21	blue
SIGMAR1	0.02238	0.03231	-0.08532	-0.05092	-0.02328	0.07551	0.11395	-0.17834	0.17834	0.77135	0.67486	0.2672	0.50832	0.76248	0.32632	0.13779	0.01961	0.01961	grey
VAMP7	-0.10799	-0.14764	0.01278	0.04212	-0.17889	0.09413	0.03853	0.32859	-0.32859	0.15974	0.05398	0.86824	0.58434	0.01923	0.22074	0.61686	1.14E-05	1.14E-05	blue
BGN	-0.05437	-0.10623	0.02143	0.07429	-0.16392	0.12034	0.02427	0.20707	-0.20707	0.47997	0.16672	0.78082	0.33421	0.03216	0.11692	0.75271	0.00658	0.00658	black
PCDHB2	-0.04278	0.03089	0.00738	-0.00201	-0.13495	0.09473	0.0069	0.08751	-0.08751	0.57849	0.68841	0.92364	0.97918	0.07845	0.21779	0.92861	0.25505	0.25505	grey
TIGD1	0.05503	-0.08659	-0.0909	-0.09169	0.14636	-0.01562	0.07515	-0.2916	0.2916	0.47472	0.26011	0.23705	0.23297	0.05611	0.83934	0.32866	0.00011	0.00011	red
FOXP3	-0.1459	-0.04344	0.08857	0.06277	-0.19278	0.16004	-0.00045	0.37463	-0.37463	0.05689	0.57261	0.24936	0.41472	0.01153	0.03654	0.9953	4.46E-07	4.46E-07	blue
THEM5	0.00652	-0.04813	0.02203	-0.06341	0.03423	0.02967	0.02378	-0.06059	0.06059	0.93251	0.5319	0.77493	0.40994	0.65671	0.70003	0.75751	0.43112	0.43112	grey
WDR25	0.15108	0.01708	-0.11549	-0.04748	0.20221	-0.14225	-0.09752	-0.07918	0.07918	0.04856	0.82454	0.13254	0.53748	0.00799	0.06346	0.20446	0.3033	0.3033	grey
OSGIN2	0.00617	-0.13776	-0.01995	0.03205	-0.27206	0.06642	0.15325	0.08545	-0.08545	0.9362	0.07236	0.79569	0.67735	0.00032	0.38805	0.04538	0.26644	0.26644	turquoise
GCA	-0.1105	-0.04213	0.08391	0.14414	-0.13797	0.16954	0.07384	0.01213	-0.01213	0.15022	0.58428	0.27519	0.05998	0.07192	0.02664	0.33713	0.87492	0.87492	turquoise
SPINDOC	0.00285	-0.25416	-0.04637	0.02023	-0.00441	-0.1165	-0.02451	0.03838	-0.03838	0.97052	0.0008	0.54701	0.79283	0.95438	0.12916	0.75035	0.61819	0.61819	grey
PAH	0.02755	-0.08971	-0.14184	0.01625	0.07365	-0.08957	-0.14092	0.21258	-0.21258	0.72061	0.24327	0.06424	0.8329	0.33837	0.244	0.06599	0.00525	0.00525	magenta
LBH	-0.03976	-0.22578	0.04311	0.06952	-0.13221	0.08391	-0.0902	0.17438	-0.17438	0.60565	0.00299	0.5756	0.36627	0.08476	0.27521	0.24067	0.02254	0.02254	turquoise
HEATR5B	-0.07954	-0.14105	0.00028	0.02655	-0.09153	0.06726	0.01185	0.19634	-0.19634	0.30109	0.06575	0.99712	0.73036	0.23378	0.38205	0.87774	0.01006	0.01006	turquoise
BAIAP3	0.05329	-0.13127	-0.17619	-0.11133	0.14611	-0.25445	-0.11418	0.17537	-0.17537	0.48882	0.08702	0.02116	0.14714	0.05654	0.00078	0.13701	0.02178	0.02178	brown
ZNF473	0.11068	-0.13701	-0.20553	-0.07351	0.05937	-0.14618	-0.08471	0.12115	-0.12115	0.14955	0.07395	0.007	0.33932	0.44053	0.05642	0.27063	0.11447	0.11447	turquoise
ZNF791	-0.06276	-0.11521	-0.16466	-0.07733	0.00824	-0.10543	-0.15378	0.28387	-0.28387	0.41482	0.13347	0.03139	0.31474	0.91482	0.16994	0.04463	0.00017	0.00017	turquoise
AKAP13	-0.10139	-0.12347	0.03251	0.07804	-0.16395	0.19137	0.01785	0.21404	-0.21404	0.18701	0.10765	0.6729	0.31034	0.03214	0.01217	0.81673	0.00494	0.00494	turquoise
CMPK2	-0.04203	0.03384	0.0446	0.06407	-0.17579	0.08599	0.13762	0.01039	-0.01039	0.58521	0.6604	0.56245	0.40513	0.02145	0.26343	0.07266	0.89273	0.89273	tan
ADAMTS1	-0.03997	-0.06587	0.05962	0.14957	-0.12433	0.11275	-0.08323	0.49598	-0.49598	0.60374	0.39198	0.43861	0.05088	0.10518	0.14205	0.27916	5.28E-12	5.28E-12	blue
LPIN1	-0.03575	-0.17583	0.04943	-0.04492	-0.02322	0.12661	-0.11755	0.00097	-0.00097	0.64251	0.02143	0.52087	0.55961	0.7631	0.09893	0.12573	0.98999	0.98999	turquoise
RPL21	0.18058	0.07448	-0.08772	-0.11791	-0.06852	-0.05304	0.06765	0.0712	-0.0712	0.0181	0.33295	0.2539	0.12457	0.3732	0.49081	0.37935	0.35477	0.35477	purple
ABCA9	-0.05869	-0.14455	-0.03467	0.08724	-0.12804	0.04203	-0.12324	0.61722	-0.61722	0.44573	0.05925	0.65262	0.25655	0.09513	0.58517	0.10829	2.47E-19	2.47E-19	blue
LZIC	-0.06767	-0.14509	-0.01036	0.03257	-0.13575	0.14706	0.04341	0.21921	-0.21921	0.37918	0.0583	0.893	0.67234	0.07667	0.05493	0.5729	0.00397	0.00397	turquoise
GOLT1A	0.15292	-0.05544	-0.12789	-0.15506	0.1232	-0.14164	0.02801	-0.35821	0.35821	0.04585	0.47138	0.09552	0.04286	0.1084	0.06461	0.71608	1.51E-06	1.51E-06	grey
NRG2	-0.03046	-0.01114	-0.19753	-0.07205	0.05318	-0.33014	-0.26512	0.46552	-0.46552	0.69251	0.88499	0.00961	0.34905	0.4897	1.03E-05	0.00046	1.40E-10	1.40E-10	brown
TSHZ2	-0.0043	-0.11851	-0.07635	-0.00539	-0.23424	0.12369	-0.01915	0.37806	-0.37806	0.95553	0.12263	0.32095	0.9442	0.00204	0.107	0.80366	3.43E-07	3.43E-07	blue
CAMSAP1	-0.01257	-0.22322	-0.07311	-0.00944	-0.18183	-0.03025	0.00488	0.15048	-0.15048	0.8704	0.00334	0.34193	0.90249	0.01731	0.69446	0.94952	0.04947	0.04947	turquoise
RBM10	0.11304	-0.12389	-0.19708	-0.12452	0.17713	-0.21151	-0.06404	-0.08356	0.08356	0.14099	0.10643	0.00978	0.10465	0.02047	0.00548	0.4053	0.27723	0.27723	green
HDAC5	0.01937	-0.14442	-0.2293	-0.13005	0.11016	-0.2256	-0.17167	0.26128	-0.26128	0.80143	0.05949	0.00255	0.09002	0.15146	0.00301	0.02476	0.00056	0.00056	brown
FOXC1	-0.09812	-0.04729	0.00858	0.06126	-0.21834	0.16553	0.23453	-0.1186	0.1186	0.20169	0.53907	0.91129	0.42609	0.00412	0.03049	0.00202	0.12235	0.12235	turquoise
GAS1	-0.06272	-0.03099	0.07163	0.14194	-0.20993	0.07401	0.06435	0.38063	-0.38063	0.41513	0.68743	0.35184	0.06403	0.00585	0.33602	0.40306	2.81E-07	2.81E-07	blue
FIG4	-0.04587	-0.05965	0.02225	-0.03475	-0.03708	-0.02686	-0.01873	0.35505	-0.35505	0.55137	0.43834	0.77273	0.65184	0.63013	0.72734	0.80792	1.89E-06	1.89E-06	turquoise
HMMR	-0.01417	0.03546	0.03826	-0.01287	-0.05794	0.1084	0.30098	-0.34521	0.34521	0.85401	0.64522	0.61933	0.86727	0.4516	0.15815	6.33E-05	3.76E-06	3.76E-06	pink
DDX3X	-0.34301	-0.16687	0.0209	0.06484	-0.12416	0.12456	-0.0409	0.25709	-0.25709	4.38E-06	0.02915	0.78615	0.39946	0.10568	0.10454	0.59532	0.00069	0.00069	turquoise
CRTC3	-0.0148	-0.21262	-0.03328	0.05698	-0.16516	0.06804	-0.03727	0.3607	-0.3607	0.84761	0.00524	0.6657	0.45915	0.03087	0.37654	0.62837	1.26E-06	1.26E-06	blue
ASMTL	-0.05588	-0.08471	-0.1745	-0.00655	0.00732	-0.10255	-0.0375	0.22363	-0.22363	0.46791	0.27063	0.02245	0.9322	0.92432	0.18197	0.62628	0.00328	0.00328	grey
SLC17A9	0.03797	0.07951	0.08869	0.00799	0.1454	0.15979	0.1356	-0.46998	0.46998	0.62196	0.30126	0.24866	0.91739	0.05777	0.03684	0.077	8.86E-11	8.86E-11	yellow
CCNY	-0.07814	-0.10039	0.01664	0.06706	-0.17673	0.03872	0.06015	0.24565	-0.24565	0.30968	0.19139	0.829	0.38351	0.02076	0.61507	0.43451	0.0012	0.0012	turquoise
ZFP36L1	-0.01674	-0.02711	0.1321	0.18143	-0.17644	0.33352	0.18995	-0.00055	0.00055	0.82799	0.72484	0.08502	0.01756	0.02097	8.29E-06	0.01283	0.99427	0.99427	black
BICD1	-0.08959	-0.179	-0.00067	-0.02811	-0.12574	0.01227	0.0565	0.08619	-0.08619	0.2439	0.01916	0.99306	0.71514	0.10127	0.87347	0.46291	0.26232	0.26232	turquoise
SNX8	-0.03805	0.00775	0.03616	0.01732	-0.0972	0.12095	0.25349	-0.11944	0.11944	0.62126	0.91983	0.63866	0.82213	0.20597	0.11507	0.00082	0.11971	0.11971	grey
KRT15	0.03392	0.02595	0.10004	-0.00067	0.02668	0.0861	0.22715	-0.45224	0.45224	0.65966	0.73618	0.19298	0.99308	0.72907	0.26281	0.00281	5.32E-10	5.32E-10	yellow
MERTK	-0.09408	-0.07179	0.02887	0.09731	-0.21878	0.05105	0.02619	0.51536	-0.51536	0.22097	0.35076	0.70781	0.20543	0.00404	0.50724	0.73388	5.50E-13	5.50E-13	blue
LRP4	-0.03109	-0.15822	0.09015	0.08452	-0.03598	0.2037	0.09706	0.05799	-0.05799	0.6865	0.03874	0.24095	0.27171	0.64038	0.00754	0.20663	0.45119	0.45119	grey
TBCB	0.05122	-0.09109	-0.13147	-0.07324	0.04357	-0.19337	-0.04267	-0.10178	0.10178	0.50587	0.23607	0.08653	0.34111	0.57149	0.01128	0.57947	0.18527	0.18527	grey
MYO1F	-0.14808	0.001	0.05255	0.06405	-0.17896	0.12418	0.08746	0.38979	-0.38979	0.05326	0.9896	0.49484	0.40527	0.01918	0.1056	0.25536	1.37E-07	1.37E-07	blue
SIDT2	-0.03077	-0.04585	0.08271	0.04642	#######	-0.02422	-0.13748	0.40752	-0.40752	0.68949	0.55152	0.28216	0.54654	0.99987	0.75317	0.07296	3.17E-08	3.17E-08	grey
PDZRN4	-0.0075	-0.00608	0.03018	0.07481	-0.0703	0.07406	-0.03511	0.41352	-0.41352	0.92242	0.93707	0.6952	0.3308	0.36087	0.33569	0.64846	1.89E-08	1.89E-08	salmon
TOMM22	-0.05113	0.12055	-0.06026	-0.07177	-0.01112	0.00226	0.13416	-0.22926	0.22926	0.50657	0.1163	0.43367	0.3509	0.88518	0.97656	0.08022	0.00256	0.00256	turquoise
PSMA1	-0.03591	-0.06807	0.03018	-0.02918	-0.05317	-0.00239	0.16219	-0.20958	0.20958	0.64097	0.37638	0.69521	0.70483	0.48976	0.97526	0.03405	0.00594	0.00594	turquoise
ANKRD53	-0.11782	0.02566	-0.0068	0.15053	-0.16546	-0.003	-0.13063	0.49924	-0.49924	0.12484	0.73903	0.92966	0.0494	0.03057	0.96892	0.08857	3.65E-12	3.65E-12	grey
VPS4A	0.07414	-0.02208	-0.07174	-0.08354	-0.01707	-0.05298	0.1521	-0.23688	0.23688	0.33522	0.77441	0.35113	0.27734	0.82464	0.49131	0.04703	0.00181	0.00181	grey
BCAR1	0.01598	-0.0618	-0.09223	-0.04418	0.12414	0.02775	0.11568	-0.3017	0.3017	0.83566	0.42195	0.23022	0.56612	0.10574	0.71867	0.13189	6.07E-05	6.07E-05	green
MRPS25	0.07525	0.03708	-0.03188	-0.12736	0.02905	0.06439	0.15045	-0.26313	0.26313	0.32801	0.63017	0.67896	0.0969	0.70604	0.40278	0.04951	0.00051	0.00051	grey
NUP160	-0.06986	-0.10883	0.04248	0.04001	-0.18612	0.13135	0.08593	0.14017	-0.14017	0.36394	0.15651	0.5812	0.60332	0.0148	0.08682	0.26377	0.06746	0.06746	turquoise
IMPA2	0.11578	0.08172	0.15199	0.1087	0.08475	0.23857	0.09408	-0.19924	0.19924	0.13156	0.28795	0.04721	0.15702	0.27043	0.00168	0.22095	0.00899	0.00899	grey
MMP3	-0.04716	0.06939	0.09282	0.08893	-0.05232	0.09905	0.07272	0.15196	-0.15196	0.54023	0.36714	0.22724	0.24743	0.49677	0.19743	0.34456	0.04724	0.04724	grey
CEMIP2	-0.03891	-0.05381	0.04994	0.11266	-0.13072	0.29221	0.09156	0.02336	-0.02336	0.61339	0.48454	0.51655	0.14235	0.08836	0.00011	0.23363	0.76173	0.76173	turquoise
LEPROT	-0.09657	-0.1621	0.06739	0.0912	-0.18201	0.15517	0.01961	0.21101	-0.21101	0.20895	0.03416	0.38112	0.23548	0.01719	0.04272	0.79909	0.0056	0.0056	turquoise
USP43	0.07462	-0.08982	-0.02595	0.05245	0.04662	-0.03778	0.14245	-0.47322	0.47322	0.33207	0.24269	0.73619	0.4957	0.54482	0.62368	0.06309	6.31E-11	6.31E-11	grey
DDX54	0.08875	0.04976	0.01395	-0.0197	0.12143	-0.0058	0.09147	-0.25862	0.25862	0.24836	0.5181	0.85632	0.79815	0.11362	0.93994	0.23409	0.00064	0.00064	green
PLPBP	0.03901	0.02907	0.019	0.08653	-0.02677	0.09262	0.06348	0.14367	-0.14367	0.61243	0.70582	0.80516	0.26047	0.72821	0.22826	0.40943	0.06083	0.06083	turquoise
SCNN1G	0.07285	-0.07775	-0.02117	0.09783	-0.06206	-0.07125	-0.04297	0.04701	-0.04701	0.34367	0.31213	0.78348	0.20305	0.42004	0.35443	0.57682	0.54148	0.54148	grey

VEZT	-0.06064	-0.14184	0.06657	0.05903	0.01667	0.12503	0.04762	-0.131	0.131	0.43073	0.06424	0.38701	0.44314	0.8287	0.10323	0.53625	0.08767	0.08767	turquoise
BLCAP	0.14292	-0.05794	-0.04771	0.00319	0.10938	-0.0796	0.1074	-0.33632	0.33632	0.06222	0.45159	0.53551	0.96699	0.15442	0.30072	0.16206	6.88E-06	6.88E-06	grey
MUTYH	-0.01698	0.02086	0.02212	-0.07819	0.17379	0.07206	0.03337	-0.26464	0.26464	0.82557	0.78652	0.77401	0.30936	0.02301	0.34899	0.66476	0.00047	0.00047	red
TROAP	0.0898	0.07524	0.01269	-0.03633	0.05374	0.01892	0.23565	-0.51375	0.51375	0.24277	0.32808	0.86912	0.63712	0.48509	0.80596	0.00192	6.67E-13	6.67E-13	pink
AP4B1	0.01456	0.00095	0.03209	-0.00328	0.10554	0.08595	0.0425	-0.2615	0.2615	0.85007	0.99019	0.67689	0.96603	0.1695	0.26365	0.58098	0.00055	0.00055	grey
MICU2	-0.00756	-0.0618	-0.11031	-0.11381	-0.01218	-0.06295	-0.09224	0.15683	-0.15683	0.92183	0.42201	0.15093	0.13828	0.8744	0.41337	0.23018	0.04052	0.04052	turquoise
NDUFA10	0.12965	0.04561	-0.14441	-0.07988	0.08344	-0.02166	0.12378	-0.19252	0.19252	0.091	0.55362	0.05951	0.29901	0.27791	0.77861	0.10675	0.01164	0.01164	turquoise
OTUD3	0.03256	-0.24597	-0.05035	-0.01071	0.10855	-0.05302	-0.11101	0.12613	-0.12613	0.67249	0.00118	0.51314	0.88941	0.15759	0.49103	0.14831	0.10021	0.10021	red
DNAJC25	-0.02893	-0.19566	-0.1502	-0.01045	-0.03096	-0.15115	-0.09653	0.11854	-0.11854	0.70719	0.01033	0.04989	0.89205	0.68769	0.04844	0.20911	0.12255	0.12255	turquoise
LRRC55	-0.09402	-0.07359	-0.03374	-0.00456	-0.18003	0.08873	-0.07724	0.4101	-0.4101	0.22127	0.33881	0.66127	0.95279	0.01847	0.24846	0.3153	2.54E-08	2.54E-08	blue
EIF5A	-0.04118	0.00401	-0.0482	-0.06597	-0.05112	-0.10425	0.16388	-0.21219	0.21219	0.59282	0.95851	0.53133	0.39126	0.50672	0.17479	0.03221	0.00533	0.00533	pink
TRAPPC11	-0.138	-0.13658	-0.03828	-0.00837	-0.02567	0.03832	-0.02289	0.22987	-0.22987	0.07187	0.07486	0.61909	0.91349	0.73896	0.61878	0.76637	0.00249	0.00249	turquoise
ASCL5	0.09968	0.12148	-0.04539	0.00431	0.12471	-0.06638	-0.05912	-0.07411	0.07411	0.19457	0.11347	0.55552	0.95539	0.10412	0.38834	0.44245	0.33538	0.33538	grey
IGDCC4	-0.11683	-0.12089	0.0165	0.05112	-0.13074	0.07233	0.09659	0.37452	-0.37452	0.12807	0.11525	0.83037	0.5067	0.08831	0.34715	0.20883	4.50E-07	4.50E-07	blue
CPS1	0.05722	-0.04925	0.08926	0.03692	0.08201	0.09715	0.1292	-0.11507	0.11507	0.45726	0.52234	0.24562	0.6316	0.28628	0.2062	0.09214	0.13395	0.13395	grey
PTGER1	0.03539	0.0246	-0.00798	-0.0765	0.02578	-0.00374	0.11132	-0.08048	0.08048	0.64588	0.74941	0.91748	0.31998	0.73786	0.96133	0.14717	0.29537	0.29537	grey
GPATCH11	0.02032	-0.11218	-0.03653	-0.02286	-0.12191	0.04802	0.00628	0.32674	-0.32674	0.79197	0.14405	0.63522	0.76668	0.11219	0.53282	0.93505	1.29E-05	1.29E-05	turquoise
STARD3	0.01082	-0.01057	-0.03283	0.03603	0.18493	-0.15839	-0.01861	-0.04353	0.04353	0.88829	0.89091	0.66993	0.63988	0.01546	0.03854	0.80914	0.57187	0.57187	grey
MAIP1	0.03124	-0.01306	-0.00347	0.068	0.0728	0.10772	0.13105	-0.2713	0.2713	0.68506	0.8654	0.96412	0.37686	0.344	0.16079	0.08755	0.00033	0.00033	turquoise
CNOT1	-0.01329	-0.0952	0.01209	0.0014	-0.11198	0.15107	0.08904	0.05583	-0.05583	0.86303	0.21552	0.87529	0.98549	0.14479	0.04856	0.24681	0.46829	0.46829	turquoise
FANCD2	-0.01439	0.04774	0.03472	-0.08619	-0.10033	0.08493	0.23417	-0.30095	0.30095	0.85183	0.53525	0.6521	0.26232	0.19169	0.2694	0.00205	6.34E-05	6.34E-05	pink
USP20	0.00119	-0.10155	-0.21179	-0.13546	0.05618	-0.15598	-0.14877	0.29972	-0.29972	0.98766	0.18628	0.00542	0.0773	0.46551	0.04163	0.05215	6.82E-05	6.82E-05	brown
TSPYL4	-0.03196	-0.20323	-0.15916	-0.07955	0.01719	-0.20611	-0.1386	0.46023	-0.46023	0.67818	0.00768	0.03759	0.30101	0.82343	0.00684	0.07063	2.40E-10	2.40E-10	brown
GALE	0.04135	0.05572	0.12224	-0.02221	0.06773	0.27971	0.19209	-0.54407	0.54407	0.59132	0.46919	0.11121	0.77305	0.37878	0.00021	0.01184	1.46E-14	1.46E-14	yellow
AP4S1	-0.00716	-0.1309	-0.13316	-0.01433	-0.01619	-0.06224	-0.16452	0.34122	-0.34122	0.92597	0.08791	0.08253	0.85243	0.83358	0.4187	0.03153	4.94E-06	4.94E-06	turquoise
TYW3	-0.05926	-0.12266	-0.08493	-0.03269	-0.06028	0.00379	-0.14526	0.29499	-0.29499	0.44133	0.10999	0.2694	0.67126	0.43354	0.96072	0.05801	8.97E-05	8.97E-05	turquoise
GMIP	-0.00196	0.03088	0.13636	0.04549	-0.04507	0.21825	0.14307	-0.06299	0.06299	0.97966	0.68846	0.07535	0.55468	0.55828	0.00413	0.06194	0.4131	0.4131	blue
EPCAM	0.11562	-0.04134	-0.01524	-0.04403	0.17274	0.03852	-0.01203	-0.46112	0.46112	0.13209	0.59133	0.84316	0.56742	0.02387	0.61689	0.88407	2.20E-10	2.20E-10	yellow
SNAP23	-0.07984	-0.09051	0.10249	0.0729	-0.16565	0.28152	0.12614	-0.0423	0.0423	0.29923	0.23907	0.18222	0.34335	0.03036	0.00019	0.10019	0.58274	0.58274	turquoise
CFAP157	-0.05981	-0.12113	-0.22317	-0.12965	0.15171	-0.20117	-0.11281	0.03316	-0.03316	0.43715	0.11453	0.00335	0.091	0.04762	0.00833	0.14181	0.66678	0.66678	grey
NUDT13	-0.00113	-0.11254	-0.01453	-0.01207	0.02727	-0.02545	0.09649	-0.2433	0.2433	0.98834	0.14277	0.85034	0.87545	0.72334	0.74113	0.20931	0.00134	0.00134	grey
ANAPC7	0.04074	-0.02099	-0.02612	-0.05384	0.15046	-0.16382	0.01128	-0.07616	0.07616	0.59676	0.78522	0.73456	0.48432	0.0495	0.03227	0.8836	0.32215	0.32215	turquoise
NDUFC2	-0.00289	-0.00978	-0.15053	-0.1264	0.078	-0.23278	0.00581	-0.05911	0.05911	0.97011	0.89893	0.04938	0.09948	0.31057	0.00218	0.93985	0.44249	0.44249	grey
ADRA2B	-0.13043	-0.07034	-0.14627	-0.01982	-0.04854	-0.04646	0.14202	0.07871	-0.07871	0.08905	0.36064	0.05627	0.79697	0.52837	0.54624	0.0639	0.30614	0.30614	grey
LGALSL	-0.04216	-0.15307	-0.12115	-0.05607	0.05945	-0.17203	-0.12298	0.1654	-0.1654	0.58405	0.04563	0.11447	0.46638	0.43986	0.02446	0.10906	0.03062	0.03062	grey
DDX10	0.01256	-0.02607	-0.00312	-0.02862	-0.14743	0.03636	0.1861	-0.08086	0.08086	0.87046	0.73505	0.96773	0.71018	0.05432	0.63682	0.01481	0.29312	0.29312	turquoise
CACNA2D	0.05223	0.12492	0.06123	0.13563	-0.03733	0.17303	0.14437	0.0247	-0.0247	0.49748	0.10354	0.42628	0.07693	0.6279	0.02363	0.05958	0.74847	0.74847	grey
PRRT3	-0.03283	-0.14558	-0.2951	-0.21628	0.1011	-0.30903	-0.19771	0.33449	-0.33449	0.66988	0.05744	8.92E-05	0.00449	0.18828	3.91E-05	0.00954	7.77E-06	7.77E-06	brown
ZBTB18	0.04472	-0.02636	-0.02658	-0.03493	0.00304	0.12127	0.03629	-0.08757	0.08757	0.56139	0.7322	0.73001	0.65012	0.96856	0.11411	0.63744	0.25473	0.25473	turquoise
SMURF2	-0.06033	-0.22679	0.04739	0.02486	-0.16393	0.08267	0.0319	0.08771	-0.08771	0.43312	0.00286	0.5382	0.74688	0.03216	0.28239	0.67878	0.25399	0.25399	turquoise
EBLN2	-0.02325	-0.09405	0.03603	0.01056	-0.06402	0.14161	0.1143	-0.02067	0.02067	0.76273	0.22111	0.63991	0.89101	0.40549	0.06467	0.13661	0.78848	0.78848	red
PRPF18	-0.00352	-0.02885	0.03839	0.02648	-0.08391	0.08147	0.10442	-0.00149	0.00149	0.96359	0.70793	0.61808	0.73096	0.27524	0.28948	0.17411	0.98459	0.98459	turquoise
ECT2	-0.03065	0.0236	0.05009	0.02679	-0.10396	0.16887	0.25362	-0.32534	0.32534	0.69067	0.75935	0.51527	0.72798	0.176	0.02725	0.00082	1.41E-05	1.41E-05	pink
TRIM35	-0.12084	-0.04024	-0.0336	-0.00272	-0.08914	-0.00048	0.01539	0.22786	-0.22786	0.11541	0.60128	0.66265	0.97185	0.24627	0.99498	0.84164	0.00272	0.00272	turquoise
UST	-0.10047	0.03033	0.1749	0.08799	-0.20347	0.31017	0.07601	0.08846	-0.08846	0.19103	0.69371	0.02214	0.25244	0.0076	3.65E-05	0.32313	0.24995	0.24995	grey
PWWP2B	0.13076	0.00312	-0.06451	-0.05035	0.12756	-0.07024	0.16246	-0.40504	0.40504	0.08824	0.96765	0.40187	0.51314	0.09637	0.36133	0.03375	3.91E-08	3.91E-08	green
KCNC4	-0.09367	-0.24057	-0.05717	-0.04963	0.17522	-0.07946	-0.15552	0.01065	-0.01065	0.22299	0.00153	0.45768	0.51916	0.02189	0.30159	0.04224	0.89009	0.89009	grey
RAB3D	0.0163	0.06359	0.06245	0.09397	0.02948	0.03276	-0.06095	-0.12288	0.12288	0.83237	0.40866	0.41709	0.22151	0.70194	0.67053	0.42844	0.10935	0.10935	grey
CFAP46	0.04394	0.11333	-0.0484	-0.08131	0.30538	-0.09943	-0.02523	-0.13447	0.13447	0.56827	0.13998	0.52962	0.29043	4.88E-05	0.19571	0.74323	0.07951	0.07951	grey
ALB	0.04373	-0.05201	0.04686	0.12727	-0.08452	0.04393	-0.10002	0.00035	-0.00035	0.5701	0.4993	0.5428	0.09714	0.27174	0.56832	0.19305	0.99638	0.99638	magenta
CTNS	-0.0876	-0.10667	0.00318	0.05257	-0.05004	-0.08634	0.08767	0.40547	-0.40547	0.25459	0.16496	0.96711	0.4947	0.51568	0.26152	0.25418	3.77E-08	3.77E-08	grey
MPP2	-0.06925	-0.17069	-0.25804	-0.11435	0.09138	-0.33007	-0.21678	0.48321	-0.48321	0.36811	0.02561	0.00066	0.13643	0.23457	1.04E-05	0.0044	2.17E-11	2.17E-11	brown
GLT1D1	-0.12851	-0.13492	-0.12776	-0.03374	-0.02032	-0.13552	-0.11971	0.39815	-0.39815	0.09392	0.0785	0.09587	0.66127	0.79192	0.07717	0.11886	6.93E-08	6.93E-08	brown
HDC	-0.05611	-0.1335	-0.00265	0.00567	-0.05318	-0.00414	-0.06403	0.44395	-0.44395	0.46602	0.08173	0.9726	0.94128	0.48967	0.95713	0.40537	1.19E-09	1.19E-09	blue
IBSP	-0.06398	-0.03827	0.05535	0.05195	-0.13329	0.10475	0.1043	-0.09374	0.09374	0.40574	0.61922	0.47214	0.49983	0.08221	0.17272	0.17457	0.22264	0.22264	grey
LYSMD4	0.01662	-0.02411	-0.02746	-0.12643	0.18	0.02092	0.00146	-0.27548	0.27548	0.82915	0.7543	0.72146	0.0994	0.01848	0.78594	0.98488	0.00027	0.00027	grey
ANKLE2	0.0009	-0.17545	0.03434	0.04345	0.06097	-0.0243	0.02176	-0.13036	0.13036	0.99068	0.02172	0.65566	0.57253	0.42829	0.75244	0.77759	0.08923	0.08923	turquoise
SFPQ	-0.0065	-0.11349	-0.07437	-0.04881	0.0349	0.00721	-0.02364	0.10999	-0.10999	0.93272	0.13941	0.33371	0.5261	0.65038	0.92548	0.7589	0.15211	0.15211	turquoise
RIC8A	-0.00962	-0.16323	-0.05567	-0.0366	-0.08739	-0.04332	0.08271	-0.02393	0.02393	0.90066	0.03291	0.46952	0.63459	0.2557	0.57372	0.28217	0.75604	0.75604	turquoise
KLRF1	-0.04238	0.07711	0.07527	0.10043	-0.06863	0.03484	-0.02618	0.15182	-0.15182	0.58203	0.31612	0.32788	0.19121	0.37244	0.65097	0.73395	0.04746	0.04746	grey
CD72	-0.06063	-0.03894	0.08111	0.05476	-0.18874	0.05165	0.06166	0.27684	-0.27684	0.43083	0.61309	0.29162	0.47687	0.01343	0.50232	0.42308	0.00025	0.00025	blue
HSD17B6	-0.00743	-0.00716	0.07621	-0.00657	-0.17207	0.17163	0.10322	0.12018	-0.12018	0.92312	0.92594	0.32183	0.93206	0.02442	0.0248	0.1791	0.11743	0.11743	black
VKORC1L1	0.0344	-0.16544	0.04963	0.01245	-0.0434	0.0194	0.0388	0.00111	-0.00111	0.65509	0.03058	0.51919	0.87158	0.57303	0.80115	0.61441	0.98847	0.98847	turquoise
TINAGL1	0.04655	-0.11948	0.11175	0.0967	0.00243	0.23708	0.21585	-0.44482	0.44482	0.54549	0.11959	0.14561	0.20833	0.97484	0.0018	0.00458	1.09E-09	1.09E-09	yellow
NCR3	-0.10056	0.04516	0.06888	0.00189	-0.10189	0.08749	-0.0679	0.31696	-0.31696	0.19065	0.55756	0.37068	0.98047	0.18481	0.25518	0.37757	2.40E-05	2.40E-05	blue
ARHGAP4	-0.0236	-0.01597	-0.09098	-0.08391	0.04832	-0.05316	-0.00024	-0.18599	0.18599	0.7593	0.83572	0.23665	0.2752	0.53024	0.48988	0.99756	0.01487	0.01487	grey
PFKFB2	-0.04534	-0.05345	-0.08918	-0.04761	0.06655	0.05203	0.09394	0.05446	-0.05446	0.556	0.48752	0.24607	0.53629	0.38711	0.49913	0.22165	0.47929	0.47929	grey
PCNX1	-0.03818	-0.14845	-0.04082	0.06018	-0.13412	-0.04181	-0.04382	0.41109	-0.41109	0.62003	0.05265	0.59605	0.43429	0.0803	0.58712	0.56929	2.33E-08	2.33E-08	turquoise
BRD9	0.02938	-0.0183	0.01193	-0.00967	-0.02551	0.00382	0.13402	-0.18084	0.18084	0.70288	0.81278	0.87696	0.90012	0.74046	0.96043	0.08053	0.01793	0.01793	grey
PTPRD	0.08083	-0.18241	0.0287	0.10141	-0.11544	-0.11524	-0.08413	0.53964	-0.53964	0.29325	0.01694	0.70938	0.18692	0.13272	0.13339	0.27398	2.62E-14	2.62E-14	blue
SCAI	-0.04356	-0.15127	-0.1281	-0.03628	-0.0215	-0.09537	-0.05804	0.33965	-0.33965	0.57156	0.04826	0.09497	0.63758	0.78017	0.21467	0.45086	5.50E-06	5.50E-06	turquoise
TMEM65	-0.00779	-0.2153	-0.06091	0.02147	-0.00634	-0.13867	-0.07613	0.16044	-0.16044	0.91948	0.00468	0.42874	0.78049	0.93443	0.07048	0.32233	0.03606	0.03606	turquoise
CEACAM2	-0.12022	-0.03623	0.05251	0.04539	-0.17781	0.08421	0.03979	0.3739	-0.3739	0.11731	0.63802	0.49518	0.55552	0.01998	0.27351	0.60535	4.72E-07	4.72E-07	blue
SLC66A1L	0.04817	0.12594	-0.1418	-0.0345	0.02344	0.00214	0.12247	-0.09576	0.09576	0.53152	0.10073	0.06431	0.65417	0.76092	0.97787	0.11055	0.21281	0.21281	grey
CMTR1	0.01343	-0.06292	-0.02742	0.04358	0.06392	-0.0478	-0.06615	0.20484	-0.20484	0.86161	0.41359	0.72185	0.57146	0.40622	0.53469	0.39	0.0072	0.0072	turquoise
TNNC2	0.1614	-0.0259	0.07304	0.07256	0.05923	0.08168	0.05334	-0.3274	0.3274	0.03495	0.73664	0.3424	0.34562	0.44156	0.28823	0.48834	1.24E-05	1.24E-05	grey
DENND4A	-0.07606	-0.17076	-0.09931	-0.02907	-0.12794	0.01241	-0.10841	0.39363	-0.39363	0.32279	0.02555	0.19625	0.70584	0.09538	0.87205	0.15814	1.00E-07	1.00E-07	turquoise
GNL3	-0.04482	0.00134	0.07915	-0.02608	-0.09187	0.17719	0.14228	-0.15203	0.15203	0.56052	0.98614	0.30344	0.73487	0.23205	0.02042	0.0634	0.04714	0.04714	turquoise
CCDC148	0.05248	-0.13753	-0.00173	0.0425	-0.00428	0.01587	-0.04283	-0.00073	0.00073	0.49542	0.07284	0.98204	0.58103	0.95571	0.83683	0.57807	0.99242	0.99242	grey
AGPAT3	-0.06441	-0.08001	-0.09002	-0.00236	0.06892	-0.00676	0.04634	0.02472	-0.02472	0.40259	0.29825	0.24164	0.9756	0.37044	0.93009	0.54726	0.74824	0.74824	turquoise
TFDP1	-0.06522	-0.01036	-0.0161	-0.00535	-0.02934	-0.05465	0.05309	0.0227	-0.0227	0.3967	0.89304	0.83448	0.9446	0.70328	0.47772	0.4904	0.76826	0.76826	turquoise
RAB1A	-0.03014	-0.10425	0.00917	0.02874	-0.0358	0.07255	0.03213	-0.01789	0.01789	0.69555	0.17479	0.90529	0.70903	0.64203	0.34567	0.67655	0.81639	0.81639	turquoise
ZBTB44	-0.06471	-0.12346	-0.02558	0.03969	-0.17764	0.10609	0.08093	0.13125	-0.13125	0.40043	0.10766	0.73986	0.60628	0.0201	0.16729	0.29268	0.08704	0.08704	turquoise
PECAM1	-0.12106	-0.09322	-0.0017	0.07277	-0.15285	0.07765	-0.10705	0.51943	-0.51943	0.11475	0.22522	0.98242	0.3442	0.04595	0.31272	0.16342	3.35E-13	3.35E-13	blue
ATXN7	-0.03083	-0.11386	0.02513	0.03821	-0.16084	0.18144	-0.0296	0.2501	-0.2501	0.68896	0.13811	0.7442	0.61978	0.0356	0.01755	0.7007	0.00097	0.00097	turquoise
SEZ6	-0.01919	-0.12597	-0.26105	-0.18793	0.14523	-0.35444	-0.24851	0.34873	-0.34873	0.80326	0.10063	0.00056	0.01384	0.05805	1.97E-06	0.00105	2.95E-06	2.95E-06	brown
STYX	-0.01717	-0.04479	0.03795	0.09933	-0.16705	0.13341	0.0874	0.10916	-0.10916	0.82359	0.56079	0.62212	0.19614	0.02898	0.08194	0.25565	0.15526	0.15526	turquoise
MGMT	-0.02582	0.03063	0.00429	0.06204	-0.07438	0.00891	0.23121	-0.24086	0.24086	0.73744	0.69089	0.95561	0.42021	0.3336	0.90796	0.00235	0.00151	0.00151	grey
ISLR	-0.08626	-0.08387	0.03536	0.0803	-0.14529	0.1762	0.06088	0.14039	-0.14039	0.26195	0.27542	0.64612	0.29648	0.05796	0.02115	0.42897	0.06704	0.06704	black
CYHR1	0.08035	-0.03045	-0.15762	-0.0772	0.13663	-0.20366	0.07915	-0.28329	0.28329	0.29614	0.69257	0.0395	0.31557	0.07477	0.00755	0.30347	0.00017	0.00017	green
MYLK	-0.10948	-0.0627	0.04162	0.06899	-0.16916	0.11894	0.03527	0.33494	-0.33494	0.15403	0.41522	0.58889	0.36989	0.02698	0.12128	0.64696	7.54E-06	7.54E-06	blue
STX16	0.02757	-0.13699	-0.07166	-0.0584	0.14331	-0.15617	-0.09112	-0.08617	0.08617	0.72041	0.074	0.35161	0.44803	0.06149	0.04137	0.23592	0.26247	0.26247	red
METTL26	0.07802	0.06894	-0.07834	-0.09395	0.09241	-0.08768	0.09388	-0.42813	0.42813	0.31046	0.37024	0.30847	0.22162	0.2293	0.25416	0.22195	5.18E-09	5.18E-09	green
PNPLA4	-0.51764	-0.11526	-0.14929	0.01297	0.04531	-0.12394	-0.04617	0.05571	-0.05571	4.17E-13	0.13331	0.05131	0.86625	0.55621	0.1063	0.54876	0.46925	0.46925	grey
SRRT	0.05998	-0.0861	-0.05549	-0.09343	0.16376	-0.07447	0.07926	-0.19355	0.19355	0.43581	0.26281	0.47104	0.22419	0.03234	0.33303	0.30279	0.0112	0.0112	red
CENPC	-0.10437	-0.09118	-0.08776	-0.09582	-0.04356	0.02572	-0.10824	0.33702	-0.33702	0.1743	0.23562	0.25368	0.21253	0.5716	0.73844	0.15879	6.56E-06	6.56E-06	turquoise
WRAP73	-0.01682	-0.01669	0.03056	0.03284	0.06765	0.0727	0.11201	-0.24707	0.24707	0.82719	0.82846	0.69156	0.66979	0.37928	0.34465	0.14468	0.00112	0.00112	green
SRP68	-0.02913	-0.20904	0.02635	-0.03334	-0.02348	-0.02798	0.02248	-0.00583	0.00583	0.70524	0.00607	0.73228	0.66505	0.76052	0.71636	0.77043	0.93965	0.93965	turquoise
IL17RC	0.10162	-0.07432	-0.15119	-0.16569	0.14713	-0.08087	0.0686	-0.21659	0.21659	0.18601	0.33404	0.04838	0.03033	0.05482	0.29306	0.37262	0.00444	0.00444	grey
GTF3C1	-0.06127	-0.13224	-0.13368	-0.01777	-0.07715	-0.0961	0.02606	0.2673	-0.2673	0.42596	0.08469	0.08132	0.81755	0.31587	0.21118	0.73508	0.00041	0.00041	turquoise
CLIP2	-0.01978	-0.13706	0.00106	0.0109	0.14304	-0.05497	-0.05126	-0.02368	0.02368	0.79739	0.07383	0.98905	0.88744	0.06198	0.47516	0.50548	0.7585	0.7585	grey
PLAAT2	0.04424	0.0358	0.09138	0.06916	0.00296	0.14486	0.19523	-0.36593	0.36593	0.56562	0.64207	0.23459	0.36874	0.96935	0.05871	0.0105	8.57E-07	8.57E-07	yellow
CBY1	-0.07143	-0.01352	-0.12652	0.02335	0.02698	-0.0889	-0.09398	-0.02254	0.02254	0.35323	0.86068	0.09915	0.76176	0.72616	0.24755	0.22148	0.7698	0.7698	grey
LRMP	-0.06615	-0.01463	0.07109	0.08258	-0.07714	0.15661	-0.00748	0.22726	-0.22726	0.39	0.8494	0.35552	0.28291	0.31593	0.0408	0.92267	0.0028	0.0028	blue
MAP3K14	0.00664	-0.18975	-0.01119	0.06684	0.01788	0.07445	-0.07713	0.27763	-0.27763	0.93129	0.01293	0.88449	0.38504	0.81644	0.33319	0.31602	0.00024	0.00024	blue
DEPDC5	-0.0752	-0.05184	-0.13031	-0.07181	0.02674	-0.13409	-0.10907	0.28286	-0.28286	0.3283	0.50074	0.08936	0.35064	0.72847	0.08037	0.1556	0.00018	0.00018	turquoise
P2RY6	-0.11137	-0.02057	0.01995	0.06759	-0.22285	0.09864	0.18838	0.10277	-0.10277	0.14699	0.7894	0.79563	0.37971	0.00339	0.19929	0.01361	0.18105	0.18105	blue
ZNF235	-0.04853	-0.19614	-0.09211	0.00512	-0.02922	0.0016	-0.08246	0.35472	-0.35472	0.52852	0.01014	0.23082	0.94697	0.70443	0.98339	0.28365	1.93E-06	1.93E-06	turquoise
ITCH	0.00384	-0.13671	0.04579	0.07118	-0.09924	0.06348	0.06983	0.08441	-0.08441	0.96022	0.07459	0.55206	0.35491	0.19656	0.40942	0.3641	0.27236	0.27236	turquoise
CENPT	0.01121	-0.04376	-0.01493	-0.02996	0.16683	-0.04925	0.0368	-0.14219	0.14219	0.8843	0.56979	0.84627	0.69726	0.02919	0.52241	0.63276	0.06357	0.06357	red
AGAP6	-0.00034	-0.06521	-0.0423	-0.02832	0.22883	-0.15411	-0.0744	-0.08187	0.08187	0.99643	0.39681	0.58275	0.71306	0.00261	0.04416	0.33351	0.28707	0.28707	red
CNNM1	0.05982	-0.0526	-0.10274	-0.03642	0.12606	-0.16478	-0.04285	-0.09564	0.09564	0.43704	0.49442	0.18117	0.63626	0.10039	0.03126	0.57792	0.21337	0.21337	grey
PBX2	-0.02764	-0.0735	-0.04165	-0.00602	0.02579	-0.06111	0.02395	0.12953	-0.12953	0.71975	0.33941	0.58857	0.93774	0.73776	0.42716	0.75581	0.09132	0.09132	turquoise
KANK3	-0.13628	-0.04643	-0.10521	0.06562	0.0354	-0.06986	-0.0933	0.41087	-0.41087	0.07551	0.54652	0.17084	0.39384	0.64573	0.36391	0.22482	2.38E-08	2.38E-08	blue
PPIL3	0.01359	-0.12582	-0.00359	-0.08957	0.05467	0.05581	0.08995	-0.03939	0.03939	0.86	0.10106	0.96282	0.24402	0.47756	0.46842	0.242	0.60899	0.60899	turquoise
CHD6	-0.01336	-0.1806	-0.08463	-0.0059	-0.03084	-0.10196	-0.16145	0.32901	-0.32901	0.86233	0.01809	0.27112	0.93893	0.68885	0.18451	0.03489	1.11E-05	1.11E-05	turquoise
CREG2	0.11195	-0.00233	0.03526	0.07959	-0.02774	0.04333	0.1298	-0.36132	0.36132	0.14491	0.97582	0.64704	0.3008	0.71874	0.57359	0.09062	1.20E-06	1.20E-06	grey
TRAF1	-0.13212	-0.1515	-0.03629	-0.01291	-0.11933	0.11219	-0.10232	0.33233	-0.33233	0.08496	0.04792	0.63747	0.86688	0.12004	0.14403	0.18295	8.96E-06	8.96E-06	blue
SEC14L4	-0.18517	-0.03243	-0.03126	0.11848	0.02106	0.07485	0.05449	-0.09996	0.09996	0.01532	0.67371	0.68483	0.12274	0.78451	0.3306	0.47901	0.19331	0.19331	grey
BTBD19	-0.08651	-0.22311	-0.0141	0.05625	-0.05472	-0.02652	0.03642	0.19031	-0.19031	0.26054	0.00336	0.85474	0.46491	0.4772	0.73062	0.63628	0.01266	0.01266	black
SOX6	0.00342	-0.20672	-0.07945	0.05426	-0.05396	0.03363	-0.03068	0.34348	-0.34348	0.96459	0.00667	0.30159	0.48088	0.48336	0.66235	0.69035	4.24E-06	4.24E-06	turquoise
SMIM22	0.14231	0.06457	0.0398	-0.02799	0.18124	0.03333	0.07256	-0.60228	0.60228	0.06335	0.40146	0.60529	0.71633	0.01768	0.6652	0.3456	2.94E-18	2.94E-18	yellow
SPTLC1	-0.06862	-0.15554	0.01845	0.07258	-0.17587	0.10684	0.0261	0.03438	-0.03438	0.37249	0.04221	0.81074	0.34548	0.0214	0.16427	0.73476	0.65533	0.65533	turquoise
TTC17	0.00813	-0.18806	-0.04227	-0.02481	-0.00395	0.0068	-0.06883	0.10733	-0.10733	0.91597	0.01377	0.58307	0.74742	0.95906	0.92969	0.37105	0.16231	0.16231	turquoise
ATR	-0.07755	-0.06541	-0.06237	-0.00633	-0.07371	0.0947	0.10494	0.00046	-0.00046	0.31339	0.39533	0.41771	0.93456	0.33803	0.21791	0.17193	0.99526	0.99526	turquoise
AHCY	0.06466	0.1434	0.01171	-0.02218	0.06407	0.02544	0.15832	-0.32206	0.32206	0.40081	0.06132	0.87921	0.77334	0.40509	0.7412	0.03863	1.74E-05	1.74E-05	grey
PLCL1	-0.10386	-0.10276	-0.01405	0.05886	-0.23435	0.01122	-0.13608	0.63889	-0.63889	0.17641	0.18109	0.8553	0.44448	0.00204	0.8842	0.07595	5.35E-21	5.35E-21	blue
CLK3	0.03016	-0.24495	-0.09875	-0.08125	0.20918	-0.11061	-0.1061	-0.09771	0.09771	0.69532	0.00124	0.19879	0.29078	0.00604	0.14979	0.16725	0.20358	0.20358	red
ZMYND19	0.00168	-0.02372	-0.07028	-0.06258	-0.02875	-0.04881	0.17649	-0.32965	0.32965	0.98262	0.75813	0.36104	0.41613	0.70891	0.52612	0.02094	1.07E-05	1.07E-05	grey

MEST	0.03119	-0.09281	0.0319	-0.02661	-0.09546	0.11777	0.06945	0.12839	-0.12839	0.68552	0.22731	0.67876	0.7297	0.21425	0.12501	0.36672	0.09423	0.09423	grey
SATB2	0.00331	-0.12738	-0.00203	0.05564	-0.04666	0.07905	0.0965	-0.11711	0.11711	0.96577	0.09686	0.97896	0.46983	0.54447	0.3041	0.20929	0.12714	0.12714	grey
CHERP	0.01297	-0.09674	-0.0939	0.00497	0.15189	-0.06658	-0.04942	-0.02779	0.02779	0.86627	0.20813	0.22186	0.94859	0.04734	0.38689	0.52096	0.71823	0.71823	grey
ATP6AP1	0.00813	-0.21874	-0.16881	-0.11496	0.02456	-0.26055	-0.12711	0.16212	-0.16212	0.91596	0.00405	0.0273	0.13435	0.7498	0.00058	0.09758	0.03414	0.03414	brown
ARHGEF9	-0.04976	-0.15592	-0.1106	-0.09495	0.07612	-0.20956	-0.16493	0.38644	-0.38644	0.51811	0.04171	0.14983	0.21671	0.32239	0.00594	0.03111	1.78E-07	1.78E-07	brown
UBE2G2	-0.08596	-0.10994	-0.12053	-0.02765	0.0924	-0.07025	-0.04602	0.23835	-0.23835	0.26361	0.15232	0.11634	0.71957	0.22936	0.36122	0.55005	0.00169	0.00169	turquoise
MED27	-0.04461	-0.08693	-0.16177	-0.09193	-0.07381	-0.2219	-0.02983	0.12256	-0.12256	0.56238	0.25821	0.03453	0.23174	0.33736	0.00354	0.69854	0.11029	0.11029	grey
BNIP3	-0.08372	-0.12562	-0.12387	0.05991	-0.01092	-0.22358	-0.17211	0.31041	-0.31041	0.27628	0.10159	0.1065	0.43631	0.88731	0.00329	0.02439	3.60E-05	3.60E-05	grey
EPS15L1	0.08774	-0.13814	-0.03654	-0.03866	0.01663	-0.00685	-0.03246	0.02861	-0.02861	0.2538	0.07158	0.63514	0.61564	0.82909	0.92911	0.67337	0.71031	0.71031	turquoise
ZNF324	-0.01507	-0.12087	-0.2155	-0.09649	0.03189	-0.14835	-0.14273	0.23392	-0.23392	0.84489	0.11532	0.00465	0.20933	0.67883	0.05281	0.06256	0.00208	0.00208	turquoise
CCDC102/	-0.06095	-0.03785	0.01602	0.13747	-0.14574	0.14339	0.16978	0.07338	-0.07338	0.42842	0.62304	0.83528	0.07298	0.05717	0.06134	0.02642	0.34018	0.34018	black
TFB2M	-0.04085	0.01837	-0.09811	-0.1315	0.02578	-0.00065	0.13277	-0.06084	0.06084	0.59573	0.81153	0.20172	0.08646	0.73782	0.99331	0.08344	0.42924	0.42924	turquoise
PODXL	-0.03389	-0.03555	0.05849	0.11907	-0.23114	0.09845	0.06999	0.06836	-0.06836	0.65988	0.64433	0.44735	0.12086	0.00235	0.20016	0.36298	0.37431	0.37431	turquoise
ATP6V0E2	-0.004	-0.07866	-0.29027	-0.16683	0.11141	-0.36985	-0.22831	0.29697	-0.29697	0.95863	0.30648	0.00012	0.02919	0.14686	6.40E-07	0.00267	8.01E-05	8.01E-05	brown
WDR13	0.0161	-0.08943	-0.22371	-0.12806	0.16889	-0.20687	0.01995	-0.27512	0.27512	0.83444	0.24475	0.00327	0.09509	0.02723	0.00663	0.79567	0.00027	0.00027	green
IL2RB	-0.10592	-0.01926	0.07293	0.10278	-0.18248	0.11554	0.05147	0.37211	-0.37211	0.16795	0.80256	0.34317	0.18101	0.0169	0.13237	0.50374	5.40E-07	5.40E-07	blue
SRPK3	-0.00531	0.0636	0.05823	-0.04129	0.1004	-0.06217	0.10033	-0.20686	0.20686	0.94509	0.40856	0.44933	0.59177	0.19134	0.41917	0.19166	0.00664	0.00664	grey
PLA2G5	-0.07764	-0.04407	0.10144	0.05121	-0.13101	0.04158	-0.01111	0.36895	-0.36895	0.31279	0.56709	0.18676	0.50596	0.08765	0.58921	0.88537	6.85E-07	6.85E-07	blue
JADE3	-0.10172	-0.12502	-0.03318	0.08168	-0.17763	0.06564	0.00331	0.13031	-0.13031	0.18555	0.10325	0.66655	0.28823	0.02011	0.39364	0.96573	0.08937	0.08937	turquoise
DAD1	0.01203	-0.04213	-0.00055	-0.00058	-0.03521	0.00472	0.07865	-0.22996	0.22996	0.8759	0.5843	0.9943	0.99398	0.6475	0.95109	0.30651	0.00248	0.00248	turquoise
MFAP5	-0.10846	0.06209	0.10823	0.11717	-0.19168	0.18497	0.06873	0.28789	-0.28789	0.15792	0.41983	0.1588	0.12695	0.01202	0.01544	0.37171	0.00013	0.00013	black
MED13	-0.06102	-0.2172	-0.07505	-0.01581	-0.08257	0.00554	-0.00857	0.2458	-0.2458	0.42789	0.00432	0.32927	0.83738	0.28299	0.94266	0.91141	0.00119	0.00119	turquoise
MAGOHB	-0.02801	-0.02869	-0.0466	-0.05453	0.00468	-0.10816	0.15138	-0.1603	0.1603	0.71611	0.70949	0.54505	0.47872	0.95151	0.15911	0.04811	0.03623	0.03623	grey
STK36	0.00248	-0.10669	-0.06784	0.02554	0.18151	-0.00418	0.01092	-0.15426	0.15426	0.97436	0.16486	0.378	0.74018	0.0175	0.95675	0.88726	0.04396	0.04396	red
TSSC4	0.04779	0.00538	-0.22326	-0.13593	0.15867	-0.17287	-0.02401	-0.13148	0.13148	0.53482	0.94433	0.00333	0.07628	0.0382	0.02376	0.75523	0.08649	0.08649	green
MTMR8	-0.04637	-0.06755	0.10837	-0.05575	-0.09682	0.20132	-0.01874	0.02514	-0.02514	0.54702	0.38001	0.15826	0.46892	0.20776	0.00828	0.80777	0.74411	0.74411	turquoise
DNAJB4	-0.04661	-0.22589	0.05966	0.11419	-0.11841	0.11183	-0.07688	0.40078	-0.40078	0.54491	0.00297	0.43826	0.13699	0.12295	0.14534	0.31757	5.58E-08	5.58E-08	blue
ZNF671	-0.09574	-0.1423	-0.17312	-0.00385	-0.00751	-0.13266	-0.19652	0.49272	-0.49272	0.21287	0.06337	0.02355	0.96012	0.92234	0.0837	0.00999	7.61E-12	7.61E-12	brown
HOXC4	0.01292	0.04685	0.0849	0.13762	0.02834	0.13056	0.17625	-0.18207	0.18207	0.8668	0.5429	0.26954	0.07266	0.71294	0.08874	0.02112	0.01715	0.01715	grey
CYB5R1	-0.0196	-0.10165	-0.04062	-0.00011	-0.0387	-0.15988	0.05297	0.07609	-0.07609	0.79912	0.18584	0.59788	0.99888	0.61528	0.03672	0.49138	0.32258	0.32258	grey
NCOA6	0.04247	-0.1695	-0.02366	0.02669	-0.03831	0.02629	0.01265	-0.01326	0.01326	0.58127	0.02668	0.75875	0.72895	0.61889	0.73291	0.8696	0.86336	0.86336	turquoise
DUOX2	-0.01863	-0.00748	0.09543	0.0468	0.09048	0.18645	0.07929	-0.1933	0.1933	0.80886	0.92261	0.21439	0.54327	0.23921	0.01462	0.3026	0.0113	0.0113	grey
APTX	0.07076	-0.09581	-0.20663	-0.06938	-0.02686	-0.1467	0.01253	-0.05553	0.05553	0.35773	0.21256	0.0067	0.36723	0.72729	0.05553	0.87079	0.4707	0.4707	grey
ZSWIM3	#######	-0.18571	-0.08914	0.04191	0.0994	-0.15203	-0.06537	0.03363	-0.03363	0.99963	0.01502	0.24626	0.58623	0.19584	0.04714	0.39559	0.66238	0.66238	grey
KDELR1	0.1043	-0.01699	-0.09425	-0.05093	0.09264	-0.06887	0.07913	-0.34122	0.34122	0.17459	0.82542	0.22016	0.50826	0.22814	0.37078	0.30361	4.95E-06	4.95E-06	grey
PRKCE	0.02414	-0.21931	-0.12367	-0.04024	-0.00659	-0.19046	-0.16382	0.42686	-0.42686	0.75401	0.00395	0.10705	0.60132	0.93187	0.01259	0.03227	5.81E-09	5.81E-09	turquoise
PAMR1	-0.0129	-0.06198	-0.03258	0.05296	-0.17564	0.17693	0.06875	0.23328	-0.23328	0.867	0.4206	0.6723	0.4915	0.02157	0.02061	0.37159	0.00214	0.00214	blue
IDE	-0.00868	-0.01863	0.04217	0.04784	-0.11355	0.09185	0.13101	0.01487	-0.01487	0.91026	0.80889	0.58392	0.53438	0.13919	0.23218	0.08764	0.84698	0.84698	turquoise
C11orf49	0.09688	-0.14433	-0.12354	-0.0993	0.14625	-0.12644	-0.09081	-0.29359	0.29359	0.20746	0.05965	0.10742	0.19631	0.0563	0.09936	0.23754	9.73E-05	9.73E-05	grey
FAP	-0.06766	-0.06735	0.09948	0.14108	-0.25382	0.13792	0.10489	0.2902	-0.2902	0.37927	0.38143	0.19546	0.06569	0.00081	0.07203	0.17214	0.00012	0.00012	black
NUP155	-0.0631	-0.05515	-0.04177	-0.02859	-0.13169	-0.04691	0.17059	0.04337	-0.04337	0.41228	0.47369	0.58749	0.71049	0.08599	0.54232	0.0257	0.57327	0.57327	turquoise
PRDM16	-0.13942	-0.0402	-0.02011	0.06054	0.05357	0.09168	-0.01954	-0.00401	0.00401	0.06895	0.60161	0.79401	0.43152	0.48652	0.23301	0.79977	0.95848	0.95848	grey
GTF2F1	0.06669	-0.07857	-0.18219	-0.07183	0.0799	-0.19901	-0.03147	-0.00149	0.00149	0.38611	0.30701	0.01708	0.35049	0.29887	0.00907	0.68286	0.98459	0.98459	green
TMEM208	0.02784	0.01594	-0.16707	-0.13585	0.15381	-0.19455	0.07496	-0.41117	0.41117	0.71773	0.83609	0.02896	0.07646	0.04459	0.01078	0.32982	2.32E-08	2.32E-08	grey
OGFR	0.0898	-0.026	-0.10813	-0.01578	0.12688	-0.09846	0.10655	-0.34388	0.34388	0.2428	0.73574	0.15921	0.83774	0.09819	0.20011	0.16541	4.12E-06	4.12E-06	green
TANK	-0.05321	-0.08203	0.02161	0.05553	-0.04509	0.10437	0.01586	-0.0375	0.0375	0.48948	0.28617	0.77907	0.47067	0.55815	0.1743	0.83688	0.62628	0.62628	turquoise
TARS2	0.04429	0.08627	-0.05717	-0.13549	0.02808	0.01162	0.13936	-0.23206	0.23206	0.56516	0.26188	0.45762	0.07723	0.71547	0.88011	0.06909	0.00226	0.00226	greenyellow
RELA	0.01436	-0.10468	0.06524	0.04887	-0.14335	0.23802	0.19975	-0.27172	0.27172	0.85213	0.173	0.39654	0.5256	0.06143	0.00172	0.00881	0.00032	0.00032	turquoise
BANP	0.05721	-0.14713	-0.0955	-0.00235	0.17962	-0.096	-0.01703	0.04698	-0.04698	0.45732	0.05482	0.21405	0.97561	0.01874	0.21163	0.82507	0.54176	0.54176	grey
DDHD2	-0.03755	-0.09818	-0.01746	0.01157	-0.09957	-0.03201	-0.02761	0.29512	-0.29512	0.62584	0.2014	0.82072	0.88062	0.19507	0.67765	0.71997	8.91E-05	8.91E-05	turquoise
TMEM117	0.11067	-0.1657	-0.03678	0.01771	-0.02228	-0.03921	-0.06302	0.17728	-0.17728	0.14957	0.03032	0.6329	0.81817	0.77236	0.61061	0.41286	0.02036	0.02036	turquoise
PLXNA2	-0.04803	-0.07617	-0.00597	-0.08261	0.07149	0.0456	-0.11971	-0.03385	0.03385	0.53273	0.3221	0.93827	0.28272	0.35276	0.55371	0.11887	0.66026	0.66026	grey
SLC1A1	0.04371	-0.09669	0.04359	0.03035	0.02203	0.20273	0.03171	-0.07768	0.07768	0.57028	0.20837	0.57136	0.69356	0.77487	0.00783	0.68052	0.31258	0.31258	grey
YAE1	-0.02838	-0.00764	-0.09402	-0.09825	0.14134	-0.10003	-0.07814	-0.09594	0.09594	0.7125	0.92096	0.22126	0.20108	0.06519	0.193	0.30969	0.21195	0.21195	grey
POLR3A	-0.00177	-0.03847	-0.06072	-0.046	-0.04183	-0.14568	0.03337	0.09943	-0.09943	0.98171	0.61739	0.43017	0.55021	0.58702	0.05728	0.66483	0.19571	0.19571	turquoise
AREL1	-0.03516	-0.09179	-0.02851	0.01862	-0.05427	-0.04059	-0.00235	0.09022	-0.09022	0.64797	0.23248	0.71127	0.80904	0.48082	0.59814	0.97563	0.2406	0.2406	turquoise
SLC12A8	0.02012	0.01251	0.09886	0.06331	-0.14006	0.18635	0.15645	0.18953	-0.18953	0.7939	0.87102	0.19827	0.41071	0.06769	0.01467	0.04101	0.01303	0.01303	black
NR1D2	-0.07831	-0.06711	-0.0189	-0.09654	-0.19877	-0.00072	-0.02558	0.20494	-0.20494	0.30867	0.38313	0.8062	0.20906	0.00915	0.99254	0.73976	0.00717	0.00717	turquoise
SAA4	-0.00868	-0.12537	0.0057	-0.07027	-0.07008	0.01716	0.04642	-0.14946	0.14946	0.91031	0.10229	0.94104	0.36106	0.36236	0.82375	0.54656	0.05104	0.05104	grey
C4A	-0.0094	-0.13273	-0.01046	0.14788	-0.12118	0.17468	-0.00672	0.184	-0.184	0.90288	0.08352	0.89195	0.05358	0.11438	0.02231	0.93053	0.01599	0.01599	grey
MS4A14	0.01337	-0.05257	0.11925	0.06505	-0.17342	0.07539	0.01895	0.32631	-0.32631	0.86219	0.4947	0.12032	0.39798	0.02331	0.32707	0.80571	1.33E-05	1.33E-05	blue
SCX	-0.04405	0.07204	-0.08483	-0.02511	0.2221	-0.1042	0.03068	-0.23937	0.23937	0.56731	0.34912	0.26994	0.74445	0.00351	0.17501	0.69037	0.00162	0.00162	green
DBF4B	-0.0064	-0.16888	-0.17295	-0.05501	0.16702	-0.24331	-0.08314	0.01994	-0.01994	0.93382	0.02723	0.02369	0.47486	0.02901	0.00134	0.27966	0.79571	0.79571	grey
DYRK1A	-0.04458	-0.16274	-0.09676	-0.00893	0.0387	0.01126	-0.09265	0.25227	-0.25227	0.56259	0.03345	0.20804	0.90767	0.61524	0.8838	0.22812	0.00087	0.00087	turquoise
JUND	0.0871	-0.10781	-0.08471	-0.08015	0.16534	0.01572	-0.03753	-0.08094	0.08094	0.25728	0.16045	0.27063	0.29734	0.03068	0.83831	0.62598	0.29261	0.29261	green
KCND1	-0.03038	-0.25031	-0.10688	0.01248	0.0498	-0.14856	-0.14624	0.38533	-0.38533	0.69324	0.00096	0.16409	0.86722	0.51772	0.05248	0.05632	1.95E-07	1.95E-07	turquoise
GEMIN7	0.00806	-0.05983	-0.09959	-0.06125	0.16294	-0.10173	0.06818	-0.29284	0.29284	0.91669	0.43695	0.19496	0.42615	0.03323	0.18549	0.37559	0.0001	0.0001	grey
HSPA1B	0.21011	-0.1103	0.07613	-0.00291	0.06338	0.12286	0.04086	-0.30885	0.30885	0.00581	0.15094	0.32235	0.96984	0.41019	0.10941	0.5957	3.96E-05	3.96E-05	grey
CILP2	0.01819	-0.00558	0.06022	0.1034	-0.09962	0.16592	0.06793	0.31789	-0.31789	0.81329	0.94229	0.43398	0.17835	0.19486	0.03009	0.37732	2.27E-05	2.27E-05	black
SPRED3	-0.00895	-0.23896	-0.16845	-0.15095	-0.04582	-0.22515	-0.13	0.19473	-0.19473	0.90751	0.00165	0.02764	0.04875	0.55175	0.00307	0.09014	0.0107	0.0107	grey
GLE1	0.01161	-0.06809	-0.11164	-0.0661	-0.16079	-0.03015	0.08362	0.11003	-0.11003	0.88021	0.37621	0.14601	0.39035	0.03565	0.69547	0.27687	0.15196	0.15196	turquoise
TMEM171	0.1322	0.10992	0.05682	-0.01858	0.07081	0.19839	0.21056	-0.40194	0.40194	0.08478	0.15239	0.46042	0.80943	0.3574	0.00929	0.00571	5.06E-08	5.06E-08	yellow
PNO1	-0.03969	0.0023	-0.0329	0.00337	-0.16616	0.10847	0.13912	0.05629	-0.05629	0.60626	0.9762	0.66924	0.9651	0.02986	0.15791	0.06956	0.46463	0.46463	turquoise
GUCY1A2	-0.06377	-0.15489	-0.07016	0.09916	-0.19043	0.02573	-0.03657	0.49774	-0.49774	0.40728	0.0431	0.36181	0.19694	0.0126	0.73834	0.63488	4.32E-12	4.32E-12	blue
PNPLA8	-0.05564	-0.24335	0.02529	0.03214	-0.05215	0.04525	-0.11753	0.19673	-0.19673	0.46977	0.00134	0.74262	0.6765	0.49816	0.55678	0.12578	0.00991	0.00991	turquoise
STX4	0.06729	0.02777	0.04341	0.09851	-0.03353	0.16032	0.18453	-0.31137	0.31137	0.38183	0.71842	0.57288	0.19988	0.66334	0.0362	0.01568	3.39E-05	3.39E-05	yellow
CEP112	-0.07958	-0.2493	-0.14101	0.07165	-0.09526	-0.14023	-0.14517	0.52208	-0.52208	0.30081	0.00101	0.06582	0.3517	0.2152	0.06734	0.05817	2.43E-13	2.43E-13	turquoise
MKRN2OS	0.00563	0.01135	0.07867	-0.02525	0.03336	0.24462	0.15111	-0.53089	0.53089	0.94173	0.88291	0.30641	0.74303	0.6649	0.00126	0.04851	8.07E-14	8.07E-14	yellow
HCK	-0.13831	0.03566	0.06451	0.04193	-0.18772	0.15987	0.06535	0.36891	-0.36891	0.07123	0.64333	0.40189	0.58607	0.01395	0.03673	0.3958	6.87E-07	6.87E-07	blue
TEAD3	-0.02203	-0.05356	0.0864	0.08773	-0.07317	0.222	0.21436	-0.21614	0.21614	0.7749	0.48656	0.26119	0.25385	0.34158	0.00352	0.00487	0.00452	0.00452	black
CTSL	-0.08847	-0.08617	-0.03666	0.06899	-0.18713	-0.09208	-0.01158	0.31567	-0.31567	0.24986	0.26245	0.63402	0.3699	0.01426	0.23097	0.88048	2.60E-05	2.60E-05	blue
STEAP3	-0.01706	0.12679	0.04799	0.04853	-0.21771	0.19064	0.24622	-0.29986	0.29986	0.82476	0.09843	0.53306	0.52845	0.00423	0.0125	0.00117	6.76E-05	6.76E-05	yellow
NALCN	0.04784	-0.16051	-0.06771	-0.03056	-0.1347	-0.06584	-0.12784	0.47508	-0.47508	0.53436	0.03598	0.37886	0.69151	0.07899	0.39223	0.09565	5.19E-11	5.19E-11	turquoise
MLH3	0.07069	-0.11304	-0.08284	0.02449	0.05616	0.05443	0.04647	-0.05744	0.05744	0.35819	0.141	0.2814	0.75054	0.46565	0.47953	0.54613	0.45555	0.45555	turquoise
RNF19A	-0.09812	-0.19605	0.02627	0.1018	-0.11282	0.12492	-0.05994	0.14563	-0.14563	0.20167	0.01017	0.73302	0.18523	0.14177	0.10353	0.43615	0.05736	0.05736	turquoise
ILKAP	0.09186	-0.01138	-0.14401	-0.07435	0.16694	-0.10418	0.0762	-0.19532	0.19532	0.23211	0.88251	0.06023	0.3338	0.02908	0.17508	0.32187	0.01046	0.01046	red
EMID1	0.01169	-0.15418	-0.03134	0.02831	-0.02377	-0.02773	-0.08436	0.32047	-0.32047	0.87942	0.04407	0.68404	0.71322	0.75762	0.71886	0.27264	1.93E-05	1.93E-05	grey
FUT3	0.09649	0.0682	0.10675	0.0562	0.03233	0.22567	0.15511	-0.49099	0.49099	0.2093	0.37542	0.16465	0.46535	0.6746	0.003	0.04279	9.24E-12	9.24E-12	yellow
PTTG1IP	0.01782	-0.11033	0.02713	0.03875	-0.05323	0.09612	0.17128	-0.21254	0.21254	0.81707	0.15083	0.72465	0.61486	0.48924	0.21106	0.02509	0.00526	0.00526	turquoise
LACC1	-0.06897	-0.08426	0.00121	-0.01806	-0.13868	0.02336	0.06514	0.28613	-0.28613	0.37009	0.27319	0.98748	0.81459	0.07046	0.76173	0.3973	0.00015	0.00015	turquoise
RTN4R	-0.04233	0.07903	0.01984	0.04683	0.09713	0.12123	0.14034	-0.40904	0.40904	0.58252	0.30417	0.79678	0.543	0.20631	0.11422	0.06714	2.78E-08	2.78E-08	yellow
ZNF487	0.01554	-0.18735	-0.04065	-0.06499	-0.01969	-0.04321	-0.05345	0.01995	-0.01995	0.84012	0.01414	0.59761	0.39838	0.79823	0.57472	0.48745	0.79567	0.79567	grey
PAG1	-0.08651	-0.23555	0.06264	0.06476	-0.19348	0.17085	-0.00902	0.19194	-0.19194	0.26057	0.00193	0.41571	0.40006	0.01123	0.02547	0.90676	0.0119	0.0119	turquoise
TCF20	0.023	0.00047	-0.02446	0.01052	-0.05918	0.02742	0.06798	0.00222	-0.00222	0.76526	0.99518	0.75084	0.89135	0.44194	0.72182	0.37702	0.97704	0.97704	turquoise
CNPY3	0.07841	0.04927	-0.05619	0.05414	-0.05794	-0.07836	-0.00766	-0.01818	0.01818	0.30801	0.52216	0.46537	0.48183	0.45161	0.30834	0.92081	0.81346	0.81346	grey
SHMT1	-0.14417	0.02631	0.00461	0.08892	-0.07334	0.09829	0.10012	-0.04225	0.04225	0.05993	0.73266	0.95226	0.24744	0.34043	0.20089	0.1926	0.58326	0.58326	grey
FASTKD2	-0.03868	0.03185	0.04242	0.06031	-0.11096	0.15121	0.14956	-0.02859	0.02859	0.61549	0.67918	0.58172	0.4333	0.1485	0.04836	0.05088	0.71049	0.71049	turquoise
SH3BGRL2	-0.00746	-0.02415	0.03122	-0.0039	0.0972	0.17465	0.0089	-0.07448	0.07448	0.92281	0.75386	0.68517	0.95963	0.20596	0.02234	0.90797	0.33295	0.33295	turquoise
MYH10	-0.09021	-0.15372	-0.04316	0.02272	-0.08946	-0.08819	-0.08642	0.58234	-0.58234	0.24066	0.04471	0.57513	0.76803	0.24458	0.2514	0.26104	6.57E-17	6.57E-17	blue
PLVAP	-0.07247	-0.17187	-0.0858	0.05784	-0.09002	-0.04138	-0.19007	0.50435	-0.50435	0.34619	0.02459	0.26448	0.45237	0.24163	0.59101	0.01277	2.02E-12	2.02E-12	blue
ABCA8	-0.06615	-0.10026	-0.04816	0.06508	-0.13074	0.05178	-0.10381	0.63424	-0.63424	0.39002	0.19196	0.53167	0.39773	0.08829	0.50123	0.17662	1.25E-20	1.25E-20	blue
RAPGEFL1	0.0559	0.04296	0.06139	0.0377	0.20762	0.06605	0.12528	-0.40006	0.40006	0.46775	0.57693	0.42506	0.62448	0.00643	0.39068	0.10253	5.92E-08	5.92E-08	yellow
SRSF3	0.00482	-0.00874	0.01387	0.00871	-0.01302	0.10275	0.11313	-0.06673	0.06673	0.95015	0.90963	0.85707	0.91002	0.86576	0.18113	0.14067	0.38585	0.38585	turquoise
C3AR1	-0.10498	0.01697	0.05613	0.05542	-0.24139	0.16126	0.10415	0.41394	-0.41394	0.17177	0.82566	0.46591	0.47153	0.00147	0.03511	0.17521	1.83E-08	1.83E-08	blue
ZNF548	-0.04411	-0.19125	-0.07888	0.05396	-0.10285	0.01402	-0.06544	0.2783	-0.2783	0.56671	0.01222	0.30513	0.48336	0.1807	0.85557	0.39508	0.00023	0.00023	turquoise
NEU1	-0.01315	-0.16321	-0.11036	-0.019	0.02459	-0.18765	-0.08672	0.17169	-0.17169	0.86446	0.03294	0.15072	0.80517	0.74957	0.01398	0.25941	0.02474	0.02474	grey
ZNF462	0.03564	-0.18649	-0.05691	0.05556	0.01385	-0.05836	-0.04071	0.19145	-0.19145	0.64351	0.0146	0.45971	0.47045	0.85734	0.44835	0.59703	0.01213	0.01213	turquoise
MTO1	-0.13124	-0.05972	-0.06007	-0.01549	-0.04339	-0.00532	-0.05769	0.34836	-0.34836	0.08707	0.43781	0.43512	0.84058	0.57311	0.94493	0.45353	3.03E-06	3.03E-06	turquoise
ZNF207	-0.00773	-0.09632	-0.02423	-0.04608	0.01381	0.05774	0.06791	-0.09646	0.09646	0.92012	0.21014	0.75311	0.54952	0.85772	0.45319	0.37749	0.20948	0.20948	turquoise
HGF	-0.06045	-0.06462	-0.02599	0.02223	-0.18114	0.15573	0.008	0.56198	-0.56198	0.4322	0.40109	0.73578	0.77291	0.01774	0.04196	0.91729	1.27E-15	1.27E-15	blue
RIPK1	-0.10963	-0.06208	0.01	-0.00297	-0.04607	0.08076	0.01216	0.24147	-0.24147	0.15346	0.4199	0.8967	0.96921	0.54959	0.29372	0.8746	0.00146	0.00146	turquoise
MRPL44	0.01623	0.03488	-0.04252	0.02321	-0.02506	0.05578	0.06914	-0.16876	0.16876	0.83308	0.65057	0.58081	0.76315	0.74492	0.46866	0.36888	0.02735	0.02735	turquoise
PEX12	-0.17256	-0.14027	-0.14994	-0.00782	0.02978	-0.11538	-0.09602	0.32739	-0.32739	0.02401	0.06727	0.05029	0.91918	0.69898	0.1329	0.21154	1.24E-05	1.24E-05	turquoise
TMEM150	0.04027	-0.07919	-0.06244	-0.02165	-0.00276	0.01886	0.06276	0.00161	-0.00161	0.60102	0.30321	0.41717	0.77864	0.97146	0.80654	0.41478	0.98338	0.98338	grey
NUDT5	0.03711	-0.00018	-0.03711	-0.10007	-0.01446	-0.02925	0.15188	-0.27086	0.27086	0.62992	0.99816	0.62992	0.19281	0.85114	0.70411	0.04737	0.00034	0.00034	turquoise
H2AFY2	-0.05168	-0.08921	-0.24376	-0.09686	0.03721	-0.07753	-0.13821	0.08937	-0.08937	0.50203	0.24592	0.00131	0.20757	0.62901	0.31352	0.07143	0.24508	0.24508	grey
PCNX3	0.02753	-0.02144	-0.08649	-0.04759	-0.00621	-0.03165	0.14891	-0.12858	0.12858	0.72079	0.78073	0.26064	0.53647	0.93578	0.68114	0.05192	0.09374	0.09374	grey
MAPK1	-0.08748	-0.11208	-0.03122	0.05504	-0.10618	0.02123	-0.04377	0.2461	-0.2461	0.25525	0.14443	0.68517	0.47462	0.1669	0.78285	0.56971	0.00118	0.00118	turquoise
MED15	-0.02522	-0.0506	-0.02821	0.01351	0.02011	-0.00904	0.09302	-0.22751	0.22751	0.74333	0.51102	0.71419	0.86081	0.79409	0.90656	0.22626	0.00277	0.00277	grey
PGM5	-0.0246	-0.0237	-0.03603	0.09242	0.01941	0.04363	-0.11272	0.4494	-0.4494	0.74945	0.75832	0.63986	0.22926	0.80109	0.571	0.14215	7.02E-10	7.02E-10	salmon
ATP8B4	-0.08523	-0.05748	0.03353	0.04302	-0.21579	0.13007	0.05142	0.554	-0.554	0.26771	0.45518	0.66328	0.57636	0.00459	0.08997	0.50419	3.84E-15	3.84E-15	blue
MAP2K5	0.03166	-0.05733	-0.16823	-0.00847	-0.00814	-0.08654	0.00641	0.17148	-0.17148	0.68103	0.45643	0.02784	0.91247	0.91583	0.26038	0.93371	0.02492	0.02492	grey
CPT1B	0.03677	-0.01315	-0.04869	-0.04791	0.28902	-0.1073	-0.06782	-0.2163	0.2163	0.63304	0.8645	0.52711	0.53379	0.00013	0.16245	0.37811	0.00449	0.00449	red
SLC16A5	-0.00548	-0.08292	0.08272	0.09447	-0.01777	0.22585	0.27878	-0.382	0.382	0.94333	0.28094	0.28208	0.21908	0.81758	0.00298	0.00022	2.53E-07	2.53E-07	yellow
IRX5	0.01574	-0.06551	0.05763	0.04744	-0.09099	0.11207	0.23232	-0.46438	0.46438	0.83808	0.39464	0.45406	0.53779	0.23659	0.14446	0.00223	1.58E-10	1.58E-10	grey
ALG14	-0.12901	0.01696	-0.15794	-0.06002	0.16751	-0.23751	-0.21468	0.22888	-0.22888	0.09264	0.82576	0.0391	0.43549	0.02853	0.00176	0.00481	0.0026	0.0026	grey
HIGD2A	0.1014	0.03113	-0.12397	-0.09294	0.17719	-0.12957	-0.02856	-0.25468	0.25468	0.18696	0.6861	0.10622	0.22664	0.02042	0.09121	0.71077	0.00078	0.00078	green
PCDHB13	-0.00878	-0.21354	0.03145	-0.01151	-0.06914	0.10826	-0.03774	0.14968	-0.14968	0.90926	0.00504	0.683	0.88123	0.36885	0.15871	0.62411	0.05071	0.05071	turquoise
MLYCD	-0.02504	-0.06961	-0.22036	-0.08278	0.14386	-0.17481	-0.19813	0.22213	-0.22213	0.74513	0.36561	0.00378	0.28173	0.06049	0.02221	0.00939	0.0035	0.0035	grey

EPHA10	0.05128	-0.04424	-0.05974	-0.07419	0.29634	-0.07202	-0.16157	-0.15421	0.15421	0.50532	0.56556	0.43766	0.33488	8.30E-05	0.34922	0.03475	0.04403	0.04403	grey
SLC29A1	0.03116	-0.08106	0.00388	0.09927	-0.04082	0.06266	-0.0345	0.17094	-0.17094	0.68576	0.29192	0.95984	0.19642	0.59602	0.41558	0.6542	0.02539	0.02539	grey
RPS25	0.04694	0.10585	-0.02092	-0.07614	-0.10001	0.07294	0.1357	-0.1815	0.1815	0.54213	0.16822	0.78598	0.32225	0.19308	0.34307	0.07677	0.01751	0.01751	purple
REXO1	-0.02558	-0.02362	-0.08582	-0.05806	0.1681	-0.04871	-0.07253	-0.1152	0.1152	0.73982	0.75913	0.26441	0.45069	0.02796	0.52698	0.3458	0.1335	0.1335	green
PCF11	-0.04098	-0.16089	-0.12707	-0.0518	0.02963	-0.05991	-0.0901	0.22153	-0.22153	0.59464	0.03554	0.09769	0.50106	0.70041	0.43633	0.24123	0.00359	0.00359	red
NHLRC1	0.04451	0.03567	-0.05053	0.00674	0.12635	-0.11378	0.07563	0.06433	-0.06433	0.56325	0.64322	0.51164	0.93027	0.09962	0.1384	0.3255	0.4032	0.4032	grey
RPS6KC1	-0.07513	-0.09604	-0.04037	-0.02751	-0.15785	0.06977	0.10524	0.21145	-0.21145	0.32876	0.21145	0.60014	0.72095	0.03922	0.3645	0.17072	0.0055	0.0055	turquoise
ATP6V0C	-0.0077	-0.05403	-0.07634	-0.02975	0.17477	-0.04539	-0.03815	-0.12756	0.12756	0.92038	0.48275	0.32098	0.69929	0.02224	0.55548	0.62032	0.0964	0.0964	green
GEM	-0.02618	-0.17072	0.06105	0.08844	-0.14087	0.20092	-0.02577	0.36865	-0.36865	0.73397	0.02558	0.42762	0.25005	0.0661	0.00841	0.7379	7.01E-07	7.01E-07	blue
MICA	0.02344	-0.02202	0.08571	0.02926	-0.07371	0.28351	0.20848	-0.30425	0.30425	0.76091	0.77497	0.26503	0.70404	0.338	0.00017	0.00621	5.22E-05	5.22E-05	yellow
ROBO4	-0.14403	-0.10384	-0.08835	#######	-0.05212	-0.13239	-0.14759	0.54213	-0.54213	0.06018	0.1765	0.2505	0.99992	0.49841	0.08432	0.05406	1.89E-14	1.89E-14	blue
OLA1	0.03385	0.07561	0.01321	0.02935	-0.08309	0.05906	0.10485	-0.21662	0.21662	0.66029	0.32566	0.8638	0.70313	0.27995	0.4429	0.1723	0.00443	0.00443	turquoise
HFE	-0.05101	-0.03483	0.01953	0.03523	-0.10688	0.25096	0.16709	-0.04261	0.04261	0.5076	0.65112	0.79981	0.64734	0.16413	0.00093	0.02893	0.58006	0.58006	turquoise
CDA	0.0986	-0.03319	0.01835	0.09561	-0.00963	0.02754	0.12738	-0.27023	0.27023	0.1995	0.66646	0.81167	0.2135	0.90054	0.72065	0.09686	0.00035	0.00035	grey
STOML1	0.02552	-0.00223	-0.12918	-0.02112	0.10419	-0.09178	-0.01875	-0.17128	0.17128	0.74039	0.97692	0.09219	0.7839	0.17505	0.23253	0.80772	0.02509	0.02509	grey
GPATCH2L	-0.02319	-0.21023	-0.04404	0.0265	-0.01407	0.00252	-0.0318	0.25079	-0.25079	0.76339	0.00578	0.56738	0.73082	0.85508	0.97394	0.67971	0.00094	0.00094	turquoise
IGFL1	-0.00696	0.06425	-0.00084	0.04699	-0.01248	0.01151	0.04683	-0.10654	0.10654	0.92798	0.40375	0.99132	0.54164	0.8713	0.88119	0.54301	0.16545	0.16545	grey
C12orf43	0.05632	-0.05873	0.01268	-0.00721	0.03988	-0.02107	0.01365	0.00452	-0.00452	0.46435	0.44542	0.86926	0.92543	0.60454	0.78448	0.85936	0.95321	0.95321	turquoise
SRBD1	-0.07949	-0.08058	-0.06571	0.00463	-0.08251	-0.0454	-0.04114	0.41292	-0.41292	0.3014	0.29477	0.39316	0.95209	0.28332	0.55547	0.59315	1.99E-08	1.99E-08	turquoise
FAM169A	-0.03806	-0.16599	-0.20463	-0.09904	0.00225	-0.23936	-0.10455	0.39905	-0.39905	0.62112	0.03003	0.00726	0.19749	0.97675	0.00162	0.17355	6.44E-08	6.44E-08	brown
HMGCS1	-0.06904	-0.00821	0.02065	-0.03461	-0.01286	0.19395	0.04979	-0.13781	0.13781	0.36958	0.91516	0.78865	0.6531	0.86746	0.01103	0.51778	0.07227	0.07227	turquoise
VAV2	-0.08459	-0.09136	-0.04895	0.05485	-0.14297	-0.06628	0.06876	-0.01894	0.01894	0.27133	0.23465	0.52488	0.47617	0.06211	0.38907	0.37154	0.80582	0.80582	grey
ACER3	0.0785	-0.09652	0.02597	0.01206	-0.0838	0.06729	0.14169	0.05504	-0.05504	0.30745	0.20917	0.73596	0.87561	0.27584	0.38186	0.06451	0.47462	0.47462	turquoise
DDX17	-0.01904	-0.13122	-0.09908	-0.007	0.06037	-0.02482	-0.06525	0.15379	-0.15379	0.80476	0.08714	0.19728	0.92762	0.43282	0.74727	0.39651	0.04462	0.04462	red
MPV17L	-0.02093	-0.09912	-0.15212	-0.05242	-0.0248	-0.07998	-0.07789	0.16845	-0.16845	0.7858	0.19713	0.04702	0.49593	0.74746	0.29838	0.31123	0.02763	0.02763	grey
SMPDL3B	-0.04644	-0.01463	0.15236	0.11351	-0.08924	0.18514	0.16574	-0.25799	0.25799	0.5464	0.84939	0.04666	0.13935	0.24574	0.01534	0.03028	0.00066	0.00066	grey
ALG2	-0.07206	-0.15176	-0.2037	-0.00183	0.031	-0.22162	-0.15389	0.20416	-0.20416	0.34897	0.04754	0.00753	0.98107	0.6873	0.00358	0.04448	0.0074	0.0074	turquoise
MTFMT	-0.02349	-0.06884	-0.1546	-0.10422	0.02927	-0.11183	0.08222	-0.01443	0.01443	0.76037	0.371	0.04348	0.17493	0.70393	0.14532	0.285	0.85138	0.85138	turquoise
MEX3C	-0.08439	-0.16317	-0.03311	0.07386	-0.0686	0.05997	-0.00484	0.27665	-0.27665	0.27244	0.03298	0.66731	0.337	0.37266	0.43591	0.94992	0.00025	0.00025	turquoise
TPD52L1	0.02626	0.11067	-0.04806	0.09598	-0.03829	-0.03112	0.04019	-0.09223	0.09223	0.73313	0.14959	0.5325	0.21174	0.61899	0.68618	0.60178	0.23023	0.23023	grey
M6PR	-0.0736	-0.14785	0.03945	0.07997	-0.14451	0.06652	-0.00982	0.20873	-0.20873	0.33873	0.05363	0.60847	0.29847	0.05933	0.38736	0.89853	0.00615	0.00615	turquoise
GOPC	-0.0346	-0.14125	0.01282	0.03711	-0.11806	-0.01911	0.00963	0.30958	-0.30958	0.65321	0.06536	0.86781	0.62988	0.12408	0.80403	0.90053	3.78E-05	3.78E-05	turquoise
MTMR1	-0.12338	-0.19363	0.01178	-0.00712	-0.12453	0.09456	0.07325	-0.00285	0.00285	0.10789	0.01116	0.87849	0.92641	0.10462	0.21861	0.34101	0.97054	0.97054	turquoise
TPI1	0.06415	-0.07795	0.01191	-0.01097	0.03644	-0.04923	0.10137	-0.40651	0.40651	0.40448	0.31086	0.87716	0.88674	0.63612	0.52254	0.18707	3.45E-08	3.45E-08	pink
TRMT1L	-0.06061	-0.13498	-0.04243	-0.02449	-0.02482	0.01491	0.0544	0.12319	-0.12319	0.43099	0.07838	0.58161	0.7505	0.7473	0.84654	0.47975	0.10846	0.10846	turquoise
SRMS	0.0018	0.02525	0.09949	0.10742	0.11792	0.16719	0.1147	-0.3844	0.3844	0.98137	0.74305	0.19543	0.16196	0.12454	0.02884	0.13523	2.10E-07	2.10E-07	yellow
DUT	-0.11555	-0.02486	-0.19795	-0.15031	0.04467	-0.15075	-0.01636	-0.078	0.078	0.13235	0.7469	0.00945	0.04972	0.5618	0.04906	0.83183	0.31058	0.31058	grey
PMS2	-0.08062	-0.12264	-0.03897	0.0042	-0.09388	0.0584	0.07668	0.12354	-0.12354	0.29454	0.11003	0.6128	0.95651	0.22195	0.44801	0.31887	0.10744	0.10744	turquoise
ARSB	-0.07254	-0.09793	0.06111	0.13116	-0.20261	0.06326	-0.01857	0.51923	-0.51923	0.34576	0.20255	0.42721	0.08728	0.00787	0.41106	0.80947	3.44E-13	3.44E-13	blue
KLHDC8A	-0.01047	-0.1659	-0.25952	-0.10558	-0.03428	-0.23721	-0.12014	0.3525	-0.3525	0.89187	0.03011	0.00061	0.16933	0.65622	0.00178	0.11755	2.26E-06	2.26E-06	brown
ZNF516	-0.02382	-0.23206	-0.19246	-0.03503	0.02836	-0.24182	-0.14064	0.38411	-0.38411	0.7571	0.00226	0.01167	0.64917	0.71273	0.00144	0.06655	2.14E-07	2.14E-07	turquoise
ARHGEF19	0.03805	-0.02664	0.10917	0.1065	-0.13153	0.16585	0.22723	-0.2442	0.2442	0.62126	0.72943	0.15522	0.16563	0.08639	0.03016	0.0028	0.00129	0.00129	grey
ERC1	0.03629	-0.06683	-0.04996	0.04028	-0.10413	-0.03081	-0.09125	0.34174	-0.34174	0.63748	0.38513	0.51636	0.60089	0.17529	0.68912	0.23524	4.77E-06	4.77E-06	turquoise
TSPOAP1	-0.01818	-0.1082	-0.19836	-0.07878	0.18149	-0.25257	-0.21116	0.23538	-0.23538	0.81341	0.15894	0.0093	0.30575	0.01752	0.00086	0.00556	0.00194	0.00194	brown
DPH6	-0.01949	-0.00092	-0.08305	-0.04417	-0.07178	0.05674	0.0968	-0.18357	0.18357	0.80022	0.99051	0.2802	0.56618	0.35087	0.46102	0.20783	0.01625	0.01625	turquoise
CLIP1	-0.01599	-0.16589	0.02245	0.05447	-0.09397	0.03959	0.00649	0.22928	-0.22928	0.83553	0.03013	0.77072	0.47916	0.2215	0.60722	0.93282	0.00256	0.00256	turquoise
TAF13	-0.09473	-0.17238	0.02384	0.04596	-0.02201	0.02675	-0.01617	0.23506	-0.23506	0.2178	0.02416	0.75693	0.55055	0.77512	0.72836	0.8337	0.00197	0.00197	turquoise
PIGU	0.04908	-0.05716	-0.13782	-0.03166	0.15466	-0.18175	0.03713	-0.18471	0.18471	0.52381	0.45702	0.07225	0.68106	0.04341	0.01736	0.62974	0.01558	0.01558	grey
DHRS3	0.06191	-0.11841	0.1565	0.12974	-0.10221	0.30278	0.21156	-0.39475	0.39475	0.42114	0.12294	0.04095	0.09078	0.18342	5.69E-05	0.00547	9.16E-08	9.16E-08	yellow
ACTG2	-0.13502	-0.00065	0.04834	0.01991	0.02255	0.08689	0.10507	0.0725	-0.0725	0.07828	0.99324	0.53012	0.79607	0.76974	0.25843	0.17143	0.34599	0.34599	salmon
ZNF823	0.0221	-0.09837	-0.03184	0.05115	0.14604	0.03293	-0.06392	-0.1846	0.1846	0.77419	0.20055	0.67935	0.50639	0.05666	0.66899	0.40625	0.01565	0.01565	grey
ZNF658	-0.19412	-0.10928	-0.15671	-0.06902	0.00268	-0.18981	-0.15914	0.40983	-0.40983	0.01096	0.1548	0.04067	0.36969	0.9722	0.0129	0.03761	2.60E-08	2.60E-08	turquoise
COLGALT2	0.0079	0.10815	0.03909	-0.06395	0.00255	0.20415	0.03065	-0.02889	0.02889	0.91832	0.15914	0.61169	0.40599	0.97364	0.0074	0.69064	0.70764	0.70764	grey
CERS5	0.06626	-0.23243	-0.12548	-0.02878	0.0115	-0.19859	-0.11198	0.057	-0.057	0.38919	0.00222	0.10198	0.70867	0.88132	0.00922	0.1448	0.459	0.459	turquoise
HCFC1R1	0.01632	-0.01966	-0.03196	0.0134	-0.04003	0.04735	0.10253	-0.16604	0.16604	0.83222	0.79859	0.67814	0.86186	0.60322	0.5386	0.18207	0.02998	0.02998	black
ITGB7	-0.0957	0.01485	0.07959	0.06283	-0.10439	0.13526	0.09739	0.1692	-0.1692	0.21309	0.84716	0.30075	0.41426	0.17422	0.07775	0.20507	0.02694	0.02694	blue
ABCD4	0.01455	-0.08775	-0.06296	0.02791	0.16939	-0.03064	-0.00971	-0.12164	0.12164	0.85022	0.25375	0.41329	0.71708	0.02677	0.69077	0.89969	0.113	0.113	red
RAB38	-0.04658	-0.1156	0.07184	0.05051	-0.17708	0.18701	0.23667	-0.21927	0.21927	0.54519	0.13216	0.35044	0.51177	0.0205	0.01432	0.00183	0.00396	0.00396	grey
PIAS2	-0.07994	-0.18648	-0.20064	-0.03443	0.09827	-0.18273	-0.14561	0.38204	-0.38204	0.29864	0.0146	0.00851	0.65482	0.20098	0.01675	0.0574	2.52E-07	2.52E-07	turquoise
PAPPA2	0.01903	-0.08086	-0.07054	-0.0881	-0.03603	-0.226	-0.17068	0.42019	-0.42019	0.80492	0.29309	0.35923	0.25188	0.63992	0.00296	0.02562	1.06E-08	1.06E-08	brown
WASF2	-0.05769	-0.13913	0.00943	0.04464	-0.1984	0.14681	0.0263	0.22797	-0.22797	0.45354	0.06954	0.90253	0.56206	0.00929	0.05535	0.73277	0.00271	0.00271	turquoise
RANBP3	-0.00841	-0.05019	-0.22048	-0.13328	0.14439	-0.23804	-0.12923	0.08712	-0.08712	0.91305	0.51449	0.00376	0.08224	0.05954	0.00172	0.09206	0.25718	0.25718	green
CH25H	-0.12575	-0.10009	-0.02227	-0.04358	-0.00034	0.05236	-0.0597	0.47626	-0.47626	0.10125	0.19273	0.77245	0.57141	0.99652	0.49642	0.43793	4.58E-11	4.58E-11	blue
TRIM33	-0.08074	-0.12948	0.02105	0.03945	-0.06379	0.10134	-0.0258	0.22511	-0.22511	0.29383	0.09143	0.78467	0.60842	0.40713	0.1872	0.7377	0.00307	0.00307	turquoise
PLEKHJ1	0.12454	0.09698	-0.02229	-0.04304	0.21319	0.00602	0.09389	-0.41535	0.41535	0.10459	0.207	0.77227	0.57616	0.00512	0.93768	0.22192	1.62E-08	1.62E-08	green
PPDPF	0.17547	0.03544	-0.0447	-0.06752	0.17735	0.01137	0.12661	-0.52893	0.52893	0.0217	0.64539	0.56155	0.38021	0.02031	0.88266	0.09892	1.03E-13	1.03E-13	green
CAPN13	-0.02579	-0.0528	-0.06062	0.00021	-0.00274	0.0222	-0.01229	-0.0461	0.0461	0.73774	0.49282	0.43095	0.99777	0.97166	0.77319	0.87329	0.54938	0.54938	grey
SUGP1	0.09766	-0.01763	-0.19423	-0.13647	0.16755	-0.19675	-0.0099	-0.09039	0.09039	0.2038	0.81902	0.01091	0.07511	0.02849	0.0099	0.89773	0.2397	0.2397	green
PNMA3	0.00197	-0.06411	-0.20159	-0.05426	0.08883	-0.226	-0.22121	0.36096	-0.36096	0.9796	0.40484	0.00819	0.48091	0.24796	0.00296	0.00364	1.23E-06	1.23E-06	brown
XPC	-0.05454	-0.12834	-0.08969	-0.07729	-0.09155	0.0379	0.00406	0.32481	-0.32481	0.47863	0.09434	0.24336	0.315	0.23371	0.62262	0.95799	1.46E-05	1.46E-05	turquoise
SEC61G	0.04637	-0.05818	-0.00575	0.03955	-0.00201	-0.15362	0.03391	-0.31186	0.31186	0.54703	0.44976	0.94052	0.60751	0.97923	0.04485	0.6597	3.29E-05	3.29E-05	grey
GALNT5	0.11111	0.04881	0.1578	0.05615	-0.04763	0.28531	0.22097	-0.31472	0.31472	0.14796	0.52608	0.03927	0.46575	0.53617	0.00016	0.00368	2.76E-05	2.76E-05	yellow
ADH6	0.04516	-0.05351	-0.12761	-0.06552	0.23048	-0.05114	-0.04548	0.10559	-0.10559	0.55754	0.48703	0.09624	0.39453	0.00242	0.50655	0.55473	0.1693	0.1693	grey
PREB	0.07964	0.00046	-0.0164	0.05057	0.00039	-0.04809	0.04268	0.09517	-0.09517	0.30044	0.9952	0.83137	0.51127	0.996	0.53221	0.57943	0.21564	0.21564	turquoise
TXLNB	-0.09111	-0.03554	0.06782	0.07042	-0.18662	0.1775	-0.01246	0.57015	-0.57015	0.23594	0.64444	0.37809	0.36009	0.01453	0.0202	0.8715	3.97E-16	3.97E-16	blue
GSTM5	-0.08222	-0.08258	-0.01368	0.02776	-0.13181	0.14023	-0.0753	0.50793	-0.50793	0.28505	0.28293	0.85903	0.71854	0.08571	0.06734	0.32764	1.33E-12	1.33E-12	blue
CHM	-0.17071	-0.19216	-0.07175	0.01993	-0.0979	-0.01064	-0.05784	0.32731	-0.32731	0.02559	0.01181	0.35107	0.79582	0.20271	0.89016	0.45236	1.24E-05	1.24E-05	turquoise
HMGN3	-0.04171	-0.13278	-0.1299	-0.06156	0.17788	-0.17839	-0.13678	0.1699	-0.1699	0.58806	0.08341	0.09037	0.42382	0.01994	0.01957	0.07443	0.02631	0.02631	grey
PPM1G	0.03731	-0.00481	-0.01917	-0.02813	-0.0426	0.06675	0.22343	-0.35709	0.35709	0.62809	0.95019	0.80343	0.71497	0.5801	0.38569	0.00331	1.63E-06	1.63E-06	turquoise
PROSER3	-0.00997	-0.17096	-0.03123	0.02331	0.01269	0.03745	0.03888	-0.07176	0.07176	0.89703	0.02538	0.68512	0.76218	0.86911	0.62679	0.6136	0.35097	0.35097	red
LRRC4B	-0.02681	-0.13784	-0.2374	-0.06887	0.19751	-0.3758	-0.24616	0.35637	-0.35637	0.72776	0.07219	0.00177	0.37076	0.00962	4.08E-07	0.00117	1.72E-06	1.72E-06	brown
GOLGA3	0.02371	-0.15675	-0.05851	-0.05268	0.10346	-0.12003	-0.04805	0.1308	-0.1308	0.75825	0.04062	0.4472	0.4938	0.17812	0.11787	0.53259	0.08815	0.08815	turquoise
NSF	0.03671	-0.14013	-0.16633	-0.09305	0.12021	-0.20046	-0.15084	0.1977	-0.1977	0.63355	0.06754	0.02969	0.22609	0.11733	0.00857	0.04891	0.00954	0.00954	turquoise
CIAO2B	0.09275	-0.00421	-0.05972	-0.10226	0.14151	-0.09002	0.06838	-0.41745	0.41745	0.22762	0.95643	0.4378	0.18324	0.06487	0.24162	0.37421	1.34E-08	1.34E-08	grey
FASTKD5	-0.07364	-0.05513	-0.07593	-0.02142	-0.03736	-0.01358	-0.05226	0.17416	-0.17416	0.33845	0.47391	0.32362	0.78097	0.62757	0.86009	0.49725	0.02272	0.02272	turquoise
SMIM5	-0.01338	-0.15948	-0.07194	-0.07784	0.23456	-0.15048	-0.05627	-0.21881	0.21881	0.86211	0.0372	0.34975	0.31154	0.00202	0.04946	0.46475	0.00404	0.00404	grey
SLC43A3	-0.0535	-0.03081	-0.05413	0.12465	-0.20477	0.07788	0.13791	0.13831	-0.13831	0.48704	0.68909	0.48197	0.10428	0.00722	0.31131	0.07204	0.07123	0.07123	black
RBM41	-0.04997	-0.14432	0.0432	0.00798	-0.04903	0.16544	0.15398	-0.15	0.15	0.51633	0.05967	0.57478	0.91746	0.52426	0.03059	0.04435	0.05021	0.05021	turquoise
HPSE	-0.12018	-0.04277	-0.06329	-0.1089	-0.13522	0.03538	0.07059	0.00217	-0.00217	0.11742	0.57858	0.41084	0.15626	0.07784	0.6459	0.35887	0.97748	0.97748	turquoise
USP32	-0.01326	-0.19172	-0.07473	0.00217	-0.14338	0.03842	0.08914	0.1549	-0.1549	0.86335	0.012	0.33133	0.97748	0.06136	0.61786	0.24627	0.04308	0.04308	turquoise
KRAS	0.01799	-0.11808	0.02981	0.07831	-0.07548	0.10565	0.03797	-0.16274	0.16274	0.81534	0.12401	0.6987	0.30861	0.3265	0.16904	0.62194	0.03345	0.03345	turquoise
RNF43	0.1728	0.04232	-0.01512	-0.0514	0.14704	0.03958	0.03366	-0.22828	0.22828	0.02382	0.5826	0.84436	0.50434	0.05497	0.60726	0.6621	0.00267	0.00267	grey
SH3YL1	0.06788	-0.05191	0.03758	0.02404	0.01016	0.19666	0.07801	-0.27036	0.27036	0.37766	0.50014	0.6256	0.75493	0.89506	0.00993	0.31048	0.00035	0.00035	grey
PSMC6	0.03837	-0.13731	0.0098	0.04123	0.04054	0.0209	0.02639	-0.20026	0.20026	0.61831	0.07331	0.89874	0.59232	0.59856	0.78615	0.7319	0.00864	0.00864	turquoise
COMP	0.02515	-0.02097	0.00432	0.06425	-0.15585	0.20853	0.09556	0.05699	-0.05699	0.74406	0.78547	0.9553	0.40376	0.0418	0.0062	0.21378	0.45905	0.45905	black
SLC35A5	-0.11323	-0.10959	-0.11361	-0.03658	-0.13941	0.04155	-0.00937	0.31092	-0.31092	0.14032	0.1536	0.13898	0.63474	0.06899	0.5895	0.90319	3.49E-05	3.49E-05	turquoise
B4GALT7	0.06426	-0.05596	-0.16342	-0.06387	0.20154	-0.1775	-0.00167	-0.17196	0.17196	0.40371	0.46724	0.0327	0.40655	0.00821	0.0202	0.98272	0.02451	0.02451	green
LGALS9B	0.07151	0.04542	0.09043	-0.04223	0.16359	0.1118	0.0893	-0.20196	0.20196	0.35263	0.55524	0.23951	0.58336	0.03252	0.14544	0.24544	0.00807	0.00807	grey
SLC35B2	0.02259	-0.06152	-0.03843	-0.02601	-0.07554	0.02484	0.13384	-0.22099	0.22099	0.76928	0.42409	0.61777	0.73557	0.32613	0.7471	0.08096	0.00368	0.00368	turquoise
WDR11	-0.11865	-0.11432	0.00964	0.03038	-0.06194	0.05507	0.06631	0.07844	-0.07844	0.1222	0.13653	0.90041	0.6932	0.42092	0.47435	0.38888	0.30782	0.30782	turquoise
SOAT1	-0.06689	-0.17349	-0.07278	0.04876	-0.10946	0.03909	-0.06328	0.42502	-0.42502	0.3847	0.02325	0.34413	0.52656	0.1541	0.61174	0.41092	6.86E-09	6.86E-09	turquoise
TCEAL3	-0.01048	-0.19471	-0.23312	-0.1243	0.05335	-0.25904	-0.12253	0.34572	-0.34572	0.89176	0.01071	0.00215	0.10528	0.48827	0.00062	0.11038	3.63E-06	3.63E-06	brown
BTBD3	-0.02141	-0.17267	-0.13304	-0.06629	0.07403	-0.20523	-0.09376	0.181	-0.181	0.78102	0.02392	0.08281	0.38902	0.33591	0.00709	0.22257	0.01783	0.01783	turquoise
DLG5	0.00112	-0.09757	0.06693	0.08993	-0.1034	0.02746	0.15553	-0.06936	0.06936	0.98841	0.20423	0.38442	0.24209	0.17836	0.72145	0.04223	0.36738	0.36738	turquoise
SURF2	0.07941	-0.04562	-0.19219	-0.1332	0.12813	-0.30714	-0.01904	-0.16048	0.16048	0.30187	0.55355	0.01179	0.08243	0.09489	4.39E-05	0.80476	0.03601	0.03601	grey
CLDN12	0.06876	-0.17669	0.00615	-0.03995	0.0907	0.03731	0.01333	-0.29896	0.29896	0.37149	0.02079	0.93638	0.60387	0.23807	0.62804	0.86261	7.13E-05	7.13E-05	turquoise
DMD	-0.06203	-0.02384	-0.033	0.05459	-0.12941	0.03274	-0.12318	0.4517	-0.4517	0.42027	0.75694	0.66831	0.47825	0.09162	0.67075	0.10847	5.61E-10	5.61E-10	turquoise
PRDM15	-0.01318	-0.1241	-0.04431	-0.01574	0.00962	-0.03467	0.00238	0.19125	-0.19125	0.86418	0.10585	0.56495	0.83806	0.90064	0.65256	0.97532	0.01222	0.01222	turquoise
PDE1B	-0.02646	-0.03331	-0.10156	-0.00025	-0.0533	-0.11936	-0.12407	0.54737	-0.54737	0.73117	0.66536	0.18623	0.99737	0.48867	0.11997	0.10593	9.43E-15	9.43E-15	blue
PCNT	-0.08216	-0.13364	-0.17993	-0.10908	0.13723	-0.24856	-0.08442	0.26877	-0.26877	0.28537	0.08141	0.01853	0.15556	0.07348	0.00105	0.27227	0.00038	0.00038	turquoise
PAICS	-0.05731	0.06541	0.01873	-0.01153	-0.05	0.01837	0.24159	-0.22149	0.22149	0.45655	0.39535	0.80791	0.88103	0.51605	0.81149	0.00146	0.0036	0.0036	turquoise
ZNF223	-0.05055	-0.15601	-0.00971	-0.02742	0.1138	0.02063	-0.04283	0.0109	-0.0109	0.51144	0.04159	0.89973	0.72186	0.13834	0.7888	0.57804	0.88751	0.88751	turquoise
CASC4	-0.09311	-0.16599	-0.01226	0.00282	-0.06013	0.03723	-0.07853	0.1542	-0.1542	0.22578	0.03002	0.87358	0.97079	0.43466	0.62877	0.30728	0.04404	0.04404	turquoise
EEF1A1	-0.01503	0.03202	0.0572	0.00603	-0.14555	0.15502	-0.02274	0.23913	-0.23913	0.84526	0.67762	0.45745	0.93762	0.05749	0.04292	0.76784	0.00163	0.00163	turquoise
NPR1	-0.09554	-0.07537	-0.07764	0.07005	-0.0133	-0.09498	-0.17055	0.58809	-0.58809	0.21384	0.32722	0.31283	0.36261	0.86289	0.21657	0.02573	2.74E-17	2.74E-17	blue
BIVM	-0.07607	-0.06748	-0.1617	-0.08124	0.09233	-0.19146	-0.03686	0.31753	-0.31753	0.3227	0.3805	0.03461	0.29081	0.22973	0.01212	0.63222	2.32E-05	2.32E-05	turquoise
TMEM47	-0.12417	-0.09411	-0.01618	0.14194	-0.11059	0.07416	-0.00697	0.20459	-0.20459	0.10564	0.2208	0.83363	0.06405	0.14988	0.33506	0.92796	0.00727	0.00727	blue
GBP3	-0.03912	-0.1469	0.20984	0.05899	-0.04395	0.21385	0.12743	-0.08716	0.08716	0.61141	0.05519	0.00587	0.44345	0.56818	0.00498	0.09673	0.25699	0.25699	grey
ADRA2C	-0.08219	-0.09052	-0.24496	0.02167	0.08068	-0.11719	-0.12099	0.21311	-0.21311	0.28521	0.23901	0.00124	0.77845	0.29416	0.12689	0.11496	0.00513	0.00513	grey
TEX264	0.03852	0.06243	-0.13408	-0.08582	0.10949	-0.03434	-0.00808	-0.30717	0.30717	0.61689	0.41729	0.08039	0.2644	0.15399	0.65568	0.91642	4.38E-05	4.38E-05	grey
CAPN1	0.02005	-0.01156	0.06896	0.02931	-0.08354	0.14694	0.21212	-0.37561	0.37561	0.7946	0.88073	0.3701	0.70354	0.27736	0.05514	0.00535	4.14E-07	4.14E-07	yellow
ADO	-0.0544	-0.11629	0.02895	0.04555	-0.16223	0.10349	0.06699	0.11153	-0.11153	0.47979	0.12984	0.70701	0.55413	0.03401	0.17799	0.38398	0.14642	0.14642	turquoise
FAM133B	-0.06596	-0.20189	0.04022	0.03896	-0.04972	0.04567	-0.03633	0.12068	-0.12068	0.39133	0.0081	0.60145	0.61288	0.5184	0.55306	0.63711	0.11587	0.11587	turquoise
AP2S1	0.03463	0.01939	-0.05869	-0.0121	-0.01731	-0.05378	0.15553	-0.25848	0.25848	0.65298	0.80129	0.44574	0.87517	0.82224	0.48481	0.04222	0.00064	0.00064	grey
NABP1	-0.07158	-0.09587	0.12321	0.11365	-0.15717	0.21217	0.1287	0.07894	-0.07894	0.35215	0.21227	0.10839	0.13887	0.04008	0.00534	0.09343	0.30474	0.30474	black
ATP6V1F	-0.02439	-0.07829	-0.04068	-0.00821	0.01724	-0.1623	0.00429	-0.05328	0.05328	0.75154	0.30875	0.59731	0.91511	0.82293	0.03393	0.95564	0.48884	0.48884	grey
AMFR	-0.00571	-0.20655	-0.05446	-0.03301	-0.04665	0.02161	-0.06598	-0.01032	0.01032	0.94094	0.00672	0.47927	0.6682	0.54462	0.77903	0.39122	0.89341	0.89341	turquoise
RBPMS2	-0.09131	-0.10456	-0.0249	0.09168	-0.02697	0.01441	-0.01024	0.28667	-0.28667	0.23494	0.17353	0.74645	0.23301	0.72618	0.85161	0.8942	0.00014	0.00014	salmon
BARX1	0.06983	-0.02414	0.00733	0.05497	-0.03901	0.01847	0.18238	-0.12921	0.12921	0.36414	0.75396	0.92418	0.47516	0.61244	0.81052	0.01697	0.09211	0.09211	grey

KDM2A	-0.02769	-0.13779	0.01719	0.04972	-0.15722	0.13424	0.11479	-0.02139	0.02139	0.71925	0.07231	0.82341	0.51844	0.04002	0.08004	0.13491	0.78128	0.78128	turquoise
NDUFA9	0.07366	0.09942	-0.13087	-0.06258	0.15797	-0.20215	-0.0502	-0.19718	0.19718	0.33831	0.19575	0.08798	0.41618	0.03907	0.00801	0.51433	0.00974	0.00974	grey
POC5	0.05531	-0.00553	-0.07719	-0.04261	0.00708	-0.13894	-0.10494	0.0651	-0.0651	0.47245	0.94277	0.31562	0.58001	0.92677	0.06994	0.17194	0.39757	0.39757	turquoise
SH3BGRL3	0.00176	0.01313	0.13015	0.02788	-0.0008	0.22758	0.14472	-0.36959	0.36959	0.98176	0.8647	0.08975	0.71738	0.99173	0.00276	0.05896	6.53E-07	6.53E-07	yellow
SNX5	-0.00699	0.04508	0.00776	0.01497	0.00551	0.03281	0.14619	-0.03495	0.03495	0.92774	0.55826	0.91979	0.84587	0.943	0.67006	0.05639	0.64993	0.64993	pink
UGT2A3	0.00172	-0.08423	0.04394	0.08936	0.06757	0.26725	-0.00429	0.05065	-0.05065	0.98216	0.27336	0.56821	0.24511	0.37988	0.00041	0.95556	0.51059	0.51059	magenta
RBM34	0.00895	-0.018	-0.0362	-0.01592	-0.01835	-0.0098	0.12624	-0.1908	0.1908	0.90747	0.81525	0.6383	0.8363	0.81169	0.89872	0.09991	0.01243	0.01243	greenyellow
ARHGAP2	0.04802	0.02321	0.14446	0.02371	0.12935	0.24209	0.20015	-0.44613	0.44613	0.53282	0.76314	0.05941	0.75827	0.09176	0.00142	0.00867	9.63E-10	9.63E-10	yellow
SNCB	0.06727	-0.03136	-0.16915	-0.06517	0.08488	-0.3314	-0.13601	0.31344	-0.31344	0.382	0.68385	0.02699	0.3971	0.2697	9.53E-06	0.07611	2.99E-05	2.99E-05	brown
SLC9A9	-0.0882	-0.04512	0.00201	0.03226	-0.17349	0.1242	-0.02936	0.58595	-0.58595	0.25133	0.55784	0.97918	0.67531	0.02325	0.10557	0.70306	3.81E-17	3.81E-17	blue
TAF5	-0.05596	-0.11701	-0.08174	-0.03381	-0.05329	-0.04801	0.08473	0.19325	-0.19325	0.46727	0.12748	0.28788	0.66063	0.48875	0.53294	0.27051	0.01133	0.01133	turquoise
BHLHE41	-0.03475	-0.13802	0.04743	0.04692	-0.14008	0.16916	0.09544	-0.01356	0.01356	0.65185	0.07183	0.53787	0.5423	0.06765	0.02698	0.21435	0.86026	0.86026	turquoise
ADAT3	0.06025	0.05105	-0.09331	-0.10902	0.20626	-0.03497	0.10926	-0.29984	0.29984	0.43371	0.50727	0.22478	0.15578	0.0068	0.64978	0.15486	6.77E-05	6.77E-05	green
RAD51AP1	-0.03235	0.08548	0.05472	0.04413	-0.03923	0.07761	0.21638	-0.29137	0.29137	0.6745	0.26631	0.47719	0.56653	0.61042	0.31297	0.00448	0.00011	0.00011	pink
ZNF638	-0.01443	-0.18252	-0.13377	-0.03727	-0.05706	-0.0472	-0.02009	0.12687	-0.12687	0.85144	0.01688	0.08111	0.62844	0.45849	0.53982	0.79425	0.09822	0.09822	red
ALKBH2	0.06068	0.15757	0.03396	0.00102	0.00019	0.09755	0.18396	-0.21706	0.21706	0.43045	0.03957	0.65923	0.98941	0.99798	0.20433	0.01602	0.00435	0.00435	purple
GTF2H5	-0.15851	-0.01617	-0.08765	0.00504	-0.02518	-0.15721	-0.01871	0.20036	-0.20036	0.03839	0.83377	0.25432	0.94782	0.74372	0.04002	0.8081	0.0086	0.0086	grey
LDLRAD3	-0.03378	-0.10084	-0.00621	0.17491	-0.22734	-0.00683	0.06766	0.27845	-0.27845	0.66098	0.18942	0.93571	0.02213	0.00279	0.92937	0.37925	0.00023	0.00023	blue
HPGDS	-0.10625	-0.06493	-0.03562	0.09306	-0.07502	0.15973	0.0886	0.30195	-0.30195	0.16662	0.39885	0.64369	0.22604	0.32949	0.0369	0.24918	5.98E-05	5.98E-05	blue
ERF	-0.04209	-0.09069	-0.10767	-0.02063	0.10348	0.06274	-0.01578	-0.07686	0.07686	0.58463	0.23816	0.16099	0.78888	0.178	0.41495	0.83769	0.31773	0.31773	green
1-Mar	-0.03787	0.2282	-0.03358	-0.02501	0.10271	-0.026	0.00394	-0.01074	0.01074	0.62286	0.00268	0.66278	0.74541	0.18128	0.73567	0.95919	0.88916	0.88916	grey
FSTL3	-0.00148	-0.00664	0.01945	0.09714	-0.14564	0.13019	0.0903	-0.01041	0.01041	0.98467	0.93136	0.80061	0.20623	0.05735	0.08967	0.24019	0.89249	0.89249	black
SLC25A12	-0.02061	-0.0412	-0.1003	-0.00676	-0.08528	-0.14535	-0.06123	0.25779	-0.25779	0.78905	0.59262	0.19178	0.93007	0.26743	0.05786	0.42628	0.00066	0.00066	turquoise
VRK1	-0.04057	0.09275	0.04709	-0.02993	-0.00466	0.03335	0.05912	-0.14876	0.14876	0.59829	0.22761	0.5408	0.69759	0.95182	0.66499	0.44241	0.05215	0.05215	turquoise
ZNF367	-0.05914	0.04152	-0.03536	-0.02457	-0.06602	0.0356	0.15064	-0.15672	0.15672	0.44226	0.58973	0.64614	0.74975	0.39091	0.64386	0.04922	0.04066	0.04066	pink
ASH1L	0.00542	-0.15269	-0.05615	-0.01257	-0.07236	0.07451	0.00874	0.20792	-0.20792	0.94394	0.04618	0.46577	0.8704	0.34698	0.33279	0.90963	0.00635	0.00635	turquoise
PSMD9	0.0159	-0.00766	0.04657	-0.00527	0.1011	-0.00876	0.05003	-0.16795	0.16795	0.83643	0.92084	0.54526	0.94546	0.18827	0.9095	0.51582	0.02811	0.02811	turquoise
MRPS22	-0.03798	0.02098	-0.00645	-0.03471	-0.10026	0.13366	0.23261	-0.14882	0.14882	0.62186	0.78532	0.93324	0.65219	0.19197	0.08137	0.0022	0.05206	0.05206	turquoise
YJEFN3	0.06576	-0.10135	-0.12032	-0.02608	0.17844	-0.09952	0.01726	-0.13036	0.13036	0.39277	0.18716	0.11698	0.73491	0.01954	0.19529	0.82271	0.08923	0.08923	red
PCMT1	-0.01886	-0.07025	-0.06557	-0.04498	-0.03024	-0.19159	-0.06619	0.25017	-0.25017	0.80662	0.3612	0.39421	0.55909	0.69456	0.01206	0.38972	0.00097	0.00097	turquoise
CDKN2C	-0.02506	-0.04607	-0.06591	0.05996	0.07072	-0.06005	0.0884	0.01207	-0.01207	0.74492	0.54964	0.39172	0.43594	0.35802	0.43531	0.25023	0.87545	0.87545	grey
CD3EAP	0.04243	-0.01542	0.02609	0.01629	-0.16552	0.11253	0.24265	-0.09487	0.09487	0.58162	0.84134	0.73485	0.83248	0.0305	0.1428	0.00139	0.21711	0.21711	turquoise
LRRC27	0.03466	-0.07243	-0.13385	-0.03332	0.10205	-0.21281	0.00437	0.12518	-0.12518	0.6527	0.34649	0.08093	0.66525	0.1841	0.0052	0.95479	0.1028	0.1028	grey
NECTIN2	0.05497	-0.12833	-0.04107	-0.0216	0.05783	0.04988	0.08123	-0.27624	0.27624	0.47518	0.09439	0.59378	0.77913	0.45243	0.51703	0.29091	0.00025	0.00025	grey
MATK	-0.11483	0.05512	-0.02525	0.0744	0.02136	0.02132	-0.00229	0.40349	-0.40349	0.13477	0.474	0.74304	0.33349	0.78159	0.78198	0.97626	4.45E-08	4.45E-08	blue
ATAT1	0.02279	-0.06637	-0.0874	-0.12044	0.16699	-0.10065	0.02773	-0.17095	0.17095	0.76737	0.38843	0.25565	0.11661	0.02903	0.19024	0.71881	0.02538	0.02538	red
EBF4	0.02614	-0.12207	-0.21311	-0.00305	0.12777	-0.24522	-0.09615	0.21614	-0.21614	0.73436	0.11172	0.00513	0.96839	0.09582	0.00123	0.21095	0.00452	0.00452	grey
OVOL1	0.0871	-0.00387	0.0803	0.03144	0.06789	0.18261	0.19078	-0.48972	0.48972	0.25729	0.95997	0.29645	0.68313	0.37763	0.01682	0.01244	1.06E-11	1.06E-11	yellow
RAB24	-0.00884	-0.11463	-0.13181	-0.05872	0.26879	-0.16836	0.00323	-0.1597	0.1597	0.90866	0.13545	0.0857	0.4455	0.00038	0.02773	0.9666	0.03694	0.03694	red
DISP2	-0.07571	-0.03009	-0.1978	-0.10982	0.03874	-0.31272	-0.12029	0.17839	-0.17839	0.325	0.69604	0.00951	0.15275	0.61496	3.12E-05	0.11708	0.01958	0.01958	brown
DMAC1	0.09428	-0.02656	-0.0828	-0.13373	0.07637	-0.07488	0.13299	-0.03513	0.03513	0.22	0.73026	0.28164	0.08121	0.32084	0.33036	0.0829	0.64831	0.64831	grey
EMG1	0.0085	-0.00494	-0.06683	-0.0448	0.07362	-0.14347	0.02745	-0.26679	0.26679	0.91211	0.94883	0.38514	0.56066	0.33859	0.06121	0.72152	0.00042	0.00042	grey
RAB5B	0.07563	-0.19516	-0.08731	-0.0099	-0.03911	0.01098	-0.07144	-0.00742	0.00742	0.32551	0.01053	0.25616	0.89775	0.61154	0.88671	0.35316	0.92331	0.92331	turquoise
MC1R	-0.00817	-0.13808	-0.13319	-0.05169	0.23142	-0.1947	-0.05977	-0.08677	0.08677	0.91549	0.0717	0.08244	0.50197	0.00232	0.01072	0.43743	0.2591	0.2591	red
COMMD9	-0.02371	-0.13639	-0.08255	-0.01586	-0.15251	-0.10602	-0.00894	0.10036	-0.10036	0.75821	0.07527	0.28309	0.8369	0.04645	0.16754	0.90759	0.19155	0.19155	grey
COL24A1	-0.07026	-0.00511	0.0589	0.03783	-0.13275	0.11405	0.03218	0.34695	-0.34695	0.36116	0.94709	0.44411	0.62324	0.08347	0.13746	0.67603	3.34E-06	3.34E-06	blue
NIPSNAP1	-0.0638	-0.0241	-0.14691	-0.0889	-0.07643	-0.13084	0.08904	-0.05788	0.05788	0.40712	0.75438	0.05519	0.24755	0.32041	0.08805	0.24684	0.4521	0.4521	turquoise
AP4E1	-0.06697	-0.16817	-0.03892	0.00597	-0.08921	0.00944	0.03476	0.17907	-0.17907	0.38413	0.02791	0.61331	0.93821	0.24592	0.90246	0.6517	0.0191	0.0191	turquoise
KCNG1	0.1067	0.051	-0.18735	-0.02028	-0.01476	-0.10669	-0.02602	0.23022	-0.23022	0.16482	0.50765	0.01414	0.79232	0.84808	0.16487	0.73553	0.00245	0.00245	grey
TBC1D23	-0.04705	-0.16079	-0.02323	0.00069	-0.12646	0.10413	0.07657	0.13011	-0.13011	0.54118	0.03565	0.76299	0.99283	0.09931	0.1753	0.31953	0.08985	0.08985	turquoise
LRWD1	0.05097	-0.1079	-0.07708	-0.0956	0.16829	-0.11062	0.00352	-0.13579	0.13579	0.50793	0.16008	0.31634	0.21357	0.02779	0.14977	0.96355	0.07659	0.07659	green
SUSD6	-0.07241	-0.19562	0.03087	0.06356	-0.14498	0.13934	-0.06405	0.20993	-0.20993	0.34665	0.01034	0.68854	0.40888	0.0585	0.06911	0.40522	0.00585	0.00585	turquoise
TMEM25	-0.03636	-0.1877	-0.14673	-0.05029	-0.07189	-0.15406	-0.16496	0.26465	-0.26465	0.63687	0.01396	0.05548	0.51363	0.35013	0.04424	0.03108	0.00047	0.00047	brown
CCRL2	0.03176	0.05277	0.20854	0.08418	-0.02835	0.34168	0.0996	-0.13382	0.13382	0.68005	0.49303	0.0062	0.27366	0.71281	4.79E-06	0.19494	0.08099	0.08099	yellow
GXYLT2	-0.04423	-0.10052	0.02987	0.06387	-0.24763	0.1448	0.04056	0.4573	-0.4573	0.56571	0.19082	0.69812	0.40659	0.00109	0.05881	0.59841	3.22E-10	3.22E-10	blue
STRN3	0.00269	-0.19271	-0.01427	0.12748	-0.03751	0.03602	-0.0081	0.11287	-0.11287	0.97218	0.01156	0.85302	0.09659	0.62621	0.63994	0.9163	0.14161	0.14161	turquoise
NHS	-0.04404	-0.02366	0.17229	0.11014	-0.07717	0.17319	0.09942	-0.15425	0.15425	0.56734	0.75874	0.02424	0.15154	0.31578	0.0235	0.19576	0.04397	0.04397	turquoise
PDCD7	-0.08686	-0.1401	-0.11856	-0.06964	-0.01197	-0.07835	-0.04076	0.22149	-0.22149	0.2586	0.06761	0.12248	0.36539	0.87649	0.30839	0.59658	0.0036	0.0036	turquoise
NAA20	0.00884	-0.10265	-0.14405	-0.06497	0.15609	-0.40112	-0.16823	0.1331	-0.1331	0.90865	0.18153	0.06016	0.39851	0.04148	5.42E-08	0.02784	0.08266	0.08266	brown
SNAPIN	-0.05885	-0.05294	-0.05705	-0.08387	-0.01431	0.08868	0.1435	-0.15897	0.15897	0.44451	0.49163	0.45862	0.27544	0.85267	0.24873	0.06114	0.03783	0.03783	greenyellow
UBE2R2	0.07757	-0.05093	-0.06013	-0.0068	-0.15357	0.04445	0.0475	-0.05993	0.05993	0.31325	0.50827	0.43465	0.92962	0.04492	0.56379	0.53729	0.43621	0.43621	turquoise
NCMAP	-0.05327	-0.09455	0.00535	0.04048	-0.05699	0.15237	-0.00268	0.0926	-0.0926	0.48898	0.21867	0.94462	0.59915	0.45911	0.04665	0.97224	0.22837	0.22837	magenta
MOAP1	0.04102	-0.16179	-0.17782	-0.02908	0.14708	-0.19355	-0.28153	0.2666	-0.2666	0.59425	0.0345	0.01997	0.70575	0.0549	0.0112	0.00019	0.00042	0.00042	turquoise
ERICH1	-0.02857	-0.00825	0.10228	0.05348	-0.03872	0.11321	0.10658	-0.00188	0.00188	0.71068	0.91471	0.18314	0.48725	0.61507	0.14039	0.16532	0.98048	0.98048	turquoise
E2F7	0.01777	0.01866	0.02866	-0.0283	-0.10213	0.05047	0.26647	-0.37205	0.37205	0.81756	0.80862	0.70978	0.71328	0.18378	0.51207	0.00043	5.43E-07	5.43E-07	pink
ZNF300	0.02731	-0.11141	0.07332	0.11134	-0.05542	0.0891	0.00247	-0.01966	0.01966	0.72296	0.14686	0.3406	0.1471	0.47156	0.24649	0.97438	0.7985	0.7985	grey
CENPO	-0.03588	0.04313	-0.03878	-0.0399	-0.04265	-0.07798	0.21513	-0.11476	0.11476	0.64126	0.57539	0.61457	0.60435	0.57962	0.31068	0.00472	0.13501	0.13501	pink
MIOS	-0.04548	-0.13764	0.00519	0.0473	-0.11407	0.09018	0.06736	0.14288	-0.14288	0.55475	0.07262	0.94629	0.53903	0.13738	0.24077	0.38138	0.06228	0.06228	turquoise
SIGLEC8	-0.04606	-0.02992	0.06901	0.07656	-0.26264	0.11738	-0.02125	0.38001	-0.38001	0.54973	0.69765	0.36982	0.31959	0.00052	0.12627	0.7826	2.95E-07	2.95E-07	blue
IFI35	0.04957	0.06073	0.08517	0.08193	-0.01369	0.07745	0.17404	-0.23936	0.23936	0.51965	0.43009	0.26805	0.28672	0.85892	0.31399	0.02282	0.00162	0.00162	tan
IGF2R	-0.03569	-0.13645	-0.01345	0.03065	-0.19237	0.063	0.05835	0.28639	-0.28639	0.64303	0.07514	0.86135	0.6907	0.01171	0.41304	0.44844	0.00015	0.00015	turquoise
DHX40	-0.03511	-0.2609	-0.13764	-0.03756	-0.0667	-0.14667	-0.08949	0.26294	-0.26294	0.64844	0.00057	0.07261	0.62571	0.38603	0.05558	0.24443	0.00051	0.00051	turquoise
BMP1	-0.06435	-0.0647	0.06687	0.10474	-0.09689	0.03849	0.12701	0.01202	-0.01202	0.40308	0.40052	0.38483	0.17278	0.20741	0.61716	0.09785	0.87601	0.87601	black
PHLPP2	-0.03013	-0.31219	-0.12037	-0.02587	0.00246	-0.12464	-0.079	0.32557	-0.32557	0.69568	3.23E-05	0.11685	0.73698	0.97455	0.10433	0.3044	1.39E-05	1.39E-05	turquoise
PLAAT3	-0.00062	-0.13766	0.0389	0.03267	-0.06939	0.0987	0.2082	-0.31553	0.31553	0.99359	0.07258	0.6135	0.67145	0.36717	0.19903	0.00628	2.63E-05	2.63E-05	yellow
NTHL1	0.12431	0.09505	-0.07532	-0.07489	0.23402	-0.05381	0.11071	-0.41059	0.41059	0.10525	0.21624	0.3275	0.33028	0.00207	0.48459	0.14944	2.44E-08	2.44E-08	green
TMEM119	-0.02698	-0.05024	-0.00052	0.03868	-0.19383	0.15229	-0.00364	0.43924	-0.43924	0.72613	0.51405	0.99461	0.61543	0.01108	0.04677	0.96234	1.86E-09	1.86E-09	blue
GLOD5	0.00097	-0.01001	-0.04987	0.02462	0.21276	0.04767	0.01338	-0.37919	0.37919	0.98995	0.89663	0.51716	0.74922	0.00521	0.53586	0.86215	3.15E-07	3.15E-07	grey
PREX2	-0.09192	-0.16226	-0.07896	0.04271	-0.15153	0.07448	-0.0515	0.54653	-0.54653	0.23182	0.03397	0.30463	0.57911	0.04788	0.33294	0.50351	1.06E-14	1.06E-14	blue
BCL2L11	-0.02204	-0.11917	-0.06311	0.0283	-0.18426	0.02531	-0.01052	0.25922	-0.25922	0.77479	0.12056	0.41219	0.71328	0.01584	0.74245	0.89134	0.00062	0.00062	turquoise
GGPS1	-0.02542	-0.03704	-0.08226	-0.06731	-0.04097	-0.00301	0.05281	-0.07788	0.07788	0.74138	0.63051	0.28478	0.38171	0.59471	0.96887	0.49276	0.31129	0.31129	turquoise
DCAF5	0.01386	-0.23163	-0.03238	0.04045	-0.00941	0.02992	-0.02214	0.10572	-0.10572	0.85718	0.0023	0.6742	0.5994	0.90276	0.69766	0.77381	0.16875	0.16875	turquoise
GAP43	0.03625	-0.10455	-0.02156	0.07106	-0.12394	-0.04513	-0.05242	0.41911	-0.41911	0.63787	0.17355	0.77955	0.35568	0.10629	0.55781	0.49588	1.16E-08	1.16E-08	grey
SPDL1	-0.07354	-0.05418	-0.02447	0.01182	-0.0951	0.01318	0.14748	-0.00695	0.00695	0.33914	0.48154	0.75068	0.8781	0.21597	0.86415	0.05423	0.92807	0.92807	turquoise
RSAD2	-0.06625	0.025	0.04748	0.0877	-0.22745	0.10943	0.15508	0.10691	-0.10691	0.38924	0.7455	0.53741	0.25403	0.00277	0.15421	0.04284	0.16398	0.16398	tan
CMA1	-0.0704	-0.05876	-0.00027	0.04607	-0.07375	0.03868	-0.14346	0.48333	-0.48333	0.36021	0.44521	0.99725	0.54965	0.33771	0.61549	0.06122	2.14E-11	2.14E-11	blue
ATRN	-0.02964	-0.16473	-0.14518	-0.04646	0.00122	-0.1671	-0.07183	0.33057	-0.33057	0.70035	0.03131	0.05815	0.54622	0.98742	0.02892	0.35053	1.01E-05	1.01E-05	turquoise
OMA1	-0.05196	-0.05665	0.11717	-0.02358	0.04629	0.21134	0.05955	-0.05741	0.05741	0.49971	0.46179	0.12696	0.75947	0.5477	0.00552	0.43909	0.45576	0.45576	turquoise
PLEKHG3	0.07866	-0.127	-0.0659	-0.02875	0.11523	-0.00611	0.02738	-0.23454	0.23454	0.30647	0.09786	0.39176	0.70898	0.1334	0.93677	0.72226	0.00202	0.00202	grey
SPG7	-0.00273	-0.08029	-0.1203	-0.07788	0.10414	-0.13579	0.01184	-0.19105	0.19105	0.97174	0.29652	0.11703	0.31129	0.17525	0.07657	0.87786	0.01231	0.01231	red
LAMP1	0.08389	-0.02039	-0.07717	-0.01828	-0.01696	-0.1161	0.05553	0.08352	-0.08352	0.27534	0.7912	0.31575	0.81239	0.82579	0.1305	0.47068	0.27746	0.27746	grey
FLNB	0.06053	-0.01892	0.12719	0.05967	-0.14401	0.1935	0.17626	-0.31661	0.31661	0.43162	0.80596	0.09736	0.43816	0.06023	0.01122	0.02111	2.45E-05	2.45E-05	turquoise
SLC3A1	0.00948	-0.03283	0.0004	0.1337	-0.00881	0.18064	-0.01162	0.08657	-0.08657	0.90201	0.66992	0.99588	0.08128	0.90897	0.01806	0.88012	0.26022	0.26022	magenta
MACO1	-0.06397	-0.15103	-0.06848	-0.00613	-0.0773	-0.0088	-0.09529	0.20286	-0.20286	0.40584	0.04863	0.37346	0.93659	0.31494	0.90909	0.21505	0.00779	0.00779	turquoise
SMIM8	-0.00212	-0.08434	-0.10563	-0.04396	-0.01275	-0.13359	-0.13068	0.35896	-0.35896	0.97807	0.27277	0.16911	0.56809	0.86854	0.08151	0.08844	1.43E-06	1.43E-06	turquoise
ENTPD2	0.03968	0.00677	-0.08419	-0.02122	0.17753	0.00174	-0.0197	-0.29423	0.29423	0.60635	0.92993	0.27361	0.78299	0.02018	0.98199	0.79819	9.38E-05	9.38E-05	grey
FBLN1	-0.07991	-0.04287	0.07614	0.16031	-0.17359	0.15135	0.08051	0.43362	-0.43362	0.29884	0.57768	0.32226	0.03622	0.02317	0.04815	0.29522	3.14E-09	3.14E-09	blue
COL12A1	-0.06759	-0.0397	0.04953	0.04634	-0.18032	0.18535	0.11949	0.13661	-0.13661	0.37974	0.60614	0.51999	0.54726	0.01827	0.01522	0.11956	0.0748	0.0748	black
CASKIN2	-0.10047	-0.19039	-0.21727	-0.0124	0.13088	-0.15869	-0.1485	0.33234	-0.33234	0.19103	0.01262	0.00431	0.87207	0.08796	0.03816	0.05257	8.96E-06	8.96E-06	grey
UGP2	-0.04275	-0.07128	0.05462	0.13103	-0.18228	0.1992	0.14937	0.08346	-0.08346	0.57874	0.35425	0.47801	0.08759	0.01703	0.009	0.05119	0.27779	0.27779	turquoise
S100PBP	-0.06023	-0.20545	-0.06286	-0.02217	-0.12538	0.02003	#######	0.30194	-0.30194	0.4339	0.00702	0.41402	0.77347	0.10227	0.79481	0.99968	5.98E-05	5.98E-05	turquoise
TIMM21	-0.13348	-0.0961	-0.15472	0.02255	0.06095	-0.17044	-0.08032	0.33948	-0.33948	0.08179	0.21119	0.04332	0.76973	0.42838	0.02583	0.29637	5.56E-06	5.56E-06	turquoise
C1orf50	0.00865	-0.05647	-0.08699	-0.0566	0.0379	0.00699	-0.01296	-0.09642	0.09642	0.91062	0.46318	0.25789	0.46217	0.62263	0.92765	0.86638	0.20963	0.20963	grey
HLA-DQB	-0.17054	0.03375	-0.02215	0.0448	-0.20845	0.1612	0.07038	0.2052	-0.2052	0.02574	0.6612	0.7737	0.56067	0.00622	0.03518	0.36034	0.0071	0.0071	blue
TM2D1	-0.05309	-0.1883	-0.03752	0.00946	-0.03113	-0.04126	-0.03206	0.09853	-0.09853	0.49045	0.01365	0.62612	0.90227	0.68605	0.59207	0.67726	0.19979	0.19979	turquoise
JPT1	0.01622	0.00216	0.06382	0.06008	0.00778	0.17115	0.25022	-0.48083	0.48083	0.83321	0.97759	0.40695	0.43504	0.91959	0.02521	0.00096	2.81E-11	2.81E-11	yellow
NKX2-2	0.02997	-0.06885	-0.32109	-0.20937	0.14233	-0.27723	-0.19823	0.30411	-0.30411	0.69716	0.37091	1.85E-05	0.00599	0.0633	0.00024	0.00935	5.26E-05	5.26E-05	brown
BECN1	-0.02877	-0.0784	-0.04971	-0.02704	0.0254	-0.00664	-0.06808	0.07604	-0.07604	0.70875	0.30809	0.51849	0.72556	0.74154	0.93129	0.37627	0.32294	0.32294	turquoise
C8orf58	-0.05722	-0.01922	0.10249	0.07046	-0.0763	0.07362	0.17035	-0.0312	0.0312	0.45723	0.80298	0.18225	0.3598	0.32127	0.33862	0.02591	0.68545	0.68545	black
CD38	-0.16675	0.03384	0.0913	0.04833	-0.15723	0.11348	0.07504	0.36588	-0.36588	0.02927	0.6604	0.23498	0.53021	0.04	0.13944	0.32933	8.61E-07	8.61E-07	blue
DUSP3	-0.08795	-0.14149	-0.07731	0.05059	-0.00506	0.01332	0.00162	0.20833	-0.20833	0.25268	0.06489	0.31489	0.51108	0.94768	0.86275	0.98319	0.00625	0.00625	turquoise
TPRKB	-0.02438	0.03571	-0.02669	-0.02178	-0.05927	0.00189	0.13661	-0.21931	0.21931	0.75157	0.64289	0.72896	0.77732	0.44125	0.98048	0.0748	0.00395	0.00395	turquoise
LRCH3	-0.0442	-0.19215	-0.06293	0.01831	-0.11113	-0.02487	0.05623	0.1331	-0.1331	0.56597	0.01181	0.41351	0.8121	0.1479	0.74682	0.46505	0.08265	0.08265	turquoise
GNAL	-0.0349	-0.15746	-0.11418	0.0363	0.01234	-0.06802	-0.10805	0.40295	-0.40295	0.65045	0.03971	0.13703	0.63741	0.87277	0.3767	0.15953	4.65E-08	4.65E-08	brown
TSC22D3	-0.02318	-0.07444	-0.04835	0.08106	-0.16276	0.02432	-0.0495	0.24397	-0.24397	0.76342	0.33322	0.53003	0.2919	0.03342	0.75215	0.52027	0.0013	0.0013	blue
EXOC6B	-0.01551	-0.17774	-0.02453	0.01205	-0.14293	0.08085	0.06958	0.08132	-0.08132	0.84039	0.02003	0.75009	0.87574	0.06219	0.29314	0.36582	0.29038	0.29038	turquoise
YOD1	-0.00811	-0.10147	0.05394	0.00371	0.04826	0.10741	0.11334	-0.30996	0.30996	0.91612	0.18664	0.48348	0.96161	0.53076	0.162	0.13994	3.70E-05	3.70E-05	turquoise
NOD1	-0.09772	-0.08516	0.01734	0.12851	-0.12884	0.10197	0.10026	0.25358	-0.25358	0.20353	0.2681	0.8219	0.09392	0.09306	0.18447	0.19199	0.00082	0.00082	blue
ATPSCKMT	-0.10412	-0.11566	-0.08757	0.04052	0.03555	-0.18003	0.01079	0.25796	-0.25796	0.17533	0.13195	0.25474	0.59872	0.64435	0.01846	0.88862	0.00066	0.00066	turquoise
SLC12A4	-0.09057	-0.14252	0.0429	0.08273	-0.22229	0.16647	0.15151	0.08921	-0.08921	0.23874	0.06295	0.57744	0.28205	0.00348	0.02954	0.04791	0.24594	0.24594	black
COQ9	0.03921	-0.00309	-0.05475	-0.06717	0.09078	0.07057	0.08898	-0.32496	0.32496	0.61066	0.96805	0.47696	0.38273	0.23769	0.35901	0.24716	1.45E-05	1.45E-05	grey
METTL14	-0.10766	-0.18026	-0.12114	-0.04415	-0.01254	-0.10295	-0.12847	0.31991	-0.31991	0.16105	0.01831	0.1145	0.56639	0.87073	0.18026	0.09402	2.00E-05	2.00E-05	turquoise
TCF4	-0.12471	-0.10434	0.0118	0.07549	-0.22328	0.08432	-0.01848	0.54508	-0.54508	0.10412	0.17441	0.87829	0.32647	0.00333	0.27285	0.81042	1.28E-14	1.28E-14	blue
STAT5B	-0.0541	-0.16664	-0.08363	-0.02808	-0.02314	-0.10135	-0.16992	0.48407	-0.48407	0.48216	0.02938	0.27681	0.71539	0.76384	0.18719	0.02629	1.98E-11	1.98E-11	turquoise
GLG1	0.00555	-0.16034	-0.06018	-0.01084	-0.13072	-0.0149	0.04974	0.17318	-0.17318	0.94253	0.03618	0.43431	0.88811	0.08834	0.84663	0.51822	0.0235	0.0235	turquoise
PCDH17	-0.05355	-0.07198	-0.18591	-0.01983	-0.07158	-0.06236	-0.10929	0.5775	-0.5775	0.48666	0.34947	0.01491	0.79683	0.35216	0.41777	0.15474	1.35E-16	1.35E-16	blue
ZNF518B	-0.1268	-0.2164	-0.04102	0.01552	-0.15717	0.03501	-0.02661	0.51045	-0.51045	0.0984	0.00447	0.59424	0.84034	0.04007	0.64944	0.72971	9.88E-13	9.88E-13	blue
FAM104A	-0.0234	-0.17337	-0.08157	-0.02546	0.043	-0.0613	0.01468	0.02414	-0.02414	0.76128	0.02335	0.28888	0.74101	0.57659	0.42573	0.84884	0.754	0.754	turquoise
SCARB2	-0.02622	-0.17441	-0.08326	0.00658	-0.08519	-0.06821	-0.03157	0.25189	-0.25189	0.73353	0.02252	0.27897	0.93195	0.26792	0.37535	0.68187	0.00089	0.00089	turquoise

ANKRD13.	-0.03384	-0.11844	-0.0442	-0.00588	-0.0626	-0.05107	-0.06325	0.34062	-0.34062	0.66033	0.12285	0.56597	0.93918	0.41602	0.50712	0.41115	5.15E-06	5.15E-06	blue
CSDE1	-0.06329	-0.08844	0.02758	0.0544	-0.15322	0.15479	0.01599	0.22171	-0.22171	0.41085	0.25001	0.72027	0.47978	0.04542	0.04322	0.83555	0.00356	0.00356	turquoise
ZC3H15	0.03477	-0.03629	0.03811	0.04675	-0.13497	0.13171	0.13298	-0.02143	0.02143	0.6516	0.63751	0.62067	0.54373	0.07839	0.08594	0.08293	0.78085	0.78085	turquoise
NAV3	-0.05002	-0.07118	0.02357	0.08005	-0.1963	0.07573	0.00398	0.5846	-0.5846	0.51591	0.35489	0.75956	0.29797	0.01007	0.32489	0.95875	4.67E-17	4.67E-17	blue
DLC1	-0.08913	-0.16673	-0.02665	0.04363	-0.17537	0.0106	-0.08005	0.63451	-0.63451	0.24632	0.02929	0.72931	0.57101	0.02178	0.89057	0.29795	1.19E-20	1.19E-20	blue
HDAC1	-0.04706	-0.04593	0.08702	-0.00493	0.07356	0.14372	-0.0045	-0.31926	0.31926	0.5411	0.55079	0.25773	0.94894	0.33901	0.06074	0.95339	2.08E-05	2.08E-05	turquoise
ZNF432	0.02852	-0.02711	-0.05591	-0.0837	0.12794	-0.04458	-0.09595	-0.06874	0.06874	0.7112	0.72486	0.46761	0.27644	0.09538	0.56257	0.21191	0.37164	0.37164	red
CBR3	0.0042	0.04573	-0.00316	0.02338	-0.02725	0.14528	0.20817	0.03866	-0.03866	0.95655	0.5526	0.96728	0.76147	0.72346	0.05798	0.00629	0.61569	0.61569	grey
UHRF1BP1	-0.09385	-0.10314	0.02798	0.09483	-0.15597	0.02592	-0.02613	0.28017	-0.28017	0.2221	0.17948	0.71636	0.21728	0.04165	0.73647	0.73442	0.00021	0.00021	turquoise
DOK1	-0.02655	0.00079	0.05829	0.03267	-0.15701	0.15668	0.2531	-0.05729	0.05729	0.73033	0.99185	0.44884	0.67146	0.04028	0.0407	0.00084	0.45671	0.45671	grey
AFAP1L1	-0.11047	-0.14839	-0.03538	0.12792	-0.10526	0.00233	0.00621	0.39897	-0.39897	0.15034	0.05275	0.64592	0.09545	0.17065	0.97586	0.93571	6.48E-08	6.48E-08	blue
UTP4	0.00408	-0.00144	0.05264	0.00203	-0.15097	0.14564	0.19862	-0.15271	0.15271	0.95775	0.98507	0.49408	0.97902	0.04872	0.05735	0.00921	0.04614	0.04614	turquoise
RYR1	0.0202	-0.12764	-0.00509	0.09104	-0.05513	0.02448	0.14475	0.08025	-0.08025	0.79317	0.09619	0.9473	0.2363	0.47392	0.7506	0.05891	0.29675	0.29675	grey
UMPS	-0.03582	0.09217	-0.14617	-0.08271	-0.08331	0.02686	0.19292	-0.1554	0.1554	0.64186	0.23053	0.05644	0.28217	0.27866	0.72727	0.01147	0.0424	0.0424	turquoise
ZNF627	-0.07333	-0.19188	-0.16053	-0.00587	-0.01497	-0.04363	-0.13074	0.21724	-0.21724	0.3405	0.01193	0.03596	0.93927	0.84593	0.57098	0.08829	0.00432	0.00432	turquoise
ZBED8	0.01325	-0.27671	-0.23861	-0.09104	0.12451	-0.28037	-0.08922	0.1658	-0.1658	0.86339	0.00025	0.00167	0.23632	0.10469	0.0002	0.24587	0.03022	0.03022	turquoise
KANK1	-0.00295	-0.18181	-0.05721	0.06198	-0.02124	-0.03063	-0.09409	0.36283	-0.36283	0.96947	0.01732	0.45737	0.42061	0.7827	0.69086	0.22092	1.08E-06	1.08E-06	turquoise
BEST4	0.01655	-0.01564	-0.04335	0.01453	0.03827	0.04137	0.09435	-0.3868	0.3868	0.82986	0.83914	0.57348	0.8504	0.61922	0.59108	0.21964	1.73E-07	1.73E-07	grey
CDKN3	0.00243	0.05541	0.04416	0.04645	-0.03942	0.02373	0.26086	-0.37382	0.37382	0.97486	0.47164	0.56632	0.54631	0.60875	0.75802	0.00057	4.75E-07	4.75E-07	pink
SPN	-0.11642	-0.06812	0.04966	0.05268	-0.19093	0.14323	0.01266	0.401	-0.401	0.12942	0.376	0.5189	0.49378	0.01237	0.06163	0.86948	5.48E-08	5.48E-08	blue
TNFRSF10	-0.07598	-0.01379	0.15198	0.05646	-0.06999	0.23765	0.23441	-0.28729	0.28729	0.32331	0.85798	0.04722	0.46324	0.36298	0.00175	0.00203	0.00014	0.00014	yellow
BTN2A2	-0.14353	-0.00943	0.06577	0.05997	-0.1361	0.17031	0.01936	0.21678	-0.21678	0.06108	0.90262	0.3927	0.43586	0.07592	0.02594	0.80159	0.0044	0.0044	blue
DNASE1L1	-0.1045	-0.0994	0.01698	-0.06966	0.00096	0.09411	0.0361	-0.11611	0.11611	0.17376	0.19585	0.82556	0.36526	0.99005	0.22082	0.63928	0.13044	0.13044	turquoise
MOSPD2	-0.0528	-0.19084	0.01011	0.06718	-0.1708	0.07258	0.08053	0.23066	-0.23066	0.49283	0.01241	0.89555	0.38262	0.02551	0.34546	0.29511	0.0024	0.0024	turquoise
BAIAP2L2	-0.02254	0.1519	-0.06827	-0.03581	0.288	0.10041	0.05047	-0.36346	0.36346	0.76977	0.04734	0.37497	0.64195	0.00013	0.19132	0.51209	1.03E-06	1.03E-06	yellow
PKP4	-0.01439	-0.05241	0.00361	0.06887	-0.07168	0.13744	0.14577	-0.19137	0.19137	0.85186	0.49602	0.96267	0.37075	0.35152	0.07303	0.05712	0.01216	0.01216	turquoise
SDHAF4	-0.0529	-0.02411	-0.06854	-0.05558	0.1086	-0.14765	-0.04356	0.01795	-0.01795	0.49199	0.75429	0.37305	0.4703	0.15739	0.05395	0.57163	0.81571	0.81571	grey
KAT7	-0.01917	-0.16783	-0.14453	-0.05394	-0.01085	-0.10737	-0.12532	0.20371	-0.20371	0.80347	0.02822	0.05929	0.48352	0.88799	0.16216	0.10242	0.00753	0.00753	turquoise
MAF	-0.03607	-0.11122	0.0474	0.09124	-0.06125	0.04795	0.03039	0.27748	-0.27748	0.63956	0.14757	0.53812	0.23527	0.42616	0.53339	0.69315	0.00024	0.00024	blue
USP28	-0.04747	-0.12221	0.03061	0.07342	-0.19276	0.12809	0.15416	0.0798	-0.0798	0.53752	0.11131	0.69102	0.33992	0.01154	0.09501	0.0441	0.29949	0.29949	turquoise
ACMSD	-0.00575	-0.1462	-0.07629	0.02334	0.22165	-0.27003	-0.14016	0.12048	-0.12048	0.9405	0.05638	0.32134	0.76192	0.00357	0.00035	0.06749	0.11649	0.11649	grey
CILP	-0.03578	-0.03487	0.01004	0.09874	-0.18243	0.04005	0.08816	0.50968	-0.50968	0.6422	0.65066	0.8963	0.19885	0.01693	0.60299	0.25154	1.08E-12	1.08E-12	blue
PARP3	-0.04066	0.02915	0.06152	0.00036	-0.04853	0.21311	0.14945	-0.30551	0.30551	0.59748	0.70508	0.42407	0.99628	0.5285	0.00513	0.05107	4.84E-05	4.84E-05	turquoise
LPXN	-0.16694	-0.06791	0.06975	0.04324	-0.1537	0.12602	0.00794	0.26043	-0.26043	0.02909	0.3775	0.36469	0.57442	0.04474	0.1005	0.91791	0.00058	0.00058	blue
HIST1H2B	0.06314	0.00201	0.12192	0.05457	-0.02374	0.10315	0.08006	-0.20136	0.20136	0.41199	0.97914	0.11216	0.47838	0.75794	0.17943	0.29792	0.00827	0.00827	grey
PDZD11	-0.03456	-0.14371	0.00303	-0.02619	-0.01373	-0.00014	0.12055	-0.36852	0.36852	0.65361	0.06077	0.96864	0.73382	0.85858	0.99855	0.11629	7.07E-07	7.07E-07	grey
DDX20	-0.17077	-0.05715	-0.00263	0.03267	-0.16215	0.05114	0.00197	0.2598	-0.2598	0.02554	0.45784	0.97279	0.67142	0.0341	0.50648	0.97956	0.0006	0.0006	turquoise
CHAF1A	-0.02534	0.16543	-0.0846	-0.09274	-0.0156	-0.01357	0.24562	-0.31404	0.31404	0.74215	0.0306	0.27126	0.22762	0.83952	0.86017	0.0012	2.88E-05	2.88E-05	pink
SLC27A5	0.06639	-0.01238	-0.28919	-0.16852	0.22922	-0.24861	-0.21471	0.02012	-0.02012	0.38828	0.8723	0.00012	0.02757	0.00256	0.00104	0.0048	0.79392	0.79392	grey
UTP25	-0.01267	-0.04863	0.02041	-0.0068	-0.18975	0.17966	0.20749	-0.11096	0.11096	0.86938	0.52761	0.79107	0.92969	0.01293	0.01871	0.00647	0.14851	0.14851	turquoise
PHF6	-0.0196	-0.1121	0.00494	0.0207	-0.14009	0.10016	0.08741	0.02203	-0.02203	0.79911	0.14437	0.94888	0.78816	0.06762	0.19243	0.25558	0.7749	0.7749	turquoise
ELL3	-0.00995	-0.00455	0.0916	-0.06801	0.09736	0.05309	-0.05902	-0.3258	0.3258	0.89727	0.95291	0.23344	0.37677	0.2052	0.4904	0.44319	1.37E-05	1.37E-05	grey
VNN1	0.05645	-0.01152	0.02785	0.03747	0.13041	0.16863	0.05219	-0.19174	0.19174	0.46334	0.88112	0.71764	0.62655	0.08912	0.02747	0.4978	0.01199	0.01199	grey
ZSCAN31	-0.09281	-0.04499	-0.03789	-0.06843	0.0024	-0.02597	-0.08444	0.20947	-0.20947	0.22727	0.55904	0.62273	0.37381	0.97517	0.736	0.27215	0.00597	0.00597	grey
THAP8	0.04536	-0.13757	-0.1272	0.00557	0.14183	-0.12143	-0.03292	0.13854	-0.13854	0.55576	0.07277	0.09734	0.94233	0.06425	0.11362	0.66903	0.07075	0.07075	grey
HARS2	0.01476	-0.09906	-0.03986	-0.06218	0.05315	0.00315	0.03684	-0.16151	0.16151	0.84806	0.19738	0.60469	0.41913	0.48996	0.96734	0.63236	0.03483	0.03483	turquoise
OLFML3	-0.10349	0.00707	-0.02578	0.03561	-0.19919	0.08212	0.08674	0.47549	-0.47549	0.17796	0.92689	0.73784	0.64377	0.009	0.28564	0.25926	4.97E-11	4.97E-11	blue
KPTN	-0.06145	0.01057	-0.17017	-0.03021	0.15893	-0.26845	-0.0673	-0.01022	0.01022	0.4246	0.89086	0.02607	0.69485	0.03787	0.00039	0.38177	0.89445	0.89445	grey
PRTFDC1	-0.06467	-0.09257	-0.01575	-0.01146	-0.06557	-0.05653	0.06178	0.03152	-0.03152	0.40071	0.22848	0.83795	0.88171	0.39421	0.46274	0.42213	0.68231	0.68231	turquoise
C9orf72	0.05003	-0.08681	-0.05361	-0.05351	-0.01856	-0.00761	#######	0.22469	-0.22469	0.51579	0.25888	0.48617	0.48699	0.8096	0.92135	0.99975	0.00313	0.00313	turquoise
PACSIN3	-0.00333	0.06865	0.119	0.15897	-0.15547	0.17121	0.27707	-0.19745	0.19745	0.96556	0.37227	0.12108	0.03782	0.04231	0.02516	0.00024	0.00964	0.00964	grey
NBEAL2	-0.00595	0.00377	0.01553	-0.05821	0.14342	0.08888	0.01905	-0.25347	0.25347	0.93839	0.96101	0.84018	0.44947	0.06129	0.24765	0.80466	0.00082	0.00082	red
CD14	-0.1307	0.05143	0.02589	0.04457	-0.15769	0.05892	0.11706	0.38635	-0.38635	0.08841	0.50415	0.73679	0.56266	0.03941	0.44402	0.12731	1.80E-07	1.80E-07	blue
DNAJC16	-0.02453	-0.19117	-0.0314	0.05114	-0.05822	0.08034	-0.03356	0.20993	-0.20993	0.75014	0.01226	0.68351	0.50652	0.44938	0.29623	0.66301	0.00585	0.00585	turquoise
LURAP1	-0.11221	-0.18557	-0.04991	0.00388	-0.08772	0.07151	-0.00249	0.37764	-0.37764	0.14398	0.0151	0.51677	0.95985	0.25395	0.35268	0.97423	3.55E-07	3.55E-07	blue
BLK	-0.06931	0.0153	0.04876	-0.00133	-0.07419	0.01707	-0.0951	0.30715	-0.30715	0.36771	0.8426	0.5265	0.98621	0.33489	0.82464	0.21598	4.38E-05	4.38E-05	blue
IMP4	0.09715	0.10568	-0.00298	-0.01257	-0.06238	0.10634	0.20919	-0.27947	0.27947	0.20619	0.16891	0.96913	0.87034	0.41762	0.16627	0.00603	0.00021	0.00021	greenyellow
ATP6V0D2	-0.1088	-0.01029	0.15464	0.14894	-0.08049	0.10405	0.09586	0.22337	-0.22337	0.15664	0.89369	0.04344	0.05186	0.29533	0.17562	0.2123	0.00332	0.00332	blue
ABHD14B	0.03948	0.13686	-0.06243	-0.14543	0.1285	0.09896	0.01189	-0.24046	0.24046	0.60813	0.07428	0.41727	0.05771	0.09394	0.19786	0.87733	0.00154	0.00154	grey
C14orf28	-0.01243	-0.12772	-0.04741	0.11382	0.01593	-0.06251	-0.03913	0.04978	-0.04978	0.87183	0.09597	0.53804	0.13825	0.83618	0.41663	0.61135	0.51788	0.51788	red
VGLL3	-0.07429	-0.09979	0.07783	0.12185	-0.24852	0.15429	0.1041	0.36387	-0.36387	0.33419	0.19409	0.31159	0.11238	0.00105	0.04391	0.1754	9.98E-07	9.98E-07	blue
SLC15A4	-0.09021	-0.12608	0.05163	0.03267	-0.1437	0.07274	-0.04976	0.06457	-0.06457	0.24063	0.10034	0.50242	0.67143	0.06079	0.34444	0.51811	0.40147	0.40147	turquoise
TVP23C	0.03909	-0.11702	-0.15265	-0.10182	0.21814	-0.24585	-0.08856	0.05812	-0.05812	0.61171	0.12744	0.04624	0.18512	0.00415	0.00119	0.24938	0.45018	0.45018	grey
SERPINE1	-0.06839	-0.15883	0.02652	0.12797	-0.18987	0.12455	0.02867	0.2231	-0.2231	0.37411	0.03799	0.73066	0.09532	0.01287	0.10456	0.70971	0.00336	0.00336	black
SLC25A17	-0.1408	-0.10572	-0.18493	-0.02368	0.01487	-0.1733	-0.02943	0.13667	-0.13667	0.06622	0.16875	0.01546	0.75851	0.84691	0.0234	0.70241	0.07468	0.07468	turquoise
SEC23B	0.02645	-0.03292	-0.10623	-0.04032	0.05182	-0.17939	-0.05932	0.20942	-0.20942	0.73128	0.66909	0.16671	0.60058	0.50086	0.01889	0.44087	0.00598	0.00598	turquoise
RBX1	-0.07122	0.06854	-0.12027	-0.01809	0.02262	-0.17776	0.03401	-0.11885	0.11885	0.35459	0.37305	0.11714	0.81433	0.76904	0.02002	0.6588	0.12156	0.12156	grey
PELI2	-0.06074	-0.17103	0.02162	0.04047	-0.11772	0.18137	-0.13399	0.18786	-0.18786	0.42999	0.02531	0.77896	0.59921	0.12517	0.0176	0.0806	0.01387	0.01387	grey
PSMC5	0.00959	-0.12956	-0.08679	-0.10529	0.02002	-0.17856	0.05952	-0.07021	0.07021	0.90092	0.09124	0.25904	0.17053	0.79494	0.01946	0.43931	0.3615	0.3615	grey
ZNF215	-0.05083	0.00107	-0.07125	-0.08232	0.08362	0.06255	-0.03708	-0.17057	0.17057	0.50906	0.98889	0.35439	0.28445	0.27688	0.4164	0.63019	0.02571	0.02571	grey
AKAP12	-0.08819	-0.13443	0.0418	0.08374	-0.22471	0.1142	-0.06803	0.34958	-0.34958	0.2514	0.07961	0.58727	0.27616	0.00313	0.13695	0.37665	2.78E-06	2.78E-06	blue
GPN2	-0.03357	-0.21385	-0.07811	-0.01332	0.06837	0.0074	-0.05757	0.08137	-0.08137	0.66291	0.00498	0.30989	0.86267	0.37426	0.9235	0.45451	0.29006	0.29006	grey
RPL13A	0.15819	0.1713	-0.0086	-0.06978	-0.04901	0.07468	0.06203	-0.14134	0.14134	0.03879	0.02508	0.91108	0.36447	0.52443	0.33164	0.42023	0.06519	0.06519	purple
AXIN2	-0.0431	0.02107	0.02804	0.02462	-0.02685	0.03155	-0.00478	0.20709	-0.20709	0.57562	0.7844	0.71577	0.74926	0.72738	0.68205	0.95055	0.00657	0.00657	grey
CADPS	0.05011	-0.07829	-0.20437	-0.1556	0.10648	-0.20254	-0.2423	0.38361	-0.38361	0.51516	0.30873	0.00733	0.04213	0.16572	0.00789	0.00141	2.23E-07	2.23E-07	brown
PSD3	-0.0166	-0.09559	0.12083	0.03094	-0.22429	0.16206	0.11902	0.0475	-0.0475	0.82935	0.21359	0.11544	0.68791	0.00319	0.0342	0.12101	0.5373	0.5373	turquoise
FBXO4	-0.13174	0.02451	-0.00656	-0.00755	-0.15878	0.00189	0.09338	0.01723	-0.01723	0.08587	0.7503	0.93214	0.9219	0.03805	0.98044	0.22443	0.82305	0.82305	grey
IST1	0.02784	-0.12916	-0.01455	-0.02801	-0.05067	0.09091	0.06279	-0.12108	0.12108	0.71775	0.09224	0.85022	0.71615	0.51044	0.23699	0.41457	0.11468	0.11468	turquoise
ZNF740	0.00097	-0.14967	-0.17503	-0.04398	0.015	-0.17836	-0.13105	0.25975	-0.25975	0.98993	0.05072	0.02203	0.56791	0.84561	0.01959	0.08754	0.0006	0.0006	turquoise
CENPU	-0.11842	0.1173	0.14692	0.02872	0.02437	0.12844	0.23927	-0.41951	0.41951	0.12292	0.12653	0.05516	0.70926	0.75168	0.09409	0.00162	1.12E-08	1.12E-08	pink
ZCCHC4	-0.02155	-0.07485	-0.03503	-0.00724	-0.01671	-0.01202	0.04199	0.01305	-0.01305	0.77964	0.33057	0.64919	0.92513	0.82827	0.87604	0.58554	0.86547	0.86547	turquoise
GSN	-0.01558	-0.08715	0.04602	0.04511	-0.04672	0.17338	0.01149	0.03642	-0.03642	0.83969	0.25702	0.55006	0.55798	0.54403	0.02334	0.88147	0.63629	0.63629	grey
DHX29	-0.04938	-0.08954	-0.08133	-0.04048	-0.05936	-0.0545	-0.00693	0.23682	-0.23682	0.52129	0.24416	0.29032	0.59911	0.44057	0.47897	0.92836	0.00182	0.00182	turquoise
CPXM2	-0.1085	-0.08776	0.03024	0.05397	-0.16241	0.23137	0.02667	0.35949	-0.35949	0.15779	0.2537	0.69459	0.48322	0.03381	0.00233	0.72911	1.37E-06	1.37E-06	blue
TMX4	-0.05201	-0.14297	-0.16239	-0.05457	-0.01791	-0.23831	-0.24445	0.51532	-0.51532	0.4993	0.06211	0.03383	0.47838	0.81614	0.0017	0.00127	5.52E-13	5.52E-13	brown
BCO2	0.05905	-0.2154	-0.15952	-0.04941	0.04263	-0.01339	0.01041	0.06977	-0.06977	0.44301	0.00466	0.03715	0.521	0.57981	0.86196	0.89255	0.3645	0.3645	grey
ABCE1	-0.15015	-0.05037	0.02061	0.02695	-0.12166	0.10172	0.06198	0.05801	-0.05801	0.04998	0.51298	0.78907	0.7264	0.11293	0.18554	0.42065	0.45106	0.45106	turquoise
LENG8	0.00819	-0.06326	-0.05727	-0.07622	0.17179	-0.02652	-0.03241	-0.13916	0.13916	0.91537	0.41107	0.45685	0.32179	0.02466	0.7306	0.67393	0.06949	0.06949	red
MARK2	0.06854	-0.13972	-0.02035	-0.01235	-0.01239	0.04096	0.12487	-0.25064	0.25064	0.37308	0.06835	0.7916	0.87263	0.87222	0.59482	0.10368	0.00094	0.00094	turquoise
PIANP	0.11908	-0.11923	-0.13949	-0.11946	-0.01902	-0.05635	-0.0527	0.26003	-0.26003	0.12085	0.12037	0.06882	0.11965	0.80494	0.46414	0.49363	0.00059	0.00059	grey
CELF3	0.00699	-0.10826	-0.27383	-0.17919	0.12015	-0.3008	-0.22189	0.39697	-0.39697	0.92769	0.15871	0.00029	0.01903	0.11751	6.40E-05	0.00354	7.63E-08	7.63E-08	brown
RBPJ	-0.00783	-0.13336	0.01285	0.01942	-0.14015	0.01834	0.01401	0.21675	-0.21675	0.91909	0.08206	0.86755	0.801	0.06751	0.81181	0.85566	0.00441	0.00441	turquoise
FCER1A	-0.08315	-0.1548	-0.02779	-0.00718	-0.12391	0.13715	-0.01939	0.39312	-0.39312	0.27961	0.04321	0.7182	0.92578	0.10638	0.07365	0.80121	1.05E-07	1.05E-07	blue
RAB9B	-0.05551	-0.12613	-0.13649	-0.00959	0.08794	-0.21564	-0.18425	0.52266	-0.52266	0.47082	0.1002	0.07506	0.90091	0.25272	0.00462	0.01585	2.26E-13	2.26E-13	brown
RRNAD1	0.08046	-0.04933	-0.1442	-0.14315	0.2605	-0.07538	0.04029	-0.33791	0.33791	0.2955	0.52173	0.05988	0.06179	0.00058	0.32715	0.6008	6.18E-06	6.18E-06	grey
C11orf86	-0.03141	-0.01643	0.1307	0.11805	0.04846	0.17205	0.15931	-0.4037	0.4037	0.68337	0.83114	0.08841	0.12411	0.52909	0.02444	0.0374	4.37E-08	4.37E-08	grey
RPL18A	0.13927	0.1538	-0.02748	-0.03534	-0.01373	0.03692	0.09516	-0.26809	0.26809	0.06926	0.04459	0.72123	0.64633	0.8585	0.63169	0.2157	0.00039	0.00039	purple
TNFRSF19	-0.10489	-0.22936	-0.14575	0.09536	-0.09544	-0.14425	-0.05346	0.44753	-0.44753	0.17213	0.00255	0.05715	0.21474	0.21435	0.05979	0.48737	8.41E-10	8.41E-10	blue
GIT2	-0.03988	-0.12526	0.04219	0.04086	-0.16931	0.08635	-0.00399	0.44617	-0.44617	0.6045	0.10261	0.58373	0.59567	0.02684	0.26143	0.95869	9.59E-10	9.59E-10	turquoise
NLRP3	-0.15749	-0.04148	0.03217	0.06185	-0.19211	0.1363	0.06516	0.47598	-0.47598	0.03966	0.59013	0.67614	0.42161	0.01183	0.07548	0.39717	4.72E-11	4.72E-11	blue
TRPC4	-0.03971	-0.15163	-0.04896	0.01961	-0.02471	-0.14324	-0.17742	0.43214	-0.43214	0.60611	0.04774	0.52486	0.79908	0.74838	0.06163	0.02026	3.59E-09	3.59E-09	turquoise
AOX1	-0.07398	-0.04172	0.00644	0.06316	-0.14288	-0.0275	-0.17662	0.59286	-0.59286	0.33625	0.58799	0.93342	0.4118	0.06229	0.72105	0.02084	1.31E-17	1.31E-17	blue
DUSP8	0.04864	-0.10697	-0.14417	-0.15689	0.30611	-0.18716	-0.28245	0.01293	-0.01293	0.52755	0.16377	0.05993	0.04044	4.67E-05	0.01424	0.00018	0.86672	0.86672	grey
ACPP	-0.04129	-0.1099	-0.1335	-0.08471	0.08186	-0.02559	0.08848	0.09146	-0.09146	0.59186	0.15247	0.08172	0.27063	0.28716	0.73968	0.24981	0.23418	0.23418	grey
VPS29	-0.02261	-0.0762	0.04528	-0.013	0.07284	-0.06676	0.01808	-0.12748	0.12748	0.76914	0.32192	0.55649	0.86596	0.34373	0.38564	0.81439	0.0966	0.0966	turquoise
PTPN7	-0.09238	-0.00329	0.07336	0.04999	-0.14624	0.14749	0.03808	0.29833	-0.29833	0.22948	0.96597	0.34031	0.51617	0.05632	0.05422	0.62098	7.40E-05	7.40E-05	blue
TBCD	-0.07153	-0.07775	-0.03862	-0.01821	0.05795	-0.04044	0.07346	0.00133	-0.00133	0.35255	0.31214	0.61601	0.81312	0.45156	0.59948	0.33966	0.98623	0.98623	grey
TTC7B	-0.10654	-0.13687	-0.08365	0.0365	-0.12675	-0.20532	-0.15517	0.61924	-0.61924	0.16546	0.07424	0.27669	0.63557	0.09853	0.00706	0.04271	1.75E-19	1.75E-19	brown
EPS8L1	0.11241	0.08689	0.09333	0.02981	0.04819	0.23072	0.22639	-0.47242	0.47242	0.14325	0.25844	0.22469	0.69868	0.53134	0.0024	0.00291	6.87E-11	6.87E-11	yellow
TLCD3A	-0.05051	-0.04358	-0.06148	-0.11035	0.15808	-0.00725	0.09188	-0.09348	0.09348	0.51176	0.57141	0.42439	0.15076	0.03892	0.925	0.23199	0.22395	0.22395	grey
KCNMB2	-0.01617	-0.10381	-0.27831	-0.17938	0.08285	-0.30465	-0.2081	0.44296	-0.44296	0.83377	0.17665	0.00023	0.0189	0.28132	5.09E-05	0.00631	1.30E-09	1.30E-09	brown
PLA2R1	0.06307	-0.10857	0.13224	0.07485	-0.13319	0.21954	0.1694	-0.13902	0.13902	0.41248	0.1575	0.08468	0.33054	0.08245	0.00391	0.02676	0.06977	0.06977	turquoise
CDT1	0.05033	0.1565	-0.00568	-0.08802	0.07401	-0.03271	0.27069	-0.52643	0.52643	0.51328	0.04095	0.94118	0.25229	0.33602	0.67109	0.00034	1.41E-13	1.41E-13	pink
ARID4B	-0.02367	-0.1555	-0.06059	-0.03151	-0.08286	0.05217	0.01372	0.17309	-0.17309	0.7586	0.04226	0.43117	0.68241	0.28131	0.49797	0.85868	0.02358	0.02358	turquoise
TRIM37	-0.08236	-0.18653	-0.15776	-0.07774	-0.04259	-0.17255	-0.07253	0.34101	-0.34101	0.28419	0.01458	0.03932	0.31218	0.58021	0.02403	0.34584	5.01E-06	5.01E-06	turquoise
AVPR2	-0.04562	-0.05753	0.00352	0.04451	0.04042	-0.05946	-0.05057	0.1966	-0.1966	0.5535	0.45485	0.96354	0.5632	0.59968	0.4398	0.51128	0.00996	0.00996	grey
TOR3A	-0.00436	-0.03584	-0.0808	-0.07895	0.02872	-0.03972	0.04396	-0.03056	0.03056	0.95484	0.64165	0.29348	0.30467	0.70924	0.60602	0.56807	0.6915	0.6915	turquoise
CASQ2	-0.13784	0.00785	0.00522	0.07276	0.02776	0.04287	-0.04413	0.29228	-0.29228	0.07219	0.91883	0.94597	0.34429	0.71855	0.5777	0.56654	0.0001	0.0001	salmon
TDP1	-0.1014	-0.06631	0.03426	0.05347	-0.12451	0.06597	0.14131	0.02141	-0.02141	0.18697	0.38887	0.65639	0.48731	0.10468	0.39131	0.06525	0.78102	0.78102	turquoise
B3GALT5	0.12436	-0.04922	0.0044	0.00477	0.21516	0.06168	0.02143	-0.21695	0.21695	0.10511	0.52262	0.95443	0.95066	0.00471	0.42288	0.78088	0.00437	0.00437	yellow
PTTG1	0.01696	0.01239	0.01546	-0.07438	-0.01174	0.00704	0.25548	-0.50923	0.50923	0.82576	0.87218	0.84095	0.33365	0.8789	0.92715	0.00074	1.14E-12	1.14E-12	pink
2-Mar	-0.08343	-0.0108	-0.02492	0.02689	-0.07941	0.01516	-0.02854	0.14908	-0.14908	0.27798	0.88848	0.74625	0.72703	0.30186	0.84398	0.71094	0.05165	0.05165	grey
KHSRP	0.05051	-0.05635	-0.04955	-0.03163	0.04841	-0.055	0.04992	-0.07505	0.07505	0.51174	0.46412	0.51983	0.68127	0.52949	0.47493	0.51668	0.32926	0.32926	turquoise
MARVELD	0.09944	-0.0674	-0.11165	-0.05282	0.11352	-0.05685	-0.05589	-0.21482	0.21482	0.19568	0.38105	0.14597	0.4926	0.1393	0.46016	0.46779	0.00478	0.00478	grey
VPS37C	0.05746	-0.03801	0.0946	0.02033	-0.12301	0.1444	0.08423	-0.10254	0.10254	0.45537	0.62156	0.21844	0.79187	0.10897	0.05953	0.27339	0.18201	0.18201	turquoise
ESYT2	-0.03933	-0.16234	0.1018	0.07677	-0.12142	0.08177	0.08644	-0.01638	0.01638	0.60958	0.03388	0.1852	0.31828	0.11365	0.2877	0.26093	0.83166	0.83166	turquoise
ZNF572	-0.04961	-0.03294	-0.09716	-0.07991	0.00247	-0.10628	0.06318	-0.08003	0.08003	0.5193	0.6689	0.20617	0.29883	0.97441	0.1665	0.41164	0.29813	0.29813	grey
CARNMT1	-0.03707	-0.02133	-0.0521	0.01427	-0.10454	0.00986	0.01001	0.12781	-0.12781	0.63028	0.78187	0.49857	0.85299	0.17357	0.89811	0.89662	0.09573	0.09573	turquoise
RANBP2	0.01029	-0.06512	-0.02469	0.00752	-0.10561	0.06346	-0.01354	0.25159	-0.25159	0.89379	0.39746	0.7486	0.92229	0.16921	0.40961	0.86048	0.0009	0.0009	turquoise

DHRS1	-0.05175	-0.01919	0.11794	0.08578	0.0897	0.0601	0.04899	-0.36217	0.36217	0.50146	0.80325	0.12446	0.26463	0.24331	0.4349	0.52461	1.13E-06	1.13E-06	grey
CFAP221	-0.03385	-0.07773	-0.10055	0.02582	0.06307	0.08905	-0.12055	0.13829	-0.13829	0.66028	0.31224	0.1907	0.73746	0.41253	0.24674	0.11628	0.07126	0.07126	magenta
SMARCAL	0.02654	-0.04547	-0.03956	0.07406	-0.07331	0.08068	0.10965	0.02005	-0.02005	0.73042	0.55479	0.60743	0.33568	0.34065	0.29415	0.1534	0.79468	0.79468	turquoise
CCDC85C	0.02764	-0.08479	-0.09712	0.01929	0.05228	-0.08289	0.0039	-0.16463	0.16463	0.71974	0.27019	0.20634	0.80228	0.49706	0.28111	0.9596	0.03142	0.03142	grey
IDS	-0.04212	-0.25731	-0.1187	-0.05482	-0.06774	-0.16399	-0.20857	0.33436	-0.33436	0.58435	0.00068	0.12204	0.47638	0.37869	0.0321	0.00619	7.84E-06	7.84E-06	turquoise
ZNF43	-0.08365	-0.1076	-0.04662	0.09676	-0.15139	-0.04222	-0.10783	0.46612	-0.46612	0.27673	0.16129	0.54488	0.20805	0.04809	0.58346	0.16039	1.32E-10	1.32E-10	blue
BCO1	0.08301	-0.06656	-0.08954	-0.03128	0.14881	-0.03869	0.04556	-0.24687	0.24687	0.28042	0.38708	0.24419	0.6846	0.05208	0.61542	0.55402	0.00113	0.00113	grey
ARHGAP1	-0.04895	0.0128	0.01019	0.01999	-0.12637	0.04996	0.2932	-0.27429	0.27429	0.52491	0.86803	0.89481	0.79528	0.09956	0.51643	9.95E-05	0.00028	0.00028	pink
KLK14	-0.13527	0.03253	-0.00602	0.03405	-0.13182	-0.08847	-0.02236	-0.00485	0.00485	0.07773	0.67274	0.93772	0.65838	0.08569	0.24986	0.77159	0.94979	0.94979	grey
RALGAPA	0.02503	-0.14964	-0.03655	-0.01009	0.07734	0.09016	-0.04427	0.0214	-0.0214	0.7452	0.05077	0.63504	0.89579	0.31467	0.24089	0.56534	0.78114	0.78114	turquoise
DNTTIP2	-0.06904	-0.08989	0.04284	0.00976	-0.12029	0.15208	0.10101	-0.05621	0.05621	0.36957	0.24232	0.57798	0.89921	0.11707	0.04707	0.18864	0.46524	0.46524	turquoise
EBF1	-0.15599	-0.09454	-0.02805	0.04106	-0.10647	-0.01846	-0.11019	0.61398	-0.61398	0.04161	0.21871	0.71567	0.59387	0.16576	0.81058	0.15137	4.28E-19	4.28E-19	blue
GDPD1	0.05376	-0.1815	-0.14628	-0.05907	0.2026	-0.24256	-0.13363	0.14644	-0.14644	0.48492	0.01751	0.05624	0.44285	0.00787	0.00139	0.08142	0.05598	0.05598	turquoise
GPC4	0.0201	-0.12587	0.0675	0.08672	-0.25151	0.26894	0.19109	-0.1052	0.1052	0.7941	0.10091	0.38038	0.25938	0.00091	0.00038	0.01229	0.17087	0.17087	turquoise
PRAG1	-0.00786	-0.06291	0.07356	0.0482	-0.06129	0.22317	0.13728	-0.16526	0.16526	0.91873	0.41371	0.339	0.53126	0.42581	0.00335	0.07338	0.03076	0.03076	turquoise
OSBPL9	-0.11334	-0.12645	0.00329	0.03466	-0.10621	0.12888	-0.01268	0.13546	-0.13546	0.13994	0.09934	0.96598	0.65267	0.16677	0.09295	0.86926	0.07731	0.07731	turquoise
NFE2L2	-0.04867	-0.14661	0.03319	0.11514	-0.1477	0.20308	0.07645	0.07836	-0.07836	0.5273	0.0557	0.66651	0.13373	0.05387	0.00772	0.32029	0.30834	0.30834	turquoise
CD79A	-0.10388	0.05364	0.01846	-0.02567	-0.09297	0.04159	-0.00734	0.30572	-0.30572	0.17634	0.4859	0.81058	0.73891	0.22647	0.58912	0.92411	4.78E-05	4.78E-05	blue
HENMT1	-0.00563	0.0198	0.17207	0.12135	-0.10259	0.22755	0.26785	-0.13291	0.13291	0.94173	0.79714	0.02442	0.11386	0.18179	0.00276	0.0004	0.0831	0.0831	grey
CCDC106	0.00397	-0.13073	-0.13749	-0.0615	0.07499	-0.19833	-0.0861	0.15469	-0.15469	0.95891	0.08834	0.07293	0.42425	0.32964	0.00931	0.26282	0.04337	0.04337	grey
CBWD2	0.01596	-0.05739	0.01199	0.01626	-0.02841	0.02921	0.08135	-0.15076	0.15076	0.83587	0.45589	0.87633	0.8328	0.71226	0.70448	0.2902	0.04905	0.04905	turquoise
MANEA	-0.08725	-0.04949	0.01854	0.02451	-0.10619	0.01406	-0.08665	0.35506	-0.35506	0.25651	0.52034	0.80983	0.75031	0.16687	0.85522	0.25976	1.89E-06	1.89E-06	turquoise
MAST4	-0.05818	-0.10544	-0.15384	-0.02876	-0.05636	-0.09052	-0.11993	0.31401	-0.31401	0.44977	0.16991	0.04454	0.70881	0.46403	0.23901	0.11819	2.89E-05	2.89E-05	turquoise
ADAMTSL	-0.0691	-0.18354	-0.15943	-0.0895	0.01593	-0.13198	-0.18239	0.39082	-0.39082	0.36917	0.0165	0.03726	0.24437	0.83619	0.08531	0.01695	1.26E-07	1.26E-07	brown
CLIC3	0.01269	0.03218	0.07573	0.03404	-0.03597	0.15625	0.21143	-0.4039	0.4039	0.86917	0.67607	0.32486	0.65845	0.64044	0.04127	0.0055	4.30E-08	4.30E-08	yellow
TOM1L1	-0.01792	-0.1281	-0.07972	-0.02398	0.10377	-0.07711	-0.06199	-0.14809	0.14809	0.81604	0.09497	0.29995	0.75551	0.17679	0.8242	0.42058	0.05324	0.05324	turquoise
DCAKD	-0.03329	-0.2566	-0.16257	-0.07871	0.10095	-0.23348	-0.19249	0.20562	-0.20562	0.66559	0.0007	0.03364	0.30618	0.1889	0.00212	0.01166	0.00697	0.00697	brown
SRSF4	-0.04568	-0.16515	0.02942	0.06091	-0.05434	0.123	0.00416	-0.03523	0.03523	0.55298	0.03088	0.70244	0.42873	0.48023	0.10901	0.95697	0.64734	0.64734	turquoise
P3H4	-0.02743	-0.07487	-0.1451	0.02816	0.02657	-0.17202	0.07108	0.0392	-0.0392	0.72178	0.33045	0.05828	0.71467	0.73009	0.02446	0.35558	0.61069	0.61069	grey
DAB2	-0.05646	-0.08823	0.04194	0.06831	-0.24509	0.16986	0.08348	0.39237	-0.39237	0.46323	0.25118	0.58602	0.37471	0.00123	0.02634	0.2777	1.11E-07	1.11E-07	blue
HMGN5	-0.11227	-0.08871	-0.10954	-0.05887	0.14642	-0.06422	-0.0763	0.30501	-0.30501	0.14376	0.24858	0.15379	0.44438	0.05602	0.40404	0.32126	4.98E-05	4.98E-05	turquoise
TTC19	-0.00614	-0.07327	-0.08062	-0.06142	0.08105	-0.16033	-0.09322	0.10681	-0.10681	0.93652	0.34092	0.29453	0.42485	0.29197	0.03619	0.22522	0.16441	0.16441	grey
PRRX1	-0.09097	-0.03377	0.07992	0.12334	-0.24402	0.12905	0.07135	0.33271	-0.33271	0.2367	0.66105	0.29875	0.10802	0.0013	0.09253	0.35375	8.74E-06	8.74E-06	black
DNAJB14	-0.09821	-0.18325	-0.05017	-0.00617	-0.03812	-0.00112	-0.05189	0.21093	-0.21093	0.20125	0.01644	0.51465	0.93616	0.62059	0.98843	0.50026	0.00562	0.00562	turquoise
STS	-0.26199	-0.11645	0.05608	0.05664	-0.17618	0.21643	0.14441	-0.04109	0.04109	0.00054	0.12932	0.46627	0.46181	0.02117	0.00447	0.0595	0.59361	0.59361	turquoise
CYP27B1	0.07411	0.11691	-0.01728	0.0349	0.02626	0.12911	0.12978	-0.24006	0.24006	0.33538	0.12779	0.82247	0.6504	0.7332	0.09236	0.09067	0.00156	0.00156	grey
ANAPC13	-0.04956	-0.02425	-0.23456	-0.0327	-0.03554	-0.07987	-0.00871	0.08888	-0.08888	0.51977	0.75286	0.00201	0.67111	0.64444	0.29905	0.91002	0.24765	0.24765	turquoise
HACE1	-0.08263	-0.12159	-0.01284	0.03183	-0.11349	-0.07727	-0.09339	0.44307	-0.44307	0.28263	0.11314	0.8676	0.67935	0.13941	0.31512	0.22439	1.29E-09	1.29E-09	turquoise
CTBP2	0.03414	-0.06377	-0.00206	0.04586	-0.00415	0.11484	0.1249	-0.27574	0.27574	0.65759	0.40735	0.97866	0.55141	0.957	0.13473	0.1036	0.00026	0.00026	turquoise
CERS1	-0.02825	-0.21627	-0.23825	-0.11018	0.09935	-0.36981	-0.18625	0.40664	-0.40664	0.7138	0.0045	0.0017	0.15142	0.19606	6.42E-07	0.01472	3.41E-08	3.41E-08	brown
CALM1	-0.04472	-0.13281	-0.10571	-0.02897	0.05597	-0.20348	-0.24665	0.28559	-0.28559	0.56138	0.08333	0.16881	0.70679	0.46718	0.0076	0.00115	0.00015	0.00015	turquoise
CD3E	-0.08509	-0.02698	0.05238	0.0886	-0.20399	0.14275	0.01192	0.36389	-0.36389	0.26849	0.72614	0.49625	0.24917	0.00745	0.06253	0.877	9.96E-07	9.96E-07	blue
KIF1BP	-0.06749	-0.22443	-0.09595	-0.02611	-0.09884	-0.1541	-0.06508	0.30808	-0.30808	0.38046	0.00317	0.21189	0.73457	0.19838	0.04418	0.3977	4.14E-05	4.14E-05	turquoise
CCNO	0.04097	0.02525	-0.05228	-0.0328	0.24037	0.03068	0.00229	-0.46837	0.46837	0.59473	0.74302	0.49711	0.67023	0.00154	0.69042	0.97633	1.05E-10	1.05E-10	grey
GPR160	0.02486	0.00269	0.11856	0.06948	0.08339	0.26344	0.02659	-0.15362	0.15362	0.74688	0.97216	0.12247	0.36654	0.2782	0.0005	0.72996	0.04486	0.04486	yellow
POMP	0.02454	0.03112	-0.07873	-0.06264	-0.04401	-0.04286	0.16901	-0.27865	0.27865	0.74999	0.68616	0.30605	0.41572	0.56761	0.57781	0.02712	0.00022	0.00022	greenyellow
PDGFB	-0.12587	-0.22013	-0.08477	0.05993	-0.11135	-0.0399	-0.09866	0.09354	-0.09354	0.10092	0.00382	0.27033	0.43618	0.14708	0.60436	0.19921	0.22366	0.22366	black
FLOT1	0.04766	-0.05188	-0.1092	-0.05679	0.01274	-0.0648	0.02533	-0.12098	0.12098	0.53587	0.50039	0.15509	0.46067	0.86863	0.39975	0.74229	0.11498	0.11498	grey
MMP13	0.03839	-0.01941	0.08291	0.13581	-0.0526	0.05047	0.21697	-0.0256	0.0256	0.6181	0.80106	0.28098	0.07654	0.49448	0.51213	0.00437	0.73965	0.73965	grey
GGA1	-0.05287	-0.00723	-0.11107	-0.07387	0.15888	-0.08202	-0.04908	-0.14955	0.14955	0.49222	0.92518	0.14811	0.33695	0.03794	0.28622	0.52384	0.0509	0.0509	red
LSM12	-0.04871	-0.0925	0.039	0.01495	-0.00882	0.11764	0.08391	-0.11241	0.11241	0.52691	0.22888	0.61256	0.84607	0.90886	0.12543	0.27519	0.14325	0.14325	turquoise
TMPRSS1	-0.02267	-0.0088	-0.03779	0.03792	-0.0419	0.03787	0.03562	-0.17944	0.17944	0.7685	0.90905	0.62359	0.62245	0.58634	0.62287	0.64371	0.01886	0.01886	grey
AIF1	-0.1059	0.00168	0.04416	0.01806	-0.19587	0.13203	0.10734	0.35616	-0.35616	0.16802	0.98262	0.56632	0.81466	0.01024	0.08518	0.16231	1.74E-06	1.74E-06	blue
TAX1BP3	0.01393	-0.02029	0.01535	0.01713	-0.11454	0.14747	0.22903	-0.20885	0.20885	0.85648	0.79225	0.84203	0.82405	0.13576	0.05424	0.00259	0.00612	0.00612	yellow
CACHD1	-0.05245	-0.17857	0.04802	0.13614	-0.20859	0.09484	0.01027	0.39241	-0.39241	0.49567	0.01945	0.53283	0.07583	0.00618	0.21723	0.89391	1.11E-07	1.11E-07	blue
TECR	0.09234	-0.04634	-0.0699	-0.07867	0.16751	-0.20196	0.03691	-0.36441	0.36441	0.22966	0.54729	0.3636	0.30644	0.02853	0.00808	0.63171	9.59E-07	9.59E-07	grey
MINK1	-0.03985	-0.10177	-0.01047	-0.08104	0.14274	-0.07493	-0.06941	0.08694	-0.08694	0.60479	0.18533	0.89185	0.292	0.06253	0.33007	0.36701	0.25816	0.25816	turquoise
ZNF792	0.00578	-0.10005	-0.02859	-0.0815	-0.10214	0.14093	0.04404	0.06114	-0.06114	0.94024	0.19291	0.71045	0.28927	0.18373	0.06598	0.56737	0.42698	0.42698	turquoise
MROH6	0.07988	0.0509	0.02256	-0.01048	0.00796	0.10345	0.25436	-0.48761	0.48761	0.29898	0.50849	0.7696	0.89179	0.91774	0.17813	0.00079	1.34E-11	1.34E-11	yellow
MIS18BP1	-0.02294	-0.05582	0.03689	0.01342	-0.12835	0.14126	0.10719	0.15332	-0.15332	0.76582	0.46832	0.63196	0.86166	0.09432	0.06534	0.16287	0.04528	0.04528	turquoise
PHTF1	-0.10447	-0.19517	-0.04015	-0.00858	-0.11933	-0.03956	-0.03032	0.24382	-0.24382	0.17388	0.01052	0.60211	0.91132	0.12005	0.60747	0.69385	0.00131	0.00131	turquoise
MTUS2	-0.05237	-0.11238	-0.16908	-0.08614	0.0966	-0.26511	-0.19558	0.41924	-0.41924	0.49632	0.14334	0.02705	0.26259	0.20879	0.00046	0.01036	1.15E-08	1.15E-08	brown
BRAF	0.02059	-0.21916	-0.03906	-0.07617	-0.07199	-0.04677	-0.07844	0.26102	-0.26102	0.78925	0.00398	0.61195	0.32206	0.34943	0.54356	0.30783	0.00056	0.00056	turquoise
NPC2	0.00882	0.00512	-0.02352	0.01081	-0.17883	0.02891	0.12426	0.04222	-0.04222	0.9088	0.947	0.76012	0.88842	0.01927	0.70739	0.10539	0.58349	0.58349	blue
HGSNAT	-0.00167	-0.18539	-0.01168	0.08667	-0.10132	0.06702	0.04835	0.17551	-0.17551	0.98269	0.0152	0.87953	0.25968	0.18731	0.3838	0.53002	0.02167	0.02167	turquoise
GAK	0.01949	0.04663	-0.07568	-0.05969	0.20932	-0.0124	0.06613	-0.31975	0.31975	0.80021	0.54475	0.32521	0.43807	0.006	0.87207	0.39011	2.02E-05	2.02E-05	grey
TCF19	-0.0757	0.09691	0.00236	-0.00536	-0.03499	0.0668	0.26414	-0.25811	0.25811	0.32509	0.20733	0.97555	0.94456	0.64956	0.38532	0.00048	0.00065	0.00065	pink
C1R	-0.08744	-0.0561	0.05246	0.10024	-0.22962	0.203	0.12702	0.28902	-0.28902	0.25547	0.46616	0.49561	0.19207	0.00252	0.00775	0.09781	0.00013	0.00013	blue
RPE	0.03236	-0.05077	0.05436	0.1127	-0.11835	0.16674	0.15659	-0.14452	0.14452	0.67434	0.50962	0.48008	0.14223	0.12314	0.02928	0.04082	0.05931	0.05931	turquoise
FRK	0.05138	-0.04978	0.01604	0.06623	-0.01676	0.10401	0.09179	-0.16498	0.16498	0.50455	0.51789	0.83506	0.38945	0.82773	0.17578	0.23245	0.03106	0.03106	turquoise
SPTB	-0.00515	-0.12283	-0.2332	-0.07607	0.08078	-0.28771	-0.22556	0.48554	-0.48554	0.94665	0.10949	0.00214	0.32269	0.2936	0.00014	0.00301	1.69E-11	1.69E-11	brown
APOA1	-0.06347	-0.07321	0.02981	-0.02075	0.04305	0.01832	0.00915	-0.0968	0.0968	0.40953	0.34129	0.69873	0.78759	0.57606	0.81201	0.90544	0.20786	0.20786	grey
ADCY7	-0.05387	-0.07836	0.07949	0.07589	-0.13015	0.06887	0.14188	0.19833	-0.19833	0.48407	0.30831	0.30139	0.32384	0.08976	0.37076	0.06416	0.00931	0.00931	turquoise
MTERF4	0.00721	-0.09114	-0.12455	-0.09127	0.08679	-0.09757	-0.11785	0.22938	-0.22938	0.92546	0.23582	0.10456	0.23512	0.25901	0.20424	0.12475	0.00255	0.00255	turquoise
ZNF497	0.06423	-0.07483	-0.27228	-0.1455	0.22502	-0.28499	-0.16382	0.14812	-0.14812	0.40391	0.33067	0.00031	0.05758	0.00309	0.00016	0.03227	0.05319	0.05319	brown
LMNTD2	0.06284	-0.08849	-0.02409	-0.05736	0.21863	-0.03695	0.03626	-0.42999	0.42999	0.41419	0.24974	0.75444	0.45619	0.00407	0.63132	0.63774	4.38E-09	4.38E-09	green
ENPP4	-0.07935	-0.14745	-0.08603	-0.02783	0.02288	-0.07263	-0.14079	0.24535	-0.24535	0.30221	0.05429	0.26325	0.71786	0.76647	0.34517	0.06625	0.00122	0.00122	turquoise
SPNS3	0.0636	0.09908	-0.06934	0.0086	0.00985	0.0977	0.01706	-0.04619	0.04619	0.40858	0.19729	0.36747	0.91108	0.89827	0.20366	0.82475	0.54854	0.54854	grey
PGD	-0.08267	-0.00106	0.04352	0.02481	-0.07609	0.08939	0.09216	0.04044	-0.04044	0.28241	0.98903	0.57192	0.74736	0.32258	0.24496	0.23059	0.59945	0.59945	turquoise
ACAN	-0.18226	0.10078	0.02924	0.11623	-0.09671	0.08117	-0.02182	0.18076	-0.18076	0.01704	0.18966	0.70419	0.13007	0.20829	0.29124	0.77692	0.01798	0.01798	grey
LTN1	-0.06483	-0.14323	-0.03384	0.02361	-0.03243	0.05306	0.00519	0.26342	-0.26342	0.39954	0.06163	0.66034	0.75923	0.67368	0.49065	0.94624	0.0005	0.0005	turquoise
RPS28	0.16004	0.15183	-0.08892	-0.06867	0.01374	-0.07982	0.07026	-0.1977	0.1977	0.03653	0.04744	0.24747	0.37218	0.8584	0.29936	0.36113	0.00954	0.00954	purple
ZNF37A	-0.09956	-0.16064	-0.07724	-0.01051	-0.05363	-0.06818	-0.10237	0.44811	-0.44811	0.19512	0.03583	0.31532	0.89146	0.48605	0.37559	0.18276	7.96E-10	7.96E-10	turquoise
GPR174	-0.06379	-0.03039	0.04669	0.06666	-0.14231	0.10367	-0.02171	0.42768	-0.42768	0.40715	0.69319	0.54428	0.38633	0.06335	0.17723	0.77805	5.40E-09	5.40E-09	blue
OXER1	-0.06028	-0.05075	-0.0932	0.08647	0.02671	0.04877	-0.07737	0.12159	-0.12159	0.43352	0.50974	0.22535	0.26079	0.72875	0.52641	0.31447	0.11314	0.11314	grey
PCSK7	-0.05617	-0.00271	0.11554	0.03817	-0.117	0.21933	0.06969	0.12522	-0.12522	0.46558	0.97195	0.13235	0.62011	0.12751	0.00395	0.36505	0.10271	0.10271	turquoise
DENND6B	0.00062	-0.01582	-0.16728	-0.08732	0.15414	-0.26212	-0.07275	-0.14714	0.14714	0.9936	0.83726	0.02875	0.25613	0.04412	0.00053	0.34436	0.0548	0.0548	red
KLHL26	0.05087	-0.1186	-0.17063	0.01968	0.08793	-0.14509	-0.08517	0.15046	-0.15046	0.50872	0.12236	0.02566	0.79833	0.2528	0.0583	0.26807	0.0495	0.0495	grey
GIN1	0.00484	-0.10505	-0.17198	-0.11088	0.06776	-0.18257	-0.10895	0.28646	-0.28646	0.94991	0.17151	0.0245	0.14879	0.37851	0.01685	0.15606	0.00015	0.00015	turquoise
VPS4B	-0.06485	-0.11546	-0.01424	0.04135	0.00984	0.06551	0.06259	0.09299	-0.09299	0.3994	0.13264	0.85329	0.59126	0.89838	0.39458	0.4161	0.22637	0.22637	turquoise
RAD51	-0.07377	0.0874	0.04266	-0.00553	-0.15238	0.07581	0.32069	-0.42809	0.42809	0.3376	0.25564	0.57959	0.94277	0.04663	0.3244	1.90E-05	5.20E-09	5.20E-09	pink
NSMAF	-0.00651	0.0102	0.02204	0.04176	-0.16691	-0.00252	0.03728	0.18782	-0.18782	0.93268	0.89468	0.77481	0.58758	0.02912	0.9739	0.62834	0.01389	0.01389	turquoise
DLK2	0.01633	-0.0507	-0.01837	0.0837	0.05389	-0.23332	0.01588	0.04485	-0.04485	0.83211	0.51019	0.81148	0.2764	0.48392	0.00213	0.83671	0.56029	0.56029	grey
GRB7	0.11899	0.01186	0.0182	0.03409	0.08179	0.10658	0.17426	-0.51858	0.51858	0.12113	0.87763	0.81327	0.65803	0.28759	0.1653	0.02263	3.72E-13	3.72E-13	yellow
FHL1	-0.0876	-0.08213	-0.08129	-0.01854	0.01037	-0.10312	-0.18223	0.57308	-0.57308	0.25459	0.28557	0.2905	0.80976	0.89296	0.17954	0.01706	2.59E-16	2.59E-16	salmon
PEX14	-0.06606	-0.06586	0.0162	0.0064	0.02054	0.05332	0.05787	-0.15049	0.15049	0.39067	0.39207	0.83342	0.93383	0.78975	0.48856	0.45218	0.04945	0.04945	grey
BOLA2B	0.10787	0.11382	-0.04978	-0.10323	0.08592	-0.12194	0.08179	-0.36214	0.36214	0.16021	0.13825	0.51788	0.17909	0.26381	0.11211	0.28757	1.13E-06	1.13E-06	grey
AGAP1	0.04056	-0.04098	-0.14171	-0.05068	-0.02256	-0.09545	-0.03229	0.13894	-0.13894	0.59836	0.5946	0.06448	0.51039	0.76959	0.21431	0.67504	0.06994	0.06994	turquoise
RPH3AL	0.03147	-0.05416	-0.22305	-0.14725	0.17271	-0.28954	-0.25314	0.21437	-0.21437	0.68287	0.48175	0.00336	0.05461	0.02389	0.00012	0.00084	0.00487	0.00487	brown
TMEM59	-0.02601	-0.17944	-0.04538	-0.04648	0.13952	-0.10145	-0.12336	0.04517	-0.04517	0.73563	0.01886	0.55565	0.54602	0.06876	0.18673	0.10794	0.55743	0.55743	turquoise
RAB18	-0.05774	-0.17469	-0.01984	0.02287	-0.02712	-0.05029	-0.08121	0.2173	-0.2173	0.45319	0.0223	0.79674	0.76652	0.72472	0.51365	0.29102	0.0043	0.0043	turquoise
MYH9	-0.07788	-0.06086	0.08898	0.08221	-0.20527	0.22648	0.0691	0.08139	-0.08139	0.31131	0.42909	0.24716	0.28509	0.00707	0.00289	0.36918	0.28996	0.28996	black
NANOS3	0.12929	-0.08519	-0.11365	-0.06955	0.01721	-0.13183	-0.0062	-0.08499	0.08499	0.09191	0.26793	0.13884	0.36604	0.82326	0.08566	0.93584	0.26906	0.26906	grey
USP31	-0.01111	-0.16298	0.01238	0.08836	-0.23429	0.04286	0.20705	0.0672	-0.0672	0.8853	0.03319	0.87232	0.25048	0.00204	0.57781	0.00659	0.38252	0.38252	turquoise
PAK5	0.00232	-0.11869	-0.27332	-0.01146	0.08384	-0.25573	-0.17595	0.30004	-0.30004	0.97593	0.12207	0.0003	0.88171	0.27559	0.00074	0.02133	6.69E-05	6.69E-05	grey
CHRDL1	-0.09637	-0.08326	-0.05548	0.06238	-0.1026	0.07617	-0.07593	0.59422	-0.59422	0.20989	0.27896	0.47106	0.41761	0.18174	0.32207	0.32363	1.06E-17	1.06E-17	blue
PAEP	0.06605	-0.00383	-0.09857	-0.05912	0.05376	-0.07324	-0.01314	-0.25863	0.25863	0.39071	0.96034	0.19961	0.44243	0.48498	0.34108	0.86456	0.00064	0.00064	grey
STPG4	0.06749	0.01372	0.05619	0.06561	-0.12975	0.04913	0.23743	-0.39625	0.39625	0.38047	0.85869	0.46543	0.39386	0.09077	0.52342	0.00177	8.10E-08	8.10E-08	grey
ZNF41	-0.03028	-0.13262	-0.04942	0.01977	-0.06439	-0.00133	-0.0179	0.23744	-0.23744	0.69424	0.08378	0.52095	0.79742	0.40278	0.98618	0.81626	0.00177	0.00177	turquoise
NOMO1	0.04551	-0.08687	0.02575	0.03314	-0.16099	-0.04568	0.02653	0.05946	-0.05946	0.5545	0.25859	0.73814	0.66702	0.03542	0.55299	0.73049	0.43978	0.43978	grey
MAZ	0.02208	-0.0293	-0.10637	-0.03658	0.07452	-0.08928	0.00532	-0.06465	0.06465	0.77443	0.70359	0.16613	0.63475	0.33271	0.24556	0.94491	0.40082	0.40082	grey
TUBD1	-0.05744	-0.21453	-0.2181	-0.10642	0.00357	-0.18502	-0.04738	0.26468	-0.26468	0.45555	0.00484	0.00416	0.16594	0.96308	0.01541	0.53831	0.00047	0.00047	turquoise
ENDOG	0.04881	-0.00994	-0.16738	-0.10039	0.14812	-0.1751	0.08898	-0.22822	0.22822	0.52614	0.89736	0.02866	0.19139	0.05318	0.02199	0.24712	0.00268	0.00268	green
FAT4	-0.09404	-0.14645	-0.00866	0.04473	-0.19332	0.1349	0.00328	0.51524	-0.51524	0.22117	0.05596	0.91044	0.56125	0.0113	0.07856	0.96604	5.58E-13	5.58E-13	blue
WWC1	-0.04106	-0.18028	-0.10415	0.10207	-0.04836	-0.01793	0.03905	-0.14217	0.14217	0.59391	0.0183	0.1752	0.18404	0.52995	0.81592	0.61211	0.0636	0.0636	grey
GPM6B	-0.14676	-0.10102	-0.12937	0.03241	-0.11256	-0.24317	-0.09538	0.5606	-0.5606	0.05544	0.18861	0.09172	0.67392	0.14269	0.00135	0.21464	1.54E-15	1.54E-15	blue
RSPH1	0.03109	-0.12801	-0.16037	-0.08147	0.34882	-0.1853	-0.11242	-0.16451	0.16451	0.6865	0.0952	0.03614	0.28945	2.93E-06	0.01525	0.14321	0.03155	0.03155	grey
SLC9A8	-0.02458	-0.0599	-0.00724	0.02475	-0.03643	-0.01912	0.05393	-0.13496	0.13496	0.74962	0.43641	0.92508	0.74794	0.63616	0.80401	0.48361	0.07843	0.07843	turquoise
SQLE	-0.03263	-0.02033	0.02461	-0.02075	-0.00429	0.12739	0.08548	-0.23226	0.23226	0.6718	0.79186	0.74934	0.7876	0.9556	0.09683	0.26628	0.00224	0.00224	grey
CHCHD5	0.12737	0.17863	-0.13397	-0.10619	0.131	-0.09824	0.08765	-0.33658	0.33658	0.09689	0.01941	0.08065	0.16687	0.08767	0.20115	0.25429	6.76E-06	6.76E-06	grey
USP3	-0.03166	-0.06014	0.03184	0.0091	-0.00516	0.15114	0.03158	-0.10054	0.10054	0.68102	0.43459	0.67934	0.90596	0.94664	0.04847	0.68175	0.19073	0.19073	turquoise
THEMIS	-0.07361	-0.03409	0.03028	0.05638	-0.1761	0.10408	-0.03458	0.45153	-0.45153	0.33869	0.65801	0.69418	0.46393	0.02123	0.1755	0.65344	5.70E-10	5.70E-10	blue
TFPT	0.05738	0.02853	-0.06112	-0.01336	0.05626	-0.04894	0.09714	-0.18699	0.18699	0.45596	0.71107	0.42709	0.86233	0.46487	0.52502	0.20626	0.01433	0.01433	grey
ZC3H12B	-0.04987	-0.20825	-0.11878	-0.01957	-0.02742	-0.11212	-0.15849	0.28931	-0.28931	0.51714	0.00627	0.12177	0.79947	0.72185	0.14427	0.03841	0.00012	0.00012	turquoise
COMMD1	0.00077	0.0266	-0.01696	0.05865	-0.11824	0.00465	0.11817	-0.00427	0.00427	0.99199	0.72985	0.82574	0.44606	0.12351	0.95186	0.12372	0.95584	0.95584	grey
WDR17	-0.06502	-0.09526	-0.25581	-0.11715	0.11	-0.26621	-0.25885	0.43132	-0.43132	0.39817	0.2152	0.00073	0.12702	0.15208	0.00043	0.00063	3.87E-09	3.87E-09	brown
TRAF5	-0.02751	0.07812	0.0786	0.05466	-0.04434	0.13972	0.06643	-0.02629	0.02629	0.72096	0.30981	0.30687	0.47763	0.56473	0.06836	0.38801	0.73283	0.73283	grey
CST2	0.08796	-0.07196	0.01991	-0.00584	-0.20876	0.22191	0.07204	0.04462	-0.04462	0.2526	0.34963	0.79605	0.93957	0.00614	0.00353	0.3491	0.56227	0.56227	black
UBLCP1	-0.07481	-0.06526	0.01062	0.02796	-0.09574	0.07454	0.00977	0.26533	-0.26533	0.3308	0.39642	0.89038	0.7166	0.21288	0.33259	0.89912	0.00045	0.00045	blue

ATXN7L1	0.02587	-0.06759	-0.00877	-0.04352	-0.01403	0.02858	-0.02546	0.08162	-0.08162	0.73697	0.37977	0.90935	0.57196	0.85553	0.71058	0.74102	0.28858	0.28858	turquoise
NDUFAF7	0.01058	-0.1034	-0.03701	-0.04801	-0.02119	0.07368	0.12542	-0.04648	0.04648	0.89073	0.17835	0.63081	0.5329	0.78327	0.33818	0.10216	0.54604	0.54604	red
NXF3	-0.01698	0.06183	0.07553	0.07905	0.13181	0.10486	0.02588	-0.14349	0.14349	0.82557	0.42178	0.3262	0.30405	0.08571	0.17226	0.73684	0.06116	0.06116	grey
FNDC4	-0.04094	-0.07655	0.00473	0.03746	-0.16428	0.10507	0.029	-0.06282	0.06282	0.59496	0.31969	0.95105	0.6267	0.03179	0.17141	0.70652	0.41435	0.41435	black
SUCO	0.0258	-0.226	-0.11977	-0.03108	0.03398	-0.08037	-0.03505	0.17506	-0.17506	0.73765	0.00296	0.11868	0.68654	0.65907	0.29607	0.64906	0.02202	0.02202	turquoise
B4GALT4	0.06137	-0.10046	-0.00884	-0.04408	0.09092	0.15108	0.11569	-0.27817	0.27817	0.42521	0.19107	0.90864	0.56703	0.23696	0.04856	0.13186	0.00023	0.00023	turquoise
CDIP1	0.023	-0.12998	-0.10308	0.01951	0.01813	-0.18884	-0.14028	0.58292	-0.58292	0.76524	0.09019	0.17972	0.80003	0.81396	0.01338	0.06724	6.03E-17	6.03E-17	brown
IGFLR1	-0.17442	0.01416	-0.01423	0.06692	-0.11359	0.00711	0.05885	0.31573	-0.31573	0.02251	0.85411	0.85343	0.38446	0.13905	0.92651	0.44449	2.59E-05	2.59E-05	blue
CSNK1D	-0.07217	-0.2447	-0.09486	-0.0613	0.08811	-0.16923	-0.07238	0.01331	-0.01331	0.34821	0.00126	0.21716	0.42574	0.25179	0.02692	0.34681	0.86286	0.86286	grey
AMH	0.18819	-0.02881	-0.19447	-0.0572	0.18484	-0.22702	-0.05326	-0.16212	0.16212	0.0137	0.70841	0.01081	0.45743	0.01551	0.00283	0.489	0.03414	0.03414	grey
NAIF1	0.10664	-0.03984	-0.09064	-0.09045	0.02313	-0.09639	-0.02177	-0.05643	0.05643	0.16507	0.60486	0.23837	0.23938	0.76396	0.20979	0.77743	0.46354	0.46354	grey
ACBD4	0.02265	-0.10565	-0.12074	-0.05852	0.32164	-0.07328	-0.06501	-0.17471	0.17471	0.76869	0.16906	0.1157	0.44707	1.79E-05	0.34082	0.39826	0.02228	0.02228	red
CCNB2	-0.00801	0.11638	0.05735	0.00953	-0.09025	0.12657	0.34265	-0.48978	0.48978	0.91718	0.12955	0.4562	0.90154	0.24041	0.09903	4.48E-06	1.06E-11	1.06E-11	pink
ADAMTS1	-0.06564	-0.03524	0.01565	0.02226	-0.16816	0.02132	0.01823	0.28201	-0.28201	0.39368	0.64724	0.83898	0.77256	0.02791	0.78198	0.8129	0.00019	0.00019	blue
ZDHHC4	-0.06451	-0.07948	-0.0559	0.01234	-0.00076	-0.07013	0.04321	-0.10783	0.10783	0.4019	0.30147	0.4677	0.87278	0.9921	0.36205	0.57473	0.16036	0.16036	grey
EHHADH	-0.00628	-0.04433	-0.07648	0.01814	0.02052	0.01704	0.09575	-0.08111	0.08111	0.93504	0.56478	0.32009	0.81388	0.78998	0.82493	0.21286	0.29162	0.29162	turquoise
USP46	-0.09816	-0.14853	-0.01957	0.02702	-0.11672	0.03955	0.08782	0.24759	-0.24759	0.2015	0.05253	0.79947	0.72569	0.12844	0.6075	0.2534	0.00109	0.00109	turquoise
RNF146	-0.04951	-0.18345	-0.07068	0.00762	-0.07054	-0.08096	-0.11445	0.44391	-0.44391	0.52018	0.01632	0.35832	0.92119	0.35927	0.2925	0.13607	1.19E-09	1.19E-09	turquoise
HHLA2	-0.02732	-0.02971	0.04099	0.07807	0.10453	0.19464	0.02573	-0.13487	0.13487	0.72281	0.69967	0.59452	0.31011	0.17364	0.01074	0.73838	0.07863	0.07863	yellow
MRPL18	0.02123	-0.07295	-0.02717	0.02225	0.02504	-0.03187	-0.01115	-0.06638	0.06638	0.78282	0.34305	0.72424	0.77272	0.74512	0.67899	0.88488	0.38833	0.38833	turquoise
GPR143	0.04389	-0.02912	-0.08135	0.08687	-0.06281	-0.1262	0.05838	-0.14509	0.14509	0.56866	0.70536	0.29016	0.25859	0.41444	0.10003	0.44814	0.0583	0.0583	grey
KCND2	-0.09738	-0.02652	0.0014	0.02066	-0.20619	0.05482	0.08954	0.33762	-0.33762	0.20513	0.73063	0.98552	0.78856	0.00682	0.47638	0.24417	6.30E-06	6.30E-06	black
KNTC1	-0.00373	-0.00669	0.12255	0.02825	-0.04824	0.09772	0.22128	-0.25976	0.25976	0.96138	0.93084	0.11029	0.71382	0.53096	0.20354	0.00363	0.0006	0.0006	pink
PIF1	8.21E-05	0.09518	0.02544	0.02408	0.09214	0.01971	0.25704	-0.32239	0.32239	0.99915	0.2156	0.74121	0.75452	0.23068	0.79801	0.00069	1.71E-05	1.71E-05	pink
ECM2	-0.0936	-0.06334	0.06188	0.12233	-0.21102	0.11279	-0.00246	0.51969	-0.51969	0.22332	0.41051	0.42136	0.11095	0.0056	0.1419	0.97455	3.25E-13	3.25E-13	blue
RAB9A	-0.15137	-0.15253	0.00225	0.1015	-0.1164	0.14414	0.1177	-0.10182	0.10182	0.04813	0.04642	0.97665	0.18651	0.12948	0.05999	0.12522	0.18514	0.18514	turquoise
NCAPH	-0.05108	0.12378	0.09832	0.03759	-0.08348	0.105	0.32925	-0.32584	0.32584	0.507	0.10676	0.20075	0.62544	0.27766	0.17168	1.10E-05	1.37E-05	1.37E-05	pink
PCIF1	-0.01168	-0.06098	-0.09402	-0.04897	-0.00496	-0.07528	-0.02676	-0.04613	0.04613	0.87945	0.42821	0.22127	0.52473	0.94862	0.32777	0.72829	0.54913	0.54913	turquoise
LMO7	0.05903	-0.0224	0.07459	-0.00799	-0.05156	0.17251	0.123	-0.34755	0.34755	0.44311	0.77123	0.33225	0.91737	0.50306	0.02406	0.10899	3.20E-06	3.20E-06	turquoise
SHPRH	-0.04746	-0.1038	-0.02094	-0.00222	-0.11378	0.06422	0.02901	0.36676	-0.36676	0.53766	0.17668	0.78577	0.97696	0.1384	0.40402	0.70642	8.06E-07	8.06E-07	turquoise
MICALL1	-0.0839	0.00591	-0.00182	0.08676	-0.20296	0.04326	0.22673	-0.08927	0.08927	0.27526	0.9389	0.98114	0.2592	0.00776	0.57424	0.00286	0.24562	0.24562	grey
RHOV	0.06701	0.065	0.12018	0.04751	-0.06642	0.16488	0.17517	-0.41927	0.41927	0.38387	0.39828	0.11742	0.53717	0.38805	0.03116	0.02193	1.15E-08	1.15E-08	yellow
WDR78	-0.07924	-0.22008	-0.13182	-0.00658	0.0278	-0.15577	-0.14241	0.31929	-0.31929	0.30291	0.00382	0.08567	0.93191	0.71812	0.0419	0.06315	2.08E-05	2.08E-05	turquoise
SNAP91	-0.00822	-0.10831	-0.2107	-0.1202	0.06884	-0.30536	-0.24227	0.53024	-0.53024	0.91498	0.1585	0.00567	0.11736	0.37094	4.88E-05	0.00141	8.76E-14	8.76E-14	brown
FGF14	-0.03409	-0.14527	-0.25319	-0.18184	0.09384	-0.33504	-0.29662	0.4155	-0.4155	0.65804	0.05798	0.00083	0.0173	0.22213	7.49E-06	8.17E-05	1.59E-08	1.59E-08	brown
KIF21A	0.09762	-0.1426	-0.15558	-0.00644	0.16123	-0.23959	-0.16324	0.13206	-0.13206	0.20399	0.06281	0.04216	0.93337	0.03514	0.0016	0.0329	0.0851	0.0851	turquoise
ANO10	0.05216	-0.01296	0.02239	-0.03283	-0.04071	0.12882	0.0734	-0.06499	0.06499	0.49808	0.86639	0.77131	0.66995	0.59708	0.0931	0.34003	0.39842	0.39842	turquoise
RAB22A	0.07236	-0.09811	0.02948	0.00375	-0.01116	0.01357	0.03485	-0.10641	0.10641	0.34697	0.20174	0.70188	0.96119	0.88478	0.86016	0.65087	0.16597	0.16597	turquoise
EYA3	0.03957	-0.19903	-0.03212	-0.02205	-0.07264	0.03909	-0.00072	0.17315	-0.17315	0.60738	0.00906	0.67663	0.77468	0.34507	0.61172	0.99256	0.02353	0.02353	turquoise
GLB1L	-0.04267	-0.11012	-0.04335	0.02891	-0.0575	-0.03308	0.05723	0.3507	-0.3507	0.57947	0.15162	0.57347	0.70737	0.45507	0.6675	0.45717	2.57E-06	2.57E-06	grey
EDC4	-0.00821	-0.14468	0.00629	-0.04787	0.04948	0.07054	-0.03221	0.06614	-0.06614	0.91512	0.05902	0.93496	0.53413	0.52041	0.35924	0.67578	0.39006	0.39006	turquoise
CCER2	0.04008	-0.03897	-0.13708	-0.06605	0.18311	-0.18367	-0.0927	-0.05222	0.05222	0.6027	0.61283	0.0738	0.39074	0.01652	0.01619	0.22787	0.49758	0.49758	grey
LPIN3	0.09937	-0.08387	0.00684	-0.00436	0.20664	0.01395	0.02024	-0.35615	0.35615	0.196	0.27545	0.92928	0.95488	0.00669	0.85631	0.79271	1.75E-06	1.75E-06	red
C11orf80	0.02115	-0.08791	-0.00445	0.00101	-0.0328	0.00641	0.13373	-0.4326	0.4326	0.78366	0.25289	0.95395	0.98951	0.6702	0.93367	0.08119	3.44E-09	3.44E-09	grey
TACC2	0.08531	-0.01845	-0.09281	-0.13145	0.14175	-0.02749	-0.03454	-0.15857	0.15857	0.26727	0.8107	0.22729	0.08658	0.0644	0.72116	0.65384	0.03831	0.03831	grey
GDI2	0.03732	-0.04769	0.04881	-0.00114	-0.16511	0.07924	0.15469	0.06017	-0.06017	0.62794	0.53563	0.52608	0.98815	0.03092	0.30288	0.04336	0.43436	0.43436	turquoise
PDE6G	-0.16978	-0.02421	0.01497	0.08874	-0.14351	0.06364	-0.07098	0.34287	-0.34287	0.02642	0.75326	0.84587	0.2484	0.06113	0.40827	0.35624	4.42E-06	4.42E-06	blue
INTS1	0.00325	-0.02372	-0.08589	-0.02907	0.11134	-0.10726	0.08384	-0.13199	0.13199	0.96634	0.75813	0.264	0.70585	0.14711	0.1626	0.27559	0.08527	0.08527	green
STAU2	0.00746	-0.10629	0.02881	0.05698	-0.12168	0.04867	0.01052	0.17726	-0.17726	0.92287	0.16645	0.70839	0.45918	0.11286	0.52729	0.89142	0.02038	0.02038	turquoise
TRPV6	0.0625	-0.04286	-0.00689	0.14704	-0.03879	0.13921	0.01955	-0.02271	0.02271	0.41675	0.57779	0.92874	0.05496	0.61447	0.06938	0.79966	0.76809	0.76809	magenta
TAS2R20	0.00144	-0.14778	-0.08186	-0.06105	0.05315	-0.08592	-0.0275	0.02606	-0.02606	0.98506	0.05375	0.28713	0.42769	0.48994	0.26385	0.72102	0.73515	0.73515	red
PAFAH1B2	-0.02625	-0.12619	-0.06087	-0.0312	-0.17086	0.00843	0.1208	-0.10952	0.10952	0.7333	0.10006	0.42902	0.68539	0.02546	0.91289	0.11552	0.15389	0.15389	turquoise
RPL36A	0.12663	0.13744	0.02439	-0.00424	-0.09083	0.12489	0.24985	-0.39268	0.39268	0.09886	0.07304	0.75153	0.95607	0.23739	0.10361	0.00098	1.08E-07	1.08E-07	purple
KRT18	0.18643	0.01608	0.01273	0.05027	0.04685	0.0856	0.15317	-0.55676	0.55676	0.01463	0.83463	0.86872	0.51381	0.5429	0.26561	0.04549	2.63E-15	2.63E-15	yellow
AGPAT4	-0.01519	-0.11894	-0.06737	0.04457	-0.09915	-0.10588	-0.09324	0.23917	-0.23917	0.84372	0.12126	0.38129	0.56271	0.19697	0.16813	0.22513	0.00163	0.00163	blue
TACR1	-0.06125	-0.02834	-0.21824	-0.11187	0.01748	-0.18227	-0.19622	0.54306	-0.54306	0.42615	0.71295	0.00414	0.1452	0.82052	0.01703	0.01011	1.67E-14	1.67E-14	brown
ZNF570	-0.10068	-0.15134	-0.06674	0.01442	-0.10322	-0.0104	-0.10577	0.47923	-0.47923	0.19012	0.04817	0.38576	0.85152	0.17912	0.89258	0.16858	3.34E-11	3.34E-11	turquoise
COLGALT1	-0.06386	-0.01309	0.0783	0.08561	-0.14902	0.09114	0.17045	-0.09728	0.09728	0.40663	0.86509	0.3087	0.26557	0.05174	0.2358	0.02582	0.20559	0.20559	black
NCKIPSD	0.06929	-0.18176	-0.0908	-0.06413	0.06229	-0.03368	-0.01592	-0.07905	0.07905	0.36786	0.01735	0.23758	0.40466	0.41833	0.66187	0.83623	0.30406	0.30406	grey
NDUFS2	0.0323	0.06952	-0.07255	-0.13827	0.02799	-0.00582	0.15484	-0.25338	0.25338	0.6749	0.36623	0.34569	0.0713	0.71633	0.93977	0.04316	0.00083	0.00083	greenyellow
TDRD9	-0.0922	-0.10824	-0.20264	-0.08251	0.09599	-0.24879	-0.09646	0.47187	-0.47187	0.23036	0.15879	0.00786	0.28334	0.21168	0.00103	0.20946	7.27E-11	7.27E-11	brown
UBQLN4	0.03565	0.03696	0.0799	0.00297	-0.13241	0.26633	0.21988	-0.2	0.2	0.64346	0.6313	0.29889	0.96928	0.08427	0.00043	0.00386	0.00872	0.00872	turquoise
NAA16	-0.03749	-0.17808	-0.01654	0.01421	0.06879	-0.00665	-0.11651	0.21842	-0.21842	0.62642	0.01979	0.83002	0.85362	0.37129	0.93118	0.12912	0.0041	0.0041	turquoise
SIX2	-0.08677	-0.10532	-0.11478	0.0536	-0.07911	-0.05838	-0.02697	0.05981	-0.05981	0.25913	0.17037	0.13495	0.48628	0.30368	0.44818	0.72619	0.43712	0.43712	grey
HABP4	-0.00126	-0.12053	-0.16146	-0.06039	-0.09684	-0.20813	-0.10889	0.38535	-0.38535	0.98696	0.11635	0.03488	0.4327	0.20766	0.0063	0.15626	1.95E-07	1.95E-07	brown
TMEM258	0.02446	0.02746	0.01151	-0.0323	-0.04537	-0.07604	0.08239	-0.31637	0.31637	0.7508	0.72148	0.88119	0.6749	0.5557	0.32292	0.28403	2.49E-05	2.49E-05	grey
CALY	0.10141	-0.14783	-0.24825	-0.18026	0.13523	-0.35534	-0.24102	0.32184	-0.32184	0.18692	0.05365	0.00106	0.01831	0.07782	1.85E-06	0.0015	1.77E-05	1.77E-05	brown
RBP5	-0.11766	-0.12266	0.00577	0.0097	0.00533	-0.03365	-0.08595	0.37675	-0.37675	0.12536	0.10998	0.94027	0.89979	0.94482	0.6622	0.26367	3.80E-07	3.80E-07	blue
PDE4D	-0.00733	-0.04785	0.00517	-0.04111	0.00087	-0.05838	-0.11283	0.23276	-0.23276	0.92418	0.53429	0.94645	0.59345	0.99098	0.44815	0.14173	0.00219	0.00219	turquoise
RNF180	-0.0276	-0.13639	-0.17193	-0.03474	-0.06963	-0.15536	-0.21339	0.61962	-0.61962	0.72005	0.07528	0.02454	0.65196	0.36551	0.04245	0.00507	1.64E-19	1.64E-19	brown
DOK6	-0.0591	-0.18119	-0.13991	-0.04715	-0.17237	-0.04846	-0.12269	0.48668	-0.48668	0.4426	0.01771	0.06799	0.54026	0.02417	0.52906	0.10991	1.49E-11	1.49E-11	turquoise
KBTBD2	-0.06371	-0.15371	0.04389	0.06306	-0.1477	0.17176	0.06245	0.01504	-0.01504	0.40778	0.04472	0.56872	0.41256	0.05387	0.02468	0.41712	0.84516	0.84516	turquoise
KANSL1L	0.02993	-0.10161	-0.05667	0.12532	-0.01763	0.17448	0.06143	0.06943	-0.06943	0.6976	0.18604	0.46162	0.10243	0.81895	0.02246	0.42476	0.3669	0.3669	turquoise
C4BPA	0.06855	0.0124	-0.02384	0.09416	-0.01523	0.11273	-0.03583	-0.10245	0.10245	0.37301	0.87217	0.75692	0.22056	0.84326	0.14211	0.64172	0.18241	0.18241	magenta
MFAP3	-0.06841	-0.18939	-0.00988	0.06625	-0.16778	0.12131	0.0233	0.36001	-0.36001	0.37398	0.0131	0.8979	0.38928	0.02827	0.114	0.76224	1.32E-06	1.32E-06	turquoise
PRSS8	0.11722	0.06181	0.11935	0.06606	-0.07978	0.2943	0.24082	-0.44816	0.44816	0.12681	0.42188	0.12	0.39062	0.29958	9.34E-05	0.00151	7.91E-10	7.91E-10	yellow
FBXL2	-0.0321	-0.21514	-0.18293	-0.11709	0.01367	-0.15467	-0.05574	0.08372	-0.08372	0.67684	0.00472	0.01663	0.12723	0.85915	0.04339	0.469	0.27633	0.27633	grey
STAC2	0.04795	-0.04474	0.04968	0.10222	-0.16194	0.02481	0.04819	-0.04824	0.04824	0.53338	0.56124	0.51878	0.1834	0.03434	0.74742	0.53137	0.53092	0.53092	grey
HPS5	-0.10024	-0.16912	0.04041	0.02127	-0.18837	0.09255	0.06218	0.28399	-0.28399	0.19207	0.02702	0.59974	0.78244	0.01361	0.2286	0.41915	0.00017	0.00017	turquoise
RNF41	0.02861	-0.21126	-0.0804	0.02295	-0.00713	-0.12083	-0.15203	0.24598	-0.24598	0.71029	0.00554	0.29587	0.76572	0.92631	0.11543	0.04714	0.00118	0.00118	turquoise
PRCP	-0.14657	-0.07945	0.01429	0.13096	-0.20317	0.06258	-0.01917	0.52657	-0.52657	0.05576	0.30162	0.85282	0.08777	0.0077	0.41616	0.80343	1.39E-13	1.39E-13	blue
TMEM82	-0.01906	0.02664	0.04279	-0.01114	0.19556	0.12704	0.10405	-0.27704	0.27704	0.80459	0.72946	0.57845	0.88505	0.01037	0.09776	0.17562	0.00024	0.00024	grey
ATG4A	-0.02444	-0.18866	-0.05415	-0.02073	0.05206	-0.04853	-0.07472	0.12453	-0.12453	0.751	0.01346	0.48177	0.78781	0.49887	0.5285	0.33142	0.10464	0.10464	turquoise
MEGF8	-0.06768	-0.13958	-0.15914	0.00537	-0.06129	-0.17794	-0.07663	0.39993	-0.39993	0.37908	0.06864	0.03762	0.94443	0.42585	0.01989	0.31919	5.98E-08	5.98E-08	turquoise
CSRNP1	-0.09981	-0.20513	-0.05955	0.02366	0.00018	0.04543	-0.09776	0.28647	-0.28647	0.19398	0.00712	0.43909	0.75868	0.99817	0.55516	0.20336	0.00015	0.00015	grey
WDR20	-0.00365	-0.16224	-0.07194	0.03157	0.07962	-0.05224	-0.11614	0.09312	-0.09312	0.96225	0.034	0.34975	0.68192	0.30058	0.49743	0.13035	0.22573	0.22573	turquoise
MUCL3	-0.05123	0.02625	0.12367	0.07639	0.02477	0.22374	0.07272	-0.16444	0.16444	0.50579	0.73326	0.10707	0.32067	0.74778	0.00326	0.34453	0.03162	0.03162	yellow
THAP4	0.11304	0.03848	-0.1043	-0.0527	0.12179	-0.05272	0.13834	-0.27547	0.27547	0.141	0.61725	0.17461	0.49358	0.11256	0.49344	0.07116	0.00027	0.00027	grey
TNFSF18	-0.04518	-0.06966	-0.03561	-0.02529	-0.12869	0.12079	-0.04273	0.37689	-0.37689	0.55737	0.36531	0.64379	0.74267	0.09345	0.11555	0.57894	3.76E-07	3.76E-07	blue
CCDC130	0.07129	-0.05162	-0.10214	-0.0662	0.27436	-0.09861	-0.02401	-0.17133	0.17133	0.35415	0.50253	0.18376	0.38964	0.00028	0.19942	0.75526	0.02505	0.02505	red
IRF2BP2	-0.06301	-0.12482	-0.17635	-0.04083	0.07909	-0.17965	-0.04762	0.09875	-0.09875	0.41295	0.10381	0.02104	0.596	0.30384	0.01871	0.53625	0.19878	0.19878	turquoise
TVP23A	-0.02295	0.01803	-0.09292	-0.01693	-0.06072	-0.02337	-0.10857	0.38943	-0.38943	0.76578	0.81489	0.22676	0.82601	0.43014	0.76155	0.15749	1.41E-07	1.41E-07	blue
VSIG1	-0.15614	0.08316	0.19158	0.02798	0.05714	0.26451	0.0909	-0.26051	0.26051	0.04142	0.27953	0.01207	0.71638	0.45788	0.00047	0.23706	0.00058	0.00058	yellow
PCGF6	-0.13299	-0.0026	0.02292	0.06145	-0.12787	-0.00696	0.13537	0.22284	-0.22284	0.08291	0.97313	0.76601	0.42461	0.09558	0.92799	0.07751	0.0034	0.0034	turquoise
MRPL27	0.00192	-0.0668	-0.14936	-0.09725	0.13659	-0.19494	0.00994	-0.26291	0.26291	0.98015	0.38537	0.05121	0.20572	0.07484	0.01062	0.89733	0.00051	0.00051	grey
CDYL2	-0.01391	-0.17699	-0.08819	0.06066	-0.14124	-0.07245	-0.0426	0.29608	-0.29608	0.85671	0.02057	0.25136	0.43059	0.06537	0.34632	0.58011	8.43E-05	8.43E-05	turquoise
PDE9A	0.00346	-0.19392	-0.25758	-0.04955	0.12333	-0.23997	-0.11997	0.07746	-0.07746	0.96419	0.01104	0.00067	0.51987	0.10804	0.00157	0.11806	0.31393	0.31393	grey
DUS3L	0.03866	0.17087	-0.10243	-0.03996	0.23063	-0.15251	-0.00161	-0.09377	0.09377	0.61563	0.02545	0.1825	0.60377	0.00241	0.04644	0.98336	0.22253	0.22253	green
CEP350	-0.01779	-0.15851	-0.02009	-0.00921	-0.10923	0.10907	0.05098	0.20576	-0.20576	0.81732	0.03839	0.7942	0.90479	0.155	0.1556	0.50785	0.00694	0.00694	turquoise
CRYBG3	-0.04153	-0.13437	0.03244	0.0897	-0.20219	0.23239	0.09462	0.29213	-0.29213	0.58966	0.07974	0.67358	0.24334	0.008	0.00222	0.21834	0.00011	0.00011	turquoise
EMC10	-0.00717	-0.14659	-0.24152	-0.09903	0.20908	-0.35963	-0.18326	0.10818	-0.10818	0.92584	0.05572	0.00146	0.19751	0.00606	1.36E-06	0.01643	0.159	0.159	brown
NBN	-0.05548	-0.02942	0.06284	0.09499	-0.1976	0.08286	0.08928	0.18374	-0.18374	0.47108	0.70246	0.41423	0.2165	0.00958	0.28126	0.24556	0.01614	0.01614	turquoise
RAB27B	-0.04643	-0.00855	0.08164	-0.00121	0.02582	0.22534	0.22282	-0.29273	0.29273	0.5465	0.91163	0.28846	0.98746	0.73741	0.00304	0.0034	0.0001	0.0001	yellow
TBC1D10C	-0.09659	0.01503	0.00273	0.0126	-0.05755	0.04546	-0.02967	0.32441	-0.32441	0.20887	0.84525	0.97176	0.87012	0.45468	0.55493	0.70009	1.50E-05	1.50E-05	blue
SDR16C5	-0.0223	0.06748	0.12975	0.04017	-0.05371	0.2322	0.17548	-0.4734	0.4734	0.7722	0.38052	0.09075	0.6019	0.48535	0.00224	0.02169	6.19E-11	6.19E-11	yellow
DTD2	-0.07972	-0.02583	-0.01649	0.09592	-0.01942	-0.01288	-0.00056	0.15085	-0.15085	0.29999	0.73737	0.83046	0.21201	0.80092	0.86719	0.9942	0.0489	0.0489	turquoise
ZFP37	-0.10271	-0.15723	-0.11592	0.05023	-0.11882	-0.12842	-0.17927	0.37035	-0.37035	0.18127	0.03999	0.1311	0.51416	0.12164	0.09415	0.01897	6.17E-07	6.17E-07	turquoise
CNOT2	0.0764	-0.17014	-0.04526	0.0565	0.06387	-0.00793	-0.03426	-0.08975	0.08975	0.32062	0.02609	0.55662	0.46297	0.40656	0.918	0.65646	0.24305	0.24305	turquoise
MAPK11	-0.10657	-0.08917	-0.18802	-0.01509	0.05277	-0.30822	-0.22176	0.36569	-0.36569	0.16533	0.24611	0.01379	0.84465	0.49309	4.11E-05	0.00356	8.73E-07	8.73E-07	brown
KIF19	0.00991	-0.06527	-0.18664	-0.16465	0.09898	-0.14136	-0.19317	0.31845	-0.31845	0.89759	0.39633	0.01451	0.0314	0.19774	0.06516	0.01136	2.19E-05	2.19E-05	brown
SRL	-0.17655	-0.02102	-0.04181	0.06397	-0.05839	0.04373	-0.0639	0.36927	-0.36927	0.02089	0.78495	0.58718	0.40585	0.44809	0.57011	0.40633	6.69E-07	6.69E-07	blue
TWSG1	-0.09295	-0.1509	0.06185	0.13877	-0.15445	0.07017	0.12064	0.22637	-0.22637	0.22658	0.04883	0.42161	0.07027	0.04369	0.3618	0.116	0.00291	0.00291	turquoise
GALNT8	0.09814	-0.02752	-0.12571	-0.10006	0.18307	-0.21289	-0.1828	0.21464	-0.21464	0.2016	0.72082	0.10134	0.19287	0.01654	0.00518	0.01671	0.00482	0.00482	brown
JOSD2	0.01162	0.02027	-0.13514	-0.01371	0.15964	-0.03874	0.15707	-0.30326	0.30326	0.88013	0.79243	0.07801	0.85875	0.03701	0.61491	0.0402	5.53E-05	5.53E-05	green
LMBR1	-0.01414	-0.17324	0.0008	-0.01094	-0.09707	-0.06952	0.0271	0.08931	-0.08931	0.85432	0.02345	0.99174	0.88708	0.20656	0.36622	0.72499	0.24538	0.24538	turquoise
RFXAP	-0.04848	-0.02529	-0.18739	-0.12641	0.03848	-0.27833	-0.16575	0.4793	-0.4793	0.52892	0.74264	0.01412	0.09946	0.61732	0.00023	0.03027	3.31E-11	3.31E-11	turquoise
SPOCK2	-0.01117	-0.14851	0.01658	-0.01426	-0.05688	-0.00049	-0.08351	0.3164	-0.3164	0.88474	0.05255	0.82953	0.85309	0.45995	0.99488	0.27754	2.49E-05	2.49E-05	blue
SETX	-0.04942	-0.1672	-0.02808	-0.01286	-0.19903	0.07521	0.00656	0.30046	-0.30046	0.52097	0.02883	0.71538	0.86744	0.00906	0.32821	0.93218	6.53E-05	6.53E-05	turquoise
CFAP69	-0.06942	-0.22669	-0.09192	0.01691	0.21646	-0.08328	-0.10298	0.11014	-0.11014	0.36697	0.00287	0.2318	0.82622	0.00446	0.27887	0.18014	0.15154	0.15154	grey
PTDSS2	0.08745	-0.05383	-0.10874	0.00809	0.04439	-0.17946	0.04065	-0.04498	0.04498	0.25537	0.48436	0.15686	0.91634	0.56426	0.01884	0.59756	0.55909	0.55909	green
AMER1	-0.05935	-0.04327	-0.02596	0.04428	-0.16616	0.16158	0.09329	0.22729	-0.22729	0.44065	0.57419	0.73611	0.56522	0.02986	0.03475	0.22487	0.00279	0.00279	turquoise
HSPA4	0.01157	-0.13802	-0.08561	-0.0255	0.00763	-0.09604	0.0121	0.07084	-0.07084	0.88066	0.07183	0.26557	0.74055	0.92109	0.21145	0.87514	0.35722	0.35722	turquoise
TCIRG1	-0.04819	0.06498	0.11985	0.10225	-0.02929	0.20626	0.24155	-0.32555	0.32555	0.5314	0.39842	0.11843	0.18327	0.70377	0.0068	0.00146	1.39E-05	1.39E-05	brown
PTK6	0.08773	0.14337	0.10734	0.05405	0.04387	0.24824	0.29398	-0.58893	0.58893	0.25384	0.06139	0.16228	0.4826	0.56883	0.00106	9.51E-05	2.41E-17	2.41E-17	yellow
MED11	-0.14405	-0.00722	-0.05352	-0.07143	0.13399	-0.1226	-0.08956	0.05167	-0.05167	0.06015	0.92536	0.48688	0.35319	0.08061	0.11015	0.24406	0.50209	0.50209	grey
TIMELESS	-0.00789	-0.06541	-0.07324	-0.07022	0.04129	-0.148	0.05622	-0.02557	0.02557	0.91847	0.39537	0.34108	0.36145	0.5918	0.05338	0.46514	0.73993	0.73993	turquoise
NLK	-0.03705	-0.1923	-0.19604	-0.11185	0.11604	-0.21826	-0.17888	0.28318	-0.28318	0.63042	0.01174	0.01018	0.14527	0.13068	0.00413	0.01924	0.00017	0.00017	turquoise
TRIB1	-0.05854	-0.16134	0.08973	0.04531	-0.06384	0.17297	0.11741	-0.15681	0.15681	0.44695	0.03502	0.24319	0.55622	0.40678	0.02368	0.12619	0.04053	0.04053	turquoise
FOXO4	-0.03945	-0.13246	-0.01442	-0.01905	-0.00875	0.13994	0.03373	0.05931	-0.05931	0.60846	0.08415	0.8515	0.80468	0.90952	0.06791	0.66137	0.441	0.441	turquoise

MAN2C1	-0.05395	-0.05808	-0.08017	-0.10249	0.1672	-0.06353	-0.03573	-0.03077	0.03077	0.48343	0.45051	0.29727	0.18224	0.02883	0.40908	0.64273	0.68951	0.68951	red
SLC25A29	0.04036	-0.08345	-0.07104	0.02923	0.28681	-0.11776	-0.11189	-0.24212	0.24212	0.60016	0.27787	0.35581	0.70431	0.00014	0.12505	0.14512	0.00142	0.00142	red
LEPR	-0.18253	-0.11171	-0.03697	0.06621	-0.18212	0.09537	-0.0408	0.51221	-0.51221	0.01687	0.14577	0.63118	0.38954	0.01712	0.2147	0.59624	8.02E-13	8.02E-13	blue
MAP1LC3I	-0.05502	-0.07077	-0.01426	-0.03668	-0.13112	0.02301	0.04978	-0.03758	0.03758	0.47478	0.35767	0.85315	0.63386	0.08736	0.76511	0.51787	0.62553	0.62553	turquoise
CPQ	-0.04917	-0.0741	-0.05928	0.00166	-0.05387	-0.14876	-0.14139	0.56015	-0.56015	0.52301	0.33542	0.4412	0.98276	0.48403	0.05215	0.06509	1.64E-15	1.64E-15	turquoise
ZC4H2	-0.0321	-0.19794	-0.13242	-0.01941	-0.09471	-0.10437	-0.15039	0.11751	-0.11751	0.67685	0.00946	0.08425	0.80102	0.2179	0.17432	0.0496	0.12585	0.12585	turquoise
PURA	-0.02862	-0.20177	-0.08257	-0.00806	-0.048	0.01412	-0.05723	0.31778	-0.31778	0.71019	0.00813	0.283	0.91665	0.53303	0.85453	0.45722	2.28E-05	2.28E-05	turquoise
GFER	0.16207	0.0497	-0.10231	-0.09116	0.08209	-0.07631	0.07006	-0.34727	0.34727	0.03419	0.51857	0.18301	0.23572	0.28581	0.32122	0.36251	3.26E-06	3.26E-06	green
YIPF6	-0.01176	-0.2177	-0.08912	-0.02536	0.00245	-0.11658	-0.0693	0.03879	-0.03879	0.87871	0.00423	0.2464	0.74197	0.97462	0.12891	0.36775	0.61447	0.61447	turquoise
SPRTN	-0.02454	-0.07566	-0.06128	-0.0014	-0.06712	0.03619	0.04294	0.16424	-0.16424	0.75	0.32531	0.42593	0.98555	0.38308	0.63837	0.57705	0.03183	0.03183	turquoise
SH2D1A	-0.09611	-0.00876	0.05486	0.05826	-0.17009	0.13627	-0.02092	0.45206	-0.45206	0.21115	0.9095	0.47603	0.44914	0.02614	0.07554	0.78591	5.41E-10	5.41E-10	blue
DLL1	-0.05261	-0.11261	-0.01005	0.02198	0.12745	-0.01485	0.00437	0.24022	-0.24022	0.49432	0.14253	0.89618	0.77534	0.09668	0.84709	0.9548	0.00155	0.00155	grey
THBS1	-0.07935	-0.10911	0.05113	0.1003	-0.25164	0.17125	0.01294	0.38802	-0.38802	0.30221	0.15545	0.50663	0.19179	0.0009	0.02512	0.86656	1.57E-07	1.57E-07	blue
GPIHBP1	-0.04112	-0.08041	-0.05926	0.1081	0.00311	-0.00823	-0.06745	0.50354	-0.50354	0.59338	0.29579	0.44135	0.15931	0.96783	0.91497	0.38075	2.22E-12	2.22E-12	blue
NIT1	0.03527	-0.0734	-0.04101	-0.07387	0.15913	0.05226	0.09647	-0.3242	0.3242	0.64697	0.34003	0.59434	0.33698	0.03763	0.49721	0.20941	1.52E-05	1.52E-05	red
ATP6V1H	-0.00422	-0.04355	-0.16577	-0.09871	-0.05858	-0.23063	-0.01402	0.07928	-0.07928	0.9563	0.57166	0.03024	0.19896	0.44658	0.00241	0.85554	0.30267	0.30267	turquoise
LRRC10B	-0.03853	-0.12577	-0.22517	-0.06047	0.13602	-0.306	-0.23564	0.35866	-0.35866	0.61679	0.1012	0.00307	0.43203	0.07609	4.70E-05	0.00192	1.46E-06	1.46E-06	brown
AGMAT	-0.03901	0.18714	0.06428	0.08933	0.11032	0.17181	0.14338	-0.22764	0.22764	0.61243	0.01425	0.40355	0.2453	0.15089	0.02464	0.06137	0.00275	0.00275	grey
C9orf64	-0.05461	-0.06919	-0.10257	0.03609	-0.07858	-0.03184	-0.00798	0.14431	-0.14431	0.47808	0.36855	0.18189	0.63937	0.30695	0.6793	0.91745	0.05969	0.05969	turquoise
SERPINF2	-0.00522	-0.09206	-0.17487	-0.07972	0.03984	-0.2497	-0.17352	0.34135	-0.34135	0.94596	0.2311	0.02216	0.3	0.60494	0.00099	0.02323	4.90E-06	4.90E-06	grey
STK17A	-0.06736	-0.12208	0.10392	0.07159	-0.24666	0.22833	0.08163	0.09154	-0.09154	0.38134	0.11169	0.17619	0.35212	0.00115	0.00267	0.2885	0.23377	0.23377	blue
UTRN	-0.14477	-0.13803	0.01275	0.03952	-0.19995	0.0933	-0.08773	0.49262	-0.49262	0.05886	0.0718	0.8685	0.60784	0.00874	0.22484	0.25385	7.70E-12	7.70E-12	turquoise
TLR5	-0.08863	-0.0614	0.03613	0.0994	-0.05947	0.10903	0.08425	0.12795	-0.12795	0.24902	0.42498	0.63893	0.19583	0.43975	0.15574	0.27327	0.09538	0.09538	grey
CCDC189	-0.01805	-0.10213	-0.13489	-0.17847	0.22217	-0.13454	-0.02381	-0.19623	0.19623	0.81472	0.18377	0.07858	0.01952	0.00349	0.07937	0.75721	0.0101	0.0101	green
CEP126	0.0042	-0.20899	-0.2482	-0.1262	0.08544	-0.31732	-0.20344	0.33013	-0.33013	0.9565	0.00608	0.00106	0.10003	0.26654	2.35E-05	0.00761	1.04E-05	1.04E-05	brown
PLEKHG4	0.03887	0.05669	-0.0448	0.07446	0.13078	0.05806	0.06417	0.0168	-0.0168	0.61377	0.46146	0.56068	0.33311	0.08821	0.45069	0.4044	0.8274	0.8274	grey
NIPAL2	0.06658	-0.08851	-0.02302	0.04764	-0.04265	0.08712	0.05698	-0.05025	0.05025	0.3869	0.24965	0.76505	0.53611	0.57969	0.2572	0.45911	0.51391	0.51391	turquoise
GPSM3	-0.07973	0.00879	0.04825	-0.03516	-0.14442	0.14437	0.04883	0.14102	-0.14102	0.2999	0.90919	0.53091	0.64798	0.05949	0.05957	0.52595	0.06581	0.06581	blue
EIF3M	0.08158	0.04123	0.05154	-0.03569	-0.1215	0.17417	0.16181	-0.33151	0.33151	0.2888	0.59232	0.50319	0.64307	0.11343	0.02271	0.03448	9.46E-06	9.46E-06	turquoise
KIF23	-0.03503	0.04424	0.04123	0.03816	-0.09991	0.09274	0.30987	-0.35315	0.35315	0.64923	0.56561	0.59232	0.62019	0.19355	0.22765	3.72E-05	2.16E-06	2.16E-06	pink
TUBGCP4	-0.03464	-0.1573	-0.10624	-0.04362	-0.0887	0.01352	0.06987	-0.03774	0.03774	0.6529	0.03991	0.16666	0.57107	0.24861	0.86064	0.36385	0.62413	0.62413	turquoise
CAMTA2	-0.06064	-0.14539	-0.12181	-0.06525	0.09925	-0.21294	-0.1566	0.36228	-0.36228	0.43077	0.05778	0.11248	0.39648	0.19654	0.00517	0.04081	1.12E-06	1.12E-06	turquoise
SUGCT	-0.06617	0.0655	0.04455	0.02577	-0.16745	0.16558	0.1019	-0.01755	0.01755	0.38982	0.39466	0.56287	0.73794	0.02859	0.03044	0.18479	0.81977	0.81977	black
TMUB2	-0.0188	-0.17338	-0.00886	-0.01359	0.08956	0.01053	0.01912	-0.17239	0.17239	0.80721	0.02334	0.90843	0.85995	0.24404	0.89126	0.80401	0.02416	0.02416	grey
WDR47	-0.10447	-0.17422	-0.04079	-0.00048	-0.13558	0.03284	-0.02635	0.35849	-0.35849	0.17388	0.02267	0.59636	0.99507	0.07704	0.66978	0.73231	1.48E-06	1.48E-06	turquoise
SAMD5	0.00349	-0.01646	-0.11924	-0.07142	0.04555	-0.03871	-0.18667	0.2265	-0.2265	0.96383	0.8308	0.12032	0.35324	0.55409	0.61517	0.0145	0.00289	0.00289	grey
NMRAL1	0.11994	0.09481	-0.02045	-0.03635	0.08653	0.01098	0.16867	-0.44158	0.44158	0.11815	0.21739	0.79061	0.63694	0.26046	0.88669	0.02743	1.49E-09	1.49E-09	green
GMPS	-0.02566	-0.05046	-0.05626	-0.03783	-0.18002	0.04826	0.19755	-0.1014	0.1014	0.73904	0.51222	0.46486	0.62323	0.01847	0.53076	0.0096	0.18696	0.18696	turquoise
ZNF430	-0.00267	-0.09227	-0.06079	-0.00465	0.00201	0.04206	-0.07864	0.19228	-0.19228	0.97232	0.23001	0.42965	0.95187	0.97918	0.58494	0.30659	0.01175	0.01175	turquoise
WDR31	-0.04534	-0.15692	-0.18426	-0.0346	0.01836	-0.11533	-0.04478	0.26554	-0.26554	0.55599	0.0404	0.01584	0.65319	0.81157	0.13308	0.56081	0.00045	0.00045	turquoise
YTHDC2	-0.10407	-0.15999	-0.06421	-0.00639	-0.01355	-0.01821	-0.06005	0.27939	-0.27939	0.17554	0.03659	0.4041	0.93388	0.86039	0.81314	0.43528	0.00022	0.00022	turquoise
HABP2	0.04475	-0.03264	-0.07271	0.11848	0.02885	0.14086	-0.02941	-0.11238	0.11238	0.56108	0.67172	0.34463	0.12274	0.70794	0.06612	0.70254	0.14337	0.14337	magenta
NT5M	-0.06079	-0.04187	-0.22427	-0.02446	0.1314	-0.31419	-0.09269	0.29556	-0.29556	0.42963	0.58664	0.00319	0.75084	0.0867	2.85E-05	0.22791	8.69E-05	8.69E-05	brown
MYLK4	-0.04858	-0.07634	-0.12394	-0.01356	-0.01601	-0.08749	-0.01541	0.22981	-0.22981	0.52809	0.32102	0.10631	0.86029	0.8354	0.2552	0.84141	0.0025	0.0025	turquoise
POLD3	-0.06498	0.02046	0.01554	0.00508	-0.02693	0.1945	0.22207	-0.12702	0.12702	0.39847	0.79051	0.8401	0.94738	0.72665	0.0108	0.00351	0.09782	0.09782	turquoise
PNLIPRP1	-0.01674	0.05763	0.08274	0.15282	-0.11097	0.07764	-0.06089	0.14939	-0.14939	0.82793	0.45402	0.282	0.04599	0.14848	0.31282	0.42887	0.05116	0.05116	magenta
RFXANK	0.06269	0.09046	-0.06706	-0.0272	0.0858	-0.04939	0.16777	-0.38206	0.38206	0.41534	0.23934	0.3835	0.72401	0.26452	0.52123	0.02829	2.52E-07	2.52E-07	green
PLA2G2A	0.02452	0.09443	0.07634	0.02349	-0.07989	0.10831	-0.00304	0.33098	-0.33098	0.75025	0.21924	0.32101	0.76038	0.29893	0.15851	0.96856	9.79E-06	9.79E-06	grey
RNF128	-0.03859	-0.0182	0.03403	0.08227	0.18112	0.06912	-0.04425	-0.20876	0.20876	0.61629	0.8132	0.65857	0.28472	0.01775	0.36906	0.56547	0.00614	0.00614	yellow
ZNF101	-0.06729	-0.0631	-0.01835	0.00291	-0.06228	0.0355	-0.12997	0.32566	-0.32566	0.38185	0.41227	0.81173	0.96983	0.41838	0.64482	0.0902	1.38E-05	1.38E-05	blue
HOXA10	0.10714	-0.08455	0.12685	0.12384	-0.10197	0.21646	0.16057	-0.25883	0.25883	0.1631	0.27157	0.09827	0.10657	0.18449	0.00446	0.03591	0.00063	0.00063	grey
ICMT	-0.07015	-0.122	-0.02967	-0.0137	-0.07515	0.01785	0.0318	0.10852	-0.10852	0.36189	0.11192	0.70004	0.85881	0.32859	0.81672	0.67967	0.15772	0.15772	turquoise
FMNL1	-0.08495	-0.06018	0.04074	0.00891	-0.12839	0.04279	0.02241	0.33287	-0.33287	0.2693	0.43424	0.59676	0.90795	0.09423	0.57841	0.77112	8.65E-06	8.65E-06	blue
UTP20	-0.02586	-0.07876	-0.00411	0.02016	-0.08515	0.04501	-0.00697	0.2301	-0.2301	0.73707	0.30585	0.95741	0.79352	0.26817	0.55884	0.92796	0.00247	0.00247	turquoise
ECI2	-0.16191	0.00952	-0.07296	-0.10302	0.06816	-0.04273	-0.12957	0.1959	-0.1959	0.03437	0.90167	0.34297	0.17997	0.3757	0.57897	0.0912	0.01023	0.01023	grey
KCNMA1	0.00127	-0.03745	-0.08956	-0.02042	0.02744	-0.20068	-0.14421	0.5164	-0.5164	0.98689	0.62678	0.24404	0.79093	0.72165	0.00849	0.05986	4.85E-13	4.85E-13	brown
ARHGAP9	-0.09577	-0.01516	0.03254	0.04922	-0.14175	0.08389	0.028	0.36747	-0.36747	0.21274	0.84399	0.67263	0.52264	0.06441	0.27535	0.71618	7.65E-07	7.65E-07	blue
RPS7	0.04636	0.12503	0.03469	-0.03764	-0.11533	0.09163	0.20005	-0.19528	0.19528	0.54713	0.10323	0.65239	0.62502	0.13308	0.23331	0.00871	0.01048	0.01048	purple
TRAPPC2	-0.27544	-0.21268	-0.0766	-0.02665	0.09322	-0.13409	-0.13987	-0.08481	0.08481	0.00027	0.00523	0.31935	0.72931	0.22523	0.08038	0.06806	0.27009	0.27009	red
PLBD1	-0.00871	0.0466	0.15438	0.09986	-0.1714	0.34384	0.29944	-0.26432	0.26432	0.91001	0.54503	0.0438	0.19377	0.025	4.14E-06	6.93E-05	0.00048	0.00048	yellow
FRRS1L	-0.01017	-0.11871	-0.23404	-0.12333	0.09636	-0.32508	-0.20369	0.49481	-0.49481	0.89498	0.12201	0.00206	0.10804	0.20994	1.44E-05	0.00754	6.03E-12	6.03E-12	brown
DENND1C	-0.03509	-0.04954	-0.07508	-0.01754	0.06324	0.02466	-0.20683	0.1727	-0.1727	0.64868	0.51994	0.32909	0.81988	0.4112	0.74883	0.00664	0.0239	0.0239	turquoise
OAS1	0.12362	0.03456	0.15828	0.15203	-0.07802	0.1688	0.26262	-0.32644	0.32644	0.10721	0.65361	0.03867	0.04714	0.31043	0.02732	0.00052	1.32E-05	1.32E-05	tan
PLEKHS1	-0.02323	0.01811	0.04213	0.15541	0.01232	0.20199	0.09085	-0.10814	0.10814	0.76302	0.81418	0.58428	0.04239	0.87299	0.00806	0.23731	0.15916	0.15916	yellow
UGDH	-0.05877	-0.15319	0.01662	0.05625	-0.15543	0.08716	0.10915	0.04144	-0.04144	0.44518	0.04546	0.82918	0.4649	0.04236	0.25698	0.1553	0.59047	0.59047	turquoise
XRRA1	-0.07693	-0.11112	-0.11983	0.02914	-0.08384	0.05087	0.07136	0.09813	-0.09813	0.31725	0.14791	0.11849	0.70515	0.2756	0.50878	0.35365	0.20164	0.20164	grey
TRIM47	-0.02179	-0.03904	0.07629	0.06598	-0.07071	0.27154	0.20324	-0.40106	0.40106	0.7773	0.6122	0.32129	0.39122	0.35809	0.00033	0.00767	5.45E-08	5.45E-08	yellow
KRI1	0.04575	-0.05288	-0.11565	-0.08369	0.04672	-0.03805	0.04089	-0.12607	0.12607	0.55242	0.49216	0.132	0.27645	0.54401	0.62126	0.59545	0.10036	0.10036	red
FIBP	0.07221	0.00944	-0.15175	-0.10667	0.01	-0.14269	0.05518	-0.20207	0.20207	0.34795	0.90247	0.04756	0.16495	0.89669	0.06264	0.47347	0.00804	0.00804	grey
NDUFS5	0.01387	0.03323	-0.02001	-0.04869	0.03258	-0.04798	0.06905	-0.25411	0.25411	0.85711	0.66617	0.79504	0.52713	0.67224	0.53316	0.36949	0.0008	0.0008	grey
EFCAB7	-0.02612	-0.16147	-0.14746	-0.02577	0.01781	-0.12222	-0.08701	0.25618	-0.25618	0.73451	0.03486	0.05427	0.73798	0.81712	0.11128	0.25779	0.00072	0.00072	turquoise
CFC1	0.0393	-0.10495	-0.27077	-0.18857	0.11674	-0.29864	-0.18015	0.25063	-0.25063	0.60984	0.17191	0.00034	0.01351	0.12836	7.26E-05	0.01838	0.00095	0.00095	brown
IWS1	0.0474	-0.06088	-0.00373	-0.00179	-0.06192	0.12736	0.1443	-0.06844	0.06844	0.53811	0.42894	0.96137	0.98151	0.42107	0.09693	0.0597	0.37376	0.37376	turquoise
CBLN3	-0.07019	-0.16178	-0.00932	-0.03955	0.00152	0.05417	-0.06354	0.08394	-0.08394	0.36164	0.03452	0.90371	0.60754	0.98425	0.48162	0.409	0.27502	0.27502	grey
CLK2	0.01239	-0.10289	-0.02547	-0.06925	0.15315	0.02848	0.07028	-0.25456	0.25456	0.87222	0.18051	0.74089	0.36813	0.04552	0.71155	0.361	0.00078	0.00078	red
CHTOP	0.05148	-0.0541	-0.09412	-0.12691	0.07019	0.0779	0.16454	-0.22385	0.22385	0.5037	0.48218	0.22078	0.09811	0.36165	0.31116	0.03152	0.00325	0.00325	greenyellow
STT3B	0.02853	-0.10675	-0.00411	-0.01857	-0.17339	0.11154	0.11176	0.07384	-0.07384	0.71111	0.16462	0.95747	0.80954	0.02333	0.14638	0.14558	0.33715	0.33715	turquoise
ZNF717	0.00601	-0.07856	-0.14946	-0.10454	-0.05517	-0.0217	0.03016	0.02411	-0.02411	0.93777	0.30711	0.05104	0.17358	0.47355	0.77812	0.69535	0.75427	0.75427	grey
RSU1	-0.09385	0.00833	0.07319	0.14277	-0.24696	0.23353	0.08248	0.14487	-0.14487	0.22212	0.91387	0.34145	0.06249	0.00113	0.00211	0.28352	0.05869	0.05869	black
SRSF5	-0.02138	-0.11244	-0.05827	-0.06455	0.10407	-0.04945	-0.10083	0.05012	-0.05012	0.78134	0.14315	0.44904	0.40157	0.17554	0.52068	0.18943	0.51504	0.51504	red
MCPH1	-0.04014	-0.03331	-0.03022	0.00122	-0.08215	-0.00505	0.07478	0.22101	-0.22101	0.60218	0.66539	0.69483	0.98737	0.28544	0.94776	0.331	0.00367	0.00367	turquoise
FBN2	-0.02856	-0.12417	0.12236	-0.02502	-0.16355	0.1981	0.0947	0.19166	-0.19166	0.71082	0.10563	0.11086	0.74527	0.03256	0.0094	0.21792	0.01203	0.01203	blue
AFMID	0.10002	0.06754	0.13145	0.06377	0.03001	0.24676	0.25473	-0.36716	0.36716	0.19305	0.3801	0.08657	0.40729	0.69676	0.00114	0.00077	7.83E-07	7.83E-07	yellow
SLC22A3	0.00377	0.04484	0.0226	0.09033	-0.06386	0.13625	0.15901	-0.18608	0.18608	0.96092	0.56031	0.76926	0.24001	0.40666	0.07557	0.03777	0.01482	0.01482	turquoise
NUTF2	0.02509	0.037	0.00548	-0.01923	-0.01445	0.05848	0.17706	-0.41622	0.41622	0.74461	0.6309	0.94328	0.8029	0.85116	0.44738	0.02052	1.50E-08	1.50E-08	greenyellow
EPHB4	0.07635	-0.00799	0.14016	0.09031	-0.10217	0.30512	0.34003	-0.38114	0.38114	0.32092	0.91743	0.06749	0.2401	0.18361	4.95E-05	5.36E-06	2.70E-07	2.70E-07	yellow
RPS4Y1	0.96623	-0.02709	-0.05379	-0.0243	0.01936	-0.05888	0.04534	-0.04597	0.04597	#######	0.72505	0.48472	0.75237	0.80151	0.44426	0.55592	0.55044	0.55044	grey
ANAPC2	-0.0477	-0.10606	-0.14726	-0.07361	0.12839	-0.13473	-0.06926	0.01289	-0.01289	0.53557	0.1674	0.0546	0.33864	0.09423	0.07893	0.36805	0.86711	0.86711	red
KCP	-0.01048	0.04404	0.09789	0.05918	0.05918	0.13879	0.1898	-0.40868	0.40868	0.89175	0.56733	0.20277	0.442	0.44193	0.07023	0.0129	2.87E-08	2.87E-08	yellow
C7	-0.03023	-0.04085	-0.04054	0.09396	-0.09559	0.10753	-0.07365	0.56998	-0.56998	0.69466	0.59581	0.59861	0.22157	0.2136	0.16155	0.33841	4.07E-16	4.07E-16	blue
ALG10B	0.03498	-0.11197	-0.0782	0.00645	0.01884	-0.04504	-0.00508	0.13064	-0.13064	0.64969	0.14482	0.30933	0.93328	0.80682	0.55856	0.94745	0.08854	0.08854	turquoise
PRKCI	0.04933	-0.04358	0.1118	0.04196	-0.15543	0.26615	0.23462	-0.24109	0.24109	0.52166	0.57141	0.14543	0.58582	0.04236	0.00043	0.00201	0.00149	0.00149	turquoise
CNNM4	0.07566	-0.03468	0.12736	0.04794	-0.03805	0.17329	0.14372	-0.34723	0.34723	0.32537	0.65253	0.09692	0.53354	0.62125	0.02341	0.06074	3.27E-06	3.27E-06	turquoise
CAMTA1	0.01592	-0.15407	-0.11579	-0.06997	0.04333	-0.0481	-0.08082	-0.01289	0.01289	0.83629	0.04423	0.13151	0.36313	0.57366	0.53211	0.29336	0.86707	0.86707	grey
KIRREL2	-0.03977	0.0348	-0.15997	-0.04086	0.03703	-0.20422	-0.22958	0.37717	-0.37717	0.60551	0.65132	0.03662	0.59571	0.63059	0.00738	0.00252	3.68E-07	3.68E-07	grey
EBF2	-0.07359	-0.0656	0.01243	0.09598	-0.20545	0.04055	-0.07765	0.55309	-0.55309	0.33877	0.39399	0.87185	0.21175	0.00702	0.5985	0.31273	4.35E-15	4.35E-15	blue
INTS5	-0.03859	-0.02486	-0.07098	-0.04873	-0.0941	0.03931	0.1087	-0.00785	0.00785	0.61631	0.74684	0.35623	0.52676	0.22086	0.60969	0.15702	0.91885	0.91885	turquoise
PRDM1	-0.10947	-0.05827	0.0643	0.03881	-0.18273	0.06132	0.08149	0.28704	-0.28704	0.15406	0.44903	0.4034	0.61429	0.01675	0.4256	0.28933	0.00014	0.00014	blue
CLTRN	-0.02181	-0.12721	-0.15686	0.00025	0.06474	-0.07864	-0.11846	0.31289	-0.31289	0.77707	0.09731	0.04048	0.99739	0.40021	0.30662	0.1228	3.09E-05	3.09E-05	grey
YKT6	0.01135	-0.0942	0.02204	0.08036	-0.11665	-0.00458	0.13155	-0.13682	0.13682	0.88285	0.22038	0.77479	0.29611	0.12867	0.95259	0.08634	0.07436	0.07436	turquoise
NFYC	0.00282	-0.03643	-0.06751	0.01601	-0.07454	0.08287	-0.0809	0.09847	-0.09847	0.97083	0.63616	0.3803	0.83533	0.33261	0.28122	0.29286	0.20008	0.20008	turquoise
NOC3L	-0.01331	-0.05986	0.07056	0.04766	-0.1944	0.10272	0.16905	0.08466	-0.08466	0.8628	0.4367	0.35909	0.53593	0.01084	0.18125	0.02708	0.27091	0.27091	turquoise
IL10RB	0.06505	-0.12548	0.05791	0.10819	0.09957	0.19086	0.17311	-0.21442	0.21442	0.39794	0.10198	0.45183	0.15899	0.19509	0.0124	0.02356	0.00486	0.00486	turquoise
ERFE	0.04786	0.10305	-0.0731	0.06495	-0.00305	0.00027	0.30456	-0.18892	0.18892	0.53416	0.17985	0.34202	0.39866	0.96837	0.99719	5.12E-05	0.01334	0.01334	grey
FTSJ1	-0.02661	-0.07079	-0.00525	0.04454	-0.15426	-0.02711	0.22281	-0.25443	0.25443	0.72978	0.35756	0.94569	0.56293	0.04395	0.72486	0.0034	0.00078	0.00078	grey
PAK1	0.0414	-0.01069	0.06329	-0.02587	-0.01246	0.18624	0.14835	-0.32171	0.32171	0.59079	0.88962	0.41083	0.73692	0.87148	0.01473	0.05282	1.78E-05	1.78E-05	turquoise
TMEM233	-0.0186	-0.07849	-0.05719	-0.04773	-0.07912	-0.05567	-0.09625	0.12659	-0.12659	0.80923	0.3075	0.45746	0.53527	0.30363	0.46954	0.21047	0.09896	0.09896	grey
CTRB2	0.01281	0.03753	0.07052	0.15626	-0.10246	0.10948	-0.06742	0.11053	-0.11053	0.86794	0.62603	0.35936	0.04126	0.18238	0.15403	0.38095	0.15009	0.15009	magenta
ZNF189	-0.10432	-0.18747	-0.12178	0.02079	0.00885	0.08036	-0.11324	0.26733	-0.26733	0.17453	0.01407	0.11257	0.78728	0.90855	0.29609	0.1403	0.00041	0.00041	turquoise
WRN	-0.11626	-0.03505	0.05923	0.02076	-0.14885	0.12004	0.12052	0.24241	-0.24241	0.12997	0.64906	0.44156	0.78758	0.05201	0.11784	0.11637	0.0014	0.0014	turquoise
ZMAT3	-0.0618	-0.03183	-0.05918	-0.01243	-0.16756	0.00473	0.00265	0.43725	-0.43725	0.42201	0.67938	0.44199	0.87179	0.02848	0.95105	0.97253	2.24E-09	2.24E-09	blue
LHFPL2	-0.00201	-0.02838	0.11736	0.1122	-0.22834	0.2135	0.12671	0.18567	-0.18567	0.97916	0.71251	0.12634	0.14399	0.00267	0.00505	0.09864	0.01504	0.01504	turquoise
PODXL2	0.06201	-0.1237	-0.14893	-0.06528	0.15297	-0.21035	-0.10827	0.15619	-0.15619	0.42038	0.10699	0.05188	0.39629	0.04578	0.00576	0.15865	0.04135	0.04135	brown
RASGRP3	-0.13994	-0.07233	-0.02096	0.09711	-0.1564	0.08605	-0.05515	0.38377	-0.38377	0.06793	0.34712	0.78551	0.20639	0.04107	0.2631	0.47375	2.20E-07	2.20E-07	blue
FFAR4	0.09607	0.03551	0.0141	0.01411	0.07341	0.12663	0.08689	-0.05093	0.05093	0.2113	0.64476	0.85473	0.85471	0.33997	0.09887	0.25847	0.50825	0.50825	grey
PRPF3	0.01739	-0.16285	-0.11873	-0.1074	-0.00184	-0.0029	0.1357	-0.19934	0.19934	0.82139	0.03332	0.12193	0.16204	0.98096	0.96998	0.07678	0.00895	0.00895	greenyellow
ANKRD12	-0.01574	-0.21495	0.0071	0.06198	-0.07285	0.04142	-0.01474	0.29178	-0.29178	0.83806	0.00475	0.92658	0.42063	0.34366	0.59061	0.84828	0.00011	0.00011	turquoise
TIMP3	-0.07962	-0.12708	-0.00245	0.10227	-0.21955	0.12042	0.08662	0.35092	-0.35092	0.30056	0.09766	0.97465	0.18317	0.00391	0.11668	0.25994	2.53E-06	2.53E-06	black
DLD	-0.03901	-0.03261	0.02725	-0.02857	0.00046	0.02208	-0.01719	0.14094	-0.14094	0.61241	0.67199	0.7235	0.71069	0.99524	0.77434	0.82337	0.06596	0.06596	turquoise
NBPF19	0.01002	-0.07771	0.00383	0.06694	-0.13773	0.08099	0.17338	0.11656	-0.11656	0.89648	0.31237	0.96035	0.38437	0.07244	0.29231	0.02334	0.12896	0.12896	turquoise
SSBP2	0.0236	-0.17466	-0.13418	-0.05095	0.01328	-0.17481	-0.26592	0.37289	-0.37289	0.75935	0.02232	0.08018	0.50808	0.86312	0.02221	0.00044	5.09E-07	5.09E-07	turquoise
DPEP2	-0.0849	-0.00271	0.10367	0.06486	-0.15322	0.09697	-0.04825	0.38813	-0.38813	0.26954	0.97189	0.17723	0.3993	0.04542	0.20705	0.53083	1.56E-07	1.56E-07	blue
DBNL	-0.01018	-0.02357	0.01482	-0.00182	0.04383	0.02961	0.0587	-0.14033	0.14033	0.89488	0.75956	0.84748	0.9811	0.56919	0.70067	0.4457	0.06715	0.06715	grey
ZXDC	-0.01772	-0.07072	-0.2213	-0.06675	0.00954	-0.00061	0.0151	0.03322	-0.03322	0.81804	0.35869	0.00363	0.38574	0.90141	0.99366	0.84464	0.66626	0.66626	turquoise
PPP1R9B	-0.02132	-0.20025	-0.09387	0.00553	-0.0173	-0.07095	-0.10108	0.23634	-0.23634	0.78199	0.00864	0.22203	0.9428	0.8223	0.35646	0.18837	0.00186	0.00186	turquoise
CCDC181	0.00125	-0.21828	-0.16514	-0.12115	0.14604	-0.28139	-0.16154	0.30963	-0.30963	0.98706	0.00413	0.03089	0.11445	0.05665	0.00019	0.03479	3.77E-05	3.77E-05	brown
CCNE2	-0.09015	-0.03603	-0.01422	-0.04122	-0.03517	-0.03331	0.16508	-0.11587	0.11587	0.24093	0.63989	0.85354	0.59247	0.64795	0.66537	0.03095	0.13126	0.13126	turquoise
SDC4	0.061	0.00168	0.08187	0.06108	-0.13407	0.30691	0.23375	-0.48692	0.48692	0.42806	0.98263	0.28711	0.42741	0.08043	4.45E-05	0.00209	1.45E-11	1.45E-11	yellow
NAGLU	-0.05296	0.03729	-0.19492	-0.08565	0.15557	-0.22177	-0.03477	0.1532	-0.1532	0.49151	0.62823	0.01063	0.26536	0.04217	0.00356	0.65162	0.04544	0.04544	grey

KITLG	-0.03297	-0.0934	0.08768	0.12601	-0.12639	0.21428	0.11791	0.02015	-0.02015	0.66859	0.22436	0.25411	0.10053	0.09952	0.00489	0.12456	0.79368	0.79368	turquoise
PSMB1	-0.02479	0.0097	-0.03596	-0.04932	0.00753	-0.10952	0.08255	-0.13008	0.13008	0.74761	0.89985	0.64058	0.52179	0.92214	0.1539	0.2831	0.08994	0.08994	grey
FGA	0.02955	-0.09898	-0.16501	0.08866	0.06097	-0.02045	-0.05595	-0.07824	0.07824	0.70119	0.19776	0.03102	0.24886	0.42829	0.79066	0.46734	0.30908	0.30908	grey
MYOM3	-0.01036	-0.01705	-0.0003	0.12661	-0.01456	0.1011	0.01856	-0.1583	0.1583	0.89299	0.82481	0.99686	0.09892	0.85006	0.18828	0.80958	0.03865	0.03865	grey
AMPH	0.00581	-0.13758	-0.11583	0.04752	0.00085	-0.2492	-0.11755	0.56919	-0.56919	0.93992	0.07274	0.13139	0.53715	0.99117	0.00101	0.12571	4.56E-16	4.56E-16	brown
WAC	0.00462	-0.18945	0.00677	0.02863	-0.09418	0.03633	-0.01433	0.20167	-0.20167	0.95215	0.01308	0.92992	0.71007	0.2205	0.63711	0.85238	0.00817	0.00817	turquoise
APH1A	0.05756	0.01952	0.00972	-0.04782	-0.07114	0.1677	0.20795	-0.36777	0.36777	0.45462	0.79994	0.89961	0.53456	0.35517	0.02835	0.00635	7.48E-07	7.48E-07	turquoise
IGSF6	-0.12767	-0.00812	0.06922	0.04248	-0.18797	0.17992	0.07728	0.37415	-0.37415	0.09611	0.916	0.36832	0.58114	0.01382	0.01854	0.31509	4.63E-07	4.63E-07	blue
ATP6V1E1	-0.02621	-0.07496	-0.11431	-0.08388	-0.05265	-0.10761	-0.00899	0.05753	-0.05753	0.7336	0.32984	0.13655	0.2754	0.49407	0.16124	0.90708	0.45481	0.45481	turquoise
SMS	-0.03608	-0.08385	-0.0453	-0.01928	-0.15037	0.00867	0.21135	-0.36114	0.36114	0.63944	0.27556	0.5563	0.80234	0.04964	0.91037	0.00552	1.22E-06	1.22E-06	pink
ITGB4	0.02437	0.05104	0.14265	0.06641	-0.08904	0.26296	0.30272	-0.44278	0.44278	0.75175	0.50736	0.06271	0.3881	0.24684	0.00051	5.71E-05	1.33E-09	1.33E-09	yellow
TOX3	0.06088	-0.03827	-0.05069	-0.01775	0.12509	0.06532	-0.11221	-0.14342	0.14342	0.42897	0.61921	0.51026	0.8178	0.10306	0.396	0.14397	0.06128	0.06128	grey
METTL3	-0.04469	-0.14531	-0.06894	-0.02364	0.02761	-0.09593	-0.10101	0.08218	-0.08218	0.56164	0.05792	0.37027	0.75891	0.71998	0.21199	0.18867	0.28525	0.28525	red
SERAC1	-0.03214	-0.14309	-0.0187	0.13521	-0.01679	-0.01386	-0.02395	0.29356	-0.29356	0.67642	0.0619	0.80817	0.07785	0.82746	0.85721	0.75585	9.75E-05	9.75E-05	magenta
ZNF496	-0.07567	-0.03166	-0.03935	-0.00868	-0.16776	0.07029	0.15652	0.12358	-0.12358	0.3253	0.68099	0.6094	0.91026	0.02829	0.36092	0.04092	0.10732	0.10732	turquoise
UBE2J2	-0.11442	-0.06587	-0.11677	0.0079	0.127	-0.0792	-0.0217	-0.07456	0.07456	0.1362	0.39198	0.12828	0.91827	0.09786	0.30316	0.77812	0.33242	0.33242	green
SLIRP	0.14173	0.06984	-0.12254	-0.0874	0.17322	-0.20071	0.03523	-0.35922	0.35922	0.06445	0.36401	0.11033	0.25566	0.02347	0.00848	0.64736	1.40E-06	1.40E-06	grey
ETAA1	-0.1418	-0.09902	-0.11186	-0.00873	-0.06174	-0.04133	0.09853	0.06465	-0.06465	0.0643	0.19755	0.14521	0.90973	0.42245	0.5915	0.1998	0.40089	0.40089	turquoise
NR4A3	-0.11362	-0.14925	0.01559	0.06289	-0.05305	0.06933	-0.06478	0.53068	-0.53068	0.13897	0.05138	0.83959	0.4138	0.49074	0.36758	0.39988	8.28E-14	8.28E-14	blue
ITGB2	-0.10793	-0.01725	0.13517	0.10431	-0.22045	0.22342	0.14477	0.23789	-0.23789	0.15998	0.82282	0.07796	0.17453	0.00376	0.00331	0.05887	0.00173	0.00173	blue
IPPK	-0.05468	-0.17533	-0.07025	-0.02887	-0.12248	-0.04638	0.00024	0.18099	-0.18099	0.47752	0.02181	0.36125	0.7078	0.11052	0.54689	0.99752	0.01784	0.01784	turquoise
AC011479	0.13994	0.02483	0.06478	0.09082	-0.02755	0.12268	0.16618	-0.25287	0.25287	0.06792	0.74714	0.39988	0.23745	0.72059	0.10992	0.02984	0.00085	0.00085	grey
SNAP29	-0.08105	-0.04371	-0.03921	0.00049	-0.08929	0.0539	0.04607	0.03947	-0.03947	0.29195	0.57029	0.61063	0.99491	0.24551	0.48378	0.54957	0.60824	0.60824	turquoise
LAMA2	-0.00038	-0.05778	0.03597	0.09819	-0.24568	0.1812	0.02324	0.44388	-0.44388	0.99611	0.45282	0.64046	0.20138	0.0012	0.0177	0.76286	1.20E-09	1.20E-09	blue
SCARB1	0.00591	-0.06671	-0.05291	-0.04403	0.10983	-0.21834	-0.06854	0.0277	-0.0277	0.93883	0.38598	0.49187	0.56745	0.15272	0.00412	0.37305	0.71909	0.71909	grey
THOP1	-0.00822	0.04149	-0.1595	-0.13234	0.21902	-0.25069	0.03841	-0.16077	0.16077	0.91498	0.59001	0.03718	0.08444	0.004	0.00094	0.6179	0.03567	0.03567	green
SS18L1	0.00921	-0.17178	-0.10245	-0.05102	0.1623	-0.18547	-0.05736	-0.01955	0.01955	0.90483	0.02467	0.18241	0.50755	0.03393	0.01516	0.45619	0.79962	0.79962	red
SNAI3	-0.04947	-0.03778	-0.09434	-0.09634	-0.0121	-0.00945	-0.1	0.27875	-0.27875	0.52052	0.62373	0.21971	0.21005	0.87519	0.90235	0.19313	0.00022	0.00022	blue
ZGPAT	0.02566	-0.02346	-0.03943	-0.02654	0.2062	-0.05452	0.04817	-0.23182	0.23182	0.73905	0.76073	0.60866	0.73044	0.00682	0.47882	0.53157	0.00228	0.00228	green
UBE2G1	-0.06376	-0.11293	-0.01894	-0.00924	-0.03753	-0.05584	-0.06413	0.30278	-0.30278	0.40742	0.14138	0.80579	0.90449	0.62604	0.46817	0.40469	5.69E-05	5.69E-05	turquoise
CRTAM	-0.0687	-0.02128	0.06568	0.09014	-0.20601	0.15414	0.0389	0.43376	-0.43376	0.37192	0.78235	0.39336	0.24103	0.00687	0.04412	0.61343	3.10E-09	3.10E-09	blue
ERH	-0.02528	-0.08336	-0.08817	-0.05376	0.04847	-0.09768	0.01177	-0.17384	0.17384	0.74277	0.2784	0.25149	0.48494	0.52896	0.20373	0.8786	0.02297	0.02297	turquoise
TNC	-0.05409	-0.07417	-0.00134	0.00685	-0.16836	0.05349	0.08275	0.19447	-0.19447	0.4823	0.33499	0.98611	0.92912	0.02772	0.48717	0.28194	0.01081	0.01081	blue
RAD51D	-0.10708	-0.06069	-0.08315	-0.00577	0.03759	-0.16435	-0.12007	0.25938	-0.25938	0.1633	0.43042	0.2796	0.94034	0.62551	0.03171	0.11775	0.00061	0.00061	turquoise
GPR3	-0.13937	-0.07754	-0.06418	-0.02093	-0.10736	-0.03396	-0.00707	0.14191	-0.14191	0.06906	0.31344	0.40433	0.78586	0.1622	0.65927	0.92686	0.06411	0.06411	grey
CRKL	-0.05365	-0.14514	-0.07031	0.00317	-0.00967	-0.03904	-0.09381	0.18287	-0.18287	0.48584	0.05822	0.36081	0.96722	0.90015	0.61215	0.22231	0.01666	0.01666	turquoise
NATD1	-0.06995	-0.13674	-0.12552	0.025	-0.13756	-0.08755	-0.0811	0.45632	-0.45632	0.36331	0.07453	0.10188	0.74547	0.07279	0.25485	0.29165	3.55E-10	3.55E-10	turquoise
NUSAP1	-0.06963	0.04669	0.03366	-0.01175	-0.07712	0.11593	0.27951	-0.3955	0.3955	0.36546	0.54428	0.66205	0.87876	0.31609	0.13104	0.00021	8.61E-08	8.61E-08	pink
PPAN	-0.00313	0.0855	-0.12746	-0.06931	0.15927	-0.04929	0.06361	-0.1921	0.1921	0.96756	0.26619	0.09664	0.36772	0.03746	0.52201	0.40846	0.01183	0.01183	green
ARID3A	-0.00292	-0.1626	-0.07469	-0.1003	0.15155	-0.14635	-0.11133	0.16873	-0.16873	0.96981	0.0336	0.33161	0.19182	0.04785	0.05613	0.14715	0.02737	0.02737	grey
IQCC	-0.01322	-0.12831	-0.18056	-0.17963	0.20533	-0.18381	-0.01349	-0.01369	0.01369	0.86373	0.09442	0.01811	0.01873	0.00706	0.0161	0.86099	0.85898	0.85898	grey
PTGFR	-0.00406	-0.07221	-0.12147	0.05868	-0.08017	0.01585	-0.03154	0.54768	-0.54768	0.95801	0.34798	0.1135	0.44582	0.29724	0.83699	0.68215	9.04E-15	9.04E-15	blue
C2orf49	-0.01657	-0.10439	0.02877	0.01752	-0.1502	0.12377	0.11119	0.06142	-0.06142	0.82969	0.17421	0.70873	0.82011	0.04989	0.10678	0.14768	0.42485	0.42485	turquoise
PEX7	-0.04395	0.06931	-0.04381	-0.10468	0.03031	-0.04932	-0.08744	0.02676	-0.02676	0.56815	0.36773	0.5694	0.17303	0.69388	0.52178	0.25544	0.72831	0.72831	grey
MLST8	0.0647	0.09233	-0.10841	-0.06762	0.15621	-0.1278	0.04444	-0.1453	0.1453	0.40052	0.2297	0.15814	0.37949	0.04132	0.09576	0.56387	0.05793	0.05793	green
PLA2G12A	-0.10321	-0.14008	-0.1317	-0.05711	0.05521	-0.13737	-0.13411	0.2822	-0.2822	0.17915	0.06764	0.08597	0.45815	0.47325	0.07318	0.08034	0.00018	0.00018	turquoise
MAPK14	-0.02298	-0.07586	0.06094	0.07055	-0.12268	0.14715	0.10798	0.10024	-0.10024	0.76547	0.32408	0.42846	0.35919	0.10994	0.05479	0.15981	0.19206	0.19206	turquoise
KCNK17	-0.14319	-0.09544	-0.24903	-0.1572	0.0525	-0.17293	-0.13839	0.40779	-0.40779	0.06171	0.21435	0.00102	0.04004	0.49522	0.02371	0.07107	3.10E-08	3.10E-08	brown
CGB5	0.10887	0.07098	0.02455	0.07694	-0.06309	0.01286	0.06619	-0.19144	0.19144	0.15635	0.35626	0.74997	0.31722	0.41237	0.86737	0.38972	0.01213	0.01213	grey
UBXN11	0.01475	-0.02849	-0.13171	-0.03676	0.24962	-0.12012	-0.05299	-0.16965	0.16965	0.84813	0.71144	0.08595	0.63314	0.00099	0.11761	0.49123	0.02654	0.02654	red
RNF20	0.04269	-0.12524	-0.09133	-0.03502	-0.15869	-0.01422	0.0103	0.23789	-0.23789	0.57932	0.10265	0.23485	0.6493	0.03816	0.85359	0.89361	0.00173	0.00173	turquoise
RREB1	0.00112	-0.12946	0.00068	0.0246	-0.01467	0.08018	0.06515	-0.09264	0.09264	0.98837	0.09149	0.99296	0.74947	0.84893	0.2972	0.3972	0.22813	0.22813	turquoise
MYOT	-0.00092	-0.13236	-0.04137	0.07338	-0.0555	-0.10087	-0.12731	0.40094	-0.40094	0.99052	0.0844	0.59114	0.34018	0.47093	0.18926	0.09705	5.50E-08	5.50E-08	grey
CRYBB3	0.00135	-0.10722	-0.02314	0.03129	0.2002	-0.02271	0.03686	-0.25372	0.25372	0.98603	0.16278	0.76391	0.68452	0.00865	0.76816	0.63221	0.00081	0.00081	grey
THY1	-0.07783	-0.05002	0.01858	0.04871	-0.15096	0.11301	0.04276	0.31375	-0.31375	0.31162	0.51585	0.80938	0.52695	0.04874	0.1411	0.57869	2.93E-05	2.93E-05	black
TMED9	0.04521	-0.07006	-0.0703	0.04149	-0.00985	-0.08841	0.15082	-0.25625	0.25625	0.55709	0.36256	0.36085	0.58999	0.89826	0.25022	0.04895	0.00072	0.00072	grey
ARL6IP4	0.02046	-0.01965	-0.05229	-0.08432	0.26012	-0.16961	-0.0955	-0.16341	0.16341	0.7905	0.7986	0.49696	0.27286	0.00059	0.02657	0.21403	0.03271	0.03271	red
POLR3G	0.02254	0.03609	-0.04579	0.01827	0.03168	-0.14587	0.11461	-0.04713	0.04713	0.76978	0.63935	0.55202	0.81251	0.68079	0.05695	0.13551	0.54045	0.54045	grey
GPRASP1	-0.02201	-0.18715	-0.21563	-0.1009	0.04615	-0.25021	-0.27755	0.56802	-0.56802	0.77505	0.01424	0.00462	0.18915	0.54892	0.00096	0.00024	5.39E-16	5.39E-16	brown
ADCY3	-0.14719	-0.13626	-0.00343	0.09192	-0.11627	0.01679	0.00521	0.41336	-0.41336	0.05472	0.07555	0.96449	0.23183	0.12991	0.82744	0.94609	1.92E-08	1.92E-08	blue
NT5DC2	-0.08258	-0.04123	0.04215	0.02999	-0.10588	0.01362	0.1345	0.08998	-0.08998	0.28293	0.59233	0.58416	0.69697	0.16813	0.85971	0.07945	0.24186	0.24186	black
BAZ1B	-0.01464	-0.21525	-0.0689	-0.02384	-0.09355	-0.01949	-0.01337	0.16957	-0.16957	0.84928	0.00469	0.37058	0.75697	0.22361	0.80021	0.86217	0.02661	0.02661	turquoise
C9orf85	0.06504	-0.09598	-0.0591	-0.01395	-0.00769	0.02391	0.06877	0.06318	-0.06318	0.39802	0.21175	0.44258	0.85625	0.92049	0.75621	0.37147	0.41171	0.41171	turquoise
RAVER2	-0.07046	-0.13783	-0.01152	0.03053	0.17584	0.00615	-0.06988	0.07304	-0.07304	0.35979	0.07221	0.88108	0.69179	0.02142	0.93638	0.36376	0.34239	0.34239	grey
PRPSAP1	-0.00531	-0.18488	0.0318	-0.01709	0.03928	-0.07188	0.0749	0.0155	-0.0155	0.94506	0.01549	0.67964	0.82441	0.60996	0.35014	0.33024	0.84049	0.84049	grey
FAM13C	-0.11973	-0.12203	-0.08239	0.06844	-0.07209	-0.05318	-0.07975	0.63819	-0.63819	0.11879	0.11184	0.28404	0.37374	0.34876	0.4897	0.29983	6.09E-21	6.09E-21	blue
CYP1B1	-0.05583	-0.06168	0.02148	0.05606	-0.24039	0.1506	-0.01246	0.44246	-0.44246	0.46827	0.42287	0.78039	0.46648	0.00154	0.04928	0.87149	1.37E-09	1.37E-09	blue
NAB1	0.02263	-0.03082	0.05123	0.0515	-0.05843	0.15162	0.18618	-0.11206	0.11206	0.76897	0.68903	0.50581	0.5035	0.4478	0.04774	0.01477	0.14449	0.14449	turquoise
TENM2	-0.16756	-0.14342	-0.15424	-0.22171	-0.09082	-0.1079	0.02794	0.40669	-0.40669	0.02848	0.06129	0.04399	0.00356	0.23747	0.16009	0.71675	3.40E-08	3.40E-08	grey
ZMYND11	-0.02205	-0.16079	-0.03038	0.01844	-0.06417	-0.02671	-0.12562	0.37381	-0.37381	0.7747	0.03566	0.69321	0.81076	0.40435	0.72875	0.1016	4.75E-07	4.75E-07	turquoise
GOLGA7B	0.05927	-0.13451	-0.24485	-0.0711	0.16954	-0.25449	-0.31032	0.0697	-0.0697	0.44131	0.07943	0.00125	0.3554	0.02664	0.00078	3.62E-05	0.36501	0.36501	brown
COX6A1	0.12066	0.01582	-0.04845	-0.07259	0.28493	-0.15187	-0.04708	-0.41843	0.41843	0.11593	0.83732	0.52913	0.34544	0.00016	0.04738	0.54089	1.23E-08	1.23E-08	grey
CDK2AP2	0.10497	0.01171	-0.01364	0.005	8.67E-05	0.0637	0.16042	-0.49628	0.49628	0.17181	0.87916	0.8595	0.94824	0.9991	0.40782	0.03608	5.10E-12	5.10E-12	yellow
GBF1	0.0378	-0.13954	-0.05834	-0.04609	-0.019	-0.09744	0.00599	0.06795	-0.06795	0.6235	0.06872	0.44847	0.54941	0.80516	0.20486	0.93804	0.3772	0.3772	turquoise
CEP85L	-0.06595	-0.10608	0.03306	-0.00904	-0.13254	0.00416	-0.04952	0.44045	-0.44045	0.39142	0.16732	0.66776	0.90655	0.08398	0.95697	0.52012	1.66E-09	1.66E-09	blue
RAB15	0.08296	-0.14228	0.07155	0.08244	0.12912	-0.04343	-0.02832	-0.07202	0.07202	0.28073	0.0634	0.3524	0.28375	0.09236	0.57274	0.71311	0.34925	0.34925	grey
CDH17	0.05829	0.04954	0.01862	0.04631	0.14529	0.10111	0.11276	-0.07764	0.07764	0.44885	0.5199	0.80899	0.54757	0.05795	0.18823	0.14199	0.31282	0.31282	grey
CCNQ	-0.03919	-0.06178	-0.18835	-0.11451	0.08954	-0.18674	0.07959	-0.18983	0.18983	0.6108	0.42216	0.01363	0.13587	0.24419	0.01446	0.30075	0.01289	0.01289	grey
UBL4A	0.02895	-0.06428	-0.07348	-0.04073	-0.12333	0.03379	0.20396	-0.22086	0.22086	0.70697	0.40357	0.33949	0.59687	0.10804	0.66083	0.00746	0.0037	0.0037	grey
DPP4	-0.04378	-0.06635	0.04455	0.04974	0.04589	-0.09571	-0.10719	0.18521	-0.18521	0.56961	0.38856	0.56284	0.51821	0.55117	0.21303	0.16287	0.0153	0.0153	grey
TFB1M	-0.06535	0.05851	0.08428	0.03128	-0.07055	0.03087	0.11535	-0.05125	0.05125	0.39573	0.44714	0.27307	0.68461	0.35918	0.68855	0.133	0.50563	0.50563	grey
HOXB7	-0.03062	0.07738	0.0142	0.06998	-0.02293	0.14186	0.26558	-0.2891	0.2891	0.69097	0.31445	0.85371	0.36306	0.76593	0.06419	0.00045	0.00013	0.00013	grey
MBOAT2	0.09842	-0.02778	0.03747	0.0163	0.01849	-0.0155	0.12341	-0.3211	0.3211	0.20032	0.71834	0.6266	0.83242	0.81033	0.84053	0.1078	1.85E-05	1.85E-05	turquoise
FAM210B	-0.01325	-0.13928	-0.04028	-0.03371	-0.07548	-0.06857	-0.02423	0.05675	-0.05675	0.86346	0.06924	0.60095	0.66155	0.32651	0.37288	0.75307	0.46098	0.46098	turquoise
SMO	-0.09038	-0.10886	-0.01729	0.12354	-0.23037	0.10734	0.068	0.32838	-0.32838	0.23977	0.15641	0.82241	0.10743	0.00244	0.16228	0.37687	1.16E-05	1.16E-05	black
ABL2	-0.06671	-0.18789	-0.01449	0.06468	-0.19352	0.0597	0.03573	0.39599	-0.39599	0.38597	0.01386	0.85077	0.40063	0.01121	0.43796	0.64266	8.27E-08	8.27E-08	blue
SLC8A1	-0.06233	-0.07378	-0.07314	-0.05677	-0.077	-0.13255	-0.12438	0.57066	-0.57066	0.41803	0.33752	0.34179	0.46082	0.31682	0.08394	0.10505	3.68E-16	3.68E-16	turquoise
NPTX2	-0.02727	-0.07196	-0.11437	0.05359	-0.04891	-0.15542	-0.07218	0.36263	-0.36263	0.72334	0.34961	0.13637	0.48632	0.52525	0.04237	0.34814	1.09E-06	1.09E-06	brown
TFF2	0.02294	0.07329	0.17649	0.04596	0.06406	0.21589	0.17592	-0.31089	0.31089	0.76589	0.34077	0.02094	0.55054	0.40517	0.00457	0.02136	3.49E-05	3.49E-05	yellow
YEATS4	0.02777	-0.07775	-0.0724	0.02504	0.14811	-0.16479	-0.13644	0.02911	-0.02911	0.71842	0.31213	0.34669	0.74512	0.0532	0.03125	0.07517	0.70544	0.70544	turquoise
GRB14	-0.03978	-0.01805	-0.17582	0.06444	0.06364	-0.0767	-0.0694	0.1933	-0.1933	0.60543	0.81474	0.02144	0.40242	0.40824	0.31873	0.36709	0.01131	0.01131	magenta
WASHC4	-0.01668	-0.12777	0.05645	0.06187	-0.09043	0.05903	0.02916	0.2507	-0.2507	0.82855	0.09585	0.46334	0.42147	0.23951	0.44316	0.70496	0.00094	0.00094	turquoise
PAK3	-0.02586	-0.12476	-0.19347	-0.0557	0.05704	-0.2587	-0.24225	0.48688	-0.48688	0.73707	0.104	0.01123	0.46933	0.45869	0.00063	0.00141	1.46E-11	1.46E-11	brown
SIRT5	-0.02891	0.00425	-0.03412	-0.00366	0.01871	0.08166	0.09384	-0.12738	0.12738	0.70739	0.95603	0.6577	0.96215	0.80804	0.28836	0.22216	0.09685	0.09685	grey
HUS1	-0.04455	-0.18418	-0.03551	0.07546	-0.01457	-0.02005	0.03373	-0.00011	0.00011	0.56286	0.01589	0.64477	0.32662	0.84994	0.79467	0.66145	0.99882	0.99882	turquoise
PPIL4	-0.07455	-0.17063	0.05445	0.05718	-0.09578	0.10028	-0.04346	0.26366	-0.26366	0.33248	0.02566	0.47933	0.45755	0.2127	0.19191	0.57252	0.00049	0.00049	turquoise
PLIN3	0.04501	-0.06291	0.11688	0.08067	-0.12933	0.09108	0.1787	-0.17718	0.17718	0.55886	0.41369	0.12789	0.29425	0.09181	0.23612	0.01936	0.02044	0.02044	turquoise
PRMT9	-0.12479	-0.16036	-0.09572	-0.05317	0.02692	-0.04504	-0.11588	0.40402	-0.40402	0.1039	0.03616	0.213	0.48973	0.72671	0.55862	0.13122	4.26E-08	4.26E-08	turquoise
CHST4	0.02347	-0.01218	0.00389	0.07642	-0.04463	0.16527	0.01231	-0.03445	0.03445	0.76062	0.87441	0.95971	0.3205	0.56216	0.03075	0.87304	0.6546	0.6546	magenta
ANKDD1B	0.10825	-0.0973	-0.00773	0.063	0.22708	0.05994	-0.02184	-0.09053	0.09053	0.15876	0.20552	0.92009	0.41301	0.00282	0.43608	0.77677	0.23898	0.23898	grey
CCDC57	-0.00206	-0.12747	-0.15848	-0.09864	0.27615	-0.21127	-0.02097	-0.12808	0.12808	0.97868	0.09661	0.03843	0.19932	0.00026	0.00554	0.78548	0.09504	0.09504	red
KDM4A	-0.01296	-0.12727	-0.00202	0.03608	-0.0416	0.03443	-0.03496	0.16002	-0.16002	0.86645	0.09715	0.97904	0.63946	0.589	0.65484	0.64983	0.03655	0.03655	turquoise
PHETA2	-0.05599	0.05084	0.07567	0.10944	-0.13013	0.13977	0.17848	-0.0433	0.0433	0.46701	0.509	0.32531	0.1542	0.0898	0.06827	0.01951	0.57389	0.57389	black
ANG	-0.00838	-0.00724	-0.0055	-0.04697	0.12881	0.1839	-0.01688	-0.30228	0.30228	0.9134	0.92517	0.94312	0.5418	0.09315	0.01605	0.82652	5.86E-05	5.86E-05	grey
PHYHD1	-0.00546	0.02756	0.0964	0.21481	-0.13341	0.22861	0.13095	0.00121	-0.00121	0.9435	0.72052	0.20974	0.00478	0.08194	0.00263	0.08779	0.98743	0.98743	grey
DCUN1D1	-0.05677	-0.13148	-0.02912	0.05294	-0.13751	0.11058	0.10747	0.07469	-0.07469	0.46082	0.0865	0.7054	0.49162	0.0729	0.1499	0.16179	0.33161	0.33161	turquoise
NR2C2	-0.07485	-0.16807	-0.05569	-0.03558	-0.11334	0.04668	-0.04346	0.26282	-0.26282	0.33058	0.028	0.46936	0.64408	0.13994	0.54437	0.57247	0.00052	0.00052	turquoise
LCP1	-0.14044	-0.01958	-0.05103	-0.03428	-0.19822	0.05326	0.04073	0.35188	-0.35188	0.06694	0.7993	0.5074	0.6562	0.00935	0.48906	0.59688	2.36E-06	2.36E-06	blue
CAAP1	0.00084	-0.03565	-0.10743	-0.08451	-0.01641	-0.044	-0.01725	0.10369	-0.10369	0.99126	0.64338	0.16194	0.27176	0.83131	0.56769	0.82277	0.17713	0.17713	turquoise
SDHD	-0.07457	0.0139	0.06296	0.02567	-0.09221	0.1513	0.07551	-0.04815	0.04815	0.33237	0.85681	0.41334	0.73893	0.23033	0.04823	0.32629	0.53171	0.53171	turquoise
METAP2	-0.00313	-0.08041	0.01492	-0.04772	-0.07426	0.03303	-0.0198	0.2105	-0.2105	0.96758	0.2958	0.84641	0.53538	0.33443	0.66803	0.7971	0.00572	0.00572	turquoise
MET	0.02438	-0.04199	0.12049	0.08626	-0.17213	0.22443	0.26217	-0.38028	0.38028	0.75159	0.58555	0.11647	0.26194	0.02437	0.00317	0.00053	2.89E-07	2.89E-07	yellow
PLBD2	0.00198	-0.17337	0.02153	0.04428	-0.10362	-0.08516	-0.12292	0.38127	-0.38127	0.97954	0.02335	0.77989	0.56521	0.17744	0.26808	0.10923	2.68E-07	2.68E-07	turquoise
CHMP1B	-0.06344	-0.22876	0.01213	0.03077	0.01072	0.06869	0.02849	0.01264	-0.01264	0.40978	0.00262	0.87489	0.68955	0.88935	0.37201	0.71146	0.8697	0.8697	turquoise
COLEC12	-0.13769	-0.05738	0.09714	0.12086	-0.16393	0.15196	0.10363	0.45057	-0.45057	0.0725	0.45603	0.20627	0.11533	0.03216	0.04724	0.17741	6.26E-10	6.26E-10	blue
ZNF385A	-0.03725	-0.03671	0.05399	0.13408	-0.0809	0.06638	0.06246	0.09635	-0.09635	0.62861	0.6336	0.48313	0.08041	0.29286	0.38834	0.41704	0.20997	0.20997	blue
NOL4	-0.03828	-0.10762	-0.23497	-0.15623	0.07213	-0.29516	-0.20953	0.44778	-0.44778	0.61911	0.16121	0.00198	0.0413	0.34849	8.89E-05	0.00595	8.21E-10	8.21E-10	brown
BRWD1	-0.02966	-0.1751	-0.10948	-0.03253	0.10152	-0.05709	-0.07166	0.20112	-0.20112	0.70012	0.02198	0.15403	0.67278	0.18642	0.45826	0.35166	0.00835	0.00835	turquoise
CNIH1	-0.0075	-0.13575	-0.0034	0.02937	-0.00236	-0.01345	-0.01971	0.0705	-0.0705	0.92248	0.07668	0.96483	0.70293	0.97556	0.86137	0.79803	0.35949	0.35949	turquoise
WDR35	-0.06383	-0.09656	-0.16917	-0.04434	-0.04582	-0.08018	-0.06365	0.37236	-0.37236	0.40687	0.20898	0.02697	0.56469	0.55175	0.29722	0.40819	5.30E-07	5.30E-07	turquoise
FGD3	-0.0257	-0.03317	0.10394	0.14247	-0.18004	0.16944	0.04597	0.29741	-0.29741	0.73864	0.66665	0.17608	0.06305	0.01845	0.02673	0.55052	7.80E-05	7.80E-05	blue
PARD3B	-0.04007	-0.07061	0.0525	0.06236	-0.16467	0.25618	-0.00446	0.32794	-0.32794	0.60285	0.35876	0.49526	0.41775	0.03138	0.00072	0.95381	1.19E-05	1.19E-05	blue
CD22	-0.05989	-0.02937	-0.00805	-0.02655	-0.08443	0.01803	-0.09052	0.36967	-0.36967	0.43648	0.703	0.91675	0.73031	0.27221	0.81491	0.23903	6.49E-07	6.49E-07	blue
PARVG	-0.11114	0.04338	0.06116	0.06456	-0.16826	0.11504	0.00362	0.38916	-0.38916	0.14784	0.57318	0.42684	0.40151	0.02782	0.13406	0.96252	1.44E-07	1.44E-07	blue
TM2D3	-0.08947	-0.25895	-0.19979	-0.09155	0.04003	-0.18286	-0.15927	0.18898	-0.18898	0.24454	0.00063	0.0088	0.23367	0.60317	0.01667	0.03745	0.01331	0.01331	turquoise
MANSC1	-0.00362	-0.08066	-0.0583	-0.06659	-0.04857	0.01586	0.03513	-0.22158	0.22158	0.96247	0.2943	0.44879	0.38686	0.52811	0.83683	0.64827	0.00359	0.00359	grey
ABCG1	-0.04939	-0.1979	0.0336	0.01838	0.03762	-0.05442	-0.066	0.06694	-0.06694	0.52119	0.00947	0.66263	0.81142	0.62522	0.47958	0.39105	0.38437	0.38437	grey
TUB	-0.02857	-0.18074	-0.1692	-0.03273	-0.01632	-0.19845	-0.14002	0.61096	-0.61096	0.71073	0.018	0.02695	0.67081	0.83224	0.00927	0.06776	7.08E-19	7.08E-19	brown
THAP3	0.06085	-0.13998	-0.11978	0.01662	0.08252	-0.03735	0.02271	-0.04708	0.04708	0.42914	0.06785	0.11865	0.8292	0.28326	0.62769	0.76813	0.54088	0.54088	grey

SLC6A17	-0.02557	-0.11311	-0.25277	-0.10881	0.03343	-0.25061	-0.15724	0.44118	-0.44118	0.73991	0.14077	0.00085	0.15658	0.66426	0.00095	0.03999	1.55E-09	1.55E-09	brown
KYAT3	-0.03427	-0.07012	0.12154	-0.04276	-0.08984	0.19681	0.10955	-0.01198	0.01198	0.65631	0.36215	0.11329	0.57865	0.2426	0.00988	0.15377	0.87637	0.87637	turquoise
RANBP6	-0.06663	-0.14883	-0.08313	-0.01764	-0.06403	-0.08704	-0.03038	0.28733	-0.28733	0.38658	0.05205	0.27974	0.81885	0.40537	0.25762	0.69325	0.00014	0.00014	turquoise
NUFIP1	0.0124	0.03391	-0.05483	-0.03451	-0.10159	0.00307	0.04645	0.02024	-0.02024	0.87214	0.65968	0.47633	0.65409	0.1861	0.96822	0.54635	0.79269	0.79269	turquoise
MFSD4B	-0.04673	-0.13328	-0.01949	-0.01181	-0.03378	0.08222	0.06389	0.13521	-0.13521	0.54392	0.08224	0.80021	0.87812	0.66095	0.28506	0.4064	0.07785	0.07785	turquoise
BTK	-0.11812	-0.02168	0.08406	0.0414	-0.22191	0.1498	0.00219	0.41242	-0.41242	0.12388	0.77837	0.27436	0.59079	0.00353	0.05051	0.97734	2.08E-08	2.08E-08	blue
HSF1	0.10937	-0.00555	-0.00752	-0.03226	-0.02982	-0.02717	0.1308	-0.39877	0.39877	0.15444	0.94254	0.92225	0.6753	0.69867	0.72429	0.08816	6.59E-08	6.59E-08	green
PLEKHA8	0.00606	-0.25694	0.00576	0.09323	-0.07325	0.05959	0.04463	0.11583	-0.11583	0.93733	0.00069	0.94035	0.22518	0.34105	0.43881	0.56219	0.13139	0.13139	turquoise
SLC16A6	-0.11119	-0.10869	-0.01085	0.0023	-0.13151	-0.01995	-0.04387	0.31327	-0.31327	0.14766	0.15706	0.88802	0.97623	0.08643	0.79562	0.56887	3.02E-05	3.02E-05	blue
SUMO1	-0.02453	-0.02202	-0.02973	0.00852	-0.02392	0.04034	0.11448	-0.28699	0.28699	0.75015	0.77495	0.69952	0.91194	0.7561	0.60041	0.13597	0.00014	0.00014	turquoise
CHFR	-0.01303	-0.03052	0.01942	0.01983	0.14937	-0.04152	-0.02056	0.06107	-0.06107	0.86564	0.69194	0.80099	0.79682	0.05119	0.58974	0.78949	0.42754	0.42754	red
SERPINB9	-0.12334	-0.12275	0.02482	0.02743	-0.18862	0.12029	0.08771	0.08373	-0.08373	0.108	0.10971	0.74731	0.72176	0.01349	0.11707	0.25398	0.27623	0.27623	blue
CCR1	-0.13404	0.00112	0.05775	0.06191	-0.21417	0.1374	0.10925	0.44407	-0.44407	0.0805	0.98839	0.45308	0.42116	0.00491	0.07312	0.15492	1.17E-09	1.17E-09	blue
GDPD3	0.06268	-0.00503	0.07646	0.00319	0.14266	0.1227	0.17256	-0.47433	0.47433	0.41542	0.94791	0.32024	0.96701	0.06269	0.10987	0.02401	5.62E-11	5.62E-11	yellow
ST7	0.05357	-0.08939	-0.01792	0.01433	0.10252	-0.12557	-0.07562	0.14237	-0.14237	0.4865	0.24495	0.81607	0.8524	0.18211	0.10175	0.32559	0.06324	0.06324	turquoise
FNBP1L	-0.02449	-0.20376	-0.05855	0.03146	0.01955	0.07366	-0.05421	0.05788	-0.05788	0.75048	0.00752	0.44682	0.68294	0.79964	0.33833	0.48129	0.4521	0.4521	turquoise
OPN3	-0.03624	-0.16785	0.13303	0.11796	-0.24201	0.2291	0.12544	-0.03498	0.03498	0.63791	0.0282	0.08282	0.12441	0.00143	0.00258	0.10209	0.64969	0.64969	turquoise
NKIRAS1	-0.06255	-0.21095	-0.18545	-0.10719	-0.05094	-0.15217	-0.10022	0.46448	-0.46448	0.41637	0.00561	0.01517	0.16289	0.50818	0.04694	0.19215	1.56E-10	1.56E-10	brown
PRR26	-0.02445	0.02645	-0.16821	-0.06238	0.24103	-0.07996	-0.15214	0.02691	-0.02691	0.75091	0.73134	0.02786	0.41767	0.0015	0.29854	0.04698	0.72682	0.72682	grey
SAP30L	0.00062	-0.21551	-0.16036	-0.05847	-0.07898	-0.10562	-0.08743	0.36914	-0.36914	0.99362	0.00464	0.03615	0.4475	0.3045	0.16917	0.25548	6.75E-07	6.75E-07	turquoise
PRKAR1A	-0.05469	-0.21222	-0.09864	-0.03068	-0.02233	-0.13304	-0.13345	0.36817	-0.36817	0.47741	0.00533	0.19931	0.6904	0.77193	0.08279	0.08184	7.26E-07	7.26E-07	turquoise
BOLA3	0.0117	0.05957	-0.0872	-0.1143	0.10583	-0.16836	0.06361	-0.30757	0.30757	0.87925	0.43893	0.25678	0.1366	0.16831	0.02772	0.40848	4.27E-05	4.27E-05	grey
PDXK	0.01296	-0.11827	-0.12632	0.00933	0.06915	-0.10713	0.14348	-0.13397	0.13397	0.86643	0.12341	0.0997	0.90363	0.36883	0.16313	0.06117	0.08066	0.08066	grey
SERTAD3	0.04171	-0.2233	0.05999	0.12342	-0.10563	0.11962	0.12169	-0.26822	0.26822	0.58808	0.00333	0.43576	0.10777	0.16913	0.11914	0.11283	0.00039	0.00039	grey
AC005726	0.0416	-0.04134	-0.01907	-0.00188	0.24751	-0.07882	-0.03589	-0.19117	0.19117	0.589	0.59141	0.80445	0.98057	0.0011	0.30551	0.64117	0.01226	0.01226	red
AAAS	0.08971	-0.04276	-0.14697	-0.04099	0.21721	-0.20327	-0.13674	-0.05383	0.05383	0.24328	0.57865	0.05508	0.59448	0.00432	0.00766	0.07453	0.48436	0.48436	grey
EPHX4	0.0359	0.10422	0.06372	0.02539	-0.03587	0.10563	0.15496	-0.4027	0.4027	0.64113	0.17493	0.40771	0.74166	0.64137	0.16913	0.043	4.75E-08	4.75E-08	yellow
XPO6	-0.00728	-0.00165	-0.03208	-0.01869	-0.09	0.01276	0.11793	-0.00035	0.00035	0.92468	0.98293	0.67699	0.80834	0.24173	0.86845	0.1245	0.99637	0.99637	turquoise
GALNT10	-0.0481	-0.0571	0.11365	0.07396	-0.14196	0.24487	0.16772	-0.09985	0.09985	0.53212	0.45824	0.13886	0.33636	0.06401	0.00125	0.02833	0.19381	0.19381	turquoise
CBX5	0.00887	-0.19799	-0.17456	-0.04952	-0.04184	-0.21876	-0.16841	0.33142	-0.33142	0.90835	0.00944	0.0224	0.5201	0.58692	0.00405	0.02768	9.52E-06	9.52E-06	turquoise
CAPS2	-0.00812	-0.17075	-0.11322	-0.01145	0.14387	-0.24716	-0.18168	0.28962	-0.28962	0.91607	0.02556	0.14035	0.88185	0.06048	0.00112	0.0174	0.00012	0.00012	brown
EXT1	-0.06024	-0.10994	0.00289	0.08342	-0.08541	0.15047	0.19157	-0.08398	0.08398	0.43382	0.15229	0.97012	0.27801	0.26669	0.04949	0.01207	0.27483	0.27483	turquoise
ELMSAN1	0.02685	-0.1915	-0.02633	0.0493	-0.05445	0.07211	-0.04748	0.05357	-0.05357	0.72742	0.01211	0.73248	0.52193	0.4794	0.3486	0.53746	0.48648	0.48648	turquoise
C4orf46	-0.09897	-0.05509	-0.10685	-0.04641	-0.03125	-0.14464	-0.02515	0.17791	-0.17791	0.19778	0.4742	0.16422	0.54666	0.68492	0.0591	0.744	0.01991	0.01991	turquoise
PIP5K1C	0.00451	-0.08473	-0.06076	0.01904	0.02858	-0.0602	-0.02686	0.11436	-0.11436	0.9533	0.27054	0.42983	0.80473	0.71062	0.43413	0.72725	0.13638	0.13638	grey
ATP11A	-0.06254	-0.07781	-0.04017	0.05717	-0.0371	0.09566	0.02337	0.04833	-0.04833	0.41642	0.31174	0.60193	0.45762	0.62994	0.21329	0.76154	0.53014	0.53014	turquoise
SELE	-0.18406	-0.01283	-0.07267	0.04348	-0.07192	0.06328	0.01069	0.43927	-0.43927	0.01596	0.86772	0.34485	0.57232	0.34989	0.4109	0.88961	1.85E-09	1.85E-09	blue
DCXR	0.03732	0.05523	-0.01764	-0.08915	0.17683	-0.18276	0.02918	-0.1791	0.1791	0.62798	0.47308	0.81886	0.24625	0.02069	0.01673	0.70483	0.01909	0.01909	grey
PROM1	-0.05528	-0.04286	0.04378	0.05909	-0.04307	0.24832	0.11501	-0.17882	0.17882	0.47269	0.57778	0.56964	0.44269	0.57596	0.00106	0.13415	0.01928	0.01928	yellow
ZNF775	0.06469	0.00559	-0.16993	-0.07515	0.13407	-0.19005	-0.03618	0.14073	-0.14073	0.40057	0.94218	0.02628	0.32862	0.08042	0.01278	0.63854	0.06636	0.06636	grey
SIAH2	0.02606	-0.0723	0.04732	0.07306	-0.13922	0.21494	0.15093	-0.19216	0.19216	0.73511	0.34738	0.53883	0.3423	0.06937	0.00475	0.04878	0.01181	0.01181	turquoise
RADX	-0.0194	-0.14318	-0.2742	-0.13559	0.08816	-0.36756	-0.24982	0.43629	-0.43629	0.80111	0.06172	0.00028	0.07703	0.25156	7.60E-07	0.00098	2.45E-09	2.45E-09	brown
ZNF621	-0.10789	-0.15306	-0.03326	-0.02891	-0.09302	0.10385	0.05254	0.11681	-0.11681	0.16013	0.04565	0.66582	0.70739	0.22624	0.17645	0.49489	0.12815	0.12815	turquoise
POLR3D	0.00335	-0.06367	0.03673	-0.01734	-0.13381	0.06299	0.15809	0.14603	-0.14603	0.96534	0.40803	0.63338	0.82188	0.08102	0.41306	0.03891	0.05667	0.05667	turquoise
FAM107A	-0.05899	-0.10343	-0.09539	0.04748	-0.06248	-0.02949	-0.14607	0.50191	-0.50191	0.44345	0.17823	0.21457	0.53742	0.41691	0.70185	0.0566	2.68E-12	2.68E-12	blue
TMA7	-0.00738	-0.07509	-0.11859	-0.21127	0.07767	-0.07837	-0.05528	-0.05344	0.05344	0.92369	0.32899	0.1224	0.00554	0.31263	0.30829	0.47266	0.48753	0.48753	purple
DENND4C	0.00101	-0.13863	-0.03137	-0.00148	-0.06024	0.10635	0.04299	0.20642	-0.20642	0.98957	0.07057	0.68375	0.98471	0.43382	0.16622	0.57668	0.00675	0.00675	turquoise
RBM6	-0.01376	-0.10234	-0.05559	-0.07061	0.07147	-0.03693	-0.07721	0.00258	-0.00258	0.85824	0.18287	0.47018	0.35875	0.35292	0.63154	0.31548	0.97329	0.97329	red
JUN	-0.00703	-0.10722	0.04129	0.09146	0.06574	0.15151	-0.00879	0.14446	-0.14446	0.92729	0.16279	0.5918	0.23418	0.39295	0.04792	0.90916	0.05942	0.05942	blue
ZNF17	-0.0422	-0.19643	-0.12486	0.02244	-0.03071	0.01851	-0.08914	0.27904	-0.27904	0.58363	0.01003	0.10371	0.77077	0.69007	0.81009	0.24626	0.00022	0.00022	turquoise
UFL1	-0.12073	-0.09107	-0.01439	0.04126	-0.02894	-0.03333	-0.08423	0.33301	-0.33301	0.11574	0.23619	0.85185	0.59206	0.70716	0.66529	0.27338	8.57E-06	8.57E-06	turquoise
AATK	-0.12813	-0.02478	-0.0268	0.00312	0.23803	-0.0828	-0.16111	-0.01491	0.01491	0.0949	0.74772	0.72792	0.96772	0.00172	0.28163	0.03528	0.84657	0.84657	grey
LAMP3	-0.09976	-0.04708	0.08685	0.11912	-0.19279	0.21242	0.1302	0.11227	-0.11227	0.1942	0.54089	0.2587	0.12071	0.01153	0.00528	0.08962	0.14375	0.14375	blue
CUL4B	-0.03765	-0.21549	-0.05114	0.01044	-0.19949	-0.01132	0.05875	0.18455	-0.18455	0.62494	0.00465	0.50655	0.89217	0.0089	0.88319	0.44531	0.01568	0.01568	turquoise
EBPL	0.11996	0.12781	-0.01631	5.62E-05	-0.04192	-0.0072	0.12227	-0.38183	0.38183	0.11809	0.09572	0.83229	0.99942	0.58618	0.92553	0.11112	2.56E-07	2.56E-07	purple
SLC40A1	-0.0048	-0.00407	0.03871	0.05301	0.04626	0.20264	0.02184	-0.04698	0.04698	0.95033	0.95787	0.61519	0.49108	0.54798	0.00786	0.77681	0.5417	0.5417	yellow
RASL10B	-0.04364	-0.14163	-0.25883	-0.05176	-0.03219	-0.2524	-0.09931	0.41346	-0.41346	0.57088	0.06463	0.00063	0.50138	0.67602	0.00087	0.19626	1.90E-08	1.90E-08	grey
FGD1	-0.07293	-0.2125	-0.1452	0.02687	-0.08783	-0.15791	-0.09835	0.42093	-0.42093	0.34313	0.00526	0.05811	0.72721	0.25332	0.03914	0.20063	9.89E-09	9.89E-09	blue
MRPL20	-0.0859	-0.02681	-0.07646	-0.02219	0.05014	-0.04214	0.0266	-0.1911	0.1911	0.26397	0.72779	0.32021	0.77333	0.5149	0.58417	0.72987	0.01229	0.01229	grey
C1orf54	-0.07748	-0.03718	0.04112	0.09809	-0.11557	0.09719	0.04623	0.23451	-0.23451	0.31384	0.62922	0.59331	0.20183	0.13226	0.20604	0.54823	0.00202	0.00202	blue
IL33	-0.12204	-0.02179	-0.01575	0.10893	-0.04616	0.12903	-0.06745	0.50726	-0.50726	0.11181	0.77729	0.83803	0.15614	0.54884	0.09258	0.3807	1.44E-12	1.44E-12	blue
ZNF491	-0.0811	-0.10593	-0.25921	-0.05667	0.11722	-0.27802	-0.16943	0.48153	-0.48153	0.29164	0.16793	0.00062	0.46159	0.1268	0.00023	0.02673	2.60E-11	2.60E-11	brown
ANKRD16	0.06838	0.03994	-0.21846	-0.09357	0.13349	-0.17272	0.01704	0.05452	-0.05452	0.3742	0.60399	0.0041	0.2235	0.08175	0.02388	0.82492	0.47881	0.47881	grey
SRD5A1	0.01566	-0.05461	-0.00732	0.00889	0.04917	-0.15249	0.07562	-0.0864	0.0864	0.8389	0.4781	0.92428	0.90815	0.52308	0.04647	0.32557	0.26119	0.26119	grey
TINAG	-0.09807	0.05476	0.10112	0.08232	0.15185	0.17199	0.08415	-0.26792	0.26792	0.20191	0.47688	0.1882	0.28444	0.04741	0.02449	0.27383	0.0004	0.0004	grey
HNRNPAB	-0.00998	-0.05172	-0.02964	-0.01103	0.01418	0.04606	0.09533	-0.22928	0.22928	0.89692	0.5017	0.70004	0.8861	0.85396	0.54966	0.21487	0.00256	0.00256	turquoise
MAOB	-0.03068	-0.05667	0.02228	0.08933	0.00263	0.06713	-0.11326	-0.043	0.043	0.69035	0.46157	0.77235	0.24529	0.97278	0.383	0.14021	0.57659	0.57659	grey
MAMSTR	0.07703	-0.04809	-0.04876	-0.00998	0.03447	-0.0149	0.11739	-0.10916	0.10916	0.31664	0.5322	0.52649	0.8969	0.65443	0.8466	0.12498	0.15524	0.15524	grey
DDX11	-0.00779	0.03376	0.00235	-0.04023	0.07609	0.03268	0.12659	-0.27392	0.27392	0.91947	0.66116	0.97571	0.60136	0.3226	0.67135	0.09897	0.00029	0.00029	grey
ZNF749	-0.01471	-0.2178	-0.16296	-0.01707	-0.07435	0.00408	0.02219	0.05576	-0.05576	0.84852	0.00421	0.03321	0.82459	0.33382	0.95776	0.77325	0.46883	0.46883	turquoise
SNW1	0.05277	-0.07549	0.00362	0.01913	-0.0872	0.08378	0.04049	-0.02279	0.02279	0.49307	0.32646	0.96256	0.8039	0.25678	0.27594	0.59898	0.7673	0.7673	turquoise
DHRSX	-0.10174	-0.07893	-0.12366	0.01064	0.01592	-0.01679	0.00039	0.08998	-0.08998	0.18549	0.3048	0.1071	0.89012	0.83629	0.8275	0.99595	0.24184	0.24184	grey
NDUFAF5	0.04178	0.01401	-0.15118	-0.07693	0.15728	-0.25181	-0.00048	0.01966	-0.01966	0.58746	0.85565	0.04841	0.31724	0.03993	0.00089	0.99504	0.7985	0.7985	grey
GRPEL2	0.03712	-0.06606	-0.14365	-0.07687	0.00426	-0.08106	-0.06686	-0.02115	0.02115	0.62981	0.39068	0.06088	0.31767	0.95589	0.29193	0.38492	0.78366	0.78366	turquoise
GAS8	0.01298	-0.09395	-0.23572	-0.10397	0.22548	-0.15662	-0.12965	-0.08407	0.08407	0.86619	0.22162	0.00191	0.17595	0.00302	0.04078	0.09101	0.2743	0.2743	grey
TMEM203	-0.01696	-0.06629	-0.14166	-0.03844	-0.09009	-0.1184	0.04365	-0.0352	0.0352	0.82573	0.389	0.06458	0.6177	0.24124	0.12299	0.57081	0.6476	0.6476	grey
FFAR2	-0.034	-0.01455	-0.17793	-0.15639	0.21348	-0.17588	-0.14044	0.18117	-0.18117	0.65883	0.85015	0.0199	0.04109	0.00506	0.02139	0.06694	0.01772	0.01772	grey
MT1E	-0.12612	0.10028	-0.02501	0.05363	-0.10181	-0.01876	0.01946	-0.1235	0.1235	0.10023	0.19191	0.74545	0.48605	0.18514	0.80754	0.80055	0.10756	0.10756	grey
ZNF829	-0.134	-0.15228	-0.09871	-0.00406	-0.12697	-0.02711	-0.04841	0.49585	-0.49585	0.08058	0.04677	0.19896	0.95802	0.09794	0.7249	0.52951	5.36E-12	5.36E-12	turquoise
MRTFA	-0.05887	-0.08049	-0.00595	0.04221	-0.12214	0.09901	-0.00162	0.10653	-0.10653	0.44438	0.2953	0.93842	0.58358	0.1115	0.19762	0.98325	0.1655	0.1655	turquoise
BAG2	-0.04355	-0.07433	0.04344	0.0773	-0.11073	-0.03393	-0.0486	0.46689	-0.46689	0.57167	0.33396	0.57267	0.31492	0.14937	0.65957	0.5279	1.22E-10	1.22E-10	salmon
DERL3	-0.18746	0.08226	0.05	0.03622	-0.08245	0.14436	0.13062	0.13333	-0.13333	0.01408	0.28481	0.51606	0.63808	0.28367	0.0596	0.08859	0.08213	0.08213	grey
PRDX2	0.06659	0.02151	-0.2371	-0.20228	0.15649	-0.33986	-0.14495	-0.08101	0.08101	0.38686	0.78008	0.00179	0.00797	0.04096	5.42E-06	0.05855	0.29222	0.29222	grey
SAMD4B	0.07573	-0.27732	-0.05279	0.07579	-0.14565	0.01492	0.06072	-0.02382	0.02382	0.32492	0.00024	0.49292	0.3245	0.05734	0.84646	0.43016	0.75713	0.75713	turquoise
SPTBN1	0.01756	-0.13135	0.00588	0.04144	-0.12038	0.24715	0.09349	-0.05431	0.05431	0.81964	0.08681	0.9392	0.59046	0.11682	0.00112	0.2239	0.48051	0.48051	turquoise
BATF2	0.01449	0.01401	0.01329	0.03681	-0.04341	0.07903	0.22223	-0.26756	0.26756	0.85083	0.22032	0.86306	0.63269	0.57289	0.3042	0.00349	0.0004	0.0004	tan
GAS6	-0.06145	0.03954	-0.02531	0.06186	-0.10984	0.11244	0.14556	0.1934	-0.1934	0.42465	0.60764	0.74248	0.42155	0.15267	0.14315	0.05749	0.01126	0.01126	blue
APOL4	-0.00989	0.0342	0.02439	0.0929	-0.09062	0.116	0.09515	0.26705	-0.26705	0.8978	0.65696	0.75154	0.22683	0.2385	0.13083	0.21575	0.00041	0.00041	blue
SKP2	-0.07254	0.00221	-0.00055	0.01975	0.00236	-0.00985	0.1704	-0.03315	0.03315	0.34576	0.97708	0.99428	0.79768	0.97551	0.89827	0.02587	0.66686	0.66686	pink
GUCA2B	0.01593	-0.07568	-0.13206	0.07174	0.06735	0.01901	-0.04418	0.00174	-0.00174	0.83617	0.32524	0.08511	0.35112	0.38147	0.80505	0.56615	0.98199	0.98199	grey
HEXD	-0.01541	-0.07311	-0.09682	-0.01614	0.21823	-0.1981	-0.02646	-0.08853	0.08853	0.84143	0.34197	0.20776	0.834	0.00414	0.0094	0.73121	0.24957	0.24957	red
DBN1	-0.02231	-0.06236	-0.15279	-0.02937	-0.07943	-0.07835	0.02635	0.01487	-0.01487	0.77208	0.41779	0.04604	0.70292	0.30177	0.30839	0.7323	0.8469	0.8469	black
DCAF16	-0.03139	-0.11777	-0.16567	-0.10467	0.02237	-0.12995	-0.06791	0.14172	-0.14172	0.68362	0.12502	0.03035	0.17308	0.77149	0.09026	0.37751	0.06447	0.06447	turquoise
MLKL	0.0582	0.00285	0.11742	0.08731	-0.05454	0.25501	0.27935	-0.25117	0.25117	0.44957	0.9705	0.12613	0.25617	0.47862	0.00076	0.00022	0.00092	0.00092	yellow
PISD	-0.10479	-0.10186	-0.1272	-0.11704	0.12262	-0.16785	-0.12445	0.15028	-0.15028	0.17257	0.18495	0.09735	0.12738	0.11009	0.0282	0.10486	0.04978	0.04978	grey
CRCT1	0.08068	0.0133	-0.07102	-0.09864	0.05985	-0.08513	0.09649	-0.1558	0.1558	0.29421	0.86293	0.35599	0.19929	0.43681	0.26829	0.20931	0.04187	0.04187	grey
HCAR2	-0.0327	-0.04207	0.15832	0.08126	-0.1417	0.11837	0.17253	-0.01522	0.01522	0.67113	0.58481	0.03862	0.29068	0.0645	0.12308	0.02404	0.84338	0.84338	grey
TSPYL5	-0.06112	-0.19778	0.03597	0.14687	-0.21871	-0.07852	-0.0516	0.47632	-0.47632	0.42716	0.00951	0.64047	0.05525	0.00406	0.30731	0.5027	4.55E-11	4.55E-11	turquoise
EIF3G	0.13135	0.11873	-0.12495	-0.09838	0.10974	-0.0316	0.10255	-0.17811	0.17811	0.0868	0.12193	0.10344	0.20049	0.15306	0.68161	0.18197	0.01977	0.01977	green
PARD6G	-0.04903	-0.05219	-0.02271	0.05386	-0.16767	-0.00461	-0.00483	0.33026	-0.33026	0.52426	0.49779	0.76815	0.48416	0.02838	0.95228	0.94996	1.03E-05	1.03E-05	blue
CTSZ	-0.06423	-0.04027	0.09624	0.12843	-0.18969	0.14302	0.14179	0.00376	-0.00376	0.40396	0.60099	0.21048	0.09411	0.01296	0.06202	0.06433	0.96112	0.96112	black
FAM222B	0.01471	-0.16524	-0.04364	-0.03132	-0.00647	0.05477	0.04528	-0.31924	0.31924	0.84851	0.03079	0.57092	0.68422	0.93311	0.47676	0.5565	2.08E-05	2.08E-05	turquoise
SLC25A37	-0.0249	-0.08339	0.154	0.0373	-0.03483	0.16597	0.2501	-0.35681	0.35681	0.74647	0.27821	0.04432	0.62811	0.6511	0.03005	0.00097	1.67E-06	1.67E-06	yellow
ZNF692	0.06164	-0.01915	-0.08086	-0.08039	0.27736	-0.12322	0.05357	-0.30855	0.30855	0.42316	0.80363	0.29313	0.29591	0.00024	0.10836	0.48647	4.03E-05	4.03E-05	red
UBE2W	-0.01868	-0.07315	0.02965	0.02373	-0.07743	-0.02805	-0.05469	0.17876	-0.17876	0.80841	0.34167	0.70024	0.75806	0.3141	0.71571	0.47742	0.01932	0.01932	turquoise
TP53I11	0.06792	-0.11226	0.05461	0.16078	0.04121	0.16668	0.13653	-0.25776	0.25776	0.37744	0.14379	0.47806	0.03567	0.5925	0.02934	0.07497	0.00066	0.00066	yellow
YIPF1	0.00073	-0.04028	-0.0256	-0.00858	-0.01495	0.06758	0.10251	-0.09485	0.09485	0.99245	0.60088	0.73957	0.91132	0.84608	0.37981	0.18215	0.21718	0.21718	turquoise
NT5C2	-0.05936	-0.14153	0.07564	0.03198	-0.10803	0.09447	0.0522	0.03367	-0.03367	0.44055	0.06483	0.3255	0.67799	0.15959	0.21906	0.49772	0.66196	0.66196	turquoise
THNSL2	-0.047	-0.15294	-0.03521	0.08461	-0.09139	-0.01858	0.04349	-0.07233	0.07233	0.54157	0.04582	0.64753	0.27121	0.23454	0.80937	0.5722	0.34715	0.34715	grey
WASL	0.03959	-0.1445	-0.02268	-0.00401	0.00149	0.03204	-0.02946	0.03515	-0.03515	0.60714	0.05934	0.76841	0.95847	0.98459	0.6774	0.70205	0.6481	0.6481	turquoise
RHBDD1	-0.02364	-0.03968	0.02868	0.05465	-0.19559	0.15801	0.16392	0.00232	-0.00232	0.75896	0.60639	0.70967	0.47778	0.01036	0.03901	0.03216	0.97603	0.97603	turquoise
ALDH1A3	-0.0402	-0.16727	0.10732	0.13664	-0.24583	0.20462	-0.00084	0.23079	-0.23079	0.60163	0.02877	0.16238	0.07474	0.00119	0.00726	0.99125	0.00239	0.00239	blue
MNS1	-0.0725	-0.07069	-0.0212	0.03154	0.06881	0.13113	0.09363	-0.09557	0.09557	0.34604	0.35821	0.78315	0.68217	0.37117	0.08735	0.2232	0.21371	0.21371	grey
ZKSCAN4	-0.04839	-0.04136	-0.05047	0.00824	0.01023	0.05711	0.04351	0.16765	-0.16765	0.52964	0.59117	0.5121	0.91482	0.89435	0.4581	0.57202	0.0284	0.0284	turquoise
PTK2B	-0.00105	-0.04009	0.05387	0.01277	-0.00853	-0.01519	0.01221	0.14452	-0.14452	0.98915	0.60268	0.48405	0.86837	0.91179	0.84366	0.87404	0.0593	0.0593	blue
EVA1C	-0.02127	-0.00517	-0.11851	0.02169	-0.00757	0.11401	0.06078	0.02032	-0.02032	0.78246	0.94648	0.12264	0.77824	0.92172	0.13759	0.42971	0.79198	0.79198	grey
GNS	0.0059	-0.16733	-0.00613	0.03978	-0.0472	-0.06229	-0.03018	0.23889	-0.23889	0.939	0.0287	0.93659	0.60547	0.53986	0.41829	0.69512	0.00165	0.00165	turquoise
LAPTM4B	0.06318	-0.09074	-0.13433	0.02748	0.00368	-0.00011	0.11736	-0.15049	0.15049	0.41164	0.23787	0.07984	0.72129	0.96191	0.99889	0.12633	0.04945	0.04945	grey
ZCCHC24	-0.07727	-0.08605	-0.03695	0.09204	-0.19006	0.14717	0.06734	0.45714	-0.45714	0.31512	0.26312	0.63137	0.23119	0.01278	0.05475	0.38148	3.27E-10	3.27E-10	blue
MRPS5	0.1304	0.00897	-0.0223	-0.0216	-0.01222	0.01914	0.1948	-0.37824	0.37824	0.08914	0.90727	0.77218	0.77916	0.87395	0.80379	0.01067	3.39E-07	3.39E-07	turquoise
SLC25A26	0.06207	0.1217	-0.09347	-0.04779	-0.12595	0.09879	0.13331	-0.16561	0.16561	0.41998	0.11281	0.22402	0.53482	0.10071	0.1986	0.08217	0.03041	0.03041	grey
FUT6	0.1333	0.08535	0.04379	0.00324	0.05519	0.13533	0.10053	-0.36107	0.36107	0.08219	0.26702	0.56955	0.96647	0.47337	0.07758	0.1908	1.22E-06	1.22E-06	yellow
MTMR4	0.03001	-0.12547	-0.0557	-0.0322	-0.02127	-0.07202	0.01434	0.15409	-0.15409	0.69679	0.10201	0.46929	0.67591	0.7824	0.34923	0.85231	0.04419	0.04419	turquoise
ADAM15	0.01677	0.01968	0.07993	0.02184	0.07517	0.14521	0.2095	-0.35341	0.35341	0.82767	0.79837	0.29873	0.7768	0.32848	0.05808	0.00596	2.12E-06	2.12E-06	yellow
ENPEP	-0.11266	-0.11701	-0.04645	0.08636	-0.15471	-0.0512	-0.02465	0.42583	-0.42583	0.14235	0.12747	0.54631	0.26138	0.04334	0.50602	0.74899	6.38E-09	6.38E-09	blue
C16orf91	0.00465	0.0378	-0.06855	-0.037	0.03552	0.00111	0.15094	-0.28606	0.28606	0.95192	0.62351	0.37302	0.63093	0.64462	0.98852	0.04877	0.00015	0.00015	grey
USP13	-0.05585	-0.11737	-0.18886	-0.08845	-0.01476	-0.165	-0.08709	0.3869	-0.3869	0.46815	0.12629	0.01337	0.24999	0.84802	0.03103	0.25737	1.72E-07	1.72E-07	turquoise
BCL2	-0.05695	-0.07736	-0.02946	0.05318	-0.18101	0.07185	-0.11026	0.58924	-0.58924	0.45935	0.31454	0.70209	0.48968	0.01782	0.35035	0.15109	2.30E-17	2.30E-17	blue
C5AR2	-0.03644	0.0724	0.02688	0.07202	-0.2047	0.0118	0.14334	0.05257	-0.05257	0.63609	0.34668	0.72711	0.34925	0.00724	0.87821	0.06145	0.49469	0.49469	grey

ZBTB47	-0.06689	-0.11417	-0.06698	0.01706	-0.11729	0.06989	-0.03174	0.42944	-0.42944	0.38473	0.13704	0.38405	0.82474	0.12656	0.36369	0.68026	4.60E-09	4.60E-09	blue
TSTD2	-0.06763	-0.16218	-0.1031	0.0157	-0.10872	-0.00944	-0.06309	0.38788	-0.38788	0.37949	0.03406	0.17963	0.83846	0.15694	0.90246	0.41233	1.59E-07	1.59E-07	turquoise
NBPF3	-0.05082	-0.18481	-0.07897	-0.06033	-0.04301	-0.10569	-0.03224	0.25423	-0.25423	0.50917	0.01553	0.30454	0.43316	0.57649	0.16889	0.67546	0.00079	0.00079	turquoise
NUPR2	0.03787	-0.03456	-0.20091	-0.14829	0.08596	-0.22855	-0.18356	0.21714	-0.21714	0.62285	0.65363	0.00842	0.0529	0.26359	0.00264	0.01625	0.00433	0.00433	brown
ITPK1	0.09246	0.01421	0.0069	0.08494	0.0967	-0.05088	-0.01419	-0.23864	0.23864	0.22905	0.85361	0.9286	0.26936	0.20832	0.50871	0.85387	0.00167	0.00167	grey
DCAF17	-0.02101	-0.07223	-0.03491	0.06425	-0.1189	0.07251	0.03149	0.17888	-0.17888	0.78501	0.34782	0.65034	0.40379	0.12141	0.34594	0.68259	0.01924	0.01924	turquoise
RAB21	0.06972	-0.1446	-0.05111	0.04461	-0.00199	-0.03421	-0.16574	0.11947	-0.11947	0.36486	0.05916	0.5068	0.56235	0.97938	0.65694	0.03028	0.1196	0.1196	turquoise
BEX5	-0.00762	-0.04899	-0.25923	-0.11976	0.13763	-0.22652	-0.22201	0.25697	-0.25697	0.92121	0.52457	0.00062	0.11872	0.07263	0.00289	0.00352	0.00069	0.00069	brown
PHTF2	-0.0825	-0.24458	-0.02089	0.07375	-0.09337	-0.13749	-0.04745	0.43272	-0.43272	0.28339	0.00126	0.78627	0.33774	0.22451	0.07294	0.53773	3.41E-09	3.41E-09	turquoise
XAB2	0.10941	0.03677	-0.18123	-0.09185	0.19078	-0.15707	0.0367	-0.20594	0.20594	0.15431	0.63301	0.01768	0.23215	0.01244	0.04021	0.63369	0.00689	0.00689	green
TBC1D31	0.01003	0.08015	-0.02606	-0.00758	-0.01861	-0.07781	0.12583	-0.03539	0.03539	0.89643	0.2974	0.73515	0.92165	0.80905	0.31176	0.10101	0.64587	0.64587	turquoise
PRF1	-0.06443	0.05571	0.05597	0.13142	-0.22355	0.05071	0.09259	0.37985	-0.37985	0.40248	0.46922	0.46712	0.08663	0.00329	0.51008	0.22841	2.99E-07	2.99E-07	blue
SLC17A4	0.04472	-0.043	-0.11417	0.06621	0.10008	0.04995	0.00827	0.08045	-0.08045	0.56135	0.57657	0.13706	0.3896	0.1928	0.51648	0.91452	0.29554	0.29554	magenta
HSD17B11	-0.03416	-0.03265	0.07728	0.0329	-0.03445	0.21301	0.10145	-0.1654	0.1654	0.65734	0.67159	0.31508	0.6692	0.6546	0.00515	0.18673	0.03063	0.03063	turquoise
FMN1	0.08158	-0.07411	0.06803	0.03073	-0.1571	0.14583	0.11889	-0.11677	0.11677	0.28882	0.33537	0.37666	0.68985	0.04017	0.05702	0.12143	0.12828	0.12828	turquoise
ALPG	0.09088	0.1416	0.01343	0.09544	0.03121	-0.0242	0.05819	-0.06575	0.06575	0.23716	0.06468	0.86156	0.21436	0.68535	0.75338	0.44965	0.39286	0.39286	grey
SH2B2	-0.05665	-0.1192	-0.00815	0.06029	-0.07894	-0.03614	0.08803	0.00088	-0.00088	0.46178	0.12047	0.91574	0.43347	0.30472	0.63888	0.25223	0.99087	0.99087	grey
GNG2	-0.08737	-0.16168	-0.15284	-0.07283	-0.02041	-0.24732	-0.27363	0.62956	-0.62956	0.25585	0.03463	0.04596	0.31784	0.79106	0.00111	0.00029	2.89E-20	2.89E-20	brown
DLG1	-0.02242	-0.13059	-0.07662	-0.05222	-0.10647	0.05546	0.12705	0.0179	-0.0179	0.77104	0.08867	0.31921	0.49759	0.16576	0.47122	0.09774	0.81623	0.81623	turquoise
SUMF1	0.00091	-0.11789	-0.00079	-0.0252	-0.06762	0.14685	0.13951	-0.22323	0.22323	0.99062	0.12462	0.9918	0.7435	0.37953	0.05529	0.06879	0.00334	0.00334	turquoise
BTBD11	-0.04073	-0.10118	-0.09048	-0.09376	0.063	-0.32949	-0.19934	0.27081	-0.27081	0.59688	0.18792	0.23921	0.22256	0.41299	1.08E-05	0.00895	0.00034	0.00034	brown
PIK3R6	-0.11135	-0.07656	0.02954	0.06578	-0.14247	0.13206	0.10709	0.32672	-0.32672	0.14708	0.31958	0.70133	0.39268	0.06305	0.08511	0.16327	1.29E-05	1.29E-05	blue
RUNX1T1	0.07311	-0.11583	-0.02389	0.01239	-0.06433	-0.09149	-0.08734	0.56154	-0.56154	0.34197	0.13139	0.75641	0.87219	0.40321	0.23402	0.256	1.35E-15	1.35E-15	turquoise
U2AF1L4	-0.02827	-0.02747	-0.07897	-0.09328	0.11681	-0.12452	-0.09759	0.03827	-0.03827	0.71358	0.72134	0.30456	0.22496	0.12812	0.10466	0.20415	0.61918	0.61918	red
GLI1	-0.03285	-0.06743	0.03863	0.03472	-0.1759	0.20569	0.01005	0.32702	-0.32702	0.6697	0.38086	0.61589	0.65215	0.02138	0.00696	0.89619	1.27E-05	1.27E-05	blue
VENTX	-0.04146	-0.03469	-0.0419	0.09733	-0.04342	0.02945	0.05767	0.41328	-0.41328	0.5903	0.6524	0.58633	0.20535	0.57283	0.70216	0.4537	1.93E-08	1.93E-08	blue
MEGF9	-0.02966	-0.16375	-0.10236	-0.06588	-0.04675	-0.06638	-0.1013	0.40393	-0.40393	0.70016	0.03235	0.18277	0.39192	0.54371	0.38838	0.18739	4.29E-08	4.29E-08	turquoise
NIPAL1	-0.01386	-0.14654	0.0183	0.10297	-0.00579	0.14303	0.12039	-0.19004	0.19004	0.85726	0.05581	0.8122	0.18018	0.94004	0.062	0.11678	0.01279	0.01279	turquoise
CA8	0.02478	-0.06058	-0.20264	-0.20112	0.20103	-0.18156	-0.14362	0.19284	-0.19284	0.74767	0.43119	0.00786	0.00835	0.00838	0.01748	0.06093	0.01151	0.01151	grey
PRPF6	0.17576	-0.00383	-0.12234	-0.05416	0.03497	-0.14438	0.07749	-0.2229	0.2229	0.02148	0.9603	0.11093	0.48168	0.6498	0.05956	0.31375	0.00339	0.00339	grey
IL1R1	-0.07261	-0.10935	0.04409	0.11082	-0.22376	0.16779	0.05015	0.40896	-0.40896	0.34531	0.15451	0.56695	0.14903	0.00326	0.02826	0.51475	2.80E-08	2.80E-08	blue
KANK4	0.01074	0.02149	0.06331	0.15857	-0.24016	0.08898	0.12046	0.11858	-0.11858	0.88913	0.7802	0.41068	0.03832	0.00156	0.24714	0.11657	0.1224	0.1224	black
LZTS2	0.02019	-0.05438	0.02316	0.0754	-0.017	0.0366	0.19507	-0.13698	0.13698	0.79322	0.47995	0.76363	0.32698	0.8253	0.63457	0.01056	0.07401	0.07401	grey
GCNA	-0.12915	-0.12844	-0.03615	0.06881	0.05012	-0.08078	-0.0728	-0.00264	0.00264	0.09227	0.09409	0.63879	0.37115	0.51507	0.29361	0.34402	0.97261	0.97261	red
CYLD	-0.0539	-0.12158	-0.1152	-0.07077	-0.09884	-0.08592	-0.08493	0.38843	-0.38843	0.48384	0.11319	0.1335	0.35767	0.1984	0.26385	0.26941	1.52E-07	1.52E-07	turquoise
MFSD2A	0.04464	-0.01231	0.05056	-0.01639	0.01052	0.04144	0.02747	-0.23393	0.23393	0.56205	0.87304	0.51135	0.83155	0.89138	0.59045	0.72135	0.00207	0.00207	grey
GANC	-0.04949	-0.05059	-0.02912	-0.05812	-0.11767	0.12243	0.06859	-0.02032	0.02032	0.52036	0.51113	0.70536	0.45017	0.12532	0.11067	0.3727	0.79191	0.79191	turquoise
RAPGEF3	0.12447	-0.15618	-0.00857	0.06178	0.0993	0.03034	0.17537	-0.18934	0.18934	0.10481	0.04137	0.91146	0.42215	0.19628	0.69366	0.02178	0.01313	0.01313	grey
CPE	-0.00263	-0.12617	-0.22887	-0.12469	0.11051	-0.30523	-0.29428	0.49679	-0.49679	0.97272	0.10009	0.0026	0.10419	0.15018	4.92E-05	9.35E-05	4.82E-12	4.82E-12	brown
RELL1	-0.0688	-0.17837	0.01197	0.04955	-0.17824	0.13537	0.0036	0.29146	-0.29146	0.37127	0.01959	0.87655	0.51985	0.01968	0.0775	0.96276	0.00011	0.00011	turquoise
TTLL7	-0.04521	-0.17083	-0.19266	-0.02384	0.07162	-0.18425	-0.13145	0.4357	-0.4357	0.55708	0.02549	0.01158	0.75693	0.35193	0.01585	0.08656	2.58E-09	2.58E-09	brown
ZNF311	0.04507	0.05956	-0.02589	0.04791	0.1209	0.16332	0.17953	-0.24199	0.24199	0.55831	0.43906	0.73681	0.53378	0.11523	0.03281	0.0188	0.00143	0.00143	grey
TCEAL2	-0.00198	-0.0853	-0.27491	-0.13413	0.11966	-0.29624	-0.23067	0.43344	-0.43344	0.97947	0.26729	0.00027	0.08029	0.11901	8.35E-05	0.0024	3.19E-09	3.19E-09	brown
MRPS23	-0.04485	-0.04522	-0.16177	-0.11904	0.01074	-0.20433	0.00712	-0.03802	0.03802	0.56025	0.55699	0.03453	0.12097	0.88911	0.00735	0.92634	0.62149	0.62149	grey
ATG2A	0.0232	-0.10409	-0.06202	-0.07978	0.0614	-0.05299	0.07637	-0.18825	0.18825	0.76324	0.17545	0.42036	0.29959	0.42502	0.49123	0.32081	0.01367	0.01367	green
FAM155A	-0.04097	-0.17356	-0.12363	0.00619	-0.12112	-0.11883	-0.06912	0.34273	-0.34273	0.59472	0.02319	0.10719	0.93592	0.11456	0.12164	0.36901	4.46E-06	4.46E-06	grey
KCNJ16	0.0144	-0.08313	-0.12118	0.08494	0.01685	-0.03107	-0.10073	0.1835	-0.1835	0.85173	0.2797	0.11437	0.26934	0.82689	0.68669	0.1899	0.01629	0.01629	magenta
KLF7	-0.10167	-0.15004	0.01877	0.09955	-0.19302	0.15945	0.05318	0.10041	-0.10041	0.18578	0.05015	0.80751	0.19514	0.01143	0.03723	0.48971	0.19132	0.19132	turquoise
CAPN15	0.04216	0.05499	0.01633	-0.0121	0.21321	0.03549	0.06673	-0.30754	0.30754	0.58406	0.47502	0.83216	0.87515	0.00511	0.64488	0.38583	4.28E-05	4.28E-05	green
STX19	0.05428	0.00153	0.12649	0.05803	0.03799	0.25871	0.12292	-0.37477	0.37477	0.48075	0.98416	0.09924	0.45088	0.62175	0.00063	0.10923	4.42E-07	4.42E-07	yellow
PIM3	0.05417	0.04648	-0.0224	0.02365	0.0915	0.06013	0.10082	-0.35607	0.35607	0.48161	0.54603	0.7712	0.75882	0.23395	0.43468	0.18951	1.76E-06	1.76E-06	grey
CREB3L2	-0.04791	-0.15278	-0.03828	0.01638	-0.08355	-0.09899	-0.12319	0.42942	-0.42942	0.53374	0.04605	0.6191	0.83163	0.27729	0.19768	0.10844	4.61E-09	4.61E-09	turquoise
CDCA7L	-0.07105	0.0285	0.10377	0.07657	-0.15018	0.32709	0.20425	-0.17513	0.17513	0.35578	0.7114	0.17681	0.31954	0.04992	1.26E-05	0.00737	0.02196	0.02196	pink
SLC38A6	-0.03878	-0.08365	0.09044	0.12968	-0.2027	-0.01444	0.0868	0.07827	-0.07827	0.61455	0.2767	0.23946	0.09095	0.00784	0.8513	0.25898	0.30889	0.30889	turquoise
PRRT1	-0.04701	-0.12623	-0.29978	-0.17485	0.19952	-0.29638	-0.15354	0.30075	-0.30075	0.54147	0.09993	6.79E-05	0.02217	0.00889	8.28E-05	0.04497	6.42E-05	6.42E-05	brown
ETV2	0.07769	0.03125	-0.19184	-0.11001	0.28196	-0.2232	-0.07793	-0.04963	0.04963	0.31246	0.68497	0.01195	0.15205	0.00019	0.00334	0.31099	0.51918	0.51918	grey
UBAP1	0.02168	-0.15713	-0.05694	0.03015	-0.10731	0.10215	0.01742	-0.06752	0.06752	0.77839	0.04013	0.45947	0.69541	0.1624	0.18369	0.8211	0.38021	0.38021	turquoise
BRAT1	-0.00136	0.00477	-0.10729	-0.05265	0.23801	-0.104	0.05255	-0.2658	0.2658	0.98593	0.95064	0.16248	0.49405	0.00172	0.17585	0.49485	0.00044	0.00044	green
ZKSCAN5	-0.04256	-0.23875	-0.08159	0.02411	-0.03037	-0.00954	0.09555	0.05747	-0.05747	0.58003	0.00166	0.28874	0.75423	0.69331	0.90146	0.21379	0.45529	0.45529	turquoise
CXXC1	-0.0502	-0.08856	-0.14314	-0.03849	0.20772	-0.18123	-0.05801	0.13026	-0.13026	0.51434	0.24938	0.0618	0.61722	0.00641	0.01768	0.45109	0.08947	0.08947	grey
ATF4	0.08808	0.03607	-0.03158	0.0839	0.1346	-0.06765	-0.13606	-0.17192	0.17192	0.25198	0.63949	0.68177	0.27526	0.07921	0.37935	0.07599	0.02455	0.02455	grey
SLC4A7	-0.04786	-0.17625	0.02615	0.08585	-0.09241	0.15079	0.02044	0.21147	-0.21147	0.53421	0.02111	0.73426	0.26421	0.22931	0.049	0.79069	0.00549	0.00549	turquoise
ZFYVE27	0.09983	-0.12603	-0.14361	-0.15471	0.22815	-0.2626	-0.08751	-0.15455	0.15455	0.19391	0.10048	0.06094	0.04334	0.00269	0.00052	0.25506	0.04355	0.04355	red
RAC1	0.00876	-0.06854	0.06301	0.02912	-0.09054	0.14402	0.2121	-0.41976	0.41976	0.90945	0.37305	0.41297	0.70536	0.2389	0.06021	0.00535	1.10E-08	1.10E-08	turquoise
KBTBD12	0.10464	0.10706	0.18954	0.06064	-0.03839	0.22452	0.19144	-0.30819	0.30819	0.17317	0.16342	0.01303	0.43077	0.61812	0.00315	0.01213	4.12E-05	4.12E-05	grey
KATNBL1	-0.0526	-0.13358	0.01008	0.02729	-0.02243	-0.01194	0.01325	-0.12197	0.12197	0.49447	0.08155	0.8959	0.72315	0.77088	0.87685	0.86344	0.11201	0.11201	red
RABAC1	0.04616	0.00662	-0.12357	-0.03475	0.20269	-0.16124	0.00964	-0.21608	0.21608	0.54885	0.9315	0.10736	0.65185	0.00784	0.03513	0.90041	0.00453	0.00453	green
RPS18	0.09034	0.19988	0.07699	0.00286	-0.07365	0.14323	0.18343	-0.17843	0.17843	0.23997	0.00876	0.31688	0.97042	0.33838	0.06165	0.01633	0.01955	0.01955	purple
ARL15	-0.13473	-0.14549	-0.14218	-0.05596	0.03141	-0.17813	-0.20365	0.45539	-0.45539	0.07893	0.05761	0.06359	0.46724	0.68345	0.01976	0.00755	3.90E-10	3.90E-10	turquoise
ESAM	-0.0871	-0.09685	-0.01602	0.02567	-0.11512	0.06987	-0.07805	0.28456	-0.28456	0.2573	0.20763	0.83524	0.73889	0.13379	0.36385	0.31023	0.00016	0.00016	blue
TRIP4	0.00799	-0.07492	-0.00505	-0.02033	-0.05584	0.03936	0.06085	-0.11978	0.11978	0.91744	0.33014	0.94777	0.79184	0.46821	0.60922	0.42914	0.11866	0.11866	turquoise
YPEL3	0.02548	-0.07943	0.0015	-0.05185	0.03021	-0.00672	0.0819	-0.18877	0.18877	0.74082	0.30172	0.9845	0.50059	0.69486	0.93052	0.28694	0.01341	0.01341	red
NARF	-0.02562	-0.17083	-0.0784	-0.02889	0.13261	-0.14208	0.0428	-0.1842	0.1842	0.73937	0.02549	0.30807	0.70761	0.08381	0.06378	0.57829	0.01588	0.01588	grey
MYCBP2	-0.06251	-0.09503	-0.03774	-0.00112	-0.14169	-0.00038	-0.07277	0.47418	-0.47418	0.41664	0.21633	0.62412	0.98842	0.06451	0.99611	0.3442	5.71E-11	5.71E-11	turquoise
SH2B1	-0.02097	-0.06567	-0.06817	-0.07417	0.15634	-0.03309	-0.02078	-0.10778	0.10778	0.78541	0.39348	0.37564	0.33497	0.04115	0.66747	0.78736	0.16058	0.16058	red
KEAP1	-0.01229	-0.00446	-0.18884	-0.0668	0.06156	-0.05208	0.0829	-0.13189	0.13189	0.87329	0.95385	0.01338	0.38531	0.42383	0.49874	0.28105	0.0855	0.0855	green
WNT4	0.03422	-0.13912	-0.25386	-0.15495	0.16398	-0.26517	-0.23074	0.2897	-0.2897	0.65682	0.06956	0.00081	0.04301	0.0321	0.00046	0.0024	0.00012	0.00012	brown
ROCK1	-0.03037	-0.2011	0.05851	0.10088	-0.17179	0.16624	0.03759	0.23443	-0.23443	0.69334	0.00835	0.44717	0.18921	0.02466	0.02978	0.62551	0.00203	0.00203	turquoise
ABLIM2	-0.04123	0.0066	-0.23184	-0.14742	0.03378	-0.27273	-0.16961	0.10769	-0.10769	0.59235	0.93168	0.00228	0.05433	0.66096	0.00031	0.02657	0.16092	0.16092	grey
C1QA	-0.10634	0.06206	0.04561	0.04549	-0.21774	0.13086	0.13207	0.28311	-0.28311	0.16627	0.42001	0.55359	0.55461	0.00422	0.08801	0.08509	0.00018	0.00018	blue
FBXO11	-0.06142	-0.17622	0.00025	0.06431	-0.14285	0.09422	0.02961	0.25151	-0.25151	0.42483	0.02114	0.99745	0.40334	0.06235	0.2203	0.70065	0.00091	0.00091	turquoise
NR1H4	0.05867	-0.13173	-0.04792	0.11312	0.02595	0.08985	0.05019	0.03112	-0.03112	0.44593	0.08589	0.53368	0.14071	0.73616	0.24255	0.51443	0.68617	0.68617	magenta
TBX2	-0.06524	-0.07999	-0.13098	0.00994	-0.02154	-0.05599	0.00484	0.2987	-0.2987	0.39656	0.29832	0.08771	0.89736	0.7798	0.46698	0.94985	7.24E-05	7.24E-05	black
SRD5A3	-0.02421	0.01315	0.17612	0.07965	0.06189	0.26155	0.17232	-0.33928	0.33928	0.75327	0.86445	0.02121	0.30039	0.42129	0.00055	0.02421	5.64E-06	5.64E-06	yellow
SASH1	-0.14928	-0.12208	-0.04963	0.04988	-0.15802	0.00768	-0.08709	0.59042	-0.59042	0.05133	0.11169	0.5192	0.51709	0.039	0.92054	0.25736	1.91E-17	1.91E-17	blue
ETNK2	-0.00234	-0.21792	-0.18308	-0.01125	-0.10406	-0.22436	-0.14552	0.27264	-0.27264	0.97582	0.00419	0.01654	0.88392	0.17558	0.00318	0.05755	0.00031	0.00031	brown
CENPBD1	-0.08714	-0.12168	-0.09883	-0.03069	-0.02769	-0.09466	-0.01292	0.27021	-0.27021	0.25711	0.11287	0.19844	0.69025	0.71922	0.21811	0.86679	0.00035	0.00035	turquoise
NXPE3	-0.11947	-0.05102	-0.13344	-0.09188	-0.15005	-0.11087	-0.13568	0.56257	-0.56257	0.11961	0.50748	0.08187	0.23202	0.05012	0.14885	0.07682	1.17E-15	1.17E-15	blue
PPFIA4	0.00286	-0.15411	0.02406	0.07077	0.07674	-0.01567	0.07718	-0.27913	0.27913	0.9704	0.04417	0.75473	0.35768	0.31848	0.83883	0.31568	0.00022	0.00022	grey
C17orf49	-0.11707	-0.10105	-0.0707	0.0036	0.05247	-0.09067	0.12665	0.17946	-0.17946	0.12729	0.18846	0.35815	0.96271	0.49553	0.23821	0.0988	0.01884	0.01884	grey
ERMAP	-0.07777	-0.14074	-0.1237	-0.04361	-0.07099	-0.06252	-0.08636	0.3552	-0.3552	0.31196	0.06635	0.10698	0.57114	0.35617	0.41662	0.26138	1.87E-06	1.87E-06	turquoise
UBXN1	0.09931	0.02827	-0.06634	-0.08423	-0.0401	-0.0202	0.1226	-0.23788	0.23788	0.19625	0.71359	0.38862	0.27339	0.60254	0.79312	0.11015	0.00173	0.00173	grey
FAM166C	0.11714	0.00142	-0.0392	-0.0952	0.21826	-0.00765	0.03821	-0.23825	0.23825	0.12704	0.98531	0.61075	0.21551	0.00413	0.92093	0.61981	0.0017	0.0017	grey
CCDC9B	-0.0025	-0.08833	-0.07021	-0.02554	-0.08934	0.02563	0.16505	-0.24793	0.24793	0.97408	0.25061	0.36148	0.74019	0.24524	0.73935	0.03098	0.00108	0.00108	yellow
TPST2	-0.14515	0.01964	0.0344	0.09132	-0.18679	-0.04945	-0.06387	0.25805	-0.25805	0.05819	0.79871	0.65508	0.23486	0.01443	0.52072	0.40656	0.00066	0.00066	magenta
OSR1	-0.02027	-0.0945	-0.03768	0.12226	-0.10298	0.11028	0.08386	0.44298	-0.44298	0.79242	0.21892	0.62468	0.11115	0.18015	0.15101	0.27549	1.30E-09	1.30E-09	blue
CHCHD10	0.15827	0.16717	-0.10157	-0.08639	0.09118	-0.04921	0.0991	-0.41512	0.41512	0.03869	0.02886	0.1862	0.26124	0.23559	0.52269	0.19718	1.65E-08	1.65E-08	green
PRELID1	0.01281	0.04093	0.02644	-0.03218	-0.0238	0.06152	0.18458	-0.3503	0.3503	0.86795	0.59508	0.73142	0.67611	0.75733	0.4241	0.01566	2.64E-06	2.64E-06	pink
EIF2B2	0.01072	-0.10823	-0.00862	-0.00074	0.03006	0.08336	0.06283	-0.22825	0.22825	0.8893	0.15881	0.91089	0.99234	0.69628	0.27841	0.41431	0.00268	0.00268	turquoise
SLC26A1	-0.02191	0.01605	-0.0882	-0.01634	0.25208	-0.07382	-0.03114	-0.12489	0.12489	0.77602	0.83492	0.2513	0.83202	0.00088	0.33731	0.68602	0.10362	0.10362	red
PDE4C	0.09829	0.10936	0.0546	0.00118	0.14866	0.13592	0.07689	-0.42884	0.42884	0.20091	0.15448	0.47813	0.9878	0.05232	0.07631	0.31754	4.86E-09	4.86E-09	grey
FHL5	-0.06722	-0.10889	-0.05779	0.0779	-0.12017	0.08283	-0.06335	0.49712	-0.49712	0.38235	0.15627	0.4528	0.31121	0.11746	0.28144	0.41045	4.64E-12	4.64E-12	blue
TMEM220	-0.09618	-0.14311	-0.03557	0.05326	0.04466	0.02082	-0.10949	0.27262	-0.27262	0.21081	0.06186	0.64414	0.489	0.56195	0.78697	0.15399	0.00031	0.00031	grey
DKK1	-0.03626	-0.18912	0.17688	0.11101	-0.1782	0.15751	0.1991	-0.2531	0.2531	0.6378	0.01324	0.02065	0.14832	0.01971	0.03965	0.00904	0.00084	0.00084	yellow
SNRK	-0.11769	-0.17005	-0.01463	0.0568	-0.12179	0.11704	-0.09457	0.33049	-0.33049	0.12526	0.02618	0.84938	0.46056	0.11255	0.12739	0.21855	1.01E-05	1.01E-05	turquoise
SEC31B	-0.03496	-0.08869	-0.07217	-0.07858	0.18889	-0.21254	-0.12294	0.15732	-0.15732	0.64991	0.24867	0.34823	0.30698	0.01335	0.00526	0.10917	0.03989	0.03989	red
FKBP2	0.0401	-0.10943	-0.16719	-0.14418	0.23543	-0.38548	-0.13248	-0.1278	0.1278	0.60257	0.15421	0.02884	0.05992	0.00194	1.93E-07	0.08412	0.09576	0.09576	grey
VCP	0.00124	-0.10456	-0.07524	-0.06344	-0.08388	-0.02563	0.01344	0.09535	-0.09535	0.98717	0.1735	0.32803	0.40972	0.2754	0.73933	0.86151	0.21479	0.21479	turquoise
SPON2	-0.08884	-0.06758	-0.06992	0.01024	-0.03766	0.08692	0.08454	0.06879	-0.06879	0.24789	0.37979	0.36347	0.89426	0.62483	0.25829	0.27162	0.37132	0.37132	black
MT1H	-0.0265	0.10433	0.16173	0.14521	-0.10075	0.19505	0.03913	0.00014	-0.00014	0.7308	0.17445	0.03457	0.05809	0.1898	0.01057	0.6114	0.9985	0.9985	magenta
PLCL2	-0.08149	-0.134	-0.10263	-0.06029	-0.00612	-0.02954	-0.11184	0.40547	-0.40547	0.28936	0.08058	0.18164	0.43345	0.93669	0.70128	0.1453	3.77E-08	3.77E-08	turquoise
BRD4	0.08074	-0.1877	-0.04836	0.02562	-0.05773	0.0093	0.01637	0.02324	-0.02324	0.2938	0.01396	0.52992	0.73946	0.45326	0.90392	0.83169	0.76282	0.76282	turquoise
INPP5A	0.009	-0.13963	0.01331	0.09526	-0.00868	0.0475	-0.01221	0.02295	-0.02295	0.90699	0.06854	0.86286	0.2152	0.91027	0.53731	0.87407	0.76579	0.76579	turquoise
TBC1D1	-0.13122	-0.08296	-0.02838	0.05075	-0.11344	0.09439	0.03125	0.12546	-0.12546	0.08712	0.28071	0.71255	0.5098	0.13958	0.21946	0.68495	0.10203	0.10203	turquoise
ARL4C	-0.02579	-0.0932	0.06397	0.08252	-0.23294	0.18218	0.13349	0.04056	-0.04056	0.73771	0.22533	0.40583	0.28327	0.00217	0.01709	0.08176	0.59842	0.59842	black
PDE10A	-0.00166	-0.18021	0.01803	0.08546	-0.00866	-0.04421	-0.11008	0.37912	-0.37912	0.9828	0.01834	0.81496	0.26638	0.91052	0.56585	0.15178	3.16E-07	3.16E-07	turquoise
EMC3	0.04923	-0.0976	-0.02557	-0.07625	-0.11363	0.10055	0.17377	-0.21775	0.21775	0.52254	0.2041	0.73995	0.32156	0.13893	0.1907	0.02302	0.00422	0.00422	turquoise
LDB2	-0.17	-0.18053	-0.10663	-0.03269	-0.1028	-0.01028	-0.15049	0.59732	-0.59732	0.02622	0.01813	0.16509	0.67125	0.18091	0.89388	0.04946	6.49E-18	6.49E-18	blue
NEPRO	-0.06515	-0.09184	-0.0612	-0.06438	-0.15004	0.06924	0.03617	0.08612	-0.08612	0.39723	0.23223	0.42655	0.40285	0.05014	0.3682	0.63863	0.26274	0.26274	turquoise
ZNF117	0.00192	-0.12481	0.04752	0.0419	0.04911	0.12132	0.1203	-0.13528	0.13528	0.98013	0.10384	0.53715	0.58632	0.52354	0.11395	0.11704	0.0777	0.0777	grey
SHISA2	-0.02041	-0.08659	-0.0037	-0.03317	-0.03628	0.16888	-0.01451	0.01875	-0.01875	0.791	0.26014	0.9617	0.66673	0.63755	0.02724	0.85059	0.80772	0.80772	black
TMEM192	-0.08116	-0.0425	-0.17203	-0.0246	-0.01851	-0.09097	-0.10499	0.34787	-0.34787	0.29133	0.58102	0.02446	0.74939	0.81009	0.2367	0.17173	3.13E-06	3.13E-06	turquoise
POU2F3	0.01078	-0.00291	0.04963	0.03089	-0.09242	0.19073	0.07918	-0.24119	0.24119	0.88875	0.96988	0.51915	0.68836	0.22925	0.01246	0.30326	0.00148	0.00148	grey
PRKCZ	0.00422	-0.01192	-0.01559	-0.0592	0.04129	0.03821	-0.03941	-0.25715	0.25715	0.95634	0.87707	0.83963	0.44184	0.59179	0.61973	0.60884	0.00069	0.00069	grey
EEF1AKMT	-0.00954	-0.0102	-0.08563	-0.09702	0.0597	-0.21357	0.02725	-0.02528	0.02528	0.90144	0.89467	0.26547	0.20682	0.43795	0.00503	0.72348	0.74278	0.74278	turquoise
ATAD2	-0.01664	-0.05513	-0.02435	-0.03672	-0.09694	-0.04558	0.17855	-0.12678	0.12678	0.82901	0.47392	0.75191	0.63346	0.20717	0.5539	0.01946	0.09846	0.09846	turquoise
AGTRAP	0.02035	0.0217	0.04446	0.08169	-0.13324	0.18286	0.21134	-0.30189	0.30189	0.79159	0.77817	0.56363	0.28813	0.08233	0.01667	0.00552	6.00E-05	6.00E-05	grey
OSBP	-0.01185	-0.11303	-0.04209	0.0281	-0.12712	0.0064	0.02537	0.05746	-0.05746	0.87778	0.14104	0.58465	0.71527	0.09756	0.93379	0.74183	0.45535	0.45535	turquoise
RPS6KA3	-0.08224	-0.17696	-0.04028	0.00807	-0.04103	0.00038	-0.08846	0.19077	-0.19077	0.28491	0.02059	0.60091	0.91653	0.59414	0.99608	0.24992	0.01244	0.01244	turquoise

ZDHHC24	-0.01246	-0.01731	-0.20812	-0.09138	0.07053	-0.21663	0.01878	-0.08125	0.08125	0.87156	0.82216	0.00631	0.23458	0.35929	0.00443	0.80743	0.2908	0.2908 green
YY1	0.03961	-0.12601	-0.01684	0.05959	-0.0304	0.03049	-0.02533	-0.05729	0.05729	0.60703	0.10053	0.827	0.43884	0.69306	0.69223	0.74223	0.45671	0.45671 turquoise
GFM2	0.06207	-0.08439	-0.07221	-0.07231	0.10464	-0.11483	-0.09573	0.10376	-0.10376	0.41996	0.27246	0.34798	0.3473	0.17319	0.13476	0.21293	0.17687	0.17687 turquoise
MTG2	0.08508	0.01759	-0.11117	-0.07016	0.14377	-0.18165	0.04018	-0.15207	0.15207	0.26852	0.81934	0.14773	0.36184	0.06065	0.01741	0.60186	0.04708	0.04708 grey
TMEM163	0.05824	0.01226	0.0294	-0.06043	0.04746	0.04735	-0.15159	-0.03856	0.03856	0.44923	0.87359	0.70271	0.43236	0.53762	0.5386	0.0478	0.61658	0.61658 grey
SALL4	0.00382	-0.15221	-0.00635	0.07638	-0.08209	-0.00834	0.09874	0.01294	-0.01294	0.96044	0.04688	0.93435	0.32077	0.28581	0.91377	0.19884	0.86656	0.86656 grey
ISM1	-0.05706	-0.07888	0.04953	0.0436	-0.19575	0.17767	0.09878	0.23501	-0.23501	0.45853	0.30514	0.51998	0.57125	0.01029	0.02008	0.19866	0.00197	0.00197 black
SIRPB1	-0.10188	0.071	0.15853	0.09314	-0.19902	0.16184	0.06936	0.2057	-0.2057	0.18485	0.35611	0.03837	0.22564	0.00906	0.03444	0.36738	0.00695	0.00695 blue
NCALD	0.01451	-0.12668	-0.18426	-0.05427	0.02933	-0.26063	-0.31058	0.52613	-0.52613	0.85057	0.09872	0.01584	0.4808	0.70331	0.00058	3.56E-05	1.47E-13	1.47E-13 brown
GOT1	0.15223	-0.07052	-0.12523	-0.1661	0.11665	-0.26101	-0.05631	-0.13571	0.13571	0.04685	0.35936	0.10269	0.02992	0.12867	0.00056	0.46446	0.07676	0.07676 grey
MB21D2	-0.0984	-0.21158	-0.02721	0.01933	-0.10096	0.05026	-0.08914	0.36486	-0.36486	0.20041	0.00547	0.72392	0.8019	0.18886	0.51383	0.24629	9.28E-07	9.28E-07 blue
LGALS1	-0.08566	-0.02185	0.02966	0.09545	-0.14438	0.11676	0.1227	0.02428	-0.02428	0.2653	0.77669	0.70017	0.2143	0.05956	0.12829	0.10986	0.75256	0.75256 black
MAN2B1	-0.06533	-0.04423	0.0248	0.04965	-0.09338	0.10858	0.05472	0.19904	-0.19904	0.39591	0.5657	0.7475	0.51897	0.22445	0.15745	0.47722	0.00906	0.00906 blue
SEZ6L	-0.03168	-0.15289	-0.22133	-0.12583	0.06327	-0.32333	-0.23539	0.46601	-0.46601	0.68081	0.0459	0.00362	0.10102	0.411	1.61E-05	0.00194	1.34E-10	1.34E-10 brown
ZNF860	-0.02441	-0.10157	-0.05244	-0.09998	-0.07945	0.02865	0.01614	-0.15966	0.15966	0.75128	0.18621	0.49572	0.19321	0.30162	0.70988	0.83401	0.03698	0.03698 turquoise
RAB5A	-0.03127	-0.13281	0.02353	-0.04083	-0.11167	0.14353	0.06441	-0.06326	0.06326	0.68472	0.08335	0.76002	0.59596	0.14593	0.06109	0.40264	0.41108	0.41108 turquoise
CLEC4E	-0.13546	0.02507	0.05987	0.03911	-0.16252	0.12029	0.00875	0.3561	-0.3561	0.07731	0.74479	0.43668	0.61157	0.03369	0.11707	0.90954	1.75E-06	1.75E-06 blue
CDK1	0.00647	0.06394	0.0222	-0.01686	-0.06102	0.05312	0.31375	-0.45391	0.45391	0.9331	0.40609	0.77322	0.82679	0.42786	0.49016	2.93E-05	4.51E-10	4.51E-10 pink
YME1L1	-0.0347	-0.10274	0.06355	0.03248	-0.12373	0.09379	0.04587	0.10309	-0.10309	0.65229	0.18116	0.40896	0.67326	0.1069	0.22239	0.55132	0.17968	0.17968 turquoise
ERP27	-0.04642	0.01383	0.08908	0.18213	-0.16477	0.10656	-0.0626	0.11871	-0.11871	0.54657	0.85751	0.24661	0.01711	0.03128	0.1654	0.41596	0.12199	0.12199 magenta
KLHL20	-0.06626	-0.10472	0.03026	0.02045	-0.08936	0.16796	0.05359	0.23321	-0.23321	0.38919	0.17283	0.69436	0.79059	0.24514	0.02811	0.48636	0.00214	0.00214 turquoise
GON7	-0.01938	-0.0901	-0.16216	0.02317	0.11701	-0.15299	-0.144	0.11486	-0.11486	0.80133	0.2412	0.03409	0.76357	0.12749	0.04575	0.06024	0.13468	0.13468 turquoise
CRISPLD1	-0.09494	-0.129	-0.01646	0.01515	-0.14624	0.12394	-0.00022	0.50667	-0.50667	0.21674	0.09266	0.83076	0.84408	0.05631	0.10629	0.99776	1.54E-12	1.54E-12 blue
MAP3K9	0.13774	-0.1592	-0.11127	-0.071	0.01486	-0.19556	-0.04653	-0.13196	0.13196	0.07241	0.03754	0.14737	0.35609	0.84701	0.01037	0.54565	0.08536	0.08536 grey
KLF12	-0.01888	-0.12246	-0.01275	-0.02193	-0.25463	0.00128	-0.09034	0.47881	-0.47881	0.80643	0.11057	0.86855	0.77585	0.00078	0.98676	0.23994	3.49E-11	3.49E-11 blue
CEP104	-0.03569	-0.15732	0.00642	0.03526	-0.08076	0.08552	-0.01654	0.1755	-0.1755	0.64304	0.03988	0.9336	0.64703	0.29369	0.26607	0.82998	0.02168	0.02168 turquoise
PVALB	-0.15526	0.13889	0.06126	0.07459	-0.11317	-0.01201	-0.00733	0.23781	-0.23781	0.04259	0.07004	0.42605	0.33225	0.14054	0.87612	0.9242	0.00174	0.00174 grey
NKD2	0.00789	-0.05385	-0.05025	0.05096	0.00657	0.01093	0.08302	0.04758	-0.04758	0.91843	0.48419	0.51394	0.50801	0.93208	0.8872	0.28036	0.5366	0.5366 black
RPRD2	0.03788	-0.08803	-0.0657	-0.06534	-0.04748	0.02886	-0.06328	0.13147	-0.13147	0.6228	0.25226	0.39327	0.39584	0.53747	0.70792	0.41095	0.08652	0.08652 turquoise
ACYP2	-0.02478	-0.12821	-0.20119	-0.13762	0.05154	-0.16622	-0.12929	0.20327	-0.20327	0.74768	0.09468	0.00832	0.07267	0.50315	0.0298	0.09193	0.00767	0.00767 brown
EIF3L	-0.06844	-0.06032	-0.03253	-0.01874	-0.11778	0.0855	-0.15382	0.38994	-0.38994	0.37376	0.43323	0.67273	0.80777	0.12497	0.26616	0.04458	1.35E-07	1.35E-07 turquoise
MEMO1	-0.03194	-0.12503	-0.0245	0.05896	-0.01661	-0.01024	0.11735	0.11097	-0.11097	0.67834	0.10323	0.75039	0.44368	0.82927	0.89421	0.12636	0.14849	0.14849 turquoise
KIAA0930	-0.07006	-0.03719	0.00274	0.06967	-0.125	-0.02806	0.09707	0.20647	-0.20647	0.36251	0.62915	0.97166	0.36524	0.10331	0.71565	0.20656	0.00674	0.00674 black
APEX1	-0.02258	-0.00338	0.03004	0.05016	-0.09773	0.07598	0.10267	-0.1271	0.1271	0.76941	0.96501	0.69655	0.51474	0.20351	0.32331	0.18148	0.0976	0.0976 turquoise
ZNF555	0.01712	-0.15449	-0.10379	-0.04734	-0.01777	-0.02058	-0.10901	0.32051	-0.32051	0.82416	0.04365	0.17672	0.53862	0.81754	0.78938	0.15581	1.92E-05	1.92E-05 turquoise
TMEM126	-0.00545	-0.04153	-0.04809	-0.03596	-0.05035	0.00203	0.05038	0.02529	-0.02529	0.94358	0.58969	0.53226	0.64053	0.51309	0.97902	0.51285	0.74261	0.74261 turquoise
RXRG	-0.05643	-0.07224	-0.173	-0.05067	0.05254	-0.19936	-0.11998	0.39895	-0.39895	0.46353	0.34778	0.02365	0.51048	0.49495	0.00894	0.11803	6.49E-08	6.49E-08 brown
LFNG	0.04635	0.09068	0.09649	0.03007	-0.00912	0.25223	0.11294	-0.45598	0.45598	0.54717	0.23819	0.20931	0.69619	0.90573	0.00087	0.14136	3.68E-10	3.68E-10 yellow
TARBP2	0.11513	-0.02385	-0.16629	-0.06028	0.2055	-0.27377	-0.06723	-0.18075	0.18075	0.13377	0.7568	0.02973	0.43354	0.00701	0.00029	0.3823	0.01799	0.01799 grey
ZFAND2A	0.09909	-0.12767	0.03948	-0.03533	0.06761	0.08323	0.07584	-0.2752	0.2752	0.19726	0.09608	0.60815	0.64641	0.37961	0.27916	0.32421	0.00027	0.00027 grey
HSPD1	0.12346	-0.01509	0.01591	0.03598	-0.01037	0.06861	0.13166	-0.29679	0.29679	0.10767	0.84469	0.83641	0.6404	0.89296	0.3726	0.08607	8.09E-05	8.09E-05 turquoise
KPNA4	-0.00115	-0.08018	0.08007	0.08654	-0.19466	0.21712	0.16908	-0.06072	0.06072	0.98807	0.29716	0.29784	0.26038	0.01073	0.00434	0.02705	0.43013	0.43013 turquoise
AP1G2	0.00464	-0.04112	0.034	0.02129	0.17828	0.01985	-0.02635	-0.35684	0.35684	0.95202	0.5933	0.65882	0.78229	0.01965	0.79667	0.73231	1.66E-06	1.66E-06 red
SSBP1	0.0005	0.01101	0.0044	-0.06582	-0.0223	-0.09126	0.13867	-0.30818	0.30818	0.99481	0.88639	0.95441	0.3924	0.77222	0.23518	0.07049	4.12E-05	4.12E-05 turquoise
NUDT11	-0.09353	-0.04887	-0.02144	0.0969	-0.15208	-0.08395	-0.05757	0.5647	-0.5647	0.22369	0.5256	0.78079	0.2074	0.04706	0.275	0.45453	8.66E-16	8.66E-16 blue
PPP1R21	-0.03825	-0.12846	-0.09446	-0.06311	-0.04944	0.02406	-0.02763	0.15626	-0.15626	0.61942	0.09404	0.21912	0.41217	0.52077	0.75477	0.71982	0.04126	0.04126 turquoise
FUCA2	0.00898	0.00447	-0.00662	-0.01322	-0.04309	-0.13561	0.05267	-0.01211	0.01211	0.90716	0.95369	0.93156	0.86374	0.57578	0.07698	0.49386	0.87512	0.87512 grey
C1RL	-0.04714	-0.03602	-0.02559	0.06295	-0.14854	0.18184	0.11576	-0.03285	0.03285	0.54034	0.63998	0.73969	0.41335	0.05251	0.0173	0.13161	0.66968	0.66968 turquoise
CXCL11	-0.1254	0.20221	0.035	0.09229	-0.17648	0.13273	0.16616	0.00318	-0.00318	0.1022	0.008	0.64947	0.22992	0.02094	0.08352	0.02986	0.96703	0.96703 blue
CYP26B1	-0.13937	-0.0742	0.02525	0.12536	-0.08314	-0.09073	-0.00245	0.3549	-0.3549	0.06907	0.3348	0.74301	0.10231	0.27963	0.23794	0.97467	1.91E-06	1.91E-06 blue
SPTAN1	0.00766	-0.14595	-0.05656	0.00818	-0.10923	0.10048	0.06241	0.06396	-0.06396	0.92084	0.05682	0.46248	0.91544	0.155	0.19101	0.4174	0.40593	0.40593 turquoise
CNTLN	-0.02924	-0.06425	-0.10155	-0.00692	-0.18772	0.0172	0.05645	0.38212	-0.38212	0.70423	0.40376	0.18631	0.92838	0.01394	0.82335	0.46336	2.51E-07	2.51E-07 blue
FAM110D	-0.10638	-0.0562	-0.11027	0.03874	-0.08009	-0.05599	-0.09569	0.50853	-0.50853	0.16609	0.46531	0.15107	0.61493	0.29776	0.46699	0.21313	1.24E-12	1.24E-12 blue
RNPS1	0.07382	-0.04954	-0.02804	-0.04458	0.01858	-0.03017	0.03756	-0.23313	0.23313	0.3373	0.51995	0.71583	0.5626	0.80936	0.69525	0.62574	0.00215	0.00215 grey
UQCC1	-0.03247	-0.08135	-0.10783	0.02047	0.07053	-0.21087	-0.03593	-0.00677	0.00677	0.67333	0.29016	0.16037	0.79048	0.3593	0.00563	0.64085	0.92995	0.92995 turquoise
MACROD2	0.02542	-0.08987	-0.05766	0.04068	0.18301	-0.04493	-0.09163	-0.03261	0.03261	0.74138	0.24244	0.45382	0.59726	0.01658	0.55955	0.2333	0.67196	0.67196 grey
LRP8	-0.02908	0.07568	0.04461	-0.00427	-0.02351	-0.02152	0.01	-0.16815	0.16815	0.70576	0.32524	0.56233	0.95576	0.7602	0.77992	0.89672	0.02792	0.02792 grey
FRMPD3	0.07404	-0.16478	-0.18309	-0.01711	0.00863	-0.31763	-0.10328	0.19872	-0.19872	0.33581	0.03126	0.01653	0.82426	0.9108	2.30E-05	0.17885	0.00917	0.00917 grey
GREM1	-0.05472	-0.02144	0.0541	0.06915	-0.18259	0.14112	0.10192	0.199	-0.199	0.47715	0.78073	0.48222	0.36881	0.01683	0.06561	0.18469	0.00907	0.00907 blue
PKNOX1	0.02574	-0.18782	-0.19633	-0.05728	-0.00308	-0.07186	-0.01991	0.30612	-0.30612	0.73829	0.0139	0.01006	0.45679	0.96816	0.35027	0.79602	4.66E-05	4.66E-05 turquoise
GZMK	-0.05595	0.00067	0.05111	0.08606	-0.1661	0.11899	-0.01691	0.46022	-0.46022	0.46733	0.99304	0.50673	0.26305	0.02991	0.12113	0.82624	2.41E-10	2.41E-10 blue
SEC24D	-0.01387	-0.17064	0.01626	0.00181	-0.02125	0.01125	-0.05559	0.28374	-0.28374	0.85708	0.02565	0.83282	0.9813	0.7826	0.88387	0.47018	0.00017	0.00017 turquoise
NLRC5	-0.04478	0.0988	0.04693	0.02184	-0.12299	0.11283	0.13956	0.07633	-0.07633	0.56082	0.19854	0.54221	0.77676	0.10903	0.14176	0.06868	0.32104	0.32104 tan
RNF25	0.04283	-0.04481	-0.1249	-0.0349	0.07052	-0.07633	0.01151	-0.07658	0.07658	0.57804	0.56057	0.10358	0.65043	0.35937	0.3211	0.88118	0.31951	0.31951 grey
P4HTM	0.05434	-0.17613	-0.19236	-0.14523	0.23864	-0.27947	-0.21869	0.00463	-0.00463	0.48029	0.0212	0.01172	0.05806	0.00167	0.00021	0.00406	0.95208	0.95208 brown
TP53BP1	-0.04967	-0.22679	-0.09142	-0.02453	-0.10375	-0.05252	-0.04061	0.2818	-0.2818	0.51884	0.00285	0.23434	0.75016	0.17687	0.4951	0.59791	0.00019	0.00019 turquoise
AURKC	0.03181	-0.08144	-0.13914	-0.02117	0.00519	-0.12024	-0.01185	0.02176	-0.02176	0.67961	0.28965	0.06952	0.7834	0.94629	0.11724	0.8777	0.77757	0.77757 grey
TOMM34	0.04004	-0.03129	-0.03099	-0.00667	0.04852	-0.03833	0.08937	-0.28794	0.28794	0.60313	0.68453	0.6874	0.93099	0.52854	0.61864	0.24509	0.00013	0.00013 turquoise
PTPN20	0.16161	-0.09633	0.0643	0.10949	0.06079	-0.02471	-0.03289	-0.07634	0.07634	0.03471	0.21006	0.40339	0.15399	0.42962	0.74838	0.66936	0.32101	0.32101 grey
NPIPA1	0.00841	-0.03767	0.02246	-0.09195	0.14999	-0.14436	-0.15806	0.03828	-0.03828	0.91305	0.6247	0.7706	0.23166	0.05023	0.05959	0.03895	0.61909	0.61909 grey
MPRIP	-0.03809	-0.23317	-0.00167	0.02954	-0.08175	0.0115	0.06018	0.19107	-0.19107	0.62089	0.00215	0.98271	0.70136	0.28777	0.88131	0.43424	0.0123	0.0123 turquoise
RIMKLA	-0.02414	-0.08957	-0.06264	-0.02232	0.16269	-0.05136	-0.19976	-0.01842	0.01842	0.75394	0.24399	0.41566	0.77202	0.0335	0.50471	0.00881	0.81099	0.81099 grey
WWTR1	-0.11161	-0.16938	-0.02992	0.13241	-0.22413	0.14212	0.06822	0.22013	-0.22013	0.14615	0.02678	0.69769	0.08428	0.00321	0.0637	0.37533	0.00381	0.00381 blue
ABCB9	0.09229	-0.10723	-0.03325	-0.07089	0.07355	-0.08154	-0.02791	-0.26816	0.26816	0.22993	0.16273	0.66598	0.35685	0.33908	0.28903	0.71709	0.00039	0.00039 grey
LTB4R2	-0.01074	-0.09196	-0.06893	-0.01663	0.20521	-0.09195	-0.01861	0.06712	-0.06712	0.88914	0.2316	0.37033	0.82907	0.00709	0.23163	0.80905	0.38308	0.38308 red
ZNF35	-0.05691	-0.15322	-0.05587	-0.05253	-0.12026	0.0963	0.03235	0.21385	-0.21385	0.45974	0.04542	0.46793	0.49499	0.11717	0.21023	0.6745	0.00498	0.00498 turquoise
KNOP1	0.00403	0.012	-0.0041	-0.0117	-0.0779	0.19397	0.26085	-0.22162	0.22162	0.95823	0.87616	0.95752	0.87929	0.31119	0.01102	0.00057	0.00358	0.00358 turquoise
HHIP	-0.07182	-0.12233	0.10017	0.01396	-0.12156	0.14277	0.04183	0.00717	-0.00717	0.35054	0.11096	0.1924	0.85624	0.11324	0.06249	0.58695	0.92584	0.92584 black
FAM200B	-0.08497	-0.21778	-0.21864	-0.11641	0.1181	-0.23056	-0.12558	0.25752	-0.25752	0.26915	0.00422	0.00407	0.12945	0.12395	0.00242	0.10171	0.00067	0.00067 brown
CYP4B1	0.03744	-0.02977	0.02861	0.05902	0.07844	0.02767	0.00957	-0.01216	0.01216	0.62688	0.69915	0.71027	0.4432	0.3078	0.71942	0.90112	0.87453	0.87453 grey
PMAIP1	-0.02166	-0.0338	0.03068	-0.01196	-0.03714	0.02989	0.08086	-0.16544	0.16544	0.77856	0.66073	0.69042	0.87659	0.62961	0.698	0.29311	0.03058	0.03058 grey
RANBP10	-0.01865	-0.11194	-0.04594	-0.02121	-0.06442	0.0381	0.02342	0.13163	-0.13163	0.80872	0.14495	0.55075	0.78305	0.40254	0.62076	0.76104	0.08613	0.08613 turquoise
TOP2B	-0.0387	-0.14648	-0.06439	-0.07151	-0.13579	0.08233	-0.03543	0.27327	-0.27327	0.61531	0.05591	0.40279	0.35264	0.07659	0.2844	0.64544	0.0003	0.0003 turquoise
GINS2	-0.07233	0.11573	-0.06328	-0.09883	-0.00679	-0.01657	0.25822	-0.41666	0.41666	0.34718	0.13173	0.4109	0.19844	0.92975	0.8297	0.00065	1.44E-08	1.44E-08 pink
MFN1	-0.10309	-0.1528	-0.0195	0.03121	-0.06706	0.10536	0.11561	-0.01255	0.01255	0.17967	0.04601	0.80011	0.68531	0.38351	0.17023	0.13213	0.87062	0.87062 turquoise
HDAC6	-0.06331	-0.19592	-0.11872	-0.04831	0.04069	-0.10055	-0.02851	0.08232	-0.08232	0.41068	0.01023	0.12196	0.53039	0.59725	0.19067	0.71123	0.28444	0.28444 red
PTP4A3	-0.05731	0.01143	-0.22967	0.00541	0.01007	-0.16369	-0.05752	0.28634	-0.28634	0.45656	0.882	0.00251	0.94405	0.89599	0.03242	0.45488	0.00015	0.00015 grey
RTN1	-0.02474	-0.12716	-0.21019	-0.1233	0.03826	-0.27097	-0.22338	0.52225	-0.52225	0.74807	0.09744	0.00579	0.10812	0.61935	0.00034	0.00332	2.38E-13	2.38E-13 brown
RPAIN	-0.03759	-0.14828	-0.17289	-0.11047	0.17725	-0.29049	-0.11979	0.13577	-0.13577	0.62549	0.05292	0.02374	0.15033	0.02038	0.00012	0.11862	0.07662	0.07662 brown
TMEM38B	-0.01466	-0.06828	-0.13536	-0.01249	0.07254	-0.27067	-0.14556	0.33287	-0.33287	0.84904	0.37491	0.07752	0.87123	0.34572	0.00034	0.05748	8.65E-06	8.65E-06 turquoise
TXNDC11	-0.03934	-0.06472	-0.08471	-0.03958	0.01811	0.0889	0.04534	-0.03351	0.03351	0.60941	0.40035	0.27064	0.60728	0.81414	0.24755	0.55592	0.66352	0.66352 turquoise
STARD13	-0.08513	-0.12296	-0.03416	0.0446	-0.17772	-0.04828	-0.04577	0.55404	-0.55404	0.26824	0.1091	0.65733	0.56245	0.02004	0.53058	0.55218	3.82E-15	3.82E-15 blue
TLR1	-0.15695	-0.06442	0.08032	0.05228	-0.18405	0.22451	0.11695	0.29581	-0.29581	0.04036	0.40257	0.29634	0.49709	0.01596	0.00316	0.12769	8.56E-05	8.56E-05 blue
BANK1	-0.12256	-0.05442	0.03477	-0.05373	-0.11665	0.11105	-0.04075	0.1781	-0.1781	0.11029	0.47964	0.65167	0.4852	0.12865	0.14817	0.59671	0.01978	0.01978 blue
BCAS3	0.00519	-0.15797	-0.17171	-0.05082	-0.03621	-0.06352	-0.05566	0.19982	-0.19982	0.94627	0.03906	0.02472	0.50914	0.63824	0.40919	0.4696	0.00878	0.00878 turquoise
ROM1	-0.08443	-0.06053	-0.19068	-0.09798	0.06213	-0.22426	-0.13284	0.16316	-0.16316	0.27226	0.4316	0.01248	0.20235	0.41951	0.00319	0.08327	0.03299	0.03299 grey
GOSR2	-0.02308	-0.17558	-0.13455	-0.01136	0.05999	-0.12183	-0.035	0.19656	-0.19656	0.76445	0.02161	0.07933	0.88274	0.43575	0.11242	0.64949	0.00997	0.00997 turquoise
CLEC2B	-0.03994	-0.00305	0.06196	0.13404	-0.15481	0.13112	0.15935	0.16055	-0.16055	0.60397	0.96843	0.42078	0.08049	0.0432	0.08737	0.03736	0.03594	0.03594 black
TRNP1	-0.0225	-0.04848	0.09682	-0.00579	0.07899	0.14483	-0.02137	-0.37621	0.37621	0.77018	0.52888	0.20774	0.94013	0.30442	0.05877	0.78144	3.96E-07	3.96E-07 grey
MMP19	-0.09664	-0.1117	0.00012	0.06866	-0.1465	0.09552	0.0169	0.43567	-0.43567	0.2086	0.1458	0.99872	0.37221	0.05587	0.21398	0.82633	2.59E-09	2.59E-09 blue
PSMB8	-0.0248	0.18059	0.03072	0.03936	-0.06852	0.17343	0.24906	-0.32006	0.32006	0.74747	0.0181	0.68999	0.60926	0.37317	0.0233	0.00102	1.98E-05	1.98E-05 tan
PSMA4	-0.06961	-0.04225	-0.03675	-0.01386	-0.03078	-0.00861	0.15344	-0.2015	0.2015	0.36564	0.58319	0.63319	0.85724	0.68938	0.91106	0.04511	0.00822	0.00822 turquoise
RAPH1	0.05646	-0.11858	0.06829	0.04779	-0.12098	0.22187	0.14109	-0.02551	0.02551	0.46326	0.12241	0.37479	0.53477	0.11497	0.00354	0.06567	0.74054	0.74054 turquoise
CELF4	-0.03414	-0.1371	-0.23298	-0.10019	0.13498	-0.34668	-0.23884	0.41022	-0.41022	0.65754	0.07376	0.00216	0.19227	0.07838	3.40E-06	0.00166	2.52E-08	2.52E-08 brown
IFNE	0.04624	0.09688	0.00993	-0.04607	-0.0932	0.03479	0.2204	-0.26471	0.26471	0.54812	0.20745	0.89748	0.54961	0.22536	0.65146	0.00377	0.00047	0.00047 grey
MRPS33	-0.00999	-0.03373	-0.08918	-0.13433	0.16925	-0.24784	-0.04405	-0.09525	0.09525	0.89678	0.6614	0.24605	0.07983	0.0269	0.00108	0.56728	0.21524	0.21524 grey
SIPA1L1	0.10512	-0.09185	0.1274	0.10127	-0.11336	0.14537	0.10333	-0.02441	0.02441	0.1712	0.23217	0.09681	0.18751	0.13986	0.05782	0.17866	0.75128	0.75128 turquoise
BRD3	-0.04381	-0.18124	-0.1062	-0.00227	-0.112	0.01209	-0.02943	0.24281	-0.24281	0.5694	0.01767	0.16684	0.97649	0.14473	0.87527	0.70236	0.00138	0.00138 turquoise
PIMREG	-0.045	0.08456	-0.03831	-0.07154	-0.04539	-0.013	0.31445	-0.36779	0.36779	0.55897	0.2715	0.61887	0.35247	0.55555	0.86597	2.81E-05	7.47E-07	7.47E-07 pink
ADAMTS3	-0.11933	-0.11352	0.00676	0.0512	-0.20558	0.09779	0.0051	0.55589	-0.55589	0.12006	0.13931	0.93005	0.506	0.00699	0.20324	0.94721	2.96E-15	2.96E-15 blue
SLIT2	-0.06951	-0.07809	-0.02355	0.05569	-0.24444	0.10302	-0.02585	0.59087	-0.59087	0.36634	0.31001	0.75981	0.46937	0.00127	0.17995	0.73717	1.78E-17	1.78E-17 blue
DHX33	-0.09241	-0.09396	-0.05429	-0.01145	-0.08008	-0.04332	0.09163	0.22483	-0.22483	0.2293	0.22154	0.48069	0.88183	0.29782	0.57368	0.23327	0.00311	0.00311 turquoise
DCK	-0.14491	-0.10241	-0.03043	-0.00572	-0.03617	-0.03936	-0.10229	0.30776	-0.30776	0.05862	0.1826	0.69277	0.94077	0.63862	0.60926	0.1831	4.23E-05	4.23E-05 turquoise
ZNF146	-0.02141	-0.1387	0.0091	0.03942	-0.15787	0.12193	0.07527	0.03608	-0.03608	0.78105	0.07042	0.90598	0.60876	0.03919	0.11215	0.32787	0.63941	0.63941 turquoise
ATG4C	-0.13401	-0.04238	0.00642	0.03045	-0.10333	0.08648	-0.0422	0.46051	-0.46051	0.08057	0.58209	0.93362	0.69258	0.17865	0.26071	0.58369	2.33E-10	2.33E-10 blue
PIAS1	-0.03097	-0.17975	-0.0768	0.01344	-0.0165	0.01744	-0.02257	0.22312	-0.22312	0.68764	0.01865	0.31808	0.86145	0.83039	0.82088	0.76949	0.00335	0.00335 turquoise
CHCHD1	0.00787	0.02101	-0.03518	-0.04962	0.03502	-0.08801	0.12545	-0.39262	0.39262	0.91858	0.78507	0.64779	0.51925	0.64933	0.25237	0.10206	1.09E-07	1.09E-07 grey
THOC3	-0.04997	-0.00301	-0.0721	-0.07047	0.05643	-0.04831	0.11424	-0.35764	0.35764	0.51629	0.96878	0.34866	0.35975	0.46353	0.53031	0.13681	1.57E-06	1.57E-06 grey
MESP1	-0.0276	-0.13892	-0.28541	-0.09421	0.13057	-0.27579	-0.09603	0.03859	-0.03859	0.72008	0.06997	0.00015	0.22034	0.08871	0.00026	0.21149	0.61629	0.61629 grey
CRTAC1	0.00987	-0.09318	-0.07546	0.10577	-0.02597	-0.04922	-0.13414	0.26277	-0.26277	0.89805	0.22546	0.32665	0.16855	0.73597	0.52262	0.08026	0.00052	0.00052 grey
SV2B	-0.06231	-0.03786	-0.09635	0.04046	-0.14208	0.00013	-0.11131	0.41993	-0.41993	0.41817	0.62295	0.20999	0.59928	0.06378	0.99869	0.14722	1.08E-08	1.08E-08 magenta
GDPGP1	-0.019	-0.19261	-0.12432	-0.07423	0.22739	-0.08539	-0.0659	-0.26026	0.26026	0.80522	0.01161	0.10522	0.33461	0.00278	0.26681	0.39177	0.00059	0.00059 grey
MCOLN1	-0.04262	-0.10763	-0.16336	-0.08428	0.07198	-0.26033	-0.1221	0.35641	-0.35641	0.57993	0.16116	0.03277	0.27308	0.34949	0.00058	0.11164	1.71E-06	1.71E-06 brown
HSD17B4	-0.08013	-0.10252	-0.01426	0.01973	-0.00831	0.04216	-0.02124	0.16428	-0.16428	0.29746	0.18209	0.85317	0.7978	0.91413	0.58407	0.78276	0.03179	0.03179 turquoise
AK9	-0.02628	-0.18449	-0.10815	-0.13073	0.05883	-0.1402	-0.12743	0.25256	-0.25256	0.73297	0.01571	0.15914	0.08831	0.4447	0.06742	0.09673	0.00086	0.00086 turquoise
VPS36	-0.03268	-0.0376	-0.00424	-0.01302	0.01596	0.03053	-0.03707	0.11601	-0.11601	0.67133	0.62541	0.95616	0.86576	0.83589	0.69185	0.63025	0.13077	0.13077 turquoise
MEOX1	-0.09464	-0.01096	-0.09749	-0.03411	-0.08372	0.09577	-0.02719	0.40757	-0.40757	0.21824	0.88689	0.2046	0.65779	0.27632	0.21274	0.72405	3.15E-08	3.15E-08 blue
DUSP4	-0.0058	0.00982	0.03377	-0.04662	-0.05251	0.09249	0.15525	-0.25404	0.25404	0.93998	0.8986	0.66108	0.54482	0.4952	0.2289	0.0426	0.0008	0.0008 grey
ID4	-0.01107	-0.12716	-0.03623	0.04828	-0.04561	-0.09394	-0.20657	0.60979	-0.60979	0.88575	0.09744	0.63803	0.53062	0.55357	0.22164	0.00671	8.60E-19	8.60E-19 brown

CFAP410	-0.00379	-0.05391	-0.2117	-0.12317	0.35794	-0.26379	-0.12855	0.0229	-0.0229	0.96073	0.48376	0.00544	0.10851	1.54E-06	0.00049	0.09381	0.76621	0.76621	green
ARFGAP1	0.07456	-0.05931	-0.08806	-0.03821	0.23833	-0.1796	0.03013	-0.23076	0.23076	0.33247	0.44098	0.25207	0.61978	0.00169	0.01875	0.69563	0.00239	0.00239	green
WNT9A	-0.07614	-0.04505	-0.10224	0.06376	-0.08229	-0.00634	0.02602	0.15155	-0.15155	0.32228	0.5585	0.18331	0.4074	0.28459	0.93444	0.73551	0.04785	0.04785	grey
TBC1D12	-0.03819	-0.16049	0.02675	0.08734	-0.08259	0.06883	0.02749	0.26927	-0.26927	0.61999	0.036	0.72836	0.25601	0.28283	0.37107	0.72116	0.00037	0.00037	turquoise
VWA5B2	-0.02668	-0.11362	-0.30028	-0.1796	0.14268	-0.30237	-0.22505	0.38158	-0.38158	0.72905	0.13895	6.60E-05	0.01875	0.06265	5.83E-05	0.00308	2.61E-07	2.61E-07	brown
C11orf98	-0.04171	0.03263	-0.02962	0.00935	0.23864	-0.07505	0.07181	-0.30226	0.30226	0.58807	0.67176	0.70059	0.90339	0.00167	0.32927	0.35061	5.87E-05	5.87E-05	green
ZNF14	-0.06059	-0.11331	-0.07891	0.00028	0.04412	-0.04558	-0.25007	0.36805	-0.36805	0.43112	0.14006	0.30496	0.99712	0.56666	0.55385	0.00097	7.33E-07	7.33E-07	turquoise
KLC4	0.09209	0.06639	-0.01423	-0.06931	0.21133	0.05232	-0.02203	-0.22252	0.22252	0.23092	0.38827	0.85346	0.36768	0.00553	0.49674	0.77486	0.00344	0.00344	grey
PIGC	0.03294	-0.04487	-0.03328	-0.02607	-0.03053	0.10498	0.10507	-0.1577	0.1577	0.6689	0.56006	0.66564	0.73502	0.6918	0.17178	0.17142	0.0394	0.0394	turquoise
CCDC3	-0.12081	-0.05343	-0.04446	0.00458	-0.02872	0.002	-0.14432	0.48977	-0.48977	0.11549	0.48763	0.56365	0.95264	0.70919	0.97931	0.05966	1.06E-11	1.06E-11	blue
NPRL3	0.08315	0.00755	-0.05917	0.03469	0.04767	-0.12467	0.06782	-0.0763	0.0763	0.2796	0.9219	0.44203	0.65236	0.53583	0.10423	0.37815	0.32127	0.32127	green
SLC5A3	0.04021	-0.14183	0.08361	0.04467	-0.05289	0.11017	0.16845	-0.12814	0.12814	0.60152	0.06425	0.27696	0.56183	0.49205	0.15145	0.02764	0.09486	0.09486	turquoise
TMC4	0.19964	-0.00102	-0.0496	-0.05036	0.19247	0.05936	0.04642	-0.49788	0.49788	0.00885	0.9894	0.51941	0.513	0.01167	0.44056	0.5466	4.26E-12	4.26E-12	yellow
S100B	-0.06929	-0.16526	-0.1277	-0.07971	-0.04599	-0.16891	-0.09626	0.45845	-0.45845	0.36781	0.03077	0.09603	0.30002	0.55028	0.02721	0.21039	2.87E-10	2.87E-10	blue
TSPAN18	-0.11429	-0.01949	-0.01917	0.04597	-0.16356	0.03679	0.05246	0.36874	-0.36874	0.13661	0.8003	0.80351	0.55047	0.03255	0.63284	0.49559	6.96E-07	6.96E-07	blue
PTPMT1	-0.05094	-0.03866	-0.003	-0.05759	-0.06866	0.01248	0.0844	-0.23621	0.23621	0.50822	0.61569	0.96898	0.45438	0.37221	0.87129	0.27241	0.00187	0.00187	grey
LHFPL4	0.03352	-0.13596	-0.28628	-0.13944	0.13975	-0.31648	-0.16336	0.33957	-0.33957	0.66338	0.07621	0.00015	0.06892	0.06831	2.48E-05	0.03277	5.53E-06	5.53E-06	brown
SLC2A6	-0.18579	-0.10058	-0.08212	-0.04007	-0.03646	-0.16716	-0.1049	0.10907	-0.10907	0.01498	0.19056	0.28561	0.60283	0.63593	0.02887	0.17209	0.1556	0.1556	grey
VHL	-0.06426	-0.14321	-0.0635	-0.04555	-0.06895	0.08502	0.05434	0.13586	-0.13586	0.40368	0.06168	0.40933	0.55412	0.37021	0.26889	0.48024	0.07643	0.07643	turquoise
ZNF595	-0.15713	-0.13981	0.0109	0.08003	-0.12476	0.07115	-0.05523	0.20204	-0.20204	0.04013	0.06818	0.88753	0.29807	0.10398	0.35509	0.47304	0.00805	0.00805	turquoise
IRF4	-0.14517	0.01306	0.04114	0.02037	-0.11092	0.08323	0.02024	0.42188	-0.42188	0.05816	0.86535	0.59316	0.79141	0.14866	0.27916	0.79278	9.09E-09	9.09E-09	blue
PCDHGA4	0.06355	-0.17485	-0.01626	0.08388	-0.13335	0.06358	0.00987	0.24703	-0.24703	0.4089	0.02218	0.83285	0.2754	0.08208	0.40875	0.89801	0.00112	0.00112	turquoise
CLEC4A	-0.17548	-0.0837	0.08147	0.02556	-0.14839	0.13584	0.02103	0.40718	-0.40718	0.02169	0.2764	0.28948	0.73998	0.05275	0.07647	0.78489	3.26E-08	3.26E-08	blue
TMEM167	-0.0055	-0.13382	-0.05587	-0.03358	-0.00543	-0.08505	-0.06231	0.22242	-0.22242	0.94305	0.08099	0.46798	0.66284	0.94383	0.26871	0.41814	0.00346	0.00346	turquoise
SLAMF7	-0.03232	0.1049	0.08773	0.03595	-0.15596	0.16679	0.12478	0.22533	-0.22533	0.67473	0.1721	0.25384	0.64068	0.04165	0.02923	0.10393	0.00305	0.00305	blue
NAV2	0.07687	-0.09295	0.05013	0.0774	-0.17477	0.06733	0.09273	-0.03237	0.03237	0.31766	0.22659	0.51494	0.31431	0.02224	0.38157	0.22769	0.67426	0.67426	turquoise
ACBD5	0.04262	-0.06011	0.05499	-0.01301	0.05569	0.09681	0.03916	-0.11002	0.11002	0.57993	0.43481	0.475	0.86586	0.46936	0.20779	0.61112	0.15199	0.15199	turquoise
DGCR2	-0.02085	-0.0313	-0.14811	0.02825	-0.01715	-0.12291	0.0251	-0.0547	0.0547	0.78669	0.68444	0.0532	0.71374	0.82377	0.10925	0.74456	0.47734	0.47734	grey
RAI2	-0.01556	-0.16026	-0.18477	-0.03849	0.03143	-0.13015	-0.323	0.5384	-0.5384	0.83993	0.03627	0.01555	0.61717	0.68325	0.08977	1.64E-05	3.08E-14	3.08E-14	brown
ENO2	-0.0201	-0.20396	-0.11464	-0.04587	0.14619	-0.21933	-0.10742	-0.13723	0.13723	0.79413	0.00746	0.13544	0.55135	0.0564	0.00395	0.16198	0.07348	0.07348	grey
SYT5	-0.00924	-0.15858	-0.22427	-0.09942	0.16552	-0.35753	-0.24747	0.2679	-0.2679	0.90452	0.0383	0.00319	0.19576	0.0305	1.58E-06	0.0011	0.0004	0.0004	brown
SEPTIN7	-0.08099	-0.14644	0.03043	0.06556	-0.13203	0.06952	-0.0182	0.27317	-0.27317	0.2923	0.05598	0.69281	0.39427	0.08519	0.36627	0.81318	0.0003	0.0003	turquoise
SLC2A10	0.02592	-0.00629	0.12068	0.12412	-0.17055	0.28068	0.23207	-0.11268	0.11268	0.73647	0.93494	0.11588	0.1058	0.02573	0.0002	0.00226	0.14227	0.14227	turquoise
PHF12	-0.04369	-0.15773	-0.05813	-0.00931	0.02677	-0.0456	-0.07415	0.0701	-0.0701	0.57041	0.03936	0.45013	0.90378	0.72821	0.55367	0.33513	0.36224	0.36224	red
KIAA2013	0.02367	-0.05694	-0.00862	0.02523	0.01493	0.06208	0.00829	-0.14432	0.14432	0.75857	0.4595	0.91095	0.74321	0.84632	0.41988	0.91431	0.05966	0.05966	turquoise
ANKRD34.	0.07848	-0.08633	-0.02939	-0.10167	-0.05797	-0.02998	-0.00465	#######	9.49E-05	0.30756	0.26153	0.70273	0.18576	0.45138	0.69706	0.9519	0.99902	0.99902	grey
ALG11	-0.04185	-0.10934	0.02515	-0.02227	-0.12919	0.09285	0.06259	0.18699	-0.18699	0.58678	0.15456	0.74402	0.77249	0.09216	0.22708	0.41604	0.01433	0.01433	turquoise
AGPAT1	0.0239	-0.1115	-0.06919	-0.05266	0.00684	-0.06655	-0.0231	0.12139	-0.12139	0.75637	0.14655	0.36854	0.49392	0.9292	0.38714	0.76428	0.11375	0.11375	turquoise
TRPC6	-0.10925	-0.18411	-0.13033	0.02504	-0.0986	-0.04	-0.10714	0.4794	-0.4794	0.15491	0.01593	0.08932	0.7451	0.19946	0.60342	0.16308	3.27E-11	3.27E-11	blue
KIAA0753	-0.02537	-0.20706	-0.17965	-0.05901	0.09539	-0.12385	-0.00409	0.12155	-0.12155	0.7419	0.00658	0.01871	0.44327	0.21459	0.10656	0.95767	0.11326	0.11326	turquoise
ZNF135	-0.05062	-0.17289	-0.14065	-0.0386	-0.05775	-0.06428	-0.08769	0.56997	-0.56997	0.51085	0.02374	0.06653	0.61617	0.45308	0.40355	0.25409	4.08E-16	4.08E-16	blue
PXMP2	-0.01477	0.07462	0.00635	-0.03572	0.15288	0.07691	0.05636	-0.16208	0.16208	0.848	0.33205	0.93429	0.6428	0.04591	0.31742	0.46408	0.03418	0.03418	grey
SLC39A11	0.05759	0.02322	0.12037	0.09182	0.00454	0.09224	0.19835	-0.47152	0.47152	0.45431	0.7631	0.11684	0.23232	0.95304	0.2302	0.00931	7.54E-11	7.54E-11	yellow
FAM172A	-0.03918	-0.16445	-0.11121	-0.00885	-0.08241	-0.06529	-0.16883	0.45125	-0.45125	0.61092	0.03161	0.1476	0.90852	0.28392	0.39623	0.02729	5.86E-10	5.86E-10	turquoise
GLP1R	-0.16551	-0.00448	-0.15721	-0.03814	-0.01644	0.00069	-0.06859	0.3869	-0.3869	0.03051	0.95367	0.04002	0.62042	0.83104	0.99281	0.37269	1.72E-07	1.72E-07	brown
PTPRO	-0.08637	-0.01843	0.10963	0.10896	-0.24875	0.19531	0.10002	0.33632	-0.33632	0.26131	0.81092	0.15346	0.156	0.00104	0.01047	0.19303	6.88E-06	6.88E-06	blue
NOL3	0.04687	-0.0851	-0.09084	-0.08538	0.22071	-0.07248	0.04995	-0.45335	0.45335	0.54271	0.26845	0.23733	0.26685	0.00372	0.34613	0.51645	4.77E-10	4.77E-10	red
ALOX5AP	-0.08358	-0.05943	0.11681	0.09373	-0.22561	0.16744	0.04452	0.22797	-0.22797	0.27712	0.44001	0.12814	0.2227	0.00301	0.0286	0.56309	0.00271	0.00271	blue
HIST3H2B	0.02704	-0.14169	-0.00437	0.04669	-0.04132	-0.00133	0.10315	-0.33799	0.33799	0.72553	0.06452	0.95474	0.54423	0.59158	0.98619	0.17944	6.15E-06	6.15E-06	grey
HMGCL	-0.04119	-0.02119	0.00908	0.01704	-0.04789	0.17132	0.13856	-0.22348	0.22348	0.59275	0.78329	0.9062	0.82491	0.5339	0.02506	0.07071	0.0033	0.0033	turquoise
AL162231	-0.01028	-0.17272	-0.30321	-0.16723	0.05229	-0.3056	-0.05634	0.2102	-0.2102	0.89379	0.02388	5.55E-05	0.0288	0.49703	4.81E-05	0.4642	0.00579	0.00579	brown
TIAM2	-0.03321	-0.11185	-0.0102	0.04051	-0.13085	0.08895	0.06482	0.11383	-0.11383	0.66632	0.14527	0.89465	0.59889	0.08803	0.2473	0.39964	0.13824	0.13824	turquoise
CSF1	-0.0768	-0.11746	-0.00096	0.11286	-0.23471	0.16817	0.08481	0.33966	-0.33966	0.3181	0.126	0.99003	0.14162	0.002	0.0279	0.27005	5.50E-06	5.50E-06	blue
MFSD12	-0.01727	-0.01915	-0.1254	-0.00356	0.03694	-0.17694	0.01179	-0.10051	0.10051	0.82265	0.80371	0.10219	0.96312	0.63148	0.02061	0.87831	0.19087	0.19087	green
FLT1	-0.12029	-0.24553	-0.07895	0.04271	-0.10554	-0.06452	-0.17001	0.36961	-0.36961	0.11708	0.00121	0.30468	0.57911	0.16949	0.40184	0.02621	6.52E-07	6.52E-07	turquoise
SPAG16	0.03281	-0.08031	-0.1074	0.00249	0.10857	-0.03517	-0.02672	-0.13004	0.13004	0.67007	0.29642	0.16207	0.97425	0.15749	0.64794	0.72862	0.09004	0.09004	grey
RAI14	-0.12659	-0.15271	0.06296	0.0315	-0.25196	0.13205	0.12632	0.14979	-0.14979	0.09896	0.04614	0.41333	0.68253	0.00089	0.08514	0.09969	0.05053	0.05053	black
MYO1D	-0.03447	-0.03679	-0.01007	-0.07467	0.06844	0.12625	0.06469	0.0023	-0.0023	0.65445	0.63285	0.89603	0.33176	0.37377	0.09987	0.40059	0.97617	0.97617	turquoise
ADAMTSL	0.02137	0.05618	0.12885	0.0671	0.07988	0.19773	0.2271	-0.51443	0.51443	0.78147	0.46548	0.09304	0.38324	0.29899	0.00953	0.00282	6.14E-13	6.14E-13	yellow
LCMT2	-0.23195	-0.14122	-0.23411	-0.06195	-0.03058	-0.16375	-0.11522	0.34709	-0.34709	0.00227	0.06541	0.00206	0.42084	0.69138	0.03235	0.13345	3.30E-06	3.30E-06	turquoise
MSL1	0.00388	-0.17223	-0.05259	-0.004	0.11174	-0.01134	0.00845	-0.03251	0.03251	0.95986	0.02429	0.49449	0.95857	0.14567	0.88299	0.91261	0.67297	0.67297	turquoise
SHROOM:	-0.02789	-0.04388	0.03789	0.02897	0.02984	0.17132	0.07684	-0.2233	0.2233	0.71729	0.5688	0.62271	0.7068	0.69844	0.02506	0.31784	0.00333	0.00333	turquoise
F11	-0.0048	-0.04102	-0.11109	0.06778	0.01763	0.04905	-0.02159	0.19796	-0.19796	0.95034	0.59422	0.14805	0.37842	0.81902	0.52406	0.77927	0.00945	0.00945	magenta
SOCS7	-0.06173	-0.18973	-0.21583	-0.03318	0.04359	-0.27114	-0.16322	0.42428	-0.42428	0.42254	0.01294	0.00458	0.66662	0.57135	0.00033	0.03292	7.34E-09	7.34E-09	turquoise
HK1	-0.02619	-0.05851	0.09485	0.06475	-0.18494	0.24057	0.25012	-0.25347	0.25347	0.73386	0.44714	0.21722	0.40011	0.01545	0.00153	0.00097	0.00082	0.00082	turquoise
CDC42SE2	-0.04705	-0.13061	-0.0455	0.00696	-0.01806	-0.00504	-0.15779	0.20096	-0.20096	0.54118	0.08862	0.55454	0.92802	0.81463	0.94779	0.03928	0.0084	0.0084	turquoise
ZCCHC18	-0.03648	-0.14934	-0.19877	-0.11115	0.02766	-0.26793	-0.23495	0.52747	-0.52747	0.63575	0.05124	0.00915	0.14782	0.71954	0.0004	0.00198	1.24E-13	1.24E-13	brown
VARS2	-0.01928	0.05377	-0.09148	-0.05281	0.08487	-0.10038	0.01465	-0.14045	0.14045	0.80232	0.48485	0.23406	0.49275	0.26975	0.19143	0.84921	0.06692	0.06692	grey
MCTP1	-0.05704	-0.05781	-0.01562	0.02078	-0.1341	0.06505	-0.00194	0.42984	-0.42984	0.45868	0.45265	0.83933	0.78738	0.08035	0.39793	0.9799	4.44E-09	4.44E-09	blue
CCDC61	0.10702	0.06821	-0.0321	-0.12296	0.15901	-0.01709	0.1597	-0.29113	0.29113	0.16354	0.37537	0.67682	0.10911	0.03778	0.82447	0.03694	0.00011	0.00011	green
DPCD	0.05454	-0.06449	-0.12798	-0.05871	0.14414	-0.18882	0.12676	-0.34019	0.34019	0.47866	0.40204	0.0953	0.44562	0.05999	0.01338	0.09852	5.30E-06	5.30E-06	grey
ZNF100	-0.06959	-0.05887	-0.02035	0.06274	0.00875	-0.03339	-0.03546	0.32005	-0.32005	0.36577	0.44439	0.79165	0.41491	0.90953	0.66465	0.6452	1.98E-05	1.98E-05	turquoise
ATP1A1	0.0107	-0.13371	-0.01332	0.09069	-0.0648	0.20881	0.03189	-0.16215	0.16215	0.88951	0.08125	0.86268	0.23811	0.39977	0.00613	0.67882	0.0341	0.0341	grey
DHRS4L2	0.0511	0.05507	-0.11367	0.0027	0.17578	-0.19629	-0.07955	-0.15973	0.15973	0.50682	0.47437	0.1388	0.97205	0.02146	0.01008	0.30101	0.0369	0.0369	grey
LYRM7	-0.01193	-0.05782	-0.12104	-0.0768	0.01735	-0.1647	-0.15753	0.39811	-0.39811	0.87695	0.45253	0.1148	0.31809	0.82181	0.03134	0.03962	6.95E-08	6.95E-08	turquoise
RHBDF2	-0.05822	-0.06082	0.13842	0.07714	-0.14316	0.13342	0.19049	-0.24819	0.24819	0.44943	0.42939	0.071	0.31597	0.06177	0.08191	0.01257	0.00106	0.00106	grey
ACADSB	-0.04805	-0.03174	-0.07643	-0.00463	0.06981	-0.11697	-0.15668	0.34316	-0.34316	0.53258	0.68028	0.32044	0.95212	0.36425	0.12761	0.04071	4.33E-06	4.33E-06	turquoise
HLA-A	-0.02763	0.12672	-0.00586	-0.00895	0.02543	-0.05501	0.09656	-0.14183	0.14183	0.71978	0.09862	0.9394	0.90754	0.74127	0.47485	0.20897	0.06424	0.06424	tan
FASTK	0.00521	-0.03524	-0.1118	-0.10088	0.2234	-0.21738	0.05821	-0.27365	0.27365	0.94608	0.64721	0.14544	0.18922	0.00331	0.00429	0.4495	0.00029	0.00029	green
HTRA3	-0.05314	0.02112	0.00833	0.04356	-0.13284	0.18898	0.01359	0.0638	-0.0638	0.49	0.78397	0.91384	0.57161	0.08327	0.01331	0.85994	0.4071	0.4071	black
MFSD9	0.05239	-0.08416	0.11677	0.06168	-0.08593	0.21695	0.18466	-0.24575	0.24575	0.49621	0.27375	0.12825	0.42287	0.26376	0.00437	0.01561	0.0012	0.0012	turquoise
DARS2	-0.03617	0.03731	0.01382	-0.03071	-0.06753	0.14557	0.23474	-0.21204	0.21204	0.63858	0.62806	0.85765	0.69009	0.38017	0.05747	0.002	0.00537	0.00537	turquoise
ESCO2	-0.1192	0.09273	0.06508	-0.017	-0.02533	0.14158	0.33625	-0.24831	0.24831	0.12044	0.22769	0.39776	0.82533	0.74228	0.06472	6.91E-06	0.00106	0.00106	pink
ATF7IP2	0.01248	0.06506	0.1356	0.06001	-0.05271	0.27413	0.12214	-0.19345	0.19345	0.87126	0.39789	0.077	0.43558	0.49353	0.00029	0.1115	0.01124	0.01124	grey
SLC27A4	0.03278	-0.0577	-0.06551	0.00829	0.00484	-0.08274	0.08048	-0.1822	0.1822	0.67038	0.45345	0.39463	0.91432	0.94992	0.28198	0.29541	0.01707	0.01707	grey
BBOF1	-0.02792	-0.21005	-0.1198	0.02794	-0.04642	-0.05522	-0.04461	0.31411	-0.31411	0.717	0.00583	0.11857	0.71678	0.54655	0.47319	0.56239	2.87E-05	2.87E-05	turquoise
VPS9D1	0.01325	-0.05602	-0.07378	-0.06397	0.14772	-0.09675	0.00981	-0.32701	0.32701	0.86344	0.46676	0.33757	0.40586	0.05384	0.20809	0.89871	1.27E-05	1.27E-05	red
C5orf24	-0.08119	-0.18813	-0.07322	0.01526	-0.04691	-0.02713	-0.04294	0.2194	-0.2194	0.29112	0.01373	0.34125	0.84292	0.54231	0.72463	0.57712	0.00394	0.00394	turquoise
RHEBL1	-0.02354	-0.11499	-0.0135	0.03112	-0.15526	-0.01042	0.13368	-0.15768	0.15768	0.75991	0.13424	0.86089	0.68617	0.04259	0.89236	0.08132	0.03943	0.03943	grey
TMEM179	-0.01751	-0.05855	0.04812	0.02334	-0.01531	0.10363	0.117	-0.40873	0.40873	0.82019	0.44684	0.53201	0.76192	0.84251	0.17739	0.1275	2.86E-08	2.86E-08	grey
TAF8	-0.00858	-0.10434	-0.05284	0.00539	-0.07739	0.02587	0.12011	0.04531	-0.04531	0.91132	0.17444	0.49248	0.94425	0.31439	0.73697	0.11763	0.55623	0.55623	turquoise
ZNF174	0.03131	-0.03778	-0.14104	-0.04127	-0.00291	-0.06859	-0.00179	0.16413	-0.16413	0.68434	0.62374	0.06577	0.59201	0.9699	0.37273	0.98151	0.03195	0.03195	turquoise
RTCA	-0.02766	-0.11255	0.01605	-0.04604	-0.02482	0.0056	0.01275	-0.01969	0.01969	0.71949	0.14276	0.83497	0.54987	0.74724	0.94207	0.8685	0.79829	0.79829	turquoise
KIAA1217	-0.04077	-0.09071	0.1017	0.09012	-0.22255	0.28246	0.23082	0.02619	-0.02619	0.59653	0.23804	0.18563	0.2411	0.00344	0.00018	0.00239	0.73388	0.73388	black
CCDC187	-0.0123	-0.13048	-0.18968	-0.06365	0.18707	-0.29232	-0.07129	0.09615	-0.09615	0.87316	0.08893	0.01296	0.40816	0.01428	0.0001	0.35412	0.21092	0.21092	grey
SLC10A3	0.0335	-0.11683	0.07302	0.03856	-0.09847	0.24367	0.1843	-0.25736	0.25736	0.66356	0.12807	0.34259	0.6166	0.20009	0.00132	0.01582	0.00068	0.00068	turquoise
PHOSPHC	0.12026	0.01382	-0.05613	-0.01199	0.09716	0.00533	0.02121	-0.04489	0.04489	0.11719	0.85765	0.46588	0.87626	0.20616	0.94485	0.78309	0.55987	0.55987	grey
ARMC10	0.07833	-0.06005	0.09434	0.05171	-0.10361	0.19491	0.2114	-0.28726	0.28726	0.30853	0.43526	0.2197	0.50181	0.17746	0.01063	0.00551	0.00014	0.00014	turquoise
WDCP	-0.0752	-0.1337	-0.06708	0.00784	-0.15172	0.07547	0.12005	0.1417	-0.1417	0.32833	0.08126	0.38336	0.91889	0.0476	0.32653	0.11783	0.0645	0.0645	turquoise
DRAP1	0.02607	-0.03876	-0.04388	-0.03595	-0.06269	-0.08603	0.12471	-0.19455	0.19455	0.735	0.61478	0.56878	0.64068	0.41534	0.26322	0.10413	0.01078	0.01078	grey
COL26A1	-0.01122	-0.10625	-0.12939	-0.04552	0.05734	-0.26735	-0.20236	0.36651	-0.36651	0.88418	0.16662	0.09167	0.55441	0.45634	0.00041	0.00795	8.21E-07	8.21E-07	brown
SEPTIN8	-0.05152	-0.16517	-0.0328	0.03495	-0.1031	0.08263	0.02866	0.20043	-0.20043	0.50337	0.03086	0.67017	0.64998	0.17962	0.28263	0.7098	0.00858	0.00858	turquoise
ZC3H13	-0.06295	-0.11154	-0.04713	-0.00914	-0.12049	0.07364	-0.03147	0.27946	-0.27946	0.41341	0.1464	0.54047	0.90555	0.11648	0.33848	0.68285	0.00021	0.00021	turquoise
LRFN4	-0.00057	0.01697	-0.06256	-0.07114	0.06808	0.00248	0.20636	-0.45555	0.45555	0.99408	0.82568	0.41627	0.35514	0.37627	0.9743	0.00677	3.83E-10	3.83E-10	green
SLK	0.06754	-0.04297	0.10506	0.0263	-0.11439	0.1711	0.21657	-0.22759	0.22759	0.38012	0.57685	0.17145	0.73281	0.13629	0.02525	0.00444	0.00276	0.00276	turquoise
PTAFR	-0.09526	-0.05526	0.12919	0.07505	-0.15691	0.25115	0.11849	0.08265	-0.08265	0.21521	0.47281	0.09217	0.32929	0.04042	0.00092	0.12271	0.2825	0.2825	blue
C1orf115	-0.03206	0.00446	-0.0426	0.01805	0.09934	0.1298	0.01022	-0.08821	0.08821	0.67725	0.95386	0.58013	0.81476	0.1961	0.09064	0.89442	0.25125	0.25125	grey
TMCC2	-0.04725	-0.14593	-0.27368	-0.09351	0.09135	-0.33874	-0.22314	0.52002	-0.52002	0.53943	0.05685	0.00029	0.2238	0.23475	5.85E-06	0.00335	3.12E-13	3.12E-13	brown
DNMT1	-0.07082	-0.03463	-0.02465	-0.04039	-0.00547	-0.00184	0.05547	0.1476	-0.1476	0.35731	0.65292	0.74891	0.59992	0.9434	0.98095	0.47119	0.05404	0.05404	turquoise
GOLGA2	-0.01037	-0.18952	-0.08624	-0.03673	-0.05348	-0.0692	-0.01485	0.11319	-0.11319	0.89293	0.01304	0.26206	0.63338	0.48725	0.3685	0.84718	0.14047	0.14047	turquoise
ZNF652	0.00481	-0.18786	-0.16374	-0.02273	0.0497	-0.10938	-0.2076	0.19597	-0.19597	0.95017	0.01388	0.03236	0.76794	0.51861	0.15439	0.00644	0.0102	0.0102	turquoise
SUPT16H	-0.03171	-0.07959	0.01388	0.03776	-0.16293	0.0634	0.07811	0.04819	-0.04819	0.68057	0.30079	0.85696	0.62389	0.03324	0.41008	0.30986	0.53135	0.53135	turquoise
NRP2	-0.01205	-0.0504	0.0432	0.08499	-0.21978	0.11814	0.18763	0.03412	-0.03412	0.87571	0.51267	0.57479	0.26904	0.00387	0.12383	0.01399	0.65773	0.65773	black
DRC3	-0.00598	-0.15133	-0.26739	-0.08597	0.17663	-0.2215	-0.19062	0.31132	-0.31132	0.93817	0.04817	0.00041	0.26354	0.02083	0.0036	0.01251	3.40E-05	3.40E-05	brown
TRIM26	-0.00312	0.00663	-0.03702	0.0076	0.03205	0.06605	0.04617	-0.0842	0.0842	0.96774	0.93142	0.63068	0.92144	0.67729	0.3907	0.54877	0.27357	0.27357	turquoise
LGALS7B	-0.03	0.01682	-0.00431	0.0234	0.13382	0.09005	0.05523	-0.19858	0.19858	0.69693	0.82717	0.95535	0.76126	0.081	0.24147	0.47305	0.00922	0.00922	grey
CDC42EP:	-0.02808	-0.2079	-0.00527	0.03049	-0.17892	-0.02326	-0.07017	0.29402	-0.29402	0.71541	0.00636	0.94546	0.69221	0.01921	0.76271	0.3618	9.49E-05	9.49E-05	turquoise
GJA5	-0.21114	-0.02943	-0.06947	-0.02386	-0.08443	0.01286	-0.05864	0.47817	-0.47817	0.00557	0.70235	0.36659	0.75677	0.27222	0.86739	0.44613	3.74E-11	3.74E-11	blue
PCSK4	0.0494	-0.01363	-0.06757	-0.04176	0.26859	-0.09892	0.05565	-0.35249	0.35249	0.52111	0.85955	0.37991	0.58756	0.00038	0.19803	0.46971	2.26E-06	2.26E-06	green
LRRN1	-0.04399	-0.03458	0.00623	0.04489	-0.16163	0.14421	0.13543	-0.05497	0.05497	0.56782	0.6534	0.93559	0.55986	0.03468	0.05986	0.07737	0.47521	0.47521	grey
BEND3	-0.00792	-0.02684	-0.01736	-0.06078	0.05047	-0.02257	0.00737	0.0347	-0.0347	0.91808	0.72748	0.82171	0.4297	0.51213	0.76954	0.92376	0.65234	0.65234	grey
DMAC2L	-0.04821	-0.08037	-0.11195	-0.05632	0.12479	-0.10933	-0.12342	0.19262	-0.19262	0.5312	0.29602	0.1449	0.46437	0.10391	0.1546	0.10779	0.0116	0.0116	turquoise
XIRP1	-0.02537	-0.20097	0.10066	0.18264	-0.12839	0.12375	0.08505	0.24213	-0.24213	0.74182	0.0084	0.19018	0.01681	0.09423	0.10683	0.26874	0.00142	0.00142	blue
BRAP	0.0862	-0.13181	-0.06224	-0.03842	0.04321	-0.12034	-0.08504	0.12229	-0.12229	0.26228	0.08571	0.41867	0.61788	0.57473	0.11694	0.26875	0.11106	0.11106	turquoise
MSH5	-0.03272	0.01686	-0.00742	-0.02557	0.20548	0.03399	0.05883	-0.24636	0.24636	0.67098	0.82671	0.92325	0.73992	0.00701	0.65897	0.44465	0.00116	0.00116	red
ALDH5A1	-0.03081	-0.0608	-0.14235	-0.04504	0.21065	-0.16306	-0.18203	0.2643	-0.2643	0.68911	0.42959	0.06327	0.55859	0.00568	0.03309	0.01718	0.00048	0.00048	turquoise
HEYL	-0.05673	-0.0853	-0.04565	0.03271	-0.22072	0.09795	-0.06238	0.44791	-0.44791	0.46113	0.26731	0.55322	0.67102	0.00372	0.20249	0.41765	8.11E-10	8.11E-10	blue
SCFD1	0.0077	-0.06862	0.01177	0.02704	0.01938	-0.08516	-0.04146	0.09142	-0.09142	0.9204	0.37251	0.87853	0.72551	0.8014	0.26812	0.59026	0.23435	0.23435	turquoise
ADAM9	-0.0397	-0.10556	0.09151	0.09014	-0.10079	0.19465	0.20027	-0.13001	0.13001	0.60613	0.16942	0.2339	0.24102	0.18961	0.01074	0.00863	0.09011	0.09011	turquoise

VCPKMT	0.00647	-0.09493	-0.02121	0.02461	0.08464	-0.02482	-0.07647	-0.08584	0.08584	0.93304	0.21681	0.783	0.7493	0.27101	0.74725	0.32015	0.26429	0.26429 red
CCND3	0.12036	-0.13066	-0.0404	-0.02349	-0.06423	-0.10785	-0.03316	0.01353	-0.01353	0.11687	0.08849	0.59984	0.76042	0.40394	0.16031	0.6668	0.86057	0.86057 grey
ARHGAP1	-0.06087	-0.0992	-0.09805	-0.09226	-0.15708	-0.07532	0.11051	0.27387	-0.27387	0.42902	0.19673	0.20202	0.23008	0.0402	0.32751	0.15017	0.00029	0.00029 turquoise
FECH	-0.14979	-0.08416	-0.08682	0.05548	-0.07458	-0.05062	-0.04137	0.50448	-0.50448	0.05054	0.27376	0.25886	0.4711	0.33229	0.51087	0.59112	1.99E-12	1.99E-12 turquoise
SGCD	-0.03434	-0.03438	-0.04407	0.01053	-0.16048	0.05	0.0075	0.48972	-0.48972	0.65568	0.65528	0.56711	0.89125	0.03601	0.51608	0.92242	1.06E-11	1.06E-11 blue
RGS13	-0.05357	-0.00217	0.02566	0.06441	0.05344	0.21255	0.01768	0.23769	-0.23769	0.48648	0.97758	0.73902	0.40263	0.48754	0.00525	0.81842	0.00175	0.00175 blue
CBX4	0.08054	-0.08614	-0.1004	-0.02145	0.16567	-0.0848	0.01366	-0.25827	0.25827	0.29501	0.26263	0.19135	0.78067	0.03035	0.27014	0.85925	0.00065	0.00065 green
FRMD5	0.01293	-0.00414	0.04426	0.01869	-0.13642	-0.00896	0.08324	-0.3399	0.3399	0.86672	0.95718	0.56538	0.80827	0.07522	0.90745	0.27909	5.41E-06	5.41E-06 grey
SOS2	-0.05503	-0.21199	-0.07823	0.0223	-0.04269	-0.00094	-0.09489	0.2934	-0.2934	0.47469	0.00538	0.30915	0.77221	0.57928	0.99027	0.21703	9.84E-05	9.84E-05 turquoise
SECTM1	0.0203	-0.01166	-0.00109	0.06105	-0.1155	0.05195	0.29515	-0.10358	0.10358	0.79219	0.8797	0.98872	0.42768	0.13249	0.49978	8.89E-05	0.17759	0.17759 grey
MAD1L1	0.05067	0.02939	-0.02823	-0.04208	0.037	-0.01309	0.07165	-0.22367	0.22367	0.51043	0.70274	0.71399	0.58477	0.63092	0.86507	0.35169	0.00327	0.00327 grey
NDUFA5	-0.01136	-0.08532	-0.1057	-0.07177	0.1674	-0.20622	-0.21354	0.118	-0.118	0.88279	0.26722	0.16885	0.35091	0.02864	0.00681	0.00504	0.12428	0.12428 turquoise
SEC61B	0.00201	0.03617	-0.1609	-0.0195	0.06412	-0.25318	-0.13465	-0.03931	0.03931	0.97919	0.63857	0.03552	0.80018	0.40473	0.00083	0.07911	0.60973	0.60973 grey
BZW2	0.0541	0.07454	0.06254	0.05159	-0.06374	0.20467	0.27723	-0.40524	0.40524	0.48221	0.33258	0.41647	0.5028	0.40752	0.00725	0.00024	3.84E-08	3.84E-08 pink
S100A8	-0.16865	0.01305	0.10323	0.04314	-0.15426	0.01549	0.07183	0.19778	-0.19778	0.02745	0.8655	0.17908	0.57531	0.04396	0.84061	0.35049	0.00952	0.00952 blue
NDUFB2	0.08354	-0.00946	-0.09751	-0.14356	0.20183	-0.27886	-0.0512	-0.11004	0.11004	0.27733	0.90227	0.20454	0.06104	0.00812	0.00022	0.50599	0.15194	0.15194 grey
NT5C3A	0.06086	-0.02373	0.08588	0.027	0.1218	-0.01967	-0.06971	-0.27394	0.27394	0.42908	0.75803	0.26404	0.72594	0.11252	0.79847	0.36491	0.00029	0.00029 grey
CKAP2	-0.02576	0.01629	-0.01313	-0.03548	-0.07374	-0.08314	0.11255	-0.07195	0.07195	0.73807	0.83252	0.86471	0.64502	0.3378	0.27967	0.14275	0.34969	0.34969 turquoise
SLC44A2	0.04826	-0.00854	0.03346	0.05803	-0.13419	0.26196	0.22511	-0.17867	0.17867	0.53074	0.91169	0.664	0.4509	0.08015	0.00054	0.00307	0.01938	0.01938 yellow
FANCM	-0.10583	-0.02917	-0.02137	-0.00549	-0.12963	0.04965	0.0758	0.19996	-0.19996	0.16832	0.7049	0.78146	0.94314	0.09106	0.51902	0.32447	0.00874	0.00874 turquoise
INPP4B	-0.02704	-0.07932	0.09	0.10394	-0.151	0.18312	0.19307	-0.25261	0.25261	0.72551	0.30245	0.24177	0.17608	0.04868	0.01651	0.0114	0.00086	0.00086 turquoise
NOP53	0.13877	0.13878	-0.08644	-0.05536	0.13179	0.01358	0.03549	-0.1835	0.1835	0.07027	0.07025	0.26094	0.47206	0.08574	0.86002	0.64491	0.01629	0.01629 green
CDCA8	-0.00998	0.05245	0.07149	-0.0181	-0.0117	0.10447	0.24234	-0.44455	0.44455	0.89696	0.49564	0.35277	0.81428	0.87932	0.1739	0.00141	1.12E-09	1.12E-09 pink
FADD	0.02524	-0.01953	-0.00685	0.04537	-0.08039	0.10579	0.2252	-0.27489	0.27489	0.74318	0.79982	0.92913	0.5557	0.29594	0.16848	0.00306	0.00027	0.00027 turquoise
GOLPH3L	0.0842	-0.04416	-0.03689	-0.03018	0.0273	0.12371	0.09463	-0.23395	0.23395	0.27352	0.56629	0.63194	0.69518	0.723	0.10696	0.21829	0.00207	0.00207 turquoise
CPSF3	-0.03706	0.01774	-0.02242	-0.03176	-0.12595	0.06394	0.26734	-0.21703	0.21703	0.63035	0.81782	0.77102	0.68008	0.10069	0.40605	0.00041	0.00435	0.00435 turquoise
PARD3	0.11742	-0.06234	0.04838	0.03817	-0.12989	0.13301	0.17156	-0.20133	0.20133	0.12613	0.41795	0.52978	0.62013	0.09042	0.08287	0.02486	0.00828	0.00828 turquoise
FAM76A	-0.03156	-0.06259	-0.02881	#######	-0.05734	0.11233	-0.03898	0.12914	-0.12914	0.682	0.41609	0.70836	0.99939	0.45629	0.14355	0.61273	0.0923	0.0923 turquoise
MRPL37	-0.03013	0.06945	0.07301	-0.00493	-0.11806	0.16453	0.29546	-0.30684	0.30684	0.69569	0.36676	0.34265	0.94896	0.12407	0.03152	8.73E-05	4.47E-05	4.47E-05 pink
PPP3R1	0.03211	-0.11397	0.00347	0.00248	-0.10621	0.08296	0.04424	-0.0543	0.0543	0.67672	0.13774	0.96405	0.97435	0.16678	0.28069	0.56556	0.4806	0.4806 turquoise
SRGAP2C	-0.107	-0.13291	0.06611	0.10489	-0.2452	0.15136	0.07178	0.23867	-0.23867	0.16362	0.0831	0.39027	0.17216	0.00123	0.04813	0.35081	0.00167	0.00167 blue
RBMX2	0.00375	-0.11585	-0.04735	-0.07126	-0.04097	0.00197	0.06649	-0.05683	0.05683	0.96118	0.13131	0.53853	0.35436	0.5947	0.9796	0.38755	0.46033	0.46033 turquoise
WDR45B	-0.08942	-0.2311	-0.00251	0.0445	0.1571	-0.04786	-0.01037	-0.24341	0.24341	0.24482	0.00236	0.97404	0.56333	0.04017	0.53415	0.89293	0.00134	0.00134 grey
GTF2H1	-0.01235	-0.10275	0.05334	0.03149	-0.17726	0.17233	0.13634	0.08151	-0.08151	0.87262	0.1811	0.48834	0.68265	0.02038	0.02421	0.07538	0.28924	0.28924 turquoise
EZH1	0.01619	-0.16542	-0.14449	-0.05822	0.00938	-0.15147	-0.13872	0.26768	-0.26768	0.83353	0.0306	0.05936	0.44944	0.90309	0.04796	0.07038	0.0004	0.0004 turquoise
OCEL1	0.13776	-0.01889	0.01066	-0.02587	0.12912	0.07211	0.09411	-0.4667	0.4667	0.07236	0.80632	0.88989	0.73693	0.09235	0.34861	0.2208	1.24E-10	1.24E-10 grey
MOCS3	-0.10824	-0.07468	-0.08187	0.03146	0.01887	-0.1227	0.07429	-0.04679	0.04679	0.15879	0.33165	0.28708	0.68291	0.80647	0.10987	0.33423	0.54338	0.54338 turquoise
GPC6	-0.07661	-0.16825	0.01803	0.05804	-0.07928	-0.11836	-0.05412	0.43427	-0.43427	0.31931	0.02783	0.81489	0.45085	0.30264	0.12312	0.48204	2.95E-09	2.95E-09 blue
CD24	-0.01659	0.11034	-0.03336	0.01883	0.02551	0.15809	0.06637	-0.12537	0.12537	0.82944	0.15081	0.66493	0.80691	0.74051	0.03891	0.38841	0.10229	0.10229 grey
TBCEL	-0.12744	-0.16424	-0.1082	0.0072	-0.17789	-0.06293	-0.04258	0.49258	-0.49258	0.0967	0.03182	0.15893	0.92558	0.01992	0.41357	0.58032	7.74E-12	7.74E-12 blue
BMP2	0.01199	-0.05181	-0.03444	0.05682	0.02709	0.14632	-0.06666	-0.11743	0.11743	0.87631	0.50094	0.65472	0.46041	0.72506	0.05619	0.38638	0.12612	0.12612 grey
MOGAT3	0.09168	0.11343	0.13525	0.08935	0.17798	0.16398	0.11507	-0.3454	0.3454	0.23305	0.13963	0.07777	0.24518	0.01986	0.03211	0.13394	3.71E-06	3.71E-06 grey
GPHN	0.02766	0.01518	-0.07087	-0.04092	0.09536	-0.04397	-0.04043	0.05001	-0.05001	0.71947	0.84381	0.35697	0.5951	0.21472	0.56796	0.59957	0.516	0.516 turquoise
WTAP	-0.06762	-0.10614	0.02766	0.05094	-0.10507	0.05278	-0.05122	0.25031	-0.25031	0.37954	0.16708	0.71952	0.5082	0.17141	0.49294	0.50589	0.00096	0.00096 turquoise
DNAAF2	-0.04017	-0.09334	-0.05725	0.00721	0.04799	0.00519	-0.08855	0.11693	-0.11693	0.6019	0.22465	0.45703	0.92543	0.53311	0.94633	0.24945	0.12773	0.12773 turquoise
MPP5	0.06572	-0.12335	0.02249	0.06608	-0.05189	0.09948	0.10951	-0.10736	0.10736	0.39307	0.10799	0.77031	0.39047	0.50027	0.19546	0.15392	0.16222	0.16222 turquoise
BLOC1S3	0.06249	-0.07573	-0.07141	-0.03837	-0.09179	0.01593	0.09173	-0.15543	0.15543	0.41685	0.32493	0.35333	0.61831	0.23247	0.8362	0.23277	0.04236	0.04236 grey
C17orf53	-0.0348	0.05273	0.00634	-0.02006	0.01818	0.00473	0.30152	-0.42856	0.42856	0.65134	0.49334	0.93445	0.79452	0.81338	0.95109	6.13E-05	4.98E-09	4.98E-09 pink
CCR2	-0.13209	-0.05976	0.03713	0.01907	-0.18228	0.14411	0.00708	0.48256	-0.48256	0.08504	0.43751	0.62975	0.8045	0.01703	0.06005	0.92676	2.33E-11	2.33E-11 blue
SMIM15	-0.03155	-0.062	-0.00155	-0.0172	0.08673	0.07198	-0.04304	-0.17059	0.17059	0.68203	0.42046	0.98396	0.82327	0.25936	0.34949	0.57616	0.0257	0.0257 turquoise
NPAS2	0.16651	-0.01981	0.05842	0.04917	0.02818	0.09432	0.25044	-0.3293	0.3293	0.0295	0.79708	0.44787	0.52301	0.71445	0.21979	0.00095	1.09E-05	1.09E-05 yellow
PAF1	0.11398	-0.31411	-0.10938	0.04459	-0.08908	-0.07187	0.00376	-0.05994	0.05994	0.13772	2.87E-05	0.15442	0.56256	0.2466	0.35023	0.96109	0.43612	0.43612 grey
NEIL2	0.03755	-0.05758	-0.11794	-0.00141	0.01073	-0.22193	0.03421	0.1909	-0.1909	0.6258	0.45439	0.12446	0.98543	0.88926	0.00353	0.65687	0.01238	0.01238 grey
CXCL1	-0.14314	-0.06285	0.00758	-0.00925	-0.131	0.13117	0.14527	-0.00123	0.00123	0.06181	0.41416	0.92163	0.90439	0.08768	0.08724	0.05799	0.98731	0.98731 grey
ZBTB26	-0.03429	-0.19216	-0.14465	-0.0779	-0.02677	-0.06047	-0.02133	0.16536	-0.16536	0.65619	0.01181	0.05909	0.31117	0.72814	0.43208	0.78182	0.03066	0.03066 turquoise
HSPA12B	-0.09341	-0.02685	-0.026	0.10067	-0.0918	-0.01702	0.03017	0.52757	-0.52757	0.22428	0.72742	0.73567	0.19017	0.2324	0.82511	0.69531	1.23E-13	1.23E-13 blue
REG1A	-0.03072	0.06365	0.09194	0.14999	-0.09236	0.18462	0.05124	-0.02001	0.02001	0.69002	0.40823	0.23172	0.05022	0.22955	0.01563	0.50573	0.79504	0.79504 magenta
TMCC3	-0.00705	-0.07669	0.02525	0.04563	0.00781	-0.05223	-0.09071	0.23494	-0.23494	0.92713	0.31876	0.74307	0.55346	0.91924	0.49752	0.23804	0.00198	0.00198 turquoise
TSHZ1	-0.02873	-0.12363	-0.15357	0.00679	0.03066	-0.06668	-0.16172	0.4158	-0.4158	0.70916	0.10718	0.04493	0.92974	0.69058	0.38619	0.03458	1.55E-08	1.55E-08 turquoise
DERL1	-0.01544	-0.09238	-0.02393	0.03679	-0.11663	-0.02223	0.07232	-0.05469	0.05469	0.84114	0.22944	0.75605	0.63282	0.12871	0.77291	0.34722	0.4774	0.4774 turquoise
GINM1	-0.0839	-0.14834	0.00635	0.08732	-0.16208	0.00528	-0.01221	0.34888	-0.34888	0.27527	0.05282	0.93435	0.25613	0.03418	0.94531	0.8741	2.92E-06	2.92E-06 turquoise
SNRNP48	0.05491	-0.1411	-0.07466	-0.01346	0.09131	0.00263	-0.01655	0.08119	-0.08119	0.47566	0.06566	0.33181	0.86126	0.23495	0.97278	0.8299	0.29112	0.29112 turquoise
MAB21L2	0.01507	0.01661	0.10767	0.09335	-0.10177	0.10303	0.06311	0.17286	-0.17286	0.84488	0.82932	0.16102	0.22459	0.18535	0.17992	0.41221	0.02377	0.02377 salmon
YPEL1	-0.14289	-0.15663	-0.08947	0.11846	-0.20294	0.02384	-0.04664	0.39258	-0.39258	0.06226	0.04077	0.24453	0.12279	0.00777	0.75698	0.5447	1.09E-07	1.09E-07 turquoise
USP4	0.01867	-0.05676	0.07932	0.00588	-0.1794	0.22959	0.14556	-0.06745	0.06745	0.80853	0.46086	0.30242	0.93915	0.01888	0.00252	0.05748	0.38072	0.38072 turquoise
CLDN1	-0.00168	-0.08048	0.06367	0.15285	-0.15762	0.18967	0.2136	-0.23206	0.23206	0.98261	0.29538	0.40804	0.04595	0.0395	0.01297	0.00503	0.00226	0.00226 yellow
LPCAT1	-0.05835	-0.14676	-0.09933	-0.02169	-0.00984	-0.19985	-0.03256	0.18498	-0.18498	0.44842	0.05544	0.19614	0.77828	0.89834	0.00878	0.67244	0.01543	0.01543 grey
SEMA3C	-0.01856	-0.05734	0.0799	0.13652	-0.21098	0.22051	0.16269	0.11384	-0.11384	0.8096	0.4563	0.29886	0.07499	0.00561	0.00375	0.0335	0.1382	0.1382 turquoise
EPS8	-0.01144	-0.09817	0.11667	0.12669	-0.15058	0.26604	0.19312	-0.15672	0.15672	0.88199	0.20144	0.12859	0.09869	0.04931	0.00044	0.01138	0.04066	0.04066 turquoise
CD207	-0.10526	-0.12095	-0.05941	-0.07354	-0.01212	0.10608	-0.00357	0.11331	-0.11331	0.17063	0.11508	0.44016	0.33911	0.87503	0.16732	0.96302	0.14005	0.14005 grey
HLA-DRA	-0.14982	0.04059	0.02467	0.05276	-0.24295	0.17568	0.1281	0.29145	-0.29145	0.05049	0.59812	0.74874	0.49311	0.00137	0.02154	0.09496	0.00011	0.00011 blue
VWA8	-0.04069	-0.0292	-0.09015	-0.03273	-0.06699	0.02759	-0.04391	0.23536	-0.23536	0.59724	0.70458	0.24096	0.67087	0.38397	0.72021	0.56854	0.00194	0.00194 turquoise
PAXIP1	-0.06907	-0.03516	-0.00275	-0.02093	-0.03502	0.03202	0.0839	0.04663	-0.04663	0.36939	0.64796	0.97155	0.78587	0.64927	0.67757	0.27526	0.54472	0.54472 turquoise
NARS2	-0.04692	-0.08674	-0.03645	-0.03453	-0.02963	-0.04104	-0.0098	0.146	-0.146	0.54228	0.25926	0.636	0.65393	0.70044	0.5941	0.89882	0.05672	0.05672 turquoise
PPIC	-0.0188	-0.03465	0.03379	0.09298	-0.13779	0.24434	0.18186	-0.08256	0.08256	0.80716	0.65278	0.66086	0.22645	0.0723	0.00128	0.01728	0.28302	0.28302 black
ZNF286A	-0.01572	-0.1644	-0.05611	0.04244	-0.07825	-0.07174	-0.00087	0.35681	-0.35681	0.83831	0.03166	0.46608	0.58152	0.30901	0.35109	0.99095	1.66E-06	1.66E-06 turquoise
EFHD1	-0.16259	-0.15665	0.01977	0.08765	-0.20434	0.11743	0.03434	0.31779	-0.31779	0.03361	0.04075	0.79742	0.25432	0.00734	0.12612	0.65566	2.28E-05	2.28E-05 blue
AK7	0.01901	-0.13011	-0.12057	0.04889	0.15147	-0.08393	-0.0882	-0.06607	0.06607	0.80505	0.08985	0.11622	0.52538	0.04796	0.27509	0.25133	0.39057	0.39057 grey
C15orf40	-0.06062	-0.16071	-0.15343	-0.08683	0.12827	-0.12482	-0.04581	-0.02566	0.02566	0.4309	0.03575	0.04513	0.25881	0.09454	0.10382	0.55187	0.73907	0.73907 grey
ELP4	-0.04258	-0.02673	0.00439	-0.00547	-0.12085	-0.09857	-0.01776	0.26964	-0.26964	0.58027	0.72856	0.95458	0.94336	0.11538	0.19961	0.81766	0.00036	0.00036 turquoise
FOLR1	0.01111	0.10735	-0.03954	0.14464	0.09086	0.1178	0.08937	0.02699	-0.02699	0.88531	0.16224	0.60763	0.05909	0.23725	0.12491	0.24505	0.72598	0.72598 grey
BET1	0.05297	-0.16623	-0.01803	0.0208	0.08547	-0.03599	-0.00792	-0.04042	0.04042	0.49137	0.02979	0.81489	0.78713	0.26633	0.64023	0.91811	0.59964	0.59964 turquoise
MSH6	-0.07274	-0.08637	-0.05753	0.00939	-0.19054	0.0593	0.13092	0.17803	-0.17803	0.34439	0.2613	0.45482	0.90299	0.01255	0.44101	0.08786	0.01982	0.01982 turquoise
DTX1	0.0029	-0.05932	-0.00649	0.00236	-0.15065	0.09204	0.00803	0.2111	-0.2111	0.96998	0.44088	0.93287	0.97558	0.04921	0.2312	0.91695	0.00558	0.00558 blue
FAM222A	-0.01094	-0.10534	-0.25146	-0.13314	0.21563	-0.34541	-0.22317	0.33786	-0.33786	0.88705	0.17029	0.00091	0.08256	0.00462	3.71E-06	0.00335	6.21E-06	6.21E-06 brown
GEMIN2	0.06266	-0.03417	0.00847	0.03335	0.00434	-0.00078	0.09546	-0.09308	0.09308	0.41557	0.6573	0.91248	0.665	0.95506	0.9919	0.21423	0.22595	0.22595 turquoise
TNFRSF13	-0.07615	-0.00737	0.05242	-0.01166	-0.04091	0.02607	-0.11574	0.24575	-0.24575	0.32222	0.92375	0.49591	0.87972	0.59526	0.73505	0.13169	0.0012	0.0012 blue
CDCA7	-0.05114	0.21462	0.11867	0.06947	-0.00722	0.30737	0.18793	-0.27119	0.27119	0.50652	0.00482	0.12212	0.3666	0.9253	4.33E-05	0.01384	0.00033	0.00033 grey
JCHAIN	-0.12899	0.00928	0.03099	0.02835	-0.14074	0.17753	0.12798	0.28655	-0.28655	0.09269	0.90413	0.68745	0.71282	0.06635	0.02018	0.0953	0.00014	0.00014 blue
ZBTB34	-0.06638	-0.26728	-0.04495	0.06504	-0.09761	0.05291	-0.03539	0.09171	-0.09171	0.38836	0.00041	0.55934	0.39805	0.20405	0.49191	0.64582	0.23287	0.23287 turquoise
EGR4	0.02896	-0.08937	-0.10339	-0.13779	0.07505	-0.17722	-0.18586	-0.00396	0.00396	0.70689	0.24509	0.17841	0.0723	0.32925	0.0204	0.01494	0.95896	0.95896 grey
VMP1	0.04053	-0.07253	0.01171	-0.00017	0.01948	0.0904	0.18864	-0.29371	0.29371	0.59866	0.34581	0.87921	0.99829	0.80037	0.23964	0.01348	9.66E-05	9.66E-05 yellow
DNAL4	0.06887	-0.02537	-0.18176	-0.02661	0.08956	-0.13255	0.00372	-0.19752	0.19752	0.37075	0.74183	0.01735	0.72972	0.24408	0.08394	0.96147	0.00961	0.00961 grey
SCD	-0.03798	-0.08265	0.05201	-0.01022	0.01261	-0.01587	-0.01184	-0.14108	0.14108	0.62189	0.28251	0.49934	0.89449	0.86999	0.83674	0.8778	0.06568	0.06568 grey
NEFM	0.0884	-0.16614	-0.08137	0.03427	0.08054	-0.30255	-0.1944	0.38076	-0.38076	0.25022	0.02987	0.29004	0.65631	0.29502	5.77E-05	0.01084	2.79E-07	2.79E-07 brown
TRAPPC2L	0.01832	0.04397	-0.17461	-0.06948	0.0566	-0.11339	0.1173	-0.23202	0.23202	0.81205	0.56798	0.02236	0.3665	0.46212	0.13977	0.12654	0.00226	0.00226 grey
MEA1	0.06337	-0.01375	-0.03872	-0.02019	-0.0433	-0.03523	0.09426	-0.19831	0.19831	0.41027	0.85833	0.61512	0.79329	0.57387	0.64737	0.22006	0.00932	0.00932 greenyellow
LMNB1	-0.08228	0.03325	0.04727	0.00991	-0.06775	0.10901	0.14987	-0.23932	0.23932	0.28468	0.66597	0.53922	0.89766	0.37861	0.15581	0.05041	0.00162	0.00162 pink
NEK4	-0.03025	-0.14892	-0.07852	-0.02559	-0.02846	0.04205	-0.01168	0.17684	-0.17684	0.69449	0.0519	0.30736	0.73971	0.71176	0.58497	0.87952	0.02068	0.02068 turquoise
HACD4	-0.14035	-0.00349	0.01962	0.02839	-0.21615	0.18	0.07527	0.42259	-0.42259	0.0671	0.96384	0.79895	0.71245	0.00452	0.01848	0.32785	8.54E-09	8.54E-09 blue
SELENOW	0.05732	0.01251	-0.04116	0.01277	0.07155	0.08521	0.10094	-0.30301	0.30301	0.45647	0.87101	0.593	0.86835	0.35239	0.26783	0.18898	5.61E-05	5.61E-05 grey
DHX57	-0.06809	-0.07935	-0.12155	-0.02743	-0.07978	-0.06235	0.03507	0.25945	-0.25945	0.37624	0.30226	0.11325	0.72177	0.29963	0.41783	0.64883	0.00061	0.00061 turquoise
SLC25A28	0.03693	-0.02023	-0.03886	-0.03026	0.01864	-0.14676	0.05844	-0.07133	0.07133	0.63157	0.79288	0.61382	0.69437	0.80877	0.05544	0.44773	0.35384	0.35384 grey
PPIP5K1	-0.03634	-0.1446	-0.13089	-0.16158	0.10779	-0.16587	-0.13968	-0.12626	0.12626	0.63698	0.05917	0.08794	0.03475	0.16055	0.03014	0.06844	0.09985	0.09985 grey
SNAPC5	-0.08126	-0.08049	-0.11093	-0.02358	-0.06464	-0.01745	-0.03365	0.03312	-0.03312	0.29068	0.29529	0.14864	0.75952	0.40095	0.82081	0.66213	0.66717	0.66717 turquoise
GTDC1	-0.06086	-0.02532	0.03449	0.15064	-0.27313	0.12291	0.12571	0.1388	-0.1388	0.42913	0.74232	0.65426	0.04923	0.0003	0.10926	0.10137	0.07022	0.07022 black
RO60	-0.01352	-0.1258	0.01637	0.03928	-0.1496	0.13855	0.05243	0.15079	-0.15079	0.86071	0.10112	0.83167	0.60995	0.05082	0.07072	0.49587	0.049	0.049 turquoise
WBP1	-0.01251	-0.09742	-0.14531	-0.04402	0.28825	-0.13265	-0.115	-0.07822	0.07822	0.871	0.20493	0.05792	0.56753	0.00013	0.0837	0.13418	0.3092	0.3092 red
TRIM16	0.07314	0.01478	0.15589	0.0703	-0.02068	0.21543	0.28061	-0.43341	0.43341	0.34176	0.84786	0.04175	0.36088	0.78837	0.00466	0.0002	3.20E-09	3.20E-09 yellow
ABHD13	-0.02858	-0.10208	-0.04107	-0.01605	-0.04377	-0.05234	-0.08925	0.30297	-0.30297	0.71058	0.18399	0.59377	0.83492	0.56972	0.4966	0.24571	5.63E-05	5.63E-05 turquoise
BRINP1	0.03551	-0.09861	-0.19018	-0.11413	0.10959	-0.18681	-0.25497	0.29901	-0.29901	0.64469	0.19941	0.01272	0.1372	0.15361	0.01443	0.00076	7.11E-05	7.11E-05 brown
FAM53B	-0.0377	-0.01342	-0.08394	-0.01706	-0.00553	-0.13042	-0.02958	0.31121	-0.31121	0.62447	0.86169	0.27506	0.82469	0.94281	0.08908	0.7009	3.43E-05	3.43E-05 blue
SPATC1L	-0.04639	-0.08583	-0.24525	-0.10581	0.10037	-0.243	-0.03761	0.02082	-0.02082	0.5468	0.26435	0.00122	0.16841	0.19149	0.00136	0.62528	0.78698	0.78698 grey
POLR2G	-0.01584	-0.05877	-0.07048	-0.05549	-0.08882	-0.03533	0.09443	-0.10227	0.10227	0.83704	0.44515	0.35965	0.47096	0.24798	0.64639	0.21925	0.18317	0.18317 grey
PSPH	-0.0446	-0.2066	-0.08587	0.00866	0.09716	-0.15698	-0.04007	0.07033	-0.07033	0.56247	0.00671	0.26412	0.91049	0.20616	0.04032	0.60279	0.36069	0.36069 grey
CENPQ	-0.00403	-0.03069	0.01725	-0.02596	-0.06967	-0.0426	0.07328	0.10961	-0.10961	0.9583	0.69024	0.82277	0.73608	0.36525	0.58011	0.34084	0.15355	0.15355 turquoise
NKAPL	-0.01363	-0.17036	-0.0305	0.01947	-0.05001	-0.08036	-0.13058	0.48571	-0.48571	0.85957	0.0259	0.69209	0.8005	0.51594	0.29609	0.0887	1.65E-11	1.65E-11 brown
SCRN2	-0.02919	-0.05945	-0.1361	-0.14428	0.21569	-0.1568	-0.05882	-0.18794	0.18794	0.70474	0.43988	0.07591	0.05974	0.00461	0.04055	0.44474	0.01383	0.01383 red
USP25	-0.06846	-0.11025	-0.02855	0.01638	-0.09975	0.08405	0.01763	0.2273	-0.2273	0.3736	0.15115	0.71086	0.83163	0.19428	0.27443	0.81895	0.00279	0.00279 turquoise
BEND5	-0.07069	-0.0942	-0.17605	-0.00798	0.10321	-0.1822	-0.36116	0.41468	-0.41468	0.35821	0.22037	0.02127	0.91746	0.17915	0.01707	1.22E-06	1.71E-08	1.71E-08 brown
LARP1B	-0.06624	-0.1098	0.00281	-0.01757	0.04577	0.05597	-0.06975	-0.01984	0.01984	0.38932	0.15282	0.97092	0.81957	0.55218	0.46714	0.36467	0.79678	0.79678 turquoise
TMEM179	-0.01427	-0.11914	-0.30664	-0.18342	0.15935	-0.31965	-0.24361	0.35082	-0.35082	0.85301	0.12065	4.52E-05	0.01634	0.03736	2.03E-05	0.00132	2.55E-06	2.55E-06 brown
NUCB1	0.04224	-0.09004	-0.06403	0.00273	-0.01701	-0.14522	-0.08029	0.1184	-0.1184	0.58334	0.24153	0.40538	0.97173	0.82521	0.05807	0.2965	0.12297	0.12297 grey
ZNF131	-0.06746	-0.12876	-0.01851	0.03385	-0.09739	0.05208	0.12366	0.15623	-0.15623	0.38065	0.09328	0.81011	0.66028	0.2051	0.49871	0.10709	0.0413	0.0413 turquoise
PTPN21	-0.03182	-0.21482	-0.05916	0.02361	-0.07637	0.04116	-0.08574	0.26298	-0.26298	0.67946	0.00478	0.4421	0.75919	0.3208	0.59301	0.26484	0.00051	0.00051 turquoise
RPL34	0.04818	0.0841	-0.00696	-0.07004	-0.04204	0.05917	0.07416	-0.14938	0.14938	0.53147	0.27411	0.928	0.36264	0.58513	0.44207	0.33507	0.05117	0.05117 purple
TBC1D5	-0.05073	-0.05608	-0.0269	-0.01171	-0.21055	0.11311	0.04485	0.33353	-0.33353	0.50994	0.46626	0.72686	0.87915	0.00571	0.14074	0.56025	8.28E-06	8.28E-06 turquoise
LIPA	-0.06762	-0.05782	0.05717	0.08568	-0.15525	0.05513	-0.00848	0.35041	-0.35041	0.37952	0.45251	0.45769	0.26518	0.04261	0.47385	0.91234	2.62E-06	2.62E-06 blue
KCNF1	-0.01125	-0.10551	-0.17787	-0.09439	0.08244	-0.14626	-0.0908	0.06456	-0.06456	0.88394	0.16963	0.01994	0.21944	0.28376	0.05629	0.23755	0.40152	0.40152 grey

HIF3A	0.14635	-0.08856	-0.12176	0.07229	0.02879	-0.00404	0.02783	0.03862	-0.03862	0.05613	0.24937	0.11265	0.34742	0.70854	0.95814	0.71786	0.61604	0.61604	grey
EXD3	0.05872	-0.03351	-0.02643	-0.0221	0.17911	0.03288	0.09102	-0.3206	0.3206	0.44554	0.66352	0.73149	0.77419	0.01908	0.66941	0.23642	1.91E-05	1.91E-05	green
RBM28	-0.00952	-0.10177	-0.06372	0.01987	0.00886	-0.10849	0.02078	0.09671	-0.09671	0.9016	0.18535	0.40766	0.79642	0.9084	0.15783	0.78729	0.20825	0.20825	turquoise
ADAMTS7	-0.06727	-0.07311	0.04013	0.09654	-0.09307	0.06506	0.10984	0.0584	-0.0584	0.38202	0.34193	0.60228	0.20907	0.22598	0.39786	0.15266	0.44803	0.44803	black
TBC1D14	-0.08638	-0.13073	-0.04199	-0.01001	-0.09773	-0.01898	-0.00763	0.31132	-0.31132	0.26129	0.08833	0.58556	0.89657	0.20349	0.80541	0.92112	3.40E-05	3.40E-05	turquoise
NET1	0.08355	0.02012	0.12704	0.05507	-0.04565	0.21557	0.21989	-0.3858	0.3858	0.27728	0.79393	0.09777	0.47435	0.55327	0.00463	0.00386	1.88E-07	1.88E-07	yellow
SKAP1	-0.11452	0.00031	-0.07983	0.05585	0.10411	0.01944	-0.03685	-0.11176	0.11176	0.13584	0.99679	0.29934	0.4681	0.17537	0.80079	0.63226	0.1456	0.1456	grey
IFT172	0.03152	-0.04146	-0.09071	-0.05137	0.14214	0.04508	0.06506	-0.3359	0.3359	0.68235	0.59031	0.23804	0.50464	0.06367	0.55823	0.39784	7.07E-06	7.07E-06	grey
INPP5K	-0.07219	-0.16797	-0.16457	-0.13006	0.03351	-0.20304	-0.16455	0.23015	-0.23015	0.34807	0.02809	0.03148	0.08998	0.66349	0.00774	0.0315	0.00246	0.00246	grey
FNDC5	-0.05848	-0.14943	-0.2005	-0.08218	0.01551	-0.21676	-0.16398	0.55273	-0.55273	0.44737	0.05109	0.00855	0.28524	0.84046	0.0044	0.03211	4.57E-15	4.57E-15	brown
SBDS	0.00438	-0.14897	0.04035	0.00353	0.00564	0.08958	-0.01955	-0.02497	0.02497	0.9547	0.05182	0.60027	0.9635	0.94169	0.24397	0.79961	0.74576	0.74576	turquoise
LANCL1	-0.03435	-0.05095	0.01002	0.09655	-0.10952	0.12993	0.02116	0.22122	-0.22122	0.6556	0.5081	0.89648	0.20902	0.15387	0.09031	0.78357	0.00364	0.00364	turquoise
CXCL16	-0.02363	-0.02564	0.09459	0.0135	-0.03044	0.21477	0.1319	-0.14004	0.14004	0.75897	0.73923	0.21846	0.86087	0.69269	0.00479	0.08548	0.06772	0.06772	grey
FUT2	0.04144	0.08676	0.09312	0.04479	0.04264	0.27235	0.19087	-0.32729	0.32729	0.59051	0.25919	0.22574	0.56081	0.57976	0.00031	0.0124	1.25E-05	1.25E-05	yellow
CHIT1	0.01112	0.03715	0.09668	0.12065	-0.07181	0.11	-0.00598	0.18686	-0.18686	0.88523	0.62954	0.20841	0.11597	0.35062	0.15209	0.93814	0.0144	0.0144	grey
NONO	-0.0036	-0.15348	-0.07168	-0.01148	-0.0765	-0.05051	0.06202	-0.01118	0.01118	0.9627	0.04505	0.35152	0.88157	0.31996	0.51181	0.42033	0.88458	0.88458	turquoise
LRRC73	0.07817	-0.11191	-0.27443	-0.12764	0.23221	-0.40384	-0.17712	0.10925	-0.10925	0.30951	0.14504	0.00028	0.09619	0.00224	4.32E-08	0.02047	0.15492	0.15492	brown
IFT57	-0.04947	-0.11177	-0.20053	-0.06858	-0.04741	-0.02603	-0.15681	0.19727	-0.19727	0.52048	0.14556	0.00854	0.37279	0.53804	0.73544	0.04054	0.00971	0.00971	turquoise
TMEM51	0.02118	-0.1256	0.074	0.16801	-0.10796	0.34934	0.1462	-0.20624	0.20624	0.78339	0.10165	0.3361	0.02805	0.15986	2.82E-06	0.05638	0.0068	0.0068	grey
EVA1A	-0.01949	0.00343	-0.06398	0.08149	-0.19581	-0.07774	0.09587	0.2255	-0.2255	0.80028	0.96446	0.40579	0.28933	0.01027	0.31217	0.21226	0.00302	0.00302	grey
C6orf223	0.01278	0.03186	0.02384	0.04889	0.08965	0.14377	0.01754	-0.4553	0.4553	0.86829	0.6791	0.75698	0.52539	0.24357	0.06066	0.81992	3.93E-10	3.93E-10	yellow
NDUFB6	0.03578	-0.01251	-0.17135	-0.08706	0.02757	-0.22778	-0.08026	-0.0074	0.0074	0.6422	0.87097	0.02504	0.25754	0.72043	0.00273	0.29669	0.92345	0.92345	grey
NSUN5	0.08652	0.0689	0.07301	0.01273	-0.00515	0.09092	0.24994	-0.40243	0.40243	0.26049	0.37058	0.34262	0.86872	0.94671	0.23696	0.00098	4.86E-08	4.86E-08	yellow
ARMCX2	-0.02702	-0.16109	-0.18826	-0.09773	-0.065	-0.19318	-0.13193	0.46411	-0.46411	0.72575	0.03531	0.01367	0.20348	0.39831	0.01136	0.08542	1.62E-10	1.62E-10	brown
PWP1	0.0161	-0.02696	0.04516	-0.01477	-0.06713	-0.00192	0.04422	-0.00291	0.00291	0.83447	0.72632	0.55752	0.84793	0.38301	0.98013	0.56581	0.96991	0.96991	turquoise
ALG1L	0.12277	0.12235	0.05234	0.05423	-0.02792	0.14011	0.14265	-0.43142	0.43142	0.10967	0.11088	0.49656	0.48114	0.71696	0.06759	0.06271	3.84E-09	3.84E-09	grey
CD70	-0.02083	0.00534	0.10383	0.12391	-0.19343	0.04613	0.18632	-0.06801	0.06801	0.78687	0.94472	0.17653	0.10637	0.01125	0.54905	0.01469	0.37681	0.37681	grey
BTN3A1	-0.08951	0.04944	0.04201	0.03745	-0.12494	0.17671	0.09349	0.21464	-0.21464	0.24434	0.52074	0.58534	0.62675	0.10349	0.02078	0.22388	0.00482	0.00482	tan
TRMT11	-0.08014	-0.02967	0.02075	-0.0271	0.00173	0.06696	0.03661	-0.05369	0.05369	0.29742	0.70012	0.78763	0.72493	0.98204	0.38424	0.6345	0.48549	0.48549	turquoise
SWI5	0.04524	-0.15707	-0.17594	-0.10212	-0.00641	-0.18192	-0.00462	0.06035	-0.06035	0.55685	0.0402	0.02134	0.18381	0.93366	0.01725	0.95218	0.43295	0.43295	grey
CREB5	-0.17339	-0.21229	-0.06927	0.10422	-0.16376	0.1174	-0.03516	0.22689	-0.22689	0.02333	0.00531	0.36796	0.17493	0.03234	0.12622	0.64803	0.00284	0.00284	blue
SSH1	-0.08484	-0.16678	0.01693	0.07481	-0.22236	0.02975	0.0234	0.35898	-0.35898	0.26991	0.02924	0.82607	0.3308	0.00347	0.69929	0.76131	1.42E-06	1.42E-06	blue
RASEF	0.07619	0.01236	0.09441	0.07286	-0.04755	0.29949	0.1548	-0.25526	0.25526	0.32196	0.87253	0.21934	0.34361	0.53684	6.91E-05	0.04321	0.00075	0.00075	yellow
SGSM3	-0.00041	0.01982	-0.01937	-0.0787	0.235	0.01201	-0.03903	-0.27299	0.27299	0.99578	0.79691	0.80145	0.3062	0.00198	0.87609	0.61232	0.0003	0.0003	grey
ERICH2	0.10455	-0.03034	-0.1001	0.10852	0.0318	-0.16373	-0.05293	-0.02785	0.02785	0.17354	0.69361	0.19268	0.1577	0.67967	0.03237	0.49171	0.71767	0.71767	grey
SEMA4F	-0.10152	-0.18441	-0.10905	0.01271	-0.0445	-0.19438	-0.0311	0.09533	-0.09533	0.18645	0.01575	0.15566	0.86893	0.56329	0.01085	0.68634	0.21489	0.21489	turquoise
RASSF1	-0.01377	-0.02805	0.02684	-0.0124	-0.02367	0.12666	0.04813	-0.01361	0.01361	0.8581	0.71574	0.72745	0.87207	0.75861	0.09878	0.53187	0.85977	0.85977	grey
MED22	0.02732	-0.13243	-0.16665	-0.07425	-0.06963	-0.10949	-0.01549	0.16532	-0.16532	0.7228	0.08424	0.02937	0.33449	0.36551	0.15399	0.84059	0.0307	0.0307	turquoise
ALKAL2	-0.00241	-0.02381	-0.12338	0.08846	-0.02506	-0.00632	-0.07976	0.20763	-0.20763	0.97508	0.75719	0.10789	0.24992	0.74491	0.93464	0.29975	0.00643	0.00643	magenta
LRRC37B	-0.01769	-0.12584	-0.20895	-0.07812	0.11552	-0.17282	-0.11999	0.27521	-0.27521	0.81838	0.10101	0.00609	0.30979	0.13242	0.0238	0.11799	0.00027	0.00027	turquoise
F2RL1	0.05177	-0.0278	0.08339	0.09716	-0.06477	0.23514	0.12846	-0.36071	0.36071	0.50129	0.71816	0.27821	0.20618	0.40001	0.00196	0.09403	1.26E-06	1.26E-06	yellow
ST6GALN/	-0.02065	-0.09249	0.07251	0.09358	-0.21934	0.08414	0.17568	-0.14079	0.14079	0.78865	0.22891	0.34593	0.22345	0.00395	0.27391	0.02154	0.06626	0.06626	grey
KBTBD11	-0.0394	-0.06856	-0.08345	-0.02479	0.09317	-0.03867	0.01771	0.05402	-0.05402	0.60891	0.3729	0.27787	0.74753	0.22547	0.61552	0.81819	0.48288	0.48288	grey
FGFR1	-0.0553	-0.09598	-0.12242	-0.03486	-0.10291	-0.14012	-0.18601	0.65489	-0.65489	0.47248	0.21172	0.11068	0.65077	0.18042	0.06756	0.01485	2.58E-22	2.58E-22	blue
DHODH	0.02368	0.1605	-0.06974	-0.01508	0.04081	0.02398	0.13674	-0.21815	0.21815	0.75854	0.03599	0.36476	0.84483	0.59615	0.75554	0.07452	0.00415	0.00415	grey
CCDC69	-0.05395	0.00355	0.06127	0.11373	-0.16792	0.07942	-0.0636	0.44229	-0.44229	0.48342	0.96323	0.42599	0.13859	0.02814	0.30181	0.40857	1.39E-09	1.39E-09	blue
CKAP2L	-0.01911	0.05614	0.05928	-0.00605	-0.16275	0.08135	0.29623	-0.34467	0.34467	0.80402	0.46583	0.4412	0.93739	0.03343	0.29017	8.36E-05	3.91E-06	3.91E-06	pink
ACCS	-0.07613	-0.06616	-0.06964	-0.09712	0.11066	-0.01164	0.11651	-0.08467	0.08467	0.32232	0.38994	0.36539	0.20635	0.1496	0.87987	0.12912	0.27086	0.27086	red
UBAP2L	0.01352	-0.11875	-0.03544	-0.07871	-0.09226	0.05567	0.08847	-0.00919	0.00919	0.86066	0.12189	0.64534	0.30615	0.23009	0.46955	0.24985	0.90506	0.90506	turquoise
AFDN	0.0193	-0.12322	0.01073	0.06229	-0.0052	0.05685	0.00154	0.04403	-0.04403	0.8022	0.10835	0.88921	0.41829	0.94614	0.4602	0.984	0.56746	0.56746	turquoise
FEM1C	0.02108	-0.17345	0.02873	0.07274	-0.01168	0.13168	-0.00117	-0.04401	0.04401	0.78438	0.02329	0.70913	0.34441	0.87953	0.08601	0.98784	0.56758	0.56758	turquoise
TFPI2	-0.03508	-0.06898	-0.06322	0.14577	-0.01274	0.06988	0.01723	0.16041	-0.16041	0.64871	0.37001	0.41139	0.05713	0.86862	0.3638	0.82299	0.0361	0.0361	grey
MRC1	-0.10907	-0.07116	0.08709	0.06567	-0.13211	0.03743	-0.00309	0.44654	-0.44654	0.15559	0.35501	0.25738	0.39346	0.085	0.62698	0.96804	9.26E-10	9.26E-10	blue
MAPK13	-0.00737	0.00488	0.02396	-0.0066	0.06993	0.01082	0.03743	-0.26867	0.26867	0.92376	0.94947	0.75577	0.93168	0.36345	0.88825	0.62696	0.00038	0.00038	grey
TRADD	0.06438	0.01522	0.04962	-0.01989	0.07579	0.18655	0.22154	-0.50126	0.50126	0.40285	0.84337	0.51924	0.79625	0.32449	0.01456	0.00359	2.89E-12	2.89E-12	yellow
UHRF1BP1	-0.08133	-0.1523	-0.06657	-0.0119	-0.10872	-0.06872	-0.06557	0.43761	-0.43761	0.29031	0.04675	0.38699	0.87728	0.15694	0.37181	0.39416	2.16E-09	2.16E-09	turquoise
CFTR	-0.04764	-0.0618	-0.03465	0.1237	-0.05768	0.15906	-0.02957	0.08866	-0.08866	0.53609	0.42198	0.65281	0.10697	0.45366	0.03771	0.701	0.24886	0.24886	magenta
IFIT1	-0.00608	0.00661	0.00416	0.1038	-0.11857	0.01545	0.09264	0.05632	-0.05632	0.93714	0.93165	0.95691	0.1767	0.12243	0.84105	0.22814	0.46436	0.46436	tan
PRDM6	-0.0524	-0.11591	0.02718	0.08445	-0.1337	0.17161	0.03957	0.35923	-0.35923	0.49613	0.13111	0.72416	0.27211	0.08128	0.02481	0.60736	1.40E-06	1.40E-06	blue
SLC30A2	0.03247	0.07602	0.03341	0.12458	-0.10237	0.11352	-0.04288	0.11639	-0.11639	0.67331	0.32301	0.66443	0.10448	0.18276	0.13933	0.57764	0.12952	0.12952	magenta
LRRC8D	-0.08084	-0.07421	-0.01519	0.01246	-0.087	-0.00106	-0.11089	0.3025	-0.3025	0.29322	0.33473	0.84368	0.87147	0.25786	0.98904	0.14876	5.79E-05	5.79E-05	turquoise
CLSPN	-0.13637	0.02331	0.07783	0.00214	-0.15394	0.13232	0.27932	-0.06894	0.06894	0.07533	0.76215	0.31165	0.97786	0.0444	0.0845	0.00022	0.37028	0.37028	pink
RYR2	-0.04954	0.00294	-0.0794	0.09187	-0.01596	-0.04395	-0.07933	0.4681	-0.4681	0.51989	0.96953	0.30194	0.23205	0.83584	0.56815	0.30236	1.08E-10	1.08E-10	grey
C11orf21	-0.06895	0.06283	0.05667	0.07959	-0.02568	0.05461	-0.00969	0.3617	-0.3617	0.37021	0.41427	0.46162	0.30079	0.7388	0.47805	0.89991	1.17E-06	1.17E-06	blue
LDLR	-0.03677	0.01063	0.08179	-0.00546	-0.09382	0.2894	0.1354	-0.28279	0.28279	0.63306	0.89024	0.28754	0.9435	0.22225	0.00012	0.07744	0.00018	0.00018	yellow
BOK	0.01899	-0.00394	-0.01437	0.08942	-0.00387	0.04121	0.15353	-0.15007	0.15007	0.80532	0.95916	0.852	0.24482	0.95993	0.5925	0.04498	0.05009	0.05009	yellow
NUDT3	-0.07586	-0.0273	-0.07663	-0.02567	-0.17565	-0.01278	-0.04963	0.17806	-0.17806	0.32406	0.72303	0.31915	0.73894	0.02156	0.86828	0.5192	0.01981	0.01981	turquoise
PRR3	-0.02585	-0.16205	-0.12865	-0.07168	0.00725	-0.12329	-0.02823	0.13844	-0.13844	0.7372	0.03421	0.09355	0.35154	0.92501	0.10814	0.71397	0.07094	0.07094	red
FAM204A	0.03598	-0.07133	-0.04362	-0.04379	-0.08	-0.06739	0.01669	0.02443	-0.02443	0.6404	0.35389	0.5711	0.56956	0.29828	0.38115	0.82843	0.75113	0.75113	turquoise
ZNF835	-0.04395	-0.13487	-0.16664	-0.01182	0.02623	-0.13599	-0.22067	0.5051	-0.5051	0.56816	0.07863	0.02938	0.87807	0.73342	0.07615	0.00373	1.85E-12	1.85E-12	brown
SLC9B2	-0.19928	-0.00183	0.05308	0.0455	-0.04147	0.1666	0.00255	-0.02808	0.02808	0.00897	0.98103	0.4905	0.55456	0.59017	0.02942	0.97364	0.71538	0.71538	grey
RASGRP1	-0.16302	-0.16157	-0.09971	-0.00577	-0.02833	0.03571	-0.09544	0.25845	-0.25845	0.03314	0.03476	0.19443	0.94029	0.71303	0.64288	0.21433	0.00064	0.00064	turquoise
CCDC80	-0.07206	-0.02217	0.03382	0.11113	-0.2107	0.17198	0.06212	0.47801	-0.47801	0.34893	0.77351	0.66055	0.14787	0.00567	0.0245	0.41957	3.80E-11	3.80E-11	blue
ACBD7	0.04087	-0.07714	0.03077	0.11001	-0.04349	0.02124	0.01825	-0.15291	0.15291	0.59557	0.31593	0.68951	0.15204	0.57222	0.7827	0.81274	0.04586	0.04586	grey
SLC39A5	0.05929	0.09215	0.02777	0.10589	0.18953	0.13719	-0.01836	-0.06848	0.06848	0.44114	0.23063	0.71843	0.16809	0.01304	0.07356	0.81165	0.37347	0.37347	magenta
DXO	-0.00074	-0.02024	-0.15849	-0.1952	0.16703	-0.25257	-0.1463	0.01712	-0.01712	0.99232	0.7927	0.03842	0.01051	0.029	0.00086	0.05621	0.82408	0.82408	red
RBM7	-0.04728	-0.13622	0.00618	0.07525	-0.15859	0.13025	0.15171	0.01954	-0.01954	0.53919	0.07564	0.93605	0.32799	0.03828	0.08952	0.04762	0.79973	0.79973	turquoise
NF1	-0.04162	-0.18805	-0.02736	0.02782	-0.096	0.02496	0.04546	0.15405	-0.15405	0.58887	0.01378	0.72247	0.71792	0.21163	0.7459	0.55489	0.04425	0.04425	turquoise
AP1G1	-0.05342	-0.1633	-0.0566	-0.02049	-0.04527	0.04282	0.02909	0.05082	-0.05082	0.48774	0.03283	0.46219	0.79025	0.55661	0.57817	0.70569	0.50915	0.50915	turquoise
SERPINA3	-0.06693	-0.09949	-0.05511	0.1479	-0.102	0.08598	0.017	-0.02061	0.02061	0.38442	0.19543	0.47407	0.05355	0.18432	0.26348	0.82536	0.78906	0.78906	magenta
LRRN2	-0.09711	-0.08088	-0.19059	-0.03849	-0.02455	-0.08637	-0.2437	0.50247	-0.50247	0.20639	0.293	0.01253	0.61724	0.74994	0.26132	0.00132	2.51E-12	2.51E-12	brown
RSL1D1	0.03313	0.0825	0.07168	0.00575	-0.23154	0.25184	0.20902	-0.09909	0.09909	0.66709	0.28338	0.3515	0.94053	0.00231	0.00089	0.00608	0.19725	0.19725	turquoise
DHX32	0.01196	-0.09609	0.03779	0.03604	-0.0897	0.11889	0.1803	-0.33296	0.33296	0.87663	0.21122	0.62359	0.63981	0.24333	0.12143	0.01828	8.60E-06	8.60E-06	turquoise
FBL	0.13869	-0.00871	0.05508	0.04768	-0.12201	0.07475	0.19224	-0.33037	0.33037	0.07045	0.90997	0.47425	0.53576	0.11189	0.33124	0.01177	1.02E-05	1.02E-05	purple
RNASE6	-0.0678	-0.03861	0.03762	0.01698	-0.23653	0.16717	0.03533	0.42277	-0.42277	0.37823	0.6161	0.62516	0.82555	0.00184	0.02886	0.64646	8.40E-09	8.40E-09	blue
BST2	0.02586	0.05612	0.09997	0.14764	-0.13125	0.15184	0.20573	-0.12745	0.12745	0.73709	0.46598	0.19329	0.05397	0.08705	0.04743	0.00694	0.09667	0.09667	tan
HRCT1	-0.00724	0.02566	-0.07744	-0.05974	-0.05483	0.04807	0.0452	-0.2251	0.2251	0.92515	0.73905	0.31405	0.43769	0.4763	0.53238	0.55719	0.00308	0.00308	grey
RIN1	0.08881	0.03647	0.01602	-0.03505	0.09481	0.04338	0.1972	-0.41933	0.41933	0.24805	0.63585	0.83524	0.64907	0.21737	0.57319	0.00973	1.14E-08	1.14E-08	green
MKKS	0.05741	-0.04966	-0.30744	-0.06405	0.172	-0.32482	-0.13543	0.13094	-0.13094	0.45577	0.51889	4.31E-05	0.40524	0.02448	1.46E-05	0.07737	0.0878	0.0878	grey
MUC16	0.09639	-0.0615	-0.02479	0.06359	-0.14816	-0.02499	0.15109	-0.21178	0.21178	0.2098	0.42426	0.7476	0.40862	0.05313	0.7456	0.04854	0.00542	0.00542	grey
CSTB	-0.03239	-0.01627	0.06004	-0.01742	0.09708	0.08935	0.1963	-0.4692	0.4692	0.67407	0.83272	0.43533	0.82108	0.20654	0.2452	0.01007	9.61E-11	9.61E-11	yellow
ZMYM2	0.01877	-0.12367	-0.03047	-0.01973	0.02121	-0.01903	-0.04339	-0.01323	0.01323	0.80752	0.10705	0.69236	0.79784	0.78306	0.80483	0.5731	0.86363	0.86363	turquoise
EVL	-0.08344	-0.09224	-0.07519	0.02864	-0.05408	-0.13093	-0.15377	0.5402	-0.5402	0.27795	0.23018	0.32836	0.70997	0.48239	0.08785	0.04464	2.43E-14	2.43E-14	blue
PDK4	-0.11431	-0.10854	-0.15384	-0.01025	0.0677	-0.17848	-0.22492	0.46297	-0.46297	0.13657	0.15763	0.04454	0.89415	0.37899	0.01951	0.0031	1.82E-10	1.82E-10	brown
STRN	0.0052	-0.07687	0.06799	0.07022	-0.16616	0.26998	0.20697	-0.02653	0.02653	0.94622	0.31767	0.37693	0.36141	0.02985	0.00036	0.00661	0.73052	0.73052	turquoise
ENGASE	0.04768	-0.06489	0.0417	0.00659	0.31551	-0.01085	0.06645	-0.18956	0.18956	0.53573	0.39913	0.58814	0.93184	2.63E-05	0.88795	0.38787	0.01302	0.01302	red
PMPCA	-0.02668	-0.06267	-0.14833	-0.12768	0.05118	-0.18737	0.01973	-0.03388	0.03388	0.72904	0.41547	0.05286	0.09608	0.50618	0.01413	0.79787	0.66002	0.66002	grey
EIF2AK4	-0.18277	-0.11353	-0.05109	0.00374	-0.20614	-0.02566	0.12924	0.18377	-0.18377	0.01672	0.13927	0.50697	0.9613	0.00683	0.73901	0.09205	0.01613	0.01613	turquoise
MEX3B	-0.07061	-0.14887	-0.01465	0.10612	-0.13897	0.01643	0.03768	0.3188	-0.3188	0.35873	0.05198	0.84914	0.16714	0.06988	0.83108	0.62465	2.14E-05	2.14E-05	black
PIGL	-0.01822	-0.11433	-0.07223	-0.0204	0.17496	-0.16533	-0.09209	0.00273	-0.00273	0.81301	0.13648	0.34781	0.79118	0.02209	0.03069	0.23093	0.97174	0.97174	grey
ATF6B	0.00852	0.00146	-0.0666	-0.07895	-0.01983	0.00156	0.07542	-0.05743	0.05743	0.91197	0.98492	0.38678	0.30469	0.79681	0.98384	0.3269	0.45557	0.45557	turquoise
PFKL	-0.03753	-0.00531	0.00047	-0.02941	0.18598	0.02937	0.07493	-0.21635	0.21635	0.62598	0.94506	0.99512	0.70255	0.01488	0.70298	0.33003	0.00448	0.00448	grey
PKP1	0.05954	0.09246	-0.00751	0.06582	-0.04675	0.02355	0.16993	-0.24824	0.24824	0.43922	0.22904	0.92231	0.39238	0.54372	0.75984	0.02628	0.00106	0.00106	grey
AKAP1	0.09722	0.01831	-0.00731	-0.04308	0.10092	0.10416	0.06394	-0.35706	0.35706	0.20587	0.81209	0.92442	0.57587	0.18906	0.17517	0.40609	1.64E-06	1.64E-06	grey
IFT20	-0.09198	-0.14324	-0.11038	-0.0564	0.25727	-0.04934	-0.14635	0.06783	-0.06783	0.23151	0.06163	0.15065	0.46374	0.00068	0.52161	0.05613	0.37806	0.37806	grey
SGSM1	0.07151	-0.15072	-0.21154	-0.10751	0.18202	-0.25395	-0.26504	0.3214	-0.3214	0.35264	0.0491	0.00548	0.16161	0.01718	0.0008	0.00046	1.82E-05	1.82E-05	brown
CCDC154	-0.02367	-0.05283	-0.1155	-0.10548	0.26549	-0.16979	-0.08365	-0.00562	0.00562	0.75865	0.49253	0.1325	0.16974	0.00045	0.0264	0.2767	0.94182	0.94182	red
ACAD10	-0.01107	-0.10615	-0.11554	-0.12464	0.19701	-0.11554	-0.09911	-0.02698	0.02698	0.8857	0.16703	0.13235	0.10431	0.0098	0.13236	0.19717	0.72616	0.72616	turquoise
SAP30BP	-0.01468	-0.14905	-0.03383	-0.04508	0.01513	-0.02149	0.05505	-0.15402	0.15402	0.8489	0.0517	0.66047	0.55819	0.84426	0.78021	0.47455	0.04429	0.04429	grey
CLDN14	0.04632	-0.02873	-0.05888	0.11207	0.14697	0.0581	0.1171	-0.15766	0.15766	0.54741	0.70911	0.4443	0.14447	0.05508	0.45033	0.12717	0.03946	0.03946	grey
DPYSL2	0.01954	-0.07497	0.00939	0.00801	-0.08592	-0.00937	-0.04422	0.29355	-0.29355	0.79974	0.32981	0.90295	0.91715	0.26384	0.9032	0.56574	9.75E-05	9.75E-05	turquoise
SAC3D1	0.02361	0.04472	-0.10754	-0.04822	0.03977	-0.13156	0.20521	-0.35323	0.35323	0.75918	0.56136	0.1615	0.53112	0.60554	0.0863	0.00709	2.15E-06	2.15E-06	green
DEPP1	-0.07232	-0.18323	0.05579	0.06984	-0.02741	-0.0778	-0.14044	0.14302	-0.14302	0.34723	0.01645	0.46857	0.36403	0.72194	0.31181	0.06693	0.06202	0.06202	grey
EMC1	-0.04107	-0.15341	-0.03359	0.03659	-0.13915	-0.00134	0.05676	0.16142	-0.16142	0.59377	0.04514	0.66276	0.63472	0.06951	0.98612	0.46091	0.03492	0.03492	turquoise
MS4A8	0.09513	-0.10925	-0.08893	-0.0568	0.29541	-0.16214	-0.21636	0.02041	-0.02041	0.21583	0.15489	0.24739	0.46059	8.76E-05	0.03412	0.00448	0.791	0.791	grey
UFM1	0.01397	-0.14078	-0.03277	-0.00499	-0.0059	-0.06663	-0.09965	-0.00273	0.00273	0.85613	0.06627	0.6705	0.94835	0.93893	0.38656	0.1947	0.97175	0.97175	turquoise
TLE1	0.04378	-0.14119	-0.14976	-0.03521	-0.0397	0.05034	0.031	0.03845	-0.03845	0.56968	0.06548	0.05058	0.64757	0.60619	0.51318	0.68729	0.61757	0.61757	grey
SEPTIN4	-0.1019	-0.0606	-0.08042	-0.00551	-0.09416	0.03075	-0.11773	0.53214	-0.53214	0.18479	0.43103	0.29574	0.94293	0.22056	0.68972	0.12513	6.88E-14	6.88E-14	blue
IGFBP5	-0.06623	-0.15399	0.02602	0.04794	-0.17583	0.14107	0.04036	0.28742	-0.28742	0.38941	0.04434	0.73547	0.53353	0.02143	0.0657	0.60016	0.00014	0.00014	blue
RPL28	0.16941	0.1399	-0.01541	-0.05332	0.01985	0.00941	0.12102	-0.38794	0.38794	0.02676	0.068	0.84149	0.48854	0.79664	0.9028	0.11485	1.58E-07	1.58E-07	purple
PHF11	-0.00732	0.02083	0.00874	-0.00111	-0.08842	0.07192	0.03066	-0.06202	0.06202	0.92428	0.78685	0.90965	0.98846	0.25012	0.34988	0.69057	0.42032	0.42032	tan
UROS	0.03124	-0.01039	-0.15531	-0.11338	-0.00508	-0.21928	0.09868	-0.00077	0.00077	0.68499	0.89275	0.04252	0.13981	0.94743	0.00396	0.19911	0.99199	0.99199	grey
KRT5	0.13728	0.04605	0.07583	0.04417	-0.12068	0.04506	0.1362	-0.15172	0.15172	0.07338	0.54978	0.32427	0.56617	0.11589	0.55841	0.07568	0.0476	0.0476	grey
NUP214	-0.00883	-0.12701	-0.11722	-0.08407	-0.11309	-0.05936	-0.02063	0.19589	-0.19589	0.90872	0.09783	0.1268	0.27429	0.14084	0.44057	0.78881	0.01024	0.01024	turquoise
PRKCSH	0.16852	-0.02742	-0.08222	-0.02624	0.10797	-0.19066	0.00647	-0.16572	0.16572	0.02757	0.72181	0.28502	0.73334	0.15983	0.0125	0.93312	0.0303	0.0303	green
TMEM45B	0.02494	-0.05424	0.05506	0.0585	0.14083	0.10561	0.08222	-0.33541	0.33541	0.74613	0.48104	0.47448	0.44723	0.06616	0.16921	0.28504	7.31E-06	7.31E-06	grey
RGS11	-0.04008	-0.11866	-0.17647	-0.12315	0.18462	-0.22503	-0.22052	0.32116	-0.32116	0.6027	0.12215	0.02095	0.10855	0.01563	0.00309	0.00375	1.84E-05	1.84E-05	brown
THSD7B	-0.0397	-0.14225	0.01683	0.05901	-0.1845	0.1599	-0.07664	0.35545	-0.35545	0.60619	0.06346	0.82707	0.4433	0.01571	0.0367	0.31913	1.84E-06	1.84E-06	blue
SPON1	-0.05466	-0.06965	0.0603	0.08031	-0.21406	0.13531	0.06072	0.42812	-0.42812	0.47763	0.36532	0.43334	0.29639	0.00493	0.07764	0.43018	5.19E-09	5.19E-09	blue

LRCH1	-0.05264	-0.01196	0.01486	0.01658	-0.10937	0.11668	0.051	0.08695	-0.08695	0.49414	0.87657	0.847	0.82956	0.15446	0.12856	0.50768	0.25811	0.25811	turquoise
WTIP	-0.04329	-0.1042	0.00604	0.16292	-0.14156	0.11138	0.16185	0.16433	-0.16433	0.57396	0.175	0.93752	0.03324	0.06476	0.14699	0.03444	0.03173	0.03173	black
RAD21	-0.02053	-0.09569	-0.01936	0.00133	-0.07885	-0.06785	0.01817	0.024	-0.024	0.7898	0.21314	0.80155	0.98621	0.3053	0.37787	0.81349	0.75534	0.75534	turquoise
ZNF229	-0.12758	-0.21964	-0.02592	0.14519	-0.10747	0.04092	-0.00329	0.18018	-0.18018	0.09635	0.0039	0.73648	0.05813	0.16179	0.59515	0.96589	0.01836	0.01836	turquoise
CKMT1B	0.01529	0.06079	-0.00219	-0.07093	0.18826	-0.07681	-0.06162	-0.3426	0.3426	0.84268	0.42966	0.97734	0.35656	0.01367	0.31805	0.42336	4.50E-06	4.50E-06	grey
NEDD1	-0.05176	-0.10394	0.0267	0.06939	-0.1339	0.0806	0.03775	0.17708	-0.17708	0.50135	0.17608	0.72885	0.36713	0.08081	0.29468	0.62403	0.02051	0.02051	turquoise
SMARCAD	-0.12863	-0.1324	-0.0604	0.00668	-0.03348	-0.00645	-0.03512	0.29766	-0.29766	0.09361	0.08429	0.43258	0.93088	0.66379	0.93327	0.64841	7.69E-05	7.69E-05	turquoise
HSPB11	-0.05328	-0.04622	-0.02596	-0.04541	0.00022	-0.08072	0.07622	-0.13303	0.13303	0.48885	0.54827	0.73606	0.55531	0.99767	0.29394	0.32174	0.08282	0.08282	turquoise
ZNF689	-0.07041	-0.06225	-0.15152	-0.05255	-0.06472	-0.08012	-0.06899	0.38123	-0.38123	0.36011	0.41859	0.0479	0.49488	0.40031	0.29756	0.36993	2.69E-07	2.69E-07	turquoise
LY6D	0.01566	0.05467	0.06068	0.08706	-0.00137	0.01249	0.11038	-0.17942	0.17942	0.83889	0.47762	0.4305	0.2575	0.98584	0.87124	0.15065	0.01887	0.01887	grey
TBCCD1	-0.10217	-0.19231	-0.03556	0.03153	-0.1422	0.17561	0.11104	0.04339	-0.04339	0.18361	0.01174	0.64427	0.68221	0.06355	0.02159	0.14821	0.57308	0.57308	turquoise
CD83	-0.09734	-0.07712	0.03942	0.01398	-0.1017	0.08092	-0.04389	0.46182	-0.46182	0.20531	0.31607	0.60869	0.85596	0.18565	0.29271	0.56866	2.05E-10	2.05E-10	blue
ZMYM4	-0.07408	-0.17665	-0.02125	0.0484	-0.13792	0.03356	-0.00497	0.23897	-0.23897	0.33559	0.02082	0.78266	0.52958	0.07203	0.66305	0.94861	0.00165	0.00165	turquoise
SUCLA2	-0.05014	-0.06239	-0.07908	-0.04617	-0.00128	-0.0884	-0.07392	0.2634	-0.2634	0.51484	0.41754	0.30391	0.54878	0.98671	0.25025	0.33662	0.0005	0.0005	turquoise
TWF2	-0.00708	0.06567	0.04621	0.02508	-0.09299	0.162	0.19583	-0.27485	0.27485	0.92677	0.39346	0.54838	0.74471	0.22641	0.03427	0.01026	0.00027	0.00027	yellow
BDP1	-0.03167	-0.13274	-0.0657	0.00219	-0.10704	-0.00801	-0.02231	0.26258	-0.26258	0.68093	0.08351	0.39321	0.9773	0.16347	0.91714	0.77214	0.00052	0.00052	turquoise
ARFGAP2	0.09001	-0.00023	-0.07976	-0.07339	-0.03663	-0.06539	0.00595	-0.13316	0.13316	0.24169	0.99759	0.29976	0.34011	0.63429	0.39549	0.93848	0.08253	0.08253	grey
MIER3	-0.01772	-0.22138	-0.05087	-0.00045	0.01916	-0.03772	-0.10008	0.20578	-0.20578	0.81809	0.00362	0.50873	0.9953	0.80355	0.62429	0.1928	0.00693	0.00693	turquoise
ERO1A	-0.00294	-0.09468	0.08843	0.05859	-0.01713	0.14504	0.12716	-0.44334	0.44334	0.96957	0.21804	0.2501	0.44652	0.82403	0.05839	0.09745	1.26E-09	1.26E-09	turquoise
PLEKHB1	0.08508	-0.18435	-0.22578	-0.1476	0.1389	-0.13019	-0.19398	-0.02121	0.02121	0.26853	0.01579	0.00299	0.05405	0.07002	0.08967	0.01101	0.78306	0.78306	grey
TIMM23	-0.04357	#######	0.0182	-0.02502	-0.10271	-0.01579	0.15497	-0.29681	0.29681	0.57153	0.99985	0.81327	0.74534	0.1813	0.83757	0.04298	8.08E-05	8.08E-05	turquoise
MAP4K2	-0.11104	-0.02913	-0.03408	-0.03688	-0.1319	-0.08994	-0.04567	0.20074	-0.20074	0.14822	0.70527	0.65816	0.63206	0.0855	0.24207	0.5531	0.00847	0.00847	blue
TRIM56	0.04095	-0.11545	-0.05938	0.01935	-0.06845	0.11914	0.10881	-0.03529	0.03529	0.59486	0.13266	0.4404	0.80169	0.37373	0.12066	0.15657	0.64678	0.64678	turquoise
APLP1	-0.04288	-0.11334	-0.27155	-0.10792	0.09584	-0.36822	-0.27193	0.43981	-0.43981	0.57759	0.13995	0.00033	0.16003	0.21243	7.23E-07	0.00032	1.76E-09	1.76E-09	brown
KLHL28	-0.00289	-0.18228	0.00089	0.05939	-0.05789	0.05923	-0.04457	0.21866	-0.21866	0.97011	0.01702	0.9908	0.44038	0.45201	0.44156	0.56271	0.00406	0.00406	turquoise
AGPS	0.02185	-0.02434	0.10469	0.11786	-0.18183	0.20944	0.1419	-0.02501	0.02501	0.7767	0.75196	0.17296	0.12471	0.01731	0.00597	0.06413	0.74545	0.74545	turquoise
RNF208	0.02648	-0.07591	-0.193	-0.10389	0.16537	-0.13748	0.00629	-0.30148	0.30148	0.73097	0.32376	0.01143	0.17629	0.03065	0.07295	0.93492	6.15E-05	6.15E-05	green
PTPN9	-0.0917	-0.14926	-0.01268	0.04393	-0.16356	0.10424	0.05787	0.12191	-0.12191	0.23294	0.05136	0.86922	0.56829	0.03255	0.17486	0.45217	0.11219	0.11219	turquoise
TMEM150	-0.11384	-0.03989	-0.11636	0.00989	-0.08914	-0.0655	-0.19072	0.61499	-0.61499	0.1382	0.60449	0.12963	0.89788	0.24629	0.3947	0.01247	3.60E-19	3.60E-19	blue
LAMTOR1	0.01875	-0.04121	-0.11975	-0.06869	-0.05727	-0.05452	0.10674	-0.11646	0.11646	0.80769	0.59256	0.11873	0.37201	0.45688	0.47878	0.16469	0.12929	0.12929	grey
PGLS	0.07532	0.01798	-0.08649	-0.05236	0.11762	-0.03524	0.13002	-0.40992	0.40992	0.32754	0.8154	0.26066	0.49638	0.1255	0.64728	0.09008	2.58E-08	2.58E-08	green
OTULINL	-0.01107	-0.11482	-0.15134	-0.12769	0.00721	-0.15711	-0.10232	0.4169	-0.4169	0.88571	0.1348	0.04816	0.09603	0.92543	0.04016	0.18297	1.41E-08	1.41E-08	brown
LMF2	-0.00014	0.09194	-0.00524	0.09907	0.10787	-0.13351	-0.05812	-0.09109	0.09109	0.99852	0.2317	0.94573	0.19735	0.1602	0.08171	0.45023	0.23608	0.23608	grey
ELAVL4	0.00564	-0.11517	-0.24474	-0.14053	0.07952	-0.26405	-0.18876	0.46977	-0.46977	0.94168	0.13361	0.00126	0.06676	0.30119	0.00048	0.01342	9.05E-11	9.05E-11	brown
COBLL1	-0.01314	-0.05521	-0.01244	0.09885	-0.17947	0.13241	0.00447	0.24843	-0.24843	0.86452	0.47323	0.87168	0.19833	0.01883	0.08429	0.95368	0.00105	0.00105	turquoise
C7orf26	-0.00048	-0.11079	-0.12768	0.00153	0.09291	-0.185	-0.09537	0.1673	-0.1673	0.99503	0.14912	0.09606	0.98414	0.2268	0.01542	0.21469	0.02873	0.02873	grey
RBM5	0.00253	-0.1445	-0.10141	-0.07961	0.11807	-0.03599	-0.1198	0.10788	-0.10788	0.9738	0.05934	0.18693	0.30064	0.12404	0.64031	0.11859	0.16019	0.16019	red
USP33	-0.07863	-0.19963	-0.02337	-0.00048	-0.0665	0.03518	-0.04973	0.20278	-0.20278	0.30668	0.00885	0.76154	0.99499	0.3875	0.64781	0.51834	0.00782	0.00782	turquoise
ZNF283	-0.00807	-0.13907	-0.06875	-0.01103	-0.05455	0.02053	0.11205	0.12922	-0.12922	0.91657	0.06967	0.3716	0.88612	0.47854	0.78984	0.14453	0.09209	0.09209	turquoise
DYNLT1	-0.00918	-0.01598	0.00391	-0.01776	-0.03938	0.00639	0.12636	-0.27095	0.27095	0.90515	0.83564	0.95954	0.81765	0.60907	0.93391	0.09958	0.00034	0.00034	turquoise
LRMDA	0.00526	-0.01799	-0.12326	0.04775	-0.16156	0.02474	0.00341	0.24368	-0.24368	0.9456	0.81536	0.10825	0.53516	0.03477	0.74806	0.96471	0.00132	0.00132	grey
C20orf27	0.03847	0.17026	-0.1196	-0.01992	0.03003	-0.17405	0.18923	-0.05481	0.05481	0.61739	0.02599	0.11922	0.79595	0.69663	0.0228	0.01318	0.47645	0.47645	grey
ATF6	0.01359	-0.06286	-0.05451	-0.06387	-0.08852	0.08375	0.06972	0.15629	-0.15629	0.85993	0.41409	0.47887	0.40659	0.2496	0.27615	0.36488	0.04122	0.04122	turquoise
SLC25A3	0.06985	-0.03076	-0.05618	-0.10331	0.08051	-0.09357	-0.01478	0.00346	-0.00346	0.36396	0.6896	0.46548	0.17872	0.29521	0.22352	0.84784	0.96422	0.96422	turquoise
CDK5RAP	0.04092	-0.0565	-0.13633	-0.07614	0.17619	-0.18333	-0.00473	-0.12606	0.12606	0.59518	0.46294	0.07542	0.32226	0.02116	0.01639	0.95107	0.10041	0.10041	grey
VBP1	-0.07129	-0.16729	-0.08384	-0.07838	-0.04702	-0.10991	-0.00371	0.10466	-0.10466	0.35417	0.02874	0.27561	0.30822	0.54138	0.1524	0.96162	0.17308	0.17308	turquoise
MYO10	0.0237	-0.15276	-0.07146	-0.02118	0.0046	-0.0802	-0.00368	0.05642	-0.05642	0.75833	0.04608	0.35302	0.78333	0.95234	0.29704	0.96192	0.46362	0.46362	turquoise
ASPDH	0.02695	-0.12411	-0.24613	-0.14913	0.1867	-0.35401	-0.23878	0.23717	-0.23717	0.72645	0.10582	0.00117	0.05156	0.01448	2.03E-06	0.00166	0.00179	0.00179	brown
PAPLN	-0.04824	-0.09033	-0.006	0.07978	-0.07002	0.15629	-0.01612	0.31225	-0.31225	0.53093	0.24	0.93788	0.29964	0.36278	0.04122	0.83429	3.21E-05	3.21E-05	blue
USP44	0.02542	-0.14643	-0.02913	0.11747	-0.10965	-0.06505	-0.15792	0.33709	-0.33709	0.74139	0.05599	0.70526	0.12599	0.1534	0.39796	0.03912	6.53E-06	6.53E-06	grey
CCZ1B	-0.10248	-0.09414	0.02611	0.00163	-0.04256	0.12089	0.1071	-0.05239	0.05239	0.18229	0.22067	0.7346	0.98312	0.58046	0.11524	0.16325	0.49617	0.49617	turquoise
ZNF573	-0.00064	-0.14483	-0.13136	-0.05758	-0.08335	0.01901	0.02067	0.30635	-0.30635	0.99342	0.05875	0.0868	0.4544	0.27844	0.80503	0.7884	4.60E-05	4.60E-05	turquoise
CCNH	0.02225	-0.09075	-0.05854	-0.02849	-0.04367	-0.03686	-0.08137	0.05818	-0.05818	0.77264	0.23781	0.44694	0.71144	0.57059	0.63216	0.29005	0.44973	0.44973	turquoise
EFCAB11	0.02007	-0.08925	-0.04601	0.10042	0.05212	-0.05321	0.04339	-0.15114	0.15114	0.79443	0.24568	0.55016	0.19128	0.49836	0.48942	0.57306	0.04847	0.04847	turquoise
ATP6V1C2	0.01607	-0.01456	-0.0405	-0.04018	0.16818	-0.03468	-0.03132	-0.18847	0.18847	0.83471	0.85009	0.59889	0.6018	0.02789	0.65246	0.68423	0.01356	0.01356	grey
EPHX3	0.01348	-0.17316	0.053	0.0641	-0.02957	0.08605	0.0792	0.0417	-0.0417	0.86105	0.02352	0.4912	0.40486	0.70104	0.26313	0.30314	0.58815	0.58815	grey
C8G	0.02134	-0.04169	-0.27836	-0.20609	0.21098	-0.23475	-0.09197	-0.16993	0.16993	0.78171	0.58824	0.00023	0.00685	0.00561	0.002	0.23157	0.02629	0.02629	grey
SNCA	-0.03933	-0.07063	-0.11242	-0.03026	0.09918	-0.16989	-0.22933	0.38864	-0.38864	0.60953	0.35866	0.14321	0.69439	0.19682	0.02632	0.00255	1.50E-07	1.50E-07	grey
NUMB	0.00183	-0.10545	0.0648	0.10868	-0.0131	0.16018	0.09039	-0.11204	0.11204	0.98104	0.16985	0.39976	0.15709	0.86496	0.03637	0.2397	0.14459	0.14459	turquoise
STARD3NI	-0.09396	-0.21358	-0.08962	-0.01637	-0.03633	-0.09255	-0.06614	0.22009	-0.22009	0.22156	0.00503	0.24373	0.8317	0.63714	0.22863	0.3901	0.00382	0.00382	turquoise
MAN2A1	-0.00226	-0.13464	0.02477	0.11545	-0.1139	0.16544	0.07536	0.19143	-0.19143	0.9766	0.07914	0.74773	0.13266	0.13799	0.03058	0.3273	0.01214	0.01214	turquoise
WASF3	-0.09322	-0.09807	-0.18749	-0.0187	-0.04527	-0.13857	-0.15461	0.67601	-0.67601	0.22525	0.2019	0.01406	0.80814	0.55658	0.07069	0.04348	3.51E-24	3.51E-24	turquoise
DCAF15	0.03161	-0.00484	-0.04971	-0.03597	0.12685	-0.07543	0.0064	-0.20681	0.20681	0.68148	0.94986	0.51851	0.64045	0.09826	0.32681	0.93377	0.00665	0.00665	red
SERPINB1	-0.02884	0.17074	0.15504	-0.02669	0.05997	0.16192	0.14135	-0.40096	0.40096	0.70809	0.02557	0.04288	0.72893	0.43585	0.03436	0.06516	5.49E-08	5.49E-08	yellow
TIMM17A	0.01965	0.07491	-0.01396	-0.05727	-0.07621	0.08226	0.19262	-0.21644	0.21644	0.79861	0.3302	0.85623	0.45683	0.32182	0.28479	0.0116	0.00446	0.00446	greenyellow
PRICKLE2	-0.05465	-0.13182	-0.13588	-0.00506	-0.17724	-0.08072	-0.10601	0.53514	-0.53514	0.47778	0.08568	0.07639	0.94766	0.02039	0.29393	0.16759	4.69E-14	4.69E-14	blue
NUP43	0.00828	-0.02917	-0.01904	-0.00211	-0.03119	-0.01594	0.16377	0.03087	-0.03087	0.91438	0.70487	0.80478	0.97816	0.68546	0.83606	0.03233	0.68853	0.68853	turquoise
ORC4	0.00735	-0.10138	-0.06353	0.0589	-0.04257	-0.01529	0.02904	-0.07683	0.07683	0.92396	0.18702	0.40906	0.44415	0.58034	0.84265	0.7061	0.3179	0.3179	turquoise
ZNF335	0.02408	-0.06604	-0.05874	-0.01635	0.13054	-0.0689	-0.05509	0.0344	-0.0344	0.75453	0.3908	0.4454	0.83191	0.08878	0.37058	0.47422	0.65513	0.65513	red
SLC66A1	-0.08041	-0.1099	-0.09409	-0.08239	0.08834	-0.06118	-0.0287	0.03803	-0.03803	0.29581	0.15247	0.22093	0.28405	0.25057	0.42666	0.70941	0.62138	0.62138	grey
ARMC9	-0.04308	-0.14521	-0.00703	0.00939	-0.07433	0.05332	0.06065	0.13282	-0.13282	0.57584	0.05809	0.92724	0.90303	0.33396	0.48856	0.43067	0.08332	0.08332	black
TUBGCP3	-0.02301	-0.01987	0.01176	-0.01215	-0.00776	-0.0025	0.02419	0.11956	-0.11956	0.76518	0.79642	0.87871	0.8747	0.91974	0.97412	0.75351	0.11932	0.11932	turquoise
PIAS3	0.00914	-0.09652	0.0472	-0.02269	-0.10473	0.11916	0.15033	-0.17794	0.17794	0.90551	0.20917	0.53983	0.76832	0.1728	0.12058	0.04969	0.01989	0.01989	turquoise
UBA5	-0.01919	-0.01193	-0.13929	-0.05588	0.0493	-0.05085	0.04242	0.13941	-0.13941	0.80325	0.87694	0.06922	0.46789	0.52195	0.50896	0.58169	0.06898	0.06898	turquoise
SPEG	-0.0495	-0.05586	-0.18022	-0.00556	0.18651	-0.27673	-0.04852	0.26484	-0.26484	0.52026	0.46806	0.01834	0.94247	0.01458	0.00025	0.52859	0.00046	0.00046	brown
PAPOLA	0.02649	-0.14948	-0.01346	0.07396	0.02388	-0.03549	-0.0882	0.13439	-0.13439	0.73089	0.05101	0.86128	0.33638	0.75652	0.64491	0.25132	0.07971	0.07971	turquoise
ISL2	-0.06626	-0.02351	-0.00242	0.03203	0.02342	0.01003	0.14352	-0.04676	0.04676	0.38923	0.76016	0.97497	0.6775	0.76113	0.89644	0.06111	0.54364	0.54364	grey
AK8	0.05405	-0.05953	-0.20824	-0.02383	0.20614	-0.14414	-0.01625	-0.03269	0.03269	0.48262	0.43925	0.00627	0.75702	0.00683	0.05998	0.83294	0.67128	0.67128	grey
RIPOR1	0.01677	-0.11483	0.00256	0.02506	-0.15835	0.09201	0.09326	0.09573	-0.09573	0.82763	0.13478	0.97349	0.74489	0.03859	0.23132	0.22505	0.21295	0.21295	black
KRT81	0.05243	-0.12229	-0.04524	-0.06157	0.01449	-0.03993	0.01655	-0.08504	0.08504	0.49582	0.11108	0.5568	0.42368	0.85075	0.60413	0.82986	0.26875	0.26875	grey
RAB7B	-0.07643	-0.03216	-0.04067	-0.01811	-0.05606	0.07598	0.06606	0.15566	-0.15566	0.32043	0.6763	0.59739	0.81417	0.46645	0.32329	0.39067	0.04205	0.04205	blue
ACKR3	-0.10642	-0.06519	0.06749	0.14306	-0.11247	0.15278	0.02625	0.21116	-0.21116	0.16594	0.39696	0.38043	0.06196	0.14301	0.04606	0.73322	0.00556	0.00556	black
INO80	-0.05977	-0.1033	-0.01613	0.01315	-0.09543	0.06485	0.03587	0.07886	-0.07886	0.43744	0.17877	0.83412	0.86445	0.21438	0.39942	0.64138	0.30524	0.30524	turquoise
IGLL5	-0.1173	0.07075	0.0358	0.00155	-0.11585	0.17706	0.10289	0.22431	-0.22431	0.12652	0.35784	0.64208	0.98396	0.13131	0.02052	0.18053	0.00318	0.00318	blue
DDC	-0.03456	0.03044	-0.13916	-0.16861	0.217	-0.20056	-0.20333	0.15972	-0.15972	0.65358	0.69265	0.06949	0.02749	0.00436	0.00853	0.00765	0.03692	0.03692	brown
PLS3	-0.04617	-0.08638	0.08214	0.13433	-0.18495	0.17982	0.15777	-0.02995	0.02995	0.54876	0.26126	0.2855	0.07983	0.01545	0.0186	0.03931	0.69742	0.69742	black
GJB4	0.01687	-0.05964	0.13134	0.14267	0.01759	0.19062	0.1718	-0.34289	0.34289	0.82669	0.43839	0.08684	0.06268	0.81942	0.01251	0.02465	4.41E-06	4.41E-06	yellow
LNX1	0.01064	-0.14501	-0.09276	0.00688	0.05091	0.02546	0.04832	-0.07939	0.07939	0.89017	0.05844	0.22753	0.92883	0.50842	0.74101	0.53029	0.302	0.302	grey
B4GALT1	-0.03577	-0.04052	0.02574	0.09995	-0.21588	0.17121	0.13223	-0.02146	0.02146	0.64227	0.59872	0.73823	0.19335	0.00457	0.02516	0.0847	0.78052	0.78052	turquoise
PLCXD1	0.03301	-0.11982	-0.00761	-0.0507	0.07428	0.09492	0.03552	-0.13931	0.13931	0.6682	0.11852	0.92127	0.51018	0.33424	0.21687	0.6446	0.06918	0.06918	grey
ASCC3	0.02674	-0.04736	-0.01161	-0.00838	-0.15438	0.02078	0.08586	0.17469	-0.17469	0.72843	0.53851	0.8802	0.91337	0.04379	0.78734	0.26418	0.0223	0.0223	turquoise
TTC13	0.04045	-0.04366	-0.1272	-0.12256	0.14148	-0.09668	-0.02835	-0.01563	0.01563	0.59935	0.57073	0.09734	0.11027	0.06492	0.20841	0.71277	0.83921	0.83921	turquoise
PANK1	0.07417	-0.08495	-0.11438	-0.03125	0.04855	-0.11084	0.04417	0.07796	-0.07796	0.33499	0.26929	0.13633	0.68488	0.52833	0.14896	0.5662	0.31078	0.31078	grey
TIGD2	-0.04667	-0.12346	-0.02579	-0.05604	-0.05769	0.0873	0.18153	-0.14354	0.14354	0.54444	0.10767	0.73778	0.46659	0.45355	0.25621	0.01749	0.06107	0.06107	turquoise
ZNF275	-0.08845	-0.19983	-0.12885	-0.04295	-0.13933	-0.02945	-0.11417	0.48753	-0.48753	0.25	0.00878	0.09305	0.57703	0.06913	0.70222	0.13705	1.36E-11	1.36E-11	turquoise
ADAM10	-0.04985	-0.09466	0.02743	0.04451	-0.15643	0.11201	0.16472	-0.10166	0.10166	0.51728	0.21814	0.72174	0.56319	0.04103	0.14467	0.03132	0.1858	0.1858	turquoise
FOS	-0.04304	-0.07454	0.07188	0.02109	0.10371	0.14783	0.04951	0.071	-0.071	0.57616	0.3326	0.35017	0.78427	0.17707	0.05365	0.52015	0.35614	0.35614	blue
PRR22	-0.02386	0.12472	-0.15545	-0.07339	0.2933	-0.19022	-0.03449	-0.17862	0.17862	0.75671	0.10408	0.04234	0.3401	9.89E-05	0.0127	0.65424	0.01941	0.01941	green
SETMAR	-0.07765	-0.06911	-0.11793	-0.12379	-0.05786	0.07015	-0.02986	0.36397	-0.36397	0.31275	0.36908	0.1245	0.10673	0.45225	0.36191	0.69822	9.91E-07	9.91E-07	grey
CLDN6	-0.16675	-0.163	-0.00455	0.07904	-0.06833	0.05671	0.03639	0.02558	-0.02558	0.02927	0.03316	0.95293	0.30412	0.37452	0.46132	0.63653	0.73979	0.73979	grey
DSE	-0.08887	-0.10372	0.08151	0.09501	-0.26971	0.11612	0.10903	0.40985	-0.40985	0.24773	0.17701	0.28924	0.21641	0.00036	0.13041	0.15575	2.60E-08	2.60E-08	blue
GPRC5A	0.00305	0.0265	0.16557	0.12349	-0.15443	0.29471	0.26551	-0.41693	0.41693	0.96841	0.73082	0.03045	0.10757	0.04372	9.12E-05	0.00045	1.41E-08	1.41E-08	yellow
MAFF	-0.07388	-0.12431	0.0162	0.04041	-0.06219	0.17609	0.07781	-0.30025	0.30025	0.3369	0.10524	0.83344	0.59976	0.41903	0.02124	0.31176	6.61E-05	6.61E-05	yellow
MRPL1	-0.05501	0.14065	-0.01146	-0.10034	-0.04844	-0.02736	0.11983	-0.12753	0.12753	0.47488	0.06653	0.88178	0.19163	0.5292	0.72243	0.1185	0.09647	0.09647	turquoise
ARHGAP8	-0.02602	0.04999	0.1036	0.06242	0.05913	0.11985	0.13671	-0.3337	0.3337	0.73549	0.51611	0.1775	0.41736	0.44236	0.11842	0.0746	8.19E-06	8.19E-06	yellow
MCU	0.02438	0.01073	0.14814	0.05792	0.05593	0.15784	0.09929	-0.34549	0.34549	0.75163	0.88923	0.05316	0.45174	0.46751	0.03922	0.19632	3.69E-06	3.69E-06	yellow
FBXW4	0.15565	-0.04896	-0.13109	-0.0443	0.17207	-0.2145	0.00995	-0.10803	0.10803	0.04206	0.52486	0.08745	0.56505	0.02442	0.00484	0.89719	0.15959	0.15959	grey
PEX6	0.03262	-0.13585	-0.07836	-0.01833	0.15674	-0.20603	-0.0536	0.07017	-0.07017	0.67189	0.07645	0.3083	0.81189	0.04063	0.00686	0.48623	0.3618	0.3618	grey
ULBP2	0.02891	-0.04308	0.03675	0.09759	-0.10736	-0.09728	0.08953	-0.26128	0.26128	0.7074	0.57588	0.63323	0.20413	0.16223	0.20558	0.24423	0.00056	0.00056	grey
IMPDH1	-0.05693	-0.01137	-0.00871	0.02588	-0.08498	-0.07295	0.13171	-0.15432	0.15432	0.45952	0.88265	0.90999	0.73688	0.26912	0.34302	0.08594	0.04388	0.04388	grey
SLC39A8	-0.1057	0.09335	0.06495	0.05789	-0.23761	0.11975	0.06247	0.17727	-0.17727	0.16884	0.2246	0.39864	0.45203	0.00175	0.11876	0.41697	0.02036	0.02036	grey
VWA5A	-0.01443	-0.18525	-0.0974	-0.10084	0.05748	-0.03048	-0.11674	0.05717	-0.05717	0.85138	0.01528	0.20503	0.18941	0.45517	0.69225	0.12835	0.45768	0.45768	turquoise
UNC5C	-0.04391	-0.11324	-0.02254	-0.02042	-0.20737	0.07767	0.01717	0.39429	-0.39429	0.5685	0.1403	0.76983	0.79091	0.0065	0.3126	0.82361	9.50E-08	9.50E-08	blue
YTHDF1	0.08565	-0.0784	-0.06872	-0.01017	0.0669	-0.09933	0.04778	-0.13948	0.13948	0.26535	0.30805	0.3718	0.89502	0.3846	0.19617	0.53485	0.06883	0.06883	turquoise
DPY19L4	-0.03848	-0.14263	0.00463	0.08049	-0.10948	0.01214	0.04613	0.09974	-0.09974	0.61729	0.06275	0.95208	0.29533	0.15404	0.87473	0.5491	0.19429	0.19429	turquoise
SENP2	0.01442	-0.16055	-0.08518	-0.02847	-0.12321	0.02655	0.1058	0.01867	-0.01867	0.85151	0.03594	0.26797	0.71165	0.10838	0.73036	0.16844	0.80851	0.80851	turquoise
CYFIP2	-0.08474	-0.08234	-0.18754	-0.06053	0.00793	-0.21666	-0.29534	0.46044	-0.46044	0.27048	0.28434	0.01404	0.43158	0.918	0.00442	8.80E-05	2.35E-10	2.35E-10	brown
ARHGEF1	-0.01678	-0.15004	-0.07351	-0.04465	0.03028	-0.02912	0.00768	0.13532	-0.13532	0.82755	0.05014	0.33934	0.56198	0.69419	0.70539	0.92056	0.07762	0.07762	turquoise
GOLGA1	-0.0683	-0.11863	-0.10021	-0.01491	-0.02873	-0.03133	-0.01165	0.21432	-0.21432	0.37471	0.12224	0.19218	0.84649	0.70918	0.68414	0.87984	0.00488	0.00488	turquoise
SLC1A3	-0.0983	0.03776	0.07282	0.13973	-0.31256	0.11378	0.15865	0.30422	-0.30422	0.20085	0.6239	0.34391	0.06834	3.15E-05	0.1384	0.03821	5.23E-05	5.23E-05	blue
CALHM3	-0.07352	0.06923	0.07724	0.02497	-0.02958	0.09611	0.04004	-0.31891	0.31891	0.33925	0.36827	0.31529	0.74585	0.70097	0.2111	0.60308	2.13E-05	2.13E-05	grey
HAAO	-0.12371	-0.03267	0.00629	0.13075	-0.16009	0.19987	-0.06124	0.36414	-0.36414	0.10696	0.67144	0.93497	0.08827	0.03648	0.00877	0.42623	9.78E-07	9.78E-07	blue
CARM1	-0.04034	-0.09721	-0.00957	0.03284	-0.03051	0.04503	0.03818	0.05344	-0.05344	0.60037	0.20592	0.90116	0.66985	0.69198	0.55862	0.62008	0.48752	0.48752	turquoise
NCBP1	0.0153	-0.10613	-0.06085	-0.01699	-0.10926	-0.01699	0.05385	0.1175	-0.1175	0.84261	0.16709	0.42916	0.82539	0.15488	0.82549	0.48423	0.12587	0.12587	turquoise
DNAH1	-0.0393	-0.01396	-0.00924	-0.06526	0.04706	0.06407	-0.05292	0.06869	-0.06869	0.60981	0.8562	0.90454	0.39644	0.54102	0.40512	0.49179	0.37203	0.37203	red
IL18R1	-0.04188	-0.16181	-0.02497	-0.01902	-0.08438	0.04625	-0.07853	0.33024	-0.33024	0.58651	0.03448	0.74582	0.80496	0.27253	0.54808	0.30729	1.03E-05	1.03E-05	blue
ATG3	-0.03482	0.03313	-0.02532	-0.07982	-0.09905	0.09429	0.1122	-0.01073	0.01073	0.6512	0.66709	0.74239	0.29936	0.19742	0.21992	0.144	0.88927	0.88927	turquoise
MRPL13	-0.00053	0.03424	-0.02937	-0.03252	-0.03217	-0.06124	0.20098	-0.31771	0.31771	0.99453	0.65658	0.70295	0.6728	0.67614	0.42622	0.00839	2.29E-05	2.29E-05	turquoise
CYSLTR2	-0.09553	-0.07063	0.04464	0.06744	-0.01321	-0.1099	-0.10365	0.38389	-0.38389	0.21391	0.35864	0.56211	0.38082	0.86386	0.15245	0.17731	2.18E-07	2.18E-07	blue

AUH	-0.06381	-0.11392	-0.11119	-0.02601	-0.02639	-0.07416	-0.10825	0.0847	-0.0847	0.40702	0.13793	0.14768	0.73564	0.73186	0.33507	0.15875	0.2707	0.2707	turquoise
WIZ	0.04304	-0.07003	-0.07624	0.02328	0.02435	-0.00538	0.01798	-0.00813	0.00813	0.5762	0.3627	0.32163	0.76243	0.75188	0.94435	0.81547	0.9159	0.9159	grey
GIMAP2	-0.0391	0.03155	0.14054	0.08505	-0.09621	0.21635	0.09137	0.07167	-0.07167	0.61162	0.68211	0.06674	0.26873	0.21065	0.00448	0.23462	0.35159	0.35159	blue
HIST4H4	0.02056	-0.18435	-0.07914	-0.03398	0.02096	-0.09412	0.06907	0.02253	-0.02253	0.78952	0.01579	0.3035	0.65902	0.78555	0.2208	0.36937	0.76994	0.76994	red
YTHDC1	-0.02885	-0.17883	-0.09179	-0.00152	0.00086	0.02192	-0.07904	0.12147	-0.12147	0.70795	0.01927	0.23248	0.98431	0.99108	0.77596	0.30413	0.1135	0.1135	turquoise
SRP19	-0.03174	-0.10852	-0.11704	-0.08263	0.085	-0.17543	-0.06454	0.03498	-0.03498	0.68028	0.1577	0.12738	0.28265	0.26902	0.02173	0.40167	0.64969	0.64969	turquoise
LRRC34	-0.00875	#######	-0.14136	-0.05953	-0.07428	-0.18814	-0.07527	0.25091	-0.25091	0.90953	0.999	0.06515	0.4393	0.3343	0.01373	0.32782	0.00093	0.00093	grey
DDX31	-0.02396	-0.11193	-0.15781	-0.10216	-0.01496	-0.10412	0.00665	-0.00972	0.00972	0.75577	0.14496	0.03926	0.18366	0.84602	0.17532	0.93121	0.89962	0.89962	turquoise
MAP2K1	-0.05281	-0.14293	-0.02107	0.02317	-0.04834	-0.00563	-0.03093	0.05624	-0.05624	0.49268	0.0622	0.78444	0.76359	0.53013	0.94177	0.68802	0.46503	0.46503	turquoise
CCKBR	-0.01456	0.01024	-0.21575	-0.08693	0.0889	-0.20718	-0.24229	0.29922	-0.29922	0.85004	0.8943	0.0046	0.25823	0.24758	0.00655	0.00141	7.02E-05	7.02E-05	brown
ELP3	-0.05065	-0.01129	0.0035	0.0326	-0.10937	0.00533	0.05765	0.26044	-0.26044	0.51057	0.88344	0.96376	0.67211	0.15446	0.94486	0.45392	0.00058	0.00058	turquoise
FCGR3B	-0.14848	0.00678	0.1002	-0.02582	-0.09797	0.11351	0.05942	0.2592	-0.2592	0.05261	0.92989	0.19225	0.73742	0.20238	0.13936	0.44013	0.00062	0.00062	grey
SUPT3H	0.07455	-0.05144	0.04169	0.06315	-0.10261	0.07154	0.08546	0.03625	-0.03625	0.33248	0.50399	0.58822	0.41192	0.18171	0.35244	0.26643	0.63787	0.63787	turquoise
MAGI2	0.01186	-0.17645	-0.14908	-0.06025	0.0133	-0.24375	-0.19308	0.47855	-0.47855	0.87768	0.02096	0.05165	0.43377	0.86294	0.00132	0.0114	3.59E-11	3.59E-11	brown
ST7L	-0.15147	-0.10966	-0.05411	-0.00772	-0.03737	-0.05138	-0.02583	0.1716	-0.1716	0.04797	0.15335	0.48215	0.92016	0.62749	0.5045	0.73735	0.02482	0.02482	turquoise
USP19	0.03423	-0.04493	-0.02096	-0.07248	-0.03733	0.08915	0.01708	0.11173	-0.11173	0.65673	0.5595	0.78553	0.34612	0.62784	0.24625	0.8245	0.1457	0.1457	turquoise
HAGHL	0.13798	0.11867	-0.07099	-0.06552	0.16536	-0.13585	0.08156	-0.30349	0.30349	0.0719	0.12212	0.35615	0.39454	0.03067	0.07645	0.28894	5.46E-05	5.46E-05	red
TSPYL2	-0.02774	-0.19781	-0.17254	-0.10026	0.11358	-0.2486	-0.25546	0.39726	-0.39726	0.7187	0.0095	0.02403	0.19197	0.1391	0.00104	0.00075	7.46E-08	7.46E-08	brown
TGFB2	-0.01815	-0.25342	0.03939	0.15232	-0.26206	0.14195	0.16001	0.05938	-0.05938	0.81372	0.00082	0.60903	0.04672	0.00054	0.06402	0.03657	0.44039	0.44039	turquoise
PAM16	0.05702	0.02044	-0.02969	-0.06424	0.26392	-0.08006	0.08495	-0.37495	0.37495	0.45885	0.79071	0.69989	0.40389	0.00049	0.29793	0.26927	4.36E-07	4.36E-07	green
HERC4	0.00268	-0.17991	0.07868	0.04301	-0.1517	0.098	0.10716	-0.10736	0.10736	0.9723	0.01854	0.30634	0.57648	0.04762	0.20224	0.163	0.1622	0.1622	turquoise
VDAC3	-0.06568	-0.03325	-0.03025	0.05665	0.03867	-0.13168	0.12645	-0.02844	0.02844	0.39337	0.66598	0.69452	0.46176	0.61552	0.08601	0.09935	0.71194	0.71194	turquoise
ZNF503	-0.03157	-0.0049	-0.16028	0.06905	-0.02229	-0.08043	-0.03083	0.21375	-0.21375	0.68185	0.9493	0.03625	0.36954	0.77227	0.29569	0.68891	0.005	0.005	grey
RAD23B	-0.00163	-0.12031	-0.03861	-0.009	-0.13351	0.00509	0.02912	0.07618	-0.07618	0.98311	0.11703	0.61612	0.90694	0.0817	0.94735	0.70533	0.32203	0.32203	turquoise
SMIM7	0.03583	-0.06055	-0.09866	-0.0751	0.07446	-0.15596	-0.02032	-0.10972	0.10972	0.64176	0.43146	0.19922	0.32892	0.33311	0.04166	0.79191	0.15314	0.15314	grey
GNPDA2	-0.12504	-0.11764	-0.14016	-0.08464	-0.03475	-0.11285	-0.14113	0.40104	-0.40104	0.10321	0.12542	0.0675	0.27103	0.65184	0.14169	0.06558	5.46E-08	5.46E-08	turquoise
NDUFA7	0.03878	0.07235	-0.1814	-0.16034	0.21918	-0.29356	-0.03394	-0.30884	0.30884	0.61451	0.34702	0.01758	0.03618	0.00397	9.74E-05	0.65945	3.96E-05	3.96E-05	green
KIF3B	0.03443	-0.13222	-0.0943	-0.03695	0.04269	-0.06241	-0.03657	0.18411	-0.18411	0.65482	0.08473	0.21989	0.63134	0.57929	0.41741	0.63484	0.01593	0.01593	turquoise
DNM3	-0.07674	-0.11988	-0.05238	0.04517	-0.16546	0.05283	0.00679	0.48891	-0.48891	0.31846	0.11835	0.49625	0.55747	0.03056	0.49251	0.92981	1.16E-11	1.16E-11	blue
CAB39	-0.03387	-0.09673	0.07871	0.08771	-0.15267	0.18896	0.14219	-0.05719	0.05719	0.66007	0.2082	0.30615	0.25395	0.04621	0.01331	0.06357	0.45747	0.45747	turquoise
HIST1H3D	0.04999	-0.12778	0.07424	0.05503	-0.04845	0.1444	0.17883	-0.20573	0.20573	0.51613	0.0958	0.33454	0.47466	0.52912	0.05952	0.01927	0.00695	0.00695	grey
CSNK1E	-0.04756	-0.09842	-0.06713	-0.01784	-0.00665	-0.07873	0.04215	-0.19441	0.19441	0.53674	0.20029	0.38303	0.81684	0.93122	0.30603	0.58409	0.01083	0.01083	grey
ZNF668	-0.05104	-0.07639	-0.07133	-0.00969	-0.00676	0.04355	0.05676	0.21955	-0.21955	0.50738	0.32068	0.35386	0.89988	0.93008	0.57167	0.4609	0.00391	0.00391	black
ZNF704	-0.02431	-0.13427	-0.00996	0.0998	-0.17191	0.205	-0.04336	0.35838	-0.35838	0.75234	0.07997	0.89717	0.19405	0.02456	0.00715	0.57339	1.49E-06	1.49E-06	turquoise
GAL3ST1	-0.01986	-0.08353	-0.26413	-0.01273	0.32827	-0.20508	-0.12234	-0.18055	0.18055	0.79656	0.2774	0.00048	0.86876	1.17E-05	0.00713	0.11091	0.01812	0.01812	grey
XPO7	-0.07564	-0.08689	-0.00786	-0.00588	-0.08868	0.02855	0.08535	0.16334	-0.16334	0.32544	0.25843	0.91878	0.93913	0.24872	0.71093	0.26704	0.03279	0.03279	turquoise
TRERF1	0.05012	-0.0512	0.04714	0.09004	-0.105	0.05785	0.12372	-0.06748	0.06748	0.51504	0.50598	0.5404	0.24152	0.17168	0.45234	0.10693	0.38048	0.38048	turquoise
SCAND1	0.04613	-0.03209	-0.1502	-0.07331	0.23787	-0.17257	0.01903	-0.26399	0.26399	0.54907	0.67691	0.04989	0.34062	0.00173	0.024	0.80489	0.00048	0.00048	green
ZNF678	0.04304	-0.04907	-0.08695	-0.0156	-0.01491	0.01737	0.00773	0.17316	-0.17316	0.57615	0.52388	0.25813	0.83957	0.84654	0.82156	0.92005	0.02352	0.02352	turquoise
KIAA1324l	-0.0073	-0.04366	-0.04385	0.0201	-0.12755	-0.10628	-0.10276	0.4242	-0.4242	0.92451	0.57074	0.56904	0.79409	0.09641	0.16651	0.18108	7.39E-09	7.39E-09	turquoise
ATXN3	0.01588	-0.12333	-0.082	0.0805	-0.05087	-0.00372	0.02037	0.06845	-0.06845	0.83671	0.10803	0.28633	0.29527	0.50877	0.96152	0.79147	0.3737	0.3737	red
TM4SF20	-0.00919	-0.08606	0.07704	0.12629	0.12673	0.15019	0.12432	-0.15751	0.15751	0.90505	0.26305	0.31657	0.09978	0.0986	0.04991	0.10521	0.03964	0.03964	grey
CHRNA1	-0.09096	0.00366	0.04365	0.05751	-0.15918	0.02509	0.02345	0.33513	-0.33513	0.23673	0.96209	0.57078	0.45501	0.03757	0.74464	0.76078	7.44E-06	7.44E-06	blue
RAB40C	0.10591	0.03505	-0.04949	-0.04978	0.2106	0.00819	-0.00194	-0.31105	0.31105	0.16798	0.64902	0.52032	0.51794	0.0057	0.91528	0.97992	3.46E-05	3.46E-05	green
MAP10	-0.06557	-0.12984	-0.14034	-0.04569	0.00943	-0.06633	0.0493	0.0341	-0.0341	0.39418	0.09052	0.06713	0.55288	0.90253	0.38869	0.52192	0.65794	0.65794	turquoise
BCLAF1	-0.03936	-0.06217	-0.03239	-0.01779	-0.09357	0.00131	-0.07881	0.31396	-0.31396	0.60924	0.41917	0.67405	0.81735	0.22352	0.9864	0.30554	2.89E-05	2.89E-05	turquoise
RRP8	0.0473	-0.0908	-0.06842	-0.15458	0.09053	0.11896	0.05003	-0.13468	0.13468	0.53901	0.23757	0.3739	0.04351	0.23898	0.12122	0.51579	0.07905	0.07905	grey
KCNJ12	-0.08532	-0.04911	0.03213	0.19442	-0.13917	0.02652	0.01201	0.35822	-0.35822	0.26722	0.52352	0.67657	0.01083	0.06947	0.73057	0.87608	1.50E-06	1.50E-06	blue
YIPF4	0.02686	-0.13066	-0.08101	-0.04596	-0.00233	0.01728	0.02169	-0.04641	0.04641	0.72731	0.0885	0.29221	0.55055	0.97591	0.82246	0.77829	0.5467	0.5467	turquoise
RTL8A	0.01178	-0.16793	-0.15536	-0.0837	0.00899	-0.10552	0.04824	-0.27711	0.27711	0.87843	0.02813	0.04245	0.27644	0.90714	0.16959	0.53092	0.00024	0.00024	grey
KAZALD1	0.01167	0.02333	0.07421	0.03873	0.11753	0.07133	0.22666	-0.2626	0.2626	0.87954	0.76202	0.33472	0.61504	0.12579	0.3539	0.00287	0.00052	0.00052	grey
C1QL1	-0.03395	-0.07938	-0.2992	-0.12249	0.10909	-0.33413	-0.1398	0.33844	-0.33844	0.65934	0.30207	7.03E-05	0.11049	0.15551	7.96E-06	0.0682	5.97E-06	5.97E-06	brown
HCFC1	-0.02465	-0.09967	-0.09313	-0.04257	-0.0693	-0.07606	0.03551	0.11072	-0.11072	0.74896	0.19463	0.22571	0.5804	0.36775	0.32276	0.64472	0.14938	0.14938	turquoise
CRISP2	0.07408	-0.02189	-0.08852	0.02602	0.12937	0.00049	0.01493	-0.00217	0.00217	0.33558	0.77623	0.2496	0.73547	0.0917	0.99492	0.84629	0.97752	0.97752	grey
TNFSF9	0.04911	0.02546	0.06203	0.08498	-0.11294	0.05369	0.15557	-0.24471	0.24471	0.52358	0.74101	0.42025	0.26909	0.14135	0.48554	0.04218	0.00126	0.00126	grey
ETFBKMT	-0.11893	-0.15831	-0.07709	-0.06947	0.06806	-0.10979	-0.13409	0.27323	-0.27323	0.12132	0.03864	0.31623	0.3666	0.37643	0.15288	0.08039	0.0003	0.0003	turquoise
MDK	0.17839	-0.05203	-0.02351	0.0045	0.02861	0.0299	0.13656	-0.37917	0.37917	0.01958	0.4991	0.76023	0.95346	0.71033	0.69787	0.07491	3.15E-07	3.15E-07	grey
PDHX	0.00433	-0.12039	-0.01097	-0.0158	-0.02969	0.0072	0.0551	-0.019	0.019	0.95515	0.11677	0.88679	0.83747	0.69983	0.92551	0.47409	0.80514	0.80514	turquoise
LHFPL3	0.02878	-0.05005	0.07752	0.08657	0.20789	0.16004	0.05607	-0.11597	0.11597	0.70867	0.5156	0.31354	0.26021	0.00636	0.03654	0.46637	0.13093	0.13093	grey
HLA-E	-0.0248	0.08823	0.06446	0.06868	-0.12143	0.14503	0.12589	0.0278	-0.0278	0.74749	0.25114	0.40225	0.3721	0.11362	0.0584	0.10087	0.71813	0.71813	tan
PTK2	-0.01538	-0.117	-0.01876	0.01364	-0.08929	-0.04648	0.06438	-0.03921	0.03921	0.84177	0.12751	0.80761	0.85945	0.2455	0.54609	0.40283	0.61063	0.61063	turquoise
LSR	0.12406	-0.01644	0.04583	-0.01448	0.01274	0.12696	0.16055	-0.54818	0.54818	0.10597	0.83095	0.55169	0.85087	0.86863	0.09799	0.03593	8.46E-15	8.46E-15	yellow
ARCN1	-0.02922	-0.13165	0.00183	-0.03028	-0.16912	0.07366	0.0932	0.07976	-0.07976	0.70439	0.08608	0.98108	0.69417	0.02702	0.33831	0.22534	0.29973	0.29973	turquoise
CDK5	-0.04907	-0.09826	-0.10522	-0.06893	0.04482	-0.24724	0.05229	-0.13122	0.13122	0.5239	0.20102	0.17081	0.37036	0.56049	0.00111	0.49697	0.08714	0.08714	grey
HSBP1	-0.00506	-0.1536	-0.15776	-0.08547	0.00137	-0.09522	-0.00395	-0.12513	0.12513	0.9476	0.04488	0.03933	0.26636	0.98577	0.2154	0.95906	0.10296	0.10296	turquoise
LDOC1	-0.06838	-0.0842	-0.22524	-0.07359	0.04822	-0.29265	-0.25715	0.38267	-0.38267	0.3742	0.27355	0.00306	0.3388	0.53116	0.0001	0.00069	2.40E-07	2.40E-07	brown
ZNF25	-0.05322	-0.17674	-0.11116	-0.01879	-0.06354	-0.12372	-0.13011	0.53936	-0.53936	0.4894	0.02075	0.14777	0.80727	0.40903	0.10693	0.08986	2.72E-14	2.72E-14	turquoise
RIN3	-0.06666	-0.01633	0.01968	0.05033	-0.1641	0.13117	0.07992	0.18192	-0.18192	0.38637	0.8321	0.79835	0.51327	0.03198	0.08726	0.29877	0.01725	0.01725	blue
PDIA2	-0.02897	0.09242	0.07584	0.11935	-0.10493	0.04343	-0.01769	0.0756	-0.0756	0.70677	0.22928	0.32421	0.11999	0.17198	0.57278	0.81834	0.32575	0.32575	magenta
CNIH2	0.00267	-0.15632	-0.23652	-0.12258	0.1205	-0.35987	-0.1759	0.33261	-0.33261	0.97232	0.04118	0.00184	0.11021	0.11644	1.34E-06	0.02138	8.80E-06	8.80E-06	brown
BTBD6	0.05374	-0.12359	-0.16387	-0.03548	0.03802	-0.25279	-0.14912	0.28234	-0.28234	0.48512	0.10729	0.03222	0.64506	0.62148	0.00085	0.05159	0.00018	0.00018	grey
PPT1	-0.07364	-0.08722	-0.00347	0.05455	-0.24738	0.00556	-0.01741	0.39976	-0.39976	0.33844	0.25666	0.96407	0.47855	0.00111	0.94244	0.82123	6.07E-08	6.07E-08	turquoise
DDX24	0.03101	-0.16672	-0.09977	-0.02917	0.0195	-0.11366	-0.17591	0.24464	-0.24464	0.68723	0.0293	0.19415	0.70485	0.80019	0.1388	0.02137	0.00126	0.00126	turquoise
VASH1	-0.08723	-0.1774	-0.13487	-0.01155	-0.09671	-0.15353	-0.12294	0.54218	-0.54218	0.25661	0.02027	0.07862	0.8808	0.20826	0.04498	0.10918	1.88E-14	1.88E-14	blue
METTL25	-0.02432	0.01849	0.00726	-0.01935	0.09784	-0.00114	-0.02256	0.11919	-0.11919	0.75218	0.81026	0.92488	0.80162	0.20298	0.98824	0.76959	0.12049	0.12049	turquoise
CA13	0.05107	-0.02652	0.05454	0.01735	0.02434	0.12914	0.1117	-0.22068	0.22068	0.50712	0.73062	0.47862	0.82178	0.75198	0.09229	0.14579	0.00373	0.00373	turquoise
TAC3	0.05316	0.18578	0.0155	0.00509	0.11752	-0.01333	0.00463	-0.07497	0.07497	0.48984	0.01499	0.84054	0.94731	0.12581	0.86265	0.95204	0.32976	0.32976	grey
DNAAF4	-0.04772	0.01336	-0.19416	-0.10464	0.04271	-0.23023	-0.06373	-0.04626	0.04626	0.5354	0.86233	0.01094	0.17317	0.57914	0.00245	0.40759	0.54796	0.54796	grey
AP3B1	0.00061	-0.02628	-0.04636	-0.00449	-0.09186	0.02035	-0.00105	0.23151	-0.23151	0.99365	0.73291	0.54706	0.95349	0.23209	0.79165	0.98912	0.00231	0.00231	turquoise
GAL	0.03224	0.05229	-0.05849	0.11706	-0.06227	-0.03912	0.22126	0.05632	-0.05632	0.67553	0.49698	0.44732	0.12732	0.41848	0.61147	0.00363	0.4644	0.4644	grey
SEC24C	-0.04188	-0.14002	0.04217	0.0364	-0.10897	0.05914	0.01226	0.16682	-0.16682	0.58652	0.06775	0.58391	0.63646	0.15597	0.44228	0.8736	0.0292	0.0292	turquoise
CNNM3	-0.01113	-0.08887	-0.16992	-0.02157	0.03073	-0.08623	-0.09811	0.20508	-0.20508	0.88517	0.24775	0.02629	0.7795	0.68987	0.26212	0.20173	0.00713	0.00713	turquoise
MUC17	-0.02797	0.07073	0.13984	0.10671	0.05615	0.23468	0.12076	-0.22517	0.22517	0.7165	0.35792	0.06813	0.16479	0.4657	0.002	0.11565	0.00307	0.00307	grey
MRPL57	0.00443	-0.03156	-0.18374	-0.17294	0.22847	-0.19415	-0.01423	-0.22987	0.22987	0.95417	0.68202	0.01615	0.0237	0.00265	0.01094	0.85341	0.00249	0.00249	green
LPAR6	-0.16257	-0.0302	0.09101	0.11485	-0.06862	0.16369	0.17414	0.02554	-0.02554	0.03363	0.69494	0.23647	0.13472	0.37253	0.03241	0.02273	0.74024	0.74024	grey
CYREN	-0.05447	-0.11384	-0.04424	0.01202	-0.04767	-0.04123	0.0241	0.09672	-0.09672	0.47923	0.13819	0.56558	0.87596	0.53585	0.59237	0.75438	0.20823	0.20823	turquoise
METAP1	-0.09905	-0.07602	0.0808	0.04196	-0.08714	0.15754	0.1696	-0.19403	0.19403	0.19745	0.32304	0.29345	0.58579	0.2571	0.0396	0.02658	0.01099	0.01099	turquoise
CBLN1	-0.11365	-0.00854	-0.19919	-0.05293	0.03847	-0.1727	-0.18599	0.48164	-0.48164	0.13886	0.91177	0.009	0.49176	0.61743	0.0239	0.01487	2.57E-11	2.57E-11	grey
AMN1	-0.07184	-0.11472	-0.0666	-0.08322	-0.00552	-0.13612	-0.0822	0.05973	-0.05973	0.35046	0.13516	0.38678	0.27918	0.94288	0.07586	0.28517	0.43771	0.43771	turquoise
DDX28	-0.14739	0.06282	-0.127	-0.09509	-0.02387	-0.08019	0.09999	-0.02067	0.02067	0.05438	0.41437	0.09787	0.21604	0.7566	0.29711	0.19318	0.78841	0.78841	grey
TXNDC17	0.07139	0.03689	-0.04398	-0.06462	0.10712	-0.0933	0.22819	-0.36626	0.36626	0.35346	0.63193	0.56789	0.40109	0.16315	0.22486	0.00268	8.36E-07	8.36E-07	grey
CFL1	0.05035	0.02962	0.05647	-0.0117	-0.05618	0.12536	0.18369	-0.33979	0.33979	0.5131	0.70053	0.46323	0.87924	0.46552	0.10232	0.01617	5.45E-06	5.45E-06	yellow
BSG	-0.00249	-0.00551	-0.13793	-0.1154	0.20441	-0.21983	-0.02526	-0.27606	0.27606	0.97423	0.94298	0.07201	0.13285	0.00732	0.00386	0.74298	0.00026	0.00026	green
KHDRBS1	-0.02737	-0.08858	-0.00688	-0.03476	-0.12301	0.09095	0.08403	-0.01402	0.01402	0.7223	0.2493	0.92888	0.65173	0.10895	0.23678	0.27454	0.85563	0.85563	turquoise
FOXRED2	-0.06151	-0.01068	-0.14517	0.03055	-0.01314	-0.12621	0.04293	0.38749	-0.38749	0.42416	0.88977	0.05816	0.69162	0.86452	0.1	0.5772	1.64E-07	1.64E-07	turquoise
NBPF12	-0.05402	-0.17174	-0.12758	-0.05337	0.01458	-0.07318	-0.06626	0.23208	-0.23208	0.48285	0.0247	0.09632	0.4881	0.84992	0.34152	0.38923	0.00225	0.00225	red
ALDH1L2	-0.00018	0.00126	0.09431	0.1208	-0.24013	0.05886	0.0367	0.5201	-0.5201	0.99819	0.98694	0.21983	0.11553	0.00156	0.44446	0.63371	3.09E-13	3.09E-13	blue
ACE	0.00834	-0.1131	-0.1069	0.0087	0.17422	-0.08168	-0.06899	-0.02288	0.02288	0.91384	0.14079	0.16403	0.91008	0.02267	0.28824	0.36994	0.76644	0.76644	grey
VSIG10	0.05953	-0.13025	0.09027	0.07728	0.05819	0.1101	0.07644	-0.33809	0.33809	0.4393	0.0895	0.2403	0.31508	0.44966	0.1517	0.32037	6.11E-06	6.11E-06	grey
MKS1	-0.04096	-0.14184	-0.16066	-0.06134	0.10024	-0.24168	-0.04071	0.02437	-0.02437	0.59482	0.06423	0.03581	0.42545	0.19209	0.00145	0.597	0.75172	0.75172	grey
C9orf24	-0.01801	-0.11973	-0.18496	-0.04581	0.15712	-0.17921	-0.14484	-0.00266	0.00266	0.8151	0.11879	0.01544	0.55184	0.04014	0.01901	0.05874	0.9725	0.9725	grey
NAA40	-0.02932	-0.18236	-0.10336	-0.05618	0.07352	-0.12549	-0.02583	-0.0698	0.0698	0.7034	0.01698	0.17851	0.4655	0.33927	0.10196	0.73736	0.36433	0.36433	red
APCDD1	0.01565	-0.03431	-0.05169	0.08747	-0.0644	0.0516	0.12494	0.12442	-0.12442	0.83902	0.65595	0.50197	0.25529	0.40272	0.50266	0.10348	0.10493	0.10493	grey
DNASE2	0.00991	-0.06359	-0.07454	0.00809	-0.09785	-0.04522	0.01877	0.08907	-0.08907	0.8976	0.40866	0.3326	0.91632	0.20296	0.55699	0.8075	0.24667	0.24667	grey
ZNF160	-0.03315	-0.14545	-0.08849	0.04719	0.03947	0.01995	-0.0857	-0.0207	0.0207	0.66685	0.05768	0.24977	0.53993	0.60823	0.79563	0.26506	0.78811	0.78811	red
MDP1	-0.07296	-0.09732	-0.18725	-0.01944	0.22594	-0.27266	-0.12254	0.11222	-0.11222	0.34293	0.20539	0.01419	0.80072	0.00296	0.00031	0.11034	0.14391	0.14391	grey
PLEKHG6	0.0675	-0.09862	0.08993	0.02605	0.11363	0.09753	0.10035	-0.41226	0.41226	0.38037	0.19939	0.2421	0.73525	0.13894	0.20441	0.1916	2.11E-08	2.11E-08	grey
FDXR	0.00706	-0.00352	-0.02398	-0.04125	0.09473	-0.13918	0.13194	-0.21567	0.21567	0.92699	0.96353	0.75551	0.59214	0.21779	0.06945	0.08539	0.00461	0.00461	grey
HAUS6	-0.00512	-0.07779	-0.0799	-0.0414	-0.06139	-0.01582	0.08378	0.16026	-0.16026	0.94699	0.31189	0.29892	0.59085	0.42509	0.83727	0.27598	0.03627	0.03627	turquoise
METTL11E	-0.0119	0.09961	0.028	-0.00955	-0.12921	0.02425	0.07819	-0.06303	0.06303	0.87726	0.19491	0.71618	0.90136	0.09211	0.75289	0.30938	0.4128	0.4128	black
NSL1	-0.09518	-0.07405	-0.11244	-0.0544	0.00123	-0.03701	-0.03878	0.27781	-0.27781	0.2156	0.33579	0.14315	0.47975	0.9873	0.63079	0.61454	0.00023	0.00023	turquoise
RPL19	0.08449	0.1662	-0.03493	-0.04179	0.12328	-0.02257	0.03116	-0.18993	0.18993	0.27189	0.02982	0.65017	0.58734	0.10819	0.76953	0.68574	0.01284	0.01284	purple
CPSF1	0.03036	0.01348	-0.01038	-0.07943	0.09737	-0.09899	0.07057	-0.24983	0.24983	0.69344	0.86112	0.89277	0.30172	0.20517	0.19771	0.35906	0.00098	0.00098	green
KRTCAP2	0.04376	0.03034	-0.05897	-0.08586	0.31365	-0.13615	0.0371	-0.40277	0.40277	0.56983	0.69359	0.44357	0.26419	2.95E-05	0.07579	0.62999	4.72E-08	4.72E-08	green
HDGF	0.09985	0.03145	0.00932	-0.04696	-0.10403	0.12912	0.27312	-0.42866	0.42866	0.19379	0.68301	0.90367	0.54194	0.17573	0.09235	0.0003	4.94E-09	4.94E-09	greenyellow
TSLP	-0.08839	-0.17036	-0.03571	-0.07406	0.04297	0.19992	-0.10281	0.03785	-0.03785	0.25028	0.0259	0.64286	0.33571	0.5768	0.00875	0.18088	0.62308	0.62308	grey
PPARGC1I	0.03678	0.00595	0.00181	0.0402	-0.03592	0.02793	0.15262	0.11608	-0.11608	0.63298	0.93845	0.98129	0.60162	0.64094	0.71689	0.04628	0.13054	0.13054	turquoise
WDR18	0.08103	0.10904	-0.16496	-0.11047	0.22891	-0.16207	0.01692	-0.25803	0.25803	0.29209	0.1557	0.03108	0.15033	0.0026	0.03419	0.82614	0.00066	0.00066	green
SLC16A12	-0.09395	-0.10324	-0.18319	-0.06801	-0.03621	-0.22447	-0.14473	0.49555	-0.49555	0.22159	0.17904	0.01647	0.37677	0.63821	0.00316	0.05893	5.54E-12	5.54E-12	brown
ZFP62	0.08612	-0.1356	-0.11587	-0.0884	0.07556	-0.00819	0.0228	-0.13693	0.13693	0.26274	0.07701	0.13126	0.25023	0.32597	0.91528	0.76723	0.07411	0.07411	turquoise
UPK1B	0.10144	-0.14454	0.0871	0.07838	0.02502	0.12333	0.08474	0.04384	-0.04384	0.18678	0.05928	0.25729	0.30822	0.74528	0.10804	0.27045	0.5691	0.5691	grey
ZNRD1	0.11125	0.06357	0.02051	-0.11207	0.05591	0.06652	0.1152	-0.38146	0.38146	0.14743	0.40881	0.79003	0.14446	0.46764	0.38735	0.13352	2.64E-07	2.64E-07	grey
EEF2KMT	0.00801	0.14164	-0.03277	-0.00959	-0.08974	0.00371	0.16884	-0.11652	0.11652	0.91717	0.06461	0.67051	0.90097	0.2431	0.96161	0.02727	0.12908	0.12908	turquoise
PLAGL1	-0.09757	-0.09872	-0.0269	0.07583	-0.02226	-0.04589	-0.06541	0.44602	-0.44602	0.20424	0.19895	0.72689	0.32424	0.77256	0.55118	0.39531	9.74E-10	9.74E-10	turquoise
LAMTOR3	-0.11197	-0.1583	-0.09245	-0.0101	0.05361	-0.02112	-0.06017	0.0208	-0.0208	0.14482	0.03865	0.22913	0.89574	0.48616	0.78391	0.43437	0.78709	0.78709	turquoise
ARRB2	-0.07535	-0.03968	0.00824	0.03275	-0.12612	0.01225	0.02041	0.45409	-0.45409	0.32736	0.60633	0.91479	0.67068	0.10023	0.8737	0.79105	4.43E-10	4.43E-10	blue
PXMP4	#######	-0.03598	-0.19672	-0.06954	0.07239	-0.16034	0.00048	0.02348	-0.02348	0.99936	0.64035	0.00991	0.36612	0.34678	0.03618	0.995	0.76053	0.76053	turquoise
EGLN3	-0.06869	-0.11255	0.05025	0.09569	0.11818	0.12528	0.02059	-0.43287	0.43287	0.37204	0.14273	0.51399	0.21312	0.12368	0.10255	0.78919	3.36E-09	3.36E-09	grey
FREM1	-0.02805	-0.11408	-0.01656	0.03926	-0.12718	0.15734	-0.07087	0.54	-0.54	0.71575	0.13735	0.8298	0.61014	0.09739	0.03986	0.35701	2.50E-14	2.50E-14	blue

MED7	-0.04198	-0.14982	-0.14335	-0.07391	0.04536	-0.05207	0.00409	-0.01036	0.01036	0.58562	0.05048	0.06142	0.33672	0.55575	0.4988	0.9577	0.89301	0.89301	turquoise
ZMIZ1	-0.03344	-0.16953	-0.06724	0.02295	-0.05144	-0.04278	-0.11513	0.3749	-0.3749	0.66415	0.02664	0.38219	0.76575	0.50406	0.5785	0.13375	4.37E-07	4.37E-07	turquoise
NRXN2	0.00068	-0.12568	-0.13494	-0.05344	-0.0973	#######	-0.02164	0.41697	-0.41697	0.99291	0.10144	0.07846	0.4876	0.20548	0.99928	0.77872	1.40E-08	1.40E-08	grey
ZNF250	-0.00947	-0.1878	-0.12075	-0.0163	-0.06834	-0.06468	-0.04315	0.05215	-0.05215	0.90217	0.0139	0.11566	0.83247	0.37445	0.40064	0.57522	0.49811	0.49811	turquoise
TAZ	0.02024	-0.05899	-0.01481	-0.07192	0.08477	0.01984	0.07946	-0.3621	0.3621	0.79269	0.44347	0.84756	0.34989	0.27029	0.79768	0.30155	1.14E-06	1.14E-06	red
MYCBP	0.01846	-0.00426	0.055	0.0272	-0.00882	0.08868	0.03539	-0.23166	0.23166	0.81064	0.95588	0.4749	0.72395	0.9088	0.24878	0.64583	0.0023	0.0023	turquoise
C17orf97	-0.0253	-0.09321	-0.185	-0.12929	0.12595	-0.11518	-0.08489	0.11399	-0.11399	0.74255	0.22531	0.01542	0.09192	0.10071	0.13359	0.26964	0.13766	0.13766	grey
CLUAP1	0.04239	0.00851	-0.01811	-0.0256	-0.01674	-0.06814	-0.01239	0.14869	-0.14869	0.58198	0.91206	0.14181	0.73959	0.82796	0.37588	0.87217	0.05227	0.05227	turquoise
CASP6	-0.06185	0.00541	0.09254	0.06033	-0.01597	0.21213	0.16808	-0.36382	0.36382	0.42158	0.94405	0.22864	0.4331	0.83578	0.00535	0.02798	1.00E-06	1.00E-06	turquoise
BCKDK	-0.04012	-0.028	0.04749	0.09605	-0.10694	0.13223	0.19764	-0.24997	0.24997	0.60239	0.7162	0.53736	0.2114	0.16388	0.08471	0.00957	0.00098	0.00098	grey
TMEM255	-0.0908	-0.01067	-0.07525	0.05932	-0.07465	-0.05407	-0.00175	0.36631	-0.36631	0.23757	0.88988	0.32799	0.44087	0.33188	0.48241	0.98189	8.34E-07	8.34E-07	blue
ARHGEF38	0.02055	-0.05837	0.02203	0.08882	0.14024	0.17955	0.00094	-0.21869	0.21869	0.78965	0.44829	0.77486	0.24798	0.06733	0.01878	0.99025	0.00406	0.00406	grey
PPP2R5B	-0.0307	-0.24917	-0.07926	-0.06496	0.08407	-0.14049	-0.12372	-0.00751	0.00751	0.69017	0.00101	0.30279	0.39857	0.27429	0.06683	0.10693	0.92232	0.92232	grey
FBXL15	0.07772	0.01021	-0.17892	-0.12906	0.25427	-0.2187	0.0337	-0.19238	0.19238	0.31233	0.89459	0.01921	0.09251	0.00079	0.00406	0.66168	0.01171	0.01171	green
UBFD1	0.02307	-0.20574	-0.09361	0.00723	-0.12877	-0.03331	0.02687	0.08081	-0.08081	0.76456	0.00694	0.22329	0.92523	0.09325	0.66535	0.72721	0.29339	0.29339	turquoise
TCAIM	0.05128	-0.07459	0.02374	-0.05369	-0.06031	0.14112	0.12065	-0.15649	0.15649	0.50534	0.33223	0.75794	0.48549	0.43325	0.06561	0.11599	0.04095	0.04095	turquoise
CSRNP3	0.00897	-0.12793	-0.17894	-0.07102	-0.02856	-0.16518	-0.08294	0.55607	-0.55607	0.90729	0.09541	0.0192	0.35594	0.71079	0.03085	0.28082	2.89E-15	2.89E-15	brown
CHN1	-0.12381	-0.0413	-0.07639	-0.03998	-0.06314	-0.17068	-0.09299	0.49697	-0.49697	0.10667	0.59176	0.32071	0.60365	0.41197	0.02562	0.22637	4.72E-12	4.72E-12	blue
TMEM11	-0.05289	-0.02555	-0.09404	-0.04472	0.00451	-0.13889	-0.02016	-0.02794	0.02794	0.49208	0.74008	0.22119	0.5614	0.95332	0.07004	0.79358	0.71678	0.71678	grey
MTX2	0.01893	0.02139	-0.05463	-0.00139	0.08202	-0.0489	0.07563	-0.27948	0.27948	0.80592	0.78129	0.4779	0.98562	0.28621	0.52536	0.32554	0.00021	0.00021	turquoise
PCGF3	-0.07454	-0.09722	-0.13517	-0.04557	0.21727	-0.11916	-0.02522	-0.09716	0.09716	0.3326	0.20588	0.07794	0.55396	0.00431	0.12059	0.74336	0.20617	0.20617	red
YBX1	-0.01217	0.04664	0.11428	0.07501	-0.1348	0.13904	0.08873	-0.01548	0.01548	0.87444	0.54472	0.13668	0.32955	0.07878	0.06972	0.24847	0.84078	0.84078	turquoise
GSDMC	-0.05831	-0.11255	0.02213	-0.00471	-0.1824	-0.03434	0.24549	-0.11901	0.11901	0.4487	0.14276	0.77391	0.95128	0.01695	0.65569	0.00121	0.12107	0.12107	grey
LY6G5C	-0.0975	-0.02853	-0.21866	-0.00524	0.16921	-0.1339	-0.20588	0.20889	-0.20889	0.20458	0.71112	0.00406	0.94577	0.02693	0.08082	0.0069	0.00611	0.00611	grey
SLC15A1	0.01593	-0.07026	-0.09623	0.0942	0.01253	0.01492	0.07028	-0.14479	0.14479	0.83621	0.36114	0.21056	0.22039	0.87082	0.8464	0.361	0.05882	0.05882	grey
NAPG	0.02346	-0.15725	0.03345	0.01152	0.02502	0.04945	0.13034	-0.07983	0.07983	0.76073	0.03997	0.66409	0.88109	0.74529	0.52065	0.08929	0.29933	0.29933	turquoise
KLHL12	0.04621	-0.01021	-0.06844	-0.05318	-0.02102	0.02715	0.13558	-0.07463	0.07463	0.5484	0.89452	0.37374	0.48969	0.7849	0.72447	0.07705	0.33202	0.33202	turquoise
ITGA6	0.00717	0.03208	0.11067	0.04897	-0.16312	0.23311	0.23517	-0.29535	0.29535	0.92586	0.67699	0.14958	0.52475	0.03303	0.00215	0.00196	8.79E-05	8.79E-05	turquoise
ANGPTL2	-0.10181	-0.07746	0.03102	0.12015	-0.18366	0.11559	0.14819	0.20849	-0.20849	0.18514	0.31392	0.68713	0.1175	0.01619	0.13219	0.05308	0.00621	0.00621	black
ZNF32	-0.01854	-0.05763	-0.13406	-0.05276	0.03699	-0.15637	-0.04363	0.10321	-0.10321	0.80976	0.454	0.08044	0.49314	0.63096	0.04112	0.57097	0.17918	0.17918	grey
B3GALNT1	-0.07643	-0.13468	-0.0049	0.03169	-0.19806	0.02975	0.06135	0.26355	-0.26355	0.32044	0.07904	0.94924	0.68076	0.00941	0.69928	0.42536	0.0005	0.0005	turquoise
RFX5	-0.11187	-0.00199	-0.039	-0.06217	-0.1299	0.06799	0.03868	0.16611	-0.16611	0.14519	0.97937	0.61256	0.41923	0.09038	0.37689	0.61549	0.0299	0.0299	turquoise
BMERB1	-0.03279	-0.17226	-0.09207	-0.0258	-0.03485	-0.12311	-0.16554	0.23981	-0.23981	0.67033	0.02426	0.23105	0.73767	0.65092	0.10868	0.03048	0.00158	0.00158	turquoise
PDIK1L	-0.05454	-0.11703	-0.05184	0.00326	-0.03837	0.03221	-0.05225	0.22871	-0.22871	0.4786	0.12741	0.50071	0.96626	0.61833	0.67578	0.49733	0.00262	0.00262	turquoise
DNAJC21	-0.00509	-0.11202	0.00628	-0.03243	-0.08766	0.04069	0.07964	0.06103	-0.06103	0.94732	0.14465	0.93499	0.67366	0.25427	0.5972	0.30047	0.42784	0.42784	turquoise
CDC34	0.11169	0.02942	-0.11451	-0.05147	0.20003	-0.11263	0.08106	-0.38105	0.38105	0.14584	0.70251	0.13586	0.50375	0.00871	0.14246	0.2919	2.72E-07	2.72E-07	green
R3HCC1	-0.04839	0.01645	-0.17306	-0.08634	0.10478	-0.22474	-0.04403	0.19502	-0.19502	0.52967	0.83087	0.0236	0.26152	0.17259	0.00312	0.56747	0.01059	0.01059	grey
OXA1L	0.02279	0.01245	0.04411	-0.00675	-0.05115	0.13099	0.10719	-0.1453	0.1453	0.76734	0.87166	0.56675	0.93022	0.5064	0.0877	0.16288	0.05794	0.05794	turquoise
ODR4	0.04528	-0.03466	-0.0454	-0.0824	0.09268	0.03138	0.04808	-0.14624	0.14624	0.55648	0.65263	0.55543	0.28395	0.22793	0.68365	0.53232	0.05632	0.05632	turquoise
PARD6B	0.13741	-0.06	0.07638	0.07677	0.02907	0.17908	0.06958	-0.4228	0.4228	0.0731	0.43563	0.32078	0.31825	0.70581	0.0191	0.36581	8.38E-09	8.38E-09	yellow
RGS17	0.10448	-0.23661	-0.15232	0.02645	0.04987	-0.2251	-0.07548	0.38971	-0.38971	0.17382	0.00183	0.04671	0.73125	0.51715	0.00308	0.3265	1.38E-07	1.38E-07	brown
MTMR2	-0.03844	-0.11948	0.04301	0.04634	-0.17211	0.12638	0.06372	0.09227	-0.09227	0.61763	0.1196	0.57649	0.5473	0.02439	0.09953	0.40767	0.23003	0.23003	turquoise
NAXD	0.02013	-0.02172	-0.18152	-0.14019	0.28441	-0.26309	-0.19221	0.04542	-0.04542	0.79379	0.77793	0.0175	0.06742	0.00016	0.00051	0.01178	0.55523	0.55523	grey
NPEPL1	0.02371	0.02094	-0.02599	0.00444	0.20336	-0.12237	0.10306	-0.22907	0.22907	0.75818	0.78573	0.73581	0.95406	0.00764	0.11082	0.17978	0.00258	0.00258	red
RNF11	-0.06862	-0.19486	-0.01083	0.04261	-0.08519	0.01061	-0.09543	0.31165	-0.31165	0.37249	0.01065	0.88823	0.58005	0.26794	0.89045	0.21441	3.34E-05	3.34E-05	turquoise
ERVMER34	0.03605	0.03878	0.01862	0.08603	-0.09114	0.14322	0.29842	-0.28129	0.28129	0.63967	0.61454	0.80903	0.2632	0.23581	0.06166	7.36E-05	0.00019	0.00019	grey
BCAS1	0.0372	0.02653	0.15714	0.06815	0.04339	0.29305	0.13166	-0.29059	0.29059	0.6291	0.73055	0.04012	0.37578	0.57312	0.0001	0.08606	0.00012	0.00012	yellow
TANC1	-0.05122	-0.02824	0.06699	0.10507	-0.13237	0.22437	0.13235	-0.06671	0.06671	0.50581	0.71389	0.384	0.17141	0.08437	0.00318	0.08441	0.386	0.386	turquoise
CGN	0.06332	-0.00448	0.03138	-0.03647	0.05395	0.2578	0.12158	-0.43868	0.43868	0.41063	0.9536	0.68373	0.6358	0.48343	0.00066	0.11317	1.96E-09	1.96E-09	yellow
SNF8	0.04795	-0.03664	-0.15576	-0.05597	0.10053	-0.17154	0.0494	-0.20137	0.20137	0.5334	0.63425	0.04192	0.46719	0.19079	0.02487	0.52109	0.00826	0.00826	grey
FAM83F	0.02979	-0.0289	-0.23659	-0.11402	0.25336	-0.29164	-0.20227	-0.0796	0.0796	0.69891	0.70748	0.00184	0.13758	0.00083	0.00011	0.00798	0.30068	0.30068	grey
TNF	-0.18244	-0.05646	0.05451	0.01747	-0.10873	0.0254	-0.02382	0.12435	-0.12435	0.01693	0.46323	0.47888	0.82055	0.1569	0.74158	0.75718	0.10514	0.10514	grey
SLC7A7	-0.03996	0.05858	0.1328	0.14924	-0.13263	0.24856	0.15194	-0.05609	0.05609	0.60381	0.44665	0.08335	0.05139	0.08376	0.00105	0.04728	0.4662	0.4662	turquoise
CLPX	-0.09364	-0.08379	-0.05273	0.00744	-0.0791	0.05843	0.0524	0.11187	-0.11187	0.22317	0.27589	0.4934	0.92304	0.30374	0.44775	0.49608	0.14518	0.14518	turquoise
AGT	-0.04555	-0.16972	-0.26744	-0.04787	0.09774	-0.17059	-0.12685	0.2329	-0.2329	0.55416	0.02647	0.00041	0.53412	0.20345	0.0257	0.09828	0.00217	0.00217	grey
SLC27A2	-0.00143	0.05752	-0.05599	-0.03233	0.18537	0.03471	-0.04144	-0.22623	0.22623	0.9852	0.4549	0.46703	0.67468	0.01521	0.65225	0.59045	0.00293	0.00293	grey
ACE2	0.04303	-0.08806	-0.02101	-0.00852	0.0914	0.05678	0.02703	0.10079	-0.10079	0.57626	0.2521	0.78501	0.91192	0.23447	0.4607	0.72564	0.18961	0.18961	grey
GTPBP4	0.03144	-0.04211	0.07309	-0.02416	-0.14621	0.13544	0.1385	-0.08236	0.08236	0.68316	0.58448	0.34206	0.75375	0.05636	0.07736	0.07084	0.28422	0.28422	turquoise
CEP192	-0.06816	-0.14168	0.04928	0.0843	-0.00555	0.07707	0.05191	0.14689	-0.14689	0.37575	0.06454	0.52211	0.273	0.94259	0.31639	0.50009	0.05522	0.05522	turquoise
ACACA	-0.1079	-0.16237	-0.06672	-0.03866	0.02802	-0.09907	-0.01102	0.08794	-0.08794	0.1601	0.03385	0.38594	0.61568	0.71598	0.19732	0.88622	0.25275	0.25275	turquoise
IMMP2L	0.01532	-0.04877	-0.08515	-0.0306	0.04937	-0.09745	-0.0751	0.09263	-0.09263	0.84238	0.52642	0.26816	0.6911	0.52136	0.20481	0.32897	0.22819	0.22819	grey
SCN9A	-0.00208	-0.15851	-0.08468	0.01957	-0.08447	-0.00058	-0.0736	0.29526	-0.29526	0.97847	0.03838	0.27084	0.79945	0.27203	0.99403	0.33875	8.84E-05	8.84E-05	grey
CLEC3B	-0.17566	-0.07679	-0.13577	-0.00713	0.03916	-0.03334	-0.1728	0.47405	-0.47405	0.02156	0.31816	0.07663	0.92622	0.61104	0.66507	0.02382	5.78E-11	5.78E-11	blue
KCTD17	-0.15791	-0.06781	-0.11576	0.0102	-0.06169	-0.1401	-0.0372	0.21916	-0.21916	0.03914	0.37816	0.13161	0.8947	0.4228	0.0676	0.62902	0.00398	0.00398	grey
CCNB1IP1	0.02878	-0.04521	-0.09357	-0.07328	0.03677	-0.03398	-0.04132	-0.17649	0.17649	0.70867	0.55711	0.22351	0.34085	0.63299	0.65907	0.59154	0.02094	0.02094	grey
CLEC7A	-0.14934	-0.02291	0.0991	0.08379	-0.22407	0.15473	0.11378	0.37269	-0.37269	0.05123	0.76619	0.19722	0.27588	0.00322	0.04331	0.13841	5.17E-07	5.17E-07	blue
MMADHC	0.01598	0.03436	0.02592	0.05332	-0.14574	0.07677	0.12052	-0.07543	0.07543	0.83569	0.65544	0.73652	0.48851	0.05717	0.31831	0.11638	0.32684	0.32684	turquoise
CYP4V2	-0.09915	-0.12952	-0.16023	0.04756	0.06851	-0.03704	-0.1057	0.27455	-0.27455	0.19699	0.09133	0.0363	0.53674	0.37328	0.63055	0.16884	0.00028	0.00028	grey
MOB4	-0.04892	-0.05934	-0.02388	0.03336	0.00663	0.006	-0.02707	0.01085	-0.01085	0.52518	0.44077	0.75652	0.66493	0.93141	0.93788	0.7252	0.88803	0.88803	turquoise
TTC23	-0.01108	-0.11321	-0.10481	0.0557	-0.09702	0.09682	0.10557	0.04295	-0.04295	0.88566	0.14041	0.17246	0.46935	0.2068	0.20778	0.16939	0.57703	0.57703	turquoise
ATP8B3	-0.05665	-0.08595	-0.02873	0.09237	-0.0957	-0.04905	0.09547	-0.18366	0.18366	0.46172	0.26367	0.70916	0.22949	0.21306	0.52404	0.21421	0.01619	0.01619	grey
ZC3H7A	-0.07557	-0.10574	-0.04177	0.02273	-0.02053	0.03911	-0.11248	0.20083	-0.20083	0.32592	0.16867	0.58754	0.76788	0.78979	0.61154	0.14301	0.00844	0.00844	turquoise
EPC2	-0.0291	-0.13934	-0.07568	0.02919	-0.12218	-0.00197	-0.06933	0.39156	-0.39156	0.70559	0.06912	0.32522	0.70467	0.1114	0.97964	0.36756	1.18E-07	1.18E-07	turquoise
PUS3	-0.05471	-0.05274	-0.1063	-0.10431	-0.09287	0.01984	0.05725	0.0586	-0.0586	0.47729	0.49325	0.16644	0.17456	0.22698	0.79679	0.45704	0.4465	0.4465	turquoise
TBC1D2	0.07938	-0.00196	0.05651	0.02179	-0.09693	0.10719	0.20743	-0.22906	0.22906	0.30203	0.9797	0.46289	0.77723	0.20723	0.16289	0.00648	0.00258	0.00258	turquoise
SULF1	-0.08693	-0.06315	0.09043	0.08911	-0.23064	0.20661	0.08001	0.16803	-0.16803	0.25825	0.41189	0.23948	0.24642	0.00241	0.0067	0.29822	0.02803	0.02803	black
SELENOH	0.05886	0.02931	-0.0633	-0.09694	0.12031	-0.06162	0.19404	-0.37521	0.37521	0.44443	0.7035	0.41075	0.20719	0.117	0.42332	0.01099	4.27E-07	4.27E-07	green
DCTN4	0.01431	-0.18455	-0.12343	-0.03901	0.00156	-0.15175	-0.134	0.29002	-0.29002	0.8526	0.01567	0.10775	0.61241	0.98386	0.04755	0.08058	0.00012	0.00012	turquoise
ERGIC3	0.14069	-0.03514	0.03337	0.02035	0.06405	-0.05429	0.12819	-0.34149	0.34149	0.06644	0.64817	0.66484	0.79164	0.40527	0.48063	0.09474	4.85E-06	4.85E-06	grey
RBCK1	0.13837	-0.02611	-0.16564	-0.08817	0.15913	-0.1694	0.03563	-0.32498	0.32498	0.07109	0.73459	0.03038	0.25147	0.03762	0.02676	0.63817	1.45E-05	1.45E-05	green
TMEM248	0.03413	-0.18571	-0.02884	0.01659	0.01697	0.02394	0.00211	-0.00625	0.00625	0.65766	0.01502	0.70807	0.82951	0.82565	0.75594	0.97814	0.93534	0.93534	turquoise
ANKRD36	-0.01027	-0.10201	0.04629	0.03418	-0.04264	0.08736	0.06673	0.02727	-0.02727	0.89399	0.18431	0.54773	0.65716	0.57976	0.25589	0.38582	0.72329	0.72329	red
TBC1D4	-0.07606	-0.01173	0.03711	0.10446	-0.20668	0.22245	0.09693	0.3562	-0.3562	0.32277	0.87896	0.62987	0.17392	0.00668	0.00345	0.20722	1.74E-06	1.74E-06	blue
MOB3C	-0.06514	-0.23838	0.02342	0.06772	-0.0743	0.09796	0.02213	0.27907	-0.27907	0.39727	0.00169	0.76113	0.37882	0.33416	0.20244	0.77384	0.00022	0.00022	turquoise
GPR183	-0.13424	-0.08079	0.09622	0.0719	-0.20631	0.19621	0.04372	0.38481	-0.38481	0.08004	0.29351	0.21061	0.35006	0.00678	0.01011	0.57022	2.03E-07	2.03E-07	blue
STARD9	-0.08685	-0.06325	-0.055	0.04936	-0.06813	-0.00809	0.01485	0.49247	-0.49247	0.25868	0.41119	0.47493	0.52147	0.37595	0.91636	0.84711	7.83E-12	7.83E-12	salmon
ZFP90	0.00322	-0.14682	-0.09369	-0.02123	-0.1098	0.00396	0.00711	0.2886	-0.2886	0.96668	0.05533	0.22292	0.78286	0.15282	0.95899	0.92651	0.00013	0.00013	turquoise
TVP23B	-0.08661	-0.12365	-0.00572	-0.01341	-0.01227	-0.11795	-0.08985	0.07799	-0.07799	0.26003	0.10712	0.94084	0.86176	0.87343	0.12443	0.24251	0.3106	0.3106	turquoise
KPNA3	-0.01015	-0.07017	-0.00041	0.02409	-0.16189	0.01627	0.02321	0.1978	-0.1978	0.89514	0.3618	0.99576	0.75444	0.0344	0.83272	0.76319	0.00951	0.00951	turquoise
UEVLD	-0.03564	-0.12327	0.01808	0.01592	-0.09345	0.09414	0.07236	0.06057	-0.06057	0.64352	0.10821	0.81439	0.83627	0.22408	0.22069	0.34694	0.43133	0.43133	turquoise
LRFN3	-0.03623	-0.15328	-0.15803	-0.08979	0.05006	-0.16005	0.02101	0.11411	-0.11411	0.63805	0.04534	0.03899	0.24285	0.51551	0.03652	0.78505	0.13726	0.13726	grey
5-Mar	-0.02535	-0.0911	0.03619	0.05235	-0.14117	0.06739	0.12883	-0.07339	0.07339	0.74203	0.23601	0.63844	0.49653	0.06551	0.38115	0.09309	0.34012	0.34012	turquoise
ARRDC2	0.06636	-0.10043	-0.03011	0.04651	0.00903	0.04213	0.06352	-0.25409	0.25409	0.38847	0.1912	0.69589	0.54578	0.90666	0.58425	0.40915	0.0008	0.0008	grey
ASXL2	-0.05418	-0.11277	-0.00801	0.02322	-0.15599	0.12586	0.07911	0.22271	-0.22271	0.48157	0.14196	0.9172	0.76307	0.04162	0.10094	0.30373	0.00341	0.00341	turquoise
FZD1	-0.07475	-0.07573	0.00996	0.10045	-0.11834	0.02571	0.02017	0.36026	-0.36026	0.33119	0.32489	0.89713	0.19113	0.12317	0.7385	0.7934	1.30E-06	1.30E-06	black
RGS4	-0.02137	-0.25046	-0.15309	-0.09588	-0.03207	-0.20482	-0.14674	0.47174	-0.47174	0.78146	0.00095	0.04561	0.21223	0.6771	0.0072	0.05546	7.37E-11	7.37E-11	brown
MFSD8	-0.07644	-0.16861	0.00548	0.01889	0.0959	-0.05118	-0.10268	0.07819	-0.07819	0.32037	0.02749	0.94328	0.80628	0.21212	0.50617	0.18143	0.30938	0.30938	turquoise
GPR68	-0.00353	-0.01185	0.04469	0.07596	-0.19727	0.13713	0.09866	0.28458	-0.28458	0.96342	0.87774	0.56167	0.32341	0.0097	0.0737	0.19919	0.00016	0.00016	black
POFUT1	-0.04709	-0.08968	-0.03865	0.06241	-0.07511	-0.03241	0.01622	0.20561	-0.20561	0.54076	0.24343	0.61578	0.41739	0.32887	0.67391	0.83323	0.00698	0.00698	turquoise
KIFC1	-0.0281	0.15599	0.02422	-0.02686	-0.01416	0.1062	0.33061	-0.43657	0.43657	0.71522	0.04161	0.75322	0.7273	0.85413	0.16681	1.00E-05	2.38E-09	2.38E-09	pink
ZFYVE21	0.05888	-0.05592	0.04281	0.09338	0.02349	0.08163	0.11296	-0.28363	0.28363	0.44431	0.46754	0.57824	0.22446	0.76039	0.28853	0.1413	0.00017	0.00017	turquoise
RMND1	-0.01521	0.04749	0.0459	-0.01874	-0.04954	0.05548	0.10946	0.0245	-0.0245	0.84347	0.53735	0.55106	0.80782	0.5199	0.47104	0.15412	0.75038	0.75038	grey
MCAM	-0.15539	-0.08114	-0.07839	0.01703	-0.09092	-0.01372	-0.1098	0.36594	-0.36594	0.04242	0.29142	0.30816	0.82499	0.23692	0.85862	0.15284	8.57E-07	8.57E-07	blue
ARNT	1.90E-05	-0.13464	0.0071	0.03315	-0.1483	0.17283	0.08963	0.23276	-0.23276	0.9998	0.07913	0.9266	0.6669	0.05289	0.02379	0.2437	0.00219	0.00219	turquoise
ARHGDIB	-0.14463	-0.06608	0.08381	0.06039	-0.20693	0.13549	0.02058	0.17479	-0.17479	0.05912	0.39053	0.27576	0.4327	0.00662	0.07723	0.7893	0.02222	0.02222	blue
TMEM270	0.00976	-0.09556	-0.09759	0.00076	0.04513	-0.17459	-0.02441	-0.22324	0.22324	0.89923	0.21376	0.2128	0.99209	0.55779	0.02238	0.75133	0.00334	0.00334	grey
PAPOLG	-0.09267	-0.16004	-0.00986	0.0616	-0.19686	0.06049	-0.00446	0.27285	-0.27285	0.22799	0.03653	0.89813	0.42351	0.00986	0.43194	0.95388	0.00031	0.00031	turquoise
FAM177A1	-0.01973	-0.08969	0.0586	0.02775	0.01495	0.10272	-0.0425	-0.14883	0.14883	0.79787	0.24339	0.44646	0.71862	0.84614	0.18124	0.58103	0.05205	0.05205	turquoise
TCEAL7	-0.08904	-0.17655	-0.10046	-0.03078	-0.08099	-0.00303	-0.08091	0.41855	-0.41855	0.2468	0.02089	0.19111	0.68945	0.2923	0.96859	0.2928	1.22E-08	1.22E-08	blue
FRS2	0.02322	-0.2348	-0.05071	0.09713	-0.05586	-0.02488	-0.10158	0.27558	-0.27558	0.76309	0.00199	0.51011	0.2063	0.46888	0.74668	0.18615	0.00026	0.00026	turquoise
ALG3	0.04879	0.03081	-0.08587	-0.06328	-0.01558	0.03348	0.29293	-0.48628	0.48628	0.52623	0.68916	0.2641	0.41093	0.83967	0.66374	0.0001	1.55E-11	1.55E-11	pink
NUDC	0.10822	-0.08472	-0.01815	0.03566	0.07629	0.00885	0.0631	-0.26555	0.26555	0.15884	0.27056	0.81372	0.64331	0.32129	0.90858	0.41227	0.00045	0.00045	grey
TATDN3	-0.07527	-0.00064	-0.16001	-0.16611	0.14719	-0.21471	-0.20932	0.26994	-0.26994	0.32788	0.99336	0.03657	0.0299	0.05472	0.0048	0.006	0.00036	0.00036	turquoise
TJP3	0.10508	0.09301	0.07973	0.02004	0.07334	0.23748	0.18939	-0.48113	0.48113	0.17137	0.2263	0.29994	0.79471	0.34044	0.00176	0.01311	2.72E-11	2.72E-11	yellow
ZNF107	-0.06472	-0.1666	0.01539	0.03608	-0.03701	0.01268	-0.03851	0.16238	-0.16238	0.40033	0.02942	0.84164	0.63941	0.63077	0.86928	0.61706	0.03384	0.03384	turquoise
TAF2	-0.05344	-0.11388	0.02576	0.04315	-0.08877	0.02461	0.0259	0.12993	-0.12993	0.48758	0.13805	0.73805	0.57518	0.24828	0.74937	0.73672	0.09032	0.09032	turquoise
SAR1A	0.00039	-0.14698	0.05982	0.02244	-0.18326	0.06756	0.11175	0.10649	-0.10649	0.99596	0.05507	0.43704	0.77085	0.01643	0.37993	0.14564	0.16568	0.16568	turquoise
GLMN	-0.11744	-0.03897	0.07448	0.05718	-0.02332	0.0218	0.06371	-0.01866	0.01866	0.12606	0.61281	0.33297	0.45754	0.76211	0.77714	0.40779	0.80862	0.80862	turquoise
POM121C	0.01505	-0.24242	-0.07307	-0.02394	0.02747	-0.11987	-0.04576	0.12506	-0.12506	0.84508	0.0014	0.34222	0.75596	0.72131	0.11837	0.55232	0.10316	0.10316	turquoise
FNDC1	-0.03524	-0.03989	0.11678	0.06217	-0.19303	0.22934	0.07277	0.22953	-0.22953	0.64722	0.60449	0.12825	0.41918	0.01142	0.00255	0.34422	0.00253	0.00253	black
ADAMTS4	-0.1268	-0.11584	0.01893	0.08721	-0.16567	0.06846	0.00383	0.35601	-0.35601	0.0984	0.13136	0.80583	0.2567	0.03035	0.37363	0.96034	1.76E-06	1.76E-06	blue
HSD17B7	0.10282	-0.01665	-0.04495	-0.15228	0.12784	0.09121	0.01643	-0.29089	0.29089	0.18084	0.82884	0.55941	0.04677	0.09565	0.23542	0.83106	0.00011	0.00011	greenyellow
RBMS3	-0.06337	-0.12996	-0.01039	0.05817	-0.24893	0.12739	-0.00574	0.53154	-0.53154	0.41023	0.09024	0.89266	0.44978	0.00103	0.09684	0.94061	7.42E-14	7.42E-14	blue
ATP6V1C1	-0.06257	-0.14069	-0.07918	-0.02553	-0.12172	-0.09582	0.01748	0.16656	-0.16656	0.41623	0.06645	0.30327	0.74029	0.11275	0.21252	0.82048	0.02946	0.02946	turquoise
SLC2A8	-0.03352	-0.0311	-0.18633	-0.0764	0.17225	-0.10508	-0.02101	0.07078	-0.07078	0.66335	0.68637	0.01468	0.32059	0.02427	0.17138	0.78501	0.35761	0.35761	grey
MAP11	0.04077	-0.08507	-0.01621	-0.10759	0.20668	-0.11476	-0.05078	-0.06541	0.06541	0.59648	0.26861	0.83337	0.1613	0.00668	0.13501	0.50955	0.39533	0.39533	red
STXBP1	0.04475	-0.13837	-0.20934	-0.12754	0.0625	-0.22669	-0.19	0.39831	-0.39831	0.56114	0.07109	0.006	0.09644	0.41675	0.00287	0.01281	6.84E-08	6.84E-08	brown
RAP2C	-0.06203	-0.14724	0.07165	0.05959	-0.12747	0.14987	0.13018	0.02664	-0.02664	0.42022	0.05464	0.35169	0.43881	0.09663	0.05041	0.08968	0.72945	0.72945	turquoise
C11orf1	0.0039	0.05233	-0.01816	-0.12674	-0.00601	0.01457	0.17713	-0.38449	0.38449	0.95967	0.49665	0.81367	0.09855	0.93786	0.84995	0.02047	2.08E-07	2.08E-07	grey

RCOR3	0.01909	-0.09888	-0.12411	-0.05327	0.06584	-0.07648	-0.01258	0.02476	-0.02476	0.80427	0.19822	0.10582	0.48894	0.39221	0.32011	0.8703	0.74792	0.74792	red
CACNB1	0.04305	-0.11879	-0.01517	0.04299	0.24673	-0.06097	0.00692	-0.28306	0.28306	0.57608	0.12176	0.8439	0.57667	0.00114	0.42828	0.92847	0.00018	0.00018	grey
SKA3	-0.00875	0.13828	0.02769	-0.0518	-0.07118	0.06693	0.30947	-0.4882	0.4882	0.90952	0.07128	0.71926	0.50104	0.35491	0.38443	3.81E-05	1.26E-11	1.26E-11	pink
COL1A2	-0.04841	-0.04704	0.07145	0.09061	-0.15893	0.16476	0.06893	0.17202	-0.17202	0.5295	0.54123	0.35306	0.23853	0.03787	0.03129	0.37032	0.02446	0.02446	black
EFCC1	-0.01987	-0.1529	-0.21816	-0.13977	0.12646	-0.05539	-0.13215	0.30979	-0.30979	0.79643	0.04587	0.00415	0.06826	0.09931	0.4718	0.0849	3.74E-05	3.74E-05	grey
MCM3AP	-0.05417	-0.1305	-0.10371	-0.02261	0.11243	-0.052	-0.0565	0.24806	-0.24806	0.48161	0.08889	0.17707	0.76912	0.14318	0.49942	0.46295	0.00107	0.00107	turquoise
RNF144B	-0.19956	-0.06539	0.05358	0.08171	-0.11113	0.15996	0.05627	0.22417	-0.22417	0.00887	0.39545	0.48643	0.28803	0.1479	0.03663	0.46479	0.0032	0.0032	blue
HOXD8	-0.14116	-0.02171	-0.03315	0.04459	-0.08296	-0.04624	0.00404	0.48993	-0.48993	0.06553	0.77811	0.66685	0.56256	0.28072	0.54817	0.95822	1.04E-11	1.04E-11	blue
PGGT1B	-0.0136	-0.09566	-0.04901	0.01035	0.04526	0.01885	-0.09245	0.17618	-0.17618	0.85989	0.21329	0.52443	0.89315	0.55666	0.80671	0.22911	0.02116	0.02116	turquoise
OCIAD1	-0.112	-0.13125	-0.13702	-0.06151	0.08603	-0.18421	-0.06732	0.07075	-0.07075	0.1447	0.08706	0.07393	0.42417	0.26325	0.01587	0.38168	0.35783	0.35783	turquoise
NOVA1	-0.03461	-0.1286	-0.2772	-0.10785	0.11876	-0.35238	-0.27231	0.51972	-0.51972	0.65314	0.09368	0.00024	0.16031	0.12186	2.28E-06	0.00031	3.24E-13	3.24E-13	brown
CNTNAP1	-0.13001	-0.14295	-0.03718	0.03527	-0.1093	0.06013	-0.03911	0.44982	-0.44982	0.09011	0.06216	0.6292	0.64702	0.15472	0.43468	0.61151	6.74E-10	6.74E-10	salmon
C6orf132	0.09543	0.07112	0.10254	0.081	-0.10771	0.30743	0.29425	-0.47198	0.47198	0.21438	0.35528	0.18204	0.29225	0.16085	4.31E-05	9.37E-05	7.19E-11	7.19E-11	yellow
C19orf18	-0.01043	-0.03436	-0.10741	-0.02068	0.15316	-0.2138	-0.17023	0.10786	-0.10786	0.89233	0.65548	0.16202	0.78834	0.0455	0.00499	0.02601	0.16027	0.16027	grey
EGR1	-0.03058	-0.12944	0.00089	-0.02667	0.03115	0.08745	0.02471	0.32251	-0.32251	0.69133	0.09153	0.99077	0.72918	0.68584	0.25541	0.74834	1.69E-05	1.69E-05	blue
COP1	0.03865	-0.0026	0.00324	-0.06449	0.01529	0.14304	0.1774	-0.2698	0.2698	0.61577	0.97303	0.96646	0.402	0.84269	0.06199	0.02027	0.00036	0.00036	turquoise
CNST	-0.05913	-0.05228	-0.09444	-0.03274	-0.00227	-0.01775	-0.09227	0.33565	-0.33565	0.44238	0.49707	0.21921	0.6708	0.97645	0.8178	0.23005	7.19E-06	7.19E-06	turquoise
TRMT10C	-0.17214	0.05228	-0.07522	-0.05322	-0.12691	0.07699	0.12153	-0.04281	0.04281	0.02436	0.49707	0.32815	0.48932	0.0981	0.31689	0.11331	0.5782	0.5782	turquoise
DNAJB12	0.03155	-0.12038	-0.07612	-0.07535	-0.11927	-0.1388	0.09175	-0.07664	0.07664	0.6821	0.11681	0.32241	0.32734	0.12024	0.07022	0.23266	0.31913	0.31913	grey
ZC3H3	0.08212	-0.04414	-0.1327	-0.04069	0.07971	-0.09845	0.09819	-0.2854	0.2854	0.28561	0.5665	0.08359	0.59724	0.30005	0.20018	0.20136	0.00015	0.00015	green
LETMD1	0.14309	-0.04955	-0.10768	-0.08531	0.16824	-0.15095	-0.11559	-0.11195	0.11195	0.0619	0.51988	0.16095	0.26725	0.02784	0.04876	0.1322	0.14489	0.14489	grey
ID3	-0.02992	-0.07348	0.07852	0.04272	-0.08505	0.04831	0.07159	0.21988	-0.21988	0.69771	0.33951	0.30732	0.57908	0.26872	0.53032	0.35212	0.00386	0.00386	black
ZNF584	-0.04811	-0.07262	-0.16162	-0.05429	-0.02539	-0.08007	-0.07668	0.12058	-0.12058	0.53205	0.34522	0.03469	0.48064	0.74169	0.29787	0.31884	0.1162	0.1162	grey
VPS33B	-0.03256	-0.07937	-0.0716	-0.06565	0.07607	-0.05833	-0.05162	-0.09527	0.09527	0.67246	0.30211	0.35203	0.39361	0.32269	0.44855	0.50251	0.21517	0.21517	turquoise
COL5A1	-0.05336	-0.06931	0.03375	0.06729	-0.1603	0.17011	0.09976	0.11891	-0.11891	0.48825	0.3677	0.66125	0.38185	0.03623	0.02612	0.19421	0.12137	0.12137	black
RBL2	-0.06412	-0.14256	0.01445	-0.00549	-0.08519	0.11086	-0.05283	0.26626	-0.26626	0.40475	0.06289	0.85117	0.94323	0.26792	0.14888	0.49253	0.00043	0.00043	turquoise
USP7	-0.00844	-0.05552	-0.01438	-0.00556	-0.04792	0.03636	-0.03096	0.06734	-0.06734	0.91274	0.47076	0.85195	0.94243	0.53364	0.63681	0.68774	0.38148	0.38148	turquoise
THRAP3	-0.0766	-0.09887	0.00106	-0.02159	-0.14385	0.08614	0.01581	0.17121	-0.17121	0.31932	0.19823	0.98907	0.77924	0.06051	0.26262	0.83742	0.02515	0.02515	turquoise
CASTOR1	-0.12172	-0.04871	-0.06406	0.02986	0.00365	-0.03259	0.01925	-0.10498	0.10498	0.11276	0.52697	0.40518	0.69827	0.96225	0.67216	0.80263	0.17176	0.17176	grey
TNFRSF8	-0.12254	0.02204	0.08017	0.11023	-0.10991	0.11607	-0.00802	0.44553	-0.44553	0.11032	0.77474	0.29724	0.15121	0.15243	0.13058	0.91709	1.02E-09	1.02E-09	blue
OGDH	0.01732	-0.07634	0.05122	0.02575	-0.03013	0.05847	-0.02403	0.00689	-0.00689	0.82209	0.32098	0.50582	0.73819	0.69563	0.44746	0.75507	0.92873	0.92873	turquoise
SUB1	-0.04355	-0.02841	-0.05559	-0.00637	-0.0142	-0.09433	0.10234	0.08424	-0.08424	0.57172	0.71223	0.47018	0.93409	0.85376	0.21972	0.1829	0.27329	0.27329	turquoise
TMEM131	-0.12319	-0.04068	-0.05654	0.00638	-0.21188	-0.03695	-0.10199	0.53224	-0.53224	0.10844	0.59729	0.46261	0.93403	0.0054	0.63141	0.18437	6.79E-14	6.79E-14	blue
NUF2	-0.03592	0.11552	0.03315	-0.07869	-0.00393	0.05746	0.27023	-0.45109	0.45109	0.6409	0.13244	0.66685	0.3063	0.95932	0.45536	0.00035	5.95E-10	5.95E-10	pink
HLA-DQA	-0.12288	0.04998	0.0128	0.02621	-0.22386	0.15625	0.08232	0.30053	-0.30053	0.10934	0.51619	0.86807	0.73365	0.00325	0.04127	0.28444	6.50E-05	6.50E-05	blue
CD300C	-0.12198	0.00791	0.05707	0.0425	-0.14876	0.15669	0.14752	0.29605	-0.29605	0.112	0.91817	0.45846	0.58098	0.05216	0.04069	0.05417	8.44E-05	8.44E-05	blue
TOGARAN	-0.02968	-0.19893	-0.08316	0.05292	-0.01702	-0.01504	-0.03467	0.27864	-0.27864	0.69995	0.0091	0.27953	0.49186	0.82516	0.84516	0.65261	0.00022	0.00022	turquoise
G6PD	-0.01115	-0.07814	-0.02689	-0.00872	-0.08986	0.04845	0.2073	-0.1485	0.1485	0.88491	0.30966	0.72702	0.90985	0.24246	0.52917	0.00652	0.05257	0.05257	grey
RNASE1	-0.13557	0.08261	0.17777	0.05167	-0.21704	0.24555	0.13637	0.10643	-0.10643	0.07707	0.28272	0.02001	0.5021	0.00435	0.00121	0.07531	0.16592	0.16592	grey
TMCC1	0.04316	-0.0832	-0.06376	0.0483	-0.10864	0.06492	0.10919	-0.08554	0.08554	0.57515	0.27928	0.40736	0.53042	0.15724	0.39889	0.15515	0.26593	0.26593	turquoise
CLTB	0.06497	0.01398	0.02665	-0.02033	0.06202	0.09155	0.11094	-0.44016	0.44016	0.3985	0.85594	0.72934	0.79188	0.4203	0.23369	0.14859	1.70E-09	1.70E-09	yellow
NIPSNAP2	-0.1449	-0.09543	-0.05275	0.01408	-0.01404	0.05774	0.00393	0.08985	-0.08985	0.05864	0.21437	0.49322	0.85498	0.85539	0.45315	0.95932	0.2425	0.2425	turquoise
AZIN1	0.0081	-0.08027	0.03635	0.05944	-0.11546	0.03959	0.02666	-0.01119	0.01119	0.91626	0.29662	0.63689	0.43998	0.13265	0.60717	0.72928	0.8845	0.8845	turquoise
SARDH	-0.11269	-0.00995	-0.08875	-0.01175	-0.08946	-0.10393	-0.10872	0.45939	-0.45939	0.14223	0.89718	0.24835	0.87875	0.24461	0.17612	0.15694	2.62E-10	2.62E-10	grey
MYL4	-0.0948	-0.11492	-0.03679	-0.04123	-0.1411	-0.06944	0.06491	0.17898	-0.17898	0.21745	0.13447	0.63282	0.59231	0.06565	0.3668	0.39895	0.01917	0.01917	grey
NEMF	-0.03827	-0.1551	0.05218	0.05233	-0.05452	0.06848	0.01484	0.18347	-0.18347	0.61924	0.0428	0.49789	0.4967	0.4788	0.3735	0.8472	0.01631	0.01631	turquoise
HDHD5	-0.04536	0.00658	-0.17107	-0.00147	0.1422	-0.17628	0.02056	-0.089	0.089	0.55581	0.93192	0.02527	0.98482	0.06355	0.02109	0.78954	0.24704	0.24704	grey
C1GALT1C	-0.02136	-0.17506	-0.03993	0.01864	0.02583	-0.05711	0.07707	-0.17198	0.17198	0.78151	0.02202	0.60406	0.80879	0.73736	0.45811	0.31638	0.0245	0.0245	turquoise
NEUROD1	-0.02114	-0.09621	-0.25067	-0.15945	0.04388	-0.21263	-0.15006	0.42615	-0.42615	0.78378	0.21066	0.00094	0.03724	0.56874	0.00524	0.05011	6.20E-09	6.20E-09	brown
FAXC	-0.00903	-0.17713	-0.22136	-0.08754	0.05278	-0.31692	-0.18442	0.29508	-0.29508	0.9067	0.02047	0.00362	0.25488	0.49295	2.41E-05	0.01575	8.93E-05	8.93E-05	brown
PRICKLE1	-0.00897	-0.11357	-0.03661	0.05187	-0.20647	0.01505	0.01167	0.47828	-0.47828	0.90728	0.13912	0.63447	0.50047	0.00674	0.84505	0.8796	3.69E-11	3.69E-11	blue
ERN2	0.04786	0.12258	0.14973	0.09402	0.08818	0.27677	0.16743	-0.38046	0.38046	0.53417	0.11022	0.05062	0.22126	0.25142	0.00025	0.02861	2.85E-07	2.85E-07	yellow
TFRC	-0.15383	-0.02918	0.00541	-0.01019	-0.09218	0.19619	0.17771	-0.07641	0.07641	0.04455	0.70477	0.94397	0.89482	0.23047	0.01012	0.02005	0.32058	0.32058	turquoise
CD3G	-0.07008	-0.03307	0.03637	0.06999	-0.19004	0.1309	-0.01176	0.40604	-0.40604	0.36242	0.66768	0.63676	0.36302	0.01279	0.0879	0.87871	3.59E-08	3.59E-08	blue
TMEM97	-0.01981	0.17565	0.11088	0.15461	-0.03671	0.25337	0.10122	-0.14501	0.14501	0.79709	0.02157	0.1488	0.04348	0.63355	0.00083	0.18775	0.05845	0.05845	magenta
PRSS33	0.10233	0.23501	0.0632	-0.08806	0.01988	0.07448	0.00274	-0.2419	0.2419	0.18291	0.00197	0.41153	0.25208	0.79639	0.33298	0.97162	0.00143	0.00143	grey
PPP2R5D	0.02412	-0.02509	-0.02434	-0.00155	-0.10369	0.03276	0.06666	0.10726	-0.10726	0.75417	0.74458	0.75198	0.98396	0.17712	0.67057	0.38633	0.16261	0.16261	turquoise
TBC1D13	-0.00838	-0.14982	-0.2187	-0.06139	-0.07786	-0.2113	-0.12383	0.3138	-0.3138	0.91337	0.05049	0.00406	0.42508	0.31142	0.00553	0.1066	2.92E-05	2.92E-05	brown
CCL2	-0.11288	-0.09832	-0.07496	0.01317	-0.14829	0.07283	-0.1036	0.39579	-0.39579	0.14158	0.20075	0.32988	0.86423	0.05291	0.34379	0.17751	8.41E-08	8.41E-08	blue
STON1	-0.07651	-0.09756	0.03287	0.06942	-0.20008	0.10104	-0.02116	0.44438	-0.44438	0.31992	0.2043	0.66956	0.36696	0.0087	0.18851	0.78354	1.14E-09	1.14E-09	blue
EDARADD	0.01484	-0.05575	-0.17733	-0.06938	-0.02196	-0.24461	-0.03824	0.45915	-0.45915	0.84721	0.46889	0.02033	0.3672	0.77557	0.00126	0.61947	2.68E-10	2.68E-10	brown
AC015871	0.08136	-0.17157	-0.09355	-0.04149	0.13118	-0.23579	-0.14296	-0.13466	0.13466	0.2901	0.02485	0.22361	0.59001	0.08722	0.0019	0.06213	0.07908	0.07908	grey
POT1	-0.00944	-0.08833	0.00848	-0.00105	-0.09888	-0.03434	0.00889	0.0436	-0.0436	0.9025	0.25064	0.9124	0.98911	0.19821	0.65566	0.90818	0.5712	0.5712	turquoise
APOBEC3A	-0.10558	0.07457	0.09097	0.03397	-0.09222	0.01205	0.05816	0.18446	-0.18446	0.16933	0.33238	0.23668	0.65912	0.23029	0.87573	0.44991	0.01573	0.01573	grey
ABI3	-0.11076	-0.02166	-0.02683	0.03514	-0.127	0.04036	-0.00314	0.40465	-0.40465	0.14923	0.77855	0.72763	0.64815	0.09788	0.60022	0.96745	4.04E-08	4.04E-08	blue
ITM2A	-0.14179	-0.05599	-0.0857	0.0294	-0.05805	0.02295	-0.18611	0.47762	-0.47762	0.06433	0.46697	0.26506	0.70267	0.45075	0.76572	0.0148	3.96E-11	3.96E-11	blue
LYVE1	-0.08256	-0.06672	0.00316	0.0551	-0.04262	0.06963	-0.04689	0.49522	-0.49522	0.28302	0.38594	0.96726	0.4741	0.57994	0.36549	0.54251	5.75E-12	5.75E-12	blue
NUBPL	0.00757	-0.04192	0.02547	0.03383	-0.05235	0.0192	0.03142	0.07402	-0.07402	0.9217	0.58621	0.7409	0.66051	0.49647	0.80319	0.68326	0.33599	0.33599	turquoise
BHMT2	-0.02425	-0.08532	0.0025	0.06607	-0.15096	0.08294	-0.0257	0.5767	-0.5767	0.75291	0.26722	0.97414	0.39058	0.04873	0.2808	0.73864	1.52E-16	1.52E-16	blue
IRF7	0.0556	-0.0571	0.03616	0.0785	0.01222	0.09322	0.15091	-0.26696	0.26696	0.47013	0.45819	0.6387	0.30745	0.87395	0.22524	0.04882	0.00042	0.00042	tan
GNA13	-0.06653	-0.16972	0.06718	0.09239	-0.19269	0.17262	0.05013	0.18606	-0.18606	0.38729	0.02647	0.38267	0.22944	0.01157	0.02396	0.51495	0.01483	0.01483	turquoise
C16orf89	-0.12164	-0.08665	-0.06814	0.07068	-0.00467	0.07909	-0.09185	0.48896	-0.48896	0.11299	0.25976	0.37589	0.35829	0.95161	0.30381	0.23218	1.16E-11	1.16E-11	blue
MAP3K21	0.06759	-0.06322	-0.12198	0.02945	0.06681	0.08231	0.01325	-0.08541	0.08541	0.37975	0.41136	0.11197	0.70215	0.38527	0.28451	0.86349	0.26669	0.26669	grey
PITPNB	-0.10046	-0.05227	0.02333	0.10641	-0.1465	0.05479	0.11171	0.02727	-0.02727	0.19109	0.49712	0.76199	0.16599	0.05588	0.47666	0.14575	0.72334	0.72334	turquoise
SLC16A1	-0.03364	0.00793	0.13317	0.07537	-0.13258	0.26853	0.29119	-0.19599	0.19599	0.66225	0.91806	0.08249	0.32723	0.08388	0.00038	0.00011	0.0102	0.0102	turquoise
ZBTB42	0.00172	0.01429	-0.0251	0.0507	0.03086	0.05015	0.086	-0.27708	0.27708	0.98215	0.85286	0.74453	0.51018	0.68867	0.51483	0.2634	0.00024	0.00024	grey
FXN	-0.03504	0.03565	-0.1528	-0.05343	-0.01543	-0.10518	-0.09529	0.1774	-0.1774	0.64911	0.64339	0.04602	0.48762	0.84124	0.17094	0.21507	0.02027	0.02027	grey
CHD7	0.02381	-0.15129	-0.00535	0.01759	-0.04287	-0.01221	-0.01806	0.01226	-0.01226	0.75724	0.04825	0.94459	0.81938	0.57768	0.87402	0.8146	0.87355	0.87355	turquoise
HYAL2	-0.13848	-0.00183	-0.01617	-0.00248	-0.05244	0.0904	0.01651	0.12584	-0.12584	0.07088	0.98107	0.83375	0.97433	0.49573	0.23967	0.83026	0.101	0.101	blue
ALDH3B1	0.04438	0.04946	0.04823	0.09452	-0.07429	0.2249	0.34175	-0.41243	0.41243	0.56439	0.5206	0.53099	0.21882	0.3342	0.0031	4.77E-06	2.08E-08	2.08E-08	yellow
TAPT1	-0.09848	-0.21355	-0.12771	0.00298	0.02509	-0.1872	-0.21872	0.30591	-0.30591	0.20001	0.00504	0.096	0.96915	0.74466	0.01422	0.00405	4.72E-05	4.72E-05	turquoise
MFAP3L	-0.03802	-0.1265	-0.04157	0.10202	-0.16174	-0.03485	-0.05669	0.43773	-0.43773	0.62152	0.09922	0.58934	0.18423	0.03456	0.65093	0.46141	2.14E-09	2.14E-09	blue
KIF20B	-0.05434	0.03643	0.0546	0.00601	-0.17641	0.05371	0.25446	-0.15961	0.15961	0.48023	0.63619	0.47819	0.93777	0.021	0.4854	0.00078	0.03705	0.03705	pink
NDST1	-0.0837	-0.18745	-0.197	-0.02196	-0.0834	-0.21554	-0.13345	0.5243	-0.5243	0.27639	0.01409	0.00981	0.7756	0.27816	0.00464	0.08183	1.84E-13	1.84E-13	turquoise
GPR146	-0.15383	-0.0792	-0.06365	0.11982	0.01065	0.04816	-0.06559	0.41739	-0.41739	0.04456	0.30313	0.40823	0.11852	0.89005	0.5316	0.39401	1.35E-08	1.35E-08	blue
RBFOX2	-0.08084	-0.16569	-0.05741	0.05459	-0.10204	-0.00785	-0.01601	0.31846	-0.31846	0.29321	0.03033	0.45576	0.47825	0.18416	0.91886	0.83535	2.19E-05	2.19E-05	blue
MMP28	-0.04226	0.05546	0.09841	0.03878	0.05334	0.14642	0.25292	-0.36664	0.36664	0.58311	0.47125	0.20036	0.61452	0.48837	0.05601	0.00085	8.14E-07	8.14E-07	yellow
COX11	-0.07244	-0.13812	-0.14344	-0.03385	0.14736	-0.24218	-0.15961	0.13811	-0.13811	0.34641	0.07162	0.06126	0.6603	0.05444	0.00142	0.03705	0.07165	0.07165	turquoise
SHOX2	-0.02942	-0.05741	0.11885	0.10819	-0.06729	-0.04225	0.02967	0.10238	-0.10238	0.70246	0.45579	0.12155	0.15899	0.38188	0.58326	0.7001	0.1827	0.1827	grey
CCM2	-0.03907	-0.10672	-0.06509	0.0514	-0.05545	-0.19225	0.00805	0.02804	-0.02804	0.61188	0.16473	0.39766	0.50432	0.47128	0.01176	0.91675	0.7158	0.7158	grey
RNF26	-0.04513	-0.04912	-0.07369	-0.02808	-0.1331	-0.01134	0.16575	-0.07469	0.07469	0.55781	0.52344	0.33811	0.71547	0.08265	0.88298	0.03027	0.3316	0.3316	turquoise
TPD52L2	0.07312	-0.06308	0.00945	0.04285	-0.09367	-0.01826	0.17896	-0.1828	0.1828	0.3419	0.41244	0.90242	0.57788	0.22301	0.81264	0.01918	0.01671	0.01671	turquoise
LIME1	0.0545	-0.01603	-0.09581	-0.03339	0.23806	-0.09547	0.01928	-0.31428	0.31428	0.47892	0.8352	0.21254	0.66459	0.00172	0.21422	0.80238	2.84E-05	2.84E-05	red
CORIN	0.05235	-0.01001	0.07213	0.06564	-0.19425	0.20662	0.08376	0.23333	-0.23333	0.49646	0.8966	0.34849	0.3937	0.0109	0.0067	0.27609	0.00213	0.00213	black
RHEX	-0.11974	-0.01221	-0.01221	0.13557	0.04168	0.12144	0.04017	0.11843	-0.11843	0.11877	0.87409	0.8741	0.07707	0.58835	0.11359	0.60191	0.1229	0.1229	grey
RNASEH1	-0.0007	-0.00879	0.01001	-0.03274	-0.17552	0.15226	0.24238	-0.06615	0.06615	0.99272	0.90913	0.89664	0.67076	0.02166	0.0468	0.0014	0.38997	0.38997	turquoise
GRB2	-0.07564	-0.12365	-0.02772	-0.01686	-0.07415	-0.02105	0.00194	0.21735	-0.21735	0.32544	0.10712	0.71895	0.82671	0.33509	0.78463	0.97988	0.0043	0.0043	turquoise
CPB1	0.01217	0.03956	0.058	0.13837	-0.12333	0.09308	-0.06263	0.14272	-0.14272	0.87444	0.60748	0.45112	0.0711	0.10803	0.22594	0.41579	0.06259	0.06259	magenta
ANXA2R	0.00215	-0.01224	0.0124	0.03324	-0.11199	0.04895	0.16172	0.18975	-0.18975	0.97776	0.87375	0.87209	0.66606	0.14475	0.52495	0.03459	0.01293	0.01293	blue
USP37	0.02395	-0.13737	0.00198	0.06341	-0.16223	0.14263	0.10624	0.18861	-0.18861	0.75584	0.07318	0.97955	0.40994	0.03401	0.06275	0.16665	0.01349	0.01349	turquoise
ARIH2	-0.07193	-0.13988	-0.1142	-0.10206	0.03026	-0.05467	-0.09903	0.19464	-0.19464	0.3498	0.06804	0.13695	0.18408	0.69441	0.47759	0.1975	0.01074	0.01074	turquoise
TUBB4B	-0.00495	-0.00351	-0.04075	-0.03762	0.05812	0.11376	0.16423	-0.41175	0.41175	0.94877	0.96371	0.59665	0.62523	0.4502	0.13846	0.03184	2.21E-08	2.21E-08	grey
APOL1	-0.03711	0.01833	-0.0322	0.08059	-0.01643	0.14496	0.26131	-0.34055	0.34055	0.62992	0.81188	0.67585	0.29471	0.83111	0.05853	0.00056	5.18E-06	5.18E-06	yellow
ARAP2	-0.08333	-0.0733	0.03222	0.04971	-0.15497	0.20168	0.16719	-0.08013	0.08013	0.27853	0.3407	0.67565	0.51846	0.04299	0.00817	0.02884	0.29751	0.29751	turquoise
CEP55	-0.01191	0.06853	0.05654	0.01806	-0.10894	0.10281	0.33332	-0.45057	0.45057	0.87715	0.37311	0.46265	0.81461	0.15609	0.18087	8.40E-06	6.26E-10	6.26E-10	pink
CORO2A	0.05464	-0.0041	0.05932	0.05048	-0.07352	0.21373	0.22454	-0.31626	0.31626	0.47784	0.9576	0.44089	0.51206	0.33924	0.005	0.00315	2.51E-05	2.51E-05	yellow
PDGFC	-0.09238	-0.06296	0.01457	0.08331	-0.275	0.22129	0.13848	0.06644	-0.06644	0.22946	0.41332	0.85003	0.27868	0.00027	0.00363	0.07087	0.38795	0.38795	black
RSL24D1	-0.03352	-0.0272	-0.05546	-0.05427	-0.10751	0.03072	0.00855	-0.04199	0.04199	0.66342	0.72396	0.47121	0.48081	0.1613	0.68997	0.91166	0.58554	0.58554	turquoise
ROR1	-0.00907	-0.13948	0.04716	0.11747	-0.20731	0.13192	0.03823	0.42985	-0.42985	0.90627	0.06884	0.54022	0.12597	0.00652	0.08545	0.61957	4.43E-09	4.43E-09	blue
MYO1G	-0.14673	0.03133	0.06931	0.05468	-0.15409	0.08922	0.05106	0.31631	-0.31631	0.05548	0.68418	0.3677	0.47751	0.04419	0.24587	0.50719	2.50E-05	2.50E-05	blue
MESP2	-0.03211	-0.01847	-0.03584	0.10439	-0.06099	0.03792	0.23913	0.03389	-0.03389	0.67677	0.81052	0.64169	0.17423	0.42811	0.6224	0.00163	0.65991	0.65991	grey
UBE2E3	0.04393	-0.00604	0.00393	0.06615	-0.10226	0.06623	0.18423	-0.20479	0.20479	0.5683	0.93751	0.95927	0.39001	0.18323	0.38945	0.01586	0.00721	0.00721	turquoise
SRSF10	-0.04747	-0.16781	0.00968	0.03932	-0.06433	0.11494	0.0468	0.04033	-0.04033	0.53755	0.02825	0.89998	0.60963	0.40322	0.1344	0.54328	0.60051	0.60051	turquoise
TAX1BP1	0.03221	-0.13573	0.06421	0.07884	-0.0267	0.09121	0.06248	-0.12394	0.12394	0.67579	0.0767	0.40407	0.30535	0.72881	0.23543	0.41687	0.10629	0.10629	turquoise
PFAS	-0.07355	-0.07897	-0.05837	-0.00147	-0.02816	-0.0692	0.0295	0.26594	-0.26594	0.33902	0.30459	0.44824	0.98474	0.71461	0.36848	0.70168	0.00044	0.00044	turquoise
ZRANB1	-0.08823	-0.19006	-0.0347	0.02775	-0.11147	0.0189	-0.02524	0.3304	-0.3304	0.25115	0.01278	0.65233	0.71865	0.14664	0.8062	0.74312	1.02E-05	1.02E-05	turquoise
RC3H2	-0.08078	-0.19479	-0.03119	0.00168	-0.22452	0.02706	0.03874	0.2671	-0.2671	0.29359	0.01068	0.68548	0.9826	0.00315	0.72537	0.61492	0.00041	0.00041	turquoise
TTC30A	-0.14696	-0.16477	-0.12801	0.05217	0.03555	-0.08501	-0.04694	0.10116	-0.10116	0.0551	0.03127	0.0952	0.49802	0.64433	0.26894	0.54213	0.18799	0.18799	turquoise
GJA4	-0.12072	0.05583	-0.07585	0.05337	-0.01781	-0.07546	-0.11829	0.46187	-0.46187	0.11575	0.46825	0.32412	0.48812	0.81712	0.32662	0.12335	2.04E-10	2.04E-10	blue
TLR10	-0.116	-0.02917	0.03392	-0.02788	-0.07731	0.05569	-0.10094	0.38812	-0.38812	0.13082	0.70493	0.65967	0.71741	0.31486	0.46939	0.18896	1.56E-07	1.56E-07	blue
COA8	0.0893	-0.04221	0.00761	0.05266	0.03315	-0.05392	0.06554	-0.34785	0.34785	0.24541	0.58361	0.92134	0.49398	0.66686	0.48365	0.39436	3.14E-06	3.14E-06	grey
PPP1R3G	-0.02098	-0.0317	0.07156	0.1461	0.03256	0.15032	0.10821	-0.30316	0.30316	0.78536	0.68062	0.35229	0.05655	0.67242	0.04972	0.15892	5.56E-05	5.56E-05	grey
COL27A1	0.02537	-0.03985	0.0038	0.06574	0.21052	0.02004	0.08404	-0.05826	0.05826	0.74191	0.60484	0.96066	0.39293	0.00571	0.79476	0.27447	0.44911	0.44911	grey
TMEM132	0.08329	-0.03597	-0.17869	-0.12419	0.03797	-0.09917	-0.15631	0.4002	-0.4002	0.27879	0.64044	0.01937	0.10558	0.62198	0.19686	0.04119	5.85E-08	5.85E-08	brown
[truncated: 1,001,582 more chars]
