# Supplementary material for: Molecular signatures of tumor progression in pancreatic adenocarcinoma identified by energy metabolism characteristics
Source: BMC Cancer. 2022 Apr 13;22:404. doi: 10.1186/s12885-022-09487-3 (PMC9006543; doi:10.1186/s12885-022-09487-3)
Supplement: Supplementary file 8 — Additional file 8. [file 12885_2022_9487_MOESM8_ESM.pdf]

Supplementary Table 3. Differential expression genes between Cluster 1 and Cluster 2

Gene_sym	FoldChage	P-value
REG4	-3.49464	4.48E-16
NKX2-8	-2.82713	2.85E-08
GKN2	-2.81283	8.59E-07
HRG	-2.79879	6.34E-08
CLCA1	-2.79696	2.16E-07
CA9	-2.72088	7.82E-18
MAGEA6	-2.69633	0.006509
APOA2	-2.66339	7.60E-05
KLK12	-2.57999	4.80E-12
PHGR1	-2.52953	1.52E-12
TFF3	-2.50035	1.89E-13
APOA4	-2.48027	7.20E-05
APOBEC1	-2.46599	2.05E-15
S100A2	-2.44393	1.68E-11
RETNLB	-2.43915	0.000666
FEZF1	-2.40539	1.86E-10
SERPINB3	-2.38995	4.89E-09
KRT14	-2.35406	1.69E-09
KRT5	-2.34926	1.52E-08
CGB5	-2.33759	8.00E-07
SLURP1	-2.33659	2.26E-07
NTF4	-2.32543	1.73E-16
MUC21	-2.31873	3.18E-08
B3GNT6	-2.28579	2.84E-08
KRT6A	-2.28189	7.54E-08
DEFA5	-2.28089	0.013884
TFF1	-2.26386	2.79E-11
SERPINB4	-2.26103	3.44E-10
PAEP	-2.25912	5.24E-11
FGF3	-2.25523	7.18E-07
PNCK	-2.23455	2.58E-12
FABP1	-2.22194	7.94E-07
C11orf86	-2.21859	6.03E-12
CALML3	-2.21477	4.11E-06
LYPD2	-2.20861	1.98E-06
CGB8	-2.2042	1.12E-08
KLHDC7B	-2.17548	3.47E-13
MAGEA3	-2.16777	0.015063
CREB3L3	-2.151	2.94E-10
ITLN1	-2.14115	3.05E-06
SPINK4	-2.13145	1.22E-06
TNNT1	-2.1222	1.79E-11
LHX1	-2.09233	5.26E-06
MAGEC2	-2.09015	0.025673
SLC10A2	-2.05518	0.000459
WFDC10B	-2.05393	4.66E-16
BTBD16	-2.05015	1.09E-21
GJB6	-2.04102	2.12E-09
ESPN	-2.03027	1.44E-19
MOGAT3	-2.01794	6.81E-10
ENSG0000	-2.00486	9.65E-10
TERT	-2.00078	8.07E-11
MOGAT2	-1.98251	9.43E-10
CASP14	-1.98085	0.003309
IFNE	-1.95986	8.97E-09
ATOH1	-1.95946	0.000312

TMEM40	-1.94442	9.72E-09
INSL4	-1.92976	0.000687
BPIFA2	-1.92565	8.46E-06
TRIM40	-1.92149	7.04E-08
C6orf15	-1.92034	1.50E-05
BTNL3	-1.90195	4.44E-05
GATA5	-1.89754	1.79E-05
UGT1A1	-1.89453	6.75E-07
LY6D	-1.88409	1.61E-05
CT83	-1.87314	0.014946
SULT1C2	-1.87273	1.96E-09
TNNI2	-1.87065	3.23E-11
IL20RB	-1.86894	8.99E-10
FABP6	-1.86763	3.30E-09
KCNA7	-1.8663	4.48E-09
LY6G6C	-1.86322	3.83E-11
BIRC7	-1.86274	2.26E-10
PAX9	-1.85384	4.13E-10
CGB7	-1.84342	1.93E-12
CST4	-1.83557	2.14E-10
WFDC3	-1.83018	1.33E-17
EGLN3	-1.82637	1.31E-16
TMPRSS15	-1.82597	0.001166
EFNA3	-1.82191	7.49E-20
NKX6-2	-1.81896	6.66E-05
CEACAM5	-1.81268	3.30E-07
CALHM3	-1.81267	5.24E-08
TNNC2	-1.81267	2.02E-13
KISS1	-1.81247	3.29E-10
COX6B2	-1.80467	6.05E-12
S100P	-1.80033	1.97E-11
CPS1	-1.79475	3.34E-10
C10orf99	-1.79027	0.000778
ITPKA	-1.78833	4.97E-17
MUC5AC	-1.78624	1.55E-06
PITX1	-1.78469	1.86E-15
HMGA2	-1.78126	9.07E-10
TRIM54	-1.78064	9.87E-12
RP1L1	-1.77859	8.07E-14
DQX1	-1.77455	1.16E-09
LHB	-1.77356	1.96E-13
CEACAM1	-1.77174	0.000288
TFF2	-1.7717	1.66E-06
DNAJB13	-1.77121	2.48E-11
PSAPL1	-1.77021	6.17E-05
TRIM15	-1.76546	2.20E-14
ANXA10	-1.76279	1.13E-09
CAPN8	-1.76184	3.73E-15
LGALS7B	-1.76155	5.14E-07
HOXB9	-1.75998	2.41E-09
FIBCD1	-1.75182	2.33E-08
KRT20	-1.74977	0.00059
WIF1	-1.73951	4.89E-06
FAM25A	-1.73407	1.23E-06
PTK6	-1.73402	1.40E-22
KNG1	-1.73196	3.58E-06
ONECUT3	-1.72237	9.49E-08
TUBAL3	-1.71908	4.09E-10

CYP24A1	-1.71901	7.73E-06
CHP2	-1.71473	0.000188
FOXH1	-1.71445	2.73E-11
KRT6C	-1.71376	7.97E-07
FAM3D	-1.71284	1.18E-07
BTNL8	-1.712	9.67E-08
AGXT	-1.71186	1.86E-06
AMN	-1.69955	4.82E-14
FAM83A	-1.69723	5.50E-07
TBX6	-1.69546	8.35E-24
MB	-1.69177	2.59E-09
PPP1R14D	-1.68876	3.51E-13
CHST5	-1.6882	1.30E-06
AXDND1	-1.68675	9.85E-08
TM4SF20	-1.68389	0.000496
LTK	-1.68339	2.09E-13
C4BPB	-1.68062	2.49E-13
DSG3	-1.67883	5.86E-06
PRSS33	-1.6787	6.89E-07
HOXC12	-1.67686	0.00768
CGB	-1.66945	0.000416
KRT13	-1.66743	2.99E-05
KRT78	-1.66315	2.34E-08
RNF183	-1.65934	1.24E-15
PDE4C	-1.65104	4.23E-14
SPACA4	-1.649	8.24E-15
IHH	-1.64517	2.41E-09
RXFP4	-1.64496	2.20E-11
PRAC2	-1.64302	5.10E-07
KRT15	-1.63945	1.11E-13
NPSR1	-1.63887	0.00039
C19orf33	-1.63817	7.26E-15
OBP2B	-1.62877	1.30E-05
LRRC31	-1.62429	5.37E-07
GPR87	-1.62243	7.34E-07
ENSG0000	-1.62222	8.40E-07
ADAP1	-1.62181	8.69E-18
IL36RN	-1.62122	0.000413
CLDN18	-1.62104	1.59E-05
TRIM7	-1.62057	7.73E-19
RNF103-C	-1.62023	3.45E-14
ENTPD8	-1.61588	4.97E-11
C6orf223	-1.61512	1.05E-13
PLA2G10	-1.61482	3.25E-12
TLX1	-1.61215	7.35E-10
SLC6A8	-1.61137	8.51E-18
TMEM238	-1.60956	4.48E-16
BIK	-1.60922	6.12E-29
TNNI3	-1.60873	5.98E-07
SYT8	-1.60732	9.01E-08
HPDL	-1.60587	5.10E-16
TMEM105	-1.59723	8.39E-17
KRT16	-1.5925	2.88E-06
CD164L2	-1.59089	5.74E-11
PKP3	-1.59068	4.94E-34
PERM1	-1.58817	5.35E-18
SPINK5	-1.58677	2.54E-11
POU5F1	-1.58553	3.56E-12

SDR16C5	-1.58337	6.59E-12
SLCO4A1	-1.57155	8.59E-17
AOC1	-1.57052	3.73E-12
TRIM31	-1.56765	4.20E-10
PIWIL1	-1.56757	3.80E-05
FOXL1	-1.56728	2.25E-15
MYEOV	-1.56496	1.48E-10
AHSG	-1.56269	1.12E-06
ALG1L	-1.56047	3.00E-12
GPA33	-1.56038	9.90E-08
SNCG	-1.55345	1.11E-10
PKP1	-1.55038	4.93E-08
CYP2S1	-1.54671	6.34E-11
LY6K	-1.54663	0.000143
BAIAP2L2	-1.54592	3.75E-15
WFDC2	-1.5435	2.59E-13
SULT2A1	-1.54299	0.008953
PHLDA2	-1.54283	1.63E-15
CCK	-1.54178	3.82E-05
EFNA2	-1.54071	1.21E-09
HCAR1	-1.53724	1.23E-09
MUC4	-1.53662	5.47E-08
CLIC3	-1.53558	6.15E-10
HOXA13	-1.53491	5.26E-05
FXYD3	-1.53429	1.76E-18
UGT2B7	-1.532	1.29E-06
DUOXA2	-1.53149	1.43E-06
CYP2C9	-1.53138	1.04E-07
S100A14	-1.52998	5.06E-11
TGM5	-1.52857	7.15E-07
NMU	-1.52758	7.82E-09
SPDEF	-1.52675	6.44E-10
TMEM82	-1.52666	9.29E-07
RNF223	-1.5228	1.04E-12
GAS2L2	-1.52275	4.14E-08
CDKN2A	-1.51681	3.73E-07
ADM	-1.51509	5.69E-15
CLCA4	-1.51406	3.50E-06
SI	-1.51368	0.007122
SLPI	-1.5101	4.41E-15
VILL	-1.50899	9.35E-16
SLC2A1	-1.50854	6.52E-16
CYP2B6	-1.5077	6.58E-06
AGR3	-1.5041	3.26E-10
PLA2G4F	-1.50173	8.20E-12
SCNN1A	-1.50001	1.66E-13
SYTL5	-1.49789	5.73E-09
ZIC2	-1.49785	6.36E-06
RBP3	-1.49707	6.32E-07
SLC5A5	-1.49662	1.63E-06
SERPINC1	-1.49467	5.09E-06
KLK3	-1.48889	0.003941
FER1L6	-1.48717	3.73E-05
TBX15	-1.48675	6.88E-10
HES2	-1.48569	2.46E-08
HOXC10	-1.48552	0.000158
MFI2	-1.48376	1.24E-13
GKN1	-1.47925	0.014607

ANXA8	-1.47841	1.79E-06
OTX1	-1.47797	1.36E-21
TEX35	-1.47722	0.00015
C2orf82	-1.47336	4.27E-20
CAPZA3	-1.47231	0.000104
ALDH3A1	-1.47157	2.76E-07
FGG	-1.46785	8.11E-05
SRMS	-1.46733	6.64E-10
SFN	-1.46384	1.27E-11
MROH6	-1.46359	1.37E-14
MUC16	-1.46355	7.69E-05
NUTM1	-1.46311	4.55E-08
ST6GALNA	-1.46311	3.16E-09
SPINK7	-1.4628	1.11E-05
KCP	-1.46272	5.93E-11
NTSR1	-1.46147	4.90E-05
ELF5	-1.46012	0.000583
OR2B6	-1.45931	5.80E-06
VSIG2	-1.45707	1.35E-10
FBN3	-1.45308	1.27E-05
FXYD4	-1.45304	2.36E-05
TINAG	-1.45125	9.65E-06
STK31	-1.45119	1.52E-10
MUC1	-1.44555	1.53E-13
HMGCS2	-1.44515	8.92E-05
FAM83E	-1.44444	8.60E-15
KRTCAP3	-1.44317	3.43E-18
OASL	-1.43992	1.61E-12
A2ML1	-1.43851	0.000688
TMPRSS4	-1.43681	1.01E-10
SLC39A4	-1.43506	4.59E-15
MYRFL	-1.43276	1.97E-07
GCKR	-1.42934	5.71E-10
BARX1	-1.42751	4.10E-05
PLEKHN1	-1.42728	2.24E-14
DHRS9	-1.4254	1.53E-05
AP006285.	-1.42436	2.53E-13
SFTA2	-1.42397	3.80E-08
GPR128	-1.42026	0.000107
ZNF488	-1.41906	3.57E-11
SPERT	-1.41836	0.000836
CBLC	-1.41764	7.86E-20
CES3	-1.41625	2.17E-08
XDH	-1.41622	5.20E-11
OVOL1	-1.41074	4.63E-11
CLRN3	-1.41052	4.40E-08
RNF39	-1.4074	3.14E-14
LGALS4	-1.40295	1.21E-08
VNN1	-1.40211	6.92E-08
RASSF7	-1.40176	4.54E-26
BCL2L14	-1.40025	3.22E-12
NR1I2	-1.4002	5.85E-07
CDHR5	-1.39864	2.17E-08
RAPGEFL1	-1.39811	1.39E-13
KRT9	-1.39423	6.18E-05
KRT19	-1.39395	2.05E-15
C2orf61	-1.3907	5.90E-08
KRT7	-1.39026	4.24E-11

EMX1	-1.38735	4.32E-05
ANKRD33	-1.38618	1.70E-05
EVPL	-1.38397	4.35E-13
ETV4	-1.38303	1.54E-17
CP	-1.38052	3.58E-07
SH2D3A	-1.38005	1.13E-19
IGSF9	-1.37992	9.39E-12
SMIM22	-1.37965	1.32E-21
HOXB13	-1.37867	0.002266
PPARG	-1.3779	4.21E-16
CITED1	-1.37773	7.71E-07
KCNN4	-1.37724	4.23E-12
ANGPTL4	-1.37573	1.27E-08
ERN2	-1.3733	4.83E-08
MSLN	-1.37285	5.77E-07
PLEK2	-1.37231	8.13E-15
KREMEN2	-1.37159	2.15E-11
AGR2	-1.37062	1.26E-09
CLCN1	-1.37059	4.83E-08
CYP2C19	-1.36792	1.05E-05
HMGA1	-1.36784	1.59E-20
KRT83	-1.36746	3.31E-05
LPCAT4	-1.36394	5.09E-24
GJB3	-1.36327	5.72E-11
PPFIA4	-1.36157	7.42E-09
COL17A1	-1.3597	2.62E-06
GPX2	-1.35963	9.90E-11
PRB1	-1.3558	0.012906
MST1R	-1.35557	3.21E-14
MISP	-1.35487	7.37E-18
HSPA6	-1.35458	2.85E-07
IRX5	-1.35366	1.86E-11
RHOV	-1.35088	3.22E-09
C8orf74	-1.35014	4.72E-07
ABHD17C	-1.3475	2.29E-20
AKR1B10	-1.34725	1.00E-05
PHACTR3	-1.34611	8.80E-07
HSD11B2	-1.34601	1.11E-09
UBE2C	-1.3449	3.04E-15
PRR15	-1.34393	2.53E-18
AQP5	-1.3435	8.66E-06
PSORS1C2	-1.34264	5.62E-08
PF4	-1.34243	1.46E-07
FABP7	-1.34024	0.000427
CYP2W1	-1.33887	1.59E-06
ALDH3B2	-1.33868	1.79E-06
DNAH2	-1.33815	2.92E-10
FA2H	-1.33685	3.47E-14
TNNT2	-1.33323	2.07E-08
ZSCAN4	-1.3325	2.38E-06
PLA2G2F	-1.33232	0.001758
CRACR2B	-1.32918	7.68E-13
PRSS21	-1.32907	8.40E-05
GSDMB	-1.32781	8.76E-15
HIST1H1D	-1.32718	1.04E-05
IFITM10	-1.32708	1.77E-11
IL1R2	-1.327	7.64E-07
LCN1	-1.32205	6.29E-06

EPS8L1	-1.32048	2.18E-10
UPK2	-1.32012	0.000314
ADAMTSL!	-1.31582	6.71E-16
C20orf85	-1.31518	0.00294
GPR35	-1.31502	3.86E-13
WISP3	-1.31445	0.000165
NUDT8	-1.31391	5.24E-18
SLC26A5	-1.31284	1.56E-10
FUT3	-1.31097	8.09E-11
ALS2CL	-1.31094	1.15E-15
SCGB1A1	-1.30888	0.024446
SERINC2	-1.3083	3.00E-19
GIP	-1.3062	0.000999
S100A6	-1.30462	3.98E-18
HIST1H2A	-1.30355	1.99E-07
PLAC8	-1.3016	2.83E-10
LAMB3	-1.30067	6.32E-11
SAPCD2	-1.30063	3.58E-18
HOXC11	-1.29625	0.000672
SPRR1B	-1.29428	0.002147
AIM1L	-1.29195	1.07E-10
SLC17A9	-1.29122	1.29E-13
EPS8L3	-1.29118	8.63E-09
ADORA2B	-1.29078	8.66E-15
ANXA8L1	-1.28983	5.04E-06
SLC22A18	-1.28845	6.72E-18
HIST1H2BI	-1.28743	6.55E-05
PSCA	-1.28742	0.000632
MYO1A	-1.2874	8.23E-06
UGT1A10	-1.28629	3.70E-05
KRT17	-1.2862	1.35E-05
HK2	-1.28567	2.40E-14
CATSPER1	-1.2848	1.04E-11
CDC42EP5	-1.28218	2.00E-15
LBX2	-1.28179	1.55E-17
LEMD1	-1.28075	3.77E-07
PPP1R1B	-1.28054	1.32E-06
GRB7	-1.27873	8.66E-16
TMPRSS11	-1.27785	0.001046
TNS4	-1.27615	1.16E-05
TJP3	-1.27402	5.10E-14
B3GNT3	-1.27362	1.43E-17
ITGB4	-1.27314	8.84E-14
GPT	-1.2727	9.51E-09
ANO7	-1.27113	2.41E-08
FOXD1	-1.27108	5.40E-07
PVRL4	-1.27034	1.60E-09
PADI1	-1.27032	0.000428
ZG16B	-1.26967	1.89E-07
C16orf74	-1.26871	1.91E-07
SPDYC	-1.26782	1.56E-08
CDHR2	-1.26732	4.69E-07
SOX21	-1.26652	0.000122
PSORS1C1	-1.26626	1.99E-10
LIPM	-1.26557	1.10E-10
CTAGE15	-1.26553	0.000105
HOXA10	-1.26469	1.28E-05
PLA2G4D	-1.2634	4.10E-11

TSPAN1	-1.26335	3.88E-13
LRRIQ4	-1.2628	1.24E-05
IQGAP3	-1.26154	1.99E-13
REP15	-1.26125	4.79E-07
BCAS1	-1.26079	1.31E-07
HIST2H2BI	-1.26059	1.32E-07
BEST4	-1.25748	1.97E-11
KRT4	-1.25493	0.001079
TMEM253	-1.25431	2.13E-08
CTSV	-1.25362	1.24E-09
ARMC3	-1.25359	4.99E-06
MIA	-1.25334	3.48E-05
KRTAP5-1	-1.25159	9.32E-10
BEAN1	-1.25114	2.02E-09
FAM83H	-1.25102	9.21E-26
ATP6V1B1	-1.24973	7.10E-09
DMBX1	-1.24582	3.63E-05
EFNA1	-1.2454	1.13E-17
MLPH	-1.2444	2.28E-17
CRCT1	-1.24396	0.001458
NQO1	-1.24315	4.08E-15
TST	-1.2429	7.00E-15
BAIAP2L1	-1.23963	1.24E-23
MAPK15	-1.23961	1.03E-08
GDPD2	-1.23914	3.03E-07
DCST2	-1.23776	3.32E-14
LRRC66	-1.23596	5.66E-08
PTGES	-1.23581	5.28E-08
MMP1	-1.23539	0.000129
NXF3	-1.23508	0.000225
PI3	-1.23416	2.87E-05
CABP4	-1.23344	2.99E-16
CAPN9	-1.23075	5.04E-05
SPIRE2	-1.22979	1.16E-12
CST6	-1.22977	7.18E-05
MMP28	-1.22726	1.06E-11
ELMO3	-1.22682	4.95E-29
DEGS2	-1.22651	1.51E-09
LYPD3	-1.22609	1.22E-06
CDC20	-1.22574	2.75E-13
PRB3	-1.22567	4.61E-06
AC114494	-1.22001	3.11E-10
RAB25	-1.21902	2.87E-19
RHOD	-1.21817	1.09E-12
ANKRD22	-1.21578	3.40E-11
EREG	-1.21557	0.000182
SDCBP2	-1.21531	1.63E-13
DUOXA1	-1.21526	3.69E-07
CDA	-1.21524	3.12E-08
HIST3H2BI	-1.21449	2.31E-08
DKK1	-1.21441	4.57E-05
FAM83C	-1.21417	0.016088
ATG9B	-1.21325	3.18E-08
CLDN4	-1.2126	8.80E-14
SLC16A3	-1.21106	1.07E-13
PRB2	-1.21078	0.005089
OFCC1	-1.21048	0.001835
TMPRSS7	-1.21009	3.65E-09

GDPD3	-1.20975	3.14E-14
ICAM5	-1.20901	9.38E-07
CCDC64B	-1.20653	1.86E-12
ID1	-1.20647	1.15E-14
RDM1	-1.20554	2.24E-12
CREG2	-1.20433	2.79E-09
KRT12	-1.20303	0.000268
SLC23A3	-1.20257	1.29E-11
S100A5	-1.20198	1.44E-13
OMP	-1.20162	1.39E-07
GALE	-1.20134	2.31E-21
B3GNT7	-1.19831	5.87E-10
EXOC3L4	-1.19738	1.26E-09
TK1	-1.19562	6.21E-15
ANO9	-1.19505	1.49E-13
CDT1	-1.19414	5.64E-17
CDH3	-1.19334	3.54E-10
TM4SF5	-1.19038	2.93E-05
LY6E	-1.18898	5.43E-13
GMDS	-1.18883	5.11E-13
CDC25C	-1.18785	1.13E-15
IFI27	-1.18769	9.27E-10
SHH	-1.18737	3.83E-07
ENSG0000	-1.18658	4.57E-10
PRSS8	-1.18575	3.77E-10
FOXL2NB	-1.18523	0.013603
ACY1	-1.18513	9.51E-22
TACSTD2	-1.18512	3.88E-09
CYP3A5	-1.18359	7.97E-09
SH2D5	-1.18277	3.16E-07
SLC9A3	-1.18245	4.38E-05
DCST1	-1.18202	4.17E-12
TROAP	-1.18135	5.09E-15
HRASLS2	-1.17986	1.93E-05
UPK3B	-1.17833	0.000288
C9orf152	-1.17726	2.05E-07
LAD1	-1.17484	1.39E-15
GPRC5A	-1.1744	7.90E-08
SPC24	-1.17226	4.34E-16
C2CD4D	-1.17134	6.44E-11
SEMA4B	-1.17081	3.65E-21
KBTBD12	-1.16921	2.39E-05
CCDC42B	-1.16877	1.58E-07
MKRN2OS	-1.16833	2.20E-19
SLC25A10	-1.16669	5.85E-18
TMEM92	-1.16492	3.50E-10
SYCE3	-1.16398	2.46E-08
MCIDAS	-1.16369	5.42E-10
ELF3	-1.16335	2.01E-14
AGMO	-1.1624	9.77E-06
TRIM29	-1.16202	2.44E-05
LRFN4	-1.16167	5.75E-15
CYP4F12	-1.16122	4.20E-08
TP73	-1.16033	7.29E-13
STYK1	-1.15539	1.89E-11
IL36B	-1.15377	0.01638
CTAGE4	-1.15333	1.66E-07
ENSG0000	-1.15305	0.000461

STAP2	-1.15276	6.11E-19
MTMR11	-1.15255	3.69E-14
YBX2	-1.15133	3.13E-05
ENSG0000	-1.15102	2.42E-06
IVL	-1.15078	0.021075
SERPINB5	-1.14787	4.41E-06
RBBP8NL	-1.1457	1.09E-08
GCNT3	-1.14558	3.55E-07
TP63	-1.14529	6.35E-05
PRAP1	-1.14395	0.001141
PADI3	-1.14349	0.002015
GJB5	-1.14311	8.81E-05
PLK1	-1.14224	2.25E-19
CAPN12	-1.1416	9.73E-09
C19orf67	-1.14079	5.50E-07
GPR20	-1.14051	3.73E-06
BATF	-1.14002	1.34E-12
HAS3	-1.13966	8.99E-07
CA14	-1.13928	4.23E-10
KLF5	-1.13873	2.74E-13
TMEM213	-1.1378	0.000243
PCSK9	-1.13757	3.00E-07
FOXM1	-1.13721	9.57E-12
TESC	-1.13658	8.99E-10
TSPO	-1.13414	6.61E-15
WNT11	-1.1338	0.000256
F12	-1.13334	6.57E-10
HYAL4	-1.13286	3.11E-05
PIK3C2G	-1.13209	0.000286
TMEM171	-1.13104	1.07E-09
MALL	-1.13093	2.59E-09
CA12	-1.1299	1.53E-06
MEP1A	-1.12974	0.001939
ABHD11	-1.12959	2.58E-28
VSIG1	-1.12953	0.000617
CENPM	-1.12893	6.98E-11
CLDN7	-1.12833	2.35E-19
CRABP2	-1.12796	1.15E-05
IL17RE	-1.12775	2.79E-15
NAPRT	-1.12711	7.17E-14
SNTN	-1.12667	3.65E-07
SLC26A9	-1.12658	0.000371
KRTAP5-1	-1.12595	8.19E-06
ATP6V0A4	-1.12561	6.62E-05
CDCA3	-1.12443	7.90E-17
XKR9	-1.12378	5.73E-09
C1orf177	-1.12263	1.42E-12
IGF2BP2	-1.12161	3.87E-13
CAPS	-1.12084	1.29E-13
BPIFB1	-1.11908	0.004582
LSR	-1.11875	7.19E-19
SLC4A11	-1.11836	3.93E-09
DKK4	-1.11694	0.00033
CDH16	-1.1167	0.000865
C11orf85	-1.11475	0.000215
KRTAP4-1	-1.11439	0.04802
GNGT1	-1.11378	0.042643
UNC93A	-1.11186	0.000359

HOXC9	-1.11079	3.70E-06
FOXQ1	-1.11055	2.15E-10
IL36G	-1.10958	0.00269
DNAH3	-1.10875	7.18E-09
KRT18	-1.10798	1.05E-17
ALPP	-1.10753	0.007043
TRIM10	-1.10714	7.52E-08
GJB4	-1.10693	8.72E-06
LMNTD2	-1.1067	2.13E-11
TRNP1	-1.10582	6.49E-12
LFNG	-1.10548	2.47E-14
IRX3	-1.10542	1.34E-05
AGPAT2	-1.10424	4.73E-19
NXNL2	-1.10398	1.16E-10
MFSD2B	-1.10377	6.65E-12
CDH17	-1.10315	0.000408
NEK2	-1.10164	2.54E-12
S100A16	-1.10051	2.69E-15
HOXA11	-1.09927	0.002135
C11orf53	-1.09829	4.35E-06
LGALS9C	-1.09673	0.000144
NOXA1	-1.09658	6.30E-10
OVOL2	-1.09544	3.52E-17
FFAR4	-1.09516	1.33E-05
CTAGE8	-1.09463	1.07E-05
ERO1L	-1.09255	6.00E-19
RP11-295I	-1.09201	7.49E-09
E2F7	-1.09179	3.97E-09
FAM72C	-1.09179	2.15E-09
LGALS9B	-1.09064	0.001097
MUC3A	-1.09058	3.49E-05
KRT3	-1.09029	0.000653
HJURP	-1.08993	1.01E-12
ATP2C2	-1.08963	1.60E-09
KLK5	-1.08935	0.025334
BBC3	-1.08885	6.67E-15
HIST1H3G	-1.0882	0.000295
FAM132A	-1.08815	1.28E-07
STX19	-1.08815	8.13E-09
SYT12	-1.08765	1.05E-06
HS3ST5	-1.08749	0.000151
EFNA4	-1.08747	5.04E-15
FUT6	-1.08679	9.01E-08
SP5	-1.08668	8.50E-06
DMBT1	-1.08665	0.0021
PPAP2C	-1.08646	6.03E-13
REEP6	-1.08599	3.32E-11
BARX2	-1.08536	8.04E-09
PLEKHG6	-1.08508	2.43E-14
STRC	-1.08487	3.15E-06
ASPHD2	-1.08474	2.56E-13
NPIPB15	-1.08457	0.000616
ASPG	-1.0845	4.39E-06
LGR6	-1.08423	4.33E-05
ZNF750	-1.08385	4.37E-05
SULT1E1	-1.08322	0.001517
ACSL5	-1.08264	1.40E-10
GDF15	-1.08241	6.98E-06

HIST1H3H	-1.08241	9.70E-06
SULT2B1	-1.08202	1.28E-06
NOX1	-1.08075	6.44E-05
PKMYT1	-1.07916	1.42E-11
CAPN5	-1.07768	4.96E-12
EPHX4	-1.07588	6.35E-10
CCNJL	-1.07485	5.31E-09
LGALS3	-1.07435	6.19E-16
SLC16A5	-1.07431	6.92E-10
UBALD2	-1.07408	1.60E-23
CCNB2	-1.07402	6.91E-13
KPNA7	-1.07367	1.84E-07
TH	-1.07348	0.001983
MMEL1	-1.07262	1.59E-09
TSTA3	-1.07228	2.99E-17
PTPRR	-1.07227	1.13E-06
PLCD3	-1.0704	1.39E-13
GREB1L	-1.07025	3.08E-07
ABCG5	-1.06908	0.000169
MUCL1	-1.06786	0.02282
TMC7	-1.06765	1.21E-11
ITGA3	-1.06642	1.51E-13
SIM2	-1.06572	9.63E-08
GALNT6	-1.06386	1.56E-09
EPHA2	-1.06374	1.61E-09
TINAGL1	-1.06349	1.87E-14
TMEM45B	-1.06295	3.70E-09
GPR37L1	-1.06161	2.34E-08
OIP5	-1.06108	3.63E-15
RPTN	-1.06044	0.042461
NPAS1	-1.06019	1.18E-09
ALG1L2	-1.05964	1.00E-12
ARHGAP4(	-1.05931	0.002876
ESRP2	-1.0587	9.46E-21
C2orf70	-1.05826	7.63E-07
EPS8L2	-1.05826	8.77E-22
BIRC5	-1.0581	1.98E-10
ORM2	-1.05802	0.000322
EN2	-1.05764	0.016723
MPST	-1.05753	6.34E-15
SMCO2	-1.05631	2.65E-11
CCL20	-1.05624	0.000219
MAL2	-1.05543	5.35E-14
SAMD10	-1.055	1.17E-23
S100A4	-1.05498	5.81E-06
PRSS22	-1.0542	3.79E-09
DUOX2	-1.05312	0.000466
CYP3A4	-1.05311	0.001324
CYP2F1	-1.05281	0.000348
SPINT1	-1.05198	7.41E-27
CCNO	-1.05194	8.57E-11
C1orf106	-1.05144	2.87E-08
KRT8	-1.05119	2.94E-17
EBP	-1.05048	8.96E-31
CDHR4	-1.05034	0.000202
SLC45A3	-1.05	5.25E-10
CNTD2	-1.04985	0.000248
NAT2	-1.04971	0.000698

S100A10	-1.04868	4.47E-18
CNTNAP2	-1.04772	0.000159
PTTG1	-1.04713	5.77E-16
TMC5	-1.04622	4.54E-08
NGEF	-1.0461	7.56E-10
HR	-1.04598	5.28E-10
HS3ST1	-1.0458	5.21E-09
C15orf48	-1.04559	5.18E-09
R3HDML	-1.04534	0.000113
KCNK1	-1.04441	8.81E-14
MICALL2	-1.04284	7.97E-15
C12orf56	-1.04254	0.000989
KLHL30	-1.04253	1.34E-07
POC1A	-1.04126	1.51E-19
ALDH3B1	-1.04088	3.00E-13
TONSL	-1.04084	1.21E-16
MUC17	-1.04027	0.012962
TMPRSS11	-1.04014	0.004672
TFAP2A	-1.03965	9.65E-08
GRTP1	-1.03886	5.07E-14
CEP55	-1.03847	1.06E-11
FAM150A	-1.03846	8.34E-05
ACRV1	-1.03757	3.58E-08
S100A11	-1.03704	1.60E-14
CDSN	-1.03679	2.07E-05
HKDC1	-1.03384	3.91E-08
ATHL1	-1.03212	1.79E-06
ARRDC1	-1.03201	4.14E-22
PDZD3	-1.03195	0.00021
TRIM16	-1.03141	1.54E-13
PPP1R13L	-1.03136	4.84E-13
ARHGEF39	-1.03037	5.34E-15
EPHA7	-1.02949	0.001221
CDX2	-1.02711	8.41E-05
MKI67	-1.02609	1.89E-11
CYP4F2	-1.0246	0.004328
GOLT1A	-1.02412	1.37E-08
TSPAN8	-1.02369	3.74E-06
PPDPF	-1.02256	1.08E-18
ARHGEF16	-1.02195	9.58E-16
TMEM139	-1.02137	4.15E-10
KCNK7	-1.01979	2.07E-08
IGF2BP1	-1.01941	0.003767
CKS2	-1.01898	8.51E-15
OOEP	-1.01788	0.004762
OCIAD2	-1.01737	7.87E-20
GRAMD2	-1.01664	1.48E-08
TSPAN15	-1.01635	2.48E-16
ESRP1	-1.01465	1.78E-17
PLLP	-1.01415	3.77E-11
GABRE	-1.01349	6.86E-07
CACNA1F	-1.01348	1.33E-08
CXCL17	-1.01316	0.001045
AQP6	-1.0127	0.000335
SLC44A4	-1.01171	7.63E-09
SLC13A5	-1.0116	0.00381
TMEM54	-1.01067	1.41E-18
CKMT1B	-1.00951	5.12E-08

EFNB1	-1.0091	3.19E-14
ISG15	-1.00882	4.82E-06
PLAC1	-1.00865	0.002715
CYP4F8	-1.00823	0.001371
MET	-1.00616	4.97E-10
KLK10	-1.00607	0.000279
HIST3H2A	-1.00567	4.30E-11
ST14	-1.00544	2.03E-19
TPX2	-1.00532	1.10E-10
HIST2H4A	-1.00473	7.13E-07
SLCO1B3	-1.00438	0.018492
FOXA3	-1.00366	3.86E-07
CRIP1	-1.0034	6.73E-11
TFR2	-1.00331	3.56E-06
CTSE	-1.0024	0.000211
ZNF695	-1.00196	8.46E-05
CKMT1A	-1.00107	9.01E-09
CENPA	-1.00039	1.96E-10
MAT1A	1.000159	0.001158
GLT8D2	1.001074	4.60E-14
HRNR	1.001109	4.84E-07
BTK	1.001196	2.73E-08
EPHB1	1.001569	1.58E-09
XG	1.001587	0.000145
ADAM12	1.001763	1.22E-05
ZFHX4	1.001885	1.68E-09
BEND6	1.002131	1.27E-11
IL1R1	1.002672	1.98E-14
SOX17	1.002833	1.68E-12
PABPC4L	1.003122	1.15E-14
SAMHD1	1.003348	2.05E-18
MZB1	1.00341	6.77E-05
NR4A1	1.003777	7.99E-07
MARK1	1.003892	2.82E-13
CADM1	1.003895	2.01E-12
RAB3B	1.004191	2.56E-07
CD180	1.004525	1.65E-07
PRKD1	1.00504	9.25E-15
MROH8	1.006208	2.26E-14
GSTA2	1.006624	0.01323
KLHL32	1.006982	0.000108
LPPR5	1.007035	1.32E-05
CHRNA1	1.007521	1.25E-05
HS3ST2	1.00856	1.00E-05
F7	1.008606	4.71E-05
SCUBE2	1.010047	1.91E-09
LPAR1	1.010585	3.16E-17
ATP1A4	1.010598	1.11E-05
EDA2R	1.01061	5.64E-14
CRTAM	1.010858	1.50E-08
C11orf21	1.010919	1.06E-06
PCLO	1.01181	9.89E-06
CHODL	1.012265	7.99E-08
SLCO2B1	1.012358	2.53E-12
MAP4K1	1.012609	8.24E-07
3-Sep	1.01273	5.56E-08
ZNF835	1.012882	1.94E-11
SIRPG	1.012891	3.82E-06

GPC6	1.013333	6.09E-10
CAPSL	1.013481	0.001539
IGSF21	1.014703	1.13E-09
SHISA3	1.014713	5.27E-06
UGT2B4	1.015217	0.028522
DOCK11	1.015806	5.09E-18
MEOX1	1.01587	5.45E-08
SLC22A16	1.016363	1.90E-06
MS4A6A	1.016957	1.45E-11
ASB16	1.017203	2.20E-09
EVI2A	1.017236	1.20E-10
SH3GL3	1.018329	0.000634
LEFTY2	1.018425	1.75E-06
GPR150	1.018705	9.15E-07
FBXL7	1.019572	3.31E-19
RD3	1.019838	8.40E-05
GCSAML	1.020047	3.12E-08
CD2	1.020061	8.79E-08
C21orf62	1.020339	0.00043
MAGI2	1.022711	7.26E-15
TTLL7	1.022732	2.89E-10
ELN	1.02363	4.52E-11
PGBD5	1.025129	3.32E-10
PCDH8	1.02566	0.000519
LILRA4	1.025901	0.000198
DBX2	1.026359	8.87E-05
CSF1R	1.027224	3.25E-13
PIK3R5	1.027281	1.18E-10
NTRK1	1.027426	4.77E-13
CD96	1.028357	1.25E-07
SEMA6D	1.028663	6.41E-13
PPY	1.02894	0.041572
ADAM22	1.029151	2.80E-15
SLC18A3	1.029153	0.001292
PLXNC1	1.029219	2.10E-16
CLEC3B	1.029346	4.37E-10
PREX1	1.029401	1.56E-20
PDE8B	1.029618	1.25E-13
SLC46A2	1.030702	3.04E-09
MPZ	1.031169	1.18E-09
AVPR1A	1.031443	2.48E-08
MRGPRF	1.031649	1.99E-09
PZP	1.032863	2.06E-10
MMP24	1.032994	5.81E-10
SLA	1.033001	6.57E-10
NGF	1.033287	1.82E-09
IL12B	1.033453	8.62E-06
ARPP21	1.033542	0.000368
GPR65	1.033662	7.04E-12
SMOC2	1.033677	1.75E-10
ARHGAP1!	1.035374	4.02E-10
SUSD5	1.035876	5.71E-10
GUCY1A2	1.036018	1.22E-13
MNDA	1.037565	1.03E-09
FPR1	1.03804	1.70E-09
SCGB2A1	1.038144	0.00027
TDRD10	1.038792	9.58E-13
CACNA1H	1.039052	2.39E-12

SLFN14	1.039273	0.000507
RGS8	1.039646	7.92E-08
MAGEH1	1.040513	1.01E-19
ADAM23	1.040828	1.97E-11
GPRIN3	1.041122	2.80E-15
BTBD11	1.04138	2.35E-06
AOC3	1.041592	1.50E-12
PLXDC2	1.041899	9.70E-15
DENND2A	1.041944	6.93E-18
PCDHGA1	1.042031	2.53E-15
INS	1.04248	0.022324
LOXHD1	1.042607	9.48E-14
KCNAB1	1.042692	3.43E-12
PALMD	1.042762	1.89E-15
ZNF804A	1.042792	5.90E-11
KCTD12	1.043162	2.58E-20
FAM124A	1.043376	3.09E-16
MYO18B	1.043436	2.95E-05
FAM19A1	1.043576	1.63E-05
KLHL34	1.043675	8.17E-06
C9orf135	1.043979	0.000404
KRT1	1.044116	0.001055
KIF6	1.044361	1.21E-09
SLC16A10	1.044378	2.54E-08
WSCD1	1.04491	4.04E-10
DTHD1	1.045674	2.24E-05
BEND4	1.046255	0.001265
FGFR1	1.046524	5.72E-28
ZNF660	1.047764	1.30E-24
SYN1	1.04795	4.28E-07
ARHGEF15	1.048312	3.12E-20
RYR3	1.048623	1.06E-11
CD93	1.048862	1.98E-22
HMCN1	1.049448	1.39E-09
RBMS3	1.049871	4.44E-21
PPAP2B	1.049917	2.66E-33
SIGLEC8	1.050043	1.58E-06
FBN1	1.050588	2.09E-10
EML1	1.050675	5.34E-21
AFF2	1.051406	2.55E-08
TCF4	1.051604	2.83E-24
MYH10	1.052281	1.58E-23
CCR1	1.052335	6.00E-12
NLRP3	1.052344	4.67E-12
ZEB2	1.052386	2.61E-19
C19orf81	1.053389	4.20E-05
S100B	1.053446	1.78E-09
CLMP	1.054222	2.43E-08
TMC3	1.054447	1.04E-08
PCYT1B	1.054518	7.15E-09
TMOD2	1.054939	4.32E-21
C10orf90	1.055004	5.04E-05
TWIST2	1.055521	3.04E-12
SASH3	1.056971	5.67E-09
SPAG6	1.057452	0.000529
NETO1	1.057656	0.00024
GSTM5	1.05797	1.32E-15
TSPAN19	1.058999	0.008595

CD99L2	1.059104	1.23E-17
NFATC2	1.059402	1.70E-15
ITGBL1	1.060017	1.59E-07
RHOH	1.060225	5.44E-07
CLU	1.060375	1.21E-07
TMEM132I	1.060514	8.04E-10
TFEC	1.060619	2.49E-09
DPEP3	1.06115	4.09E-05
GIMAP1	1.061259	1.02E-14
RGL1	1.061422	7.29E-31
CST7	1.061477	8.26E-11
BEX4	1.06167	6.49E-12
RBP7	1.061804	3.78E-10
C1QTNF7	1.061935	2.01E-10
STEAP1B	1.062459	9.29E-08
DDR2	1.063712	2.08E-13
TCEAL7	1.063811	3.55E-12
ALLC	1.063818	7.04E-06
ZNF492	1.064319	7.50E-10
BACE1	1.064735	2.78E-19
PTPRN2	1.064861	5.28E-06
TMCC2	1.065016	5.19E-16
KCNN2	1.065086	4.59E-10
TMEM151/	1.065168	7.30E-05
CLEC4E	1.065178	7.56E-07
ZNF521	1.065183	1.65E-15
TECTA	1.06547	3.89E-22
BEST3	1.065515	0.000188
MYCT1	1.065927	5.50E-21
DCDC1	1.066075	9.16E-08
LRRK2	1.066326	2.71E-11
CCL4	1.067197	6.31E-11
CDKL2	1.06722	3.03E-07
NXPH3	1.067476	1.10E-11
PAH	1.067502	0.002748
MYRIP	1.068499	4.27E-07
ENSG0000	1.068876	9.18E-11
TAL1	1.069796	7.37E-18
KLHL6	1.069855	2.08E-08
CXCR2	1.070188	4.47E-05
NPPC	1.070623	0.003581
PPM1K	1.070844	8.83E-20
FAM78A	1.070941	2.39E-13
CCL2	1.071124	2.18E-08
GFRA1	1.071181	3.00E-06
ACACB	1.071468	9.12E-16
PARM1	1.071505	2.24E-07
NCKAP1L	1.071506	4.54E-11
MS4A4E	1.071777	1.16E-09
ITGAL	1.071902	2.64E-08
FXYD1	1.073083	2.77E-11
CD69	1.073293	1.26E-06
CCL8	1.07361	2.14E-05
MUM1L1	1.074556	6.64E-06
PTPN5	1.074629	1.49E-06
CPNE4	1.076001	3.92E-05
PDZRN3	1.076258	8.94E-18
NUPR1L	1.077607	0.000282

CD163	1.077883	1.40E-07
GNG7	1.078275	1.78E-10
SLC25A53	1.078307	6.32E-18
CCDC151	1.078636	2.24E-07
SYNE3	1.079071	4.40E-18
DPPA4	1.079186	1.83E-05
IKZF3	1.079425	1.03E-06
GPNMB	1.079462	3.69E-11
FAM110D	1.079845	1.29E-11
KDR	1.080631	8.93E-24
CETP	1.080688	1.85E-09
GPR112	1.080958	0.000115
SLCO4C1	1.08116	7.74E-05
GPR183	1.081184	4.73E-09
ATP8A1	1.082126	4.04E-10
GABRA5	1.082518	0.000179
OTUD7A	1.082695	4.20E-19
RGS11	1.083249	7.67E-08
RNASE6	1.084452	2.63E-12
BACH2	1.084489	1.72E-11
RERGL	1.085769	1.23E-05
IL10RA	1.086613	9.55E-15
ATP8B4	1.087123	1.88E-18
ATP8B2	1.088637	2.40E-28
CYP7B1	1.091034	2.12E-13
SLC5A4	1.091875	7.67E-09
CPXM1	1.091993	1.61E-09
DOK6	1.092171	8.00E-15
GPR123	1.092738	0.000113
FCER1A	1.092936	5.68E-07
LPAR4	1.093703	1.26E-13
ENSG0000	1.094063	0.000927
PDE1A	1.094871	3.43E-21
LRRC15	1.09604	0.000135
CYP19A1	1.096462	5.15E-08
CD37	1.096691	2.37E-07
MAB21L1	1.097531	2.90E-10
FPR3	1.097694	9.80E-10
CPED1	1.097897	6.20E-11
HCN4	1.097917	1.07E-05
FLI1	1.098519	5.64E-18
GAS7	1.099009	2.35E-21
CCDC69	1.099289	1.77E-11
ZAP70	1.099299	4.00E-07
GRAP2	1.099512	1.95E-13
ITGA7	1.100088	1.15E-13
TRPC4	1.100218	2.79E-12
THSD7A	1.10034	1.21E-14
CHRM5	1.1011	7.39E-13
KY	1.101823	1.04E-05
HSD11B1	1.101954	6.58E-11
PTGDR	1.102158	8.56E-13
FBLN2	1.102217	1.37E-12
COL4A6	1.102937	6.78E-07
NYAP1	1.102966	3.76E-08
MS4A4A	1.103194	8.28E-11
GFPT2	1.103366	6.64E-11
MPDZ	1.104483	1.58E-23

ITPR1	1.104713	7.47E-23
CYYR1	1.105156	3.36E-23
SCML4	1.105207	2.88E-08
TBX5	1.105492	0.000618
LHFP	1.105811	2.26E-24
LRRC70	1.106261	4.28E-16
TM6SF1	1.106483	6.59E-16
NLRP1	1.109211	1.25E-11
SCIMP	1.109808	4.24E-10
MFAP5	1.110108	5.68E-07
CD3E	1.11082	8.50E-08
CERKL	1.112022	2.04E-18
EML6	1.112426	2.35E-11
KCNH1	1.112579	1.05E-09
ACTN3	1.113205	4.89E-07
CTRL	1.113758	0.007228
CXorf21	1.113795	3.04E-12
ARHGEF6	1.11383	1.98E-20
DLG4	1.113853	1.46E-14
NPR1	1.115673	6.85E-18
NEURL1	1.116367	0.00012
IL10	1.116733	2.27E-10
CRISPLD1	1.116743	3.12E-14
PRSS35	1.116874	1.53E-10
DOCK3	1.117726	7.50E-08
KCTD8	1.118157	0.000492
IGSF10	1.118275	2.46E-08
GZMM	1.118322	6.17E-08
PRRT3	1.11851	1.36E-10
UPB1	1.11853	5.24E-09
C15orf26	1.118662	1.71E-06
SOCS2	1.118977	4.49E-25
SV2C	1.119851	4.29E-08
DPY19L2	1.120058	1.42E-08
TSPAN33	1.122001	5.91E-12
GPAM	1.122465	2.56E-16
A1BG	1.122507	6.83E-09
CPT1C	1.123052	4.75E-18
NKX6-1	1.123381	2.43E-05
SCN11A	1.123422	1.17E-10
PIK3CG	1.123684	1.95E-10
CD84	1.124011	1.79E-10
PACRG	1.12421	4.36E-07
ERG	1.124349	6.81E-24
FGL2	1.124542	4.18E-11
CX3CR1	1.12459	1.74E-08
GABRB2	1.124689	1.65E-06
TMEM108	1.125058	3.14E-17
ABCB5	1.125602	2.83E-07
NKAIN1	1.125934	7.00E-08
ST8SIA1	1.125982	5.30E-11
EPHA3	1.126076	5.41E-11
ATP2B2	1.126336	1.16E-07
RUNX1T1	1.127524	6.99E-21
LRRC4C	1.127763	1.27E-11
FLRT2	1.127912	2.54E-08
RSPH4A	1.128826	1.82E-07
TLR8	1.129415	9.16E-08

OLFML3	1.129466	1.11E-15
KCNG3	1.129683	2.49E-06
DOCK2	1.129699	3.83E-11
NUGGC	1.130528	1.37E-06
ELMO1	1.130805	6.19E-14
ASPN	1.130988	8.26E-11
FGF9	1.131136	4.02E-10
STAT4	1.131169	6.94E-15
EGR3	1.131503	3.97E-10
FAM20A	1.13152	8.02E-14
FAT4	1.132504	5.16E-14
CD302	1.132938	1.23E-19
CCDC178	1.13318	6.17E-08
GREB1	1.133425	1.78E-13
ASCL1	1.13383	9.67E-05
CLUL1	1.134681	3.29E-08
ANKRD55	1.134843	2.85E-06
NGFR	1.134846	1.34E-06
ID4	1.136166	1.98E-20
FAM171B	1.136629	4.99E-17
CD8A	1.136787	3.03E-09
CAMK2N2	1.137217	1.63E-05
LMOD1	1.13742	9.94E-09
HSPB8	1.138678	2.72E-13
ERVFRD-1	1.139427	6.41E-09
MICU3	1.140219	5.27E-17
LILRA1	1.140456	2.03E-12
SFRP2	1.140931	3.56E-08
TBL1Y	1.140959	0.027333
USP51	1.141684	3.93E-22
KLRB1	1.141902	1.21E-09
WDR63	1.142037	1.38E-08
EPYC	1.142801	0.006093
CTTNBP2	1.142954	3.82E-12
KIAA1377	1.143003	2.07E-12
TENM3	1.143226	8.16E-16
C10orf105	1.143279	1.26E-07
ASB2	1.14418	8.10E-07
MCF2L2	1.144485	2.72E-13
SULT1C4	1.144808	3.11E-14
FAM169A	1.145099	8.53E-10
SLAMF1	1.14527	2.69E-08
TIGIT	1.145896	4.13E-07
TIAM1	1.146832	8.87E-16
HSD17B14	1.146943	7.93E-14
CD27	1.147541	4.21E-07
CECR1	1.148168	1.81E-13
TAGAP	1.148301	2.00E-10
ECM2	1.149312	2.50E-18
RBFOX3	1.150111	1.59E-06
PLCL1	1.150313	1.27E-24
PCDHB5	1.151247	4.97E-11
CD226	1.152208	1.08E-12
DLC1	1.152797	7.21E-31
CCL11	1.152964	1.81E-08
ICOS	1.153084	2.99E-07
SYT9	1.155305	5.31E-06
GCH1	1.155444	2.45E-12

KCND3	1.155789	3.62E-12
EYA4	1.155965	8.31E-08
CD5	1.156406	2.32E-09
TMEM170I	1.156524	5.47E-29
GYPE	1.157195	9.35E-16
GUCY1A3	1.157387	1.10E-17
SAMD11	1.157419	9.16E-14
NEFH	1.157529	4.60E-11
C1QTNF9	1.157669	3.20E-08
ARNT2	1.158002	1.00E-13
TMEM155	1.158399	1.29E-07
PDE2A	1.158785	3.98E-18
SLC45A2	1.158823	4.53E-07
CD3G	1.159	3.08E-08
BNC2	1.159131	2.78E-12
BEX2	1.160029	8.95E-07
MADCAM1	1.160201	2.32E-06
TMEM150	1.16051	3.92E-22
PABPC5	1.160642	1.35E-17
NRROS	1.160656	2.23E-21
ASXL3	1.160811	5.74E-14
KLRG1	1.161086	1.32E-15
THBS1	1.161184	2.83E-10
PODN	1.161465	1.11E-17
PRPH	1.161691	4.52E-06
EBF1	1.163267	1.36E-21
CCDC184	1.163429	7.20E-10
ZNF781	1.163815	1.15E-17
BRINP1	1.164059	1.28E-09
GXYLT2	1.16448	7.18E-13
KCTD4	1.164578	0.000551
SIT1	1.165256	8.43E-07
ARG2	1.16576	1.73E-10
TRAF3IP3	1.166535	5.54E-09
NCR3	1.166589	3.13E-06
CD200R1	1.166835	1.34E-12
MRC1	1.167392	1.22E-08
COL21A1	1.167834	1.10E-10
RASA4B	1.167865	1.33E-06
CCDC110	1.168034	1.45E-10
SPSB4	1.168642	3.44E-08
CCR8	1.168666	2.15E-07
KLRK1	1.168693	2.22E-08
KRT40	1.169314	0.000148
GPR6	1.169994	0.017375
RECK	1.170406	8.92E-29
CRISPLD2	1.170556	8.32E-16
PCDH17	1.170704	1.80E-19
GPR158	1.170719	5.85E-06
HDC	1.171147	9.72E-10
C2orf73	1.171277	1.03E-07
SEMA5A	1.171818	2.95E-19
PSD	1.173999	9.12E-11
PREX2	1.174056	8.06E-20
SHANK1	1.174508	1.70E-09
IL16	1.175592	7.14E-12
TGFBR3	1.175935	2.35E-18
PDE4B	1.176658	6.86E-18

C17orf51	1.177386	6.17E-26
SRSF12	1.178412	8.96E-15
TMEM119	1.178857	5.25E-15
DHH	1.179266	2.57E-11
PDGFRA	1.180182	8.43E-16
HRH2	1.180351	5.00E-13
ALAS2	1.180922	9.63E-06
CD1C	1.181278	8.03E-08
DCN	1.18168	7.64E-16
TGFB3	1.182249	1.58E-14
KRT75	1.182727	0.002706
TMEM132I	1.18315	2.17E-11
PHOX2A	1.183509	0.000807
DAAM2	1.183856	3.31E-13
DPEP1	1.184552	5.70E-06
CMTM5	1.184955	1.48E-06
MEOX2	1.18613	1.04E-11
DKK2	1.186401	1.85E-10
FAM171A2	1.186718	6.14E-11
KCNH3	1.186912	4.63E-09
GBX1	1.18776	0.034109
GRIA4	1.187926	1.62E-07
ASPDH	1.188078	9.03E-08
MYH1	1.188646	2.19E-06
CYR61	1.189019	6.12E-11
PTPRD	1.189642	7.57E-17
PPP2R2B	1.189651	1.39E-10
PRKAR2B	1.190139	2.56E-16
LINGO4	1.190251	1.59E-06
GPR12	1.190908	0.000127
SERPINF1	1.191061	4.32E-16
USP2	1.191169	1.70E-09
MMP19	1.191894	1.46E-14
S1PR1	1.192384	5.52E-22
JAM2	1.193341	3.10E-26
TDRD6	1.193495	7.40E-15
ANGPT1	1.193535	2.76E-16
PYGM	1.194129	1.74E-12
PDE3A	1.194697	1.96E-18
MAGEE1	1.195154	6.79E-13
OR52N4	1.195334	3.24E-06
PLEK	1.196794	1.79E-12
SHE	1.197836	7.13E-25
TLR7	1.198026	4.23E-11
CD48	1.199394	1.04E-07
MYBPC2	1.199779	8.25E-07
GPR141	1.200264	2.31E-09
ICA1L	1.200417	7.01E-15
NPY	1.200423	0.000276
GPRASP2	1.200598	7.21E-21
RYR2	1.200792	6.27E-13
SLC26A4	1.201117	3.69E-11
MAP1B	1.202585	4.57E-17
SMIM18	1.20339	4.37E-06
SCAMP5	1.203824	8.72E-10
TRIM55	1.204097	7.43E-07
GAPT	1.205271	1.12E-08
PDZD2	1.205423	3.74E-12

PRSS3	1.205434	5.35E-05
CXCR6	1.205519	4.82E-11
CCDC67	1.206031	3.00E-10
EFEMP1	1.206108	1.69E-14
RND2	1.207045	7.86E-10
WASF3	1.208152	1.06E-33
SERPINA9	1.20833	0.001481
NRXN3	1.209215	2.26E-09
SYT11	1.209266	6.79E-16
SERPINA6	1.210088	0.000239
AMHR2	1.210619	0.000111
RTP5	1.210806	0.000277
RIC3	1.211408	1.18E-06
MS4A2	1.212671	9.96E-11
SOX5	1.214465	4.33E-19
CNTN4	1.214491	1.40E-19
GPIHBP1	1.215304	1.28E-14
SLC35F1	1.216682	5.87E-14
ICAM3	1.216867	1.29E-08
MYO3A	1.218122	3.52E-07
CLIP3	1.218252	9.18E-20
CHRNA6	1.21874	1.95E-08
ZNF385D	1.219395	1.66E-09
ANGPTL3	1.219762	7.33E-07
RAPGEF4	1.219946	5.74E-18
ABCA6	1.219966	9.99E-16
KIF26A	1.221451	8.21E-15
FGF12	1.221974	4.70E-10
SGCD	1.222271	6.34E-13
C16orf96	1.222907	9.12E-12
LAMA2	1.223058	4.78E-15
CILP2	1.22372	3.57E-08
ITGA4	1.224193	2.67E-14
CRMP1	1.225436	2.34E-14
ZNF812	1.225488	3.69E-08
SLC18A2	1.227013	3.26E-12
GATSL2	1.227154	4.69E-19
RASA4	1.227705	5.59E-14
TRIM61	1.229112	5.64E-16
DACH2	1.229411	3.02E-05
AMPD1	1.229872	1.11E-05
GLT1D1	1.230004	4.02E-10
GPR18	1.231927	6.88E-07
LEPR	1.232578	3.64E-18
SPOCK3	1.233074	6.37E-05
ENAM	1.233313	2.27E-06
FIBIN	1.233915	1.07E-10
TRIM58	1.233955	4.26E-11
DOCK10	1.234102	8.25E-19
CALCRL	1.235347	2.75E-22
SPARCL1	1.235424	9.73E-22
PAK7	1.236416	3.34E-05
RASSF2	1.236655	2.62E-19
RNF180	1.236759	5.19E-24
WISP2	1.236823	2.45E-07
CLEC4D	1.236831	4.62E-08
NALCN	1.238102	1.46E-14
SETBP1	1.238692	1.94E-28

GPR133	1.238799	1.19E-16
FAM133A	1.238878	8.02E-09
CD40LG	1.239351	1.17E-07
DIRAS3	1.239852	4.67E-11
APBB1	1.2403	9.14E-18
NAV3	1.241546	8.06E-22
CCIN	1.242231	5.23E-13
PTGDR2	1.242498	7.22E-07
ALOX15	1.243193	1.24E-06
CYSLTR1	1.243371	3.94E-16
PIRT	1.24338	9.88E-05
BHMT2	1.245106	1.55E-24
CSF2RB	1.246595	4.21E-13
GABRA1	1.247195	0.002787
CACNA2D	1.247544	2.15E-22
SCN4B	1.247741	9.93E-23
RAB9B	1.248792	1.24E-15
PEG10	1.249352	3.54E-07
IGFN1	1.249566	2.17E-06
CD1E	1.250805	9.25E-09
GIMAP8	1.251631	1.34E-24
LRCH2	1.253639	9.48E-24
FFAR3	1.254093	4.04E-08
MYOT	1.254096	6.80E-10
ALDH1L2	1.255088	1.05E-14
SRRM4	1.255194	7.55E-06
INSRR	1.255323	3.49E-09
TESPA1	1.25607	1.03E-09
GALNT15	1.256347	3.55E-12
NEFM	1.256422	4.70E-08
BCL2	1.257025	9.24E-21
RCSD1	1.257042	4.98E-12
ASB9	1.257581	1.58E-08
ZDHHC15	1.258658	2.97E-28
LSAMP	1.259298	8.07E-21
NEXN	1.259376	7.86E-15
KCNH2	1.25992	3.48E-07
SYNPO2	1.260225	1.72E-11
CTNNA3	1.260666	2.33E-09
LRRN2	1.260749	8.83E-15
RPH3A	1.261366	6.03E-06
CH25H	1.261856	8.37E-15
CCR5	1.262236	6.10E-13
SYNE1	1.262771	4.88E-34
GPC3	1.264785	2.11E-18
APOLD1	1.265438	5.77E-23
MAS1	1.265589	0.000331
PDE3B	1.265643	8.65E-13
MAL	1.265741	3.10E-07
TMTC1	1.266616	3.73E-17
CCDC141	1.266881	3.81E-08
NPTX2	1.267122	2.09E-08
ARHGEF26	1.268208	2.14E-14
ARHGAP3(	1.268823	0.000315
CACNA2D	1.268972	8.06E-11
ACSM5	1.269009	5.02E-15
NYAP2	1.269963	2.05E-07
F13A1	1.270417	7.72E-12

PCDHB4	1.27117	1.36E-15
CLEC6A	1.272434	5.46E-05
KLRC4-KLI	1.272612	8.16E-09
NPY5R	1.272908	1.11E-05
NDRG4	1.273305	9.87E-11
P2RY13	1.273669	1.64E-12
KIF19	1.273934	1.85E-08
MOXD1	1.274032	1.68E-16
SIM1	1.274072	0.00231
TMEM236	1.274127	7.82E-10
TEK	1.274506	1.27E-25
PRUNE2	1.274539	6.45E-07
ZNF80	1.275542	6.70E-06
FBLN5	1.275664	9.68E-22
DCC	1.275685	2.46E-08
PAIP2B	1.275978	9.28E-08
PLA2G2A	1.276039	0.001437
SNPH	1.276606	5.60E-15
MAN1A1	1.277367	3.07E-26
ZSCAN1	1.277728	3.19E-08
AFF3	1.277865	4.17E-08
MS4A7	1.278631	5.25E-16
RGS18	1.280629	5.15E-13
MAN1C1	1.280777	3.91E-34
FHL5	1.281137	1.19E-15
FAM131C	1.281254	3.55E-07
KCNT2	1.281261	2.77E-16
FAM180A	1.281522	3.09E-16
VWC2	1.282281	2.68E-05
KCTD16	1.282484	3.61E-11
CECR2	1.282566	5.18E-11
GIMAP7	1.283152	1.40E-15
PRR18	1.28323	2.12E-07
PCDHA6	1.284512	5.02E-11
CASS4	1.285142	5.49E-19
LGI2	1.285592	2.41E-16
FAM184A	1.2858	1.18E-17
IL24	1.285918	3.94E-07
OSBPL6	1.28677	7.45E-14
PTCHD2	1.286874	2.94E-07
TMED6	1.287151	6.41E-07
SEMA3G	1.287499	7.77E-21
CNTN1	1.287667	7.32E-11
SPACA3	1.289408	0.000195
FAM107A	1.290571	6.68E-13
ATP4A	1.291901	0.000926
MRO	1.291916	4.66E-14
5-Sep	1.293397	4.22E-19
AOAH	1.294282	6.81E-15
CARTPT	1.294902	0.000854
SLC1A2	1.294916	1.08E-05
TKTL1	1.295865	1.06E-08
KCNQ2	1.295961	1.11E-05
MPPED2	1.296338	8.50E-17
ADCY2	1.29676	8.34E-13
UGT2B28	1.296974	0.019767
NTS	1.297959	0.000206
ADAM33	1.298069	5.15E-16

FCRL5	1.298136	1.21E-05
IKZF1	1.298588	1.56E-10
ZNF366	1.300493	5.22E-17
FMO2	1.301532	1.28E-11
CACNB2	1.301587	8.87E-11
PADI4	1.301725	9.16E-08
EBF3	1.301729	1.73E-23
SERPINI1	1.302305	8.71E-16
FREM1	1.302729	1.56E-10
CTRB1	1.304089	0.026735
PCBP3	1.304683	1.82E-19
LAMB4	1.30545	5.52E-19
TMEM132	1.306	7.08E-09
CALB1	1.306092	1.42E-06
HBB	1.306156	5.93E-06
IGJ	1.30627	6.28E-06
TNFSF18	1.306409	7.22E-09
RGAG1	1.306742	3.14E-09
CTGF	1.308232	9.94E-17
SLC9A9	1.308882	3.99E-24
UNC45B	1.309174	1.25E-10
LIFR	1.309231	3.93E-21
RNF150	1.311903	2.18E-17
TTC24	1.312382	6.30E-06
KCNJ8	1.313507	5.46E-20
GABRB3	1.315031	3.90E-08
CMKLR1	1.315691	4.61E-18
CHRNA3	1.316007	3.10E-09
PTGDS	1.316113	4.80E-10
CCR6	1.316393	5.99E-09
LMNTD1	1.317327	0.001419
LONRF2	1.318368	1.09E-13
GHSR	1.318412	0.000122
LY9	1.318465	2.18E-08
IRF4	1.318866	2.73E-08
ARHGAP2	1.319738	9.35E-21
RADIL	1.32046	1.62E-14
GAS1	1.321404	5.55E-10
MMP16	1.321462	1.91E-22
PTPRC	1.321717	1.78E-10
TENM1	1.322215	3.45E-06
SPHKAP	1.322256	2.18E-06
POU6F2	1.322629	9.57E-06
PLN	1.322916	6.23E-08
NAP1L6	1.324187	7.33E-09
LPPR4	1.324286	1.19E-16
GPD1	1.324377	7.56E-08
ELAVL3	1.324409	1.16E-08
FAM159B	1.326429	0.000113
PLXNA4	1.32748	5.57E-12
RTN4RL1	1.328059	5.07E-10
PCDH9	1.328078	1.25E-15
SYT6	1.328535	2.00E-05
MYO16	1.329296	3.05E-11
GIMAP6	1.329627	2.40E-23
CELF2	1.330024	7.11E-20
EMCN	1.330412	1.94E-22
VAT1L	1.331279	5.29E-15

GLIPR1L2	1.331305	6.90E-12
VIP	1.332014	3.83E-05
SH2D1A	1.334058	8.15E-09
HSF5	1.33423	6.21E-07
SLCO5A1	1.334723	1.07E-13
SPATA4	1.335524	3.14E-07
GPR34	1.335549	8.15E-16
ADARB2	1.336627	8.55E-07
SDPR	1.336862	2.11E-18
FAM65B	1.33714	2.01E-09
ENSG0000	1.339135	1.33E-14
CORO2B	1.33925	2.58E-22
CASQ2	1.339512	1.00E-06
SLC7A8	1.340054	2.52E-14
FOXI2	1.340093	4.56E-11
SHISA9	1.340278	4.60E-07
COL26A1	1.341215	6.71E-10
FNDC5	1.341638	4.40E-22
ATP6V1G2	1.341849	1.54E-23
THBS4	1.343169	1.08E-10
HBA2	1.343588	8.01E-06
DCLK1	1.343731	5.17E-14
GRPR	1.344911	2.18E-06
ZBTB16	1.344926	2.30E-09
SPTA1	1.344982	2.60E-09
POMC	1.346961	3.63E-09
NACAD	1.347333	3.07E-12
HCN1	1.347536	5.54E-07
KCNH7	1.347553	5.66E-08
TLL1	1.348339	3.61E-15
EGF	1.348376	7.70E-05
ADAMTS1	1.348484	3.36E-17
DTNA	1.348781	6.69E-15
GFRA2	1.349585	5.79E-16
FLT3	1.34962	4.25E-08
GLP2R	1.349873	2.56E-13
ADAMTS3	1.349929	1.34E-18
FAM46C	1.350209	6.16E-16
KIAA0319	1.350666	1.04E-10
GRIK2	1.352444	1.06E-10
SELP	1.352691	3.44E-13
ADCYAP1F	1.352822	1.60E-14
NGB	1.353299	5.72E-05
BVES	1.354466	7.23E-15
SLC32A1	1.355345	0.002218
ADD2	1.356444	5.67E-15
LILRB5	1.356563	4.65E-13
RCAN2	1.356718	4.03E-26
MPEG1	1.357952	8.88E-16
CCDC158	1.358381	8.43E-14
EOMES	1.359149	5.83E-10
ADAMTSL	1.359254	5.73E-21
TUB	1.360964	1.44E-25
CDH19	1.361027	5.68E-07
SPON1	1.362269	1.02E-15
TUBB1	1.362409	3.52E-14
DPYS	1.363353	5.10E-08
PPP1R16B	1.364128	1.63E-16

SST	1.364453	2.53E-05
GAP43	1.365374	1.08E-10
PDE7B	1.365546	2.93E-24
NAP1L5	1.368842	2.34E-20
GMNC	1.369286	4.80E-05
KCNS2	1.36941	1.55E-10
TCEAL6	1.370907	6.01E-08
NOVA2	1.371879	6.95E-16
NRG2	1.3732	3.29E-12
HAP1	1.373483	4.06E-09
FOLR2	1.374321	2.65E-16
ADAMTS8	1.375345	3.07E-08
CD209	1.375513	9.41E-13
BRSK2	1.376947	5.42E-07
HGF	1.377803	4.75E-21
PDGFRL	1.379061	4.80E-19
WDFY4	1.380131	1.77E-11
PCDHA2	1.381059	2.31E-11
MAST1	1.382655	2.73E-09
P2RY8	1.383069	2.29E-15
ESR1	1.383235	3.98E-21
TMEM130	1.388137	1.82E-11
MFAP4	1.388832	1.45E-18
SALL2	1.389045	4.27E-20
FILIP1	1.390663	3.19E-23
CAND2	1.391724	1.40E-17
ASPA	1.391812	1.34E-17
COLEC12	1.392048	5.67E-12
FAM198A	1.392138	7.66E-22
SAMD3	1.392616	4.27E-18
C2orf71	1.392756	5.27E-06
EDNRB	1.395001	4.73E-26
NOS1	1.395701	3.17E-08
FAM57B	1.396475	3.34E-08
NPFFR2	1.396648	8.38E-07
PHACTR1	1.398534	1.55E-24
XCR1	1.398541	5.83E-08
RGS7BP	1.399023	4.74E-13
CCR2	1.400332	2.48E-13
CLEC9A	1.401083	4.39E-12
NBEA	1.401213	1.06E-16
OPRD1	1.401293	1.58E-08
FAM218A	1.402949	7.09E-16
KCNQ5	1.404364	9.30E-14
TMC2	1.40475	3.96E-16
JAKMIP1	1.405357	1.40E-14
S100A12	1.406324	5.82E-09
LRRC2	1.406417	1.09E-15
TPO	1.407475	5.57E-08
STAR	1.408175	6.48E-12
VIT	1.408794	1.67E-07
EPB41L3	1.409436	1.05E-27
RASGEF1C	1.409866	5.28E-10
LAX1	1.410985	2.11E-10
CFC1	1.411767	0.000513
SCML2	1.412573	2.22E-19
MAP2	1.412745	2.60E-15
P2RY10	1.414421	1.49E-08

ITK	1.415164	4.47E-09
PYHIN1	1.415232	4.29E-11
SLCO1C1	1.415921	9.47E-17
RORB	1.416281	1.47E-17
GPR174	1.416405	2.72E-08
PEBP4	1.4166	2.54E-10
CLEC2L	1.416733	5.37E-11
KCNK17	1.418133	8.41E-11
GIMAP5	1.4184	5.04E-16
SFTPA1	1.419222	2.87E-05
THEMIS	1.420938	5.82E-10
ERP27	1.421021	2.51E-05
LCN8	1.421772	0.034444
C1orf127	1.422363	5.05E-07
ACVR1C	1.42326	6.48E-14
DCT	1.423367	3.71E-14
CNR1	1.423488	4.38E-11
SGSM1	1.423849	5.95E-12
CD1B	1.424859	6.56E-08
ABI3BP	1.426201	1.08E-17
FHL1	1.427783	3.83E-19
TXLNB	1.427888	4.20E-19
NTRK2	1.42949	9.21E-13
EPHA5	1.429854	5.12E-10
NLGN1	1.42989	2.56E-11
FAM13C	1.430275	7.76E-24
FBLN1	1.430662	9.04E-20
SELE	1.430755	3.29E-07
CSMD3	1.430854	2.91E-05
GDF10	1.431419	2.37E-09
LRFN5	1.432379	2.00E-19
HFM1	1.433899	1.57E-10
P2RX2	1.434486	5.70E-08
CD28	1.435464	6.12E-12
TIMD4	1.436171	6.94E-07
RBPJL	1.43649	0.00719
GPM6A	1.437505	3.66E-08
SYT5	1.439176	1.50E-07
ITGAD	1.43959	2.35E-11
PNOC	1.441252	1.11E-10
MAPK8IP1	1.441858	1.87E-14
NLRP7	1.443225	3.41E-06
MMRN1	1.443503	1.38E-09
FMO1	1.443974	9.26E-16
ST8SIA5	1.444567	9.79E-13
STMN4	1.444893	2.21E-05
KCNG2	1.446105	2.85E-10
AMTN	1.447197	0.000344
CHRNB3	1.447406	0.000313
GPR114	1.447928	5.32E-12
TMEM215	1.448056	3.54E-06
AK5	1.448769	1.54E-09
SLC22A17	1.449397	8.18E-18
TNFSF8	1.45007	1.67E-11
KCNIP1	1.450325	2.59E-12
STAB2	1.452246	1.25E-11
LECT1	1.452887	8.10E-06
IL7R	1.452945	5.44E-12

KIAA1324	1.452971	8.39E-07
SEMA3D	1.453116	7.49E-10
CXCL13	1.45569	0.000165
KIRREL2	1.455754	2.14E-06
RUNDC3B	1.456954	1.13E-18
GRIK3	1.458161	4.68E-12
ZNF728	1.458713	4.28E-13
JPH4	1.458958	3.49E-17
RBM24	1.459141	1.13E-08
AMER2	1.459912	1.78E-05
ACKR1	1.46034	4.85E-09
MBOAT4	1.46079	3.90E-10
GPR64	1.460804	1.48E-08
GPM6B	1.461143	8.94E-21
ABCB1	1.461973	6.08E-13
SVEP1	1.462346	1.04E-19
HAND1	1.462534	0.001642
ALK	1.463713	1.13E-14
PNMA3	1.463804	1.81E-11
RASD1	1.463863	4.05E-10
PGM5	1.464999	2.05E-10
GPLD1	1.4653	1.55E-20
CALN1	1.466254	9.74E-06
ABCA9	1.466797	2.29E-22
RSPO3	1.467005	1.07E-10
CNTFR	1.467333	1.74E-06
CCKBR	1.470797	1.46E-06
LINGO3	1.470956	6.70E-08
OSR1	1.471117	2.32E-10
IGSF1	1.472086	4.12E-10
GPR88	1.4723	5.14E-12
IL33	1.472459	3.03E-16
AKAP6	1.47345	3.39E-14
CPEB1	1.473542	2.00E-24
CR1	1.476005	5.66E-10
GPR171	1.477466	4.78E-12
CCL19	1.478303	3.17E-06
CD200	1.479088	8.08E-25
KRT72	1.479572	4.73E-05
GP1BA	1.481483	1.14E-11
VIPR2	1.481683	3.78E-09
PLA2G1B	1.482155	0.007079
P2RY12	1.482602	2.55E-13
BCHE	1.483633	1.68E-18
FGF14	1.4852	2.87E-11
BANF2	1.485411	0.008763
CTNND2	1.485908	8.98E-09
TMEM252	1.486334	9.78E-07
ABAT	1.489439	1.43E-15
KCNMA1	1.489691	1.68E-15
GABRB1	1.492158	2.77E-07
EDARADD	1.493301	3.48E-13
COL25A1	1.493695	1.50E-11
FCRL3	1.494225	8.26E-07
KCNJ5	1.494382	5.22E-08
PRKCB	1.494678	4.11E-11
SLC6A15	1.494855	3.32E-07
HPCAL4	1.494898	1.43E-09

KSR2	1.495329	6.21E-14
DHRS2	1.496021	1.09E-08
P2RY14	1.496303	5.76E-14
FSTL4	1.498389	4.66E-08
HTR1F	1.498495	1.81E-12
PPP1R42	1.500498	2.48E-08
PCDH19	1.501327	7.73E-23
INS-IGF2	1.501835	0.000892
GADL1	1.502535	3.48E-06
SYT16	1.502751	3.30E-08
DMGDH	1.503421	8.56E-33
DSCAML1	1.505246	8.69E-10
CLVS2	1.506168	5.54E-07
TENM2	1.506941	8.01E-15
APOH	1.507708	1.60E-06
TNXB	1.507883	4.14E-16
WNT9B	1.508504	7.51E-14
TMEM72	1.509876	1.95E-06
FRMPD1	1.510229	9.58E-09
SFRP4	1.510687	1.69E-15
ATP8A2	1.511793	2.53E-18
SLIT2	1.513121	3.62E-26
CHST8	1.514507	1.29E-09
SDK1	1.515435	3.63E-21
NAP1L3	1.516993	1.78E-26
CXXC4	1.517715	1.37E-10
C16orf89	1.518546	2.17E-11
NPHS1	1.518925	1.12E-06
GP2	1.519844	0.004128
TRAT1	1.519978	1.87E-10
MAP6	1.522846	1.16E-14
CHST9	1.52322	2.40E-08
PRRG3	1.524479	1.15E-18
CASKIN1	1.525013	3.21E-08
SLC8A1	1.52522	1.06E-24
GPR162	1.525745	1.55E-22
BOC	1.527545	6.81E-30
FXYD6	1.527817	2.38E-23
TIFAB	1.528357	6.97E-11
MAFB	1.528368	1.24E-17
CDH22	1.532115	7.18E-07
CR2	1.532355	0.001291
NEGR1	1.533606	8.96E-31
MAMLD1	1.533774	1.37E-19
SV2A	1.534091	6.07E-19
AGBL4	1.534723	2.05E-09
DNASE1L3	1.534975	9.08E-10
CYP1B1	1.536461	1.87E-17
CELA3B	1.537284	0.010549
C2orf40	1.537723	3.18E-14
ISL1	1.539989	2.19E-10
GZMK	1.540649	1.71E-11
SLC17A6	1.541057	8.29E-05
MUSK	1.541386	4.71E-09
ZFR2	1.542279	1.67E-10
MDS2	1.542863	1.22E-10
KCNA3	1.543884	1.14E-13
PLD5	1.544064	2.35E-09

PRG4	1.544158	3.30E-16
MAP1LC3(	1.544421	1.10E-15
CELF5	1.547321	8.08E-12
AQP4	1.549362	4.93E-09
GNAZ	1.550974	1.24E-13
WDR17	1.551376	2.00E-11
MCOLN3	1.551699	9.58E-12
ART4	1.55227	7.24E-17
C1orf158	1.554121	4.35E-05
TMEM255,	1.555717	1.46E-16
ENPP2	1.558158	1.10E-17
FOXP2	1.558769	3.52E-19
C10orf82	1.55886	3.64E-09
SV2B	1.559037	1.88E-10
SYT3	1.560745	1.60E-13
C1QL1	1.564375	1.59E-09
SBK2	1.564463	1.10E-07
FAM222A	1.564472	4.85E-10
KCNK2	1.565784	2.46E-14
SRPX	1.566196	4.73E-26
LCN6	1.567946	7.89E-10
CHRNA2	1.569379	6.47E-05
SLC10A6	1.569798	1.04E-19
KCNN3	1.569867	1.06E-33
IL6	1.570667	2.03E-08
SCRT1	1.571438	1.43E-07
DLK1	1.571888	1.69E-05
AGTR1	1.572247	1.79E-18
RGS4	1.572673	4.26E-18
XPNPEP2	1.572909	4.60E-08
EBF2	1.573957	1.88E-18
CCDC80	1.574694	1.13E-21
KIAA1644	1.575287	5.06E-15
KCNJ11	1.575387	4.74E-11
ARC	1.575922	2.38E-08
RANBP3L	1.577054	4.51E-15
MAPK10	1.577444	1.01E-23
GLP1R	1.577902	1.96E-09
PM20D1	1.579395	1.44E-07
HEPACAM	1.580968	1.11E-05
BMPER	1.581498	2.37E-12
CADPS	1.58224	5.17E-12
CCNA1	1.582244	6.50E-16
GPR113	1.582601	5.60E-14
CPA2	1.582962	0.005036
PTGER3	1.58314	5.38E-27
PLA2G2D	1.58468	5.87E-07
CCL14	1.584935	6.96E-13
PACSIN1	1.585731	3.05E-11
NRSN1	1.586393	9.09E-09
HTR2A	1.589382	8.04E-17
PIPOX	1.58949	4.05E-16
C15orf59	1.589587	6.10E-22
ZNF536	1.590069	2.25E-10
CELA3A	1.590517	0.006688
DGKB	1.592525	3.69E-13
CABP7	1.592935	1.27E-08
ANGPTL1	1.595593	2.40E-14

ADAMTSL3	1.59628	7.60E-22
11-Mar	1.596784	0.000468
ABCD2	1.59816	8.99E-16
ST8SIA3	1.598932	3.93E-07
S100Z	1.600205	4.87E-15
WNT4	1.600736	1.91E-09
PHYHIPL	1.600781	7.37E-14
CCL21	1.602953	1.45E-08
CCKAR	1.603618	2.63E-05
C1orf95	1.603869	7.75E-11
BAIAP3	1.604909	9.87E-11
ZCCHC5	1.611349	3.00E-11
HSPB6	1.613039	2.01E-18
MAGEL2	1.614155	1.46E-19
TRIM9	1.615276	1.10E-11
NAT8L	1.615501	2.62E-10
JAKMIP2	1.618909	1.44E-17
UCHL1	1.619513	9.44E-17
SYT7	1.620016	5.43E-11
CLEC10A	1.620715	1.99E-15
PRRT4	1.623215	5.03E-10
TMEM178I	1.625441	3.58E-12
CSRNP3	1.631025	7.76E-25
PLCXD3	1.632118	7.42E-12
ENSG0000	1.632236	7.92E-14
LCN10	1.633015	9.28E-09
CRB1	1.633034	1.37E-10
ZNF483	1.634006	3.47E-17
PHOX2B	1.635868	1.37E-05
CTNNA2	1.637672	8.07E-11
AQP12A	1.637841	0.001012
KCNK3	1.638585	1.99E-09
NAP1L2	1.641021	1.54E-15
SCN4A	1.641532	6.12E-16
NHSL2	1.643517	1.82E-25
SNCB	1.644609	3.11E-08
ADAMTS1	1.646091	8.24E-15
NUDT10	1.647099	2.55E-22
ATP2B3	1.648164	1.71E-14
SYT10	1.648676	1.23E-06
PTGFR	1.648802	2.58E-20
CPA5	1.651047	2.83E-07
TNFRSF13	1.651818	3.28E-07
MAGEE2	1.652687	2.60E-15
SLC38A8	1.653258	3.03E-07
LBP	1.653638	3.13E-06
CNKSR2	1.654375	2.36E-24
CUZD1	1.654892	0.000221
LIX1	1.656194	1.44E-11
MTUS2	1.656652	4.20E-10
LYVE1	1.65671	1.26E-16
ZNF157	1.657066	1.74E-14
CD36	1.657088	9.93E-17
BHLHE22	1.657327	1.79E-28
AQP8	1.658031	0.001236
TMEM151I	1.658996	1.60E-13
DNAI2	1.661587	4.69E-12
IRX1	1.665946	1.49E-05

TCERG1L	1.666037	1.03E-07
CNGA3	1.666052	8.45E-11
LMO3	1.666138	7.52E-17
ENSG0000	1.666375	7.36E-06
PEG3	1.666452	4.66E-26
TNNT3	1.666992	5.50E-10
B3GAT1	1.667246	6.30E-11
SCN2B	1.669117	8.41E-19
MAPK8IP2	1.67136	1.81E-11
GALNT16	1.672175	1.00E-19
FGF7	1.67219	1.84E-16
PNMAL1	1.675477	6.09E-17
ANGPTL7	1.675507	1.40E-06
KCNC2	1.676219	1.06E-06
BRINP2	1.676953	3.97E-10
CNTN6	1.679201	2.26E-11
ARX	1.679693	3.03E-07
RGAG4	1.679933	7.33E-22
WT1	1.680577	2.45E-13
TPH2	1.681527	5.99E-07
ELOVL4	1.683537	6.05E-26
NIM1K	1.685259	1.12E-17
MYH2	1.68635	9.80E-08
FAM92B	1.686492	3.33E-09
HLF	1.689783	1.51E-20
NEUROD2	1.691611	4.29E-06
SLITRK2	1.69193	1.66E-13
SELL	1.692433	1.74E-16
GNG2	1.698312	2.98E-28
DYNC1I1	1.698918	7.10E-19
NFASC	1.699681	1.72E-18
WNK3	1.700171	3.17E-21
GALNT8	1.700774	6.92E-14
CACNA1I	1.70152	2.22E-11
RGS22	1.701536	2.29E-32
KIAA1045	1.702883	8.30E-19
AOX1	1.704287	9.92E-15
ANKRD7	1.704455	5.20E-13
CCR4	1.704738	4.94E-13
NANOS1	1.70611	1.40E-18
PGR	1.708048	1.31E-25
TDRD9	1.709545	1.24E-15
DLGAP3	1.710698	3.01E-15
RIMS4	1.713824	1.89E-13
PCDHA1	1.717086	6.14E-11
PCDHB1	1.720124	6.14E-10
KCNJ3	1.721758	1.29E-08
PRLR	1.72177	1.65E-13
PMP2	1.72335	1.79E-07
DIRAS1	1.723587	9.91E-13
RSPO1	1.723971	5.46E-13
SPTB	1.724375	7.11E-17
ADAMTS1	1.725196	1.80E-14
FLG	1.726737	3.08E-15
RASGRP2	1.729106	3.44E-18
FBXL16	1.729765	1.39E-12
RNF182	1.73012	6.75E-14
DNAH9	1.731386	9.28E-15

EFR3B	1.734232	1.56E-24
CPA1	1.737372	0.020488
SLC5A7	1.738	2.55E-07
CBLN1	1.739076	6.06E-15
CTRC	1.739695	0.001381
TEX11	1.741232	3.69E-10
GNG4	1.742604	5.10E-12
SYT14	1.74379	7.52E-10
RIIAD1	1.745155	1.58E-10
CPE	1.747068	8.71E-15
PRIMA1	1.747664	7.19E-12
C6orf58	1.747819	2.01E-06
TMEM145	1.747996	3.14E-12
UBE2QL1	1.753233	1.33E-20
AMY2A	1.753304	0.005856
CXCR5	1.753949	3.33E-06
COL6A5	1.755297	6.08E-10
CILP	1.757464	1.64E-12
LMO1	1.760239	1.08E-08
FBLL1	1.760433	3.56E-10
KCNH5	1.762014	5.25E-06
BTBD17	1.773638	9.07E-08
VSTM2A	1.773746	1.81E-08
ASB4	1.774442	3.03E-10
KLHL1	1.777531	3.06E-06
RFPL2	1.778387	4.07E-10
GLRA3	1.781573	6.15E-10
HS3ST4	1.782055	5.08E-07
ATP1A3	1.784244	1.33E-15
PDZD4	1.788102	1.38E-22
GC	1.789232	1.28E-07
LRRC4B	1.790878	5.85E-17
LPL	1.793239	2.32E-20
KCNA2	1.795712	4.92E-16
TMOD1	1.79981	1.30E-14
VPREB3	1.800609	1.64E-07
SORCS1	1.803525	3.38E-14
OGDHL	1.803639	2.36E-10
RGS9	1.804173	6.47E-16
CDO1	1.805214	3.80E-28
PDZRN4	1.806122	1.57E-11
AQP12B	1.807562	9.89E-05
RPRML	1.807766	9.37E-07
SLC12A1	1.809398	1.31E-10
TCF23	1.809636	1.05E-11
STAP1	1.809808	3.98E-12
KIAA2022	1.810864	2.08E-20
FP325317.	1.812328	6.30E-12
SLC35F3	1.812728	2.46E-11
DCAF12L2	1.813068	2.61E-10
FOSB	1.814441	9.33E-13
KRT222	1.815528	2.80E-16
LGI1	1.819613	6.12E-11
BMP5	1.820135	1.49E-10
GPRASP1	1.820152	3.38E-31
CD79B	1.820432	5.28E-11
NRK	1.82227	4.22E-23
TRPM3	1.82295	5.60E-17

PDK4	1.824029	2.68E-20
CADM3	1.824206	1.56E-14
CLC	1.825125	6.89E-07
RXFP3	1.826779	5.33E-11
TSPAN11	1.828036	7.65E-22
SCN3A	1.828361	5.93E-19
SLC35F4	1.829178	1.78E-11
DLG2	1.831196	1.24E-36
FIGF	1.832023	2.47E-16
ADCYAP1	1.832453	4.45E-12
TMEM59L	1.832627	6.01E-14
CHRDL2	1.833748	6.42E-10
CNTN2	1.834135	7.93E-14
ST6GALNA	1.834524	3.24E-20
RASGRF1	1.838384	6.26E-13
MYOC	1.838848	2.63E-06
DPYSL5	1.840879	9.65E-10
MROH2B	1.841734	8.43E-12
NPAS4	1.843891	2.73E-14
OMD	1.845779	1.16E-18
GCG	1.847379	0.000185
RET	1.848532	8.46E-22
ANKS1B	1.849912	2.21E-15
RELN	1.851225	5.69E-12
MAPT	1.851441	2.32E-16
SCRG1	1.851518	1.18E-16
LRRTM2	1.853226	5.80E-22
CD22	1.853234	1.11E-12
DCX	1.856761	1.92E-12
SLC6A17	1.858333	4.16E-11
CLEC4G	1.85849	9.58E-11
SLC7A2	1.867777	1.15E-18
LRRTM4	1.871123	1.23E-12
SYCN	1.873682	0.003291
TSPAN7	1.873747	1.67E-19
FTCD	1.874914	1.52E-12
ABCC9	1.879058	2.14E-40
SYNGR4	1.879123	1.02E-08
FAIM3	1.88094	1.84E-17
ENSG0000	1.881985	4.02E-16
FAM180B	1.882406	2.63E-12
FAM163A	1.882708	3.67E-13
PCSK1N	1.883575	2.69E-08
NKX2-2	1.884013	6.34E-08
FEV	1.886421	2.96E-09
CACNA1G	1.886509	4.51E-19
ERICH3	1.888852	4.06E-10
IGFBPL1	1.888874	1.34E-15
MEDAG	1.889207	8.25E-17
FABP4	1.891033	1.16E-08
MSI1	1.892989	9.53E-17
F10	1.895088	3.40E-19
HHATL	1.895296	8.93E-08
GRM7	1.897789	4.10E-17
C5orf38	1.899136	2.42E-10
DPT	1.902234	8.73E-21
TLR10	1.902342	3.12E-15
AMPH	1.90268	1.06E-24

NRCAM	1.905494	1.60E-19
RXRG	1.90558	3.62E-10
AP3B2	1.909438	1.44E-13
GHR	1.910316	5.81E-39
LGALS12	1.910712	9.54E-15
SLC29A4	1.910744	3.82E-14
LINGO2	1.912426	4.21E-12
GALNT13	1.912439	1.73E-22
COL14A1	1.916854	8.56E-33
C12orf42	1.921385	2.34E-09
CD79A	1.921769	8.51E-10
ZCCHC18	1.922006	1.06E-24
DNAJC6	1.927508	7.31E-21
CALY	1.927735	3.52E-09
CERS1	1.930337	4.61E-17
GRIA1	1.930843	1.64E-12
SLC4A8	1.931186	7.55E-21
TSPEAR	1.933536	2.38E-11
SORCS3	1.934652	5.91E-12
MYL7	1.935098	8.20E-07
ATP1B2	1.935846	7.43E-21
PNLIPRP1	1.937003	0.00088
4-Mar	1.938143	4.99E-12
CHL1	1.941484	3.59E-23
PAX6	1.942168	7.78E-17
PCSK1	1.942387	4.69E-11
TNMD	1.947452	7.80E-06
LHFPL4	1.951648	4.99E-09
CLSTN2	1.957855	1.05E-32
AMY2B	1.958172	3.09E-08
RAB39A	1.958843	4.39E-22
KL	1.959907	1.90E-19
GLYATL3	1.960993	0.010253
SLIT1	1.963534	6.35E-13
FSTL5	1.964139	8.16E-09
GNAO1	1.964392	1.11E-23
DNAI1	1.965946	2.21E-11
GDAP1L1	1.968502	6.51E-12
SERPINI2	1.97023	0.000176
RIMS2	1.972859	2.22E-12
SCN7A	1.973043	1.71E-18
TNFRSF13	1.97359	1.89E-11
NCAM1	1.97789	1.45E-14
CXCL12	1.980256	6.84E-34
KIAA0408	1.981138	1.10E-14
GPX3	1.981644	9.25E-20
CTD-2207	1.982437	8.58E-07
CTSG	1.983687	1.90E-15
NR4A3	1.996709	1.13E-19
OXGR1	1.997661	2.98E-13
RAB3C	1.998143	6.98E-18
C4orf50	1.998545	4.57E-15
FGF10	1.99924	3.94E-21
PEX5L	1.999642	3.75E-13
CEL	2.001579	0.000146
MTMR7	2.007099	3.06E-25
SNTG2	2.007622	2.59E-16
PPM1E	2.011095	1.40E-18

G6PC2	2.015024	4.69E-07
ZNF831	2.018058	3.46E-16
GALR1	2.018788	1.98E-13
CNIH2	2.021871	3.99E-13
JPH3	2.022461	3.45E-17
CDH8	2.025428	2.34E-15
KIF5A	2.026918	3.53E-21
CHD5	2.027372	2.45E-16
PCDH10	2.030374	8.98E-23
SLIT3	2.031048	4.12E-40
UNC5D	2.038945	1.53E-09
EIF4E1B	2.040687	2.49E-07
SCN3B	2.041302	3.12E-20
KCNB2	2.04162	1.08E-14
LRRC10B	2.042471	4.46E-16
CRH	2.043832	1.18E-05
RAB39B	2.048906	2.03E-17
PTF1A	2.049457	0.000435
PTX3	2.051987	5.71E-13
SLC6A4	2.054784	3.40E-17
SLC12A5	2.062598	2.56E-18
PENK	2.065497	4.14E-13
ABCA8	2.065804	3.13E-32
PPP4R4	2.068049	1.96E-13
FCRL2	2.069567	2.21E-10
DDX25	2.070425	2.05E-12
CCR7	2.072251	4.04E-17
P2RX5	2.07328	1.38E-12
GRIK5	2.075474	3.27E-20
ATP1A2	2.077743	3.81E-14
NAT16	2.079398	7.85E-12
MPP2	2.082296	2.31E-28
CA10	2.082577	6.57E-11
CXorf57	2.082605	1.63E-22
IAPP	2.083859	1.70E-06
SNAP25	2.084365	1.10E-15
ANKRD34(	2.09085	3.00E-10
MPPED1	2.095451	1.29E-09
ANK2	2.097367	3.38E-34
BTLA	2.101189	9.07E-19
KCNJ6	2.101959	1.25E-13
PAK3	2.104172	5.68E-16
BRS3	2.104638	8.83E-08
CSF3	2.108163	1.05E-08
ANGPTL5	2.109376	1.28E-18
TACR1	2.119054	2.60E-21
VWC2L	2.120449	4.04E-11
CACNA2D	2.12137	9.05E-17
KLB	2.126962	4.98E-13
EFNB3	2.128005	1.55E-22
PHF21B	2.128664	3.97E-17
MRAP	2.129999	1.07E-08
LRRN3	2.132555	1.33E-24
TRDN	2.134327	1.38E-08
FRMPD4	2.135767	3.78E-16
DAZL	2.137174	6.82E-11
TAGLN3	2.137586	3.72E-14
CACNG2	2.143172	1.47E-06

PLIN4	2.143981	5.19E-12
CYP46A1	2.148731	6.54E-25
GPR98	2.14895	5.75E-17
PAPPA2	2.151896	2.95E-13
MAPK4	2.15408	1.13E-17
PLP1	2.155755	1.30E-14
CDH18	2.156054	3.26E-10
NLGN4X	2.158392	3.13E-32
PI16	2.158642	2.63E-11
GRM4	2.160984	7.46E-12
CD19	2.1621	2.21E-10
NOVA1	2.164733	1.22E-25
USP41	2.165152	3.19E-13
FFAR1	2.165455	4.13E-10
COL6A6	2.170607	6.51E-22
LRRC4	2.17083	3.48E-24
C18orf42	2.172229	5.72E-11
ELAVL4	2.179558	4.00E-17
HAO1	2.179917	1.89E-07
CRYBA2	2.182552	2.17E-09
COL19A1	2.1841	6.19E-11
BSN	2.190947	2.05E-21
CELA2A	2.197623	0.000246
C1orf168	2.199203	2.23E-13
CACNG5	2.204337	5.66E-11
CRHBP	2.204948	6.15E-23
CELA2B	2.215273	2.38E-05
GRIA3	2.216231	2.16E-20
REEP2	2.222145	2.62E-22
RTN1	2.223006	2.29E-20
SH3GL2	2.228124	2.18E-13
HMGCLL1	2.234639	2.01E-17
RIPPLY2	2.234784	2.31E-10
ADH1B	2.23598	4.46E-18
DUSP26	2.243098	1.12E-14
PCP4	2.251219	1.51E-15
SLC30A8	2.258123	1.05E-09
UNC79	2.266602	1.46E-16
KIF1A	2.269827	6.06E-15
CPB1	2.275726	0.001282
FAM19A4	2.275808	7.61E-13
NRXN1	2.279353	8.07E-21
GAD2	2.290647	3.91E-10
PNLIP	2.291275	0.004486
LGI3	2.291488	5.07E-16
EML5	2.292037	3.88E-23
PRSS1	2.293357	9.74E-06
NEUROD1	2.300546	2.56E-12
SCGN	2.302591	3.42E-12
SLITRK1	2.30446	1.68E-12
SFRP1	2.306731	3.17E-21
VGF	2.308809	1.54E-13
NLRP4	2.310004	2.11E-06
IRX2	2.318836	8.06E-14
OTOGL	2.319584	3.75E-18
UCN3	2.319767	2.48E-12
GLYAT	2.321917	1.25E-06
SLC8A3	2.322517	7.09E-28

GRIA2	2.323052	5.22E-13
NECAB2	2.336658	8.23E-21
CMA1	2.33955	4.06E-14
KHDRBS2	2.343445	3.57E-17
PAX5	2.344482	3.91E-11
C7	2.348747	3.29E-28
SCG5	2.373397	3.16E-17
SLC16A12	2.378944	1.41E-16
ATRNL1	2.380012	1.95E-22
CBLN4	2.381984	8.54E-26
KLHL14	2.382788	3.62E-17
ERO1LB	2.383516	2.47E-23
FCRLA	2.38935	1.80E-11
C14orf180	2.389871	9.14E-11
AVPR1B	2.391029	1.01E-14
GRM1	2.396275	1.73E-21
BAI3	2.398388	7.41E-27
BPIFB4	2.401352	2.73E-07
PROK1	2.417421	4.13E-14
CIDEA	2.422706	7.10E-06
FCER2	2.423353	7.41E-09
ACSL6	2.426244	9.02E-25
CLEC17A	2.429739	1.02E-15
GLRA1	2.430555	6.27E-11
GABRG2	2.433498	1.24E-12
KIF5C	2.442875	1.22E-28
SMYD1	2.443878	3.35E-05
CNR2	2.444452	1.76E-12
GJD2	2.448745	3.51E-11
PPP1R1A	2.452062	1.39E-13
INSM1	2.456918	1.34E-12
CNTNAP5	2.458685	2.07E-15
CDH10	2.470266	3.66E-16
AMER3	2.471364	5.07E-14
OGN	2.474875	3.29E-28
BEX1	2.483447	7.65E-16
MS4A1	2.483978	2.60E-10
HAS1	2.485304	2.47E-16
SPTBN4	2.494398	4.91E-26
CELF4	2.495273	4.64E-22
TNR	2.496207	1.29E-19
SCRT2	2.499849	2.26E-10
CHRNB2	2.50404	4.73E-19
SNAP91	2.504375	1.03E-23
KCNB1	2.505325	1.39E-18
CNTN5	2.513847	7.20E-16
C22orf42	2.516752	3.88E-13
XKR4	2.517859	3.48E-23
CHRDL1	2.524267	1.67E-25
TCEAL5	2.528541	1.19E-23
GCK	2.531704	1.19E-19
MYT1L	2.531746	7.00E-17
BLK	2.536962	2.28E-13
NOL4	2.539733	5.25E-15
IQSEC3	2.550417	2.66E-33
KCNMB2	2.55984	8.33E-18
SERPINA1(	2.560779	5.61E-16
CAMK2B	2.57148	2.89E-17

SYP	2.572561	4.10E-23
CNTNAP4	2.57263	2.09E-15
TCL1A	2.586215	3.57E-08
HMP19	2.589387	1.30E-18
SLC4A10	2.590762	7.03E-21
CACNG7	2.596501	7.89E-14
CLGN	2.600598	4.03E-24
ZDHHC22	2.610085	9.80E-17
STXBP5L	2.628049	1.74E-17
SNTG1	2.63047	5.84E-15
IGF1	2.642815	1.54E-27
FMN2	2.645873	1.76E-25
CTXN2	2.648998	4.91E-14
INA	2.649107	6.29E-20
ABCC8	2.65968	7.31E-15
KCNK16	2.661513	9.27E-12
MYT1	2.675823	1.65E-21
PTPRT	2.709561	2.74E-28
GPHA2	2.713078	6.20E-09
UNC80	2.717904	2.44E-17
CDK5R2	2.723364	6.67E-21
TMEM196	2.726121	1.21E-16
HS6ST3	2.746276	2.93E-22
PRLHR	2.749078	3.84E-18
RFX6	2.751344	4.27E-16
ATCAY	2.752563	4.93E-21
SERTM1	2.75491	5.64E-18
KCNA1	2.760943	1.95E-22
KCNA5	2.76307	6.32E-25
FAM129C	2.781324	1.47E-15
ST18	2.784886	1.55E-21
MGAT4C	2.787159	4.53E-24
UNC13A	2.787434	8.35E-28
GPR142	2.799224	6.67E-21
CACNA1A	2.816638	5.31E-28
SPX	2.817562	7.45E-16
TUSC5	2.819034	1.21E-09
FRRS1L	2.829678	3.28E-24
SCG3	2.83429	3.27E-18
SEZ6	2.835216	1.30E-21
KCNC1	2.835408	4.71E-19
CHGA	2.839295	1.12E-15
DPP6	2.841996	1.66E-23
PLIN1	2.84677	9.00E-18
TCEAL2	2.849397	2.13E-22
LEP	2.861981	4.80E-12
SLC7A10	2.880826	1.34E-12
SSTR3	2.895582	3.09E-19
AMY1B	2.903486	3.01E-05
ASTN1	2.916145	6.51E-25
SSTR2	2.926726	9.90E-34
ADCY1	2.9545	2.20E-37
SLC38A4	2.955157	3.62E-26
TTR	2.971286	1.01E-12
CD300LG	2.973095	1.55E-20
APLP1	2.974448	3.80E-28
RUNDC3A	2.975061	9.40E-24
SVOP	2.984555	1.77E-19

SCARA5	2.990879	2.93E-24
GPR148	3.016943	4.50E-16
MAP3K15	3.023179	7.05E-24
CELF3	3.027338	4.20E-21
FCRL1	3.028315	3.29E-15
SCG2	3.028728	4.29E-26
CACNA1B	3.032027	1.99E-25
SEZ6L	3.049655	4.17E-22
ACTL6B	3.064094	2.27E-19
XKR7	3.067037	1.31E-20
LRRC53	3.075983	6.17E-07
SLC8A2	3.110962	1.48E-23
PCSK2	3.116182	7.17E-17
FAM135B	3.124312	4.11E-26
RIMBP2	3.136749	2.04E-26
PTPRN	3.150571	7.13E-24
TMEM179	3.16787	2.22E-20
VWA5B2	3.173828	4.67E-24
CHGB	3.179958	2.52E-18
SFTPC	3.185298	6.11E-13
CPLX2	3.205347	2.22E-19
SHISA7	3.227826	1.26E-21
TMEM132[illegible]	3.315805	4.53E-24
ADIPOQ	3.376921	2.65E-09
SYT4	3.388724	7.32E-25
THRSP	3.391312	1.39E-18
TMEM63C	3.39778	1.94E-28
GPR119	3.400535	3.43E-19
GAST	3.41577	8.54E-12
DSCAM	3.425546	2.70E-29
MMD2	3.462076	9.87E-12
KCNH6	3.462887	4.13E-24
AGTR2	3.545843	4.47E-19
SLC7A14	3.615483	2.66E-30
CSN1S1	4.76573	8.00E-22
