# Supplementary material for: Molecular signatures of tumor progression in pancreatic adenocarcinoma identified by energy metabolism characteristics
Source: BMC Cancer. 2022 Apr 13;22:404. doi: 10.1186/s12885-022-09487-3 (PMC9006543; doi:10.1186/s12885-022-09487-3)
Supplement: Supplementary file 9 — Additional file 9. [file 12885_2022_9487_MOESM9_ESM.pdf]

Supplementary Table 4. The univariate Cox proportional hazard regression analysis result of 743 co-expression DEGs

Symbol	p.value	HR	Low 95%CI	High 95%CI
PLEKHN1	0.00168913	1.03025863	1.01126612	1.04960784
PERM1	0.00275246	1.07232312	1.02441114	1.12247597
RNF223	0.00425377	1.06499426	1.01999402	1.11197982
MMEL1	0.1346027	1.04310788	0.98700324	1.10240169
ARHGEF16	0.05497449	1.00990029	0.99979013	1.02011267
RBP7	0.09198477	0.98366269	0.96499511	1.00269139
ANGPTL7	0.53594202	0.99134749	0.96443979	1.01900591
EPHA2	0.00238634	1.00182984	1.0006487	1.00301237
PADI1	0.01015571	1.00412657	1.00097857	1.00728447
PLA2G2D	0.6110143	0.99333084	0.96804489	1.01927728
GALE	0.04936388	1.00471707	1.0000131	1.00944318
MAN1C1	0.01508462	0.95000874	0.91151933	0.99012339
FAM110D	0.09316126	0.95788442	0.91095839	1.00722774
SFN	0.03168555	1.00071613	1.00006278	1.00136991
SERINC2	0.23943223	1.00080734	0.99946262	1.00215387
TINAGL1	0.08947708	1.00167841	0.99974149	1.00361908
TMEM54	0.18435564	1.00129413	0.99938397	1.00320793
GJB5	5.44E-07	1.02124362	1.01287977	1.02967653
GJB4	4.89E-05	1.03381139	1.01735224	1.05053682
GJB3	3.12E-05	1.0094663	1.00500029	1.01395215
GRIK3	0.33651028	0.92641931	0.79271562	1.08267419
SLC2A1	0.06671202	1.00084362	0.99994189	1.00174616
TSPAN1	0.00598015	1.00096287	1.00027624	1.00164998
TAL1	0.05357643	0.81070065	0.65511544	1.00323623
PODN	0.86921584	0.99952916	0.99394154	1.0051482
TACSTD2	0.06501537	1.00048745	0.99996968	1.00100549
LEPR	0.76698116	0.9940431	0.95552651	1.03411226
PDE4B	0.58382297	0.98754109	0.94422294	1.03284654
PTGER3	0.72580098	1.01484048	0.93463937	1.10192361
NEGR1	0.63106684	0.98605214	0.93111477	1.0442309
NEXN	0.68751137	1.00245289	0.99055723	1.0144914
PTGFR	0.7326754	0.98628614	0.91115238	1.06761545
TGFBR3	0.71476782	1.00326206	0.98589128	1.02093889
EPHX4	0.01071255	1.05645711	1.01282154	1.10197263
PALMD	0.94166419	0.99802302	0.94650256	1.05234786
S1PR1	0.60404645	0.99763185	0.9887322	1.00661161
GSTM5	0.70244114	1.01276869	0.94894323	1.08088701
EPS8L3	0.13441947	1.00216403	0.99933151	1.00500458
KCNA3	0.25926803	0.91967109	0.79514966	1.06369272
OLFML3	0.62163553	1.00077465	0.99770181	1.00385696
NGF	0.38366572	0.94770488	0.83983052	1.06943545
CD2	0.72559486	0.99854538	0.99046145	1.00669528
MTMR11	0.17744361	1.00234915	0.99893704	1.00577291
S100A10	0.00693652	1.00041351	1.00011331	1.0007138
S100A11	0.00071874	1.00017362	1.00007301	1.00027424
S100A12	0.20801449	1.04080238	0.97798217	1.10765781
S100A6	0.02442745	1.00004773	1.00000616	1.0000893
S100A16	0.00028438	1.00086221	1.00039647	1.00132817
S100A14	0.21062513	1.00024473	0.99986159	1.00062802
NPR1	0.08259488	0.96490527	0.92675713	1.00462372
ATP8B2	0.0318231	0.97872478	0.95969511	0.99813178
SHE	0.58081795	0.97207439	0.87910282	1.07487838
TDRD10	0.10285443	0.77148496	0.56486361	1.05368629
EFNA4	0.02170628	1.00932347	1.00135732	1.017353
EFNA1	0.4083216	1.00044775	0.99938673	1.00150989
MUC1	0.11453994	1.00036418	0.9999119	1.00081666
RAB25	0.09436023	1.00127682	0.99978101	1.00277487
CRABP2	0.00387695	1.00090727	1.00029146	1.00152346
FCRL5	0.36579223	0.94978502	0.84944502	1.06197759
FCRL3	0.33645905	0.96612957	0.90057697	1.03645372
CD1C	0.49006526	0.99403501	0.97728959	1.01106735
CD1B	0.84163767	0.98385556	0.83866816	1.15417732
CD1E	0.42352578	0.97815938	0.92666552	1.03251469
MNDA	0.2921211	1.00648036	0.99445727	1.01864882
PYHIN1	0.49473843	0.96617002	0.87528517	1.06649186

CADM3	0.09472606	0.96467557	0.92482889	1.00623906
ACKR1	0.94818456	1.00011458	0.99666455	1.00357656
FCER1A	0.61295886	0.99569249	0.97917729	1.01248625
IGSF9	0.13018775	1.0118422	0.99653185	1.02738778
CD84	0.78745991	1.00459368	0.97167426	1.03862838
SLAMF1	0.52858073	0.97790003	0.91223838	1.04828792
CD48	0.41564038	0.99581069	0.98579555	1.00592758
LY9	0.36723986	0.94040379	0.82282366	1.07478592
FCRLA	0.36678379	0.98839548	0.96365596	1.01377013
DDR2	0.10311	1.01133335	0.99772094	1.02513149
RCSD1	0.38899653	0.99240308	0.97533274	1.00977218
DPT	0.954347	0.99991172	0.9968942	1.00293838
SELP	0.99216637	0.99991515	0.98311976	1.01699747
SELL	0.35827526	0.9972602	0.99143987	1.0031147
SELE	0.70317787	1.00430992	0.98233677	1.02677458
FMO2	0.93881397	1.00119208	0.97119457	1.03211613
FMO1	0.81117679	1.00434459	0.96925268	1.040707
TNFSF18	0.83008535	0.99225952	0.9242776	1.06524161
ANGPTL1	0.55720302	0.9932528	0.97105231	1.01596084
RGL1	0.65006462	0.99565535	0.97710138	1.01456163
HMCN1	0.12735945	1.0280182	0.99214017	1.06519366
PRG4	0.28130291	0.98033378	0.94554408	1.0164035
RGS18	0.8788266	0.9942863	0.92367481	1.07029578
PTPRC	0.64948954	0.99823757	0.99067239	1.00586052
LAD1	0.07041875	1.0011505	0.99990419	1.00239836
ELF3	0.02957796	1.00136837	1.0001356	1.00260267
LAX1	0.6218046	0.9840158	0.9229917	1.04907453
LEMD1	0.10102121	1.00873459	0.99830423	1.01927392
SLC45A3	0.31714536	1.00316576	0.99697249	1.0093975
CTSE	0.14776853	1.0001743	0.99993829	1.00041036
IL10	0.64079205	0.95397377	0.78266735	1.16277491
IL24	0.40615186	0.9887994	0.96286675	1.01543048
C4BPB	0.28898808	1.00623555	0.99473993	1.01786402
CR2	0.95959429	1.0000992	0.99626855	1.00394459
CR1	0.40083618	0.95284633	0.85130391	1.06650061
LAMB3	0.00229511	1.00085903	1.00030676	1.0014116
HSD11B1	0.23047074	1.00474703	0.99699981	1.01255444
TRAF3IP3	0.32103164	0.97106896	0.91636348	1.02904026
KCNK2	0.88394371	0.99393193	0.91593411	1.07857177
CAPN8	0.39055302	1.00081743	0.99895246	1.00268587
CAPN9	0.74409919	0.99914633	0.99403598	1.00428295
KCNK1	0.09433155	1.00413483	0.99929242	1.0090007
MAP1LC3C	0.54765645	0.96038415	0.84182399	1.09564198
NLRP3	0.63301781	0.98076327	0.9056014	1.06216333
GREB1	0.28692748	1.06410894	0.9491113	1.1930401
OSR1	0.4630393	0.98077383	0.93121738	1.03296751
KRTCAP3	0.205805	1.00183315	0.99899475	1.00467962
XDH	0.01068843	1.00960153	1.00222137	1.01703604
VIT	0.76296464	0.98133937	0.86826886	1.10913451
CYP1B1	0.89163753	1.00035657	0.99523882	1.00550064
EFEMP1	0.5404384	1.00067425	0.99851706	1.00283609
PLEK	0.99871808	1.00000906	0.98902187	1.0111183
ADD2	0.03556631	0.7829526	0.62322435	0.98361813
HK2	0.11758657	1.00233072	0.99941262	1.00525735
CD8A	0.9918142	0.99989202	0.97947699	1.02073257
MAL	0.14606309	0.96066506	0.91006534	1.01407813
ZAP70	0.25242068	0.97467228	0.93277896	1.01844713
AFF3	0.00818907	0.80321001	0.68278622	0.94487307
IL1R1	0.30405026	1.00208789	0.9981101	1.00608152
SULT1C2	0.86870229	0.99967299	0.99580395	1.00355707
SULT1C4	0.86979768	0.99582352	0.94721551	1.04692593
ARHGAP15	0.34574765	0.96989906	0.91019552	1.0335188
ZEB2	0.66670996	1.00975811	0.9661048	1.05538389
CD302	0.94413605	0.99868947	0.96272089	1.03600188
SCN7A	0.80069552	0.99596922	0.96522251	1.02769534
CHRNA1	0.4236301	1.06737374	0.90981775	1.2522142
ITGA4	0.82774226	0.99551874	0.95604918	1.03661777

CERKL	0.04090647	0.95673947	0.91702529	0.99817357
PDE1A	0.73047474	1.00687644	0.96837486	1.04690881
CALCRL	0.49650723	1.00551063	0.98970903	1.02156452
STAT4	0.05442628	0.93180868	0.86710336	1.00134248
PLCL1	0.28311953	0.88834588	0.71563349	1.10274103
AOX1	0.53915373	0.99425117	0.97612313	1.01271588
CD28	0.40060366	0.96881096	0.8998114	1.04310155
ICOS	0.74852407	0.98578098	0.90314182	1.07598179
ADAM23	0.56015332	0.97820449	0.90831015	1.0534772
CXCR2	0.623196	1.01900449	0.94528021	1.09847867
IHH	0.46730232	1.00214271	0.99637553	1.00794326
SLC23A3	0.28872197	1.06845939	0.94543216	1.20749592
DOCK10	0.11723379	0.96442551	0.92169231	1.00913998
B3GNT7	0.7211361	0.99939782	0.99609771	1.00270886
NGEF	0.00080585	1.02860057	1.01177347	1.04570752
UGT1A10	0.01975243	1.01090214	1.00172751	1.02016079
MLPH	0.4081147	1.00147918	0.99797802	1.00499263
TWIST2	0.69179905	1.00997178	0.96161979	1.060755
GPR35	0.308543	1.00642475	0.99409508	1.01890736
CHL1	0.31812257	0.96240548	0.89265936	1.03760108
CNTN4	0.63771305	0.9785099	0.89391455	1.0711109
IL17RE	0.01857806	1.01560527	1.00259476	1.02878461
PPARG	0.1465084	1.00457571	0.99840407	1.01078551
MKRN2OS	0.01807967	1.0684563	1.01138079	1.12875278
GALNT15	0.44289246	1.01612306	0.9754462	1.05849619
ZNF385D	0.81539913	1.01296593	0.90912975	1.12866176
EOMES	0.49297362	0.96080575	0.85702604	1.07715243
RBMS3	0.70230124	1.01004358	0.95958723	1.063153
CCR4	0.54495774	0.98609138	0.94237115	1.03183996
SUSD5	0.76971065	1.01387396	0.92453149	1.11185008
VILL	0.10306084	1.00259427	0.99947602	1.00572224
CX3CR1	0.63947702	0.98357408	0.91772185	1.05415161
CCR8	0.98276033	0.99847785	0.86962477	1.14642321
CLEC3B	0.02368752	0.98119174	0.9651823	0.99746674
CXCR6	0.6832232	1.01016698	0.96226055	1.06045844
XCR1	0.25779607	0.9119134	0.7773078	1.06982852
CCR1	0.19638612	1.01240261	0.99364635	1.03151293
CCR2	0.96664022	0.99900072	0.95327344	1.04692147
CCR5	0.97247715	1.00048827	0.9731249	1.02862108
LRRC2	0.03448238	0.76366569	0.59478301	0.98050091
ALS2CL	0.01524904	1.00736697	1.00141201	1.01335734
MST1R	0.03638959	1.0051871	1.00032786	1.01006995
ACY1	0.10243113	1.0436506	0.99148762	1.09855793
SEMA3G	0.02174077	0.95928436	0.92582573	0.99395215
DNASE1L3	0.14204969	0.92158024	0.82639361	1.02773078
FAM107A	0.27211452	0.98405704	0.95623267	1.01269104
FAM3D	0.2562211	0.99928365	0.99804825	1.00052059
GXYLT2	0.56618444	1.00687363	0.98358311	1.03071565
PDZRN3	0.10470319	0.976461	0.94876332	1.00496729
EPHA3	0.78953952	1.00209609	0.98680582	1.01762328
STX19	0.16088561	1.01678089	0.99340052	1.04071154
ABI3BP	0.7778018	1.00241006	0.98578972	1.01931063
TRAT1	0.53986501	0.96953539	0.87822231	1.07034275
CD96	0.55518414	0.98599398	0.94086015	1.03329293
BTLA	0.34603912	0.95737391	0.87444336	1.04816943
CCDC80	0.50073774	1.00079385	0.99848481	1.00310823
CD200R1	0.73646528	0.9621357	0.7684336	1.20466506
BOC	0.30063854	0.9885089	0.96711025	1.01038102
TIGIT	0.58000829	0.98435146	0.9308703	1.04090527
LSAMP	0.44486109	1.01027378	0.98412718	1.03711505
CLDN18	0.54616453	1.00011123	0.99975005	1.00047254
SLC9A9	0.64613292	0.99045958	0.95074677	1.0318312
AGTR1	0.05549892	0.90745504	0.82159699	1.00228538
GPR171	0.5283524	0.9865727	0.94597472	1.02891299
P2RY14	0.39635182	0.9698201	0.90351715	1.04098856
P2RY13	0.69976662	0.99082401	0.94547305	1.0383503
P2RY12	0.80662872	1.01115722	0.92519838	1.10510237

KCNAB1	0.81586034	0.98913377	0.90223392	1.08440349
PTX3	0.68434982	0.99859225	0.99183303	1.00539753
BCHE	0.24237468	0.95233248	0.87746015	1.03359354
KLHL6	0.59377238	0.98857949	0.94772221	1.03119817
IGF2BP2	3.80E-05	1.02274169	1.01185736	1.03374311
MUC4	0.26253132	1.00440631	0.99671103	1.01216101
NRROS	0.16039218	0.96111861	0.90934711	1.0158376
S100P	0.06202607	1.00030581	0.99998462	1.00062711
HS3ST1	0.03253669	1.01059826	1.00087717	1.02041376
C1QTNF7	0.3867426	1.03168437	0.96132625	1.10719192
SLIT2	0.94735106	0.99925095	0.97727189	1.02172433
LGI2	0.29815351	0.96648263	0.90636225	1.03059088
TLR10	0.31798929	0.96475669	0.89915463	1.03514507
RHOH	0.33079528	0.97907532	0.93822553	1.02170368
SHISA3	0.31629991	0.95680716	0.87764599	1.04310845
OCIAD2	0.00301919	1.00420246	1.00142325	1.00698939
PDGFRA	0.53331746	1.00221522	0.99526208	1.00921694
KDR	0.11446256	0.98327311	0.96289217	1.00408545
NMU	0.18392249	1.00342509	0.99837712	1.00849859
STAP1	0.47286163	0.98861206	0.95817458	1.02001643
ADAMTS3	0.61897955	1.053029	0.85900906	1.29087123
CXCL13	0.61042967	0.99931898	0.99670332	1.0019415
TMEM150C	0.27301826	0.98084438	0.94750296	1.01535904
SLC10A6	0.31233373	0.91206942	0.76293157	1.09036074
SPARCL1	0.86933768	1.00007931	0.99913475	1.00102476
MMRN1	0.06841611	0.9842233	0.96753207	1.00120247
ADH1B	0.41524427	0.9969695	0.98971644	1.00427571
EMCN	0.256339	0.97852892	0.94253422	1.01589822
DKK2	0.42679807	0.98405245	0.94579893	1.02385316
FAT4	0.61361637	1.01925407	0.94652495	1.09757155
PABPC4L	0.2233549	1.17199126	0.90778343	1.51309605
TLL1	0.54731945	1.03715322	0.92096593	1.16799847
ANXA10	0.10785057	1.00070781	0.9998451	1.00157127
TENM3	0.78024254	0.99491898	0.95994569	1.03116643
ADAMTS16	0.67535538	0.98841891	0.93597861	1.0437973
FBXL7	0.7267141	0.99411076	0.96172124	1.02759111
IL7R	0.66333413	0.99813559	0.98978424	1.00655741
LIFR	0.10621768	0.96250628	0.91889843	1.00818362
C7	0.88353626	0.99994877	0.99926359	1.00063442
FGF10	0.04370924	1.06553454	1.00179255	1.1333323
GZMK	0.54916915	0.9946048	0.97715416	1.01236708
GAPT	0.33033302	0.96992861	0.91209729	1.0314267
LRRC70	0.92657458	1.01375828	0.75809054	1.35565053
CD180	0.53078409	0.98477948	0.93866527	1.03315916
ZNF366	0.59962863	0.965105	0.84524026	1.10196794
TMEM171	0.98807013	0.99988018	0.98429798	1.01570906
BHMT2	0.9679361	0.99873317	0.93887179	1.06241124
THBS4	0.82124888	0.99972284	0.99732176	1.0021297
PITX1	0.09783972	1.00697932	0.9987213	1.01530562
MZB1	0.70003813	0.99842167	0.99043078	1.00647703
PCDHGA12	0.97450562	1.00255129	0.85751027	1.17212484
CSF1R	0.9657305	1.00014538	0.99353462	1.00680013
CCDC69	0.41261818	0.99473592	0.98225505	1.00737537
SGCD	0.23838554	1.02128545	0.98614638	1.05767663
TIMD4	0.59283054	0.98421112	0.9284422	1.04332992
ITK	0.35694206	0.97428549	0.921754	1.02981079
EBF1	0.43209991	0.97818772	0.92582101	1.03351642
SLIT3	0.30501835	0.99103888	0.97413871	1.00823225
DOCK2	0.6669993	0.9929616	0.96152189	1.02542932
HRH2	0.83767788	0.98298268	0.83412532	1.15840501
CDHR2	0.68616522	1.00111243	0.99572801	1.00652596
GFPT2	0.41160517	1.00373901	0.99483675	1.01272093
IRF4	0.36376637	0.9727094	0.9163262	1.03256197
FOXQ1	0.08523369	1.00321107	0.99955515	1.00688037
F13A1	0.11186726	1.00300047	0.9993028	1.00671182
TFAP2A	0.03267438	1.03629298	1.00294332	1.07075158
PHACTR1	0.02462456	0.82379336	0.69566158	0.97552535

RNF39	0.14660783	1.00903368	0.99685563	1.02136051
TRIM31	0.34664896	1.00212123	0.9977086	1.00655338
TRIM10	0.28084888	1.06465067	0.9500786	1.19303923
TRIM15	0.59790929	1.00161992	0.99561316	1.00766292
SFTA2	9.52E-05	1.00392585	1.00195229	1.0059033
PSORS1C1	0.35014076	1.02336523	0.97496455	1.07416869
PSORS1C2	0.72250254	1.01528905	0.93372653	1.10397618
POU5F1	0.87570715	1.00075397	0.99134717	1.01025002
NCR3	0.23602864	0.95194538	0.87747742	1.03273313
SLC44A4	0.56384757	1.00035714	0.9991448	1.00157096
TNXB	0.58155235	0.99058255	0.95780373	1.02448316
SPDEF	0.97491048	0.99997122	0.99817905	1.0017666
PI16	0.77057918	0.99870087	0.99001311	1.00746487
C6orf223	0.69901103	1.00269209	0.98912019	1.01645021
COL21A1	0.76688022	0.98557357	0.89529401	1.08495674
BEND6	0.05155506	1.14621937	0.99907979	1.31502895
FILIP1	0.69277195	0.97971075	0.88498691	1.08457328
PRSS35	0.20535862	0.8910991	0.74546992	1.06517725
CNR1	0.37366184	0.95645539	0.86709557	1.05502432
BACH2	0.19274387	0.92240537	0.81684075	1.04161265
FHL5	0.83801897	1.00353868	0.97012059	1.03810793
SCML4	0.06434248	0.78065602	0.60050257	1.0148563
SLC16A10	0.17498209	1.08767423	0.96329142	1.22811768
PLN	0.64044057	1.0014279	0.9954498	1.0074419
THEMIS	0.66014797	0.97282608	0.8604055	1.09993553
LAMA2	0.19702622	1.00632931	0.9967291	1.01602198
SAMD3	0.24401476	0.86816519	0.68439937	1.10127335
MOXD1	0.581136	1.00209781	0.99466538	1.00958578
PDE7B	0.35374321	0.95609821	0.86954986	1.05126093
TXLNB	0.71354429	1.04953449	0.81076377	1.35862342
MYCT1	0.823908	0.99630931	0.96438457	1.02929089
TAGAP	0.82589688	0.99706048	0.97124718	1.02355984
SMOC2	0.35403999	0.99897142	0.99679964	1.00114792
ADAP1	0.29583868	1.00397286	0.99653725	1.01146395
LFNG	0.03219578	1.0030379	1.00025778	1.00582575
THSD7A	0.35239169	1.03620861	0.961371	1.11687193
MEOX2	0.81620134	0.99565514	0.95976038	1.03289235
AGR2	0.71959869	1.00001872	0.99991652	1.00012093
AGR3	0.37095568	1.00067299	0.99919928	1.00214888
GPNMB	0.64118117	1.00044292	0.99858156	1.00230775
PRR15	0.31654472	1.00230938	0.99779521	1.00684398
AOAH	0.53620567	0.9925782	0.96942343	1.01628603
ELMO1	0.00396161	0.94892357	0.91567757	0.98337665
GPR141	0.52211445	1.06302751	0.88158626	1.28181161
SFRP4	0.88426657	0.9999159	0.99878418	1.0010489
IKZF1	0.34920877	0.98511435	0.95466201	1.01653807
ABHD11	0.20639288	1.00382719	0.99789684	1.00979277
CLDN4	0.00828454	1.00117341	1.00030221	1.00204537
ELN	0.53087389	0.99905734	0.99611482	1.00200855
FGL2	0.40834	1.00307266	0.99580424	1.01039413
CD36	0.04576834	0.97453874	0.95018645	0.99951517
HGF	0.88698174	1.00474254	0.94127868	1.07248533
SEMA3D	0.66551896	1.02840253	0.90577142	1.1676365
ADAM22	0.53134824	0.97919594	0.91681273	1.04582393
BAIAP2L1	0.09897774	1.00224343	0.99957884	1.00491513
KPNA7	0.19678016	1.02014511	0.98971316	1.05151279
CYP3A5	0.59934979	1.00083235	0.99773049	1.00394385
MUC3A	0.74670066	1.00057636	0.99708404	1.00408091
PIK3CG	0.90958285	0.99638915	0.93608566	1.06057744
TFEC	0.7410941	1.01430361	0.93235117	1.10345955
MET	3.62E-07	1.00806533	1.00495063	1.01118969
CPED1	0.55439021	1.00797014	0.98178806	1.03485044
KCP	0.02114001	1.0264562	1.00392099	1.04949726
AKR1B10	0.08126908	1.0012538	0.99984434	1.00266526
FAM180A	0.25658685	1.02402224	0.98287616	1.06689082
TMEM139	1.27E-05	1.03233696	1.01769001	1.04719471
GIMAP8	0.72426591	0.99558879	0.971432	1.02034629

GIMAP7	0.2945264	0.99556572	0.98732737	1.00387281
GIMAP6	0.56830915	0.99513071	0.97858334	1.01195788
GIMAP1	0.19214688	0.96250505	0.90878669	1.01939871
GIMAP5	0.38592501	0.90979806	0.73474779	1.12655326
AOC1	0.87587959	0.99982143	0.99758337	1.0020645
SHH	0.39979115	0.99340706	0.97822812	1.00882153
BLK	0.35109757	0.98774089	0.96246092	1.01368486
DLC1	0.97950542	0.99968972	0.97629857	1.0236413
PDGFRL	0.88132096	1.00077931	0.99059657	1.01106672
LPL	0.1800602	0.99165097	0.97956967	1.00388128
GFRA2	0.01455125	0.65762783	0.46986299	0.92042653
EBF2	0.57497702	0.97686216	0.90011332	1.06015504
SCARA5	0.79478408	0.99742535	0.97823646	1.01699063
NUGGC	0.98490847	1.00124328	0.88029142	1.13881391
PNOC	0.90245756	0.99579183	0.9308502	1.06526418
FGFR1	0.0811346	0.98729363	0.9732075	1.00158364
SFRP1	0.01160435	0.98658346	0.9762892	0.99698627
SOX17	0.09293074	0.93839588	0.87131076	1.01064611
SDR16C5	0.04327188	1.00435573	1.00013143	1.00859787
BHLHE22	0.43352547	0.97328687	0.90952057	1.04152381
CYP7B1	0.65124978	1.00848178	0.97221776	1.04609847
PREX2	0.93758798	1.00342825	0.921048	1.09317675
CRISPLD1	0.91393843	0.99808546	0.96399307	1.03338356
ZFHX4	0.12094076	1.10465748	0.9740833	1.25273492
FABP4	0.14854614	0.99763261	0.99442979	1.00084574
MMP16	0.7740766	0.98092038	0.86002047	1.11881616
ESRP1	0.00093839	1.00986643	1.00400962	1.01575741
ANGPT1	0.33515976	1.05421864	0.94688666	1.17371696
MAL2	0.02423326	1.00113471	1.00014769	1.00212269
COL14A1	0.59244476	0.999042	0.99554215	1.00255415
FAM83A	0.00185625	1.00397437	1.0014697	1.00648531
SLA	0.71541573	0.99685401	0.98011071	1.01388333
PSCA	0.00888444	1.00031108	1.00007805	1.00054417
GPIHBP1	0.15573395	0.94094155	0.86508976	1.02344408
MROH6	0.00363604	1.00854069	1.00277662	1.0143379
IL33	0.94801001	0.9996674	0.98972154	1.00971321
PTPRD	0.25997482	0.94348116	0.85264922	1.04398933
MPDZ	0.93308154	0.99758671	0.94288173	1.05546562
FREM1	0.31677683	0.96097991	0.88893972	1.03885827
BNC2	0.32467898	1.03399357	0.96744616	1.10511855
TEK	0.86303259	1.00214736	0.97801993	1.02687002
CCL19	0.71811186	0.9998343	0.99893512	1.00073429
CCL21	0.40093492	0.99962899	0.99876379	1.00049493
SIT1	0.35170533	0.98864235	0.96515895	1.01269712
RECK	0.69722476	1.00725854	0.97122123	1.04463302
NTRK2	0.5130906	0.98426565	0.93858109	1.03217387
GAS1	0.04790542	1.00480458	1.0000442	1.00958762
OGN	0.83215223	0.99924417	0.9922815	1.00625569
OMD	0.46966717	1.00430626	0.99267563	1.01607315
ASPN	0.04681894	1.00162024	1.00002278	1.00322026
ECM2	0.20880948	1.01432926	0.99207225	1.03708559
NR4A3	0.75458335	0.99776976	0.98389942	1.01183564
C9orf152	0.88825899	0.99950588	0.99263879	1.00642049
SVEP1	0.52744837	1.00675011	0.98596064	1.02797795
LPAR1	0.82768655	0.99771066	0.97733138	1.01851489
RNF183	0.96336136	1.00103467	0.95782556	1.04619301
TNFSF8	0.52172274	0.98517983	0.94119279	1.03122263
PTGES	0.00053969	1.00283936	1.00123009	1.00445121
FAM78A	0.24776602	0.97470895	0.93327447	1.017983
PTGDS	0.67530704	0.9996898	0.99823963	1.00114208
CLIC3	0.0038803	1.00329033	1.00105594	1.00552971
CELF2	0.22516175	0.98286896	0.95580483	1.01069943
MRC1	0.7930668	0.99933007	0.99433896	1.00434623
PLXDC2	0.20874105	1.00523997	0.99708175	1.01346494
CXCL12	0.89613892	0.99964069	0.99426139	1.00504909
ZNF488	3.80E-05	1.11037871	1.05642105	1.16709231
WDFY4	0.38696434	0.98021765	0.93683627	1.02560786

DKK1	0.00540455	1.00474579	1.00139982	1.00810295
FAM13C	0.3541397	0.92600217	0.78702443	1.08952148
HKDC1	0.06727809	1.00654965	0.99953576	1.01361276
TSPAN15	0.07997045	1.00227233	0.99972896	1.00482217
LIPM	0.01188806	1.08497798	1.01817397	1.15616511
ANKRD22	0.00065797	1.01013645	1.00429189	1.01601502
CH25H	0.38431979	0.99215235	0.97469712	1.00992018
COL17A1	0.00013375	1.0026964	1.00131183	1.00408289
GPAM	0.57965231	1.0076279	0.98089472	1.03508967
ACSL5	0.01699524	1.00287771	1.00051403	1.00524698
GFRA1	0.20566502	0.97598063	0.93991603	1.01342902
CLRN3	0.42925142	1.00143247	0.99788445	1.0049931
EBF3	0.62649327	0.97481762	0.87965905	1.08027013
PKP3	0.00958306	1.00373304	1.00090738	1.00656668
ANO9	0.91802997	1.00078448	0.98594969	1.01584248
CDHR5	0.65021049	0.99883213	0.99380009	1.00388965
EPS8L2	0.30004082	1.00247723	0.99779739	1.00717901
MUC5AC	0.68228528	0.99970636	0.99830159	1.0011131
BRSK2	0.06026356	0.89522512	0.79760819	1.00478909
SYT8	0.68242147	1.00034957	0.99867615	1.0020258
TNNT3	0.16429153	0.90642923	0.78923542	1.04102518
C11orf21	0.55081774	0.95489366	0.82053213	1.11125679
SLC22A18	0.5394075	1.00327403	0.99285534	1.01380205
PHLDA2	0.25362332	1.00091546	0.99934425	1.00248915
SCUBE2	0.22365684	1.01354927	0.99181508	1.03575974
LYVE1	0.76852042	0.99650345	0.9735279	1.02002123
SPON1	0.55591269	1.00098312	0.99771499	1.00426196
PTPN5	0.69686037	0.94992783	0.7335803	1.23008057
FIBIN	0.17808822	1.00406519	0.99815409	1.0100113
WT1	0.55378164	0.97475164	0.89563508	1.06085702
LRRC4C	0.3373284	0.93682068	0.81989222	1.07042483
MPEG1	0.72525349	0.99837893	0.98938655	1.00745305
MS4A2	0.73792633	1.01408512	0.93431392	1.10066713
MS4A6A	0.82856703	1.0010987	0.99119788	1.01109841
MS4A4E	0.0717665	0.8181097	0.6575158	1.0179276
MS4A4A	0.67907352	1.00196094	0.99270522	1.01130297
MS4A7	0.77073042	0.99836016	0.98740092	1.00944103
MS4A1	0.37139662	0.99604522	0.98742826	1.00473739
CD5	0.24157321	0.98532307	0.96123922	1.01001033
RASGRP2	0.19186534	0.98050891	0.95194864	1.00992605
OVOL1	0.00312808	1.0388116	1.01290314	1.06538275
CST6	0.02512709	1.00226528	1.00028242	1.00425208
CATSPER1	0.02430882	1.04482723	1.00570643	1.08546977
RHOD	2.41E-05	1.00456625	1.00244457	1.00669242
CABP4	0.09287197	1.04480619	0.99273557	1.09960799
NUDT8	0.45802967	1.00538213	0.99122964	1.01973669
ALDH3B1	0.0128754	1.00872805	1.00184383	1.01565957
MYEOV	0.00102204	1.00637862	1.00256729	1.01020444
FOLR2	0.93522991	0.99976395	0.99408792	1.00547239
PDE2A	0.01920652	0.93124095	0.87733468	0.9884594
SLCO2B1	0.81440608	1.00133666	0.99023105	1.01256682
CAPN5	0.11830482	1.00273958	0.99930305	1.00618793
DLG2	0.24845039	0.85048985	0.64599794	1.11971408
GUCY1A2	0.6670697	0.97677007	0.8775745	1.08717809
ZBTB16	0.22859562	0.97225701	0.92873098	1.01782294
IL10RA	0.40926762	0.99242058	0.97464753	1.01051774
TMPRSS4	0.012548	1.00420429	1.00090188	1.00751759
SCN4B	0.28823498	0.97176207	0.92172496	1.02451551
SCN2B	0.78832718	0.97397809	0.80344618	1.18070549
CD3E	0.4235097	0.99627746	0.98721977	1.00541825
CD3G	0.55266003	0.98250338	0.92689395	1.04144912
TRIM29	0.020699	1.00396722	1.00060513	1.0073406
CRTAM	0.98558284	0.99870807	0.86804589	1.14903811
CLMP	0.06649546	1.01144039	0.99922609	1.02380401
VSIG2	0.40060347	1.00052746	0.99929797	1.00175846
FLI1	0.50735827	0.98813678	0.9538811	1.02362264
BARX2	0.00334883	1.02180225	1.0071847	1.03663196

ST14	0.15099911	1.00103569	0.99962238	1.002451
ADAMTS8	0.60003653	0.99192158	0.96229883	1.02245621
SCNN1A	0.86789593	1.0000772	0.99916794	1.00098728
CD27	0.32876763	0.99232048	0.97708572	1.00779278
CD163	0.12029479	1.0030667	0.99919926	1.0069491
APOBEC1	0.94037278	0.99983998	0.99565618	1.00404136
CLEC4E	0.65428253	1.0066101	0.97799895	1.03605826
KLRG1	0.20925867	0.90687279	0.77853391	1.05636793
KLRB1	0.32801646	0.9889389	0.96714135	1.01122771
CD69	0.76864792	0.99769214	0.98244958	1.01317118
STYK1	0.02006322	1.03033456	1.00470467	1.05661827
BCL2L14	0.20600445	1.0221717	0.98801484	1.05750939
APOLD1	0.52943149	0.99554558	0.9817897	1.00949419
GPRC5A	0.00252495	1.00158451	1.000556	1.00261408
ART4	0.5226783	0.97987604	0.92066228	1.04289822
RERGL	0.91497294	0.99596233	0.92467193	1.07274907
PDE3A	0.76295669	1.00470449	0.97452298	1.03582073
ABCC9	0.19479682	0.97469855	0.93766011	1.01320004
TSPAN11	0.14307344	0.9775654	0.94832518	1.00770719
LRRK2	0.58467971	1.02187183	0.94558586	1.10431223
DHH	0.02601742	0.66763546	0.46778389	0.95286972
AQP5	0.3094827	1.00063936	0.99940671	1.00187352
GALNT6	0.59116413	1.003196	0.99158348	1.01494451
KRT7	1.05E-05	1.00087832	1.00048763	1.00126918
KRT8	0.00109557	1.00052884	1.00021132	1.00084646
KRT18	0.00027238	1.0007419	1.00034238	1.00114158
NCKAP1L	0.6534174	0.99488299	0.97285218	1.01741271
TESPA1	0.21958419	0.94549445	0.86456501	1.03399947
MMP19	0.85729132	1.00101184	0.99003844	1.01210686
AVPR1A	0.54671822	1.01237074	0.97269116	1.05366899
TSPAN8	0.03624304	1.00030133	1.00001931	1.00058343
NAV3	0.37978461	1.05003008	0.94164843	1.17088621
DCN	0.30953905	1.00063475	0.99941085	1.00186015
PLXNC1	0.24561304	0.9864798	0.96407027	1.00941024
IGF1	0.43892829	0.96941357	0.89608022	1.04874837
GLT8D2	0.22762105	1.00747869	0.99535781	1.01974717
ALDH1L2	0.15188961	1.0286734	0.98965501	1.06923014
CMKLR1	0.88063676	1.00185386	0.97792462	1.02636864
TMEM119	0.7428009	0.99935974	0.99554412	1.00318999
HSPB8	0.34791852	0.99489718	0.98432576	1.00558213
OASL	0.00112378	1.01137604	1.00451602	1.0182829
FLT3	0.73977651	0.98656853	0.91090498	1.06851701
MEDAG	0.86055101	1.00038959	0.99605155	1.00474653
DCLK1	0.89605984	1.00412384	0.94400254	1.06807413
FAM124A	0.91429426	1.00847113	0.86486231	1.17592594
PCDH17	0.72009723	0.99348017	0.95856329	1.02966894
KLF5	0.00735579	1.00232882	1.00062534	1.00403521
EDNRB	0.56241412	0.99342283	0.97148766	1.01585327
GPC6	0.34357915	0.99170419	0.97475477	1.00894834
GPR18	0.29349644	0.94928967	0.86144966	1.04608651
GPR183	0.5117652	1.00218112	0.99567947	1.00872521
GRTP1	0.12645133	1.01414737	0.99603935	1.0325846
RNASE6	0.75527145	0.99924484	0.99450939	1.00400284
CMA1	0.34495878	0.96113703	0.88523631	1.04354552
CTSG	0.37933438	0.98700532	0.95863895	1.01621105
PRKD1	0.45268666	0.98818021	0.95798454	1.01932765
PTGDR	0.87270245	0.97452371	0.7107163	1.33625254
LGALS3	0.003551	1.00093575	1.00030659	1.0015653
GPX2	0.95089092	1.00001081	0.9996669	1.00035483
PLEK2	0.00458649	1.00367534	1.00113273	1.00622441
BATF	0.87508895	1.00044478	0.99491335	1.00600696
TGFB3	0.91021579	0.99971937	0.99485443	1.0046081
FLRT2	0.99152065	0.99974606	0.95399859	1.04768727
GPR65	0.79467678	0.98993046	0.91728071	1.06833415
FBLN5	0.46748042	0.99793146	0.99237287	1.00352118
IFI27	0.00181166	1.00120896	1.0004492	1.00196929
SYNE3	0.48342833	0.94961386	0.82177252	1.09734319

TCL1A	0.50292215	0.9972385	0.98920273	1.00533955
EML1	0.17665001	0.96500674	0.91641058	1.0161799
AMN	0.28297899	1.00497364	0.99591275	1.01411696
EXOC3L4	0.83294755	1.00278074	0.97723626	1.02899295
LPCAT4	0.06458322	1.00227288	0.99986263	1.00468893
THBS1	0.03560918	1.00074973	1.00005043	1.00144952
SPINT1	0.07857785	1.00131683	0.99984966	1.00278615
RHOV	0.0009318	1.00744394	1.00302993	1.01187738
ITPKA	0.14515281	1.00948478	0.99674539	1.02238699
FGF7	0.0841779	1.01376752	0.99815668	1.02962251
ATP8B4	0.84983523	1.02190162	0.81659093	1.27883237
HDC	0.22650849	0.93347605	0.83493414	1.04364824
GCNT3	0.02264231	1.00248298	1.00034761	1.00462291
CILP	0.54361941	1.00222945	0.99505264	1.00945802
ABHD17C	0.01752326	1.00394439	1.00068891	1.00721046
IL16	0.29252851	0.98191217	0.94909919	1.01585958
TM6SF1	0.91898548	0.99649501	0.93130342	1.06625005
PLIN1	0.32705816	0.99174559	0.97544186	1.00832183
SEMA4B	0.02295392	1.003015	1.00041606	1.00562069
MSLN	0.32825045	1.00023339	0.99976555	1.00070145
ZG16B	0.67060666	1.00079313	0.99714339	1.00445623
PRSS22	0.14765139	1.00264358	0.99906668	1.00623329
SMIM22	0.29556371	1.00164958	0.99856085	1.00474786
C16orf89	0.41408146	0.99173284	0.97217134	1.01168794
PLA2G10	0.1051528	1.0107565	0.99776022	1.02392207
TMC7	0.00520372	1.03465185	1.01022186	1.05967263
TMC5	0.13846992	1.00276087	0.99911001	1.00642506
ACSM5	0.4483389	0.88804724	0.653341	1.20706936
HS3ST2	0.41284772	0.98392563	0.94649419	1.02283739
ERN2	0.45527663	1.00144096	0.99766284	1.00523339
PRKCB	0.25554718	0.97492374	0.93316374	1.01855254
CD19	0.37374214	0.99316945	0.9782827	1.00828275
TBX6	0.24383471	1.01197936	0.99191488	1.03244972
GDPD3	0.40779263	1.00576761	0.99216502	1.0195567
ITGAL	0.37574491	0.99093895	0.97117997	1.01109993
PRSS8	0.03017409	1.0018943	1.00018146	1.00361007
CETP	0.08231192	0.92360128	0.84439985	1.01023149
PLLP	0.07948536	1.01261713	0.99852623	1.02690687
BEAN1	0.00163468	1.07517456	1.02775647	1.12478039
ELMO3	0.32398751	1.00260628	0.99743374	1.00780565
HSD11B2	0.16479561	1.00145911	0.99940102	1.00352143
ESRP2	0.04074118	1.01525979	1.00063814	1.0300951
CDH3	0.00314308	1.00562295	1.00188786	1.00937197
NQO1	0.18010841	1.00089649	0.99958591	1.00220879
FA2H	0.22599784	1.00267107	0.99835064	1.0070102
CRISPLD2	0.69423062	1.00052616	0.99790567	1.00315352
FOXL1	0.21229035	1.00328019	0.9981306	1.00845634
SERPINF1	0.90909649	0.99995103	0.99911077	1.000792
ASPA	0.41223897	0.93711247	0.80235208	1.09450677
P2RX5	0.22250084	0.97961666	0.94773413	1.01257174
GP1BA	0.44055788	0.97549433	0.9158869	1.03898111
SCIMP	0.61359966	0.98839276	0.94460079	1.03421494
CLEC10A	0.36620847	0.99010562	0.96897922	1.01169262
CLDN7	0.28408891	1.00093322	0.99922634	1.00264302
ARHGEF15	0.13035266	0.96529341	0.92211065	1.01049844
MYH10	0.42175652	0.99475608	0.98207781	1.00759802
PIK3R5	0.41109119	0.98037835	0.93513084	1.02781521
GLP2R	0.47902333	0.98041356	0.92816113	1.03560762
GAS7	0.90640015	1.00095417	0.98516729	1.01699402
TRIM16	0.0112553	1.00829978	1.00187587	1.01476488
MFAP4	0.8268952	0.99986912	0.99869691	1.00104271
EVI2A	0.45936262	1.00577815	0.99054441	1.02124617
CCL2	0.76223724	0.99950451	0.99630055	1.00271878
CCL11	0.93869866	0.99929269	0.98143446	1.01747587
CCL8	0.42280372	1.01749026	0.9752535	1.06155624
MMP28	6.96E-06	1.00808332	1.00455056	1.0116285
CCL14	0.78620913	1.01470711	0.91310887	1.12760981

CCL4	0.95173308	0.99917226	0.97273726	1.02632565
GRB7	0.9917911	1.00001159	0.99780554	1.00222252
IKZF3	0.41713842	0.98751686	0.95800231	1.01794071
GSDMB	0.01354553	1.00900847	1.00185095	1.01621711
RAPGEFL1	0.00241188	1.00827764	1.00292281	1.01366106
TNS4	0.00234689	1.00298462	1.00106087	1.00491208
CCR7	0.21658809	0.99194007	0.9792886	1.00475497
KRT15	0.67609504	1.00293508	0.98924026	1.01681949
KRT19	0.00024692	1.00022559	1.00010496	1.00034623
KRT16	0.00159978	1.00087149	1.00033017	1.0014131
KRT17	0.1035805	1.00010907	0.99997774	1.00024041
AOC3	0.67295227	1.00133046	0.99516758	1.00753151
MEOX1	0.70214245	0.99498025	0.96964669	1.0209757
PLCD3	0.0046654	1.0114722	1.00351097	1.01949658
NGFR	0.12509411	0.99337946	0.98498283	1.00184767
ITGA3	0.00322176	1.00202347	1.00067669	1.00337206
TMEM92	0.00099145	1.02296179	1.00923215	1.03687821
CD79B	0.36626301	0.99715672	0.99101652	1.00333497
FAM20A	0.07671533	0.96989601	0.93761862	1.00328455
ABCA8	0.3985732	0.98256411	0.94324549	1.0235217
ABCA9	0.32483226	0.95229067	0.86398604	1.04962058
ABCA6	0.88422549	0.99225545	0.89366747	1.10171949
SLC16A5	0.02866953	1.00764101	1.00079365	1.01453522
ITGB4	0.00418297	1.00133274	1.0004205	1.00224581
EVPL	0.02247735	1.00746536	1.00105072	1.01392111
ST6GALNAC:	0.74921295	0.99969018	0.99779278	1.0015912
SLC16A3	0.02634669	1.0038329	1.00044982	1.00722743
COLEC12	0.70479679	1.00261442	0.98916233	1.01624945
ARHGAP28	0.61862545	1.05864356	0.84586997	1.32493908
ZNF521	0.17195824	1.02282105	0.99023502	1.0564794
TCF4	0.34066857	1.01368572	0.98573555	1.04242839
BCL2	0.29613086	0.9772004	0.93582169	1.02040872
SERPINB5	0.02080014	1.00336465	1.00051109	1.00622636
MADCAM1	0.58795138	0.99215586	0.96429038	1.02082657
GZMM	0.31986634	0.98073716	0.94386182	1.01905316
MISP	0.04459828	1.00166379	1.00004011	1.0032901
EFNA2	0.10487958	1.01257961	0.99739531	1.02799508
ADAMTSL5	0.05429141	1.04180375	0.9992457	1.08617436
LINGO3	0.03314171	0.77127077	0.60734515	0.97944075
TJP3	0.0818006	1.00476916	0.99939974	1.01016744
STAP2	0.31719102	1.00322038	0.99691982	1.00956076
FUT6	0.2618412	1.00766429	0.99431458	1.02119323
FUT3	0.05642149	1.00349191	0.99990463	1.00709207
SH2D3A	0.01205273	1.01544474	1.00336716	1.0276677
CD209	0.91837058	1.00206572	0.96329009	1.04240219
ICAM3	0.18624172	0.95044892	0.8814569	1.02484098
B3GNT3	0.00895846	1.0031127	1.00077758	1.00545327
FXYD3	0.24658095	1.00049938	0.99965481	1.00134467
LSR	0.58983928	1.00025946	0.99931643	1.00120338
CD22	0.3693598	0.99267173	0.9768574	1.00874207
HSPB6	0.43518996	0.99896608	0.99637392	1.00156498
C19orf33	0.00381454	1.00058576	1.00018889	1.00098278
MAP4K1	0.31719914	0.98729333	0.96286072	1.01234592
CAPN12	0.02700512	1.02742557	1.0030815	1.05236046
LGALS4	0.6545168	1.00005533	0.99981302	1.0002977
CYP2S1	0.60767177	1.00048757	0.99862749	1.00235112
CEACAM5	0.28301696	1.00009718	0.99991977	1.00027462
CD79A	0.35816673	0.99890976	0.99658823	1.00123671
KCNN4	0.02319175	1.00237669	1.00032451	1.00443308
CBLC	0.15324098	1.00209479	0.99922112	1.00497672
PPP1R13L	0.02124069	1.00662635	1.00098589	1.01229859
SULT2B1	0.00863296	1.01692112	1.00426612	1.02973559
FAM83E	0.63192067	1.00083554	0.99742129	1.00426147
CD37	0.29247259	0.9965742	0.99022721	1.00296187
KLK10	0.00036534	1.00343391	1.00154399	1.00532739
SIGLEC8	0.70175338	0.98986194	0.93956219	1.0428545
FPR1	0.63959884	1.00299111	0.99053075	1.01560822

FPR3	0.30013545	1.00426772	0.99621022	1.01239039
LILRB5	0.88596963	0.99194626	0.88815898	1.10786177
LILRA4	0.38897556	0.9540813	0.85731348	1.06177162
CDC42EP5	0.5652197	1.00069099	0.99833805	1.00304948
LILRA1	0.69046341	0.94601899	0.7199218	1.24312381
EPS8L1	0.02123597	1.00338951	1.0005051	1.00628224
TMEM238	0.27289437	0.99380244	0.98281923	1.0049084
SDCBP2	0.00954765	1.00300113	1.00073079	1.00527662
SIRPG	0.44483396	0.97867379	0.92602424	1.03431676
CPXM1	0.66009315	0.9991839	0.99555491	1.00282612
ADAM33	0.50390414	0.98489046	0.94188536	1.02985911
RASSF2	0.44105236	0.99532093	0.98351571	1.00726785
OVOL2	0.5736507	1.00625668	0.98463085	1.0283575
CD93	0.52568172	1.00180252	0.99624599	1.00739003
CST7	0.83205917	0.998928	0.98907438	1.0088798
ID1	0.19094154	1.00075426	0.99962408	1.00188571
SAMHD1	0.65427754	1.00106927	0.99639793	1.00576252
LBP	0.0958327	0.97062497	0.93715772	1.00528738
PPP1R16B	0.12423111	0.97584712	0.94589689	1.00674567
PI3	0.21863488	1.00053064	0.99968533	1.00137668
SLPI	0.46322206	1.00007821	0.99986926	1.00028721
PREX1	0.17174289	0.9887999	0.97295572	1.00490209
NFATC2	0.35442801	0.98973939	0.96836856	1.01158184
BCAS1	0.55821042	1.00140098	0.99671891	1.00610504
CBLN4	0.35134806	0.96416941	0.8929509	1.04106804
CASS4	0.65742679	0.96635468	0.83070293	1.12415804
ZNF831	0.0870275	0.84591476	0.69838159	1.02461432
SLCO4A1	0.04029797	1.00695838	1.0003068	1.01365418
SLC17A9	0.61046853	1.00272823	0.99227181	1.01329483
PTK6	0.00302317	1.00608417	1.0020587	1.01012581
SRMS	0.40247572	1.00926227	0.98771192	1.03128282
JAM2	0.56977182	0.98996695	0.95613584	1.02499512
CYYR1	0.77267924	0.99741	0.98001341	1.0151154
ADAMTS1	0.55549449	1.00174614	0.99595281	1.00757317
ERG	0.7727633	0.99431928	0.95660515	1.03352028
TFF2	0.35205081	1.00003977	0.99995601	1.00012354
TFF1	0.42208828	1.00002438	0.99996486	1.00008389
S100B	0.05500276	0.98918109	0.97825122	1.00023307
VPREB3	0.340335	0.99623371	0.98853645	1.00399091
NEFH	0.4963081	0.97263134	0.89789874	1.05358397
SLC5A4	0.82783375	0.97118796	0.74624068	1.2639435
CSF2RB	0.8029818	1.00141653	0.99034224	1.01261466
TST	0.64960082	1.00040135	0.99867103	1.00213467
MPST	0.91414121	1.00049979	0.99145313	1.009629
BAIAP2L2	0.11339033	1.00217092	0.99948396	1.00486511
GRAP2	0.0931232	0.88072772	0.7593794	1.02146743
TNFRSF13C	0.30914002	0.98981973	0.97049224	1.00953213
BIK	0.01408769	1.00449263	1.00090432	1.0080938
TSPO	0.04709033	1.00076467	1.00000983	1.00152009
FBLN1	0.86143389	1.00012464	0.99872599	1.00152524
P2RY8	0.21858591	0.98103749	0.95156858	1.01141902
TLR7	0.94390723	0.99762828	0.93377446	1.06584857
TLR8	0.52797594	1.01498544	0.96916557	1.06297156
GPM6B	0.1240633	0.9525924	0.8954187	1.01341673
CXorf21	0.91906389	1.00259078	0.95378194	1.05389736
SRPX	0.49387719	0.99813593	0.99281496	1.00348541
GPR34	0.78927898	1.00245171	0.98460974	1.020617
NUDT10	0.53986365	0.96920154	0.876954	1.07115267
EFNB1	0.00350053	1.00421781	1.00138475	1.00705887
NHSL2	0.38023846	0.93502367	0.80472861	1.08641503
CYSLTR1	0.00129525	1.06261253	1.0240145	1.10266544
P2RY10	0.41936587	0.98180203	0.93899854	1.02655667
GPR174	0.52845433	0.97048274	0.88415784	1.06523599
BTK	0.52832151	0.9904502	0.9613455	1.02043604
TCEAL7	0.19600737	0.98755563	0.96898693	1.00648016
NRK	0.32776692	1.0461038	0.95580831	1.14492953
VSIG1	0.57546741	1.00074271	0.99814577	1.0033464

CHRDL1	0.33931865	0.99627421	0.9886752	1.00393163
DOCK11	0.83158752	0.99693732	0.96914906	1.02552235
TMEM255A	0.21036591	0.94806917	0.87216784	1.0305759
SH2D1A	0.46349937	0.98237949	0.93678007	1.03019854
SASH3	0.39070716	0.99607794	0.98717969	1.0050564
GPC3	0.31441643	0.99322013	0.98014279	1.00647195
CD40LG	0.39032908	0.96542137	0.89093619	1.04613374
ARHGEF6	0.86743694	0.99779819	0.97230374	1.02396112
