# Supplementary material for: Molecular signatures of tumor progression in pancreatic adenocarcinoma identified by energy metabolism characteristics
Source: BMC Cancer. 2022 Apr 13;22:404. doi: 10.1186/s12885-022-09487-3 (PMC9006543; doi:10.1186/s12885-022-09487-3)
Supplement: Supplementary file 10 — Additional file 10. [file 12885_2022_9487_MOESM10_ESM.pdf]

Supplementary Table 5. GO and KEGG pathways significantly involved in the 743 co-expression DEGs

ID	descriptior	GeneRatio	BgRatio	pValue	FDR	qvalue	geneID	size	DB	enrichmentRatio
hsa04640	Hematopc	22/335	99/8011	7.15E-11	7.84E-09	7.06E-09	CD2/CD1C	22	pathway_K	0.065672
hsa04061	Viral prote	22/335	100/8011	8.82E-11	7.84E-09	7.06E-09	IL10/IL24/(	22	pathway_K	0.065672
hsa04060	Cytokine-(	39/335	294/8011	8.91E-11	7.84E-09	7.06E-09	LEPR/NGF/	39	pathway_K	0.116418
hsa05144	Malaria	15/335	50/8011	9.11E-10	6.01E-08	5.42E-08	ACKR1/SEI	15	pathway_K	0.044776
hsa04062	Chemokin	27/335	189/8011	1.69E-08	8.92E-07	8.04E-07	CXCR2/CC	27	pathway_K	0.080597
hsa04514	Cell adhes	23/335	147/8011	3.60E-08	1.58E-06	1.43E-06	NEGR1/CD	23	pathway_K	0.068657
hsa05340	Primary im	11/335	38/8011	2.53E-07	9.55E-06	8.61E-06	PTPRC/CD	11	pathway_K	0.032836
hsa04015	Rap1 signa	26/335	210/8011	5.77E-07	1.90E-05	1.71E-05	EPHA2/NG	26	pathway_K	0.077612
hsa04151	PI3K-Akt s	34/335	354/8011	4.26E-06	0.000125	0.000113	EPHA2/NG	34	pathway_K	0.101493
hsa04014	Ras signali	25/335	232/8011	1.20E-05	0.000316	0.000285	EPHA2/PL/	25	pathway_K	0.074627
hsa04360	Axon guid	20/335	181/8011	6.35E-05	0.001524	0.001374	EPHA2/EFI	20	pathway_K	0.059701
hsa04662	B cell recep	11/335	82/8011	0.000567	0.011844	0.010673	CR2/PRKC	11	pathway_K	0.032836
hsa04010	MAPK sign	25/335	295/8011	0.000583	0.011844	0.010673	EPHA2/NG	25	pathway_K	0.074627
hsa04672	Intestinal i	8/335	49/8011	0.000864	0.016296	0.014685	IL10/ITGA4	8	pathway_K	0.023881
hsa04080	Neuroactiv	27/335	340/8011	0.000967	0.016629	0.014985	GRIK3/LEP	27	pathway_K	0.080597
hsa04512	ECM-recep	11/335	88/8011	0.001037	0.016629	0.014985	LAMB3/IT(	11	pathway_K	0.032836
hsa04064	NF-kappa	12/335	102/8011	0.001071	0.016629	0.014985	ZAP70/IL1	12	pathway_K	0.035821
hsa04660	T cell recep	12/335	104/8011	0.001271	0.018649	0.016805	PTPRC/IL1	12	pathway_K	0.035821
hsa05150	Staphyloc	10/335	96/8011	0.006612	0.091865	0.082781	SELP/IL10/	10	pathway_K	0.029851
hsa05230	Central ca	8/335	69/8011	0.007776	0.102646	0.092496	SLC2A1/HI	8	pathway_K	0.023881
hsa05310	Asthma	5/335	31/8011	0.008605	0.108175	0.097478	FCER1A/IL	5	pathway_K	0.014925
hsa00982	Drug meta	8/335	72/8011	0.009995	0.11506	0.103682	GSTM5/FM	8	pathway_K	0.023881
hsa05146	Amoebiasi	10/335	102/8011	0.010024	0.11506	0.103682	CD1C/CD1	10	pathway_K	0.029851
hsa00140	Steroid ho	7/335	60/8011	0.012076	0.132835	0.1197	HSD11B1/	7	pathway_K	0.020896
hsa00980	Metabolisr	8/335	76/8011	0.013643	0.143809	0.129589	GSTM5/HS	8	pathway_K	0.023881
hsa00340	Histidine n	4/335	23/8011	0.014163	0.143809	0.129589	AOC1/ALD	4	pathway_K	0.01194
hsa04340	Hedgehog	6/335	50/8011	0.017229	0.162775	0.14668	IHH/BOC/S	6	pathway_K	0.01791
hsa04960	Aldosteror	5/335	37/8011	0.01801	0.162775	0.14668	SFN/SCNN	5	pathway_K	0.014925
hsa04670	Leukocyte	10/335	112/8011	0.018497	0.162775	0.14668	ITGA4/CLD	10	pathway_K	0.029851
hsa05145	Toxoplasm	10/335	112/8011	0.018497	0.162775	0.14668	IL10/LAME	10	pathway_K	0.029851
hsa04510	Focal adhe	15/335	199/8011	0.019483	0.165917	0.149511	LAMB3/IT(	15	pathway_K	0.044776
hsa05218	Melanoma	7/335	72/8011	0.030199	0.249141	0.224505	PDGFRA/F	7	pathway_K	0.020896
hsa04020	Calcium si	14/335	193/8011	0.031374	0.250989	0.22617	PTGER3/PT	14	pathway_K	0.041791
hsa00052	Galactose	4/335	31/8011	0.038798	0.292651	0.263713	GALE/HK2	4	pathway_K	0.01194
hsa00512	Mucin type	4/335	31/8011	0.038798	0.292651	0.263713	GALNT15/	4	pathway_K	0.01194
hsa01521	EGFR tyros	7/335	79/8011	0.046544	0.332268	0.299412	PDGFRA/K	7	pathway_K	0.020896
hsa05414	Dilated ca	8/335	96/8011	0.04714	0.332268	0.299412	ITGA4/SG(	8	pathway_K	0.023881
hsa05202	Transcripti	13/335	186/8011	0.047826	0.332268	0.299412	SLC45A3/F	13	pathway_K	0.038806
GO:00603	cell chemc	52/673	304/18670	4.38E-21	2.03E-17	1.56E-17	ARHGEF16	52	geneontol	0.077266
GO:00300	lymphocyt	55/673	353/18670	2.85E-20	6.61E-17	5.07E-17	PLA2G2D/	55	geneontol	0.081724
GO:00421	T cell activ	61/673	464/18670	1.40E-18	2.16E-15	1.66E-15	PLA2G2D/	61	geneontol	0.090639
GO:00509	leukocyte	62/673	499/18670	1.21E-17	1.40E-14	1.08E-14	PDE4B/S1I	62	geneontol	0.092125
GO:00700	chemokine	25/673	88/18670	3.83E-16	3.55E-13	2.72E-13	ACKR1/CX	25	geneontol	0.037147
GO:00457	positive re	52/673	403/18670	1.20E-15	9.23E-13	7.08E-13	S100A10/T	52	geneontol	0.077266
GO:00706	leukocyte	44/673	298/18670	1.48E-15	9.79E-13	7.51E-13	PLA2G2D/	44	geneontol	0.065379
GO:00302	T cell differ	39/673	240/18670	2.47E-15	1.43E-12	1.10E-12	PLA2G2D/	39	geneontol	0.057949
GO:19908	response t	25/673	97/18670	4.74E-15	2.19E-12	1.68E-12	ACKR1/CX	25	geneontol	0.037147
GO:19908	cellular res	25/673	97/18670	4.74E-15	2.19E-12	1.68E-12	ACKR1/CX	25	geneontol	0.037147
GO:00321	positive re	45/673	323/18670	6.30E-15	2.63E-12	2.02E-12	PTGER3/S1	45	geneontol	0.066865
GO:00466	lymphocyt	41/673	272/18670	6.81E-15	2.63E-12	2.02E-12	PLA2G2D/	41	geneontol	0.060921
GO:00329	mononucl	41/673	274/18670	8.81E-15	3.14E-12	2.41E-12	PLA2G2D/	41	geneontol	0.060921
GO:00509	regulation	36/673	217/18670	1.55E-14	5.11E-12	3.92E-12	S1PR1/S10	36	geneontol	0.053492
GO:00071	leukocyte	45/673	337/18670	3.04E-14	9.40E-12	7.21E-12	PLA2G2D/	45	geneontol	0.066865
GO:00305	leukocyte	36/673	224/18670	4.23E-14	1.23E-11	9.40E-12	PDE4B/S1I	36	geneontol	0.053492
GO:00430	extracellul	50/673	422/18670	1.27E-13	3.47E-11	2.66E-11	DDR2/DPT	50	geneontol	0.074294
GO:00508	regulation	42/673	314/18670	2.12E-13	5.45E-11	4.18E-11	PLA2G2D/	42	geneontol	0.062407
GO:00509	positive re	27/673	135/18670	3.09E-13	7.54E-11	5.78E-11	S1PR1/S10	27	geneontol	0.040119
GO:00072	positive re	42/673	319/18670	3.64E-13	8.42E-11	6.46E-11	PTGER3/PT	42	geneontol	0.062407
GO:00512	regulation	53/673	485/18670	5.99E-13	1.32E-10	1.01E-10	PLA2G2D/	53	geneontol	0.078752
GO:00301	extracellul	45/673	368/18670	7.32E-13	1.54E-10	1.18E-10	DDR2/DPT	45	geneontol	0.066865
GO:00421	B cell proli	22/673	95/18670	2.20E-12	4.43E-10	3.40E-10	FCRL3/MN	22	geneontol	0.032689
GO:00197	calcium-m	33/673	218/18670	2.73E-12	5.27E-10	4.04E-10	PTGFR/SEL	33	geneontol	0.049034
GO:00315	cell-substr	43/673	354/18670	3.02E-12	5.59E-10	4.29E-10	TACSTD2/	43	geneontol	0.063893
GO:00026	regulation	31/673	196/18670	3.90E-12	6.94E-10	5.33E-10	S100A14/S	31	geneontol	0.046062
GO:00975	myeloid le	32/673	210/18670	4.90E-12	8.40E-10	6.44E-10	PDE4B/S1(	32	geneontol	0.047548
GO:00550	calcium io	50/673	471/18670	8.17E-12	1.35E-09	1.04E-09	PTGER3/PT	50	geneontol	0.074294
GO:00514	regulation	42/673	357/18670	1.55E-11	2.47E-09	1.90E-09	PTGER3/PT	42	geneontol	0.062407
GO:00506	regulation	31/673	208/18670	1.91E-11	2.94E-09	2.26E-09	PLA2G2D/	31	geneontol	0.046062
GO:00329	regulation	31/673	209/18670	2.16E-11	3.21E-09	2.47E-09	PLA2G2D/	31	geneontol	0.046062
GO:00706	regulation	32/673	222/18670	2.22E-11	3.21E-09	2.47E-09	PLA2G2D/	32	geneontol	0.047548
GO:19030	regulation	38/673	304/18670	2.41E-11	3.39E-09	2.60E-09	PLA2G2D/	38	geneontol	0.056464

GO:00508(	positive re	44/673	394/18670	2.89E-11	3.94E-09	3.02E-09	CD2/FCRL3	44	geneontol	0.065379
GO:00068	cellular cal	47/673	458/18670	1.13E-10	1.47E-08	1.13E-08	PTGER3/PT	47	geneontol	0.069837
GO:00026(	positive re	42/673	380/18670	1.14E-10	1.47E-08	1.13E-08	CD2/FCRL3	42	geneontol	0.062407
GO:00026	positive re	23/673	128/18670	1.74E-10	2.18E-08	1.67E-08	S100A14/S	23	geneontol	0.034175
GO:19030	positive re	30/673	218/18670	3.01E-10	3.66E-08	2.81E-08	PTPRC/NL	30	geneontol	0.044577
GO:00181(	peptidyl-ty	40/673	363/18670	3.51E-10	4.16E-08	3.19E-08	EPHA2/TA	40	geneontol	0.059435
GO:00182	peptidyl-ty	40/673	366/18670	4.49E-10	5.19E-08	3.98E-08	EPHA2/TA	40	geneontol	0.059435
GO:00716	granulocyt	22/673	123/18670	4.75E-10	5.37E-08	4.12E-08	PDE4B/S10	22	geneontol	0.032689
GO:00466	alpha-beta	23/673	138/18670	8.22E-10	9.02E-08	6.92E-08	PLA2G2D/	23	geneontol	0.034175
GO:00224(	positive re	32/673	255/18670	8.38E-10	9.02E-08	6.92E-08	PTPRC/IL1	32	geneontol	0.047548
GO:00199	second-m	44/673	439/18670	9.54E-10	1.00E-07	7.70E-08	PTGER3/PT	44	geneontol	0.065379
GO:00508	positive re	28/673	202/18670	9.97E-10	1.03E-07	7.87E-08	PTPRC/NL	28	geneontol	0.041605
GO:00024	immune re	46/673	473/18670	1.05E-09	1.05E-07	8.07E-08	PDE4B/MU	46	geneontol	0.068351
GO:00433	negative T	8/673	12/18670	1.19E-09	1.17E-07	9.01E-08	PTPRC/ZA	8	geneontol	0.011887
GO:00975	granulocyt	23/673	141/18670	1.27E-09	1.23E-07	9.41E-08	PDE4B/S10	23	geneontol	0.034175
GO:00725(	cellular div	47/673	493/18670	1.32E-09	1.24E-07	9.53E-08	PTGER3/PT	47	geneontol	0.069837
GO:00421	B cell activ	35/673	310/18670	2.38E-09	2.20E-07	1.69E-07	FCRL3/MN	35	geneontol	0.052006
GO:00726	lymphocyt	20/673	111/18670	2.56E-09	2.33E-07	1.79E-07	S1PR1/ZAI	20	geneontol	0.029718
GO:00071(	cell-matrix	29/673	225/18670	2.80E-09	2.49E-07	1.91E-07	S100A10/D	29	geneontol	0.043091
GO:00329	regulation	37/673	343/18670	2.93E-09	2.56E-07	1.96E-07	TACSTD2/	37	geneontol	0.054978
GO:00026	regulation	20/673	114/18670	4.16E-09	3.50E-07	2.69E-07	S100A14/C	20	geneontol	0.029718
GO:00108	regulation	28/673	215/18670	4.16E-09	3.50E-07	2.69E-07	TACSTD2/	28	geneontol	0.041605
GO:00026	negative re	44/673	463/18670	4.95E-09	4.09E-07	3.14E-07	PLA2G2D/	44	geneontol	0.065379
GO:00305	neutrophil	19/673	104/18670	5.04E-09	4.10E-07	3.14E-07	PDE4B/S10	19	geneontol	0.028232
GO:00509(	leukocyte	10/673	25/18670	6.94E-09	5.51E-07	4.23E-07	SELP/SELL	10	geneontol	0.014859
GO:00450	T cell selec	13/673	47/18670	7.02E-09	5.51E-07	4.23E-07	LY9/PTPRC	13	geneontol	0.019316
GO:00224(	regulation	40/673	403/18670	7.41E-09	5.72E-07	4.39E-07	PLA2G2D/	40	geneontol	0.059435
GO:19902(	neutrophil	20/673	118/18670	7.71E-09	5.85E-07	4.49E-07	PDE4B/S10	20	geneontol	0.029718
GO:00329	regulation	39/673	388/18670	8.07E-09	6.03E-07	4.62E-07	TACSTD2/	39	geneontol	0.057949
GO:00301	B cell diffe	21/673	131/18670	9.16E-09	6.73E-07	5.17E-07	FCRL3/PTF	21	geneontol	0.031204
GO:00433(	positive T	11/673	34/18670	1.65E-08	1.20E-06	9.17E-07	LY9/PTPRC	11	geneontol	0.016345
GO:00315	actin cytos	18/673	101/18670	1.92E-08	1.34E-06	1.03E-06	S1PR1/PLE	18	geneontol	0.026746
GO:00466	alpha-beta	18/673	101/18670	1.92E-08	1.34E-06	1.03E-06	PLA2G2D/	18	geneontol	0.026746
GO:00450(	negative th	7/673	11/18670	2.23E-08	1.54E-06	1.18E-06	PTPRC/ZA	7	geneontol	0.010401
GO:00308	regulation	14/673	61/18670	2.57E-08	1.75E-06	1.34E-06	FCRL3/MN	14	geneontol	0.020802
GO:00456	regulation	23/673	169/18670	4.42E-08	2.96E-06	2.27E-06	CD2/FCRL3	23	geneontol	0.034175
GO:00512	positive re	34/673	334/18670	5.44E-08	3.60E-06	2.76E-06	FCRL3/PTF	34	geneontol	0.05052
GO:00025	monocyte	14/673	65/18670	6.08E-08	3.97E-06	3.04E-06	S100A12/S	14	geneontol	0.020802
GO:00485(	digestive t	20/673	134/18670	7.09E-08	4.56E-06	3.50E-06	IHH/CLDN	20	geneontol	0.029718
GO:00024(	dendritic c	9/673	24/18670	7.84E-08	4.97E-06	3.81E-06	CXCR2/CC	9	geneontol	0.013373
GO:00072(	Rho protei	25/673	203/18670	8.61E-08	5.38E-06	4.13E-06	ARHGEF16	25	geneontol	0.037147
GO:00072(	phospholip	17/673	100/18670	9.77E-08	6.03E-06	4.63E-06	PTGER3/S1	17	geneontol	0.02526
GO:00355	G protein-	7/673	13/18670	1.09E-07	6.63E-06	5.09E-06	GPR171/P2	7	geneontol	0.010401
GO:00506	regulation	36/673	378/18670	1.20E-07	7.19E-06	5.52E-06	SFN/TACST	36	geneontol	0.053492
GO:00716	mononucle	16/673	90/18670	1.22E-07	7.24E-06	5.55E-06	S100A12/S	16	geneontol	0.023774
GO:00074(	axonogene	41/673	468/18670	1.59E-07	9.19E-06	7.05E-06	EPHA2/NE	41	geneontol	0.060921
GO:00022	lymphocyt	23/673	181/18670	1.59E-07	9.19E-06	7.05E-06	CD1C/LY9/	23	geneontol	0.034175
GO:00506	epithelial c	39/673	434/18670	1.66E-07	9.46E-06	7.26E-06	EPHA2/SFI	39	geneontol	0.057949
GO:00421	regulation	21/673	156/18670	2.05E-07	1.15E-05	8.81E-06	PLA2G2D/	21	geneontol	0.031204
GO:00451	cellular ext	13/673	61/18670	2.06E-07	1.15E-05	8.81E-06	SELP/SELL	13	geneontol	0.019316
GO:00506	positive re	19/673	130/18670	2.11E-07	1.16E-05	8.91E-06	FCRL3/PTF	19	geneontol	0.028232
GO:00420	T cell proli	23/673	184/18670	2.15E-07	1.17E-05	8.97E-06	PLA2G2D/	23	geneontol	0.034175
GO:00329	positive re	19/673	131/18670	2.38E-07	1.28E-05	9.84E-06	FCRL3/PTF	19	geneontol	0.028232
GO:20001(	regulation	15/673	83/18670	2.41E-07	1.28E-05	9.85E-06	EFNA1/IL1	15	geneontol	0.022288
GO:00363	dendritic c	9/673	27/18670	2.55E-07	1.34E-05	1.03E-05	CXCR2/CC	9	geneontol	0.013373
GO:00551	digestive s	20/673	146/18670	2.99E-07	1.55E-05	1.19E-05	IHH/CLDN	20	geneontol	0.029718
GO:00716	regulation	11/673	44/18670	3.19E-07	1.64E-05	1.26E-05	S100A14/C	11	geneontol	0.016345
GO:00108	positive re	18/673	121/18670	3.35E-07	1.70E-05	1.31E-05	S100A10/V	18	geneontol	0.026746
GO:00355	purinergic	8/673	21/18670	3.67E-07	1.83E-05	1.40E-05	GPR171/P2	8	geneontol	0.011887
GO:00450(	thymic T c	8/673	21/18670	3.67E-07	1.83E-05	1.40E-05	PTPRC/ZA	8	geneontol	0.011887
GO:00482	lymphocyt	13/673	64/18670	3.73E-07	1.84E-05	1.41E-05	CCR2/CXC	13	geneontol	0.019316
GO:00026	positive re	15/673	87/18670	4.57E-07	2.23E-05	1.71E-05	S100A14/C	15	geneontol	0.022288
GO:00312	T cell costi	12/673	56/18670	5.69E-07	2.74E-05	2.10E-05	CD28/ICOS	12	geneontol	0.017831
GO:00455	regulation	19/673	139/18670	6.10E-07	2.88E-05	2.21E-05	CD2/PTPR	19	geneontol	0.028232
GO:00706(	positive re	19/673	139/18670	6.10E-07	2.88E-05	2.21E-05	FCRL3/PTF	19	geneontol	0.028232
GO:00510	actin filam	20/673	153/18670	6.43E-07	3.01E-05	2.31E-05	TACSTD2/	20	geneontol	0.029718
GO:00350	regulation	19/673	140/18670	6.82E-07	3.16E-05	2.42E-05	ARHGEF16	19	geneontol	0.028232
GO:00312	lymphocyt	12/673	57/18670	6.97E-07	3.20E-05	2.45E-05	CD28/ICOS	12	geneontol	0.017831
GO:00074	axon guida	28/673	276/18670	8.70E-07	3.95E-05	3.03E-05	EPHA2/NE	28	geneontol	0.041605
GO:00720(	renal syste	29/673	293/18670	9.27E-07	4.16E-05	3.19E-05	TACSTD2/	29	geneontol	0.043091
GO:00974	neuron pro	28/673	277/18670	9.36E-07	4.17E-05	3.20E-05	EPHA2/NE	28	geneontol	0.041605
GO:00514	positive re	9/673	31/18670	9.63E-07	4.25E-05	3.26E-05	S1PR1/GPI	9	geneontol	0.013373
GO:00615	actin filam	20/673	157/18670	9.75E-07	4.26E-05	3.27E-05	TACSTD2/	20	geneontol	0.029718

GO:00246	adaptive ir	33/673	361/18670	1.02E-06	4.39E-05	3.37E-05	CD1C/CD1	33	geneontol	0.049034
GO:00507	regulation	40/673	485/18670	1.07E-06	4.57E-05	3.50E-05	PTGER3/S	40	geneontol	0.059435
GO:00016	urogenital	31/673	330/18670	1.21E-06	5.10E-05	3.91E-05	TACSTD2/	31	geneontol	0.046062
GO:00324	response t	31/673	330/18670	1.21E-06	5.10E-05	3.91E-05	PDE4B/PT	31	geneontol	0.046062
GO:00435	positive re	9/673	32/18670	1.30E-06	5.41E-05	4.15E-05	P2RY12/PI	9	geneontol	0.013373
GO:00508	antigen re	30/673	316/18670	1.44E-06	5.97E-05	4.58E-05	PDE4B/FC	30	geneontol	0.044577
GO:00518	positive re	21/673	176/18670	1.55E-06	6.36E-05	4.88E-05	CD28/ICO	21	geneontol	0.031204
GO:00506	positive re	23/673	206/18670	1.61E-06	6.53E-05	5.01E-05	IL10/OSR1	23	geneontol	0.034175
GO:00434	protein kir	27/673	269/18670	1.67E-06	6.74E-05	5.17E-05	EPHA2/XD	27	geneontol	0.040119
GO:00026	regulation	38/673	458/18670	1.72E-06	6.85E-05	5.26E-05	FCRL3/CD	38	geneontol	0.056464
GO:00466	positive re	12/673	62/18670	1.81E-06	7.14E-05	5.48E-05	PTPRC/NL	12	geneontol	0.017831
GO:00430	regulation	39/673	479/18670	1.99E-06	7.81E-05	5.99E-05	ARHGEF16	39	geneontol	0.057949
GO:00016	ameboidal	38/673	461/18670	2.01E-06	7.81E-05	5.99E-05	EPHA2/TA	38	geneontol	0.056464
GO:19021	regulation	27/673	272/18670	2.07E-06	7.99E-05	6.13E-05	TAL1/CD2	27	geneontol	0.040119
GO:19055	macropha	11/673	53/18670	2.36E-06	9.02E-05	6.92E-05	CX3CR1/C	11	geneontol	0.016345
GO:00716	positive re	8/673	26/18670	2.40E-06	9.11E-05	6.99E-05	S100A14/	8	geneontol	0.011887
GO:00017	morphoge	22/673	196/18670	2.47E-06	9.30E-05	7.13E-05	EPHA2/TA	22	geneontol	0.032689
GO:00226	regulation	39/673	484/18670	2.56E-06	9.56E-05	7.33E-05	TACSTD2/	39	geneontol	0.057949
GO:00140	positive re	14/673	87/18670	2.59E-06	9.61E-05	7.37E-05	SELP/CD2	14	geneontol	0.020802
GO:00027	regulation	18/673	139/18670	2.66E-06	9.68E-05	7.43E-05	FCRL3/PTF	18	geneontol	0.026746
GO:00507	positive re	19/673	153/18670	2.67E-06	9.68E-05	7.43E-05	PTGER3/S	19	geneontol	0.028232
GO:00611	morphoge	21/673	182/18670	2.68E-06	9.68E-05	7.43E-05	EPHA2/TA	21	geneontol	0.031204
GO:00022	response t	31/673	343/18670	2.73E-06	9.80E-05	7.52E-05	PDE4B/PT	31	geneontol	0.046062
GO:00617	leukocyte	10/673	44/18670	2.83E-06	0.000101	7.74E-05	SELP/SELL	10	geneontol	0.014859
GO:00450	positive th	6/673	13/18670	2.96E-06	0.000105	8.04E-05	PTPRC/ZA	6	geneontol	0.008915
GO:01100	regulation	26/673	261/18670	2.99E-06	0.000105	8.04E-05	TACSTD2/	26	geneontol	0.038633
GO:00508	regulation	12/673	65/18670	3.05E-06	0.000106	8.14E-05	FCRL3/PTF	12	geneontol	0.017831
GO:00018	kidney dev	27/673	278/18670	3.14E-06	0.000108	8.32E-05	TACSTD2/	27	geneontol	0.040119
GO:00018	negative re	28/673	296/18670	3.46E-06	0.000119	9.10E-05	EPHA2/CD	28	geneontol	0.041605
GO:00070	actin filam	34/673	400/18670	3.56E-06	0.000121	9.30E-05	TACSTD2/	34	geneontol	0.05052
GO:00313	positive re	33/673	383/18670	3.70E-06	0.000125	9.60E-05	NEGR1/EP	33	geneontol	0.049034
GO:00902	positive re	9/673	36/18670	3.83E-06	0.000127	9.78E-05	P2RY12/PI	9	geneontol	0.013373
GO:19026	regulation	9/673	36/18670	3.83E-06	0.000127	9.78E-05	IL1R1/CXC	9	geneontol	0.013373
GO:00508	B cell rece	17/673	129/18670	3.94E-06	0.00013	9.99E-05	FCRL3/MN	17	geneontol	0.02526
GO:00071	heterophil	10/673	46/18670	4.36E-06	0.000142	0.000109	CADM3/SI	10	geneontol	0.014859
GO:00336	regulation	10/673	46/18670	4.36E-06	0.000142	0.000109	EPHA2/EF	10	geneontol	0.014859
GO:19013	regulation	35/673	422/18670	4.44E-06	0.000144	0.00011	EPHA2/NP	35	geneontol	0.052006
GO:00718	leukocyte	15/673	104/18670	4.70E-06	0.000151	0.000116	EFNA1/IL1	15	geneontol	0.022288
GO:00336	cell adhesi	12/673	68/18670	5.00E-06	0.000158	0.000122	EPHA2/EF	12	geneontol	0.017831
GO:00509	positive ch	12/673	68/18670	5.00E-06	0.000158	0.000122	S1PR1/CC	12	geneontol	0.017831
GO:00326	regulation	19/673	160/18670	5.20E-06	0.000164	0.000126	CD2/SLAM	19	geneontol	0.028232
GO:00019	regulation	16/673	119/18670	5.80E-06	0.000181	0.000139	S100A10/I	16	geneontol	0.023774
GO:00085	epidermis	37/673	464/18670	5.87E-06	0.000182	0.00014	EPHA2/SF	37	geneontol	0.054978
GO:19026	positive re	8/673	29/18670	5.99E-06	0.000185	0.000142	IL1R1/CXC	8	geneontol	0.011887
GO:00987	cell-cell ac	26/673	273/18670	6.79E-06	0.000205	0.000158	NEXN/CAI	26	geneontol	0.038633
GO:00330	T cell diffe	12/673	70/18670	6.83E-06	0.000205	0.000158	PTPRC/ZA	12	geneontol	0.017831
GO:00326	tumor nec	19/673	163/18670	6.84E-06	0.000205	0.000158	CD2/SLAM	19	geneontol	0.028232
GO:19035	regulation	19/673	163/18670	6.84E-06	0.000205	0.000158	CD2/SLAM	19	geneontol	0.028232
GO:00027	regulation	18/673	149/18670	7.19E-06	0.000215	0.000165	CD1C/CD1	18	geneontol	0.026746
GO:00074	mesoderm	17/673	135/18670	7.34E-06	0.000218	0.000167	EPHA2/TA	17	geneontol	0.02526
GO:00421	positive re	14/673	95/18670	7.52E-06	0.000222	0.00017	PTPRC/ZA	14	geneontol	0.020802
GO:00900	regulation	8/673	30/18670	7.92E-06	0.000232	0.000178	CXCR2/SL	8	geneontol	0.011887
GO:00518	regulation	24/673	244/18670	8.85E-06	0.000258	0.000198	EPHA2/XD	24	geneontol	0.035661
GO:00070	cell-substr	14/673	97/18670	9.63E-06	0.000278	0.000214	S100A10/L	14	geneontol	0.020802
GO:00457	regulation	32/673	383/18670	9.78E-06	0.000281	0.000216	EPHA2/NP	32	geneontol	0.047548
GO:00512	negative re	32/673	384/18670	1.03E-05	0.000294	0.000225	PODN/TA	32	geneontol	0.047548
GO:00346	response t	8/673	31/18670	1.03E-05	0.000294	0.000225	PTGFR/TG	8	geneontol	0.011887
GO:00717	tumor nec	19/673	168/18670	1.06E-05	0.0003	0.00023	CD2/SLAM	19	geneontol	0.028232
GO:00023	B cell activ	12/673	73/18670	1.07E-05	0.0003	0.00023	PTPRC/IL1	12	geneontol	0.017831
GO:00703	positive re	22/673	215/18670	1.11E-05	0.000311	0.000238	SLAMF1/P	22	geneontol	0.032689
GO:00327	positive re	7/673	23/18670	1.13E-05	0.000314	0.000241	NLRP3/CD	7	geneontol	0.010401
GO:00017	leukocyte	13/673	86/18670	1.19E-05	0.000328	0.000252	PDE4B/CX	13	geneontol	0.019316
GO:00435	positive re	33/673	405/18670	1.20E-05	0.000328	0.000252	ARHGEF16	33	geneontol	0.049034
GO:00024	lymphocyt	30/673	352/18670	1.26E-05	0.000341	0.000262	CD1C/CD1	30	geneontol	0.044577
GO:00326	negative re	6/673	16/18670	1.26E-05	0.000341	0.000262	SLAMF1/IL	6	geneontol	0.008915
GO:00326	interferon-	15/673	113/18670	1.32E-05	0.000356	0.000273	PDE4B/CD	15	geneontol	0.022288
GO:00355	purinergic	8/673	32/18670	1.33E-05	0.000357	0.000274	GPR171/P	8	geneontol	0.011887
GO:00315	ruffle orga	10/673	52/18670	1.39E-05	0.000371	0.000284	TACSTD2/	10	geneontol	0.014859
GO:00510	regulation	29/673	338/18670	1.54E-05	0.0004	0.000307	ARHGEF16	29	geneontol	0.043091
GO:00355	G protein-	7/673	24/18670	1.55E-05	0.0004	0.000307	GPR171/P	7	geneontol	0.010401
GO:00508	positive re	7/673	24/18670	1.55E-05	0.0004	0.000307	PTPRC/TR	7	geneontol	0.010401
GO:00900	positive re	7/673	24/18670	1.55E-05	0.0004	0.000307	CXCR2/TH	7	geneontol	0.010401
GO:20010	regulation	7/673	24/18670	1.55E-05	0.0004	0.000307	KDR/CXCL	7	geneontol	0.010401

GO:00244	productior	26/673	286/18670	1.55E-05	0.0004	0.000307	FCRL3/PTF	26	geneontol	0.038633
GO:00451	cell fate cc	25/673	270/18670	1.65E-05	0.000422	0.000324	TAL1/LY9/	25	geneontol	0.037147
GO:00069	cellular de	10/673	53/18670	1.66E-05	0.000423	0.000324	MNDA/CX	10	geneontol	0.014859
GO:00326	interleukin	8/673	33/18670	1.71E-05	0.000432	0.000331	NLRP3/CD	8	geneontol	0.011887
GO:00726	T cell migr	11/673	65/18670	1.87E-05	0.000471	0.000361	S1PR1/ZAI	11	geneontol	0.016345
GO:00028	regulation	17/673	145/18670	1.91E-05	0.000476	0.000365	CD1C/CD1	17	geneontol	0.02526
GO:00400	negative re	32/673	396/18670	1.91E-05	0.000476	0.000365	PODN/TAC	32	geneontol	0.047548
GO:00028	regulation	18/673	160/18670	1.94E-05	0.000479	0.000368	CD1C/CD1	18	geneontol	0.026746
GO:00326	regulation	10/673	54/18670	1.97E-05	0.000486	0.000373	SLAMF1/IL	10	geneontol	0.014859
GO:00507	positive re	20/673	192/18670	2.13E-05	0.000523	0.000401	TAL1/EFN	20	geneontol	0.029718
GO:19016	response t	20/673	193/18670	2.30E-05	0.00056	0.00043	PTGFR/TG	20	geneontol	0.029718
GO:00026	regulation	5/673	11/18670	2.31E-05	0.00056	0.00043	FGL2/CCL	5	geneontol	0.007429
GO:00435	regulation	10/673	55/18670	2.34E-05	0.000563	0.000432	P2RY12/PI	10	geneontol	0.014859
GO:00026	negative re	15/673	120/18670	2.74E-05	0.000653	0.000501	FCRL3/CD	15	geneontol	0.022288
GO:00226	gland mor	15/673	120/18670	2.74E-05	0.000653	0.000501	EPHA2/ELI	15	geneontol	0.022288
GO:00326	interleukin	10/673	56/18670	2.75E-05	0.000654	0.000502	SLAMF1/IL	10	geneontol	0.014859
GO:00466	regulation	13/673	93/18670	2.83E-05	0.000667	0.000512	PTPRC/NL	13	geneontol	0.019316
GO:00480	phosphatic	19/673	181/18670	3.06E-05	0.000719	0.000552	NGF/SELP	19	geneontol	0.028232
GO:00456	positive re	13/673	94/18670	3.17E-05	0.000742	0.000569	PTPRC/NL	13	geneontol	0.019316
GO:00018	positive re	35/673	464/18670	3.37E-05	0.000785	0.000602	PDE4B/CD	35	geneontol	0.052006
GO:00326	regulation	7/673	27/18670	3.62E-05	0.000839	0.000644	NLRP3/CD	7	geneontol	0.010401
GO:00026	positive re	21/673	216/18670	3.78E-05	0.00087	0.000668	CD1C/CD1	21	geneontol	0.031204
GO:00458	positive re	17/673	153/18670	3.84E-05	0.000873	0.00067	TSPAN1/S	17	geneontol	0.02526
GO:00480	inositol lip	19/673	184/18670	3.84E-05	0.000873	0.00067	NGF/SELP	19	geneontol	0.028232
GO:00024	dendritic c	5/673	12/18670	3.85E-05	0.000873	0.00067	FGL2/CCL	5	geneontol	0.007429
GO:20001	negative re	9/673	47/18670	3.89E-05	0.000878	0.000674	EFNA1/CX	9	geneontol	0.013373
GO:00140	regulation	15/673	124/18670	4.04E-05	0.000908	0.000696	SELP/CD2	15	geneontol	0.022288
GO:00455	positive re	12/673	83/18670	4.06E-05	0.000908	0.000696	PTPRC/NL	12	geneontol	0.017831
GO:00027	regulation	20/673	201/18670	4.13E-05	0.00092	0.000706	CD1C/CD1	20	geneontol	0.029718
GO:00467	protein au	22/673	235/18670	4.43E-05	0.00098	0.000752	DDR2/PTP	22	geneontol	0.032689
GO:00322	regulation	13/673	97/18670	4.45E-05	0.00098	0.000752	TACSTD2/	13	geneontol	0.019316
GO:19037	positive re	9/673	48/18670	4.63E-05	0.001017	0.00078	P2RY12/PI	9	geneontol	0.013373
GO:00300	contractile	13/673	98/18670	4.96E-05	0.001078	0.000827	TACSTD2/	13	geneontol	0.019316
GO:00431	stress fiber	13/673	98/18670	4.96E-05	0.001078	0.000827	TACSTD2/	13	geneontol	0.019316
GO:00518	regulation	10/673	60/18670	5.12E-05	0.001103	0.000846	S100A10/	10	geneontol	0.014859
GO:00901	regulation	10/673	60/18670	5.12E-05	0.001103	0.000846	S100A10/	10	geneontol	0.014859
GO:00015	ossificatior	31/673	398/18670	5.18E-05	0.00111	0.000852	EPHA2/S1	31	geneontol	0.046062
GO:00465	regulation	22/673	238/18670	5.37E-05	0.001145	0.000878	ARHGEF16	22	geneontol	0.032689
GO:00420	gliogenesi	25/673	290/18670	5.44E-05	0.001155	0.000886	TAL1/LEPF	25	geneontol	0.037147
GO:00026	negative re	9/673	49/18670	5.50E-05	0.001157	0.000887	CD200R1/	9	geneontol	0.013373
GO:00485	digestive t	9/673	49/18670	5.50E-05	0.001157	0.000887	IHH/PDGF	9	geneontol	0.013373
GO:00507	regulation	23/673	256/18670	5.69E-05	0.001193	0.000915	TAL1/EFN	23	geneontol	0.034175
GO:00162	O-glycan	10/673	61/18670	5.93E-05	0.001237	0.000949	MUC1/B3C	10	geneontol	0.014859
GO:00350	positive re	7/673	29/18670	5.98E-05	0.001242	0.000953	GPR35/LP	7	geneontol	0.010401
GO:00024	compleme	5/673	13/18670	6.06E-05	0.001253	0.000962	CR2/CR1/	5	geneontol	0.007429
GO:00302	bone mine	14/673	114/18670	6.13E-05	0.001261	0.000967	S1PR1/DD	14	geneontol	0.020802
GO:00434	positive re	23/673	258/18670	6.42E-05	0.00131	0.001005	NGF/S100	23	geneontol	0.034175
GO:01200	regulation	18/673	175/18670	6.42E-05	0.00131	0.001005	EPHA2/TA	18	geneontol	0.026746
GO:00326	regulation	13/673	101/18670	6.82E-05	0.001386	0.001063	PDE4B/CD	13	geneontol	0.019316
GO:20001	negative re	28/673	349/18670	7.10E-05	0.001435	0.001101	PODN/TAC	28	geneontol	0.041605
GO:00017	mesoderm	11/673	75/18670	7.39E-05	0.001487	0.001141	EPHA2/TA	11	geneontol	0.016345
GO:00604	regulation	18/673	177/18670	7.45E-05	0.001493	0.001145	EPHA2/TA	18	geneontol	0.026746
GO:00327	positive re	7/673	30/18670	7.56E-05	0.001504	0.001154	PDE4B/PTI	7	geneontol	0.010401
GO:00018	mesoneph	13/673	102/18670	7.57E-05	0.001504	0.001154	TACSTD2/	13	geneontol	0.019316
GO:00303	negative re	27/673	334/18670	8.38E-05	0.001657	0.001271	PODN/TAC	27	geneontol	0.040119
GO:00488	homeostas	22/673	246/18670	8.78E-05	0.001729	0.001326	TAL1/PDE	22	geneontol	0.032689
GO:00072	Ras protei	33/673	448/18670	8.88E-05	0.00173	0.001327	ARHGEF16	33	geneontol	0.049034
GO:00703	ERK1 and	26/673	317/18670	8.90E-05	0.00173	0.001327	EPHA2/SL	26	geneontol	0.038633
GO:00140	phosphatic	16/673	148/18670	8.96E-05	0.00173	0.001327	SELP/CD2	16	geneontol	0.023774
GO:00435	regulation	10/673	64/18670	9.04E-05	0.00173	0.001327	P2RY12/PI	10	geneontol	0.014859
GO:00465	positive re	10/673	64/18670	9.04E-05	0.00173	0.001327	NGF/GPR3	10	geneontol	0.014859
GO:00509	negative re	10/673	64/18670	9.04E-05	0.00173	0.001327	SEMA3G/	10	geneontol	0.014859
GO:00308	positive re	8/673	41/18670	9.11E-05	0.00173	0.001327	FCRL3/PTF	8	geneontol	0.011887
GO:00468	bone remc	12/673	90/18670	9.12E-05	0.00173	0.001327	EPHA2/LEI	12	geneontol	0.017831
GO:00017	immunolo	5/673	14/18670	9.15E-05	0.00173	0.001327	DOCK2/C	5	geneontol	0.007429
GO:00433	CD8-posit	5/673	14/18670	9.15E-05	0.00173	0.001327	EOMES/TN	5	geneontol	0.007429
GO:00703	regulation	25/673	300/18670	9.39E-05	0.001767	0.001355	EPHA2/SL	25	geneontol	0.037147
GO:00483	mesoderm	11/673	77/18670	9.44E-05	0.001767	0.001355	EPHA2/TA	11	geneontol	0.016345
GO:00357	endothelia	7/673	31/18670	9.46E-05	0.001767	0.001355	KDR/CXCL	7	geneontol	0.010401
GO:00336	integrin ac	6/673	22/18670	9.76E-05	0.001808	0.001387	SELP/PLEK	6	geneontol	0.008915
GO:00486	reproducti	32/673	431/18670	9.77E-05	0.001808	0.001387	GJB5/GJB3	32	geneontol	0.047548
GO:00083	regulation	16/673	150/18670	0.000105	0.001939	0.001488	PALMD/AI	16	geneontol	0.023774
GO:00614	reproducti	32/673	434/18670	0.000111	0.002033	0.00156	GJB5/GJB3	32	geneontol	0.047548

GO:003103	actomyosi	19/673	199/18670	0.000111	0.002033	0.00156	TACSTD2/	19	geneontol	0.028232
GO:000228	T cell activ	13/673	106/18670	0.000113	0.002057	0.001578	CD1C/LY9/	13	geneontol	0.019316
GO:005076	positive re	34/673	474/18670	0.000117	0.002118	0.001625	NEGR1/NC	34	geneontol	0.05052
GO:000170	formation	14/673	121/18670	0.000118	0.002129	0.001634	EPHA2/TA	14	geneontol	0.020802
GO:005086	regulation	18/673	184/18670	0.000123	0.002211	0.001697	FCRL3/MN	18	geneontol	0.026746
GO:003469	response t	6/673	23/18670	0.000128	0.002289	0.001756	TGFBR3/PI	6	geneontol	0.008915
GO:006044	branching	6/673	23/18670	0.000128	0.002289	0.001756	FGF10/HG	6	geneontol	0.008915
GO:001086	positive re	8/673	43/18670	0.00013	0.002309	0.001771	SELE/P2RY	8	geneontol	0.011887
GO:000736	gastrulatio	18/673	185/18670	0.000132	0.002333	0.00179	EPHA2/TA	18	geneontol	0.026746
GO:004358	skin develo	31/673	419/18670	0.000133	0.002345	0.0018	EPHA2/SFI	31	geneontol	0.046062
GO:200102	positive re	5/673	15/18670	0.000133	0.002345	0.0018	KDR/SMO	5	geneontol	0.007429
GO:004210	positive re	10/673	67/18670	0.000134	0.002346	0.0018	PTPRC/CD	10	geneontol	0.014859
GO:190339	regulation	10/673	67/18670	0.000134	0.002346	0.0018	S100A10/F	10	geneontol	0.014859
GO:004303	regulation	9/673	55/18670	0.00014	0.002436	0.001869	PTPRC/IL1	9	geneontol	0.013373
GO:004208	cytokine b	14/673	123/18670	0.000141	0.002436	0.001869	PTPRC/IL1	14	geneontol	0.020802
GO:200102	negative re	7/673	33/18670	0.000144	0.002486	0.001908	IL10/OSR1	7	geneontol	0.010401
GO:003027	regulation	19/673	203/18670	0.000144	0.002486	0.001908	S1PR1/DD	19	geneontol	0.028232
GO:000704	cell-substr	11/673	81/18670	0.00015	0.002569	0.001971	S100A10/F	11	geneontol	0.016345
GO:004804	focal adhe	11/673	81/18670	0.00015	0.002569	0.001971	S100A10/F	11	geneontol	0.016345
GO:004210	cytokine m	14/673	124/18670	0.000153	0.002604	0.001998	PTPRC/IL1	14	geneontol	0.020802
GO:003808	vascular er	8/673	44/18670	0.000154	0.002604	0.001998	XDH/PDGI	8	geneontol	0.011887
GO:004211	macropha	12/673	95/18670	0.000155	0.002612	0.002004	PTPRC/IL1	12	geneontol	0.017831
GO:000238	immunogl	9/673	56/18670	0.000162	0.002725	0.002091	PTPRC/IL1	9	geneontol	0.013373
GO:003157	membrane	6/673	24/18670	0.000166	0.002777	0.002131	CD2/S100/	6	geneontol	0.008915
GO:000338	epithelial c	7/673	34/18670	0.000176	0.002935	0.002252	RAB25/IHH	7	geneontol	0.010401
GO:000743	salivary gla	7/673	34/18670	0.000176	0.002935	0.002252	FGF10/HG	7	geneontol	0.010401
GO:004887	multicellula	34/673	485/18670	0.00018	0.00299	0.002294	SFN/LEPR/	34	geneontol	0.05052
GO:190027	regulation	8/673	45/18670	0.000181	0.002991	0.002295	SELE/P2RY	8	geneontol	0.011887
GO:007026	cornificatic	13/673	112/18670	0.000198	0.00326	0.002501	LIPM/PKP3	13	geneontol	0.019316
GO:190290	regulation	27/673	352/18670	0.000199	0.003268	0.002507	TACSTD2/	27	geneontol	0.040119
GO:000269	negative re	17/673	175/18670	0.000204	0.003341	0.002563	PLA2G2D/	17	geneontol	0.02526
GO:007190	positive re	26/673	334/18670	0.000206	0.003358	0.002577	NGF/S100/	26	geneontol	0.038633
GO:000271	regulation	11/673	84/18670	0.000209	0.003382	0.002595	IL10/NLRP	11	geneontol	0.016345
GO:005149	regulation	11/673	84/18670	0.000209	0.003382	0.002595	TACSTD2/	11	geneontol	0.016345
GO:003106	hair follicle	6/673	25/18670	0.000211	0.003396	0.002605	FGF10/FOX	6	geneontol	0.008915
GO:005092	positive re	6/673	25/18670	0.000211	0.003396	0.002605	S1PR1/CCI	6	geneontol	0.008915
GO:007167	regulation	8/673	46/18670	0.000212	0.003397	0.002606	S100A14/C	8	geneontol	0.011887
GO:000270	negative re	7/673	35/18670	0.000214	0.003397	0.002606	FCRL3/IL10	7	geneontol	0.010401
GO:004833	mesoderm	7/673	35/18670	0.000214	0.003397	0.002606	TAL1/TRIM	7	geneontol	0.010401
GO:005105	positive re	10/673	71/18670	0.00022	0.003484	0.002673	NGF/GPR3	10	geneontol	0.014859
GO:000237	immunogl	18/673	193/18670	0.000224	0.003537	0.002713	FCRL3/PTF	18	geneontol	0.026746
GO:003010	regulation	23/673	281/18670	0.00023	0.003617	0.002775	TSPAN1/S	23	geneontol	0.034175
GO:004203	regulation	13/673	114/18670	0.000236	0.003708	0.002845	PTPRC/IL1	13	geneontol	0.019316
GO:004340	regulation	26/673	337/18670	0.000237	0.003708	0.002845	NGF/S100/	26	geneontol	0.038633
GO:001051	positive re	9/673	59/18670	0.000244	0.003802	0.002917	SELE/AGTI	9	geneontol	0.013373
GO:006019	positive re	10/673	72/18670	0.000247	0.003835	0.002943	SELE/AGTI	10	geneontol	0.014859
GO:004663	positive re	8/673	47/18670	0.000248	0.003835	0.002943	NLRP3/ZA	8	geneontol	0.011887
GO:001076	regulation	24/673	301/18670	0.000251	0.003867	0.002967	TACSTD2/	24	geneontol	0.035661
GO:000282	positive re	12/673	100/18670	0.000252	0.003867	0.002967	CD1C/CD1	12	geneontol	0.017831
GO:002260	digestive s	12/673	100/18670	0.000252	0.003867	0.002967	PTGER3/M	12	geneontol	0.017831
GO:003261	interleukin	13/673	115/18670	0.000258	0.003938	0.003021	MNDA/PY	13	geneontol	0.019316
GO:190397	regulation	5/673	17/18670	0.000259	0.003938	0.003021	CX3CR1/C	5	geneontol	0.007429
GO:190370	regulation	33/673	475/18670	0.000263	0.003992	0.003063	TAL1/CD2/	33	geneontol	0.049034
GO:005092	regulation	6/673	26/18670	0.000266	0.004029	0.003091	S1PR1/CCI	6	geneontol	0.008915
GO:004877	tissue rem	17/673	179/18670	0.000267	0.004033	0.003094	EPHA2/LEI	17	geneontol	0.02526
GO:003261	interleukin	12/673	101/18670	0.000277	0.004165	0.003196	MNDA/PY	12	geneontol	0.017831
GO:006056	epithelial t	25/673	322/18670	0.000282	0.004226	0.003242	EPHA2/TA	25	geneontol	0.037147
GO:000189	embryonic	11/673	87/18670	0.000286	0.004268	0.003275	GJB5/IL10/	11	geneontol	0.016345
GO:000176	establishm	4/673	10/18670	0.000295	0.004341	0.00333	DOCK2/CC	4	geneontol	0.005944
GO:001404	regulation	4/673	10/18670	0.000295	0.004341	0.00333	LRRK2/EDI	4	geneontol	0.005944
GO:007220	cell prolife	4/673	10/18670	0.000295	0.004341	0.00333	OSR1/SHH	4	geneontol	0.005944
GO:009704	dendritic c	4/673	10/18670	0.000295	0.004341	0.00333	CCL19/CCI	4	geneontol	0.005944
GO:200066	regulation	4/673	10/18670	0.000295	0.004341	0.00333	CCL19/CCI	4	geneontol	0.005944
GO:004255	myelinatio	14/673	132/18670	0.000296	0.004341	0.003331	MAL/HGF/	14	geneontol	0.020802
GO:000803	cell recogr	19/673	215/18670	0.000303	0.004402	0.003377	NEXN/IGSI	19	geneontol	0.028232
GO:001097	regulation	34/673	499/18670	0.000305	0.004402	0.003377	NEGR1/NC	34	geneontol	0.05052
GO:000743	salivary gla	7/673	37/18670	0.000307	0.004402	0.003377	FGF10/HG	7	geneontol	0.010401
GO:003808	peptidyl-ty	7/673	37/18670	0.000307	0.004402	0.003377	KDR/ITK/B	7	geneontol	0.010401
GO:004311	regulation	7/673	37/18670	0.000307	0.004402	0.003377	NPR1/CXC	7	geneontol	0.010401
GO:200024	regulation	7/673	37/18670	0.000307	0.004402	0.003377	PDGFRA/S	7	geneontol	0.010401
GO:200040	positive re	7/673	37/18670	0.000307	0.004402	0.003377	ITGA4/CCI	7	geneontol	0.010401
GO:000110	response t	26/673	343/18670	0.000312	0.004456	0.003419	GJB3/PTGI	26	geneontol	0.038633
GO:190188	regulation	11/673	88/18670	0.000316	0.004504	0.003456	EPHA2/S10	11	geneontol	0.016345

GO:00508	negative re	18/673	199/18670	0.000326	0.004632	0.003554	PLA2G2D/	18	geneontol	0.026746
GO:00430	positive re	6/673	27/18670	0.000332	0.004701	0.003607	IL10/STAP	6	geneontol	0.008915
GO:00071	negative re	23/673	289/18670	0.000344	0.004847	0.003719	PLA2G2D/	23	geneontol	0.034175
GO:00072	ensheathm	14/673	134/18670	0.000346	0.004847	0.003719	MAL/HGF/	14	geneontol	0.020802
GO:00083	axon ensh	14/673	134/18670	0.000346	0.004847	0.003719	MAL/HGF/	14	geneontol	0.020802
GO:00485	embryonic	5/673	18/18670	0.000347	0.004847	0.003719	IHH/PDGF	5	geneontol	0.007429
GO:00507	negative re	15/673	150/18670	0.000348	0.004847	0.003719	CD84/PTP	15	geneontol	0.022288
GO:00098	embryonic	9/673	62/18670	0.000358	0.004974	0.003816	IHH/FGF1	9	geneontol	0.013373
GO:00482	macropha	7/673	38/18670	0.000365	0.005057	0.00388	STAP1/CM	7	geneontol	0.010401
GO:00487	mesenchy	19/673	219/18670	0.000382	0.005278	0.00405	TGFBR3/E	19	geneontol	0.028232
GO:00343	adherens j	11/673	90/18670	0.000385	0.005307	0.004072	S100A10/	11	geneontol	0.016345
GO:00027	positive re	12/673	105/18670	0.000398	0.005453	0.004184	CD1C/CD1	12	geneontol	0.017831
GO:00028	positive re	12/673	105/18670	0.000398	0.005453	0.004184	CD1C/CD1	12	geneontol	0.017831
GO:00026	regulation	9/673	63/18670	0.000404	0.005488	0.004211	FCRL3/PTF	9	geneontol	0.013373
GO:00466	regulation	9/673	63/18670	0.000404	0.005488	0.004211	NLRP3/ZA	9	geneontol	0.013373
GO:20004	regulation	9/673	63/18670	0.000404	0.005488	0.004211	ITGA4/CC	9	geneontol	0.013373
GO:00017	myeloid de	6/673	28/18670	0.00041	0.005516	0.004232	CD2/SLAM	6	geneontol	0.008915
GO:00351	embryonic	6/673	28/18670	0.00041	0.005516	0.004232	OSR1/AFF	6	geneontol	0.008915
GO:00455	regulation	6/673	28/18670	0.00041	0.005516	0.004232	FCRL3/SFR	6	geneontol	0.008915
GO:00485	embryonic	30/673	428/18670	0.000426	0.005713	0.004383	EPHA2/GJ	30	geneontol	0.044577
GO:19055	regulation	7/673	39/18670	0.000431	0.005767	0.004425	CX3CR1/C	7	geneontol	0.010401
GO:00352	organ grov	18/673	204/18670	0.00044	0.005862	0.004498	LEPR/TGFE	18	geneontol	0.026746
GO:00019	positive re	8/673	51/18670	0.000442	0.005862	0.004498	S100A10/K	8	geneontol	0.011887
GO:00725	purine-cor	8/673	51/18670	0.000442	0.005862	0.004498	PDE4B/XD	8	geneontol	0.011887
GO:00106	epithelial c	26/673	351/18670	0.000444	0.005868	0.004503	EPHA2/TA	26	geneontol	0.038633
GO:00017	establishm	4/673	11/18670	0.000451	0.00593	0.00455	DOCK2/C	4	geneontol	0.005944
GO:00330	positive re	4/673	11/18670	0.000451	0.00593	0.00455	IHH/IL7R/	4	geneontol	0.005944
GO:00025	chronic inf	5/673	19/18670	0.000458	0.005968	0.004579	IL10/CXCL	5	geneontol	0.007429
GO:00026	negative re	5/673	19/18670	0.000458	0.005968	0.004579	SLIT2/STA	5	geneontol	0.007429
GO:00466	positive re	5/673	19/18670	0.000458	0.005968	0.004579	PTPRC/ZA	5	geneontol	0.007429
GO:19048	positive re	11/673	92/18670	0.000466	0.006065	0.004653	TNFSF18/	11	geneontol	0.016345
GO:00019	leukocyte	12/673	107/18670	0.000474	0.006143	0.004713	CD1C/CD1	12	geneontol	0.017831
GO:00019	regulation	10/673	78/18670	0.000478	0.00618	0.004741	CD1C/CD1	10	geneontol	0.014859
GO:00604	mesenchy	22/673	278/18670	0.000495	0.006384	0.004898	TGFBR3/E	22	geneontol	0.032689
GO:00487	epidermis	6/673	29/18670	0.000501	0.006409	0.004918	FGF10/FO	6	geneontol	0.008915
GO:00508	regulation	6/673	29/18670	0.000501	0.006409	0.004918	FCRL3/STA	6	geneontol	0.008915
GO:19000	regulation	6/673	29/18670	0.000501	0.006409	0.004918	TACSTD2/	6	geneontol	0.008915
GO:00075	digestion	14/673	139/18670	0.000504	0.006416	0.004923	PTGER3/C	14	geneontol	0.020802
GO:00901	epithelium	26/673	354/18670	0.000505	0.006416	0.004923	EPHA2/TA	26	geneontol	0.038633
GO:00091	cyclic nucl	7/673	40/18670	0.000506	0.006423	0.004928	EPHA2/PD	7	geneontol	0.010401
GO:00327	positive re	9/673	65/18670	0.000512	0.006479	0.004971	PDE4B/CD	9	geneontol	0.013373
GO:00068	calcium io	30/673	434/18670	0.000536	0.006738	0.00517	PDE4B/FC	30	geneontol	0.044577
GO:00487	gland deve	30/673	434/18670	0.000536	0.006738	0.00517	EPHA2/TG	30	geneontol	0.044577
GO:00609	kidney mo	11/673	94/18670	0.000562	0.007044	0.005404	TACSTD2/	11	geneontol	0.016345
GO:00514	positive re	19/673	226/18670	0.000564	0.007058	0.005415	S100A10/	19	geneontol	0.028232
GO:00019	endothelia	17/673	191/18670	0.000569	0.007101	0.005448	EPHA2/IL1	17	geneontol	0.02526
GO:00300	myeloid ce	29/673	416/18670	0.000576	0.007166	0.005498	EPHA2/TA	29	geneontol	0.043091
GO:00702	regulation	8/673	53/18670	0.000578	0.007167	0.005499	EFNA1/IL1	8	geneontol	0.011887
GO:00068	xenobiotic	13/673	125/18670	0.000582	0.007207	0.005529	GSTM5/S1	13	geneontol	0.019316
GO:01500	regulation	7/673	41/18670	0.000592	0.007253	0.005565	PTPRC/CD	7	geneontol	0.010401
GO:00025	regulation	5/673	20/18670	0.000592	0.007253	0.005565	FGL2/CCL	5	geneontol	0.007429
GO:00336	positive re	5/673	20/18670	0.000592	0.007253	0.005565	P2RY12/C	5	geneontol	0.007429
GO:00433	CD4-posit	5/673	20/18670	0.000592	0.007253	0.005565	LY9/IRF4/	5	geneontol	0.007429
GO:00017	B cell hom	6/673	30/18670	0.000608	0.007402	0.005679	DOCK10/	6	geneontol	0.008915
GO:00107	regulation	6/673	30/18670	0.000608	0.007402	0.005679	PPARG/AG	6	geneontol	0.008915
GO:01100	regulation	11/673	95/18670	0.000615	0.007469	0.005731	TACSTD2/	11	geneontol	0.016345
GO:00901	tissue mig	26/673	360/18670	0.000648	0.007841	0.006016	EPHA2/TA	26	geneontol	0.038633
GO:00019	regulation	16/673	176/18670	0.00065	0.007841	0.006016	IL10/XDH/	16	geneontol	0.023774
GO:00326	regulation	8/673	54/18670	0.000657	0.007841	0.006016	PDE4B/PT	8	geneontol	0.011887
GO:00028	positive re	4/673	12/18670	0.000657	0.007841	0.006016	CD28/CNF	4	geneontol	0.005944
GO:00360	response t	4/673	12/18670	0.000657	0.007841	0.006016	MST1R/ST	4	geneontol	0.005944
GO:00360	cellular res	4/673	12/18670	0.000657	0.007841	0.006016	MST1R/ST	4	geneontol	0.005944
GO:00426	regulation	4/673	12/18670	0.000657	0.007841	0.006016	FGFR1/SO	4	geneontol	0.005944
GO:19040	epithelial c	12/673	111/18670	0.000662	0.007874	0.006041	IL10/ITGA	12	geneontol	0.017831
GO:19010	regulation	7/673	42/18670	0.000689	0.008173	0.006271	IL10/CD36	7	geneontol	0.010401
GO:00423	molting cy	12/673	112/18670	0.000717	0.008414	0.006456	EPS8L3/FG	12	geneontol	0.017831
GO:00426	hair cycle	12/673	112/18670	0.000717	0.008414	0.006456	EPS8L3/FG	12	geneontol	0.017831
GO:00022	T cell diffe	9/673	68/18670	0.000718	0.008414	0.006456	LY9/NLRP	9	geneontol	0.013373
GO:00359	cellular res	9/673	68/18670	0.000718	0.008414	0.006456	XDH/PDG	9	geneontol	0.013373
GO:19021	positive re	14/673	144/18670	0.000718	0.008414	0.006456	PTPRC/NL	14	geneontol	0.020802
GO:00072	activation	6/673	31/18670	0.000731	0.008542	0.006554	SELE/P2RY	6	geneontol	0.008915
GO:00016	ureteric bu	11/673	97/18670	0.000734	0.008559	0.006567	TACSTD2/	11	geneontol	0.016345
GO:00482	eosinophil	5/673	21/18670	0.000754	0.008753	0.006716	LGALS3/C	5	geneontol	0.007429

GO:00519(	regulation	5/673	21/18670	0.000754	0.008753	0.006716	KDR/LRRK:	5	geneontol	0.007429
GO:0043€	keratinocy	7/673	43/18670	0.000797	0.009172	0.007037	SFN/FGF1(	7	geneontol	0.010401
GO:00508	regulation	11/673	98/18670	0.000801	0.009172	0.007037	PLEK/ZAP7	11	geneontol	0.016345
GO:00601	regulation	11/673	98/18670	0.000801	0.009172	0.007037	SELE/AGTI	11	geneontol	0.016345
GO:00721(	mesoneph	11/673	98/18670	0.000801	0.009172	0.007037	TACSTD2/	11	geneontol	0.016345
GO:00721(	mesoneph	11/673	98/18670	0.000801	0.009172	0.007037	TACSTD2/	11	geneontol	0.016345
GO:00458	positive re	14/673	146/18670	0.000823	0.009408	0.007218	SLC45A3/I	14	geneontol	0.020802
GO:00312	biomineral	15/673	163/18670	0.000836	0.009529	0.007311	S1PR1/DD	15	geneontol	0.022288
GO:00019	positive re	8/673	56/18670	0.000842	0.009553	0.007329	CD1C/CD1	8	geneontol	0.011887
GO:00606	regulation	8/673	56/18670	0.000842	0.009553	0.007329	TACSTD2/	8	geneontol	0.011887
GO:00091(	glycoprote	25/673	348/18670	0.000881	0.009976	0.007654	MAN1C1/I	25	geneontol	0.037147
GO:00027(	regulation	9/673	70/18670	0.000889	0.009984	0.00766	CD1C/CD1	9	geneontol	0.013373
GO:00105	regulation	9/673	70/18670	0.000889	0.009984	0.00766	SELE/AGTI	9	geneontol	0.013373
GO:00507(	positive re	9/673	70/18670	0.000889	0.009984	0.00766	PTPRC/PP/	9	geneontol	0.013373
GO:00061	purine nuc	7/673	44/18670	0.000919	0.010142	0.007781	PDE4B/XD	7	geneontol	0.010401
GO:00104(	mesenchy	7/673	44/18670	0.000919	0.010142	0.007781	OSR1/IHH,	7	geneontol	0.010401
GO:00316	regulation	4/673	13/18670	0.000922	0.010142	0.007781	PTGER3/N	4	geneontol	0.005944
GO:00336	regulation	4/673	13/18670	0.000922	0.010142	0.007781	SELP/PLEK	4	geneontol	0.005944
GO:00431	negative re	4/673	13/18670	0.000922	0.010142	0.007781	SLIT2/ANG	4	geneontol	0.005944
GO:00450	positive re	4/673	13/18670	0.000922	0.010142	0.007781	PTPRC/CD	4	geneontol	0.005944
GO:00601(	positive re	4/673	13/18670	0.000922	0.010142	0.007781	PPARG/ST.	4	geneontol	0.005944
GO:00603	heart trab	4/673	13/18670	0.000922	0.010142	0.007781	TGFBR3/TI	4	geneontol	0.005944
GO:19051	positive re	4/673	13/18670	0.000922	0.010142	0.007781	PPARG/ST.	4	geneontol	0.005944
GO:01200	positive re	11/673	100/18670	0.000948	0.010386	0.007968	EPS8L3/P2	11	geneontol	0.016345
GO:00017	neural cre	8/673	57/18670	0.000949	0.010386	0.007968	ZEB2/SEM	8	geneontol	0.011887
GO:00976	STAT casca	15/673	166/18670	0.001008	0.011002	0.008441	TNFSF18/I	15	geneontol	0.022288
GO:00108	regulation	6/673	33/18670	0.001034	0.01121	0.008601	SFN/FGF1(	6	geneontol	0.008915
GO:00199	cGMP-me	6/673	33/18670	0.001034	0.01121	0.008601	NPR1/CD3	6	geneontol	0.008915
GO:00466	alpha-bet	6/673	33/18670	0.001034	0.01121	0.008601	PTPRC/ZA	6	geneontol	0.008915
GO:00327(	positive re	10/673	86/18670	0.00104	0.01122	0.008609	CD2/PTPR	10	geneontol	0.014859
GO:00705	response t	10/673	86/18670	0.00104	0.01122	0.008609	PTGFR/TG	10	geneontol	0.014859
GO:00025	acute infla	18/673	220/18670	0.001067	0.011483	0.008811	PTGER3/C	18	geneontol	0.026746
GO:00487	branching	14/673	150/18670	0.001073	0.01152	0.008839	EPHA2/TA	14	geneontol	0.020802
GO:00510	regulation	26/673	373/18670	0.001088	0.01166	0.008946	TGFBR3/N	26	geneontol	0.038633
GO:00023(	cytokine p	11/673	102/18670	0.001118	0.011956	0.009173	IL10/NLRP	11	geneontol	0.016345
GO:00360	CD8-posit	5/673	23/18670	0.001175	0.012393	0.009509	EOMES/TN	5	geneontol	0.007429
GO:00518	mitochond	5/673	23/18670	0.001175	0.012393	0.009509	KDR/LRRK:	5	geneontol	0.007429
GO:00614	establishm	5/673	23/18670	0.001175	0.012393	0.009509	SFN/CLDN	5	geneontol	0.007429
GO:19010	negative re	5/673	23/18670	0.001175	0.012393	0.009509	IL10/HGF/	5	geneontol	0.007429
GO:19032(	negative re	5/673	23/18670	0.001175	0.012393	0.009509	IL10/HGF/	5	geneontol	0.007429
GO:20010	negative re	5/673	23/18670	0.001175	0.012393	0.009509	IL10/HGF/	5	geneontol	0.007429
GO:00025	myeloid le	17/673	204/18670	0.001189	0.012448	0.00955	EPHA2/TA	17	geneontol	0.02526
GO:00457(	positive re	17/673	204/18670	0.001189	0.012448	0.00955	IL10/CYP1	17	geneontol	0.02526
GO:19029(	positive re	17/673	204/18670	0.001189	0.012448	0.00955	S100A10/I	17	geneontol	0.02526
GO:00099	cell-cell re	9/673	73/18670	0.001206	0.012548	0.009627	DOCK2/C(	9	geneontol	0.013373
GO:00024	inflammat	7/673	46/18670	0.001206	0.012548	0.009627	IL10/CD28	7	geneontol	0.010401
GO:00226(	ovulation	7/673	46/18670	0.001206	0.012548	0.009627	PTX3/SLIT	7	geneontol	0.010401
GO:00326	regulation	11/673	103/18670	0.001212	0.012582	0.009653	MNDA/PY	11	geneontol	0.016345
GO:00607	cell fate cc	6/673	34/18670	0.001218	0.012601	0.009668	EOMES/TF	6	geneontol	0.008915
GO:00018	placenta d	14/673	152/18670	0.001219	0.012601	0.009668	GJB5/GJB3	14	geneontol	0.020802
GO:00610	regulation	10/673	88/18670	0.001243	0.012759	0.009789	TAL1/EFN/	10	geneontol	0.014859
GO:19035	positive re	10/673	88/18670	0.001243	0.012759	0.009789	CD2/PTPR	10	geneontol	0.014859
GO:19037	regulation	10/673	88/18670	0.001243	0.012759	0.009789	P2RY12/PI	10	geneontol	0.014859
GO:00712	cellular res	17/673	205/18670	0.001254	0.012793	0.009815	PDE4B/IL1	17	geneontol	0.02526
GO:00092	cyclic nucl	4/673	14/18670	0.001255	0.012793	0.009815	PDE4B/PD	4	geneontol	0.005944
GO:00508(	positive re	4/673	14/18670	0.001255	0.012793	0.009815	TRAT1/TES	4	geneontol	0.005944
GO:00099	epidermal	25/673	358/18670	0.001312	0.01332	0.01022	EPHA2/SFI	25	geneontol	0.037147
GO:00015	retinoid m	11/673	104/18670	0.001313	0.01332	0.01022	CRABP2/C	11	geneontol	0.016345
GO:00313	negative re	16/673	188/18670	0.001315	0.01332	0.01022	TACSTD2/	16	geneontol	0.023774
GO:00433(	CD4-posit	9/673	74/18670	0.00133	0.013438	0.01031	PLA2G2D/	9	geneontol	0.013373
GO:00464	positive re	10/673	89/18670	0.001356	0.013678	0.010494	TNFSF18/I	10	geneontol	0.014859
GO:00109	positive re	21/673	281/18670	0.001361	0.013696	0.010508	NEGR1/N(	21	geneontol	0.031204
GO:00028(	regulation	5/673	24/18670	0.001441	0.014376	0.01103	IL10/CD28	5	geneontol	0.007429
GO:00456(	positive re	5/673	24/18670	0.001441	0.014376	0.01103	SFN/SFRP	5	geneontol	0.007429
GO:00603	trabecula f	5/673	24/18670	0.001441	0.014376	0.01103	TGFBR3/SI	5	geneontol	0.007429
GO:00716	positive re	5/673	24/18670	0.001441	0.014376	0.01103	S100A14/(	5	geneontol	0.007429
GO:01500	neuroinfla	9/673	75/18670	0.001464	0.01457	0.011179	PTPRC/CD	9	geneontol	0.013373
GO:00326	regulation	10/673	90/18670	0.001478	0.014647	0.011237	MNDA/PY	10	geneontol	0.014859
GO:00457	positive re	10/673	90/18670	0.001478	0.014647	0.011237	DDR2/OSI	10	geneontol	0.014859
GO:00322	positive re	8/673	61/18670	0.001491	0.014749	0.011316	S100A10/I	8	geneontol	0.011887
GO:00024	T cell med	11/673	106/18670	0.001534	0.015137	0.011613	CD1C/CD1	11	geneontol	0.016345
GO:00081	actin poly	17/673	209/18670	0.001546	0.015227	0.011683	PLEK/ADD	17	geneontol	0.02526
GO:00017	microglial	7/673	48/18670	0.001559	0.015229	0.011685	PTPRC/ST/	7	geneontol	0.010401

GO:00022(	leukocyte a	7/673	48/18670	0.001559	0.015229	0.011685	PTPRC/STA	7	geneontol	0.010401
GO:00027	positive re	7/673	48/18670	0.001559	0.015229	0.011685	CD1C/CD1	7	geneontol	0.010401
GO:00352	exocrine sy	7/673	48/18670	0.001559	0.015229	0.011685	FGF10/HG	7	geneontol	0.010401
GO:00072	JAK-STAT	14/673	156/18670	0.001564	0.015242	0.011694	TNFSF18/F	14	geneontol	0.020802
GO:00305(	regulation	9/673	76/18670	0.001608	0.01564	0.012	S1PR1/DD	9	geneontol	0.013373
GO:00107	negative re	24/673	344/18670	0.001644	0.015834	0.012149	TACSTD2/	24	geneontol	0.035661
GO:00022(	lymphocyt	8/673	62/18670	0.001659	0.015834	0.012149	DOCK10/G	8	geneontol	0.011887
GO:00326	interleukin	8/673	62/18670	0.001659	0.015834	0.012149	PDE4B/PTI	8	geneontol	0.011887
GO:00107	macropha	6/673	36/18670	0.001659	0.015834	0.012149	PPARG/AG	6	geneontol	0.008915
GO:00327	positive re	6/673	36/18670	0.001659	0.015834	0.012149	CD28/TIGI	6	geneontol	0.008915
GO:00351	hindlimb n	6/673	36/18670	0.001659	0.015834	0.012149	OSR1/AFF3	6	geneontol	0.008915
GO:00900	foam cell c	6/673	36/18670	0.001659	0.015834	0.012149	PPARG/AG	6	geneontol	0.008915
GO:00017	endoderm	4/673	15/18670	0.001662	0.015834	0.012149	EOMES/PC	4	geneontol	0.005944
GO:00600	regulation	4/673	15/18670	0.001662	0.015834	0.012149	PPARG/ST	4	geneontol	0.005944
GO:19000	positive re	4/673	15/18670	0.001662	0.015834	0.012149	EPS8L3/P2	4	geneontol	0.005944
GO:00321(	negative re	25/673	365/18670	0.001711	0.016263	0.012478	PTPRC/IL1	25	geneontol	0.037147
GO:00023	mature B c	5/673	25/18670	0.001749	0.016473	0.012639	DOCK10/L	5	geneontol	0.007429
GO:00720	glomerulus	5/673	25/18670	0.001749	0.016473	0.012639	OSR1/PDG	5	geneontol	0.007429
GO:00726	eosinophil	5/673	25/18670	0.001749	0.016473	0.012639	LGALS3/CC	5	geneontol	0.007429
GO:00900	regulation	5/673	25/18670	0.001749	0.016473	0.012639	S100A14/C	5	geneontol	0.007429
GO:00140	regulation	12/673	124/18670	0.001751	0.016473	0.012639	SLC45A3/F	12	geneontol	0.017831
GO:00027(	negative re	7/673	49/18670	0.001764	0.016527	0.012681	CD84/PTPI	7	geneontol	0.010401
GO:00083	glial cell m	7/673	49/18670	0.001764	0.016527	0.012681	CX3CR1/C	7	geneontol	0.010401
GO:00712	cellular res	17/673	212/18670	0.001801	0.016843	0.012923	PDE4B/IL1	17	geneontol	0.02526
GO:00026	regulation	14/673	159/18670	0.001873	0.017393	0.013344	PTGER3/C	14	geneontol	0.020802
GO:00605	muscle tiss	27/673	408/18670	0.001874	0.017393	0.013344	TGFBR3/S1	27	geneontol	0.040119
GO:00303	embryonic	12/673	125/18670	0.001875	0.017393	0.013344	CRABP2/C	12	geneontol	0.017831
GO:00351	embryonic	12/673	125/18670	0.001875	0.017393	0.013344	CRABP2/C	12	geneontol	0.017831
GO:00343	adherens j	13/673	142/18670	0.001899	0.017577	0.013486	S100A10/C	13	geneontol	0.019316
GO:00488	axon exter	6/673	37/18670	0.001921	0.017648	0.01354	SEMA3G/S	6	geneontol	0.008915
GO:00603	head morp	6/673	37/18670	0.001921	0.017648	0.01354	IHH/PDGF	6	geneontol	0.008915
GO:19022	neuron pro	6/673	37/18670	0.001921	0.017648	0.01354	SEMA3G/S	6	geneontol	0.008915
GO:00064	protein O-	11/673	109/18670	0.001921	0.017648	0.01354	MUC1/B3C	11	geneontol	0.016345
GO:00101	body morp	7/673	50/18670	0.001988	0.018225	0.013983	IHH/PDGF	7	geneontol	0.010401
GO:00343	cell junctio	21/673	290/18670	0.002002	0.018316	0.014053	EPHA2/S1	21	geneontol	0.031204
GO:00973(	response t	18/673	233/18670	0.002029	0.018525	0.014213	PTGFR/TG	18	geneontol	0.026746
GO:00701(	regulation	10/673	94/18670	0.002054	0.018715	0.014359	S1PR1/DD	10	geneontol	0.014859
GO:00450	regulation	29/673	452/18670	0.002058	0.018715	0.014359	S100A14/N	29	geneontol	0.043091
GO:00161(	diterpenoi	11/673	110/18670	0.002067	0.018763	0.014396	CRABP2/C	11	geneontol	0.016345
GO:00106	regulation	21/673	291/18670	0.002087	0.018888	0.014492	EPHA2/TA	21	geneontol	0.031204
GO:00023(	T cell linea	5/673	26/18670	0.002101	0.018888	0.014492	LY9/IRF4/S	5	geneontol	0.007429
GO:00335(	regulation	5/673	26/18670	0.002101	0.018888	0.014492	SFN/CLDN	5	geneontol	0.007429
GO:00514	negative re	5/673	26/18670	0.002101	0.018888	0.014492	TACSTD2/	5	geneontol	0.007429
GO:00607(	cell differe	5/673	26/18670	0.002101	0.018888	0.014492	GJB5/EOM	5	geneontol	0.007429
GO:00456(	positive re	25/673	371/18670	0.002132	0.019066	0.014628	NEGR1/NC	25	geneontol	0.037147
GO:00080(	regulation	15/673	179/18670	0.002138	0.019066	0.014628	PLEK/ADD	15	geneontol	0.022288
GO:00487	appendag	15/673	179/18670	0.002138	0.019066	0.014628	CRABP2/C	15	geneontol	0.022288
GO:00601	limb devel	15/673	179/18670	0.002138	0.019066	0.014628	CRABP2/C	15	geneontol	0.022288
GO:19051	regulation	4/673	16/18670	0.002154	0.019174	0.014711	PPARG/ST	4	geneontol	0.005944
GO:00605	respiratory	16/673	198/18670	0.002242	0.019918	0.015282	HSD11B1/	16	geneontol	0.023774
GO:00725	endothelia	8/673	65/18670	0.002254	0.019935	0.015295	IL10/ITGA4	8	geneontol	0.011887
GO:00308	regulation	15/673	180/18670	0.002257	0.019935	0.015295	PLEK/ADD	15	geneontol	0.022288
GO:00714(	cellular res	15/673	180/18670	0.002257	0.019935	0.015295	GSTM5/S1	15	geneontol	0.022288
GO:00433	regulation	17/673	217/18670	0.002305	0.020283	0.015562	TGFBR3/IL	17	geneontol	0.02526
GO:00603	bone deve	17/673	217/18670	0.002305	0.020283	0.015562	TAL1/PTPF	17	geneontol	0.02526
GO:00712	cellular res	18/673	236/18670	0.002333	0.020492	0.015723	PDE4B/IL1	18	geneontol	0.026746
GO:00019	positive re	11/673	112/18670	0.002386	0.020916	0.016048	IL10/ITGA4	11	geneontol	0.016345
GO:00085	female gor	10/673	96/18670	0.002404	0.021038	0.016141	PTX3/SLIT	10	geneontol	0.014859
GO:00721	mesoneph	8/673	66/18670	0.002486	0.021661	0.016619	TACSTD2/	8	geneontol	0.011887
GO:00327	positive re	7/673	52/18670	0.002503	0.021661	0.016619	MNDA/PY	7	geneontol	0.010401
GO:00550	sodium ior	7/673	52/18670	0.002503	0.021661	0.016619	NPR1/SCN	7	geneontol	0.010401
GO:00614	renal syste	5/673	27/18670	0.002504	0.021661	0.016619	OSR1/PDG	5	geneontol	0.007429
GO:00614	kidney vas	5/673	27/18670	0.002504	0.021661	0.016619	OSR1/PDG	5	geneontol	0.007429
GO:19016	regulation	5/673	27/18670	0.002504	0.021661	0.016619	CCR2/CXC	5	geneontol	0.007429
GO:00300	actin filam	15/673	182/18670	0.002511	0.021686	0.016639	ADD2/VILL	15	geneontol	0.022288
GO:00456	positive re	6/673	39/18670	0.002537	0.021868	0.016778	SFN/SFRP4	6	geneontol	0.008915
GO:00488	stem cell c	19/673	257/18670	0.002546	0.021904	0.016806	TAL1/TACS	19	geneontol	0.028232
GO:00107	negative re	10/673	97/18670	0.002596	0.022255	0.017075	TACSTD2/	10	geneontol	0.014859
GO:00303	osteoclast	10/673	97/18670	0.002596	0.022255	0.017075	EPHA2/EFI	10	geneontol	0.014859
GO:00330(	muscle cel	18/673	239/18670	0.002674	0.022883	0.017557	TGFBR3/S1	18	geneontol	0.026746
GO:00091(	glycoprote	27/673	419/18670	0.002718	0.022918	0.017584	MAN1C1/I	27	geneontol	0.040119
GO:00197	secondary	8/673	67/18670	0.002735	0.022918	0.017584	FMO2/FM(	8	geneontol	0.011887
GO:00107	positive re	4/673	17/18670	0.002737	0.022918	0.017584	AGTR1/CD	4	geneontol	0.005944

GO:00312(	pseudopo	4/673	17/18670	0.002737	0.022918	0.017584	RAB25/CC	4	geneontol	0.005944
GO:00316	positive re	4/673	17/18670	0.002737	0.022918	0.017584	HGF/ZNF4	4	geneontol	0.005944
GO:00316	heat gene	4/673	17/18670	0.002737	0.022918	0.017584	PTGER3/N	4	geneontol	0.005944
GO:00429	lipoprotei	4/673	17/18670	0.002737	0.022918	0.017584	PPARG/CD	4	geneontol	0.005944
GO:00448	lipoprotei	4/673	17/18670	0.002737	0.022918	0.017584	PPARG/CD	4	geneontol	0.005944
GO:00460(	cGMP met	4/673	17/18670	0.002737	0.022918	0.017584	NPR1/PDE	4	geneontol	0.005944
GO:00901	negative re	4/673	17/18670	0.002737	0.022918	0.017584	TACSTD2/	4	geneontol	0.005944
GO:00351(	appendag	13/673	148/18670	0.002738	0.022918	0.017584	CRABP2/C	13	geneontol	0.019316
GO:00351(	limb morp	13/673	148/18670	0.002738	0.022918	0.017584	CRABP2/C	13	geneontol	0.019316
GO:00603	bone mor	11/673	114/18670	0.002743	0.022918	0.017598	VIT/IHH/T	11	geneontol	0.016345
GO:00507(	interleukin	7/673	53/18670	0.002796	0.02323	0.017823	NLRP3/CD	7	geneontol	0.010401
GO:00506(	cytokine s	18/673	240/18670	0.002797	0.02323	0.017823	CD2/S100	18	geneontol	0.026746
GO:00313	regulation	10/673	98/18670	0.0028	0.02323	0.017823	CD1C/CD1	10	geneontol	0.014859
GO:00507(	regulation	10/673	98/18670	0.0028	0.02323	0.017823	PTPRC/C4	10	geneontol	0.014859
GO:00708	divalent m	30/673	483/18670	0.002821	0.023358	0.017921	PDE4B/FC	30	geneontol	0.044577
GO:00022	activation	22/673	319/18670	0.002898	0.023955	0.018379	S100A14/	22	geneontol	0.032689
GO:00343	cell junctio	18/673	241/18670	0.002924	0.024127	0.018511	EPHA2/S1	18	geneontol	0.026746
GO:00466	female sex	11/673	115/18670	0.002937	0.024127	0.018511	PTX3/SLIT	11	geneontol	0.016345
GO:19037(	positive re	15/673	185/18670	0.002937	0.024127	0.018511	TAL1/PTP	15	geneontol	0.022288
GO:00026	regulation	5/673	28/18670	0.002959	0.024127	0.018511	IL1R1/ITG	5	geneontol	0.007429
GO:00108	T cell chen	5/673	28/18670	0.002959	0.024127	0.018511	CCR2/CXC	5	geneontol	0.007429
GO:00605	prostate g	5/673	28/18670	0.002959	0.024127	0.018511	FGF10/SH	5	geneontol	0.007429
GO:00850	extracellul	5/673	28/18670	0.002959	0.024127	0.018511	IHH/TNXB	5	geneontol	0.007429
GO:00459	regulation	12/673	132/18670	0.00296	0.024127	0.018511	IL10/OSR1	12	geneontol	0.017831
GO:00313	positive re	8/673	68/18670	0.003004	0.024443	0.018753	CD1C/CD1	8	geneontol	0.011887
GO:00017(	cell fate sp	10/673	99/18670	0.003017	0.024501	0.018798	IHH/EOM	10	geneontol	0.014859
GO:00450	positive re	25/673	381/18670	0.003031	0.024574	0.018854	S100A14/	25	geneontol	0.037147
GO:00071	homophili	14/673	168/18670	0.003115	0.025209	0.019342	NEXN/CAD	14	geneontol	0.020802
GO:00027(	positive re	12/673	133/18670	0.00315	0.025451	0.019527	CD1C/CD1	12	geneontol	0.017831
GO:00971	extrinsic a	17/673	224/18670	0.003203	0.025832	0.019819	NGF/PTPR	17	geneontol	0.02526
GO:19013	negative re	15/673	187/18670	0.003253	0.026185	0.02009	EPHA2/NP	15	geneontol	0.022288
GO:00605	skeletal m	14/673	169/18670	0.003287	0.026197	0.020099	CHRNA1/	14	geneontol	0.020802
GO:00109	negative re	6/673	41/18670	0.00329	0.026197	0.020099	C4BPB/CR	6	geneontol	0.008915
GO:00351	forelimb m	6/673	41/18670	0.00329	0.026197	0.020099	CRABP2/C	6	geneontol	0.008915
GO:00971	ruffle asse	6/673	41/18670	0.00329	0.026197	0.020099	TACSTD2/	6	geneontol	0.008915
GO:19033	negative re	6/673	41/18670	0.00329	0.026197	0.020099	C4BPB/CR	6	geneontol	0.008915
GO:00426	ovulation	8/673	69/18670	0.003294	0.026197	0.020099	PTX3/SLIT	8	geneontol	0.011887
GO:00702	lymphocyt	8/673	69/18670	0.003294	0.026197	0.020099	EFNA1/IL1	8	geneontol	0.011887
GO:00725	divalent in	30/673	489/18670	0.003376	0.026806	0.020567	PDE4B/FC	30	geneontol	0.044577
GO:00900	positive re	4/673	18/18670	0.00342	0.027061	0.020762	S100A14/	4	geneontol	0.005944
GO:01500	negative re	4/673	18/18670	0.00342	0.027061	0.020762	PTPRC/CD	4	geneontol	0.005944
GO:00001	activation	13/673	152/18670	0.003451	0.027257	0.020913	PTPRC/FG	13	geneontol	0.019316
GO:00322	negative re	5/673	29/18670	0.003471	0.027322	0.020963	TACSTD2/	5	geneontol	0.007429
GO:19001	negative re	5/673	29/18670	0.003471	0.027322	0.020963	CLDN18/C	5	geneontol	0.007429
GO:00465	developm	10/673	101/18670	0.00349	0.027365	0.020996	PTX3/SLIT	10	geneontol	0.014859
GO:00140	mesenchy	9/673	85/18670	0.003494	0.027365	0.020996	ZEB2/SEM	9	geneontol	0.013373
GO:00488	stem cell c	9/673	85/18670	0.003494	0.027365	0.020996	PTPRC/ZE	9	geneontol	0.013373
GO:00327	negative re	8/673	70/18670	0.003604	0.028133	0.021585	SLAMF1/IL	8	geneontol	0.011887
GO:00720	regulation	8/673	70/18670	0.003604	0.028133	0.021585	PTPRC/CX	8	geneontol	0.011887
GO:00507(	regulation	29/673	472/18670	0.003832	0.029866	0.022915	SLC2A1/C	29	geneontol	0.043091
GO:00303	lung devel	14/673	172/18670	0.003851	0.029961	0.022987	HSD11B1/	14	geneontol	0.020802
GO:00107	positive re	13/673	154/18670	0.003859	0.029975	0.022998	NGF/S100	13	geneontol	0.019316
GO:00071	establishm	16/673	210/18670	0.004016	0.031086	0.02385	RHOH/FG	16	geneontol	0.023774
GO:00507(	regulation	16/673	210/18670	0.004016	0.031086	0.02385	CD2/IL10/	16	geneontol	0.023774
GO:00217	olfactory b	5/673	30/18670	0.004043	0.031141	0.023893	EOMES/SL	5	geneontol	0.007429
GO:00466	regulation	5/673	30/18670	0.004043	0.031141	0.023893	PTPRC/ZA	5	geneontol	0.007429
GO:19032(	regulation	5/673	30/18670	0.004043	0.031141	0.023893	IL10/HGF/	5	geneontol	0.007429
GO:00069(	phagocyto	24/673	369/18670	0.004056	0.031187	0.023928	LEPR/SLA	24	geneontol	0.035661
GO:00067	terpenoid	11/673	120/18670	0.004079	0.031315	0.024026	CRABP2/C	11	geneontol	0.016345
GO:00147(	striated m	25/673	390/18670	0.004096	0.031394	0.024087	TGFBR3/S	25	geneontol	0.037147
GO:19040	positive re	17/673	230/18670	0.004188	0.031789	0.02439	IL10/CYP1	17	geneontol	0.02526
GO:00325	regulation	24/673	370/18670	0.004195	0.031789	0.02439	NGF/CRAB	24	geneontol	0.035661
GO:00508	positive re	6/673	43/18670	0.004198	0.031789	0.02439	ZAP70/TR	6	geneontol	0.008915
GO:00016	gastric aci	4/673	19/18670	0.00421	0.031789	0.02439	PTGER3/N	4	geneontol	0.005944
GO:00017	mesoderm	4/673	19/18670	0.00421	0.031789	0.02439	TRIM15/F	4	geneontol	0.005944
GO:00023	mature B	4/673	19/18670	0.00421	0.031789	0.02439	DOCK10/L	4	geneontol	0.005944
GO:00023(	alpha-bet	4/673	19/18670	0.00421	0.031789	0.02439	LY9/IRF4/	4	geneontol	0.005944
GO:00450	regulation	4/673	19/18670	0.00421	0.031789	0.02439	PTPRC/CD	4	geneontol	0.005944
GO:20002	positive re	4/673	19/18670	0.00421	0.031789	0.02439	STAP1/BA	4	geneontol	0.005944
GO:19035	negative re	8/673	72/18670	0.004292	0.032359	0.024827	SLAMF1/IL	8	geneontol	0.011887
GO:00507	positive re	12/673	139/18670	0.004508	0.033927	0.026031	CD2/IL10/	12	geneontol	0.017831
GO:00424	hormone	17/673	232/18670	0.004567	0.034319	0.026331	NGF/CRAB	17	geneontol	0.02526
GO:00016(	fever gene	3/673	10/18670	0.004628	0.034326	0.026337	PTGER3/C	3	geneontol	0.004458

GO:00022	T cell activ	3/673	10/18670	0.004628	0.034326	0.026337	FGL2/LGAI	3	geneontol	0.004458
GO:00326	interleukin	3/673	10/18670	0.004628	0.034326	0.026337	CD84/IL10	3	geneontol	0.004458
GO:00430	penile erec	3/673	10/18670	0.004628	0.034326	0.026337	CNR1/AVF	3	geneontol	0.004458
GO:00519	positive re	3/673	10/18670	0.004628	0.034326	0.026337	KDR/DCN/	3	geneontol	0.004458
GO:00611	lung secre	3/673	10/18670	0.004628	0.034326	0.026337	FGF10/SPD	3	geneontol	0.004458
GO:00726	interleukin	3/673	10/18670	0.004628	0.034326	0.026337	CCL19/CC	3	geneontol	0.004458
GO:19043	regulation	3/673	10/18670	0.004628	0.034326	0.026337	SHH/SFRP	3	geneontol	0.004458
GO:00017	in utero er	24/673	373/18670	0.004635	0.034326	0.026337	GJB5/GJB3	24	geneontol	0.035661
GO:00311	animal org	8/673	73/18670	0.004672	0.034334	0.026342	TGFBR3/IL	8	geneontol	0.011887
GO:00619	glial cell ac	7/673	58/18670	0.004674	0.034334	0.026342	PTPRC/ST/	7	geneontol	0.010401
GO:00026	positive re	5/673	31/18670	0.00468	0.034334	0.026342	PTGER3/CI	5	geneontol	0.007429
GO:00091	purine ribc	5/673	31/18670	0.00468	0.034334	0.026342	PDE4B/PD	5	geneontol	0.007429
GO:00219	olfactory lc	5/673	31/18670	0.00468	0.034334	0.026342	EOMES/SL	5	geneontol	0.007429
GO:20004	positive re	5/673	31/18670	0.00468	0.034334	0.026342	ITGA4/CCI	5	geneontol	0.007429
GO:00346	response t	21/673	312/18670	0.004714	0.034482	0.026456	SELE/TNFS	21	geneontol	0.031204
GO:20004	regulation	6/673	44/18670	0.004716	0.034482	0.026456	ITGA4/CCI	6	geneontol	0.008915
GO:00303	respiratory	14/673	176/18670	0.004723	0.034482	0.026456	HSD11B1/	14	geneontol	0.020802
GO:00902	regulation	20/673	292/18670	0.004738	0.034541	0.026502	TGFBR3/XI	20	geneontol	0.029718
GO:00610	somite dev	9/673	89/18670	0.004753	0.034593	0.026541	ZEB2/IHH/	9	geneontol	0.013373
GO:00463	positive re	12/673	140/18670	0.004773	0.034634	0.026573	SLAMF1/Z	12	geneontol	0.017831
GO:00720	kidney epi	12/673	140/18670	0.004773	0.034634	0.026573	TACSTD2/	12	geneontol	0.017831
GO:00072	JNK cascac	16/673	214/18670	0.004816	0.034887	0.026767	SLAMF1/Z	16	geneontol	0.023774
GO:00435	endothelia	19/673	273/18670	0.004915	0.035551	0.027276	EPHA2/EFI	19	geneontol	0.028232
GO:00421	neurotrans	10/673	106/18670	0.004934	0.03563	0.027337	IL10/CYP1	10	geneontol	0.014859
GO:00140	positive re	8/673	74/18670	0.005077	0.036495	0.028001	PPARG/CC	8	geneontol	0.011887
GO:00720	nephron tu	8/673	74/18670	0.005077	0.036495	0.028001	TACSTD2/	8	geneontol	0.011887
GO:00023	lymphoid	4/673	20/18670	0.005114	0.036495	0.028001	SHH/FLT3/	4	geneontol	0.005944
GO:00076	copulation	4/673	20/18670	0.005114	0.036495	0.028001	CNR1/AVF	4	geneontol	0.005944
GO:00105	positive re	4/673	20/18670	0.005114	0.036495	0.028001	CCL19/CC	4	geneontol	0.005944
GO:19039	regulation	4/673	20/18670	0.005114	0.036495	0.028001	PTPRC/ST/	4	geneontol	0.005944
GO:00016	metanephi	9/673	90/18670	0.005117	0.036495	0.028001	OSR1/CXC	9	geneontol	0.013373
GO:00140	neural cres	9/673	90/18670	0.005117	0.036495	0.028001	ZEB2/SEM	9	geneontol	0.013373
GO:00016	branching	7/673	59/18670	0.005142	0.036506	0.028009	TACSTD2/	7	geneontol	0.010401
GO:00327	positive re	7/673	59/18670	0.005142	0.036506	0.028009	MNDA/PY	7	geneontol	0.010401
GO:20003	regulation	7/673	59/18670	0.005142	0.036506	0.028009	ITGA4/KDI	7	geneontol	0.010401
GO:00359	endoderm	6/673	45/18670	0.005279	0.037423	0.028713	LAMB3/IT(	6	geneontol	0.008915
GO:00075	skeletal mu	13/673	160/18670	0.005326	0.037699	0.028925	CHRNA1/E	13	geneontol	0.019316
GO:00720	nephron d	12/673	142/18670	0.005343	0.037758	0.02897	TACSTD2/	12	geneontol	0.017831
GO:00092	ribonuclec	5/673	32/18670	0.005385	0.037886	0.029068	PDE4B/PD	5	geneontol	0.007429
GO:00603	face morpl	5/673	32/18670	0.005385	0.037886	0.029068	PDGFRA/C	5	geneontol	0.007429
GO:00613	heart trabe	5/673	32/18670	0.005385	0.037886	0.029068	TGFBR3/S1	5	geneontol	0.007429
GO:00510	positive re	14/673	179/18670	0.005476	0.038465	0.029512	NGF/S100/	14	geneontol	0.020802
GO:00455	mast cell a	7/673	60/18670	0.005644	0.039546	0.030342	S100A12/(	7	geneontol	0.010401
GO:00443	cellular res	12/673	143/18670	0.005647	0.039546	0.030342	CXCL13/F/	12	geneontol	0.017831
GO:00160	immunogl	16/673	218/18670	0.005741	0.040147	0.030803	PTPRC/IL1	16	geneontol	0.023774
GO:00507	regulation	6/673	46/18670	0.00589	0.040974	0.031437	NLRP3/LPI	6	geneontol	0.008915
GO:00509	negative c	6/673	46/18670	0.00589	0.040974	0.031437	SEMA3G/S	6	geneontol	0.008915
GO:00607	labyrinthin	6/673	46/18670	0.00589	0.040974	0.031437	GJB5/IL10/	6	geneontol	0.008915
GO:00308	regulation	13/673	162/18670	0.005904	0.040974	0.031437	ADD2/VILI	13	geneontol	0.019316
GO:00357	CD4-posit	9/673	92/18670	0.005909	0.040974	0.031437	PLA2G2D/	9	geneontol	0.013373
GO:00027	innate imn	20/673	298/18670	0.005913	0.040974	0.031437	S100A14/N	20	geneontol	0.029718
GO:00720	nephron e	8/673	76/18670	0.005968	0.041291	0.03168	TACSTD2/	8	geneontol	0.011887
GO:00024	acute infla	4/673	21/18670	0.00614	0.04138	0.031748	CXCR2/CN	4	geneontol	0.005944
GO:00302	maintenan	4/673	21/18670	0.00614	0.04138	0.031748	MUC4/TFF	4	geneontol	0.005944
GO:00358	regulation	4/673	21/18670	0.00614	0.04138	0.031748	NPR1/AGT	4	geneontol	0.005944
GO:00607	labyrinthin	4/673	21/18670	0.00614	0.04138	0.031748	GJB5/IL10/	4	geneontol	0.005944
GO:00721	cell prolife	4/673	21/18670	0.00614	0.04138	0.031748	OSR1/SHH	4	geneontol	0.005944
GO:01401	positive re	4/673	21/18670	0.00614	0.04138	0.031748	CCR2/CXC	4	geneontol	0.005944
GO:20003	regulation	4/673	21/18670	0.00614	0.04138	0.031748	SHH/FGFR	4	geneontol	0.005944
GO:00019	regulation	5/673	33/18670	0.006162	0.04138	0.031748	CD1C/CD1	5	geneontol	0.007429
GO:00351	embryonic	5/673	33/18670	0.006162	0.04138	0.031748	CRABP2/C	5	geneontol	0.007429
GO:00507	positive re	5/673	33/18670	0.006162	0.04138	0.031748	NLRP3/LPI	5	geneontol	0.007429
GO:00702	regulation	5/673	33/18670	0.006162	0.04138	0.031748	EFNA1/IL7	5	geneontol	0.007429
GO:19018	positive re	5/673	33/18670	0.006162	0.04138	0.031748	EPHA2/S1(	5	geneontol	0.007429
GO:20010	regulation	5/673	33/18670	0.006162	0.04138	0.031748	IL10/HGF/	5	geneontol	0.007429
GO:00030	heart morp	18/673	259/18670	0.006178	0.04138	0.031748	TGFBR3/S1	18	geneontol	0.026746
GO:00022	alpha-beta	7/673	61/18670	0.006183	0.04138	0.031748	LY9/NLRP3	7	geneontol	0.010401
GO:00022	alpha-beta	7/673	61/18670	0.006183	0.04138	0.031748	LY9/NLRP3	7	geneontol	0.010401
GO:00507	interleukin	7/673	61/18670	0.006183	0.04138	0.031748	NLRP3/CD	7	geneontol	0.010401
GO:00026	negative re	3/673	11/18670	0.006195	0.04138	0.031748	FCRL3/IL3	3	geneontol	0.004458
GO:00029	regulation	3/673	11/18670	0.006195	0.04138	0.031748	PTPRC/C4I	3	geneontol	0.004458
GO:00316	positive re	3/673	11/18670	0.006195	0.04138	0.031748	PTGER3/N	3	geneontol	0.004458
GO:00357	natural kill	3/673	11/18670	0.006195	0.04138	0.031748	PIK3CG/C(	3	geneontol	0.004458

GO:00431	positive re	3/673	11/18670	0.006195	0.04138	0.031748	CXCR2/PD	3	geneontol	0.004458
GO:00453	type I inte	3/673	11/18670	0.006195	0.04138	0.031748	IL10/TLR7/	3	geneontol	0.004458
GO:19059	regulation	3/673	11/18670	0.006195	0.04138	0.031748	FGFR1/SO	3	geneontol	0.004458
GO:00060	glycosami	10/673	110/1867C	0.006396	0.042658	0.03273	B3GNT7/H	10	geneontol	0.014859
GO:00197	B cell med	16/673	221/1867C	0.006526	0.043468	0.03335	PTPRC/IL1	16	geneontol	0.023774
GO:00613	trabecula	6/673	47/18670	0.006551	0.043507	0.033381	TGFBR3/S	6	geneontol	0.008915
GO:00988	bone grow	6/673	47/18670	0.006551	0.043507	0.033381	LEPR/DDR	6	geneontol	0.008915
GO:00458	negative re	23/673	363/18670	0.00659	0.043679	0.033513	SFN/NGF/	23	geneontol	0.034175
GO:00508	T cell rece	15/673	202/1867C	0.006617	0.043679	0.033513	PDE4B/PT	15	geneontol	0.022288
GO:00507	regulation	14/673	183/1867C	0.006627	0.043679	0.033513	NGF/EFNA	14	geneontol	0.020802
GO:00512	negative re	12/673	146/1867C	0.006643	0.043679	0.033513	PLA2G2D/	12	geneontol	0.017831
GO:00513	response t	12/673	146/1867C	0.006643	0.043679	0.033513	IL10/FAM1	12	geneontol	0.017831
GO:19048	regulation	12/673	146/1867C	0.006643	0.043679	0.033513	TNFSF18/	12	geneontol	0.017831
GO:20002	regulation	12/673	146/1867C	0.006643	0.043679	0.033513	CNR1/LFN	12	geneontol	0.017831
GO:00613	renal tubu	8/673	78/18670	0.006972	0.045607	0.034991	TACSTD2/	8	geneontol	0.011887
GO:00706	negative re	8/673	78/18670	0.006972	0.045607	0.034991	PLA2G2D/	8	geneontol	0.011887
GO:00720	nephron m	8/673	78/18670	0.006972	0.045607	0.034991	TACSTD2/	8	geneontol	0.011887
GO:19030	negative re	11/673	129/1867C	0.007006	0.045607	0.034991	PLA2G2D/	11	geneontol	0.016345
GO:00364	cell death	5/673	34/18670	0.007015	0.045607	0.034991	IL10/HGF/	5	geneontol	0.007429
GO:00425	tumor nec	5/673	34/18670	0.007015	0.045607	0.034991	IL10/CX3C	5	geneontol	0.007429
GO:00425	regulation	5/673	34/18670	0.007015	0.045607	0.034991	IL10/CX3C	5	geneontol	0.007429
GO:00485	embryonic	5/673	34/18670	0.007015	0.045607	0.034991	IHH/PDGF	5	geneontol	0.007429
GO:00508	synapse o	25/673	408/1867C	0.00718	0.046614	0.035764	EFNA1/IG	25	geneontol	0.037147
GO:00308	prostate g	6/673	48/18670	0.007264	0.046828	0.035928	FGF10/SH	6	geneontol	0.008915
GO:00468	regulation	6/673	48/18670	0.007264	0.046828	0.035928	LEPR/S1P	6	geneontol	0.008915
GO:00027	positive re	9/673	95/18670	0.007271	0.046828	0.035928	PTPRC/NL	9	geneontol	0.013373
GO:00355	calcium-m	4/673	22/18670	0.007294	0.046828	0.035928	PTGFR/SE	4	geneontol	0.005944
GO:00420	interleukin	4/673	22/18670	0.007294	0.046828	0.035928	PTPRC/CD	4	geneontol	0.005944
GO:00434	regulation	4/673	22/18670	0.007294	0.046828	0.035928	ITGA4/LG	4	geneontol	0.005944
GO:00483	immunogl	4/673	22/18670	0.007294	0.046828	0.035928	LAX1/IL33	4	geneontol	0.005944
GO:00713	cellular re	4/673	22/18670	0.007294	0.046828	0.035928	PTGFR/PP	4	geneontol	0.005944
GO:00217	developm	19/673	284/1867C	0.007421	0.047581	0.036507	TAL1/S1P	19	geneontol	0.028232
GO:00015	vasculoge	8/673	79/18670	0.007519	0.048143	0.036937	EPHA2/TG	8	geneontol	0.011887
GO:00096	response t	21/673	326/18670	0.00767	0.049038	0.037624	PTPRC/NL	21	geneontol	0.031204
GO:00019	cell killing	13/673	168/1867C	0.007942	0.05023	0.038539	S100A12/	13	geneontol	0.019316
GO:00420	type 2 imn	5/673	35/18670	0.007946	0.05023	0.038539	IL10/NLRP	5	geneontol	0.007429
GO:00456	positive re	7/673	64/18670	0.008028	0.05023	0.038539	PPARG/LP	7	geneontol	0.010401
GO:00019	T cell med	6/673	49/18670	0.008032	0.05023	0.038539	CD1C/CD1	6	geneontol	0.008915
GO:00032	regulation	6/673	49/18670	0.008032	0.05023	0.038539	KDR/SCN2	6	geneontol	0.008915
GO:19030	regulation	6/673	49/18670	0.008032	0.05023	0.038539	PTX3/CCL	6	geneontol	0.008915
GO:00082	opsonizati	3/673	12/18670	0.008041	0.05023	0.038539	C4BPB/PT	3	geneontol	0.004458
GO:00315	hemidesm	3/673	12/18670	0.008041	0.05023	0.038539	LAMB3/CC	3	geneontol	0.004458
GO:00455	positive re	3/673	12/18670	0.008041	0.05023	0.038539	CD27/NCK	3	geneontol	0.004458
GO:00465	saliva secr	3/673	12/18670	0.008041	0.05023	0.038539	FGF10/AQ	3	geneontol	0.004458
GO:00604	regulation	3/673	12/18670	0.008041	0.05023	0.038539	PTGER3/N	3	geneontol	0.004458
GO:00606	branching	3/673	12/18670	0.008041	0.05023	0.038539	IL10/ST14/	3	geneontol	0.004458
GO:00704	thrombin-	3/673	12/18670	0.008041	0.05023	0.038539	PLEK/MET	3	geneontol	0.004458
GO:00725	T-helper 1	3/673	12/18670	0.008041	0.05023	0.038539	LY9/IRF4/	3	geneontol	0.004458
GO:00972	hepatocyt	3/673	12/18670	0.008041	0.05023	0.038539	PIK3CG/K	3	geneontol	0.004458
GO:19041	positive re	3/673	12/18670	0.008041	0.05023	0.038539	KDR/DCN/	3	geneontol	0.004458
GO:19055	negative re	3/673	12/18670	0.008041	0.05023	0.038539	CD200R1/	3	geneontol	0.004458
GO:00717	response t	12/673	150/1867C	0.008181	0.051037	0.039158	CXCL13/F	12	geneontol	0.017831
GO:00486	regulation	13/673	169/1867C	0.00833	0.051897	0.039818	S1PR1/NP	13	geneontol	0.019316
GO:00027	regulation	29/673	500/1867C	0.008364	0.052037	0.039925	SLC2A1/C	29	geneontol	0.043091
GO:19012	negative re	15/673	208/1867C	0.008573	0.052897	0.040585	NGF/IL10/	15	geneontol	0.022288
GO:00305	male genit	4/673	23/18670	0.008582	0.052897	0.040585	PDGFRA/F	4	geneontol	0.005944
GO:00358	renal sodi	4/673	23/18670	0.008582	0.052897	0.040585	NPR1/AGT	4	geneontol	0.005944
GO:00487	positive re	4/673	23/18670	0.008582	0.052897	0.040585	PPARG/SH	4	geneontol	0.005944
GO:19007	regulation	4/673	23/18670	0.008582	0.052897	0.040585	XDH/ADA	4	geneontol	0.005944
GO:19034	regulation	4/673	23/18670	0.008582	0.052897	0.040585	LRRK2/ED	4	geneontol	0.005944
GO:20000	regulation	4/673	23/18670	0.008582	0.052897	0.040585	SFRP4/SFR	4	geneontol	0.005944
GO:00507	positive re	18/673	268/18670	0.00867	0.053241	0.040849	CD2/IL10/	18	geneontol	0.026746
GO:00060	aminoglyc	10/673	115/1867C	0.008672	0.053241	0.040849	B3GNT7/H	10	geneontol	0.014859
GO:00329	collagen m	10/673	115/1867C	0.008672	0.053241	0.040849	IHH/PPAR	10	geneontol	0.014859
GO:00140	neural cre	8/673	81/18670	0.008711	0.053407	0.040976	ZEB2/SEM	8	geneontol	0.011887
GO:00606	ureteric bu	7/673	65/18670	0.008725	0.053423	0.040989	TACSTD2/	7	geneontol	0.010401
GO:00017	endoderm	6/673	50/18670	0.008856	0.05408	0.041493	LAMB3/IT	6	geneontol	0.008915
GO:00451	intermedia	6/673	50/18670	0.008856	0.05408	0.041493	SHH/KRT1	6	geneontol	0.008915
GO:00712	cellular re	15/673	209/1867C	0.008939	0.054433	0.041763	GJB3/PTG	15	geneontol	0.022288
GO:00027	MyD88-de	5/673	36/18670	0.00896	0.054433	0.041763	TLR10/CD	5	geneontol	0.007429
GO:00104	regulation	5/673	36/18670	0.00896	0.054433	0.041763	SPDEF/FG	5	geneontol	0.007429
GO:00604	lung epith	5/673	36/18670	0.00896	0.054433	0.041763	FGF10/SP	5	geneontol	0.007429
GO:00105	regulation	16/673	229/1867C	0.009048	0.054841	0.042077	EPHA2/EF	16	geneontol	0.023774

GO:00459	negative re	17/673	249/18670	0.009059	0.054841	0.042077	NPR1/KCN	17	geneontol	0.02526
GO:00015	regulation	25/673	416/18670	0.009063	0.054841	0.042077	SFN/NGF/	25	geneontol	0.037147
GO:00486	smooth m	13/673	171/18670	0.00915	0.055297	0.042427	S1PR1/NP	13	geneontol	0.019316
GO:00302	entry into	11/673	134/18670	0.009225	0.055455	0.042548	EPHA2/SL	11	geneontol	0.016345
GO:00444	entry into	11/673	134/18670	0.009225	0.055455	0.042548	EPHA2/SL	11	geneontol	0.016345
GO:00518	entry into	11/673	134/18670	0.009225	0.055455	0.042548	EPHA2/SL	11	geneontol	0.016345
GO:00518	entry into	11/673	134/18670	0.009225	0.055455	0.042548	EPHA2/SL	11	geneontol	0.016345
GO:00326	regulation	8/673	82/18670	0.009357	0.056178	0.043102	EPHA2/AC	8	geneontol	0.011887
GO:00713	cellular res	19/673	291/18670	0.009497	0.056943	0.043689	TNFSF18/	19	geneontol	0.028232
GO:00328	positive re	13/673	172/18670	0.009584	0.057391	0.044033	SLAMF1/X	13	geneontol	0.019316
GO:00027	regulation	6/673	51/18670	0.009739	0.058017	0.044514	PTPRC/IL1	6	geneontol	0.008915
GO:00028	regulation	6/673	51/18670	0.009739	0.058017	0.044514	PTPRC/IL1	6	geneontol	0.008915
GO:00451	intermedia	6/673	51/18670	0.009739	0.058017	0.044514	SHH/KRT1	6	geneontol	0.008915
GO:00514	positive re	6/673	51/18670	0.009739	0.058017	0.044514	S100A10/S	6	geneontol	0.008915
GO:00215	telencepha	17/673	251/18670	0.009763	0.058042	0.044532	SLC2A1/ZE	17	geneontol	0.02526
GO:00160	cell growth	28/673	484/18670	0.009768	0.058042	0.044532	SFN/NGF/	28	geneontol	0.041605
GO:19011	carbohydr	14/673	192/18670	0.009927	0.05859	0.044953	PDE4B/PD	14	geneontol	0.020802
GO:00330	regulation	4/673	24/18670	0.01001	0.05859	0.044953	IHH/IL7R/S	4	geneontol	0.005944
GO:00341	toll-like re	4/673	24/18670	0.01001	0.05859	0.044953	FCRL3/LILI	4	geneontol	0.005944
GO:00440	regulation	4/673	24/18670	0.01001	0.05859	0.044953	NPR1/AGT	4	geneontol	0.005944
GO:19025	regulation	4/673	24/18670	0.01001	0.05859	0.044953	XDH/ADAI	4	geneontol	0.005944
GO:19030	positive re	4/673	24/18670	0.01001	0.05859	0.044953	CCL19/CC	4	geneontol	0.005944
GO:00016	temperatu	13/673	173/18670	0.010033	0.05859	0.044953	LEPR/PTGI	13	geneontol	0.019316
GO:00703	positive re	13/673	173/18670	0.010033	0.05859	0.044953	SLAMF1/X	13	geneontol	0.019316
GO:00344	substrate a	9/673	100/18670	0.010058	0.05859	0.044953	TACSTD2/	9	geneontol	0.013373
GO:00352	segmentat	9/673	100/18670	0.010058	0.05859	0.044953	OSR1/ZEB	9	geneontol	0.013373
GO:20010	regulation	9/673	100/18670	0.010058	0.05859	0.044953	IL10/OSR1	9	geneontol	0.013373
GO:00031	regulation	5/673	37/18670	0.01006	0.05859	0.044953	FGF10/SHI	5	geneontol	0.007429
GO:00511	striated m	19/673	293/18670	0.010168	0.05859	0.044953	CAPN9/PD	19	geneontol	0.028232
GO:00028	regulation	3/673	13/18670	0.010176	0.05859	0.044953	CNR1/CCR	3	geneontol	0.004458
GO:00104	negative re	3/673	13/18670	0.010176	0.05859	0.044953	SPDEF/SO	3	geneontol	0.004458
GO:00108	positive re	3/673	13/18670	0.010176	0.05859	0.044953	FGF10/FGI	3	geneontol	0.004458
GO:00312	positive re	3/673	13/18670	0.010176	0.05859	0.044953	CCL21/CC	3	geneontol	0.004458
GO:00325	regulation	3/673	13/18670	0.010176	0.05859	0.044953	CDHR2/CD	3	geneontol	0.004458
GO:00450	positive re	3/673	13/18670	0.010176	0.05859	0.044953	TNFRSF13	3	geneontol	0.004458
GO:00508	white fat c	3/673	13/18670	0.010176	0.05859	0.044953	PPARG/FG	3	geneontol	0.004458
GO:00607	spongiotro	3/673	13/18670	0.010176	0.05859	0.044953	GJB5/PHLI	3	geneontol	0.004458
GO:00996	protein loc	3/673	13/18670	0.010176	0.05859	0.044953	ADAM22/I	3	geneontol	0.004458
GO:00996	neurotrans	3/673	13/18670	0.010176	0.05859	0.044953	ADAM22/I	3	geneontol	0.004458
GO:19039	negative re	3/673	13/18670	0.010176	0.05859	0.044953	OSR1/ANC	3	geneontol	0.004458
GO:20000	positive re	3/673	13/18670	0.010176	0.05859	0.044953	SFRP1/DKI	3	geneontol	0.004458
GO:00109	negative re	12/673	155/18670	0.01048	0.060262	0.046236	EFNA1/NG	12	geneontol	0.017831
GO:00326	regulation	6/673	52/18670	0.010682	0.06135	0.047071	CD28/TIGI	6	geneontol	0.008915
GO:00105	negative re	8/673	84/18670	0.010755	0.061693	0.047333	LEPR/IL10/	8	geneontol	0.011887
GO:00464	regulation	11/673	137/18670	0.010795	0.061845	0.04745	TNFSF18/I	11	geneontol	0.016345
GO:00513	negative re	27/673	466/18670	0.01083	0.061968	0.047545	SFN/LEPR/	27	geneontol	0.040119
GO:00519	regulation	17/673	254/18670	0.010901	0.062298	0.047798	PDE4B/FCI	17	geneontol	0.02526
GO:20001	negative re	13/673	175/18670	0.01098	0.062674	0.048086	EPHA2/NP	13	geneontol	0.019316
GO:00308	regulation	12/673	156/18670	0.010994	0.062675	0.048087	SFN/OSR1	12	geneontol	0.017831
GO:00027	negative re	5/673	38/18670	0.011249	0.063893	0.049022	PTPRC/C4I	5	geneontol	0.007429
GO:00507	positive re	5/673	38/18670	0.011249	0.063893	0.049022	NLRP3/LPI	5	geneontol	0.007429
GO:19040	positive re	5/673	38/18670	0.011249	0.063893	0.049022	ITGA4/SFR	5	geneontol	0.007429
GO:00309	forebrain c	23/673	381/18670	0.011402	0.064624	0.049582	SLC2A1/ZE	23	geneontol	0.034175
GO:00605	developme	16/673	235/18670	0.011406	0.064624	0.049582	S1PR1/NG	16	geneontol	0.023774
GO:00456	regulation	8/673	85/18670	0.01151	0.064995	0.049867	SFN/SFRP4	8	geneontol	0.011887
GO:00507	positive re	8/673	85/18670	0.01151	0.064995	0.049867	NGF/CRAE	8	geneontol	0.011887
GO:00720	stem cell p	10/673	120/18670	0.011524	0.064995	0.049867	PTPRC/CX	10	geneontol	0.014859
GO:00027	negative re	4/673	25/18670	0.011584	0.064995	0.049867	IL10/CD96	4	geneontol	0.005944
GO:00094	toxin meta	4/673	25/18670	0.011584	0.064995	0.049867	FMO2/FM	4	geneontol	0.005944
GO:00518	positive re	4/673	25/18670	0.011584	0.064995	0.049867	S100A10/K	4	geneontol	0.005944
GO:19058	negative re	4/673	25/18670	0.011584	0.064995	0.049867	EFNA1/NG	4	geneontol	0.005944
GO:20003	positive re	4/673	25/18670	0.011584	0.064995	0.049867	ITGA4/THE	4	geneontol	0.005944
GO:00507	regulation	6/673	53/18670	0.011689	0.065506	0.05026	NLRP3/LPI	6	geneontol	0.008915
GO:00071	G protein-	17/673	256/18670	0.011716	0.065581	0.050317	GRIK3/PTG	17	geneontol	0.02526
GO:00067	isoprenoid	11/673	139/18670	0.011951	0.066811	0.051261	CRABP2/C	11	geneontol	0.016345
GO:00467	viral entry	10/673	121/18670	0.012171	0.067961	0.052143	EPHA2/SL	10	geneontol	0.014859
GO:00019	hair follicle	8/673	86/18670	0.012302	0.068612	0.052642	FGF10/FO	8	geneontol	0.011887
GO:00305	positive re	5/673	39/18670	0.012531	0.06924	0.053124	OSR1/TFA	5	geneontol	0.007429
GO:00487	regulation	5/673	39/18670	0.012531	0.06924	0.053124	SLC45A3/I	5	geneontol	0.007429
GO:00508	regulation	5/673	39/18670	0.012531	0.06924	0.053124	TRAT1/TES	5	geneontol	0.007429
GO:00075	mesoderm	3/673	14/18670	0.012609	0.06924	0.053124	FGFR1/SO	3	geneontol	0.004458
GO:00107	regulation	3/673	14/18670	0.012609	0.06924	0.053124	NPR1/PDE	3	geneontol	0.004458
GO:00312	regulation	3/673	14/18670	0.012609	0.06924	0.053124	CCL21/CC	3	geneontol	0.004458

GO:00343	triglycerid	3/673	14/18670	0.012609	0.06924	0.053124	LPL/GPIHB	3	geneontol	0.004458
GO:00358	positive re	3/673	14/18670	0.012609	0.06924	0.053124	NPR1/AVP	3	geneontol	0.004458
GO:00466	gamma-d	3/673	14/18670	0.012609	0.06924	0.053124	PTPRC/NC	3	geneontol	0.004458
GO:00482	behavioral	3/673	14/18670	0.012609	0.06924	0.053124	THBS4/TH	3	geneontol	0.004458
GO:00509	induction	3/673	14/18670	0.012609	0.06924	0.053124	FGF10/CX	3	geneontol	0.004458
GO:00609	cardiac cel	3/673	14/18670	0.012609	0.06924	0.053124	SOX17/DK	3	geneontol	0.004458
GO:19020	positive re	3/673	14/18670	0.012609	0.06924	0.053124	MAL/SFRP	3	geneontol	0.004458
GO:00510	positive re	25/673	428/1867	0.012627	0.069254	0.053135	CD2/NPR1	25	geneontol	0.037147
GO:00510	negative r	16/673	238/1867	0.012753	0.069822	0.053571	PTGER3/C	16	geneontol	0.023774
GO:00301	collagen fi	6/673	54/18670	0.012761	0.069822	0.053571	DDR2/DPT	6	geneontol	0.008915
GO:00485	response t	23/673	385/1867	0.012784	0.069866	0.053604	IL10/PPAR	23	geneontol	0.034175
GO:00100	glial cell di	15/673	218/1867	0.012825	0.069924	0.053649	TAL1/SLC	15	geneontol	0.022288
GO:00322	regulation	15/673	218/1867	0.012825	0.069924	0.053649	ADD2/VILL	15	geneontol	0.022288
GO:00017	somitogen	7/673	70/18670	0.01289	0.070196	0.053858	ZEB2/LFN	7	geneontol	0.010401
GO:00487	skeletal sy	16/673	239/1867	0.013229	0.071546	0.054893	OSR1/VIT/	16	geneontol	0.023774
GO:00302	glycosami	12/673	160/1867	0.013249	0.071546	0.054893	B3GNT7/H	12	geneontol	0.017831
GO:00019	positive re	4/673	26/18670	0.013308	0.071546	0.054893	CD1C/CD1	4	geneontol	0.005944
GO:00107	regulation	4/673	26/18670	0.013308	0.071546	0.054893	STAP1/CM	4	geneontol	0.005944
GO:00301	positive re	4/673	26/18670	0.013308	0.071546	0.054893	SELP/PLEK	4	geneontol	0.005944
GO:00316	negative r	4/673	26/18670	0.013308	0.071546	0.054893	IL10/GPR3	4	geneontol	0.005944
GO:00606	embryonic	4/673	26/18670	0.013308	0.071546	0.054893	GJB5/IL10/	4	geneontol	0.005944
GO:00607	prostate g	4/673	26/18670	0.013308	0.071546	0.054893	FGF10/SH	4	geneontol	0.005944
GO:00725	T-helper 1	4/673	26/18670	0.013308	0.071546	0.054893	LY9/NLRP	4	geneontol	0.005944
GO:00995	synaptic m	4/673	26/18670	0.013308	0.071546	0.054893	SPARCL1/	4	geneontol	0.005944
GO:19000	positive re	4/673	26/18670	0.013308	0.071546	0.054893	SELP/PLEK	4	geneontol	0.005944
GO:00508	positive re	11/673	142/1867	0.013858	0.074101	0.056854	FCRL3/PTF	11	geneontol	0.016345
GO:00425	positive re	7/673	71/18670	0.013869	0.074101	0.056854	TNFSF18/I	7	geneontol	0.010401
GO:00326	interleukin	6/673	55/18670	0.0139	0.074101	0.056854	CD28/TIGI	6	geneontol	0.008915
GO:00610	positive re	6/673	55/18670	0.0139	0.074101	0.056854	TAL1/EFN	6	geneontol	0.008915
GO:00019	regulation	5/673	40/18670	0.013907	0.074101	0.056854	AGTR1/AV	5	geneontol	0.007429
GO:00028	negative r	5/673	40/18670	0.013907	0.074101	0.056854	PTPRC/C4	5	geneontol	0.007429
GO:00715	semaphori	5/673	40/18670	0.013907	0.074101	0.056854	SEMA3G/S	5	geneontol	0.007429
GO:00192	regulation	24/673	410/1867	0.014	0.074101	0.056854	RGL1/SLC	24	geneontol	0.035661
GO:00224	molting cy	8/673	88/18670	0.014007	0.074101	0.056854	FGF10/FO	8	geneontol	0.011887
GO:00224	hair cycle	8/673	88/18670	0.014007	0.074101	0.056854	FGF10/FO	8	geneontol	0.011887
GO:00341	regulation	8/673	88/18670	0.014007	0.074101	0.056854	LEPR/S1P	8	geneontol	0.011887
GO:00435	regulation	8/673	88/18670	0.014007	0.074101	0.056854	ZEB2/SFR	8	geneontol	0.011887
GO:00987	skin epide	8/673	88/18670	0.014007	0.074101	0.056854	FGF10/FO	8	geneontol	0.011887
GO:19040	regulation	8/673	88/18670	0.014007	0.074101	0.056854	ITGA4/KD	8	geneontol	0.011887
GO:00104	negative r	17/673	262/1867	0.014454	0.076377	0.0586	SFN/NGF/	17	geneontol	0.02526
GO:00319	response t	12/673	162/1867	0.014501	0.076541	0.058726	IL10/FAM1	12	geneontol	0.017831
GO:00326	chemokine	8/673	89/18670	0.014921	0.078577	0.060288	EPHA2/AC	8	geneontol	0.011887
GO:00600	roof of mc	8/673	89/18670	0.014921	0.078577	0.060288	TGFBR3/O	8	geneontol	0.011887
GO:19030	positive re	6/673	56/18670	0.015107	0.07946	0.060966	ARHGEF16	6	geneontol	0.008915
GO:00028	regulation	4/673	27/18670	0.015187	0.07946	0.060966	IL1R1/CCR	4	geneontol	0.005944
GO:00508	positive re	4/673	27/18670	0.015187	0.07946	0.060966	SELP/PLEK	4	geneontol	0.005944
GO:00602	regulation	4/673	27/18670	0.015187	0.07946	0.060966	LEPR/NMU	4	geneontol	0.005944
GO:00722	metaneph	4/673	27/18670	0.015187	0.07946	0.060966	OSR1/CXC	4	geneontol	0.005944
GO:00024	germinal c	3/673	15/18670	0.015346	0.07946	0.060966	KLHL6/CX	3	geneontol	0.004458
GO:00327	positive re	3/673	15/18670	0.015346	0.07946	0.060966	NLRP3/IRF	3	geneontol	0.004458
GO:00343	primary al	3/673	15/18670	0.015346	0.07946	0.060966	SULT1C4/	3	geneontol	0.004458
GO:00468	negative r	3/673	15/18670	0.015346	0.07946	0.060966	CLDN18/S	3	geneontol	0.004458
GO:00480	regulation	3/673	15/18670	0.015346	0.07946	0.060966	ZEB2/IHH/	3	geneontol	0.004458
GO:00517	positive re	3/673	15/18670	0.015346	0.07946	0.060966	KDR/LRRK	3	geneontol	0.004458
GO:00720	metaneph	3/673	15/18670	0.015346	0.07946	0.060966	OSR1/SHH	3	geneontol	0.004458
GO:00901	epithelial	3/673	15/18670	0.015346	0.07946	0.060966	CYP1B1/IH	3	geneontol	0.004458
GO:00901	positive re	3/673	15/18670	0.015346	0.07946	0.060966	CSF1R/LPL	3	geneontol	0.004458
GO:20000	regulation	3/673	15/18670	0.015346	0.07946	0.060966	SFRP1/DK	3	geneontol	0.004458
GO:00902	regulation	5/673	41/18670	0.015382	0.079555	0.061038	FCRL3/PLN	5	geneontol	0.007429
GO:00488	sensory sy	22/673	371/1867	0.015843	0.081851	0.0628	EPHA2/CY	22	geneontol	0.032689
GO:00164	protein pr	20/673	328/1867	0.015865	0.081872	0.062816	NGF/CTSE	20	geneontol	0.029718
GO:00329	negative r	7/673	73/18670	0.015985	0.082126	0.063011	PLA2G2D/	7	geneontol	0.010401
GO:00436	response t	7/673	73/18670	0.015985	0.082126	0.063011	PPARG/ZN	7	geneontol	0.010401
GO:00506	negative r	7/673	73/18670	0.015985	0.082126	0.063011	PLA2G2D/	7	geneontol	0.010401
GO:00603	endochon	7/673	73/18670	0.015985	0.082126	0.063011	VIT/IHH/C	7	geneontol	0.010401
GO:00514	stress-act	18/673	286/1867	0.01605	0.082367	0.063196	SLAMF1/X	18	geneontol	0.026746
GO:00314	keratinizat	15/673	224/1867	0.016072	0.082387	0.063211	SFN/LIPM	15	geneontol	0.022288
GO:00325	response t	9/673	108/1867	0.016093	0.082405	0.063225	GJB3/OSR	9	geneontol	0.013373
GO:00109	regulation	23/673	394/1867	0.016386	0.083812	0.064305	PDE4B/FC	23	geneontol	0.034175
GO:00716	anatomica	12/673	165/1867	0.016546	0.084536	0.06486	TAL1/S1P	12	geneontol	0.017831
GO:00456	negative r	15/673	225/1867	0.016669	0.085074	0.065272	EFNA1/NG	15	geneontol	0.022288
GO:00022	toll-like re	11/673	146/1867	0.016748	0.085382	0.065509	S100A14/	11	geneontol	0.016345
GO:00463	regulation	13/673	185/1867	0.016801	0.085556	0.065643	SLAMF1/Z	13	geneontol	0.019316

GO:00518	membrane	8/673	91/18670	0.016878	0.085853	0.06587	SCN7A/KD	8	geneontol	0.011887
GO:00026	positive re	5/673	42/18670	0.016957	0.08588	0.065891	PTPRC/CD	5	geneontol	0.007429
GO:00104	regulation	5/673	42/18670	0.016957	0.08588	0.065891	IL10/OSR1	5	geneontol	0.007429
GO:00105	regulation	5/673	42/18670	0.016957	0.08588	0.065891	CCL19/CC	5	geneontol	0.007429
GO:00970	dendritic c	5/673	42/18670	0.016957	0.08588	0.065891	IRF4/CCL1	5	geneontol	0.007429
GO:00027	positive re	18/673	288/18670	0.017105	0.086198	0.066135	CD2/IL10/	18	geneontol	0.026746
GO:00435	positive re	7/673	74/18670	0.017125	0.086198	0.066135	ZEB2/CCL	7	geneontol	0.010401
GO:00031	aortic valv	4/673	28/18670	0.017225	0.086198	0.066135	EFNA1/SLI	4	geneontol	0.005944
GO:00181	keratan su	4/673	28/18670	0.017225	0.086198	0.066135	B3GNT7/C	4	geneontol	0.005944
GO:00326	regulation	4/673	28/18670	0.017225	0.086198	0.066135	IL10/LILRA	4	geneontol	0.005944
GO:00343	protein-lip	4/673	28/18670	0.017225	0.086198	0.066135	AGTR1/LP	4	geneontol	0.005944
GO:00343	plasma lip	4/673	28/18670	0.017225	0.086198	0.066135	AGTR1/LP	4	geneontol	0.005944
GO:00426	regulation	4/673	28/18670	0.017225	0.086198	0.066135	EPS8L3/CD	4	geneontol	0.005944
GO:19026	negative re	4/673	28/18670	0.017225	0.086198	0.066135	SEMA3G/S	4	geneontol	0.005944
GO:19037	negative re	4/673	28/18670	0.017225	0.086198	0.066135	OSR1/SFR	4	geneontol	0.005944
GO:19049	regulation	4/673	28/18670	0.017225	0.086198	0.066135	ITGA4/CCI	4	geneontol	0.005944
GO:00022	hematopo	12/673	166/18670	0.017273	0.086254	0.066178	TAL1/PTPI	12	geneontol	0.017831
GO:00902	negative re	12/673	166/18670	0.017273	0.086254	0.066178	TGFBR3/XI	12	geneontol	0.017831
GO:00025	platelet de	10/673	128/18670	0.017491	0.087155	0.066869	SELP/PLEK	10	geneontol	0.014859
GO:00303	negative re	13/673	186/18670	0.017491	0.087155	0.066869	NPR1/PPA	13	geneontol	0.019316
GO:00420	T-helper c	6/673	58/18670	0.017738	0.088287	0.067738	LY9/NLRP	6	geneontol	0.008915
GO:00020	epithelial c	14/673	207/18670	0.018147	0.089989	0.069044	EPHA2/SFI	14	geneontol	0.020802
GO:00074	peripheral	7/673	75/18670	0.018321	0.089989	0.069044	NGF/LAM	7	geneontol	0.010401
GO:00347	cellular ho	10/673	129/18670	0.018372	0.089989	0.069044	CRABP2/C	10	geneontol	0.014859
GO:00017	neutrophil	3/673	16/18670	0.018391	0.089989	0.069044	PDE4B/CX	3	geneontol	0.004458
GO:00022	T-helper c	3/673	16/18670	0.018391	0.089989	0.069044	LY9/IRF4/E	3	geneontol	0.004458
GO:00024	antigen pr	3/673	16/18670	0.018391	0.089989	0.069044	CD1C/CD1	3	geneontol	0.004458
GO:00108	positive re	3/673	16/18670	0.018391	0.089989	0.069044	CCR2/CXC	3	geneontol	0.004458
GO:00108	regulation	3/673	16/18670	0.018391	0.089989	0.069044	PPARG/CD	3	geneontol	0.004458
GO:00308	negative re	3/673	16/18670	0.018391	0.089989	0.069044	MNDA/IL1	3	geneontol	0.004458
GO:00312	pseudopo	3/673	16/18670	0.018391	0.089989	0.069044	CCL21/CC	3	geneontol	0.004458
GO:00330	negative re	3/673	16/18670	0.018391	0.089989	0.069044	CXCR2/CC	3	geneontol	0.004458
GO:00336	cell-cell ac	3/673	16/18670	0.018391	0.089989	0.069044	ITGA4/CX	3	geneontol	0.004458
GO:00480	hepatocyte	3/673	16/18670	0.018391	0.089989	0.069044	MST1R/HC	3	geneontol	0.004458
GO:00507	negative re	3/673	16/18670	0.018391	0.089989	0.069044	EFNA1/NG	3	geneontol	0.004458
GO:00705	dendrite se	3/673	16/18670	0.018391	0.089989	0.069044	NEXN/IGS	3	geneontol	0.004458
GO:00722	metanephi	3/673	16/18670	0.018391	0.089989	0.069044	OSR1/PDG	3	geneontol	0.004458
GO:00456	positive re	5/673	43/18670	0.018636	0.091093	0.069891	PPARG/SH	5	geneontol	0.007429
GO:00720	nephron tu	8/673	93/18670	0.019012	0.092836	0.071228	TACSTD2/	8	geneontol	0.011887
GO:00094	response t	18/673	292/18670	0.019378	0.09396	0.07209	GSTM5/S1	18	geneontol	0.026746
GO:00069	substrate-	4/673	29/18670	0.019425	0.09396	0.07209	PTPRC/P2	4	geneontol	0.005944
GO:00072	nitric oxide	4/673	29/18670	0.019425	0.09396	0.07209	CD36/GUC	4	geneontol	0.005944
GO:00106	epithelial s	4/673	29/18670	0.019425	0.09396	0.07209	MUC4/TFF	4	geneontol	0.005944
GO:00343	protein-cc	4/673	29/18670	0.019425	0.09396	0.07209	AGTR1/LP	4	geneontol	0.005944
GO:00447	modulatio	4/673	29/18670	0.019425	0.09396	0.07209	PTX3/CSF1	4	geneontol	0.005944
GO:00610	negative re	4/673	29/18670	0.019425	0.09396	0.07209	PTPRC/GP	4	geneontol	0.005944
GO:00725	T-helper 1	4/673	29/18670	0.019425	0.09396	0.07209	LY9/NLRP	4	geneontol	0.005944
GO:01200	negative re	4/673	29/18670	0.019425	0.09396	0.07209	TACSTD2/	4	geneontol	0.005944
GO:00517	interaction	14/673	209/18670	0.019549	0.094458	0.072473	EPHA2/SL	14	geneontol	0.020802
GO:00149	myotube c	9/673	112/18670	0.01995	0.096197	0.073807	SHH/BARX	9	geneontol	0.013373
GO:00508	negative re	9/673	112/18670	0.01995	0.096197	0.073807	PLA2G2D/	9	geneontol	0.013373
GO:00519	negative re	19/673	315/18670	0.020349	0.097878	0.075097	EFNA1/NG	19	geneontol	0.028232
GO:00034	endochon	5/673	44/18670	0.02042	0.097878	0.075097	DDR2/VIT	5	geneontol	0.007429
GO:00140	oligodend	5/673	44/18670	0.02042	0.097878	0.075097	SHH/LPAR	5	geneontol	0.007429
GO:00514	regulation	5/673	44/18670	0.02042	0.097878	0.075097	CCL21/TG	5	geneontol	0.007429
GO:19026	regulation	5/673	44/18670	0.02042	0.097878	0.075097	SEMA3G/S	5	geneontol	0.007429
GO:00060	aminoglyc	12/673	170/18670	0.020426	0.097878	0.075097	B3GNT7/H	12	geneontol	0.017831
GO:00022	CD4-posit	6/673	60/18670	0.020666	0.098926	0.0759	LY9/NLRP	6	geneontol	0.008915
GO:00068	nitric oxide	7/673	77/18670	0.020886	0.099876	0.076629	IL10/CYP1	7	geneontol	0.010401
GO:00424	odontoger	10/673	132/18670	0.021211	0.101309	0.077729	OSR1/PDG	10	geneontol	0.014859
GO:00507	negative re	18/673	295/18670	0.021229	0.101309	0.077729	EFNA1/NG	18	geneontol	0.026746
GO:00106	positive re	12/673	171/18670	0.021277	0.101431	0.077823	RAB25/KD	12	geneontol	0.017831
GO:00613	renal tubu	8/673	95/18670	0.021333	0.101597	0.07795	TACSTD2/	8	geneontol	0.011887
GO:00026	positive re	3/673	17/18670	0.021745	0.102203	0.078414	IL1R1/ITG	3	geneontol	0.004458
GO:00073	segment s	3/673	17/18670	0.021745	0.102203	0.078414	OSR1/ME	3	geneontol	0.004458
GO:00108	regulation	3/673	17/18670	0.021745	0.102203	0.078414	CCR2/CXC	3	geneontol	0.004458
GO:00303	stabilizatio	3/673	17/18670	0.021745	0.102203	0.078414	KCNK2/KC	3	geneontol	0.004458
GO:00359	chondrocy	3/673	17/18670	0.021745	0.102203	0.078414	DDR2/IHH	3	geneontol	0.004458
GO:00433	CD4-posit	3/673	17/18670	0.021745	0.102203	0.078414	LY9/IRF4/E	3	geneontol	0.004458
GO:00450	regulation	3/673	17/18670	0.021745	0.102203	0.078414	TNFRSF13	3	geneontol	0.004458
GO:00612	retina vasc	3/673	17/18670	0.021745	0.102203	0.078414	CYP1B1/P	3	geneontol	0.004458
GO:20008	negative re	3/673	17/18670	0.021745	0.102203	0.078414	NTRK2/BC	3	geneontol	0.004458
GO:00034	chondrocy	4/673	30/18670	0.021792	0.102203	0.078414	VIT/IHH/C	4	geneontol	0.005944

GO:00326(	interferon-	4/673	30/18670	0.021792	0.102203	0.078414	IL10/LILRA	4	geneontol	0.005944
GO:00433	positive re	4/673	30/18670	0.021792	0.102203	0.078414	NLRP3/CC	4	geneontol	0.005944
GO:00482(	response t	4/673	30/18670	0.021792	0.102203	0.078414	THBS4/ED	4	geneontol	0.005944
GO:00508(	negative re	4/673	30/18670	0.021792	0.102203	0.078414	MNDA/IL1	4	geneontol	0.005944
GO:00514	positive re	4/673	30/18670	0.021792	0.102203	0.078414	CCL21/TGI	4	geneontol	0.005944
GO:00326	regulation	11/673	152/18670	0.021897	0.102591	0.078712	SLAMF1/IL	11	geneontol	0.016345
GO:00085	fibroblast	9/673	114/18670	0.022111	0.103385	0.079321	FAT4/FGF1	9	geneontol	0.013373
GO:00454	endothelia	9/673	114/18670	0.022111	0.103385	0.079321	S1PR1/XDI	9	geneontol	0.013373
GO:00060	glycosamir	6/673	61/18670	0.022246	0.103548	0.079446	OGN/OMD	6	geneontol	0.008915
GO:00341	heterotypi	6/673	61/18670	0.022246	0.103548	0.079446	CD2/PTPR	6	geneontol	0.008915
GO:00456(	regulation	6/673	61/18670	0.022246	0.103548	0.079446	SFN/SFRP	6	geneontol	0.008915
GO:00486	animal org	6/673	61/18670	0.022246	0.103548	0.079446	FGF10/SHI	6	geneontol	0.008915
GO:00085	regulation	7/673	78/18670	0.022257	0.103548	0.079446	IHH/FGF1(	7	geneontol	0.010401
GO:00028	negative re	5/673	45/18670	0.022313	0.103598	0.079485	PTPRC/C4I	5	geneontol	0.007429
GO:00325	response t	5/673	45/18670	0.022313	0.103598	0.079485	CLDN4/CA	5	geneontol	0.007429
GO:00421	neurotrans	11/673	153/18670	0.022856	0.106015	0.081339	IL10/CYP1	11	geneontol	0.016345
GO:00075	hemostasis	20/673	341/18670	0.023016	0.106518	0.081725	SELP/C4BF	20	geneontol	0.029718
GO:00426	muscle cel	22/673	385/18670	0.023057	0.106518	0.081725	CAPN9/BC	22	geneontol	0.032689
GO:00104(	regulation	12/673	173/18670	0.023057	0.106518	0.081725	TAL1/IL10/	12	geneontol	0.017831
GO:00165	negative re	12/673	173/18670	0.023057	0.106518	0.081725	EPHA2/NF	12	geneontol	0.017831
GO:00466	response t	10/673	134/18670	0.023274	0.107411	0.082411	P2RY12/PI	10	geneontol	0.014859
GO:00301	regulation	7/673	79/18670	0.02369	0.109226	0.083803	SELP/PLEK	7	geneontol	0.010401
GO:00487(	oligodend	8/673	97/18670	0.023848	0.109843	0.084276	SLC45A3/F	8	geneontol	0.011887
GO:00328	glomerulus	6/673	62/18670	0.023905	0.109996	0.084394	OSR1/PDG	6	geneontol	0.008915
GO:00604	mammary	5/673	46/18670	0.024316	0.111273	0.085374	EPHA2/ELI	5	geneontol	0.007429
GO:00027	regulation	4/673	31/18670	0.024327	0.111273	0.085374	NLRP3/IL1	4	geneontol	0.005944
GO:00066	phosphatic	4/673	31/18670	0.024327	0.111273	0.085374	PLA2G2D/	4	geneontol	0.005944
GO:00458	positive re	4/673	31/18670	0.024327	0.111273	0.085374	IHH/SHH/	4	geneontol	0.005944
GO:19028	positive re	4/673	31/18670	0.024327	0.111273	0.085374	IL10/WT1/	4	geneontol	0.005944
GO:19033	positive re	4/673	31/18670	0.024327	0.111273	0.085374	S100A10/	4	geneontol	0.005944
GO:20003	regulation	13/673	195/18670	0.024708	0.112907	0.086627	IL10/XDH/	13	geneontol	0.019316
GO:20012	regulation	11/673	155/18670	0.024868	0.113523	0.0871	PTPRC/MA	11	geneontol	0.016345
GO:01500(	visual syste	21/673	366/18670	0.024977	0.113645	0.087194	EPHA2/CY	21	geneontol	0.031204
GO:00064	protein gly	16/673	258/18670	0.02518	0.113645	0.087194	MAN1C1/I	16	geneontol	0.023774
GO:00434	macromol	16/673	258/18670	0.02518	0.113645	0.087194	MAN1C1/I	16	geneontol	0.023774
GO:19000	regulation	7/673	80/18670	0.025185	0.113645	0.087194	SELP/PLEK	7	geneontol	0.010401
GO:00328	regulation	15/673	237/18670	0.025243	0.113645	0.087194	SLAMF1/X	15	geneontol	0.022288
GO:00034	chondrocy	3/673	18/18670	0.02541	0.113645	0.087194	VIT/COL21	3	geneontol	0.004458
GO:00034	growth pla	3/673	18/18670	0.02541	0.113645	0.087194	VIT/COL21	3	geneontol	0.004458
GO:00071	negative re	3/673	18/18670	0.02541	0.113645	0.087194	CCR2/P2R	3	geneontol	0.004458
GO:00093	oligosacch	3/673	18/18670	0.02541	0.113645	0.087194	SLC2A1/ST	3	geneontol	0.004458
GO:00108	cholestero	3/673	18/18670	0.02541	0.113645	0.087194	PPARG/CD	3	geneontol	0.004458
GO:00310(	positive re	3/673	18/18670	0.02541	0.113645	0.087194	SDR16C5/	3	geneontol	0.004458
GO:00361	phosphatic	3/673	18/18670	0.02541	0.113645	0.087194	PLA2G2D/	3	geneontol	0.004458
GO:00420	interferon-	3/673	18/18670	0.02541	0.113645	0.087194	TNFRSF13	3	geneontol	0.004458
GO:00510	regulation	3/673	18/18670	0.02541	0.113645	0.087194	IL33/CD22	3	geneontol	0.004458
GO:00518	negative re	3/673	18/18670	0.02541	0.113645	0.087194	FAM107A/	3	geneontol	0.004458
GO:00702	negative re	3/673	18/18670	0.02541	0.113645	0.087194	EFNA1/IL7	3	geneontol	0.004458
GO:00901	chondrocy	3/673	18/18670	0.02541	0.113645	0.087194	VIT/COL21	3	geneontol	0.004458
GO:00901	regulation	3/673	18/18670	0.02541	0.113645	0.087194	CSF1R/LPL	3	geneontol	0.004458
GO:00970	craniofacia	3/673	18/18670	0.02541	0.113645	0.087194	MMP16/FF	3	geneontol	0.004458
GO:01500	positive re	3/673	18/18670	0.02541	0.113645	0.087194	STAP1/IL3	3	geneontol	0.004458
GO:20006	negative re	3/673	18/18670	0.02541	0.113645	0.087194	OVOL1/OV	3	geneontol	0.004458
GO:00075	excretion	6/673	63/18670	0.025645	0.114365	0.087746	NPR1/AGT	6	geneontol	0.008915
GO:00343	protein loc	6/673	63/18670	0.025645	0.114365	0.087746	FGF10/AN	6	geneontol	0.008915
GO:19043	positive re	6/673	63/18670	0.025645	0.114365	0.087746	ARHGEF16	6	geneontol	0.008915
GO:00022(	somatic re	5/673	47/18670	0.026431	0.116856	0.089657	PTPRC/IL1	5	geneontol	0.007429
GO:00022(	somatic di	5/673	47/18670	0.026431	0.116856	0.089657	PTPRC/IL1	5	geneontol	0.007429
GO:00069	acute-pha	5/673	47/18670	0.026431	0.116856	0.089657	PTGER3/CI	5	geneontol	0.007429
GO:00149	positive re	5/673	47/18670	0.026431	0.116856	0.089657	NR4A3/LP	5	geneontol	0.007429
GO:00433	regulation	5/673	47/18670	0.026431	0.116856	0.089657	NLRP3/IRF	5	geneontol	0.007429
GO:00451	isotype sw	5/673	47/18670	0.026431	0.116856	0.089657	PTPRC/IL1	5	geneontol	0.007429
GO:00616	pri-miRNA	5/673	47/18670	0.026431	0.116856	0.089657	IL10/PPAR	5	geneontol	0.007429
GO:00701(	positive re	5/673	47/18670	0.026431	0.116856	0.089657	OSR1/TFA	5	geneontol	0.007429
GO:00702	T cell apop	5/673	47/18670	0.026431	0.116856	0.089657	EFNA1/IL7	5	geneontol	0.007429
GO:00485	negative re	8/673	99/18670	0.026563	0.117263	0.089969	PTX3/TRIM	8	geneontol	0.011887
GO:00022	pattern rec	13/673	197/18670	0.026573	0.117263	0.089969	S100A14/F	13	geneontol	0.019316
GO:00316	regulation	10/673	137/18670	0.026633	0.117414	0.090085	IL10/GPR3	10	geneontol	0.014859
GO:00484(	cell matura	12/673	177/18670	0.026943	0.118448	0.090879	TAL1/IHH/	12	geneontol	0.017831
GO:00313	negative re	15/673	239/18670	0.026944	0.118448	0.090879	PTPRC/IL1	15	geneontol	0.022288
GO:00703(	regulation	15/673	239/18670	0.026944	0.118448	0.090879	SLAMF1/X	15	geneontol	0.022288
GO:00031	aortic valv	4/673	32/18670	0.027033	0.118498	0.090917	EFNA1/SLI	4	geneontol	0.005944
GO:00425	retinoic ac	4/673	32/18670	0.027033	0.118498	0.090917	CRABP2/A	4	geneontol	0.005944

GO:00488	regulation	4/673	32/18670	0.027033	0.118498	0.090917	SEMA3G/S	4	geneontol	0.005944
GO:00164	somatic di	6/673	64/18670	0.027468	0.120177	0.092205	PTPRC/IL1	6	geneontol	0.008915
GO:00300	lamellipod	6/673	64/18670	0.027468	0.120177	0.092205	EPHA2/S1I	6	geneontol	0.008915
GO:00310	regenerati	13/673	198/18670	0.027544	0.120398	0.092374	TGFBR3/IL	13	geneontol	0.019316
GO:00512	protein po	17/673	283/18670	0.028107	0.122744	0.094175	ADD2/VILL	17	geneontol	0.02526
GO:00462	nitric oxide	7/673	82/18670	0.028367	0.123744	0.094942	IL10/CYP1	7	geneontol	0.010401
GO:00302	keratinocy	18/673	305/18670	0.02839	0.123744	0.094942	EPHA2/SFI	18	geneontol	0.026746
GO:00080	neuron rec	5/673	48/18670	0.028659	0.124685	0.095664	NEXN/IGSI	5	geneontol	0.007429
GO:00603	face devel	5/673	48/18670	0.028659	0.124685	0.095664	PDGFRA/C	5	geneontol	0.007429
GO:00900	regulation	15/673	241/18670	0.028729	0.12487	0.095806	TGFBR3/N	15	geneontol	0.022288
GO:00725	reactive ox	17/673	284/18670	0.028939	0.125665	0.096416	IL10/XDH/	17	geneontol	0.02526
GO:00331	regulation	10/673	139/18670	0.029057	0.126058	0.096717	PLCL1/HG	10	geneontol	0.014859
GO:00190	viral life cy	19/673	328/18670	0.029293	0.126298	0.096902	EPHA2/SL/	19	geneontol	0.028232
GO:00060	aminoglyc	6/673	65/18670	0.029374	0.126298	0.096902	OGN/OMD	6	geneontol	0.008915
GO:00511	negative re	6/673	65/18670	0.029374	0.126298	0.096902	PI16/SHH/	6	geneontol	0.008915
GO:00027	positive re	3/673	19/18670	0.029385	0.126298	0.096902	NLRP3/IL1	3	geneontol	0.004458
GO:00034	growth pla	3/673	19/18670	0.029385	0.126298	0.096902	VIT/COL21	3	geneontol	0.004458
GO:00426	muscle cel	3/673	19/18670	0.029385	0.126298	0.096902	FGF10/DKI	3	geneontol	0.004458
GO:00465	negative re	3/673	19/18670	0.029385	0.126298	0.096902	PTX3/TRIM	3	geneontol	0.004458
GO:00515	keratinocy	3/673	19/18670	0.029385	0.126298	0.096902	FGF10/FGF	3	geneontol	0.004458
GO:00604	negative re	3/673	19/18670	0.029385	0.126298	0.096902	PTGER3/N	3	geneontol	0.004458
GO:00720	kidney me	3/673	19/18670	0.029385	0.126298	0.096902	OSR1/SHH	3	geneontol	0.004458
GO:00508	defense re	8/673	101/18670	0.029487	0.126618	0.097147	EPHA2/NL	8	geneontol	0.011887
GO:00423	keratan su	4/673	33/18670	0.029911	0.128203	0.098363	B3GNT7/C	4	geneontol	0.005944
GO:00606	mammary	4/673	33/18670	0.029911	0.128203	0.098363	EPHA2/FG	4	geneontol	0.005944
GO:00425	regulation	7/673	83/18670	0.030055	0.12858	0.098653	TNFSF18/I	7	geneontol	0.010401
GO:00481	regulation	7/673	83/18670	0.030055	0.12858	0.098653	S100A6/DI	7	geneontol	0.010401
GO:00713	cellular res	12/673	180/18670	0.030156	0.128896	0.098895	PPARG/IRF	12	geneontol	0.017831
GO:00447	multi-mult	14/673	222/18670	0.030733	0.13124	0.100693	SLC2A1/PT	14	geneontol	0.020802
GO:00030	regulation	5/673	49/18670	0.031004	0.13215	0.101392	AGTR1/AV	5	geneontol	0.007429
GO:20012	positive re	5/673	49/18670	0.031004	0.13215	0.101392	PTPRC/MA	5	geneontol	0.007429
GO:00326	interleukin	11/673	161/18670	0.03167	0.134867	0.103476	SLAMF1/IL	11	geneontol	0.016345
GO:00481	fibroblast	7/673	84/18670	0.031809	0.135212	0.10374	S100A6/DI	7	geneontol	0.010401
GO:00508	regulation	7/673	84/18670	0.031809	0.135212	0.10374	SELP/PLEK	7	geneontol	0.010401
GO:00190	viral genor	9/673	122/18670	0.032438	0.137681	0.105635	CD28/CXC	9	geneontol	0.013373
GO:00485	embryonic	17/673	288/18670	0.03245	0.137681	0.105635	EPHA2/EFI	17	geneontol	0.02526
GO:19035	positive re	22/673	399/18670	0.032575	0.138084	0.105945	CD2/IL10/I	22	geneontol	0.032689
GO:00072	integrin-m	8/673	103/18670	0.032624	0.138168	0.106009	PLEK/ITGA	8	geneontol	0.011887
GO:00450	regulatory	4/673	34/18670	0.032964	0.139099	0.106723	PLA2G2D/	4	geneontol	0.005944
GO:00718	cellular res	4/673	34/18670	0.032964	0.139099	0.106723	PDE4B/NR	4	geneontol	0.005944
GO:00718	cellular res	4/673	34/18670	0.032964	0.139099	0.106723	PDE4B/NR	4	geneontol	0.005944
GO:00970	energy hor	4/673	34/18670	0.032964	0.139099	0.106723	LEPR/NMU	4	geneontol	0.005944
GO:00108	lipid locali	22/673	400/18670	0.033353	0.1394	0.106954	PLA2G2D/	22	geneontol	0.032689
GO:00308	positive re	6/673	67/18670	0.033441	0.1394	0.106954	SFN/ZEB2/	6	geneontol	0.008915
GO:00604	lung morp	5/673	50/18670	0.033464	0.1394	0.106954	FGF10/SHI	5	geneontol	0.007429
GO:00343	primary al	7/673	85/18670	0.033631	0.1394	0.106954	CYP1B1/SU	7	geneontol	0.010401
GO:19039	negative re	7/673	85/18670	0.033631	0.1394	0.106954	PTX3/TRIM	7	geneontol	0.010401
GO:20010	reactive ni	7/673	85/18670	0.033631	0.1394	0.106954	IL10/CYP1	7	geneontol	0.010401
GO:00020	sprouting	12/673	183/18670	0.033637	0.1394	0.106954	EPHA2/IL1	12	geneontol	0.017831
GO:00023	cytokine se	3/673	20/18670	0.033668	0.1394	0.106954	IL10/NLRP	3	geneontol	0.004458
GO:00028	positive re	3/673	20/18670	0.033668	0.1394	0.106954	IL1R1/CCR	3	geneontol	0.004458
GO:00100	response t	3/673	20/18670	0.033668	0.1394	0.106954	FIBIN/LRRI	3	geneontol	0.004458
GO:00230	signal tran	3/673	20/18670	0.033668	0.1394	0.106954	PDGFRA/S	3	geneontol	0.004458
GO:00312	negative re	3/673	20/18670	0.033668	0.1394	0.106954	CCR2/P2R	3	geneontol	0.004458
GO:00341	negative re	3/673	20/18670	0.033668	0.1394	0.106954	CLDN18/S	3	geneontol	0.004458
GO:00360	fucosylatic	3/673	20/18670	0.033668	0.1394	0.106954	LFNG/FUT	3	geneontol	0.004458
GO:00425	positive re	3/673	20/18670	0.033668	0.1394	0.106954	CCR2/THB	3	geneontol	0.004458
GO:00487	smooth m	3/673	20/18670	0.033668	0.1394	0.106954	OSR1/IHH	3	geneontol	0.004458
GO:00517	nitric-oxid	3/673	20/18670	0.033668	0.1394	0.106954	KDR/LRRK	3	geneontol	0.004458
GO:00517	regulation	3/673	20/18670	0.033668	0.1394	0.106954	KDR/LRRK	3	geneontol	0.004458
GO:00602	definitive	3/673	20/18670	0.033668	0.1394	0.106954	TAL1/TGFE	3	geneontol	0.004458
GO:00901	chemokine	3/673	20/18670	0.033668	0.1394	0.106954	CSF1R/LPL	3	geneontol	0.004458
GO:19055	positive re	3/673	20/18670	0.033668	0.1394	0.106954	P2RY12/CI	3	geneontol	0.004458
GO:00016	osteoblast	14/673	225/18670	0.03387	0.140111	0.107499	EPHA2/DD	14	geneontol	0.020802
GO:00356	multicellul	13/673	204/18670	0.033918	0.140186	0.107557	S1PR1/NPI	13	geneontol	0.019316
GO:00313	positive re	16/673	268/18670	0.03406	0.140612	0.107884	TAL1/PLEK	16	geneontol	0.023774
GO:00069	humoral ir	20/673	356/18670	0.034082	0.140612	0.107884	S100A12/F	20	geneontol	0.029718
GO:00991	postsynap	11/673	163/18670	0.034206	0.140997	0.10818	EFNA1/DC	11	geneontol	0.016345
GO:00192	sensory pe	8/673	104/18670	0.034275	0.141157	0.108302	IL10/CCR2	8	geneontol	0.011887
GO:00488	dendrite m	10/673	143/18670	0.034366	0.141404	0.108491	EFNA1/DC	10	geneontol	0.014859
GO:00700	glycosylati	16/673	269/18670	0.03506	0.144132	0.110585	MAN1C1/I	16	geneontol	0.023774
GO:19012	regulation	18/673	313/18670	0.035314	0.145049	0.111288	NGF/IL10/	18	geneontol	0.026746
GO:00072	tyrosine pl	7/673	86/18670	0.035521	0.145404	0.11156	TNFSF18/I	7	geneontol	0.010401

GO:00086	extrinsic ap	7/673	86/18670	0.035521	0.145404	0.11156	NGF/MAL/	7	geneontol	0.010401
GO:00480	ephrin rec	7/673	86/18670	0.035521	0.145404	0.11156	EPHA2/EFI	7	geneontol	0.010401
GO:00358	modificatio	11/673	164/18670	0.035526	0.145404	0.11156	S100A12/F	11	geneontol	0.016345
GO:00508	multicellul	6/673	68/18670	0.035605	0.145597	0.111709	SFN/CLDN	6	geneontol	0.008915
GO:00193	arachidoni	5/673	51/18670	0.036043	0.146843	0.112665	CYP1B1/PT	5	geneontol	0.007429
GO:00481	positive re	5/673	51/18670	0.036043	0.146843	0.112665	S100A6/DI	5	geneontol	0.007429
GO:00482	positive re	5/673	51/18670	0.036043	0.146843	0.112665	SELE/SFRP	5	geneontol	0.007429
GO:00486	regulation	5/673	51/18670	0.036043	0.146843	0.112665	SHH/SOX1	5	geneontol	0.007429
GO:00018	tissue hom	14/673	227/18670	0.036086	0.146843	0.112665	S1PR1/IHH	14	geneontol	0.020802
GO:00075	blood coa	19/673	336/18670	0.0361	0.146843	0.112665	SELP/C4BF	19	geneontol	0.028232
GO:00327	positive re	4/673	35/18670	0.036192	0.14696	0.112754	CD36/CCL	4	geneontol	0.005944
GO:00988	regulation	4/673	35/18670	0.036192	0.14696	0.112754	NPR1/AGT	4	geneontol	0.005944
GO:00430	camera-ty	18/673	314/18670	0.03626	0.147106	0.112867	EPHA2/CY	18	geneontol	0.026746
GO:00310	stress-acti	18/673	315/18670	0.037224	0.150886	0.115767	SLAMF1/X	18	geneontol	0.026746
GO:00109	negative re	15/673	250/18670	0.037852	0.152914	0.117322	SFN/NGF/	15	geneontol	0.022288
GO:00159	long-chain	6/673	69/18670	0.037857	0.152914	0.117322	PLA2G2D/	6	geneontol	0.008915
GO:00713	cellular res	6/673	69/18670	0.037857	0.152914	0.117322	GJB3/OSR	6	geneontol	0.008915
GO:20002	positive re	6/673	69/18670	0.037857	0.152914	0.117322	LFNG/SHH	6	geneontol	0.008915
GO:00060	ethanol m	3/673	21/18670	0.038256	0.153989	0.118147	SULT1C4/	3	geneontol	0.004458
GO:00361	phosphati	3/673	21/18670	0.038256	0.153989	0.118147	PLA2G2D/	3	geneontol	0.004458
GO:00902	positive re	3/673	21/18670	0.038256	0.153989	0.118147	CXCL12/L	3	geneontol	0.004458
GO:19033	negative re	3/673	21/18670	0.038256	0.153989	0.118147	FAM107A/	3	geneontol	0.004458
GO:00456	regulation	9/673	126/18670	0.038685	0.155536	0.119335	DDR2/TWI	9	geneontol	0.013373
GO:00164	somatic re	5/673	52/18670	0.038741	0.155536	0.119335	PTPRC/IL1	5	geneontol	0.007429
GO:19000	regulation	5/673	52/18670	0.038741	0.155536	0.119335	TACSTD2/	5	geneontol	0.007429
GO:00714	cellular res	19/673	339/18670	0.038932	0.156166	0.119818	SLC2A1/PT	19	geneontol	0.028232
GO:00016	eye develo	20/673	362/18670	0.039471	0.158191	0.121372	EPHA2/CY	20	geneontol	0.029718
GO:19004	regulation	7/673	88/18670	0.039508	0.158204	0.121381	IL10/CD36	7	geneontol	0.010401
GO:20005	positive re	4/673	36/18670	0.039596	0.158419	0.121547	NLRP3/CC	4	geneontol	0.005944
GO:00519	regulation	6/673	70/18670	0.040197	0.160686	0.123285	GRIK3/CCF	6	geneontol	0.008915
GO:00435	negative re	10/673	147/18670	0.040317	0.161025	0.123546	NGF/CHL1	10	geneontol	0.014859
GO:00510	regulation	23/673	432/18670	0.041092	0.163978	0.125811	S100A12/D	23	geneontol	0.034175
GO:00346	cellular res	11/673	168/18670	0.041166	0.164132	0.12593	IL10/CYP1	11	geneontol	0.016345
GO:20000	regulation	15/673	253/18670	0.041312	0.164571	0.126266	TACSTD2/	15	geneontol	0.022288
GO:00060	alcohol me	20/673	364/18670	0.041399	0.164777	0.126424	LEPR/CYP1	20	geneontol	0.029718
GO:00020	blood vess	5/673	53/18670	0.041559	0.16499	0.126588	EPHA2/AG	5	geneontol	0.007429
GO:00327	negative re	5/673	53/18670	0.041559	0.16499	0.126588	SLAMF1/IL	5	geneontol	0.007429
GO:00507	negative re	5/673	53/18670	0.041559	0.16499	0.126588	PTPRC/SFF	5	geneontol	0.007429
GO:00516	localizatio	10/673	148/18670	0.041909	0.166006	0.127367	CD2/PTPR	10	geneontol	0.014859
GO:00075	muscle org	22/673	410/18670	0.041919	0.166006	0.127367	TGFBR3/S	22	geneontol	0.032689
GO:00508	coagulatio	19/673	342/18670	0.041923	0.166006	0.127367	SELP/C4BF	19	geneontol	0.028232
GO:00003	response t	14/673	232/18670	0.042078	0.16648	0.127731	IL10/CYP1	14	geneontol	0.020802
GO:00068	lipid trans	20/673	365/18670	0.042388	0.167562	0.128561	PLA2G2D/	20	geneontol	0.029718
GO:19035	negative re	13/673	211/18670	0.04262	0.167671	0.128645	CD84/IL10	13	geneontol	0.019316
GO:00507	negative re	11/673	169/18670	0.042667	0.167671	0.128645	PTPRC/IL1	11	geneontol	0.016345
GO:00017	organ indu	3/673	22/18670	0.043145	0.167671	0.128645	FGF10/FGF	3	geneontol	0.004458
GO:00065	melanin m	3/673	22/18670	0.043145	0.167671	0.128645	ZEB2/CDH	3	geneontol	0.004458
GO:00108	positive re	3/673	22/18670	0.043145	0.167671	0.128645	CD36/LPL/	3	geneontol	0.004458
GO:00198	antigen pr	3/673	22/18670	0.043145	0.167671	0.128645	CD1C/CD1	3	geneontol	0.004458
GO:00326	regulation	3/673	22/18670	0.043145	0.167671	0.128645	NLRP3/IRF	3	geneontol	0.004458
GO:00350	negative re	3/673	22/18670	0.043145	0.167671	0.128645	MET/DLC1	3	geneontol	0.004458
GO:00460	cAMP met	3/673	22/18670	0.043145	0.167671	0.128645	EPHA2/PD	3	geneontol	0.004458
GO:00603	regulation	3/673	22/18670	0.043145	0.167671	0.128645	TGFB3/CIL	3	geneontol	0.004458
GO:00604	lung epith	3/673	22/18670	0.043145	0.167671	0.128645	FGF10/SPD	3	geneontol	0.004458
GO:00605	intestinal e	3/673	22/18670	0.043145	0.167671	0.128645	SPDEF/KLF	3	geneontol	0.004458
GO:00714	cellular res	3/673	22/18670	0.043145	0.167671	0.128645	PPARG/CD	3	geneontol	0.004458
GO:00903	positive re	3/673	22/18670	0.043145	0.167671	0.128645	SDR16C5/	3	geneontol	0.004458
GO:00140	Schwann c	4/673	37/18670	0.043176	0.167671	0.128645	LAMA2/AD	4	geneontol	0.005944
GO:00310	embryonic	4/673	37/18670	0.043176	0.167671	0.128645	IHH/FGF1	4	geneontol	0.005944
GO:00329	secretion b	4/673	37/18670	0.043176	0.167671	0.128645	FGF10/AG	4	geneontol	0.005944
GO:00430	T cell hom	4/673	37/18670	0.043176	0.167671	0.128645	SIT1/NCK	4	geneontol	0.005944
GO:00457	positive re	4/673	37/18670	0.043176	0.167671	0.128645	NMU/CNR	4	geneontol	0.005944
GO:00459	positive re	4/673	37/18670	0.043176	0.167671	0.128645	SLC45A3/F	4	geneontol	0.005944
GO:19053	semi-luna	4/673	37/18670	0.043176	0.167671	0.128645	EFNA1/SLI	4	geneontol	0.005944
GO:00163	dendrite d	14/673	233/18670	0.043357	0.168175	0.129032	EFNA1/IGS	14	geneontol	0.020802
GO:00720	nephron e	8/673	109/18670	0.043379	0.168175	0.129032	TACSTD2/	8	geneontol	0.011887
GO:00510	positive re	10/673	149/18670	0.043543	0.168529	0.129303	S100A12/T	10	geneontol	0.014859
GO:19039	regulation	10/673	149/18670	0.043543	0.168529	0.129303	CD28/PTX	10	geneontol	0.014859
GO:00027	positive re	5/673	54/18670	0.044498	0.172081	0.132028	NLRP3/IL1	5	geneontol	0.007429
GO:00192	transmissi	6/673	72/18670	0.045148	0.174305	0.133735	S1PR1/SCN	6	geneontol	0.008915
GO:00518	regulation	6/673	72/18670	0.045148	0.174305	0.133735	KDR/LRRK	6	geneontol	0.008915
GO:00163	morphoge	10/673	150/18670	0.04522	0.174436	0.133835	OSR1/ZEB	10	geneontol	0.014859
GO:00032	cardiac ch	11/673	171/18670	0.045783	0.176459	0.135388	TGFBR3/K	11	geneontol	0.016345

GO:00718(	response t	4/673	38/18670	0.046933	0.17956	0.137767	PDE4B/NR	4	geneontol	0.005944
GO:00718(	response t	4/673	38/18670	0.046933	0.17956	0.137767	PDE4B/NR	4	geneontol	0.005944
GO:00442	sulfur com	5/673	55/18670	0.047558	0.17956	0.137767	OGN/OMD	5	geneontol	0.007429
GO:00302	myofibril a	6/673	73/18670	0.04776	0.17956	0.137767	CAPN9/PD	6	geneontol	0.008915
GO:00456	regulation	6/673	73/18670	0.04776	0.17956	0.137767	SLC45A3/F	6	geneontol	0.008915
GO:00027	negative re	2/673	10/18670	0.048192	0.17956	0.137767	C4BPB/CR	2	geneontol	0.002972
GO:00028	negative re	2/673	10/18670	0.048192	0.17956	0.137767	C4BPB/CR	2	geneontol	0.002972
GO:00069	substrate-	2/673	10/18670	0.048192	0.17956	0.137767	P2RY12/M	2	geneontol	0.002972
GO:00069	hypotonic	2/673	10/18670	0.048192	0.17956	0.137767	AQP5/TSP	2	geneontol	0.002972
GO:00074	foregut m	2/673	10/18670	0.048192	0.17956	0.137767	SHH/SOX1	2	geneontol	0.002972
GO:00107	regulation	2/673	10/18670	0.048192	0.17956	0.137767	GUCY1A2/	2	geneontol	0.002972
GO:00109	negative re	2/673	10/18670	0.048192	0.17956	0.137767	MUC1/TFA	2	geneontol	0.002972
GO:00303	poly-N-ac	2/673	10/18670	0.048192	0.17956	0.137767	B3GNT7/B	2	geneontol	0.002972
GO:00357(	endothelia	2/673	10/18670	0.048192	0.17956	0.137767	CXCL13/F(	2	geneontol	0.002972
GO:00480	regulation	2/673	10/18670	0.048192	0.17956	0.137767	ZEB2/CDH	2	geneontol	0.002972
GO:00483(	positive re	2/673	10/18670	0.048192	0.17956	0.137767	PTPRC/CD	2	geneontol	0.002972
GO:00485	notochord	2/673	10/18670	0.048192	0.17956	0.137767	EPHA2/EFI	2	geneontol	0.002972
GO:00508(	positive re	2/673	10/18670	0.048192	0.17956	0.137767	STAP1/PRI	2	geneontol	0.002972
GO:00515	response t	2/673	10/18670	0.048192	0.17956	0.137767	BCHE/FOL	2	geneontol	0.002972
GO:00602	regulation	2/673	10/18670	0.048192	0.17956	0.137767	PDE3A/IGF	2	geneontol	0.002972
GO:00605	prostatic b	2/673	10/18670	0.048192	0.17956	0.137767	FGF10/SHI	2	geneontol	0.002972
GO:00607(	regulation	2/673	10/18670	0.048192	0.17956	0.137767	SHH/SERP	2	geneontol	0.002972
GO:00609	cardiac mu	2/673	10/18670	0.048192	0.17956	0.137767	DKK1/WT1	2	geneontol	0.002972
GO:00612	retina vasc	2/673	10/18670	0.048192	0.17956	0.137767	CYP1B1/AI	2	geneontol	0.002972
GO:00700	regulation	2/673	10/18670	0.048192	0.17956	0.137767	SLIT2/SLIT	2	geneontol	0.002972
GO:00701	regulation	2/673	10/18670	0.048192	0.17956	0.137767	TFAP2A/AS	2	geneontol	0.002972
GO:00703	response t	2/673	10/18670	0.048192	0.17956	0.137767	CD36/LBP	2	geneontol	0.002972
GO:00709	neutrophil	2/673	10/18670	0.048192	0.17956	0.137767	DNASE1L3	2	geneontol	0.002972
GO:00712	cellular res	2/673	10/18670	0.048192	0.17956	0.137767	CD36/LBP	2	geneontol	0.002972
GO:00716	smooth m	2/673	10/18670	0.048192	0.17956	0.137767	SLIT2/LPAI	2	geneontol	0.002972
GO:19017	regulation	2/673	10/18670	0.048192	0.17956	0.137767	LRRK2/PRI	2	geneontol	0.002972
GO:19058	regulation	2/673	10/18670	0.048192	0.17956	0.137767	PDE3A/IGF	2	geneontol	0.002972
GO:20005	regulation	2/673	10/18670	0.048192	0.17956	0.137767	CXCL13/F(	2	geneontol	0.002972
GO:00074	axonal fas	3/673	23/18670	0.048329	0.17956	0.137767	CNTN4/EP	3	geneontol	0.004458
GO:00075	sex determ	3/673	23/18670	0.048329	0.17956	0.137767	WT1/DHH	3	geneontol	0.004458
GO:00095	detection (	3/673	23/18670	0.048329	0.17956	0.137767	NLRP3/CR	3	geneontol	0.004458
GO:00163	calcium-in	3/673	23/18670	0.048329	0.17956	0.137767	CLDN18/C	3	geneontol	0.004458
GO:00451(	intermedia	3/673	23/18670	0.048329	0.17956	0.137767	SHH/KRT1	3	geneontol	0.004458
GO:00513	negative re	3/673	23/18670	0.048329	0.17956	0.137767	CCR2/P2R	3	geneontol	0.004458
GO:00604	lung cell d	3/673	23/18670	0.048329	0.17956	0.137767	FGF10/SPD	3	geneontol	0.004458
GO:00721	metanephr	3/673	23/18670	0.048329	0.17956	0.137767	OSR1/CXC	3	geneontol	0.004458
GO:00901	regulation	3/673	23/18670	0.048329	0.17956	0.137767	KDR/LRRK	3	geneontol	0.004458
GO:01060	neuron pro	3/673	23/18670	0.048329	0.17956	0.137767	CNTN4/EP	3	geneontol	0.004458
GO:19018(	negative re	3/673	23/18670	0.048329	0.17956	0.137767	FAM107A/	3	geneontol	0.004458
GO:20001	negative re	7/673	92/18670	0.048333	0.17956	0.137767	SFN/NGF/	7	geneontol	0.010401
GO:00709	neuron de	19/673	348/18670	0.048399	0.179662	0.137845	NGF/IL10/	19	geneontol	0.028232
GO:00162(	regulation	10/673	152/18670	0.048704	0.180648	0.138602	TGFBR3/K(	10	geneontol	0.014859
GO:00030	vascular p	11/673	173/18670	0.04905	0.181641	0.139363	NPR1/CXC	11	geneontol	0.016345
GO:00550(	striated mu	11/673	173/18670	0.04905	0.181641	0.139363	CAPN9/PD	11	geneontol	0.016345
GO:00031	endotheliu	9/673	132/18670	0.049514	0.18273	0.140199	S1PR1/XDI	9	geneontol	0.013373
GO:00086	hexose tra	8/673	112/18670	0.049541	0.18273	0.140199	SLC2A1/SL	8	geneontol	0.011887
GO:00518	modificatio	8/673	112/18670	0.049541	0.18273	0.140199	PTX3/CSF1	8	geneontol	0.011887
GO:19035	mucopolys	8/673	112/18670	0.049541	0.18273	0.140199	B3GNT7/H	8	geneontol	0.011887
GO:19038	negative re	8/673	112/18670	0.049541	0.18273	0.140199	CLDN18/C	8	geneontol	0.011887
GO:00620	collagen-c	57/718	406/19717	1.57E-18	6.10E-16	5.56E-16	TINAGL1/F	57	geneontol	0.079387
GO:00098	external si	53/718	393/19717	1.53E-16	2.98E-14	2.71E-14	LEPR/TGFE	53	geneontol	0.073816
GO:00312	anchored (	23/718	170/19717	6.00E-08	7.79E-06	7.10E-06	NEGR1/CD	23	geneontol	0.032033
GO:00451	membrane	31/718	315/19717	5.61E-07	4.67E-05	4.26E-05	SLC2A1/S1	31	geneontol	0.043175
GO:00988	membrane	31/718	316/19717	6.01E-07	4.67E-05	4.26E-05	SLC2A1/S1	31	geneontol	0.043175
GO:00985	membrane	31/718	328/19717	1.34E-06	8.67E-05	7.91E-05	SLC2A1/S1	31	geneontol	0.043175
GO:00059	cell-cell ju	36/718	459/19717	1.43E-05	0.000794	0.000724	EPHA2/GJI	36	geneontol	0.050139
GO:00056	interstitial	5/718	12/19717	4.04E-05	0.001965	0.001792	VIT/ABI3BI	5	geneontol	0.006964
GO:00466	anchored (	10/718	59/19717	4.81E-05	0.002081	0.001897	CD2/EFNA	10	geneontol	0.013928
GO:00056(	basement	12/718	95/19717	0.00017	0.006627	0.006043	HMCN1/L/	12	geneontol	0.016713
GO:00017	immunolo	7/718	36/19717	0.000274	0.009678	0.008825	ZAP70/CD	7	geneontol	0.009749
GO:00057	Golgi lume	12/718	102/19717	0.000334	0.010831	0.009877	NGF/MUC	12	geneontol	0.016713
GO:00059(	microvillus	10/718	83/19717	0.000852	0.025482	0.023238	CD302/S1(	10	geneontol	0.013928
GO:00347(	sodium ch	5/718	23/19717	0.001231	0.033303	0.03037	GRIK3/SCN	5	geneontol	0.006964
GO:00099	basal plas	6/718	34/19717	0.001284	0.033303	0.03037	TACSTD2/	6	geneontol	0.008357
GO:00444	extracellul	7/718	51/19717	0.002368	0.05757	0.052499	LAMB3/EL	7	geneontol	0.009749
GO:00163	apicolatera	4/718	18/19717	0.003548	0.081195	0.074043	CLDN4/MI	4	geneontol	0.005571
GO:00971	tetraspani	3/718	10/19717	0.004763	0.102941	0.093874	TSPAN15/	3	geneontol	0.004178
GO:00163	basolatera	16/718	217/19717	0.006059	0.124048	0.113122	SLC2A1/T/	16	geneontol	0.022284

GO:00701(	tight juncti	11/718	128/19717	0.00713	0.133654	0.121882	EPHA2/TA	11	geneontol	0.01532
GO:00986:	protein co	5/718	34/19717	0.007319	0.133654	0.121882	ITGA4/CD2	5	geneontol	0.006964
GO:00426:	mast cell g	4/718	22/19717	0.007559	0.133654	0.121882	CXCR2/PIK	4	geneontol	0.005571
GO:00451	basal part	6/718	51/19717	0.010213	0.168043	0.153242	TACSTD2/	6	geneontol	0.008357
GO:00312:	intrinsic cc	4/718	24/19717	0.010368	0.168043	0.153242	GPIHBP1/F	4	geneontol	0.005571
GO:00163:	apical plas	20/718	318/19717	0.012943	0.193942	0.176859	SLC2A1/M	20	geneontol	0.027855
GO:00015	voltage-ga	3/718	14/19717	0.012963	0.193942	0.176859	SCN7A/SC	3	geneontol	0.004178
GO:00310!	platelet alp	8/718	91/19717	0.017843	0.25083	0.228737	SELP/MMF	8	geneontol	0.011142
GO:00448!	plasma me	9/718	109/19717	0.018055	0.25083	0.228737	SLC2A1/SE	9	geneontol	0.012535
GO:00325(	ruffle mem	8/718	94/19717	0.021281	0.285457	0.260314	EPHA2/EP!	8	geneontol	0.011142
GO:00017:	ruffle	12/718	172/19717	0.023771	0.296404	0.270297	EPHA2/EP!	12	geneontol	0.016713
GO:00347(	potassium	8/718	96/19717	0.023818	0.296404	0.270297	GRIK3/KCN	8	geneontol	0.011142
GO:00451	apical part	22/718	384/19717	0.024898	0.296404	0.270297	SLC2A1/M	22	geneontol	0.030641
GO:00083(	integrin cc	4/718	31/19717	0.025145	0.296404	0.270297	ITGA4/ITG	4	geneontol	0.005571
GO:00312!	cell project	20/718	345/19717	0.02821	0.308812	0.281612	EPHA2/EP!	20	geneontol	0.027855
GO:00436	axon termi	9/718	119/19717	0.029891	0.308812	0.281612	GRIK3/KCN	9	geneontol	0.012535
GO:00160	dystrophin	3/718	19/19717	0.030167	0.308812	0.281612	SGCD/KRT	3	geneontol	0.004178
GO:00313(	anchored (	3/718	19/19717	0.030167	0.308812	0.281612	GPIHBP1/F	3	geneontol	0.004178
GO:00906(	glycoprote	3/718	19/19717	0.030167	0.308812	0.281612	SGCD/KRT	3	geneontol	0.004178
GO:00347(	cation cha	14/718	220/19717	0.031009	0.309294	0.282051	GRIK3/PDE	14	geneontol	0.019499
GO:01500:	distal axor	17/718	285/19717	0.032411	0.315194	0.287431	GRIK3/KCN	17	geneontol	0.023677
GO:00310!	platelet alp	6/718	67/19717	0.0349	0.331123	0.301958	MMRN1/F	6	geneontol	0.008357
GO:00059:	bicellular t	9/718	123/19717	0.035871	0.332235	0.302971	TACSTD2/	9	geneontol	0.012535
GO:00431!	terminal bo	5/718	51/19717	0.037416	0.338486	0.308672	GRIK3/NM	5	geneontol	0.006964
GO:00059:	connexin c	3/718	21/19717	0.039253	0.339401	0.309506	GJB5/GJB4	3	geneontol	0.004178
GO:00198!	extrinsic cc	11/718	165/19717	0.039262	0.339401	0.309506	S100A10/S	11	geneontol	0.01532
GO:00198!	extrinsic cc	17/718	295/19717	0.042654	0.356863	0.325431	S100A10/S	17	geneontol	0.023677
GO:00315:	brush bord	5/718	53/19717	0.043117	0.356863	0.325431	KCNK1/CD	5	geneontol	0.006964
GO:00165:	sarcoplasn	6/718	71/19717	0.044434	0.360101	0.328383	XDH/HK2/	6	geneontol	0.008357
GO:00015:	microfibril	2/718	10/19717	0.049088	0.372398	0.339598	MFAP4/AD	2	geneontol	0.002786
GO:00442:	juxtaparan	2/718	10/19717	0.049088	0.372398	0.339598	KCNAB1/D	2	geneontol	0.002786
GO:00315:	microvillus	3/718	23/19717	0.049562	0.372398	0.339598	S100P/CDI	3	geneontol	0.004178
GO:00347(	ion channe	17/718	301/19717	0.049817	0.372398	0.339598	GRIK3/PDE	17	geneontol	0.023677
GO:00055:	glycosamir	36/672	229/17697	3.86E-13	2.71E-10	2.35E-10	PLA2G2D/	36	geneontol	0.053571
GO:00082(	heparin bi	30/672	169/17697	1.48E-12	5.20E-10	4.49E-10	PLA2G2D/	30	geneontol	0.044643
GO:00052(	extracellula	29/672	163/17697	3.30E-12	7.74E-10	6.69E-10	TINAGL1/F	29	geneontol	0.043155
GO:00048!	cytokine re	22/672	96/17697	7.57E-12	1.33E-09	1.15E-09	LEPR/IL1R	22	geneontol	0.032738
GO:00016:	G protein-	12/672	26/17697	4.83E-11	5.66E-09	4.89E-09	CXCR2/GP	12	geneontol	0.017857
GO:00049!	chemokine	12/672	26/17697	4.83E-11	5.66E-09	4.89E-09	CXCR2/GP	12	geneontol	0.017857
GO:00199!	C-C chem	11/672	24/17697	3.47E-10	3.49E-08	3.01E-08	ACKR1/CX	11	geneontol	0.016369
GO:00199!	cytokine b	23/672	128/17697	4.74E-10	4.17E-08	3.60E-08	LEPR/TGFE	23	geneontol	0.034226
GO:00199!	chemokine	12/672	32/17697	9.16E-10	7.16E-08	6.18E-08	ACKR1/ITG	12	geneontol	0.017857
GO:00047	protein tyr	23/672	134/17697	1.21E-09	8.53E-08	7.37E-08	EPHA2/EFI	23	geneontol	0.034226
GO:00164!	C-C chem	10/672	23/17697	4.26E-09	2.73E-07	2.35E-07	CXCR2/CC	10	geneontol	0.014881
GO:19016(	sulfur com	31/672	250/17697	6.92E-09	4.04E-07	3.49E-07	PLA2G2D/	31	geneontol	0.046131
GO:00047	transmeml	15/672	62/17697	7.46E-09	4.04E-07	3.49E-07	EPHA2/EFI	15	geneontol	0.022321
GO:00085:	G protein-	22/672	146/17697	3.30E-08	1.66E-06	1.43E-06	NPR1/CAL	22	geneontol	0.032738
GO:00191!	transmeml	16/672	79/17697	3.63E-08	1.70E-06	1.47E-06	EPHA2/TG	16	geneontol	0.02381
GO:00016!	peptide re	22/672	152/17697	6.93E-08	3.05E-06	2.63E-06	NPR1/CAL	22	geneontol	0.032738
GO:00016(	G protein-	7/672	13/17697	1.55E-07	6.06E-06	5.23E-06	GPR171/P2	7	geneontol	0.010417
GO:00450:	G protein-	7/672	13/17697	1.55E-07	6.06E-06	5.23E-06	GPR171/P2	7	geneontol	0.010417
GO:00016	purinergic	8/672	20/17697	3.48E-07	1.22E-05	1.06E-05	GPR171/P2	8	geneontol	0.011905
GO:00165(	nucleotide	8/672	20/17697	3.48E-07	1.22E-05	1.06E-05	GPR171/P2	8	geneontol	0.011905
GO:00150:	coreceptor	11/672	44/17697	5.33E-07	1.78E-05	1.54E-05	TGFBR3/CI	11	geneontol	0.016369
GO:00508:	cell adhesi	42/672	499/17697	1.27E-06	4.06E-05	3.51E-05	ARHGEF16	42	geneontol	0.0625
GO:00355(	purinergic	8/672	25/17697	2.53E-06	7.72E-05	6.67E-05	GPR171/P2	8	geneontol	0.011905
GO:00302	carbohydr	27/672	271/17697	5.02E-06	0.000147	0.000127	TINAGL1/S	27	geneontol	0.040179
GO:00423	chemokine	12/672	66/17697	6.11E-06	0.000172	0.000149	S100A14/C	12	geneontol	0.017857
GO:00051	integrin bi	17/672	132/17697	1.06E-05	0.000287	0.000248	ADAM23/I	17	geneontol	0.025298
GO:00986:	cell adhesi	11/672	59/17697	1.16E-05	0.000302	0.000261	TACSTD2/	11	geneontol	0.016369
GO:00986:	cell-cell ac	10/672	50/17697	1.51E-05	0.00038	0.000328	TACSTD2/	10	geneontol	0.014881
GO:00198:	growth fac	17/672	137/17697	1.75E-05	0.000423	0.000366	TGFBR3/IL	17	geneontol	0.025298
GO:00306	Rac guany	6/672	17/17697	2.53E-05	0.000594	0.000513	EPS8L3/DO	6	geneontol	0.008929
GO:00480:	CCR chem	9/672	43/17697	2.75E-05	0.000625	0.00054	CCR2/CXC	9	geneontol	0.013393
GO:00986	cadherin b	6/672	19/17697	5.21E-05	0.001144	0.000988	TACSTD2/	6	geneontol	0.008929
GO:00080(	chemokine	9/672	49/17697	8.19E-05	0.001744	0.001507	CXCL13/C(	9	geneontol	0.013393
GO:00480	receptor li	36/672	482/17697	9.19E-05	0.0019	0.001641	NGF/TNFS	36	geneontol	0.053571
GO:00049!	icosanoid	5/672	15/17697	0.00017	0.003416	0.002951	PTGER3/PT	5	geneontol	0.00744
GO:00050(	Rho guany	11/672	79/17697	0.000188	0.003672	0.003172	ARHGEF16	11	geneontol	0.016369
GO:00055	collagen b	10/672	67/17697	0.000204	0.003885	0.003357	PODN/DD	10	geneontol	0.014881
GO:00508	extracellula	9/672	57/17697	0.000274	0.005062	0.004373	TINAGL1/S	9	geneontol	0.013393
GO:00047	non-meml	8/672	46/17697	0.000301	0.005432	0.004693	ZAP70/ITK	8	geneontol	0.011905
GO:00049!	prostaglan	4/672	10/17697	0.00036	0.006178	0.005338	PTGER3/PT	4	geneontol	0.005952

GO:00717	lipopeptid	4/672	10/17697	0.00036	0.006178	0.005338	CD1C/CD1	4	geneontol	0.005952
GO:00422	metalloen	12/672	103/17697	0.000529	0.008581	0.007413	MMEL1/AI	12	geneontol	0.017857
GO:00508	guanyl-nu	19/672	214/17697	0.000539	0.008581	0.007413	ARHGEF16	19	geneontol	0.028274
GO:00499	prostanoic	4/672	11/17697	0.000549	0.008581	0.007413	PTGER3/P	4	geneontol	0.005952
GO:00507	RAGE rece	4/672	11/17697	0.000549	0.008581	0.007413	S100A12/S	4	geneontol	0.005952
GO:00050	scavenger	8/672	51/17697	0.000623	0.009514	0.00822	TINAGL1/F	8	geneontol	0.011905
GO:00432	laminin bir	6/672	29/17697	0.000659	0.00986	0.008519	TINAGL1/S	6	geneontol	0.008929
GO:00041	3',5'-cyclic	4/672	12/17697	0.000799	0.011705	0.010113	PDE4B/PD	4	geneontol	0.005952
GO:00381	pattern re	5/672	21/17697	0.000954	0.013682	0.011821	CD36/CLE	5	geneontol	0.00744
GO:00170	Rho GTPas	16/672	177/17697	0.00119	0.016168	0.013969	ARHGEF16	16	geneontol	0.02381
GO:00300	extracellul	5/672	22/17697	0.001196	0.016168	0.013969	PODN/PR	5	geneontol	0.00744
GO:00504	transformi	5/672	22/17697	0.001196	0.016168	0.013969	TGFBR3/N	5	geneontol	0.00744
GO:00380	cargo rece	10/672	85/17697	0.001401	0.018582	0.016055	TINAGL1/F	10	geneontol	0.014881
GO:00198	immunogl	5/672	24/17697	0.001813	0.0236	0.020389	FCER1A/M	5	geneontol	0.00744
GO:00051	cytokine a	18/672	220/17697	0.00189	0.024163	0.020876	TNFSF18/I	18	geneontol	0.026786
GO:00433	proteoglyc	6/672	36/17697	0.002158	0.027087	0.023402	PLA2G2D/	6	geneontol	0.008929
GO:00016	G protein-	21/672	280/17697	0.002427	0.029938	0.025865	S1PR1/S10	21	geneontol	0.03125
GO:00080	phosphori	10/672	94/17697	0.002987	0.036123	0.031209	PDE4B/PD	10	geneontol	0.014881
GO:00051	cytokine r	21/672	286/17697	0.00312	0.036123	0.031209	TGFBR3/N	21	geneontol	0.03125
GO:00019	fibronectir	5/672	27/17697	0.003134	0.036123	0.031209	ITGA4/CCI	5	geneontol	0.00744
GO:00468	ephrin rec	5/672	27/17697	0.003134	0.036123	0.031209	EFNA4/EF	5	geneontol	0.00744
GO:00041	calcium-d	4/672	17/17697	0.003303	0.037453	0.032358	CAPN8/CA	4	geneontol	0.005952
GO:00041	endopepti	28/672	427/17697	0.003684	0.040625	0.035098	MMEL1/C	28	geneontol	0.041667
GO:00041	3',5'-cyclic	5/672	28/17697	0.003698	0.040625	0.035098	PDE4B/PD	5	geneontol	0.00744
GO:00052	sodium ch	6/672	41/17697	0.004244	0.045898	0.039655	GRIK3/KC	6	geneontol	0.008929
GO:00041	cyclic-nucl	5/672	29/17697	0.004332	0.046138	0.039862	PDE4B/PD	5	geneontol	0.00744
GO:00483	Rac GTPas	8/672	69/17697	0.004502	0.047241	0.040815	EPS8L3/D	8	geneontol	0.011905
GO:00199	structural	3/672	10/17697	0.005355	0.055364	0.047833	MAL/MAL	3	geneontol	0.004464
GO:00050	Ras guany	12/672	137/17697	0.006015	0.061283	0.052946	ARHGEF16	12	geneontol	0.017857
GO:00083	signaling	4/672	20/17697	0.006144	0.061699	0.053306	CD36/COL	4	geneontol	0.005952
GO:00452	CXCR cher	3/672	11/17697	0.007158	0.07087	0.061229	CXCL13/C	3	geneontol	0.004464
GO:00018	compleme	4/672	21/17697	0.007365	0.070923	0.061275	CR2/CR1/	4	geneontol	0.005952
GO:00518	glycolipid	4/672	21/17697	0.007365	0.070923	0.061275	CD1C/SELI	4	geneontol	0.005952
GO:00718	lipoprotei	5/672	33/17697	0.007643	0.071643	0.061897	CD36/LPL/	5	geneontol	0.00744
GO:00718	protein-lip	5/672	33/17697	0.007643	0.071643	0.061897	CD36/LPL/	5	geneontol	0.00744
GO:00483	calcium-d	7/672	61/17697	0.008132	0.075225	0.064992	S100A11/S	7	geneontol	0.010417
GO:00336	sialic acid	3/672	12/17697	0.009277	0.083501	0.072142	SELP/SELE	3	geneontol	0.004464
GO:00082	metallope	14/672	181/17697	0.0093	0.083501	0.072142	MMEL1/AI	14	geneontol	0.020833
GO:00080	growth fac	13/672	163/17697	0.009383	0.083501	0.072142	NGF/IL10/	13	geneontol	0.019345
GO:00012	DNA-bind	27/672	439/17697	0.009791	0.086041	0.074337	ELF3/ZEB2	27	geneontol	0.040179
GO:00170	peptide h	6/672	49/17697	0.010229	0.086925	0.0751	LEPR/NPR	6	geneontol	0.008929
GO:00171	fibroblast	4/672	23/17697	0.010263	0.086925	0.0751	TGFBR3/C	4	geneontol	0.005952
GO:00466	sphingolip	4/672	23/17697	0.010263	0.086925	0.0751	S1PR1/SEL	4	geneontol	0.005952
GO:00355	signaling	8/672	80/17697	0.010874	0.091006	0.078626	CD28/TRA	8	geneontol	0.011905
GO:00167	oxidoredu	3/672	13/17697	0.011724	0.095837	0.0828	XDH/AOX	3	geneontol	0.004464
GO:00397	co-recept	3/672	13/17697	0.011724	0.095837	0.0828	DKK2/DKK	3	geneontol	0.004464
GO:00171	Wnt-prote	5/672	37/17697	0.012401	0.100207	0.086576	MST1R/SF	5	geneontol	0.00744
GO:00050	SH3/SH2 a	6/672	52/17697	0.013541	0.108149	0.093437	CD28/STA	6	geneontol	0.008929
GO:00083	steroid hy	5/672	38/17697	0.013846	0.108149	0.093437	CYP1B1/C	5	geneontol	0.00744
GO:00420	chemoattr	5/672	38/17697	0.013846	0.108149	0.093437	FGF10/GP	5	geneontol	0.00744
GO:00451	bioactive l	3/672	14/17697	0.014507	0.112068	0.096823	S1PR1/LPA	3	geneontol	0.004464
GO:00152	channel ac	27/672	456/17697	0.015472	0.116172	0.100368	GJB4/GJB3	27	geneontol	0.040179
GO:00425	hormone	9/672	102/17697	0.015506	0.116172	0.100368	LEPR/NPR	9	geneontol	0.013393
GO:00167	transferase	7/672	69/17697	0.015534	0.116172	0.100368	SULT1C2/S	7	geneontol	0.010417
GO:00044	phosphati	4/672	26/17697	0.015843	0.116252	0.100437	PLCL1/CC	4	geneontol	0.005952
GO:00228	passive tra	27/672	457/17697	0.015875	0.116252	0.100437	GJB4/GJB3	27	geneontol	0.040179
GO:00017	phosphoty	5/672	40/17697	0.017064	0.122714	0.10602	SHE/ZAP7	5	geneontol	0.00744
GO:00018	opsonin bi	3/672	15/17697	0.01763	0.122714	0.10602	CR1/PTX3/	3	geneontol	0.004464
GO:00083	mechanos	3/672	15/17697	0.01763	0.122714	0.10602	TMEM150	3	geneontol	0.004464
GO:00445	S100 prote	3/672	15/17697	0.01763	0.122714	0.10602	S100A11/S	3	geneontol	0.004464
GO:00704	oligosacch	3/672	15/17697	0.01763	0.122714	0.10602	SELP/SELL	3	geneontol	0.004464
GO:00454	chemorep	4/672	27/17697	0.018054	0.124428	0.107502	SEMA3G/S	4	geneontol	0.005952
GO:00046	phosphol	4/672	28/17697	0.020446	0.13955	0.120566	PLCL1/CC	4	geneontol	0.005952
GO:00301	low-densit	3/672	16/17697	0.021098	0.141256	0.122041	CD36/THB	3	geneontol	0.004464
GO:00433	heparan s	3/672	16/17697	0.021098	0.141256	0.122041	PLA2G2D/	3	geneontol	0.004464
GO:00016	virus recep	7/672	74/17697	0.022065	0.144969	0.125248	EPHA2/SL	7	geneontol	0.010417
GO:01040	hijacked m	7/672	74/17697	0.022065	0.144969	0.125248	EPHA2/SL	7	geneontol	0.010417
GO:00050	transmem	3/672	17/17697	0.024911	0.16126	0.139323	EPHA2/EF	3	geneontol	0.004464
GO:19907	protein tyr	8/672	93/17697	0.025003	0.16126	0.139323	ARHGEF16	8	geneontol	0.011905
GO:00306	protein bir	12/672	170/17697	0.029004	0.184106	0.159061	CD28/MLP	12	geneontol	0.017857
GO:00381	neuropilin	3/672	18/17697	0.029069	0.184106	0.159061	SEMA3G/S	3	geneontol	0.004464
GO:00708	growth fac	10/672	134/17697	0.031695	0.198944	0.171881	IL10/EFEM	10	geneontol	0.014881
GO:00050	ephrin rec	3/672	19/17697	0.03357	0.208848	0.180438	EPHA2/EF	3	geneontol	0.004464

GO:00049	monooxyg	8/672	99/17697	0.034606	0.213403	0.184373	FMO2/FM(	8	geneontol	0.011905
GO:00051	epidermal	4/672	33/17697	0.035247	0.215467	0.186157	EFEMP1/A	4	geneontol	0.005952
GO:00052	voltage-ga	3/672	20/17697	0.03841	0.232779	0.201113	SCN7A/SC	3	geneontol	0.004464
GO:00046	phospholip	8/672	102/17697	0.040234	0.24004	0.207387	PLA2G2D/	8	geneontol	0.011905
GO:00061	peptidase	14/672	219/17697	0.040291	0.24004	0.207387	NGF/MAL/	14	geneontol	0.020833
GO:00431	alcohol bir	7/672	85/17697	0.0426	0.251664	0.217429	RBP7/CRA	7	geneontol	0.010417
GO:00453	protein ph	5/672	51/17697	0.043501	0.254846	0.220179	SHE/ZAP7	5	geneontol	0.00744
GO:00304	peptidase	12/672	182/17697	0.045193	0.262569	0.22685	NGF/PI16/	12	geneontol	0.017857
GO:00170	sodium ch	4/672	36/17697	0.046459	0.262934	0.227166	SCN4B/SC	4	geneontol	0.005952
GO:00421	SH2 doma	4/672	36/17697	0.046459	0.262934	0.227166	LAX1/DLC	4	geneontol	0.005952
GO:00081	sulfotransf	5/672	52/17697	0.046689	0.262934	0.227166	SULT1C2/S	5	geneontol	0.00744
GO:00309	receptor ty	6/672	69/17697	0.046752	0.262934	0.227166	ARHGEF16	6	geneontol	0.008929
GO:00162	channel re	10/672	144/17697	0.047978	0.267689	0.231274	KCNAB1/P	10	geneontol	0.014881
