# Supplementary material for: Molecular signatures of tumor progression in pancreatic adenocarcinoma identified by energy metabolism characteristics
Source: BMC Cancer. 2022 Apr 13;22:404. doi: 10.1186/s12885-022-09487-3 (PMC9006543; doi:10.1186/s12885-022-09487-3)
Supplement: Supplementary file 11 — Additional file 11. [file 12885_2022_9487_MOESM11_ESM.pdf]

Supplementary Table 6. List of prognostic co-expression DEGs

Symbol	p.value	HR	Low 95%CI	High 95%CI
PLEKHN1	0.001689	1.030259	1.011266	1.049608
PERM1	0.002752	1.072323	1.024411	1.122476
RNF223	0.004254	1.064994	1.019994	1.11198
EPHA2	0.002386	1.00183	1.000649	1.003012
GJB5	5.44E-07	1.021244	1.01288	1.029677
GJB4	4.89E-05	1.033811	1.017352	1.050537
GJB3	3.12E-05	1.009466	1.005	1.013952
TSPAN1	0.00598	1.000963	1.000276	1.00165
S100A10	0.006937	1.000414	1.000113	1.000714
S100A11	0.000719	1.000174	1.000073	1.000274
S100A16	0.000284	1.000862	1.000396	1.001328
CRABP2	0.003877	1.000907	1.000291	1.001523
LAMB3	0.002295	1.000859	1.000307	1.001412
AFF3	0.008189	0.80321	0.682786	0.944873
NGEF	0.000806	1.028601	1.011773	1.045708
IGF2BP2	3.80E-05	1.022742	1.011857	1.033743
OCIAD2	0.003019	1.004202	1.001423	1.006989
SFTA2	9.52E-05	1.003926	1.001952	1.005903
ELMO1	0.003962	0.948924	0.915678	0.983377
CLDN4	0.008285	1.001173	1.000302	1.002045
MET	3.62E-07	1.008065	1.004951	1.01119
TMEM139	1.27E-05	1.032337	1.01769	1.047195
ESRP1	0.000938	1.009866	1.00401	1.015757
FAM83A	0.001856	1.003974	1.00147	1.006485
PSCA	0.008884	1.000311	1.000078	1.000544
MROH6	0.003636	1.008541	1.002777	1.014338
PTGES	0.00054	1.002839	1.00123	1.004451
CLIC3	0.00388	1.00329	1.001056	1.00553
ZNF488	3.80E-05	1.110379	1.056421	1.167092
DKK1	0.005405	1.004746	1.0014	1.008103
ANKRD22	0.000658	1.010136	1.004292	1.016015
COL17A1	0.000134	1.002696	1.001312	1.004083
PKP3	0.009583	1.003733	1.000907	1.006567
OVOL1	0.003128	1.038812	1.012903	1.065383
RHOD	2.41E-05	1.004566	1.002445	1.006692
MYEOV	0.001022	1.006379	1.002567	1.010204
BARX2	0.003349	1.021802	1.007185	1.036632
GPRC5A	0.002525	1.001585	1.000556	1.002614
KRT7	1.05E-05	1.000878	1.000488	1.001269
KRT8	0.001096	1.000529	1.000211	1.000846
KRT18	0.000272	1.000742	1.000342	1.001142
OASL	0.001124	1.011376	1.004516	1.018283
KLF5	0.007356	1.002329	1.000625	1.004035
LGALS3	0.003551	1.000936	1.000307	1.001565
PLEK2	0.004586	1.003675	1.001133	1.006224
IFI27	0.001812	1.001209	1.000449	1.001969
RHOV	0.000932	1.007444	1.00303	1.011877
TMC7	0.005204	1.034652	1.010222	1.059673
BEAN1	0.001635	1.075175	1.027756	1.12478
CDH3	0.003143	1.005623	1.001888	1.009372
MMP28	6.96E-06	1.008083	1.004551	1.011629
RAPGEFL1	0.002412	1.008278	1.002923	1.013661
TNS4	0.002347	1.002985	1.001061	1.004912
KRT19	0.000247	1.000226	1.000105	1.000346
KRT16	0.0016	1.000871	1.00033	1.001413
PLCD3	0.004665	1.011472	1.003511	1.019497

ITGA3	0.003222	1.002023	1.000677	1.003372
TMEM92	0.000991	1.022962	1.009232	1.036878
ITGB4	0.004183	1.001333	1.000421	1.002246
B3GNT3	0.008958	1.003113	1.000778	1.005453
C19orf33	0.003815	1.000586	1.000189	1.000983
SULT2B1	0.008633	1.016921	1.004266	1.029736
KLK10	0.000365	1.003434	1.001544	1.005327
SDCBP2	0.009548	1.003001	1.000731	1.005277
PTK6	0.003023	1.006084	1.002059	1.010126
EFNB1	0.003501	1.004218	1.001385	1.007059
CYSLTR1	0.001295	1.062613	1.024014	1.102665
