# Supplementary material for: Molecular signatures of tumor progression in pancreatic adenocarcinoma identified by energy metabolism characteristics
Source: BMC Cancer. 2022 Apr 13;22:404. doi: 10.1186/s12885-022-09487-3 (PMC9006543; doi:10.1186/s12885-022-09487-3)
Supplement: Supplementary file 12 — Additional file 12. [file 12885_2022_9487_MOESM12_ESM.pdf]

Supplementary Table 7. Significantly enriched pathways in both groups

Term	ES	NES	NP	FDR	FWER
KEGG_NEUROACTIVE_LIGAND_RECEPTOR_INTERACTION	-0.4735	-1.8204	0	0.2591	0.236
KEGG_INTESTINAL_IMMUNE_NETWORK_FOR_IGA_PRODUCTION	-0.6288	-1.6002	0.0786	0.8446	0.701
KEGG_ASTHMA	-0.6128	-1.5185	0.1105	0.9382	0.826
KEGG_GLYCINE_SERINE_AND_THREONINE_METABOLISM	-0.4617	-1.4693	0.0765	0.9412	0.88
KEGG_CALCIUM_SIGNALING_PATHWAY	-0.3679	-1.4579	0.0371	0.8007	0.892
KEGG_AUTOIMMUNE_THYROID_DISEASE	-0.5757	-1.4026	0.1694	0.8845	0.924
KEGG_PRIMARY_IMMUNODEFICIENCY	-0.5993	-1.3887	0.184	0.8128	0.932
KEGG_TYPE_I_DIABETES_MELLITUS	-0.5061	-1.3835	0.1972	0.7297	0.937
KEGG_HEMATOPOIETIC_CELL_LINEAGE	-0.455	-1.3508	0.2139	0.7573	0.95
KEGG_COMPLEMENT_AND_COAGULATION_CASCADES	-0.3908	-1.3071	0.1695	0.8302	0.971
KEGG_CELL_ADHESION_MOLECULES_CAMS	-0.3721	-1.2849	0.2035	0.8276	0.975
KEGG_TYPE_II_DIABETES_MELLITUS	-0.3685	-1.2828	0.1285	0.7654	0.975
KEGG_ALLOGRAFT_REJECTION	-0.5396	-1.2728	0.2733	0.7352	0.978
KEGG_TRYPTOPHAN_METABOLISM	-0.3611	-1.2636	0.1448	0.708	0.979
KEGG_PRIMARY_BILE_ACID_BIOSYNTHESIS	-0.4633	-1.2466	0.2162	0.7078	0.979
KEGG_VASCULAR_SMOOTH_MUSCLE_CONTRACTION	-0.3319	-1.2456	0.1777	0.6664	0.979
KEGG_GRAFT_VERSUS_HOST_DISEASE	-0.5271	-1.2376	0.3133	0.6458	0.981
KEGG_ABC_TRANSPORTERS	-0.3453	-1.212	0.219	0.6718	0.987
KEGG_MATURITY_ONSET_DIABETES_OF_THE_YOUNG	-0.4612	-1.1998	0.2974	0.6649	0.987
KEGG_CHEMOKINE_SIGNALING_PATHWAY	-0.3266	-1.1969	0.2785	0.6386	0.987
KEGG_TASTE_TRANSDUCTION	-0.3754	-1.1715	0.2481	0.6632	0.99
KEGG_CYTOKINE_CYTOKINE_RECEPTOR_INTERACTION	-0.315	-1.1277	0.3391	0.7303	0.995
KEGG_JAK_STAT_SIGNALING_PATHWAY	-0.3097	-1.0941	0.3789	0.7745	0.997
KEGG_GLYCOSPHINGOLIPID_BIOSYNTHESIS_GANGLIO_SERIES	-0.3801	-1.0337	0.4393	0.8896	0.999
KEGG_FC_EPSILON_RI_SIGNALING_PATHWAY	-0.2787	-1.0316	0.4158	0.8596	0.999
KEGG_PRION_DISEASES	-0.3129	-1.0183	0.4473	0.8576	1
KEGG_LONG_TERM_DEPRESSION	-0.2579	-1.0129	0.4447	0.8383	1
KEGG_TAURINE_AND_HYPOTAURINE_METABOLISM	-0.427	-1.0057	0.4836	0.8248	1
KEGG_ALDOSTERONE_REGULATED_SODIUM_REABSORPTION	-0.2792	-1.0009	0.4635	0.8071	1
KEGG_PROXIMAL_TUBULE_BICARBONATE_RECLAMATION	-0.3385	-0.995	0.4569	0.7918	1
KEGG_T_CELL_RECEPTOR_SIGNALING_PATHWAY	-0.2971	-0.9684	0.5031	0.8208	1
KEGG_ADIPOCYTOKINE_SIGNALING_PATHWAY	-0.2407	-0.8979	0.6173	0.9454	1
KEGG_NICOTINATE_AND_NICOTINAMIDE_METABOLISM	-0.2618	-0.8897	0.625	0.9339	1
KEGG_BETA_ALANINE_METABOLISM	-0.2832	-0.8689	0.6524	0.9474	1
KEGG_PPAR_SIGNALING_PATHWAY	-0.2326	-0.8542	0.6831	0.9503	1
KEGG_FATTY_ACID_METABOLISM	-0.2582	-0.8136	0.6821	1	1
KEGG_TYROSINE_METABOLISM	-0.2282	-0.7939	0.7564	1	1
KEGG_CARDIAC_MUSCLE_CONTRACTION	-0.2187	-0.7928	0.7186	0.9961	1
KEGG_BUTANOATE_METABOLISM	-0.2451	-0.7918	0.7329	0.9722	1
KEGG_OLFACTORY_TRANSDUCTION	-0.2089	-0.7861	0.8073	0.9585	1
KEGG_PROPANOATE_METABOLISM	-0.2538	-0.7719	0.7	0.9613	1
KEGG_PHOSPHATIDYLINOSITOL_SIGNALING_SYSTEM	-0.2118	-0.7638	0.7778	0.9531	1
KEGG_GLYCOSAMINOGLYCAN_DEGRADATION	-0.2608	-0.7561	0.7258	0.9443	1
KEGG_LEISHMANIA_INFECTION	-0.2521	-0.752	0.6727	0.9302	1
KEGG_RENIN_ANGIOTENSIN_SYSTEM	-0.2755	-0.7425	0.7954	0.9253	1
KEGG_ANTIGEN_PROCESSING_AND_PRESENTATION	-0.253	-0.7343	0.7135	0.9174	1
KEGG_B_CELL_RECEPTOR_SIGNALING_PATHWAY	-0.2321	-0.7306	0.6901	0.904	1
KEGG_NATURAL_KILLER_CELL_MEDIATED_CYTOTOXICITY	-0.2063	-0.6982	0.7613	0.933	1
KEGG_VIRAL_MYOCARDITIS	-0.2174	-0.6903	0.7879	0.9245	1
KEGG_REGULATION_OF_AUTOPHAGY	-0.2376	-0.6897	0.826	0.9068	1
KEGG_GNRH_SIGNALING_PATHWAY	-0.1626	-0.6609	0.9807	0.9253	1
KEGG_VALINE_LEUCINE_AND_ISOLEUCINE_DEGRADATION	-0.1948	-0.6204	0.8801	0.9526	1
KEGG_ALPHA_LINOLENIC_ACID_METABOLISM	-0.1966	-0.5967	0.9538	0.9569	1
KEGG_SPHINGOLIPID_METABOLISM	-0.1739	-0.5815	0.9583	0.9523	1
KEGG_ARACHIDONIC_ACID_METABOLISM	-0.155	-0.556	0.9716	0.9538	1
KEGG_GLYCOSAMINOGLYCAN_BIOSYNTHESIS_HEPARAN_SULFATE	-0.1693	-0.5237	0.99	0.956	1
KEGG_RIBOSOME	0.1291	0.2644	0.9761	0.9998	1
KEGG_GLYCOSAMINOGLYCAN_BIOSYNTHESIS_CHONDROITIN_SULFATE	0.159	0.443	0.9858	0.9919	1
KEGG_VASOPRESSIN_REGULATED_WATER_REABSORPTION	0.1661	0.5436	0.9739	0.9585	1
KEGG_LINOLEIC_ACID_METABOLISM	0.1922	0.5488	0.9561	0.9631	1
KEGG_NITROGEN_METABOLISM	0.1757	0.5529	0.9874	0.9684	1
KEGG_TERPENOID_BACKBONE_BIOSYNTHESIS	0.246	0.5976	0.8667	0.9417	1
KEGG_OTHER_GLYCAN_DEGRADATION	0.2242	0.6021	0.9027	0.9451	1
KEGG_RIBOFLAVIN_METABOLISM	0.2028	0.6056	0.9449	0.9495	1
KEGG_LYSOSOME	0.171	0.6081	0.8852	0.9551	1
KEGG_TOLL_LIKE_RECEPTOR_SIGNALING_PATHWAY	0.1826	0.6194	0.8913	0.952	1
KEGG_PANTOTHENATE_AND_COA_BIOSYNTHESIS	0.2134	0.6486	0.9194	0.9279	1
KEGG_LYSINE_DEGRADATION	0.1959	0.6511	0.8912	0.9326	1
KEGG_INOSITOL_PHOSPHATE_METABOLISM	0.1966	0.6605	0.8723	0.9295	1
KEGG_PYRUVATE_METABOLISM	0.1928	0.662	0.8902	0.9354	1
KEGG_MTOR_SIGNALING_PATHWAY	0.1981	0.6858	0.8598	0.9126	1
KEGG_MAPK_SIGNALING_PATHWAY	0.1622	0.7025	0.9458	0.8984	1
KEGG_PHENYLALANINE_METABOLISM	0.2201	0.7167	0.8569	0.8855	1
KEGG_GLYOXYLATE_AND_DICARBOXYLATE_METABOLISM	0.2616	0.7452	0.7645	0.8518	1
KEGG_CYTOSOLIC_DNA_SENSING_PATHWAY	0.2447	0.773	0.7067	0.8176	1
KEGG_PROTEIN_EXPORT	0.3	0.7843	0.7063	0.8067	1
KEGG_PARKINSONS_DISEASE	0.2518	0.7854	0.6578	0.8119	1
KEGG_ETHER_LIPID_METABOLISM	0.226	0.8017	0.7725	0.7928	1

KEGG_CYSTEINE_AND_METHIONINE_METABOLISM	0.2322	0.8074	0.7397	0.7911	1
KEGG_ALANINE_ASPARTATE_AND_GLUTAMATE_METABOLISM	0.2332	0.8104	0.7269	0.7935	1
KEGG_RETINOL_METABOLISM	0.2558	0.8153	0.6935	0.7929	1
KEGG_RNA_POLYMERASE	0.286	0.822	0.6646	0.7901	1
KEGG_DRUG_METABOLISM_CYTOCHROME_P450	0.2552	0.8321	0.627	0.7809	1
KEGG_FOLATE_BIOSYNTHESIS	0.3431	0.843	0.6437	0.7694	1
KEGG_HISTIDINE_METABOLISM	0.2555	0.8483	0.6742	0.7678	1
KEGG_STEROID_BIOSYNTHESIS	0.3448	0.8604	0.6143	0.755	1
KEGG_GLYCOSYLPHOSPHATIDYLINOSITOL_GPI_ANCHOR_BIOSYNTHESIS	0.2988	0.8624	0.6228	0.759	1
KEGG_VIBRIO_CHOLERAE_INFECTION	0.2397	0.8625	0.6645	0.7665	1
KEGG_MELANOMA	0.2247	0.8647	0.6619	0.7703	1
KEGG_GAP_JUNCTION	0.2261	0.8836	0.618	0.7435	1
KEGG_METABOLISM_OF_XENOBIOTICS_BY_CYTOCHROME_P450	0.2763	0.8858	0.5656	0.7481	1
KEGG_INSULIN_SIGNALING_PATHWAY	0.2268	0.8921	0.6126	0.745	1
KEGG_PURINE_METABOLISM	0.2192	0.8988	0.6074	0.7413	1
KEGG_OXIDATIVE_PHOSPHORYLATION	0.2931	0.9008	0.5392	0.746	1
KEGG_DILATED_CARDIOMYOPATHY	0.2346	0.9047	0.5663	0.7473	1
KEGG_SELENOAMINO_ACID_METABOLISM	0.2817	0.9184	0.5396	0.7305	1
KEGG_MELANOGENESIS	0.226	0.9244	0.6037	0.7279	1
KEGG_PEROXISOME	0.2611	0.9384	0.5234	0.7096	1
KEGG_NOD_LIKE_RECEPTOR_SIGNALING_PATHWAY	0.2895	0.9496	0.5234	0.6973	1
KEGG_GLYCEROLIPID_METABOLISM	0.256	0.9569	0.494	0.6928	1
KEGG_HEDGEHOG_SIGNALING_PATHWAY	0.2625	0.9582	0.5146	0.6984	1
KEGG_LONG_TERM_POTENTIATION	0.2632	0.9619	0.5041	0.7001	1
KEGG_ARGININE_AND_PROLINE_METABOLISM	0.256	0.9665	0.5085	0.6997	1
KEGG_TGF_BETA_SIGNALING_PATHWAY	0.2758	0.9675	0.5115	0.706	1
KEGG_STEROID_HORMONE_BIOSYNTHESIS	0.2897	0.9704	0.4927	0.7091	1
KEGG_NON_SMALL_CELL_LUNG_CANCER	0.2801	0.977	0.4758	0.7053	1
KEGG_GLIOMA	0.2661	0.9861	0.498	0.6968	1
KEGG_AMYOTROPHIC_LATERAL_SCLEROSIS_ALS	0.2806	1.0185	0.4266	0.6448	1
KEGG_ASCORBATE_AND_ALDARATE_METABOLISM	0.38	1.0197	0.4469	0.6507	1
KEGG_EPITHELIAL_CELL_SIGNALING_IN_HELICOBACTER_PYLORI_INFECTION	0.2825	1.0276	0.4168	0.6456	1
KEGG_FC_GAMMA_R_MEDIATED_PHAGOCYTOSIS	0.2874	1.0363	0.4187	0.6382	1
KEGG_ERBB_SIGNALING_PATHWAY	0.2692	1.039	0.424	0.6422	1
KEGG_PROGESTERONE_MEDIATED_OOCYTE_MATURATION	0.2844	1.0427	0.4029	0.6447	1
KEGG_HYPERTROPHIC_CARDIOMYOPATHY_HCM	0.2728	1.063	0.3376	0.6166	0.998
KEGG_GLYCEROPHOSPHOLIPID_METABOLISM	0.2584	1.0635	0.3529	0.6243	0.998
KEGG_VEGF_SIGNALING_PATHWAY	0.2755	1.0732	0.3574	0.6151	0.998
KEGG_ACUTE_MYELOID_LEUKEMIA	0.324	1.0801	0.3968	0.6122	0.998
KEGG_PENTOSE_AND_GLUCURONATE_INTERCONVERSIONS	0.38	1.0806	0.3531	0.6202	0.998
KEGG_SNARE_INTERACTIONS_IN_VESICULAR_TRANSPORT	0.3312	1.0807	0.4009	0.6291	0.998
KEGG_NEUROTROPHIN_SIGNALING_PATHWAY	0.29	1.0953	0.3785	0.6098	0.997
KEGG_LEUKOCYTE_TRANSENDOTHELIAL_MIGRATION	0.2954	1.1006	0.3278	0.6093	0.997
KEGG_ECM_RECEPTOR_INTERACTION	0.326	1.1029	0.3732	0.614	0.997
KEGG_GLYCOSAMINOGLYCAN_BIOSYNTHESIS_KERATAN_SULFATE	0.3832	1.1076	0.3286	0.6147	0.997
KEGG_O_GLYCAN_BIOSYNTHESIS	0.3584	1.1145	0.3439	0.6115	0.996
KEGG_CITRATE_CYCLE_TCA_CYCLE	0.3993	1.1333	0.3541	0.5844	0.993
KEGG_PROSTATE_CANCER	0.315	1.1376	0.3224	0.586	0.992
KEGG_DORSO_VENTRAL_AXIS_FORMATION	0.3718	1.1411	0.2965	0.5891	0.992
KEGG_AMINO_SUGAR_AND_NUCLEOTIDE_SUGAR_METABOLISM	0.3466	1.157	0.3074	0.5673	0.99
KEGG_GLYCOSPHINGOLIPID_BIOSYNTHESIS_GLOBO_SERIES	0.4112	1.1781	0.2802	0.5366	0.987
KEGG_HUNTINGTONS_DISEASE	0.3293	1.1834	0.299	0.5366	0.986
KEGG_ALZHEIMERS_DISEASE	0.3295	1.1915	0.2713	0.5305	0.985
KEGG_SULFUR_METABOLISM	0.436	1.1992	0.2449	0.5259	0.984
KEGG_FRUCTOSE_AND_MANNOSE_METABOLISM	0.3686	1.2018	0.2449	0.531	0.984
KEGG_SYSTEMIC_LUPUS_ERYTHEMATOSUS	0.3524	1.2053	0.2793	0.5338	0.984
KEGG_COLORECTAL_CANCER	0.3476	1.2092	0.2619	0.5364	0.984
KEGG_UBIQUITIN_MEDIATED_PROTEOLYSIS	0.3558	1.2109	0.2627	0.5435	0.983
KEGG_RENAL_CELL_CARCINOMA	0.3525	1.2178	0.2587	0.5412	0.981
KEGG_VALINE_LEUCINE_AND_ISOLEUCINE_BIOSYNTHESIS	0.4909	1.22	0.2546	0.5482	0.981
KEGG_FOCAL_ADHESION	0.3194	1.2222	0.218	0.5547	0.98
KEGG_RIG_I_LIKE_RECEPTOR_SIGNALING_PATHWAY	0.3738	1.2294	0.2322	0.5516	0.979
KEGG_ENDOMETRIAL_CANCER	0.3659	1.2486	0.2202	0.5218	0.976
KEGG_NON_HOMOLOGOUS_END_JOINING	0.4968	1.249	0.2337	0.5327	0.976
KEGG_LIMONENE_AND_PINENE_DEGRADATION	0.5096	1.2539	0.2416	0.5357	0.975
KEGG_CHRONIC_MYELOID_LEUKEMIA	0.3643	1.2597	0.2231	0.5372	0.975
KEGG_BIOSYNTHESIS_OF_UNSATURATED_FATTY_ACIDS	0.407	1.2597	0.2173	0.5498	0.975
KEGG_BASAL_CELL_CARCINOMA	0.3571	1.2753	0.1591	0.5301	0.971
KEGG_APOPTOSIS	0.3615	1.2762	0.2061	0.5414	0.97
KEGG_AMINOACYL_TRNA_BIOSYNTHESIS	0.4622	1.3137	0.2303	0.4756	0.963
KEGG_OOCYTE_MEIOSIS	0.3629	1.3246	0.1431	0.4659	0.961
KEGG_ARRHYTHMOGENIC_RIGHT_VENTRICULAR_CARDIOMYOPATHY_ARVC	0.3497	1.3257	0.1543	0.4758	0.961
KEGG_BASAL_TRANSCRIPTION_FACTORS	0.4372	1.3297	0.1371	0.4796	0.958
KEGG_N_GLYCAN_BIOSYNTHESIS	0.4114	1.3427	0.1423	0.463	0.951
KEGG_DRUG_METABOLISM_OTHER_ENZYMES	0.4136	1.3524	0.1213	0.4565	0.949
KEGG_WNT_SIGNALING_PATHWAY	0.3327	1.363	0.0932	0.4482	0.945
KEGG_REGULATION_OF_ACTIN_CYTOSKELETON	0.3341	1.3747	0.1013	0.4382	0.941
KEGG_ENDOCYTOSIS	0.3429	1.3896	0.0958	0.4232	0.933
KEGG_GLUTATHIONE_METABOLISM	0.4081	1.4104	0.1127	0.395	0.92
KEGG_STARCH_AND_SUCROSE_METABOLISM	0.4188	1.4318	0.0694	0.3663	0.908

KEGG_PANCREATIC_CANCER	0.4113	1.4439	0.087	0.3584	0.899
KEGG_SMALL_CELL_LUNG_CANCER	0.4119	1.4532	0.0755	0.3537	0.892
KEGG_DNA_REPLICATION	0.5886	1.4564	0.1417	0.3616	0.889
KEGG_NOTCH_SIGNALING_PATHWAY	0.4108	1.4565	0.0734	0.376	0.889
KEGG_PORPHYRIN_AND_CHLOROPHYLL_METABOLISM	0.4432	1.4615	0.0679	0.3792	0.885
KEGG_HOMOLOGOUS_RECOMBINATION	0.5349	1.4635	0.1145	0.3919	0.883
KEGG_PYRIMIDINE_METABOLISM	0.4191	1.469	0.0947	0.399	0.879
KEGG_MISMATCH_REPAIR	0.564	1.4698	0.1032	0.4163	0.878
KEGG_PATHWAYS_IN_CANCER	0.3548	1.4728	0.0557	0.4302	0.876
KEGG_AXON_GUIDANCE	0.3775	1.4908	0.0422	0.4097	0.858
KEGG_RNA_DEGRADATION	0.4843	1.5139	0.0713	0.3771	0.831
KEGG_GALACTOSE_METABOLISM	0.4844	1.5619	0.0363	0.2969	0.762
KEGG_TIGHT_JUNCTION	0.3918	1.5771	0.0238	0.2871	0.733
KEGG_NUCLEOTIDE_EXCISION_REPAIR	0.5291	1.5829	0.0373	0.2958	0.727
KEGG_CIRCADIAN_RHYTHM_MAMMAL	0.6363	1.5917	0.028	0.2991	0.708
KEGG_CELL_CYCLE	0.4846	1.5932	0.0692	0.3189	0.703
KEGG_ONE_CARBON_POOL_BY_FOLATE	0.5663	1.6078	0.0343	0.311	0.674
KEGG_GLYCOSPHINGOLIPID_BIOSYNTHESIS_LACTO_AND_NEOLACTO_SERIES	0.4834	1.6203	0.0202	0.3122	0.659
KEGG_BASE_EXCISION_REPAIR	0.5884	1.6358	0.056	0.3117	0.629
KEGG_GLYCOLYSIS_GLUCONEOGENESIS	0.4729	1.6847	0.0142	0.239	0.538
KEGG_SPLICEOSOME	0.5326	1.6869	0.0379	0.2645	0.53
KEGG_BLADDER_CANCER	0.4965	1.7177	0.0083	0.2353	0.462
KEGG_ADHERENS_JUNCTION	0.4994	1.7193	0.0146	0.2709	0.461
KEGG_PATHOGENIC_ESCHERICHIA_COLI_INFECTION	0.5396	1.7772	0.0128	0.2054	0.338
KEGG_PENTOSE_PHOSPHATE_PATHWAY	0.5837	1.7919	0.0084	0.2265	0.308
KEGG_PROTEASOME	0.7068	1.8144	0.0122	0.2477	0.268
KEGG_P53_SIGNALING_PATHWAY	0.519	1.9044	0.002	0.1372	0.115
KEGG_THYROID_CANCER	0.6689	2.1488	0	0.0084	0.004
